# S-SCAM is essential for synapse formation

**DOI:** 10.3389/fncel.2023.1182493

**Published:** 2023-11-16

**Authors:** Nina Wittenmayer, Andonia Petkova-Tuffy, Maximilian Borgmeyer, Chungku Lee, Jürgen Becker, Andreas Böning, Sebastian Kügler, JeongSeop Rhee, Julio S. Viotti, Thomas Dresbach

**Affiliations:** ^1^Institute of Anatomy and Embryology, University Medical Center Göttingen, Göttingen, Germany; ^2^Institute for Translational Medicine, MSH Medical School Hamburg, Hamburg, Germany; ^3^Department of Molecular Neurobiology, Synaptic Physiology Group, Max Planck Institute for Multidisciplinary Sciences, Göttingen, Germany; ^4^Institute of Anatomy and Cell Biology, University Medical Center Göttingen, Göttingen, Germany; ^5^Department of Neurology, University Medical Center Göttingen, Göttingen, Germany; ^6^University of Bordeaux, CNRS, IINS, UMR 5297, Bordeaux, France

**Keywords:** synapse formation, scaffolding protein, neuronal morphology, synaptic transmission, spine

## Abstract

Synapse formation is critical for the wiring of neural circuits in the developing brain. The synaptic scaffolding protein S-SCAM/MAGI-2 has important roles in the assembly of signaling complexes at post-synaptic densities. However, the role of S-SCAM in establishing the entire synapse is not known. Here, we report significant effects of RNAi-induced S-SCAM knockdown on the number of synapses in early stages of network development *in vitro*. *In vivo* knockdown during the first three postnatal weeks reduced the number of dendritic spines in the rat brain neocortex. Knockdown of S-SCAM in cultured hippocampal neurons severely reduced the clustering of both pre- and post-synaptic components. This included synaptic vesicle proteins, pre- and post-synaptic scaffolding proteins, and cell adhesion molecules, suggesting that entire synapses fail to form. Correspondingly, functional and morphological characteristics of developing neurons were affected by reducing S-SCAM protein levels; neurons displayed severely impaired synaptic transmission and reduced dendritic arborization. A next-generation sequencing approach showed normal expression of housekeeping genes but changes in expression levels in 39 synaptic signaling molecules in cultured neurons. These results indicate that S-SCAM mediates the recruitment of all key classes of synaptic molecules during synapse assembly and is critical for the development of neural circuits in the developing brain.

## Introduction

Synapses are asymmetric intercellular junctions. They are specialized for synaptic transmission, thus mediating information processing by neural circuits. Synapse formation is a highly orchestrated process involving the initial axo-dendritic contact and the induction of pre- and post-synaptic differentiation, which involves the accumulation of pre- and post-synaptic synaptic proteins and synaptic vesicles at the nascent synaptic contact. The synapse differentiation process is followed by a prolonged step of structural and functional maturation (Garner et al., 2006). During all steps of synaptogenesis, signaling by trans-synaptic cell adhesion molecules in cooperation with pre- and post-synaptic scaffolding proteins mediates the bidirectional organization of synaptic components and determines the final properties of the developing synapse (Sudhof, 2017, 2018).

Scaffolding proteins are multi-domain molecules equipped to bind, recruit, and anchor a large number of binding partners to a certain site of action (Kim and Sheng, 2004). One of the scaffolding proteins acting at post-synaptic sites is called the synaptic scaffolding molecule (S-SCAM). S-SCAM was originally identified and named based on its interactions with three other post-synaptic proteins, i.e., post-synaptic cell adhesion molecules of the neuroligin family, N-methyl-d-aspartate type glutamate receptors (NMDAR), and the protein SAPAP/GKAP (Hirao et al., 1998). S-SCAM is also called MAGI-2 for membrane-associated guanylate kinase inverted-2 (Wu et al., 2000), AIP-1 for atropine-interacting protein 1 (Wood et al., 1998), and ARIP-1 for activin receptor-interacting protein 1 (Shoji et al., 2000). S-SCAM is equipped with multiple domains for protein-protein interactions, including one guanylate kinase (GK) domain, two WW domains, and six PDZ domains (Hirao et al., 1998; Nagashima et al., 2015). S-SCAM interacts with numerous interaction partners at both excitatory and inhibitory synapses (reviewed in Nagashima et al., 2015). Knockdown (KD) and overexpression experiments in mature hippocampal cultures implicate S-SCAM in maintaining synaptic strength by stabilizing the GluA2-containing pool of AMPA receptors at excitatory synapses (Danielson et al., 2012) and in the maintenance of inhibitory synapses (Shin et al., 2020). Thus, S-SCAM is important for the structural and functional maintenance of synapses.

There are three alternative splicing variants, S-SCAMα, -β, and -γ, which start with different initiation methionines. Mice lacking the longest variant, S-SCAMα, died within 24 h after birth. In cultured neurons obtained from these mice, the number of synapses was normal, but dendritic spines were abnormally elongated (Iida et al., 2007). Two distinct mouse lines lacking all three S-SCAM isoforms live for up to 3 weeks or 3 months, depending on the line, after which the mice die of renal failure, highlighting the crucial role of S-SCAM in the kidney (Balbas et al., 2014; Ihara et al., 2014). Thus, the importance of S-SCAM for synapse formation in the brain is still unknown. Interestingly, mutations in the human S-SCAM gene have been associated with neurological diseases such as infantile spasm (Marshall et al., 2008), schizophrenia (Walsh et al., 2008), and epilepsy (DiFrancesco et al., 2019). Transgenic mice overexpressing S-SCAM in excitatory forebrain neurons, thus mimicking part of the schizophrenia-associated S-SCAM gene duplication (Walsh et al., 2008), show schizophrenia-related behavioral phenotypes, hyperglutamatergic function, and a reduction in the number of dendritic spines (Zhang et al., 2015).

These data highlight the importance of S-SCAM for maintaining the integrity of cell–cell junctions and proper brain function. However, its role in synapse assembly has remained elusive. Here, we use a triple isoform knockdown approach to reduce the levels of all three S-SCAM isoforms in immature hippocampal cultures, i.e., during the onset of synapse formation, and in the neonatal rat brain, in order to study the importance of S-SCAM for synaptogenesis.

## Materials and methods

### DNA constructs

For performing RNA interference-mediated knockdown of S-SCAM/MAGI-2, the hairpin sequences used were cloned into pFUGW, which coexpresses EGFP (for detailed cloning information, see Stan et al., 2010). Two different shRNA sequences (Oligo#1 and Oligo#2) were used in the article. Their specificity and efficiency were previously demonstrated (Oligo#1: Danielson et al., 2012; Oligo#2: Stan et al., 2010). Oligo#2 was used in all experiments; in addition, Oligo#1 was used in Figure 2. S-SCAM/MAGI2 rescue constructs were generated by introducing silent mutations in the S-SCAM/MAGI2 RNAi targeting sequence and subcloning of the mutated S-SCAM/MAGI2 cDNA in pcDNA3_mOrange2 (Prof. Thomas Kuner, Heidelberg). The coding region for the rescue constructs was followed by an IRES and mOrange2. Detailed information about the sequences of the shRNAs, the mutations introduced into the rescue constructs, and the complete conservation of the shRNA targeted sequences between rat and mouse S-SCAM is summarized in Supplementary Data S3.

### Primary neuronal cell culture, transfection, and infection with recombinant lentiviruses

Conventional dissociated hippocampal neurons, as opposed to micro-island cultures (see below), were prepared from embryonic day 19 rats as previously described (Wittenmayer et al., 2009). Briefly, hippocampi were dissected in HBSS and digested with trypsin for 20 min at 37°C. Hippocampi were triturated, and dissociated cells were counted and plated in DMEM medium with the addition of B-27 supplement, glutamine, and penicillin/streptomycin. After 4 h, the medium was changed to a neurobasal medium with the addition of B-27 supplement, glutamine, and penicillin/streptomycin (Invitrogen). High-density cultures (40,000 neurons/well) were plated on poly-l-lysine-coated 13-mm glass coverslips in 24-well plates. At day *in vitro* (DIV) 2, neuronal cultures were transfected with calcium phosphate (Dresbach et al., 2003) or infected with lentiviruses. Neurons were processed for immunocytochemistry at DIV9/10 or DIV17.

Micro-island cultures of autaptic hippocampal neurons were prepared and cultured as described previously (Burgalossi et al., 2012). In brief, astrocytes were obtained from mouse cortices from P0 WT animals using digestion with 0.25% trypsin (Gibco) for 20 min at 37°C. The cells were plated in T75 culture flasks in DMEM medium (Gibco) containing 10% FBS (PAA) and penicillin/streptomycin (Gibco). The medium was exchanged the day after plating, and cells were allowed to grow for 7–10 days. Following this, cells were collected from the flask using trypsin digestion and plated at a density of 12,000 cells/well on 32-mm coverslips. The coverslips used for micro-island cultures were first coated with agarose (Sigma-Aldrich) and then with a coating solution containing poly-d-lysine (Sigma-Aldrich), acetic acid, and collagen (BD), using a custom-made stamp to generate 200 μm × 200 μm substrate islands. Hippocampi from postnatal day 1 (P1) mice were dissected free of meninges and separately collected in ice-cold Hanks' balanced salt solution (HBSS; Gibco). They were incubated in papain solution containing 2 mg cysteine, 10 ml DMEM (Gibco), 1 mM CaCl_2_, and 0.5 mM EDTA, along with 20–25 units of papain (Worthington Biomedical Corporation); 45 min for hippocampi and 60 min for striatum at 37°C). After washing, cells were triturated and counted in a Fuchs-Rosenthal or Naubauer chamber. The cells were plated in pre-warmed neurobasal medium (Gibco) supplemented with B-27 (Gibco), glutamax (Gibco), and penicillin/streptomycin (Gibco) at a density of 25,000 cells/well on 18 mm coverslips for high-density cultures or 4,000 cells/well on a 32 mm coverslip for micro-island cultures.

Recombinant lentiviruses for delivery of expression plasmids were generated as described (Rubinson et al., 2003). Neurons were transduced using lentivirus particles delivering either control shRNAs or knockdown shRNAs directed against S-SCAM/MAGI2α, -β, and -γ. The time windows for transduction and analysis are indicated for each experiment. To confirm that the constructs reduced the protein expression of S-SCAM/MAGI2, total cell lysates from cultures transduced at DIV2 were collected at DIV9. To confirm the functionality of rescue constructs, HEK293 cells were cotransfected with either control or the S-SCAM/MAGI2 knockdown FUGW plasmids and rescue constructs for either S-SCAM/MAGI2α, -β, or -γ. Antibodies used for Western blotting were rabbit polyclonal MAGI2 (1:1000, M2441, Sigma-Aldrich, RRID:AB_477175), GFP (1:2000, Abcam, RRID:AB_305564), and mouse monoclonal tubulin (1:2000, Sigma-Aldrich, RRID:AB_477593).

### Immunocytochemistry

Depending on the antibodies, cells were fixed in either 4% paraformaldehyde in PBS, pH 7.4, for 10 or 20 min at RT or in ice-cold methanol for 20 min at −20°C. Cells were washed in PBS and first permeabilized for 30 min in PBS containing 2% normal goat serum and 0.2% Triton X-100 (Sigma-Aldrich). Incubation with primary antibodies was performed overnight at 4°C and with secondary antibodies for 1 h at RT. Polyclonal antibodies used were VGAT (1:1000, Synaptic Systems, RRID:AB_887869), MAGI2 (1:1000, M2441, Sigma-Aldrich, RRID:AB_477175), PSD95 (1:600, Invitrogen, 15386994), Bassoon (1:500, Synaptic Systems, RRID:AB_2661779), GFP (1:2000, Abcam, RRID:AB_305564), GFP (1:500, Synaptic Systems; RRID:AB_2713983), and VAMP2 (1:1000, Synaptic Systems, 104202). Monoclonal antibodies used were pan-Shank (1:800, NeuroMab, RRID:AB_10672418), Bassoon (1:800, Stressgene, RRID:AB_2038857), VGlut1 (1:1000, Synaptic Systems, RRID:AB_887879), Synaptophysin (1:800, Synaptic Systems, RRID:AB_887824), Neuroligin (1:600, Synaptic Systems, RRID:AB_1210390), Gephyrin (1:1000, Synaptic Systems, mAB7A, RRID:AB_2810214), and PSD95 (1:500, Abcam, RRID:AB_300453).

### Lentivirus delivery *in vivo*

Lentiviral vectors were applied to the rat neonatal cortex by means of a fine-bore glass capillary (μTip, WPI, # TIP30TW1LS01) attached to a 5 μl Hamilton syringe. The shape of the μ-tip auto-limits the penetration depth, thereby avoiding tissue damage. In total 2 μl of viral suspension were injected into the cortex of neonates at P3. All animal experiments were conducted according to approved experimental animal licenses issued by the responsible animal welfare authority (Niedersächsisches Landesamt für Verbraucherschutz und Lebensmittelsicherheit, LAVES) and controlled by the local animal welfare committee and veterinarians of the University Medical Center Göttingen.

### Immunohistochemistry

P19 rats were transcardially perfused with 15 ml of PBS, followed by 20 ml of 4% paraformaldehyde (PFA) dissolved in PBS. The brains were removed and stored in 4% PFA at 4°C for 2–3 h for post-fixation. PFA was removed by three washes in PBS before the cortex was cut into 50-μm-thick sections using a vibratome (Mikrom International GmbH). Immunostaining was performed using primary antibodies against GFP (1:750; Medical & Biological Laboratories Co., RRID:AB_591817) and Cy2-coupled secondary antibodies (1:500; Jackson ImmunoResearch, RRID:AB_2340673). All incubation steps were performed on a horizontal shaker. Brain sections were permeabilized and blocked for 2 h at room temperature in PBS containing 5% normal goat serum and 1% Triton X-100. Primary antibodies were applied overnight at 4°C in PBS containing 1% normal goat serum and 0.2% Triton X-100, followed by three 15-min washes using PBS supplemented with 2% normal goat serum. All subsequent steps were performed at room temperature. Secondary antibodies were applied in PBS containing 1% normal goat serum and 0.2% Triton X-100. After three 15-min incubations with washing solution (1% normal goat serum in PBS) and three 10-min rinsing steps using PBS, the brain sections were mounted on glass slides (H868; Roth) and embedded in Slow Fade Gold (S36936; Invitrogen), using 100-μm spacers to prevent the tissue from being squeezed by the coverslip (1871; Roth). Specimens were sealed with commercially available nail polish and stored protected from light at 4°C.

### Electrophysiology

For whole-cell voltage-clamp recordings of autaptic cultures, cells were held at −70 mV, and holding voltage was increased to 0 mV (2 ms) to depolarize cells (MultiClamp 700B amplifier, Axon Instruments, Molecular Devices) under the control of the Clampex program 10.1 (Molecular Devices). Recordings were done at room temperature. The flow of extracellular solution was maintained during recordings, and pharmacological solutions were applied if required. Hypertonic sucrose solution (0.5 M in external solution) was used to trigger the fusion of the readily releasable pool of vesicles (RRP). NBQX (HelloBio) and bicuculline (Tocris) were used to inhibit AMPA and GABA receptors, respectively. Miniature excitatory post-synaptic currents (mEPSCs) were recorded in the presence of 300 nM tetrodotoxin (TTX; Tocris). The extracellular solution contained 140 mM NaCl, 2.4 mM KCl, 10 mM Hepes, 10 mM Glucose, 4 mM CaCl_2_, 4 mM MgCl_2_ (320 mOsmol/liter), and the patch-pipette solution for recording contained 136 mM KCl, 17.8 mM Hepes, 1 mM EGTA, 4.6 mM MgCl_2_, 4 mM NaATP, 0.3 mM Na_2_GTP, 15 mM creatine phosphate, and 5 U/ml phosphocreatine kinase (315–320 mOsmol/liter), pH 7.4. Cells were visualized through an inverted microscope (Olympus or Zeiss). Custom-made manipulators controlled the movement of the microelectrode. All reagents, including 0.5 M hypertonic sucrose solution, were applied with a custom-built fast flow system consisting of an array of flow pipes controlled by a stepper motor that allows complete and rapid solution exchange with time constants of ~30 ms. Pressure was on for 20 ms. Measurements were performed and registered using an Axon 700B amplifier (Axon Instruments), and signals were converted with an Axon Instrument digitizer. The recording rate was 10 kHz. Microelectrodes were made by using a Sutter 2,000 filament-based horizontal puller and were used only if they had pipette resistances of 2.5–4.5 MΩ. Serial resistance was compensated by 35–60%, and cells with serial resistances below 12 MΩ were analyzed. All chemicals were purchased from Sigma-Aldrich unless mentioned otherwise. All autaptic electrophysiology data were analyzed using Axograph (Ver. 1.5.4) software (Axograph Scientific). Recordings of S-SCAM KD and control autaptic neurons were performed from DIV11 to DIV14.

Whole-cell patch clamp recordings of conventional dissociated cultured hippocampal neurons were made at 37°C ± 0.5°C using a HEKA EPC-10 USB amplifier and the software Patchmaster (HEKA Electronics). During recordings, cultures were continuously perfused with an extracellular solution consisting of (in mM): NaCl 125.0, KCl 2.5, NaHCO_3_ 25.0, NaH_2_PO_4_ 1.25, CaCl_2_ 2.0, MgCl_2_ 1.0, glucose 20.0 (pH 7.4), with the addition of 1 μM TTX. The intracellular solution consisted of (in mM): 150 K^+^-D-Gluconate, 10 NaCl, 3 Mg-ATP, 0.3 Na-GTP, 10 Hepes, and 0.05 EGTA (pH 7.3). Cells were voltage-clamped at −70 mV, and a liquid junction potential of 13 mV was corrected online. Recordings were discarded if the series resistance was >10 MΩ. Recordings were digitized at 20 kHz and filtered at 2.9 kHz. mEPSCs in conventional cultures were detected by the software MiniAnalysis (Synaptosoft). Recordings of S-SCAM KD and control neurons in conventional cultures were performed at DIV9–10.

### Cell lysis and western blotting analyses

Transfected HEK293 cells were harvested and resuspended in ice-cold RIPA buffer (10 mM Tris-HCl, pH 8.0, 1 mM EDTA, 0.5 mM EGTA, 1% Triton X-100, 0.1% sodium deoxylcholate, 0.1% SDS, 140 mM NaCl, complete protease inhibitor [Roche]). After 15 min incubation on ice, cell fragments were homogenized by passing the suspension through a syringe. After centrifugation at 13,000 rpm for 5 min, the protein concentration of the supernatant was determined by a Bradford assay using BSA as a standard. Equal amounts of proteins were separated by SDS-PAGE and transferred onto the PVDF membrane. Membranes were blocked with 10% fetal calf serum in PBS. The following antibodies were used: rabbit anti-MAGI-2 (1:1000, M2441, Sigma-Aldrich, RRID:AB_477175), mouse anti-tubulin (1:5000, T9026, Sigma-Aldrich, RRID:AB_477593), and guinea pig anti-GFP (1:600, 132004, SynapticSystems, RRID:AB_11041999). Western blotting samples were obtained from DIV14 mouse primary hippocampal mass cultures (800,000 cells per well) after transducing with virus particles at DIV2 as described (Ripamonti et al., 2017). Samples were separated on 8% SDS-PAGE gels and then transferred to nitrocellulose membranes (Millipore). The membranes were blocked for 1 h at room temperature with 5% non-fat dry milk in TBST (Tris-buffered saline containing 20 mM Tris-HCl, pH 7.4, 137 mM NaCl, and 0.1% Tween-20). The membranes were probed with antibodies to MAGI-2 (rabbit anti-MAGI-2, Sigma-Aldrich, M2441, 1:250, RRID:AB_477175) or β-actin (rabbit anti-β-actin, Synaptic Systems, 251 003, 1:1000, RRID:AB_11042458) and corresponding horseradish peroxidase (HRP) conjugated goat anti-rabbit IgG (H+L) (Jackson ImmunoResearch Laboratories, 111-035-144, 1:10000, RRID:AB_2307391). The blots were developed using enhanced chemiluminescence (ECL) substrates (Amersham Pharmacia Biotech, RPN2106) and ChemoCam Imager 3.2 (Intas Science Imaging).

### Deep sequencing

At DIV2, hippocampal neurons were infected with lentiviruses delivering knockdown shRNAs (Oligo#2) directed against S-SCAM/MAGI2. As a control, an empty shRNA vector was used. At DIV9, RNA was isolated using the TRIzol^®^ Reagent (Life Technologies) according to manufacturer instructions. RNA quality was assessed by measuring the RIN (RNA Integrity Number) using an Agilent 2100 Bioanalyzer (Agilent Technologies, Palo Alto, CA). Library preparation for RNA-Seq was performed using the TruSeq™ RNA Sample Prep Kit v2 (Illumina, Cat. No. RS-122-2002), starting from 3,000 ng of total RNA. Accurate quantitation of cDNA libraries was performed using the QuantiFluor™ dsDNA System (Promega). The size range of final cDNA libraries was determined by applying the DNA 1,000 chip to the Bioanalyzer 2,100 from Agilent (average 350 bp). cDNA libraries were amplified and sequenced by using the cBot and HiSeq2000 from Illumina (SR; 1 × 50 bp; ~30–40 million reads per sample). Sequence images were transformed with Illumina software BaseCaller to bcl files, which were demultiplexed to fastq files with CASAVA v1.8.2. Quality check was done via FastQC (v. 0.10.0, Babraham Bioinformatics). Read alignment was performed using STAR v2.3.0 to the Rattus norvegicus v5 reference genome. Data were converted and sorted by Samtools 1.2, and reads per gene were counted via htseq version 0.6. Data analysis was performed using R (3.2.1), and differential gene expression was performed using DESeq2 (v1.8.1). Candidate genes were filtered to a minimum FDR-corrected *p*-value < 0.05. Sequence data have been deposited in NCBI's Gene Expression Omnibus and are accessible through GEO Series accession number GSE228093. For functional analysis, Gene Ontology (GO; Ashburner et al., 2000) enrichment was tested using g:Profiler (Reimand et al., 2007) against a statistical domain scope of all annotated genes. Significance was assessed using the g:SCS threshold. To decrease redundancy in GO terms, an upper limit of term size was set to 1,000. To investigate the interaction between differentially expressed gene products, we used STRING (Szklarczyk et al., 2015). The displayed genes were limited to those in the same functional network as S-SCAM (Magi2) and only those with a high confidence interaction score (minimum of 0.77).

### qPCR

Methods for qPCR were basically the same as described (Becker et al., 2012). In brief, total RNA was extracted using Nucleozol (Macherey-Nagel, Cat. No. 740404.200) directly from the culture plates, and 1 μg RNA was transcribed using the Omniscript RT kit (Qiagen, Cat. No. 205113). qPCR was performed on a MIC qPCR cycler (Bio Molecular Systems) using Fast SYBR Green Master Mix (Thermo Fisher Scientific, Cat. No. 4385616). All kits were used as suggested by the manufacturers. All samples were tested in triplicates, and relative expression was determined using the mean Ct of the triplicates and normalization to β-actin or α-tubulin according to the 2^−ΔΔCT^ method (Livak and Schmittgen, 2001). The resulting values were log transformed to calculate the log_2_ fold change. Primers are listed in Supplementary Data S2.

### Microscopy

Images were acquired using a SPOT-cooled CCD camera (Visitron Systems) attached to a Zeiss Axiovert 200M. Images were collected using 40 × and 63 × Plan-Neofluar oil objectives, and digital images were analyzed with Metamorph software. Image acquisition and analysis were done in a blind manner. For measuring puncta number and intensity per given neuron, four to five dendritic segments were used, using the proximal 25 μm of dendrites starting from the soma and their average values.

### Quantification of synaptic puncta

Dissociated neurons were plated on poly-l-lysine-coated 13-mm glass coverslips in 24-well plates. Cells were fixed and stained with neuronal markers Bassoon or Synaptophysin as presynaptic markers and PSD95 as a post-synaptic marker. Image analyses were conducted in Opera Phenix at 60 × magnification using Harmony software. Proximity localization of post and presynapse was quantified using OpenView software (Tsuriel et al., 2006). Postsynapses were automatically detected using the BoxpunctaEx function. An 8 × 8 pixel box was placed around the maximum of each postsynapse. Proximity-localized presynapses were detected using the MatchSet function. The number of synapses was counted along the selected dendritic segment.

### Spine analysis

Confocal images were acquired using a laser-scanning confocal microscope (Leica TCS SP5, Leica Microsystems CMS, Germany) equipped with 488 nm (Ar) and 561/633 nm (He–Ne) lasers for excitation of the respective Alexa fluorophores and a 63 × /1.4 NA oil-immersion objective. The spine analysis was conducted using the Dendritic Spine Counter 1.4.7 plugin in Fiji/ImageJ software. The average spine size was calibrated manually, allowing for automatic detection of spines along the dendrite. The automatically generated results were adjusted manually if necessary. The dendrite was traced using the Polyline tool, and the number and classes of spines were quantified along the tranced stretch.

### Statistical analyses

All experimental data were collected at least from triplicate experiments using independent batches of neuron cultures. All data values represent means ± SEM. For multiple group comparisons, one- or two-way ANOVA with Tukey's multiple comparison *post-hoc* test was used using GraphPad Prism software.

## Results

### Loss of function of S-SCAM causes a severe reduction of synapse formation in hippocampal cultures

**Figure 1:**
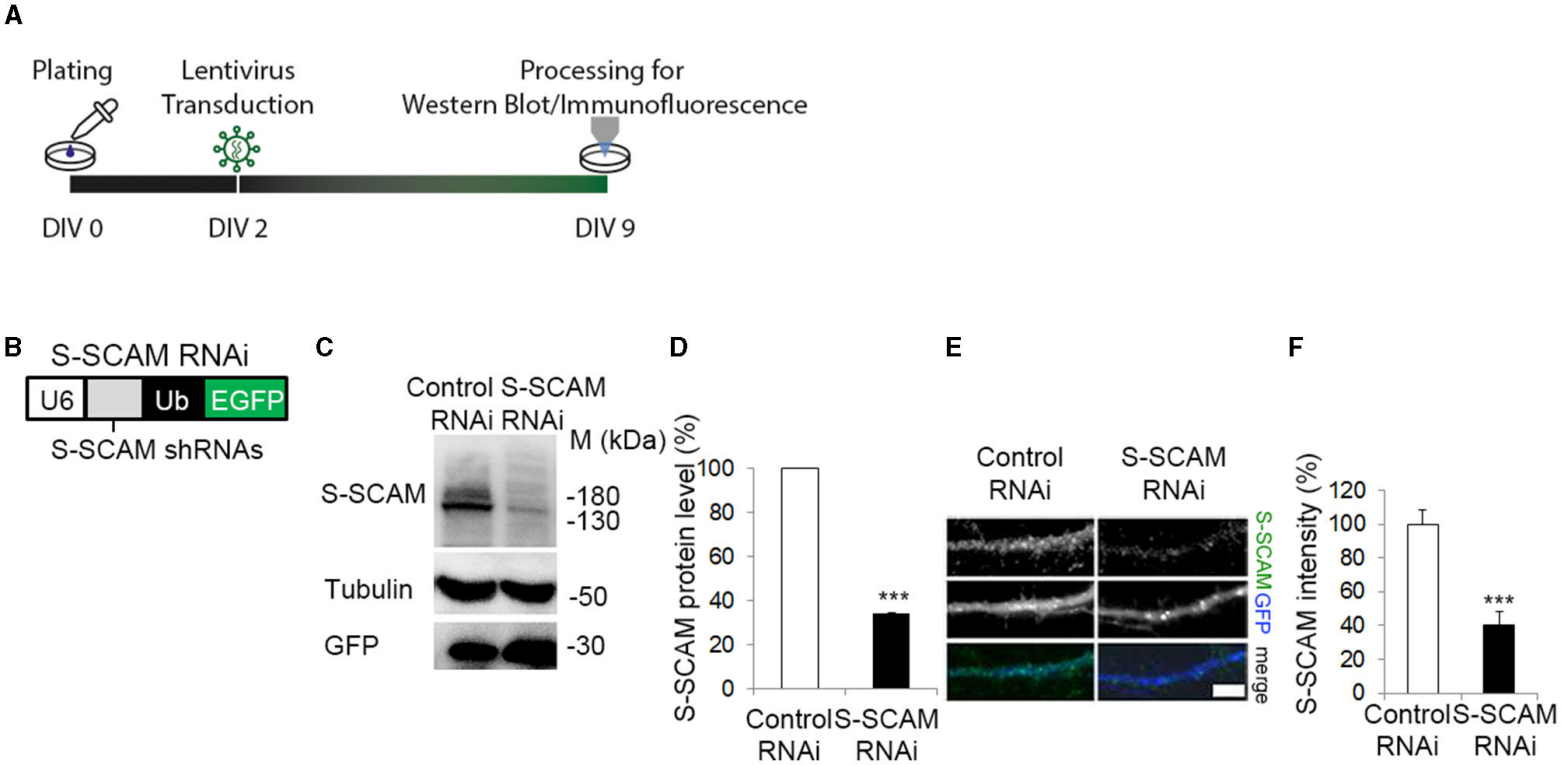
ShRNA mediated knockdown of S-SCAM in cultured hippocampal neurons. **(A)** Schematic diagram of the experimental timeline. **(B)** Design of lentiviral shRNA vectors for knockdown of all three S-SCAM isoforms. U6, U6 RNA polymerase III promotor; Ub, ubiquitin promotor. **(C–F)** Specificity and efficacy of S-SCAM-RNAi. **(C)** Western blot confirming knockdown of endogenous S-SCAM at protein level in low-density cultures of hippocampal neurons. **(D)** Semi-quantitative analysis of band intensities. **(E)** Representative images of S-SCAM knockdown neurons immunostained for S-SCAM and GFP. Bar, 4 μm. Quantification of the data is provided in **(F)**. ^***^p ≤ 0.001, unpaired *t*-test.

To study the role of S-SCAM during synapse formation, we performed a shRNAi-mediated knockdown of all three S-SCAM isoforms in cultured rat hippocampal neurons. We expressed shRNAs globally in these cultures using lentiviral transduction and quantified endogenous S-SCAM protein levels by Western blotting and immunofluorescence intensity (Figure 1). When shRNA targeting S-SCAM mRNA called Oligo#2 (Stan et al., 2010; for further description, see section Materials and methods) was expressed from DIV2 to DIV9, it specifically reduced S-SCAM protein levels to about 35% (Figures 1C, D) compared to a mismatched control shRNA (control RNAi). By determining immunofluorescence intensity, S-SCAM RNAi effectively reduced S-SCAM levels to about 40% in dendrites (Figures 1E, F).

**Figure 2:**
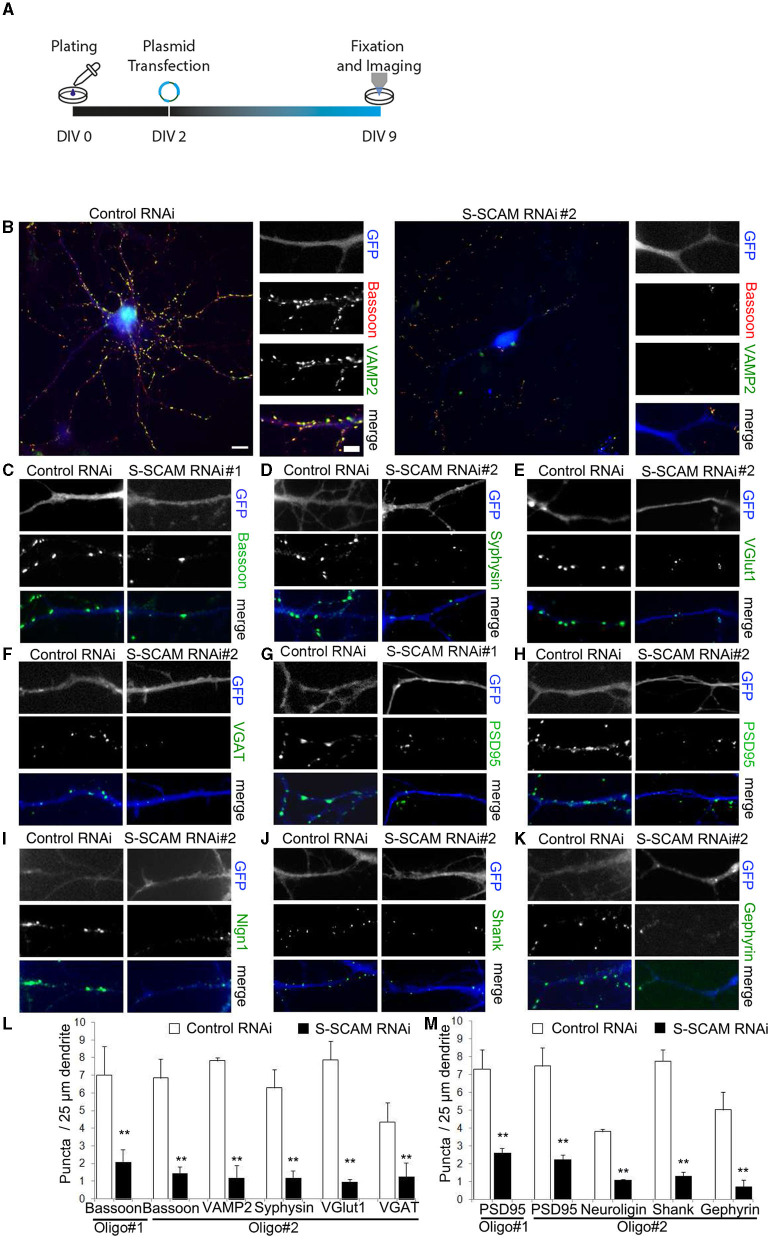
Loss of function of S-SCAM causes a severe reduction of synapse formation. **(A)** Schematic diagram of the experimental timeline. **(B–K)** Representative images of hippocampal neurons that were transfected with S-SCAM knockdown or control vectors and analyzed by immunofluorescence with antibodies to the presynaptic proteins Bassoon, VAMP2, Synaptophysin, VGlut1, VGAT, and the post-synaptic proteins PSD95, Neuroligin-1/2/3/4, pan-Shank, and Gephyrin. Bar, 10 μm, bar in zoom, 3 μm. **(L, M)** Summary graphs of the effect of S-SCAM knockdown on the density of presynaptic **(L)** and post-synaptic **(M)** markers. A 100–155 puncta (10–15 cells) were quantified. *N* = 3 independent culture experiments. **p < 0.01, two-tailed unpaired *t*-test.

To test whether knockdown of S-SCAM alters synapse formation in neurons, we sparsely transfected cultured hippocampal neurons with shRNA-expressing vectors at DIV2. In this approach, we used two different shRNA sequences previously used to knock down S-SCAM, termed Oligo#1 (Danielson et al., 2012) and Oligo#2 (Stan et al., 2010; for further description, see Section Materials and Methods). We immunostained transfected neurons at DIV9 for the presynaptic marker proteins Bassoon, VAMP2, Synaptophysin, VGlut1, or VGAT and quantified the density of immunofluorescence puncta on dendrites of the transfected neurons (Figure 2). We observed a significant reduction in the dendritic puncta density of all tested presynaptic proteins between 71 and 83% when compared to controls (Oligo#1: Bassoon 71 ± 3.3%; Oligo#2: Bassoon 79.8 ± 2.6%, VAMP2 80 ± 3.5%, Synaptophysin 78 ± 2%, VGlut1 83 ± 1.1%, VGAT 71 ± 3.2%). Similar results were obtained when we transduced hippocampal neurons with lentiviruses expressing shRNAs targeting S-SCAM mRNA at DIV2 and analyzed the Bassoon puncta density at DIV9 (Supplementary Figures S1A–C). Consistent with the reduction in the puncta density of presynaptic proteins, S-SCAM RNAi transfection greatly reduced the number of puncta for post-synaptic markers PSD95, Neuroligin, Shank, and Gephyrin (Figure 2; Oligo#1: PSD95 65 ± 3.7%; Oligo#2: PSD95#2 70 ± 1.7%, Neuroligin 71 ± 0.5%, Shank: 83 ± 6%, Gephyrin: 86 ± 5%) in dendrites. In addition, the number of sites double-positive for Bassoon and PSD95 was significantly reduced upon knockdown (Supplementary Figures S1D–F). Furthermore, the percentage of VAMP2 puncta positive for PSD95 was significantly reduced (Supplementary Figures S1G, H). Note that the number of VAMP2 puncta is strongly reduced upon knockdown (Figures 2B, L). Of the remaining VAMP2 puncta, only a fraction is aligned with the post-synaptic marker PSD95, a marker for excitatory post-synaptic sites. This indicates that the few VAMP2 puncta remaining upon S-SCAM knockdown are either located at inhibitory synapses or at orphan presynaptic sites. Together, these data suggest that S-SCAM plays an essential role during the *de novo* formation of excitatory and inhibitory synapses in developing neurons.

### Each S-SCAM isoform rescues the reduction in synapse density independently

**Figure 3:**
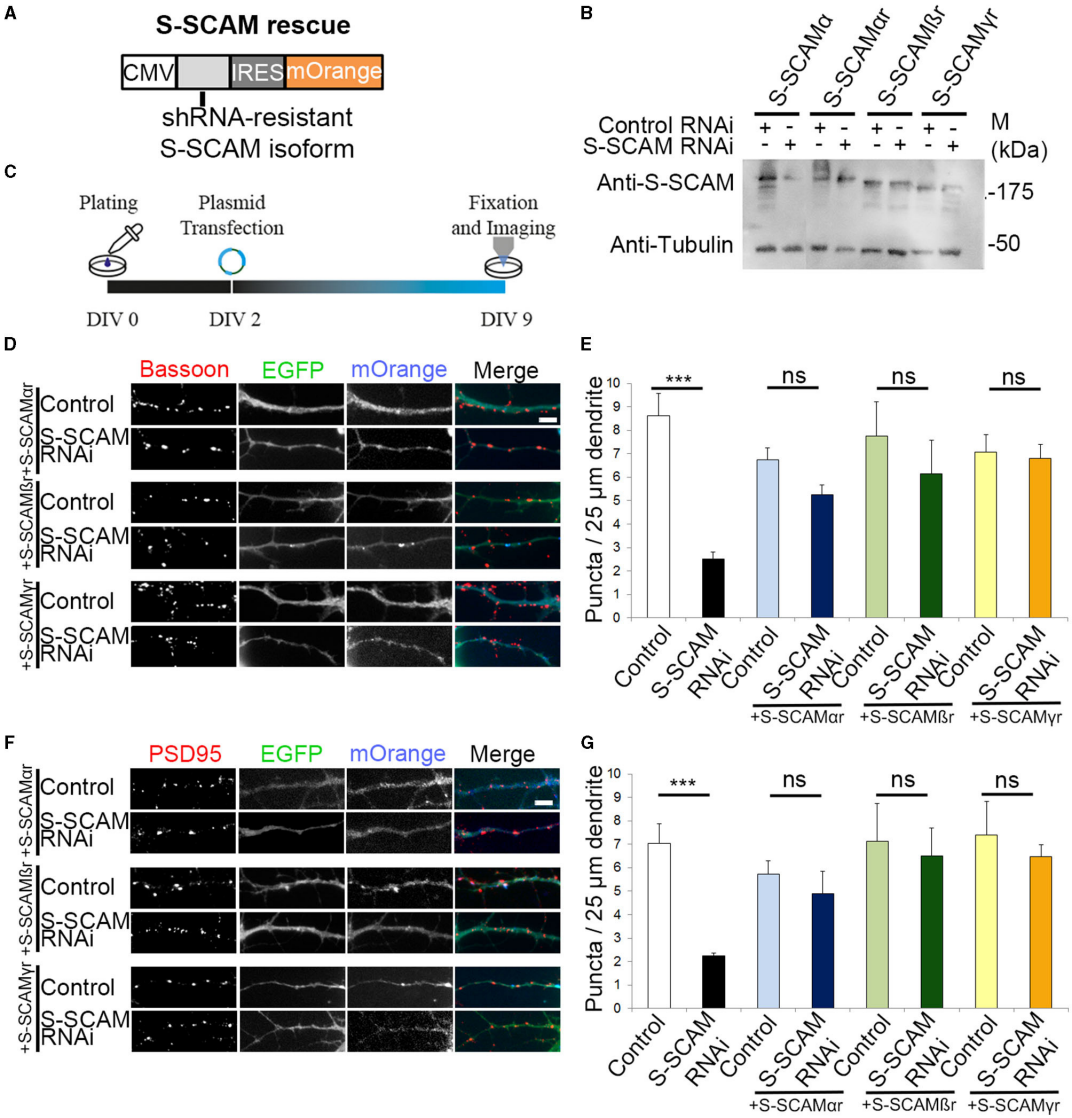
Reversal of synapse loss by overexpression of S-SCAMα, -β, or -γ in S-SCAM knockdown neurons. **(A)** Design of expression vectors for overexpression of S-SCAMα, -β, or -γ in S-SCAM knockdown neurons. CMV, cytomegalovirus promotor; IRES, internal ribosome entry sequence. **(B)** Verification of S-SCAM rescue vectors. HEK293 cells were transfected with S-SCAM RNAi or control plasmids and cotransfected with either S-SCAMα or S-SCAMα, -β, or -γ rescue vectors (S-SCAMαr, S-SCAMβr, and S-SCAMγr) containing silent mutations. Anti-tubulin blot is shown as a loading control. **(C)** Schematic diagram of the experimental timeline. **(D–G)** Rescue experiments with S-SCAMαr, -βr, and -γr. Representative images of S-SCAM RNAi or control neurons cotransfected with S-SCAMαr, S-SCAMβr, or S-SCAMγr stained for Bassoon **(D)** or PSD95 **(F)**. Bar, 4 μm. Quantification of the rescue experiments for Bassoon **(E)** and PSD95 **(G)** dendritic puncta density. A 85–125 puncta (10–12 cells) were quantified. *N* = 3 independent culture experiments. ***p < 0.001, ANOVA Tukey's test.

After confirming that the two shRNAs have similar effects on Bassoon and PSD95, we used Oligo#2 for all subsequent experiments. We next confirmed the specificity of the S-SCAM RNAi effect. We performed “rescue” experiments with S-SCAM RNAi-resistant S-SCAMα, -β, or -γ isoforms (designated S-SCAMαr, -βr, or -γr). For this purpose, we generated expression vectors that contain the S-SCAM RNAi-resistant isoforms followed by an internal ribosomal entry site (IRES) and mOrange2 for visualization of transfected neurons (Figure 3A). S-SCAM knockdown resistance was confirmed in HEK293 cells by Western blotting (Figure 3B). We then coexpressed the S-SCAM RNAi plasmid with S-SCAMαr, -βr, or -γr constructs in immature hippocampal cultures by transfecting on DIV2, and immunostained for the presynaptic protein Bassoon or for the post-synaptic marker PSD95 at DIV9 (Figure 3C). Restoring S-SCAM protein levels with either S-SCAMα, -β, or the shortest isoform, S-SCAMγ, during early synaptogenesis rescued the reduction in the dendritic puncta density of Bassoon and PSD95 to normal levels (Figures 3D–G). Thus, first, the effect of S-SCAM RNAi on synapse formation was specifically related to the loss of S-SCAM proteins and not due to the off-target effects of the shRNAs. Second, both shorter S-SCAM isoforms, missing the PDZ0 and parts of the GK domain, are able to compensate for the defect in synapse formation. This indicates that an incomplete GK domain, the WW domain, and PDZ1-5 of S-SCAM are sufficient for their function in synaptogenesis.

### S-SCAM is necessary for proper dendritic arborization and a normal number of synapses in advanced cultures

**Figure 4:**
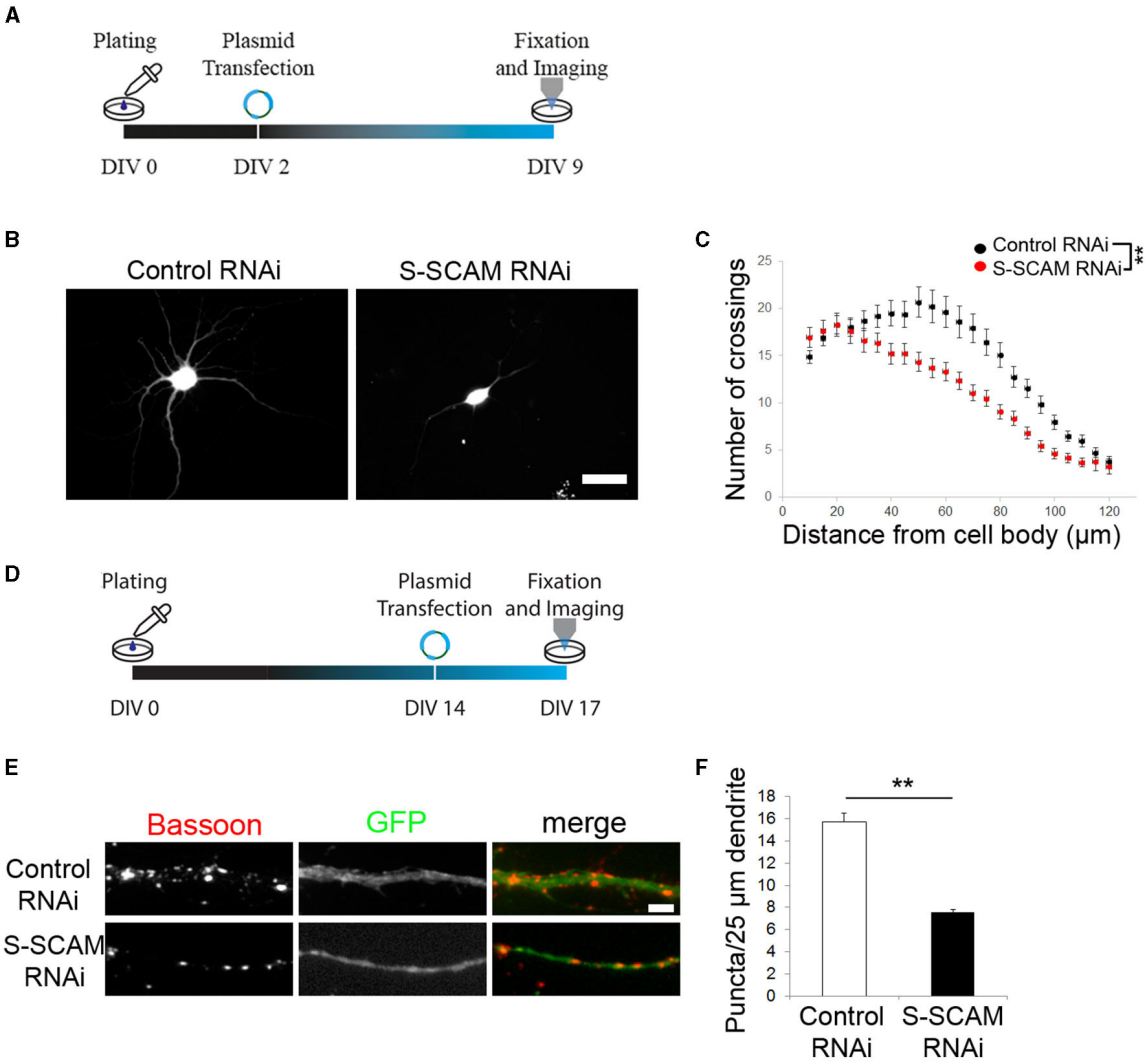
Loss of function of S-SCAM affects neuronal morphology and synaptic maintenance. **(A, D)** Schematic diagram of the experimental timeline. **(B, C)** Sholl analysis revealed a loss of neuronal processes upon S-SCAM knockdown. One-way ANOVA, **P < 0.01. **(E, F)** Neurons in older cultures (DIV14) were transfected with S-SCAM knockdown or control constructs and immunostained for Bassoon. Quantification shows a reduction in Bassoon puncta density, control RNAi, *n* = 96; S-SCAM RNAi, *n* = 112. *N* = 3 independent culture experiments; **p < 0.01, two-tailed unpaired *t*-test.

Given the dramatic reduction in synaptic markers upon S-SCAM knockdown, we assessed the corresponding morphological changes in more detail, namely the effect of S-SCAM downregulation on dendritic complexity and synapse number in advanced cultures. Transfection of neurons with shRNA plasmids was carried out at DIV2, and the signal of eGFP expression in transfected neurons allowed imaging of neuronal morphology at DIV9/10 (Figures 4A–C). Morphometric analysis revealed that the reduction of S-SCAM protein levels caused a reduced dendritic arborization complexity. The complexity of the dendritic tree was assessed using Sholl analysis, and it revealed that neurons transfected with S-SCAM RNAi vectors showed a significantly reduced number of crossings over the entire measured distance (Figure 4C).

To rule out that the striking changes in neuronal morphology were the consequence of KD-induced cell death, we tested neurons transfected on DIV2 for apoptosis at DIV10 (8 days after transfection) and quantified the percentage of viable neurons. The percentage of dying neurons did not significantly differ between S-SCAM RNAi and control neurons (control 5%, S-SCAM RNAi 6.3%; Supplementary Figure S2). In addition, we tested neurons transduced by lentiviral vectors on DIV2 for apoptotic cells on DIV10. Despite screening entire coverslips, we did not observe any apoptotic cells in control neurons transduced with GFP-expressing lentivirus or neurons transduced with S-SCAM shRNA lentivirus (Supplementary Figure S2). These results substantiate that the observed phenotype after knocking down S-SCAM is indeed due to the absence of the protein and not caused by cell death.

In addition, we asked whether S-SCAM knockdown also affects synapses in mature cultures. We transfected cultured hippocampal neurons with shRNA vectors at DIV14 and immunostained for Bassoon at DIV17 (Figures 4D–F). The dendritic Bassoon puncta density was reduced by 50% 3 days after S-SCAM RNAi vector transfection (Figures 4E, F), demonstrating that S-SCAM protein is necessary for maintaining a normal number of synapses in mature cultures. In summary, these results show the importance of S-SCAM protein for distinct developmental characteristics of hippocampal neurons, such as neuronal and synapse morphology, as well as the stability of synapses.

### Loss of function of S-SCAM impairs excitatory synaptic transmission

**Figure 5:**
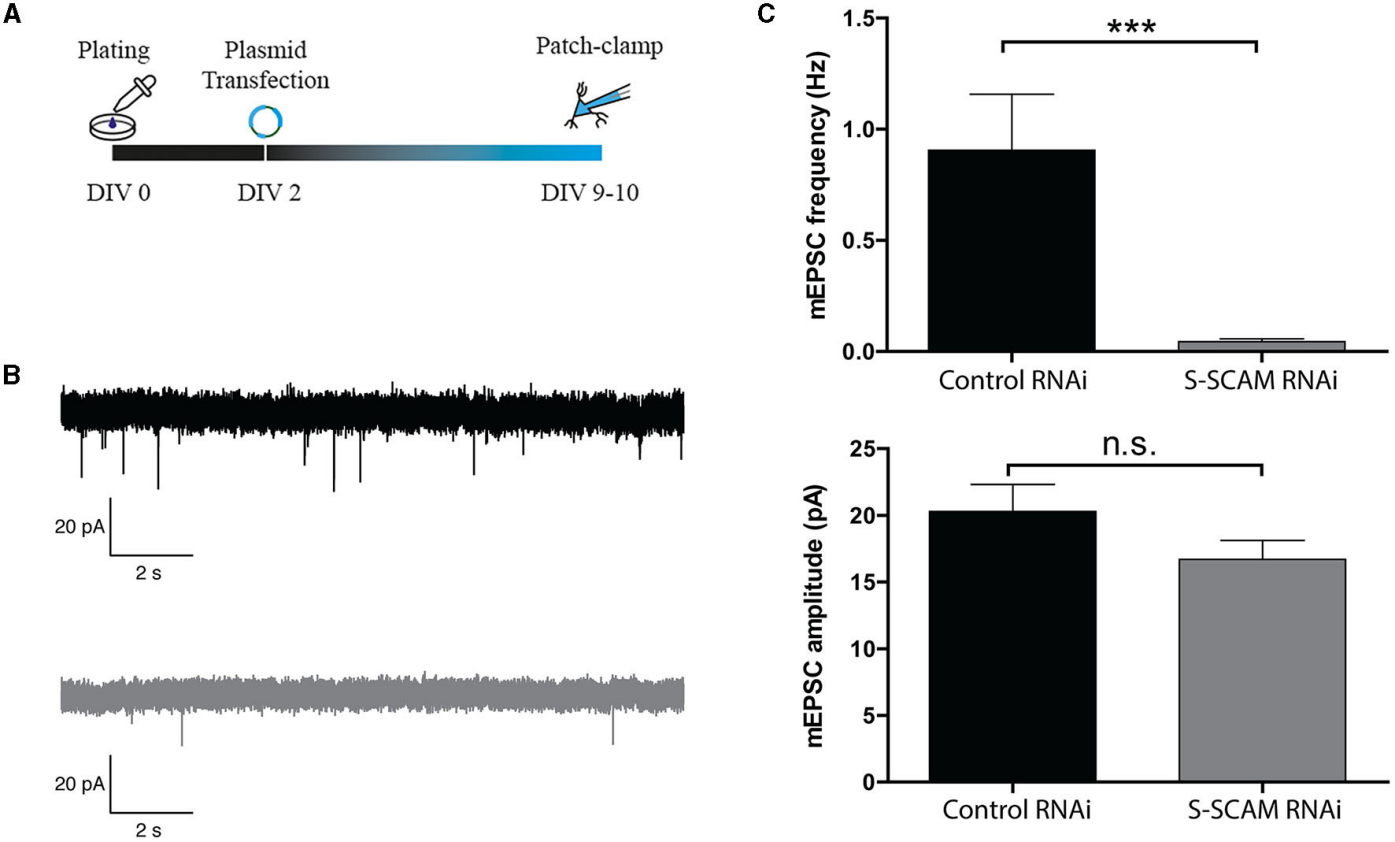
S-SCAM knockdown reduces spontaneous synaptic activity in conventional hippocampal cultures via a post-synaptic effect. **(A)** Schematic diagram of the experimental timeline. **(B)** Example traces of mEPSC events in DIV9–10 neurons infected with control RNAi (black) or S-SCAM RNAi (gray). **(C)** S-SCAM RNAi reduces mEPSC frequency, whereas the amplitude of mEPSC events between S-SCAM knockdown and control neurons is unchanged. *n* = 13 for S-SCAM RNAi and control neurons. *N* = 4 independent culture experiments. ***p < 0.001, Mann–Whitney *U* test.

To determine whether the changes in morphological synapse numbers reflect changes in the number of functional synapses per neuron, we examined synaptic transmission in cultured hippocampal neurons that were sparsely transfected with S-SCAM RNAi or control vectors on DIV2. Since it is known that S-SCAM can also localize to presynaptic compartments (Mok et al., 2002), this approach had the advantage of excluding any potential presynaptic effect of S-SCAM knockdown. Low transfection efficiency ensured that the majority of analyzed synaptic inputs originated presynaptically from untransfected S-SCAM RNAi neurons, and thus since the neurons patched were positive for transfection, only post-synaptic effects were detectable. At DIV9, the mEPSC frequency was clearly reduced following the knockdown of S-SCAM when compared with control neurons (Figures 5A–C). In contrast, the mEPSC amplitude following S-SCAM RNAi was unchanged (Figures 5B, C). This indicates that most synapses are lost, but the few remaining synapses are functional.

**Figure 6:**
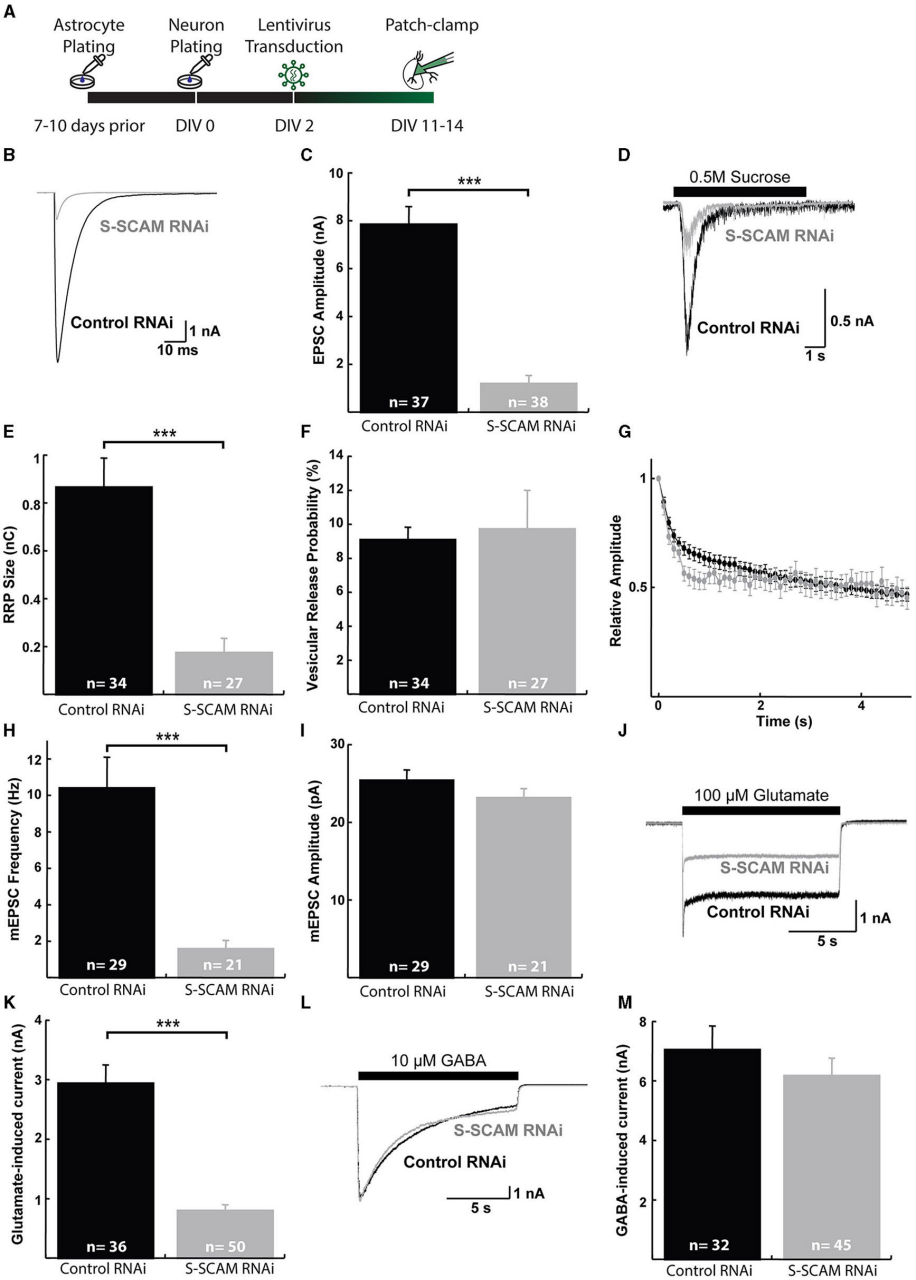
S-SCAM knockdown reduces spontaneous and evoked excitatory synaptic activity in autaptic hippocampal micro-island cultures. **(A)** Schematic diagram of the experimental timeline. **(B)** Example traces of evoked EPSC responses. **(C)** Mean EPSC amplitude measured in neurons after lentivirus expression of the control RNAi and SSCAM knockdown constructs. **(D)** Example traces of current responses induced by the application of 0.5 M sucrose solution. **(E)** Mean RRP sizes as estimated by the charge integral measured inward currents during the application of 0.5 M sucrose solution. **(F)** Calculated mean vesicular release probabilities calculated by dividing the charge transfer during a single EPSC by the charge transfer measured during RRP release. **(G)** Evoked EPSC depression during 10 Hz stimulation train. Data were normalized to the first response in the train. **(H)** Mean mEPSC frequencies. **(I)** Mean mEPSC amplitudes. **(J)** Example traces of current responses induced by the application of 100 μM glutamate solution. **(K)** Mean peak amplitudes of responses to exogenous 100 μM glutamate. **(L)** Example traces of current responses induced by the application of 10 μM GABA solution. **(M)** Mean peak amplitudes of responses to exogenous 10 μM GABA. ***p < 0.001, two-tailed unpaired *t*-test; n, neurons recorded.

We also evaluated the effect of the reduction of S-SCAM in evoked synaptic transmission by using mouse autaptic hippocampal cultures that were transduced with S-SCAM RNAi or control lentiviruses on DIV2 and analyzed between DIV11 and DIV14 (Figure 6A). Western blotting of mouse cultured neurons confirmed the knockdown of S-SCAM protein (Supplementary Figure S3). As expected, the evoked EPSC amplitude and mEPSC frequency recorded from glutamatergic autaptic neurons were markedly reduced upon knockdown of S-SCAM when compared with control neurons (Figures 6B, C, H). In contrast, the mEPSC amplitude following S-SCAM RNAi was unchanged (Figure 6I). Furthermore, the readily releasable pool in the S-SCAM RNAi neurons, measured by 0.5 M hypertonic sucrose solution, dramatically decreased, corresponding to the reduction in EPSC amplitudes (Figures 6D–F). Based on the unchanged vesicular release probability and mEPSC amplitude, it can be predicted that the reduction in synaptic strength reflects a decrease in the number of functional excitatory synapses. In particular, the decrease in current induced by exogenous application of glutamate, but not GABA, onto the S-SCAM RNAi neuron supports this assumption (Figures 6J–M). Note that the recorded glutamatergic autaptic neurons do not have GABAergic synapses. The response to exogenous GABA reveals that they have extra-synaptic GABA receptors in their membranes and are vital. Furthermore, since mEPSC amplitudes and relative amplitudes of evoked responses to 10-Hz trains are also unchanged (Figures 6G, I), the active synapses in autaptic cultured neurons remaining upon S-SCAM knockdown seem functionally normal. Together, these results show that S-SCAM knockdown leads to a massive reduction in the number of synapses per neuron, while the remaining synapses on these neurons display normal synaptic strength.

### Profiling gene expression in primary cultured hippocampal neurons upon S-SCAM downregulation

**Figure 7:**
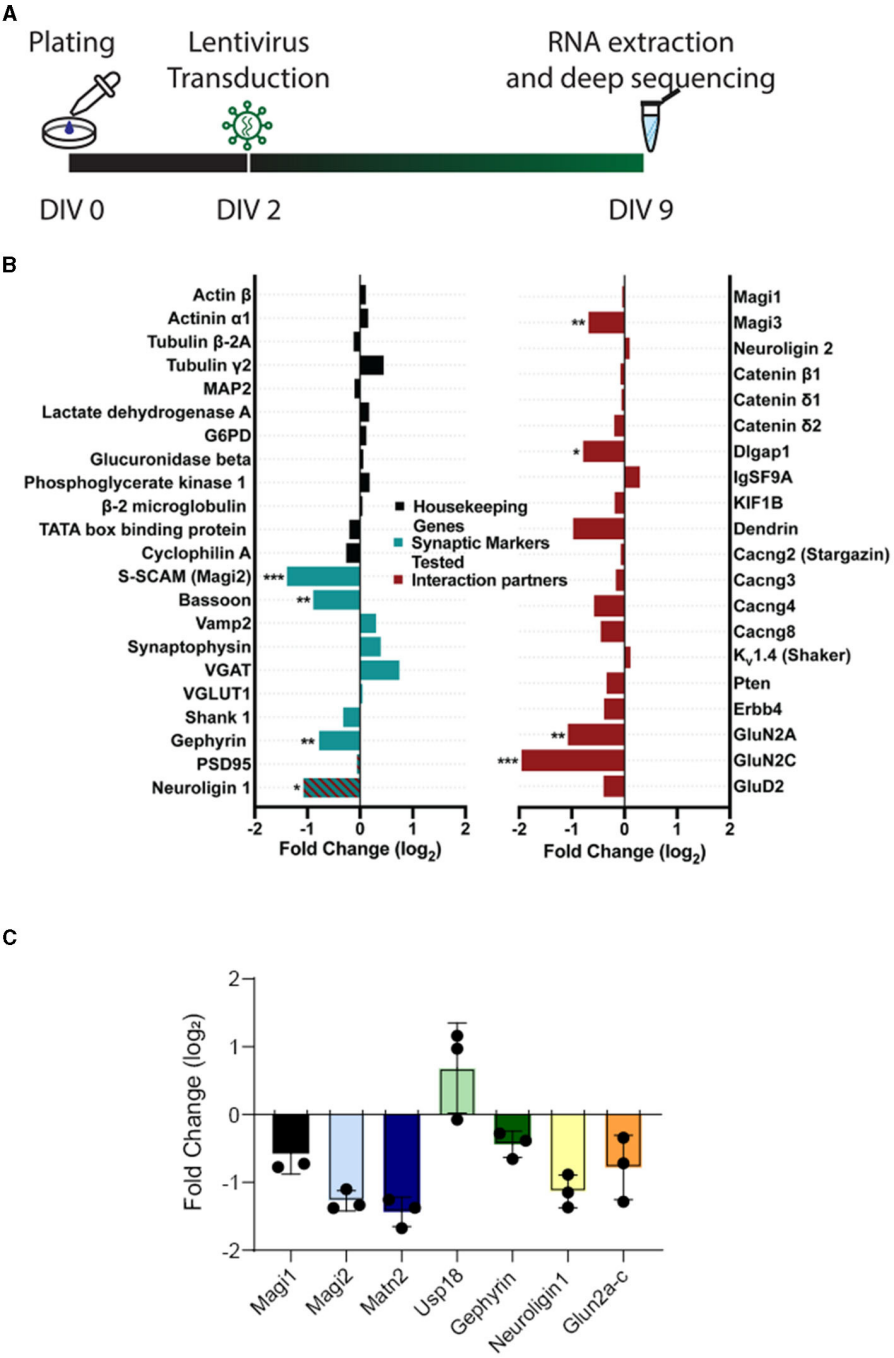
Expression of housekeeping genes is unchanged, whereas expression of signaling proteins is altered upon S-SCAM knockdown. **(A)** Schematic diagram of the experimental timeline. **(B)** Selected results of the deep sequencing analysis of neuronal cultures treated with S-SCAM shRNA or control shRNA. The graph shows the log_2_-fold change in the shRNA-treated cultures compared to control cultures of housekeeping genes (black), synaptic marker proteins tested by immunofluorescence in our study (green), and known S-SCAM interaction partners (red). *p < 0.05; **p < 0.01; ***p < 0.001, false discovery rate. *N* = 3 independent experiments. **(C)** RT-PCR of selected RNAs from Figure 7B. RNA was isolated from *n* = 3 cultures transduced with SCAM-RNAi or EGFP. Ct values were normalized to α-tubulin (δct). RNAi values were normalized to EGFP (δδct), and the fold change (FC) was calculated. Values are plotted as log_2_FC.

Given the dramatic effects of S-SCAM triple isoform knockdown on synapse formation and neuronal morphology, we examined gene expression in cultures transduced with control or knockdown shRNA. We isolated RNA from DIV9 primary cultured hippocampal neurons transduced with S-SCAM RNAi or control viruses at DIV2. More than 30 million high-quality reads were obtained in each sample. Read alignment was performed using STAR v2.3.0 to the Rattus norvegicus v5 reference genome. Deep sequencing revealed that S-SCAM was reduced to 38% of control (−1.39 log_2_ fold change), which corresponds well with the ca. 60% reduction in S-SCAM protein estimated by Western blotting and immunofluorescence (see Figure 1). RNA levels of housekeeping genes including lactate dehydrogenase, GAPDH, actin, and neurofilament light and heavy chains were not changed (Figure 7; Supplementary Data S1).

Deep sequencing also revealed changes in the expression of synaptic markers previously tested in this article (Figure 2), namely a decrease in Bassoon, Gephyrin, and neuroligin-1 (Figure 7B). Furthermore, some of the known interaction partners of S-SCAM showed reduced expression, including neuroligin-1, Magi3, Dlgap1, GluN2A, and GluN2C. Overall, 544 genes were differentially expressed by the reduction in S-SCAM levels (Figure 7B; Supplementary Data S1). A quantitative real-time PCR (qPCR) analysis of selected mRNAs isolated from DIV9 neurons transduced with S-SCAM RNAi or control viruses at DIV2 confirmed the trends revealed by deep sequencing (Figure 7C). Moreover, similar results were obtained when cultures treated over distinct time windows (i.e., transduction at DIV2 followed by analysis at DIV16 and transduction at DIV14 followed by analysis at DIV17) were analyzed by qPCR (Supplementary Figure S4).

**Figure 8:**
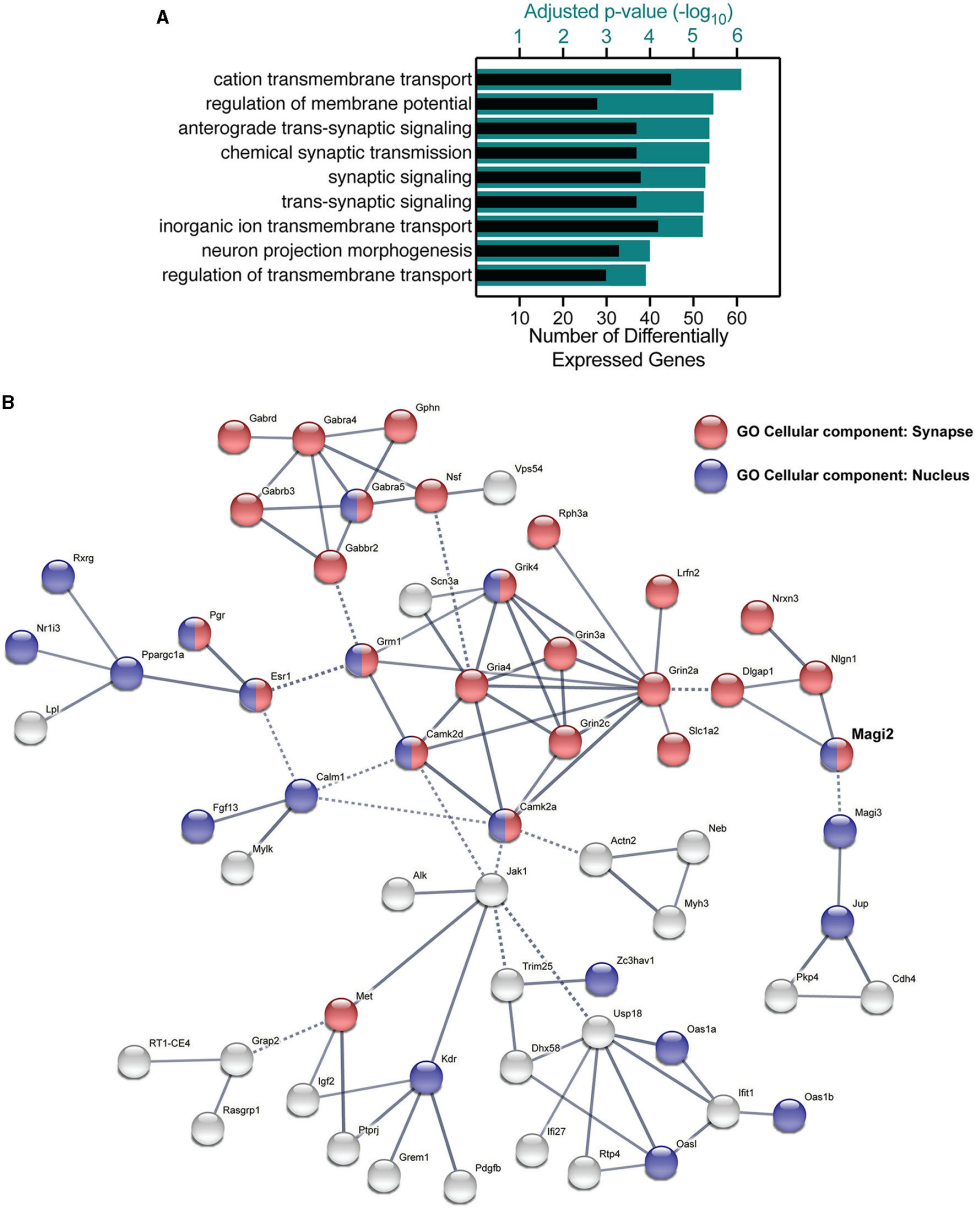
Gene ontology and functional clustering of differentially expressed genes by the knockdown of S-SCAM. **(A)** The GO terms for biological processes with the lowest adjusted *p*-value (teal bars) by using the g:Profiler tool, and the number of differentially expressed genes in each category (black bars). **(B)** STRING network analysis of differentially expressed genes functionally connected to S-SCAM (MAGI-2, in bold). Blue nodes represent protein products present in the nucleus, red nodes indicate synaptic proteins, and green nodes indicate proteins present in cell junctions but not proven to be synaptic. Nodes that have more than one color are proteins that localize to both subcellular compartments, whereas gray nodes indicate proteins that localize to none of these three compartments. Edges represented by full lines are functional associations within a cluster, whereas edges represented by dotted lines are connections between clusters.

A gene enrichment analysis using g:Profiler (Reimand et al., 2007) yielded several significant biological process terms in Gene Ontology (Ashburner et al., 2000) related to synaptic transmission and neuronal functioning (Figure 8A), namely, anterograde trans-synaptic signaling, chemical synaptic signaling, synaptic signaling, and trans-synaptic signaling figured in the most significantly changed terms. These terms had adjusted *p*-values between –log_10_ 5.2 and –log_10_ 5.3 and included 38–39 genes. The most significant term in our analysis was cation transmembrane transport, including 46 genes whose expression was modified by the knockdown of S-SCAM. Of those 46, 13 included genes also involved in synaptic transmission (e.g., GluN2A, GluN2C, and GluN3A). Other enriched terms involved with neuronal function included terms related to the regulation of membrane potential and neuron projection morphogenesis. Similar results were observed when analyzing molecular function Gene Ontology terms using the GOrilla web application (Supplementary Figure S5; Eden et al., 2009).

We investigated the interaction between differentially expressed gene products using STRING (Szklarczyk et al., 2015). Most of the nodes observed in the functional network of S-SCAM were gene products present in the nucleus and/or synapse (Figure 8B). Notably, one cluster in the network was formed by glutamate receptor subunits (Grik4, Grin3a, Grin2a, Grin2c, Gria4, and Grm1), calmodulin kinase subunits (Camk2a and Camk2d), a glutamate transporter (Slc1a2), a synaptic adhesion-like molecule (Lrfn2), a regulator of exocytosis (Rph3a), and a sodium channel subunit (Scn3a). One other cluster included GABA receptor subunits (Gabra4, Gabra5, Gabrb3, Gabbr2, and Gabrd), Gephyrin (Gphn), a vesicle-fusing ATPase (Nsf), as well as a Golgi-associated protein (Vps54). These clusters give us a hint of how S-SCAM is affecting excitatory and inhibitory synapses, respectively. What is still untested is whether the knockdown of S-SCAM affects synapse formation *in vivo*, which we decided to verify by analyzing spine density.

### S-SCAM is necessary for spinogenesis *in vitro* and *in vivo*

**Figure 9:**
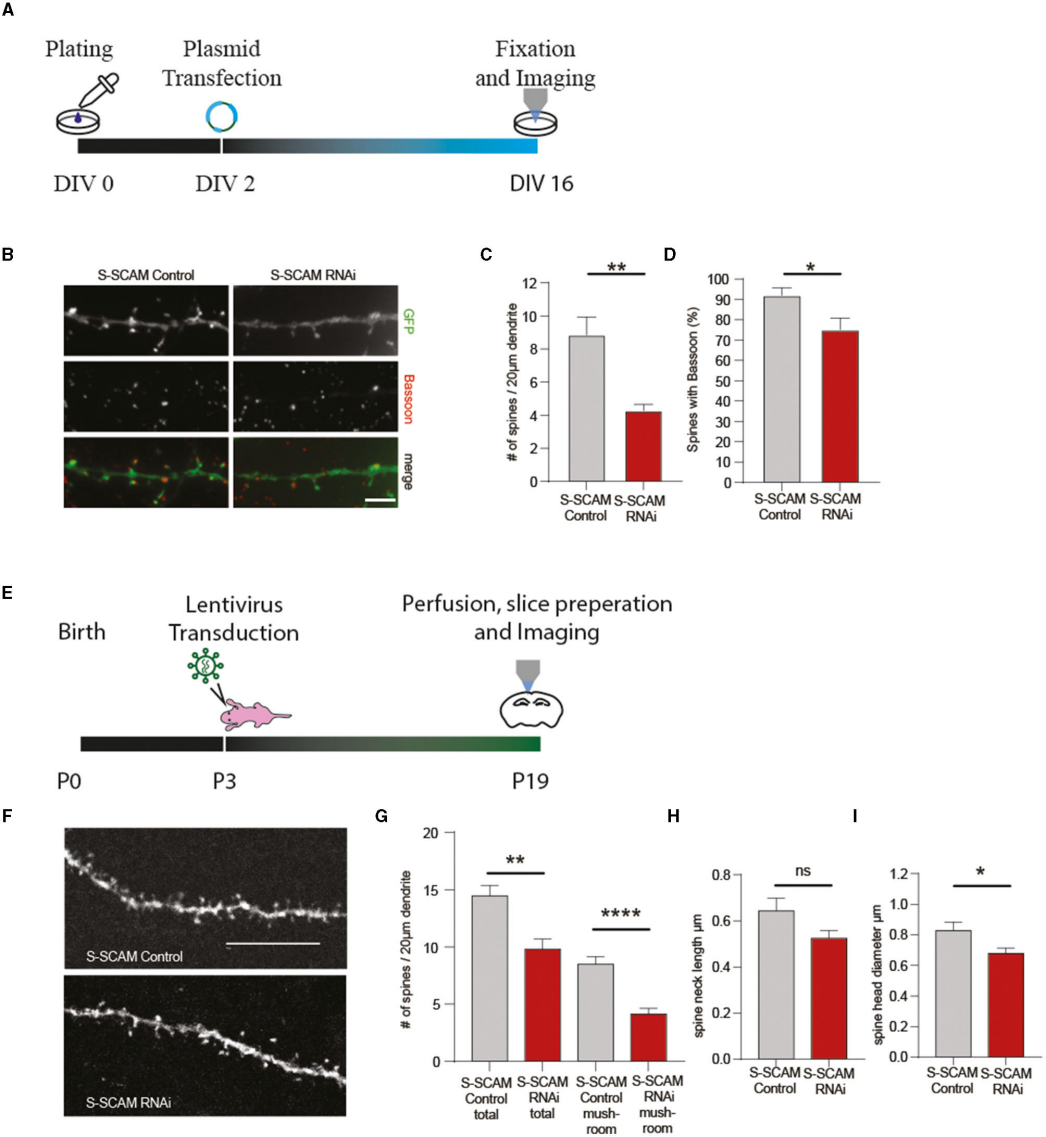
S-SCAM is involved in spine formation *in vitro* and *in vivo*. **(A, E)** Schematic diagram of the experimental timeline. **(B)** Images of dendrites of hippocampal neurons transfected with S-SCAM knockdown or control constructs and immunostained for Bassoon. Bar, 5 μm. **(C)** Quantification of the effect of S-SCAM knockdown on dendritic spine density, control RNAi, *n* = 124; S-SCAM RNAi, *n* = 134. *N* = 3 independent culture experiments; **p < 0.01, unpaired *t*-test. **(D)** Quantification of the effect of S-SCAM knockdown on the percentage of spines with Bassoon staining; control RNAi, *n* = 155; S-SCAM RNAi, *n* = 136. *N* = 3 independent culture experiments. *p < 0.05, unpaired *t*-test. **(F–I)** Lentiviral particles expressing shRNAs against S-SCAM or control shRNAs were injected into the cortex of P3 rat brains **(E)**. Brains were analyzed at P19 by immunohistochemistry with an antibody against GFP, and confocal images of dendrites of cortical neurons were analyzed **(F)**. Bar, 10 μm. Quantification of the effect of S-SCAM knockdown on dendritic spine density, number of mature mushroom spines **(G)**, average spine neck length **(H)**, and spine head diameter **(I)**; control RNAi, *n* = 13 neurites S-SCAM RNAi, *n* = 16 neurites Mean ± SEM (*N* = 2 control RNAi-injected animals; *N* = 4 S-SCAM RNAi-injected animals); **p < 0.01, ^****^p ≤ 0.001, unpaired *t*-test.

To address whether the observed effect in synaptogenesis is accompanied by reduced spine formation, we transfected hippocampal neurons with shRNA plasmids at DIV2 and labeled neurons with an antibody against GFP and Bassoon at DIV9. Confocal microscopy revealed that the number of dendritic spines was significantly reduced after knocking down S-SCAM (Figures 9A–C). Additionally, the number of spines positive for presynaptic terminals (Bassoon signal) was reduced (Figure 9D), demonstrating the importance of S-SCAM protein for spinogenesis and synaptogenesis *in vitro*. To validate the results obtained in cultured neuronal cells and to examine if the knockdown of S-SCAM also interferes with the formation of spines within a normal functional network, we aimed to test the effects of the knockdown shRNAs *in vivo*. To this end, we verified by Western blotting and qPCR that the shRNA knocks down S-SCAM in rat cultured cortical neurons (Supplementary Figure S6). We then injected lentiviral particles expressing a mismatch or the respective shRNA targeting S-SCAM into the cerebral cortex of 3-day-old rats. The brains were collected and analyzed by immunohistochemistry at P19. Viral particles were spread out after injection into the cortex and sparsely labeled neurons in all cortical layers. To substantiate the finding of decreased spinogenesis *in vitro*, we counted spines in this more native environment. In accordance with our culture experiments, we detected a significant decrease in spine number after the knockdown of S-SCAM (Figure 9). These results demonstrate that decreasing the levels of S-SCAM in the first 3 weeks after birth results in a dramatic alteration of neuronal spine formation despite their contact with a normal microenvironment.

## Discussion

In this study, we demonstrate that reducing S-SCAM levels leads to drastic effects on three different levels: (1) expression of neuronal proteins is strongly changed, (2) neuronal dendrites become underdeveloped, and (3) synapses are lost both in neuronal cultures and *in vivo*. We also show that, despite these drastic changes, neuronal integrity and survival are unaffected.

### Role of S-SCAM in developing cultured neurons

S-SCAM is thought to regulate synapse composition. This was initially predicted based on the large number of binding partners for this post-synaptic scaffolding protein (reviewed in Nagashima et al., 2015). Later, it turned out that triple isoform knockdown of all three S-SCAM isoforms in developing cultures reduced the capacity of post-synaptic neuroligin-1 to recruit the synaptic vesicle marker VAMP-2 in the axons of young neurons (Stan et al., 2010), but whether or not active zones formed under these conditions remained untested. A different knockdown study, performed in mature culture stages, found that the number of clusters of certain synaptic proteins was reduced in cultured neurons after reducing S-SCAM levels (Danielson et al., 2012). Based on this study, S-SCAM was proposed to be critically involved in recruiting AMPA-type glutamate receptors. The same knockdown approach revealed a reduction in the clusters of several proteins of inhibitory synapses in advanced cultures (Shin et al., 2020). This suggested that S-SCAM is required for the maintenance of a normal protein composition at excitatory and inhibitory synapses. In this study, we corroborate these studies and show, in addition, that all pre- and post-synaptic protein classes tested here failed to cluster upon S-SCAM triple isoform knockdown in young neuronal cultures (DIV9), suggesting that S-SCAM is required for synapse assembly.

Upon knockdown after DIV14, Danielson et al. (2012) found reduced clustering of Bassoon and PSD95; Shin et al. (2020) found reduced clustering of VGAT, NL2, and Gephyrin. At this developmental stage (DIV14–DIV17), the primary effect of the knockdown is likely on synapse maintenance, although one cannot exclude in these studies or in our study that the reduction in the number of synapses at this developmental stage is, in addition, caused by inhibiting ongoing synapse formation. Our data support and extend these studies by revealing that pre- and post-synaptic proteins, including SV proteins and scaffolding proteins, fail to accumulate at synapses when S-SCAM is knocked down in developing cultures. In these cultures, functional synapses become detectable by electrophysiology after DIV5 (Mozhayeva et al., 2002; Wittenmayer et al., 2009). Early knockdown of S-SCAM (to ca. 40%) led to a dramatic reduction in the clustering of pre- and post-synaptic proteins by DIV9, i.e., during the onset of synaptogenesis. This suggests that synapses do not assemble, although we cannot exclude a scenario in which synapses form and disassemble rapidly before DIV9. In any case, the fact that Bassoon, Synaptophysin, VAMP2, VGlut1, Shanks, PSD95, and Neuroligins, as well as VGAT and Gephyrin, all fail to cluster before DIV9 strongly suggests that S-SCAM is required for synaptogenesis in this time window.

This is supported by our electrophysiological analysis, which reveals strong effects of the knockdown on synaptic transmission, as evidenced by at least 80% reductions in mini frequency observed in both conventional cultures and autaptic micro-island cultures. Reduced mini-frequency *per se* can be caused by a reduction in the number of synapses or the release probability observed at each synapse. In autaptic cultures, the readily releasable pool of vesicles was reduced equally strongly as the mini frequency, while the release probability was unchanged. This indicates that the knockdown of S-SCAM dramatically reduced the number of functional synapses.

Our study also shows that the few synapses that form despite the knockdown of S-SCAM are functionally normal, as observed by normal EPSC amplitude, plasticity, and vesicular release probability. These synapses may represent a pool of synapses that is independent of S-SCAM or at least require smaller concentrations of S-SCAM than others. The unchanged EPSC amplitude indicates that the quantal size of these synapses is unaffected, i.e., there is no change in the presence and density of post-synaptic receptors as well as the neurotransmitter content of the synaptic vesicles. The absence of change in plasticity and vesicular release probability indicates that there is no obvious change in the vesicular release machinery of these synapses. This contrasts with a previous report (Danielson et al., 2012), which shows a slight reduction in the amplitude of electrophysiological responses. Another difference between this report and ours is that we observe a 95% reduction in mEPSC frequency in comparison to 56% of Danielson et al. (2012). These differences may reflect the different culture stages tested and suggest that at later culture stages, S-SCAM may have a primary role in receptor recruitment while its role in synapse formation or maintenance can be compensated for by other proteins. Alternatively, different knockdown efficacies of the shRNAs or different specificities for S-SCAM isoforms may contribute to these differences.

In addition to the reduction of mEPSC frequency, the reduction in evoked EPSC amplitude, RRP size, and glutamate-induced currents corroborate the reduction in synapse numbers observed by immunofluorescence. Furthermore, since our cultures were sparsely transfected, we ensured that most synaptic contacts came from untransfected neurons. Even in these conditions, we observed a reduction of transmission, supporting the idea that S-SCAM is necessary post-synaptically to ensure synapse formation and/or maintenance in developing neuronal cultures. The validity of our approach was tested in three ways: first, DeepSeq analysis revealed that the expression of housekeeping genes was unaffected by the KD; second, the number of apoptotic cells was unchanged, indicating that the overall viability of the neurons was unaffected; and third, rescue experiments indicated that the KD-induced loss of synapses was indeed caused by the lack of S-SCAM since RNAi-resistant versions of S-SCAMα, -β, or -γ restored normal synapse numbers.

### Short S-SCAM isoforms

Whether or not S-SCAM-β or S-SCAM-γ can rescue synapse formation had not been known before. Iida et al. (2007) expressed all three isoforms in cultured neurons from S-SCAMα knockout mice. However, cultured neurons from these mice have a normal number of synapses. In addition, these neurons have abnormally long spines. This spine morphology phenotype could be rescued by S-SCAMα, but not by S-SCAM-β or S-SCAM-γ (Iida et al., 2007), indicating that only the longest isoform, S-SCAMα, regulates spine morphology in this context. Our data add to this picture the insight that all S-SCAM isoforms can rescue synapse formation or stability. Thus, the N-terminal PDZ domain and most of the guanylate kinase domain (which are missing in the shorter isoforms) are required for regulating spine length (Iida et al., 2007), but are not required for the synapse-forming or stabilizing action of S-SCAM. The expression of these isoforms could account for the observation that cultures from S-SCAMα knockout mice have a normal number of synapses and that morphologically normal synapses, as revealed by electron microscopy, do exist in the neocortex of S-SCAMα knockout mice (Iida et al., 2007).

### Role of S-SCAM in the brain

The role of S-SCAM in the brain has remained elusive. Knockout of the largest S-SCAM isoform did not reveal synapse assembly deficits *in vivo*, although animals died neonatally (Iida et al., 2007). Curiously, S-SCAM triple knockout mice survive for up to 3 weeks or 3 months, depending on the line, and die of renal failure (Balbas et al., 2014; Ihara et al., 2014). Thus, the role of S-SCAM for synapses in the brain has remained unclear. Increased levels of S-SCAM in excitatory neurons of transgenic mice cause a reduction in the number of spines *in vivo* (Zhang et al., 2015). Interestingly, these transgenic mice show, in addition to a reduced number of dendritic spines, enhanced basal glutamatergic synaptic transmission and schizophrenia-like endophenotypes (Zhang et al., 2015), which is consistent with the duplication of the S-SCAM gene in schizophrenia (Walsh et al., 2008). These observations make S-SCAM an important protein to study. However, whether S-SCAM is essential for synapse formation in the brain has remained elusive. In particular, a correlation between reduced S-SCAM levels and reduced synapse numbers was observed in cultures (Danielson et al., 2012; Shin et al., 2020), and this study had not been demonstrated *in vivo*. In this study, we show that S-SCAM shRNA expression in cortical neurons leads to reduced synapse numbers *in vivo*. In this experiment, shRNA-expressing neurons were surrounded by unaffected neurons and thereby exposed to the native environment of signaling molecules and factors. This experiment, therefore, establishes that the effect on synaptogenesis is cell-autonomous and not restricted to the neuronal cell culture system and reveals the importance of S-SCAM for synapse formation in the brain. Remarkably, since increased expression of S-SCAM in a mouse model of schizophrenia also decreases the number of spines (Zhang et al., 2015), our data indicate that manipulating the expression levels of S-SCAM in either direction causes a reduction in the number of spines *in vivo*.

### Mechanisms

Overall, three interconnected mechanisms could underlie the loss of synapses observed upon knockdown of S-SCAM. These include (i) failure to recruit direct and indirect binding partners of S-SCAM to synapses; (ii) failure to recruit these proteins also entails the failure to recruit the synapse-forming and synapse-stabilizing functions provided by these proteins; and (iii) aberrant gene expression and impaired synapse-to-nucleus signaling.

First, the loss of direct and indirect physical interactions of S-SCAM alone should already have significant effects on synaptic composition. For example, S-SCAM binds directly to Neuroligins and SAPAP/GKAP (Kim et al., 1997; Hirao et al., 1998; Iida et al., 2007). Both Neuroligins and SAPAP/GKAP also bind to the scaffolding proteins PSD95 and Prosaps/Shanks (Grabrucker et al., 2014; Nagashima et al., 2015; Monteiro and Feng, 2017; Bai et al., 2022), all of which fail to accumulate at synapses in the S-SCAM knockdown.

Second, failure to recruit these direct and indirect binding partners is expected to have further, secondary consequences for synapse composition and integrity because of the key functions that these molecules provide. For example, TARPS, another family of direct S-SCAM-binding partners, recruits AMPA receptors (Danielson et al., 2012), while neuroligin-1 recruits the GlunN1 NMDA receptor subunit (Chih et al., 2005; Budreck et al., 2013). Lack of recruitment of these S-SCAM-binding partners can account for the failure to recruit both families of glutamate receptors. This situation may be further exacerbated by the reduction in the expression levels of GluN2 NMDA receptor subunits observed in our DeepSeq analysis. GluN2 subunits determine biophysical NMDA receptor properties, and the GluN2A subunit is known to be incorporated into synapses at advanced maturational stages (see, e.g., McKay et al., 2018). Thus, reduced expression of GluN2 subunits may itself impair the maturation of initially formed synapses or may reflect impaired maturation. In addition to preventing GluN1 recruitment, reduced accumulation of Neuroligins is also expected to impair the recruitment of synaptic vesicles, as initially shown by the knockdown of Neuroligins by Chih et al. (2005). This is further supported by our previous observation that S-SCAM activates the synaptic vesicle recruiting potential of neuroligin-1 (Stan et al., 2010). In this scenario, loss of β-catenin, another direct interaction partner of S-SCAM, may further impair vesicle accumulation by reducing the recruitment of N-cadherin, which co-operates with S-SCAM and neuroligin-1 in inducing the accumulation of synaptic vesicles in young networks (Stan et al., 2010). Knockdown of S-SCAM also caused a reduction in the expression levels and synaptic accumulation of the presynaptic scaffold protein Bassoon. Bassoon stabilizes presynaptic terminals by reducing proteasomal degradation and autophagy (Waites et al., 2013; Okerlund et al., 2017; Gundelfinger et al., 2022). Thus, reduced expression of Bassoon and failure to recruit and anchor Bassoon at synapses may weaken synapses and significantly decrease their lifetime by causing over-activity of degradative pathways.

Overall, the failure of pre- and post-synaptic assembly can be explained by (i) reduced recruitment of the multiple interaction partners of S-SCAM and (ii) loss of the important functions of these proteins for synapse assembly. This highlights the importance of S-SCAM for synapse assembly as a key scaffolding protein. The aberrant gene expression we detected is likely to contribute to a third factor. While the expression of housekeeping genes was unchanged and a large number of synaptic proteins and S-SCAM interaction partners showed unchanged expression too, 544 genes did show up- or downregulated expression. Some of the downregulated genes, including neuroligin-1, Dlgap1 (SAPAP/GKAP), Magi3, GluN2A, and GluN2C, encode S-SCAM interaction partners. On the other hand, the expression of the majority of S-SCAM interaction partners was unaffected (e.g., axins, erbB4, β-catenin, γ-catenin, IgSF9b, PSD95, Tamalin, Stargazin, SynArfGEF, and others; see Supplementary Data S1). The downregulation of the cell adhesion molecules neuroligin-1, neurexin-3, and cadherin-4 may contribute to the failure to assemble synapses.

It is unclear whether the gene expression changes that were observed occurred as a general response to the severely reduced synaptic activity—for example, by reduced NMDA receptor-dependent fast signal-to-nucleus calcium signaling (Panayotis et al., 2015)—or perhaps were a more specific response to impaired signal-to-nucleus protein trafficking caused by the absence of certain S-SCAM-binding proteins. Prosap/Shank family proteins, which bind to S-SCAM directly and failed to cluster in the knockdown, include the isoform Prosap2/SHANK3. Prosap2/Shank3 translocates to the nucleus in an activity-dependent manner and binds to Lapser 1 and Abi-1, i.e., proteins that also undergo synapse-to-nucleus translocation (Grabrucker et al., 2014; Panayotis et al., 2015). Lapser 1, in turn, binds to the direct S-SCAM binding partner β-catenin, which may itself translocate to the nucleus to regulate gene expression (Thyssen et al., 2006; Schmeisser et al., 2009; Wisniewska, 2013). Impairment of synapse-to-nucleus signaling could also occur in the presynaptic inputs of cells with reduced S-SCAM levels. On the presynaptic side, the transcriptional co-repressor and Bassoon binding partner CtBP1 undergo activity-dependent shuttling from the active zones to the nucleus: in active neurons, CtBP1 is retained in the presynaptic compartment via an interaction with the scaffolding protein Bassoon, while reduced activity leads to the release of CtBP1 from Bassoon, followed by its nuclear translocation and action as a transcriptional repressor (Ivanova et al., 2015). Upon S-SCAM knockdown, the observed reduction in expression levels and synaptic accumulation of Bassoon in presynaptic inputs could release CtBP1 from the presynaptic compartment, allowing it to repress its target genes in afferent cells. This is consistent with our finding that GluN2A, one of the target genes of CtBP1 (Ivanova et al., 2015), was downregulated in cultures where S-SCAM was globally knocked down by viral shRNA delivery.

Thus, S-SCAM is directly associated with multiple binding partners predicted to promote synapse-to-nucleus signaling. Overall, our data indicate that S-SCAM is required for synapse assembly *in vitro* and *in vivo*. The presence of S-SCAM may promote the formation or stability of synapses in developing networks, both through the action of S-SCAM as a major scaffolding protein and by maintaining a suitable gene expression profile.

## Data availability statement

Sequence data presented in the study are deposited in the GEO repository and can be accessed via the following link: https://www.ncbi.nlm.nih.gov/geo/query/acc.cgi?acc=GSE228093.

## Ethics statement

The animal study was approved by Niedersächsisches Landesamt für Verbraucherschutz und Lebensmittelsicherheit, LAVES. The study was conducted in accordance with the local legislation and institutional requirements.

## Author contributions

Conceptualization, methodology, data curation, writing—original draft preparation, and project administration: NW, JB, JV, and TD. Validation: NW, JV, and TD. Formal analysis and writing—review and editing: NW, AP, MB, JB, SK, JR, JV, and TD. Investigation: NW, AP, MB, CL, AB, SK, JR, and JV. Resources, supervision, and funding acquisition: TD. Visualization: NW, AP, MB, CL, and JV. All authors have read and agreed to the published version of the manuscript.
