# Supplementary material for: S-SCAM is essential for synapse formation
Source: Front Cell Neurosci. 2023 Nov 16;17:1182493. doi: 10.3389/fncel.2023.1182493 (PMC10690602; doi:10.3389/fncel.2023.1182493)
Supplement: Supplementary file 1 [file Data_Sheet_1.zip › Data Sheet 1/Supplementary Data S2 - List of pPCR Primers.pdf]

S2_Data: List of Primers used.

Primer	Forward Sequence (5‘-3‘)	Reverse Sequence (5‘-3‘)
Matn2 version1	GAGGGCTTTGTCCTCGCTGA	GGGAAACTGCCAAGGAATCT
Matn2 version2	GTGCAACGAAGGATTTGCTC	CATCCATGATCCGAGGAGGC
Usp18	TGTTTGTTGGGTGACCTGGAAGGAT	TGCAGCAGATGTGGGTACAGGAGAG
Magi1	CTGACGCACAATCAAGTCGTGG	CTCTTCTTGGGAACTGGTAGCC
Magi2 version1	CGAGAGCCATTTATGAAAGTAGGC	AGACTCCATCCTCCGAAGGTGA
Magi2 version2	CACCGAAAGGAAAACGAAGGC	TCCGATTTTATGGGGCACGG
Gephyrin	AAACCACGACCATCAAATCCG	GGTGCAAACCCTGTTCCGC
Neuroligin1 version1	CACTCGAACTTTGGCTCACC	GGAAAGGCTGATGTGACTGG
Neuroligin1 version1	CGTGGACGTGCTTCCCTG	CAG CAA TGT GGT GAG AGT GG
NR2a-c	GGGGTTCTGCATCGACATCC	GACAGCAAAGAAGGCCCACAC
ß-Actin	CCGCGAGTACAACCTTCTTG	ATCGTCATCCATGGCGAACTG
Tubulin	CGGGCAGTGTTTGTAGACTTGG	CTCCTTGCCAATGGTGTAGTGC
