# Supplementary material for: S-SCAM is essential for synapse formation
Source: Front Cell Neurosci. 2023 Nov 16;17:1182493. doi: 10.3389/fncel.2023.1182493 (PMC10690602; doi:10.3389/fncel.2023.1182493)
Supplement: Supplementary file 1 [file Data_Sheet_1.zip › Data Sheet 1/Suppl. Figure S6 Legend.pdf]

**S6_Fig: Knockdown of S-SCAM in cortical neurons.**

(A) Schematic diagram of the experimental timeline. (B) Gene expression data (qPCR) for S-SCAM/MAGI-2 in cortical cultures after lentivirus expression of GFP (Control) or S-SCAM shRNA (S-SCAM RNAi), each with three replicates. Values were calculated using the $2^{-\Delta\Delta CT}$- method, normalized to beta-actin expression, and log2 transformed to show the log2 fold change. N = 3 independent culture experiments; mean ± SEM. (C) Western blot confirming knockdown of endogenous S-SCAM at the protein level in cultures of cortical neurons (viral transduction DIV2, analysis DIV9). S-SCAM protein levels of neurons after lentivirus expression of mismatch shRNA (Control RNAi) or S-SCAM shRNA (S-SCAM RNAi).
