# Supplementary material for: S-SCAM is essential for synapse formation
Source: Front Cell Neurosci. 2023 Nov 16;17:1182493. doi: 10.3389/fncel.2023.1182493 (PMC10690602; doi:10.3389/fncel.2023.1182493)
Supplement: Supplementary file 1 [file Data_Sheet_1.zip › Data Sheet 1/Suppl. Figure S3 Legend.pdf]

S3_Fig: Western blot analysis of S-SCAM expression in conventional cultured hippocampal mouse neurons. The expression of S-SCAM was effectively suppressed in neurons transduced with lentivirus expressing Oligo#1 or Oligo#2. N = 2 culture experiments.
