# Supplementary material for: S-SCAM is essential for synapse formation
Source: Front Cell Neurosci. 2023 Nov 16;17:1182493. doi: 10.3389/fncel.2023.1182493 (PMC10690602; doi:10.3389/fncel.2023.1182493)
Supplement: Supplementary file 1 [file Data_Sheet_1.zip › Data Sheet 1/Suppl. Figure S4 Legend.pdf]

S4_Fig: Gene-expression data of hippocampal cultures transduced with S-SCAM shRNA or GFP for various time windows.

(A) Schematic diagram of the experimental timeline for the results shown in panel B. (B) qPCR analysis of Magi2 (S-SCAM), Magi1, Matn2, Usp18, Gephyrin, Neuroligin1, and GluN2a-c in DIV16 samples infected on DIV2 with control EGFP or S-SCAM RNAi vectors. Values were calculated using the $2^{-\Delta\Delta CT}$- method, normalized to beta-actin expression, and log2 transformed to show the log2 fold change. N = 3 independent culture experiments; mean ± SEM. (C) Schematic diagram of the experimental timeline for the results shown in panel D. (D) qPCR analysis of Magi2 (S-SCAM), Magi1, Matn2, Usp18, Gephyrin, Neuroligin1, and GluN2a-c in DIV17 samples infected on DIV14 with control EGFP or S-SCAM RNAi vectors. Values were calculated using the $2^{-\Delta\Delta CT}$-method, normalized to beta-actin expression, and log2 transformed to show the log2 fold change. N = 3 independent culture experiments; mean ± SEM. (E) Schematic diagram of the experimental timeline for the results shown in panel F. (F) qPCR analysis of Magi2 (S-SCAM) in DIV9 samples infected on DIV2 with control EGFP or S-SCAM RNAi vectors. The level of knockdown for S-SCAM/Magi-2 was -2.01844 ± 0.060743 (SEM) for DIV2-DIV16, -1.16993 ± 0.243452 (SEM) for DIV14-DIV17, and -1.66153 ± 0.221041 (SEM) for DIV2-DIV9. Values were calculated using the $2^{-\Delta\Delta CT}$-method, normalized to beta-actin expression, and log2 transformed to show the log2 fold change. N = 3 independent culture experiments; mean ± SEM.
