# Supplementary material for: S-SCAM is essential for synapse formation
Source: Front Cell Neurosci. 2023 Nov 16;17:1182493. doi: 10.3389/fncel.2023.1182493 (PMC10690602; doi:10.3389/fncel.2023.1182493)
Supplement: Supplementary file 1 [file Data_Sheet_1.zip › Data Sheet 1/Supplementary Data S3 - shRNA sequences.pdf]

shRNA name	Full sequence	Sense strand	Loop	Location mouse	Location rat	Identity mouse genome	Identity rat genome
Oligo #1	GTACAGAACCTGAGCCATATTCAAGAGATATGGCTCAGGTTCTGTAC	GTACAGAACCTGAGCCATA	TTCAAGAGA	Exon 10	Exon 8	100%	100%
Oligo #2	GCCAGTCTATCATCAACATGCGTGAAGCCACAGATGGCATGTTGATGATAGACTGGC	GCCAGTCTATCATCAACATGC	GTGAAGCCACAGATG	Exon 17	Exon 15	100%	100%
Rescue / Control shRNA	GTCAATCGATAATAAATATGCGTGAAGCCACAGATGGCATATTTATTATCGATTGAC	GTCAATCGATAATAAATATGC	GTGAAGCCACAGATG	Exon 17	Exon 15	71.40%	71.40%
