# Supplementary material for: S-SCAM is essential for synapse formation
Source: Front Cell Neurosci. 2023 Nov 16;17:1182493. doi: 10.3389/fncel.2023.1182493 (PMC10690602; doi:10.3389/fncel.2023.1182493)
Supplement: Supplementary file 1 [file Data_Sheet_1.zip › Data Sheet 1/Suppl. Figure S1 Legend.pdf]

S1_Fig: Effect of S-SCAM knockdown on synapse density and misalignment of pre- and postsynaptic terminals.

(A, D) Schematic diagram of the experimental timeline. (B) Hippocampal cultured neurons were infected with S-SCAM RNAi or control RNAi viruses (green) and immunostained for the presynaptic protein Bassoon (red) at DIV9/10. Bar, 4 µm. (C) Quantification of the dendritic number of Bassoon puncta revealed a significant reduction of the formation of presynaptic terminals upon knockdown of S-SCAM. The data shown are mean ± SEM (N = 2 independent culture experiments). *: $p<0.05$, two-tailed unpaired *t* test. (E) Images of hippocampal neurons transfected with S-SCAM RNAi, control RNAi and EGFP vectors. Cells were immunostained for Bassoon and PSD95. Bar, 10 µm. (F) Quantification of PSD95 and Bassoon positive puncta, categorized as synapses/µm. Cells transfected with EGFP (n=25), control RNAi (n=20), or S-SCAM RNAi (n=17). Mean ± SEM (N = 3 independent culture experiments unpaired), **: $p<0.01$, ***: $p<0.001$, two-tailed unpaired *t* test. (G) Images of hippocampal neurons transfected with S-SCAM RNAi and control RNAi vectors. Cells were immunostained for VAMP2 and PSD95. Bar, 4 µm. (H) Quantification of VAMP2 puncta positive for PSD95. 10 – 15 cells were quantified. Mean ± SEM (N = 2 independent culture experiments). **: $p<0.01$, two-tailed unpaired *t* test.
