# Supplementary material for: S-SCAM is essential for synapse formation
Source: Front Cell Neurosci. 2023 Nov 16;17:1182493. doi: 10.3389/fncel.2023.1182493 (PMC10690602; doi:10.3389/fncel.2023.1182493)
Supplement: Supplementary file 1 [file Data_Sheet_1.zip › Data Sheet 1/Suppl. Figure S2 Legend.pdf]

S2_Fig: In situ detection of DNA fragmentation in S-SCAM knockdown hippocampal cultures. (A, D) Schematic diagram of the experimental timeline. (B, C) Neurons were transfected with S-SCAM RNAi and Control RNAi vectors. At DIV10, apoptotic cells were detected by the TdT-mediated dUTP nick end labeling method using TACS2 TdT-DAB *in situ* Apoptosis Detection Kit (Trevigen, Gaithersburg, MD, USA). Transfected neurons were identified by GFP signal. For TACS control untransfected cells treated with TACS nuclease and TdT enzyme were used. Control RNAi, n = 102; S-SCAM RNAi, n = 130; Bar, 10 μm. (E) Images of neuronal cultures treated with S-SCAM shRNA or control EGFP shRNA, non-transduced neurons treated with TACS nuclease and TdT enzyme and non-transduced neurons without TdT enzyme are shown. Only non-transduced neurons treated with TACS nuclease and TdT enzyme displayed apotopic cells.
