# Supplementary material for: S-SCAM is essential for synapse formation
Source: Front Cell Neurosci. 2023 Nov 16;17:1182493. doi: 10.3389/fncel.2023.1182493 (PMC10690602; doi:10.3389/fncel.2023.1182493)
Supplement: Supplementary file 1 [file Data_Sheet_1.zip › Data Sheet 1/Supplementary Data S1 - DeepSeq analysis.pdf]

ensembl_gene_id	rgd_symbo	description	entrezge	chrom	start_positio	end_position	length	baseMean	log2Fol	padj
ENSRNOG00000008697	**Nov**	nephroblastoma overexpressed (Nov)	81526	7	95015159	95022176	7018	984,46	-1,35	4,82E-13
ENSRNOG00000012442	**RGD13052**	Protein RGD1305254 [Source:UniPro	308797	1	147000790	147067480	66691	255,79	-1,75	4,17E-12
ENSRNOG00000019711	**Isoc1**	isochorismatase domain containing 1	364879	18	53008862	53028768	19907	1982,08	-1,83	1,81E-11
ENSRNOG00000014046	**Sertm1**	serine-rich and transmembrane domai	690333	2	163721932	163741483	19552	506,42	-1,40	2,01E-09
ENSRNOG00000020644	**Nsg2**	neuron specific gene family member 2	497878	10	15762454	15822361	59908	28137,06	-1,13	2,01E-09
ENSRNOG00000047088	**Rab10**	RAB10, member RAS oncogene famil	50993	6	37479074	37531801	52728	7832,81	-1,08	2,55E-09
ENSRNOG00000008203	**Synpr**	synaptoporin (Synpr), mRNA [Source:	66030	15	18562899	18741366	178468	613,50	-1,63	3,31E-09
ENSRNOG00000013962	**Magi2**	membrane associated guanylate kinas	113970	4	10987824	11595537	607714	1204,91	-1,39	3,31E-09
ENSRNOG00000010803	**Gabra5**	gamma-aminobutyric acid (GABA) A re	29707	1	113844283	113955085	110803	2368,02	-1,64	5,85E-09
ENSRNOG00000003280	**Grin2c**	glutamate receptor, ionotropic, N-meth	24411	10	104480212	104497817	17606	143,81	-1,95	1,09E-08
ENSRNOG00000005007	**Scn3a**	sodium channel, voltage-gated, type II	497770	3	58167493	58277870	110378	1121,47	-1,05	2,75E-08
ENSRNOG00000018445	**Agt**	angiotensinogen (serpin peptidase inh	24179	19	68026228	68038550	12323	367,38	-1,89	4,13E-08
ENSRNOG00000020684	**Vat1**	vesicle amine transport protein 1 hom	287721	10	89164915	89172532	7618	4291,53	1,12	4,91E-08
ENSRNOG00000001187	**Oasl**	2'-5'-oligoadenylate synthetase-like (O	304545	12	49237542	49249899	12358	24,75	2,53	1,57E-07
ENSRNOG00000014290	**Grm1**	glutamate receptor, metabotropic 1 (G	24414	1	6413420	6818380	404961	213,91	-1,60	1,77E-07
ENSRNOG00000014856	**Etnk1**	ethanolamine kinase 1 (Etnk1), mRNA	312828	4	241606529	241650653	44125	4840,02	-1,12	2,22E-07
ENSRNOG00000020773	**Map3k11**	mitogen-activated protein kinase kinas	309168	1	227978441	227991739	13299	720,80	1,04	2,70E-07
ENSRNOG00000013948	**Zc3hav1**	zinc finger CCCH type, antiviral 1 (Zc3	252832	4	65836588	65876491	39904	299,23	2,07	4,65E-07
ENSRNOG00000018851		protein phosphatase 2 (formerly 2A), regulatory		18	36647303	36757305	110003	2599,32	-1,47	2,15E-06
ENSRNOG00000008934	**Tmem65**	transmembrane protein 65 (Tmem65),	500874	7	99270342	99310255	39914	1632,78	-0,98	2,66E-06
ENSRNOG00000013024	**Csgalnact1**	chondroitin sulfate N-acetylgalactosan	306375	16	22868157	22963258	95102	344,76	-1,42	3,38E-06
ENSRNOG00000013781	**Kcnq5**	potassium voltage-gated channel, KQ	259273	9	26413937	26561055	147119	584,81	-0,97	5,56E-06
ENSRNOG00000019662	**Tm6sf1**	transmembrane 6 superfamily membe	361600	1	144605597	144647529	41933	365,25	-1,20	1,04E-05
ENSRNOG00000013140	**Pdzd2**	PDZ domain containing 2 (Pdzd2), mR	65034	2	82185700	82288609	102910	758,15	-1,46	1,16E-05
ENSRNOG00000006060	**Matn2**	Protein Matn2 [Source:UniProtKB/TrE	299996	7	73174202	73376715	202514	181,50	-1,97	1,55E-05
ENSRNOG00000006729		sodium/potassium/calcium exchanger	314396	6	135252727	135364932	112206	148,42	-1,81	1,55E-05
ENSRNOG00000011589	**Camk2d**	calcium/calmodulin-dependent protein	24246	2	250252085	250485218	233134	1796,04	-0,96	1,55E-05
ENSRNOG00000008758	**Tspan18**	tetraspanin 18 (Tspan18), mRNA [Sou	311210	3	88799485	88939055	139571	269,09	-1,45	1,79E-05
ENSRNOG00000024322	**Shroom2**	shroom family member 2 (Shroom2), r	317435	X	23895809	24065973	170165	2026,35	-1,38	1,79E-05
ENSRNOG00000013023	**Gltscr2**	glioma tumor suppressor candidate re	292624	1	79100888	79108702	7815	2648,53	0,82	1,79E-05
ENSRNOG00000009263	**Ifi27**	interferon, alpha-inducible protein 27 (	170512	6	136548421	136554959	6539	4703,02	2,17	1,79E-05
ENSRNOG00000034200		Protein Atp8a1 [Source:UniProtKB/Tr	289615	14	41770499	42002866	232368	1747,77	-1,20	1,85E-05
ENSRNOG00000034025		protein tyrosine phosphatase, receptor type, J [S		3	85950332	86099287	148956	710,58	-0,99	1,92E-05
ENSRNOG00000026053		Gremlin-1 [Source:UniProtKB/Swiss-P	50566	3	111781603	111791645	10043	49,92	-1,60	2,41E-05
ENSRNOG00000002336	**Gabra4**	gamma-aminobutyric acid (GABA) A re	140675	14	38966016	39040596	74581	334,56	-0,97	2,67E-05
ENSRNOG00000050924	**Kcnc4**	potassium voltage gated channel, Sha	684516	2	229694932	229715013	20082	311,49	-1,29	3,26E-05
ENSRNOG00000042753	**Fgf13**	fibroblast growth factor 13 (Fgf13), mR	84488	X	142078585	142189620	111036	3363,63	-1,19	3,26E-05

ENSRNOG00000007410	**Dab1**	Dab, reelin signal transducer, homolog	266729	5	127881438	128125734	244297	892,86	-1,27	3,48E-05
ENSRNOG00000037198	**Usp18**	ubiquitin specific peptidase 18 (Usp18	312688	4	220895597	220922138	26542	339,26	2,21	3,48E-05
ENSRNOG00000028624	**Kif26b**	kinesin family member 26B (Kif26b), n	305012	13	101800843	102194975	394133	344,29	-1,20	3,67E-05
ENSRNOG00000003686	**Tbl1x**	transducin (beta)-like 1 X-linked (Tbl1x	302711	X	45257868	45286546	28679	2386,25	-0,94	3,67E-05
ENSRNOG00000008431	**Gabbr2**	gamma-aminobutyric acid (GABA) B r	83633	5	66802348	67142203	339856	3518,57	-1,13	3,89E-05
ENSRNOG00000000435	**Ppt2**	palmitoyl-protein thioesterase 2 (Ppt2)	54398	20	6460813	6469830	9018	654,26	0,81	3,90E-05
ENSRNOG00000003756	**Lancl3**	LanC lantibiotic synthetase componen	302540	X	15139374	15271030	131657	54,35	-1,83	3,90E-05
ENSRNOG00000000170	**Slc30a4**	solute carrier family 30 (zinc transport	64469	3	121344030	121366262	22233	1286,39	-1,13	3,90E-05
ENSRNOG00000009523	**Rab11fip2**	RAB11 family interacting protein 2 (cla	308003	1	288422015	288457832	35818	294,41	-1,09	3,94E-05
ENSRNOG00000000129	**Phf24**	Protein RGD1559864 [Source:UniPro	500446	5	62902399	62910270	7872	1434,10	-1,05	3,98E-05
ENSRNOG00000018111	**Slc12a5**	solute carrier family 12 member 5 [So	171373	3	167610336	167647352	37017	1726,52	-0,96	3,98E-05
ENSRNOG00000009001	**Mtss1**	metastasis suppressor 1 (Mtss1), mRN	362918	7	99419337	99557952	138616	5025,65	-0,96	3,98E-05
ENSRNOG00000023370	**Bst2**	bone marrow stromal cell antigen 2 (B	378947	16	19800348	19803919	3572	509,54	2,17	3,98E-05
ENSRNOG00000002714	**Mettl22**	Protein Mettl22 [Source:UniProtKB/Tr	287054	10	6026812	6043628	16817	932,01	0,89	4,44E-05
ENSRNOG00000021266	**Prokr2**	prokineticin receptor 2 (Prokr2), mRNA	192649	3	131503826	131511943	8118	121,45	-1,71	5,49E-05
ENSRNOG00000007377	**Slit3**	slit homolog 3 (Drosophila) (Slit3), mR	83467	10	20195980	20392415	196436	335,97	-1,15	5,49E-05
ENSRNOG00000005840	**Cacfd1**	calcium channel flower domain contair	296599	3	10920444	10928043	7600	554,49	0,94	5,49E-05
ENSRNOG00000015071	**Zim1**	zinc finger, imprinted 1 (Zim1), mRNA	308322	1	71646069	71659128	13060	90,31	-1,42	6,14E-05
ENSRNOG00000006631	**Sema3e**	sema domain, immunoglobulin domair	296789	4	17288508	17563239	274732	91,73	-1,43	7,56E-05
ENSRNOG00000012690	**Nipa2**	non imprinted in Prader-Willi/Angelma	308667	1	115354415	115378274	23860	880,14	-0,95	7,68E-05
ENSRNOG00000046864	**Acot4**	acyl-CoA thioesterase 4 (Acot4), mRN	681337	6	118021372	118026536	5165	78,03	-1,45	7,78E-05
ENSRNOG00000001372	**Oas1b**	2-5 oligoadenylate synthetase 1B (Oa	246268	12	43116714	43127900	11187	388,78	2,10	8,63E-05
ENSRNOG00000001374	**Rasal1**	RAS protein activator like 1 (GAP1 like	360814	12	43274639	43307089	32451	512,15	-1,33	8,92E-05
ENSRNOG00000016704	**Pcyox1**	prenylcysteine oxidase 1 (Pcyox1), mR	246302	4	182758202	182769261	11060	3962,53	-0,87	8,92E-05
ENSRNOG00000010960	**Ankh**	ANKH inorganic pyrophosphate transp	114506	2	99796579	99921970	125392	1816,30	-0,86	8,92E-05
ENSRNOG00000023720	**Ntm**	neurotrimin (Ntm), mRNA [Source:Ref	50864	8	30074875	30510958	436084	1134,30	-1,08	9,51E-05
ENSRNOG00000008022		apoptotic peptidase activating factor 1 [Source:N		7	31792818	31872984	80167	1202,88	-0,95	9,60E-05
ENSRNOG00000031890	**Ncam1**	neural cell adhesion molecule 1 (Ncan	24586	8	52458775	52522326	63552	14499,97	-1,06	1,24E-04
ENSRNOG00000012681		lectin, galactoside-binding, soluble, 9 (	25476	10	66863439	66886611	23173	304,53	2,03	1,24E-04
ENSRNOG00000025494	**Nsun7**	NOP2/Sun domain family, member 7 (	305339	14	43346771	43374401	27631	25,09	-1,73	1,67E-04
ENSRNOG00000007079	**Met**	met proto-oncogene (Met), mRNA [So	24553	4	45382347	45459517	77171	961,69	-1,47	1,70E-04
ENSRNOG00000011123	**Fam81a**	family with sequence similarity 81, me	315789	8	76497582	76554483	56902	1284,39	-0,99	1,98E-04
ENSRNOG00000017679	**Cckbr**	cholecystokinin B receptor (Cckbr), mR	25706	1	177269044	177279480	10437	46,95	-1,70	2,08E-04
ENSRNOG00000012181	**Lpl**	lipoprotein lipase (Lpl), mRNA [Source	24539	16	22431494	22455871	24378	546,74	-0,99	2,31E-04
ENSRNOG00000001229	**Col18a1**	collagen, type XVIII, alpha 1 (Col18a1	85251	20	14388743	14495241	106499	208,60	-1,35	2,79E-04
ENSRNOG00000032002	**Hapln1**	hyaluronan and proteoglycan link prot	29331	2	18229924	18291848	61925	224,34	-1,81	2,80E-04
ENSRNOG00000014284	**Elof1**	elongation factor 1 homolog (S. cerevi	691193	8	23180772	23185661	4890	1509,59	0,84	2,80E-04
ENSRNOG00000019532	**Emc10**	ER membrane protein complex subun	292878	1	101560506	101566824	6319	7016,74	1,01	2,80E-04

ENSRNOG00000015380	**Jup**	junction plakoglobin (Jup), mRNA [Sou	81679	10	88073766	88100379	26614	1545,62	-1,04	2,95E-04
ENSRNOG00000002129	**Lrrc8b**	leucine rich repeat containing 8 family,	305135	14	5409635	5471379	61745	795,89	-1,46	3,12E-04
ENSRNOG00000013515	**Ptpru**	protein tyrosine phosphatase, recepto	116680	5	153601583	153675513	73931	561,63	-1,53	3,63E-04
ENSRNOG00000016677	**Csnk1g3**	casein kinase 1, gamma 3 (Csnk1g3),	64823	18	48065995	48126390	60396	912,43	-1,19	3,67E-04
ENSRNOG00000005723	**Grin3a**	glutamate receptor, ionotropic, N-meth	191573	5	69420058	69564696	144639	661,58	-1,43	3,68E-04
ENSRNOG00000006393	**Sirt6**	sirtuin 6 (Sirt6), mRNA [Source:RefSe	299638	7	11106748	11112173	5426	1818,80	0,76	3,68E-04
ENSRNOG00000002341	**Trim25**	tripartite motif-containing 25 (Trim25),	494338	10	73754576	73769423	14848	132,88	1,74	3,68E-04
ENSRNOG00000003875	**Ocrl**	oculocerebrorenal syndrome of Lowe	317576	X	134814622	134865807	51186	807,15	-0,97	3,81E-04
ENSRNOG00000016366	**Colec12**	collectin sub-family member 12 (Colec	361289	18	910945	1096503	185559	44,06	-1,95	3,95E-04
ENSRNOG00000018881	**Rnasek**	ribonuclease, RNase K (Rnasek), mRI	287453	10	56592533	56594249	1717	2710,09	0,69	3,95E-04
ENSRNOG00000006783		Protein Neb [Source:UniProtKB/TrEM	311029	3	42756012	42835971	79960	66,25	1,29	3,95E-04
ENSRNOG00000027540	**Fam102b**	family with sequence similarity 102, m	365903	2	231243585	231302559	58975	275,97	-1,20	3,98E-04
ENSRNOG00000007845	**Supt4h1**	suppressor of Ty 4 homolog 1 (S. cere	287608	10	75063840	75070031	6192	1146,60	0,84	4,25E-04
ENSRNOG00000007646	**Sipa1l1**	signal-induced proliferation-associatec	246212	6	114214346	114304630	90285	1234,73	-1,01	4,38E-04
ENSRNOG00000013202	**Akap7**	A kinase (PRKA) anchor protein 7 (Ak	361458	1	22621734	22744656	122923	1417,99	-0,99	4,46E-04
ENSRNOG00000026577	**Cpne4**	copine IV (Cpne4), mRNA [Source:Re	367160	8	112491426	112972591	481166	1029,34	-0,93	4,51E-04
ENSRNOG00000017140		Protein Zfp185 [Source:UniProtKB/Tr	689949	1	148728594	148761025	32432	106,25	-1,66	5,52E-04
ENSRNOG00000008908	**Slc35a1**	solute carrier family 35 (CMP-sialic ac	313139	5	54688835	54707429	18595	349,72	-1,14	5,52E-04
ENSRNOG00000021262	**Slc23a2**	solute carrier family 23 (ascorbic acid	50622	3	131132125	131224768	92644	2040,62	-0,94	5,52E-04
ENSRNOG00000011655	**Dcaf10**	DDB1 and CUL4 associated factor 10	313242	5	65409106	65446189	37084	772,19	-0,77	5,87E-04
ENSRNOG00000007968	**RGD13053**	similar to RIKEN cDNA 2510039O18 (	313699	5	168409903	168416143	6241	1888,51	0,81	6,27E-04
ENSRNOG00000026310	**March1**	membrane-associated ring finger (C3H	361135	16	24885332	25407999	522668	141,02	-1,11	6,39E-04
ENSRNOG00000008911	**Draxin**	Protein Draxin [Source:UniProtKB/TrE	691317	5	168572013	168585976	13964	572,31	-1,13	6,91E-04
ENSRNOG00000014104	**Myo5b**	myosin Vb (Myo5b), mRNA [Source:R	25132	18	69568405	69866976	298572	451,06	-1,39	6,99E-04
ENSRNOG00000004084	**Fam84a**	family with sequence similarity 84, me	313969	6	48123855	48128328	4474	1001,43	-1,05	7,08E-04
ENSRNOG00000002595	**Dpp10**	dipeptidylpeptidase 10 (Dpp10), mRN	363972	13	44555442	44766710	211269	375,38	-1,22	7,56E-04
ENSRNOG00000003905	**Nsf**	N-ethylmaleimide-sensitive factor (Nsf	60355	10	91645027	91751625	106599	5879,27	-1,07	7,62E-04
ENSRNOG00000025383	**Pdha1l1**	pyruvate dehydrogenase (lipoamide) a	29554	X	37640725	37654356	13632	1152,15	-1,26	7,81E-04
ENSRNOG00000008451	**Fut8**	fucosyltransferase 8 (alpha (1,6) fucos	432392	6	109693324	109922266	228943	1066,26	-0,68	8,46E-04
ENSRNOG00000009892	**Adamts15**	ADAM metallopeptidase with thrombo	300474	8	32003564	32026941	23378	166,92	-1,40	1,10E-03
ENSRNOG00000011142	**Cyb5b**	cytochrome b5 type B (outer mitochon	80773	19	50221836	50255693	33858	2201,59	-0,73	1,15E-03
ENSRNOG00000001369		2'-5' oligoadenylate synthetase 1A (Oa	192281	12	43064584	43106931	42348	204,05	1,85	1,17E-03
ENSRNOG00000008157	**Syn2**	synapsin II (Syn2), transcript variant 1	29179	4	210326340	210483842	157503	1590,36	-1,12	1,19E-03
ENSRNOG00000002895	**Nagpa**	N-acetylglucosamine-1-phosphodieste	360476	10	9342962	9351287	8326	266,09	0,88	1,23E-03
ENSRNOG00000020887	**Tmem101**	transmembrane protein 101 (Tmem10	303564	10	89879760	89883460	3701	866,81	0,69	1,24E-03
ENSRNOG00000014761	**Rasd2**	RASD family, member 2 (Rasd2), mRI	171099	19	25770392	25781108	10717	444,82	-1,26	1,33E-03
ENSRNOG00000027309		contactin associated protein-like 3 [Source:MGI		17	2188225	2271053	82829	258,57	-1,24	1,34E-03
ENSRNOG00000010183	**Fam198b**	family with sequence similarity 198, m	310540	2	198087637	198158770	71134	44,17	-1,41	1,37E-03

ENSRNOG00000004641	**Sstr4**	somatostatin receptor 4 (Sstr4), mRNA	25555	3	149151003	149152866	1864	109,89	-1,08	1,39E-03
ENSRNOG00000004281	**Cobl**	cordon-bleu WH2 repeat protein (Cobl	305497	14	92142188	92292689	150502	554,18	-1,25	1,43E-03
ENSRNOG00000003031		plasma membrane calcium-transportir	29600	13	55157902	55204057	46156	1134,92	-1,46	1,43E-03
ENSRNOG00000020369	**Igf2**	insulin-like growth factor 2 (Igf2), trans	24483	1	222722922	222730430	7509	29,84	-1,77	1,44E-03
ENSRNOG00000006636	**Otud6b**	OTU domain containing 6B (Otud6b),	297911	5	33018928	33034090	15163	447,47	-1,43	1,44E-03
ENSRNOG00000018500		FERM domain containing 4A [Source:MGI Symb		17	79297096	79342250	45155	1017,09	-0,78	1,44E-03
ENSRNOG00000015134	**Map3k12**	mitogen activated protein kinase kinas	25579	7	141894814	141901161	6348	2117,15	-0,68	1,47E-03
ENSRNOG00000024730	**Ppm1e**	protein phosphatase, Mg2+/Mn2+ dep	360593	10	75421043	75552393	131351	2015,94	-0,87	1,52E-03
ENSRNOG00000048929		Protein Stk19 [Source:UniProtKB/TrE	361800	20	6589829	6596880	7052	293,51	0,84	1,52E-03
ENSRNOG00000017077	**LOC10091**	sorting nexin 7 (Snx7), mRNA [Source	310815	2	239065719	239150147	84429	212,50	-1,26	1,52E-03
ENSRNOG00000017707		abl interactor 2 [Source:RefSeq pepti	286928	9	67013845	67098832	84988	5399,92	-1,00	1,58E-03
ENSRNOG00000029012	**Shisa6**	shisa family member 6 (Shisa6), mRN	497926	10	52504105	52791629	287525	195,04	-0,98	1,66E-03
ENSRNOG00000021032	**Sphk2**	sphingosine kinase 2 (Sphk2), mRNA	308589	1	102771805	102776109	4305	1486,49	0,99	1,74E-03
ENSRNOG00000028992	**Acan**	aggrecan (Acan), mRNA [Source:RefS	58968	1	141732130	141793202	61073	133,90	-0,90	1,85E-03
ENSRNOG00000027233	**Trpc5**	transient receptor potential cation chai	140933	X	114081723	114216237	134515	466,43	-1,15	1,92E-03
ENSRNOG00000003741	**Nptx1**	neuronal pentraxin I (Nptx1), mRNA [S	266777	10	108290473	108295779	5307	174,23	-1,32	1,92E-03
ENSRNOG00000030714	**Bsn**	bassoon (presynaptic cytomatrix prote	29138	8	116227802	116319071	91270	4792,19	-0,89	2,04E-03
ENSRNOG00000009079	**Prkar2b**	protein kinase, cAMP dependent regul	24679	6	59936129	59968013	31885	4754,76	-0,85	2,09E-03
ENSRNOG00000023548	**Sned1**	sushi, nidogen and EGF-like domains	316638	9	100037560	100093411	55852	80,99	-1,62	2,09E-03
ENSRNOG00000007970		plexin C1 [Source:MGI Symbol;Acc:MGI:189012		7	35914416	36063727	149312	3179,12	-1,27	2,09E-03
ENSRNOG00000009403	**Dcaf17**	DDB1 and CUL4 associated factor 17	499807	3	64142533	64167836	25304	189,07	-1,03	2,09E-03
ENSRNOG00000033824		Glycerol-3-phosphate dehydrogenase, mitochon		3	48327068	48426568	99501	400,98	-0,83	2,09E-03
ENSRNOG00000027230	**Fhod3**	formin homology 2 domain containing	1E+08	18	16916474	17086632	170159	841,87	-1,20	2,29E-03
ENSRNOG00000010213	**Fgd5**	Protein Fgd5 [Source:UniProtKB/TrEM	362402	4	189299931	189387281	87351	88,76	-1,47	2,30E-03
ENSRNOG00000011132	**LOC10036**	RIKEN cDNA D430041D05 gene [Sou	1E+08	3	100703018	100819225	116208	1674,06	-0,83	2,39E-03
ENSRNOG00000005971	**Gpr176**	G protein-coupled receptor 176 (Gpr1	117257	3	116588548	116686280	97733	168,33	-1,04	2,49E-03
ENSRNOG00000004992		RAB1, member RAS oncogene family [Source:M		14	104208410	104236221	27812	3443,27	-0,64	2,50E-03
ENSRNOG00000006503	**Frmd3**	FERM domain containing 3 (Frmd3), r	298141	5	94097585	94202350	104766	175,64	-1,01	2,52E-03
ENSRNOG00000010320	**Efnb3**	ephrin B3 (Efnb3), mRNA [Source:Ref	360546	10	55906787	55912782	5996	5811,47	-1,05	2,61E-03
ENSRNOG00000005634	**Lingo2**	leucine rich repeat and Ig domain cont	313156	5	56027971	56030029	2059	128,82	-1,13	2,67E-03
ENSRNOG00000019885	**Magi3**	membrane associated guanylate kinas	245903	2	225923270	226123408	200139	1185,13	-0,68	2,71E-03
ENSRNOG00000007091	**Ly6e**	lymphocyte antigen 6 complex, locus I	362934	7	116253795	116257488	3694	1576,08	0,72	2,71E-03
ENSRNOG00000039587	**Wdr13**	WD repeat domain 13 (Wdr13), mRNA	317370	X	15897039	15904909	7871	1451,21	-0,76	2,73E-03
ENSRNOG00000008337	**Gjd2**	gap junction protein, delta 2 (Gjd2), m	50564	3	112040926	112043925	3000	206,51	-1,07	2,85E-03
ENSRNOG00000037146	**Ctxn2**	cortexin 2 (Ctxn2), mRNA [Source:Ref	1E+08	3	123940156	123940597	442	122,73	1,15	2,91E-03
ENSRNOG00000009355	**Ogfr**	opioid growth factor receptor (Ogfr), m	83525	3	179794351	179800638	6288	3574,72	0,63	3,02E-03
ENSRNOG00000011211	**Pex5l**	peroxisomal biogenesis factor 5-like (F	286937	2	138979958	139185885	205928	216,60	-1,11	3,03E-03
ENSRNOG00000005392	**Ngfr**	nerve growth factor receptor (Ngfr), m	24596	10	83198415	83216629	18215	33,49	-1,55	3,15E-03

ENSRNOG00000003148	**Timp2**	TIMP metallopeptidase inhibitor 2 (Tim	29543	10	106973463	107021070	47608	6683,81	-0,94	3,15E-03
ENSRNOG00000008787	**Foxj3**	forkhead box J3 (Foxj3), mRNA [Sourc	313554	5	142408836	142478023	69188	1555,16	-0,75	3,15E-03
ENSRNOG00000006911	**Sptb**	spectrin, beta, erythrocytic (Sptb), mR	314251	6	109073106	109181206	108101	577,45	-1,06	3,26E-03
ENSRNOG00000011000	**Rims1**	regulating synaptic membrane exocyto	84556	9	27284840	27813498	528659	1527,91	-0,76	3,34E-03
ENSRNOG00000022919	**Chst8**	carbohydrate (N-acetylgalactosamine	308511	1	91681357	91822788	141432	269,86	-0,82	3,61E-03
ENSRNOG00000026846		RALBP1 associated Eps domain conta	363466	X	34228233	34401250	173018	203,97	-0,81	3,74E-03
ENSRNOG00000017619	**Aldh1a1**	aldehyde dehydrogenase 1 family, me	24188	1	245521322	245562969	41648	368,96	1,58	3,94E-03
ENSRNOG00000008526	**Pdzd3**	PDZ domain containing 3 (Pdzd3), mF	500986	8	47196475	47200923	4449	67,94	-1,46	4,00E-03
ENSRNOG00000009652	**Prkci**	protein kinase C, iota (Prkci), mRNA [S	84006	2	135636956	135697732	60777	1866,08	-0,70	4,00E-03
ENSRNOG00000019358	**Esr1**	estrogen receptor 1 (Esr1), mRNA [So	24890	1	42667185	42931332	264148	21,54	-1,59	4,06E-03
ENSRNOG00000016245	**Neto2**	neuropilin (NRP) and tolloid (TLL)-like	307757	19	33456905	33493500	36596	1291,99	-0,78	4,21E-03
ENSRNOG00000029814	**Prtn3**	proteinase 3 (Prtn3), mRNA [Source:R	314615	7	12813434	12816673	3240	86,59	1,06	4,21E-03
ENSRNOG00000046254	**Emr1**	EGF-like module containing, mucin-lik	316137	9	8434335	8593090	158756	28,12	-1,68	4,30E-03
ENSRNOG00000019254	**Dyrk1b**	dual-specificity tyrosine-(Y)-phosphory	308468	1	86329130	86336571	7442	659,58	0,87	4,30E-03
ENSRNOG00000000379	**Dnajc12**	DnaJ (Hsp40) homolog, subfamily C, r	619393	20	8200164	8222140	21977	279,70	0,96	4,30E-03
ENSRNOG00000001469	**Eln**	elastin (Eln), mRNA [Source:RefSeq m	25043	12	26978753	27022484	43732	1012,11	-1,08	4,37E-03
ENSRNOG00000022428	**Flrt1**	fibronectin leucine rich transmembrane	499308	1	229338914	229340938	2025	193,56	-0,88	4,37E-03
ENSRNOG00000028366		gephyrin [Source:RefSeq peptide;Acc	64845	6	110978550	111237656	259107	1770,61	-0,78	4,37E-03
ENSRNOG00000001225	**Fam207a**	family with sequence similarity 207, m	294333	20	14030705	14064390	33686	1550,17	0,71	4,39E-03
ENSRNOG00000003183	**Fmod**	fibromodulin (Fmod), mRNA [Source:F	64507	13	55928423	55939039	10617	541,58	-1,12	4,40E-03
ENSRNOG00000016999	**Grp**	gastrin releasing peptide (Grp), mRNA	171101	18	60759080	60772845	13766	205,25	-1,51	4,90E-03
ENSRNOG00000030418	**Tmem47**	transmembrane protein 47 (Tmem47),	501569	X	48984317	49010851	26535	1930,31	-1,08	4,90E-03
ENSRNOG00000033942	**Grin2a**	glutamate receptor, ionotropic, N-meth	24409	10	4527220	4936773	409554	154,90	-1,07	4,90E-03
ENSRNOG00000018712		Uncharacterized protein [Source:UniProtKB/TrE		18	55428528	55529045	100518	2302,84	-1,29	4,93E-03
ENSRNOG00000002763	**Ulk2**	unc-51 like autophagy activating kinas	303206	10	47845649	47922223	76575	1961,93	-0,81	4,93E-03
ENSRNOG00000011016		solute carrier family 7 (cationic amino	64554	16	54171102	54224384	53283	156,37	-1,34	5,03E-03
ENSRNOG00000012037	**Galnt7**	UDP-N-acetyl-alpha-D-galactosamine	29750	16	35751116	35876429	125314	252,06	-0,84	5,04E-03
ENSRNOG00000018095	**Nkiras2**	NFKB inhibitor interacting Ras-like 2 (l	287707	10	88331030	88335057	4028	1183,71	0,70	5,12E-03
ENSRNOG00000046515	**St6gal2**	ST6 beta-galactosamide alpha-2,6-sia	301155	9	6048734	6101607	52874	461,43	-1,06	5,58E-03
ENSRNOG00000012759		collagen, type XIX, alpha 1 [Source:M	367236	9	29339207	29668271	329065	87,79	-0,94	5,58E-03
ENSRNOG00000026884	**Snx17**	sorting nexin 17 (Snx17), mRNA [Sour	298836	6	36359401	36361303	1903	1717,84	0,60	5,58E-03
ENSRNOG00000025806	**Prr3**	proline rich 3 (Prr3), mRNA [Source:R	361788	20	5398413	5404790	6378	2109,81	0,65	5,62E-03
ENSRNOG00000007329		FERM domain containing 6 (Frmd6), r	257646	6	102768949	102802463	33515	108,59	-0,99	5,66E-03
ENSRNOG00000004524	**Desi2**	desumoylating isopeptidase 2 (Desi2),	289277	13	100554069	100597418	43350	768,23	-0,77	5,77E-03
ENSRNOG00000013875	**Fbxl17**	F-box and leucine-rich repeat protein 1	316663	9	110083540	110508540	425001	1076,04	-0,75	5,88E-03
ENSRNOG00000009525	**Rnf114**	ring finger protein 114 (Rnf114), mRNA	362277	3	170399735	170411498	11764	3745,31	0,80	5,88E-03
ENSRNOG00000003121	**Rtn4rl1**	reticulon 4 receptor-like 1 (Rtn4rl1), m	303311	10	61748820	61822593	73774	790,66	-0,97	6,01E-03
ENSRNOG00000012108	**Thsd1**	thrombospondin, type I, domain contai	364630	16	74316449	74346243	29795	161,95	0,86	6,01E-03

ENSRNOG00000050137	**Ajap1**	adherens junction associated protein 1	687031	5	174137054	174194344	57291	463,36	-0,91	6,01E-03
ENSRNOG00000014443	**Pde5a**	phosphodiesterase 5A, cGMP-specific	171115	2	246262030	246402956	140927	199,40	-1,05	6,04E-03
ENSRNOG00000001915	**Chodl**	chondrolectin (Chodl), mRNA [Source:	288289	11	21183908	21206141	22234	179,40	-0,93	6,04E-03
ENSRNOG00000000411	**Nus1**	nuclear undecaprenyl pyrophosphate s	294400	20	35336831	35363789	26959	1536,44	-0,77	6,04E-03
ENSRNOG00000033894	**Nrg3**	Protein Nrg3 [Source:UniProtKB/TrEM	498596	16	15663031	15697361	34331	332,56	-0,72	6,04E-03
ENSRNOG00000046045				14	80370225	80370277	53	61,39	-1,12	6,12E-03
ENSRNOG00000007581	**Slc17a8**	solute carrier family 17 (vesicular gluta	266767	7	30314802	30371888	57087	17,90	-1,49	6,16E-03
ENSRNOG00000013301		Protein Bean1 [Source:UniProtKB/TrE	361358	19	950519	983660	33142	65,51	-1,28	6,16E-03
ENSRNOG00000002214	**Klhl8**	kelch-like family member 8 (Klhl8), mR	289457	14	7159371	7204942	45572	782,68	-0,83	6,16E-03
ENSRNOG00000046581	**LOC10091**	Protein LOC100911595 [Source:UniP	1E+08	5	72166934	72169087	2154	84,33	-1,24	6,20E-03
ENSRNOG00000022704	**Esyt3**	Protein Esyt3 [Source:UniProtKB/TrE	363120	8	106981123	107027925	46803	26,45	-1,42	6,24E-03
ENSRNOG00000046829	**Kdr**	kinase insert domain receptor (Kdr), m	25589	14	34557361	34618109	60749	85,91	-1,60	6,41E-03
ENSRNOG00000001712		ALG3, alpha-1,3- mannosyltransferase	287983	11	87058552	87064028	5477	662,24	0,68	6,41E-03
ENSRNOG00000003005	**Rasgef1c**	RasGEF domain family, member 1C (I	360519	10	35164536	35230687	66152	169,73	-1,25	6,64E-03
ENSRNOG00000005345	**Vsnl1**	visinin-like 1 (Vsnl1), mRNA [Source:F	24877	6	46751474	46872211	120738	1017,49	-0,80	6,64E-03
ENSRNOG00000010185	**RGD13069**	similar to CG31122-PA (RGD1306941	316406	9	63871338	63881044	9707	581,33	-0,79	6,76E-03
ENSRNOG00000011157	**Jak1**	Janus kinase 1 (Jak1), mRNA [Source	84598	5	123865518	123966233	100716	3394,81	-1,05	6,81E-03
ENSRNOG00000003869	**Sod3**	superoxide dismutase 3, extracellular	25352	14	61071313	61077045	5733	152,32	-0,86	6,81E-03
ENSRNOG00000019582	**Mthfd1l**	methylenetetrahydrofolate dehydroger	361472	1	41876363	42065388	189026	732,85	-0,66	6,96E-03
ENSRNOG00000001249	**Col6a1**	Protein Col6a1 [Source:UniProtKB/Tr	294337	20	14817563	14835866	18304	109,01	-1,48	6,97E-03
ENSRNOG00000001247	**Clip1**	CAP-GLY domain containing linker pro	65201	12	40226383	40332760	106378	1409,40	-0,73	6,97E-03
ENSRNOG00000007125	**Vps54**	vacuolar protein sorting 54 homolog (S	286932	14	106220349	106274484	54136	595,28	-0,81	7,12E-03
ENSRNOG00000007887	**Elk4**	ELK4, ETS-domain protein (SRF acce	304786	13	53860253	53883004	22752	1288,93	-0,75	7,15E-03
ENSRNOG00000024568	**Ndufs7**	NADH dehydrogenase (ubiquinone) Fe	362837	7	12488477	12495748	7272	1883,96	0,54	7,15E-03
ENSRNOG00000015970	**Tbc1d13**	Protein Tbc1d13 [Source:UniProtKB/T	499768	3	14054961	14068345	13385	1063,11	0,68	7,15E-03
ENSRNOG00000007575	**Lppr1**	lipid phosphate phosphatase-related p	298062	5	72309081	72594906	285826	992,02	-1,00	7,27E-03
ENSRNOG00000005278	**Trhde**	thyrotropin-releasing hormone degradi	366894	7	57265684	57693174	427491	133,18	-0,92	7,29E-03
ENSRNOG00000032798	**Slco3a1**	solute carrier organic anion transporte	140915	1	136802247	137070858	268612	481,74	-0,86	7,46E-03
ENSRNOG00000046112	**Dedd2**	Protein LOC687118; RCG54082, isofo	687118	1	83342209	83356711	14503	497,25	0,81	7,60E-03
ENSRNOG00000004693	**Pbx1**	pre-B-cell leukemia homeobox 1 (Pbx1	304947	13	91029200	91306878	277679	1910,33	-0,67	7,88E-03
ENSRNOG00000008615	**Mal2**	mal, T-cell differentiation protein 2 (Ma	362911	7	94768727	94801520	32794	949,64	-0,99	7,97E-03
ENSRNOG00000047080	**Gng4**	Guanine nucleotide-binding protein su	114118	17	91930878	91950536	19659	78,34	-1,23	8,02E-03
ENSRNOG00000010124		tripartite motif-containing protein 2 [So	361970	2	202561569	202624484	62916	2029,04	-1,04	8,02E-03
ENSRNOG00000023403	**Gtpbp3**	GTP binding protein 3 (Gtpbp3), mRN	290633	16	19758699	19763790	5092	377,62	0,90	8,24E-03
ENSRNOG00000006649	**Thrb**	thyroid hormone receptor beta (Thrb),	24831	15	12953151	13147289	194139	492,22	-0,84	8,71E-03
ENSRNOG00000012638	**Ciao1**	cytosolic iron-sulfur protein assembly 1	29231	3	126196997	126202542	5546	1436,34	0,69	8,71E-03
ENSRNOG00000009640	**Psmf1**	proteasome (prosome, macropain) inh	689852	3	153590719	153615931	25213	1539,92	0,91	8,71E-03
ENSRNOG00000004873	**Prkch**	protein kinase C, eta (Prkch), mRNA [S	81749	6	105972486	106109232	136747	91,08	-1,05	8,78E-03

ENSRNOG00000018366	**RGD13108**	Protein RGD1310819 [Source:UniPro	301351	9	43896224	43935094	38871	162,81	-0,88	8,78E-03
ENSRNOG00000017621	**Spns1**	spinster homolog 1 (Drosophila) (Spns	361648	1	204753275	204760552	7278	2557,71	0,65	8,78E-03
ENSRNOG00000036688	**Arhgdia**	Rho GDP dissociation inhibitor (GDI) a	360678	10	109347156	109350645	3490	16116,51	0,69	8,78E-03
ENSRNOG00000018116	**Kcna3**	potassium voltage-gated channel, sha	29731	2	229233084	229234661	1578	144,56	-1,09	8,88E-03
ENSRNOG00000003699		endonuclease V [Source:MGI Symbol;Acc:MGI:2		10	108235192	108251018	15827	364,36	0,68	8,98E-03
ENSRNOG00000029914	**Cacna1i**	calcium channel, voltage-dependent, T	56827	7	121545990	121604663	58674	707,40	-1,06	9,07E-03
ENSRNOG00000004900	**Crhr1**	corticotropin releasing hormone recep	58959	10	91953470	91994397	40928	126,64	-1,07	9,27E-03
ENSRNOG00000015288	**Dip2c**	DIP2 disco-interacting protein 2 homol	307067	17	65192742	65571872	379131	2089,09	-0,66	9,28E-03
ENSRNOG00000012933	**Prcc**	papillary renal cell carcinoma (transloc	310687	2	206636545	206661988	25444	2513,04	0,58	9,28E-03
ENSRNOG00000049665		Uncharacterized protein [Source:UniProtKB/TrE		1	154731975	154737611	5637	1265,40	0,72	9,28E-03
ENSRNOG00000047219		ubiquitin protein ligase E3B [Source:MGI Symbo		12	49737720	49780944	43225	1950,79	0,74	9,28E-03
ENSRNOG00000008053	**LOC69188**	ATPase, aminophospholipid transporte	691889	15	43903107	44204457	301351	491,07	-0,72	9,28E-03
ENSRNOG00000011116	**Snx25**	Protein Snx25 [Source:UniProtKB/TrE	306471	16	49076062	49159354	83293	805,65	-0,77	9,56E-03
ENSRNOG00000005711	**Ptprd**	Protein Ptprd [Source:UniProtKB/TrEI	313278	5	96910961	97206174	295214	2245,76	-0,59	9,56E-03
ENSRNOG00000011508	**Zc3h12b**	Protein Zc3h12b; RCG36219 [Source	296864	X	65883054	65916508	33455	118,56	-0,83	9,67E-03
ENSRNOG00000013257	**Hecw2**	HECT, C2 and WW domain containing	316395	9	60142808	60357086	214279	618,47	-0,86	9,76E-03
ENSRNOG00000010957	**Braf**	Protein Braf [Source:UniProtKB/TrEM	114486	4	67206868	67327589	120722	371,70	-0,64	9,76E-03
ENSRNOG00000013844	**Fam172a**	family with sequence similarity 172, m	294606	2	5174044	5531001	356958	1163,95	-0,75	9,81E-03
ENSRNOG00000016538	**Itga8**	integrin, alpha 8 (Itga8), mRNA [Sourc	364786	17	80956113	81304978	348866	132,27	-1,12	9,84E-03
ENSRNOG00000005404	**Rasgrp1**	RAS guanyl releasing protein 1 (calciu	29434	3	115539782	115600270	60489	144,11	-1,09	9,87E-03
ENSRNOG00000014686	**Kcnd3**	potassium voltage-gated channel, Sha	65195	2	227345046	227557341	212296	412,42	-0,83	9,87E-03
ENSRNOG00000019050	**Ifit1**	interferon-induced protein with tetratric	56824	1	260165588	260166976	1389	23,79	1,55	9,88E-03
ENSRNOG00000008428	**Drd2**	D(2) dopamine receptor [Source:UniProtKB/Swi		8	52298097	52363812	65716	118,68	-1,32	9,90E-03
ENSRNOG00000015615	**Tnfrsf11a**	tumor necrosis factor receptor superfa	498206	13	30966261	30996894	30634	63,34	-1,14	1,01E-02
ENSRNOG00000022975		Nfat activating molecule with ITAM mo	362966	7	123842487	123861991	19505	29,55	-1,33	1,02E-02
ENSRNOG00000045535	**LOC67888**	similar to mammalian retrotransposon	679038	X	74428118	74429328	1211	1339,91	0,54	1,02E-02
ENSRNOG00000020518	**Fam189b**	family with sequence similarity 189, m	310640	2	207911887	207917765	5879	875,28	0,78	1,03E-02
ENSRNOG00000002981		voltage-dependent T-type calcium cha	29717	10	81951133	82017400	66268	1174,01	-0,86	1,05E-02
ENSRNOG00000004614	**Lypd6b**	LY6/PLAUR domain containing 6B (Ly	362133	3	40398135	40555551	157417	114,58	-0,93	1,08E-02
ENSRNOG00000048217	**Bbc3**	Bcl-2 binding component 3 (Bbc3), mF	317673	1	79520747	79521829	1083	699,53	0,58	1,08E-02
ENSRNOG00000015007	**Ski**	Protein RGD1565591 [Source:UniPro	313757	5	176018126	176083961	65836	1713,90	-0,86	1,08E-02
ENSRNOG00000016167	**Spata2L**	spermatogenesis associated 2-like (Sp	498963	19	66737483	66741950	4468	324,00	-0,69	1,08E-02
ENSRNOG00000027773		SMEK homolog 1, suppressor of mek1	314388	6	134261555	134302289	40735	924,13	-0,62	1,14E-02
ENSRNOG00000026447	**Ptchd2**	Protein Ptchd2 [Source:UniProtKB/Tr	313705	5	168710726	168741342	30617	425,50	-0,63	1,14E-02
ENSRNOG00000001211	**RGD13030**	homolog of zebrafish ES1 (RGD1303(	294326	20	13414047	13422143	8097	3047,74	0,55	1,17E-02
ENSRNOG00000005148	**Scrt2**	scratch homolog 2, zinc finger protein	366229	3	153934774	153946487	11714	205,85	1,20	1,17E-02
ENSRNOG00000008873	**Ino80b**	INO80 complex subunit B (Ino80b), m	500225	4	178639684	178643232	3549	688,75	0,66	1,17E-02
ENSRNOG00000019648	**Col6a3**	Protein Col6a3 [Source:UniProtKB/Tr	367313	9	97620136	97670009	49874	66,37	-1,16	1,17E-02

ENSRNOG00000016737	**Tcerg1l**	transcription elongation regulator 1-like	361669	1	217478056	217666989	188934	338,44	-0,82	1,19E-02
ENSRNOG00000045764	**Cxxc4**	CXXC finger protein 4 (Cxxc4), mRNA	83824	2	257941014	257965727	24714	1022,17	-0,72	1,19E-02
ENSRNOG00000049067	**Igip**	Protein Igip [Source:UniProtKB/TrEMI	1E+08	18	28831401	28832489	1089	119,29	-0,99	1,20E-02
ENSRNOG00000020061	**Tmem198**	Protein Tmem198; RCG23725, isoforr	1E+08	9	82444082	82448691	4610	1159,63	0,68	1,20E-02
ENSRNOG00000016207	**Galnt1**	UDP-N-acetyl-alpha-D-galactosamine	79214	18	15906881	15947004	40124	1891,85	-0,78	1,22E-02
ENSRNOG00000028895	**Rtp4**	receptor (chemosensory) transporter p	360733	11	80084427	80096284	11858	134,94	1,50	1,22E-02
ENSRNOG00000005479		excitatory amino acid transporter 2 iso	29482	3	99282853	99309441	26589	1815,35	-1,01	1,23E-02
ENSRNOG00000016388	**Sphkap**	SPHK1 interactor, AKAP domain conta	316561	9	88793220	88894918	101699	1115,48	-0,74	1,23E-02
ENSRNOG00000000701	**Iscu**	iron-sulfur cluster scaffold homolog (E	288740	12	50403338	50409181	5844	1814,97	0,71	1,23E-02
ENSRNOG00000023473	**RGD15612**	Protein RGD1561238 [Source:UniPro	502091	16	64425173	64429157	3985	111,33	0,86	1,23E-02
ENSRNOG00000003841	**Kcnh1**	potassium voltage-gated channel, sub	65198	13	115478550	115789683	311134	264,01	-1,12	1,25E-02
ENSRNOG00000000831	**Gtf2h4**	general transcription factor II H, polype	294236	20	5679700	5685350	5651	548,40	0,59	1,25E-02
ENSRNOG00000018644	**Slc6a7**	solute carrier family 6 (neurotransmitte	117100	18	55539473	55557598	18126	196,71	-0,93	1,34E-02
ENSRNOG00000016456		interleukin-33 precursor [Source:RefS	361749	1	255386570	255397660	11091	242,60	-1,16	1,34E-02
ENSRNOG00000014745		Protein LOC685778 [Source:UniProtK	685778	16	87742772	87744028	1257	73,63	-1,17	1,38E-02
ENSRNOG00000009571		WAS/WASL interacting protein family,	259242	4	149287016	149335996	48981	313,85	-1,06	1,40E-02
ENSRNOG00000000851		BCL2-associated athanogene 6 (Bag6	94342	20	7198938	7211343	12406	8311,65	0,56	1,41E-02
ENSRNOG00000008218	**Atp6v0e2**	ATPase, H+ transporting V0 subunit e	436582	4	142831842	142835730	3889	11447,74	0,66	1,41E-02
ENSRNOG00000019902	**Folr1**	folate receptor 1 (adult) (Folr1), mRNA	171049	1	173124247	173133377	9131	96,01	-1,51	1,43E-02
ENSRNOG00000024264	**Amz1**	archaelysin family metallopeptidase 1	304317	12	17975719	17994561	18843	277,63	-1,18	1,43E-02
ENSRNOG00000007804	**C1galt1**	core 1 synthase, glycoprotein-N-acety	65044	4	33766334	33796913	30580	124,90	-1,11	1,43E-02
ENSRNOG00000020233	**Rabac1**	Rab acceptor 1 (prenylated) (Rabac1)	83583	1	83095962	83099071	3110	5923,83	0,63	1,43E-02
ENSRNOG00000025424	**Yrdc**	yrdC domain containing (E.coli) (Yrdc)	319113	5	146615909	146620749	4841	1177,89	0,71	1,43E-02
ENSRNOG00000002190	**RGD15630**	Protein RGD1563065 [Source:UniPro	289633	14	45938969	46347907	408939	868,53	-1,15	1,45E-02
ENSRNOG00000030910	**Grik4**	glutamate receptor, ionotropic, kainate	24406	8	45279686	45568161	288476	474,97	-0,78	1,45E-02
ENSRNOG00000005504		Protein Pkp4 [Source:UniProtKB/TrEN	295625	3	50393961	50530402	136442	1916,16	-0,67	1,45E-02
ENSRNOG00000018674		neurotrophic tyrosine kinase, receptor,	29613	1	140868286	141239954	371669	706,05	-0,82	1,48E-02
ENSRNOG00000020588	**Efna4**	ephrin A4 (Efna4), mRNA [Source:Ref	310643	2	208065969	208070220	4252	319,99	0,64	1,48E-02
ENSRNOG00000004709	**Foxn3**	forkhead box N3 (Foxn3), mRNA [Sou	314374	6	132416694	132806362	389669	789,32	-0,85	1,49E-02
ENSRNOG00000016196	**Dlgap1**	discs, large (Drosophila) homolog-ass	65040	9	118045333	118613312	567980	1966,63	-0,78	1,49E-02
ENSRNOG00000002915	**Trim11**	tripartite motif-containing 11 (Trim11),	360534	10	45078796	45091444	12649	680,93	0,66	1,49E-02
ENSRNOG00000025463	**LOC10012**	expressed sequence AI593442 [Sourc	1E+08	8	55822041	55828684	6644	1944,17	-0,67	1,54E-02
ENSRNOG00000004473	**Ppargc1a**	peroxisome proliferator-activated rece	83516	14	63195104	63286096	90993	256,99	-0,96	1,57E-02
ENSRNOG00000015567		solute carrier family 9, subfamily A (NH	24783	9	47069438	47174866	105429	47,31	-1,38	1,59E-02
ENSRNOG00000004763	**Sirpa**	signal-regulatory protein alpha (Sirpa)	25528	3	127864146	127901869	37724	3152,86	-1,15	1,59E-02
ENSRNOG00000005024	**Cdkn2aipn**	CDKN2A interacting protein N-termina	287278	10	37195757	37205191	9435	1677,74	0,54	1,60E-02
ENSRNOG00000003878	**Thsd7b**	thrombospondin, type I, domain contai	289007	13	51235509	51990395	754887	277,36	-0,91	1,60E-02
ENSRNOG00000005109	**Rprm**	reprimo, TP53 dependent G2 arrest m	680110	3	44691990	44692319	330	998,06	0,77	1,61E-02

ENSRNOG00000043103	**Frrs1l**	DOMON domain-containing protein FF	366376	5	78154766	78183558	28793	132,34	-0,84	1,62E-02
ENSRNOG00000032576	**Nlgn1**	neuroligin 1 (Nlgn1), mRNA [Source:R	116647	2	130772394	131486085	713692	399,42	-1,07	1,64E-02
ENSRNOG00000004147		ATP-binding cassette, subfamily A (AE	303638	10	98033537	98099024	65488	310,34	-1,02	1,64E-02
ENSRNOG00000033162		acidic mammalian chitinase precursor	113901	2	228174327	228221354	47028	28,08	-1,45	1,70E-02
ENSRNOG00000020480	**Fads1**	fatty acid desaturase 1 (Fads1), mRN	84575	1	233178776	233193796	15021	7874,46	-1,01	1,70E-02
ENSRNOG00000014132	**Mypop**	Myb-related transcription factor, partne	499090	1	81159192	81169783	10592	284,65	0,72	1,75E-02
ENSRNOG00000011931	**Smarca2**	SWI/SNF related, matrix associated, a	361745	1	251867337	252034211	166875	3808,20	-0,57	1,76E-02
ENSRNOG00000010363		28S ribosomal protein S23, mitochond	360594	10	74570150	74577912	7763	1060,57	0,74	1,80E-02
ENSRNOG00000012671	**Gan**	gigaxonin (Gan), mRNA [Source:RefS	307893	19	60482638	60528944	46307	387,78	-0,62	1,81E-02
ENSRNOG00000013920	**Arhgap26**	Rho GTPase activating protein 26 (Arl	307459	18	31493583	31887220	393638	243,18	-0,80	1,81E-02
ENSRNOG00000015616	**Rgs14**	regulator of G-protein signaling 14 (Rg	114705	17	11887270	11901352	14083	218,10	-1,37	1,82E-02
ENSRNOG00000016322	**Camk2n1**	calcium/calmodulin-dependent protein	287005	5	160625616	160627396	1781	944,45	-1,06	1,82E-02
ENSRNOG00000007666	**Cacnb4**	calcium channel, voltage-dependent, b	58942	3	43054753	43191213	136461	243,80	-0,87	1,82E-02
ENSRNOG00000005159	**Fam135b**	Protein Fam135b [Source:UniProtKB/	315069	7	112609758	112768320	158563	232,08	-0,79	1,82E-02
ENSRNOG00000022802	**Tmem184b**	transmembrane protein 184B (Tmem1	362959	7	120586166	120629422	43257	1641,30	0,57	1,82E-02
ENSRNOG00000033646		G protein-coupled receptor 123 [Source:MGI Sy		1	219095294	219133257	37964	311,14	-0,93	1,82E-02
ENSRNOG00000006831	**Pgr**	progesterone receptor (Pgr), mRNA [S	25154	8	7113895	7172761	58867	57,57	-1,18	1,82E-02
ENSRNOG00000030712	**RT1-A**	RT1 class Ia, locus A2 (RT1-A2), mRN	24974	20	7433433	7436985	3553	713,53	1,37	1,83E-02
ENSRNOG00000000723	**RT1-CE5**	RT1 class I, locus CE5 (RT1-CE5), tra	309607	20	6968850	6973135	4286	171,29	1,15	1,84E-02
ENSRNOG00000023109	**Icoslg**	Protein Icoslg [Source:UniProtKB/TrE	499415	20	13502479	13510230	7752	63,90	-1,21	1,85E-02
ENSRNOG00000003572	**LOC10090**	ADAM metallopeptidase with thrombo	287899	10	35868734	36072447	203714	90,56	-1,32	1,85E-02
ENSRNOG00000010084	**Ythdf3**	YTH domain family, member 3 (Ythdf3	361920	2	120415579	120448585	33007	1215,50	-0,90	1,85E-02
ENSRNOG00000048405	**Scamp3**	secretory carrier membrane protein 3	65169	2	207905687	207911470	5784	3117,45	0,62	1,85E-02
ENSRNOG00000011338	**Cecr5**	cat eye syndrome chromosome regior	312680	4	220134732	220167528	32797	671,31	0,56	1,86E-02
ENSRNOG00000020646	**Sdhaf2**	succinate dehydrogenase complex as	361726	1	233493738	233518545	24808	730,52	-0,85	1,88E-02
ENSRNOG00000023809		opioid binding protein/cell adhesion molecule-lik		8	29724777	29974284	249508	2621,21	-0,69	1,88E-02
ENSRNOG00000042821	**Cd59**	CD59 molecule, complement regulato	25407	3	100650405	100667801	17397	790,64	-0,69	1,88E-02
ENSRNOG00000002793	**Sstr2**	somatostatin receptor 2 (Sstr2), mRN	54305	10	101816029	101823322	7294	316,67	-0,83	1,89E-02
ENSRNOG00000025151		Protein Ildr2 [Source:UniProtKB/TrEM	685277	13	89364845	89404282	39438	163,40	-0,73	1,90E-02
ENSRNOG00000017833	**Actn2**	actinin alpha 2 (Actn2), mRNA [Source	291245	17	68050946	68143522	92577	102,69	-0,90	1,92E-02
ENSRNOG00000000283	**Dgcr14**	DiGeorge syndrome critical region ger	360741	11	90315984	90324889	8906	770,85	0,62	1,92E-02
ENSRNOG00000004461	**Flywch2**	Protein LOC100910745; RCG33321, i	1E+08	10	12931766	12934651	2886	232,02	0,65	1,92E-02
ENSRNOG00000010267	**Klhdc10**	kelch domain containing 10 (Klhdc10),	312199	4	57477673	57531126	53454	1322,41	-0,67	1,94E-02
ENSRNOG00000016070	**Trpc3**	transient receptor potential cation chai	60395	2	142945396	143011755	66360	139,69	-1,14	2,00E-02
ENSRNOG00000023496		family with sequence similarity 189, m	680344	1	126661515	127069249	407735	223,92	-1,16	2,01E-02
ENSRNOG00000019201		NAD(P)H-hydrate epimerase [Source	295229	2	206827344	206829392	2049	1440,54	0,53	2,01E-02
ENSRNOG00000002215	**Mylk**	myosin light chain kinase (Mylk), mRN	288057	11	72103710	72313911	210202	107,46	-1,32	2,02E-02
ENSRNOG00000017439	**Cgnl1**	cingulin-like 1 (Cgnl1), mRNA [Source	315795	8	74802444	74948694	146251	88,23	-1,31	2,03E-02

ENSRNOG00000017197	**Pdgfb**	platelet-derived growth factor beta pol	24628	7	121205506	121223140	17635	111,79	-0,81	2,03E-02
ENSRNOG00000050933	**LOC68009**	Histone H4 Osteogenic growth peptide	64627	17	45677621	45677932	312	54,24	1,32	2,05E-02
ENSRNOG00000018316		GRB2-related adaptor protein 2 [Source:MGI Sy		7	121831634	121854018	22385	18,80	-1,34	2,08E-02
ENSRNOG00000020748		Microtubule-associated protein 4 [Source:UniPr		8	117366079	117515387	149309	7572,60	-0,69	2,08E-02
ENSRNOG00000031090	**LOC10036**	RT1 class I, locus CE7 (RT1-CE7), mR	368153	20	6718907	6779286	60380	199,79	1,32	2,08E-02
ENSRNOG00000017444	**Nrsn1**	neurensin 1 (Nrsn1), mRNA [Source:R	291129	17	43671713	43689288	17576	375,12	-0,98	2,15E-02
ENSRNOG00000017189	**Kcng2**	potassium voltage-gated channel, sub	307234	18	71933314	71999524	66211	71,12	-1,41	2,15E-02
ENSRNOG00000005592	**Brinp2**	bone morphogenetic protein/retinoic a	286895	13	80864668	80964493	99826	1532,42	-0,71	2,15E-02
ENSRNOG00000046330		ring finger protein 130 [Source:MGI Symbol;Acc		10	35306180	35372337	66158	629,11	-0,72	2,21E-02
ENSRNOG00000020107	**Stk11ip**	serine/threonine kinase 11 interacting	301535	9	82487970	82503147	15178	1359,62	0,85	2,21E-02
ENSRNOG00000018570	**C1qtnf3**	C1q and tumor necrosis factor related	294806	2	83741942	83764184	22243	31,76	-1,43	2,22E-02
ENSRNOG00000024796	**Lrrc47**	leucine rich repeat containing 47 (Lrrc	362672	5	174789385	174799217	9833	2499,50	0,63	2,22E-02
ENSRNOG00000039744		RT1 class I, locus CE4 (RT1-CE4), mR	414783	20	6994667	6997483	2817	64,99	1,31	2,22E-02
ENSRNOG00000020078	**Vstm4**	V-set and transmembrane domain con	361112	16	11056305	11135814	79510	79,03	-1,31	2,25E-02
ENSRNOG00000002347	**Rgl1**	ral guanine nucleotide dissociation stir	289080	13	74881865	75145652	263788	1243,00	-0,66	2,25E-02
ENSRNOG00000004755	**Commd9**	COMM domain containing 9 (Commd9	295956	3	98121989	98136421	14433	1805,35	0,53	2,25E-02
ENSRNOG00000016315	**Cnpy3**	canopy FGF signaling regulator 3 (Cnp	685174	9	15450330	15465055	14726	2212,56	0,54	2,27E-02
ENSRNOG00000002626	**Ca10**	Protein Ca10 [Source:UniProtKB/TrEI	1E+08	10	80633889	81150503	516615	53,13	-1,01	2,30E-02
ENSRNOG00000014010	**Gfra2**	GDNF family receptor alpha 2 (Gfra2),	25136	15	56280942	56372307	91366	226,86	-0,75	2,30E-02
ENSRNOG00000042523	**Ube2d2**	ubiquitin-conjugating enzyme E2D 2 (Ube2d2), r		18	28306950	28315393	8444	2047,43	-0,64	2,30E-02
ENSRNOG00000014751		proto-oncogene tyrosine-protein kinas	24716	4	216130625	216172348	41724	166,37	-1,26	2,30E-02
ENSRNOG00000010083	**Prpsap1**	phosphoribosyl pyrophosphate synthe	64390	10	105092064	105113772	21709	1661,50	0,54	2,30E-02
ENSRNOG00000007118	**Eva1a**	eva-1 homolog A (Eva1a), mRNA [Sou	500221	4	177462081	177511382	49302	44,72	-1,40	2,33E-02
ENSRNOG00000003704	**Fam184b**	Protein Fam184b [Source:UniProtKB/	289671	14	70013085	70127477	114393	284,28	-0,73	2,34E-02
ENSRNOG00000020663	**Pygo2**	pygopus 2 (Pygo2), mRNA [Source:Re	295251	2	208171771	208176564	4794	2406,79	0,54	2,36E-02
ENSRNOG00000001465		iduronate 2-sulfatase [Source:MGI Symbol;Acc:I		8	69158390	69174456	16067	4336,51	-0,60	2,38E-02
ENSRNOG00000016161	**Lman2**	lectin, mannose-binding 2 (Lman2), m	290994	17	11907302	11925365	18064	2171,86	0,52	2,38E-02
ENSRNOG00000050638		Uncharacterized protein [Source:UniProtKB/TrE		6	114195301	114196932	1632	492,06	-0,92	2,38E-02
ENSRNOG00000007467	**Ace**	angiotensin I converting enzyme (pept	24310	10	93922062	93957568	35507	70,67	-1,13	2,39E-02
ENSRNOG00000002783	**Serpinc1**	serpin peptidase inhibitor, clade C (an	304917	13	83700732	83727844	27113	48,08	-1,03	2,39E-02
ENSRNOG00000022804	**Foxp4**	forkhead box P4 (Foxp4), mRNA [Sou	363185	9	13993012	14049324	56313	963,59	0,74	2,39E-02
ENSRNOG00000030597	**Ankrd52**	ankyrin repeat domain 52 (Ankrd52), r	362811	7	2774352	2788002	13651	2920,62	0,79	2,39E-02
ENSRNOG00000023410	**Apol9a**	apolipoprotein L 9a (Apol9a), mRNA [S	503164	7	118924874	118931634	6761	84,02	1,41	2,39E-02
ENSRNOG00000003260	**Nr1i3**	nuclear receptor subfamily 1, group I,	65035	13	94213148	94218142	4995	119,01	-0,76	2,40E-02
ENSRNOG00000008976		cadherin 4 [Source:MGI Symbol;Acc:MGI:99218		3	181834313	181967627	133315	976,91	-1,08	2,46E-02
ENSRNOG00000007871	**Cabp7**	calcium binding protein 7 (Cabp7), mR	360970	14	85644750	85655064	10315	171,78	-1,30	2,47E-02
ENSRNOG00000014096	**Nr3c1**	nuclear receptor subfamily 3, group C, member		18	31408742	31430004	21263	701,61	-0,84	2,47E-02
ENSRNOG00000026055	**Neurod6**	neuronal differentiation 6 (Neurod6), n	500137	4	150564266	150567512	3247	2983,80	-0,72	2,48E-02

ENSRNOG00000020117	**LOC10091**	breast cancer metastasis-suppressor	293668	1	227256375	227265638	9264	44,28	0,98	2,48E-02
ENSRNOG00000013305		ATPase, Ca++ transporting, type 2C,	170699	8	113331596	113398299	66704	3739,61	-0,70	2,50E-02
ENSRNOG00000001449	**Pom121**	POM121 transmembrane nucleoporin	113975	12	26321893	26339241	17349	2747,79	0,59	2,50E-02
ENSRNOG00000016368	**Ppp1r14c**	protein phosphatase 1, regulatory (inh	171010	1	41159898	41272168	112271	1833,90	-0,76	2,50E-02
ENSRNOG00000003398	**Tomm40l**	translocase of outer mitochondrial me	304971	13	94218782	94221471	2690	256,29	-0,62	2,50E-02
ENSRNOG00000048025	**Cstf2**	cleavage stimulation factor, 3' pre-RN	683927	X	104716668	104742710	26043	1429,73	-0,57	2,52E-02
ENSRNOG00000013578	**Trem2**	triggering receptor expressed on myel	301227	9	13532920	13539372	6453	18,67	-1,41	2,53E-02
ENSRNOG00000033169	**Cpeb4**	cytoplasmic polyadenylation element	303010	10	15882113	15940027	57915	2033,20	-0,69	2,54E-02
ENSRNOG00000009323	**Fam214b**	family with sequence similarity 214, m	298201	5	63002711	63010762	8052	1596,43	0,53	2,54E-02
ENSRNOG00000049497	**Eif6**	eukaryotic translation initiation factor	305506	3	157722079	157728326	6248	2308,06	0,56	2,54E-02
ENSRNOG00000025061	**RGD13081**	LOC361719 (RGD1308106), mRNA [S	361719	1	229594278	229616177	21900	1298,23	0,67	2,54E-02
ENSRNOG00000005932	**Megf9**	multiple EGF-like-domains 9 (Megf9),	313270	5	90674194	90783779	109586	1376,10	-0,68	2,57E-02
ENSRNOG00000012549	**Hs2st1**	heparan sulfate 2-O-sulfotransferase	292155	2	268996211	269129114	132904	2723,08	-0,58	2,61E-02
ENSRNOG00000011680	**Il16**	interleukin 16 (Il16), mRNA [Source:R	116996	1	146710947	146799931	88985	39,11	-1,35	2,63E-02
ENSRNOG00000038980	**Lypd6**	Protein Lypd6; RCG37726, isoform CF	679564	3	40794415	40818938	24524	84,66	-1,01	2,63E-02
ENSRNOG00000003217	**Lgals3bp**	lectin, galactoside-binding, soluble, 3	245955	10	107050332	107059670	9339	642,23	1,22	2,63E-02
ENSRNOG00000006513	**LOC49793**	RIKEN cDNA 2310047M10 gene [Sou	497934	10	55385181	55387034	1854	574,13	0,67	2,67E-02
ENSRNOG00000037957	**Aifm3**	apoptosis-inducing factor, mitochondri	303786	11	90417093	90431999	14907	35,05	-1,19	2,71E-02
ENSRNOG00000003149	**Farsa**	phenylalanyl-tRNA synthetase, alpha	288917	19	36944878	36954400	9523	1952,43	0,49	2,71E-02
ENSRNOG00000007636	**Cadps2**	Protein Cadps2 [Source:UniProtKB/T	312166	4	50109970	50640382	530413	415,61	-0,71	2,73E-02
ENSRNOG00000029866	**Dennd6b**	DENN/MADD domain containing 6B (I	362983	7	129850713	129862537	11825	557,62	0,65	2,74E-02
ENSRNOG00000026271	**Tmem245**	Transmembrane protein 245 [Source:	298020	5	78073998	78141161	67164	482,16	-0,70	2,76E-02
ENSRNOG00000008683	**Alk**	anaplastic lymphoma receptor tyrosine	266802	6	32580734	33082961	502228	116,58	-0,77	2,80E-02
ENSRNOG00000005124	**Plekhh2**	pleckstrin homology domain containin	313866	6	7731647	7830438	98792	110,88	-0,79	2,83E-02
ENSRNOG00000016088	**Cdk10**	cyclin-dependent kinase 10 (Cdk10), t	361434	19	66729784	66737478	7695	1070,86	0,52	2,83E-02
ENSRNOG00000015583	**Dnal4**	dynein, axonemal, light chain 4 (Dnal4	294376	7	120978397	120991231	12835	1142,44	0,61	2,85E-02
ENSRNOG00000018247	**Dhx58**	DEXH (Asp-Glu-X-His) box polypeptid	303538	10	88393941	88405108	11168	160,42	1,34	2,91E-02
ENSRNOG00000033627	**Panx2**	pannexin 2 (Panx2), mRNA [Source:R	362979	7	129728892	129738977	10086	969,28	-0,85	2,97E-02
ENSRNOG00000013042	**Htr1b**	5-hydroxytryptamine (serotonin) recep	25075	8	88666819	88667979	1161	62,44	-0,90	2,98E-02
ENSRNOG00000000489	**Nudt3**	nudix (nucleoside diphosphate linked	294292	20	9358099	9410646	52548	7739,81	0,45	2,98E-02
ENSRNOG00000018498	**Hectd2**	HECT domain containing E3 ubiquitin	309514	1	262443066	262510127	67062	278,59	-0,85	2,98E-02
ENSRNOG00000033528	**Tll1**	tolloid-like 1 (Tll1), mRNA [Source:Ref	678743	16	27269834	27466134	196301	50,42	-1,00	3,00E-02
ENSRNOG00000008196	**Parp12**	Poly (ADP-ribose) polymerase family,	362343	4	66654422	66704543	50122	293,52	1,06	3,00E-02
ENSRNOG00000008276	**Adcy1**	adenylate cyclase 1 (brain) (Adcy1), m	305509	14	80947153	81055801	108649	4273,64	-0,83	3,00E-02
ENSRNOG00000007328	**Mrpl50**	mitochondrial ribosomal protein L50 (M	362517	5	69275963	69280730	4768	312,57	-0,78	3,01E-02
ENSRNOG00000010067	**Lypla2**	lysophospholipase II (Lypla2), mRNA [	83510	5	158079858	158084485	4628	2707,02	0,65	3,05E-02
ENSRNOG00000011323	**Lgi3**	leucine-rich repeat LGI family, membe	306013	15	55944095	55951223	7129	124,22	-1,16	3,07E-02
ENSRNOG00000023538	**Aldh5a1**	aldehyde dehydrogenase 5 family, me	291133	17	44000891	44023629	22739	791,84	-0,82	3,07E-02

ENSRNOG00000000705		uncharacterized protein LOC360824 [	360824	12	50604426	50644721	40296	189,41	-0,83	3,08E-02
ENSRNOG00000029292	**LOC50087**	Ab1-152 (LOC500877), mRNA [Sourc	500877	7	101676672	101706856	30185	32,72	-1,12	3,08E-02
ENSRNOG00000022216	**Abr**	active BCR-related (Abr), mRNA [Sou	287537	10	63306286	63449250	142965	7098,19	-0,67	3,19E-02
ENSRNOG00000005978		double C2-like domain-containing prot	81820	10	64054072	64078438	24367	349,86	-0,82	3,19E-02
ENSRNOG00000020675	**Ccdc97**	coiled-coil domain containing 97 (Ccd	292724	1	83764847	83772603	7757	1814,18	0,46	3,26E-02
ENSRNOG00000013642	**Lrrc41**	leucine rich repeat containing 41 (Lrrc	362566	5	138711342	138732260	20919	2941,20	0,46	3,26E-02
ENSRNOG00000007582	**Zswim4**	zinc finger, SWIM-type containing 4 (Z	304655	19	36296871	36321978	25108	1270,54	0,64	3,29E-02
ENSRNOG00000046276	**Myh3**	myosin, heavy chain 3, skeletal muscl	24583	10	53372408	53396227	23820	18,94	-1,37	3,31E-02
ENSRNOG00000042905	**RT1-T24-4**	RT1 class I, locus T24, gene 4 (RT1-T	414784	20	5287623	5295621	7999	58,19	1,26	3,34E-02
ENSRNOG00000000886	**Caln1**	calneuron 1 (Caln1), mRNA [Source:R	363909	12	31252691	31680890	428200	362,76	-0,88	3,41E-02
ENSRNOG00000003357	**Col3a1**	collagen, type III, alpha 1 (Col3a1), ml	84032	9	51689492	51725414	35923	210,27	-1,20	3,44E-02
ENSRNOG00000004317	**Vipr2**	vasoactive intestinal peptide receptor :	29555	6	152876207	152945274	69068	41,00	-1,09	3,44E-02
ENSRNOG00000033099	**Dcc**	deleted in colorectal carcinoma (Dcc),	25311	18	65693812	66392627	698816	483,69	-0,81	3,44E-02
ENSRNOG00000019560	**Pde2a**	phosphodiesterase 2A, cGMP-stimula	81743	1	172724783	172816712	91930	3520,32	-0,76	3,44E-02
ENSRNOG00000014971	**Mas1**	MAS1 oncogene (Mas1), mRNA [Sour	25153	1	51644722	51675365	30644	55,48	-1,21	3,45E-02
ENSRNOG00000001368	**Rph3a**	rabphilin 3A (Rph3a), mRNA [Source:I	171039	12	42939357	43014054	74698	1660,43	-0,94	3,52E-02
ENSRNOG00000012302	**Gucy1a3**	guanylate cyclase 1, soluble, alpha 3 (	497757	2	200453505	200514967	61463	3211,12	-0,73	3,53E-02
ENSRNOG00000042603	**Ttc39b**	tetratricopeptide repeat domain 39B (T	298186	5	105326437	105422721	96285	117,71	-0,84	3,56E-02
ENSRNOG00000003611	**Dynlt3**	dynein light chain Tctex-type 3 (Dynlt3	363448	X	15414615	15423884	9270	454,27	-0,94	3,58E-02
ENSRNOG00000018285	**Kcna2**	potassium voltage-gated channel, sha	25468	2	229304210	229308358	4149	253,39	-0,66	3,58E-02
ENSRNOG00000005985	**Kcnma1**	potassium large conductance calcium-	83731	15	339099	1012524	673426	445,44	-0,89	3,61E-02
ENSRNOG00000021478	**Tpd52l1**	tumor protein D52-like 1 (Tpd52l1), ml	689256	1	29910327	30031363	121037	86,19	-0,78	3,61E-02
ENSRNOG00000017766	**Car12**	carbonic anyhydrase 12 (Car12), mRN	363085	8	72072135	72125119	52985	22,75	-1,24	3,61E-02
ENSRNOG00000016385	**Gabrd**	gamma-aminobutyric acid (GABA) A re	29689	5	176255724	176267624	11901	68,89	-1,03	3,61E-02
ENSRNOG00000014479	**Cttnbp2nl**	CTTNBP2 N-terminal like (Cttnbp2nl),	310760	2	226917956	226964542	46587	1508,78	-0,69	3,61E-02
ENSRNOG00000046947	**Ak5**	similar to adenylate kinase 5 isoform 1	365985	2	276349770	276531713	181944	863,99	-0,85	3,66E-02
ENSRNOG00000046527		PHD finger protein 20-like protein 1 [S	314964	7	108561906	108700209	138304	842,18	-0,68	3,66E-02
ENSRNOG00000004516	**Itgbl1**	integrin, beta-like 1 (Itgbl1), mRNA [Sc	498564	15	113495871	113759207	263337	41,32	-1,22	3,69E-02
ENSRNOG00000033026	**Dclk3**	doublecortin-like kinase 3 (Dclk3), mR	316023	8	119040830	119093605	52776	107,90	-1,19	3,69E-02
ENSRNOG00000003729	**Drg2**	Protein Drg2; RCG32787 [Source:Uni	497915	10	46573720	46587107	13388	1399,59	0,51	3,69E-02
ENSRNOG00000050646	**Fem1a**	fem-1 homolog a (C. elegans) (Fem1a	316131	9	9790762	9792726	1965	1441,07	0,60	3,69E-02
ENSRNOG00000050795				2	58069136	58069479	344	142,80	-0,78	3,77E-02
ENSRNOG00000032922	**Dclk1**	doublecortin-like kinase 1 (Dclk1), trar	83825	2	164277278	164355201	77924	17412,81	-0,51	3,77E-02
ENSRNOG00000036829	**Nckap1l**	NCK associated protein 1 like (Nckap1	315348	7	142849494	142894585	45092	20,23	-1,33	3,77E-02
ENSRNOG00000006876	**Msx1**	msh homeobox 1 (Msx1), mRNA [Sou	81710	14	77690990	77694787	3798	137,82	-1,01	3,77E-02
ENSRNOG00000010349	**Ghrl**	ghrelin/obestatin prepropeptide (Ghrl),	59301	4	208970556	208974465	3910	68,06	-0,87	3,77E-02
ENSRNOG00000017265	**Tmem131**	Protein Tmem131 [Source:UniProtKB	316335	9	43006795	43103045	96251	2587,69	-0,61	3,79E-02
ENSRNOG00000016576	**Lrrc16a**	leucine rich repeat containing 16A (Lrr	306941	17	44916816	45196987	280172	1545,35	-0,80	3,80E-02

ENSRNOG00000024801	**Slc35a2**	solute carrier family 35 (UDP-galactos	1E+08	X	16234421	16242948	8528	1215,49	0,67	3,80E-02
ENSRNOG00000047574	**Nrxn3**	neurexin 3 (Nrxn3), mRNA [Source:RefSeq mRN		6	123170183	123337131	166949	592,47	-0,82	3,83E-02
ENSRNOG00000001052		solute carrier family 25, member 30 [Source:MG		15	61519038	61534942	15905	506,70	-0,68	3,88E-02
ENSRNOG00000017235	**Atp6v0d1**	ATPase, H+ transporting, lysosomal V	291969	19	48347545	48391661	44117	9910,75	0,48	3,90E-02
ENSRNOG00000014336	**Mcm5**	minichromosome maintenance comple	291885	19	25637492	25681915	44424	948,52	0,65	3,92E-02
ENSRNOG00000025110	**Vwa3a**	von Willebrand factor A domain contai	293449	1	197606342	197667768	61427	111,12	-1,06	3,92E-02
ENSRNOG00000014798	**RGD13095**	UPF0705 protein C11orf49 homolog [	295930	3	86803733	87050021	246289	2647,34	0,50	3,93E-02
ENSRNOG00000021157		cathepsin S [Source:MGI Symbol;Acc:MGI:1073		2	217144872	217169058	24187	29,29	-1,33	3,94E-02
ENSRNOG00000002908	**Slc35a4**	solute carrier family 35, member A4 (S	257647	18	29211202	29214370	3169	2326,13	0,51	3,96E-02
ENSRNOG00000002827	**Rbfox1**	RNA binding protein, fox-1 homolog (C	302920	10	7109396	7450683	341288	1945,76	-0,62	3,97E-02
ENSRNOG00000020694	**Icam5**	intercellular adhesion molecule 5, tele	313785	8	22107090	22114077	6988	784,46	-0,80	3,97E-02
ENSRNOG00000015741	**Slc2a13**	solute carrier family 2 (facilitated gluco	171147	7	132108134	132430525	322392	290,79	-0,73	3,97E-02
ENSRNOG00000047102	**Popdc3**	popeye domain containing 3 (Popdc3)	641520	20	52006183	52033990	27808	34,64	-1,09	3,98E-02
ENSRNOG00000003829	**Ap3s1**	adaptor-related protein complex 3, sig	302290	18	40378507	40443852	65346	776,61	-0,62	3,98E-02
ENSRNOG00000048213		Protein LOC678880 [Source:UniProtK	678880	X	74394343	74394696	354	358,35	0,57	3,99E-02
ENSRNOG00000010378	**Slc4a5**	solute carrier family 4, sodium bicarbo	297386	4	179507406	179590667	83262	23,11	-1,33	3,99E-02
ENSRNOG00000009967	**Otof**	Otoferlin [Source:UniProtKB/Swiss-Pr	84573	6	37140887	37236346	95460	31,11	-1,33	4,00E-02
ENSRNOG00000017332	**Dapk2**	death-associated protein kinase 2 (Da	300799	8	71583273	71610443	27171	18,25	-1,30	4,00E-02
ENSRNOG00000000582	**LOC10091**	D-aspartate oxidase (Ddo), mRNA [Sc	685325	20	50384344	50403396	19053	29,03	-1,23	4,02E-02
ENSRNOG00000032569	**Lingo3**	leucine rich repeat and Ig domain cont	690755	7	11905008	11914418	9411	196,85	-0,88	4,05E-02
ENSRNOG00000018457	**Ppp2r4**	protein phosphatase 2A activator, regu	362102	3	14334896	14365358	30463	6992,28	0,62	4,05E-02
ENSRNOG00000017071	**Babam1**	BRISC and BRCA1 A complex membe	290631	16	19652971	19658999	6029	1572,65	0,64	4,05E-02
ENSRNOG00000011977	**Sema5a**	sema domain, seven thrombospondin	310207	2	105049343	105480370	431028	716,55	-1,09	4,06E-02
ENSRNOG00000046863	**C1ql2**	complement component 1, q subcomp	288979	13	41484469	41486004	1536	27,23	-1,08	4,06E-02
ENSRNOG00000018276	**Mgat4a**	mannosyl (alpha-1,3-)-glycoprotein be	367252	9	43734664	43828381	93718	336,55	-0,81	4,06E-02
ENSRNOG00000011803	**Lrfn2**	leucine rich repeat and fibronectin type	316205	9	12776272	12807638	31367	566,54	-0,80	4,06E-02
ENSRNOG00000014938	**Gabrb3**	gamma-aminobutyric acid (GABA) A re	24922	1	114022939	114275043	252105	1753,61	-0,65	4,06E-02
ENSRNOG00000002989	**Nmt1**	N-myristoyltransferase 1 (Nmt1), mRN	259274	10	90898210	90933049	34840	2245,99	0,47	4,06E-02
ENSRNOG00000013514	**Maf1**	MAF1 homolog (S. cerevisiae) (Maf1),	315093	7	117405317	117408349	3033	3871,96	0,47	4,06E-02
ENSRNOG00000017949	**Mvb12a**	multivesicular body subunit 12A (Mvb1	290635	16	19833920	19838783	4864	723,10	0,58	4,06E-02
ENSRNOG00000021403	**Rhob**	ras homolog family member B (Rhob),	64373	6	43473992	43474582	591	2254,78	0,67	4,06E-02
ENSRNOG00000049380		Protein LOC100360601 [Source:UniP	1E+08	11	37478384	37484677	6294	112,93	0,75	4,06E-02
ENSRNOG00000010529	**Thbs2**	thrombospondin 2 (Thbs2), mRNA [Sc	292406	1	57842346	57871988	29643	59,91	-1,21	4,06E-02
ENSRNOG00000010886	**Nfe2l3**	Protein Nfe2l3 [Source:UniProtKB/TrE	312331	4	145871588	145898997	27410	21,36	-1,15	4,06E-02
ENSRNOG00000003268	**Maml1**	mastermind like 1 (Drosophila) (Maml1	303101	10	35527493	35561786	34294	767,10	0,48	4,06E-02
ENSRNOG00000017641	**Rnf44**	ring finger protein 44 (Rnf44), mRNA [	361212	17	12589090	12596028	6939	1700,45	0,71	4,06E-02
ENSRNOG00000002322	**RGD13105**	Uncharacterized protein C1orf115 hon	360894	13	107456595	107466329	9735	35,72	-1,10	4,12E-02
ENSRNOG00000003553	**Efemp1**	EGF-containing fibulin-like extracellula	305604	14	112892387	112984799	92413	62,83	-1,32	4,14E-02

ENSRNOG00000007019	**Asic2**	acid-sensing (proton-gated) ion chann	25364	10	67916010	68180348	264339	913,39	-0,66	4,14E-02
ENSRNOG00000020495	**Eif3k**	eukaryotic translation initiation factor 3	292762	1	89103168	89108197	5030	1329,35	0,50	4,14E-02
ENSRNOG00000016588	**Myadm**	myeloid-associated differentiation mar	369016	1	63430413	63438759	8347	928,73	0,55	4,16E-02
ENSRNOG00000018865	**Adamts12**	ADAM metallopeptidase with thrombo:	294809	2	83352798	83650251	297454	141,08	-1,08	4,19E-02
ENSRNOG00000040166	**Ankrd34b**	ankyrin repeat domain 34B (Ankrd34b	499506	2	41203747	41219671	15925	116,80	-0,69	4,21E-02
ENSRNOG00000018168	**Klc4**	kinesin light chain 4 (Klc4), mRNA [So	316226	9	15554537	15568069	13533	1120,62	0,61	4,21E-02
ENSRNOG00000049361		growth arrest specific 7 (Gas7), mRNA	85246	10	53885814	53988130	102317	10298,60	-0,65	4,23E-02
ENSRNOG00000008026	**C2cd4c**	C2 calcium-dependent domain contair	500798	7	13142884	13145045	2162	296,54	-0,87	4,25E-02
ENSRNOG00000025612	**Sez6l**	seizure related 6 homolog (mouse)-like	304554	12	51848836	51998895	150060	3150,01	-0,76	4,25E-02
ENSRNOG00000023540	**Pygo1**	pygopus 1 (Pygo1), mRNA [Source:Re	691857	8	73464585	73482239	17655	234,66	-0,68	4,25E-02
ENSRNOG00000013343	**Myg1**	melanocyte proliferating gene 1 (Myg1	300258	7	141725674	141732906	7233	1053,26	0,51	4,27E-02
ENSRNOG00000004660	**Fzd6**	frizzled family receptor 6 (Fzd6), mRN	282581	7	78317823	78349530	31708	53,14	-1,12	4,28E-02
ENSRNOG00000013742	**Large**	like-glycosyltransferase (Large), mRN.	361368	19	23595328	24054765	459438	3805,87	-0,77	4,28E-02
ENSRNOG00000039464	**Tmem229a**	transmembrane protein 229A (Tmem2	685756	4	52115167	52120186	5020	1448,77	-1,02	4,29E-02
ENSRNOG00000009414	**Creld1**	cysteine-rich with EGF-like domains 1	312638	4	208737583	208747189	9607	1841,17	0,60	4,29E-02
ENSRNOG00000015736	**Dhrs3**	dehydrogenase/reductase (SDR family	313689	5	166490225	166524733	34509	149,37	-1,06	4,38E-02
ENSRNOG00000042833		predicted gene 884 [Source:MGI Symbol;Acc:M(		10	91377230	91433099	55870	286,40	-0,80	4,42E-02
ENSRNOG00000045738	**Ak4**	adenylate kinase 4 (Ak4), mRNA [Sou	29223	5	124130328	124189158	58831	596,87	-0,78	4,42E-02
ENSRNOG00000003224	**Nudt16l1**	nudix (nucleoside diphosphate linked r	497867	10	9596797	9598729	1933	2380,86	0,49	4,54E-02
ENSRNOG00000048264	**LOC68482**	histone cluster 1, H1d [Source:MGI Sy	684828	17	45494208	45655399	161192	567,26	0,62	4,54E-02
ENSRNOG00000050183	**RT1-CE1**	RT1 class I, locus1 (RT1-CE1), mRNA	309600	20	6851806	6872656	20851	121,17	1,22	4,54E-02
ENSRNOG00000002509	**Gnl3l**	guanine nucleotide binding protein-like	1E+08	X	20785115	20818062	32948	2095,80	-0,62	4,54E-02
ENSRNOG00000020499	**Scaf1**	SR-related CTD-associated factor 1 (S	56081	1	102062545	102073215	10671	4911,28	0,51	4,57E-02
ENSRNOG00000006312	**Zfp202**	zinc finger protein 202 (Zfp202), mRN.	500981	8	61469449	61489701	20253	224,53	-0,65	4,58E-02
ENSRNOG00000023770	**Tcfl5**	Protein Tcfl5 [Source:UniProtKB/TrEM	311715	3	179826168	179845122	18955	132,81	-0,91	4,64E-02
ENSRNOG00000008897	**Gga1**	golgi associated, gamma adaptin ear c	300066	7	120098422	120111258	12837	1834,24	0,46	4,69E-02
ENSRNOG00000004140	**Pacrgl**	Protein Pacrgl; Similar to RIKEN cDNA	360947	14	66781647	66802061	20415	101,90	-0,77	4,70E-02
ENSRNOG00000003470	**Fundc1**	FUN14 domain containing 1 (Fundc1),	363442	X	6329908	6346563	16656	416,87	-0,74	4,70E-02
ENSRNOG00000031700	**Map3k5**	mitogen-activated protein kinase kinas	365057	1	16961080	17160990	199911	226,75	-0,92	4,70E-02
ENSRNOG00000001418	**Znhit1**	zinc finger, HIT-type containing 1 (Znh	1E+08	12	24732249	24744501	12253	690,61	0,65	4,70E-02
ENSRNOG00000003520	**Prdx2**	peroxiredoxin 2 (Prdx2), mRNA [Sourc	29338	19	37060473	37065716	5244	13602,59	0,53	4,72E-02
ENSRNOG00000010422	**Kcnab1**	potassium voltage-gated channel, sha	29737	2	175102652	175398300	295649	566,79	-0,63	4,74E-02
ENSRNOG00000002934	**Atp1b1**	ATPase, Na+/K+ transporting, beta 1 p	25650	13	87621449	87641967	20519	11378,64	-0,69	4,76E-02
ENSRNOG00000019735	**Dph2**	DPH2 homolog (S. cerevisiae) (Dph2)	298452	5	140512447	140515407	2961	596,63	0,56	4,76E-02
ENSRNOG00000019958	**Tmem151b**	transmembrane protein 151B (Tmem1	301253	9	16732153	16734756	2604	1277,97	0,81	4,77E-02
ENSRNOG00000010806	**Npvf**	neuropeptide VF precursor (Npvf), mR	60570	4	145061114	145064806	3693	29,22	-1,24	4,79E-02
ENSRNOG00000011300		laminin, alpha 3 [Source:MGI Symbol;	307582	18	3867377	3939230	71854	30,39	-1,03	4,79E-02
ENSRNOG00000029528		cystathionine beta synthase (Cbs), mF	24250	20	12550129	12574252	24124	302,47	-0,98	4,80E-02

ENSRNOG00000019468	**Rce1**	RCE1 homolog, prenyl protein proteas	309153	1	226727307	226730406	3100	887,56	0,61	4,85E-02
ENSRNOG00000006957	**Gria4**	glutamate receptor, ionotropic, AMPA 4 (Gria4), I		8	1542037	2069626	527590	1023,09	-0,67	4,87E-02
ENSRNOG00000001171	**Coq5**	coenzyme Q5 homolog, methyltransfe	304542	12	48872069	48888982	16914	785,30	0,61	4,87E-02
ENSRNOG00000017553	**Smcr7l**	Smith-Magenis syndrome chromosom	315141	7	121458674	121463161	4488	621,98	0,51	4,89E-02
ENSRNOG00000001222	**Ube2g2**	ubiquitin-conjugating enzyme E2G 2 (I	294331	20	13866883	13887894	21012	1259,97	0,55	4,89E-02
ENSRNOG00000020531	**Fen1**	flap structure-specific endonuclease 1	84490	1	233196202	233200570	4369	448,95	0,66	4,89E-02
ENSRNOG00000038784		piezo-type mechanosensitive ion chan	307380	18	57577565	57948727	371163	20,51	-1,17	4,94E-02
ENSRNOG00000017163	**Pfkp**	phosphofructokinase, platelet (Pfkp), r	60416	17	70227568	70290412	62845	1611,87	-0,46	4,94E-02
ENSRNOG00000007175	**Mier1**	mesoderm induction early response 1,	313418	5	126512781	126559155	46375	829,92	-0,75	4,97E-02
ENSRNOG00000003848	**Med12**	mediator complex subunit 12 (Med12)	679693	X	72027347	72050290	22944	867,70	-0,71	4,97E-02
ENSRNOG00000004060	**Calm1**	calmodulin 1 (Calm1), mRNA [Source:	24242	6	133444274	133454391	10118	33181,14	-0,65	4,97E-02
ENSRNOG00000002428	**Lpar4**	lysophosphatidic acid receptor 4 (Lpar	302378	X	78238142	78251630	13489	145,66	-1,06	4,99E-02
ENSRNOG00000004537	**Rxrg**	retinoid X receptor gamma (Rxrg), mR	83574	13	90460380	90511040	50661	158,02	-0,70	4,99E-02
ENSRNOG00000017858	**Tsr3**	TSR3, 20S rRNA accumulation (Tsr3),	360494	10	14415604	14418166	2563	1112,27	0,58	5,01E-02
ENSRNOG00000024239	**Fam89b**	family with sequence similarity 89, me	309170	1	228018858	228020412	1555	4704,50	0,84	5,05E-02
ENSRNOG00000020016	**LOC10036**	late endosomal/lysosomal adaptor, MA	308869	1	173182228	173187799	5572	477,76	0,51	5,09E-02
ENSRNOG00000020845	**Tyrobp**	Tyro protein tyrosine kinase binding pr	361537	1	90031117	90034967	3851	24,72	-1,25	5,09E-02
ENSRNOG00000005360	**Dhrs7b**	dehydrogenase/reductase (SDR famil	287380	10	46837698	46884558	46861	1084,65	0,52	5,11E-02
ENSRNOG00000024832	**Gpr158**	G protein-coupled receptor 158 (Gpr1	291352	17	89902429	90335831	433403	338,05	-0,53	5,12E-02
ENSRNOG00000012807	**C1qa**	complement component 1, q subcomp	298566	5	159023754	159026611	2858	83,70	-1,24	5,12E-02
ENSRNOG00000011054	**Laptm5**	lysosomal protein transmembrane 5 (L	89783	5	152749594	152771632	22039	37,76	-1,25	5,17E-02
ENSRNOG00000006051		Uncharacterized protein [Source:UniProtKB/TrE		15	1043193	1043501	309	54,86	-0,94	5,19E-02
ENSRNOG00000019129	**Fcgbp**	Fc fragment of IgG binding protein (Fc	1E+08	1	86220276	86263136	42861	67,42	0,85	5,19E-02
ENSRNOG00000028782	**RGD15654**	uncharacterized protein LOC500843 is	500843	7	64146261	64159524	13264	194,58	-0,69	5,19E-02
ENSRNOG00000042173	**Shisa9**		1E+08	10	1661444	1686478	25035	243,49	-0,95	5,19E-02
ENSRNOG00000009378	**Rpl4**	ribosomal protein L4 (Rpl4), mRNA [S	64302	8	68827907	68833042	5136	7603,76	0,62	5,19E-02
ENSRNOG00000000777	**RT1-S3**	RT1 class Ib, locus S3 (RT1-S3), mRN	294228	20	5274290	5276827	2538	447,12	1,28	5,21E-02
ENSRNOG00000005606	**Med1**	mediator complex subunit 1 (Med1), m	497991	10	85901212	85941797	40586	1688,38	-0,49	5,21E-02
ENSRNOG00000007021	**Zdhhc18**	zinc finger, DHHC-type containing 18 (	362613	5	155520908	155546083	25176	476,26	0,50	5,21E-02
ENSRNOG00000013520	**Mat2a**	methionine adenosyltransferase II, alp	171347	4	165067990	165073559	5570	2898,64	-1,02	5,21E-02
ENSRNOG00000016691	**Nadk2**	NAD kinase 2, mitochondrial (Nadk2),	365699	2	77550016	77592156	42141	684,14	-0,91	5,21E-02
ENSRNOG00000046390	**Tceal6**	Protein LOC679974; Protein Tceal3 [S	501628	2	122107325	122109244	1920	71,53	0,94	5,21E-02
ENSRNOG00000005726		protein piccolo isoform 2 [Source:Ref	56768	4	16428814	16643899	215086	2028,41	-0,84	5,23E-02
ENSRNOG00000011460	**RGD13099**	Protein RGD1309903 [Source:UniPro	292947	1	13988001	14063960	75960	1008,62	-0,55	5,25E-02
ENSRNOG00000023257	**Adamts9**	ADAM metallopeptidase with thrombo	312566	4	188409401	188604215	194815	477,73	-1,02	5,28E-02
ENSRNOG00000006542	**Atp6v0c**	ATPase, H+ transporting, lysosomal V	170667	10	13352992	13358042	5051	20261,37	0,54	5,28E-02
ENSRNOG00000009155	**Ndufs3**	NADH dehydrogenase (ubiquinone) F	295923	3	86430806	86437983	7178	2275,48	0,46	5,28E-02
ENSRNOG00000014592	**Zbtb46**	zinc finger and BTB domain containing	311718	3	180600938	180636854	35917	260,79	-0,70	5,28E-02

ENSRNOG00000000963	**Tmem132c**	Protein Tmem132c [Source:UniProtKl	363915	12	34713829	35029422	315594	141,52	-1,09	5,31E-02
ENSRNOG00000010551	**Lhx2**	LIM homeobox 2 (Lhx2), mRNA [Sour	296706	3	27870027	27887928	17902	2637,18	-0,60	5,31E-02
ENSRNOG00000013267	**Helz2**	Protein Pric285 [Source:UniProtKB/Tr	296474	3	180439964	180453229	13266	68,24	1,10	5,31E-02
ENSRNOG00000018641	**Camkv**	CaM kinase-like vesicle-associated (C	79011	8	116070039	116084385	14347	6116,58	-0,80	5,31E-02
ENSRNOG00000000456	**Psmb8**	proteasome (prosome, macropain) sul	24968	20	6070357	6073320	2964	159,17	1,18	5,32E-02
ENSRNOG00000020266	**Eef2**	eukaryotic translation elongation facto	29565	7	11568907	11574177	5271	48534,06	0,53	5,34E-02
ENSRNOG00000000816	**Ppp1r18**	protein phosphatase 1, regulatory sub	361790	20	5469936	5494142	24207	963,88	0,50	5,34E-02
ENSRNOG00000018382		inositol polyphosphate-4-phosphatase	116699	19	40508509	40860035	351527	91,25	-0,86	5,34E-02
ENSRNOG00000015406	**Pgm5**	phosphoglucomutase 5 (Pgm5), mRN.	679990	1	249856475	250039462	182988	47,96	-1,13	5,43E-02
ENSRNOG00000037080	**Adamts17**	Protein Adamts17 [Source:UniProtKB	293004	1	128878220	129192208	313989	199,93	-0,87	5,43E-02
ENSRNOG00000008738	**Tp53i11**	tumor protein p53 inducible protein 11	311209	3	88784314	88799475	15162	3703,21	0,56	5,43E-02
ENSRNOG00000010996	**Mob1b**	MOB kinase activator 1B (Mob1b), mF	360920	14	20870121	20902822	32702	664,34	-0,71	5,47E-02
ENSRNOG00000014789	**Ddn**	dendrin (Ddn), mRNA [Source:RefSeq	25113	X	114727118	114993826	266709	2488,99	-0,97	5,50E-02
ENSRNOG00000007038	**Cntn5**	contactin 5 (Cntn5), mRNA [Source:Re	114589	8	7793982	8501166	707185	706,68	-0,64	5,53E-02
ENSRNOG00000001872	**LOC10036**	RIKEN cDNA 4930451C15 gene [Sou	1E+08	11	90469499	90485776	16278	18,93	-1,20	5,56E-02
ENSRNOG00000016216	**Sft2d3**	SFT2 domain containing 3 (Sft2d3), m	364835	18	24407121	24407774	654	99,83	0,73	5,56E-02
ENSRNOG00000008626	**Manea**	mannosidase, endo-alpha (Manea), m	140808	5	45469290	45491135	21846	473,54	-1,10	5,57E-02
ENSRNOG00000006802	**Lrrn1**	leucine rich repeat neuronal 1 (Lrrn1),	500280	4	204141294	204144345	3052	1719,83	-0,60	5,63E-02
ENSRNOG00000007476	**Clmp**	CXADR-like membrane protein (Clmp)	286939	8	46020808	46148061	127254	1092,26	-0,91	5,63E-02
ENSRNOG00000007989	**Chst1**	carbohydrate (keratan sulfate Gal-6) s	295934	3	88201237	88216361	15125	1935,13	-0,76	5,63E-02
ENSRNOG00000013128	**Tmem179**	transmembrane protein 179 (Tmem17	314472	6	146081901	146092800	10900	1489,90	0,77	5,63E-02
ENSRNOG00000027645	**Syndig1l**	synapse differentiation inducing 1-like	685882	6	117134070	117155831	21762	83,90	-0,74	5,63E-02
ENSRNOG00000004535	**Kcng3**	potassium voltage-gated channel, sub	171011	6	6749225	6797238	48014	40,94	-1,10	5,69E-02
ENSRNOG00000004554	**Dcn**	decorin (Dcn), mRNA [Source:RefSeq	29139	7	38783714	38823738	40025	31,38	-1,23	5,73E-02
ENSRNOG00000009460	**Pdzd8**	PDZ domain containing 8 (Pdzd8), mF	308000	1	287818525	287876134	57610	1702,63	-0,47	5,73E-02
ENSRNOG00000011068	**Papss2**	3'-phosphoadenosine 5'-phosphosulfa	294103	1	258376175	258461336	85162	115,01	-1,19	5,73E-02
ENSRNOG00000045623	**Capn10**	calpain 10 (Capn10), mRNA [Source:F	63834	9	99767655	99776487	8833	1572,31	0,55	5,73E-02
ENSRNOG00000004117	**Kcnv1**	potassium channel, subfamily V, mem	60326	7	84030573	84037747	7175	188,27	-0,89	5,75E-02
ENSRNOG00000021181	**Sf3b4**	splicing factor 3b, subunit 4 (Sf3b4), m	295270	2	217799411	217804163	4753	2207,52	0,46	5,80E-02
ENSRNOG00000017516	**Bcl9**	Protein Bcl9 [Source:UniProtKB/TrEM	310704	2	218817615	218828968	11354	1705,88	-0,57	5,83E-02
ENSRNOG00000008081	**Ddx27**	DEAD (Asp-Glu-Ala-Asp) box polypep	362274	3	169927524	169946720	19197	1339,13	0,54	5,83E-02
ENSRNOG00000016653	**Ngef**	neuronal guanine nucleotide exchange	246217	9	94290729	94323295	32567	1154,65	-0,54	5,91E-02
ENSRNOG00000049388	**LOC68375**	RIKEN cDNA 1110059M19 gene [Sou	683753	X	131703447	131705128	1682	46,54	-1,21	5,91E-02
ENSRNOG00000006556	**Slc5a6**	solute carrier family 5 (sodium/multivit	170551	6	36500814	36512084	11271	1386,44	0,65	5,92E-02
ENSRNOG00000023576	**RGD13056**	similar to RIKEN cDNA 1500015O10 (	363225	9	50191737	50212523	20787	67,77	-1,08	5,92E-02
ENSRNOG00000028941	**Zbed3**	zinc finger, BED-type containing 3 (Zb	361881	2	44199667	44210609	10943	807,80	0,54	5,93E-02
ENSRNOG00000000498	**Anks1a**	ankyrin repeat and sterile alpha motif	309639	20	9686588	9840938	154351	1856,89	0,60	5,97E-02
ENSRNOG00000009300	**Tlk1**	tousled-like kinase 1 (Tlk1), mRNA [Sc	311118	3	63799371	63897975	98605	1183,40	-0,68	5,98E-02

ENSRNOG00000010545	**Mrap2**	melanocortin 2 receptor accessory prc	363112	8	94308773	94364872	56100	82,32	-0,76	5,98E-02
ENSRNOG00000014363	**Arhgef3**	Rho guanine nucleotide exchange fac	290541	16	2925678	2994155	68478	239,45	-0,99	5,98E-02
ENSRNOG00000019202	**PVR**	poliovirus receptor (PVR), mRNA [Sou	25066	1	82085299	82100950	15652	184,46	0,83	5,98E-02
ENSRNOG00000019478	**Irf9**	interferon regulatory factor 9 (Irf9), mF	305896	15	38173131	38178333	5203	694,19	1,16	5,98E-02
ENSRNOG00000027756	**RGD15601**	Protein RGD1560175 [Source:UniPro	303946	11	65542190	65581149	38960	404,89	-0,87	5,98E-02
ENSRNOG00000050585	**Pgam1**	phosphoglycerate mutase 1 (brain) (P	24642	1	268610676	268618285	7610	5481,79	0,52	5,98E-02
ENSRNOG00000009795	**Nfib**	nuclear factor I/B (Nfib), mRNA [Sourc	29227	5	104469935	104676353	206419	1153,18	-0,60	5,99E-02
ENSRNOG00000016695		72 kDa type IV collagenase precursor	81686	19	26630488	26658966	28479	387,24	0,67	5,99E-02
ENSRNOG00000029127	**Parp16**	poly (ADP-ribose) polymerase family,	315760	8	70401838	70424172	22335	106,38	-0,80	6,03E-02
ENSRNOG00000001890	**Txnrd2**	thioredoxin reductase 2 (Txnrd2), mRI	50551	11	89762307	89809957	47651	516,22	0,59	6,09E-02
ENSRNOG00000026775	**Pmpca**	peptidase (mitochondrial processing) a	296588	3	9195490	9203287	7798	1837,82	0,51	6,13E-02
ENSRNOG00000009183		SUZ RNA binding domain containing 1	500575	5	163350635	163375124	24490	3832,47	0,51	6,17E-02
ENSRNOG00000016403	**Olah**	oleoyl-ACP hydrolase (Olah), mRNA [	64669	17	80520857	80545393	24537	19,41	-1,24	6,17E-02
ENSRNOG00000023816	**Aph1a**	APH1A gamma secretase subunit (Ap	365872	2	217496482	217499990	3509	2992,49	0,42	6,17E-02
ENSRNOG00000002360	**Gabrg1**	gamma-aminobutyric acid (GABA) A re	140674	14	39777522	39858765	81244	422,56	-1,10	6,21E-02
ENSRNOG00000013078	**Zcchc7**	zinc finger, CCHC domain containing 7	298086	5	64759112	64938825	179714	243,20	-0,63	6,21E-02
ENSRNOG00000019181		synaptopodin (Synpo), mRNA [Source	60324	18	55076843	55130938	54096	360,78	-0,66	6,24E-02
ENSRNOG00000019263	**Sp3**	Protein Sp3 [Source:UniProtKB/TrEM	367846	3	66119807	66153955	34149	457,99	-0,63	6,24E-02
ENSRNOG00000030371		cytochrome c oxidase subunit II (mitoc	26198	MT	7006	7689	684	97866,26	-0,57	6,32E-02
ENSRNOG00000047314	**Tk1**	thymidine kinase 1, soluble (Tk1), mR	24834	10	106458084	106468147	10064	605,28	0,74	6,32E-02
ENSRNOG00000008865	**Stac**	Protein Stac; Src homology three (SH	363152	8	119169640	119287534	117895	32,38	-0,95	6,37E-02
ENSRNOG00000003334	**Klhl21**	kelch-like family member 21 (Klhl21),	313743	5	172740028	172748683	8656	1399,83	0,57	6,39E-02
ENSRNOG00000015627	**Slc35a3**	solute carrier family 35 (UDP-N-acetylglucosami		2	237492884	237513169	20286	281,65	-0,63	6,39E-02
ENSRNOG00000029028	**Mknk2**	MAP kinase-interacting serine/threonir	299618	7	12075366	12086471	11106	4951,95	0,83	6,39E-02
ENSRNOG00000042702	**Nipa1**	non imprinted in Prader-Willi/Angelma	308668	1	115392570	115410234	17665	696,05	-0,75	6,39E-02
ENSRNOG00000027405	**Ccp110**	centriolar coiled coil protein 110kDa (C	361634	1	195365370	195387930	22561	761,04	0,46	6,41E-02
ENSRNOG00000043304	**Apcdd1**	Protein Apcdd1 [Source:UniProtKB/Tr	689616	18	57494474	57522393	27920	341,90	-0,84	6,41E-02
ENSRNOG00000049853	**Rgag1**	Protein LOC100912526 [Source:UniP	1E+08	X	112820558	112825273	4716	38,78	-1,01	6,41E-02
ENSRNOG00000010557	**Smarcd2**	SWI/SNF related, matrix associated, a	83833	10	94203168	94212034	8867	404,59	0,60	6,44E-02
ENSRNOG00000001428	**Alkbh4**	alkB, alkylation repair homolog 4 (E. c	288587	12	25568554	25574483	5930	327,84	0,73	6,45E-02
ENSRNOG00000023363	**Ing4**	inhibitor of growth family, member 4 (I	297597	4	224572632	224581190	8559	2708,79	0,80	6,45E-02
ENSRNOG00000001134	**Rfc5**	replication factor C (activator 1) 5 (Rfc	304528	12	46752012	46761552	9541	806,40	0,65	6,45E-02
ENSRNOG00000013123	**Scube2**	Protein Scube2-ps1 [Source:UniProtK	499241	1	181421062	181479784	58723	28,04	-1,09	6,45E-02
ENSRNOG00000019813	**Ppp4c**	protein phosphatase 4, catalytic subur	171366	1	205198901	205205448	6548	1644,10	0,51	6,48E-02
ENSRNOG00000014684	**Npr1**	natriuretic peptide receptor A/guanylat	24603	2	209271276	209286963	15688	82,24	-1,00	6,48E-02
ENSRNOG00000019924	**Thop1**	thimet oligopeptidase 1 (Thop1), mRN	64517	7	11668513	11680853	12341	2266,19	0,55	6,48E-02
ENSRNOG00000023272		Ribonucleoside-diphosphate reductas	362720	6	60756211	60761048	4838	165,76	0,77	6,48E-02
ENSRNOG00000024907	**Tnrc6b**	trinucleotide repeat containing 6B (Tnr	192178	7	121922421	122139717	217297	4055,26	-0,44	6,48E-02

ENSRNOG00000026610	**Akirin1**	akirin 1 (Akirin1), mRNA [Source:RefS	595134	5	145205746	145221146	15401	1116,79	-0,54	6,48E-02
ENSRNOG00000011024	**Zdhhc20**	zinc finger, DHHC-type containing 20 (	305923	15	41950094	42006537	56444	372,97	-0,77	6,49E-02
ENSRNOG00000008447	**Tmem132d**	transmembrane protein 132D (Tmem1	288750	12	33851101	34515311	664211	82,23	-0,92	6,51E-02
ENSRNOG00000004560	**LOC10091**	calcium channel, voltage-dependent, N	257648	3	1727956	1910340	182385	2719,11	-0,65	6,51E-02
ENSRNOG00000008564	**Tmem222**	transmembrane protein 222 (Tmem22	313021	5	155127137	155138924	11788	1521,49	0,50	6,51E-02
ENSRNOG00000009037	**Sulf1**	sulfatase 1 (Sulf1), mRNA [Source:Re	171396	5	10836868	10919822	82955	114,28	-1,19	6,51E-02
ENSRNOG00000025558	**Palm2**	Protein LOC100910655 [Source:UniP	1E+08	5	78911292	78931548	20257	151,38	-0,77	6,51E-02
ENSRNOG00000004517	**Igf1**	insulin-like growth factor 1 (Igf1), trans	24482	7	28528240	28602541	74302	48,76	-1,07	6,51E-02
ENSRNOG00000008716	**Nefh**	neurofilament, heavy polypeptide (Nef	24587	14	85859193	85869178	9986	213,20	0,88	6,51E-02
ENSRNOG00000006979	**Hpca**	hippocalcin (Hpca), mRNA [Source:Re	29177	5	151030063	151038262	8200	1479,94	-1,06	6,53E-02
ENSRNOG00000012095	**Pkia**	protein kinase (cAMP-dependent, cata	114906	2	116338940	116411705	72766	4146,70	-0,74	6,53E-02
ENSRNOG00000023316		Protein Grhl1 [Source:UniProtKB/TrEI	313993	6	60606789	60662227	55439	18,65	-1,07	6,54E-02
ENSRNOG00000007520	**Zcchc3**	Protein Zcchc3; RCG37285 [Source:U	690005	3	154226833	154228035	1203	324,70	0,64	6,55E-02
ENSRNOG00000016372	**Slc12a7**	solute carrier family 12 (potassium/chl	308069	1	33511432	33564814	53383	336,29	0,91	6,56E-02
ENSRNOG00000010981	**Gtf3c5**	general transcription factor IIIC, polype	362095	3	12495485	12515790	20306	1081,91	0,57	6,57E-02
ENSRNOG00000016795	**Rabl6**	RAB, member RAS oncogene family-li	362084	3	2788190	2814110	25921	3142,73	0,48	6,57E-02
ENSRNOG00000042062	**LOC68065**	RIKEN cDNA B930041F14 gene [Sou	680656	5	176614966	176615643	678	102,11	0,72	6,57E-02
ENSRNOG00000009896	**Tmem199**	transmembrane protein 199 (Tmem19	303332	10	65863303	65867897	4595	1034,67	0,88	6,59E-02
ENSRNOG00000024144	**Cdipt**	CDP-diacylglycerol--inositol 3-phospha	192260	1	205389729	205393866	4138	3257,18	0,62	6,59E-02
ENSRNOG00000046955	**Cbx6**	chromobox homolog 6 (Cbx6), mRNA	315136	7	121041261	121048988	7728	5475,74	0,73	6,59E-02
ENSRNOG00000003172	**Serpinf1**	serpin peptidase inhibitor, clade F (alp	287526	10	61954921	61969094	14174	109,07	-1,22	6,61E-02
ENSRNOG00000000487	**Grm4**	glutamate receptor, metabotropic 4 (G	24417	20	8986551	9013774	27224	173,55	-0,76	6,75E-02
ENSRNOG00000012703	**Crh**	corticotropin releasing hormone (Crh),	81648	2	124182919	124184783	1865	29,47	-1,21	6,75E-02
ENSRNOG00000016274	**Zfp580**	Protein Zfp580 [Source:UniProtKB/Tr	308336	1	76196682	76197200	519	654,85	0,70	6,75E-02
ENSRNOG00000020230	**Pias4**	protein inhibitor of activated STAT, 4 (F	362827	7	11581929	11595424	13496	984,02	0,66	6,75E-02
ENSRNOG00000029386	**RT1-N1**	RT1 class Ib, locus N2 (RT1-N2), mRN	24748	20	5245094	5248485	3392	30,27	1,00	6,75E-02
ENSRNOG00000018849	**Tcerg1**	transcription elongation regulator 1 (Tc	307474	18	36491823	36552727	60905	2799,99	-0,46	6,80E-02
ENSRNOG00000024310	**Kcnf1**	potassium voltage-gated channel, sub	298908	6	52191821	52193338	1518	395,98	-0,72	6,80E-02
ENSRNOG00000024689	**Hopx**	HOP homeobox (Hopx), mRNA [Sourc	171160	14	33145766	33153536	7771	238,77	-1,02	6,81E-02
ENSRNOG00000001203	**Rrp1**	ribosomal RNA processing 1 (Rrp1), m	309674	20	13153171	13164412	11242	1568,54	0,64	6,84E-02
ENSRNOG00000005727	**Galnt3**	UDP-N-acetyl-alpha-D-galactosamine	366061	3	58806687	58830432	23746	33,91	-0,92	6,87E-02
ENSRNOG00000048222		NLR family, CARD domain containing	291861	19	10897691	10959365	61675	76,03	1,06	6,89E-02
ENSRNOG00000005206		potassium voltage-gated channel, KQ	29682	7	106665371	106956100	290730	463,15	-0,99	6,94E-02
ENSRNOG00000015596	**Efhd1**	EF-hand domain family, member D1 (E	501181	9	94084446	94131498	47053	145,60	-1,12	6,94E-02
ENSRNOG00000032539	**Spag8**	sperm associated antigen 8 (Spag8), r	362508	5	63672205	63674417	2213	32,03	-0,93	6,96E-02
ENSRNOG00000005335	**Galnt13**	UDP-N-acetyl-alpha-D-galactosamine	311039	3	45130269	45710879	580611	244,08	-0,65	6,97E-02
ENSRNOG00000008025	**Bend5**	BEN domain containing 5 (Bend5), mR	362564	5	135084714	135138954	54241	394,44	-0,55	6,98E-02
ENSRNOG00000048309	**Uqcc**	ubiquinol-cytochrome c reductase com	683512	3	157749599	157840990	91392	1235,67	0,59	6,99E-02

ENSRNOG00000045561	**LOC69034**	expressed sequence AI462493 [Sourc	690344	1	232013921	232014699	779	327,38	0,58	7,02E-02
ENSRNOG00000004928	**Sntg2**	syntrophin, gamma 2 (Sntg2), mRNA [	298936	6	57803704	58004693	200990	33,92	-0,92	7,05E-02
ENSRNOG00000026573	**Ophn1**	oligophrenin 1 (Ophn1), mRNA [Sourc	312108	X	69063674	69437472	373799	563,72	-0,55	7,07E-02
ENSRNOG00000028930	**Dab2**	disabled 2, mitogen-responsive phosp	79128	2	75489435	75510919	21485	100,54	-0,96	7,09E-02
ENSRNOG00000007057		Uncharacterized protein [Source:UniProtKB/TrE		5	150394968	150411673	16706	112,00	-0,93	7,12E-02
ENSRNOG00000007988	**Timm22**	translocase of inner mitochondrial mer	79463	10	63451314	63458296	6983	1070,23	0,57	7,12E-02
ENSRNOG00000018297	**Ocln**	occludin (Ocln), mRNA [Source:RefSe	83497	2	49686710	49736838	50129	98,57	-1,00	7,15E-02
ENSRNOG00000016758	**Loxl2**	lysyl oxidase-like 2 (Loxl2), mRNA [So	290350	15	55059673	55092976	33304	33,84	-1,02	7,19E-02
ENSRNOG00000007765	**Frzb**	frizzled-related protein (Frzb), mRNA [	295691	3	74196166	74229331	33166	18,60	-1,21	7,24E-02
ENSRNOG00000050210		RT1 class I, locus CE10 (RT1-CE10),	414792	20	155870	158859	2990	187,60	1,11	7,24E-02
ENSRNOG00000000990	**Pdap1**	PDGFA associated protein 1 (Pdap1),	64527	12	13309878	13320105	10228	6213,95	0,65	7,31E-02
ENSRNOG00000002585	**Cul4b**	cullin 4B (Cul4b), mRNA [Source:RefS	302502	X	124917170	124954871	37702	1158,42	-0,79	7,31E-02
ENSRNOG00000003909	**Esyt1**	extended synaptotagmin-like protein 1	29579	7	2897632	2914841	17210	410,40	0,89	7,31E-02
ENSRNOG00000004110	**Trib2**	tribbles homolog 2 (Drosophila) (Trib2	313974	6	49774736	49798090	23355	2171,56	-0,45	7,31E-02
ENSRNOG00000004171	**Dnah9**	Protein Dnah9 [Source:UniProtKB/TrE	117251	10	52103139	52465432	362294	244,11	-0,79	7,31E-02
ENSRNOG00000005284	**Itpka**	inositol-trisphosphate 3-kinase A (Itpka	81677	3	118093293	118102049	8757	171,95	-1,11	7,31E-02
ENSRNOG00000007801	**R3hdm2**	R3H domain containing 2 (R3hdm2), r	362894	7	70870219	70978626	108408	2853,81	-0,65	7,31E-02
ENSRNOG00000008720	**Wbp1**	WW domain binding protein 1 (Wbp1),	297381	4	178636944	178639343	2400	1263,18	0,62	7,31E-02
ENSRNOG00000010589	**Fam134b**	family with sequence similarity 134, m	619558	2	97821407	97972800	151394	1176,68	-0,69	7,31E-02
ENSRNOG00000013603	**Dffa**	DNA fragmentation factor, alpha subur	114214	5	169573104	169586002	12899	1240,75	0,83	7,31E-02
ENSRNOG00000017136		Synaptotagmin-17 [Source:UniProtKE	192189	1	194982174	195048211	66038	292,79	-0,88	7,31E-02
ENSRNOG00000017708				4	182898783	182899852	1070	6713,87	0,49	7,31E-02
ENSRNOG00000020010	**Kctd13**	potassium channel tetramerization dor	293497	1	205341000	205359303	18304	2400,56	-0,57	7,31E-02
ENSRNOG00000022393	**RGD15647**	similar to RIKEN cDNA C230052I12 (F	502320	1	92784851	92791055	6205	95,37	-0,70	7,31E-02
ENSRNOG00000029811	**Kcne2**	potassium voltage-gated channel, Isk-	171138	11	36049570	36050026	457	21,23	-1,02	7,31E-02
ENSRNOG00000034084	**Ggct**	gamma-glutamyl cyclotransferase (Gg	362368	4	149842892	149850088	7197	193,86	-0,74	7,31E-02
ENSRNOG00000036806	**Psme3**	proteasome (prosome, macropain) act	25634	10	89034248	89109170	74923	2890,23	0,67	7,31E-02
ENSRNOG00000046207	**Cbx4**	Protein Cbx4 [Source:UniProtKB/TrEI	501403	10	107803835	107809057	5223	930,92	0,58	7,31E-02
ENSRNOG00000046293		nephronectin [Source:MGI Symbol;Acc:MGI:214		2	256542683	256607414	64732	144,13	-0,89	7,31E-02
ENSRNOG00000047706		Uncharacterized protein [Source:UniProtKB/TrE		20	5857674	5878729	21056	973,34	1,05	7,31E-02
ENSRNOG00000005457	**Lamp5**	lysosomal-associated membrane prote	362220	3	135507575	135519611	12037	22,60	-1,10	7,32E-02
ENSRNOG00000005861		hydroxysteroid 11-beta dehydrogenas	25116	13	116482032	116528373	46342	291,22	-0,98	7,32E-02
ENSRNOG00000013947	**Rabl2a**	RAB, member of RAS oncogene famil	362987	7	130241551	130250156	8606	500,37	-0,67	7,34E-02
ENSRNOG00000016326	**Cx3cl1**	chemokine (C-X3-C motif) ligand 1 (C:	89808	19	10638461	10647952	9492	2461,13	-0,79	7,34E-02
ENSRNOG00000027871		Protein Rapgefl1 [Source:UniProtKB/TrEMBL;A		10	86545853	86561330	15478	1297,87	-0,76	7,34E-02
ENSRNOG00000001829	**Ube2v2**	ubiquitin-conjugating enzyme E2 varia	287927	11	92611596	92645498	33903	664,00	-1,16	7,34E-02
ENSRNOG00000004378	**Abca5**	ATP-binding cassette, subfamily A (AE	286970	10	98282739	98351500	68762	414,64	-0,61	7,34E-02
ENSRNOG00000006327	**Rcc2**	Protein Rcc2 [Source:UniProtKB/TrEI	298594	5	162927687	162941028	13342	3560,64	0,74	7,34E-02

ENSRNOG00000009522	**Adra1a**	adrenoceptor alpha 1A (Adra1a), mRN	29412	15	48203098	48295045	91948	25,58	-1,09	7,34E-02
ENSRNOG00000012094	**Ltbp2**	latent transforming growth factor beta	59106	6	116956933	117049306	92374	121,04	-1,12	7,34E-02
ENSRNOG00000013545	**Polr2e**	polymerase (RNA) II (DNA directed) p	690966	7	12702978	12706900	3923	2107,54	0,65	7,34E-02
ENSRNOG00000015397	**Cpne7**	copine VII (Cpne7), mRNA [Source:Re	361433	19	66634979	66651622	16644	177,57	-1,01	7,34E-02
ENSRNOG00000020161	**Zbtb7a**	zinc finger and BTB domain containing	117107	7	11599413	11612150	12738	601,11	0,58	7,34E-02
ENSRNOG00000039902	**Lbh**	limb bud and heart development (Lbh)	683626	6	34010039	34031267	21229	1857,87	0,46	7,34E-02
ENSRNOG00000017330		predicted gene 9905 [Source:MGI Symbol;Acc:M		1	197883912	197891408	7497	345,59	0,61	7,36E-02
ENSRNOG00000018094	**Sv2c**	synaptic vesicle glycoprotein 2c (Sv2c	29643	2	45631737	45828663	196927	379,22	-1,11	7,36E-02
ENSRNOG00000018109	**Clic4**	chloride intracellular channel 4 (Clic4),	83718	5	157335185	157394574	59390	3638,52	-0,94	7,36E-02
ENSRNOG00000013980	**Tfpt**	TCF3 (E2A) fusion partner (Tfpt), mRN	85423	1	63155152	63164554	9403	560,93	0,59	7,39E-02
ENSRNOG00000010598	**Hs3st1**	heparan sulfate (glucosamine) 3-O-su	84406	14	75872397	75873335	939	530,69	0,60	7,43E-02
ENSRNOG00000048411	**Uhrf1**	ubiquitin-like with PHD and ring finger	316129	9	9731078	9749712	18635	574,60	0,63	7,43E-02
ENSRNOG00000008086	**Dpf3**	D4, zinc and double PHD fingers, fami	299186	6	114855815	114948893	93079	55,01	-0,86	7,47E-02
ENSRNOG00000008001	**Rab3b**	RAB3B, member RAS oncogene famil	81755	5	132343181	132408834	65654	548,22	-0,96	7,50E-02
ENSRNOG00000003707	**Zmym3**	zinc finger, MYM-type 3 (Zmym3), mR	317260	X	72150498	72165290	14793	3414,14	-0,51	7,51E-02
ENSRNOG00000004640	**Mtfp1**	mitochondrial fission process 1 (Mtfp1	289745	14	85011888	85015728	3841	409,24	0,67	7,51E-02
ENSRNOG00000012437		Protein Fam73a [Source:UniProtKB/T	362058	2	276170622	276223258	52637	727,93	-0,58	7,51E-02
ENSRNOG00000012439	**Bid**	BH3 interacting domain death agonist	64625	4	220531877	220554316	22440	735,23	-0,50	7,51E-02
ENSRNOG00000014675	**Pi4k2a**	phosphatidylinositol 4-kinase type 2 al	114554	1	268790066	268814981	24916	754,81	0,51	7,53E-02
ENSRNOG00000019915	**Xrcc1**	X-ray repair complementing defective	84495	1	82673671	82701080	27410	1659,72	0,52	7,56E-02
ENSRNOG00000023969		hect domain and RLD 6 [Source:MGI Symbol;Ac		4	153429563	153471906	42344	22,39	1,14	7,56E-02
ENSRNOG00000004292	**Sptssa**	serine palmitoyltransferase, small sub	500651	6	85080146	85091608	11463	1047,52	-0,50	7,57E-02
ENSRNOG00000049911		Protein LOC100912203 [Source:UniP	29224	11	37455173	37457389	2217	119,50	0,62	7,57E-02
ENSRNOG00000020892	**Dpf2**	D4, zinc and double PHD fingers famil	361711	1	228186741	228201255	14515	2392,61	0,44	7,58E-02
ENSRNOG00000010597	**Slc5a7**	solute carrier family 5 (sodium/choline	85426	9	4343096	4379724	36629	29,59	-1,11	7,62E-02
ENSRNOG00000024711	**Sdk2**	sidekick cell adhesion molecule 2 (Sdk	360652	10	101978490	102096741	118252	545,91	-0,72	7,62E-02
ENSRNOG00000000780	**Ppp1r11**	protein phosphatase 1, regulatory (inh	294207	20	4131645	4135152	3508	1869,50	0,48	7,63E-02
ENSRNOG00000003084	**Parp1**	poly (ADP-ribose) polymerase 1 (Parp	25591	13	103859760	103891948	32189	3592,78	0,44	7,63E-02
ENSRNOG00000015325		phosphatidylinositol glycan anchor biosynthesis,		6	20554557	20580952	26396	152,21	-0,75	7,65E-02
ENSRNOG00000018622		Protein Trim27; Tripartite motif protein 27 (Predic		17	57844089	57856502	12414	1879,77	0,51	7,65E-02
ENSRNOG00000046791	**Sh3rf3**	Protein Sh3rf3 [Source:UniProtKB/TrE	294557	20	30171737	30524777	353041	1022,12	-0,57	7,65E-02
ENSRNOG00000004726	**Mapkapk2**	mitogen-activated protein kinase-activ	289014	13	52865897	52870983	5087	1691,54	0,55	7,70E-02
ENSRNOG00000023208	**RGD13098**	uncharacterized protein LOC366360 |	366360	5	62402428	62409756	7329	762,90	-0,97	7,76E-02
ENSRNOG00000024567	**LOC68367**	RIKEN cDNA E130309D02 gene [Sou	683674	12	15340705	15353986	13282	954,16	0,70	7,82E-02
ENSRNOG00000025481	**Dctn2**	dynactin 2 (p50) (Dctn2), mRNA [Sour	299850	7	70730174	70745437	15264	8237,82	0,49	7,82E-02
ENSRNOG00000008194	**Znfx1**	zinc finger, NFX1-type containing 1 (Z	296384	3	169948332	170007642	59311	1003,74	0,81	7,84E-02
ENSRNOG00000012885	**Ccdc28a**	coiled-coil domain containing 28A (Cc	361454	1	15072111	15083325	11215	104,78	0,69	7,84E-02
ENSRNOG00000016054	**Tab2**	TGF-beta activated kinase 1/MAP3K7	308267	1	3696211	3770496	74286	2198,50	-0,61	7,84E-02

ENSRNOG00000018268	**Hhip**	Hedgehog-interacting protein (Hhip), r	291936	19	42430120	42518700	88581	135,42	-1,01	7,84E-02
ENSRNOG00000025160	**Tmem56**	transmembrane protein 56 (Tmem56),	365924	2	242813588	242867287	53700	764,49	-0,62	7,84E-02
ENSRNOG00000007818		Protein Slc45a4; Similar to KIAA1126	315054	7	114746106	114771962	25857	598,89	0,65	7,89E-02
ENSRNOG00000009145	**Klf12**	Kruppel-like factor 12 (Klf12), mRNA [S	306110	15	88095615	88331655	236041	319,85	-0,67	7,89E-02
ENSRNOG00000009422	**Hmgcl**	3-hydroxymethyl-3-methylglutaryl-CoA	79238	5	158059688	158073434	13747	799,25	0,54	7,89E-02
ENSRNOG00000012988	**Lix1**	Lix1 homolog (chicken) (Lix1), mRNA	292381	1	60083352	60139891	56540	882,96	-0,59	7,89E-02
ENSRNOG00000017075	**Slc35e2b**	solute carrier family 35, member E2B	313765	5	176499465	176521324	21860	591,90	-0,49	7,89E-02
ENSRNOG00000017923	**Pnck**	pregnancy upregulated non-ubiquitous	29660	1	152900172	152904072	3901	396,82	-0,85	7,89E-02
ENSRNOG00000019930	**Rhot2**	ras homolog family member T2 (Rhot2	287156	10	15018888	15024226	5339	1734,77	0,52	7,89E-02
ENSRNOG00000021573	**Dpy19l3**	dpy-19-like 3 (C. elegans) (Dpy19l3), r	308519	1	93139685	93199944	60260	1006,59	-0,62	7,89E-02
ENSRNOG00000034026	**Lclat1**	Protein Lclat1; Similar to lysocardiolipi	362702	6	34278820	34364478	85659	233,19	0,54	7,89E-02
ENSRNOG00000046253		Uncharacterized protein [Source:UniProtKB/TrE		5	176528187	176542116	13930	820,56	0,46	7,89E-02
ENSRNOG00000048176				16	7739075	7739201	127	33,67	-0,92	7,89E-02
ENSRNOG00000006985	**Ccnk**	cyclin K (Ccnk), mRNA [Source:RefSe	500715	6	141261013	141283937	22925	1084,13	0,42	7,93E-02
ENSRNOG00000008377	**Wdtc1**	WD and tetratricopeptide repeats 1 (W	313020	5	155153929	155201755	47827	2462,21	0,44	7,93E-02
ENSRNOG00000014084	**Sp1**	Sp1 transcription factor (Sp1), mRNA	24790	7	141806042	141836498	30457	1436,79	-0,45	7,93E-02
ENSRNOG00000027839	**Ptk2b**	protein tyrosine kinase 2 beta (Ptk2b),	50646	15	48646435	48766707	120273	1276,52	-0,91	7,93E-02
ENSRNOG00000033361	**Slc39a5**	solute carrier family 39 (zinc transporte	362812	7	2792339	2797763	5425	433,56	0,68	7,93E-02
ENSRNOG00000004268	**Zfp386**	zinc finger protein 386 (Kruppel-like) (Z	25165	6	152840231	152854884	14654	211,28	-0,87	7,94E-02
ENSRNOG00000010248	**Serpini1**	serpin peptidase inhibitor, clade I (neu	116459	2	193348764	193446630	97867	1146,12	-0,71	7,94E-02
ENSRNOG00000013196	**Dok5**	docking protein 5 (Dok5), mRNA [Sour	502694	3	174447259	174579714	132456	1183,97	-0,55	7,94E-02
ENSRNOG00000017215	**LOC10090**	uncharacterized protein LOC307249	307249	18	73693017	73737785	44769	477,88	-0,64	7,94E-02
ENSRNOG00000018289	**RGD15606**	parkinson protein 7 (Park7), transcript	117287	5	171566144	171582474	16331	4229,41	0,53	7,94E-02
ENSRNOG00000025669	**Tmem104**	transmembrane protein 104 (Tmem10	303670	10	104503014	104566633	63620	233,05	0,58	7,94E-02
ENSRNOG00000012026	**Psmc2**	proteasome (prosome, macropain) 26	25581	4	9868268	9882578	14311	4161,79	0,45	7,97E-02
ENSRNOG00000017659	**Hs3st2**	heparan sulfate (glucosamine) 3-O-su	293451	1	198266876	198393452	126577	498,18	-0,76	7,97E-02
ENSRNOG00000020261	**Fam53c**	Protein Fam53c [Source:UniProtKB/T	685405	18	27275541	27283115	7575	1259,39	0,62	7,97E-02
ENSRNOG00000001324	**Asmt**	acetylserotonin O-methyltransferase (A	246281	12	20506355	20511204	4850	54,99	0,90	8,05E-02
ENSRNOG00000003069	**Cd38**	CD38 molecule (Cd38), mRNA [Sourc	25668	14	71745755	71785718	39964	110,56	-0,99	8,07E-02
ENSRNOG00000005154	**Fam150b**	family with sequence similarity 150, m	679566	6	58505391	58514834	9444	19,55	-1,16	8,08E-02
ENSRNOG00000000641	**Nrbf2**	nuclear receptor binding factor 2 (Nrbf	58839	20	24818944	24837210	18267	71,74	0,70	8,10E-02
ENSRNOG00000012770	**Spata9**	spermatogenesis associated 9 (Spata	294594	2	2728276	2765300	37025	69,82	-0,82	8,16E-02
ENSRNOG00000018434	**Stab1**	Protein Stab1 [Source:UniProtKB/TrE	1E+08	16	7149123	7179494	30372	47,00	-1,15	8,17E-02
ENSRNOG00000026622	**RGD13107**	LOC363070 (RGD1310727), mRNA [S	363070	8	62094551	62097943	3393	483,98	0,87	8,17E-02
ENSRNOG00000039470				18	30273856	30275746	1891	201,82	-0,76	8,19E-02
ENSRNOG00000006868	**Ube2z**	ubiquitin-conjugating enzyme E2Z (Ub	303478	10	83674573	83693693	19121	5052,79	0,56	8,19E-02
ENSRNOG00000009419		receptor-type tyrosine-protein phospha	171357	15	16953386	17647514	694129	1712,42	-0,69	8,21E-02
ENSRNOG00000012946	**Mov10**	Moloney leukemia virus 10 (Mov10), m	310756	2	226697325	226717439	20115	230,88	0,84	8,37E-02

ENSRNOG00000048511	**Garem**	Protein LOC679154 [Source:UniProtK	679154	18	14769101	14791340	22240	207,57	-0,61	8,37E-02
ENSRNOG00000049530		RCG62717; Uncharacterized protein [Source:U		2	203633992	203634802	811	285,07	-0,58	8,37E-02
ENSRNOG00000000464	**Rxrb**	retinoid X receptor beta (Rxrb), mRNA	361801	20	5902519	5908469	5951	2193,91	0,54	8,38E-02
ENSRNOG00000005097		Cyclin-dependent kinase-like 3 [Sourc	60396	10	37231600	37315393	83794	351,26	-0,55	8,38E-02
ENSRNOG00000014454	**Ap1m1**	adaptor-related protein complex 1, mu	306332	16	19148666	19164135	15470	3391,55	0,52	8,38E-02
ENSRNOG00000010218	**Eif5**	eukaryotic translation initiation factor 5	56783	6	144405139	144412325	7187	1487,33	-0,56	8,42E-02
ENSRNOG00000017753	**Ercc2**	excision repair cross-complementing r	308415	1	81559883	81573643	13761	999,15	0,47	8,42E-02
ENSRNOG00000018854	**Nppc**	natriuretic peptide C (Nppc), mRNA [S	114593	9	93458753	93462953	4201	145,44	-0,88	8,44E-02
ENSRNOG00000015450	**Ppp1r16a**	protein phosphatase 1, regulatory sub	362944	7	117722085	117745228	23144	1338,05	0,49	8,45E-02
ENSRNOG00000048151		dyskeratosis congenita 1, dyskerin [Source:MGI		1	151564298	151578545	14248	2962,64	0,45	8,45E-02
ENSRNOG00000039086	**Ccdc153**	coiled-coil domain containing 153 (Ccc	300663	8	47189965	47196423	6459	102,77	-1,14	8,45E-02
ENSRNOG00000039969	**Dsc2**	desmocollin 2 (Dsc2), mRNA [Source:	291760	18	11625848	11700836	74989	20,05	-1,00	8,45E-02
ENSRNOG00000012240	**Supt6h**	suppressor of Ty 6 homolog (S. cerevi	303281	10	66129656	66166766	37111	6292,82	0,57	8,52E-02
ENSRNOG00000005825	**Lyz2**	lysozyme 2 (Lyz2), mRNA [Source:Re	25211	7	60337951	60343246	5296	33,52	-1,14	8,53E-02
ENSRNOG00000012999	**Phb2**	prohibitin 2 (Phb2), mRNA [Source:Re	114766	4	224248364	224252647	4284	4360,61	0,56	8,53E-02
ENSRNOG00000016727	**Akr1a1**	aldo-keto reductase family 1, member	78959	5	139278242	139295142	16901	26909,02	0,57	8,53E-02
ENSRNOG00000042556	**Bmyc**	brain expressed myelocytomatosis on	311807	3	2898075	2898834	760	1154,75	0,52	8,54E-02
ENSRNOG00000003332	**Nt5m**	5',3'-nucleotidase, mitochondrial (Nt5m	287368	10	45972898	45999748	26851	288,98	0,55	8,64E-02
ENSRNOG00000015157	**Smtnl2**	smoothelin-like 2 (Smtnl2), mRNA [So	679629	10	58690457	58711606	21150	129,13	-1,00	8,66E-02
ENSRNOG00000004212	**Fbxw9**	F-box and WD repeat domain containi	288921	19	37149394	37156298	6905	1430,64	0,49	8,66E-02
ENSRNOG00000007437	**Irf5**	interferon regulatory factor 5 (Irf5), mF	296953	4	56572293	56583432	11140	20,18	-1,03	8,69E-02
ENSRNOG00000005083	**Rtfdc1**	replication termination factor 2 domain	296410	3	176519321	176548882	29562	2928,35	0,65	8,72E-02
ENSRNOG00000009882		Serine/threonine-protein phosphatase	24674	2	260462929	260735288	272360	3503,68	-0,62	8,72E-02
ENSRNOG00000015231	**Mrpl44**	mitochondrial ribosomal protein L44 (M	301552	9	85293975	85299104	5130	1017,21	0,51	8,72E-02
ENSRNOG00000019559	**Eaf1**	ELL associated factor 1 (Eaf1), mRNA	306261	16	7523081	7537830	14750	1152,81	0,84	8,72E-02
ENSRNOG00000020144	**RGD13101**	similar to cDNA sequence BC017158	361654	1	206733725	206763393	29669	869,38	0,61	8,72E-02
ENSRNOG00000033667	**Lmbr1l**	limb region 1 like (Lmbr1l), mRNA [So	300215	X	115098755	115107826	9072	427,90	0,96	8,72E-02
ENSRNOG00000003186	**Anks3**	ankyrin repeat and sterile alpha motif	302937	10	9575751	9596420	20670	950,02	0,69	8,73E-02
ENSRNOG00000029038	**Gemin8**	gem (nuclear organelle) associated pr	363462	X	30198510	30217341	18832	492,97	0,45	8,75E-02
ENSRNOG00000015090	**Nubp2**	nucleotide binding protein 2 (Nubp2),	287125	10	14060220	14063916	3697	1088,35	0,57	8,75E-02
ENSRNOG00000006500	**Snf8**	SNF8, ESCRT-II complex subunit (Snf	287645	10	83661391	83673470	12080	1303,48	0,45	8,80E-02
ENSRNOG00000020918	**Ccnd1**	cyclin D1 (Ccnd1), mRNA [Source:Ref	58919	1	224960136	224969658	9523	3573,10	0,63	8,80E-02
ENSRNOG00000013399	**LOC50103**	cDNA sequence AF529169 [Source:M	501033	8	96475723	96488337	12615	94,59	-0,90	8,85E-02
ENSRNOG00000018797	**Myrip**	myosin VIIA and Rab interacting prote	360034	8	128203900	128335868	131969	413,21	-0,84	8,85E-02
ENSRNOG00000042977	**Mtx1**	Metaxin 1 (Mtx1), mRNA [Source:RefS	295241	2	207937066	207942555	5490	1412,45	0,41	8,87E-02
ENSRNOG00000024177		transducer of ERBB2, 2 [Source:MGI Symbol;Ac		7	123063582	123064214	633	104,44	0,82	8,87E-02
ENSRNOG00000024743	**L3mbtl2**	l(3)mbt-like 2 (Drosophila) (L3mbtl2),	300320	7	122871438	122892514	21077	1511,42	0,61	8,87E-02
ENSRNOG00000027468	**Slc6a15**	solute carrier family 6 (neutral amino a	282712	7	45349000	45402668	53669	1050,01	-0,82	8,92E-02

ENSRNOG00000010487	**Dnajb14**	DnaJ (Hsp40) homolog, subfamily B, r	499716	2	261656714	261699682	42969	333,65	-0,51	8,92E-02
ENSRNOG00000037580	**Psmd8**	proteasome (prosome, macropain) 26	292766	1	89292313	89299144	6832	5444,20	0,57	8,94E-02
ENSRNOG00000007031	**Trim9**	tripartite motif-containing 9 (Trim9), ml	155812	6	102107774	102207427	99654	3323,59	-0,69	8,94E-02
ENSRNOG00000009040	**Gas2l1**	growth arrest-specific 2 like 1 (Gas2l1	360973	14	85986064	85995815	9752	751,93	0,57	8,98E-02
ENSRNOG00000021081	**Vps72**	vacuolar protein sorting 72 homolog (S	310661	2	215772302	215783863	11562	2557,21	0,51	8,98E-02
ENSRNOG00000006628	**Dusp16**	dual specificity phosphatase 16 (Dusp	297682	4	232753988	232798358	44371	153,83	-0,70	8,99E-02
ENSRNOG00000018860	**Mtg1**	Mitochondrial GTPase 1 [Source:UniF	1E+08	1	219521209	219533905	12697	634,71	0,53	9,11E-02
ENSRNOG00000004744	**Fam84b**	Protein Fam84b [Source:UniProtKB/T	315019	7	101707859	101708791	933	98,72	-0,82	9,11E-02
ENSRNOG00000013412		cAMP responsive element binding pro	81646	9	71278917	71346034	67118	243,77	-0,58	9,11E-02
ENSRNOG00000019154	**Zmynd8**	zinc finger, MYND-type containing 8 (Z	296374	3	168659098	168756457	97360	2279,51	0,43	9,11E-02
ENSRNOG00000001003	**Trappc5**	trafficking protein particle complex 5 (T	363858	12	4390645	4394259	3615	2085,26	0,63	9,18E-02
ENSRNOG00000010325	**Ptger3**	prostaglandin E receptor 3 (subtype E	24929	2	282680844	282760556	79713	80,50	-1,09	9,18E-02
ENSRNOG00000020619	**Eif3g**	eukaryotic translation initiation factor 3	298700	8	21967952	21971979	4028	2423,30	0,64	9,18E-02
ENSRNOG00000021579	**Mpp6**	membrane protein, palmitoylated 6 (M	362359	4	144519340	144598644	79305	1051,24	-0,52	9,18E-02
ENSRNOG00000008279	**Mrps15**	mitochondrial ribosomal protein S15 (M	298517	5	147824053	147834646	10594	860,18	0,63	9,18E-02
ENSRNOG00000009288	**Fgf14**	fibroblast growth factor 14 (Fgf14), mF	63851	15	113762510	114450472	687963	327,86	-0,80	9,18E-02
ENSRNOG00000012367		protocadherin-7 precursor [Source:Re	360942	14	54787857	55216817	428961	760,98	-0,73	9,21E-02
ENSRNOG00000011523	**H2afy**	H2A histone family, member Y (H2afy)	29384	17	11440883	11502763	61881	4198,91	0,42	9,27E-02
ENSRNOG00000001607	**Adamts1**	ADAM metallopeptidase with thrombo	79252	11	28965542	28974878	9337	71,85	-1,09	9,28E-02
ENSRNOG00000011158	**Ppp2r2a**	protein phosphatase 2, regulatory sub	117104	15	47862028	47921334	59307	3005,04	-0,48	9,28E-02
ENSRNOG00000043364	**Zfp362**	zinc finger protein 362 (Zfp362), mRN	297879	5	150689530	150711442	21913	966,59	0,51	9,28E-02
ENSRNOG00000000368	**Grik2**	glutamate receptor, ionotropic, kainate	54257	20	55397877	55872312	474436	681,71	-0,81	9,31E-02
ENSRNOG00000024039	**Serinc5**	serine incorporator 5 (Serinc5), mRNA	170907	2	41398821	41516426	117606	2860,02	-0,70	9,31E-02
ENSRNOG00000003999	**Adcy3**	adenylate cyclase 3 (Adcy3), mRNA [S	64508	6	38378060	38454925	76866	796,24	-0,50	9,35E-02
ENSRNOG00000010539	**Mcat**	malonyl CoA:ACP acyltransferase (mit	315173	7	124401456	124412526	11071	1006,90	0,58	9,35E-02
ENSRNOG00000004333		Protein Flywch1 [Source:UniProtKB/T	360488	10	12910768	12927188	16421	1841,78	0,55	9,40E-02
ENSRNOG00000017556	**Chrm4**	cholinergic receptor, muscarinic 4 (Ch	25111	3	87531940	87539674	7735	290,54	-0,81	9,41E-02
ENSRNOG00000014604	**Sigmar1**	sigma non-opioid intracellular receptor	29336	5	62645891	62648669	2779	1903,93	0,47	9,45E-02
ENSRNOG00000022860	**Swi5**	SWI5 recombination repair homolog (y	499779	3	16656851	16665969	9119	5161,86	0,43	9,45E-02
ENSRNOG00000021101	**Sema6c**	sema domain, transmembrane domain	29744	2	215831094	215840455	9362	1944,73	0,73	9,49E-02
ENSRNOG00000016367	**Vasp**	vasodilator-stimulated phosphoprotein	361517	1	81437102	81452365	15264	1068,46	0,52	9,52E-02
ENSRNOG00000003209	**Pcp4l1**	Purkinje cell protein 4-like 1 (Pcp4l1),	685448	13	94169878	94192679	22802	137,09	-0,81	9,55E-02
ENSRNOG00000029415	**RGD15657**	RGD1565784 (RGD1565784), mRNA	497874	10	14358284	14359586	1303	886,21	0,50	9,55E-02
ENSRNOG00000007018	**Mapkbp1**	mitogen activated protein kinase bindi	362197	3	118316138	118364509	48372	1421,70	-0,45	9,59E-02
ENSRNOG00000014915	**Gsr**	glutathione reductase (Gsr), mRNA [S	116686	16	61859601	61901971	42371	1495,10	0,58	9,60E-02
ENSRNOG00000015967	**Sh3bgrl3**	SH3 domain binding glutamic acid-rich	298544	5	156043499	156044872	1374	1493,91	0,53	9,61E-02
ENSRNOG00000019247		pleckstrin homology domain-containin	314634	7	11951787	11954190	2404	1418,15	0,69	9,67E-02
ENSRNOG00000043143		echinoderm microtubule associated protein like		6	141509418	141619631	110214	656,01	-0,67	9,67E-02

ENSRNOG00000008400		promyelocytic leukemia [Source:MGI Symbol;Ac		8	62781117	62810211	29095	314,04	0,91	9,71E-02
ENSRNOG00000017513	**Fam73b**	family with sequence similarity 73, me	296623	3	14281314	14303962	22649	1576,64	0,46	9,73E-02
ENSRNOG00000018582	**Exosc6**	Protein Exosc6 [Source:UniProtKB/Tr	307850	19	54016153	54016971	819	321,78	0,85	9,73E-02
ENSRNOG00000015474	**Nelfa**	negative elongation factor complex me	305455	14	82778077	82802146	24070	808,97	0,45	9,73E-02
ENSRNOG00000017158	**Eif4g2**	eukaryotic translation initiation factor 4	361628	1	182868165	182880312	12148	4278,54	-0,56	9,73E-02
ENSRNOG00000018227	**Hiatl1**	hippocampus abundant transcript-like	306687	17	4842401	4894767	52367	909,28	-0,58	9,73E-02
ENSRNOG00000018801		kelch domain containing 4 [Source:MGI Symbol;		19	65335010	65362339	27330	1340,25	0,66	9,73E-02
ENSRNOG00000026060	**Arsi**	arylsulfatase family, member I (Arsi), r	307404	18	55414598	55420577	5980	44,87	-1,00	9,73E-02
ENSRNOG00000026866	**LOC10091(**	synapsin III (Syn3), mRNA [Source:Re	29130	7	23553826	23958150	404325	669,78	-0,45	9,73E-02
ENSRNOG00000036742	**Uqcrc2**	ubiquinol cytochrome c reductase core	293448	1	197480625	197511182	30558	2659,69	-0,60	9,73E-02
ENSRNOG00000010938	**Slc7a10**	solute carrier family 7 (neutral amino a	114518	1	92563400	92579262	15863	262,04	-0,80	9,78E-02
ENSRNOG00000012952	**Lrig1**	Protein Lrig1 [Source:UniProtKB/TrEM	312574	4	191131078	191158729	27652	1007,19	0,47	9,78E-02
ENSRNOG00000011271	**Mcc**	mutated in colorectal cancers (Mcc), n	307449	18	35348808	35448391	99584	61,38	-0,78	9,80E-02
ENSRNOG00000019506	**Dnajb2**	DnaJ (Hsp40) homolog, subfamily B, r	689593	9	82205777	82213929	8153	2673,93	0,42	9,80E-02
ENSRNOG00000015393	**Slc32a1**	solute carrier family 32 (GABA vesicul	83612	3	160500060	160504727	4668	2749,00	0,75	9,85E-02
ENSRNOG00000003897	**Col1a1**	collagen, type I, alpha 1 (Col1a1), mR	29393	10	82563434	82580363	16930	505,24	-1,04	9,86E-02
ENSRNOG00000007442	**Ubl7**	ubiquitin-like 7 (bone marrow stromal	300744	8	62472012	62489306	17295	1670,44	0,49	9,87E-02
ENSRNOG00000024839	**Tysnd1**	trypsin domain containing 1 (Tysnd1),	365571	20	33110493	33116211	5719	849,90	0,59	9,87E-02
ENSRNOG00000019740	**Hdgfrp3**	hepatoma-derived growth factor, relate	252941	1	144646635	144694902	48268	2420,03	-0,52	9,87E-02
ENSRNOG00000021132	**Trmt112**	tRNA methyltransferase 11-2 homolog	293700	1	229158449	229159630	1182	1508,45	0,66	9,87E-02
ENSRNOG00000028302	**Smarcb1**	SWI/SNF related, matrix associated, a	361825	20	15836278	15858383	22106	2830,83	0,38	9,87E-02
ENSRNOG00000005487	**Chpt1**	choline phosphotransferase 1 (Chpt1)	362866	7	29150843	29177859	27017	762,06	-0,81	9,90E-02
ENSRNOG00000029042		NADH dehydrogenase subunit 6 (mito	26203	MT	13543	14061	519	13090,15	-0,62	9,95E-02
ENSRNOG00000037627	**Trappc1**	trafficking protein particle complex 1 (T	287427	10	55667071	55668657	1587	1850,63	0,49	9,95E-02
ENSRNOG00000013818		ras-specific guanine nucleotide-releas	114513	2	40664646	40835991	171346	356,40	-0,81	9,96E-02
ENSRNOG00000024722		Uncharacterized protein [Source:UniProtKB/TrE		6	122076962	122260333	183372	237,64	-0,72	9,98E-02
ENSRNOG00000001492	**Slc8a2**	solute carrier family 8 (sodium/calcium	140447	1	79296259	79320748	24490	1227,64	-0,72	9,98E-02
ENSRNOG00000001796	**Dgkg**	diacylglycerol kinase, gamma (Dgkg),	25666	11	85059234	85251061	191828	184,13	-0,94	9,98E-02
ENSRNOG00000007040	**Timm17a**	translocase of inner mitochondrial mer	54311	13	57174166	57185658	11493	1015,77	0,54	9,98E-02
ENSRNOG00000008598	**Btbd7**	BTB (POZ) domain containing 7 (Btbd	362772	6	135867662	135916888	49227	783,50	-0,49	9,98E-02
ENSRNOG00000015822	**Klf13**	Kruppel-like factor 13 (Klf13), mRNA [	499171	1	125883341	125914101	30761	201,79	0,59	1,00E-01
ENSRNOG00000020595	**Rps11**	ribosomal protein S11 (Rps11), mRNA	81774	1	102182159	102184324	2166	7487,11	0,52	1,00E-01
ENSRNOG00000021179	**Naa40**	N(alpha)-acetyltransferase 40, NatD c	361718	1	229468108	229485469	17362	993,93	0,56	1,00E-01
ENSRNOG00000028649	**Tox3**	TOX high mobility group box family me	291908	19	29134067	29241334	107268	888,86	-0,58	1,00E-01
ENSRNOG00000011029	**Sec11a**	SEC11 homolog A (S. cerevisiae) (Sec	65166	1	143685596	143711945	26350	2004,03	0,42	1,00E-01
ENSRNOG00000024135	**Zmat1**	Protein Zmat1; RCG38044, isoform Cl	308654	X	105695032	105724457	29426	120,36	-0,68	1,00E-01
ENSRNOG00000033496		Protein Igdcc4 [Source:UniProtKB/TrE	363081	8	70300509	70322185	21677	2401,09	-0,92	1,00E-01
ENSRNOG00000007859	**LOC10036**	coiled-coil domain containing 41 (Ccdc	366872	7	35838379	35911635	73257	174,31	-0,79	1,01E-01

ENSRNOG00000026490		dynamin 3 (Dnm3), mRNA [Source:Re	171574	13	84804759	85272009	467251	1590,58	-0,51	1,01E-01
ENSRNOG00000001254	**Col6a2**	collagen, type VI, alpha 2 (Col6a2), m	361821	20	14931526	14959214	27689	203,51	-1,08	1,01E-01
ENSRNOG00000018491	**Chchd5**	coiled-coil-helix-coiled-coil-helix doma	296147	3	128353804	128354758	955	351,13	0,54	1,01E-01
ENSRNOG00000024905	**Drc1**	dynein regulatory complex subunit 1 h	362712	6	37237529	37272329	34801	122,13	-0,91	1,02E-01
ENSRNOG00000002125	**Ccni**	cyclin I (Ccni), mRNA [Source:RefSeq	289500	14	16267528	16290318	22791	8341,53	-0,44	1,02E-01
ENSRNOG00000025079	**Fam126b**	family with sequence similarity 126, m	316415	9	65213175	65249294	36120	142,66	-0,73	1,02E-01
ENSRNOG00000028974	**LOC100912099**			1	172475052	172475901	850	564,28	0,53	1,02E-01
ENSRNOG00000005809	**Arhgdib**	Rho, GDP dissociation inhibitor (GDI)	362456	4	235170740	235189433	18694	188,66	0,84	1,02E-01
ENSRNOG00000000476	**Zbtb22**	zinc finger and BTB domain containing	309630	20	7534957	7538123	3167	1305,53	0,45	1,02E-01
ENSRNOG00000024961	**Usp35**	Ubiquitin carboxyl-terminal hydrolase	308834	1	168491301	168507039	15739	334,19	0,57	1,02E-01
ENSRNOG00000013179	**Tinagl1**	tubulointerstitial nephritis antigen-like	94174	5	152101199	152110578	9380	31,61	-0,98	1,02E-01
ENSRNOG00000017204		PQ-loop repeat-containing protein 1 [Source:Un		18	72002641	72038286	35646	1254,83	0,56	1,02E-01
ENSRNOG00000027799	**Tmie**	transmembrane inner ear (Tmie), mRN	501061	8	118480842	118495446	14605	78,31	-1,08	1,02E-01
ENSRNOG00000036687	**Alyref**	Aly/REF export factor (Alyref), mRNA	690585	10	109363929	109367692	3764	1838,71	0,49	1,02E-01
ENSRNOG00000003491		protein kinase C alpha type [Source:F	24680	10	95919265	96311060	391796	1091,65	-0,66	1,03E-01
ENSRNOG00000009990	**Zranb2**	zinc finger, RAN-binding domain containing 2 (Z		2	282777936	282791456	13521	2131,73	-0,68	1,03E-01
ENSRNOG00000006198	**Prr11**	proline rich 11 (Prr11), mRNA [Source	360591	10	75687241	75706849	19609	121,23	0,71	1,03E-01
ENSRNOG00000009596		zinc finger, SWIM-type containing 8 (Z	361004	15	3737671	3749885	12215	3641,53	0,42	1,03E-01
ENSRNOG00000019138	**Clec11a**	C-type lectin domain family 11, membe	29313	1	101355487	101358626	3140	131,72	-0,61	1,03E-01
ENSRNOG00000019666	**Ubxn1**	UBX domain protein 1 (Ubxn1), mRNA	293719	1	232005791	232009563	3773	4292,96	0,46	1,03E-01
ENSRNOG00000023317	**Colgalt1**	collagen beta(1-O)galactosyltransfera	290637	16	19900825	19913462	12638	1585,16	0,71	1,03E-01
ENSRNOG00000028530		predicted gene 22 [Source:MGI Symbol;Acc:MG		19	65787859	65798862	11004	65,61	-1,01	1,03E-01
ENSRNOG00000008348	**Cdc34**	cell division cycle 34 (Cdc34), mRNA [	299602	7	13068994	13074382	5389	1537,57	0,80	1,03E-01
ENSRNOG00000016845	**Gabra3**	gamma-aminobutyric acid (GABA) A re	24947	1	148143256	148373351	230096	455,88	-0,66	1,04E-01
ENSRNOG00000004085	**Tcaim**	T cell activation inhibitor, mitochondria	363169	8	131000653	131031922	31270	543,47	-0,73	1,04E-01
ENSRNOG00000011900		Fragile X mental retardation protein 1	24948	1	150408231	150445610	37380	533,03	-0,88	1,04E-01
ENSRNOG00000032703	**Rasgrp3**	RAS guanyl releasing protein 3 (calciu	313874	6	30966364	31029468	63105	22,49	-0,96	1,04E-01
ENSRNOG00000049452		Protein Nebl; RCG55853, isoform CRA_b [Sour		17	85874473	86219535	345063	186,83	-0,79	1,04E-01
ENSRNOG00000016429		glutamate receptor, metabotropic 5 (G	24418	1	157535334	158095289	559956	1814,68	-0,70	1,05E-01
ENSRNOG00000033835		dynamin 1 (Dnm1), mRNA [Source:Re	140694	3	16686951	16730913	43963	4910,63	-0,63	1,05E-01
ENSRNOG00000002013	**Sidt1**	SID1 transmembrane family, member	288109	11	65429664	65524944	95281	355,35	-0,68	1,05E-01
ENSRNOG00000005114	**Tmem74**	Protein Tmem74 [Source:UniProtKB/	500864	7	82695682	82696599	918	194,04	-0,65	1,05E-01
ENSRNOG00000014058	**Tomm22**	translocase of outer mitochondrial me	300075	7	120892307	120894054	1748	3464,55	0,49	1,05E-01
ENSRNOG00000019109	**Thap11**	THAP domain containing 11 (Thap11),	307806	19	48692347	48694164	1818	1175,82	0,43	1,05E-01
ENSRNOG00000024953		Protein RGD1559441 [Source:UniPro	500410	5	29582046	29624703	42658	455,55	0,67	1,05E-01
ENSRNOG00000026392	**Cdh12**	Protein Cdh12 [Source:UniProtKB/Tr	301887	2	91863900	92268636	404737	112,16	0,62	1,05E-01
ENSRNOG00000029197	**Diablo**	diablo, IAP-binding mitochondrial prot	288753	12	40372113	40385230	13118	494,79	0,43	1,05E-01
ENSRNOG00000048516	**LOC68368**	transmembrane protein 29 [Source:M	683684	X	21552599	21553213	615	31,52	-0,93	1,05E-01

ENSRNOG00000007332	**Sstr3**	somatostatin receptor 3 (Sstr3), mRN/	171044	7	119749198	119756392	7195	28,28	-1,10	1,05E-01
ENSRNOG00000006694	**Cav1**	caveolin 1, caveolae protein (Cav1), tr	25404	4	45203434	45236460	33027	304,48	-0,87	1,06E-01
ENSRNOG00000042728	**Fbrs**	Protein Fbrs; RCG40094, isoform CR/	691899	1	205933261	205938141	4881	639,20	0,45	1,06E-01
ENSRNOG00000014197	**Tmem51**	transmembrane protein 51 (Tmem51),	500578	5	164281861	164331199	49339	114,33	-1,11	1,06E-01
ENSRNOG00000026810	**LOC30840**	Protein LOC308401 [Source:UniProtK	308401	1	80991850	80994591	2742	51,28	-0,88	1,06E-01
ENSRNOG00000008323	**Pitpnm3**	Protein Pitpnm3 [Source:UniProtKB/T	287467	10	58363189	58434010	70822	203,26	-0,84	1,06E-01
ENSRNOG00000012121	**Sdf2**	stromal cell derived factor 2 (Sdf2), ml	287470	10	66119399	66129873	10475	2143,32	0,40	1,06E-01
ENSRNOG00000021261	**Rassf2**	Ras association (RalGDS/AF-6) doma	311437	3	131075323	131109910	34588	1940,77	-0,46	1,07E-01
ENSRNOG00000038999	**RT1-A1**	RT1 class Ia, locus A1 (RT1-A1), mRNA [Source		20	7474639	7478155	3517	417,37	1,02	1,07E-01
ENSRNOG00000047453		CAS1 domain containing 1 [Source:MGI Symbol		4	29552031	29602488	50458	464,06	-0,70	1,07E-01
ENSRNOG00000012820		adducin 3 (gamma) (Add3), transcript	25230	1	281267469	281375203	107735	2983,19	-0,72	1,08E-01
ENSRNOG00000027194	**Sgsm2**	small G protein signaling modulator 2	303304	10	61425575	61466833	41259	2121,41	-0,46	1,09E-01
ENSRNOG00000039876	**LOC68141**	Protein Hnrnpa0 [Source:UniProtKB/T	498696	17	9112369	9113277	909	4534,30	0,53	1,10E-01
ENSRNOG00000040303		Protein 1110001A16Rik; RCG62056, isoform CF		6	1506267	1507727	1461	138,03	-0,60	1,10E-01
ENSRNOG00000047741	**E2f2**	Protein LOC684111 [Source:UniProtK	684111	5	158290338	158310319	19982	58,32	0,89	1,12E-01
ENSRNOG00000020636		serine peptidase inhibitor, Kunitz type,	292770	1	88380000	88402455	22456	1169,29	0,58	1,12E-01
ENSRNOG00000034268	**Smarcd1**	SWI/SNF related, matrix associated, a	363002	X	115860833	115871373	10541	4826,58	0,52	1,12E-01
ENSRNOG00000010488	**Zmiz1**	zinc finger, MIZ-type containing 1 (Zmi	361103	16	1726706	1932218	205513	7188,46	-0,77	1,12E-01
ENSRNOG00000012144	**Kcng1**	potassium voltage-gated channel, sub	296395	3	171047847	171178647	130801	759,70	-0,79	1,12E-01
ENSRNOG00000018414	**Csf1r**	colony stimulating factor 1 receptor (C	307403	18	55662900	55689297	26398	38,12	-1,10	1,13E-01
ENSRNOG00000007842	**Aup1**	ancient ubiquitous protein 1 (Aup1), m	680423	4	178572182	178575201	3020	2022,49	0,45	1,13E-01
ENSRNOG00000011150	**Arsb**	arylsulfatase B (Arsb), mRNA [Source	25227	2	42559883	42719688	159806	2631,38	-0,65	1,13E-01
ENSRNOG00000002418	**Tgfb2**	transforming growth factor, beta 2 (Tg	81809	13	109681282	109791576	110295	1613,32	-0,66	1,13E-01
ENSRNOG00000015727	**Loxl4**	lysyl oxidase-like 4 (Loxl4), mRNA [So	309380	1	269286675	269305014	18340	19,79	-1,10	1,13E-01
ENSRNOG00000021882	**Tmed9**	transmembrane emp24 protein transp	361207	17	11668171	11672682	4512	4262,00	0,59	1,13E-01
ENSRNOG00000050748	**Dpp9**	Protein Dpp9 [Source:UniProtKB/TrEI	301130	9	9846698	9871136	24439	1182,22	0,49	1,13E-01
ENSRNOG00000002436	**Mmd**	monocyte to macrophage differentiatic	303439	10	77617043	77645125	28083	3913,86	-0,56	1,14E-01
ENSRNOG00000011861	**Aadat**	aminoadipate aminotransferase (Aada	29416	16	32665787	32702406	36620	112,03	-1,01	1,14E-01
ENSRNOG00000017109	**Rassf7**	Ras association (RalGDS/AF-6) doma	293623	1	221121400	221123321	1922	90,28	0,78	1,14E-01
ENSRNOG00000028270	**Ctu2**	cytosolic thiouridylase subunit 2 homo	292069	19	66009931	66014982	5052	542,29	0,59	1,14E-01
ENSRNOG00000006207	**Bloc1s4**	biogenesis of lysosomal organelles co	364183	14	87207216	87208516	1301	597,20	0,50	1,15E-01
ENSRNOG00000036649	**Pwwp2b**	PWWP domain containing 2B (Pwwp2	361671	1	218506152	218524764	18613	156,87	0,58	1,15E-01
ENSRNOG00000019268	**Pelp1**	proline, glutamate and leucine rich prc	360552	10	56739145	56756177	17033	2438,84	0,46	1,15E-01
ENSRNOG00000017213	**Cdk11b**	cyclin-dependent kinase 11B (Cdk11b), mRNA [S		5	176542671	176553642	10972	796,31	0,48	1,15E-01
ENSRNOG00000007047	**Eps8**	Epidermal growth factor receptor kinas	312812	4	235734054	235794456	60403	644,82	-0,64	1,16E-01
ENSRNOG00000026691	**Coq4**	coenzyme Q4 homolog (S. cerevisiae)	366013	3	13692575	13700907	8333	487,25	0,65	1,16E-01
ENSRNOG00000043094	**Oxct1**	3-oxoacid CoA transferase 1 (Oxct1),	690163	2	72891713	73037614	145902	2898,70	-1,03	1,16E-01
ENSRNOG00000005108	**Wfs1**	Wolfram syndrome 1 (wolframin) (Wfs	83725	14	78613738	78630689	16952	639,18	-0,66	1,17E-01

ENSRNOG00000021356	**Dnah2**	Protein Dnah2 [Source:UniProtKB/TrE	303242	10	55768511	55892139	123629	66,08	-0,80	1,17E-01
ENSRNOG00000000457	**Tap1**	transporter 1, ATP-binding cassette, su	24811	20	6058583	6069216	10634	685,16	0,90	1,17E-01
ENSRNOG00000002165	**Fam175a**	family with sequence similarity 175, m	289468	14	10372640	10386976	14337	72,33	-0,73	1,17E-01
ENSRNOG00000020683	**Hnrnpul1**	heterogeneous nuclear ribonucleoprot	361522	1	83774021	83808198	34178	5699,48	-0,54	1,17E-01
ENSRNOG00000023220	**Cnot11**	CCR4-NOT transcription complex, sub	363221	9	45958835	45968530	9696	772,85	0,65	1,17E-01
ENSRNOG00000010905	**Dlx5**	distal-less homeobox 5 (Dlx5), mRNA	25431	4	32255323	32259589	4267	558,29	0,70	1,17E-01
ENSRNOG00000007110	**Ankrd6**	ankyrin repeat domain 6 (Ankrd6), mR	500430	5	52640129	52778272	138144	1650,76	-0,67	1,18E-01
ENSRNOG00000020533	**Htra1**	HtrA serine peptidase 1 (Htra1), mRN/	65164	1	208531988	208581539	49552	521,43	-0,81	1,18E-01
ENSRNOG00000007033	**Sorcs2**	sortilin-related VPS10 domain containi	305438	14	79176694	79547823	371130	2173,48	-0,63	1,18E-01
ENSRNOG00000014714	**Trpv6**	transient receptor potential cation char	114246	4	135705697	135721360	15664	260,53	-0,78	1,18E-01
ENSRNOG00000026942	**RGD13115**	Protein RGD1311595 [Source:UniPro	309307	1	255151574	255211033	59460	821,23	-0,57	1,18E-01
ENSRNOG00000045524	**Slc39a3**	solute carrier family 39 (zinc transporte	314637	7	11712392	11720741	8350	2002,37	0,57	1,19E-01
ENSRNOG00000047896	**Kctd10**	potassium channel tetramerization dor	494521	12	49784215	49801889	17675	3952,89	0,65	1,19E-01
ENSRNOG00000050996	**Kctd4**	potassium channel tetramerization dor	691835	15	61719150	61722354	3205	151,60	-0,83	1,19E-01
ENSRNOG00000020255	**Sars**	seryl-tRNA synthetase (Sars), mRNA [	266975	2	230689716	230705606	15891	7183,89	0,51	1,19E-01
ENSRNOG00000031169	**Nkap**	NFKB activating protein (Nkap), mRN/	298342	X	123887440	123907038	19599	564,53	0,61	1,19E-01
ENSRNOG00000001047	**Map2k7**	mitogen activated protein kinase kinas	363855	12	4700990	4707936	6947	534,47	0,60	1,19E-01
ENSRNOG00000015717	**Ptpre**	protein tyrosine phosphatase, recepto	114767	1	214879098	214919139	40042	344,99	-0,77	1,19E-01
ENSRNOG00000024278	**Rbm42**	RNA binding motif protein 42 (Rbm42)	361545	1	90254914	90264844	9931	2271,01	0,44	1,19E-01
ENSRNOG00000033426	**Cdc37**	cell division cycle 37 (Cdc37), mRNA [	114562	8	22217096	22229971	12876	5199,27	0,65	1,20E-01
ENSRNOG00000038202	**Calml4**	calmodulin-like 4 (Calml4), mRNA [So	691455	8	67482026	67494105	12080	74,53	-1,09	1,20E-01
ENSRNOG00000018599	**Gga2**	golgi associated, gamma adaptin ear (	293455	1	198729100	198856172	127073	1781,80	0,74	1,20E-01
ENSRNOG00000008709	**Arhgap32**	Protein Arhgap32 [Source:UniProtKB/	315530	8	33257762	33406716	148955	2885,54	-0,48	1,21E-01
ENSRNOG00000008456	**Thoc5**	THO complex 5 (Thoc5), mRNA [Sour	360972	14	85820114	85849561	29448	1678,28	0,42	1,21E-01
ENSRNOG00000012378	**Dbnl**	drebrin-like (Dbnl), transcript variant 1	83527	14	86721144	86735820	14677	2892,82	0,47	1,21E-01
ENSRNOG00000008990		angiomotin-like 1 [Source:MGI Symbo	315430	8	12987687	13029539	41853	382,72	0,56	1,22E-01
ENSRNOG00000022623	**Ttll12**	tubulin tyrosine ligase-like family, mem	300105	7	124461238	124479484	18247	1286,52	0,39	1,22E-01
ENSRNOG00000023712	**Stox1**	storkhead box 1 (Stox1), mRNA [Sour	294388	20	34061421	34137583	76163	25,12	-0,89	1,22E-01
ENSRNOG00000037613	**Kdm6b**	lysine (K)-specific demethylase 6B (Kc	363630	10	55743758	55750732	6975	1990,28	0,47	1,22E-01
ENSRNOG00000001185	**RGD13118**	Uncharacterized protein C12orf43 hon	288704	12	49226757	49232350	5594	4855,34	0,57	1,22E-01
ENSRNOG00000017392	**Fgf2**	fibroblast growth factor 2 (Fgf2), mRN.	54250	2	143689407	143742070	52664	35,14	-1,04	1,22E-01
ENSRNOG00000018046	**Lgals8**	lectin, galactoside-binding, soluble, 8 (	116641	17	68233213	68257626	24414	385,43	-0,69	1,22E-01
ENSRNOG00000049066	**Mrfap1**	Morf4 family associated protein 1 (Mrf	282585	14	87168521	87170315	1795	10011,42	0,56	1,22E-01
ENSRNOG00000009726	**Grip2**	glutamate receptor interacting protein	171571	4	188120214	188170081	49868	769,93	-0,47	1,23E-01
ENSRNOG00000013067	**Phf21b**	PHD finger protein 21B (Phf21b), mRN	300117	7	125370846	125438318	67473	290,64	0,66	1,23E-01
ENSRNOG00000013917	**Igsf10**	immunoglobulin superfamily, member	310448	2	168967575	168996281	28707	492,67	-0,91	1,23E-01
ENSRNOG00000049719		Uncharacterized protein [Source:UniProtKB/TrE		4	17005068	17031483	26416	377,69	-0,75	1,23E-01
ENSRNOG00000033747	**Sp110**	SP110 nuclear body protein (Sp110), r	301570	9	92324922	92346959	22038	188,12	0,92	1,23E-01

ENSRNOG00000028149		Protein Kansl1l [Source:UniProtKB/Tr	367289	9	73506962	73593827	86866	186,08	-0,68	1,23E-01
ENSRNOG00000005957	**Slc4a7**	solute carrier family 4, sodium bicarbo	117955	15	15875420	15950530	75111	436,25	-0,46	1,23E-01
ENSRNOG00000014528	**Zswim6**	Protein Zswim6 [Source:UniProtKB/Tr	310062	2	58070319	58098435	28117	827,50	-0,61	1,23E-01
ENSRNOG00000009683	**Sdcbp**	syndecan binding protein (Sdcbp), mR	83841	5	24256128	24283025	26898	3474,79	-0,69	1,23E-01
ENSRNOG00000004143	**Adipor1**	adiponectin receptor 1 (Adipor1), mRN	289036	13	56297571	56316893	19323	4189,77	0,59	1,24E-01
ENSRNOG00000007805	**Mybl2**	myeloblastosis oncogene-like 2 (Mybl2	296344	3	165617194	165645609	28416	497,13	0,75	1,24E-01
ENSRNOG00000006984	**Mapk11**	mitogen-activated protein kinase 11 (M	689314	7	129807476	129814393	6918	697,64	0,53	1,24E-01
ENSRNOG00000008291	**Thumpd2**	THUMP domain containing 2 (Thumpd	313851	6	3710781	3762198	51418	148,59	-0,54	1,24E-01
ENSRNOG00000010894	**Tmem203**	transmembrane protein 203 (Tmem20	311800	3	2456385	2457213	829	592,39	0,46	1,24E-01
ENSRNOG00000012422	**Tnik**	TRAF2 and NCK interacting kinase (T	294917	2	133688980	134082229	393250	2503,84	-0,57	1,24E-01
ENSRNOG00000045825	**Ppp1r3d**	protein phosphatase 1, regulatory sub	689995	3	183137515	183140586	3072	461,69	0,56	1,24E-01
ENSRNOG00000005442	**Tspan9**	tetraspanin 9 (Tspan9), mRNA [Source	312728	4	231011142	231085237	74096	1631,74	-0,53	1,24E-01
ENSRNOG00000002737	**Sh3bp5l**	SH3 binding domain protein 5 like (Sh	690898	10	43561167	43575473	14307	1856,34	0,56	1,25E-01
ENSRNOG00000021902	**Rmi2**	Protein Rmi2 [Source:UniProtKB/TrEI	497856	10	3733421	3738516	5096	43,48	0,86	1,25E-01
ENSRNOG00000002278	**Tec**	tec protein tyrosine kinase (Tec), mRN	84492	14	37729490	37838224	108735	71,54	-0,78	1,26E-01
ENSRNOG00000019496	**Stat5a**	signal transducer and activator of tran	24918	10	88558936	88583261	24326	61,53	0,87	1,27E-01
ENSRNOG00000001432	**Dtx2**	deltex homolog 2 (Drosophila) (Dtx2),	304591	12	25686614	25726212	39599	355,66	0,50	1,28E-01
ENSRNOG00000002443	**Mfap3**	microfibrillar-associated protein 3 (Mfa	287299	10	42738203	42750690	12488	802,55	-0,60	1,28E-01
ENSRNOG00000008992	**Grik3**	glutamate receptor, ionotropic, kainate	298521	5	147265647	147480507	214861	1069,16	-0,73	1,28E-01
ENSRNOG00000049063		solute carrier family 16 (monocarboxyl	687808	20	21772669	21801047	28379	29,59	-0,84	1,29E-01
ENSRNOG00000003279	**Syce2**	synaptonemal complex central elemer	364976	19	36953712	36976721	23010	48,13	-0,76	1,29E-01
ENSRNOG00000043286	**Cpne2**	copine II (Cpne2), mRNA [Source:Ref	498972	19	10855268	10877311	22044	638,83	-0,71	1,29E-01
ENSRNOG00000017882	**Camk1d**	calcium/calmodulin-dependent protein	307124	17	78187614	78591805	404192	885,20	-0,61	1,29E-01
ENSRNOG00000017893	**Baiap3**	Protein Baiap3 [Source:UniProtKB/Tr	685297	10	14418873	14427212	8340	30,45	-0,90	1,29E-01
ENSRNOG00000019824	**LOC10091**	elongation factor RNA polymerase II (I	306347	16	20449840	20497154	47315	555,43	0,43	1,29E-01
ENSRNOG00000020178		coatomer subunit epsilon [Source:Ref	290659	16	20714916	20725117	10202	3669,71	0,57	1,29E-01
ENSRNOG00000020882	**Shkbp1**	Sh3kbp1 binding protein 1 (Shkbp1), r	292735	1	85366002	85379188	13187	448,91	0,68	1,29E-01
ENSRNOG00000018708	**Ppp1ca**	protein phosphatase 1, catalytic subur	24668	1	226310595	226314221	3627	8686,72	0,48	1,29E-01
ENSRNOG00000021245	**RGD13117**	similar to RIKEN cDNA 1700037H04 (	311428	3	130187020	130201122	14103	1714,94	0,63	1,30E-01
ENSRNOG00000012061		protein kinase C, beta (Prkcb), transcr	25023	1	199295067	199639137	344071	1950,42	-0,79	1,30E-01
ENSRNOG00000020412	**Sf3b2**	splicing factor 3b, subunit 2 (Sf3b2), m	293671	1	227575699	227595347	19649	7845,32	0,47	1,31E-01
ENSRNOG00000013869	**Kcnj4**	potassium inwardly-rectifying channel,	116649	7	120710791	120738030	27240	438,71	-0,65	1,31E-01
ENSRNOG00000017585	**Abt1**	activator of basal transcription 1 (Abt1	306960	17	45800802	45802895	2094	887,25	0,69	1,31E-01
ENSRNOG00000010210	**Slc7a11**	solute carrier family 7 (anionic amino a	310392	2	158930294	159004611	74318	2543,21	-1,06	1,31E-01
ENSRNOG00000031207	**LOC50003**	uncharacterized protein LOC500035		500035	4	39988834	39997113	8280	1177,16	1,02	1,32E-01
ENSRNOG00000008047		ketohexokinase [Source:RefSeq pept	25659	6	36626194	36636426	10233	321,47	0,67	1,32E-01
ENSRNOG00000014272	**LOC36705**	ribosomal protein L35 (Rpl35), mRNA	296709	3	28516640	28519600	2961	5205,60	0,47	1,32E-01
ENSRNOG00000023237		Uncharacterized protein [Source:UniProtKB/TrE		5	148800259	148800906	648	22,17	0,96	1,32E-01

ENSRNOG00000028187	**Sp9**	trans-acting transcription factor 9 (Sp9	366078	3	66454296	66456826	2531	392,01	0,61	1,32E-01
ENSRNOG00000007054	**Dhx33**	DEAH (Asp-Glu-Ala-His) box polypept	287464	10	57367234	57383697	16464	606,88	-0,52	1,32E-01
ENSRNOG00000006619		dnaJ homolog subfamily C member 9	364240	15	8457759	8462062	4304	908,08	0,49	1,32E-01
ENSRNOG00000049083	**Nicn1**	nicolin 1 (Nicn1), mRNA [Source:RefS	619581	8	116415838	116420979	5142	1647,91	0,56	1,32E-01
ENSRNOG00000018945	**Nutf2**	nuclear transport factor 2 (Nutf2), mRN	291981	19	48697685	48718957	21273	2567,17	0,57	1,33E-01
ENSRNOG00000025936	**Rpl6**	ribosomal protein L6 (Rpl6), mRNA [Source:RefS		12	42744695	42749109	4415	631,75	0,49	1,33E-01
ENSRNOG00000013855	**Zmym1**	zinc finger, MYM-type 1 (Zmym1), mR	313604	5	148876980	148894226	17247	239,91	-0,68	1,33E-01
ENSRNOG00000015514	**Bcat1**	branched chain amino acid transamina	29592	4	243444839	243491574	46736	22958,42	0,58	1,33E-01
ENSRNOG00000036682	**Pycr1**	pyrroline-5-carboxylate reductase 1 (P	287877	10	109411300	109414672	3373	346,03	-0,84	1,34E-01
ENSRNOG00000002894	**Mnt**	MNT, MAX dimerization protein (Mnt),	287521	10	61406977	61422227	15251	2060,20	0,51	1,34E-01
ENSRNOG00000006049	**Rfx1**	regulatory factor X, 1 (influences HLA	288906	19	36158698	36189544	30847	606,04	0,55	1,34E-01
ENSRNOG00000019522	**Narfl**	nuclear prelamin A recognition factor-li	360496	10	14956675	14965648	8974	639,05	0,46	1,34E-01
ENSRNOG00000024066	**Fundc2**	FUN14 domain containing 2 (Fundc2),	361288	18	447545	453204	5660	518,41	0,54	1,34E-01
ENSRNOG00000007151	**Cdk14**	cyclin-dependent kinase 14 (Cdk14), r	362316	4	25732186	26326121	593936	2035,97	-0,36	1,35E-01
ENSRNOG00000039861	**LOC69061**	RIKEN cDNA 4930404N11 gene [Sour	690617	7	11382219	11383981	1763	449,26	0,56	1,35E-01
ENSRNOG00000019342	**Sult1a1**	sulfotransferase family, cytosolic, 1A,	83783	1	205079584	205083124	3541	43,73	-0,87	1,35E-01
ENSRNOG00000020706	**Kcnn3**	potassium intermediate/small conducta	54263	2	208267083	208411808	144726	251,98	-0,62	1,35E-01
ENSRNOG00000020659	**Mrpl4**	mitochondrial ribosomal protein L4 (Mr	363023	8	22078081	22084096	6016	1941,23	0,45	1,35E-01
ENSRNOG00000006591	**Fam163b**	family with sequence similarity 163, m	685169	3	11020099	11054772	34674	412,31	-0,63	1,36E-01
ENSRNOG00000018698		WW domain containing adaptor with coiled-coil [		17	62664132	62722879	58748	1123,63	-0,44	1,36E-01
ENSRNOG00000002277		nuclear receptor corepressor 1 [Sourc	54299	10	48417491	48559314	141824	4233,31	-0,54	1,36E-01
ENSRNOG00000000575	**Ascc1**	activating signal cointegrator 1 comple	294512	20	31364396	31450468	86073	1134,48	0,39	1,36E-01
ENSRNOG00000007713	**Tmcc3**	transmembrane and coiled-coil domai	314751	7	35536671	35608377	71707	880,79	-0,62	1,36E-01
ENSRNOG00000004330	**Chrdl1**	chordin-like 1 (Chrdl1), mRNA [Source	363455	X	112998868	113102972	104105	140,05	-0,71	1,36E-01
ENSRNOG00000018403	**Atg4b**	autophagy related 4B, cysteine peptida	316640	9	100564123	100573271	9149	2150,61	0,63	1,37E-01
ENSRNOG00000006526	**Sema3c**	sema domain, immunoglobulin domain	296787	4	14293372	14464552	171181	699,53	-0,93	1,37E-01
ENSRNOG00000012543	**Mcm3**	minichromosome maintenance comple	316273	9	25770288	25788415	18128	709,41	0,63	1,37E-01
ENSRNOG00000001585	**Nrip1**	nuclear receptor interacting protein 1 (	304157	11	18314226	18397564	83339	824,23	-0,77	1,38E-01
ENSRNOG00000009512	**Esd**	esterase D (Esd), transcript variant 2,	290401	15	60479262	60498753	19492	1002,96	-0,46	1,38E-01
ENSRNOG00000000157	**Atg12**	autophagy related 12 (Atg12), mRNA	361321	18	40367827	40378153	10327	2749,11	0,64	1,38E-01
ENSRNOG00000049355	**Arxes2**	Signal peptidase complex subunit 3 [S	317409	X	106598456	106598998	543	87,27	-0,68	1,38E-01
ENSRNOG00000005874	**Tle2**	transducin-like enhancer of split 2 (E(s	299636	7	11155631	11170879	15249	291,94	0,55	1,38E-01
ENSRNOG00000011613	**Ppip5k2**	diphosphoinositol pentakisphosphate k	501194	9	110786864	110800883	14020	249,63	0,66	1,38E-01
ENSRNOG00000017406	**Atrnl1**	Protein Atrnl1 [Source:UniProtKB/TrE	307992	1	285926442	286113279	186838	1564,70	-0,56	1,38E-01
ENSRNOG00000038166		prostaglandin reductase 2 (Ptgr2), mR	299194	6	117514754	117546551	31798	1349,22	-0,76	1,38E-01
ENSRNOG00000012504		WAS protein family homolog [Source:N	367328	9	113443347	113459271	15925	1160,72	0,39	1,38E-01
ENSRNOG00000039852	**Cactin**	cactin, spliceosome C complex subuni	500790	7	11427936	11432741	4806	754,10	0,40	1,38E-01
ENSRNOG00000003675	**Ptchd1**	patched domain containing 1 (Ptchd1)	317517	X	42866040	42917474	51435	82,23	-0,66	1,39E-01

ENSRNOG00000034040	**Rnf113a1**	ring finger protein 113A1 (Rnf113a1), r	313450	X	124602718	124603842	1125	282,78	0,61	1,39E-01
ENSRNOG00000015762	**Abtb1**	ankyrin repeat and BTB (POZ) domair	297432	4	186019189	186025416	6228	426,21	0,71	1,39E-01
ENSRNOG00000001217	**Lrrc3**	leucine rich repeat containing 3 (Lrrc3	246773	20	13659350	13660300	951	118,02	-0,73	1,39E-01
ENSRNOG00000004909	**Cnot2**	CCR4-NOT transcription complex, sub	299805	7	59542042	59598859	56818	814,11	-0,49	1,39E-01
ENSRNOG00000009960	**Puf60**	poly-U binding splicing factor 60 (Puf6	84401	7	117114908	117125839	10932	5750,48	0,38	1,39E-01
ENSRNOG00000010106	**Faxc**	failed axon connections (Faxc), mRNA	366333	5	40729491	40787422	57932	298,66	-0,44	1,39E-01
ENSRNOG00000013281	**Mib1**	mindbomb E3 ubiquitin protein ligase	307594	18	2003879	2127899	124021	2096,67	-0,64	1,39E-01
ENSRNOG00000014619	**Dhx38**	DEAH (Asp-Glu-Ala-His) box polypept	292007	19	52935420	52952669	17250	1497,56	0,54	1,39E-01
ENSRNOG00000027260	**Adprh**	ADP-ribosylarginine hydrolase (Adprh	25371	11	67241832	67248168	6337	1624,21	0,41	1,39E-01
ENSRNOG00000034191	**Fmo1**	flavin containing monooxygenase 1 (F	25256	13	85607536	85639986	32451	200,26	-1,05	1,40E-01
ENSRNOG00000048129	**LOC68210**	cDNA sequence CK137956 [Source:M	682102	5	149782638	149824585	41948	36,16	-1,00	1,40E-01
ENSRNOG00000029678		zinc finger protein 354B [Source:MGI Symbol;Ac		10	36185559	36194975	9417	26,85	-0,88	1,40E-01
ENSRNOG00000021410	**Negr1**	neuronal growth regulator 1 (Negr1), mRNA [Sou		2	281883336	282071757	188422	572,03	-0,57	1,41E-01
ENSRNOG00000049785		Protein Ranbp3 [Source:UniProtKB/T	501281	9	9341273	9378345	37073	1998,70	0,46	1,41E-01
ENSRNOG00000011904	**Trappc4**	trafficking protein particle complex 4 (T	367073	8	47342732	47345957	3226	1206,28	0,41	1,42E-01
ENSRNOG00000019079	**Polr1c**	polymerase (RNA) I polypeptide C (Po	301246	9	16017436	16021548	4113	1062,79	0,52	1,42E-01
ENSRNOG00000029336	**Zfp180**	zinc finger protein 180 (Zfp180), trans	246279	1	82186002	82205285	19284	1307,60	-0,62	1,43E-01
ENSRNOG00000001079		Sn1-specific diacylglycerol lipase beta	304289	12	15156222	15197954	41733	614,11	0,58	1,43E-01
ENSRNOG00000027156	**Morn4**	MORN repeat containing 4 (Morn4), m	293950	1	268775555	268785855	10301	2916,41	0,67	1,43E-01
ENSRNOG00000011634	**Xkr6**	XK, Kell blood group complex subunit-	305960	15	50706269	50932560	226292	88,52	-0,66	1,43E-01
ENSRNOG00000008634	**Cc2d1b**	coiled-coil and C2 domain containing	313478	5	132057675	132070250	12576	777,50	0,51	1,44E-01
ENSRNOG00000021802	**Isg15**	ISG15 ubiquitin-like modifier (Isg15), r	298693	5	177098680	177099967	1288	305,58	1,04	1,44E-01
ENSRNOG00000010667	**Ept1**	selenoprotein I (Seli), mRNA [Source:I	362713	6	37285873	37297955	12083	888,16	-0,68	1,44E-01
ENSRNOG00000015415	**LOC10091**	ras homolog family member Q (Rhoq)	85428	6	20521668	20557098	35431	946,55	-0,46	1,44E-01
ENSRNOG00000049150		LOC100125377 protein; RCG58522; Uncharacte		20	55379701	55381505	1805	35,29	-0,92	1,44E-01
ENSRNOG00000015339	**Shoc2**	soc-2 (suppressor of clear) homolog (	309548	1	282112968	282163537	50570	1313,82	-0,46	1,45E-01
ENSRNOG00000021054	**Men1**	multiple endocrine neoplasia I (Men1),	29417	1	228692516	228698421	5906	2098,05	0,37	1,45E-01
ENSRNOG00000013570		Uncharacterized protein [Source:UniProtKB/TrE		8	114753015	114811529	58515	879,32	0,55	1,46E-01
ENSRNOG00000046599	**RGD15616**	similar to Kelch domain containing 4 (I	307917	19	65371365	65371835	471	121,59	0,79	1,46E-01
ENSRNOG00000049772	**Gemin7l1**	gem (nuclear organelle) associated pr	499391	1	81739987	81749508	9522	94,58	0,66	1,46E-01
ENSRNOG00000005209	**Spred1**	sprouty-related, EVH1 domain contain	296072	3	115352442	115414136	61695	647,07	-0,43	1,46E-01
ENSRNOG00000017414	**Irf7**	interferon regulatory factor 7 (Irf7), mR	293624	1	221166927	221169993	3067	109,77	1,05	1,46E-01
ENSRNOG00000020487	**Coa3**	cytochrome C oxidase assembly facto	498000	10	88997136	88998122	987	848,34	0,54	1,46E-01
ENSRNOG00000022043	**Yipf2**	Yip1 domain family, member 2 (Yipf2),	363027	8	22675736	22679913	4178	474,52	0,65	1,46E-01
ENSRNOG00000002529	**Rap2c**	RAP2C, member of RAS oncogene fa	302495	X	138277499	138290616	13118	1358,84	-0,46	1,46E-01
ENSRNOG00000006411	**Cav2**	caveolin 2 (Cav2), mRNA [Source:Ref	363425	4	45179465	45186995	7531	159,78	-0,88	1,46E-01
ENSRNOG00000009336	**Mapk15**	mitogen-activated protein kinase 15 (M	286997	7	117041464	117046797	5334	53,97	-0,85	1,46E-01
ENSRNOG00000011414	**Psmc3**	proteasome (prosome, macropain) 26	29677	3	86585691	86591072	5382	5485,85	0,48	1,46E-01

ENSRNOG00000014208	**Dctn3l1**	dynactin 3 (Dctn3), mRNA [Source:Re	362504	5	62622817	62630771	7955	1487,20	0,60	1,46E-01
ENSRNOG00000032546	**Dot1l**	DOT1-like histone H3K79 methyltrans	362831	7	11953820	11992519	38700	1528,51	0,47	1,46E-01
ENSRNOG00000036719	**Samd10**	sterile alpha motif domain containing 1	499957	3	180801648	180805061	3414	878,69	0,65	1,46E-01
ENSRNOG00000047057	**Gpr26**	G protein-coupled receptor 26 (Gpr26)	192153	1	210974995	210991992	16998	161,66	-0,63	1,46E-01
ENSRNOG00000000663	**Tfip11**	tuftelin interacting protein 11 (Tfip11), r	288718	12	52077899	52090133	12235	1416,20	0,54	1,46E-01
ENSRNOG00000017183	**Ctdp1**	CTD (carboxy-terminal domain, RNA p	291414	18	71828756	71889824	61069	1008,76	0,44	1,46E-01
ENSRNOG00000048258	**Cisd2**	CDGSH iron sulfur domain 2 (Cisd2), r	295457	2	259106753	259130712	23960	1502,84	-0,38	1,47E-01
ENSRNOG00000002473	**RGD13078**	Protein RGD1307830 [Source:UniPro	304863	13	72548442	72574290	25849	113,37	-0,65	1,47E-01
ENSRNOG00000033854	**Adh5**	alcohol dehydrogenase 5 (class III), ch	1E+08	2	262262880	262275335	12456	3525,04	0,45	1,47E-01
ENSRNOG00000037951	**Nhsl2**	Protein Nhsl2 [Source:UniProtKB/TrE	317253	X	72805923	72817514	11592	77,97	-0,84	1,47E-01
ENSRNOG00000045943	**Rnf170**	Protein Rnf170; Similar to ring finger p	364654	16	70353877	70375249	21373	229,58	-0,53	1,47E-01
ENSRNOG00000000465	**Slc39a7**	solute carrier family 39 (zinc transporte	294281	20	5898748	5902119	3372	3171,09	0,36	1,48E-01
ENSRNOG00000025832	**Zer1**	zyg-11 related, cell cycle regulator (Ze	311842	3	14014737	14042463	27727	3427,15	0,41	1,48E-01
ENSRNOG00000008890	**Slc18a2**	solute carrier family 18 (vesicular mon	25549	1	287783563	287817632	34070	115,25	-0,68	1,48E-01
ENSRNOG00000025000	**Cep104**	glycine-, glutamate-, thienylcyclohexyl	246295	5	174757446	174786094	28649	1264,26	0,46	1,48E-01
ENSRNOG00000011876	**Fxr2**	fragile X mental retardation, autosoma	287433	10	55983452	56003515	20064	3513,20	0,60	1,48E-01
ENSRNOG00000016010	**Mul1**	mitochondrial E3 ubiquitin protein ligas	298576	5	160603417	160612730	9314	1027,30	0,57	1,48E-01
ENSRNOG00000007630	**Rsl24d1**	ribosomal L24 domain containing 1 (R	363099	8	73286446	73295207	8762	1492,68	0,44	1,49E-01
ENSRNOG00000031431	**Cdca8**	cell division cycle associated 8 (Cdca8	500545	5	146680981	146700100	19120	447,01	0,72	1,49E-01
ENSRNOG00000004362	**Rps6ka5**	ribosomal protein S6 kinase, polypepti	314384	6	133782184	133959292	177109	911,25	-0,51	1,49E-01
ENSRNOG00000011008	**Bet1**	Bet1 golgi vesicular membrane traffick	29631	4	28992532	29002927	10396	709,21	-0,70	1,49E-01
ENSRNOG00000012035	**Rnf181**	ring finger protein 181 (Rnf181), mRNA	297337	4	164992852	164999568	6717	1542,32	0,59	1,49E-01
ENSRNOG00000004026	**Atp2b1**	ATPase, Ca++ transporting, plasma m	29598	7	41154017	41259749	105733	3202,89	-0,50	1,49E-01
ENSRNOG00000016789	**Actr1b**	ARP1 actin-related protein 1 homolog	316333	9	42919562	42929225	9664	4801,72	0,47	1,50E-01
ENSRNOG00000009377	**Nelfb**	negative elongation factor complex me	311796	3	2395734	2412431	16698	3342,60	0,44	1,50E-01
ENSRNOG00000011295	**Anapc2**	anaphase promoting complex subunit	296558	3	2471941	2483659	11719	4876,81	0,57	1,50E-01
ENSRNOG00000023079	**Nras**	neuroblastoma ras oncogene (Nras), r	24605	2	224983750	224991378	7629	815,23	-0,55	1,50E-01
ENSRNOG00000019097	**Bap1**	BRCA1 associated protein-1 (ubiquitin	306257	16	7265075	7273901	8827	6890,09	0,55	1,51E-01
ENSRNOG00000001089		probable palmitoyltransferase ZDHHC	304291	12	15324906	15339325	14420	799,68	0,55	1,51E-01
ENSRNOG00000009730	**Cyp7b1**	cytochrome P450, family 7, subfamily	25429	2	122442002	122610354	168353	945,00	-0,89	1,51E-01
ENSRNOG00000001402	**Lrch4**	leucine-rich repeats and calponin hom	360779	12	24144769	24155758	10990	295,58	0,64	1,51E-01
ENSRNOG00000006324	**Trpc6**	transient receptor potential cation chai	89823	8	6799564	6904624	105061	22,44	-0,96	1,51E-01
ENSRNOG00000003242	**Gulp1**	GULP, engulfment adaptor PTB doma	314543	9	50936854	51211196	274343	142,91	-0,74	1,52E-01
ENSRNOG00000017346	**Myt1**	myelin transcription factor 1 (Myt1), m	362291	3	181019141	181049952	30812	1767,61	0,50	1,52E-01
ENSRNOG00000020363	**Med25**	mediator complex subunit 25 (Med25)	292889	1	101939597	101952474	12878	3119,32	0,46	1,52E-01
ENSRNOG00000022141		CTD small phosphatase-like protein 2	311368	3	120446018	120496804	50787	343,91	-0,56	1,53E-01
ENSRNOG00000001086	**Vps37b**	vacuolar protein sorting 37 homolog B	288659	12	39857153	39894365	37213	2968,78	0,48	1,54E-01
ENSRNOG00000017784	**Lrif1**	ligand dependent nuclear receptor inte	310775	2	228900177	228915197	15021	141,34	-0,78	1,54E-01

ENSRNOG00000020700	**Rnaseh2c**	Protein Rnaseh2c; RCG47232 [Sourc	1E+08	1	227897736	227898678	943	372,08	0,72	1,54E-01
ENSRNOG00000008915	**Prima1**	proline rich membrane anchor 1 (Prim	690195	6	136279361	136330870	51510	17,85	-0,97	1,54E-01
ENSRNOG00000020390	**Tmem161a**	transmembrane protein 161A (Tmem1	364535	16	20836631	20846231	9601	878,00	0,44	1,54E-01
ENSRNOG00000022248	**Fbxl16**	F-box and leucine-rich repeat protein	494223	10	14996746	14999921	3176	1541,97	-0,45	1,55E-01
ENSRNOG00000009046	**Phf13**	PHD finger protein 13 (Phf13), mRNA	313742	5	172719997	172726441	6445	868,97	0,53	1,55E-01
ENSRNOG00000019463	**Mad2l1bp**	MAD2L1 binding protein (Mad2l1bp), r	316237	9	16112968	16117289	4322	340,80	0,55	1,55E-01
ENSRNOG00000021098	**Rasgrp2**	RAS guanyl releasing protein 2 (calciu	361714	1	228761043	228776703	15661	861,08	-0,74	1,55E-01
ENSRNOG00000011489	**R3hdm4**	R3H domain containing 4 (R3hdm4), r	362840	7	12781190	12788308	7119	5605,51	0,52	1,55E-01
ENSRNOG00000042499	**LOC10091**	thymosin, beta 10 (Tmsb10), mRNA [S	50665	4	165645783	165646842	1060	7445,57	0,68	1,55E-01
ENSRNOG00000011771	**LOC10036**	protein tyrosine phosphatase type IVA	29463	9	36028652	36036214	7563	53,17	-0,66	1,56E-01
ENSRNOG00000021058	**Grwd1**	glutamate-rich WD repeat containing 1	308592	1	102892342	102898043	5702	567,96	0,59	1,56E-01
ENSRNOG00000001454	**Eif4h**	eukaryotic translation initiation factor 4	288599	12	27093554	27110196	16643	14452,80	0,45	1,56E-01
ENSRNOG00000050500	**Tob2**	transducer of ERBB2, 2 (Tob2), mRNA	315159	7	123055915	123056955	1041	418,35	0,61	1,56E-01
ENSRNOG00000013165	**Bet1l**	Bet1 golgi vesicular membrane traffick	54400	1	220528199	220530992	2794	1128,82	0,58	1,56E-01
ENSRNOG00000014934	**Fam63b**	Protein Fam63b [Source:UniProtKB/T	363089	8	75993155	76047318	54164	524,60	-0,54	1,56E-01
ENSRNOG00000024426		maestro [Source:MGI Symbol;Acc:MGI:2152817		18	68984920	68993186	8267	111,79	-0,86	1,56E-01
ENSRNOG00000006632	**Rps6ka3**	ribosomal protein S6 kinase polypeptid	501560	X	37777188	37876841	99654	372,70	-0,81	1,56E-01
ENSRNOG00000005180		V-set and transmembrane domain containing 2A		14	99295236	99319815	24580	505,39	-0,66	1,57E-01
ENSRNOG00000013330	**Cdhr1**	cadherin-related family member 1 (Cd	93662	16	14230688	14250567	19880	196,33	-0,49	1,57E-01
ENSRNOG00000028814	**Oasl2**	2'-5' oligoadenylate synthetase-like 2 (	304549	12	49263773	49276267	12495	255,18	1,02	1,57E-01
ENSRNOG00000005546		tRNA-splicing ligase RtcB homolog [Source:Uni		7	60070558	60072604	2047	102,38	0,70	1,58E-01
ENSRNOG00000005334	**Acvr2a**	activin A receptor, type IIA (Acvr2a), m	29263	3	38113728	38197535	83808	1134,20	-0,41	1,58E-01
ENSRNOG00000042696	**Minos1**	mitochondrial inner membrane organiz	362641	5	161285672	161311999	26328	1650,96	0,49	1,58E-01
ENSRNOG00000000805	**Gja1**	gap junction protein, alpha 1 (Gja1), m	24392	20	39612108	39624547	12440	12425,92	-0,99	1,58E-01
ENSRNOG00000013452	**Rcn1**	reticulocalbin 1, EF-hand calcium bind	362182	3	102026257	102040504	14248	2599,07	-0,58	1,58E-01
ENSRNOG00000026930	**Ndufa9**	NADH dehydrogenase (ubiquinone) 1	362440	4	232213354	232241728	28375	3653,71	0,49	1,58E-01
ENSRNOG00000050258	**Ccnd3**	cyclin D3 (Ccnd3), mRNA [Source:Ref	25193	9	14326184	14332305	6122	1479,81	0,59	1,58E-01
ENSRNOG00000010617	**Scube1**	signal peptide, CUB domain, EGF-like	315174	7	124490468	124608680	118213	733,28	-0,49	1,58E-01
ENSRNOG00000017206	**Igfbp5**	insulin-like growth factor binding prote	25285	9	79920613	79937408	16796	21714,13	-0,92	1,58E-01
ENSRNOG00000047756		myocyte enhancer factor 2a (Mef2a), r	309957	1	129276149	129406965	130817	1400,39	-0,47	1,58E-01
ENSRNOG00000008688	**Usp45**	ubiquitin specific peptidase 45 (Usp45	313098	5	40573111	40639486	66376	143,00	-0,76	1,59E-01
ENSRNOG00000009819	**Vezf1**	Protein Vezf1 [Source:UniProtKB/TrE	287615	10	74727293	74736259	8967	937,55	-0,48	1,59E-01
ENSRNOG00000032902	**LOC10091**	Y box binding protein 1 (Ybx1), mRNA [Source:F		10	40219875	40221373	1499	1528,42	-0,63	1,59E-01
ENSRNOG00000048389	**Fgf18**	fibroblast growth factor 18 (Fgf18), mF	29369	10	17932880	17948600	15721	29,91	-0,85	1,59E-01
ENSRNOG00000050220		Uncharacterized protein [Source:UniProtKB/TrE		6	23843134	24308904	465771	1624,88	-0,51	1,59E-01
ENSRNOG00000046857		oxysterol-binding protein-related prote	259221	18	4089699	4273391	183693	2165,53	-0,37	1,59E-01
ENSRNOG00000008423	**Gpr22**	G protein-coupled receptor 22 (Gpr22)	298944	6	59683771	59690751	6981	222,75	-0,95	1,59E-01
ENSRNOG00000012721	**Ednra**	endothelin receptor type A (Ednra), mF	24326	19	44812398	44874904	62507	213,20	-0,82	1,59E-01

ENSRNOG00000019118	**Slc13a3**	solute carrier family 13 (sodium-deper	64846	3	168266579	168329649	63071	541,18	-0,97	1,59E-01
ENSRNOG00000037371		Protein XAF1 [Source:UniProtKB/TrE	679600	10	58601351	58614067	12717	121,68	0,89	1,59E-01
ENSRNOG00000001072	**Cers4**	ceramide synthase 4 (Cers4), mRNA [	304208	12	6568235	6602867	34633	1027,03	-0,71	1,60E-01
ENSRNOG00000003229		tetraspanin 7 (Tspan7), mRNA [Source	363447	X	14051181	14072173	20993	12222,34	-0,53	1,60E-01
ENSRNOG00000007199	**Rapgef3**	Rap guanine nucleotide exchange fac	59326	7	139423980	139445473	21494	255,16	-0,79	1,60E-01
ENSRNOG00000030408	**Dnajb12**	DnaJ (Hsp40) homolog, subfamily B, r	294513	20	31249072	31266937	17866	1060,05	0,39	1,61E-01
ENSRNOG00000049862		ring finger protein 157 [Source:MGI Symbol;Acc		10	104958004	105030267	72264	1449,92	-0,75	1,61E-01
ENSRNOG00000003239	**Cant1**	calcium activated nucleotidase 1 (Can	246272	10	107067504	107080520	13017	1355,38	0,42	1,63E-01
ENSRNOG00000016102	**Ebf3**	early B-cell factor 3 (Ebf3), mRNA [So	116543	1	216447390	216565356	117967	63,27	-0,97	1,63E-01
ENSRNOG00000036698	**Nploc4**	nuclear protein localization 4 homolog	140639	10	109148086	109198455	50370	2508,91	0,46	1,63E-01
ENSRNOG00000037563	**Cd68**	Cd68 molecule (Cd68), mRNA [Source	287435	10	56014365	56016243	1879	46,16	-1,02	1,63E-01
ENSRNOG00000050071	**Cdc45**	cell division cycle 45 (Cdc45), mRNA [	287961	11	89431161	89456061	24901	196,02	0,59	1,63E-01
ENSRNOG00000018385	**Chrm1**	cholinergic receptor, muscarinic 1 (Ch	25229	1	231819886	231821268	1383	244,85	-0,78	1,63E-01
ENSRNOG00000027161	**LOC10036**	similar to RIKEN cDNA 1110059E24 (I	361740	1	246496970	246553306	56337	41,34	-0,77	1,63E-01
ENSRNOG00000036954	**Ddx23**	DEAD (Asp-Glu-Ala-Asp) box polypep	300208	X	114828731	114845972	17242	3385,85	0,46	1,63E-01
ENSRNOG00000016907	**Ppp5c**	protein phosphatase 5, catalytic subur	65179	1	80191565	80215807	24243	3579,53	0,46	1,63E-01
ENSRNOG00000017440	**Bgn**	biglycan (Bgn), mRNA [Source:RefSe	25181	1	153067994	153080160	12167	387,52	-0,92	1,63E-01
ENSRNOG00000050510	**LOC10036**	RAB1B, member RAS oncogene famil	81754	1	227413058	227413663	606	1162,16	0,52	1,63E-01
ENSRNOG00000004697	**Baalc**	brain and acute leukemia, cytoplasmic	140720	7	78416267	78489775	73509	654,06	-0,55	1,63E-01
ENSRNOG00000033096	**Macrod2**	Protein Macrod2 [Source:UniProtKB/	685695	3	142644462	142719566	75105	41,04	-0,92	1,63E-01
ENSRNOG00000013312	**Kcnt2**	potassium channel, subfamily T, meml	304827	13	62144528	62533203	388676	217,26	-0,79	1,63E-01
ENSRNOG00000014776	**Adcy7**	adenylate cyclase 7 (Adcy7), mRNA [	84420	19	30730189	30752411	22223	27,29	-0,98	1,63E-01
ENSRNOG00000000938	**Ran**	RAN, member RAS oncogene family (	84509	12	33247059	33250220	3162	8323,04	0,48	1,63E-01
ENSRNOG00000004612	**Ppp1cb**	protein phosphatase 1, catalytic subur	25594	6	33416541	33448276	31736	3911,75	-0,63	1,63E-01
ENSRNOG00000011287	**Minpp1**	multiple inositol-polyphosphate phospl	29688	1	258276252	258301852	25601	326,43	0,62	1,63E-01
ENSRNOG00000022980	**Sdhd**	succinate dehydrogenase complex, su	363061	8	53625447	53634926	9480	2167,44	0,40	1,63E-01
ENSRNOG00000016973	**RGD13072**	chromosome 1 open reading frame, hi	292228	1	47618088	47644800	26713	981,82	0,48	1,64E-01
ENSRNOG00000022429	**RGD13120**	similar to DD1 (RGD1312005), mRNA	291580	18	53346501	53361030	14530	345,23	0,76	1,64E-01
ENSRNOG00000032376	**LOC68876**	expressed sequence AI429214 [Sourc	688765	16	58418185	58419063	879	38,09	-0,77	1,64E-01
ENSRNOG00000010727	**Ube2r2**	similar to ubiquitin-conjugating enzym	689226	5	62002658	62061597	58940	2160,60	0,43	1,64E-01
ENSRNOG00000011033	**Tekt2**	tektin 2 (testicular) (Tekt2), mRNA [So	298532	5	148109214	148112751	3538	23,07	-0,91	1,64E-01
ENSRNOG00000018194	**Srrm1**	serine/arginine repetitive matrix 1 (Srr	313620	5	157444419	157476909	32491	3204,71	0,38	1,64E-01
ENSRNOG00000004534	**Spdya**	speedy/RINGO cell cycle regulator far	192209	6	33364981	33410446	45466	34,81	-0,91	1,64E-01
ENSRNOG00000013389	**Rgs7bp**	regulator of G-protein signaling 7 bindi	294715	2	54201509	54742295	540787	199,19	-0,58	1,64E-01
ENSRNOG00000007345	**Amot**	Protein Amot [Source:UniProtKB/TrEI	300289	X	116893710	116931649	37940	506,26	-0,54	1,65E-01
ENSRNOG00000007456	**Calb1**	calbindin 1 (Calb1), mRNA [Source:Re	83839	5	34217914	34242322	24409	539,70	-0,92	1,66E-01
ENSRNOG00000011292	**Col1a2**	collagen, type I, alpha 2 (Col1a2), mR	84352	4	31405253	31440179	34927	298,88	-0,96	1,66E-01
ENSRNOG00000005452		serine/threonine-protein phosphatase	117256	14	78644679	78672624	27946	1813,85	-0,65	1,66E-01

ENSRNOG00000019891	**Sgta**	small glutamine-rich tetratricopeptide r	64667	7	11689394	11705305	15912	4057,76	0,36	1,66E-01
ENSRNOG00000036841	**Cbx5**	chromobox homolog 5 (Cbx5), mRNA	300266	7	142605589	142623004	17416	815,84	-0,61	1,66E-01
ENSRNOG00000001360	**Stag3**	stromal antigen 3 (Stag3), mRNA [Sou	114522	12	21656555	21686862	30308	28,92	-0,93	1,67E-01
ENSRNOG00000001385	**Plbd2**	phospholipase B domain containing 2	246120	12	43452899	43472080	19182	2240,05	0,42	1,67E-01
ENSRNOG00000028717	**Ndufb7**	NADH dehydrogenase (ubiquinone) 1	361385	19	35680097	35684435	4339	1944,48	0,52	1,67E-01
ENSRNOG00000049585	**Tnc**	tenascin C (Tnc), mRNA [Source:RefS	116640	5	83891006	83950462	59457	5372,66	-0,94	1,67E-01
ENSRNOG00000005309		sodium channel protein type 8 subunit	29710	7	140395741	140485138	89398	1520,83	-0,55	1,67E-01
ENSRNOG00000008845	**Pdrg1**	p53 and DNA damage regulated 1 (Pd	296278	3	154890051	154896217	6167	1498,09	0,35	1,67E-01
ENSRNOG00000015079		dolichyl-diphosphooligosaccharide--pr	313648	5	160417822	160424369	6548	3056,97	0,41	1,68E-01
ENSRNOG00000006645		Protein Ryr3 [Source:UniProtKB/TrEM	170546	3	110710790	110851485	140696	354,96	-0,60	1,68E-01
ENSRNOG00000004827	**Papola**	poly (A) polymerase alpha (Papola), m	314417	6	138807510	138859620	52111	3039,02	-0,44	1,69E-01
ENSRNOG00000016244	**Mical2**	microtubule associated monooxygena	365352	1	184071797	184148676	76880	880,08	-0,58	1,69E-01
ENSRNOG00000009137	**Ubxn2b**	UBX domain protein 2B (Ubxn2b), mR	312965	5	24070790	24093957	23168	466,74	-0,46	1,69E-01
ENSRNOG00000015153	**Ints3**	Protein Ints3 [Source:UniProtKB/TrEM	361988	2	209198463	209248534	50072	2409,27	0,43	1,69E-01
ENSRNOG00000022845	**Cep70**	centrosomal protein 70 (Cep70), mRN	367153	8	106921077	106973347	52271	135,85	-0,75	1,69E-01
ENSRNOG00000002615	**Pmm2**	phosphomannomutase 2 (Pmm2), mR	302915	10	5857624	5878777	21154	919,97	0,51	1,70E-01
ENSRNOG00000002944	**RGD13045**	similar to RIKEN cDNA 2310033P09 (	303180	10	45315048	45317467	2420	373,04	0,45	1,70E-01
ENSRNOG00000008530	**Stoml1**	Protein Stoml1 [Source:UniProtKB/Tr	300748	8	62813283	62820361	7079	1142,67	0,51	1,70E-01
ENSRNOG00000016508	**Palmd**	palmdelphin (Palmd), mRNA [Source:I	310811	2	238543610	238596898	53289	153,89	-0,80	1,70E-01
ENSRNOG00000028521	**Plekhm1**	pleckstrin homology domain containin	303584	10	91219140	91267042	47903	1227,35	0,43	1,70E-01
ENSRNOG00000028733		cAMP-dependent protein kinase type I	25521	12	19611758	19708677	96920	9035,64	-0,53	1,70E-01
ENSRNOG00000043044		calponin 2 [Source:MGI Symbol;Acc:M	690976	7	12952278	12957474	5197	472,99	0,66	1,70E-01
ENSRNOG00000016001	**Chmp1a**	charged multivesicular body protein 1A	365024	19	66707177	66715457	8281	4747,53	0,42	1,70E-01
ENSRNOG00000015140	**Trmt13**	tRNA methyltransferase 13 homolog (S	499697	2	237588550	237601699	13150	99,22	-0,71	1,70E-01
ENSRNOG00000003504	**Rnaseh2a**	ribonuclease H2, subunit A (Rnaseh2a	364974	19	37050610	37060302	9693	1389,13	0,37	1,70E-01
ENSRNOG00000008703	**RGD15642**	ribonucleic acid binding protein S1 (Rr	287113	10	13603389	13613703	10315	4393,39	0,51	1,70E-01
ENSRNOG00000009255	**RGD13091**	uncharacterized protein LOC315463		315463	8	22679828	22682797	2970	2701,20	0,55	1,70E-01
ENSRNOG00000010841	**Col8a2**	Protein Col8a2 [Source:UniProtKB/Tr	313592	5	148097757	148100436	2680	29,97	-0,99	1,70E-01
ENSRNOG00000014252	**Irf2bp1**	interferon regulatory factor 2 binding p	308404	1	81171966	81174667	2702	2092,83	0,42	1,70E-01
ENSRNOG00000021866	**Bola3**	bolA family member 3 (Bola3), mRNA	297388	4	179636751	179646197	9447	596,02	0,45	1,70E-01
ENSRNOG00000013419	**Hykk**	hydroxylysine kinase (Hykk), mRNA [S	300723	8	58090149	58111525	21377	56,44	-0,84	1,70E-01
ENSRNOG00000020996		Protein Vps51 [Source:UniProtKB/TrE	1E+08	1	228359230	228375920	16691	2650,13	0,37	1,70E-01
ENSRNOG00000004137	**Ubtd2**	Protein Ubtd2 [Source:UniProtKB/TrE	287178	10	17267426	17282835	15410	341,77	-0,43	1,71E-01
ENSRNOG00000006102	**Pole4**	polymerase (DNA-directed), epsilon 4,	362385	4	178170534	178176345	5812	1198,03	-0,44	1,71E-01
ENSRNOG00000019214	**Rbx1**	ring-box 1, E3 ubiquitin protein ligase	300084	7	122677395	122686075	8681	2509,81	0,42	1,71E-01
ENSRNOG00000024917	**Als2cr12**	amyotrophic lateral sclerosis 2 (juvenil	316413	9	65472508	65500565	28058	18,23	-0,97	1,71E-01
ENSRNOG00000029238	**Fam203a**	family with sequence similarity 203, m	315094	7	117422049	117424835	2787	930,69	0,44	1,71E-01
ENSRNOG00000016977	**Calb2**	calbindin 2 (Calb2), mRNA [Source:Re	117059	19	52310641	52337700	27060	2921,13	-0,85	1,71E-01

ENSRNOG00000015914	**U2af2**	Protein U2af2 [Source:UniProtKB/TrE	308335	1	76206354	76223195	16842	6056,40	0,34	1,71E-01
ENSRNOG00000016827	**Slc38a3**	solute carrier family 38, member 3 (Slc	252919	8	115761371	115777467	16097	4015,70	-0,67	1,71E-01
ENSRNOG00000000702	**Sart3**	squamous cell carcinoma antigen reco	304582	12	50410338	50438125	27788	1606,97	0,49	1,71E-01
ENSRNOG00000007142	**B3gat1**	beta-1,3-glucuronyltransferase 1 (gluc	117108	8	27797386	27823230	25845	3581,23	-0,39	1,71E-01
ENSRNOG00000005480	**Ybx3**	Y box binding protein 3 (Ybx3), mRNA	83807	4	228170272	228193494	23223	1670,12	0,61	1,72E-01
ENSRNOG00000009513	**Akr1b1**	aldo-keto reductase family 1, member	24192	4	61426190	61440279	14090	2429,61	0,35	1,72E-01
ENSRNOG00000012404	**Thrsp**	thyroid hormone responsive (Thrsp), n	25357	1	168587726	168592099	4374	67,68	-0,97	1,72E-01
ENSRNOG00000050166		Uncharacterized protein [Source:UniProtKB/TrE		12	37852519	37859251	6733	233,98	-0,86	1,72E-01
ENSRNOG00000018533	**Iffo1**	intermediate filament family orphan 1 (	362437	4	224676331	224693515	17185	749,30	0,49	1,72E-01
ENSRNOG00000031036	**Mdfic**	MyoD family inhibitor domain containir	362325	4	41790495	41870159	79665	271,63	-0,70	1,72E-01
ENSRNOG00000019725	**Actr1a**	ARP1 actin-related protein 1 homolog	294010	1	273554811	273573479	18669	15809,24	0,53	1,73E-01
ENSRNOG00000037480	**Ddx51**	DEAD (Asp-Glu-Ala-Asp) box polypep	304570	12	53805410	53810335	4926	579,21	0,55	1,73E-01
ENSRNOG00000049873	**Ap1s3**	adaptor-related protein complex 1, sig	367304	9	85139124	85197998	58875	47,70	-0,71	1,73E-01
ENSRNOG00000018994	**Psmc4**	proteasome (prosome, macropain) 26	117262	1	86194912	86203260	8349	3426,68	0,55	1,75E-01
ENSRNOG00000001113	**Mmd2**	monocyte to macrophage differentiatic	304301	12	16049294	16105827	56534	4821,62	-0,86	1,75E-01
ENSRNOG00000014399	**Aamp**	angio-associated, migratory cell protei	301512	9	81327383	81333189	5807	6121,90	0,38	1,75E-01
ENSRNOG00000014616	**Iars**	isoleucyl-tRNA synthetase (Iars), mRN	306804	17	17414466	17461081	46616	2452,79	0,37	1,75E-01
ENSRNOG00000031591				18	56194141	56194647	507	906,05	0,41	1,75E-01
ENSRNOG00000020360	**Clcc1**	chloride channel CLIC-like 1 (Clcc1), r	170927	2	230923419	230952835	29417	595,95	-0,64	1,75E-01
ENSRNOG00000009492	**RGD13054**	similar to Nef associated protein 1 (RC	298072	5	66505324	66513879	8556	86,06	0,66	1,75E-01
ENSRNOG00000012558	**Orc2**	origin recognition complex, subunit 2 (	301430	9	65158929	65188753	29825	336,10	-0,64	1,75E-01
ENSRNOG00000024525	**Alkbh8**	alkB, alkylation repair homolog 8 (E. c	366783	6	28777894	28847493	69600	232,47	-0,54	1,75E-01
ENSRNOG00000032414	**Tmem161b**	Protein Tmem161b [Source:UniProtK	309953	2	12379283	12410781	31499	77,56	-0,72	1,75E-01
ENSRNOG00000004417	**Fam117a**	family with sequence similarity 117, m	497983	10	82964187	83008064	43878	79,07	-0,73	1,75E-01
ENSRNOG00000003336		myosin binding protein H (Mybph), mF	83708	13	56089936	56097582	7647	314,51	-0,50	1,76E-01
ENSRNOG00000007097	**Gpatch3**	Protein Gpatch3 [Source:UniProtKB/T	362615	5	155486208	155495158	8951	181,46	0,52	1,76E-01
ENSRNOG00000009480	**Slc24a3**	solute carrier family 24 (sodium/potass	85267	3	145760074	146259491	499418	2358,89	-0,53	1,76E-01
ENSRNOG00000034107	**LOC68128**	RIKEN cDNA 1110038F14 gene [Sour	681282	7	118007781	118009440	1660	449,84	0,48	1,76E-01
ENSRNOG00000010119	**Zmat3**	zinc finger, matrin type 3 (Zmat3), mR	64394	2	138382954	138411897	28944	2964,24	0,55	1,76E-01
ENSRNOG00000039740	**Cenpk**	centromere protein K (Cenpk), mRNA	294712	2	53441765	53464775	23011	78,93	-0,71	1,76E-01
ENSRNOG00000003493	**Prp2l1**	Protein Prp2l1 [Source:UniProtKB/TrE	287750	10	91440546	91451034	10489	214,85	-0,70	1,77E-01
ENSRNOG00000016043	**Aqp4**	aquaporin 4 (Aqp4), transcript variant	25293	18	6720759	6737507	16749	8357,11	-0,88	1,77E-01
ENSRNOG00000006368	**Lrrn3**	leucine rich repeat neuronal 3 (Lrrn3),	81514	6	70956995	70987862	30868	1520,07	-0,77	1,77E-01
ENSRNOG00000028103	**Psmd3**	proteasome (prosome, macropain) 26	287670	10	86395552	86407493	11942	4953,62	0,43	1,77E-01
ENSRNOG00000029095	**Trabd**	TraB domain containing (Trabd), mRN	300142	7	129744197	129755170	10974	1041,21	0,41	1,77E-01
ENSRNOG00000038372	**Ndufs2**	NADH dehydrogenase (ubiquinone) F	289218	13	94233721	94246253	12533	5538,43	0,46	1,77E-01
ENSRNOG00000009681		flotillin 2 (Flot2), transcript variant 1, m	83764	10	66303632	66327749	24118	2692,64	0,54	1,77E-01
ENSRNOG00000010616	**Ndor1**	NADPH dependent diflavin oxidoreduc	311799	3	2448121	2456341	8221	717,38	0,55	1,78E-01

ENSRNOG00000011027	**Uba5**	ubiquitin-like modifier activating enzym	300968	8	111967198	111981855	14658	1153,62	0,53	1,78E-01
ENSRNOG00000014079	**Stat1**	signal transducer and activator of tran:	25124	9	53998205	54038463	40259	2028,81	0,87	1,78E-01
ENSRNOG00000020368	**Fbxl12**	F-box and leucine-rich repeat protein 1	313782	8	21712700	21717320	4621	259,73	0,42	1,78E-01
ENSRNOG00000000073	**Tmed5**	transmembrane emp24 protein transp	289883	14	2612362	2623810	11449	301,35	-0,71	1,78E-01
ENSRNOG00000010397		Protein Agbl3 [Source:UniProtKB/TrE	500076	4	62032377	62107166	74790	35,26	-0,82	1,78E-01
ENSRNOG00000001719	**Psmd2**	proteasome (prosome, macropain) 26	287984	11	87006148	87016773	10626	9503,72	0,44	1,79E-01
ENSRNOG00000013323	**Rnf217**	ring finger protein 217 (Rnf217), mRN	292188	1	29757234	29846858	89625	527,66	-0,53	1,80E-01
ENSRNOG00000025999	**Pnma3**	paraneoplastic Ma antigen 3 (Pnma3),	293840	1	148793053	148794860	1808	108,41	-0,63	1,80E-01
ENSRNOG00000017905		microtubule-associated protein 1 light	64862	19	64367214	64383493	16280	6445,33	0,45	1,80E-01
ENSRNOG00000012348	**Exosc4**	exosome component 4 (Exosc4), mRN	300045	7	117377932	117380674	2743	628,22	0,41	1,80E-01
ENSRNOG00000015517	**Zfp444**	zinc finger protein 444 (Zfp444), mRN	292569	1	72775799	72793062	17264	1222,46	0,38	1,80E-01
ENSRNOG00000017994	**Nfatc2ip**	nuclear factor of activated T-cells, cytc	308983	1	204767892	204782663	14772	174,38	0,71	1,80E-01
ENSRNOG00000020391	**Nip7**	NIP7, nucleolar pre-rRNA processing	192180	19	50121523	50123631	2109	641,86	-0,41	1,80E-01
ENSRNOG00000021175	**Otub1**	OTU domain, ubiquitin aldehyde bindi	293705	1	229442242	229450521	8280	4347,50	0,50	1,80E-01
ENSRNOG00000045788		Uncharacterized protein [Source:UniProtKB/TrE		16	3946693	3954852	8160	81,56	-0,65	1,80E-01
ENSRNOG00000021899	**LOC10091**	transmembrane protein 115 (Tmem11	363136	8	115646345	115650714	4370	1036,16	0,41	1,80E-01
ENSRNOG00000017369	**Mustn1**	musculoskeletal, embryonic nuclear p	290553	16	6889473	6891725	2253	59,50	-0,78	1,80E-01
ENSRNOG00000011994	**Perp**	PERP, TP53 apoptosis effector (Perp)	292949	1	15786756	15799199	12444	30,15	-0,88	1,80E-01
ENSRNOG00000012356	**Slc36a1**	solute carrier family 36 (proton/amino	155205	10	40381908	40411783	29876	725,50	0,62	1,80E-01
ENSRNOG00000000645	**Reep3**	receptor accessory protein 3 (Reep3),	294375	20	25003934	25086732	82799	1812,95	-0,68	1,80E-01
ENSRNOG00000002106	**Usp46**	ubiquitin specific peptidase 46 (Usp46	289584	14	36514023	36582331	68309	2197,55	-0,38	1,80E-01
ENSRNOG00000004526	**Cox7a2l**	cytochrome c oxidase subunit VIIa pol	298762	6	6650357	6664094	13738	5224,38	0,48	1,80E-01
ENSRNOG00000006037		synaptosomal-associated protein 25 (	25012	3	136083976	136269421	185446	4685,24	-0,53	1,80E-01
ENSRNOG00000008557	**Abcb8**	ATP-binding cassette, subfamily B (MI	362302	4	7307908	7323157	15250	1506,53	0,39	1,80E-01
ENSRNOG00000008764	**Otud5**	OTU domain containing 5 (Otud5), mF	363452	X	16252448	16284933	32486	2661,72	-0,38	1,80E-01
ENSRNOG00000017548	**Fam53a**	family with sequence similarity 53, me	305461	14	83064832	83104049	39218	246,13	0,46	1,80E-01
ENSRNOG00000018588				17	39477612	39478533	922	2484,27	0,65	1,80E-01
ENSRNOG00000019266	**Tmem223**	transmembrane protein 223 (Tmem22	690285	1	231908980	231910341	1362	1617,05	0,77	1,80E-01
ENSRNOG00000024601	**Taf11**	TAF11 RNA polymerase II, TATA box b	303433	20	9678689	9684785	6097	694,12	0,44	1,80E-01
ENSRNOG00000030467	**Ube2l6**	ubiquitin-conjugating enzyme E2L 6 (U	295704	3	78712108	78727220	15113	313,59	0,95	1,80E-01
ENSRNOG00000033473	**Rpl36**	ribosomal protein L36 (Rpl36), mRNA	58927	9	9438155	9438769	615	44,66	0,81	1,80E-01
ENSRNOG00000037664	**Tbrg4**	transforming growth factor beta regula	360977	14	80510245	80519011	8767	1147,24	0,38	1,80E-01
ENSRNOG00000023271		presenilins-associated rhomboid-like p	287979	11	87581684	87607945	26262	852,60	0,46	1,81E-01
ENSRNOG00000031997		tubulin tyrosine ligase-like family, member 7 [Sou		2	271298255	271377377	79123	456,25	-0,64	1,81E-01
ENSRNOG00000049203	**LOC10091**	peroxisomal biogenesis factor 5 (Pex5	312703	4	224003342	224029468	26127	87,14	0,58	1,81E-01
ENSRNOG00000050381	**Dus3l**	dihydrouridine synthase 3-like (S. cere	301122	9	9286526	9291060	4535	750,11	0,42	1,81E-01
ENSRNOG00000003542	**Hcfc1r1**	host cell factor C1 regulator 1 (XPO1-	287097	10	12817362	12818424	1063	1700,85	0,50	1,81E-01
ENSRNOG00000003915	**Tmem206**	transmembrane protein 206 (Tmem20	305070	13	114572850	114597636	24787	1246,71	0,57	1,81E-01

ENSRNOG00000009409	**Fbxo2**	F-box protein 2 (Fbxo2), mRNA [Sourc	85273	5	168630521	168635950	5430	2075,04	-0,84	1,81E-01
ENSRNOG00000049471	**Steap3**	STEAP family member 3, metalloredu	170824	13	41363990	41380914	16925	92,16	-0,89	1,81E-01
ENSRNOG00000012810	**Rfesd**	Rieske (Fe-S) domain containing (Rfe:	361871	2	2767847	2777556	9710	142,36	-0,67	1,82E-01
ENSRNOG00000037566	**RGD15603**	Protein RGD1560398 [Source:UniPro	498192	12	49345461	49349115	3655	481,29	0,64	1,82E-01
ENSRNOG00000045829	**Thbs1**	thrombospondin 1 (Thbs1), mRNA [Source:RefS		3	116415715	116422101	6387	449,25	-0,49	1,82E-01
ENSRNOG00000046922		phosphatase and actin regulator 4 [So	682920	5	154203401	154248493	45093	409,01	0,41	1,82E-01
ENSRNOG00000013456	**Ighmbp2**	immunoglobulin mu binding protein 2 (	29532	1	225376771	225399917	23147	729,74	0,50	1,82E-01
ENSRNOG00000025209	**Plxnd1**	plexin D1 (Plxnd1), mRNA [Source:Re	312652	4	211138020	211177703	39684	1627,79	-0,72	1,82E-01
ENSRNOG00000005607	**Kctd5**	potassium channel tetramerization do	287109	10	13235963	13261942	25980	2669,64	0,45	1,83E-01
ENSRNOG00000007947	**Fam13a**	family with sequence similarity 13, me	362378	4	154006120	154102125	96006	95,84	-0,65	1,83E-01
ENSRNOG00000017372	**Higd2a**	HIG1 hypoxia inducible domain family,	290999	17	12684712	12685636	925	1576,17	0,53	1,83E-01
ENSRNOG00000039417	**Dda1**	DET1 and DDB1 associated 1 (Dda1),	682635	16	19739276	19746866	7591	2693,53	0,51	1,83E-01
ENSRNOG00000045863	**Naa16**	Protein LOC686240; RCG36843 [Sou	686240	15	65262054	65319826	57773	139,98	-0,68	1,83E-01
ENSRNOG00000005418	**Trap1**	TNF receptor-associated protein 1 (Tr	287069	10	10478499	10512148	33650	2384,96	0,49	1,83E-01
ENSRNOG00000022943		diacylglycerol kinase alpha [Source:R	140866	7	3116798	3142227	25430	139,23	-0,59	1,83E-01
ENSRNOG00000017051	**Tmem110**	transmembrane protein 110 (Tmem11	361110	16	6829242	6885526	56285	547,93	0,42	1,84E-01
ENSRNOG00000001456	**Nup153**	nucleoporin 153 (Nup153), mRNA [So	25281	17	19523818	19575973	52156	1896,75	-0,48	1,84E-01
ENSRNOG00000003343	**Canx**	calnexin (Canx), mRNA [Source:RefSe	29144	10	35562608	35595454	32847	10918,36	-0,72	1,84E-01
ENSRNOG00000009398	**Tceanc2**	transcription elongation factor A (SII) N	366431	5	130594909	130632086	37178	337,28	0,59	1,84E-01
ENSRNOG00000021463	**Ppara**	peroxisome proliferator activated rece	25747	7	126330833	126392748	61916	53,76	-0,74	1,84E-01
ENSRNOG00000013438	**LOC50002**	Yae1 domain containing 1 (Yae1d1), n	306994	17	47667539	47674056	6518	401,73	-0,88	1,84E-01
ENSRNOG00000011044	**Clmn**	calmin (Clmn), mRNA [Source:RefSeq	299285	6	137672546	137764455	91910	90,57	-0,64	1,84E-01
ENSRNOG00000015354	**Aox1**	aldehyde oxidase 1 (Aox1), mRNA [So	54349	9	64726637	64804786	78150	696,05	-0,96	1,84E-01
ENSRNOG00000018590	**Drg1**	developmentally regulated GTP bindin	305470	14	84148278	84164306	16029	1485,80	0,49	1,84E-01
ENSRNOG00000023274	**Lrrc28**	leucine rich repeat containing 28 (Lrrc	361588	1	129551867	129669841	117975	862,26	0,62	1,84E-01
ENSRNOG00000029855	**Letmd1**	LETM1 domain containing 1 (Letmd1)	681352	7	139913936	139923419	9484	1286,15	0,47	1,84E-01
ENSRNOG00000037835	**LOC69043**	predicted gene 216 [Source:MGI Sym	690437	9	81409927	81416667	6741	126,66	-0,87	1,84E-01
ENSRNOG00000006244	**Lztfl1**	leucine zipper transcription factor-like	316102	8	131928287	131943478	15192	287,57	-0,63	1,84E-01
ENSRNOG00000047537	**LOC10091**	Protein LOC100912373; RCG54864 [	1E+08	5	33163149	33182241	19093	25,81	-0,89	1,84E-01
ENSRNOG00000000708	**Sgsm1**	small G protein signaling modulator 1	288743	12	51103675	51169362	65688	1012,43	-0,49	1,85E-01
ENSRNOG00000003803	**Zdhhc17**	zinc finger, DHHC-type containing 17 (	366889	7	53664302	53726422	62121	676,44	-0,52	1,85E-01
ENSRNOG00000016177	**Scara3**	scavenger receptor class A, member 3	364405	15	48953960	48986864	32905	214,24	-0,79	1,85E-01
ENSRNOG00000018212	**Abhd17a**	abhydrolase domain containing 17A (A	299617	7	12160068	12166498	6431	3232,14	0,59	1,85E-01
ENSRNOG00000003661	**Hn1**	hematological and neurological expres	287828	10	104204896	104222931	18036	11089,20	0,49	1,85E-01
ENSRNOG00000008678	**Antxr1**	anthrax toxin receptor 1 (Antxr1), mRN	362393	4	183514052	183699805	185754	644,02	-0,51	1,85E-01
ENSRNOG00000042717	**RGD15663**	uncharacterized protein LOC365871 [	365871	2	217478839	217482049	3211	69,09	-0,79	1,85E-01
ENSRNOG00000009567	**Mrpl10**	mitochondrial ribosomal protein L10 (M	691075	10	84766862	84775278	8417	1527,89	0,43	1,85E-01
ENSRNOG00000010793	**Spryd3**	SPRY domain containing 3 (Spryd3), r	315327	7	141517639	141534847	17209	6203,69	0,60	1,85E-01

ENSRNOG00000028879	**Slc4a8**	solute carrier family 4, sodium bicarbo	315311	7	140201594	140275775	74182	464,48	-0,59	1,85E-01
ENSRNOG00000013484	**Gsta1**	glutathione S-transferase A3 (Gsta3),	24421	9	26283551	26300195	16645	517,82	-0,97	1,85E-01
ENSRNOG00000039024	**Spice1**	spindle and centriole associated prote	288111	11	65358818	65400932	42115	188,26	-0,45	1,86E-01
ENSRNOG00000008409	**Myo1f**	myosin IF (Myo1f), mRNA [Source:Re	314654	7	18618342	18669048	50707	19,24	-0,97	1,86E-01
ENSRNOG00000046912		Nuclear receptor subfamily 1 group D	259241	15	12792822	12816505	23684	302,19	-0,56	1,86E-01
ENSRNOG00000000175	**Mier2**	mesoderm induction early response 1,	362841	7	13203327	13219032	15706	999,37	0,39	1,86E-01
ENSRNOG00000008065		WW, C2 and coiled-coil domain contai	303039	10	20535873	20689272	153400	2913,68	-0,64	1,86E-01
ENSRNOG00000002209	**Arap2**	ArfGAP with RhoGAP domain, ankyrin	305367	14	48906157	49090667	184511	260,62	-0,95	1,86E-01
ENSRNOG00000005690	**Lmcd1**	LIM and cysteine-rich domains 1 (Lmc	494021	4	207494655	207554217	59563	129,29	-0,95	1,86E-01
ENSRNOG00000019939		G1/S-specific cyclin-D2 [Source:RefS	64033	4	231905237	231927558	22322	1321,75	-0,53	1,86E-01
ENSRNOG00000020281	**Kif22**	kinesin family member 22 (Kif22), mRNA [Sourc		1	205441133	205449441	8309	232,54	0,60	1,86E-01
ENSRNOG00000010280	**Pde8b**	phosphodiesterase 8B (Pde8b), mRN/	309962	2	43879335	43984030	104696	145,58	-0,68	1,87E-01
ENSRNOG00000017057		TBC1 domain family, member 22a [So	678774	7	126793081	127154298	361218	408,80	0,57	1,88E-01
ENSRNOG00000031979		ATP synthase F0 subunit 6 (mitochon	26197	MT	7919	8599	681	120429,61	-0,54	1,88E-01
ENSRNOG00000029470		Protein LOC685619 [Source:UniProtK	685619	3	168347846	168350355	2510	321,33	0,41	1,89E-01
ENSRNOG00000002523		epsin 2 (Epn2), transcript variant 2, ml	60443	10	47567991	47646897	78907	3440,59	-0,51	1,89E-01
ENSRNOG00000046803	**Arhgef16**	Rho guanine nucleotide exchange fac	687105	5	175098148	175120197	22050	20,62	-0,96	1,89E-01
ENSRNOG00000003134	**Slc4a4**	solute carrier family 4, sodium bicarbo	84484	14	20386111	20723823	337713	1793,04	-0,70	1,89E-01
ENSRNOG00000046479	**Chaf1a**	Protein Chaf1a [Source:UniProtKB/Tr	363333	9	9988293	10013841	25549	628,68	0,48	1,89E-01
ENSRNOG00000026336	**Tmem255b**	transmembrane protein 255B (Tmem2	290877	16	80692961	80697619	4659	180,76	-0,96	1,89E-01
ENSRNOG00000028261	**Tppp**	tubulin polymerization promoting prote	361466	1	33295461	33315340	19880	242,63	-0,59	1,90E-01
ENSRNOG00000007731	**LOC69199**	uncharacterized protein LOC691995		691995	10	57414744	57416295	1552	3360,96	0,74	1,90E-01
ENSRNOG00000014231	**Pnoc**	prepronociceptin (Pnoc), mRNA [Sour	25516	15	52547686	52574352	26667	157,16	0,64	1,90E-01
ENSRNOG00000021138	**Cers2**	ceramide synthase 2 (Cers2), mRNA [	310667	2	215986084	215994332	8249	1260,64	0,50	1,90E-01
ENSRNOG00000029805		Uncharacterized protein [Source:UniProtKB/TrE		1	85916136	85928618	12483	52,79	-0,70	1,90E-01
ENSRNOG00000031439	**RGD1563834**			19	55073108	55073544	437	23,49	0,83	1,90E-01
ENSRNOG00000048719		dysbindin [Source:RefSeq peptide;Ac	641528	17	22099335	22151097	51763	202,68	0,45	1,90E-01
ENSRNOG00000017969	**Olfm3**	olfactomedin 3 (Olfm3), mRNA [Sourc	252920	2	236054946	236097056	42111	461,09	-0,70	1,90E-01
ENSRNOG00000020551	**Bles03**	basophilic leukemia expressed protein	266609	1	227735894	227738143	2250	1742,13	0,38	1,90E-01
ENSRNOG00000008822	**Fkbp1a**	FK506 binding protein 1a (Fkbp1a), m	25639	3	153399455	153418856	19402	11807,59	0,41	1,91E-01
ENSRNOG00000007684	**Zc3h3**	zinc finger CCCH type containing 3 (Z	300032	7	116679040	116765271	86232	873,06	0,43	1,91E-01
ENSRNOG00000013621	**Rnd1**	Rho family GTPase 1 (Rnd1), mRNA [	362993	X	114852416	114859487	7072	918,67	-0,66	1,91E-01
ENSRNOG00000002592	**Rps6ka6**	ribosomal protein S6 kinase polypeptic	317203	X	82611372	82710712	99341	172,52	-0,67	1,91E-01
ENSRNOG00000006396	**Sdc1**	syndecan 1 (Sdc1), mRNA [Source:Re	25216	6	43667444	43689898	22455	753,11	0,51	1,91E-01
ENSRNOG00000048617	**Ensa**	endosulfine alpha (Ensa), transcript va	60334	2	217239506	217246826	7321	3495,23	0,47	1,91E-01
ENSRNOG00000033271		Cc2-36; Uncharacterized protein [Source:UniPr		5	74014076	74023281	9206	200,59	-0,93	1,92E-01
ENSRNOG00000042307		Protein Rybp; RING1 and YY1 binding	312603	4	197179661	197179912	252	167,18	-0,55	1,92E-01
ENSRNOG00000012601	**Leprotl1**	leptin receptor overlapping transcript-l	361160	16	61424726	61435440	10715	1304,80	0,43	1,92E-01

ENSRNOG00000000693		SV2 related protein [Source:MGI Symbol;Acc:M(		12	50074360	50132727	58368	3398,91	0,46	1,92E-01
ENSRNOG00000020715	**Ddb1**	damage-specific DNA binding protein	64470	1	233603576	233628839	25264	11715,12	0,48	1,92E-01
ENSRNOG00000013285	**Imp4**	IMP4, U3 small nucleolar ribonucleopr	316317	9	37456907	37462041	5135	2197,30	0,50	1,93E-01
ENSRNOG00000004783	**Fam171b**	Protein Fam171b [Source:UniProtKB/	499821	3	77735636	77787817	52182	1542,84	-0,76	1,93E-01
ENSRNOG00000008146	**Tox2**	TOX high mobility group box family me	311615	3	165765480	165890364	124885	403,70	0,48	1,93E-01
ENSRNOG00000012368	**Dnajc17**	DnaJ (Hsp40) homolog, subfamily C, r	311329	3	117530720	117564237	33518	237,76	0,51	1,93E-01
ENSRNOG00000017241	**Dpcd**	deleted in primary ciliary dyskinesia (Dpcd), mRI		1	272738456	272756533	18078	1344,75	0,57	1,93E-01
ENSRNOG00000019306	**Syt12**	synaptotagmin XII (Syt12), mRNA [So	191595	1	226557953	226587017	29065	95,62	-0,56	1,93E-01
ENSRNOG00000042355	**Tarbp2**	TAR (HIV-1) RNA binding protein 2 (Ta	363006	7	141913739	141918852	5114	1447,96	0,62	1,93E-01
ENSRNOG00000047712	**Dcx**	doublecortin (Dcx), mRNA [Source:Re	84394	X	113551874	113628192	76319	17062,12	-0,76	1,93E-01
ENSRNOG00000050044		Protein tyrosine phosphatase type IVA	85237	5	151775119	151784416	9298	2602,67	-0,33	1,93E-01
ENSRNOG00000050846		Mitochondrial import inner membrane	84384	1	177477126	177478411	1286	507,99	0,44	1,93E-01
ENSRNOG00000002052	**Ccdc80**	coiled-coil domain containing 80 (Ccd	64387	11	64747816	64781404	33589	539,01	-0,41	1,94E-01
ENSRNOG00000005736	**Eif1ax**	eukaryotic translation initiation factor 1	302697	X	37884994	37900391	15398	3389,08	-0,52	1,94E-01
ENSRNOG00000006467	**Eif2b2**	eukaryotic translation initiation factor 2	84005	6	116505786	116512219	6434	1931,63	0,49	1,94E-01
ENSRNOG00000010799	**Ccrn4l**	CCR4 carbon catabolite repression 4-l	310395	2	159759666	159779874	20209	554,02	-0,42	1,94E-01
ENSRNOG00000012579	**Tlcd1**	TLC domain containing 1 (Tlcd1), mRI	287472	10	66193481	66195513	2033	258,31	-0,85	1,94E-01
ENSRNOG00000013002	**Gpbp1**	GC-rich promoter binding protein 1 (G	294734	2	62047278	62114558	67281	1306,82	-0,68	1,94E-01
ENSRNOG00000013965	**Noc3l**	nucleolar complex associated 3 homol	361753	1	264962014	264990545	28532	153,58	-0,69	1,94E-01
ENSRNOG00000016660	**Cox5b**	cytochrome c oxidase subunit Vb (Co)	94194	9	42912193	42914547	2355	3595,70	0,44	1,94E-01
ENSRNOG00000020778	**Chrnb2**	cholinergic receptor, nicotinic, beta 2 (	54239	2	208511421	208519636	8216	1416,34	0,65	1,94E-01
ENSRNOG00000029658		ring finger protein 213 [Source:MGI Sy	303735	10	108134733	108229040	94308	793,05	0,79	1,94E-01
ENSRNOG00000032773				3	87249550	87250818	1269	163,89	0,70	1,94E-01
ENSRNOG00000049149		MACRO domain containing 2 [Source:MGI Syml		3	142006787	142284349	277563	20,53	-0,88	1,94E-01
ENSRNOG00000050563		Activated RNA polymerase II transcrip	192269	2	82656091	82670880	14790	4053,10	-0,58	1,94E-01
ENSRNOG00000010029	**Ubald2**	Protein Fam100b [Source:UniProtKB/	287840	10	105054510	105058301	3792	1162,11	0,58	1,95E-01
ENSRNOG00000014871		Zic family member 4 (Zic4), mRNA [So	315882	8	98243374	98252520	9147	43,24	-0,75	1,95E-01
ENSRNOG00000020379	**Cog8**	component of oligomeric golgi comple	291990	19	50110028	50121317	11290	1285,30	0,44	1,95E-01
ENSRNOG00000001842	**Mrps35**	mitochondrial ribosomal protein S35 (I	297727	4	245584690	245651496	66807	725,11	0,46	1,95E-01
ENSRNOG00000003313	**LOC10091(**	glycine receptor, alpha 2 (Glra2), mRN	24397	X	30995485	31214065	218581	211,48	-0,63	1,95E-01
ENSRNOG00000004232		G2/M phase-specific E3 ubiquitin-prot	299002	6	81659540	81685196	25657	257,96	-0,72	1,95E-01
ENSRNOG00000005689	**Yeats4**	YEATS domain containing 4 (Yeats4),	299810	7	60289460	60296600	7141	1180,79	0,48	1,95E-01
ENSRNOG00000048891		proline rich 14-like [Source:MGI Symb	305466	14	83923291	83976596	53306	809,97	0,42	1,95E-01
ENSRNOG00000002579	**Parm1**	prostate androgen-regulated mucin-lik	286894	14	17888361	17918277	29917	198,78	-0,80	1,95E-01
ENSRNOG00000008450	**LOC10035!**	ribonucleotide reductase M2 (Rrm2), r	1E+08	19	36367878	36370407	2530	122,18	0,64	1,95E-01
ENSRNOG00000011611	**Mapk1ip1l**	mitogen-activated protein kinase 1 inte	361028	15	28019022	28042563	23542	1848,45	0,38	1,95E-01
ENSRNOG00000011203	**Farp1**	FERM, RhoGEF (Arhgef) and pleckstr	306183	15	109829728	110020013	190286	4675,14	-0,39	1,96E-01
ENSRNOG00000016294	**Cd4**	Cd4 molecule (Cd4), mRNA [Source:F	24932	4	224400830	224425954	25125	22,20	-0,94	1,96E-01

ENSRNOG00000003563	**LOC10091**	bleomycin hydrolase (Blmh), mRNA [S	287552	10	62896489	62939897	43409	1422,70	0,41	1,96E-01
ENSRNOG00000004971	**Txlng**	taxilin gamma (Txlng), mRNA [Source	302680	X	33946696	33984036	37341	90,43	-0,60	1,96E-01
ENSRNOG00000027962	**Smg7**	SMG7 nonsense mediated mRNA dec	360855	13	75229500	75270238	40739	2485,65	-0,38	1,96E-01
ENSRNOG00000009871	**Piwil2**	piwi-like RNA-mediated gene silencing	306011	15	55760795	55839495	78701	41,94	-0,76	1,97E-01
ENSRNOG00000015529	**Cdca3**	cell division cycle associated 3 (Cdca3	297594	4	224365696	224369280	3585	551,18	0,59	1,97E-01
ENSRNOG00000019721	**Ypel3**	yippee-like 3 (Drosophila) (Ypel3), mR	293491	1	205190393	205193684	3292	2587,07	0,48	1,97E-01
ENSRNOG00000003235	**Mgat4b**	mannosyl (alpha-1,3-)-glycoprotein be	303100	10	35487146	35496940	9795	3467,44	0,39	1,97E-01
ENSRNOG00000017149	**Fam131b**	family with sequence similarity 131, m	500102	4	136508270	136517460	9191	2481,21	-0,56	1,97E-01
ENSRNOG00000045740		thioredoxin-related transmembrane pr	682967	18	86613012	86642612	29601	423,55	-0,72	1,97E-01
ENSRNOG00000014743	**Hagh**	hydroxyacyl glutathione hydrolase (Ha	24439	10	14032159	14046519	14361	811,87	0,51	1,97E-01
ENSRNOG00000021220	**Cpxm1**	carboxypeptidase X (M14 family), mer	296156	3	129414247	129421045	6799	645,04	-0,88	1,97E-01
ENSRNOG00000008257	**Mfap2**	microfibrillar-associated protein 2 (Mfa	313662	5	163246454	163252003	5550	585,97	0,53	1,97E-01
ENSRNOG00000012007	**Slc38a7**	solute carrier family 38, member 7 (Slc	291840	19	9607720	9621918	14199	995,12	0,53	1,98E-01
ENSRNOG00000026861		zinc finger and SCAN domain containi	499083	1	66661623	66668933	7311	426,88	-0,43	2,00E-01
ENSRNOG00000004466	**Fam210b**	family with sequence similarity 210, m	296408	3	176429999	176438295	8297	305,23	-0,48	2,00E-01
ENSRNOG00000005828	**Skp1**	S-phase kinase-associated protein 1 (	287280	10	37373099	37383454	10356	12295,16	-0,43	2,00E-01
ENSRNOG00000017837		zinc finger protein 553 [Source:MGI Symbol;Acc		1	205673587	205677062	3476	771,54	0,49	2,00E-01
ENSRNOG00000030238		fibronectin type III domain containing 5	260327	5	151057693	151062818	5126	282,98	-0,82	2,00E-01
ENSRNOG00000012357	**Unc45a**	unc-45 homolog A (C. elegans) (Unc45	308759	1	143071176	143086028	14853	2084,89	0,51	2,01E-01
ENSRNOG00000019634	**Eif4e2**	eukaryotic translation initiation factor 4	363275	9	94034066	94050247	16182	1303,06	0,40	2,01E-01
ENSRNOG00000026364	**Tanc2**	tetratricopeptide repeat, ankyrin repea	303599	10	93567079	93880565	313487	3499,75	-0,37	2,01E-01
ENSRNOG00000009340	**Zbtb6**	zinc finger and BTB domain containing	366029	3	26919902	26923316	3415	665,68	-0,55	2,01E-01
ENSRNOG00000027016	**Cobll1**	Protein Cobll1 [Source:UniProtKB/TrE	311088	3	57773840	57848396	74557	224,03	-0,73	2,01E-01
ENSRNOG00000001963	**Mx2**	myxovirus (influenza virus) resistance	286918	11	41546197	41570078	23882	1403,54	0,90	2,01E-01
ENSRNOG00000002863	**Cacna1e**	calcium channel, voltage-dependent, F	54234	13	76850596	77306502	455907	1228,99	-0,58	2,01E-01
ENSRNOG00000019798	**Stub1**	STIP1 homology and U-box containing	287155	10	15010698	15012978	2281	1826,68	0,44	2,01E-01
ENSRNOG00000006991	**Adrm1**	adhesion regulating molecule 1 (Adrm	65138	3	181565742	181570520	4779	3541,74	0,56	2,02E-01
ENSRNOG00000014758	**Lgi1**	leucine-rich, glioma inactivated 1 (Lgi1	252892	1	264436334	264477681	41348	525,22	-0,47	2,02E-01
ENSRNOG00000020246	**Myl9**	myosin, light chain 9, regulatory (Myl9	296313	3	158832629	158838991	6363	608,29	0,59	2,02E-01
ENSRNOG00000001457	**Rfc2**	replication factor C (activator 1) 2 (Rfc	116468	12	27130771	27143874	13104	696,84	0,50	2,02E-01
ENSRNOG00000009309	**Meaf6**	MYST/Esa1-associated factor 6 (Meaf	362594	5	146848980	146870851	21872	1379,00	0,51	2,02E-01
ENSRNOG00000011352	**Furin**	furin (paired basic amino acid cleaving	54281	1	143137723	143150202	12480	1728,64	0,47	2,02E-01
ENSRNOG00000016410	**Prelid1**	PRELI domain containing 1 (Prelid1),	290995	17	11943926	11946985	3060	4053,48	0,52	2,02E-01
ENSRNOG00000020723	**Pten**	phosphatase and tensin homolog (Pte	50557	1	258651829	258717009	65181	1335,40	-0,41	2,02E-01
ENSRNOG00000001603	**N6amt1**	N-6 adenine-specific DNA methyltrans	288309	11	30629574	30641919	12346	300,54	0,47	2,02E-01
ENSRNOG00000014055		N-acetyltransferase 1 (Nat1), transcrip	116631	16	23854943	23875351	20409	46,23	-0,87	2,02E-01
ENSRNOG00000015148		ELKS/RAB6-interacting/CAST family r	259269	16	3260477	3645293	384817	652,37	-0,60	2,02E-01
ENSRNOG00000032574	**Scrib**	scribbled planar cell polarity protein (S	362938	7	117091481	117114469	22989	2642,65	0,40	2,02E-01

ENSRNOG00000032878		CXXC-type zinc finger protein 5 [Sour	291670	18	28365065	28368322	3258	620,10	-0,65	2,02E-01
ENSRNOG00000037687	**Rspo2**	R-spondin 2 (Rspo2), mRNA [Source:I	500863	7	81992732	82077521	84790	120,32	-0,75	2,03E-01
ENSRNOG00000013817	**Wnt8b**	wingless-type MMTV integration site fa	293990	1	271703670	271707467	3798	29,89	-0,94	2,03E-01
ENSRNOG00000042022	**H1f0**	H1 histone family, member 0 (H1f0), m	24437	7	120252161	120254014	1854	3585,24	0,49	2,03E-01
ENSRNOG00000012397	**Zmym4**	zinc finger, MYM-type 4 (Zmym4), mR	313598	5	148648498	148766683	118186	2733,01	-0,32	2,04E-01
ENSRNOG00000024212	**Papd5**	PAP associated domain containing 5 (	307745	19	30800163	30857233	57071	1221,13	-0,43	2,04E-01
ENSRNOG00000000875		four and a half LIM domains 1 (Fhl1), t	25177	X	153788934	153803150	14217	4791,03	-0,47	2,04E-01
ENSRNOG00000021507	**LOC10091(**	Protein LOC100910839 [Source:UniP	1E+08	1	114425908	114534783	108876	183,91	-0,80	2,04E-01
ENSRNOG00000007312	**Arl8b**	ADP-ribosylation factor-like 8B (Arl8b)	500282	4	217450494	217494706	44213	5598,00	-0,39	2,04E-01
ENSRNOG00000011521	**Filip1**	filamin A interacting protein 1 (Filip1), i	246776	8	86812192	86958261	146070	100,98	-0,90	2,04E-01
ENSRNOG00000016603	**Rtn2**	reticulon 2 (Rtn2), mRNA [Source:Ref!	308410	1	81461962	81474845	12884	4794,41	0,55	2,04E-01
ENSRNOG00000017840	**Pik3c3**	phosphatidylinositol 3-kinase, catalytic	65052	18	22694942	22778627	83686	868,49	0,43	2,04E-01
ENSRNOG00000020656	**Pfkfb4**	6-phosphofructo-2-kinase/fructose-2,6	54283	8	117088862	117126898	38037	113,30	0,53	2,04E-01
ENSRNOG00000020906	**Pola2**	polymerase (DNA directed), alpha 2, a	85242	1	228231867	228255908	24042	293,29	0,43	2,04E-01
ENSRNOG00000020950	**LOC10091(**	synovial apoptosis inhibitor 1, synovio	361712	1	228345143	228351660	6518	1722,26	0,46	2,04E-01
ENSRNOG00000030719	**Csmd1**	CUB and Sushi multiple domains 1 (C	364634	16	78123917	78434380	310464	1222,73	-0,51	2,04E-01
ENSRNOG00000040281	**Zc3h10**	zinc finger CCCH type containing 10 (.	685928	7	2942063	2946149	4087	491,04	0,48	2,04E-01
ENSRNOG00000020938	**Ppp1r15a**	protein phosphatase 1, regulatory sub	171071	1	102590679	102593734	3056	901,40	0,52	2,04E-01
ENSRNOG00000046500	**Irgq**	immunity-related GTPase family, Q (Ir	292708	1	82656242	82664190	7949	8504,04	0,64	2,05E-01
ENSRNOG00000015791	**Zdhhc12**	zinc finger, DHHC-type containing 12 (	366014	3	14008557	14011260	2704	340,73	0,43	2,05E-01
ENSRNOG00000021705	**LOC10091'**	Protein LOC100911699 [Source:UniP	1E+08	12	39058751	39059536	786	50,08	-0,69	2,05E-01
ENSRNOG00000004518	**Cacnb1**	calcium channel, voltage-dependent, b	50688	10	85742631	85763137	20507	2239,20	0,49	2,05E-01
ENSRNOG00000006096	**Slc26a7**	solute carrier family 26 (anion exchan	297910	5	32679000	32816595	137596	31,24	-0,90	2,05E-01
ENSRNOG00000007177	**Kdelc2**	KDEL (Lys-Asp-Glu-Leu) containing 2	315664	8	56547250	56564862	17613	734,49	-0,69	2,05E-01
ENSRNOG00000001959	**Mx1**	myxovirus (influenza virus) resistance	24575	11	41398738	41420543	21806	57,72	0,91	2,05E-01
ENSRNOG00000014448		Aryl hydrocarbon receptor nuclear translocator-li		1	185007568	185105409	97842	939,95	-0,41	2,05E-01
ENSRNOG00000004068	**Ncoa1**	nuclear receptor coactivator 1 (Ncoa1)	313929	6	38483478	38734322	250845	3631,93	-0,35	2,05E-01
ENSRNOG00000004099	**R3hdm1**	R3H domain containing 1 (R3hdm1), r	304763	13	49897739	50035934	138196	3581,38	-0,32	2,05E-01
ENSRNOG00000007090	**Cacna1c**	calcium channel, voltage-dependent, L	24239	4	216566486	217184742	618257	1052,51	-0,47	2,05E-01
ENSRNOG00000008169		Sodium/potassium/calcium exchanger	84550	5	109317349	109564102	246754	508,23	-0,50	2,05E-01
ENSRNOG00000012406	**Pcbp4**	poly(rC) binding protein 4 (Pcbp4), mF	363133	8	114519757	114529993	10237	7675,34	0,58	2,05E-01
ENSRNOG00000012954	**Eefsec**	eukaryotic elongation factor, selenocy	500255	4	185435789	185630486	194698	822,65	0,43	2,05E-01
ENSRNOG00000013085	**Nkain3**	Na+/K+ transporting ATPase interactir	689576	5	38786929	39151521	364593	125,76	-0,59	2,05E-01
ENSRNOG00000017608	**C2cd3**	C2 calcium-dependent domain contair	293148	1	171583397	171680309	96913	1180,10	0,53	2,05E-01
ENSRNOG00000020204	**Srp19**	signal recognition particle 19 (Srp19),	291685	18	26828311	26834643	6333	587,74	-0,50	2,05E-01
ENSRNOG00000021185	**Bola1**	bolA family member 1 (Bola1), mRNA	365875	2	217825097	217826113	1017	391,90	0,67	2,05E-01
ENSRNOG00000033299		ATP synthase F0 subunit 8 (mitochon	26196	MT	7758	7961	204	32688,08	-0,58	2,05E-01
ENSRNOG00000047246	**Chtf8**	CTF8, chromosome transmission fidelity factor 8		19	49941868	49952168	10301	3996,80	0,38	2,05E-01

ENSRNOG00000000513	**Mapk14**	mitogen activated protein kinase 14 (M	81649	20	7968539	8028708	60170	1855,88	0,57	2,05E-01
ENSRNOG00000019105	**Dpp8**	dipeptidylpeptidase 8 (Dpp8), mRNA [	315758	8	70219642	70273860	54219	6344,32	-0,41	2,05E-01
ENSRNOG00000019184	**Npr3**	natriuretic peptide receptor C/guanylat	25339	2	82741077	82801473	60397	219,61	-0,80	2,05E-01
ENSRNOG00000021007	**Snx15**	sorting nexin 15 (Snx15), mRNA [Sour	293691	1	228421392	228430527	9136	879,04	0,45	2,05E-01
ENSRNOG00000023423	**Aim1l**	Protein Aim1l [Source:UniProtKB/TrEl	298543	5	155987651	156007400	19750	24,47	-0,77	2,05E-01
ENSRNOG00000008414		basigin (Ok blood group) (Bsg), transc	25246	7	13044573	13051789	7217	14768,50	0,52	2,05E-01
ENSRNOG00000004666	**Prr5l**	proline rich 5 like (Prr5l), mRNA [Sour	362171	3	97950825	98029048	78224	48,19	-0,72	2,06E-01
ENSRNOG00000013290	**Nrip3**	nuclear receptor interacting protein 3 (	361625	1	181370497	181396068	25572	1829,65	-0,54	2,06E-01
ENSRNOG00000005397	**Maea**	macrophage erythroblast attacher (Ma	298982	14	83349565	83383727	34163	2944,65	0,48	2,06E-01
ENSRNOG00000000803		protein phosphatase 1, regulatory subunit 10 [Sc		20	5428220	5441580	13361	1474,87	0,35	2,06E-01
ENSRNOG00000002456	**LOC69028**	hepatic leukemia factor [Source:MGI S	690286	10	77701829	77777898	76070	49,72	-0,66	2,06E-01
ENSRNOG00000021028	**Atg2a**	autophagy related 2A (Atg2a), mRNA	689688	1	228549959	228617368	67410	1213,35	0,38	2,06E-01
ENSRNOG00000019802	**Zfp428**	zinc finger protein 428 (Zfp428), mRN	361519	1	82633765	82642244	8480	2118,28	0,60	2,06E-01
ENSRNOG00000020474	**Pin1**	peptidylprolyl cis/trans isomerase, NIM	298696	8	21725297	21736676	11380	4177,29	0,59	2,06E-01
ENSRNOG00000020847	**Elp6**	elongator acetyltransferase complex s	363150	8	117869570	117884873	15304	801,65	0,45	2,06E-01
ENSRNOG00000017020	**Inpp5d**	inositol polyphosphate-5-phosphatase	54259	9	94464377	94569982	105606	29,54	-0,91	2,07E-01
ENSRNOG00000025324	**Spire1**	spire homolog 1 (Drosophila) (Spire1),	307348	18	62411829	62542260	130432	3906,16	-0,38	2,08E-01
ENSRNOG00000001293	**Get4**	golgi to ER traffic protein 4 homolog (S	288518	12	19455694	19472738	17045	2264,09	0,36	2,08E-01
ENSRNOG00000009779	**Krt8**	keratin 8 (Krt8), mRNA [Source:RefSe	25626	7	141393012	141400521	7510	17,75	-0,94	2,08E-01
ENSRNOG00000014154	**Ilf2**	interleukin enhancer binding factor 2 (	310612	2	209288460	209308315	19856	3755,21	0,61	2,09E-01
ENSRNOG00000001730	**Acap2**	ArfGAP with coiled-coil, ankyrin repea	619382	11	76151876	76262551	110676	1276,39	-0,50	2,09E-01
ENSRNOG00000002553	**Smim14**	small integral membrane protein 14 (S	364154	14	44233124	44276792	43669	2776,88	0,60	2,09E-01
ENSRNOG00000023492		growth regulation by estrogen in breas	498819	18	1710684	1818209	107526	31,16	-0,74	2,09E-01
ENSRNOG00000015084	**Necab2**	N-terminal EF-hand calcium binding p	170928	19	62834865	62861089	26225	1124,70	-0,60	2,09E-01
ENSRNOG00000019697	**Ankrd28**	Protein Ankrd28 [Source:UniProtKB/T	306264	16	7740393	7820041	79649	934,95	-0,51	2,09E-01
ENSRNOG00000009078	**Mrpl37**	mitochondrial ribosomal protein L37 (M	56281	5	130507127	130517640	10514	1640,52	0,40	2,09E-01
ENSRNOG00000012818	**Ksr1**	kinase suppressor of ras 1 (Ksr1), mR	360573	10	65166925	65300246	133322	270,43	-0,44	2,10E-01
ENSRNOG00000018122	**Tspan17**	tetraspanin 17 (Tspan17), mRNA [Sou	306771	17	12482948	12490502	7555	1514,09	-0,84	2,10E-01
ENSRNOG00000000522	**Cpne5**	copine V (Cpne5), mRNA [Source:Ref	309650	20	8664244	8745116	80873	739,51	-0,73	2,10E-01
ENSRNOG00000021886	**B4galt7**	xylosylprotein beta 1,4-galactosyltrans	364675	17	11657452	11666049	8598	1136,99	0,43	2,10E-01
ENSRNOG00000008712	**RGD13045**	uncharacterized protein LOC298104	298104	5	83497715	83521432	23718	191,34	-0,76	2,10E-01
ENSRNOG00000016930	**LOC68574**	ubiquitin-conjugating enzyme E2S (Ub	292588	1	75951968	75956026	4059	4617,34	0,59	2,10E-01
ENSRNOG00000048242	**Txlna**	taxilin alpha (Txlna), mRNA [Source:R	682457	5	151561492	151575118	13627	1464,71	0,58	2,10E-01
ENSRNOG00000021938	**Lrrtm4**	leucine rich repeat transmembrane ne	500219	4	175391879	175393435	1557	265,79	-0,57	2,10E-01
ENSRNOG00000015554		Protein Ankdd1a [Source:UniProtKB/	1E+08	8	70722175	70742752	20578	40,77	-0,75	2,11E-01
ENSRNOG00000009715	**Me1**	malic enzyme 1, NADP(+)-dependent,	24552	8	93769078	93863618	94541	3211,22	-0,57	2,11E-01
ENSRNOG00000027992		Down syndrome cell adhesion molecu	171119	11	40530632	40775978	245347	1194,30	-0,45	2,11E-01
ENSRNOG00000012424	**Adam23**	ADAM metallopeptidase domain 23 (A	301460	9	72283127	72437355	154229	1212,01	-0,55	2,11E-01

ENSRNOG00000000414	**LOC10036**	centrosomal protein 85-like [Source:M	1E+08	20	36333903	36441228	107326	198,12	-0,56	2,11E-01
ENSRNOG00000004793	**Ncln**	nicalin (Ncln), mRNA [Source:RefSeq	314648	7	11248842	11258770	9929	3565,73	0,42	2,11E-01
ENSRNOG00000014226		phospholipase C, eta 2 [Source:MGI S	313756	5	175842863	175871527	28665	735,47	-0,50	2,11E-01
ENSRNOG00000003864	**Gipc1**	GIPC PDZ domain containing family, r	83823	19	35765788	35777379	11592	3033,92	0,60	2,12E-01
ENSRNOG00000012280	**Ptx3**	pentraxin 3, long (Ptx3), mRNA [Sourc	689388	2	177457263	177463073	5811	398,56	-0,74	2,12E-01
ENSRNOG00000038330	**RGD13595**	similar to protein C33A12.3 (RGD135	361941	2	148271821	148296647	24827	45,93	-0,71	2,12E-01
ENSRNOG00000002983	**Nfix**	nuclear factor I/X (CCAAT-binding trar	81524	19	36797568	36890319	92752	3182,76	-0,49	2,12E-01
ENSRNOG00000009712	**Gale**	UDP-galactose-4-epimerase (Gale), m	114860	5	158075304	158079806	4503	360,77	0,58	2,12E-01
ENSRNOG00000015479	**Mrps34**	mitochondrial ribosomal protein S34 (I	287126	10	14073017	14074148	1132	1370,52	0,64	2,12E-01
ENSRNOG00000021794	**Znhit2**	zinc finger, HIT-type containing 2 (Znh	309177	1	228359846	228361096	1251	525,06	0,55	2,12E-01
ENSRNOG00000024707	**Tp73**	tumor protein p73 (Tp73), mRNA [Sou	362675	5	174843033	174901875	58843	75,65	-0,88	2,12E-01
ENSRNOG00000027954	**Cdh15**	cadherin 15 (Cdh15), mRNA [Source:I	361432	19	66375691	66396016	20326	47,72	-0,72	2,12E-01
ENSRNOG00000018310	**Eno4**	enolase family member 4 (Eno4), mRI	292138	1	287467133	287490511	23379	43,07	-0,69	2,13E-01
ENSRNOG00000032656	**Ptprt**	protein tyrosine phosphatase, recepto	362263	3	163763405	164548723	785319	1056,42	-0,64	2,13E-01
ENSRNOG00000008644	**Nkx2-1**	NK2 homeobox 1 (Nkx2-1), mRNA [Sc	25628	6	86945224	86948414	3191	261,03	0,58	2,13E-01
ENSRNOG00000010154	**Zc3hc1**	zinc finger, C3HC-type containing 1 (Z	296957	4	57445868	57468238	22371	1342,69	0,52	2,13E-01
ENSRNOG00000016631	**Zfp276**	Protein Zfp276 [Source:UniProtKB/Tr	307924	19	66759591	66772638	13048	276,25	0,45	2,13E-01
ENSRNOG00000010169	**Atpaf1**	ATP synthase mitochondrial F1 compl	313510	5	138426066	138451527	25462	516,52	0,46	2,13E-01
ENSRNOG00000016055	**Fkrp**	fukutin related protein (Fkrp), mRNA [	308390	1	79980703	79986302	5600	1026,03	0,55	2,13E-01
ENSRNOG00000022725	**LOC10091**	IBA57, iron-sulfur cluster assembly ho	363611	10	45262185	45270970	8786	199,36	0,49	2,13E-01
ENSRNOG00000007006	**Slc3a1**	solute carrier family 3, member 1 (Slc	29484	6	8217523	8251124	33602	686,75	-0,44	2,13E-01
ENSRNOG00000042283	**LOC10035**	DNA segment, Chr 3, ERATO Doi 254	1E+08	2	142490960	142495476	4517	260,94	-0,59	2,13E-01
ENSRNOG00000002176	**Pvrl3**	poliovirus receptor-related 3 (Pvrl3), m	288124	11	62049050	62144719	95670	706,22	-0,45	2,14E-01
ENSRNOG00000003720	**Prrx1**	paired related homeobox 1 (Prrx1), m	266813	13	86039713	86107193	67481	322,21	-0,73	2,14E-01
ENSRNOG00000004890	**Adcy8**	adenylate cyclase 8 (brain) (Adcy8), m	29241	7	105301842	105540451	238610	348,60	-0,57	2,14E-01
ENSRNOG00000008641	**Gnpnat1**	glucosamine-phosphate N-acetyltrans	498486	15	23685449	23697742	12294	227,15	-0,57	2,14E-01
ENSRNOG00000012216	**Tgfbi**	transforming growth factor, beta induc	116487	17	10576349	10605606	29258	18,17	-0,90	2,14E-01
ENSRNOG00000012812	**Sharpin**	SHANK-associated RH domain intera	81859	7	117400782	117404993	4212	926,24	0,35	2,14E-01
ENSRNOG00000016723	**Phpt1**	phosphohistidine phosphatase 1 (Php	296571	3	2778458	2779837	1380	2544,49	0,40	2,14E-01
ENSRNOG00000018502		vacuolar fusion protein MON1 homolo	315999	8	116011554	116036515	24962	710,20	0,50	2,14E-01
ENSRNOG00000020689	**Cpeb3**	Protein Cpeb3 [Source:UniProtKB/Tr	309510	1	263170252	263287406	117155	375,41	0,62	2,14E-01
ENSRNOG00000020936	**Nradd**	neurotrophin receptor associated deat	246143	8	118231775	118234748	2974	77,17	0,70	2,14E-01
ENSRNOG00000026914		Dynein heavy chain 1, axonemal [Source:UniPr		16	7274368	7337315	62948	62,89	-0,87	2,14E-01
ENSRNOG00000027959	**Ppp1r12c**	protein phosphatase 1, regulatory sub	499076	1	75625471	75648495	23025	3051,16	0,46	2,14E-01
ENSRNOG00000031789	**Rangap1**	RAN GTPase activating protein 1 (Ra	362965	7	122915003	122942464	27462	6391,52	0,62	2,14E-01
ENSRNOG00000032522	**RGD15607**	Protein RGD1560784 [Source:UniPro	363449	X	15675897	15676895	999	96,22	0,63	2,14E-01
ENSRNOG00000007971	**Wbp2**	WW domain binding protein 2 (Wbp2),	192645	10	103647548	103654798	7251	3519,84	0,39	2,14E-01
ENSRNOG00000013970	**Cdt1**	chromatin licensing and DNA replicati	292071	19	66090081	66095027	4947	343,02	0,57	2,14E-01

ENSRNOG00000018790	**Kcnh4**	potassium voltage-gated channel, sub	114032	10	88441734	88461552	19819	118,46	-0,64	2,14E-01
ENSRNOG00000002753	**Adam11**	ADAM metallopeptidase domain 11 (A	360638	10	90521652	90536723	15072	486,86	-0,54	2,15E-01
ENSRNOG00000038406	**Tmem132b**	transmembrane protein 132B (Tmem1	304458	12	38141550	38271429	129880	391,83	-0,75	2,15E-01
ENSRNOG00000021090	**Pygm**	phosphorylase, glycogen, muscle (Pyg	24701	1	228744114	228758930	14817	103,52	-0,91	2,15E-01
ENSRNOG00000030705	**Cpsf1**	cleavage and polyadenylation specific	366952	7	117649865	117660364	10500	1768,95	0,36	2,15E-01
ENSRNOG00000038044	**Tsc22d2**	TSC22 domain family, member 2 (Tsc2	499624	2	168025537	168072788	47252	528,38	-0,35	2,15E-01
ENSRNOG00000006893	**Ppm1k**	protein phosphatase, Mg2+/Mn2+ dep	312381	4	153516057	153540007	23951	288,46	-0,57	2,15E-01
ENSRNOG00000010086	**Plagl2**	pleiomorphic adenoma gene-like 2 (Pl	296281	3	155374856	155388034	13179	430,02	0,62	2,15E-01
ENSRNOG00000010466	**Chpf2**	chondroitin polymerizing factor 2 (Chp	296733	4	7120978	7126440	5463	1320,77	0,42	2,15E-01
ENSRNOG00000050998		Protein LOC686860 [Source:UniProtK	686860	20	6755278	6758464	3187	18,44	0,92	2,15E-01
ENSRNOG00000000423	**LOC10036**	serine/threonine kinase 19 (Stk19), ml	1E+08	20	6396797	6399301	2505	68,24	0,62	2,16E-01
ENSRNOG00000013072	**Plxna4a**	Protein Plxna4a [Source:UniProtKB/T	312213	4	59192168	59552548	360381	1848,02	-0,41	2,16E-01
ENSRNOG00000049959	**Igsf21**	immunoglobin superfamily, member 2	298591	5	162230228	162245527	15300	640,49	-0,66	2,16E-01
ENSRNOG00000016222	**Ppp1r3g**	Protein Ppp1r3g; RCG63346 [Source	291069	17	31464150	31465193	1044	25,24	-0,88	2,16E-01
ENSRNOG00000017285	**Tab1**	TGF-beta activated kinase 1/MAP3K7	315139	7	121340948	121371923	30976	1845,62	0,47	2,16E-01
ENSRNOG00000043199		blood vessel epicardial substance [Source:MGI S		20	52052641	52086539	33899	38,98	-0,69	2,16E-01
ENSRNOG00000017918	**Iglon5**	Protein Iglon5 [Source:UniProtKB/TrE	308557	1	99516991	99532725	15735	572,57	0,55	2,16E-01
ENSRNOG00000029194	**Dhx30**	DEAH (Asp-Glu-Ala-His) box helicase	367172	8	117515770	117542943	27174	3687,01	0,51	2,16E-01
ENSRNOG00000036745	**Gna13**	guanine nucleotide binding protein (G	303634	10	97371692	97404295	32604	412,59	-0,55	2,16E-01
ENSRNOG00000007500	**Top1mt**	topoisomerase (DNA) I, mitochondrial	300029	7	116583001	116606332	23332	399,61	0,50	2,17E-01
ENSRNOG00000015357	**Bmper**	BMP-binding endothelial regulator (Bm	300455	8	24409998	24655248	245251	456,80	-0,83	2,17E-01
ENSRNOG00000020441		vacuolar protein sorting 25 homolog (S	287715	10	88972339	89007992	35654	2578,26	0,56	2,17E-01
ENSRNOG00000028545	**Ahnak2**	Protein Ahnak2 [Source:UniProtKB/Tr	314478	6	146342143	146344449	2307	46,96	-0,73	2,17E-01
ENSRNOG00000007975	**Ncoa2**	nuclear receptor coactivator 2 (Ncoa2)	83724	5	10456313	10547228	90916	1346,68	-0,41	2,17E-01
ENSRNOG00000024635	**Cramp1l**	Protein Cramp1l [Source:UniProtKB/T	287127	10	14143850	14187194	43345	425,34	0,50	2,17E-01
ENSRNOG00000021008	**Kctd16**	potassium channel tetramerization dor	291618	18	33119771	33404042	284272	37,54	-0,85	2,18E-01
ENSRNOG00000001414	**Serpine1**	serpin peptidase inhibitor, clade E (ne:	24617	12	24653385	24663763	10379	51,35	-0,69	2,18E-01
ENSRNOG00000032274				MT	3835	3903	69	539,96	-0,77	2,18E-01
ENSRNOG00000049142	**Hdgfrp2**	hepatoma-derived growth factor-relate	171073	9	9959298	9978830	19533	3260,23	0,43	2,18E-01
ENSRNOG00000001339		26S proteasome non-ATPase regulatc	161475	12	40668828	40698959	30132	1210,34	0,32	2,18E-01
ENSRNOG00000003679	**Med13**	mediator complex subunit 13 (Med13)	303403	10	73600909	73685836	84928	1706,37	-0,51	2,19E-01
ENSRNOG00000004410		paired box 6 (Pax6), mRNA [Source:R	25509	3	102327021	102348393	21373	312,79	-0,55	2,19E-01
ENSRNOG00000009207	**Spata2**	spermatogenesis associated 2 (Spata2	114210	3	170381599	170389997	8399	1710,75	0,52	2,19E-01
ENSRNOG00000016553	**Vwa5b1**	von Willebrand factor A domain contai	313653	5	160746778	160801678	54901	59,49	-0,75	2,19E-01
ENSRNOG00000006403	**Pvrl1**	Poliovirus receptor-related 1; Protein F	192183	8	46715098	46774064	58967	1081,56	-0,44	2,19E-01
ENSRNOG00000014719	**Snx19**	sorting nexin 19 (Snx19), mRNA [Sour	315478	8	31526777	31563215	36439	1369,35	-0,42	2,19E-01
ENSRNOG00000017343	**LOC10091**	F-box and WD repeat domain containi	309444	1	272756541	272852823	96283	739,15	0,43	2,19E-01
ENSRNOG00000022609	**Mrps10**	mitochondrial ribosomal protein S10 (N	363187	9	14558418	14567371	8954	230,71	0,50	2,19E-01

ENSRNOG00000027726	**Tars2**	threonyl-tRNA synthetase 2, mitochon	310672	2	217350915	217368084	17170	891,24	0,32	2,19E-01
ENSRNOG00000029707		NADH dehydrogenase subunit 4 (mito	26201	MT	10160	11537	1378	66211,15	-0,51	2,19E-01
ENSRNOG00000010780	**Dlc1**	deleted in liver cancer 1 (Dlc1), mRNA	58834	16	58878663	58926658	47996	258,49	-0,44	2,20E-01
ENSRNOG00000011139	**Arg2**	arginase 2 (Arg2), mRNA [Source:Ref	29215	6	114998810	115025299	26490	561,96	0,69	2,20E-01
ENSRNOG00000016011	**Plekhg1**	pleckstrin homology domain containin	679812	1	41780838	41866419	85582	250,46	-0,63	2,20E-01
ENSRNOG00000027359	**Slc25a2**	solute carrier family 25 (mitochondrial	291640	18	30470724	30471521	798	28,65	-0,70	2,20E-01
ENSRNOG00000004201		regulating synaptic membrane exocyto	116839	7	77655910	78157335	501426	532,93	-0,44	2,20E-01
ENSRNOG00000004221	**Lgr5**	leucine rich repeat containing G protei	299802	7	58459666	58596253	136588	100,99	-0,82	2,20E-01
ENSRNOG00000011684		methylglutaconyl-CoA hydratase, mito	361215	17	14407791	14501277	93487	1546,64	0,58	2,20E-01
ENSRNOG00000043053	**Zfp639**	zinc finger protein 639 (Zfp639), mRN	683504	2	138565913	138573817	7905	352,75	-0,43	2,20E-01
ENSRNOG00000009805		wiskott-Aldrich syndrome protein famil	313024	5	155028091	155046935	18845	250,59	0,44	2,20E-01
ENSRNOG00000000532	**Cmtr1**	cap methyltransferase 1 (Cmtr1), mRN	309656	20	10524633	10568580	43948	3566,02	0,40	2,21E-01
ENSRNOG00000006973	**RGD13079**	similar to RIKEN cDNA C430008C19 (	314788	7	40343617	40354037	10421	186,97	-0,45	2,22E-01
ENSRNOG00000009862		olfactomedin 1 (Olfm1), mRNA [Sourc	93667	3	12115220	12152486	37267	6521,65	-0,43	2,22E-01
ENSRNOG00000023807	**Ccnyl1**	Protein Ccnyl1 [Source:UniProtKB/Trl	316452	9	71146078	71167668	21591	168,59	-0,52	2,22E-01
ENSRNOG00000009346	**Zbtb26**	zinc finger and BTB domain containin	311910	3	26927108	26937120	10013	217,99	-0,55	2,22E-01
ENSRNOG00000018200	**Gad2**	glutamate decarboxylase 2 (Gad2), m	24380	17	90851697	90914893	63197	1394,26	-0,44	2,22E-01
ENSRNOG00000018413	**Per3**	period circadian clock 3 (Per3), mRNA	78962	5	171665221	171700125	34905	305,13	-0,58	2,22E-01
ENSRNOG00000017980	**Itgal**	integrin, alpha L (Itgal), mRNA [Source	308995	1	205742369	205780279	37911	17,59	-0,91	2,22E-01
ENSRNOG00000022868	**Ell3**	elongation factor RNA polymerase II-li	296102	3	119941853	119945947	4095	63,46	-0,58	2,23E-01
ENSRNOG00000020793	**Ruvbl2**	RuvB-like 2 (E. coli) (Ruvbl2), mRNA [	292907	1	102493177	102506032	12856	1719,22	0,43	2,23E-01
ENSRNOG00000022540	**Trex1**	three prime repair exonuclease 1 (Tre	1E+08	8	117147877	117149221	1345	445,06	0,42	2,24E-01
ENSRNOG00000000711	**RGD13065**	Protein RGD1306556 [Source:UniPro	288744	12	51253965	51327952	73988	162,40	-0,49	2,24E-01
ENSRNOG00000021400	**Magee2**	melanoma antigen, family E, 2 (Magee	302392	X	76592888	76595012	2125	31,72	-0,79	2,24E-01
ENSRNOG00000001062	**LOC10090**	SET domain containing (lysine methyl	689820	12	39429983	39445521	15539	281,62	0,47	2,24E-01
ENSRNOG00000038066	**Fam155b**	Protein Fam155b [Source:UniProtKB/	688841	X	70452283	70474813	22531	232,93	0,64	2,24E-01
ENSRNOG00000003736		collagen, type V, alpha 2 precursor [S	85250	9	51758657	51823228	64572	961,20	0,61	2,25E-01
ENSRNOG00000001343	**Bcl7a**	Protein LOC690085 [Source:UniProtK	690085	12	40532067	40567658	35592	2304,57	0,57	2,25E-01
ENSRNOG00000021871		GCN1 general control of amino-acid s	690632	12	48527293	48587142	59850	4454,19	0,33	2,25E-01
ENSRNOG00000019501	**Rmnd1**	required for meiotic nuclear division 1	292268	1	42291016	42314989	23974	259,92	-0,54	2,25E-01
ENSRNOG00000047035	**Fadd**	Fas (TNFRSF6)-associated via death	266610	1	224600338	224606008	5671	383,69	-0,39	2,25E-01
ENSRNOG00000014338	**Slc25a25**	solute carrier family 25 (mitochondrial	246771	3	16791267	16801399	10133	1392,57	0,62	2,25E-01
ENSRNOG00000027400	**Vwc2l**	von Willebrand factor C domain-conta	501160	9	77609107	77799130	190024	59,64	-0,61	2,25E-01
ENSRNOG00000020580	**LOC50095**	RIKEN cDNA A230050P20 gene [Sou	500956	8	21946553	21952190	5638	584,57	0,36	2,26E-01
ENSRNOG00000028556	**Usp30**	ubiquitin specific peptidase 30 (Usp30	304579	12	50044991	50068680	23690	1683,88	0,46	2,26E-01
ENSRNOG00000024338	**LOC69027**	RIKEN cDNA 2410004P03 gene [Sou	690276	6	52026112	52032239	6128	81,45	-0,91	2,26E-01
ENSRNOG00000003742		Cyclin-dependent kinase-like 5; Protei	1E+08	X	35927782	36100916	173135	440,41	-0,76	2,26E-01
ENSRNOG00000018823		Nischarin [Source:UniProtKB/Swiss-Prot;Acc:Q		16	7182945	7219131	36187	18117,73	0,34	2,26E-01

ENSRNOG00000011852		myosin VI [Source:MGI Symbol;Acc:M	315840	8	87169192	87264964	95773	1502,84	-0,72	2,26E-01
ENSRNOG00000029068	**Wdr44**	WD repeat domain 44 (Wdr44), mRNA	246152	X	121006921	121111573	104653	428,90	-0,39	2,26E-01
ENSRNOG00000016478	**Eif5a**	eukaryotic translation initiation factor 5	287444	10	56272204	56276711	4508	12554,64	0,64	2,27E-01
ENSRNOG00000047154		Protein LOC100910839 [Source:UniP	1E+08	1	112865798	113069722	203925	100,42	-0,85	2,27E-01
ENSRNOG00000013386	**Psmb5**	proteasome (prosome, macropain) sul	29425	15	37143059	37147105	4047	5257,82	0,59	2,27E-01
ENSRNOG00000019737	**Sema4a**	sema domain, immunoglobulin domair	310630	2	207201927	207219874	17948	544,30	-0,51	2,27E-01
ENSRNOG00000026649	**Dnmt3a**	DNA (cytosine-5-)-methyltransferase 3	444984	6	38044960	38149613	104654	1761,46	-0,34	2,27E-01
ENSRNOG00000038427		predicted gene 1043 [Source:MGI Symbol;Acc:M		14	78331241	78344307	13067	29,57	-0,73	2,27E-01
ENSRNOG00000047061		zinc finger protein 773 [Source:MGI Symbol;Acc		1	71238918	71243045	4128	93,07	-0,56	2,27E-01
ENSRNOG00000011652		signal peptide peptidase-like 2A precu	311401	3	125887346	125929935	42590	451,07	-0,51	2,27E-01
ENSRNOG00000004829	**Immp1l**	Protein Immp1l; RCG27139, isoform C	691145	3	102617787	102646088	28302	273,00	-0,56	2,28E-01
ENSRNOG00000037960	**LOC68949**	RIKEN cDNA 6330403A02 gene [Sour	689499	13	103732725	103737132	4408	118,18	-0,68	2,28E-01
ENSRNOG00000008338		BSD domain containing 1 [Source:MGI Symbol;/		5	151382663	151410825	28163	2376,31	0,51	2,28E-01
ENSRNOG00000048261		zinc finger protein 52 [Source:MGI Symbol;Acc:M		1	63824978	63833865	8888	39,85	-0,89	2,28E-01
ENSRNOG00000009285	**Tssc1**	tumor suppressing subtransferable ca	362721	6	56832526	56837116	4591	626,89	0,49	2,28E-01
ENSRNOG00000039528	**Fam32a**	family with sequence similarity 32, me	498600	16	19164840	19169329	4490	3993,04	0,56	2,29E-01
ENSRNOG00000003863	**LOC10091**	similar to RIKEN cDNA 2310036O22 (	288920	19	37118343	37121759	3417	209,74	0,46	2,29E-01
ENSRNOG00000008965	**Socs2**	suppressor of cytokine signaling 2 (So	84607	7	36558017	36560623	2607	290,79	-0,56	2,29E-01
ENSRNOG00000013620		cell division protein kinase 13 isoform	306998	17	47890049	47978811	88763	1355,19	-0,46	2,29E-01
ENSRNOG00000001692	**Chaf1b**	chromatin assembly factor 1, subunit E	288242	11	37690511	37710590	20080	234,76	0,54	2,29E-01
ENSRNOG00000003866	**Cxcr4**	chemokine (C-X-C motif) receptor 4 (C	60628	13	50394711	50395760	1050	833,49	0,63	2,29E-01
ENSRNOG00000004036	**Snx12**	sorting nexin 12 (Snx12), mRNA [Sour	363478	X	71845291	71979356	134066	781,26	0,38	2,29E-01
ENSRNOG00000001130	**Nos1**	nitric oxide synthase 1, neuronal (Nos	24598	12	46049288	46209569	160282	685,67	-0,66	2,29E-01
ENSRNOG00000017052	**Pianp**	PILR alpha associated neural protein (	312711	4	224529480	224535510	6031	1822,22	0,54	2,29E-01
ENSRNOG00000022767	**Elfn1**	extracellular leucine-rich repeat and fil	288512	12	18741107	18805200	64094	760,79	-0,73	2,29E-01
ENSRNOG00000043381	**Cyth1**	cytohesin 1 (Cyth1), mRNA [Source:R	116691	10	106832874	106916333	83460	1768,43	0,54	2,30E-01
ENSRNOG00000024647	**Ppp4r2**	protein phosphatase 4, regulatory sub	297486	4	197767067	197805270	38204	1744,33	-0,50	2,30E-01
ENSRNOG00000018067	**Smn1**	survival of motor neuron 1, telomeric (	64301	2	49519267	49530311	11045	851,40	0,39	2,30E-01
ENSRNOG00000045791	**Eif4a3**	eukaryotic translation initiation factor 4	688288	10	108027036	108037029	9994	3230,60	0,50	2,31E-01
ENSRNOG00000003562	**Susd4**	sushi domain containing 4 (Susd4), m	289335	13	106115286	106237584	122299	1298,37	-0,38	2,31E-01
ENSRNOG00000005413	**Creb3l1**	cAMP responsive element binding pro	362165	3	87591105	87632647	41543	182,74	-0,89	2,31E-01
ENSRNOG00000019758	**Ipo13**	importin 13 (Ipo13), mRNA [Source:Re	116458	5	140517789	140538029	20241	1688,23	0,41	2,31E-01
ENSRNOG00000014234	**Hif1an**	hypoxia-inducible factor 1, alpha subu	309434	1	271752379	271762020	9642	796,14	0,66	2,31E-01
ENSRNOG00000011775	**Mfap3l**	microfibrillar-associated protein 3-like	306424	16	32571991	32587004	15014	463,55	-0,76	2,31E-01
ENSRNOG00000014859	**Rnf152**	ring finger protein 152 (Rnf152), mRN	293561	13	30348244	30424288	76045	882,05	-0,43	2,31E-01
ENSRNOG00000020717		biorientation of chromosomes in cell d	287173	10	16152118	16158451	6334	2414,27	0,38	2,31E-01
ENSRNOG00000030644		NADH dehydrogenase subunit 1 (mito	26193	MT	2740	3694	955	69290,92	-0,49	2,31E-01
ENSRNOG00000050320		Uncharacterized protein [Source:UniProtKB/TrE		16	59704269	59717933	13665	18,86	-0,80	2,31E-01

ENSRNOG00000002708	**Phf8**	PHD finger protein 8 (Phf8), mRNA [S	317425	X	22281689	22386870	105182	1513,50	0,62	2,31E-01
ENSRNOG00000012832	**Mfsd5**	major facilitator superfamily domain co	315329	7	141678906	141681186	2281	1189,90	0,44	2,31E-01
ENSRNOG00000020467	**Nrep**	neuronal regeneration related protein	338475	18	25900067	25926253	26187	3846,90	-0,45	2,31E-01
ENSRNOG00000015150	**Spg7**	spastic paraplegia 7 homolog (human)	353231	19	66586319	66618933	32615	1336,94	0,44	2,31E-01
ENSRNOG00000050401	**Vbp1**	Protein LOC681825 [Source:UniProtK	681825	1	154704717	154723069	18353	2825,52	0,59	2,32E-01
ENSRNOG00000000419	**Cfb**	complement component 2 (C2), mRNA	24231	20	6616019	6641139	25121	607,23	0,83	2,32E-01
ENSRNOG00000015187	**Acot8**	acyl-CoA thioesterase 8 (Acot8), mRN	170588	3	167446049	167457779	11731	562,15	0,42	2,32E-01
ENSRNOG00000003726		sex comb on midleg-like 2 (Drosophila) [Source:		X	35646943	35722945	76003	29,40	-0,69	2,32E-01
ENSRNOG00000004821	**Sntb1**	syntrophin, beta 1 (Sntb1), mRNA [So	299940	7	96019799	96286153	266355	224,08	-0,80	2,32E-01
ENSRNOG00000010769		predicted gene 9934 [Source:MGI Symbol;Acc:M		1	164153042	164164771	11730	36,73	0,77	2,32E-01
ENSRNOG00000021887	**Cyb561d2**	cytochrome b561 family, member D2 (	363137	8	115651589	115654110	2522	1114,36	0,50	2,32E-01
ENSRNOG00000001349	**Mcm7**	minichromosome maintenance comple	288532	12	21364250	21372100	7851	2077,45	0,55	2,32E-01
ENSRNOG00000016539	**Rab24**	RAB24, member RAS oncogene famil	361208	17	11946939	11949132	2194	1410,56	0,43	2,32E-01
ENSRNOG00000008000	**Syt13**	synaptotagmin XIII (Syt13), mRNA [Sc	80977	3	88516918	88555227	38310	358,74	-0,71	2,32E-01
ENSRNOG00000011575		A disintegrin and metalloproteinase wi	361412	19	56829758	56925970	96213	128,38	-0,58	2,33E-01
ENSRNOG00000017495	**Klhdc3**	kelch domain containing 3 (Klhdc3), m	363192	9	15519048	15525376	6329	4699,34	0,47	2,33E-01
ENSRNOG00000020475	**Sart1**	squamous cell carcinoma antigen reco	29678	1	227691827	227700489	8663	1664,71	0,35	2,33E-01
ENSRNOG00000000847	**Csnk2b**	casein kinase 2, beta polypeptide (Csı	81650	20	7181952	7186247	4296	3528,58	0,49	2,33E-01
ENSRNOG00000021112	**Cdc42se1**	CDC42 small effector 1 (Cdc42se1), n	499672	2	215902738	215904577	1840	445,22	0,51	2,33E-01
ENSRNOG00000024170	**Phf5a**	PHD finger protein 5A (Phf5a), mRNA	192246	7	123070683	123077281	6599	1440,62	0,38	2,33E-01
ENSRNOG00000021824	**Dnajb1**	DnaJ (Hsp40) homolog, subfamily B, r	361384	19	35726334	35730075	3742	2685,09	0,44	2,33E-01
ENSRNOG00000001032	**Ccz1**	CCZ1 vacuolar protein trafficking and	360768	12	14682886	14706374	23489	749,34	-0,45	2,35E-01
ENSRNOG00000001820		mediator of RNA polymerase II transcı	312849	4	245120185	245126317	6133	268,91	-0,58	2,35E-01
ENSRNOG00000003873	**Cpd**	carboxypeptidase D (Cpd), mRNA [So	25306	10	63024264	63088488	64225	1726,85	-0,54	2,35E-01
ENSRNOG00000004854	**Has2**	hyaluronan synthase 2 (Has2), mRNA	25694	7	97055458	97081461	26004	63,57	0,68	2,35E-01
ENSRNOG00000010634		multiple EGF-like-domains 11 [Source:MGI Sym		8	69722316	69888354	166039	1465,94	-0,76	2,35E-01
ENSRNOG00000018760	**Mpp7**	membrane protein, palmitoylated 7 (M	307035	17	61845053	62033794	188742	54,57	-0,71	2,35E-01
ENSRNOG00000003442	**Adora1**	adenosine A1 receptor (Adora1), mRN	29290	13	56097928	56131500	33573	1107,66	-0,46	2,35E-01
ENSRNOG00000042037	**Smim7**	small integral membrane protein 7 (Sn	688495	16	18838290	18842195	3906	2915,02	0,33	2,36E-01
ENSRNOG00000000899		beta 1,3-galactosyltransferase-like [Source:MGI		12	8503727	8573900	70174	380,08	-0,49	2,36E-01
ENSRNOG00000001128	**Tesc**	Protein Tesc [Source:UniProtKB/TrEN	288689	12	45973046	46006207	33162	52,45	-0,89	2,36E-01
ENSRNOG00000021817	**Irak2**	interleukin-1 receptor-associated kinas	362418	4	208890974	208947452	56479	58,42	-0,68	2,36E-01
ENSRNOG00000001726	**Tmem44**	Protein Tmem44; Similar to transmem	288028	11	76805943	76840991	35049	868,10	0,45	2,36E-01
ENSRNOG00000001114	**Wipi2**	WD repeat domain, phosphoinositide i	288498	12	15997276	16025704	28429	1462,39	0,66	2,36E-01
ENSRNOG00000002349	**Gabra2**	gamma-aminobutyric acid (GABA-A) r	289606	14	39473412	39609387	135976	873,97	-0,68	2,36E-01
ENSRNOG00000005437	**Hrsp12**	heat-responsive protein 12 (Hrsp12), r	65151	7	73422767	73436388	13622	807,27	-0,84	2,36E-01
ENSRNOG00000050780		folylpolyglutamyl synthetase [Source:MGI Symb		3	17058730	17068931	10202	626,63	0,42	2,36E-01
ENSRNOG00000008555	**RGD15622**	ribosomal protein S20 (Rps20), mRNA	122772	5	21486034	21487196	1163	93,37	0,52	2,36E-01

ENSRNOG00000028036	**Adamts7**	ADAM metallopeptidase with thrombo	315879	8	97035311	97074365	39055	868,86	0,41	2,37E-01
ENSRNOG00000032394	**Tymp**	thymidine phosphorylase (Tymp), mRI	315219	7	130029367	130032479	3113	182,06	0,48	2,37E-01
ENSRNOG00000048061	**Brcc3**	BRCA1/BRCA2-containing complex, s	316794	9	8866553	8867428	876	253,90	-0,64	2,37E-01
ENSRNOG00000018379		zinc finger protein 688 [Source:MGI Symbol;Acc		1	205864686	205867558	2873	255,45	0,40	2,37E-01
ENSRNOG00000036691	**Fam195b**	family with sequence similarity 195, m	360677	10	109313274	109315325	2052	2669,38	0,52	2,37E-01
ENSRNOG00000017718	**Tmco6**	transmembrane and coiled-coil domai	291661	18	29282952	29289547	6596	395,27	0,44	2,37E-01
ENSRNOG00000024825	**Fam163a**	family with sequence similarity 163, m	498257	13	78666728	78677673	10946	62,38	-0,87	2,37E-01
ENSRNOG00000027796		neurexin I [Source:MGI Symbol;Acc:MGI:10963		6	24408199	24715174	306976	1608,45	-0,38	2,37E-01
ENSRNOG00000011632	**Cct5**	chaperonin containing Tcp1, subunit 5	294864	2	104340316	104351466	11151	7231,72	0,52	2,38E-01
ENSRNOG00000022432	**Tmem59l**	transmembrane protein 59-like (Tmem	306349	16	20547483	20551145	3663	2248,37	0,58	2,38E-01
ENSRNOG00000019811	**LOC10036**	translocase of inner mitochondrial mei	54312	16	8241068	8267099	26032	675,18	0,49	2,39E-01
ENSRNOG00000019959	**Kcnc3**	potassium voltage gated channel, Sha	117101	1	101659056	101673261	14206	1299,57	-0,48	2,39E-01
ENSRNOG00000021689	**Rps6kb2**	ribosomal protein S6 kinase, polypepti	361696	1	226276853	226283539	6687	401,94	0,61	2,39E-01
ENSRNOG00000045816		Glutamate receptor 1 [Source:UniProtKB/TrEMI		10	42257921	42571220	313300	3032,82	-0,55	2,39E-01
ENSRNOG00000005502	**Ankib1**	ankyrin repeat and IBR domain contai	368062	4	27376703	27500432	123730	1748,67	-0,43	2,39E-01
ENSRNOG00000004155	**Samd14**	sterile alpha motif domain containing 1	287637	10	82639539	82647961	8423	2304,50	0,50	2,39E-01
ENSRNOG00000021839	**RGD15635**	Protein RGD1563556 [Source:UniPro	315409	8	6254657	6302976	48320	147,51	0,57	2,39E-01
ENSRNOG00000029399	**Bcam**	basal cell adhesion molecule (Luthera	78958	1	81939822	81954480	14659	195,13	-0,49	2,39E-01
ENSRNOG00000015439	**Man2a1**	mannosidase, alpha, class 2A, membe	25478	9	111831033	111987870	156838	434,17	-0,41	2,39E-01
ENSRNOG00000024863	**Fam76b**	family with sequence similarity 76, me	367021	8	12306043	12324965	18923	388,86	-0,56	2,39E-01
ENSRNOG00000007032	**Amigo2**	adhesion molecule with Ig like domain	300186	7	138328447	138330009	1563	112,98	-0,56	2,40E-01
ENSRNOG00000010260	**Dixdc1**	DIX domain containing 1 (Dixdc1), mF	363062	8	53688641	53740876	52236	772,13	-0,38	2,40E-01
ENSRNOG00000019169	**RGD15622**	similar to RIKEN cDNA 0610039J04 (I	292100	19	68313479	68327797	14319	1133,72	0,46	2,41E-01
ENSRNOG00000014019	**Tbc1d7**	TBC1 domain family, member 7 (Tbc1	361227	17	25727575	25744428	16854	388,20	0,44	2,41E-01
ENSRNOG00000021005	**Mrpl16**	mitochondrial ribosomal protein L16 (M	293754	1	235210618	235215431	4814	588,44	0,44	2,41E-01
ENSRNOG00000004259	**Dohh**	deoxyhypusine hydroxylase/monooxy	314644	7	11358019	11362757	4739	2834,39	0,59	2,42E-01
ENSRNOG00000014987	**Mdfi**	MyoD family inhibitor (Mdfi), mRNA [S	501097	9	14090319	14108948	18630	17,99	-0,87	2,42E-01
ENSRNOG00000047248		Uncharacterized protein [Source:UniProtKB/TrE		5	10836117	10836626	510	28,50	-0,89	2,42E-01
ENSRNOG00000030927	**Grid2ip**	glutamate receptor, ionotropic, delta 2	288484	12	15261534	15291234	29701	37,68	-0,84	2,42E-01
ENSRNOG00000000569	**MGC11271**	platelet receptor Gi24 [Source:RefSe	690899	20	31709059	31731463	22405	624,09	-0,81	2,42E-01
ENSRNOG00000009590	**Stox2**	storkhead box 2 (Stox2), transcript var	306459	16	47836086	47942379	106294	4092,19	-0,31	2,43E-01
ENSRNOG00000010204		transmembrane protein 9 precursor [S	289046	13	57910156	57927429	17274	1875,71	0,40	2,43E-01
ENSRNOG00000000257	**Smpd3**	sphingomyelin phosphodiesterase 3, r	94338	19	49107658	49191221	83564	5069,33	-0,40	2,43E-01
ENSRNOG00000005365		Kv channel-interacting protein 1 (Kcnip	65023	10	18444651	18663340	218690	352,56	-0,53	2,43E-01
ENSRNOG00000008289	**Slc25a3**	solute carrier family 25 (mitochondrial	245959	7	31904034	31911476	7443	8780,77	0,36	2,43E-01
ENSRNOG00000008630	**Psma7**	proteasome (prosome, macropain) sul	29674	3	181692291	181698641	6351	6569,44	0,40	2,43E-01
ENSRNOG00000012135		myeloid/lymphoid or mixed-lineage leukemia (tri		10	85395401	85411740	16340	1215,99	0,41	2,43E-01
ENSRNOG00000018993	**Ilk**	integrin-linked kinase (Ilk), mRNA [Sou	170922	1	177584793	177591044	6252	3148,64	0,34	2,43E-01

ENSRNOG00000020134	**Upf1**	Protein LOC684558 [Source:UniProtK	684558	16	20677669	20697168	19500	2177,75	0,38	2,43E-01
ENSRNOG00000048237	**Tcta**	T-cell leukemia translocation altered (	306587	8	116427899	116430966	3068	1272,87	0,50	2,43E-01
ENSRNOG00000017091	**Slc25a46**	solute carrier family 25, member 46 (S	291709	18	24086615	24114920	28306	1065,62	-0,48	2,43E-01
ENSRNOG00000013535		cadherin-6 precursor [Source:RefSeq	25409	2	81528704	81594434	65731	664,44	-0,49	2,43E-01
ENSRNOG00000005353	**Rybp**	RING1 and YY1 binding protein (Rybp), mRNA [		4	197180863	197228554	47692	423,03	-0,41	2,44E-01
ENSRNOG00000021009	**Gapdhs**	glyceraldehyde-3-phosphate dehydrog	66020	1	90334994	90349297	14304	96,88	0,53	2,44E-01
ENSRNOG00000012634	**Fbxo10**	F-box only protein 10 (Predicted), isof	362511	5	65100612	65126031	25420	1297,44	0,53	2,44E-01
ENSRNOG00000015078	**Ifitm3**	interferon induced transmembrane pro	361673	1	220730714	220731861	1148	261,13	0,87	2,44E-01
ENSRNOG00000023381	**Proca1**	protein interacting with cyclin A1 (Proc	497959	10	66167747	66180504	12758	226,13	0,68	2,44E-01
ENSRNOG00000024159	**Fcer1g**	Fc fragment of IgE, high affinity I, rece	25441	13	94228761	94233191	4431	19,37	-0,86	2,44E-01
ENSRNOG00000002922	**Adora2b**	adenosine A2B receptor (Adora2b), m	29316	10	48358060	48374870	16811	156,62	-0,66	2,44E-01
ENSRNOG00000013102	**Entpd2**	ectonucleoside triphosphate diphosph	64467	3	2599308	2604728	5421	1493,86	-0,86	2,44E-01
ENSRNOG00000017326		C-terminal binding protein 2 (Ctbp2), r	81717	1	211993864	212033074	39211	927,91	-0,36	2,46E-01
ENSRNOG00000031739	**Fam71e1**	Protein Fam71e1 [Source:UniProtKB/	361564	1	101567063	101571409	4347	138,46	0,51	2,46E-01
ENSRNOG00000022286	**Dmrtc1b**	Protein Dmrtc1b [Source:UniProtKB/T	680068	X	74474144	74477761	3618	96,43	-0,58	2,46E-01
ENSRNOG00000003973	**Cyb5r1**	cytochrome b5 reductase 1 (Cyb5r1),	304805	13	56286702	56293106	6405	717,43	0,41	2,46E-01
ENSRNOG00000037300	**RGD15622**	Protein RGD1562276 [Source:UniPro	360567	10	61132535	61150418	17884	68,96	0,59	2,46E-01
ENSRNOG00000046585		Protein LOC100359642 [Source:UniP	1E+08	3	156559482	156596179	36698	683,79	0,35	2,47E-01
ENSRNOG00000017259	**Tacc3**	transforming, acidic coiled-coil contain	360962	14	83025668	83039430	13763	617,06	0,54	2,47E-01
ENSRNOG00000030547	**Dpp6**	dipeptidylpeptidase 6 (Dpp6), mRNA [	29272	4	4062161	4724198	662038	2713,22	-0,49	2,47E-01
ENSRNOG00000007930		Uncharacterized protein [Source:UniProtKB/TrE		3	154686207	154687101	895	302,52	0,66	2,47E-01
ENSRNOG00000000581	**Cdc40**	cell division cycle 40 (Cdc40), mRNA [	361859	20	50156118	50219885	63768	603,22	-0,43	2,47E-01
ENSRNOG00000016384	**Pik3cb**	phosphatidylinositol-4,5-bisphosphate	85243	8	106701471	106771882	70412	333,32	-0,49	2,47E-01
ENSRNOG00000002873	**Fam183b**	Protein Fam183b [Source:UniProtKB/	287346	10	44496938	44506773	9836	56,92	-0,88	2,47E-01
ENSRNOG00000014348	**Cacng8**	calcium channel, voltage-dependent, g	140729	1	63321710	63341943	20234	530,30	-0,45	2,47E-01
ENSRNOG00000004198	**Stxbp6**	syntaxin binding protein 6 (amisyn) (S	362734	6	74671542	74901751	230210	248,49	-0,56	2,48E-01
ENSRNOG00000011009	**Cmtm4**	CKLF-like MARVEL transmembrane d	498902	19	783437	822756	39320	693,75	-0,34	2,48E-01
ENSRNOG00000011361	**Slc37a4**	solute carrier family 37 (glucose-6-pho	29573	8	47335910	47341992	6083	1013,43	0,35	2,48E-01
ENSRNOG00000003567	**Ppox**	protoporphyrinogen oxidase (Ppox), m	289219	13	94276960	94281095	4136	698,48	0,42	2,48E-01
ENSRNOG00000004246	**Spag7**	sperm associated antigen 7 (Spag7), r	303260	10	57030583	57036740	6158	725,57	0,35	2,48E-01
ENSRNOG00000007104		inositol 1,4,5-trisphosphate receptor, t	25262	4	204714826	205047900	333075	913,82	-0,68	2,48E-01
ENSRNOG00000008802	**Dnajc11**	DnaJ (Hsp40) homolog, subfamily C, r	362666	5	172668492	172712646	44155	2450,08	0,44	2,48E-01
ENSRNOG00000009656	**Rspo1**	R-spondin 1 (Rspo1), mRNA [Source:I	313589	5	146755088	146776423	21336	18,13	-0,88	2,48E-01
ENSRNOG00000017193	**Lingo1**	leucine rich repeat and Ig domain cont	315691	8	59844165	59860388	16224	4764,84	-0,55	2,48E-01
ENSRNOG00000050837		Uncharacterized protein [Source:UniProtKB/TrE		X	19212358	19226625	14268	353,61	0,56	2,48E-01
ENSRNOG00000020075	**Eef1g**	eukaryotic translation elongation facto	293725	1	232100965	232111615	10651	9449,07	0,46	2,48E-01
ENSRNOG00000020217		PAXIP1-associated glutamate-rich pro	293500	1	205429226	205431124	1899	449,34	0,42	2,48E-01
ENSRNOG00000012701	**Map7**	microtubule-associated protein 7 (Map	293016	1	17239998	17290726	50729	246,50	-0,50	2,48E-01

ENSRNOG00000039107	**Mfrp**	membrane frizzled-related protein (Mfı	315597	8	47054088	47059250	5163	122,71	-0,67	2,49E-01
ENSRNOG00000019751	**Cyb5r2**	cytochrome b5 reductase 2 (Cyb5r2),	365345	1	178970105	178978337	8233	142,89	-0,70	2,50E-01
ENSRNOG00000017622	**Npm3**	Protein Npm3 [Source:UniProtKB/TrE	502389	1	272934815	272936286	1472	469,60	0,36	2,51E-01
ENSRNOG00000020597	**RGD13050**	similar to RIKEN cDNA 2310057M21 (	309029	1	208958026	208974579	16554	249,90	0,60	2,51E-01
ENSRNOG00000019357	**Tax1bp3**	Tax1 (human T-cell leukemia virus typ	360564	10	59482718	59487235	4518	1238,10	0,44	2,52E-01
ENSRNOG00000023400	**Dtx3l**	deltex 3-like (Drosophila) (Dtx3l), mRN	498089	11	70846234	70853376	7143	171,35	0,79	2,52E-01
ENSRNOG00000015370	**Slc35g2**	solute carrier family 35, member G2 (S	315957	8	108269640	108296902	27263	161,80	-0,74	2,52E-01
ENSRNOG00000002840	**Atp5b**	ATP synthase, H+ transporting, mitoch	171374	7	2484095	2490338	6244	35907,24	0,33	2,53E-01
ENSRNOG00000025899	**Thada**	thyroid adenoma associated (Thada),	313865	6	7364891	7677173	312283	720,20	-0,40	2,53E-01
ENSRNOG00000021216	**Pex11b**	peroxisomal biogenesis factor 11 beta	310682	2	218249072	218257960	8889	1375,38	0,36	2,53E-01
ENSRNOG00000007356	**Chmp3**	charged multivesicular body protein 3	282834	4	164228440	164274202	45763	1157,18	0,50	2,53E-01
ENSRNOG00000008790	**Gdap1l1**	ganglioside-induced differentiation-ass	311616	3	166016380	166034837	18458	2231,57	0,43	2,53E-01
ENSRNOG00000020726	**Sipa1**	signal-induced proliferation-associatec	361710	1	227941671	227953682	12012	59,90	-0,56	2,53E-01
ENSRNOG00000000440	**Pbx2**	pre-B-cell leukemia homeobox 2 (Pbx:	406164	20	6437161	6442086	4926	812,02	0,43	2,53E-01
ENSRNOG00000009033	**Cntn2**	contactin 2 (axonal) (Cntn2), mRNA [S	25356	13	54360710	54389586	28877	525,77	-0,56	2,53E-01
ENSRNOG00000014237	**Zfp503**	zinc finger protein 503 (Zfp503), mRN.	305687	15	2357098	2360886	3789	185,23	0,50	2,53E-01
ENSRNOG00000019949	**Mrps12**	mitochondrial ribosomal protein S12 (N	292758	1	88101812	88104707	2896	561,29	0,53	2,53E-01
ENSRNOG00000021096	**Tmem143**	transmembrane protein 143 (Tmem14	308593	1	102962983	102979034	16052	264,47	0,38	2,53E-01
ENSRNOG00000021787	**Zfp217**	zinc finger protein 217 (Zfp217), mRN.	311764	3	173096854	173107574	10721	176,99	0,60	2,53E-01
ENSRNOG00000031367	**Hprt1**	hypoxanthine phosphoribosyltransfera	24465	X	153249883	153281841	31959	74,23	-0,62	2,53E-01
ENSRNOG00000001555	**Btg3**	BTG family, member 3 (Btg3), mRNA [	54230	11	20528293	20544036	15744	279,82	-0,40	2,54E-01
ENSRNOG00000004713	**Kcnj16**	potassium inwardly-rectifying channel,	29719	10	99084253	99086021	1769	25,12	-0,85	2,54E-01
ENSRNOG00000004889	**Vangl2**	VANGL planar cell polarity protein 2 (V	289229	13	94905108	94928701	23594	1162,29	0,39	2,54E-01
ENSRNOG00000005766	**Tbc1d20**	TBC1 domain family, member 20 (Tbc	362237	3	154120296	154137199	16904	917,83	0,41	2,54E-01
ENSRNOG00000011567	**Rpusd4**	RNA pseudouridylate synthase domaiı	315550	8	36485875	36495292	9418	394,64	0,36	2,54E-01
ENSRNOG00000013703	**Siah2**	siah E3 ubiquitin protein ligase 2 (Siah	140593	2	168292053	168309802	17750	435,73	-0,49	2,54E-01
ENSRNOG00000014397	**Zic2**	Zic family member 2 (Zic2), mRNA [Sc	361096	15	112296546	112301371	4826	297,79	-0,43	2,54E-01
ENSRNOG00000015237	**Gle1**	GLE1 RNA export mediator (Gle1), mF	362098	3	13842293	13871572	29280	1106,10	0,55	2,54E-01
ENSRNOG00000019107	**LOC100365590**			9	66515715	66515976	262	96,81	-0,62	2,54E-01
ENSRNOG00000025711	**LOC49977(**	DNA segment, Chr 2, Wayne State Ur	499770	3	14096394	14101124	4731	548,51	0,36	2,54E-01
ENSRNOG00000046191	**Mb21d1**	Protein LOC682147 [Source:UniProtK	682147	8	85345187	85353097	7911	33,02	0,67	2,54E-01
ENSRNOG00000050697	**Ctsz**	cathepsin Z (Ctsz), mRNA [Source:Re	252929	3	178574619	178585389	10771	62,08	-0,87	2,54E-01
ENSRNOG00000016164	**Fcrl2**	Fc receptor-like 2 (Fcrl2), mRNA [Sou	310694	2	205998855	206009505	10651	171,24	-0,75	2,54E-01
ENSRNOG00000008067		REST corepressor 1 [Source:MGI Symbol;Acc:M		6	144866567	144901118	34552	264,09	0,41	2,55E-01
ENSRNOG00000000695	**Ssh1**	Protein Ssh1 [Source:UniProtKB/TrEI	304580	12	50192169	50229287	37119	367,33	0,46	2,55E-01
ENSRNOG00000006966	**Nfia**	nuclear factor I/A (Nfia), mRNA [Sourc	25492	5	120364845	120695028	330184	1457,70	-0,57	2,55E-01
ENSRNOG00000009716	**Casc3**	cancer susceptibility candidate 3 (Cas	259170	10	86520779	86540730	19952	1967,93	0,52	2,55E-01
ENSRNOG00000011419	**Aldh6a1**	aldehyde dehydrogenase 6 family, me	81708	6	117379212	117399815	20604	2365,52	-0,64	2,55E-01

ENSRNOG00000015413	**Qpctl**	glutaminyl-peptide cyclotransferase-lik	292687	1	81314398	81323467	9070	1027,45	0,39	2,55E-01
ENSRNOG00000024867	**Eme2**	Protein Eme2 [Source:UniProtKB/TrE	302982	10	14070214	14072952	2739	416,04	0,41	2,55E-01
ENSRNOG00000004910	**Eif2d**	eukaryotic translation initiation factor 2	498225	13	53011087	53030016	18930	716,85	0,32	2,56E-01
ENSRNOG00000020030	**Crlf1**	cytokine receptor-like factor 1 (Crlf1), i	290655	16	20530521	20541796	11276	285,76	0,46	2,56E-01
ENSRNOG00000001417	**Plod3**	procollagen-lysine, 2-oxoglutarate 5-di	288583	12	24728207	24738766	10560	1137,76	0,39	2,56E-01
ENSRNOG00000015518	**Rbp4**	retinol binding protein 4, plasma (Rbp4	25703	1	264287001	264294216	7216	31,74	-0,67	2,56E-01
ENSRNOG00000017018		RIKEN cDNA A630007B06 gene [Sou	361773	1	284987105	285009140	22036	402,81	-0,75	2,56E-01
ENSRNOG00000010457	**Vash1**	Protein Vash1 [Source:UniProtKB/TrE	503052	6	119922878	119934446	11569	1903,53	0,51	2,56E-01
ENSRNOG00000011373	**Reep4**	receptor accessory protein 4 (Reep4),	306014	15	55958424	55962009	3586	96,73	0,59	2,57E-01
ENSRNOG00000009054	**Elmod1**	ELMO/CED-12 domain containing 1 (E	315670	8	57066609	57123000	56392	3330,92	-0,50	2,57E-01
ENSRNOG00000016396	**Fbxo11**	F-box protein 11 (Fbxo11), mRNA [Sou	301674	6	21634307	21658631	24325	1692,33	-0,33	2,57E-01
ENSRNOG00000045756		Uncharacterized protein [Source:UniProtKB/TrE		6	121480947	121537048	56102	167,12	-0,59	2,57E-01
ENSRNOG00000016147	**Slc17a6**	solute carrier family 17 (vesicular gluta	84487	1	108040511	108080510	40000	263,69	-0,47	2,57E-01
ENSRNOG00000005438	**Pcsk2**	proprotein convertase subtilisin/kexin t	25121	3	144059121	144362761	303641	1118,44	-0,56	2,57E-01
ENSRNOG00000013591	**Tmem9b**	TMEM9 domain family, member B (Tm	293415	1	181347009	181364639	17631	1635,48	-0,44	2,57E-01
ENSRNOG00000025067	**Mocs3**	molybdenum cofactor synthesis 3 (Mo	311655	3	171125683	171127643	1961	407,20	0,55	2,57E-01
ENSRNOG00000032701	**Slc6a8**	solute carrier family 6 (neurotransmitte	50690	1	152879305	152888609	9305	2292,15	-0,46	2,57E-01
ENSRNOG00000039472				18	30262285	30264692	2408	101,47	-0,62	2,57E-01
ENSRNOG00000045840	**RGD15623**	Histone H4 Osteogenic growth peptide	64627	4	235021876	235022187	312	66,11	-0,65	2,57E-01
ENSRNOG00000021285	**Celsr1**	Protein Celsr1 [Source:UniProtKB/TrE	300128	7	126548437	126625585	77149	555,55	-0,72	2,58E-01
ENSRNOG00000007600	**Igsf1**	immunoglobulin superfamily, member	302822	X	136852224	136867466	15243	2313,74	-0,83	2,58E-01
ENSRNOG00000010972	**Neurog2**	Protein Neurog2; RCG28897 [Source	295475	2	251308041	251308832	792	23,63	-0,75	2,58E-01
ENSRNOG00000009278	**Ift88**	intraflagellar transport 88 homolog (Ch	305918	15	41537251	41630808	93558	419,09	0,47	2,58E-01
ENSRNOG00000020869	**mrpl9**	mitochondrial ribosomal protein L9 (mi	310653	2	215163444	215168154	4711	1523,66	0,53	2,58E-01
ENSRNOG00000008116	**Ppap2b**	phosphatidic acid phosphatase type 2	192270	5	128551196	128626137	74942	5609,31	-0,77	2,58E-01
ENSRNOG00000013166	**Wnt4**	wingless-type MMTV integration site fa	84426	5	159405834	159424674	18841	71,41	-0,84	2,58E-01
ENSRNOG00000002318	**Limch1**	LIM and calponin homology domains 1	305332	14	42560956	42868253	307298	3196,44	-0,34	2,58E-01
ENSRNOG00000011782	**Got2**	glutamic-oxaloacetic transaminase 2, i	25721	19	9572757	9598443	25687	5881,42	0,39	2,59E-01
ENSRNOG00000014581	**LOC100910**	trafficking protein particle complex 2-lil	292074	19	66131866	66135551	3686	794,10	0,52	2,59E-01
ENSRNOG00000023456	**Rnf41**	ring finger protein 41 (Rnf41), mRNA [	362814	7	2809468	2831145	21678	3400,48	0,47	2,59E-01
ENSRNOG00000045961	**Lyrm7**	LYR motif containing 7 (Lyrm7), mRNA	686506	10	39955373	39974260	18888	469,06	-0,38	2,59E-01
ENSRNOG00000029450		brain-specific angiogenesis inhibitor 1	362931	7	115675214	115733670	58457	4245,20	-0,48	2,60E-01
ENSRNOG00000026953	**Gpr88**	G-protein coupled receptor 88 (Gpr88)	64443	2	237334865	237339419	4555	666,23	-0,51	2,61E-01
ENSRNOG00000003144		G-protein coupled receptor family C gr	287805	10	103052022	103073771	21750	54,89	-0,84	2,61E-01
ENSRNOG00000001250	**Lfng**	LFNG O-fucosylpeptide 3-beta-N-acet	170905	12	18113995	18122013	8019	1622,08	0,55	2,61E-01
ENSRNOG00000029862		SPC24, NDC80 kinetochore complex componen		8	22834762	22839400	4639	275,26	0,73	2,61E-01
ENSRNOG00000006762	**Nhp2l1**	NHP2 non-histone chromosome prote	300092	7	123267419	123272386	4968	425,48	0,34	2,61E-01
ENSRNOG00000017319	**Mertk**	c-mer proto-oncogene tyrosine kinase	65037	3	128669942	128777039	107098	196,03	-0,83	2,61E-01

ENSRNOG00000043093	**Ap1m2**	adaptor-related protein complex 1, mu	367038	8	22373292	22391178	17887	37,07	0,81	2,61E-01
ENSRNOG00000047247		protein tyrosine phosphatase, receptor type, S [S		9	9596330	9638569	42240	18871,18	-0,37	2,61E-01
ENSRNOG00000018895	**Mchr1**	melanin-concentrating hormone recep	83567	7	122432538	122435016	2479	70,31	-0,71	2,62E-01
ENSRNOG00000019596	**Smg9**	SMG9 nonsense mediated mRNA dec	365215	1	82523064	82545403	22340	719,40	0,46	2,62E-01
ENSRNOG00000009954	**Cacul1**	CDK2-associated, cullin domain 1 (Ca	365493	1	289157890	289215140	57251	1659,21	-0,36	2,62E-01
ENSRNOG00000023601	**Elavl4**	ELAV (embryonic lethal, abnormal visi	432358	5	133839926	133919410	79485	1076,05	-0,33	2,62E-01
ENSRNOG00000011032	**Lhfpl2**	lipoma HMGIC fusion partner-like 2 (L	294643	2	42942597	43023910	81314	859,02	-0,73	2,63E-01
ENSRNOG00000016066	**Bambi**	BMP and activin membrane-bound inh	83837	17	54939072	54943877	4806	130,96	-0,54	2,63E-01
ENSRNOG00000017284	**Nop16**	NOP16 nucleolar protein (Nop16), mR	306768	17	12685785	12690719	4935	1670,81	0,43	2,63E-01
ENSRNOG00000023458	**Dcaf12**	DDB1 and CUL4 associated factor 12	313296	X	131415634	131419442	3809	654,03	0,67	2,63E-01
ENSRNOG00000023463	**Parp9**	poly (ADP-ribose) polymerase family,	303905	11	70812299	70845022	32724	298,21	0,71	2,63E-01
ENSRNOG00000039858	**Mfsd12**	major facilitator superfamily domain cc	362824	7	11383866	11390540	6675	937,36	0,49	2,63E-01
ENSRNOG00000004899	**Kcns3**	potassium voltage-gated channel, dela	83588	6	46515729	46571013	55285	56,74	-0,58	2,63E-01
ENSRNOG00000020947	**Egln2**	EGL nine homolog 2 (C. elegans) (Egl	308457	1	85180395	85188174	7780	2865,38	0,47	2,63E-01
ENSRNOG00000023152	**Tmem201**	Protein Tmem201 [Source:UniProtKB	691426	5	170318703	170339591	20889	856,15	0,41	2,63E-01
ENSRNOG00000023216	**Ccdc17**	coiled-coil domain containing 17 (Ccdc	500528	5	139238283	139241972	3690	91,62	-0,63	2,63E-01
ENSRNOG00000046214	**Cyp27b1**	cytochrome P450, family 27, subfamily	114700	7	70512763	70517707	4945	38,23	0,80	2,63E-01
ENSRNOG00000030771	**Dgkb**	diacylglycerol kinase, beta (Dgkb), mR	54248	6	67113446	67868708	755263	333,12	-0,78	2,64E-01
ENSRNOG00000003533	**Clcn4**	chloride channel, voltage-sensitive 4 (	60586	X	25427594	25491603	64010	2247,17	-0,40	2,64E-01
ENSRNOG00000012503	**Dnajc16**	DnaJ (Hsp40) homolog, subfamily C, r	362652	5	164037421	164064397	26977	709,70	0,44	2,64E-01
ENSRNOG00000010038	**Psmc5**	proteasome (prosome, macropain) 26	81827	10	94197162	94203113	5952	2903,21	0,48	2,65E-01
ENSRNOG00000018408		Uncharacterized protein C19orf47 hom	292739	1	85578686	85602677	23992	810,37	0,33	2,65E-01
ENSRNOG00000007374	**Tac1**	tachykinin, precursor 1 (Tac1), transcri	24806	4	33499891	33507855	7965	41,40	-0,82	2,66E-01
ENSRNOG00000021548	**Rassf1**	Ras association (RalGDS/AF-6) doma	363140	8	115663409	115674549	11141	609,16	0,45	2,66E-01
ENSRNOG00000027592	**Rerg**	Protein Rerg; Similar to RAS-like, estr	502916	4	235327142	235433770	106629	78,51	-0,76	2,66E-01
ENSRNOG00000012835	**Espl1**	extra spindle pole bodies homolog 1 (S	315330	7	141693056	141719909	26854	418,04	0,50	2,66E-01
ENSRNOG00000012046	**Prmt5**	protein arginine methyltransferase 5 (F	364382	15	37041740	37051125	9386	2973,53	0,44	2,66E-01
ENSRNOG00000014142	**Ogfrl1**	opioid growth factor receptor-like 1 (O	316290	9	28469367	28483091	13725	414,37	-0,42	2,67E-01
ENSRNOG00000019572	**Lsm4**	LSM4 homolog, U6 small nuclear RNA	290647	16	20363467	20368886	5420	1506,03	0,50	2,67E-01
ENSRNOG00000010319	**Lcp1**	lymphocyte cytosolic protein 1 (Lcp1),	306071	15	60932334	60988849	56516	22,87	-0,85	2,67E-01
ENSRNOG00000017127	**LOC10036**	ribosomal protein L28 (Rpl28), mRNA	64638	1	75912910	75914436	1527	2702,20	0,46	2,67E-01
ENSRNOG00000033114	**Zfp187**	Protein Zfp187 [Source:UniProtKB/Tr	266792	17	58263506	58268024	4519	198,62	-0,53	2,67E-01
ENSRNOG00000021691	**Ccdc92**	coiled-coil domain containing 92 (Ccdc	1E+08	12	39085289	39112093	26805	1374,49	0,61	2,67E-01
ENSRNOG00000002693	**Nme1**	NME/NM23 nucleoside diphosphate ki	191575	10	81487115	81496485	9371	3384,36	0,48	2,67E-01
ENSRNOG00000037690	**Sertad3**	SERTA domain containing 3 (Sertad3)	499108	1	85498045	85501763	3719	92,47	0,49	2,69E-01
ENSRNOG00000002542	**Heatr6**	HEAT repeat containing 6 (Heatr6), ml	497972	10	70678105	70706990	28886	583,25	0,41	2,69E-01
ENSRNOG00000004901		kanadaptin [Source:RefSeq peptide;A	298805	6	36035092	36064887	29796	972,14	0,48	2,69E-01
ENSRNOG00000015334	**Fcho2**	FCH domain only 2 (Fcho2), mRNA [S	309129	2	48110537	48211938	101402	631,53	-0,39	2,69E-01

ENSRNOG00000019319	**Fchsd2**	FCH and double SH3 domains 2 (Fchs	308864	1	172337554	172585548	247995	3330,95	-0,35	2,69E-01
ENSRNOG00000042094	**Prr13**	proline rich 13 (Prr13), mRNA [Source	363004	7	141857064	141860364	3301	833,94	0,44	2,69E-01
ENSRNOG00000020213	**Tubg1**	tubulin, gamma 1 (Tubg1), mRNA [Sou	252921	10	88829361	88835951	6591	820,00	0,43	2,69E-01
ENSRNOG00000013263	**Ccdc151**	Protein Ccdc151 [Source:UniProtKB/T	315465	8	23055948	23069138	13191	29,41	-0,72	2,69E-01
ENSRNOG00000021528	**RGD15615**	tumor suppressor candidate 2 (Tusc2)	501052	8	115675223	115678441	3219	1982,19	0,40	2,69E-01
ENSRNOG00000045789	**Zfp192**	zinc finger protein 192 (Zfp192), mRN	306974	17	58372076	58390621	18546	306,54	-0,55	2,69E-01
ENSRNOG00000012409	**Exosc3**	exosome component 3 (Exosc3), mRN	313243	5	65377252	65380455	3204	225,68	0,52	2,69E-01
ENSRNOG00000015664	**Tmem8b**	Protein Tmem8b [Source:UniProtKB/T	313490	5	63703638	63716765	13128	700,42	0,48	2,69E-01
ENSRNOG00000025372	**Glce**	Protein Glce; RCG57892 [Source:Uni	363073	8	66744887	66757177	12291	402,05	-0,44	2,69E-01
ENSRNOG00000047287	**Nucks1**	nuclear casein kinase and cyclin-depe	64709	13	53753077	53778581	25505	1105,77	-0,32	2,69E-01
ENSRNOG00000010875	**LOC69193**	RIKEN cDNA 5730508B09 gene [Sour	691931	2	251559192	251589087	29896	60,49	-0,66	2,70E-01
ENSRNOG00000020930	**Atxn7l3**	Protein Atxn7l3 [Source:UniProtKB/Tr	287734	10	90023643	90026969	3327	2218,84	0,45	2,70E-01
ENSRNOG00000043023	**LOC10036**	microseminoprotein, prostate associat	1E+08	5	63609317	63610270	954	204,37	0,53	2,70E-01
ENSRNOG00000003643	**Tab3**	Protein LOC317546 [Source:UniProtK	317546	X	54264950	54287819	22870	165,89	-0,59	2,70E-01
ENSRNOG00000008019	**Oxr1**	oxidation resistance 1 (Oxr1), transcri	117520	7	80737304	80812054	74751	1258,12	-0,59	2,70E-01
ENSRNOG00000022946	**Slc22a3**	solute carrier family 22 (organic cation	29504	1	51234472	51321364	86893	123,81	-0,78	2,70E-01
ENSRNOG00000039388	**Prrt4**	proline-rich transmembrane protein 4 (	500059	4	56209884	56220344	10461	339,71	-0,36	2,70E-01
ENSRNOG00000007083	**Gpn2**	GPN-loop GTPase 2 (Gpn2), mRNA [S	362614	5	155495838	155503005	7168	363,50	0,45	2,71E-01
ENSRNOG00000010171	**Elk1**	ELK1, member of ETS oncogene fami	314436	X	2112120	2119843	7724	969,08	-0,42	2,71E-01
ENSRNOG00000015956	**Usp29**	ubiquitin specific peptidase 29 (Usp29	361495	1	71382517	71397426	14910	1903,15	-0,48	2,71E-01
ENSRNOG00000021384	**Ankrd44**	ankyrin repeat domain 44 (Ankrd44), r	301415	9	60910883	61210388	299506	760,50	-0,46	2,71E-01
ENSRNOG00000042274		F-box protein 31 (Fbxo31), mRNA [So	498959	19	64224824	64363108	138285	1509,08	0,49	2,71E-01
ENSRNOG00000022267	**Pabpc1l2a**	Protein Pabpc1l2b [Source:UniProtKE	302405	X	74699763	74700365	603	261,47	-0,44	2,71E-01
ENSRNOG00000022472	**Snap47**	synaptosomal-associated protein, 47 (	303183	10	45512878	45554396	41519	860,66	0,49	2,71E-01
ENSRNOG00000000161	**Chm**	choroideremia (Rab escort protein 1) (	24942	X	84610339	84767421	157083	228,16	-0,70	2,71E-01
ENSRNOG00000002069	**Slc35a5**	solute carrier family 35, member A5 (S	498081	11	64716028	64736221	20194	697,63	-0,64	2,71E-01
ENSRNOG00000008924	**Arhgef12**	Rho guanine nucleotide exchange fac	367072	8	45745243	45863838	118596	2671,79	-0,30	2,71E-01
ENSRNOG00000013092	**Lonrf3**	LON peptidase N-terminal domain anc	298322	X	123088023	123121296	33274	413,20	-0,66	2,71E-01
ENSRNOG00000036696	**Hgs**	hepatocyte growth factor-regulated tyr	56084	10	109232696	109250719	18024	3734,94	0,30	2,71E-01
ENSRNOG00000018126	**Abca1**	ATP-binding cassette, subfamily A (AE	313210	5	74023652	74112802	89151	4040,55	-0,81	2,71E-01
ENSRNOG00000014635	**Clta**	clathrin, light chain A (Clta), mRNA [Sc	83800	5	64013646	64031649	18004	8371,52	0,43	2,72E-01
ENSRNOG00000016302	**Cnnm1**	cyclin M1 (Cnnm1), mRNA [Source:Re	309387	1	270631411	270687644	56234	558,93	0,62	2,72E-01
ENSRNOG00000019630	**Hacl1**	2-hydroxyacyl-CoA lyase 1 (Hacl1), m	85255	16	7646580	7682624	36045	415,99	-0,44	2,72E-01
ENSRNOG00000043065	**Prpf40b**	PRP40 pre-mRNA processing factor 4	363000	X	115471636	115485028	13393	1016,33	-0,36	2,72E-01
ENSRNOG00000014690	**Pigt**	phosphatidylinositol glycan anchor bio	296360	3	167125865	167135023	9159	3708,16	0,29	2,72E-01
ENSRNOG00000027606	**Neurl1b**	neuralized homolog 1B (Drosophila) (N	303019	10	16945337	16972071	26735	365,81	-0,70	2,72E-01
ENSRNOG00000017272	**Mrps27**	mitochondrial ribosomal protein S27 (N	361883	2	48760336	48828144	67809	623,18	0,37	2,73E-01
ENSRNOG00000014310	**Tmtc4**	transmembrane and tetratricopeptide r	290501	15	112720474	112776171	55698	748,55	-0,52	2,74E-01

ENSRNOG00000001628	**Pcp4**	Purkinje cell protein 4 (Pcp4), transcrip	25510	11	40377162	40438069	60908	479,24	-0,79	2,74E-01
ENSRNOG00000018128	**Unc50**	unc-50 homolog (C. elegans) (Unc50)	192356	9	43726249	43734121	7873	1255,97	0,33	2,75E-01
ENSRNOG00000009439	**Eef1a1**	eukaryotic translation elongation facto	171361	8	85388157	85390106	1950	10695,20	0,46	2,75E-01
ENSRNOG00000013064		Protein Lrp8 [Source:UniProtKB/TrEM	362558	5	131252608	131320255	67648	1444,08	-0,42	2,75E-01
ENSRNOG00000013845	**Mrpl21**	mitochondrial ribosomal protein L21 (M	309140	1	225399844	225408322	8479	461,80	0,42	2,75E-01
ENSRNOG00000020414	**Axin1**	axin 1 (Axin1), mRNA [Source:RefSeq	79257	10	15324330	15376252	51923	1339,50	0,32	2,75E-01
ENSRNOG00000037430	**Chfr**	checkpoint with forkhead and ring fing	288734	12	54299049	54332973	33925	1218,25	-0,64	2,75E-01
ENSRNOG00000006483	**Heatr5a**	Protein Heatr5a [Source:UniProtKB/T	362737	6	82211313	82299967	88655	553,07	-0,41	2,76E-01
ENSRNOG00000020013	**Psrc1**	proline/serine-rich coiled-coil 1 (Psrc1)	691380	2	230646557	230650999	4443	233,23	0,48	2,76E-01
ENSRNOG00000027374		RIKEN cDNA 9330101J02 gene [Source:MGI Sy		1	218907327	218944361	37035	30,24	-0,84	2,76E-01
ENSRNOG00000022071	**Itga2b**	Protein Itga2b [Source:UniProtKB/TrE	685269	10	90186663	90203600	16938	26,23	-0,81	2,76E-01
ENSRNOG00000004670	**Ddx56**	DEAD (Asp-Glu-Ala-Asp) box helicase	289780	14	87340632	87348794	8163	857,91	0,43	2,77E-01
ENSRNOG00000005483	**Rpap1**	RNA polymerase II associated protein	311338	3	118019055	118135613	116559	896,89	0,34	2,77E-01
ENSRNOG00000014367	**Ephb6**	Eph receptor B6 (Ephb6), mRNA [Sou	312275	4	135690323	135705579	15257	1361,10	-0,51	2,77E-01
ENSRNOG00000014522	**Mlycd**	malonyl-CoA decarboxylase (Mlycd), r	85239	19	62781487	62797312	15826	1174,78	0,40	2,77E-01
ENSRNOG00000019635	**Cops8**	COP9 signalosome subunit 8 (Cops8)	363283	9	97453701	97463536	9836	2778,63	0,53	2,77E-01
ENSRNOG00000020376	**Obfc1**	oligonucleotide/oligosaccharide-bindin	294025	1	274714591	274748404	33814	410,65	0,51	2,77E-01
ENSRNOG00000027623	**LOC68003**	DNA segment, Chr 5, ERATO Doi 579	680039	14	78899825	78964642	64818	2922,88	-0,33	2,77E-01
ENSRNOG00000000281	**Prodh**	proline dehydrogenase (oxidase) 1 (Pr	680409	11	90150469	90167638	17170	466,09	-0,75	2,77E-01
ENSRNOG00000010816	**Kcnk15**	potassium channel, subfamily K, mem	156873	3	166421477	166427695	6219	229,62	-0,48	2,78E-01
ENSRNOG00000011409	**Hebp2**	heme binding protein 2 (Hebp2), mRN	308632	1	15398929	15404431	5503	41,17	0,64	2,78E-01
ENSRNOG00000015075	**Stc1**	stanniocalcin 1 (Stc1), mRNA [Source	81801	15	54622230	54632873	10644	102,05	-0,58	2,78E-01
ENSRNOG00000016309	**Rgp1**	Protein Rgp1 [Source:UniProtKB/TrEI	313493	5	63605359	63613117	7759	486,64	0,49	2,78E-01
ENSRNOG00000006110	**Jph1**	junctophilin 1 (Jph1), mRNA [Source:F	297748	5	1413721	1507659	93939	65,90	-0,65	2,78E-01
ENSRNOG00000018938	**Mkx**	Protein Mkx [Source:UniProtKB/TrEM	291228	17	62322717	62332383	9667	35,08	0,68	2,78E-01
ENSRNOG00000007436	**LOC10015**	RIKEN cDNA 1600002H07 gene [Sou	1E+08	10	13402892	13407969	5078	350,85	0,35	2,78E-01
ENSRNOG00000029449	**Ror1**	receptor tyrosine kinase-like orphan re	362550	5	122797878	123141864	343987	20,66	-0,77	2,78E-01
ENSRNOG00000027066	**Stk32a**	serine/threonine kinase 32A (Stk32a),	364858	18	37253265	37362844	109580	43,41	-0,84	2,78E-01
ENSRNOG00000000934	**Piwil1**	piwi-like RNA-mediated gene silencing	363912	12	33532887	33553715	20829	17,75	0,79	2,79E-01
ENSRNOG00000007088	**Xpot**	exportin, tRNA (Xpot), mRNA [Source:	314879	7	63912373	63951227	38855	2540,99	-0,30	2,79E-01
ENSRNOG00000013423	**Npap60**	nuclear pore associated protein (Npap	25497	7	125551331	125564950	13620	1604,87	0,45	2,79E-01
ENSRNOG00000024266		HAUS augmin-like complex, subunit 5	1E+08	1	90268937	90279586	10650	343,67	0,46	2,79E-01
ENSRNOG00000017550	**Fbln7**	Protein Fbln7 [Source:UniProtKB/TrE	296145	3	128571581	128604148	32568	19,12	-0,76	2,79E-01
ENSRNOG00000021911	**Camlg**	calcium modulating ligand (Camlg), m	81715	17	11631232	11642058	10827	1673,58	0,34	2,79E-01
ENSRNOG00000023148		collagen, type XI, alpha 1 (Col11a1), n	25654	2	234955506	235148332	192827	428,78	-0,60	2,79E-01
ENSRNOG00000036644	**Tnfrsf22**	Protein Tnfrsf22 [Source:UniProtKB/TrEMBL;Ac		1	223705814	223714178	8365	21,56	0,82	2,79E-01
ENSRNOG00000006197		calumenin (Calu), transcript variant 1,	64366	4	56392772	56420272	27501	4708,68	-0,38	2,79E-01
ENSRNOG00000003955	**Spata7**	spermatogenesis associated 7 (Spata	192225	6	131817567	131862859	45293	292,82	-0,37	2,80E-01

ENSRNOG00000016584	**Mamdc4**	MAM domain containing 4 (Mamdc4),	252882	3	2767936	2776522	8587	144,58	0,58	2,80E-01
ENSRNOG00000029682	**Clic1**	chloride intracellular channel 1 (Clic1),	406864	20	7113249	7122091	8843	674,56	0,60	2,80E-01
ENSRNOG00000000495	**Uhrf1bp1**	Protein Uhrf1bp1 [Source:UniProtKB/	309637	20	9633546	9673740	40195	1019,90	0,32	2,81E-01
ENSRNOG00000010880	**Gpr27**	G protein-coupled receptor 27 (Gpr27)	65275	4	196629031	196630164	1134	537,75	0,51	2,81E-01
ENSRNOG00000017460	**Imp3**	IMP3, U3 small nucleolar ribonucleopr	315697	8	60176548	60177436	889	1022,52	0,43	2,81E-01
ENSRNOG00000029500	**Tapbp**	TAP binding protein (tapasin) (Tapbp),	25217	20	7526668	7534619	7952	358,28	0,63	2,81E-01
ENSRNOG00000030759	**Nhs**	Nance-Horan syndrome (congenital ca	317494	X	34966098	35015678	49581	106,15	-0,51	2,81E-01
ENSRNOG00000031769	**Chchd7**	Protein LOC684258; RCG30521, isofo	684258	5	21622037	21624182	2146	191,57	0,46	2,81E-01
ENSRNOG00000014768		Protein RGD1565505 [Source:UniPro	680671	1	253622233	254026042	403810	241,33	-0,70	2,81E-01
ENSRNOG00000012989	**Serinc2**	serine incorporator 2 (Serinc2), mRNA	313057	5	152187838	152210009	22172	319,85	0,57	2,82E-01
ENSRNOG00000012098		adenylate cyclase activating polypepti	24167	4	150314846	150360099	45254	7559,19	-0,50	2,82E-01
ENSRNOG00000013408	**Npas2**	neuronal PAS domain protein 2 (Npas	316351	9	45590021	45769792	179772	381,05	-0,64	2,82E-01
ENSRNOG00000014660	**Usp53**	ubiquitin specific peptidase 53 (Usp53	295425	2	246460175	246521759	61585	310,31	-0,61	2,82E-01
ENSRNOG00000005482	**Sap30bp**	SAP30 binding protein (Sap30bp), mR	360662	10	103769674	103802319	32646	2126,21	0,51	2,83E-01
ENSRNOG00000010064		multidrug resistance-associated protei	170924	15	107127439	107356997	229559	695,20	-0,75	2,83E-01
ENSRNOG00000013704	**Cps1**	carbamoyl-phosphate synthetase 1 (C	497840	9	73072857	73183868	111012	50,25	0,66	2,83E-01
ENSRNOG00000028206	**Fam109a**	Sesquipedalian-1 [Source:UniProtKB/	288664	12	42097550	42098254	705	396,13	0,47	2,83E-01
ENSRNOG00000045782	**LOC10091**	Protein LOC100910991 [Source:UniP	1E+08	20	18950944	19004486	53543	35,73	-0,84	2,83E-01
ENSRNOG00000031033		NADH dehydrogenase subunit 2 (mito	26194	MT	3904	4942	1039	68105,13	-0,57	2,83E-01
ENSRNOG00000015329	**Kpna2**	karyopherin alpha 2 (Kpna2), transcrip	85245	10	94940857	94952889	12033	282,58	0,62	2,83E-01
ENSRNOG00000018745	**Sgsm3**	small G protein signaling modulator 3	362963	7	122187869	122217328	29460	1653,57	0,38	2,83E-01
ENSRNOG00000001662		Dual specificity tyrosine-phosphorylati	25255	11	38457362	38548315	90954	2076,62	-0,36	2,84E-01
ENSRNOG00000004515	**Pes1**	pescadillo ribosomal biogenesis factor	289740	14	84895325	84911703	16379	2491,06	0,34	2,84E-01
ENSRNOG00000010668	**Anxa6**	annexin A6 (Anxa6), mRNA [Source:R	79125	10	40092956	40129924	36969	1807,81	0,40	2,84E-01
ENSRNOG00000017839	**Ercc1**	excision repair cross-complementing r	292673	1	81523234	81534459	11226	749,76	0,42	2,84E-01
ENSRNOG00000021318	**Epas1**	endothelial PAS domain protein 1 (Epa	29452	6	20299621	20377837	78217	3455,88	-0,66	2,84E-01
ENSRNOG00000023020	**Fdx1l**	ferredoxin 1-like (Fdx1l), mRNA [Sourc	313786	8	22143206	22148122	4917	740,62	0,55	2,84E-01
ENSRNOG00000025787	**Spag6**	sperm associated antigen 6 (Spag6), mRNA [So		11	91480026	91563635	83610	51,97	-0,79	2,84E-01
ENSRNOG00000032437	**Pard3**	par-3 (partitioning defective 3) homolo	81918	19	70690939	71248880	557942	1125,70	0,36	2,84E-01
ENSRNOG00000046799	**Phb**	prohibitin (Phb), mRNA [Source:RefSe	25344	10	83284046	83296819	12774	979,75	0,34	2,84E-01
ENSRNOG00000000665	**Pitpnb**	phosphatidylinositol transfer protein, b	114561	12	53020772	53079031	58260	2444,69	-0,37	2,84E-01
ENSRNOG00000014568	**Ndufb10**	NADH dehydrogenase (ubiquinone) 1	681418	10	13905856	13908017	2162	1648,34	0,38	2,84E-01
ENSRNOG00000006867		ets variant 1 (Etv1), transcript variant 2	362733	6	68061144	68152515	91372	1481,89	-0,38	2,84E-01
ENSRNOG00000013000	**Ldhb**	lactate dehydrogenase B (Ldhb), mRN	24534	4	240918028	240936001	17974	10693,50	0,44	2,84E-01
ENSRNOG00000007591	**Slc45a3**	solute carrier family 45, member 3 (Slc	304785	13	53816774	53837091	20318	21,26	0,71	2,84E-01
ENSRNOG00000014369	**Slc27a4**	solute carrier family 27 (fatty acid trans	311839	3	13707126	13720047	12922	3032,10	0,48	2,84E-01
ENSRNOG00000000996	**Arpc1a**	actin related protein 2/3 complex, subu	81824	12	13346433	13363473	17041	4349,23	0,45	2,84E-01
ENSRNOG00000006227	**Ifih1**	interferon induced with helicase C don	499801	3	55223298	55269581	46284	135,35	0,74	2,85E-01

ENSRNOG00000009351	**Srp68**	signal recognition particle 68 (Srp68),	363707	10	104791135	104818559	27425	1370,69	0,38	2,85E-01
ENSRNOG00000007147	**Cyp46a1**	cytochrome P450, family 46, subfamily	362782	6	141416875	141443679	26805	2005,44	-0,43	2,85E-01
ENSRNOG00000019702	**Med29**	mediator complex subunit 29 (Med29)	292751	1	86583618	86589021	5404	878,58	0,47	2,85E-01
ENSRNOG00000017795	**Hspbp1**	HSPA binding protein, cytoplasmic coc	246146	1	75779793	75803081	23289	3410,02	0,47	2,85E-01
ENSRNOG00000010840	**Adamtsl3**	ADAMTS-like 3 (Adamtsl3), mRNA [Sc	308787	1	145170122	145490043	319922	67,97	-0,52	2,86E-01
ENSRNOG00000017233	**Mmachc**	methylmalonic aciduria (cobalamin de	313520	5	139351762	139357898	6137	287,71	0,43	2,86E-01
ENSRNOG00000017741	**Nudt5**	nudix (nucleoside diphosphate linked i	361274	17	78043454	78066592	23139	506,34	0,44	2,86E-01
ENSRNOG00000031965	**Impdh2**	IMP (inosine 5'-monophosphate) dehy	301005	8	116691532	116696120	4589	3041,00	0,39	2,86E-01
ENSRNOG00000049997		serine/threonine-protein phosphatase	500711	6	136618491	136715054	96564	60,69	-0,57	2,86E-01
ENSRNOG00000020388	**Inpp5f**	inositol polyphosphate-5-phosphatase	309008	1	207085286	207164061	78776	1191,44	-0,35	2,86E-01
ENSRNOG00000009163	**Fam133b**	family with sequence similarity 133, m	362320	4	27632197	27658496	26300	380,82	-0,59	2,86E-01
ENSRNOG00000000662		SRR1 domain containing [Source:MGI	288717	12	52073011	52078001	4991	276,62	0,51	2,86E-01
ENSRNOG00000007956	**Styx**	serine/threonine/tyrosine interacting pi	1E+08	15	23654558	23681602	27045	35,54	-0,61	2,86E-01
ENSRNOG00000021276	**Bmp2**	bone morphogenetic protein 2 (Bmp2)	29373	3	132822229	132830717	8489	29,78	-0,75	2,86E-01
ENSRNOG00000028292	**Gart**	phosphoribosylglycinamide formyltran:	288259	11	35389226	35414461	25236	2870,39	0,48	2,86E-01
ENSRNOG00000032930	**Trim26**	tripartite motif-containing 26 (Trim26),	309586	20	4239932	4248757	8826	1031,99	0,50	2,86E-01
ENSRNOG00000048800		Glucocorticoid receptor [Source:UniPi	24413	18	32327621	32348467	20847	193,36	-0,60	2,87E-01
ENSRNOG00000021198	**Hist1h2bq**	histone cluster 1, H2bh (Hist1h2bh), m	64647	17	45534129	45542936	8808	1536,03	0,58	2,87E-01
ENSRNOG00000042878	**Wdr74**	WD repeat domain 74 (Wdr74), mRNA	690229	1	231871409	231876109	4701	964,78	0,45	2,87E-01
ENSRNOG00000000048	**Gak**	cyclin G associated kinase (Gak), mRI	81659	14	2095648	2168937	73290	2634,94	0,35	2,87E-01
ENSRNOG00000000560	**Lrrc20**	leucine rich repeat containing 20 (Lrrc	499430	20	32881662	32985238	103577	502,20	0,38	2,87E-01
ENSRNOG00000002705	**Vps4b**	vacuolar protein sorting 4 homolog B (	360834	13	31975737	32000936	25200	1011,34	0,36	2,87E-01
ENSRNOG00000003310	**Tmem63a**	transmembrane protein 63a (Tmem63	289318	13	104218357	104250973	32617	56,53	0,76	2,87E-01
ENSRNOG00000003349	**LOC10091**	collagen, type XXIII, alpha 1 (Col23a1	353303	10	36513061	36800649	287589	21,25	-0,77	2,87E-01
ENSRNOG00000005472	**Sp4**	Sp4 transcription factor (Sp4), mRNA	25162	6	155047728	155108173	60446	533,14	-0,32	2,87E-01
ENSRNOG00000006384	**Ddx58**	DEAD (Asp-Glu-Ala-Asp) box polypep	297989	5	61054910	61080662	25753	250,68	0,78	2,87E-01
ENSRNOG00000007590		eyes absent 1 homolog (Drosophila) [Source:MC		5	4349987	4485882	135896	52,08	-0,67	2,87E-01
ENSRNOG00000007756		similar to RIKEN cDNA 9430023L20 (I	300240	7	140730296	140739408	9113	1464,42	0,35	2,87E-01
ENSRNOG00000008625		Protein Rimkla; RCG50366 [Source:UniProtKB/		5	142327166	142350279	23114	210,06	-0,58	2,87E-01
ENSRNOG00000009324	**Wdr54**	WD repeat domain 54 (Wdr54), mRNA	500226	4	179436792	179443385	6594	1429,81	0,44	2,87E-01
ENSRNOG00000012460	**Cntf**	ciliary neurotrophic factor (Cntf), mRN	25707	1	236752366	236754389	2024	94,81	-0,71	2,87E-01
ENSRNOG00000014626	**Katnb1**	katanin p80 (WD repeat containing) su	291852	19	10320410	10338205	17796	1520,26	0,32	2,87E-01
ENSRNOG00000019459	**Oaz1**	ornithine decarboxylase antizyme 1 (C	25502	7	11919843	11922302	2460	9148,66	0,49	2,87E-01
ENSRNOG00000026791	**Pgbd5**	piggyBac transposable element derive	292098	19	67835422	67900208	64787	839,12	-0,53	2,87E-01
ENSRNOG00000048113	**Cbx8**	chromobox homolog 8 (Cbx8), mRNA	303731	10	107762003	107764441	2439	537,09	0,38	2,87E-01
ENSRNOG00000048513		Uncharacterized protein [Source:UniProtKB/TrE		11	15184325	15252957	68633	185,93	0,57	2,87E-01
ENSRNOG00000036658	**Tbcd**	tubulin folding cofactor D (Tbcd), mRN	1E+08	10	110229340	110380958	151619	3519,26	0,43	2,87E-01
ENSRNOG00000031053		NADH dehydrogenase subunit 4L (mit	26200	MT	9870	10166	297	11947,97	-0,52	2,88E-01

ENSRNOG00000024712	**Insc**	inscuteable homolog (Drosophila) (Ins	293166	1	191272625	191380855	108231	18,44	0,76	2,88E-01
ENSRNOG00000031730	**LOC68076**	transcription elongation factor B (SIII),	64525	5	2052950	2058835	5886	154,91	-0,54	2,88E-01
ENSRNOG00000010389		NDRG family member 2 (Ndrg2), trans	171114	15	32118800	32127444	8645	9968,45	-0,69	2,88E-01
ENSRNOG00000030157	**Dhx16**	DEAH (Asp-Glu-Ala-His) box polypept	294232	20	5466494	5483190	16697	1191,76	0,33	2,88E-01
ENSRNOG00000030483	**Rabggta**	Rab geranylgeranyltransferase, alpha	58983	15	38284054	38290398	6345	784,24	0,32	2,88E-01
ENSRNOG00000049683	**Sh3gl1**	SH3-domain GRB2-like 1 (Sh3gl1), ml	81922	9	10018263	10040574	22312	2768,07	0,29	2,88E-01
ENSRNOG00000029195	**Uba7**	ubiquitin-like modifier activating enzym	301000	8	116108502	116117308	8807	76,17	0,81	2,89E-01
ENSRNOG00000046918	**Apoo**	apolipoprotein O (Apoo), mRNA [Sour	363474	X	64430818	64534955	104138	317,86	-0,50	2,89E-01
ENSRNOG00000006931	**Eepd1**	endonuclease/exonuclease/phosphata	315500	8	26673119	26779323	106205	1295,08	-0,51	2,89E-01
ENSRNOG00000009166	**LOC10090**	DEAD (Asp-Glu-Ala-Asp) box helicase	373065	6	136522732	136539893	17162	2459,69	0,46	2,89E-01
ENSRNOG00000019180	**Acsl4**	acyl-CoA synthetase long-chain family	113976	X	112046207	112109823	63617	2327,85	-0,61	2,90E-01
ENSRNOG00000015236	**Mybbp1a**	MYB binding protein (P160) 1a (Mybb	60571	10	58739483	58749744	10262	3102,17	0,30	2,90E-01
ENSRNOG00000010458		phosphatidylinositol-4,5-bisphosphate	170911	2	138491967	138521470	29504	556,89	-0,44	2,90E-01
ENSRNOG00000017467	**Rab4a**	RAB4A, member RAS oncogene famil	25532	19	67296402	67323422	27021	1306,16	0,41	2,90E-01
ENSRNOG00000046633		Uncharacterized protein [Source:UniProtKB/TrE		1	268523263	268526180	2918	124,30	0,55	2,90E-01
ENSRNOG00000002935	**Ankrd40**	ankyrin repeat domain 40 (Ankrd40), r	690586	10	81865469	81878114	12646	1238,03	0,33	2,91E-01
ENSRNOG00000008040	**Fam64a**	family with sequence similarity 64, me	360559	10	58354690	58359530	4841	280,72	0,66	2,91E-01
ENSRNOG00000017037	**Otud3**	OTU domain containing 3 (Otud3), mF	500572	5	161086486	161108351	21866	220,07	0,51	2,91E-01
ENSRNOG00000009822	**Tlr2**	toll-like receptor 2 (Tlr2), mRNA [Sour	310553	2	202253257	202258683	5427	29,30	-0,83	2,91E-01
ENSRNOG00000009281	**Commd1**	copper metabolism (Murr1) domain co	289831	14	107724470	107835993	111524	1112,54	0,39	2,91E-01
ENSRNOG00000033133	**Nae1**	NEDD8 activating enzyme E1 subunit	84019	19	646856	673674	26819	648,99	-0,55	2,91E-01
ENSRNOG00000004752		sodium leak channel non-selective prc	266760	15	113117194	113455059	337866	581,99	-0,45	2,91E-01
ENSRNOG00000009080	**Atp6v1d**	ATPase, H+ transporting, lysosomal V	299159	6	111407090	111421805	14716	3952,07	0,43	2,91E-01
ENSRNOG00000022660	**Ccdc73**	Protein Ccdc73 [Source:UniProtKB/Tr	499848	3	101493266	101614072	120807	19,68	-0,71	2,91E-01
ENSRNOG00000025864	**Lemd2**	LEM domain containing 2 (Lemd2), ml	361807	20	8789657	8800115	10459	1911,81	0,44	2,91E-01
ENSRNOG00000009090	**Dad1**	defender against cell death 1 (Dad1),	192275	15	36679092	36699071	19980	3642,62	0,54	2,91E-01
ENSRNOG00000049849		family with sequence similarity 199, X-linked [So		X	107574114	107601860	27747	57,28	-0,70	2,91E-01
ENSRNOG00000006858	**Pigy**	phosphatidylinositol glycan anchor bio	502782	4	153407117	153409393	2277	209,58	-0,55	2,91E-01
ENSRNOG00000006209	**Mbd6**	methyl-CpG binding domain protein 6	362892	7	70745585	70751297	5713	743,50	0,44	2,92E-01
ENSRNOG00000006943	**Zcchc10**	zinc finger, CCHC domain containing	360524	10	38445574	38455988	10415	116,70	-0,46	2,92E-01
ENSRNOG00000025860	**Ccdc135**	coiled-coil domain containing 135 (Cc	291853	19	10342364	10360068	17705	28,34	-0,73	2,92E-01
ENSRNOG00000017286	**Ephx2**	epoxide hydrolase 2, cytoplasmic (Epl	65030	15	48799895	48836847	36953	556,51	-0,69	2,92E-01
ENSRNOG00000003098	**Prom1**	prominin 1 (Prom1), transcript variant	60357	14	71563324	71668091	104768	239,37	-0,65	2,93E-01
ENSRNOG00000003779	**Dedd**	death effector domain-containing (Dec	83631	13	94345399	94348787	3389	639,28	0,37	2,93E-01
ENSRNOG00000011858	**Unc5d**	unc-5 homolog D (C. elegans) (Unc5d	306534	16	66680943	67223786	542844	466,31	-0,49	2,93E-01
ENSRNOG00000013738	**LOC10017**	RIKEN cDNA C330018D20 gene [Sou	1E+08	18	51225484	51241458	15975	199,69	-0,48	2,93E-01
ENSRNOG00000029608		Uncharacterized protein [Source:UniProtKB/TrE		1	29390335	29610546	220212	265,11	-0,50	2,93E-01
ENSRNOG00000033722		ring finger protein 207 [Source:MGI Sy	691246	5	173040220	173052658	12439	23,57	-0,77	2,93E-01

ENSRNOG00000036604	**Ifit2**	interferon-induced protein with tetratric	294091	1	260116148	260122209	6062	24,22	0,83	2,93E-01
ENSRNOG00000001864	**Tmem191c**	Protein Tmem191c [Source:UniProtKI	680867	11	91030347	91032188	1842	69,02	0,53	2,93E-01
ENSRNOG00000002413	**Gpc4**	glypican 4 (Gpc4), mRNA [Source:Ref	317322	X	139402263	139512716	110454	1357,00	-0,53	2,93E-01
ENSRNOG00000017283		potassium channel, subfamily T, meml	60444	3	8673193	8725541	52349	155,44	-0,66	2,93E-01
ENSRNOG00000006305	**Slc38a2**	solute carrier family 38, member 2 (Sl	29642	7	137716131	137728300	12170	3827,14	-0,28	2,93E-01
ENSRNOG00000011457	**Lrrc26**	leucine rich repeat containing 26 (Lrrc	311803	3	2487839	2489165	1327	33,86	0,83	2,93E-01
ENSRNOG00000019168	**Nxt2**	nuclear transport factor 2-like export fa	315352	X	111960845	111967052	6208	120,52	-0,61	2,93E-01
ENSRNOG00000020431	**Matk**	megakaryocyte-associated tyrosine ki	60450	7	11492662	11497686	5025	613,08	-0,49	2,93E-01
ENSRNOG00000020652	**Tgfb1**	transforming growth factor, beta 1 (Tgf	59086	1	83742151	83758471	16321	28,01	-0,76	2,93E-01
ENSRNOG00000030628	**LOC50065**	eukaryotic translation initiation factor 4	287436	10	56016895	56022402	5508	9180,83	0,46	2,93E-01
ENSRNOG00000037327	**F8a1**	coagulation factor VIII-associated 1 (F	501661	1	153316591	153318105	1515	1929,04	0,57	2,93E-01
ENSRNOG00000005758		BTB (POZ) domain containing 11 [Source:MGI S		7	24194086	24465369	271284	201,65	-0,58	2,93E-01
ENSRNOG00000002053		Fraser syndrome 1 homolog (human) [Source:M		14	14413367	14794704	381338	324,86	-0,43	2,93E-01
ENSRNOG00000002258	**Tmem150c**	transmembrane protein 150C (Tmem1	360916	14	11093499	11110144	16646	799,99	-0,58	2,93E-01
ENSRNOG00000005031	**Surf6**	surfeit 6 (Surf6), mRNA [Source:RefSe	303076	3	10803319	10814066	10748	510,96	0,43	2,93E-01
ENSRNOG00000008757	**Tmem218**	transmembrane protein 218 (Tmem21	300516	8	39687930	39703272	15343	671,68	0,58	2,93E-01
ENSRNOG00000008966	**Slco5a1**	solute carrier organic anion transporte	312907	5	10704801	10824591	119791	250,22	0,45	2,93E-01
ENSRNOG00000009084	**Rpusd3**	RNA pseudouridylate synthase domai	362416	4	208663811	208668038	4228	288,02	0,41	2,93E-01
ENSRNOG00000009766	**Gpr180**	G protein-coupled receptor 180 (Gpr1	306165	15	106783002	106806560	23559	714,59	0,40	2,93E-01
ENSRNOG00000010277	**Idh3a**	isocitrate dehydrogenase 3 (NAD+) al	114096	8	57745463	57764540	19078	2844,33	-0,32	2,93E-01
ENSRNOG00000011020	**Eif3l**	eukaryotic translation initiation factor 3	300069	7	120314744	120325674	10931	2615,59	0,48	2,93E-01
ENSRNOG00000011705	**Stmn2**	stathmin-like 2 (Stmn2), mRNA [Sourc	84510	2	115167506	115215213	47708	35241,81	-0,50	2,93E-01
ENSRNOG00000011789	**Cdon**	cell adhesion associated, oncogene re	50938	8	36673132	36725976	52845	480,91	-0,43	2,93E-01
ENSRNOG00000011952	**Samm50**	sorting and assembly machinery comp	300111	7	125047478	125071017	23540	2274,34	0,35	2,93E-01
ENSRNOG00000018985	**Adrbk1**	adrenergic, beta, receptor kinase 1 (A	25238	1	226406333	226414442	8110	2240,79	-0,46	2,93E-01
ENSRNOG00000020056	**Fam134c**	family with sequence similarity 134, m	360632	10	88805098	88829208	24111	2791,17	0,55	2,93E-01
ENSRNOG00000023334	**Parp14**	poly (ADP-ribose) polymerase family,	303903	11	71194913	71226980	32068	321,80	0,73	2,93E-01
ENSRNOG00000038976	**Galntl6**	UDP-N-acetyl-alpha-D-galactosamine	361142	16	35390465	35619230	228766	77,05	-0,65	2,93E-01
ENSRNOG00000004494	**Lta4h**	leukotriene A4 hydrolase (Lta4h), mRN	299732	7	34353506	34385290	31785	1153,62	0,44	2,93E-01
ENSRNOG00000002943	**Mrpl55**	mitochondrial ribosomal protein L55 (M	287356	10	45308880	45312028	3149	1048,68	0,49	2,94E-01
ENSRNOG00000007221	**Dut**	deoxyuridine triphosphatase (Dut), tra	497778	3	124038162	124049164	11003	478,29	0,42	2,94E-01
ENSRNOG00000036859	**Gdpd5**	Protein Gdpd5; Putative uncharacteriz	499211	1	170582634	170619908	37275	1044,12	0,48	2,94E-01
ENSRNOG00000001103		Protein Sdk1 [Source:UniProtKB/TrEM	304297	12	16473809	16728950	255142	320,89	-0,49	2,94E-01
ENSRNOG00000001168	**Rsph1**	radial spoke head 1 homolog (Chlamy	361818	20	12177328	12195013	17686	121,60	-0,55	2,94E-01
ENSRNOG00000028919	**Zfp868**	zinc finger protein 868 (Zfp868), mRN	494340	16	21432872	21438285	5414	180,02	-0,50	2,94E-01
ENSRNOG00000000459	**Psmb9**	proteasome (prosome, macropain) sul	24967	20	6052972	6058393	5422	399,53	0,74	2,94E-01
ENSRNOG00000009175	**Jagn1**	jagunal homolog 1 (Drosophila) (Jagn	502872	4	208697312	208701997	4686	1513,24	0,38	2,94E-01
ENSRNOG00000011979	**Mrps24**	mitochondrial ribosomal protein S24 (M	498406	14	86640006	86644150	4145	1566,99	0,60	2,94E-01

ENSRNOG00000047132	**Zfp182**	Protein LOC100911831 [Source:UniP	1E+08	X	979734	994442	14709	78,75	-0,52	2,94E-01
ENSRNOG00000009585	**Tcf20**	transcription factor 20 (Tcf20), mRNA [	366964	7	123655990	123753836	97847	3448,55	-0,40	2,94E-01
ENSRNOG00000003338	**Pmp22**	peripheral myelin protein 22 (Pmp22),	24660	10	49319543	49346994	27452	2573,27	0,53	2,95E-01
ENSRNOG00000020769	**Crebrf**	CREB3 regulatory factor (Crebrf), mRI	303016	10	16590829	16621974	31146	116,76	-0,68	2,95E-01
ENSRNOG00000033372		kelch-like protein 24 [Source:RefSeq	303803	11	87677908	87705842	27935	1256,93	-0,52	2,95E-01
ENSRNOG00000026260	**Rpl11**	ribosomal protein L11 (Rpl11), mRNA	362631	5	158155378	158158916	3539	5623,43	0,48	2,95E-01
ENSRNOG00000008898	**Trmt44**	tRNA methyltransferase 44 homolog (S	305443	14	79987434	80004327	16894	228,98	0,50	2,96E-01
ENSRNOG00000008960	**Fgf22**	fibroblast growth factor 22 (Fgf22), mF	170579	7	12999989	13001879	1891	114,11	0,44	2,96E-01
ENSRNOG00000014165	**Ssr1**	signal sequence receptor, alpha (Ssr1	361233	17	29410635	29426436	15802	2623,83	-0,45	2,96E-01
ENSRNOG00000016488	**Pltp**	phospholipid transfer protein (Pltp), ml	296371	3	167489818	167507385	17568	562,64	-0,73	2,96E-01
ENSRNOG00000019914	**Fam57b**	family with sequence similarity 57, me	293493	1	205239664	205246007	6344	2288,85	0,48	2,96E-01
ENSRNOG00000001488	**Psmb1**	proteasome (prosome, macropain) sul	94198	1	58398991	58418276	19286	6219,75	0,40	2,96E-01
ENSRNOG00000016984		autophagy related 13 (Atg13), mRNA [	362164	3	87278370	87313656	35287	3252,93	0,49	2,96E-01
ENSRNOG00000006682	**Nav3**	neuron navigator 3 (Nav3), mRNA [So	314814	7	52215845	52510816	294972	1121,52	-0,36	2,96E-01
ENSRNOG00000047085	**Lrrtm2**	leucine rich repeat transmembrane ne	685472	18	27725459	27727003	1545	193,92	-0,64	2,96E-01
ENSRNOG00000001253	**Eif3b**	eukaryotic translation initiation factor 3	288516	12	18278837	18303294	24458	5390,80	0,41	2,98E-01
ENSRNOG00000007399	**Nudc**	nudC nuclear distribution protein (Nud	29648	5	155443982	155457375	13394	468,38	0,52	2,98E-01
ENSRNOG00000018241	**Ank1**	ankyrin 1, erythrocytic (Ank1), mRNA [	306570	16	73314002	73459822	145821	690,24	-0,35	2,98E-01
ENSRNOG00000032997				MT	14062	14130	69	75,10	-0,62	2,98E-01
ENSRNOG00000021230	**Ubox5**	U-box domain containing 5 (Ubox5), m	296161	3	129628377	129670003	41627	557,82	0,52	2,98E-01
ENSRNOG00000039668	**Col8a1**	collagen, type VIII, alpha 1 (Col8a1), r	304021	11	48073158	48201270	128113	35,42	-0,81	2,98E-01
ENSRNOG00000004642	**Lmx1a**	LIM homeobox transcription factor 1 a	289201	13	90560341	90699341	139001	30,80	-0,81	2,98E-01
ENSRNOG00000017953	**Gfod2**	glucose-fructose oxidoreductase doma	307801	19	48549438	48591884	42447	944,65	0,55	2,98E-01
ENSRNOG00000018334	**Ctu1**	cytosolic thiouridylase subunit 1 (Ctu1	292847	1	100668356	100674148	5793	760,16	0,44	2,98E-01
ENSRNOG00000018363	**RGD13098**	Protein RGD1309823 [Source:UniPro	313525	5	139661184	139661837	654	207,34	0,53	2,98E-01
ENSRNOG00000024104	**Mbd1**	methyl-CpG binding domain protein 1	291439	18	69390260	69404711	14452	1502,78	0,41	2,98E-01
ENSRNOG00000004252	**LOC10091**	eukaryotic translation initiation factor 3	299899	6	20146737	20231688	84952	318,17	0,46	2,98E-01
ENSRNOG00000010604		TAF15 RNA polymerase II, TATA box b	287571	10	70322902	70355481	32580	215,55	0,43	2,98E-01
ENSRNOG00000012683	**Abhd17c**	abhydrolase domain containing 17C (A	361601	1	147177238	147218200	40963	1401,76	-0,44	2,98E-01
ENSRNOG00000016023	**Kank1**	KN motif and ankyrin repeat domains	309429	1	250540559	250659565	119007	1047,61	-0,79	2,98E-01
ENSRNOG00000034221	**RGD15626**	similar to RIKEN cDNA 1700009P17 (	498278	13	94107559	94123450	15892	134,22	-0,52	2,98E-01
ENSRNOG00000017150	**Med10**	mediator complex subunit 10 (Med10)	290939	1	37504218	37509877	5660	1627,39	0,39	2,98E-01
ENSRNOG00000026833	**Ube4a**	ubiquitination factor E4A (Ube4a), mR	315608	8	47847869	47887561	39693	4129,20	0,45	2,98E-01
ENSRNOG00000028501	**Zc3h18**	zinc finger CCCH-type containing 18 (	292067	19	65917257	65952371	35115	1508,62	0,31	2,98E-01
ENSRNOG00000038881	**Hcls1**	hematopoietic cell specific Lyn substra	288077	11	69828261	69850286	22026	25,91	-0,74	2,99E-01
ENSRNOG00000019677	**Arid3b**	AT rich interactive domain 3B (Bright li	367092	8	62346554	62391111	44558	149,98	0,55	2,99E-01
ENSRNOG00000007839	**Slc16a7**	solute carrier family 16 (monocarboxyl	29735	7	68640932	68744670	103739	214,17	-0,72	2,99E-01
ENSRNOG00000017913	**Atg16l1**	autophagy related 16-like 1 (S. cerevis	363278	9	94599373	94634548	35176	1398,50	0,65	2,99E-01

ENSRNOG00000021082	**Kdelr1**	KDEL (Lys-Asp-Glu-Leu) endoplasmic	361577	1	102938310	102949196	10887	3046,89	0,36	2,99E-01
ENSRNOG00000030404	**Arrb1**	arrestin, beta 1 (Arrb1), mRNA [Sourc	25387	1	170706120	170772100	65981	1166,43	-0,35	2,99E-01
ENSRNOG00000034134	**Cpm**	carboxypeptidase M (Cpm), mRNA [S	314855	7	60654168	60714583	60416	67,65	-0,82	2,99E-01
ENSRNOG00000019161	**Cpeb1**	cytoplasmic polyadenylation element b	293056	1	144114219	144199174	84956	160,65	-0,68	3,00E-01
ENSRNOG00000004109	**Zfpm2**	zinc finger protein, multitype 2 (Zfpm2	314930	7	79657649	79976680	319032	342,55	-0,46	3,00E-01
ENSRNOG00000016580	**Rps23**	ribosomal protein S23 (Rps23), mRNA	124323	2	19699472	19701042	1571	1951,94	0,36	3,01E-01
ENSRNOG00000006370	**RGD13108**	similar to RIKEN cDNA 9130401M01 (	314992	7	98498309	98509170	10862	343,25	0,46	3,01E-01
ENSRNOG00000007427	**Entpd6**	ectonucleoside triphosphate diphosph	85260	3	152905477	152927857	22381	2008,99	0,44	3,01E-01
ENSRNOG00000029134		latrophilin 1 (Lphn1), mRNA [Source:R	65096	19	36021690	36047247	25558	10557,12	-0,48	3,01E-01
ENSRNOG00000031138	**Irgm**	immunity-related GTPase family, M (Ir	303090	10	33997566	34005503	7938	300,61	0,74	3,01E-01
ENSRNOG00000001494	**Napa**	N-ethylmaleimide-sensitive factor atta	140673	1	79260708	79279642	18935	3005,30	0,48	3,01E-01
ENSRNOG00000017022	**Cerk**	ceramide kinase (Cerk), mRNA [Sourc	300129	7	126726021	126768650	42630	2414,60	-0,27	3,01E-01
ENSRNOG00000046360	**Dact3**	dapper, antagonist of beta-catenin, ho	499088	1	80048066	80059888	11823	3261,37	0,54	3,01E-01
ENSRNOG00000000431	**Atf6b**	activating transcription factor 6 beta (A	406169	20	6493788	6501639	7852	2473,24	0,45	3,02E-01
ENSRNOG00000009236	**Necap1**	NECAP endocytosis associated 1 (Ne	312694	4	222730478	222745559	15082	5726,19	0,58	3,02E-01
ENSRNOG00000017416	**Ppic**	peptidylprolyl isomerase C (Ppic), mR	291463	18	47585436	47598040	12605	408,27	-0,68	3,02E-01
ENSRNOG00000023768	**Rundc1**	Protein Rundc1 [Source:UniProtKB/T	303552	10	89137045	89145851	8807	1170,43	0,39	3,02E-01
ENSRNOG00000030312		BEN domain-containing protein 6 [So	363212	9	37980049	38032680	52632	705,39	-0,51	3,02E-01
ENSRNOG00000048843	**Tsfm**	Ts translation elongation factor, mitoch	679068	7	70489452	70495163	5712	457,57	0,36	3,02E-01
ENSRNOG00000027914	**Plscr3**	phospholipid scramblase 3 (Plscr3), m	360549	10	56198996	56203509	4514	621,73	0,45	3,03E-01
ENSRNOG00000007539	**Rsad2**	radical S-adenosyl methionine domain	65190	6	54376868	54388860	11993	142,41	0,81	3,04E-01
ENSRNOG00000033262	**Reep6**	receptor accessory protein 6 (Reep6),	362835	7	12409855	12416524	6670	185,39	-0,68	3,04E-01
ENSRNOG00000024786	**Asb6**	ankyrin repeat and SOCS box-contain	296627	3	15019073	15023681	4609	1071,00	0,55	3,05E-01
ENSRNOG00000020049	**Slc7a6os**	solute carrier family 7, member 6 oppo	246187	19	49047661	49056771	9111	1030,75	0,38	3,05E-01
ENSRNOG00000002795	**Cog1**	component of oligomeric golgi comple	303652	10	101847319	101860914	13596	969,36	0,36	3,05E-01
ENSRNOG00000018288		nuclear receptor coactivator 6 (Ncoa6)	116464	3	157287951	157328147	40197	2265,21	-0,48	3,05E-01
ENSRNOG00000049661	**Prr24**	proline rich 24 (Prr24), mRNA [Source	1E+08	1	79475096	79475482	387	206,32	0,39	3,05E-01
ENSRNOG00000025141	**Plk1s1**	Protein Plk1s1 [Source:UniProtKB/Tr	311502	3	147486152	147591738	105587	145,86	-0,63	3,06E-01
ENSRNOG00000001068	**Rac1**	ras-related C3 botulinum toxin substra	363875	12	15133521	15153741	20221	5834,05	0,27	3,06E-01
ENSRNOG00000017508	**Suv420h2**	suppressor of variegation 4-20 homolo	308345	1	75861337	75868943	7607	536,67	0,34	3,06E-01
ENSRNOG00000021084	**Mpeg1**	macrophage expressed 1 (Mpeg1), m	64552	1	235887051	235889195	2145	22,76	-0,81	3,06E-01
ENSRNOG00000049828	**Crlf2**	cytokine receptor-like factor 2 (Crlf2),	171499	14	1459625	1464324	4700	232,41	0,41	3,06E-01
ENSRNOG00000000968	**Rnf6**	ring finger protein (C3H2C3 type) 6 (R	304271	12	12679023	12685173	6151	773,42	0,52	3,06E-01
ENSRNOG00000029205	**Zfp354c**	zinc finger protein 354C (Zfp354c), m	78972	10	36082344	36093357	11014	436,02	-0,51	3,07E-01
ENSRNOG00000001880	**Dgcr6**	DiGeorge syndrome critical region ger	303794	11	90168058	90173103	5046	3411,89	0,47	3,07E-01
ENSRNOG00000007542	**Scg5**	secretogranin V (7B2 protein) (Scg5),	25719	3	111811719	111856163	44445	1290,29	0,61	3,07E-01
ENSRNOG00000011316	**Fam167a**	family with sequence similarity 167, m	498533	15	50391387	50405856	14470	415,09	-0,54	3,07E-01
ENSRNOG00000016780		Protein RGD1310951 [Source:UniPro	313202	5	81059693	81131097	71405	365,19	-0,60	3,07E-01

ENSRNOG00000008696	**RGD15633**	uncharacterized protein LOC299700 [	299700	7	26602287	26607557	5271	326,12	0,55	3,07E-01
ENSRNOG00000017919		nucleoporin 133 [Source:MGI Symbol;	292085	19	67397820	67418668	20849	513,75	0,42	3,07E-01
ENSRNOG00000017951		RAN binding protein 9 [Source:MGI Symbol;Acc		17	25970453	26062435	91983	1563,16	-0,35	3,07E-01
ENSRNOG00000014254	**Cpt1a**	carnitine palmitoyltransferase 1a, liver	25757	1	225437255	225497583	60329	2533,48	-0,67	3,08E-01
ENSRNOG00000019578	**LOC36692**	ribosomal protein S16 (Rps16), mRNA	140655	1	86620608	86623553	2946	15881,99	0,54	3,08E-01
ENSRNOG00000010867	**Msantd1**	Myb/SANT-like DNA-binding domain c	498394	14	81783158	81789350	6193	72,19	-0,51	3,08E-01
ENSRNOG00000020702	**Cyb561a3**	cytochrome b561 family, member A3 (	361729	1	233566985	233588172	21188	160,56	0,63	3,08E-01
ENSRNOG00000027463	**Adamts3**	ADAM metallopeptidase with thrombo	305253	14	19773136	19977447	204312	816,35	-0,43	3,09E-01
ENSRNOG00000026111	**Morn5**	MORN repeat containing 5 (Morn5), m	362122	3	20688321	20716464	28144	523,70	0,44	3,09E-01
ENSRNOG00000007738	**Hm13**	histocompatibility 13 (Hm13), mRNA [	311545	3	154555181	154594098	38918	2999,05	0,36	3,09E-01
ENSRNOG00000021693	**Fam149a**	Protein Fam149a [Source:UniProtKB/	361153	16	49773192	49828133	54942	172,49	-0,46	3,09E-01
ENSRNOG00000020427	**Mef2bnb**	MEF2B neighbor (Mef2bnb), mRNA [S	688966	16	20872444	20879027	6584	828,01	0,50	3,10E-01
ENSRNOG00000020982	**LOC68778**	Finkel-Biskis-Reilly murine sarcoma vi	29752	1	228355748	228357261	1514	3343,92	0,55	3,10E-01
ENSRNOG00000012734	**Dcun1d1**	DCN1, defective in cullin neddylation 1	310324	2	142111797	142274371	162575	123,30	-0,59	3,10E-01
ENSRNOG00000005610	**Yipf4**	Yip1 domain family, member 4 (Yipf4),	362699	6	32015277	32026693	11417	534,14	0,59	3,10E-01
ENSRNOG00000028674	**Fbxw5**	F-box and WD repeat domain containi	362081	3	2708665	2712619	3955	1522,95	0,32	3,11E-01
ENSRNOG00000018646	**Hbegf**	heparin-binding EGF-like growth facto	25433	18	29036863	29046746	9884	301,02	-0,45	3,12E-01
ENSRNOG00000001490	**Pdcd2**	programmed cell death 2 (Pdcd2), mR	58934	1	58443078	58448568	5491	614,91	0,44	3,12E-01
ENSRNOG00000013786	**Tsc22d3**	TSC22 domain family, member 3 (Tsc2	83514	3	52711812	52715431	3620	1324,82	0,43	3,12E-01
ENSRNOG00000014793	**Gpr149**	G protein-coupled receptor 149 (Gpr1	192251	2	172232556	172307498	74943	55,24	-0,63	3,12E-01
ENSRNOG00000018518		proline rich 14 [Source:MGI Symbol;Acc:MGI:23		1	205910770	205914314	3545	506,43	0,35	3,12E-01
ENSRNOG00000020445	**Pdia2**	protein disulfide isomerase family A, m	287164	10	15376461	15379535	3075	20,01	-0,75	3,12E-01
ENSRNOG00000037204	**Lyrm9**	LYR motif containing 9 (Lyrm9), mRNA	497962	10	65531688	65545716	14029	625,54	0,50	3,12E-01
ENSRNOG00000014486	**Rfx3**	regulatory factor X, 3 (influences HLA	361746	1	253103266	253254214	150949	379,61	-0,74	3,12E-01
ENSRNOG00000028368	**Etnk2**	ethanolamine kinase 2 (Etnk2), mRNA	360843	13	55534037	55551850	17814	90,73	-0,69	3,12E-01
ENSRNOG00000000893	**Tmem248**	transmembrane protein 248 (Tmem24	288616	12	31870778	31895659	24882	1011,92	0,37	3,12E-01
ENSRNOG00000002388	**Trim41**	tripartite motif-containing 41 (Trim41),	303088	10	33939418	33950502	11085	1793,22	0,41	3,12E-01
ENSRNOG00000005218	**Sf3a1**	splicing factor 3a, subunit 1 (Sf3a1), m	305479	14	85074847	85095596	20750	3380,67	0,29	3,12E-01
ENSRNOG00000011646	**Rem2**	RAS (RAD and GEM) like GTP binding	64626	15	37006429	37010908	4480	245,89	0,63	3,12E-01
ENSRNOG00000019659	**Aspa**	aspartoacylase (Aspa), mRNA [Source	79251	10	59607015	59627427	20413	52,66	0,78	3,12E-01
ENSRNOG00000029466	**RGD15626**	uncharacterized protein LOC498831 [	498831	18	17157409	17429811	272403	166,64	-0,45	3,12E-01
ENSRNOG00000042848	**Jam2**	junctional adhesion molecule 2 (Jam2	619374	11	27864806	27913271	48466	2654,79	-0,54	3,12E-01
ENSRNOG00000003673	**Nup85**	nucleoporin 85 (Nup85), mRNA [Sourc	287830	10	104144379	104162637	18259	1027,14	0,37	3,12E-01
ENSRNOG00000042720	**Mrpl28**	Protein Mrpl28; Similar to 39S ribosom	497876	10	15309344	15312226	2883	2229,16	0,43	3,12E-01
ENSRNOG00000050657		cytokine receptor-like factor 3 [Source	54395	10	67041696	67060708	19013	243,16	0,59	3,12E-01
ENSRNOG00000001739	**Mfi2**	antigen p97 (melanoma associated) id	288038	11	75210907	75232739	21833	23,70	-0,71	3,13E-01
ENSRNOG00000014739	**Wfdc2**	WAP four-disulfide core domain 2 (Wf	286888	3	167197728	167203405	5678	167,47	0,51	3,13E-01
ENSRNOG00000007657	**Col27a1**	collagen, type XXVII, alpha 1 (Col27a1	298101	5	83170172	83284999	114828	233,40	-0,60	3,13E-01

ENSRNOG00000018898	**Mpi**	mannose phosphate isomerase (mapp	300741	8	62101461	62109361	7901	1058,88	0,51	3,13E-01
ENSRNOG00000022724	**Cnih3**	cornichon homolog 3 (Drosophila) (Cn	690252	13	104655193	104761214	106022	107,48	-0,48	3,13E-01
ENSRNOG00000006154	**Pde1a**	phosphodiesterase 1A, calmodulin-de	81529	3	73364538	73820966	456429	260,56	-0,64	3,13E-01
ENSRNOG00000000042	**Xpr1**	xenotropic and polytropic retrovirus re	289424	13	77853049	77991192	138144	2134,41	-0,36	3,13E-01
ENSRNOG00000001129	**Fbxo21**	F-box protein 21 (Fbxo21), mRNA [So	360818	12	46075020	46106396	31377	3594,14	0,41	3,14E-01
ENSRNOG00000018989	**Epdr1**	ependymin related 1 (Epdr1), mRNA [	291180	17	56066897	56091673	24777	1580,78	0,42	3,14E-01
ENSRNOG00000033688		centrosomal protein 170B [Source:MG	500726	6	146294896	146315899	21004	4762,47	0,46	3,14E-01
ENSRNOG00000027124	**Tdg**	thymine-DNA glycosylase (Tdg), mRN	114521	7	27313358	27333042	19685	4572,77	0,52	3,14E-01
ENSRNOG00000025808	**Aars2**	alanyl-tRNA synthetase 2, mitochondri	301254	9	16570774	16769589	198816	542,88	0,35	3,15E-01
ENSRNOG00000000150	**Dnajc1**	Protein Dnajc1 [Source:UniProtKB/Tr	1E+08	17	86711161	86865487	154327	236,95	0,44	3,15E-01
ENSRNOG00000000852	**Prrc2a**	proline-rich coiled-coil 2A (Prrc2a), mF	294250	20	7213149	7226658	13510	15473,99	0,38	3,15E-01
ENSRNOG00000012841	**Alg11**	ALG11, alpha-1,2-mannosyltransferas	361174	16	74487293	74494498	7206	325,57	-0,40	3,15E-01
ENSRNOG00000016998	**Atxn1**	ataxin 1 (Atxn1), mRNA [Source:RefS	25049	17	21272818	21559271	286454	729,66	-0,38	3,15E-01
ENSRNOG00000025596	**Ago4**	argonaute RISC catalytic component 4	298533	5	148272608	148328239	55632	296,19	-0,52	3,16E-01
ENSRNOG00000047524	**LOC10091**	phosphatidylinositol glycan anchor bio	1E+08	8	23221470	23222714	1245	331,54	0,36	3,16E-01
ENSRNOG00000009220	**Tmem17**	transmembrane protein 17 (Tmem17),	360985	14	107366212	107371630	5419	398,79	0,39	3,17E-01
ENSRNOG00000012193	**Cited2**	Cbp/p300-interacting transactivator, w	114490	1	14515629	14518063	2435	2439,29	0,66	3,17E-01
ENSRNOG00000020321	**Nop9**	NOP9 nucleolar protein (Nop9), mRN	290235	15	38320522	38328222	7701	692,10	0,41	3,17E-01
ENSRNOG00000046333		Uncharacterized protein [Source:UniProtKB/TrE		1	285233699	285247448	13750	439,11	-0,39	3,17E-01
ENSRNOG00000049783	**RGD13057**	REST corepressor 1 (Rcor1), mRNA [	314458	6	144864180	144864566	387	82,28	0,49	3,17E-01
ENSRNOG00000014901	**Uggt1**	UDP-glucose glycoprotein glucosyltra	171129	9	42350134	42459613	109480	2286,14	-0,31	3,17E-01
ENSRNOG00000006093	**LRRTM1**	leucine rich repeat transmembrane ne	679668	4	173006774	173022018	15245	1674,90	-0,41	3,17E-01
ENSRNOG00000022552	**Sh2d3c**	SH2 domain containing 3C (Sh2d3c),	362111	3	17095296	17130517	35222	752,61	0,35	3,17E-01
ENSRNOG00000024602	**Plekha7**	pleckstrin homology domain containin	499249	1	192397589	192463974	66386	78,87	-0,61	3,17E-01
ENSRNOG00000014688	**Prkcg**	protein kinase C, gamma (Prkcg), mR	24681	1	63399153	63425610	26458	1835,73	-0,50	3,17E-01
ENSRNOG00000013532	**Pgam2**	phosphoglycerate mutase 2 (muscle) (	24959	14	86736789	86738938	2150	197,05	0,57	3,17E-01
ENSRNOG00000014625	**Atp5d**	ATP synthase, H+ transporting, mitoc	245965	7	12596918	12600125	3208	7429,81	0,57	3,17E-01
ENSRNOG00000000804	**Mrps18b**	mitochondrial ribosomal protein S18B	294230	20	5442393	5448452	6060	841,72	0,40	3,18E-01
ENSRNOG00000003262	**Cacng4**	calcium channel, voltage-dependent, g	140725	10	95688305	95746619	58315	2457,31	-0,57	3,18E-01
ENSRNOG00000017219	**Vac14**	Vac14 homolog (S. cerevisiae) (Vac14	307842	19	51753774	51855971	102198	1786,57	0,44	3,18E-01
ENSRNOG00000026981	**Zfp282**	zinc finger protein 282 (Zfp282), mRN	297065	4	142223904	142250349	26446	2149,18	0,39	3,18E-01
ENSRNOG00000010172	**Mkrn3**	Protein Mkrn3 [Source:UniProtKB/TrE	292988	1	124200481	124202260	1780	745,84	0,62	3,18E-01
ENSRNOG00000020322	**Asb1**	ankyrin repeat and SOCS box-contain	316628	9	98344295	98360345	16051	1072,94	0,56	3,19E-01
ENSRNOG00000011507	**Pick1**	protein interacting with PRKCA 1 (Pick	84591	7	120459071	120478332	19262	1111,61	0,39	3,19E-01
ENSRNOG00000000104	**Thoc3**	THO complex 3 (Thoc3), mRNA [Sour	290519	17	12803773	12812964	9192	1390,52	0,40	3,21E-01
ENSRNOG00000007027	**Hgf**	hepatocyte growth factor (Hgf), mRNA	24446	4	15410059	15478680	68622	90,04	-0,78	3,21E-01
ENSRNOG00000010664	**Wdr73**	WD repeat domain 73 (Wdr73), mRN	308751	1	143664127	143672463	8337	853,32	0,43	3,21E-01
ENSRNOG00000005769	**Smg8**	Protein Smg8 [Source:UniProtKB/TrE	287596	10	75712430	75721227	8798	651,35	0,36	3,21E-01

ENSRNOG00000006086	**Lynx1**	Ly6/neurotoxin 1 (Lynx1), mRNA [Sour	300018	7	115891669	115892310	642	26,55	-0,66	3,21E-01
ENSRNOG00000010756	**Tp53**	tumor protein p53 (Tp53), mRNA [Sou	24842	10	55932658	55944086	11429	2221,46	0,46	3,21E-01
ENSRNOG00000018282	**Gda**	guanine deaminase (Gda), mRNA [So	83585	1	246270809	246346023	75215	491,66	-0,53	3,21E-01
ENSRNOG00000028208	**Smcr7**	Protein Smcr7; Similar to Smith-Mage	497916	10	46743660	46747788	4129	917,15	0,37	3,21E-01
ENSRNOG00000005783		Protein Dlg5 [Source:UniProtKB/TrEM	305645	15	115167	223675	108509	1932,87	-0,39	3,21E-01
ENSRNOG00000005464	**Lgalsl**	lectin, galactoside-binding-like (Lgalsl)	360983	14	104777094	104784596	7503	2171,13	-0,38	3,21E-01
ENSRNOG00000012140	**Cep89**	centrosomal protein 89kDa (Cep89), n	292811	1	92791362	92833455	42094	592,63	0,40	3,21E-01
ENSRNOG00000013679	**Sema4d**	sema domain, immunoglobulin domair	306790	17	15591157	15613214	22058	711,57	-0,36	3,21E-01
ENSRNOG00000019082	**Sbk1**	SH3-binding domain kinase 1 (Sbk1),	113907	1	68159188	68181024	21837	2547,90	0,50	3,22E-01
ENSRNOG00000012994	**Pus3**	pseudouridylate synthase 3 (Pus3), m	315554	8	36783458	36785648	2191	220,85	-0,47	3,22E-01
ENSRNOG00000004996	**Zcrb1**	zinc finger CCHC-type and RNA bindir	362990	7	134255943	134269279	13337	788,64	0,43	3,22E-01
ENSRNOG00000046502	**Lonp1**	lon peptidase 1, mitochondrial (Lonp1)	170916	9	9425780	9438104	12325	2822,93	0,27	3,22E-01
ENSRNOG00000009625	**Dpysl2**	dihydropyrimidinase-like 2 (Dpysl2), m	25416	15	48053084	48120298	67215	35847,43	-0,40	3,22E-01
ENSRNOG00000015560		RIKEN cDNA 2210018M11 gene [Source:MGI S		1	169748678	169814867	66190	764,47	-0,30	3,22E-01
ENSRNOG00000019741	**Isyna1**	inositol-3-phosphate synthase 1 (Isyna	290651	16	20445208	20448042	2835	604,58	0,59	3,22E-01
ENSRNOG00000019035	**Trpm8**	transient receptor potential cation chai	171384	9	95085452	95170098	84647	19,85	-0,72	3,23E-01
ENSRNOG00000027151	**Lrrc58**	leucine rich repeat containing 58 (Lrrc	303919	11	68841297	68853498	12202	1164,58	-0,46	3,23E-01
ENSRNOG00000001142	**Prkab1**	protein kinase, AMP-activated, beta 1	83803	12	48117572	48127947	10376	879,26	0,33	3,23E-01
ENSRNOG00000014044	**Pank4**	pantothenate kinase 4 (Pank4), mRNA	171053	5	175823559	175840289	16731	815,72	0,40	3,23E-01
ENSRNOG00000043185	**Cntnap5c**	contactin associated protein-like 5C (Cntnap5c),		13	11957533	12804561	847029	46,64	0,54	3,23E-01
ENSRNOG00000048712	**Ccdc137**	coiled-coil domain containing 137 (Ccc	688298	10	109223663	109230044	6382	818,15	0,50	3,23E-01
ENSRNOG00000049232	**Tcf7l2**	transcription factor 7-like 2 (T-cell spec	679869	1	284095287	284118928	23642	348,40	-0,70	3,23E-01
ENSRNOG00000008961	**Mapre3**	microtubule-associated protein, RP/EE	298848	6	36695665	36706479	10815	2481,66	0,39	3,24E-01
ENSRNOG00000021042	**Psmd4**	proteasome (prosome, macropain) 26	83499	2	215692998	215702195	9198	3380,00	0,36	3,24E-01
ENSRNOG00000021692	**LOC362473**			5	13996706	13998575	1870	59,45	0,53	3,24E-01
ENSRNOG00000000598	**Tube1**	tubulin, epsilon 1 (Tube1), mRNA [Sou	361856	20	45947864	45965854	17991	253,91	0,36	3,24E-01
ENSRNOG00000002862	**Clcn5**	chloride channel, voltage-sensitive 5 (	25749	X	16955709	16981815	26107	140,79	0,52	3,24E-01
ENSRNOG00000003983	**Pfdn2**	prefoldin subunit 2 (Pfdn2), mRNA [Sc	685607	13	94353202	94371423	18222	2151,36	0,36	3,24E-01
ENSRNOG00000005115	**Asf1b**	anti-silencing function 1B histone chap	304648	19	36055967	36070506	14540	217,78	0,60	3,24E-01
ENSRNOG00000006019	**G0s2**	G0/G1switch 2 (G0s2), mRNA [Source	289388	13	116559387	116560299	913	166,55	-0,79	3,24E-01
ENSRNOG00000007662	**Zfp800**	zinc finger protein 800 (Zfp800), mRN	500057	4	55246413	55267999	21587	229,55	-0,39	3,24E-01
ENSRNOG00000019294	**Stk16**	serine/threonine kinase 16 (Stk16), ml	286927	9	82180274	82183501	3228	1766,13	0,36	3,24E-01
ENSRNOG00000019974	**Uba52**	ubiquitin A-52 residue ribosomal prote	64156	16	20524449	20526604	2156	12225,24	0,52	3,24E-01
ENSRNOG00000026226	**Hook1**	hook homolog 1 (Drosophila) (Hook1),	313370	5	118887278	118948329	61052	271,82	-0,64	3,24E-01
ENSRNOG00000002879	**Psen2**	presenilin 2 (Psen2), mRNA [Source:F	81751	13	103521935	103547127	25193	726,03	0,62	3,24E-01
ENSRNOG00000012685	**Adck1**	aarF domain containing kinase 1 (Adc	366698	6	120923362	121019893	96532	541,89	0,49	3,24E-01
ENSRNOG00000021119	**Pdcd2l**	programmed cell death 2-like (Pdcd2l)	689637	1	91189390	91201359	11970	413,35	0,50	3,24E-01
ENSRNOG00000023814		Uncharacterized protein [Source:UniProtKB/TrE		4	222281854	222293504	11651	325,18	-0,46	3,24E-01

ENSRNOG00000027590	**Jakmip3**	janus kinase and microtubule interacti	365380	1	218277853	218347771	69919	293,57	-0,56	3,24E-01
ENSRNOG00000031208	**Mgat1**	mannosyl (alpha-1,3-)-glycoprotein be	81519	10	34296107	34313527	17421	1726,24	0,30	3,24E-01
ENSRNOG00000013972	**Pef1**	penta-EF hand domain containing 1 (F	297900	5	152039075	152062360	23286	1840,20	0,42	3,24E-01
ENSRNOG00000016815	**Tmem135**	transmembrane protein 135 (Tmem13	293098	1	159198116	159407174	209059	1416,76	-0,38	3,24E-01
ENSRNOG00000033531	**Cacna2d1**	calcium channel, voltage-dependent, a	25399	4	15684840	16105448	420609	1968,76	-0,41	3,24E-01
ENSRNOG00000049952		guanylyl cyclase domain containing 1	687713	20	16287174	16289504	2331	515,56	0,46	3,24E-01
ENSRNOG00000028872		retinoic acid induced 14 (Rai14), mRN	294804	2	84073868	84128487	54620	453,96	-0,61	3,25E-01
ENSRNOG00000047409		Similar to contactin associated protein-like 2 isof		4	141668088	141694942	26855	172,33	-0,40	3,25E-01
ENSRNOG00000001823		ST6 beta-galactosamide alpha-2,6-sia	25197	11	79754197	79808019	53823	1295,23	0,58	3,25E-01
ENSRNOG00000008118	**Sync**	syncoilin, intermediate filament proteir	362606	5	151209094	151231142	22049	106,77	-0,59	3,25E-01
ENSRNOG00000008970	**Pcdh17**	protocadherin 17 (Pcdh17), mRNA [Sc	306055	15	71182073	71271037	88965	1296,51	-0,36	3,25E-01
ENSRNOG00000015143	**Siah1a**	seven in absentia 1A (Siah1a), mRNA	140941	19	32403161	32404557	1397	1137,28	-0,35	3,25E-01
ENSRNOG00000032258	**Swt1**	Swt1 RNA endoribonuclease homolog	289088	13	73690645	73748003	57359	152,75	-0,43	3,25E-01
ENSRNOG00000039471	**Pcdhb17**	Protein Pcdhb17; RCG49511 [Source	548104	18	30269373	30271772	2400	108,01	-0,48	3,25E-01
ENSRNOG00000002903	**Specc1**	sperm antigen with calponin homology	303208	10	48031612	48234767	203156	479,96	-0,53	3,26E-01
ENSRNOG00000034184	**Zfp583**	zinc finger protein 583 (Zfp583), mRN	499068	1	72645124	72656306	11183	114,98	-0,43	3,26E-01
ENSRNOG00000007660	**Fntb**	farnesyltransferase, CAAX box, beta (	64511	6	109278424	109361572	83149	1187,89	0,47	3,26E-01
ENSRNOG00000011386	**Nhsl1**	Protein Nhsl1 [Source:UniProtKB/TrE	308631	1	15256105	15388904	132800	1130,74	0,38	3,26E-01
ENSRNOG00000018547	**Mrpl46**	mitochondrial ribosomal protein L46 (M	293054	1	141445111	141453185	8075	700,78	0,35	3,26E-01
ENSRNOG00000009050	**Amn**	amnion associated transmembrane pr	314459	6	144682579	144690020	7442	20,65	-0,77	3,26E-01
ENSRNOG00000021150	**Plcb3**	phospholipase C, beta 3 (phosphatidy	29322	1	229200438	229215707	15270	594,96	-0,53	3,26E-01
ENSRNOG00000036683	**Sirt7**	sirtuin 7 (Sirt7), mRNA [Source:RefSe	303745	10	109389098	109395794	6697	437,74	0,52	3,27E-01
ENSRNOG00000009566	**Phf12**	PHD finger protein 12 (Phf12), mRNA	303274	10	66762563	66810434	47872	1622,45	0,42	3,27E-01
ENSRNOG00000023657	**Gprin3**	Protein Gprin3 [Source:UniProtKB/TrE	502784	4	154510003	154512291	2289	26,17	-0,72	3,27E-01
ENSRNOG00000019825	**Zdhhc24**	zinc finger, DHHC-type containing 24 (	293665	1	227068278	227074385	6108	445,50	0,43	3,28E-01
ENSRNOG00000015332	**Thoc1**	THO complex 1 (Thoc1), mRNA [Sour	291797	18	1185635	1219998	34364	230,44	-0,52	3,28E-01
ENSRNOG00000001039	**Eif2b1**	eukaryotic translation initiation factor 2	64514	12	39315127	39323400	8274	1178,03	0,50	3,28E-01
ENSRNOG00000011058	**Utrn**	utrophin (Utrn), mRNA [Source:RefSe	25600	1	8098753	8608676	509924	564,60	-0,50	3,28E-01
ENSRNOG00000014587	**Mid2**	midline 2 (Mid2), mRNA [Source:RefS	363502	3	52482853	52580984	98132	225,03	-0,44	3,28E-01
ENSRNOG00000017003	**RGD15608**	Protein RGD1560871 [Source:UniPro	362398	4	186458289	186495366	37078	4905,77	-0,37	3,28E-01
ENSRNOG00000008597	**Tada3**	transcriptional adaptor 3 (Tada3), mRN	291150	4	208615470	208626923	11454	1264,96	0,42	3,28E-01
ENSRNOG00000020489	**Tmem145**	Protein Tmem145 [Source:UniProtKB	292722	1	83438613	83448233	9621	1170,32	-0,45	3,28E-01
ENSRNOG00000008421	**Klhl5**	kelch-like family member 5 (Klhl5), mF	305351	14	44618848	44658817	39970	2299,35	-0,41	3,28E-01
ENSRNOG00000014166	**LOC10091**	SPARC related modular calcium bindi	292401	1	57434174	57565314	131141	27,63	-0,77	3,28E-01
ENSRNOG00000002364	**Rnf112**	ring finger protein 112 (Rnf112), mRN	24916	10	47491745	47497296	5552	1066,89	-0,56	3,28E-01
ENSRNOG00000030151	**Thsd7a**	thrombospondin, type I, domain contai	500032	4	38500796	38938890	438095	468,90	-0,37	3,28E-01
ENSRNOG00000006968	**Mrpl19**	mitochondrial ribosomal protein L19 (M	297372	4	177390795	177395024	4230	536,23	0,42	3,28E-01
ENSRNOG00000008424	**Aagab**	alpha- and gamma-adaptin binding pro	171435	8	68247490	68284415	36926	774,56	0,43	3,28E-01

ENSRNOG00000018325	**Cnppd1**	cyclin Pas1/PHO80 domain containing	316530	9	82108127	82114523	6397	1459,52	0,50	3,28E-01
ENSRNOG00000024244	**Ttyh3**	tweety homolog 3 (Drosophila) (Ttyh3)	304315	12	18080074	18108489	28416	17120,26	0,31	3,29E-01
ENSRNOG00000017829	**Ssu72**	SSU72 RNA polymerase II CTD phosp	298681	5	176628574	176658329	29756	2046,78	0,42	3,29E-01
ENSRNOG00000008354	**Slc9a8**	solute carrier family 9, subfamily A (NH	311651	3	170326024	170373076	47053	781,43	0,56	3,29E-01
ENSRNOG00000022289	**Aip**	aryl-hydrocarbon receptor-interacting	282827	1	226232510	226240238	7729	1328,52	0,36	3,29E-01
ENSRNOG00000015708	**E2f4**	E2F transcription factor 4, p107/p130-	1E+08	19	48118651	48125266	6616	778,60	0,38	3,29E-01
ENSRNOG00000013248	**Wwc2**	WW and C2 domain containing 2 (Ww	498630	16	47091900	47254470	162571	919,92	-0,34	3,29E-01
ENSRNOG00000020737	**Cdc25a**	cell division cycle 25A (Cdc25a), mRN	171102	8	117306200	117324072	17873	578,12	0,52	3,30E-01
ENSRNOG00000005386		KIT ligand (Kitlg), transcript variant 1,	60427	7	42337401	42383921	46521	683,72	-0,73	3,31E-01
ENSRNOG00000003951	**Psmc1**	proteasome (prosome, macropain) 26	117263	6	133350565	133362938	12374	4965,47	0,47	3,31E-01
ENSRNOG00000028236	**RGD13091**	uncharacterized protein LOC289084	289084	13	74240136	74408344	168209	638,76	-0,33	3,31E-01
ENSRNOG00000035596	**Mir207**	rno-mir-207 [Source:miRBase;Acc:MI0003479]		5	61562680	61562757	78	41,13	0,63	3,31E-01
ENSRNOG00000009783	**LOC10090**	calcium/calmodulin-dependent protein	171140	15	3669216	3724735	55520	1636,08	-0,34	3,32E-01
ENSRNOG00000012826	**Creb3l2**	cAMP responsive element binding pro	362339	4	64691125	64803069	111945	334,71	0,49	3,32E-01
ENSRNOG00000014320	**Inhba**	inhibin beta-A (Inhba), mRNA [Source:	29200	17	49965505	49978776	13272	56,04	-0,76	3,32E-01
ENSRNOG00000015496	**Tpm4**	tropomyosin 4 (Tpm4), mRNA [Source	24852	16	19244266	19258182	13917	1081,43	0,47	3,32E-01
ENSRNOG00000021441	**Reln**	reelin (Reln), mRNA [Source:RefSeq r	24718	4	9349481	9775752	426272	5213,58	-0,67	3,32E-01
ENSRNOG00000026569		zinc finger prtoein 943 [Source:MGI Symbol;Acc		1	64950262	64962383	12122	81,93	-0,73	3,32E-01
ENSRNOG00000000421	**Skiv2l**	superkiller viralicidic activity 2-like (S.	294260	20	6599256	6609917	10662	1388,47	0,30	3,32E-01
ENSRNOG00000020833	**Cspg5**	chondroitin sulfate proteoglycan 5 (ne	50568	8	117686009	117699634	13626	5557,66	-0,44	3,32E-01
ENSRNOG00000002949	**Pgs1**	Protein Pgs1; Similar to phosphatidylg	303698	10	106632598	106663922	31325	1293,67	0,45	3,32E-01
ENSRNOG00000020961		lysine (K)-specific methyltransferase 2B [Source		1	90178012	90197133	19122	2130,38	0,36	3,32E-01
ENSRNOG00000048141	**Rbm4**	RNA binding motif protein 4 (Rbm4), n	293663	1	226903519	226913778	10260	850,47	0,46	3,32E-01
ENSRNOG00000009811	**Cnih1**	cornichon family AMPA receptor auxili	289994	15	27512057	27523878	11822	1854,48	0,30	3,33E-01
ENSRNOG00000019297	**Homer2**	homer homolog 2 (Drosophila) (Home	29547	1	144387111	144478036	90926	478,75	-0,51	3,33E-01
ENSRNOG00000000879	**Slc9a6**	Sodium/hydrogen exchanger [Source	302863	X	153620186	153673347	53162	1789,75	-0,40	3,33E-01
ENSRNOG00000007548	**Polr3f**	polymerase (RNA) III (DNA directed) p	311487	3	145114599	145130969	16371	664,52	-0,47	3,33E-01
ENSRNOG00000000242		synergin, gamma [Source:MGI Symbol;Acc:MGI		10	70890012	71271499	381488	2626,64	0,33	3,33E-01
ENSRNOG00000001283	**Anapc7**	anaphase promoting complex subunit	304490	12	41494331	41520882	26552	1548,33	0,36	3,33E-01
ENSRNOG00000001410	**Gigyf1**	GRB10 interacting GYF protein 1 (Gig	304378	12	24252933	24261868	8936	1185,48	0,46	3,33E-01
ENSRNOG00000004756	**Syt2**	synaptotagmin II (Syt2), mRNA [Sourc	24805	13	56621946	56629657	7712	31,06	-0,60	3,33E-01
ENSRNOG00000010555	**Phyhip**	phytanoyl-CoA 2-hydroxylase interacti	290356	15	56490752	56501997	11246	19,95	-0,72	3,33E-01
ENSRNOG00000017657	**Phf23**	PHD finger protein 23 (Phf23), mRNA	360550	10	56350259	56354352	4094	1860,84	0,41	3,33E-01
ENSRNOG00000018023	**Bin3**	bridging integrator 3 (Bin3), mRNA [Sc	361065	15	55524836	55544843	20008	569,55	0,42	3,34E-01
ENSRNOG00000036735	**Cables2**	Protein Cables2 [Source:UniProtKB/T	311703	3	181470268	181483108	12841	549,96	-0,54	3,34E-01
ENSRNOG00000016574	**Fiz1**	FLT3-interacting zinc finger 1 (Fiz1), m	292584	1	76148778	76155738	6961	930,60	0,28	3,34E-01
ENSRNOG00000016470	**Ndufa10l1**	NADH dehydrogenase (ubiquinone) 1	316632	9	99282166	99316927	34762	1308,81	0,43	3,34E-01
ENSRNOG00000042410				19	41576281	41576782	502	63,99	0,61	3,34E-01

ENSRNOG00000047545	**Adra2a**	adrenoceptor alpha 2A (Adra2a), mRN	25083	1	282178475	282181273	2799	385,46	-0,47	3,34E-01
ENSRNOG00000008425	**LOC68570**	neuron navigator 1 [Source:MGI Symb	685707	13	57316374	57463205	146832	6166,69	-0,50	3,34E-01
ENSRNOG00000001273		Protein Psmg3; RCG42703, isoform C	1E+08	12	18890327	18893168	2842	1144,25	0,47	3,35E-01
ENSRNOG00000042332	**Slc44a5**	solute carrier family 44, member 5 (Slc	365962	2	279103883	279195711	91829	263,12	-0,62	3,35E-01
ENSRNOG00000007206		RIKEN cDNA 4930452B06 gene [Sour	361016	15	21650963	21865186	214224	64,01	-0,53	3,36E-01
ENSRNOG00000010814	**Bmpr1a**	bone morphogenetic protein receptor,	81507	16	9084570	9114523	29954	1882,53	-0,47	3,36E-01
ENSRNOG00000014790	**Cryga**	crystallin, gamma A (Cryga), mRNA [S	684028	9	70740873	70742647	1775	33,35	0,64	3,36E-01
ENSRNOG00000017981	**Mcm10**	minichromosome maintenance comple	307126	17	78890310	78911578	21269	311,22	0,48	3,36E-01
ENSRNOG00000025602	**Cdk4**	cyclin-dependent kinase 4 (Cdk4), mR	94201	7	70527541	70530390	2850	8656,67	0,52	3,36E-01
ENSRNOG00000032635	**LOC10091**	ribosomal protein L8 (Rpl8), mRNA [S	26962	3	8751686	8752525	840	670,69	0,47	3,36E-01
ENSRNOG00000012219	**Snrnp40**	small nuclear ribonucleoprotein 40 (U5	313056	5	152294724	152327111	32388	1235,39	0,40	3,36E-01
ENSRNOG00000020115	**Ilkap**	integrin-linked kinase-associated serin	64538	9	98190648	98212635	21988	1440,83	0,39	3,36E-01
ENSRNOG00000014549	**Arhgef26**	Protein Arhgef26 [Source:UniProtKB/	310460	2	46226750	46352443	125694	1049,97	-0,32	3,37E-01
ENSRNOG00000029735	**Pid1**	phosphotyrosine interaction domain co	501174	9	91403610	91419329	15720	302,25	-0,49	3,37E-01
ENSRNOG00000002736	**Rabgap1l**	RAB GTPase activating protein 1-like	304914	13	82918223	83505642	587420	292,92	-0,62	3,37E-01
ENSRNOG00000006263	**Sh2d1a**	SH2 domain containing 1A (Sh2d1a),	501502	X	128978818	129007044	28227	97,15	-0,57	3,37E-01
ENSRNOG00000010873	**Pithd1**	Protein Pithd1 [Source:UniProtKB/TrE	298557	5	158088178	158097818	9641	1746,19	0,43	3,37E-01
ENSRNOG00000011951	**Plk2**	polo-like kinase 2 (Plk2), mRNA [Sour	83722	2	60970300	60976058	5759	2365,90	-0,42	3,38E-01
ENSRNOG00000015499		serine incorporator 4 (Serinc4), mRNA	311358	3	119957712	119963592	5881	1035,48	0,54	3,38E-01
ENSRNOG00000018187	**LOC10090**	Rac GTPase-activating protein 1 (Rac	315298	X	115782548	115811946	29399	226,56	0,49	3,38E-01
ENSRNOG00000018226		Protein Zcchc14 [Source:UniProtKB/T	365018	19	64385403	64425619	40217	1586,90	-0,33	3,38E-01
ENSRNOG00000020206	**Ctsd**	cathepsin D (Ctsd), mRNA [Source:Re	171293	1	222436922	222448798	11877	12610,83	0,41	3,38E-01
ENSRNOG00000001700	**Dfnb31**	deafness, autosomal recessive 31 (Df	313255	5	83349718	83431410	81693	485,52	-0,51	3,39E-01
ENSRNOG00000001081	**Ogfod2**	2-oxoglutarate and iron-dependent oxy	288657	12	39779493	39782802	3310	232,62	0,42	3,39E-01
ENSRNOG00000010222	**Glud1**	glutamate dehydrogenase 1 (Glud1), r	24399	16	8980462	9014001	33540	12731,73	-0,26	3,39E-01
ENSRNOG00000015845	**Fam129b**	family with sequence similarity 129, m	362115	3	17257993	17307290	49298	1569,71	0,33	3,39E-01
ENSRNOG00000010386	**H2afx**	H2A histone family, member X (H2afx)	500987	8	47284623	47285952	1330	2237,99	0,48	3,39E-01
ENSRNOG00000025796		RIKEN cDNA 4930432K21 gene [Sour	304654	19	36239995	36260885	20891	156,63	0,58	3,39E-01
ENSRNOG00000007918	**Tbxas1**	thromboxane A synthase 1, platelet (T	24886	4	66481264	66651601	170338	30,70	0,66	3,39E-01
ENSRNOG00000014805	**Wrap73**	WD repeat containing, antisense to TF	366515	5	174926481	174935895	9415	616,77	0,41	3,39E-01
ENSRNOG00000016883		ectonucleoside triphosphate diphosph	309390	1	270893394	270935069	41676	69,50	0,58	3,39E-01
ENSRNOG00000017765	**Net1**	neuroepithelial cell transforming 1 (Ne	307098	17	71714423	71724531	10109	501,60	0,44	3,39E-01
ENSRNOG00000027940	**Lppr3**	lipid phosphate phosphatase-related p	314614	7	12822277	12833539	11263	6938,91	0,35	3,39E-01
ENSRNOG00000030294	**Eml4**	echinoderm microtubule associated pr	313861	6	6573579	6629032	55454	1475,75	-0,48	3,39E-01
ENSRNOG00000010540	**Mrpl45**	mitochondrial ribosomal protein L45 (M	287656	10	85047890	85059050	11161	1463,12	0,29	3,39E-01
ENSRNOG00000005668	**Ndufa8**	NADH dehydrogenase (ubiquinone) 1	296658	3	20672277	20685979	13703	2674,88	0,33	3,40E-01
ENSRNOG00000000483	**Syngap1**	synaptic Ras GTPase activating protei	192117	20	7594614	7623865	29252	7503,92	-0,42	3,40E-01
ENSRNOG00000002365	**Itm2a**	integral membrane protein 2A (Itm2a),	317218	X	78689821	78695786	5966	71,02	-0,69	3,40E-01

ENSRNOG00000005652	**Cntn4**	contactin 4 (Cntn4), mRNA [Source:Re	116658	4	202737092	203254911	517820	122,73	-0,66	3,40E-01
ENSRNOG00000005669	**Car8**	carbonic anhydrase 8 (Car8), mRNA [S	297814	5	26010376	26106856	96481	124,75	-0,72	3,40E-01
ENSRNOG00000011518	**LOC50171**	dual specificity phosphatase 26 (putati	306527	16	64455739	64463016	7278	2020,82	0,45	3,40E-01
ENSRNOG00000013282	**Mctp1**	Protein Mctp1-ps1 [Source:UniProtKE	309928	2	3613029	3972025	358997	24,45	-0,72	3,40E-01
ENSRNOG00000013429	**Tbl3**	transducin (beta)-like 3 (Tbl3), mRNA	287120	10	13883208	13888420	5213	1187,42	0,31	3,40E-01
ENSRNOG00000015301		elongator acetyltransferase complex s	307545	18	16303487	16340055	36569	2668,04	0,54	3,40E-01
ENSRNOG00000048528		Uncharacterized protein [Source:UniProtKB/TrE		10	71159197	71182350	23154	100,79	0,53	3,40E-01
ENSRNOG00000048554				X	21229334	21230037	704	202,68	0,41	3,40E-01
ENSRNOG00000000606		protocadherin 15 (Pcdh15), mRNA [Sc	294350	20	17136666	17521597	384932	362,96	-0,77	3,40E-01
ENSRNOG00000028477	**Med8**	mediator complex subunit 8 (Med8), m	362575	5	141030635	141031519	885	260,86	0,47	3,40E-01
ENSRNOG00000000437	**Agpat1**	1-acylglycerol-3-phosphate O-acyltran	406165	20	6450325	6457628	7304	4812,80	0,38	3,40E-01
ENSRNOG00000001036	**Rsph10b**	radial spoke head 10 homolog B (Chla	288478	12	14709091	14756095	47005	158,35	-0,75	3,40E-01
ENSRNOG00000008223	**Cnr1**	cannabinoid receptor 1 (brain) (Cnr1),	25248	5	53881982	53904008	22027	7760,16	-0,54	3,40E-01
ENSRNOG00000009140	**C1qtnf4**	C1q and tumor necrosis factor related	311184	3	86422687	86427145	4459	2590,16	0,50	3,40E-01
ENSRNOG00000010915	**Ermp1**	endoplasmic reticulum metallopeptida	373544	1	255038060	255072206	34147	2572,58	-0,56	3,40E-01
ENSRNOG00000011366	**Pigs**	phosphatidylinositol glycan anchor bio	303277	10	66032984	66047520	14537	1710,88	0,35	3,40E-01
ENSRNOG00000019604		Phospholipase D2 [Source:UniProtKB/TrEMBL;		10	56909460	56926683	17224	457,01	-0,57	3,40E-01
ENSRNOG00000020340	**Brd8**	bromodomain containing 8 (Brd8), mR	291691	18	27112312	27132659	20348	2291,83	0,46	3,40E-01
ENSRNOG00000021287	**Hexim2**	hexamethylene bis-acetamide inducibl	303580	10	90988728	90994489	5762	298,60	0,36	3,40E-01
ENSRNOG00000021292	**LOC681419**			17	51093019	51094298	1280	166,88	0,42	3,40E-01
ENSRNOG00000010744	**Nrp1**	neuropilin 1 (Nrp1), mRNA [Source:Re	246331	19	71984687	72136034	151348	962,97	-0,47	3,41E-01
ENSRNOG00000015423	**Ccna2**	cyclin A2 (Ccna2), mRNA [Source:Ref	114494	2	142891080	142897322	6243	569,49	0,53	3,41E-01
ENSRNOG00000019961	**Tcte1**	t-complex-associated testis expressed	316242	9	16737240	16746455	9216	538,27	0,46	3,41E-01
ENSRNOG00000014613	**Ddah1**	dimethylarginine dimethylaminohydrol	64157	2	270160834	270289319	128486	3640,58	0,33	3,41E-01
ENSRNOG00000018556	**Tomm40**	translocase of outer mitochondrial mei	308416	1	81883632	81895516	11885	1678,97	0,36	3,41E-01
ENSRNOG00000019936	**Anapc15**	anaphase promoting complex subunit	293155	1	173173929	173176117	2189	750,33	0,38	3,41E-01
ENSRNOG00000024972	**Cox10**	Protoheme IX farnesyltransferase, mit	691853	10	50208977	50341923	132947	244,78	0,46	3,41E-01
ENSRNOG00000027731	**Ubxn10**	UBX domain protein 10 (Ubxn10), mR	298577	5	160901917	160907746	5830	26,55	-0,68	3,41E-01
ENSRNOG00000042195	**Pabpn1**	poly(A) binding protein, nuclear 1 (Pab	116697	15	37442586	37447197	4612	4343,82	0,41	3,41E-01
ENSRNOG00000046048	**Fam60a**	family with sequence similarity 60, member A (Fa		16	18319807	18335595	15789	63,08	0,63	3,41E-01
ENSRNOG00000047134	**Gtf2f1**	general transcription factor IIF, polype	316123	9	9033678	9043039	9362	2405,27	0,41	3,41E-01
ENSRNOG00000005101		CTAGE family, member 5 [Source:MG	1E+08	6	89744666	89812332	67667	792,49	0,41	3,41E-01
ENSRNOG00000020544	**Sdr39u1**	short chain dehydrogenase/reductase	361044	15	38452084	38455017	2934	967,21	0,37	3,42E-01
ENSRNOG00000010830	**Slc25a4**	solute carrier family 25 (mitochondrial	85333	16	48981575	48985393	3819	19626,33	0,36	3,42E-01
ENSRNOG00000016678	**Angptl2**	angiopoietin-like 2 (Angptl2), mRNA [S	171100	3	17596289	17626134	29846	82,00	-0,60	3,42E-01
ENSRNOG00000018281	**Uqcrfs1**	ubiquinol-cytochrome c reductase, Rie	291103	17	36991598	36996097	4500	5001,35	0,35	3,42E-01
ENSRNOG00000022753	**Mast3**	microtubule associated serine/threonir	688540	16	20257645	20271835	14191	1555,07	-0,42	3,42E-01
ENSRNOG00000032297	**Msmo1**	methylsterol monooxygenase 1 (Msmo	140910	16	26738367	26754806	16440	3710,08	-0,55	3,42E-01

ENSRNOG00000046639	**Itgae**	integrin, alpha E (Itgae), mRNA [Sourc	83577	10	59278907	59450774	171868	187,73	-0,60	3,42E-01
ENSRNOG00000038449		tigger transposable element derived 2 [Source:M		4	154355197	154356777	1581	125,84	-0,42	3,42E-01
ENSRNOG00000029220	**RGD13063**	Protein RGD1306353; RCG23367 [So	305911	15	40894102	40907132	13031	349,26	-0,46	3,42E-01
ENSRNOG00000013663	**Tmem86a**	transmembrane protein 86A (Tmem86	308602	1	104238667	104243015	4349	299,62	0,41	3,42E-01
ENSRNOG00000019205	**Gnpat**	glyceronephosphate O-acyltransferase	84470	19	68327864	68354395	26532	2080,18	0,45	3,42E-01
ENSRNOG00000011946	**Ptn**	pleiotrophin (Ptn), mRNA [Source:RefS	24924	4	64063954	64156273	92320	14800,58	-0,50	3,42E-01
ENSRNOG00000014395	**Gli3**	GLI family zinc finger 3 (Gli3), mRNA [	140588	17	50360049	50629477	269429	587,29	-0,47	3,42E-01
ENSRNOG00000013370	**LOC10091**	growth factor, augmenter of liver reger	27100	10	13876328	13878685	2358	591,37	0,46	3,42E-01
ENSRNOG00000017307	**Prss23**	protease, serine, 23 (Prss23), mRNA [	308807	1	160037254	160045625	8372	225,21	-0,51	3,42E-01
ENSRNOG00000021295	**Rae1**	RAE1 RNA export 1 homolog (S. pom	362281	3	177082075	177096826	14752	1263,59	0,33	3,42E-01
ENSRNOG00000026108	**Tcof1**	Treacher Collins-Franceschetti syndro	291571	18	55316277	55350440	34164	921,88	-0,51	3,42E-01
ENSRNOG00000047194	**Arl13b**	ADP-ribosylation factor-like 13B (Arl13	304037	7	1111829	1181765	69937	401,40	-0,50	3,43E-01
ENSRNOG00000010859	**Zyg11b**	Protein Zyg11b [Source:UniProtKB/Tr	362559	5	131695011	131725479	30469	1748,67	-0,40	3,43E-01
ENSRNOG00000023351		dishevelled associated activator of morphogenes		9	12381132	12423558	42427	285,56	-0,54	3,43E-01
ENSRNOG00000003251	**B3galt2**	UDP-Gal:betaGlcNAc beta 1,3-galacto	686081	13	65424610	65433621	9012	308,74	-0,68	3,43E-01
ENSRNOG00000012749	**C1qb**	complement component 1, q subcomp	29687	5	159008941	159014494	5554	73,90	-0,63	3,43E-01
ENSRNOG00000008193		complement component (3b/4b) recep	54243	13	118373053	118420106	47054	434,01	-0,32	3,43E-01
ENSRNOG00000014152	**Kcnip3**	Kv channel interacting protein 3, calse	65199	3	127245518	127307875	62358	107,62	-0,62	3,43E-01
ENSRNOG00000000957	**RGD15624**	Protein LOC100361103; Protein LOC1	79449	12	12104737	12106565	1829	479,04	0,30	3,44E-01
ENSRNOG00000049215	**Cbx2**	chromobox homolog 2 (Cbx2), mRNA	303730	10	107745908	107754738	8831	456,90	0,44	3,44E-01
ENSRNOG00000008937	**Csrp1**	cysteine and glycine-rich protein 1 (Cs	29276	13	57601914	57623352	21439	2749,01	0,61	3,44E-01
ENSRNOG00000018319	**Pisd**	Protein Pisd [Source:UniProtKB/TrEM	681361	14	83999012	84034630	35619	1370,11	0,29	3,44E-01
ENSRNOG00000011553	**Actl6a**	actin-like 6A (Actl6a), mRNA [Source:F	361925	2	138764113	138780203	16091	665,78	0,30	3,44E-01
ENSRNOG00000006472	**Hspa2**	heat shock protein 2 (Hspa2), mRNA [	60460	6	108843800	108846300	2501	465,85	-0,46	3,44E-01
ENSRNOG00000008100	**Rgs20**	regulator of G-protein signaling 20 (Rg	362477	5	19197565	19275849	78285	630,54	-0,51	3,44E-01
ENSRNOG00000010827	**Ptbp2**	polypyrimidine tract binding protein 2 (	310820	2	241308282	241367644	59363	1914,89	-0,51	3,44E-01
ENSRNOG00000032350	**Kcnip4**	Kv channel interacting protein 4 (Kcnip	259243	14	66629284	66779107	149824	118,52	-0,51	3,44E-01
ENSRNOG00000014575	**Rtcd1**	RNA terminal phosphate cyclase doma	295395	2	237659375	237680650	21276	2166,02	0,41	3,45E-01
ENSRNOG00000021847		Spindle and kinetochore-associated pı	361047	15	41918700	41937666	18967	301,64	0,42	3,45E-01
ENSRNOG00000004791	**Arf6**	ADP-ribosylation factor 6 (Arf6), mRN	79121	6	101150428	101151574	1147	1735,96	0,30	3,45E-01
ENSRNOG00000013919	**Ift46**	intraflagellar transport 46 homolog (Ch	300675	8	47699718	47716194	16477	1144,50	0,44	3,45E-01
ENSRNOG00000006674	**Fam101b**	family with sequence similarity 101, m	287534	10	63810534	63816787	6254	49,02	-0,65	3,45E-01
ENSRNOG00000006784	**Sema4f**	sema domain, immunoglobulin domair	29745	4	178414272	178440640	26369	1482,52	0,54	3,45E-01
ENSRNOG00000026964	**Gas8**	growth arrest specific 8 (Gas8), mRNA	361438	19	67025621	67045030	19410	237,19	0,49	3,45E-01
ENSRNOG00000046202	**Metrnl**	meteorin, glial cell differentiation regul	316842	10	110473811	110487800	13990	133,49	-0,69	3,45E-01
ENSRNOG00000050106	**Rcc1**	regulator of chromosome condensatio	682908	5	154176778	154193158	16381	780,09	0,38	3,45E-01
ENSRNOG00000016911		transmembrane BAX inhibitor motif co	24822	X	115538427	115573914	35488	8605,30	0,36	3,46E-01
ENSRNOG00000004449	**E2f6**	E2F transcription factor 6 (E2f6), mRN	313978	6	51811952	51828124	16173	914,05	-0,42	3,47E-01

ENSRNOG00000007281	**Flnc**	filamin C, gamma (Flnc), mRNA [Sour	362332	4	56478247	56505940	27694	427,87	-0,73	3,47E-01
ENSRNOG00000020704	**Dak**	dihydroxyacetone kinase 2 homolog (S	361730	1	233590129	233603337	13209	246,20	0,43	3,47E-01
ENSRNOG00000023851	**Igsf3**	immunoglobulin superfamily, member	295325	2	223212465	223299218	86754	3251,13	-0,49	3,47E-01
ENSRNOG00000043447	**Qars**	glutaminyl-tRNA synthetase (Qars), m	290868	8	116642373	116650404	8032	2618,98	0,41	3,47E-01
ENSRNOG00000011969	**Dock9**	dedicator of cytokinesis 9 (Dock9), mF	259237	15	111337693	111600206	262514	2225,31	-0,36	3,47E-01
ENSRNOG00000016491	**Fam110d**	family with sequence similarity 110, m	500563	5	156229920	156232883	2964	847,07	0,57	3,47E-01
ENSRNOG00000022790		Uncharacterized protein [Source:UniProtKB/TrE		10	45094429	45171867	77439	18,69	-0,71	3,47E-01
ENSRNOG00000002054		glomulin, FKBP associated protein (Gl	289437	14	3205772	3249112	43341	278,83	-0,46	3,48E-01
ENSRNOG00000048902		Influenza virus NS1A binding protein (Predicted)		13	73671622	73675647	4026	818,65	-0,49	3,48E-01
ENSRNOG00000019716	**Ntf3**	neurotrophin 3 (Ntf3), transcript varian	81737	4	225638802	225707718	68917	122,05	-0,67	3,48E-01
ENSRNOG00000011268	**Chd5**	chromodomain helicase DNA binding	691589	5	173072897	173119043	46147	3428,07	-0,39	3,48E-01
ENSRNOG00000018070	**Rnf182**	ring finger protein 182 (Rnf182), mRN	498726	17	26191715	26240098	48384	310,75	-0,40	3,49E-01
ENSRNOG00000046443		methenyltetrahydrofolate synthetase d	687539	19	63770618	63777888	7271	179,48	0,54	3,49E-01
ENSRNOG00000004613	**Gpm6b**	glycoprotein m6b (Gpm6b), mRNA [Sc	192179	X	30000365	30041765	41401	16218,93	-0,48	3,50E-01
ENSRNOG00000042786		vesicle transport through interaction w	65277	1	283705427	283882015	176589	397,25	0,30	3,50E-01
ENSRNOG00000009514	**Mme**	membrane metallo-endopeptidase (Mı	24590	2	173197265	173275026	77762	21,04	-0,74	3,51E-01
ENSRNOG00000015654	**Ghr**	growth hormone receptor (Ghr), mRN/	25235	2	72182692	72346118	163427	102,63	-0,45	3,51E-01
ENSRNOG00000009849	**N6amt2**	N-6 adenine-specific DNA methyltrans	290279	15	41658553	41671647	13095	333,72	0,36	3,51E-01
ENSRNOG00000023318	**Tigd3**	tigger transposable element derived 3	309174	1	228181887	228184306	2420	176,20	-0,40	3,51E-01
ENSRNOG00000025030	**Dffb**	DNA fragmentation factor, 40kDa, beta	84359	5	174741404	174753526	12123	342,97	0,32	3,51E-01
ENSRNOG00000000133	**Txndc15**	thioredoxin domain containing 15 (Txn	307180	17	11147557	11548829	401273	2483,00	0,44	3,52E-01
ENSRNOG00000007766	**Pqbp1**	polyglutamine binding protein 1 (Pqbp	302557	X	16128379	16234365	105987	1181,53	0,33	3,52E-01
ENSRNOG00000012563	**Arhgap29**	Rho GTPase activating protein 29 (Arl	310833	2	243589277	243648767	59491	122,37	-0,67	3,52E-01
ENSRNOG00000028014	**Cryzl1**	crystallin, zeta (quinone reductase)-lik	288256	11	35456478	35497959	41482	123,82	-0,47	3,52E-01
ENSRNOG00000043180				1	181825607	181825899	293	35,39	-0,61	3,52E-01
ENSRNOG00000011732	**LOC36594**	proteasome (prosome, macropain) sul	85492	3	28115650	28176238	60589	6064,30	0,34	3,52E-01
ENSRNOG00000018232	**Srf**	serum response factor (c-fos serum re	501099	9	15642594	15652177	9584	1861,59	0,39	3,52E-01
ENSRNOG00000002993	**Zc3h11a**	zinc finger CCCH-type containing 11A	360845	13	55250213	55288525	38313	661,82	-0,38	3,52E-01
ENSRNOG00000006708	**Tbc1d24**	TBC1 domain family, member 24 (Tbc	287110	10	13367060	13392774	25715	2802,81	-0,39	3,52E-01
ENSRNOG00000014668	**RGD62109**	UPF0183 protein C16orf70 homolog [	207123	19	48045945	48074039	28095	886,18	0,64	3,52E-01
ENSRNOG00000018577	**Neil1**	nei endonuclease VIII-like 1 (E. coli) (I	367090	8	60387398	60393242	5845	168,56	0,53	3,53E-01
ENSRNOG00000011981	**Slc39a13**	solute carrier family 39 (zinc transport	295928	3	86593277	86601182	7906	790,84	0,32	3,53E-01
ENSRNOG00000014480	**Sys1**	Protein Sys1; RCG32134, isoform CR	685079	3	167089540	167092595	3056	819,20	0,55	3,53E-01
ENSRNOG00000029280	**Gpaa1**	glycosylphosphatidylinositol anchor at	300046	7	117381997	117385579	3583	1639,06	0,38	3,53E-01
ENSRNOG00000047908		Uncharacterized protein [Source:UniProtKB/TrE		1	223809194	223827625	18432	52,36	0,61	3,53E-01
ENSRNOG00000016036	**Rprd1a**	Protein Rprd1a [Source:UniProtKB/Tr	291736	18	16212386	16256735	44350	538,97	-0,44	3,53E-01
ENSRNOG00000017012	**Coq7**	coenzyme Q7 homolog, ubiquinone (y	25249	1	195121560	195137541	15982	543,07	0,33	3,53E-01
ENSRNOG00000013160	**Sash1**	Protein RGD1566017 [Source:UniPro	365037	1	4470756	4631378	160623	949,78	-0,55	3,53E-01

ENSRNOG00000017986	**Zfp458**	zinc finger protein 458 (Zfp458), trans	499563	2	106640560	106649928	9369	43,20	-0,74	3,53E-01
ENSRNOG00000029812	**Mex3a**	Protein Mex3a [Source:UniProtKB/Trl	310631	2	207296030	207300710	4681	757,83	0,46	3,53E-01
ENSRNOG00000049181		SH2B adaptor protein 1 (Sh2b1), trans	89817	1	204857974	204865893	7920	2801,17	0,30	3,53E-01
ENSRNOG00000026994	**Afg3l1**	AFG3(ATPase family gene 3)-like 1 (S	361436	19	66982824	67009036	26213	1411,20	0,27	3,54E-01
ENSRNOG00000050841		acetylcholinesterase [Source:MGI Symbol;Acc:M		12	24487852	24491390	3539	131,30	-0,69	3,54E-01
ENSRNOG00000032311		tubulin tyrosine ligase-like family, member 8 [Sou		7	129597599	129634762	37164	17,94	-0,70	3,54E-01
ENSRNOG00000008782	**Pnisr**	PNN-interacting serine/arginine-rich pr	297942	5	40646030	40672914	26885	1169,02	-0,43	3,54E-01
ENSRNOG00000022303	**Pdf**	Protein Pdf [Source:UniProtKB/TrEMI	690214	19	50108584	50109917	1334	255,46	0,40	3,54E-01
ENSRNOG00000000956	**Rasl11a**	RAS-like family 11 member A (Rasl11a	304268	12	12093851	12098070	4220	72,78	-0,58	3,54E-01
ENSRNOG00000019482	**Gnao1**	guanine nucleotide binding protein (G	50664	19	11487578	11643367	155790	11975,18	-0,31	3,54E-01
ENSRNOG00000050600		zinc finger protein 872 [Source:MGI Symbol;Acc		8	23245924	23250062	4139	55,73	-0,50	3,55E-01
ENSRNOG00000021872	**Zfp213**	zinc finger protein 213 (Zfp213), mRN/	287094	10	12706830	12713743	6914	665,59	0,33	3,55E-01
ENSRNOG00000005762	**Rab22a**	RAB22A, member RAS oncogene fam	366265	3	177802660	177843968	41309	713,20	0,32	3,56E-01
ENSRNOG00000012620	**Syngr3**	synaptogyrin 3 (Syngr3), mRNA [Sour	302975	10	13868395	13873149	4755	3189,27	0,39	3,56E-01
ENSRNOG00000018815	**Plk1**	polo-like kinase 1 (Plk1), mRNA [Sour	25515	1	199178259	199188238	9980	547,38	0,54	3,56E-01
ENSRNOG00000006853	**Elavl2**	ELAV (embryonic lethal, abnormal vision, Droso		5	113450855	113577382	126528	936,45	-0,44	3,56E-01
ENSRNOG00000013317	**RGD15652**	similar to RIKEN cDNA 4931414P19 (	498514	15	37109233	37134202	24970	173,53	0,44	3,56E-01
ENSRNOG00000014722	**Raph1**	Ras association (RalGDS/AF-6) and p	363239	9	67107597	67184496	76900	933,10	-0,51	3,56E-01
ENSRNOG00000017963		Uncharacterized protein [Source:UniProtKB/TrE		1	39260120	39281445	21326	80,82	-0,63	3,56E-01
ENSRNOG00000019900	**Slc35b2**	solute carrier family 35 (adenosine 3'-p	501103	9	16712121	16715753	3633	1648,76	0,35	3,56E-01
ENSRNOG00000021526	**Slc25a34**	solute carrier family 25, member 34 (S	298606	5	163890565	163894419	3855	121,57	-0,74	3,56E-01
ENSRNOG00000023721	**LOC50268**	RIKEN cDNA 6820408C15 gene [Sou	502684	3	154241684	154259517	17834	53,31	-0,75	3,56E-01
ENSRNOG00000025624		Rho GTPase activating protein 20 (Arl	367085	8	54764682	54845836	81155	1126,34	-0,34	3,56E-01
ENSRNOG00000050946	**Fam110a**	family with sequence similarity 110, m	311535	3	153826435	153829327	2893	474,72	0,39	3,56E-01
ENSRNOG00000037505	**Ulk1**	unc-51 like autophagy activating kinas	360827	12	53646799	53673053	26255	2527,01	0,32	3,57E-01
ENSRNOG00000004749	**Slc30a1**	solute carrier family 30 (zinc transport	58976	13	115236547	115240420	3874	1234,40	0,46	3,57E-01
ENSRNOG00000004016	**Msl3**	male-specific lethal 3 homolog (Droso	317464	X	27420566	27438237	17672	592,78	-0,41	3,57E-01
ENSRNOG00000023908	**Egfem1**	EGF-like and EMI domain containing 1	310269	2	136659205	136807811	148607	61,00	-0,55	3,57E-01
ENSRNOG00000039924	**Triqk**	triple QxxK/R motif containing (Triqk),	500413	5	31193979	31266991	73013	389,40	-0,49	3,58E-01
ENSRNOG00000028801	**Gsap**	gamma-secretase activating protein (G	311984	4	10416405	10511054	94650	25,14	-0,63	3,58E-01
ENSRNOG00000006388	**Pygl**	phosphorylase, glycogen, liver (Pygl),	64035	6	102045139	102091169	46031	59,43	-0,50	3,58E-01
ENSRNOG00000012736	**Sssca1**	Sjogren syndrome/scleroderma autoa	689397	1	228020766	228022549	1784	2180,48	0,42	3,58E-01
ENSRNOG00000037871	**Sfxn5**	sideroflexin 5 (Sfxn5), mRNA [Source:	261737	4	181575048	181691696	116649	2036,35	-0,60	3,58E-01
ENSRNOG00000050671	**Ier5**	immediate early response 5 (Ier5), mR	498256	13	77678740	77679666	927	446,46	0,41	3,58E-01
ENSRNOG00000000010	**Cbln1**	cerebellin 1 precursor (Cbln1), mRNA	498922	19	31617915	31621724	3810	795,57	-0,51	3,59E-01
ENSRNOG00000010352	**Dnajc3**	DnaJ (Hsp40) homolog, subfamily C, r	63880	15	107610234	107649355	39122	469,53	-0,40	3,59E-01
ENSRNOG00000020289	**Akt1s1**	AKT1 substrate 1 (proline-rich) (Akt1s	292887	1	101913514	101916583	3070	1975,58	0,49	3,59E-01
ENSRNOG00000032134	**Uqcrc1**	ubiquinol-cytochrome c reductase core	301011	8	117030911	117042818	11908	5795,89	0,39	3,59E-01

ENSRNOG00000028077	**Wdr52**	Protein Wdr52 [Source:UniProtKB/TrE	363782	11	65270160	65342227	72068	44,22	-0,74	3,59E-01
ENSRNOG00000005248	**Slc1a4**	solute carrier family 1 (glutamate/neut	305540	14	104316565	104346057	29493	4244,91	-0,41	3,59E-01
ENSRNOG00000010520	**Wrap53**	WD repeat containing, antisense to TF	287432	10	55914381	55931438	17058	309,00	0,39	3,59E-01
ENSRNOG00000013383	**Tmub1**	transmembrane and ubiquitin-like dom	362301	4	7269540	7271681	2142	1020,05	0,34	3,59E-01
ENSRNOG00000017194	**Prdx1**	peroxiredoxin 1 (Prdx1), mRNA [Sourc	117254	5	139332537	139348214	15678	1162,20	-0,56	3,59E-01
ENSRNOG00000049054	**Ntsr2**	neurotensin receptor 2 (Ntsr2), mRNA	64636	6	51646002	51652450	6449	866,45	-0,74	3,59E-01
ENSRNOG00000005257	**Prkaca**	protein kinase, cAMP-dependent, cata	25636	19	36073104	36096875	23772	4025,47	0,33	3,59E-01
ENSRNOG00000007069	**Adhfe1**	alcohol dehydrogenase, iron containin	362474	5	14202076	14228623	26548	204,90	-0,66	3,60E-01
ENSRNOG00000007675	**Gpr63**	G protein-coupled receptor 63 (Gpr63)	297952	5	43958430	43999913	41484	152,09	-0,51	3,60E-01
ENSRNOG00000018462	**Rabep2**	rabaptin, RAB GTPase binding effecto	80754	1	204820053	204836433	16381	444,16	0,48	3,60E-01
ENSRNOG00000022373	**Dennd4b**	Protein Dennd4b [Source:UniProtKB/	361987	2	209061387	209075586	14200	1316,30	0,30	3,60E-01
ENSRNOG00000000396	**Ddx50**	DEAD (Asp-Glu-Ala-Asp) box polypep	361848	20	34024312	34053709	29398	795,20	-0,31	3,60E-01
ENSRNOG00000014340	**Pdcd7**	programmed cell death 7 (Pdcd7), mR	363082	8	70553737	70568103	14367	1158,07	0,44	3,60E-01
ENSRNOG00000013218		sin3 associated polypeptide [Source:M	680122	16	35925240	35930358	5119	597,80	0,49	3,61E-01
ENSRNOG00000024259	**Tmem54**	transmembrane protein 54 (Tmem54),	362605	5	151023461	151029951	6491	23,30	-0,72	3,61E-01
ENSRNOG00000011440	**Ccdc39**	coiled-coil domain containing 39 (Ccdc	310315	2	139933459	139971211	37753	249,25	-0,40	3,61E-01
ENSRNOG00000012183	**Glrx**	glutaredoxin (thioltransferase) (Glrx), r	64045	2	2604443	2614390	9948	1877,17	-0,38	3,61E-01
ENSRNOG00000000164	**Lamp2**	lysosomal-associated membrane prote	24944	X	124809053	124852469	43417	2610,88	-0,57	3,61E-01
ENSRNOG00000002262	**Enoph1**	enolase-phosphatase 1 (Enoph1), mR	305177	14	11116183	11142029	25847	1674,80	0,42	3,61E-01
ENSRNOG00000020658	**Aarsd1**	alanyl-tRNA synthetase domain contai	619440	10	89115999	89129102	13104	2122,54	0,45	3,61E-01
ENSRNOG00000028211	**Akap17a**	A kinase (PRKA) anchor protein 17A (.	288526	12	20512073	20516115	4043	2015,41	0,44	3,61E-01
ENSRNOG00000019570	**Gng3**	guanine nucleotide binding protein (G	114117	1	231972086	231973852	1767	4343,09	0,49	3,61E-01
ENSRNOG00000002194	**Coq2**	coenzyme Q2 4-hydroxybenzoate poly	498332	14	10528340	10547770	19431	1245,33	0,34	3,61E-01
ENSRNOG00000004708	**Aard**	alanine and arginine rich domain conta	246323	7	92232534	92237373	4840	69,09	-0,62	3,61E-01
ENSRNOG00000017714	**Usp3**	ubiquitin specific peptidase 3 (Usp3), r	363084	8	71875689	71890546	14858	503,83	-0,37	3,61E-01
ENSRNOG00000022537	**E2f8**		308607	1	105245744	105259620	13877	164,24	0,49	3,61E-01
ENSRNOG00000050052	**Cox19**	cytochrome c oxidase assembly homo	304330	12	19381857	19391336	9480	1680,05	0,34	3,61E-01
ENSRNOG00000006494	**Tubg2**	tubulin, gamma 2 (Tubg2), mRNA [Sou	680991	10	88867752	88874081	6330	212,88	0,45	3,61E-01
ENSRNOG00000012295		phospholipase A2, group VI (cytosolic,	360426	7	120512623	120552754	40132	710,30	-0,50	3,61E-01
ENSRNOG00000000021				4	219073024	219074225	1202	201,86	0,43	3,61E-01
ENSRNOG00000036661	**Rab40b**	Rab40b, member RAS oncogene fami	303754	10	110143366	110171085	27720	198,50	-0,47	3,61E-01
ENSRNOG00000043350	**Cap2**	CAP, adenylate cyclase-associated pr	116653	17	19345324	19492224	146901	481,01	-0,40	3,61E-01
ENSRNOG00000003797	**Mrps7**	mitochondrial ribosomal protein S7 (M	113958	10	104122365	104125390	3026	845,74	0,32	3,62E-01
ENSRNOG00000026051		Uncharacterized protein [Source:UniProtKB/TrE		20	5236559	5238631	2073	32,56	0,59	3,62E-01
ENSRNOG00000019435	**Psd**	pleckstrin and Sec7 domain containing	171381	1	273490272	273505122	14851	9863,36	-0,51	3,62E-01
ENSRNOG00000007900	**Tspan33**	tetraspanin 33 (Tspan33), mRNA [Sou	500065	4	56748444	56771953	23510	494,27	-0,47	3,62E-01
ENSRNOG00000026974	**Dbndd1**	dysbindin (dystrobrevin binding proteir	361437	19	67012418	67021168	8751	935,49	0,34	3,62E-01
ENSRNOG00000047726	**Inhbb**	inhibin beta-B (Inhbb), mRNA [Source	25196	13	40555699	40561389	5691	623,79	-0,39	3,62E-01

ENSRNOG00000004980	**Rangrf**	RAN guanine nucleotide release facto	287419	10	55301089	55302460	1372	235,38	-0,54	3,63E-01
ENSRNOG00000004373		ATP-dependent RNA helicase DDX39,	89827	19	35825685	35834039	8355	928,44	0,43	3,63E-01
ENSRNOG00000005223	**Hnmt**	histamine N-methyltransferase (Hnmt)	81676	3	896390	928854	32465	47,84	-0,52	3,63E-01
ENSRNOG00000006454	**Rph3al**	rabphilin 3A-like (without C2 domains)	171123	10	63905252	64016749	111498	49,92	-0,75	3,63E-01
ENSRNOG00000019962	**Sars2**	seryl-tRNA synthetase 2, mitochondria	292759	1	88104901	88116638	11738	259,07	0,40	3,63E-01
ENSRNOG00000034233	**Fyttd1**	forty-two-three domain containing 1 (F	360726	11	74198837	74221579	22743	1635,54	-0,51	3,63E-01
ENSRNOG00000014303		low-density lipoprotein receptor-relate	292462	1	3400376	3428890	28515	5975,07	0,42	3,63E-01
ENSRNOG00000019325	**Rnf185**	ring finger protein 185 (Rnf185), mRN,	360967	14	84336669	84373469	36801	1394,26	0,41	3,63E-01
ENSRNOG00000029330	**Car5b**	carbonic anhydrase 5b, mitochondrial	302669	X	32609747	32652918	43172	31,93	-0,58	3,63E-01
ENSRNOG00000032074		homeobox protein ARX [Source:RefS	317268	X	62957758	62969937	12180	1912,78	0,44	3,63E-01
ENSRNOG00000016683	**Zfp668**	zinc finger protein 668 (Zfp668), mRN,	309002	1	206334471	206336683	2213	372,72	0,36	3,63E-01
ENSRNOG00000019237	**Dab2ip**	DAB2 interacting protein (Dab2ip), mF	192126	3	20200937	20371266	170330	4130,97	0,35	3,63E-01
ENSRNOG00000005767		tubby-like protein 3 [Source:MGI Symt	688749	4	226844164	226883755	39592	350,18	0,42	3,63E-01
ENSRNOG00000012184	**Urgcp**	upregulator of cell proliferation (Urgcp	305493	14	86649972	86660719	10748	1373,04	0,27	3,63E-01
ENSRNOG00000010953	**Icmt**	isoprenylcysteine carboxyl methyltrans	170818	5	173028513	173035369	6857	611,42	0,37	3,63E-01
ENSRNOG00000003719	**Atpaf2**	ATP synthase mitochondrial F1 compl	303190	10	46523866	46539216	15351	747,85	0,37	3,64E-01
ENSRNOG00000017079	**Pgm2l1**	phosphoglucomutase 2-like 1 (Pgm2l1	685076	1	171438702	171487835	49134	2674,61	-0,65	3,64E-01
ENSRNOG00000027487	**Trim28**	tripartite motif-containing 28 (Trim28),	116698	1	66355606	66362276	6671	7743,25	0,31	3,65E-01
ENSRNOG00000008570	**Cadps**	Ca++-dependent secretion activator ((	26989	15	19278581	19669176	390596	3146,62	-0,36	3,65E-01
ENSRNOG00000020541	**Nprl3**	nitrogen permease regulator-like 3 (S.	360505	10	15516050	15556474	40425	531,49	0,31	3,65E-01
ENSRNOG00000043167		integrin alpha 9 [Source:MGI Symbol;Acc:MGI:1		8	126493595	126791845	298251	43,91	-0,56	3,65E-01
ENSRNOG00000004622	**Calcrl**	calcitonin receptor-like (Calcrl), mRNA	25029	3	78271011	78366562	95552	151,38	-0,74	3,65E-01
ENSRNOG00000047543	**Rpgrip1**	Protein Rpgrip1 [Source:UniProtKB/T	305850	15	32370343	32384859	14517	24,32	-0,69	3,65E-01
ENSRNOG00000002151	**Tagln3**	transgelin 3 (Tagln3), transcript varian	63837	11	60668378	60681692	13315	2637,34	0,48	3,65E-01
ENSRNOG00000006505		loss of heterozygosity, 12, chromosom	362452	4	232678816	232748292	69477	512,38	0,31	3,65E-01
ENSRNOG00000008631	**Gpc5**	glypican 5 (Gpc5), mRNA [Source:Ref	306157	15	104018023	104084185	66163	244,07	-0,46	3,65E-01
ENSRNOG00000048145	**Sstr1**	somatostatin receptor 1 (Sstr1), mRN/	25033	6	88784408	88787878	3471	601,51	0,49	3,65E-01
ENSRNOG00000002440	**Ralb**	v-ral simian leukemia viral oncogene h	116546	13	40612012	40621240	9229	675,97	0,30	3,65E-01
ENSRNOG00000004807	**Arf2**	ADP-ribosylation factor 2 (Arf2), mRN,	79119	10	91783781	91805554	21774	3689,03	0,55	3,65E-01
ENSRNOG00000005578	**Zbtb44**	zinc finger and BTB domain containinç	363035	8	32191761	32243080	51320	362,93	-0,44	3,65E-01
ENSRNOG00000006965	**Aff4**	AF4/FMR2 family, member 4 (Aff4), m	303132	10	38473786	38555300	81515	7426,53	0,31	3,65E-01
ENSRNOG00000040205	**Zcchc24**	zinc finger, CCHC domain containing :	361104	16	1973079	2024105	51027	4090,58	-0,46	3,65E-01
ENSRNOG00000043189	**Trub2**	TruB pseudouridine (psi) synthase hor	366012	3	13681673	13691949	10277	1177,78	0,36	3,65E-01
ENSRNOG00000007235	**LOC68996**	ATP synthase, H+ transporting, mitoch	29754	10	83700817	83703415	2599	2283,90	0,37	3,66E-01
ENSRNOG00000049270	**Ppp2r3b**	protein phosphatase 2, regulatory sub	682033	14	1472151	1478801	6651	834,69	0,33	3,66E-01
ENSRNOG00000014065	**Zfp653**	zinc finger protein 653 (Zfp653), mRN,	300446	8	23121360	23139663	18304	384,36	0,33	3,66E-01
ENSRNOG00000014429	**Arfgap2**	ADP-ribosylation factor GTPase activa	362162	3	86790400	86802349	11950	2539,80	0,32	3,66E-01
ENSRNOG00000033915	**Gpt**	glutamic-pyruvate transaminase (alani	81670	7	117747058	117749910	2853	271,94	0,44	3,66E-01

ENSRNOG00000011781	**Oplah**	5-oxoprolinase (ATP-hydrolysing) (Opl	116684	7	117339712	117354917	15206	398,39	-0,61	3,67E-01
ENSRNOG00000007291	**Terf1**	telomeric repeat binding factor (NIMA-	297758	5	2843154	2872363	29210	197,95	-0,46	3,67E-01
ENSRNOG00000012074	**Ifngr1**	interferon gamma receptor 1 (Ifngr1), i	116465	1	16607955	16626390	18436	935,57	0,47	3,68E-01
ENSRNOG00000032780	**Ict1**	immature colon carcinoma transcript 1	303673	10	104316049	104322545	6497	672,82	0,36	3,68E-01
ENSRNOG00000017125	**Fam53b**	family with sequence similarity 53, me	309060	1	211708688	211798528	89841	855,74	0,30	3,68E-01
ENSRNOG00000024082	**Gldn**	gliomedin (Gldn), mRNA [Source:RefS	315675	8	57446589	57490037	43449	41,64	-0,71	3,68E-01
ENSRNOG00000009549	**Fbxo3**	F-box protein 3 (Fbxo3), mRNA [Sourc	690634	3	100608785	100640793	32009	1219,14	-0,35	3,68E-01
ENSRNOG00000012147	**Nop14**	NOP14 nucleolar protein (Nop14), mR	289724	14	82027602	82048732	21131	931,12	0,37	3,68E-01
ENSRNOG00000003924	**Pi4k2b**	phosphatidylinositol 4-kinase type 2 be	305419	14	60685764	60712615	26852	173,80	-0,39	3,68E-01
ENSRNOG00000003870	**C1qtnf2**	C1q and tumor necrosis factor related	497886	10	28909018	28925541	16524	59,63	0,57	3,68E-01
ENSRNOG00000011258	**Masp2**	mannan-binding lectin serine peptidas	64459	5	169071761	169086477	14717	102,74	-0,54	3,68E-01
ENSRNOG00000036760		transmembrane channel-like protein 5	365360	1	195224593	195282791	58199	58,08	-0,60	3,68E-01
ENSRNOG00000023633	**Crabp1**	cellular retinoic acid binding protein 1	25061	8	57926233	57934271	8039	261,54	-0,54	3,69E-01
ENSRNOG00000031287	**Cacna2d3**	calcium channel, voltage-dependent, a	306243	16	4812761	5730476	917716	976,09	-0,39	3,69E-01
ENSRNOG00000012573	**Dlgap2**	discs, large (Drosophila) homolog-ass	116681	16	79447602	79750039	302438	294,43	-0,38	3,69E-01
ENSRNOG00000019940	**Ssr2**	signal sequence receptor, beta (Ssr2),	295235	2	207354107	207362738	8632	3987,94	0,36	3,69E-01
ENSRNOG00000003917	**Uck2**	uridine-cytidine kinase 2 (Uck2), mRN	304944	13	90035481	90087109	51629	677,30	0,49	3,70E-01
ENSRNOG00000006551	**Dcaf15**	DDB1 and CUL4 associated factor 15	304653	19	36189603	36197003	7401	1103,98	0,45	3,70E-01
ENSRNOG00000004813	**Rtcb**	RNA 2',3'-cyclic phosphate and 5'-OH	362855	7	24063301	24082716	19416	1391,38	0,44	3,70E-01
ENSRNOG00000008296	**Tpgs1**	tubulin polyglutamylase complex subu	691093	7	13080966	13086888	5923	2303,25	0,54	3,70E-01
ENSRNOG00000036790	**Olfml1**	olfactomedin-like 1 (Olfml1), mRNA [S	361621	1	178792340	178816630	24291	701,93	-0,59	3,70E-01
ENSRNOG00000049550		Uncharacterized protein [Source:UniProtKB/TrE		7	121452810	121453022	213	160,14	0,43	3,70E-01
ENSRNOG00000050639		NACHT and WD repeat domain containing 1 [Sc		16	18741609	18783744	42136	1256,11	-0,64	3,70E-01
ENSRNOG00000005034	**Arhgef25**	Rho guanine nucleotide exchange fac	314904	7	70646953	70653981	7029	642,63	-0,42	3,71E-01
ENSRNOG00000011040	**LOC680142**			6	62442691	62444516	1826	372,11	-0,49	3,71E-01
ENSRNOG00000025269	**Slc25a44**	solute carrier family 25, member 44 (S	365841	2	207174512	207189211	14700	2037,96	0,38	3,71E-01
ENSRNOG00000031731	**Fam216a**	family with sequence similarity 216, m	288667	12	41560318	41569453	9136	1186,07	0,33	3,71E-01
ENSRNOG00000033639	**Zfp777**	zinc finger protein 777 (Zfp777), mRN	502764	4	142354004	142376510	22507	1177,44	0,31	3,72E-01
ENSRNOG00000017399	**Acrbp**	acrosin binding protein (Acrbp), mRNA	500316	4	224581828	224585861	4034	42,14	0,66	3,72E-01
ENSRNOG00000003847	**Gid4**	Protein LOC687192; RCG33430, isofo	687192	10	46539787	46563377	23591	588,20	-0,37	3,72E-01
ENSRNOG00000006614	**Rfx7**	regulatory factor X, 7 (Rfx7), mRNA [S	315804	8	73807892	73927996	120105	1968,69	-0,30	3,72E-01
ENSRNOG00000022309	**Frem1**	Fras1 related extracellular matrix 1 (Fr	298185	5	105042311	105160290	117980	52,89	-0,54	3,73E-01
ENSRNOG00000006983	**Nr2c1**	nuclear receptor subfamily 2, group C,	252924	7	35137598	35189641	52044	321,65	-0,42	3,73E-01
ENSRNOG00000017646	**RGD13096**	Protein RGD1309651 [Source:UniPro	361424	19	64906830	64925543	18714	57,52	-0,73	3,73E-01
ENSRNOG00000017115	**Chchd6**	coiled-coil-helix-coiled-coil-helix doma	297436	4	183964605	184187039	222435	1494,21	0,39	3,74E-01
ENSRNOG00000006420	**Rbm38**	RNA binding motif protein 38 (Rbm38)	366262	3	177104361	177116960	12600	578,46	0,53	3,74E-01
ENSRNOG00000021330	**Sprtn**	SprT-like N-terminal domain (Sprtn), m	292101	19	68363402	68367942	4541	334,88	0,56	3,75E-01
ENSRNOG00000010424		Fanconi anemia, complementation gro	691105	5	62973516	62981572	8057	150,76	0,41	3,75E-01

ENSRNOG00000042261	**Hdgf**	hepatoma-derived growth factor (Hdgf), mRNA [		2	206507465	206687513	180049	2560,79	0,27	3,75E-01
ENSRNOG00000006961	**Snrpb**	small nuclear ribonucleoprotein polype	171365	3	129196990	129204596	7607	3623,13	0,41	3,76E-01
ENSRNOG00000026204	**Dclre1a**	DNA cross-link repair 1A (Dclre1a), ml	292127	1	284716585	284735397	18813	353,38	-0,37	3,76E-01
ENSRNOG00000008691	**Ick**	intestinal cell kinase (Ick), mRNA [Sou	84411	8	84982701	85037684	54984	1078,19	-0,30	3,76E-01
ENSRNOG00000033611	**Wdr83os**	WD repeat domain 83 opposite strand	288925	19	37169959	37171458	1500	2355,98	0,42	3,76E-01
ENSRNOG00000009995	**Rapgef6**	Rap guanine nucleotide exchange fac	303141	10	39648119	39812677	164559	413,13	0,49	3,77E-01
ENSRNOG00000011238	**Tiparp**	Protein Tiparp; TCDD-inducible poly(A	310467	2	176688181	176710351	22171	336,67	-0,46	3,77E-01
ENSRNOG00000003802	**Pttg1**	pituitary tumor-transforming 1 (Pttg1),	64193	10	28861945	28867675	5731	288,36	0,60	3,77E-01
ENSRNOG00000007026	**LOC68940**	similar to H2A histone family, member	498894	14	80320991	80339834	18844	523,56	0,43	3,78E-01
ENSRNOG00000009491	**Phc3**	polyhomeotic homolog 3 (Drosophila)	310258	2	135727661	135791251	63591	689,19	-0,32	3,78E-01
ENSRNOG00000039530	**Ccl27**	chemokine (C-C motif) ligand 27 (Ccl2	362505	5	62683432	62687950	4519	606,76	0,45	3,79E-01
ENSRNOG00000032677	**Tssc4**	tumor suppressing subtransferable ca	361682	1	223116610	223118239	1630	1341,33	0,28	3,79E-01
ENSRNOG00000017033	**Nckap5l**	NCK-associated protein 5-like (Nckap	315297	X	115586236	115597266	11031	1440,50	0,36	3,80E-01
ENSRNOG00000010174		ectonucleotide pyrophosphatase/phos	301261	9	18326766	18336443	9678	378,03	0,55	3,80E-01
ENSRNOG00000000845	**Abhd16a**	abhydrolase domain containing 16A (A	361796	20	7153000	7168192	15193	1525,70	0,41	3,80E-01
ENSRNOG00000001367	**Gpc2**	glypican 2 (Gpc2), mRNA [Source:Ref	171517	12	21649636	21655969	6334	2315,96	0,49	3,80E-01
ENSRNOG00000007922	**Cldn19**	claudin 19 (Cldn19), mRNA [Source:R	298487	5	142113780	142118475	4696	85,60	-0,67	3,80E-01
ENSRNOG00000009094	**Nudt4**	nudix (nucleoside diphosphate linked	94267	7	36695609	36711834	16226	4934,70	-0,39	3,80E-01
ENSRNOG00000009663	**Apex1**	APEX nuclease (multifunctional DNA r	79116	15	31682396	31684507	2112	3590,78	0,30	3,80E-01
ENSRNOG00000017386	**Il11**	interleukin 11 (Il11), mRNA [Source:Re	171040	1	75891524	75897820	6297	25,48	-0,58	3,80E-01
ENSRNOG00000010768	**Kpna4**	karyopherin alpha 4 (importin alpha 3)	361959	2	185044071	185101033	56963	1505,69	-0,50	3,80E-01
ENSRNOG00000021267	**Gpcpd1**	glycerophosphocholine phosphodieste	362219	3	131661271	131700120	38850	711,89	-0,53	3,80E-01
ENSRNOG00000002244	**Pdgfra**	platelet derived growth factor receptor	25267	14	35356435	35409311	52877	3533,26	-0,71	3,81E-01
ENSRNOG00000023650	**Cops4**	COP9 signalosome subunit 4 (Cops4)	360915	14	10666123	10696464	30342	2253,31	0,30	3,81E-01
ENSRNOG00000027171	**Ndst2**	N-deacetylase/N-sulfotransferase (hep	114002	15	3726914	3737357	10444	597,64	0,29	3,81E-01
ENSRNOG00000013746	**Senp3**	Sumo1/sentrin/SMT3 specific peptidas	303245	10	56023253	56032029	8777	2113,09	0,29	3,81E-01
ENSRNOG00000001632		bromodomain and WD repeat domain	304061	11	39870304	39961008	90705	575,78	0,41	3,81E-01
ENSRNOG00000023989	**Actr5**	ARP5 actin-related protein 5 homolog	362258	3	160477100	160490585	13486	454,38	0,34	3,81E-01
ENSRNOG00000003496	**Tbc1d9**	TBC1 domain family, member 9 (with	304645	19	35307319	35408165	100847	4436,49	0,44	3,81E-01
ENSRNOG00000006068		transmembrane protein 117 [Source:M	500921	7	135545200	136048699	503500	138,85	-0,49	3,81E-01
ENSRNOG00000018295	**RGD13099**	similar to 2610301G19Rik protein (RG	306007	15	55545044	55560184	15141	5471,90	0,37	3,81E-01
ENSRNOG00000006079	**Psd4**	pleckstrin and Sec7 domain containing	311785	3	1471353	1507762	36410	49,73	-0,63	3,82E-01
ENSRNOG00000011895		Bq135360; Protein Saal1 [Source:Uni	687992	1	103793445	103816239	22795	235,54	0,35	3,82E-01
ENSRNOG00000005301		translation initiation factor eIF-2B subu	117019	6	36364317	36369267	4951	1036,93	0,27	3,82E-01
ENSRNOG00000021240	**Atrn**	attractin (Atrn), mRNA [Source:RefSec	83526	3	129934530	130067263	132734	7015,84	-0,33	3,82E-01
ENSRNOG00000016234	**Ciapin1**	cytokine induced apoptosis inhibitor 1	307649	19	10591807	10607103	15297	2416,09	0,51	3,83E-01
ENSRNOG00000020744	**Adar**	adenosine deaminase, RNA-specific (.	81635	2	208485313	208508301	22989	4540,21	0,43	3,83E-01
ENSRNOG00000009019	**Slc6a6**	solute carrier family 6 (neurotransmitte	29464	4	188176251	188245992	69742	1365,33	-0,35	3,83E-01

ENSRNOG00000020087	**Zfp90**	zinc finger protein 90 (Zfp90), mRNA [	498945	19	49466084	49476869	10786	185,62	-0,37	3,83E-01
ENSRNOG00000005587	**Suclg1**	succinate-CoA ligase, alpha subunit (S	114597	4	165937918	165967275	29358	3982,87	0,30	3,83E-01
ENSRNOG00000008855	**Gjb2**	gap junction protein, beta 2 (Gjb2), mF	394266	15	41224500	41230461	5962	23,66	-0,72	3,83E-01
ENSRNOG00000002126	**Ncam2**	neural cell adhesion molecule 2 (Ncan	288280	11	24122738	24352502	229765	707,67	-0,61	3,83E-01
ENSRNOG00000014811		cysteine and histidine rich 1-like (RGD	315097	7	117697254	117710445	13192	1729,14	0,25	3,83E-01
ENSRNOG00000015409	**LOC10091**	ubiquitin specific peptidase 5 (isopepti	297593	4	224350336	224365479	15144	3350,00	0,38	3,83E-01
ENSRNOG00000036689	**P4hb**	prolyl 4-hydroxylase, beta polypeptide	25506	10	109329599	109341105	11507	10365,84	0,46	3,83E-01
ENSRNOG00000016512	**Sema3b**	sema domain, immunoglobulin domair	363142	8	115710332	115717175	6844	235,49	-0,64	3,85E-01
ENSRNOG00000020620	**Ppp1r32**	protein phosphatase 1, regulatory sub	252958	1	233464614	233473161	8548	62,79	-0,63	3,85E-01
ENSRNOG00000002471	**Polq**	polymerase (DNA directed), theta (Pol	288079	11	69681013	69786646	105634	56,44	0,61	3,85E-01
ENSRNOG00000004306	**Zbtb39**	zinc finger and BTB domain containing	299510	7	71209250	71216714	7465	764,65	0,34	3,85E-01
ENSRNOG00000007934	**LOC10091**	leucine rich repeat and fibronectin type	1E+08	7	119883205	119885667	2463	1304,53	-0,45	3,85E-01
ENSRNOG00000018464	**Lipt1**	lipoyltransferase 1 (Lipt1), mRNA [Sou	316342	9	44164007	44169366	5360	36,89	-0,54	3,85E-01
ENSRNOG00000027849	**Wipf2**	WAS/WASL interacting protein family,	360620	10	86591015	86607288	16274	535,48	0,28	3,85E-01
ENSRNOG00000042485	**Bdp1**	B double prime 1, subunit of RNA poly	294687	2	49486780	49499375	12596	41,99	0,52	3,85E-01
ENSRNOG00000034007	**Nr3c2**	nuclear receptor subfamily 3, group C,	25672	19	45289894	45639834	349941	285,18	-0,50	3,86E-01
ENSRNOG00000010091	**Efcab14**	EF-hand calcium binding domain 14 (E	298425	5	138378955	138416616	37662	911,98	-0,45	3,86E-01
ENSRNOG00000022698	**Vsig10**	V-set and immunoglobulin domain con	304529	12	46787917	46819404	31488	77,30	0,49	3,86E-01
ENSRNOG00000006744	**Acyp1**	acylphosphatase 1, erythrocyte (comn	299203	6	116453047	116459981	6935	399,40	-0,39	3,86E-01
ENSRNOG00000029510	**Plxnb1**	Protein Plxnb1 [Source:UniProtKB/TrE	316009	8	117190501	117208237	17737	2478,11	-0,52	3,86E-01
ENSRNOG00000034124	**Erich2**	glutamate-rich 2 (Erich2), mRNA [Sou	499806	3	63420706	63456910	36205	42,28	-0,63	3,87E-01
ENSRNOG00000012727	**Trpc7**	transient receptor potential cation char	282822	17	10316807	10456371	139565	33,75	-0,60	3,87E-01
ENSRNOG00000001396	**Mospd3**	motile sperm domain containing 3 (Mc	288557	12	24182698	24186561	3864	1175,60	0,38	3,87E-01
ENSRNOG00000030273	**Zfp563**	zinc finger protein 563 (Zfp563), mRN.	314584	7	15593481	15601988	8508	237,01	-0,43	3,87E-01
ENSRNOG00000013733	**Ppp4r1**	protein phosphatase 4, regulatory sub	140943	9	113066545	113105527	38983	2975,78	0,33	3,87E-01
ENSRNOG00000017512	**Aldh3b1**	aldehyde dehydrogenase 3 family, me	309147	1	226018064	226038888	20825	40,53	-0,62	3,87E-01
ENSRNOG00000001807	**Sspn**	sarcospan (Sspn), mRNA [Source:Ref	500364	4	244453710	244486642	32933	359,04	-0,62	3,88E-01
ENSRNOG00000006885	**LOC10091**	Protein Dbx2 [Source:UniProtKB/TrEI	541457	7	136605951	136636284	30334	120,56	-0,65	3,88E-01
ENSRNOG00000012031	**St8sia2**	ST8 alpha-N-acetyl-neuraminide alpha	117523	1	136353283	136423629	70347	1219,56	-0,47	3,88E-01
ENSRNOG00000018416	**Ttbk1**	tau tubulin kinase 1 (Ttbk1), mRNA [S	316229	9	15761885	15798787	36903	1837,28	0,41	3,88E-01
ENSRNOG00000004499	**Gpr135**	G protein-coupled receptor 135 (Gpr1	314213	6	104261635	104263008	1374	88,16	-0,50	3,88E-01
ENSRNOG00000008444	**Cbl**	Protein Cbl [Source:UniProtKB/TrEMI	500985	8	47107861	47183368	75508	747,70	-0,31	3,88E-01
ENSRNOG00000011498	**Psip1**	PC4 and SFRS1 interacting protein 1	313323	5	105568811	105600711	31901	3603,79	-0,52	3,88E-01
ENSRNOG00000012228	**Skap2**	src kinase associated phosphoprotein	155183	4	146485400	146635663	150264	52,10	-0,51	3,88E-01
ENSRNOG00000030269		ATPase, Ca++ transporting, plasma membrane		4	209001178	209104640	103463	2987,14	-0,36	3,88E-01
ENSRNOG00000042233	**Rpl41**	ribosomal protein L41 (Rpl41), mRNA	124440	7	2946560	2947551	992	10622,14	0,51	3,88E-01
ENSRNOG00000012831	**Slc25a26**	solute carrier family 25 (S-adenosylme	362403	4	191036169	191129236	93068	283,49	0,48	3,88E-01
ENSRNOG00000012923	**Rprd1b**	regulation of nuclear pre-mRNA doma	311591	3	161050868	161097240	46373	1260,25	0,35	3,88E-01

ENSRNOG00000015076	**Cyp26b1**	cytochrome P450, family 26, subfamily	312495	4	180848038	180864857	16820	271,37	-0,48	3,88E-01
ENSRNOG00000016777	**R3hcc1**	Protein R3hcc1 [Source:UniProtKB/Tr	361064	15	55096848	55113932	17085	2278,86	0,29	3,88E-01
ENSRNOG00000021312	**Heatr2**	HEAT repeat containing 2 (Heatr2), ml	304332	12	19532497	19570964	38468	378,29	0,39	3,88E-01
ENSRNOG00000045651	**LOC10090**	acidic (leucine-rich) nuclear phosphop	25379	3	69745835	69780603	34769	702,52	0,35	3,88E-01
ENSRNOG00000047505	**Tubb4a**	tubulin, beta 4A class IVa (Tubb4a), m	29213	9	8960772	8968172	7401	7996,92	0,54	3,88E-01
ENSRNOG00000038297	**Plekhd1**	pleckstrin homology domain containing	500685	6	112512063	112543110	31048	146,78	-0,58	3,88E-01
ENSRNOG00000006076	**Steap2**	STEAP family member 2, metalloredu	312052	4	25414631	25434255	19625	231,27	-0,54	3,88E-01
ENSRNOG00000024823	**Aifm2**	apoptosis-inducing factor, mitochondri	361843	20	33137031	33152531	15501	394,72	-0,56	3,88E-01
ENSRNOG00000049761	**Htr6**	5-hydroxytryptamine (serotonin) recep	64354	5	161242626	161257707	15082	24,57	-0,62	3,88E-01
ENSRNOG00000003254	**Il23a**	Interleukin 23, alpha subunit p19 (Il23a	155140	7	2689128	2691242	2115	23,89	-0,61	3,88E-01
ENSRNOG00000012427	**Veph1**	ventricular zone expressed PH domain	361954	2	177231518	177513886	282369	254,38	-0,65	3,89E-01
ENSRNOG00000020916	**Tmub2**	transmembrane and ubiquitin-like dom	303567	10	90017228	90021390	4163	2129,96	0,45	3,89E-01
ENSRNOG00000026110	**Scml4**	sex comb on midleg-like 4 (Drosophila	309859	20	49258821	49350697	91877	282,72	-0,57	3,89E-01
ENSRNOG00000026299	**Mysm1**	Protein Mysm1 [Source:UniProtKB/Tr	298247	5	117853355	117885569	32215	195,32	-0,51	3,89E-01
ENSRNOG00000045843	**Rusc2**	Protein LOC684259 [Source:UniProtK	684259	5	63395643	63407290	11648	5841,16	0,35	3,89E-01
ENSRNOG00000003923	**Rab21**	RAB21, member RAS oncogene famil	299799	7	58270894	58295672	24779	1303,58	-0,37	3,89E-01
ENSRNOG00000009910	**Swap70**	SWAP switching B-cell complex 70 (S	293410	1	181861342	181922717	61376	813,90	-0,50	3,89E-01
ENSRNOG00000017468	**Trappc6a**	trafficking protein particle complex 6A	680465	1	81683535	81690450	6916	406,24	0,51	3,89E-01
ENSRNOG00000018794	**Card9**	caspase recruitment domain family, m	64171	3	9159494	9167941	8448	58,31	0,53	3,89E-01
ENSRNOG00000000258	**Prmt7**	protein arginine methyltransferase 7 (F	361402	19	49056853	49106846	49994	1446,35	0,48	3,89E-01
ENSRNOG00000006779	**Crot**	carnitine O-octanoyltransferase (Crot)	83842	4	22018242	22053050	34809	450,64	-0,55	3,89E-01
ENSRNOG00000013823	**Cers3**	ceramide synthase 3 (Cers3), mRNA [	499174	1	128723370	128856461	133092	41,99	0,70	3,89E-01
ENSRNOG00000007533	**Rab27a**	RAB27A, member RAS oncogene fam	50645	8	73311542	73366647	55106	63,05	-0,72	3,90E-01
ENSRNOG00000034089	**Ttc21a**	Protein Ttc21a-ps1 [Source:UniProtK	301065	8	127824200	127857313	33114	51,13	-0,58	3,90E-01
ENSRNOG00000003071	**Vamp4**	vesicle-associated membrane protein	364033	13	85352970	85375551	22582	1207,70	-0,44	3,90E-01
ENSRNOG00000016190	**Coq9**	coenzyme Q9 homolog (S. cerevisiae)	498909	19	10578663	10591651	12989	1177,73	0,30	3,90E-01
ENSRNOG00000007728	**Gsdmd**	gasdermin D (Gsdmd), mRNA [Source	315084	7	116782280	116786859	4580	109,71	0,58	3,91E-01
ENSRNOG00000014933	**Dnttip1**	deoxynucleotidyltransferase, terminal,	171437	3	167398374	167421692	23319	1095,15	0,27	3,91E-01
ENSRNOG00000001979	**Rcan1**	regulator of calcineurin 1 (Rcan1), mR	266766	11	36145270	36155595	10326	2669,22	0,26	3,92E-01
ENSRNOG00000012499		retinoic acid receptor, gamma (Rarg),	685072	7	141637841	141660460	22620	144,58	0,44	3,92E-01
ENSRNOG00000031917				11	27338577	27339430	854	798,31	0,33	3,92E-01
ENSRNOG00000011254		family with sequence similarity 76, me	362618	5	154755365	154784446	29082	881,55	0,32	3,92E-01
ENSRNOG00000015128	**Sae1**	SUMO1 activating enzyme subunit 1 (	308384	1	79530175	79585932	55758	4523,70	0,37	3,92E-01
ENSRNOG00000008610	**Mbip**	MAP3K12 binding inhibitory protein 1	362740	6	86758207	86775445	17239	164,83	-0,62	3,92E-01
ENSRNOG00000024594	**Fhdc1**	FH2 domain containing 1 (Fhdc1), mR	295161	2	202860614	202896104	35491	270,29	-0,34	3,92E-01
ENSRNOG00000001884	**Ranbp1**	RAN binding protein 1 (Ranbp1), mRN	360739	11	89984523	89992756	8234	1815,18	0,37	3,92E-01
ENSRNOG00000004089		ectonucleotide pyrophosphatase/phosphodiester		7	95120407	95180901	60495	269,80	-0,67	3,92E-01
ENSRNOG00000004139		nudE nuclear distribution gene E hom	170845	10	55153459	55186775	33317	2811,27	0,61	3,92E-01

ENSRNOG00000008921	**Dynll2**	dynein light chain LC8-type 2 (Dynll2),	140734	10	74829855	74837879	8025	17356,75	0,44	3,92E-01
ENSRNOG00000010076	**Pkp1**	plakophilin 1 (Pkp1), mRNA [Source:R	304822	13	57753232	57800892	47661	116,25	-0,71	3,92E-01
ENSRNOG00000022008	**Taf1a**	TATA box binding protein (Tbp)-associ	360893	13	100975811	101010930	35120	256,76	0,48	3,92E-01
ENSRNOG00000031397	**Stk32b**	serine/threonine kinase 32B (Stk32b),	305431	14	77807749	78072471	264723	543,25	-0,36	3,92E-01
ENSRNOG00000037478	**Noc4l**	nucleolar complex associated 4 homol	360828	12	53810417	53814955	4539	866,66	0,41	3,92E-01
ENSRNOG00000020667	**Shisa5**	shisa family member 5 (Shisa5), mRN	301013	8	117145034	117147664	2631	1210,77	0,46	3,93E-01
ENSRNOG00000031420	**Hyal2**	hyaluronoglucosaminidase 2 (Hyal2),	64468	8	115681546	115685238	3693	1053,84	0,41	3,93E-01
ENSRNOG00000039559	**Fam219a**	family with sequence similarity 219, m	691024	5	62425370	62474180	48811	3071,93	0,32	3,93E-01
ENSRNOG00000007972		Putative hydrolase RBBP9 [Source:U	29459	3	145131500	145138290	6791	399,73	-0,46	3,93E-01
ENSRNOG00000031671	**Rasgef1a**	Protein Rasgef1a [Source:UniProtKB/	312664	4	216061371	216070484	9114	325,76	-0,46	3,93E-01
ENSRNOG00000040275	**Tmem210**	Protein Tmem210; RCG45731, isoforr	1E+08	3	2486107	2487079	973	21,52	0,72	3,94E-01
ENSRNOG00000019956	**Kdm4a**	lysine (K)-specific demethylase 4A (Kc	313539	5	140755996	140802764	46769	4760,38	0,36	3,94E-01
ENSRNOG00000027869	**Sox5**	SRY (sex determining region Y)-box 5 (Sox5), m		4	242271079	242371664	100586	302,46	-0,37	3,94E-01
ENSRNOG00000039871	**RGD13060**	similar to HT021 (RGD1306063), mRN	289928	15	16900269	16915111	14843	256,67	-0,39	3,94E-01
ENSRNOG00000014948	**Osgin1**	oxidative stress induced growth inhibit	171493	19	62825694	62834030	8337	147,23	-0,69	3,94E-01
ENSRNOG00000016281	**Col4a1**	collagen, type IV, alpha 1 (Col4a1), ml	290905	16	82987857	83097435	109579	1153,21	-0,27	3,94E-01
ENSRNOG00000047009	**Pag1**	phosphoprotein associated with glycos	64019	2	114018219	114030540	12322	153,94	-0,51	3,94E-01
ENSRNOG00000018061		Protein Tmem80; Transmembrane protein 80 [S		1	221234795	221243404	8610	792,09	0,36	3,94E-01
ENSRNOG00000010219	**Ralgds**	ral guanine nucleotide dissociation stir	29622	3	12460022	12481565	21544	3055,58	0,31	3,95E-01
ENSRNOG00000029191	**LOC68506**	guanylate binding protein 7 [Source:M	685067	2	266748515	266769711	21197	41,09	0,69	3,95E-01
ENSRNOG00000031232	**Nrp2**	neuropilin 2 (Nrp2), mRNA [Source:Re	81527	9	69307750	69419329	111580	1204,39	-0,43	3,95E-01
ENSRNOG00000001470	**Limk1**	LIM domain kinase 1 (Limk1), mRNA [	65172	12	27037225	27070940	33716	2530,59	0,50	3,95E-01
ENSRNOG00000003549	**Cnpy2**	canopy FGF signaling regulator 2 (Cnp	685814	7	2716178	2720781	4604	1513,17	0,36	3,95E-01
ENSRNOG00000003935		SERTA domain-containing protein 4 [S	360899	13	116128415	116133122	4708	218,89	-0,42	3,95E-01
ENSRNOG00000005580	**Itgb4**	integrin, beta 4 (Itgb4), mRNA [Source	25724	10	103725177	103761362	36186	27,72	-0,63	3,95E-01
ENSRNOG00000011066	**March6**	Protein March6 [Source:UniProtKB/Tr	294862	2	104209952	104281884	71933	4403,84	-0,36	3,95E-01
ENSRNOG00000012938	**Psmb3**	proteasome (prosome, macropain) sul	29676	10	85437464	85444560	7097	2633,57	0,54	3,95E-01
ENSRNOG00000013764	**Fam118a**	family with sequence similarity 118, m	300120	7	125652051	125678576	26526	335,95	0,56	3,95E-01
ENSRNOG00000046172		RIKEN cDNA 0610009D07 gene [Source:MGI S		6	39886871	39895187	8317	690,72	-0,35	3,95E-01
ENSRNOG00000011540	**Vta1**	Vps20-associated 1 homolog (S. cere	292640	1	10447776	10495500	47725	2634,27	0,41	3,95E-01
ENSRNOG00000014508	**Mgll**	monoglyceride lipase (Mgll), mRNA [S	29254	4	185912083	186013125	101043	2048,68	-0,68	3,95E-01
ENSRNOG00000016423	**Tacc1**	transforming, acidic coiled-coil contain	306562	16	71284600	71335563	50964	2981,15	0,27	3,95E-01
ENSRNOG00000017836	**Rrp36**	Protein Rrp36; RCG43434, isoform CI	1E+08	9	15525626	15532535	6910	428,98	0,40	3,95E-01
ENSRNOG00000017299		PHD and ring finger domains 1 (Phrf1)	245925	1	221133586	221166439	32854	2317,59	0,26	3,96E-01
ENSRNOG00000032463	**Rap1a**	RAP1A, member of RAS oncogene fa	295347	2	227614907	227691823	76917	1082,02	-0,33	3,96E-01
ENSRNOG00000012358	**Pcyt1b**	phosphate cytidylyltransferase 1, choli	286936	X	63347319	63411196	63878	827,59	-0,42	3,96E-01
ENSRNOG00000018657	**Scrn3**	secernin 3 (Scrn3), mRNA [Source:Re	311731	3	66507926	66525084	17159	93,00	-0,49	3,96E-01
ENSRNOG00000037718	**Wdr16**	WD repeat domain 16 (Wdr16), mRNA	303233	10	54216435	54257416	40982	511,01	-0,71	3,96E-01

ENSRNOG00000020543		nitric oxide synthase-interacting protei	292894	1	102120682	102135946	15265	1423,67	0,37	3,96E-01
ENSRNOG00000019007	**Rpl14**	ribosomal protein L14 (Rpl14), mRNA	65043	8	128438421	128441259	2839	4301,80	0,28	3,96E-01
ENSRNOG00000030475	**Mum1l1**	melanoma associated antigen (mutate	501630	X	110446523	110450318	3796	43,77	-0,63	3,96E-01
ENSRNOG00000046949	**Kcnb1**	potassium voltage gated channel, Sha	25736	3	170010756	170094516	83761	869,98	-0,30	3,96E-01
ENSRNOG00000048915	**Twf2**	twinfilin actin-binding protein 2 (Twf2),	684352	8	114259815	114272429	12615	757,19	0,41	3,96E-01
ENSRNOG00000001342		WD repeat domain 66 [Source:MGI Sy	304498	12	40586228	40648587	62360	67,22	-0,70	3,96E-01
ENSRNOG00000009061	**RGD13106**	UPF0704 protein C6orf165 homolog [	366347	5	54717643	54762483	44841	19,39	-0,71	3,96E-01
ENSRNOG00000020862	**Ptpn23**	protein tyrosine phosphatase, non-rec	117552	8	117972419	117994931	22513	2187,36	0,28	3,96E-01
ENSRNOG00000026504	**Fam114a1**	Protein Fam114a1 [Source:UniProtKE	498366	14	44756277	44815155	58879	96,69	-0,51	3,96E-01
ENSRNOG00000042389	**Bcl10**	B-cell CLL/lymphoma 10 (Bcl10), mRN	83477	2	270330540	270340108	9569	897,53	0,29	3,96E-01
ENSRNOG00000032150	**Adcy2**	adenylate cyclase 2 (brain) (Adcy2), m	81636	1	38887240	39080014	192775	1905,45	-0,51	3,97E-01
ENSRNOG00000010904	**Kansl2**	KAT8 regulatory NSL complex subunit	300206	X	114700526	114716824	16299	1601,50	0,37	3,98E-01
ENSRNOG00000028166	**Asmtl**	acetylserotonin O-methyltransferase-li	288527	12	20517050	20519715	2666	1297,11	0,40	3,98E-01
ENSRNOG00000016452	**Creb3**	cAMP responsive element binding pro	298400	5	63588152	63592782	4631	1996,25	0,51	3,98E-01
ENSRNOG00000049087	**Zfp622**	zinc finger protein 622 (Zfp622), mRN.	294846	2	97978551	97992639	14089	431,43	0,52	3,98E-01
ENSRNOG00000002841	**Cdc42bpa**	CDC42 binding protein kinase alpha ((	114116	13	103238053	103453991	215939	3640,25	-0,34	3,98E-01
ENSRNOG00000011339	**Slk**	STE20-like kinase (Slk), mRNA [Sourc	54308	1	274792996	274845812	52817	532,48	-0,51	3,98E-01
ENSRNOG00000005872		Protein Tcf7; Transcription factor 7, T-(	363595	10	37389827	37419231	29405	49,15	-0,54	3,99E-01
ENSRNOG00000010872	**Ckb**	creatine kinase, brain (Ckb), mRNA [S	24264	6	144270887	144273773	2887	35446,02	0,33	3,99E-01
ENSRNOG00000011891	**Atp6v1b2**	ATPase, H transporting, lysosomal V1	117596	16	22221014	22244664	23651	13417,98	0,56	3,99E-01
ENSRNOG00000020249	**P4htm**	Protein P4htm; Similar to prolyl-4-hydr	301008	8	116709604	116727218	17615	871,17	0,42	3,99E-01
ENSRNOG00000005850	**Gdap1**	ganglioside-induced differentiation-ass	312890	5	1323590	1342573	18984	1017,21	-0,51	3,99E-01
ENSRNOG00000020618	**Rpl13a**	ribosomal protein L13A (Rpl13a), mRN	317646	1	102186220	102188911	2692	1839,27	0,42	3,99E-01
ENSRNOG00000045543	**Frat2**	Protein LOC679110 [Source:UniProtK	679110	1	268542217	268542909	693	114,41	0,56	3,99E-01
ENSRNOG00000005853	**Tacr1**	tachykinin receptor 1 (Tacr1), mRNA [S	24807	4	177782405	178095041	312637	67,84	-0,62	4,00E-01
ENSRNOG00000018468	**Ldb1**	LIM domain binding 1 (Ldb1), mRNA [	309447	1	273215591	273223127	7537	3541,74	0,28	4,00E-01
ENSRNOG00000024544	**Six3**	SIX homeobox 3 (Six3), mRNA [Sourc	78974	6	8811894	8814540	2647	164,58	0,40	4,00E-01
ENSRNOG00000007643	**Ccm2**	cerebral cavernous malformation 2 (C	305505	14	80446452	80492850	46399	2027,66	0,42	4,00E-01
ENSRNOG00000015221		eukaryotic translation initiation factor 3	293427	1	180523036	180532243	9208	5034,81	0,41	4,00E-01
ENSRNOG00000016702	**Gsg1l**	Germ cell-specific gene 1-like protein	499263	1	204256603	204329093	72491	324,99	-0,61	4,00E-01
ENSRNOG00000001687	**Crybg3**	Protein Crybg3; Putative uncharacteri:	288204	11	46134583	46196066	61484	143,57	-0,45	4,01E-01
ENSRNOG00000003597	**Tuba4a**	tubulin, alpha 4A (Tuba4a), mRNA [So	316531	9	82184866	82188549	3684	1624,13	-0,30	4,01E-01
ENSRNOG00000003879	**Rnf167**	ring finger protein 167 (Rnf167), mRN.	360554	10	57013969	57018254	4286	2300,00	0,43	4,01E-01
ENSRNOG00000004940	**Rnf215**	ring finger protein 215 (Rnf215), mRN.	305478	14	85050141	85056544	6404	1210,12	0,28	4,01E-01
ENSRNOG00000013806	**Mplkip**	M-phase specific PLK1 interacting pro	684996	17	48011755	48046661	34907	552,18	0,38	4,01E-01
ENSRNOG00000016046	**Hecw1**	HECT, C2 and WW domain containing	291209	17	50929146	51469507	540362	1224,32	-0,47	4,01E-01
ENSRNOG00000016392	**Zfp593**	zinc finger protein 593 (Zfp593), mRN.	298546	5	156221537	156223865	2329	595,92	0,34	4,01E-01
ENSRNOG00000016717	**Gas2**	growth arrest-specific 2 (Gas2), mRN/	499156	1	108304939	108405240	100302	57,80	-0,52	4,01E-01

ENSRNOG00000017397	**Upf3a**	UPF3 regulator of nonsense transcript	361176	16	81009176	81020604	11429	852,55	0,30	4,01E-01
ENSRNOG00000021224	**Mrps26**	mitochondrial ribosomal protein S26 (M	362216	3	129591392	129593055	1664	1072,15	0,29	4,01E-01
ENSRNOG00000022576	**Nudt12**	nudix (nucleoside diphosphate linked m	367323	9	105282133	105294901	12769	221,98	-0,48	4,01E-01
ENSRNOG00000027008		interferon gamma induced GTPase [S	303163	10	43407161	43446882	39722	262,34	0,68	4,01E-01
ENSRNOG00000014025	**Rasgrf1**	RAS protein-specific guanine nucleotic	192213	8	96778646	96905924	127279	792,97	-0,46	4,01E-01
ENSRNOG00000021137	**Kctd15**	potassium channel tetramerization dor	499129	1	91645833	91659091	13259	1284,10	-0,36	4,01E-01
ENSRNOG00000022599		Protein RGD1308908 [Source:UniPro	288514	12	18941636	18965326	23691	1973,04	0,28	4,01E-01
ENSRNOG00000008755	**Acox1**	acyl-CoA oxidase 1, palmitoyl (Acox1)	50681	10	103537778	103562620	24843	4661,29	-0,35	4,01E-01
ENSRNOG00000039567	**Trmt10c**	tRNA methyltransferase 10 homolog C	304012	11	50229232	50234308	5077	417,62	-0,46	4,01E-01
ENSRNOG00000014366	**Shc3**	SHC (Src homology 2 domain containi	114858	17	15743125	15862911	119787	975,66	-0,41	4,01E-01
ENSRNOG00000028448	**Elovl1**	ELOVL fatty acid elongase 1 (Elovl1),	679532	5	141046751	141049945	3195	559,73	0,34	4,01E-01
ENSRNOG00000017227	**Ppme1**	protein phosphatase methylesterase 1	361613	1	171538086	171582879	44794	2665,31	0,38	4,01E-01
ENSRNOG00000020038	**Chpf**	chondroitin polymerizing factor (Chpf),	316533	9	82438465	82443159	4695	3978,44	0,35	4,01E-01
ENSRNOG00000001852		endoplasmic reticulum-Golgi intermed	297728	4	246724725	246734389	9665	91,88	-0,58	4,01E-01
ENSRNOG00000003205	**Ldb2**	LIM domain binding 2 (Ldb2), mRNA [	289664	14	70817550	71145367	327818	3002,11	-0,44	4,02E-01
ENSRNOG00000015192	**Mrps5**	mitochondrial ribosomal protein S5 (M	296134	3	127150028	127166611	16584	965,16	0,28	4,02E-01
ENSRNOG00000016483	**Myo16**	myosin XVI (Myo16), mRNA [Source:F	192253	16	84026826	84387681	360856	2506,51	-0,35	4,02E-01
ENSRNOG00000012194	**Morc4**	Protein Morc4 [Source:UniProtKB/TrE	315914	3	53431375	53449418	18044	49,55	-0,48	4,02E-01
ENSRNOG00000019729	**Jmjd8**	jumonji domain containing 8 (Jmjd8), r	360498	10	15008912	15011811	2900	743,18	0,43	4,02E-01
ENSRNOG00000037245	**Gdi1**	GDP dissociation inhibitor 1 (Gdi1), m	25183	1	152140883	152147543	6661	22522,80	0,40	4,02E-01
ENSRNOG00000001640	**Tomm70a**	translocase of outer mitochondrial mei	304017	11	48672524	48706365	33842	674,44	-0,49	4,03E-01
ENSRNOG00000014477	**Acvr2b**	activin A receptor, type IIB (Acvr2b), m	25366	8	127293865	127325514	31650	194,08	0,48	4,03E-01
ENSRNOG00000020159	**Dalrd3**	DALR anticodon binding domain conta	363146	8	116700446	116703321	2876	510,25	0,30	4,03E-01
ENSRNOG00000021099	**Lysmd1**	LysM, putative peptidoglycan-binding,	499671	2	215799038	215804338	5301	1054,55	0,47	4,03E-01
ENSRNOG00000005249	**Snx6**	sorting nexin 6 (Snx6), mRNA [Source	362738	6	85166825	85210033	43209	1748,71	0,32	4,03E-01
ENSRNOG00000007111		nardilysin 1 (Nrd1), mRNA [Source:Re	25499	5	132471876	132534126	62251	3632,77	0,32	4,03E-01
ENSRNOG00000012747	**Spock1**	sparc/osteonectin, cwcv and kazal-like	306759	17	9966212	9981063	14852	1057,96	-0,40	4,03E-01
ENSRNOG00000014597	**Irs1**	insulin receptor substrate 1 (Irs1), mR	25467	9	87782499	87835248	52750	429,98	-0,32	4,03E-01
ENSRNOG00000019276	**RGD73502**	SEL1 domain containing protein RGD	307480	18	31076952	31090582	13631	1635,74	0,29	4,03E-01
ENSRNOG00000019579	**Edc3**	Protein Edc3 [Source:UniProtKB/TrEM	315708	8	62263516	62302963	39448	808,55	0,40	4,03E-01
ENSRNOG00000009870	**Tmem88**	transmembrane protein 88 (Tmem88),	497936	10	55740605	55742300	1696	54,13	0,50	4,03E-01
ENSRNOG00000048277		serrate RNA effector molecule homolog (Arabido		12	24473101	24486246	13146	1796,05	0,34	4,03E-01
ENSRNOG00000027859	**Tmem26**	transmembrane protein 26 (Tmem26),	309724	20	23740900	23789900	49001	45,98	0,60	4,03E-01
ENSRNOG00000011631	**Fst**	follistatin (Fst), mRNA [Source:RefSec	24373	2	65577804	65583544	5741	113,74	-0,47	4,03E-01
ENSRNOG00000011914	**Dtnb**	dystrobrevin, beta (Dtnb), mRNA [Sou	362715	6	37788347	37986143	197797	627,13	-0,29	4,03E-01
ENSRNOG00000046343		RIKEN cDNA D630023F18 gene [Sou	1E+08	9	70719963	70735720	15758	108,54	0,53	4,03E-01
ENSRNOG00000000047	**Cd82**	Cd82 molecule (Cd82), mRNA [Source	83628	3	89026124	89070127	44004	716,30	-0,68	4,04E-01
ENSRNOG00000018033	**Ddx19a**	DEAD (Asp-Glu-Ala-Asp) box polypep	292022	19	54077938	54100118	22181	1571,59	0,28	4,04E-01

ENSRNOG00000027833	**Fnip2**	folliculin interacting protein 2 (Fnip2), r	310538	2	197564860	197670260	105401	624,55	-0,32	4,04E-01
ENSRNOG00000026050	**Epb41l4a**	erythrocyte membrane protein band 4.	307514	18	26284360	26371053	86694	432,96	-0,29	4,05E-01
ENSRNOG00000000091	**Smad9**	SMAD family member 9 (Smad9), mR	85435	2	163609443	163625679	16237	71,68	-0,46	4,05E-01
ENSRNOG00000009759	**Cln5**	ceroid-lipofuscinosis, neuronal 5 (Cln5	306128	2	86329690	86336522	6833	92,05	0,50	4,05E-01
ENSRNOG00000014030	**Synm**	synemin, intermediate filament protein	308709	1	129760413	129787766	27354	1279,33	-0,48	4,05E-01
ENSRNOG00000017178	**Hydin**	Protein Hydin [Source:UniProtKB/TrE	292017	19	51863280	51954772	91493	88,78	-0,70	4,05E-01
ENSRNOG00000029071	**Unc5c**	unc-5 homolog C (C. elegans) (Unc5c	362049	2	265573556	265919845	346290	325,66	-0,41	4,05E-01
ENSRNOG00000013519	**Tll2**	tolloid-like 2 (Tll2), mRNA [Source:Ref	365460	1	267791567	267916973	125407	23,80	-0,63	4,06E-01
ENSRNOG00000026211	**Mri1**	methylthioribose-1-phosphate isomera	288912	19	36336479	36342471	5993	867,23	0,37	4,06E-01
ENSRNOG00000011927		syndecan-3 precursor [Source:RefSe	116673	5	152628005	152658676	30672	9152,78	-0,37	4,06E-01
ENSRNOG00000019577	**Adamts19**	ADAM metallopeptidase with thrombo:	361332	18	53154607	53337093	182487	28,16	-0,65	4,06E-01
ENSRNOG00000001862	**Ube2l3**	ubiquitin-conjugating enzyme E2L 3 (Ube2l3), m		11	91100273	91139662	39390	4510,90	0,25	4,07E-01
ENSRNOG00000006180	**Pum2**	pumilio homolog 2 (Drosophila) (Pum2	298874	6	43569102	43648162	79061	4949,43	-0,39	4,07E-01
ENSRNOG00000007905		integrin, alpha 7 (Itga7), mRNA [Sourc	81008	7	3325443	3354361	28919	333,94	-0,63	4,07E-01
ENSRNOG00000017819	**Cd14**	CD14 molecule (Cd14), mRNA [Sourc	60350	18	29265353	29266946	1594	23,40	-0,70	4,07E-01
ENSRNOG00000019087	**Fam174a**	family with sequence similarity 174, m	301634	9	102475547	102493695	18149	1085,00	0,35	4,07E-01
ENSRNOG00000020570	**Spred3**	sprouty-related, EVH1 domain contain	308478	1	89279385	89288184	8800	284,93	0,44	4,07E-01
ENSRNOG00000045698	**Lin7c**	lin-7 homolog C (C. elegans) (Lin7c), r	60442	3	107611000	107621494	10495	2284,25	-0,62	4,07E-01
ENSRNOG00000003201				X	72916124	72919512	3389	17,69	-0,62	4,07E-01
ENSRNOG00000006986	**RGD13046**	similar to RIKEN cDNA 2700097O09 (	314128	6	85483144	85531929	48786	100,04	-0,39	4,07E-01
ENSRNOG00000007237	**Id2**	inhibitor of DNA binding 2 (Id2), mRNA	25587	6	53083849	53086239	2391	4555,80	-0,32	4,07E-01
ENSRNOG00000007644	**Mbtps2**	membrane-bound transcription factor p	302705	X	40569195	40616823	47629	352,51	-0,46	4,07E-01
ENSRNOG00000008877	**Lsm14b**	Protein Lsm14b [Source:UniProtKB/T	1E+08	3	181701084	181710021	8938	2006,78	0,44	4,07E-01
ENSRNOG00000013413	**Rorb**	RAR-related orphan receptor B (Rorb)	309288	1	241465471	241535552	70082	215,11	-0,53	4,07E-01
ENSRNOG00000014689		Protein Zbtb4 [Source:UniProtKB/TrE	287441	10	56127304	56133262	5959	953,28	-0,36	4,07E-01
ENSRNOG00000020902	**G6pc3**	glucose 6 phosphatase, catalytic, 3 (G	303565	10	89921496	89925728	4233	971,53	0,35	4,07E-01
ENSRNOG00000001873	**P2rx6**	purinergic receptor P2X, ligand-gated	25041	11	90486479	90496513	10035	179,19	-0,54	4,07E-01
ENSRNOG00000005960		expressed sequence AW551984 [Source:MGI Sy		8	43303075	43322826	19752	472,58	-0,43	4,08E-01
ENSRNOG00000007649	**Dnm2**	dynamin 2 (Dnm2), mRNA [Source:Re	25751	8	22513045	22594143	81099	2135,17	-0,28	4,08E-01
ENSRNOG00000013795	**Asb7**	ankyrin repeat and SOCS box-contain	365277	1	128632992	128674393	41402	317,86	0,36	4,08E-01
ENSRNOG00000004220	**Ebag9**	estrogen receptor binding site associa	299864	7	83579394	83597339	17946	327,91	-0,31	4,08E-01
ENSRNOG00000033740	**Lurap1l**	leucine rich adaptor protein 1-like (Lur	362535	5	102512705	102558783	46079	126,51	0,42	4,08E-01
ENSRNOG00000006844	**Dnajc14**	DnaJ (Hsp40) homolog, subfamily C, r	114481	7	3201890	3213835	11946	1029,77	0,49	4,08E-01
ENSRNOG00000008870	**Atf7ip**	Protein Atf7ip [Source:UniProtKB/TrE	312800	4	234777034	234818058	41025	1991,16	0,27	4,08E-01
ENSRNOG00000023261	**Ube2q2l**	ubiquitin-conjugating enzyme E2Q fan	317341	X	11568936	11570054	1119	25,05	-0,59	4,08E-01
ENSRNOG00000016541	**Enc1**	ectodermal-neural cortex 1 (Enc1), mF	294674	2	47160036	47171848	11813	19292,60	-0,41	4,08E-01
ENSRNOG00000003392	**Grsf1**	G-rich RNA sequence binding factor 1	305256	14	20949817	20966234	16418	2201,09	-0,27	4,09E-01
ENSRNOG00000010748	**Mtus1**	microtubule associated tumor suppres	306487	16	54044790	54098279	53490	292,11	-0,34	4,09E-01

ENSRNOG00000021663	**RGD15618**	similar to RIKEN cDNA 3110035E14 (I	500393	5	14155561	14181247	25687	470,66	-0,62	4,09E-01
ENSRNOG00000014353	**Sympk**	symplekin (Sympk), mRNA [Source:Re	292683	1	81190967	81219274	28308	2153,01	0,29	4,09E-01
ENSRNOG00000019585	**Kat8**	K (lysine) acetyltransferase 8 (Kat8), r	310194	1	206383425	206395703	12279	792,96	0,42	4,10E-01
ENSRNOG00000019552	**Lsm7**	LSM7 homolog, U6 small nuclear RNA	362829	7	11892677	11895103	2427	1290,74	0,45	4,10E-01
ENSRNOG00000023850	**Tbrg1**	transforming growth factor beta regula	300521	8	40115616	40123274	7659	119,55	0,44	4,10E-01
ENSRNOG00000018529	**Csnk1g2**	casein kinase 1, gamma 2 (Csnk1g2),	65278	7	12112483	12130793	18311	3426,98	0,44	4,10E-01
ENSRNOG00000016254	**Sema4c**	sema domain, immunoglobulin domair	301346	9	42783321	42793776	10456	1448,90	0,42	4,10E-01
ENSRNOG00000018453	**Nop2**	NOP2 nucleolar protein (Nop2), mRNA	314969	4	224663955	224675686	11732	570,23	0,34	4,10E-01
ENSRNOG00000037250	**Ubl4a**	ubiquitin-like 4A (Ubl4a), mRNA [Sour	293864	1	152081056	152083906	2851	1761,44	0,39	4,10E-01
ENSRNOG00000004196	**LOC10091**	ribosomal protein S29 (Rps29), mRNA	25348	6	100914108	100915476	1369	2248,46	0,40	4,10E-01
ENSRNOG00000007884	**Bcap29**	B-cell receptor-associated protein 29 (	298943	6	59555828	59595518	39691	448,49	-0,55	4,10E-01
ENSRNOG00000018117	**Ndufv1**	NADH dehydrogenase (ubiquinone) fla	293655	1	226124946	226130037	5092	3617,27	0,29	4,11E-01
ENSRNOG00000017342	**Zdhhc7**	zinc finger, DHHC-type containing 7 (Z	170906	19	63480614	63497760	17147	1088,26	0,36	4,11E-01
ENSRNOG00000021553	**Nckap5**	Protein Nckap5 [Source:UniProtKB/Tr	363974	13	47371394	47850408	479015	375,25	0,40	4,11E-01
ENSRNOG00000016748	**Poll**	polymerase (DNA directed), lambda (F	361767	1	272729885	272738343	8459	1225,54	0,35	4,11E-01
ENSRNOG00000002093	**Tgfbr3**	transforming growth factor, beta recep	29610	14	3509601	3683814	174214	97,24	-0,57	4,11E-01
ENSRNOG00000007284	**Slc2a1**	solute carrier family 2 (facilitated gluco	24778	5	141964114	141992634	28521	989,59	-0,47	4,11E-01
ENSRNOG00000013320	**RGD15619**	Protein RGD1561916 [Source:UniPro	500441	5	62411598	62423642	12045	126,59	-0,58	4,11E-01
ENSRNOG00000021100	**Tnfaip8l2**	tumor necrosis factor, alpha-induced p	310663	2	215804336	215820349	16014	27,85	-0,61	4,11E-01
ENSRNOG00000014908	**Sf3b5**	splicing factor 3b, subunit 5 (Sf3b5), m	680891	1	8739602	8740282	681	1998,59	0,39	4,11E-01
ENSRNOG00000049714		ArfGAP with SH3 domain, ankyrin repeat and PH		5	158326041	158364909	38869	39,03	-0,62	4,11E-01
ENSRNOG00000001888	**Arvcf**	armadillo repeat gene deleted in velo-(	303798	11	89830069	89887729	57661	2044,66	-0,39	4,12E-01
ENSRNOG00000013865	**Zmym6**	zinc finger, MYM-type 6 (Zmym6), mR	362602	5	148909099	148956295	47197	486,43	-0,46	4,12E-01
ENSRNOG00000014193	**LOC10091**	ligase I, DNA, ATP-dependent (Lig1), r	81513	1	76727601	76762379	34779	203,49	0,37	4,12E-01
ENSRNOG00000016201	**Mrps9**	mitochondrial ribosomal protein S9 (M	301371	9	49317820	49375391	57572	886,27	0,35	4,12E-01
ENSRNOG00000016451	**Cd1d1**	CD1d1 molecule (Cd1d1), mRNA [Sou	25109	2	205728725	205732232	3508	21,48	0,65	4,12E-01
ENSRNOG00000007775	**Cmpk1**	cytidine monophosphate (UMP-CMP)	298410	5	137551830	137578247	26418	2512,76	-0,28	4,12E-01
ENSRNOG00000018484	**Plk3**	polo-like kinase 3 (Plk3), mRNA [Sour	58936	5	139790975	139796149	5175	422,96	0,45	4,12E-01
ENSRNOG00000021003		SAC3 domain containing 1 [Source:MGI Symbol		1	228418634	228420960	2327	675,85	0,46	4,12E-01
ENSRNOG00000011467	**Uck1**	uridine-cytidine kinase 1 (Uck1), mRN	311864	3	16620744	16626627	5884	1919,32	0,28	4,12E-01
ENSRNOG00000017723	**Cept1**	choline/ethanolamine phosphotransfer	310773	2	228528581	228569428	40848	475,07	-0,47	4,12E-01
ENSRNOG00000022274	**Slc2a8**	solute carrier family 2, (facilitated gluc	85256	3	17356862	17366510	9649	1125,11	0,31	4,12E-01
ENSRNOG00000027238	**Jrk**	jerky homolog (mouse) (Jrk), mRNA [S	315073	7	115847177	115851860	4684	565,71	0,35	4,12E-01
ENSRNOG00000030579		zinc finger protein 853 [Source:MGI Symbol;Acc		12	15364519	15369102	4584	402,86	-0,35	4,12E-01
ENSRNOG00000006813	**Sumf1**	sulfatase modifying factor 1 (Sumf1), r	362409	4	204606895	204688360	81466	756,44	0,52	4,12E-01
ENSRNOG00000011542	**Apopt1**	apoptogenic 1, mitochondrial (Apopt1)	299341	6	144205803	144230967	25165	827,87	0,41	4,12E-01
ENSRNOG00000018337	**Caly**	calcyon neuron-specific vesicular prot	192349	1	219452504	219463693	11190	3203,67	0,36	4,12E-01
ENSRNOG00000018856		collagen, type IV, alpha 6 [Source:MG	363458	X	110869703	111224978	355276	212,69	-0,49	4,12E-01

ENSRNOG00000023668	**Scyl1**	SCY1-like 1 (S. cerevisiae) (Scyl1), m	293684	1	228048825	228062617	13793	2445,02	0,28	4,12E-01
ENSRNOG00000030124	**Ptpn11**	protein tyrosine phosphatase, non-rec	25622	12	42762630	42822760	60131	7421,41	-0,24	4,12E-01
ENSRNOG00000000781	**Rnf39**	ring finger protein 39 (Rnf39), mRNA [	171387	20	4135089	4140578	5490	132,42	0,60	4,12E-01
ENSRNOG00000004810		phospholipase C, beta 1 (phosphoinos	24654	3	134642689	134801508	158820	1071,93	-0,41	4,12E-01
ENSRNOG00000005749	**Foxred2**	FAD-dependent oxidoreductase doma	315112	7	119167570	119179999	12430	225,12	-0,36	4,12E-01
ENSRNOG00000012333	**Kbtbd11**	kelch repeat and BTB (POZ) domain c	306617	16	79275079	79276980	1902	413,54	0,33	4,12E-01
ENSRNOG00000025580	**LOC67953!**	ubiquitin-conjugating enzyme E2 varia	296390	3	170494658	170517510	22853	262,75	0,38	4,13E-01
ENSRNOG00000007299	**Caap1**	caspase activity and apoptosis inhibitc	500501	5	117425195	117477566	52372	254,77	-0,43	4,13E-01
ENSRNOG00000027175	**Cnpy4**	canopy FGF signaling regulator 4 (Cnp	363886	12	21386612	21392755	6144	339,65	0,47	4,13E-01
ENSRNOG00000001216	**Trpm2**	transient receptor potential cation chai	294329	20	13606332	13651945	45614	102,14	0,45	4,13E-01
ENSRNOG00000005517	**LOC68894**	ribosomal protein S26 (Rps26), mRNA	27139	7	3025736	3027286	1551	122,08	0,38	4,13E-01
ENSRNOG00000010731	**Gpm6a**	glycoprotein m6a (Gpm6a), mRNA [Sc	306439	16	39144855	39253973	109119	17321,09	-0,36	4,13E-01
ENSRNOG00000045779	**Rgag4**	Protein LOC100911832; RCG36266 [	1E+08	X	72803663	72805468	1806	359,88	-0,44	4,14E-01
ENSRNOG00000009233	**Ankrd49**	ankyrin repeat domain 49 (Ankrd49), r	315434	8	13240207	13244890	4684	243,57	-0,42	4,14E-01
ENSRNOG00000010763	**Fam181b**	family with sequence similarity 181, m	499205	1	164164156	164165644	1489	1091,63	0,51	4,14E-01
ENSRNOG00000001228	**Pofut2**	protein O-fucosyltransferase 2 (Pofut2	309686	20	14281734	14292670	10937	974,73	0,36	4,14E-01
ENSRNOG00000005347	**Fjx1**	four jointed box 1 (Drosophila) (Fjx1),	366140	3	98940094	98941443	1350	2274,02	0,40	4,14E-01
ENSRNOG00000019987	**Scand1**	SCAN domain-containing 1 (Scand1),	362252	3	159308252	159309116	865	2705,15	0,58	4,15E-01
ENSRNOG00000008177	**Pim2**	pim-2 oncogene (Pim2), mRNA [Sourc	317366	X	16243856	16249125	5270	835,72	0,40	4,15E-01
ENSRNOG00000034140	**Htra4**	HtrA serine peptidase 4 (Htra4), mRN	306564	16	71438259	71451530	13272	54,87	-0,62	4,15E-01
ENSRNOG00000005070	**Spopl**	speckle-type POZ protein-like (Spopl),	296532	3	396612	427083	30472	142,81	-0,51	4,15E-01
ENSRNOG00000019349	**Sf3a2**	splicing factor 3a, subunit 2 (Sf3a2), m	299620	7	11945541	11951564	6024	1036,21	0,34	4,15E-01
ENSRNOG00000000610	**Cisd1**	CDGSH iron sulfur domain 1 (Cisd1),	294362	20	20659781	20673155	13375	3127,74	0,37	4,16E-01
ENSRNOG00000002414	**Tfcp2l1**	transcription factor CP2-like 1 (Tfcp2l1	304741	13	39741256	39797758	56503	82,11	-0,64	4,16E-01
ENSRNOG00000004165	**Prkd1**	protein kinase D1 (Prkd1), mRNA [Sou	85421	6	80601006	80914043	313038	403,20	-0,46	4,16E-01
ENSRNOG00000019551	**Psmb6**	proteasome (prosome, macropain) sul	29666	10	56888980	56891231	2252	875,87	0,50	4,16E-01
ENSRNOG00000036917	**Amn1**	antagonist of mitotic exit network 1 hoi	302032	4	247795755	247822690	26936	673,76	-0,29	4,16E-01
ENSRNOG00000039297	**Mrpl52**	mitochondrial ribosomal protein L52 (M	361037	15	36955394	36958618	3225	1401,38	0,38	4,16E-01
ENSRNOG00000039556	**Rpp25l**	ribonuclease P/MRP 25 subunit-like (F	298002	5	62620312	62621688	1377	335,29	0,31	4,16E-01
ENSRNOG00000001930	**Ccdc50**	coiled-coil domain containing 50 (Ccd	288022	11	83920150	83976562	56413	518,62	-0,47	4,16E-01
ENSRNOG00000020430	**Wdr11**	Protein Wdr11 [Source:UniProtKB/TrE	309016	1	209788417	209832826	44410	1983,23	0,39	4,16E-01
ENSRNOG00000016921	**Klhl11**	kelch-like family member 11 (Klhl11), r	287706	10	88171942	88182499	10558	1051,43	-0,41	4,16E-01
ENSRNOG00000016374	**Fgfr2**	fibroblast growth factor receptor 2 (Fgf	25022	1	207626734	207731841	105108	2190,00	-0,48	4,17E-01
ENSRNOG00000000962	**Slc15a4**	solute carrier family 15 (oligopeptide tr	246280	12	34660965	34680418	19454	1028,65	0,31	4,17E-01
ENSRNOG00000006871	**Slc25a14**	solute carrier family 25 (mitochondrial	85263	X	135541374	135579545	38172	576,48	0,53	4,17E-01
ENSRNOG00000004078	**Eno3**	enolase 3, beta, muscle (Eno3), mRN	25438	10	57023888	57029247	5360	75,51	0,64	4,18E-01
ENSRNOG00000013687	**Ablim1**	Protein Ablim1 [Source:UniProtKB/TrE	307989	1	285260913	285379156	118244	543,40	-0,38	4,18E-01
ENSRNOG00000011151	**Tenm4**	teneurin transmembrane protein 4 (Te	308831	1	167615228	168094355	479128	2409,53	-0,42	4,18E-01

ENSRNOG00000018946	**Trim33**	Protein Trim33 [Source:UniProtKB/TrI	365894	2	225244514	225287933	43420	788,90	-0,49	4,19E-01
ENSRNOG00000045636	**Fasn**	fatty acid synthase (Fasn), mRNA [Sou	50671	10	109579049	109597219	18171	18535,96	-0,45	4,19E-01
ENSRNOG00000007967	**Sdhb**	succinate dehydrogenase complex, su	298596	5	163197045	163217312	20268	5160,42	0,31	4,20E-01
ENSRNOG00000000279	**Rtn4ip1**	reticulon 4 interacting protein 1 (Rtn4ip	309912	20	50523654	50565353	41700	252,66	0,45	4,20E-01
ENSRNOG00000011261	**Ttc14**	tetratricopeptide repeat domain 14 (Tt	310314	2	139921787	139931470	9684	207,85	-0,50	4,20E-01
ENSRNOG00000020729	**Stc2**	stanniocalcin 2 (Stc2), mRNA [Source	63878	10	16432189	16441955	9767	48,59	-0,61	4,20E-01
ENSRNOG00000024930	**Smim19**	Protein Smim19 [Source:UniProtKB/T	1E+08	16	74042995	74055583	12589	727,45	-0,34	4,20E-01
ENSRNOG00000038228	**Serp2**	stress-associated endoplasmic reticulu	498546	15	62376861	62400750	23890	2229,43	0,50	4,20E-01
ENSRNOG00000010628	**Sec13**	SEC13 homolog (S. cerevisiae) (Sec1	297522	4	208982590	208995976	13387	1011,63	0,28	4,20E-01
ENSRNOG00000042086	**Rab26**	RAB26, member RAS oncogene famil	171111	10	13711256	13715749	4494	73,89	-0,59	4,21E-01
ENSRNOG00000004487	**Nek2l1**	NIMA (never in mitosis gene a)-relatec	114482	13	115071864	115082965	11102	28,66	0,61	4,21E-01
ENSRNOG00000001658	**Kcnj6**	potassium inwardly-rectifying channel,	25743	11	38617934	38693513	75580	139,78	-0,49	4,21E-01
ENSRNOG00000004741	**RGD15600**	ribosomal protein L19 (Rpl19), mRNA	81767	10	85767184	85774814	7631	5668,88	0,35	4,21E-01
ENSRNOG00000005346	**Tmem128**	transmembrane protein 128 (Tmem12	360952	14	77252258	77261289	9032	1525,16	0,24	4,21E-01
ENSRNOG00000007496	**Mtmr4**	myotubularin related protein 4 (Mtmr4)	287607	10	75192507	75215438	22932	3544,28	0,40	4,21E-01
ENSRNOG00000010795	**Cnot4**	CCR4-NOT transcription complex, sub	312227	4	62288220	62389482	101263	756,44	-0,28	4,21E-01
ENSRNOG00000011216	**Tbl1xr1**	transducin (beta)-like 1 X-linked recep	365755	2	126968262	127093734	125473	2419,43	-0,31	4,21E-01
ENSRNOG00000015588	**Nol3**	nucleolar protein 3 (apoptosis repress	85383	19	48100723	48102390	1668	435,25	-0,37	4,21E-01
ENSRNOG00000018317	**Aak1**	AP2 associated kinase 1 (Aak1), mRN	500244	4	183225152	183365259	140108	3361,54	-0,38	4,21E-01
ENSRNOG00000019859	**Pla2g15**	phospholipase A2, group XV (Pla2g15	361401	19	48996419	49013714	17296	954,92	0,52	4,21E-01
ENSRNOG00000024250		Protein Wwc3 [Source:UniProtKB/TrE	317439	X	25328900	25397389	68490	528,86	-0,30	4,21E-01
ENSRNOG00000031656	**Ccnh**	cyclin H (Ccnh), mRNA [Source:RefSe	84389	2	13446659	13467469	20811	770,98	-0,45	4,21E-01
ENSRNOG00000047097		Protein LOC686590 [Source:UniProtK	686590	4	187092863	187194136	101274	1693,75	-0,41	4,21E-01
ENSRNOG00000015816	**Bbs7**	Bardet-Biedl syndrome 7 (Bbs7), mRN	361930	2	142898506	142938346	39841	314,30	-0,51	4,21E-01
ENSRNOG00000000841	**Ddx39b**	DEAD (Asp-Glu-Ala-Asp) box polypep	114612	20	6886514	6898958	12445	7241,38	0,22	4,21E-01
ENSRNOG00000015250		methyltransferase like 14 (Mettl14), m	295428	2	246923971	246940051	16081	532,06	-0,35	4,21E-01
ENSRNOG00000022953	**Ccdc163**	coiled-coil domain containing 163 (Ccc	298442	5	139358209	139364237	6029	103,97	0,52	4,21E-01
ENSRNOG00000024349	**Dos**	downstream of Stk11 (Dos), mRNA [S	314622	7	12605828	12611153	5326	2957,04	0,41	4,21E-01
ENSRNOG00000013360	**Prkcsh**	protein kinase C substrate 80K-H (Prk	300445	8	23069740	23081287	11548	4557,92	0,35	4,21E-01
ENSRNOG00000050736				6	122710038	122713987	3950	48,47	-0,49	4,21E-01
ENSRNOG00000010103	**Eif4b**	eukaryotic translation initiation factor 4	300253	7	141476183	141497938	21756	7975,66	0,47	4,21E-01
ENSRNOG00000022237		GTPase activating RANGAP domain-like 3 [Sou		3	17370765	17488893	118129	1052,60	-0,35	4,22E-01
ENSRNOG00000000474	**Rgl2**	ral guanine nucleotide dissociation stir	294283	20	7517123	7524877	7755	998,46	0,32	4,22E-01
ENSRNOG00000007300	**C1qtnf6**	C1q and tumor necrosis factor related	315114	7	119734790	119741304	6515	236,76	-0,63	4,22E-01
ENSRNOG00000010902	**Tceb3**	transcription elongation factor B (SIII),	25562	5	158112243	158128181	15939	1855,37	0,32	4,22E-01
ENSRNOG00000013610	**Chrna5**	cholinergic receptor, nicotinic, alpha 5	25102	8	58143974	58172330	28357	102,10	-0,43	4,22E-01
ENSRNOG00000021702	**Mcmdc2**	minichromosome maintenance domair	500392	5	13787106	13839762	52657	67,07	-0,53	4,22E-01
ENSRNOG00000022929		Myotubularin-related protein 12 [Sour	310155	2	82385608	82452818	67211	274,84	-0,42	4,22E-01

ENSRNOG00000033575	**Emid1**	EMI domain containing 1 (Emid1), mR	685462	14	86034840	86073361	38522	280,75	-0,64	4,22E-01
ENSRNOG00000036934	**Abhd12**	abhydrolase domain containing 12 (Ab	499913	3	152989212	153051233	62022	3742,61	0,24	4,22E-01
ENSRNOG00000042271	**LOC10036**	Protein LOC100360645 [Source:UniP	1E+08	10	48664388	48665064	677	11257,21	0,26	4,22E-01
ENSRNOG00000002991	**Srr**	serine racemase (Srr), mRNA [Source	303306	10	61477875	61493655	15781	621,69	-0,34	4,22E-01
ENSRNOG00000009106	**C2cd4b**	Protein C2cd4b [Source:UniProtKB/Tı	501015	8	77923036	77924058	1023	25,44	-0,57	4,22E-01
ENSRNOG00000024751	**H2afy2**	H2A histone family, member Y2 (H2afy	361844	20	33152565	33200486	47922	2983,00	0,33	4,22E-01
ENSRNOG00000006221	**Uba3**	ubiquitin-like modifier activating enzym	117553	4	194051849	194072884	21036	861,07	-0,48	4,22E-01
ENSRNOG00000006298	**Cpne3**	copine III (Cpne3), mRNA [Source:Ref	313087	5	38183598	38214447	30850	291,68	-0,50	4,22E-01
ENSRNOG00000017181		Protein Malt1 [Source:UniProtKB/TrEl	307366	18	60305268	60356704	51437	68,61	-0,51	4,22E-01
ENSRNOG00000020970	**Rundc3a**	RUN domain containing 3A (Rundc3a)	303569	10	90130416	90139535	9120	4646,55	0,37	4,22E-01
ENSRNOG00000020979	**Psmb4**	proteasome (prosome, macropain) sul	58854	2	215536872	215539684	2813	10807,54	0,46	4,22E-01
ENSRNOG00000042879		SEH1-like (S. cerevisiae [Source:MGI	1E+08	18	62705167	62795437	90271	941,06	0,40	4,22E-01
ENSRNOG00000047811	**Ier2**	immediate early response 2 (Ier2), mF	494344	19	36751280	36751945	666	325,02	0,37	4,22E-01
ENSRNOG00000010085	**Cldn10**	claudin 10 (Cldn10), mRNA [Source:R	290485	15	107450003	107539150	89148	186,38	-0,68	4,23E-01
ENSRNOG00000012164	**Lppr2**	lipid phosphate phosphatase-related p	300443	8	23003383	23013242	9860	3950,92	0,36	4,23E-01
ENSRNOG00000026324	**Stard6**	StAR-related lipid transfer (START) dc	291527	18	68130750	68147692	16943	25,92	-0,56	4,23E-01
ENSRNOG00000029370	**Abhd3**	abhydrolase domain containing 3 (Abh	291793	18	1925174	1979805	54632	2519,96	-0,57	4,23E-01
ENSRNOG00000009691	**Lrrn2**	leucine rich repeat neuronal 2 (Lrrn2),	289020	13	54735286	54801198	65913	3560,62	-0,34	4,24E-01
ENSRNOG00000007574	**Padi2**	peptidyl arginine deiminase, type II (Pa	29511	5	163141202	163182415	41214	148,57	-0,62	4,24E-01
ENSRNOG00000009968		excision repair cross-complementing r	310071	2	58518981	58556315	37335	951,97	0,43	4,24E-01
ENSRNOG00000012160	**Syk**	spleen tyrosine kinase (Syk), mRNA [S	25155	17	14705881	14759165	53285	41,22	-0,54	4,24E-01
ENSRNOG00000026745		acyl-CoA synthetase long-chain family	117243	10	39431075	39488943	57869	1287,15	-0,27	4,24E-01
ENSRNOG00000015285	**Lrp4**	low density lipoprotein receptor-relatec	83469	3	87062818	87116798	53981	2193,61	-0,47	4,24E-01
ENSRNOG00000020254	**Per2**	period circadian clock 2 (Per2), mRNA	63840	9	98231204	98273371	42168	451,86	-0,31	4,24E-01
ENSRNOG00000001191	**Tchp**	trichoplein, keratin filament binding (Tc	304547	12	49431572	49445524	13953	773,24	0,33	4,24E-01
ENSRNOG00000010868	**Rims4**	regulating synaptic membrane exocytc	266976	3	166431305	166490895	59591	547,13	-0,42	4,24E-01
ENSRNOG00000042326	**Smpdl3b**	sphingomyelin phosphodiesterase, aci	362619	5	154592756	154614208	21453	103,88	-0,43	4,24E-01
ENSRNOG00000048718	**Ccdc71**	coiled-coil domain containing 71 (Ccdc	498678	8	116590946	116594850	3905	805,58	0,35	4,24E-01
ENSRNOG00000002345	**Rasgef1b**	Protein Rasgef1b [Source:UniProtKB/	1E+08	14	11944914	11968889	23976	187,62	-0,45	4,25E-01
ENSRNOG00000001357	**Mblac1**	metallo-beta-lactamase domain contai	304346	12	21393471	21394732	1262	215,25	0,30	4,25E-01
ENSRNOG00000011280	**Mllt3**	myeloid/lymphoid or mixed-lineage leu	114510	5	110089251	110183696	94446	368,30	-0,42	4,25E-01
ENSRNOG00000014109	**Psmd13**	proteasome (prosome, macropain) 26	365388	1	220561620	220574027	12408	3752,97	0,29	4,25E-01
ENSRNOG00000033583	**LOC10091**	zinc finger protein 938 [Source:MGI Sy	1E+08	7	10060906	10063355	2450	25,08	-0,66	4,25E-01
ENSRNOG00000013461	**Ralbp1**	ralA binding protein 1 (Ralbp1), mRNA	84014	9	113111338	113130700	19363	1755,72	0,46	4,25E-01
ENSRNOG00000012301	**RGD13103**	similar to RIKEN cDNA 1700026D08 (	308794	1	146836113	146860740	24628	31,25	-0,68	4,25E-01
ENSRNOG00000002121	**Lrrc8d**	leucine rich repeat containing 8 family,	305131	14	5077924	5093047	15124	1846,14	-0,26	4,26E-01
ENSRNOG00000012475	**Prr5**	proline rich 5 (renal) (Prr5), mRNA [Sc	315189	7	125317939	125338782	20844	397,06	-0,42	4,26E-01
ENSRNOG00000008566	**Mrpl15**	mitochondrial ribosomal protein L15 (M	297799	5	19392158	19402384	10227	1263,23	0,36	4,26E-01

ENSRNOG00000007792	**Gcc1**	Protein Gcc1; RCG28300 [Source:Un	1E+08	4	55457677	55461006	3330	439,07	0,38	4,27E-01
ENSRNOG00000019636	**Bscl2**	Berardinelli-Seip congenital lipodystro	361722	1	231974121	231983756	9636	1118,44	0,29	4,27E-01
ENSRNOG00000020977	**LOC10036**	cAMP-regulated phosphoprotein 19 (A	60336	8	118444306	118444644	339	39,14	0,57	4,27E-01
ENSRNOG00000003506	**Gosr2**	golgi SNAP receptor complex membe	64154	10	91503272	91522573	19302	3251,81	0,32	4,27E-01
ENSRNOG00000004911	**Psmd14**	proteasome (prosome, macropain) 26	311078	3	54249938	54344885	94948	2095,88	0,26	4,27E-01
ENSRNOG00000008766	**Grin2b**	glutamate receptor, ionotropic, N-meth	24410	4	233824034	234258927	434894	1321,35	-0,39	4,27E-01
ENSRNOG00000011774	**Fblim1**	filamin binding LIM protein 1 (Fblim1),	362650	5	163841377	163867441	26065	113,10	0,43	4,27E-01
ENSRNOG00000012650	**Mrpl3**	mitochondrial ribosomal protein L3 (M	300974	8	112989706	113013066	23361	1762,03	0,36	4,27E-01
ENSRNOG00000019473	**Dcun1d2**	DCN1, defective in cullin neddylation	688913	16	81075538	81102636	27099	331,73	0,37	4,27E-01
ENSRNOG00000020875	**Celf3**	CUGBP, Elav-like family member 3 (C	499669	2	215197408	215211497	14090	4976,10	0,44	4,27E-01
ENSRNOG00000021919	**Rhoj**	ras homolog family member J (Rhoj),	299145	6	107702937	107785229	82293	140,21	0,36	4,27E-01
ENSRNOG00000029145				MT	15348	15415	68	566,25	-0,49	4,27E-01
ENSRNOG00000000138	**Glis1**	GLIS family zinc finger 1 (Predicted), i	298732	5	130892706	131081379	188674	31,88	-0,66	4,28E-01
ENSRNOG00000005929	**Them6**	thioesterase superfamily member 6 (T	300015	7	115861665	115864841	3177	621,00	0,57	4,28E-01
ENSRNOG00000013474	**Casz1**	Protein Casz1 [Source:UniProtKB/TrE	313713	5	169401538	169425050	23513	35,30	-0,55	4,28E-01
ENSRNOG00000012036	**Pcsk5**	proprotein convertase subtilisin/kexin	116548	1	243339733	243618637	278905	178,51	-0,48	4,28E-01
ENSRNOG00000009935	**Xpo1**	exportin 1, CRM1 homolog (yeast) (X	85252	14	108088677	108124346	35670	3692,09	-0,47	4,28E-01
ENSRNOG00000015632	**Taf1c**	TATA box binding protein (Tbp)-associ	361420	19	62994282	63000801	6520	396,01	0,32	4,28E-01
ENSRNOG00000016879		low density lipoprotein receptor class	679578	18	63182678	63218616	35939	97,12	-0,46	4,28E-01
ENSRNOG00000032206	**Cntnap5b**	contactin associated protein-like 5B (C	301650	13	26870922	27774438	903517	22,31	-0,64	4,28E-01
ENSRNOG00000036835	**Copz1**	coatomer protein complex, subunit ze	315345	7	142680779	142706987	26209	3552,69	-0,42	4,28E-01
ENSRNOG00000047578	**Ccdc28b**	coiled coil domain containing 28B (Cc	682445	5	151552470	151556886	4417	1185,96	0,37	4,28E-01
ENSRNOG00000000341	**Nid2**	nidogen 2 (osteonidogen) (Nid2), mRN	302248	15	8984788	9041180	56393	326,91	-0,34	4,28E-01
ENSRNOG00000002163	**Klf3**	Kruppel-like factor 3 (basic) (Klf3), mR	114845	14	44952288	44974726	22439	1555,18	-0,28	4,28E-01
ENSRNOG00000019742	**Stat3**	signal transducer and activator of tran	25125	10	88584612	88638724	54113	5143,31	0,44	4,29E-01
ENSRNOG00000002146	**Pkd2**	polycystic kidney disease 2 (autosom	498328	14	6581342	6624595	43254	752,67	-0,38	4,29E-01
ENSRNOG00000007404		coiled-coil domain containing 23 (Ccd	362578	5	142055067	142060684	5618	1153,00	0,32	4,29E-01
ENSRNOG00000007817	**Kctd6**	potassium channel tetramerization do	305792	15	22452635	22456037	3403	268,97	-0,36	4,29E-01
ENSRNOG00000013656	**Lpar1**	lysophosphatidic acid receptor 1 (Lpa	116744	5	79707135	79824358	117224	58,94	-0,58	4,29E-01
ENSRNOG00000017560	**Mdk**	midkine (Mdk), mRNA [Source:RefSe	81517	3	87539673	87541584	1912	2172,38	-0,48	4,29E-01
ENSRNOG00000028856		PBX/knotted 1 homeobox 2 (Pknox2),	680549	8	39366205	39463886	97682	591,24	-0,27	4,29E-01
ENSRNOG00000029316	**Gtf2f2**	general transcription factor IIF, polype	81674	15	61647681	61771945	124265	332,27	-0,35	4,29E-01
ENSRNOG00000007781	**Stau1**	staufen double-stranded RNA binding	84496	3	169863711	169909847	46137	2675,26	-0,24	4,29E-01
ENSRNOG00000014882	**Fgf11**	fibroblast growth factor 11 (Fgf11), mR	170632	10	56150464	56153978	3515	218,14	0,38	4,30E-01
ENSRNOG00000016983	**Myh7**	myosin, heavy chain 7, cardiac muscl	29557	15	37521021	37542688	21668	49,27	-0,51	4,30E-01
ENSRNOG00000011535	**Gcsh**	glycine cleavage system protein H (an	171133	19	60313300	60324057	10758	1316,42	-0,42	4,30E-01
ENSRNOG00000021831	**Kcnrg**	potassium channel regulator (Kcnrg),	305947	15	45738076	45742807	4732	90,60	-0,40	4,30E-01
ENSRNOG00000017164		Protein Afap1l2 [Source:UniProtKB/Tr	292130	1	285111675	285205944	94270	315,10	-0,64	4,30E-01

ENSRNOG00000047303	**LOC68298**	mediator of RNA polymerase II transcr	682988	5	154313151	154313705	555	223,67	0,42	4,30E-01
ENSRNOG00000008474	**Acox3**	acyl-CoA oxidase 3, pristanoyl (Acox3	83522	14	79942668	79981341	38674	710,78	0,41	4,30E-01
ENSRNOG00000008091	**Gins1**	GINS complex subunit 1 (Psf1 homolo	499914	3	153056224	153077643	21420	160,11	0,50	4,30E-01
ENSRNOG00000015600	**Prpf6**	pre-mRNA processing factor 6 (Prpf6)	366276	3	180807600	180871660	64061	2739,65	0,41	4,30E-01
ENSRNOG00000017596		thioredoxin-like 4A [Source:MGI Symbol;Acc:MG		18	72067550	72082824	15275	1455,49	0,29	4,30E-01
ENSRNOG00000017895	**Eno1**	enolase 1, (alpha) (Eno1), transcript v	24333	5	170916358	170927263	10906	2354,27	0,32	4,31E-01
ENSRNOG00000046005	**LOC10091**	stearoyl-CoA desaturase (delta-9-des	83792	1	271502294	271515353	13060	133001,98	-0,43	4,31E-01
ENSRNOG00000010921	**Taf1d**	TATA box binding protein (Tbp)-associ	363017	8	13802188	13810920	8733	186,41	-0,59	4,31E-01
ENSRNOG00000032729	**Bex2**	brain expressed X-linked 2 (Bex2), mR	363498	X	106563910	106565438	1529	4878,93	0,35	4,31E-01
ENSRNOG00000047853	**LOC68702**	differentially expressed in B16F10 1 [S	687029	8	129531024	129533588	2565	928,91	-0,40	4,31E-01
ENSRNOG00000009815	**Pnma2**	paraneoplastic Ma antigen 2 (Pnma2),	305977	15	48006292	48013952	7661	1326,26	-0,38	4,31E-01
ENSRNOG00000004950	**Ubxn2a**	UBX domain protein 2A (Ubxn2a), mR	685859	6	40892611	40914105	21495	29,00	-0,56	4,32E-01
ENSRNOG00000005061	**Dlst**	dihydrolipoamide S-succinyltransferas	299201	6	116597173	116620312	23140	4735,04	0,39	4,32E-01
ENSRNOG00000007141	**Preb**	prolactin regulatory element binding (P	58842	6	36599703	36603487	3785	1546,52	0,26	4,32E-01
ENSRNOG00000011164	**Coq6**	coenzyme Q6 homolog, monooxygena	299195	6	117458618	117469955	11338	1255,78	0,29	4,32E-01
ENSRNOG00000011865	**Fam96b**	family with sequence similarity 96, me	680987	19	545968	547833	1866	1053,25	0,38	4,32E-01
ENSRNOG00000019455	**Ap4b1**	adaptor-related protein complex 4, bet	310746	2	225717430	225729412	11983	982,55	0,52	4,32E-01
ENSRNOG00000019568	**Jund**	jun D proto-oncogene (Jund), mRNA [	24518	16	20342643	20343668	1026	2488,19	0,42	4,32E-01
ENSRNOG00000039576	**RGD15656**	suppressor of variegation 3-9 homolog	302553	X	15953775	15967467	13693	543,83	0,27	4,32E-01
ENSRNOG00000003065	**Glyr1**	glyoxylate reductase 1 homolog (Arab	360477	10	9492832	9528292	35461	6408,62	0,33	4,32E-01
ENSRNOG00000031485	**LOC67876**	dipeptidylpeptidase 3 (Dpp3), mRNA [	114591	1	227097125	227120710	23586	366,44	0,30	4,33E-01
ENSRNOG00000001170	**Cox6a1**	cytochrome c oxidase, subunit VIa, po	25282	12	48817758	48820811	3054	15776,27	0,55	4,34E-01
ENSRNOG00000046408		Uncharacterized protein [Source:UniProtKB/TrE		2	103675923	103767985	92063	2653,48	-0,25	4,34E-01
ENSRNOG00000047019		BBSome interacting protein 1 [Source	1E+08	1	282062619	282076548	13930	282,73	-0,46	4,34E-01
ENSRNOG00000050570	**Nrbp1**	nuclear receptor binding protein 1 (Nrb	619579	6	36306032	36316531	10500	2460,53	-0,27	4,34E-01
ENSRNOG00000000438	**Rnf5**	ring finger protein 5, E3 ubiquitin prote	407784	20	6445602	6448046	2445	4626,82	0,44	4,34E-01
ENSRNOG00000003927	**Cd55**	Cd55 molecule (Cd55), mRNA [Source	64036	13	52179638	52206115	26478	18,52	-0,61	4,34E-01
ENSRNOG00000032994		myomesin family, member 3 [Source:M	313625	5	157876941	157930025	53085	19,17	-0,64	4,34E-01
ENSRNOG00000006114	**LOC10091**	RIKEN cDNA A330021E22 gene [Sou	1E+08	4	25445688	25516719	71032	193,73	-0,36	4,34E-01
ENSRNOG00000011991	**Slc10a7**	solute carrier family 10, member 7 (Slc	291942	19	43751269	43972967	221699	110,74	0,41	4,34E-01
ENSRNOG00000025705	**Armcx2**	armadillo repeat containing, X-linked 2	367903	X	105506651	105511422	4772	3518,64	0,39	4,34E-01
ENSRNOG00000002890	**Ankrd45**	Protein Ankrd45 [Source:UniProtKB/T	289152	13	83874383	83886577	12195	206,83	-0,34	4,34E-01
ENSRNOG00000013351		syntaxin binding protein 5 (tomosyn) (	81022	1	5535192	5682141	146950	1310,10	-0,33	4,34E-01
ENSRNOG00000026128	**Cpne8**	copine VIII (Cpne8), mRNA [Source:R	362988	7	131298537	131472968	174432	279,62	-0,50	4,35E-01
ENSRNOG00000013356	**Snapin**	SNAP-associated protein (Snapin), tra	295217	2	209309041	209311590	2550	1982,31	-0,44	4,35E-01
ENSRNOG00000023828	**LOC68087**	cDNA sequence BC024139 [Source:M	680875	7	117166480	117173038	6559	26,59	-0,64	4,35E-01
ENSRNOG00000009601	**LOC10090**	transient receptor potential cation char	89821	8	102955430	103005393	49964	520,26	-0,46	4,35E-01
ENSRNOG00000009760	**Palm**	paralemmin (Palm), mRNA [Source:Re	170673	7	12877762	12963915	86154	8031,10	0,37	4,35E-01

ENSRNOG00000010047	**LOC10036**	DNA-damage-inducible transcript 4-lik	140582	2	261430991	261433647	2657	109,39	-0,65	4,35E-01
ENSRNOG00000010244	**Sarm1**	sterile alpha and TIR motif containing	287545	10	65877570	65901277	23708	2350,13	0,36	4,35E-01
ENSRNOG00000017628	**Tagln**	transgelin (Tagln), mRNA [Source:Ref	25123	8	48847018	48852863	5846	2684,10	0,53	4,35E-01
ENSRNOG00000018426	**LOC10091**	apolipoprotein C-I (Apoc1), transcript v	25292	1	81872117	81875399	3283	121,31	-0,66	4,35E-01
ENSRNOG00000018971	**Mob3a**	MOB kinase activator 3A (Mob3a), mR	362833	7	12046195	12063525	17331	1762,91	0,42	4,35E-01
ENSRNOG00000032240	**Gbp5**	guanylate binding protein 5 (Gbp5), m	362050	2	266707528	266725950	18423	98,12	0,66	4,35E-01
ENSRNOG00000048898		WAS protein family, member 3 [Source	682937	12	12403205	12439680	36476	1283,21	-0,25	4,35E-01
ENSRNOG00000000231	**Kctd17**	potassium channel tetramerization dor	300317	7	119637883	119648656	10774	2423,56	0,34	4,36E-01
ENSRNOG00000000936		syntaxin 2 (Stx2), mRNA [Source:RefS	25130	12	33261860	33285512	23653	378,08	0,31	4,36E-01
ENSRNOG00000018181	**Stk25**	serine/threonine kinase 25 (Stk25), ml	373542	9	100420926	100433656	12731	4907,62	0,37	4,36E-01
ENSRNOG00000037241	**Emd**	emerin (Emd), mRNA [Source:RefSeq	25437	1	152192966	152196018	3053	1323,39	0,26	4,36E-01
ENSRNOG00000012658	**Pdlim3**	PDZ and LIM domain 3 (Pdlim3), mRN	114108	16	49271217	49303147	31931	25,75	0,65	4,36E-01
ENSRNOG00000017023	**Tcf25**	Protein Tcf25 [Source:UniProtKB/TrEI	292082	19	66890051	66921560	31510	8107,62	0,32	4,37E-01
ENSRNOG00000023688	**Drd1**	dopamine receptor D1 (Drd1), mRNA	24316	17	13212535	13214770	2236	87,06	-0,61	4,37E-01
ENSRNOG00000000033	**Tmcc2**	Protein Tmcc2; Similar to RIKEN cDN	305095	13	54201418	54238500	37083	3672,38	0,47	4,37E-01
ENSRNOG00000018690	**Rgs17**	regulator of G-protein signaling 17 (Rg	308118	1	43822671	43918302	95632	258,93	-0,46	4,37E-01
ENSRNOG00000027990	**Crip1**	cysteine-rich protein 1 (intestinal) (Crip	691657	6	146959581	146961385	1805	86,27	-0,67	4,37E-01
ENSRNOG00000012714	**RGD15641**	similar to Protein C21orf58 (RGD1564	499419	20	15085295	15096866	11572	24,23	-0,66	4,37E-01
ENSRNOG00000001931	**Fgf12**	fibroblast growth factor 12 (Fgf12), mR	170630	11	78947043	79210052	263010	548,02	-0,33	4,37E-01
ENSRNOG00000002004	**Prdm8**	Protein Prdm8 [Source:UniProtKB/TrE	305198	14	12997501	13000902	3402	1514,00	0,45	4,37E-01
ENSRNOG00000005902	**Ccdc34**	coiled-coil domain containing 34 (Ccd	362187	3	107756836	107791987	35152	329,79	-0,57	4,37E-01
ENSRNOG00000005451	**Dnah11**	Protein Dnah11 [Source:UniProtKB/Tr	117253	6	154698899	154906395	207497	41,91	-0,64	4,37E-01
ENSRNOG00000021392	**Noc2l**	nucleolar complex associated 2 homol	313777	5	177134961	177146499	11539	1965,15	0,37	4,37E-01
ENSRNOG00000019713	**Wdr24**	WD repeat domain 24 (Wdr24), mRNA	360497	10	15004021	15008803	4783	721,79	0,41	4,37E-01
ENSRNOG00000000840	**Atp6v1g2**	ATPase, H+ transporting, lysosomal V	368044	20	6901681	6903894	2214	3838,21	0,38	4,38E-01
ENSRNOG00000001094	**Zfp316**	zinc finger protein 316 (Zfp316), mRN	304293	12	15394499	15406460	11962	1201,73	0,30	4,38E-01
ENSRNOG00000001117	**LOC10090**	Protein LOC100909844 [Source:UniP	678726	12	15764431	15781260	16830	54,38	0,47	4,38E-01
ENSRNOG00000001148	**Rplp0**	ribosomal protein, large, P0 (Rplp0), n	64205	12	48588977	48592275	3299	34452,40	0,42	4,38E-01
ENSRNOG00000001235	**Gna12**	guanine nucleotide binding protein (G	81663	12	17890088	17970852	80765	6946,12	-0,36	4,38E-01
ENSRNOG00000001241	**Iqce**	Protein Iqce [Source:UniProtKB/TrEM	304318	12	18028345	18065162	36818	619,73	0,30	4,38E-01
ENSRNOG00000003267	**Pycr2**	pyrroline-5-carboxylate reductase fam	364064	13	104182940	104186733	3794	1394,35	0,40	4,38E-01
ENSRNOG00000003328	**Cdip1**	cell death-inducing p53 target 1 (Cdip	360480	10	9734342	9756549	22208	3810,13	0,27	4,38E-01
ENSRNOG00000005018		Sodium channel protein type 2 subunit alpha [S		3	58322638	58454329	131692	2309,41	-0,33	4,38E-01
ENSRNOG00000005076	**Bcorl1**	BCL6 co-repressor-like 1 (Bcorl1), mR	302810	X	135259167	135305318	46152	846,58	-0,32	4,38E-01
ENSRNOG00000005530	**Rps6kl1**	Protein Rps6kl1 [Source:UniProtKB/T	299202	6	116581249	116594985	13737	851,21	0,35	4,38E-01
ENSRNOG00000007655	**Zfp174**	Protein Zfp174 [Source:UniProtKB/Tr	287074	10	10685777	10691026	5250	120,42	0,36	4,38E-01
ENSRNOG00000008138		RIKEN cDNA 4931429I11 gene [Source:MGI Sy		8	43571129	43623841	52713	26,14	-0,64	4,38E-01
ENSRNOG00000016671		Protein Dtna-ps1 [Source:UniProtKB/	307548	18	14341044	14457596	116553	978,17	-0,37	4,38E-01

ENSRNOG00000017174	**LOC10091(**	quaking (Qk), mRNA [Source:RefSeq mRNA;Ac		1	52104976	52209819	104844	170,61	-0,37	4,38E-01
ENSRNOG00000019614	**Ptpn22**	protein tyrosine phosphatase, non-rec	295338	2	225765776	225814130	48355	22,38	0,60	4,38E-01
ENSRNOG00000019861	**Tollip**	toll interacting protein (Tollip), mRNA [	361677	1	221737399	221758265	20867	4431,59	0,38	4,38E-01
ENSRNOG00000020130	**Arhgef1**	Rho guanine nucleotide exchange fac	60323	1	83035506	83053988	18483	794,97	0,31	4,38E-01
ENSRNOG00000020202	**Asrgl1**	asparaginase like 1 (Asrgl1), mRNA [S	246307	1	232246223	232267037	20815	4245,04	-0,40	4,38E-01
ENSRNOG00000020481	**Pafah1b3**	platelet-activating factor acetylhydrola	114113	1	83427896	83430421	2526	2367,31	0,42	4,38E-01
ENSRNOG00000020705	**Rnls**	renalase, FAD-dependent amine oxida	361751	1	259052393	259323697	271305	59,75	0,44	4,38E-01
ENSRNOG00000020713	**Osbpl5**	oxysterol binding protein-like 5 (Osbpl	361686	1	223725412	223779450	54039	1296,72	0,26	4,38E-01
ENSRNOG00000021317	**Cct2**	chaperonin containing TCP1, subunit 2	299809	7	60110076	60122818	12743	4825,67	0,35	4,38E-01
ENSRNOG00000025818	**Zxdc**	Protein Zxdc [Source:UniProtKB/TrEM	362399	4	186823293	186853893	30601	683,80	0,39	4,38E-01
ENSRNOG00000026065	**Slit1**	slit homolog 1 (Drosophila) (Slit1), mR	65047	1	268298680	268445090	146411	1957,94	-0,36	4,38E-01
ENSRNOG00000026937	**Arsk**	arylsulfatase family, member K (Arsk),	365619	2	2836108	2891776	55669	149,24	-0,45	4,38E-01
ENSRNOG00000029207	**Abcb4**	ATP-binding cassette, subfamily B (MI	24891	4	22071627	22128783	57157	171,13	-0,62	4,38E-01
ENSRNOG00000029910		Golgin subfamily A member 4 [Source	501069	8	126393942	126483268	89327	1762,70	0,37	4,38E-01
ENSRNOG00000030091	**Dusp14l1**	dual specificity phosphatase 14 (Dusp	360580	10	71277398	71295766	18369	40,65	-0,61	4,38E-01
ENSRNOG00000037298	**RGD13090**	uncharacterized protein LOC292874 |	292874	1	101411207	101424622	13416	143,21	-0,46	4,38E-01
ENSRNOG00000038102	**Elmod3**	ELMO/CED-12 domain containing 3 (E	297342	4	165194110	165235495	41386	280,02	0,39	4,38E-01
ENSRNOG00000047014	**Homer1**	homer homolog 1 (Drosophila) (Home	29546	2	42107429	42208021	100593	1451,28	-0,40	4,38E-01
ENSRNOG00000048288	**Taf12**	TAF12 RNA polymerase II, TATA box b	682902	5	154126886	154141447	14562	427,13	0,44	4,38E-01
ENSRNOG00000012091	**Ppa2**	pyrophosphatase (inorganic) 2 (Ppa2)	310856	2	257065673	257142749	77077	832,05	0,27	4,38E-01
ENSRNOG00000014485	**Pdcd6**	programmed cell death 6 (Pdcd6), mR	308061	1	33002331	33017997	15667	1214,87	0,38	4,38E-01
ENSRNOG00000022448	**Htra2**	HtrA serine peptidase 2 (Htra2), mRN/	297376	4	178568773	178571933	3161	1103,79	0,25	4,38E-01
ENSRNOG00000031816	**Nckipsd**	NCK interacting protein with SH3 dom	301009	8	116946761	116956823	10063	630,80	0,34	4,38E-01
ENSRNOG00000007290	**Atp1a2**	ATPase, Na+/K+ transporting, alpha 2	24212	13	95173347	95198290	24944	13582,87	-0,48	4,38E-01
ENSRNOG00000030629	**Camkmt**	calmodulin-lysine N-methyltransferase	299521	6	8279200	8656790	377591	320,01	0,46	4,38E-01
ENSRNOG00000015447	**LOC10091:**	calcium binding and coiled coil domair	246047	7	143218503	143233218	14716	42,48	0,55	4,38E-01
ENSRNOG00000018586	**Fam134a**	family with sequence similarity 134, m	363252	9	82115051	82120826	5776	5683,82	0,27	4,38E-01
ENSRNOG00000018639	**Zfand2b**	zinc finger, AN1 type domain 2B (Zfan	363253	9	82140185	82143100	2916	593,59	0,34	4,38E-01
ENSRNOG00000018840	**Rnf40**	ring finger protein 40, E3 ubiquitin prot	266712	1	206060191	206074819	14629	1496,63	0,28	4,38E-01
ENSRNOG00000019288	**Ogfod1**	2-oxoglutarate and iron-dependent oxy	307657	19	11399734	11425518	25785	383,46	0,43	4,38E-01
ENSRNOG00000019749	**Ube2j2**	ubiquitin-conjugating enzyme E2, J2 (I	298689	5	176848287	176862700	14414	1943,85	0,29	4,38E-01
ENSRNOG00000025108		ankyrin repeat domain 35 [Source:MG	365881	2	218284432	218303371	18940	54,59	-0,43	4,38E-01
ENSRNOG00000046793		Uncharacterized protein [Source:UniProtKB/TrE		9	94147593	94213434	65842	249,57	-0,43	4,38E-01
ENSRNOG00000002755	**Pafah1b1**	platelet-activating factor acetylhydrola	83572	10	61186184	61299247	113064	11089,77	-0,35	4,38E-01
ENSRNOG00000020602	**Ndufa13**	NADH dehydrogenase [ubiquinone] 1	1E+08	16	21191327	21198326	7000	1523,84	0,42	4,39E-01
ENSRNOG00000003947	**Ntn1**	netrin 1 (Ntn1), mRNA [Source:RefSe	114523	10	54507536	54711662	204127	529,36	0,50	4,39E-01
ENSRNOG00000008554		solute carrier family 9 (sodium/hydrog	363115	8	101787033	102485049	698017	118,92	0,40	4,39E-01
ENSRNOG00000020058	**Celsr2**	cadherin, EGF LAG seven-pass G-typ	83465	2	230654782	230677391	22610	5566,28	0,31	4,39E-01

ENSRNOG00000004846	**Atp6v1c1**	ATPase, H+ transporting, lysosomal V	299971	7	78536556	78573997	37442	1602,74	-0,38	4,39E-01
ENSRNOG00000018618	**Nfatc3**	nuclear factor of activated T-cells, cytc	361400	19	48906477	48980756	74280	618,42	0,36	4,39E-01
ENSRNOG00000019433	**Rab3a**	RAB3A, member RAS oncogene famil	25531	16	20292237	20296302	4066	7123,15	0,38	4,39E-01
ENSRNOG00000021109	**Ccdc114**	coiled-coil domain containing 114 (Ccc	308594	1	102985867	103016208	30342	58,43	-0,66	4,40E-01
ENSRNOG00000036668	**Ogfod3**	2-oxoglutarate and iron-dependent ox	303749	10	109965251	109993035	27785	392,92	0,34	4,40E-01
ENSRNOG00000025235	**Tmem130**	transmembrane protein 130 (Tmem13	304280	12	13876610	13900956	24347	11028,21	0,48	4,40E-01
ENSRNOG00000047086		Protein LOC683536 [Source:UniProtK	116725	3	181117373	181165752	48380	114,99	0,42	4,40E-01
ENSRNOG00000050453		F-box protein 27 [Source:MGI Symbol;Acc:MGI:		1	88055288	88069759	14472	73,87	0,43	4,40E-01
ENSRNOG00000002563	**Mcts1**	malignant T cell amplified sequence 1	302500	X	124979248	124990620	11373	938,07	-0,26	4,40E-01
ENSRNOG00000001885	**Trmt2a**	tRNA methyltransferase 2 homolog A (	287953	11	89979612	89984220	4609	974,45	0,31	4,40E-01
ENSRNOG00000019857	**Gng7**	guanine nucleotide binding protein (G	58979	7	11792645	11793928	1284	473,47	-0,54	4,40E-01
ENSRNOG00000016976	**Clcn7**	chloride channel, voltage-sensitive 7 (	29233	10	14308276	14332371	24096	1183,59	0,33	4,40E-01
ENSRNOG00000047394	**Ufd1l**	ubiquitin fusion degradation 1 like (yea	84478	11	89406803	89430406	23604	2398,42	0,31	4,40E-01
ENSRNOG00000023376	**Riok3**	RIO kinase 3 (Riok3), mRNA [Source:	361293	18	3574641	3600214	25574	2232,82	0,31	4,41E-01
ENSRNOG00000013506	**Zfand6**	zinc finger, AN1-type domain 6 (Zfand	293067	1	147672440	147750196	77757	676,00	-0,46	4,41E-01
ENSRNOG00000021213	**Lix1l**	Lix1 homolog (mouse)-like (Lix1l), mR	499677	2	218213052	218238919	25868	3344,95	0,25	4,41E-01
ENSRNOG00000015021	**Carkd**	carbohydrate kinase domain containin	361185	16	82791870	82809822	17953	1182,68	0,26	4,41E-01
ENSRNOG00000027787	**Cdc6**	cell division cycle 6 (Cdc6), mRNA [Sc	360621	10	86614893	86628265	13373	139,41	0,43	4,41E-01
ENSRNOG00000023534	**Haus8**	HAUS augmin-like complex, subunit 8	290626	16	19512405	19529269	16865	319,75	0,37	4,42E-01
ENSRNOG00000006241	**March7**	membrane-associated ring finger (C3H	311059	3	51400038	51432100	32063	665,03	-0,58	4,42E-01
ENSRNOG00000007152	**Bhlhe40**	basic helix-loop-helix family, member e	79431	4	205170129	205175829	5701	433,60	-0,50	4,42E-01
ENSRNOG00000012227	**Stambp**	Stam binding protein (Stambp), mRNA	171565	4	179839859	179864344	24486	679,72	0,30	4,43E-01
ENSRNOG00000041717				9	59757832	59758012	181	112,68	-0,51	4,43E-01
ENSRNOG00000006956		Protein Adamtsl1 [Source:UniProtKB/	362539	5	108500765	108737201	236437	113,90	-0,48	4,43E-01
ENSRNOG00000001345	**Mapkapk5**	mitogen-activated protein kinase-activ	498183	12	42377614	42396574	18961	1531,36	0,24	4,43E-01
ENSRNOG00000003225	**Nudcd2**	NudC domain containing 2 (Nudcd2),	287199	10	25741398	25746616	5219	584,81	-0,43	4,43E-01
ENSRNOG00000007648	**Rab11b**	RAB11B, member RAS oncogene fam	79434	7	18776594	18789732	13139	8091,87	0,23	4,43E-01
ENSRNOG00000017155	**Nck2**	NCK adaptor protein 2 (Nck2), mRNA	316369	9	49915383	50040941	125559	1489,94	0,41	4,43E-01
ENSRNOG00000018047	**Gorasp1**	golgi reassembly stacking protein 1 (G	56082	8	127811974	127824020	12047	1699,42	0,25	4,43E-01
ENSRNOG00000020444	**Hcn3**	hyperpolarization-activated cyclic nucl	114245	2	207875210	207888575	13366	622,68	-0,35	4,44E-01
ENSRNOG00000028670	**RGD15627**	similar to Putative protein C21orf62 hc	498060	11	34833565	34848516	14952	93,87	-0,55	4,44E-01
ENSRNOG00000017916	**Rexo1**	REX1, RNA exonuclease 1 homolog (	314630	7	12189793	12209185	19393	2217,27	0,34	4,45E-01
ENSRNOG00000050061		Solute carrier family 25 member 51 [S	313241	5	65453389	65464585	11197	36,60	0,58	4,45E-01
ENSRNOG00000014375	**Bai2**	brain-specific angiogenesis inhibitor 2	313058	5	151919830	151950961	31132	8043,64	-0,40	4,45E-01
ENSRNOG00000001009	**Bri3**	brain protein I3 (Bri3), mRNA [Source:	304284	12	14374635	14383317	8683	1525,33	0,45	4,45E-01
ENSRNOG00000010290		zinc and ring finger 3 [Source:MGI Symbol;Acc:N		14	86199006	86256716	57711	228,43	-0,35	4,45E-01
ENSRNOG00000020063	**Nfkbib**	nuclear factor of kappa light polypeptic	81525	1	88122206	88129977	7772	443,62	0,46	4,45E-01
ENSRNOG00000006139	**Stx18**	syntaxin 18 (Stx18), mRNA [Source:R	360953	14	77384817	77475005	90189	774,89	0,36	4,46E-01

ENSRNOG00000008492	**Ccdc19**	coiled-coil domain containing 19 (Ccdc	304984	13	95428891	95452273	23383	54,77	-0,65	4,46E-01
ENSRNOG00000010676	**Smarce1**	SWI/SNF related, matrix associated, a	303518	10	86913923	86933996	20074	2009,81	0,31	4,46E-01
ENSRNOG00000005522	**Sh3yl1**	SH3 domain containing, Ysc84-like 1 (	362724	6	58531557	58574823	43267	474,23	0,48	4,46E-01
ENSRNOG00000014866	**Pign**	phosphatidylinositol glycan anchor bio	309051	13	30676676	30814931	138256	199,08	-0,49	4,46E-01
ENSRNOG00000028585	**Tceal8**	transcription elongation factor A (SII)-li	367909	X	106411719	106413855	2137	1270,78	-0,39	4,46E-01
ENSRNOG00000000658	**Acacb**	acetyl-CoA carboxylase beta (Acacb),	116719	12	49914427	50000977	86551	38,95	-0,66	4,46E-01
ENSRNOG00000000994	**Stxbp2**	syntaxin binding protein 2 (Stxbp2), m	81804	12	4342248	4353273	11026	96,33	0,49	4,46E-01
ENSRNOG00000004742	**Chp1**	calcineurin-like EF-hand protein 1 (Ch	64152	3	117902988	117938471	35484	530,17	0,47	4,46E-01
ENSRNOG00000015953	**Oaz2**	ornithine decarboxylase antizyme 2 (C	501454	8	70899390	70913165	13776	7032,49	0,29	4,46E-01
ENSRNOG00000019014	**Ndst1**	N-deacetylase/N-sulfotransferase (hep	29633	18	55189496	55226907	37412	2843,90	0,22	4,46E-01
ENSRNOG00000029061	**Hirip3**	HIRA interacting protein 3 (Hirip3), mF	361650	1	205278318	205281341	3024	637,42	0,43	4,46E-01
ENSRNOG00000031081	**Stat2**	signal transducer and activator of tran	288774	7	2670243	2685670	15428	1121,65	0,53	4,46E-01
ENSRNOG00000038085	**Rpe**	ribulose-5-phosphate-3-epimerase (Rp	501157	9	73595054	73615031	19978	547,02	-0,35	4,46E-01
ENSRNOG00000045860	**U2af1**	U2 small nuclear RNA auxiliary factor	687575	20	12582586	12593473	10888	1990,89	0,22	4,46E-01
ENSRNOG00000048406	**Trnau1ap**	tRNA selenocysteine 1 associated pro	65241	5	154159835	154173989	14155	1231,35	0,28	4,46E-01
ENSRNOG00000049900		interferon regulatory factor 2 binding p	679357	19	70172659	70174944	2286	736,64	0,28	4,46E-01
ENSRNOG00000001443	**Rhbdd2**	rhomboid domain containing 2 (Rhbdd	360793	12	26089458	26099384	9927	967,46	0,30	4,46E-01
ENSRNOG00000037283	**Ssr4**	signal sequence receptor, delta (Ssr4)	29435	1	152743845	152747716	3872	2266,46	0,44	4,46E-01
ENSRNOG00000001501	**Snrpa**	small nuclear ribonucleoprotein polype	292729	1	85210610	85219232	8623	806,84	0,39	4,47E-01
ENSRNOG00000005256	**Zc3h15**	zinc finger CCCH-type containing 15 (	362154	3	77550919	77570542	19624	450,69	-0,57	4,47E-01
ENSRNOG00000008479	**Slc8a1**	solute carrier family 8 (sodium/calcium	29715	6	4220847	4481722	260876	1385,56	-0,42	4,47E-01
ENSRNOG00000011829	**Rpgrip1l**	Rpgrip1-like (Rpgrip1l), mRNA [Source	307724	19	28182502	28274100	91599	619,35	-0,42	4,47E-01
ENSRNOG00000012836		Hepatoma-derived growth factor [Sou	114499	2	206679280	206686097	6818	1801,29	0,25	4,47E-01
ENSRNOG00000034116	**Gk**	glycerol kinase (Gk), mRNA [Source:R	79223	X	54426883	54503311	76429	342,92	-0,37	4,47E-01
ENSRNOG00000000249	**Mettl23**	methyltransferase like 23 (Mettl23), m	287918	10	105446488	105451151	4664	236,41	-0,29	4,47E-01
ENSRNOG00000005274	**Vrk1**	vaccinia related kinase 1 (Vrk1), mRN	362779	6	139031649	139098523	66875	379,99	0,37	4,47E-01
ENSRNOG00000008049	**Max**	MYC associated factor X (Max), mRN	60661	6	109378844	109403735	24892	1921,44	0,31	4,47E-01
ENSRNOG00000008812	**Tmem214**	transmembrane protein 214 (Tmem21	362711	6	36684558	36692304	7747	1821,01	0,28	4,47E-01
ENSRNOG00000014903	**Zfyve27**	zinc finger, FYVE domain containing 2	309376	1	268867697	268888292	20596	2622,95	0,34	4,47E-01
ENSRNOG00000029394		dual specificity protein phosphatase 8	361679	1	221943575	221950984	7410	3018,83	0,37	4,47E-01
ENSRNOG00000049612		Uncharacterized protein [Source:UniProtKB/TrE		4	57753251	57766927	13677	31,01	-0,53	4,47E-01
ENSRNOG00000002979	**Tbx19**	T-box 19 (Tbx19), mRNA [Source:Ref	304935	13	88283232	88305361	22130	20,92	0,56	4,47E-01
ENSRNOG00000007492	**Rpn2**	ribophorin II (Rpn2), mRNA [Source:R	64701	3	158274304	158321446	47143	4366,25	0,25	4,47E-01
ENSRNOG00000008140	**LOC10036**	ribosomal protein L15 (Rpl15), mRNA	245981	15	12250806	12252870	2065	4134,16	0,47	4,47E-01
ENSRNOG00000014371	**Cdh13**	cadherin 13 (Cdh13), mRNA [Source:I	192248	19	62076973	62720152	643180	4608,85	-0,44	4,47E-01
ENSRNOG00000016369	**Eci2**	enoyl-CoA delta isomerase 2 (Eci2), m	291075	17	32456608	32473003	16396	607,46	-0,47	4,47E-01
ENSRNOG00000018829	**RGD13081**	similar to RIKEN cDNA 1110020A23 (I	287452	10	56589622	56592266	2645	921,52	0,42	4,47E-01
ENSRNOG00000037435	**Tmem196**	transmembrane protein 196 (Tmem19	500750	6	156784820	156847093	62274	79,79	-0,51	4,47E-01

ENSRNOG00000009334	**Knstrn**	kinetochore-localized astrin/SPAG5 bi	311325	3	117157780	117177432	19653	216,79	0,49	4,48E-01
ENSRNOG00000014637	**Atxn10**	ataxin 10 (Atxn10), mRNA [Source:Re	170821	7	125942296	126065027	122732	10165,32	0,33	4,48E-01
ENSRNOG00000010028	**Polr3d**	polymerase (RNA) III (DNA directed) p	306012	15	55852988	55857657	4670	1320,21	0,39	4,48E-01
ENSRNOG00000013460	**Zfp319**	zinc finger protein 319 (Zfp319), mRN	291849	19	10103086	10107065	3980	733,28	0,29	4,48E-01
ENSRNOG00000003187		armadillo repeat containing, X-linked 5 [Source:M	X		106865066	106866769	1704	272,78	0,50	4,49E-01
ENSRNOG00000011879	**Nfat5**	nuclear factor of activated T-cells 5, to	307820	19	49315752	49403632	87881	1247,56	-0,49	4,49E-01
ENSRNOG00000014302	**Dlgap3**	discs, large (Drosophila) homolog-ass	286923	5	149027227	149073369	46143	2141,47	-0,47	4,49E-01
ENSRNOG00000014999	**Tnpo1**	transportin 1 (Tnpo1), mRNA [Source:	309126	2	48243521	48302954	59434	1713,45	-0,37	4,49E-01
ENSRNOG00000040242	**Epm2a**	epilepsy, progressive myoclonus type	114005	1	7100639	7224740	124102	103,53	-0,54	4,49E-01
ENSRNOG00000026107		cDNA sequence BC030336 [Source:MGI Symbo		1	197530179	197596476	66298	96,64	-0,45	4,49E-01
ENSRNOG00000039928		Uncharacterized protein [Source:UniProtKB/TrE		13	45417135	45418216	1082	31,84	-0,49	4,49E-01
ENSRNOG00000001198	**Fam222a**	family with sequence similarity 222, m	498193	12	49539795	49585147	45353	221,45	0,49	4,49E-01
ENSRNOG00000004005		calcium/calmodulin-dependent protein	24245	14	86893087	86982260	89174	12854,06	-0,40	4,49E-01
ENSRNOG00000012051		non-SMC condensin I complex, subunit H [Sourc		3	126109413	126130107	20695	403,00	0,45	4,49E-01
ENSRNOG00000027770	**Trpm3**	transient receptor potential cation cha	309407	1	247642339	248041913	399575	424,73	-0,43	4,49E-01
ENSRNOG00000009881	**Fam161a**	family with sequence similarity 161, m	289833	14	107861019	107874812	13794	44,49	-0,46	4,49E-01
ENSRNOG00000003972	**LOC10036**	thyroid stimulating hormone receptor (	25360	6	124425325	124561154	135830	248,46	-0,47	4,50E-01
ENSRNOG00000020047	**Tut1**	terminal uridylyl transferase 1, U6 snR	499314	1	232089082	232100064	10983	825,41	0,33	4,50E-01
ENSRNOG00000001035	**Gtf2h3**	general transcription factor IIH, polype	288651	12	39298571	39315037	16467	777,33	0,36	4,51E-01
ENSRNOG00000027332	**Gemin6**	gem (nuclear organelle) associated pr	362688	6	2884038	2888452	4415	173,21	0,32	4,51E-01
ENSRNOG00000027434	**Fitm2**	fat storage-inducing transmembrane p	311617	3	166047354	166053866	6513	871,34	0,27	4,51E-01
ENSRNOG00000049629	**Reep2**	receptor accessory protein 2 (Reep2),	682105	18	27351033	27353125	2093	3274,82	0,29	4,51E-01
ENSRNOG00000022771	**Arhgap23**	Protein Arhgap23 [Source:UniProtKB/	303501	10	85144259	85233279	89021	3347,35	0,34	4,51E-01
ENSRNOG00000018671	**Commd4**	COMM domain containing 4 (Commd4	363068	8	60403480	60406936	3457	2149,37	0,34	4,51E-01
ENSRNOG00000024040	**Mb21d2**	Mab-21 domain containing 2 (Mb21d2	498100	11	78476278	78575233	98956	1222,08	-0,40	4,51E-01
ENSRNOG00000021671	**Ldoc1l**	Protein Ldoc1l [Source:UniProtKB/TrE	300114	7	125195454	125196185	732	671,59	0,27	4,52E-01
ENSRNOG00000000555	**Eif4ebp2**	eukaryotic translation initiation factor 4	361845	20	32841631	33048246	206616	2247,18	0,31	4,52E-01
ENSRNOG00000001837	**Sst**	somatostatin (Sst), mRNA [Source:Re	24797	11	80374262	80375447	1186	5862,64	0,47	4,52E-01
ENSRNOG00000006818	**Arl6ip5**	ADP-ribosylation-like factor 6 interacti	66028	4	194077679	194100520	22842	4593,58	0,34	4,52E-01
ENSRNOG00000014801	**Exog**	endo/exonuclease (5'-3'), endonucleas	301062	8	127340145	127359922	19778	744,88	0,48	4,52E-01
ENSRNOG00000023643	**Mmp17**	matrix metallopeptidase 17 (Mmp17),	288626	12	32636907	32663678	26772	404,08	-0,47	4,52E-01
ENSRNOG00000028207		collagen beta(1-O)galactosyltransferase 2 [Sour		13	74834548	74873161	38614	321,09	-0,47	4,52E-01
ENSRNOG00000028883	**Rpl37a**	ribosomal protein L37a (Rpl37a), transcript varia		10	9642913	9643191	279	371,02	0,35	4,52E-01
ENSRNOG00000033916	**LOC10036**	40S ribosomal protein S21 [Source:U	1E+08	5	163318971	163319321	351	87,62	0,59	4,52E-01
ENSRNOG00000046883	**LOC50128**	DNA segment, Chr 17, Wayne State U	501282	9	9874668	9882697	8030	2477,18	0,28	4,52E-01
ENSRNOG00000047222		importin 8 [Source:MGI Symbol;Acc:MGI:24446		4	247360262	247417663	57402	543,51	0,37	4,52E-01
ENSRNOG00000001867	**Snap29**	synaptosomal-associated protein 29 (	116500	11	90883358	90914051	30694	1504,82	-0,33	4,52E-01
ENSRNOG00000007509		excitatory amino acid transporter 4 [S	84012	7	13893842	13915011	21170	164,64	-0,42	4,52E-01

ENSRNOG00000003203	**Hexim1**	hexamethylene bis-acetamide inducibl	498008	10	90972403	90974513	2111	1368,58	0,42	4,52E-01
ENSRNOG00000005930	**Nnmt**	nicotinamide N-methyltransferase (Nn	300691	8	51518448	51531215	12768	35,54	-0,59	4,52E-01
ENSRNOG00000015054	**Rcbtb2**	regulator of chromosome condensatio	290363	15	58766754	58796461	29708	854,11	-0,30	4,52E-01
ENSRNOG00000017021	**Galnt18**	UDP-N-acetyl-alpha-D-galactosamine	293181	1	183278639	183588615	309977	705,11	-0,52	4,52E-01
ENSRNOG00000048725		LSM2 homolog, U6 small nuclear RNA	684148	20	7040457	7043830	3374	458,22	0,45	4,52E-01
ENSRNOG00000006995	**Ano6**	anoctamin 6 (Ano6), mRNA [Source:R	315272	7	136781652	136973259	191608	871,99	-0,31	4,53E-01
ENSRNOG00000047322	**Crb3**	crumbs homolog 3 (Drosophila) (Crb3	301112	9	8981383	8984483	3101	54,81	0,43	4,53E-01
ENSRNOG00000024494		Protein Gm872 [Source:UniProtKB/TrEMBL;Acc		7	33772559	33862925	90367	53,16	-0,65	4,54E-01
ENSRNOG00000004001	**Osgepl1**	O-sialoglycoprotein endopeptidase-like	314548	9	52711225	52762415	51191	137,70	-0,44	4,54E-01
ENSRNOG00000046889		diazepam binding inhibitor (GABA rece	25045	13	41225979	41283626	57648	17587,98	-0,52	4,54E-01
ENSRNOG00000005765	**Ap4s1**	adaptor-related protein complex 4, sig	366618	6	82026136	82066641	40506	431,37	0,35	4,55E-01
ENSRNOG00000009667	**Mlst8**	MTOR associated protein, LST8 homo	64226	10	13656236	13661987	5752	1016,01	0,35	4,55E-01
ENSRNOG00000014051	**Pgrmc2**	progesterone receptor membrane com	361940	2	147587585	147603481	15897	1844,42	-0,27	4,55E-01
ENSRNOG00000021984	**Rgs7**	regulator of G-protein signaling 7 (Rgs	54296	13	97560710	97772203	211494	1051,04	-0,37	4,55E-01
ENSRNOG00000016037	**Mafb**	v-maf avian musculoaponeurotic fibros	54264	3	162573583	162574554	972	139,11	0,37	4,55E-01
ENSRNOG00000007686	**Sept3**	septin 3 (Sept3), mRNA [Source:RefS	56003	7	123495523	123511261	15739	5492,12	-0,39	4,55E-01
ENSRNOG00000019627	**Mybpc2**	myosin binding protein C, fast-type (M	292879	1	101572091	101595570	23480	19,51	0,60	4,56E-01
ENSRNOG00000001088	**Rfc3**	replication factor C (activator 1) 3 (Rfc	288414	12	1439597	1450351	10755	646,36	0,33	4,56E-01
ENSRNOG00000003541	**Pggt1b**	protein geranylgeranyltransferase type	81746	18	39829209	39871483	42275	291,89	-0,38	4,56E-01
ENSRNOG00000015516	**Cbwd1**	COBW domain containing 1 (Cbwd1),	171057	1	250093369	250134935	41567	104,40	-0,43	4,56E-01
ENSRNOG00000049943		Fam60a protein; Protein Fam60a; RC	686611	4	247533906	247542952	9047	25,41	0,61	4,56E-01
ENSRNOG00000001194		ribosomal RNA processing 1 homolog	309673	20	13016119	13039473	23355	550,34	0,32	4,56E-01
ENSRNOG00000006797	**Zfp280c**	Protein Zfp981 [Source:UniProtKB/Tr	302812	X	135436456	135501067	64612	173,34	-0,48	4,56E-01
ENSRNOG00000009948	**Tmem55b**	transmembrane protein 55B (Tmem55	364298	15	31684579	31688424	3846	2081,62	0,32	4,56E-01
ENSRNOG00000010180	**Gid8**	GID complex subunit 8 (Gid8), mRNA	296466	3	179916193	179922710	6518	1488,25	0,24	4,56E-01
ENSRNOG00000013502	**Eps15l1**	epidermal growth factor receptor path	361120	16	18988863	19064343	75481	1759,04	0,28	4,56E-01
ENSRNOG00000020692	**Tmem216**	transmembrane protein 216 (Tmem21	361727	1	233548121	233551813	3693	317,25	0,33	4,56E-01
ENSRNOG00000033316	**Foxo4**	forkhead box O4 (Foxo4), mRNA [Sou	302415	X	72008079	72014636	6558	717,18	0,37	4,56E-01
ENSRNOG00000009156	**Tra2a**	transformer 2 alpha homolog (Drosopl	500116	4	143594598	143613358	18761	1026,90	-0,35	4,57E-01
ENSRNOG00000021412	**Slfn13**	schlafen family member 13 (Slfn13), m	303378	10	69956876	69968233	11358	152,09	0,56	4,57E-01
ENSRNOG00000001781	**Lmln**	leishmanolysin-like (metallopeptidase	363795	11	73982199	74046319	64121	499,38	0,49	4,57E-01
ENSRNOG00000006989	**Vamp2**	vesicle-associated membrane protein	24803	10	55418597	55422463	3867	23461,57	0,30	4,57E-01
ENSRNOG00000001554	**RGD15638**	uncharacterized protein LOC360692		360692	11	20731051	20783321	52271	107,83	-0,47	4,57E-01
ENSRNOG00000015300	**Spsb3**	splA/ryanodine receptor domain and S	302981	10	14064726	14069831	5106	1358,96	0,27	4,57E-01
ENSRNOG00000023344	**Rpl23a**	ribosomal protein L23a (Rpl23a), mRN	360572	10	66190636	66193322	2687	1203,40	0,27	4,58E-01
ENSRNOG00000046262		RIKEN cDNA 3110082J24 gene [Source:MGI Sy		6	37453680	37453976	297	17,88	0,57	4,58E-01
ENSRNOG00000032075	**Kcnh3**	potassium voltage-gated channel, sub	27150	X	115293665	115414776	121112	1133,21	-0,51	4,58E-01
ENSRNOG00000004531	**Tceanc**	transcription elongation factor A (SII) N	367782	X	29896994	29898070	1077	36,92	-0,55	4,58E-01

ENSRNOG00000018377	**LOC100359956**			19	40711194	40712425	1232	76,22	0,40	4,58E-01
ENSRNOG00000001135	**Wsb2**	WD repeat and SOCS box-containing	288692	12	46762964	46783174	20211	4494,32	-0,40	4,59E-01
ENSRNOG00000001707	**Vwa5b2**	von Willebrand factor A domain contai	303812	11	87064398	87081268	16871	443,93	0,36	4,59E-01
ENSRNOG00000006490	**Neo1**	Neogenin [Source:UniProtKB/TrEMBL	81735	8	63421646	63524303	102658	8868,55	-0,38	4,59E-01
ENSRNOG00000047211	**Fzd3**	frizzled family receptor 3 (Fzd3), mRN	266715	15	52344566	52411769	67204	979,50	-0,47	4,59E-01
ENSRNOG00000050543		scaffold attachment factor B1 [Source	64196	9	9447353	9467936	20584	3165,02	0,28	4,59E-01
ENSRNOG00000007049	**Bcl11a**	B-cell CLL/lymphoma 11A (zinc finger	305589	14	108548150	108632982	84833	1873,54	0,36	4,59E-01
ENSRNOG00000017032	**Atp5a1**	ATP synthase, H+ transporting, mitoch	65262	18	73830653	73838477	7825	22260,50	0,33	4,59E-01
ENSRNOG00000002343	**Uchl1**	ubiquitin carboxyl-terminal esterase L1	29545	14	42928247	42938965	10719	19855,65	0,40	4,60E-01
ENSRNOG00000012294	**Heph**	hephaestin (Heph), mRNA [Source:Re	117240	X	66393130	66488431	95302	42,57	0,59	4,60E-01
ENSRNOG00000012623	**Arf4**	ADP-ribosylation factor 4 (Arf4), mRN/	79120	16	2313285	2330255	16971	9349,90	0,40	4,60E-01
ENSRNOG00000002864	**Nacc1**	nucleus accumbens associated 1, BEI	171454	19	36761771	36777149	15379	2364,11	0,32	4,60E-01
ENSRNOG00000019920	**Doc2a**	double C2-like domains, alpha (Doc2a	65031	1	205264653	205268291	3639	305,79	-0,51	4,60E-01
ENSRNOG00000008024	**Pxk**	PX domain containing serine/threonine	306203	15	22515247	22583048	67802	967,22	0,41	4,60E-01
ENSRNOG00000011596		transient receptor potential cation channel, subfa		3	125783791	125840900	57110	620,42	-0,41	4,60E-01
ENSRNOG00000047255	**Fosl2**	fos-like antigen 2 (Fosl2), transcript va	25446	6	35422541	35440258	17718	372,14	-0,59	4,60E-01
ENSRNOG00000000815	**Smpdl3a**	sphingomyelin phosphodiesterase, aci	294422	20	42510561	42532381	21821	273,22	-0,61	4,61E-01
ENSRNOG00000007326	**Prepl**	prolyl endopeptidase-like (Prepl), mRN	298771	6	8251542	8278693	27152	3053,45	-0,30	4,61E-01
ENSRNOG00000021048	**LOC69105(**	Protein LOC691056 [Source:UniProtKB/TrEMBI		1	102862636	102871754	9119	2821,56	0,37	4,61E-01
ENSRNOG00000014750		DNA segment, Chr 14, Abbott 1 expre:	680155	16	3019039	3073894	54856	517,47	-0,53	4,61E-01
ENSRNOG00000000517	**Kctd20**	Protein Kctd20; Similar to RIKEN cDN	294307	20	8459922	8468395	8474	606,22	0,39	4,61E-01
ENSRNOG00000006824	**Hdac11**	histone deacetylase 11 (Hdac11), mRI	297453	4	187747332	187764590	17259	1403,12	0,36	4,61E-01
ENSRNOG00000011135	**Gtf2b**	general transcription factor IIB (Gtf2b)	81673	2	267245407	267257873	12467	626,56	0,32	4,61E-01
ENSRNOG00000036832	**Itga5**	integrin, alpha 5 (fibronectin receptor,	315346	7	142750121	142773323	23203	63,75	0,42	4,61E-01
ENSRNOG00000036833	**Zfp385a**	zinc finger protein 385A (Zfp385a), tra	685474	7	142719645	142739423	19779	1285,38	0,34	4,61E-01
ENSRNOG00000004161	**Mtif2**	mitochondrial translational initiation fa	305606	14	113621601	113631935	10335	120,63	-0,37	4,61E-01
ENSRNOG00000046428	**Fam211b**	family with sequence similarity 211, m	1E+08	20	16236028	16242159	6132	291,50	0,32	4,61E-01
ENSRNOG00000049531	**Usp19**	ubiquitin specific peptidase 19 (Usp19	361190	8	116625536	116635791	10256	3810,29	0,26	4,61E-01
ENSRNOG00000019772	**Dnpep**	aspartyl aminopeptidase (Dnpep), mR	301529	9	82274791	82283660	8870	3770,00	0,22	4,61E-01
ENSRNOG00000019826	**Sil1**	SIL1 homolog, endoplasmic reticulum	291673	18	27777578	28014973	237396	359,96	0,35	4,61E-01
ENSRNOG00000037655		Glutamyl-tRNA(Gln) amidotransferase	361974	2	203993423	204077216	83794	521,22	0,52	4,61E-01
ENSRNOG00000031995		small integral membrane protein 15 (S	1E+08	2	58399574	58404108	4535	385,05	-0,59	4,61E-01
ENSRNOG00000005099		topoisomerase (DNA) III alpha [Source:MGI Syn		10	46754952	46790713	35762	242,00	0,35	4,62E-01
ENSRNOG00000014148	**Ap5m1**	adaptor-related protein complex 5, mu	305861	15	29856055	29878317	22263	322,63	-0,44	4,62E-01
ENSRNOG00000018704	**Nolc1**	nucleolar and coiled-body phosphoprc	64896	1	273260272	273270982	10711	4832,83	0,28	4,62E-01
ENSRNOG00000045948		ADP-ribosylation factor-like protein 10	306767	17	12693071	12701000	7930	2313,96	0,40	4,62E-01
ENSRNOG00000009808	**Ift80**	intraflagellar transport 80 homolog (Ch	295106	2	184867652	184961866	94215	201,49	-0,48	4,63E-01
ENSRNOG00000037238	**Rnmtl1**	RNA methyltransferase like 1 (Rnmtl1)	360569	10	63609355	63616033	6679	297,64	0,36	4,63E-01

ENSRNOG00000003324		Uncharacterized protein [Source:UniProtKB/TrE		10	9652576	9653493	918	28,19	0,52	4,63E-01
ENSRNOG00000009942	**Cib2**	calcium and integrin binding family me	300719	8	57704422	57720624	16203	500,04	-0,40	4,63E-01
ENSRNOG00000016849	**Ppp2r5d**	Protein LOC100909464 [Source:UniP	363193	9	15493418	15517044	23627	3196,13	0,32	4,63E-01
ENSRNOG00000008735		WD repeat-containing and planar cell	305552	14	106464351	106823366	359016	75,46	-0,46	4,63E-01
ENSRNOG00000010081		transmembrane protein 144 [Source:I	361968	2	197996572	198023399	26828	81,09	-0,51	4,63E-01
ENSRNOG00000015093	**Sparcl1**	SPARC-like 1 (hevin) (Sparcl1), mRN	25434	14	6985393	7016295	30903	25231,60	-0,43	4,63E-01
ENSRNOG00000050091	**Efcab1**	EF hand calcium binding domain 1 (Ef	301957	11	93274702	93287932	13231	106,27	-0,63	4,63E-01
ENSRNOG00000050367				15	91342348	91342452	105	41,88	0,54	4,63E-01
ENSRNOG00000021125	**Prdx5**	peroxiredoxin 5 (Prdx5), mRNA [Sourc	113898	1	229155316	229158301	2986	3820,46	0,39	4,64E-01
ENSRNOG00000002976	**Maats1**	Protein Maats1 [Source:UniProtKB/Tr	303920	11	67063853	67106609	42757	89,63	-0,39	4,64E-01
ENSRNOG00000037483	**Ep400**	E1A binding protein p400 (Ep400), mF	304569	12	53687001	53793498	106498	4264,68	-0,29	4,64E-01
ENSRNOG00000004869		Protein Dach2 [Source:UniProtKB/TrE	302333	X	84868693	85429932	561240	54,28	-0,63	4,64E-01
ENSRNOG00000006569	**Itgb8**	integrin, beta 8 (Itgb8), mRNA [Source	362800	6	156003756	156082274	78519	560,43	-0,57	4,64E-01
ENSRNOG00000013913	**Piezo1**	family with sequence similarity 38, me	361430	19	66014941	66075579	60639	50,86	-0,57	4,64E-01
ENSRNOG00000043328	**Chmp2a**	charged multivesicular body protein 2	365191	1	66352534	66354940	2407	1802,70	0,33	4,64E-01
ENSRNOG00000017822	**Mgea5**	meningioma expressed antigen 5 (hya	154968	1	272938859	272971539	32681	3844,84	-0,33	4,64E-01
ENSRNOG00000019979	**B3galt6**	UDP-Gal:betaGal beta 1,3-galactosylt	298690	5	176900220	176901197	978	208,43	-0,36	4,65E-01
ENSRNOG00000018044	**Phyh**	phytanoyl-CoA 2-hydroxylase (Phyh),	114209	17	78953406	78970294	16889	470,18	-0,41	4,65E-01
ENSRNOG00000020608	**Ppan**	peter pan homolog (Drosophila) (Ppar	298699	8	21962180	21966157	3978	725,76	0,26	4,65E-01
ENSRNOG00000007408	**Ebf4**	early B-cell factor 4 (Ebf4), mRNA [So	680751	3	129335397	129391670	56274	208,24	-0,49	4,65E-01
ENSRNOG00000047046		perilipin 4 [Source:MGI Symbol;Acc:MGI:19297(		9	9950800	9958221	7422	26,87	-0,63	4,65E-01
ENSRNOG00000001596		ATP synthase lipid-binding protein, mi	114630	3	67289968	67292590	2623	7669,41	0,34	4,65E-01
ENSRNOG00000002026	**Dnajc28**	DnaJ (Hsp40) homolog, subfamily C, r	360699	11	35378904	35381723	2820	61,07	-0,50	4,65E-01
ENSRNOG00000014180		Uncharacterized protein [Source:UniProtKB/TrE		9	14234280	14237235	2956	219,38	0,39	4,65E-01
ENSRNOG00000015547	**Jak2**	Janus kinase 2 (Jak2), mRNA [Source	24514	1	254646614	254705434	58821	1067,02	-0,40	4,65E-01
ENSRNOG00000016364	**Gba2**	glucosidase beta 2 (Gba2), mRNA [Sc	298399	5	63592680	63604807	12128	2308,93	0,42	4,65E-01
ENSRNOG00000019028	**Znrf1**	Protein Znrf1; RCG51260, isoform CR	690769	19	54557436	54640013	82578	1438,97	0,36	4,65E-01
ENSRNOG00000023760	**Plekhm3**	Protein Plekhm3 [Source:UniProtKB/	316455	9	70935176	71068434	133259	293,15	0,41	4,65E-01
ENSRNOG00000024757	**Endod1**	Protein Endod1; RCG31867 [Source:	363015	8	12846527	12873120	26594	309,01	0,34	4,65E-01
ENSRNOG00000038420	**Atxn7l3b**	ataxin 7-like 3B (Atxn7l3b), mRNA [So	1E+08	7	55624344	55624637	294	1605,38	0,50	4,66E-01
ENSRNOG00000003634	**Zfp354a**	zinc finger protein 354A (Zfp354a), mF	24522	10	36359537	36370635	11099	106,15	-0,40	4,66E-01
ENSRNOG00000009495	**Src**	v-src avian sarcoma (Schmidt-Ruppin	83805	3	158125180	158138447	13268	2169,39	0,28	4,66E-01
ENSRNOG00000019590	**Smg5**	Protein Smg5 [Source:UniProtKB/TrE	681012	2	207111216	207137075	25860	1874,33	0,33	4,66E-01
ENSRNOG00000000536		MAM domain-containing glycosylphos	309659	20	10707850	10766161	58312	753,61	-0,42	4,66E-01
ENSRNOG00000006087	**Rnf20**	ring finger protein 20, E3 ubiquitin pro	313216	5	69386859	69411969	25111	1650,76	0,41	4,66E-01
ENSRNOG00000018665	**Bud13**	BUD13 homolog (S. cerevisiae) (Bud1	300687	8	49198988	49214820	15833	415,83	0,32	4,66E-01
ENSRNOG00000017427	**Pfn2**	profilin 2 (Pfn2), mRNA [Source:RefSe	81531	2	167359674	167365375	5702	184,74	0,52	4,66E-01
ENSRNOG00000028404	**Ppp1r1b**	protein phosphatase 1, regulatory (inh	360616	10	86100811	86109846	9036	1009,81	-0,54	4,66E-01

ENSRNOG00000007906	**Bub1b**	Protein Bub1b [Source:UniProtKB/TrE	171576	3	116912841	116964033	51193	458,66	0,51	4,67E-01
ENSRNOG00000013729	**RGD13062**	Protein RGD1306271 [Source:UniPro	312246	4	65654521	65777795	123275	1727,20	-0,33	4,67E-01
ENSRNOG00000014352	**Smim12**	small integral membrane protein 12 (S	685634	5	149083705	149083983	279	184,98	0,37	4,67E-01
ENSRNOG00000020280	**Armc6**	armadillo repeat containing 6 (Armc6),	306352	16	20789968	20799539	9572	875,81	0,36	4,67E-01
ENSRNOG00000050806	**LOC10036**	SPT2, Suppressor of Ty, domain conta	1E+08	1	104183116	104194154	11039	176,71	0,42	4,67E-01
ENSRNOG00000016109	**Neurl4**	neuralized homolog 4 (Drosophila) (Ne	303248	10	56257734	56269348	11615	3980,49	0,31	4,67E-01
ENSRNOG00000011555	**Lrrc40**	leucine rich repeat containing 40 (Lrrc	310946	2	283518779	283533188	14410	550,07	-0,38	4,67E-01
ENSRNOG00000014323	**Extl2**	exostosin-like glycosyltransferase 2 (E	310803	2	237037494	237057929	20436	519,34	-0,32	4,68E-01
ENSRNOG00000020693	**Tmem138**	transmembrane protein 138 (Tmem13	361728	1	233569913	233576952	7040	339,25	0,31	4,68E-01
ENSRNOG00000022586	**Cdk9**	cyclin-dependent kinase 9 (Cdk9), mR	362110	3	17081165	17086012	4848	2146,55	0,24	4,68E-01
ENSRNOG00000014634	**Gtpbp1**	GTP binding protein 1 (Gtpbp1), mRN	300077	7	120914233	120938682	24450	2128,74	0,29	4,68E-01
ENSRNOG00000030821	**Wibg**	within bgcn homolog (Drosophila) (Wi	366790	7	3145368	3165714	20347	829,75	0,30	4,69E-01
ENSRNOG00000049882	**Adcyap1**	adenylate cyclase activating polypepti	24166	9	121156295	121162350	6056	938,93	0,58	4,69E-01
ENSRNOG00000001426	**Prkrip1**	Prkr interacting protein 1 (IL11 inducib	498171	12	25515337	25533620	18284	563,67	0,39	4,69E-01
ENSRNOG00000013331	**Sdha**	succinate dehydrogenase complex, su	157074	1	32971779	32996749	24971	3216,20	-0,26	4,69E-01
ENSRNOG00000021085	**Sf1**	splicing factor 1 (Sf1), transcript variar	117855	1	228723306	228736875	13570	4634,88	0,22	4,69E-01
ENSRNOG00000027459	**Zbtb45**	zinc finger and BTB domain containing	308366	1	66375406	66379533	4128	822,35	0,26	4,69E-01
ENSRNOG00000046316	**Tomm6**	Protein Tomm6; RCG43475 [Source:U	681123	9	14237733	14238645	913	2485,41	0,46	4,69E-01
ENSRNOG00000049488		Uncharacterized protein [Source:UniProtKB/TrE		1	149594171	149606009	11839	107,73	-0,37	4,69E-01
ENSRNOG00000009850	**St3gal4**	ST3 beta-galactoside alpha-2,3-sialylt	363040	8	36283787	36333597	49811	696,66	-0,30	4,69E-01
ENSRNOG00000025245	**RGD13119**	similar to RIKEN cDNA 1810055G02 (	309145	1	225819324	225828032	8709	671,45	-0,30	4,69E-01
ENSRNOG00000036814	**Atp6v0a1**	ATPase, H+ transporting, lysosomal V	29757	10	88712837	88766228	53392	12382,65	0,33	4,69E-01
ENSRNOG00000006870	**Mtdh**	metadherin (Mtdh), mRNA [Source:Re	170910	7	72939139	72994607	55469	729,28	-0,34	4,69E-01
ENSRNOG00000013492	**Cap1**	CAP, adenylate cyclase-associated pr	64185	5	144350205	144376518	26314	8968,97	0,32	4,69E-01
ENSRNOG00000048699	**Vps37d**	vacuolar protein sorting 37 homolog D	687208	12	26650992	26656442	5451	1145,33	0,33	4,69E-01
ENSRNOG00000016292	**Pdzd9**	PDZ domain containing 9 (Pdzd9), mF	308954	1	197510331	197523570	13240	32,30	-0,52	4,69E-01
ENSRNOG00000032446	**Recql4**	RecQ protein-like 4 (Recql4), mRNA [S	300057	7	117753871	117761035	7165	173,14	0,36	4,69E-01
ENSRNOG00000003120	**Prelp**	proline/arginine-rich end leucine-rich r	84400	13	55804884	55814122	9239	112,66	-0,44	4,69E-01
ENSRNOG00000014658		zinc finger protein 423 [Source:RefSe	94188	19	31295760	31396060	100301	772,33	-0,38	4,69E-01
ENSRNOG00000046472	**Arfgap3**	ADP-ribosylation factor GTPase activa	503165	7	124136869	124149920	13052	681,29	-0,37	4,69E-01
ENSRNOG00000005620	**Lcp2**	lymphocyte cytosolic protein 2 (Lcp2),	155918	10	18875179	18921942	46764	28,91	0,62	4,69E-01
ENSRNOG00000008101	**Tmem251**	Protein Tmem251; RCG63028 [Sourc	1E+08	6	135828702	135829916	1215	288,26	-0,34	4,69E-01
ENSRNOG00000025285	**Gfm2**	G elongation factor, mitochondrial 2 (G	294672	2	47063820	47094441	30622	640,23	-0,26	4,70E-01
ENSRNOG00000000127	**Kpna6**	karyopherin alpha 6 (importin alpha 7)	362607	5	151581763	151594918	13156	1320,69	0,33	4,70E-01
ENSRNOG00000003550	**Acbd6**	acyl-CoA binding domain containing 6	289125	13	78130762	78265044	134283	1738,90	0,30	4,70E-01
ENSRNOG00000006895	**Vps53**	vacuolar protein sorting 53 homolog (S	287535	10	63672733	63790222	117490	1638,85	0,37	4,70E-01
ENSRNOG00000010223	**Syp**	synaptophysin (Syp), mRNA [Source:F	24804	X	16485674	16500711	15038	17090,99	0,39	4,70E-01
ENSRNOG00000012008	**S100a3**	S100 calcium binding protein A3 (S10	114216	2	209427357	209430124	2768	82,40	-0,63	4,70E-01

ENSRNOG00000015225	**Gramd3**	GRAM domain containing 3 (Gramd3)	307288	18	50688264	50786064	97801	1602,90	-0,52	4,70E-01
ENSRNOG00000017315		HAUS augmin-like complex subunit 7	293844	1	153101834	153120880	19047	369,92	-0,36	4,70E-01
ENSRNOG00000018795	**Rpl18a**	ribosomal protein L18A (Rpl18a), mRN	290641	16	20150821	20152827	2007	20559,90	0,52	4,70E-01
ENSRNOG00000021497	**Akt3**	v-akt murine thymoma viral oncogene	29414	13	99533234	99800302	267069	2016,13	-0,32	4,70E-01
ENSRNOG00000022500	**Rwdd4**	RWD domain containing 4A (Rwdd4),	502084	16	47578235	47594453	16219	259,26	-0,37	4,70E-01
ENSRNOG00000028683		Alpha-N-acetylgalactosaminide alpha-	29758	2	278255750	278355379	99630	74,03	-0,40	4,70E-01
ENSRNOG00000038973	**Acd**	adrenocortical dysplasia homolog (mo	307798	19	48532000	48534691	2692	972,36	0,29	4,70E-01
ENSRNOG00000013229	**Mthfs**	5,10-methenyltetrahydrofolate synthet	300886	8	96071796	96121083	49288	123,17	-0,47	4,70E-01
ENSRNOG00000015249	**RGD15622**	TAF8 RNA polymerase II, TATA box bi	299169	9	14436067	14450890	14824	544,78	0,32	4,70E-01
ENSRNOG00000028581	**Ccdc138**	Protein Ccdc138 [Source:UniProtKB/	499442	20	29897985	29974911	76927	55,91	-0,60	4,70E-01
ENSRNOG00000001083	**Kdelr2**	KDEL (Lys-Asp-Glu-Leu) endoplasmic	304290	12	15232539	15250771	18233	2923,62	0,38	4,70E-01
ENSRNOG00000021128	**Kcnj11**	potassium inwardly rectifying channel,	83535	1	103188484	103189656	1173	89,42	0,40	4,70E-01
ENSRNOG00000019549	**Akap12**	A kinase (PRKA) anchor protein 12 (A	83425	1	42162163	42252614	90452	1941,44	0,33	4,70E-01
ENSRNOG00000021745	**Bhlhe22**	basic helix-loop-helix family, member e	365748	2	122425612	122428723	3112	3226,91	-0,58	4,71E-01
ENSRNOG00000027017	**Rnasel**	ribonuclease L (2',5'-oligoisoadenylate	359726	13	76158771	76166016	7246	94,54	0,35	4,71E-01
ENSRNOG00000015581		Protein Dnahc6 [Source:UniProtKB/Tr	117250	4	165697844	165895140	197297	35,56	-0,63	4,71E-01
ENSRNOG00000018421	**Aen**	apoptosis enhancing nuclease (Aen),	361594	1	141559326	141568691	9366	892,18	0,33	4,71E-01
ENSRNOG00000024376	**Zfp111**	zinc finger protein 111 (Zfp111), mRNA	170849	1	82300647	82307037	6391	243,11	-0,35	4,71E-01
ENSRNOG00000017060	**Ryr2**	ryanodine receptor 2, cardiac (Ryr2), t	689560	17	67286594	67704766	418173	849,94	-0,45	4,71E-01
ENSRNOG00000018550	**RGD13081**	similar to RIKEN cDNA 1200014J11 (F	360563	10	59352867	59382579	29713	615,27	0,39	4,71E-01
ENSRNOG00000008133	**Mfng**	MFNG O-fucosylpeptide 3-beta-N-ace	315119	7	119970579	119988416	17838	245,85	0,47	4,71E-01
ENSRNOG00000012739	**Brf2**	BRF2, RNA polymerase III transcriptio	306542	16	68763911	68768629	4719	482,79	0,40	4,72E-01
ENSRNOG00000021298	**Dstyk**	dual serine/threonine and tyrosine pro	304791	13	54263966	54318716	54751	1529,14	0,49	4,72E-01
ENSRNOG00000032178	**Cenpa**	centromere protein A (Cenpa), mRNA	298850	6	36888797	36896348	7552	138,87	0,48	4,72E-01
ENSRNOG00000047433				6	2292319	2295746	3428	170,88	-0,49	4,72E-01
ENSRNOG00000018132		HMG box domain containing 3 [Source:MGI Syn		18	55689442	55731997	42556	1555,02	0,31	4,72E-01
ENSRNOG00000018102	**Coa5**	cytochrome C oxidase assembly facto	503252	9	43716386	43726016	9631	401,96	0,32	4,72E-01
ENSRNOG00000013712	**Tex261**	testis expressed 261 (Tex261), mRNA	297392	4	180035951	180042794	6844	2903,84	0,30	4,72E-01
ENSRNOG00000020199	**RGD13115**	similar to RIKEN cDNA 9430015G10 (	313775	5	177016247	177033648	17402	448,07	0,40	4,72E-01
ENSRNOG00000025416	**Dpy19l4**	Protein Dpy19l4 [Source:UniProtKB/T	297824	5	29230401	29280664	50264	175,16	-0,44	4,73E-01
ENSRNOG00000033090	**Ltbp1**	latent transforming growth factor beta	59107	6	31097090	31491806	394717	98,98	0,54	4,73E-01
ENSRNOG00000002645	**Gemin5**	gem (nuclear organelle) associated pr	691231	10	43347904	43392988	45085	872,98	0,30	4,73E-01
ENSRNOG00000009102	**Fermt2**	fermitin family member 2 (Fermt2), mF	289992	15	23771818	23840399	68582	2829,44	-0,29	4,73E-01
ENSRNOG00000013774	**Lmnb1**	lamin B1 (Lmnb1), mRNA [Source:Ref	116685	18	50979875	51017524	37650	2116,69	0,29	4,73E-01
ENSRNOG00000027115	**Zc2hc1c**	zinc finger, C2HC-type containing 1C (	314321	6	116437584	116445658	8075	32,28	-0,47	4,73E-01
ENSRNOG00000000920	**Phkg1**	phosphorylase kinase, gamma 1 (Phk	29353	12	32392860	32406743	13884	30,95	-0,59	4,73E-01
ENSRNOG00000001682		E3 ubiquitin-protein ligase TTC3 [Sou	360702	11	38186073	38330440	144368	10691,22	-0,56	4,73E-01
ENSRNOG00000004972	**Upp1**	uridine phosphorylase 1 (Upp1), mRN	289801	14	89113731	89132096	18366	64,52	0,57	4,73E-01

ENSRNOG00000007268		Protein RGD1561796 [Source:UniPro	360483	10	10635218	10645243	10026	111,64	0,46	4,73E-01
ENSRNOG00000008850	**Ttc5**	tetratricopeptide repeat domain 5 (Ttc!	305837	15	31530805	31548395	17591	2149,57	0,41	4,73E-01
ENSRNOG00000008892	**Parp2**	poly (ADP-ribose) polymerase 2 (Parp	290027	15	31571841	31582073	10233	516,49	-0,31	4,73E-01
ENSRNOG00000009953	**Snx16**	sorting nexin 16 (Snx16), mRNA [Sour	64088	2	113278410	113299752	21343	634,62	-0,31	4,73E-01
ENSRNOG00000009993	**Dcps**	decapping enzyme, scavenger (Dcps)	266605	8	36340779	36393331	52553	726,66	0,23	4,73E-01
ENSRNOG00000011228	**Layn**	layilin (Layn), mRNA [Source:RefSeq I	500996	8	54047076	54063936	16861	39,07	-0,60	4,73E-01
ENSRNOG00000012111	**Poli**	polymerase (DNA directed), iota (Poli)	291526	18	64930367	64950189	19823	122,47	-0,33	4,73E-01
ENSRNOG00000012524	**Zfp91**	zinc finger protein 91 (Zfp91), mRNA [	246282	1	236755856	236792548	36693	3936,58	-0,32	4,73E-01
ENSRNOG00000014278	**Zfp710**	zinc finger protein 710 (Zfp710), mRN.	293044	1	142815665	142822208	6544	360,87	0,26	4,73E-01
ENSRNOG00000015543		uncharacterized protein LOC310362 |	310362	2	72849783	72859924	10142	167,20	-0,41	4,73E-01
ENSRNOG00000019407	**Dmap1**	DNA methyltransferase 1-associated p	298447	5	140323215	140331218	8004	707,58	0,41	4,73E-01
ENSRNOG00000020904	**Cdc42ep2**	CDC42 effector protein (Rho GTPase	309175	1	228203835	228212662	8828	129,44	-0,34	4,73E-01
ENSRNOG00000028832	**Sorcs3**	sortilin-related VPS10 domain contain	294043	1	275416587	276058327	641741	575,11	-0,54	4,73E-01
ENSRNOG00000029588	**LOC10036**	LRRG00136-like (LOC100364769), m	1E+08	1	224556182	224592483	36302	27,29	0,63	4,73E-01
ENSRNOG00000047183	**Hist1h2bk**	histone cluster 1, H2bk (Hist1h2bk), m	680312	17	58942757	58943137	381	109,45	0,52	4,73E-01
ENSRNOG00000049171	**Pspn**	persephin (Pspn), mRNA [Source:Ref!	25525	9	9045406	9045964	559	47,82	0,47	4,73E-01
ENSRNOG00000049614		inter-alpha (globulin) inhibitor H5 [Source:MGI S		17	73726031	73742064	16034	340,46	-0,41	4,73E-01
ENSRNOG00000002389	**Morf4l2**	mortality factor 4 like 2 (Morf4l2), mRN	317413	X	107278245	107289323	11079	7382,74	0,29	4,73E-01
ENSRNOG00000023651		Uncharacterized protein [Source:UniProtKB/TrE		2	8807641	8903858	96218	80,97	-0,48	4,73E-01
ENSRNOG00000001291	**Adap1**	ArfGAP with dual PH domains 1 (Adap	171097	12	19402559	19454794	52236	4374,03	-0,50	4,73E-01
ENSRNOG00000008016	**Ckap4**	cytoskeleton-associated protein 4 (Cka	362859	7	25084905	25093002	8098	1986,35	0,27	4,73E-01
ENSRNOG00000010034	**Asah1**	N-acylsphingosine amidohydrolase (ac	84431	16	53712315	53743716	31402	1445,87	-0,45	4,73E-01
ENSRNOG00000019425	**Gabarapl2**	GABA(A) receptor-associated protein	64670	19	54972028	54982863	10836	944,50	0,27	4,73E-01
ENSRNOG00000019973	**LOC10091**	ADP-ribosylation factor-like 3 (Arl3), m	64664	1	273718181	273753577	35397	1925,21	0,27	4,73E-01
ENSRNOG00000036666	**LOC61957**	cDNA sequence BC017643 [Source:M	619574	10	110011632	110018450	6819	852,21	0,45	4,73E-01
ENSRNOG00000043193	**Smim1**	small integral membrane protein 1 (Sn	500595	5	174800870	174803561	2692	33,39	-0,63	4,73E-01
ENSRNOG00000005842	**Zfp280d**	zinc finger protein 280D (Zfp280d), mF	315798	8	74134797	74211059	76263	795,03	-0,39	4,73E-01
ENSRNOG00000012099	**Papd4**	PAP associated domain containing 4 (	361878	2	41944123	41995951	51829	549,25	-0,46	4,73E-01
ENSRNOG00000003786	**Tspan6**	tetraspanin 6 (Tspan6), mRNA [Source	302313	X	104555384	104562260	6877	1684,26	-0,46	4,73E-01
ENSRNOG00000011864	**Gm2a**	GM2 ganglioside activator (Gm2a), mF	282838	10	40277005	40289541	12537	1924,46	-0,36	4,73E-01
ENSRNOG00000003746		Gap junction beta-1 protein [Source:L	29584	X	72123958	72131894	7937	27,04	0,63	4,76E-01
ENSRNOG00000017703	**Unc93b1**	unc-93 homolog B1 (C. elegans) (Unc	361689	1	226043713	226054651	10939	51,80	-0,57	4,76E-01
ENSRNOG00000018751	**Cog7**	component of oligomeric golgi comple	293456	1	198858776	199170252	311477	1021,69	0,34	4,76E-01
ENSRNOG00000010372		Protein Foxred1; RCG22732 [Source:UniProtKE		8	36421216	36428627	7412	235,38	-0,34	4,76E-01
ENSRNOG00000047440		Uncharacterized protein [Source:UniProtKB/TrE		16	76755110	77059453	304344	32,94	-0,51	4,76E-01
ENSRNOG00000037352	**RGD15627**	similar to RIKEN cDNA 1110012L19 (F	292328	8	69193023	69196651	3629	356,96	0,26	4,76E-01
ENSRNOG00000010237	**Map7d1**	MAP7 domain containing 1 (Map7d1),	681287	5	148018369	148042648	24280	6767,74	0,34	4,76E-01
ENSRNOG00000013837		transformation related protein 53 bindi	296099	3	119695001	119770850	75850	5314,53	0,29	4,76E-01

ENSRNOG00000016272	**LOC67895**	endothelial differentiation-related facto	296570	3	2762582	2766887	4306	2869,55	0,36	4,76E-01
ENSRNOG00000048096		Protein Plekha6 [Source:UniProtKB/TrEMBL;Ac		13	55133392	55151200	17809	364,77	-0,29	4,76E-01
ENSRNOG00000004538	**Brd1**	bromodomain containing 1 (Brd1), mR	315210	7	129366021	129413531	47511	2141,89	0,37	4,76E-01
ENSRNOG00000009576	**Xpa**	xeroderma pigmentosum, complemen	298074	5	66266229	66309390	43162	69,77	-0,44	4,76E-01
ENSRNOG00000032699		Protein tweety homolog 1 [Source:Un	292597	1	74795009	74813522	18514	15712,33	-0,42	4,76E-01
ENSRNOG00000045899		PET117 homolog (S. cerevisiae) [Source:MGI Sy		3	144934673	144939289	4617	58,16	0,39	4,76E-01
ENSRNOG00000005550	**Lrfn5**	leucine rich repeat and fibronectin type	314164	6	92602956	92952468	349513	930,41	-0,35	4,76E-01
ENSRNOG00000002381	**Bmp3**	bone morphogenetic protein 3 (Bmp3)	25667	14	12306370	12331030	24661	98,02	-0,53	4,76E-01
ENSRNOG00000014875	**Ash2l**	ash2 (absent, small, or homeotic)-like	290829	16	70676024	70697836	21813	1646,49	0,37	4,76E-01
ENSRNOG00000017851	**Etfb**	electron-transfer-flavoprotein, beta pol	292845	1	99549585	99563745	14161	1418,64	0,32	4,76E-01
ENSRNOG00000018441	**Ggt7**	gamma-glutamyltransferase 7 (Ggt7),	156275	3	157373962	157397348	23387	1823,79	0,31	4,76E-01
ENSRNOG00000026900	**Ppil2**	peptidylprolyl isomerase (cyclophilin)-l	360746	11	91200042	91222633	22592	819,27	0,37	4,76E-01
ENSRNOG00000017279	**Tmem50a**	transmembrane protein 50A (Tmem50	298552	5	157006370	157021310	14941	5211,03	0,32	4,77E-01
ENSRNOG00000026186	**Syde2**	Protein Syde2-ps1 [Source:UniProtKE	308021	2	270386600	270418420	31821	153,06	-0,37	4,77E-01
ENSRNOG00000004483	**Ptprr**	protein tyrosine phosphatase, recepto	94202	7	59041711	59335373	293663	469,14	-0,36	4,78E-01
ENSRNOG00000005246	**Tfap2c**	transcription factor AP-2 gamma (Tfap	362280	3	176625472	176633352	7881	20,14	-0,59	4,78E-01
ENSRNOG00000048861	**LOC49812**	RIKEN cDNA 2510002D24 gene [Sou	498122	11	89401176	89405419	4244	445,13	0,27	4,78E-01
ENSRNOG00000011827	**LOC10036**	Proteasome subunit beta type-6 [Sou	1E+08	2	115773840	115774556	717	19,25	0,61	4,78E-01
ENSRNOG00000018243	**Ubfd1**	ubiquitin family domain containing 1 (U	293454	1	199088152	199099547	11396	1415,17	0,42	4,78E-01
ENSRNOG00000050188		Uncharacterized protein [Source:UniProtKB/TrE		10	95171418	95171732	315	176,78	-0,35	4,78E-01
ENSRNOG00000000185		3-mercaptopyruvate sulfurtransferase	192172	7	119614631	119622095	7465	1693,38	0,42	4,79E-01
ENSRNOG00000009453	**Mob3b**	MOB kinase activator 3B (Mob3b), mF	366352	5	55007530	55201845	194316	55,19	-0,50	4,79E-01
ENSRNOG00000011160		synaptic vesicle glycoprotein 2B [Sou	117556	1	137657430	137732099	74670	2298,95	-0,46	4,79E-01
ENSRNOG00000005818		RIKEN cDNA 9330182L06 gene [Source:MGI Sy		4	21496030	21689226	193197	1243,53	-0,30	4,79E-01
ENSRNOG00000010517	**Fam126a**	family with sequence similarity 126, m	499975	4	7670532	7731263	60732	630,35	-0,37	4,79E-01
ENSRNOG00000015683		Protein Pitpnc1 [Source:UniProtKB/Tr	498015	10	95176728	95298724	121997	239,58	-0,32	4,79E-01
ENSRNOG00000047525	**Zcchc18**		679126	X	107513881	107514894	1014	4911,20	0,44	4,79E-01
ENSRNOG00000039754		ras-related protein Rab-7b [Source:R	501854	13	53536348	53553149	16802	125,51	-0,53	4,79E-01
ENSRNOG00000001738	**Eif4g1**	Protein LOC100911431 [Source:UniP	287986	11	86980027	86998442	18416	300,09	0,38	4,79E-01
ENSRNOG00000003919	**Rps6kb1**	ribosomal protein S6 kinase, polypepti	83840	10	76234215	76275377	41163	656,43	-0,39	4,79E-01
ENSRNOG00000007349		activating signal cointegrator 1 comple	498402	14	85555815	85592750	36936	632,49	0,30	4,79E-01
ENSRNOG00000008345	**Clcn6**	chloride channel, voltage-sensitive 6 (	295586	5	168471564	168502323	30760	2708,79	0,33	4,79E-01
ENSRNOG00000010591	**Kdm5a**	lysine (K)-specific demethylase 5A (Kc	312678	4	219981483	220015022	33540	287,87	0,47	4,79E-01
ENSRNOG00000014330	**Pcmt1**	protein-L-isoaspartate (D-aspartate) O	25604	1	3433956	3464919	30964	1801,56	0,38	4,79E-01
ENSRNOG00000016842		Protein Pifo [Source:UniProtKB/TrEM	691223	2	228037986	228055019	17034	22,44	-0,62	4,79E-01
ENSRNOG00000022514	**Tor2a**	torsin family 2, member A (Tor2a), mR	362112	3	17132599	17136663	4065	971,58	0,28	4,79E-01
ENSRNOG00000025005	**RGD15606**	similar to novel protein (RGD1560672	498270	13	92891869	92899444	7576	69,43	-0,60	4,79E-01
ENSRNOG00000026328	**Gpr137c**	G protein-coupled receptor 137C (Gpr	305812	15	23512965	23580026	67062	167,10	-0,40	4,79E-01

ENSRNOG00000025350	**Ppp1r13l**	Protein Ppp1r13l [Source:UniProtKB/	686781	1	81545967	81556717	10751	130,89	0,36	4,79E-01
ENSRNOG00000014235	**Zgpat**	zinc finger, CCCH-type with G patch d	296478	3	180575440	180591733	16294	684,52	0,30	4,80E-01
ENSRNOG00000048252		Uncharacterized protein [Source:UniProtKB/TrE		6	37299180	37323881	24702	174,18	-0,38	4,80E-01
ENSRNOG00000019164	**Uba1**	ubiquitin-like modifier activating enzym	314432	X	2516603	2535075	18473	14724,32	0,34	4,80E-01
ENSRNOG00000001295	**S100b**	S100 calcium binding protein B (S100	25742	20	15287748	15296457	8710	13697,32	-0,56	4,80E-01
ENSRNOG00000014667	**Dll1**	delta-like 1 (Drosophila) (Dll1), mRNA	84010	1	242107688	242115797	8110	1150,65	0,28	4,80E-01
ENSRNOG00000037302	**LOC10091**	RAD51 recombinase (Rad51), mRNA	499870	3	117468881	117493555	24675	72,03	0,54	4,80E-01
ENSRNOG00000006426	**Syt1**	synaptotagmin I (Syt1), mRNA [Source	25716	7	50097034	50650569	553536	4488,52	-0,32	4,81E-01
ENSRNOG00000013713	**LOC49924**	cDNA sequence BC051019 [Source:M	499240	1	181324235	181335547	11313	17,78	-0,60	4,81E-01
ENSRNOG00000019062	**Pura**	Transcriptional activator protein Pur-al	307498	18	28817976	28818941	966	302,55	0,30	4,81E-01
ENSRNOG00000047924	**Zbtb7c**	zinc finger and BTB domain containing	679155	18	71136512	71590024	453513	179,92	-0,52	4,81E-01
ENSRNOG00000006056		Protein Asap2 [Source:UniProtKB/TrE	362719	6	60104140	60213869	109730	1113,89	-0,36	4,82E-01
ENSRNOG00000014426		lysyl oxidase (Lox), mRNA [Source:Re	24914	18	46715872	46791367	75496	86,99	-0,60	4,82E-01
ENSRNOG00000023086	**LOC10036**	cAMP-regulated phosphoprotein 19 (Arpp19), m		8	81566087	81585228	19142	150,47	0,32	4,82E-01
ENSRNOG00000013358	**Aqp11**	aquaporin 11 (Aqp11), mRNA [Source:	286758	1	168910173	168920364	10192	235,58	-0,44	4,83E-01
ENSRNOG00000026163	**Cpt1c**	carnitine palmitoyltransferase 1c (Cpt1	308579	1	102019484	102034000	14517	808,89	0,31	4,83E-01
ENSRNOG00000019100		kinesin family member 2C (Kif2c), tran	171529	5	139821001	139846482	25482	335,45	0,45	4,83E-01
ENSRNOG00000001808	**Asun**	asunder, spermatogenesis regulator (/	690728	4	245015507	245046757	31251	878,58	-0,32	4,83E-01
ENSRNOG00000010474	**Copg1**	coatomer protein complex, subunit ga	297428	4	185062828	185087265	24438	5527,62	0,35	4,83E-01
ENSRNOG00000019529	**Btbd1**	BTB (POZ) domain containing 1 (Btbd	293060	1	144554178	144591200	37023	5613,95	-0,41	4,83E-01
ENSRNOG00000023269				13	94680739	94682046	1308	45,53	0,46	4,83E-01
ENSRNOG00000026679	**Scn4b**	sodium channel, voltage-gated, type I\	315611	8	48067555	48082728	15174	28,59	-0,52	4,83E-01
ENSRNOG00000007369	**Shisa4**	shisa family member 4 (Shisa4), mRN	360848	13	57243999	57248373	4375	3642,67	0,38	4,83E-01
ENSRNOG00000014445		heterochromatin protein 1, binding protein 3 [So		5	160336477	160358537	22061	7190,22	0,30	4,83E-01
ENSRNOG00000024964		Protein LOC681371 [Source:UniProtk	681371	10	66425460	66451772	26313	376,48	-0,37	4,83E-01
ENSRNOG00000030912				6	79550301	79550848	548	18,42	-0,62	4,83E-01
ENSRNOG00000000521	**Cdkn1a**	cyclin-dependent kinase inhibitor 1A ((	114851	20	8592437	8602879	10443	1922,63	0,52	4,84E-01
ENSRNOG00000002874		Uncharacterized protein [Source:UniProtKB/TrE		6	118377074	118391069	13996	503,01	-0,35	4,84E-01
ENSRNOG00000008102		diphthamine biosynthesis 7 [Source:MGI Symb		3	2151723	2160261	8539	632,99	0,33	4,84E-01
ENSRNOG00000005016		transmembrane emp24 domain-contai	305502	14	87351932	87356305	4374	1224,23	0,46	4,84E-01
ENSRNOG00000003990	**Grb2**	growth factor receptor bound protein 2	81504	10	104019958	104087249	67292	1916,21	0,39	4,84E-01
ENSRNOG00000007124	**Krcc1**	lysine-rich coiled-coil 1 (Krcc1), mRNA	312437	4	163912884	163925867	12984	427,39	-0,59	4,84E-01
ENSRNOG00000010532	**Fam206a**	Protein Fam206a; RCG31985 [Source	298018	5	77711085	77714698	3614	76,79	-0,44	4,84E-01
ENSRNOG00000031491				5	29485995	29486849	855	95,06	0,35	4,84E-01
ENSRNOG00000003813	**Kcnk10**	potassium channel, subfamily K, mem	65272	6	131633746	131760720	126975	758,89	-0,39	4,84E-01
ENSRNOG00000004032		RIKEN cDNA 2310067B10 gene [Source:MGI S		10	103942825	103965175	22351	1218,21	0,27	4,84E-01
ENSRNOG00000008392	**Sbf1**	SET binding factor 1 (Sbf1), mRNA [S	300147	7	129946917	129973401	26485	5547,77	0,32	4,84E-01
ENSRNOG00000012811	**Spint1**	serine peptidase inhibitor, Kunitz type	311331	3	117599950	117612634	12685	36,82	-0,50	4,84E-01

ENSRNOG00000014642	**Morn1**	MORN repeat containing 1 (Morn1), m	298676	5	175945054	176002862	57809	217,47	0,45	4,84E-01
ENSRNOG00000011227	**Atp1b2**	ATPase, Na+/K+ transporting, beta 2 p	24214	10	55951263	55957495	6233	8432,43	-0,48	4,84E-01
ENSRNOG00000013216	**Usb1**	U6 snRNA biogenesis 1 (Usb1), mRN/	307643	19	10089672	10102534	12863	328,32	0,29	4,85E-01
ENSRNOG00000011073	**Htt**	huntingtin (Htt), mRNA [Source:RefSe	29424	14	81796508	81942093	145586	2953,69	0,34	4,85E-01
ENSRNOG00000001868	**LOC10091**	v-crk avian sarcoma virus CT10 oncog	287942	11	90833008	90867428	34421	596,40	0,38	4,85E-01
ENSRNOG00000008642	**Snx11**	sorting nexin 11 (Snx11), transcript va	303493	10	84421495	84432020	10526	1072,14	0,25	4,85E-01
ENSRNOG00000048101	**Zfp397**	zinc finger protein 397 (Zfp397), mRN	1E+08	18	15625143	15629505	4363	75,25	-0,49	4,85E-01
ENSRNOG00000032261	**Cyp2d4**	cytochrome P450, family 2, subfamily	171522	7	123583985	123593008	9024	92,26	-0,52	4,85E-01
ENSRNOG00000003374	**Ccdc85a**	coiled-coil domain containing 85A (Cc	289855	14	112409633	112636341	226709	205,12	-0,43	4,85E-01
ENSRNOG00000010556		inturned planar cell polarity effector ho	361938	2	147064054	147130412	66359	100,48	-0,39	4,85E-01
ENSRNOG00000011552	**Mon1b**	MON1 homolog b (yeast) (Mon1b), mF	307868	19	56763615	56770441	6827	735,71	0,24	4,85E-01
ENSRNOG00000014621	**Rab8a**	RAB8A, member RAS oncogene famil	117103	16	19213379	19235107	21729	1834,82	0,31	4,85E-01
ENSRNOG00000015283	**Nt5c1a**	5'-nucleotidase, cytosolic IA (Nt5c1a),	313574	5	144678889	144693912	15024	63,63	-0,61	4,85E-01
ENSRNOG00000001762	**Pcyt1a**	phosphate cytidylyltransferase 1, choli	140544	11	74632947	74676225	43279	1810,09	-0,24	4,85E-01
ENSRNOG00000000062	**Pcgf3**	polycomb group ring finger 3 (Pcgf3),	305624	14	2242209	2295509	53301	834,86	-0,35	4,86E-01
ENSRNOG00000002309	**Hlx**	H2.0-like homeobox (Hlx), mRNA [Sou	364069	13	107315404	107320809	5406	17,53	0,60	4,86E-01
ENSRNOG00000006829				3	42975030	42975468	439	136,67	0,37	4,86E-01
ENSRNOG00000019106	**LOC10036**	ribosomal protein S17 (Rps17), mRNA	29286	1	144109690	144112274	2585	297,77	0,39	4,86E-01
ENSRNOG00000013987	**Sbno2**	strawberry notch homolog 2 (Drosophi	314619	7	12641927	12685802	43876	970,38	0,26	4,86E-01
ENSRNOG00000018771	**Ncapd2**	Condensin complex subunit 1 [Source	362438	4	224700041	224722925	22885	1355,45	0,34	4,86E-01
ENSRNOG00000020425	**Stim1**	stromal interaction molecule 1 (Stim1)	361618	1	173562502	173722020	159519	1751,21	0,33	4,86E-01
ENSRNOG00000022491	**Wdr76**	Protein Wdr76 [Source:UniProtKB/TrE	311361	3	119988996	120018334	29339	55,93	-0,41	4,86E-01
ENSRNOG00000034242	**Vcp**	valosin-containing protein (Vcp), mRN	116643	5	62952000	62971402	19403	15679,75	0,29	4,86E-01
ENSRNOG00000036648	**LOC68069**	RIKEN cDNA 6430531B16 gene [Sou	680693	1	219230245	219236189	5945	18,11	-0,61	4,86E-01
ENSRNOG00000038150	**RGD15603**	RGD1560394 (RGD1560394), mRNA	289728	14	82737925	82739530	1606	1887,37	0,48	4,86E-01
ENSRNOG00000001575		glutamate receptor, ionotropic, kainate	29559	11	31426908	31828130	401223	332,70	0,49	4,86E-01
ENSRNOG00000009823	**Lsmd1**	LSM domain containing 1 (Lsmd1), ml	287429	10	55739689	55740766	1078	758,23	0,44	4,86E-01
ENSRNOG00000033893	**Cacna1h**	calcium channel, voltage-dependent, T	114862	10	14547465	14605140	57676	1644,65	-0,35	4,86E-01
ENSRNOG00000001754	**Wdr53**	WD repeat domain 53 (Wdr53), mRNA	498097	11	74871569	74879908	8340	178,86	0,34	4,87E-01
ENSRNOG00000004520	**Wdr60**	WD repeat domain 60 (Wdr60), mRNA	314523	6	153007323	153061842	54520	397,65	0,27	4,87E-01
ENSRNOG00000005260	**Acp1**	acid phosphatase 1, soluble (Acp1), tr	24161	6	58515990	58531060	15071	143,29	-0,42	4,87E-01
ENSRNOG00000005330	**Crebbp**	CREB binding protein (Crebbp), mRN/	54244	10	10350046	10473909	123864	1434,51	-0,24	4,87E-01
ENSRNOG00000009864	**Pla2g12a**	phospholipase A2, group XIIA (Pla2g1	362039	2	74606742	74622974	16233	493,24	0,31	4,87E-01
ENSRNOG00000015002	**Abhd15**	abhydrolase domain containing 15 (Ab	303343	10	62340336	62345727	5392	41,99	-0,55	4,87E-01
ENSRNOG00000019746	**Paf1**	Paf1, RNA polymerase II associated fa	361531	1	86577752	86583329	5578	2221,80	0,31	4,87E-01
ENSRNOG00000031017				10	85338680	85339284	605	20,89	0,55	4,87E-01
ENSRNOG00000048230	**LOC30030**	uncharacterized protein LOC300308		299668	7	20432424	20644702	212279	31,85	-0,59	4,87E-01
ENSRNOG00000002680	**Lamc1**	laminin, gamma 1 (Lamc1), mRNA [Sc	117036	13	75622195	75749168	126974	2418,87	-0,30	4,87E-01

ENSRNOG00000002120	**Spata18**	spermatogenesis associated 18 (Spat	289586	14	36904437	36931140	26704	42,68	-0,61	4,87E-01
ENSRNOG00000014075	**Clybl**	citrate lyase beta like (Clybl), mRNA [S	306198	15	111996346	112224034	227689	86,13	-0,56	4,87E-01
ENSRNOG00000021215	**Rbm8a**	RNA binding motif protein 8A (Rbm8a)	295284	2	218242220	218244257	2038	1498,22	0,37	4,87E-01
ENSRNOG00000000105	**Cplx2**	complexin 2 (Cplx2), mRNA [Source:R	116657	17	12883635	12892106	8472	2276,85	-0,34	4,88E-01
ENSRNOG00000000247	**Mfsd11**	major facilitator superfamily domain cc	360667	10	105455233	105474239	19007	876,48	0,44	4,88E-01
ENSRNOG00000003111	**Ovca2**	ovarian tumor suppressor candidate 2	497954	10	61733771	61734889	1119	462,37	0,33	4,88E-01
ENSRNOG00000008417	**Giot1**	gonadotropin inducible ovarian transcr	171090	7	13266211	13267989	1779	31,27	-0,59	4,88E-01
ENSRNOG00000010977	**Igfbp6**	insulin-like growth factor binding prote	25641	7	141546160	141551065	4906	26,99	-0,61	4,88E-01
ENSRNOG00000019655	**Atp6v0b**	ATPase, H+ transporting, lysosomal V	298451	5	140507405	140508839	1435	4354,92	0,51	4,88E-01
ENSRNOG00000047133	**Jrkl**	jerky homolog-like (mouse) (Jrkl), mRI	315417	8	11843756	11845330	1575	154,54	-0,37	4,88E-01
ENSRNOG00000002818	**Eftud2**	Protein Eftud2 [Source:UniProtKB/TrE	287739	10	90705843	90752820	46978	3696,35	0,21	4,88E-01
ENSRNOG00000050445	**Gde1**	glycerophosphodiester phosphodieste	60418	1	195338336	195350223	11888	4035,07	0,28	4,88E-01
ENSRNOG00000001136	**Pebp1**	phosphatidylethanolamine binding pro	29542	12	46846901	46851098	4198	19119,45	0,31	4,88E-01
ENSRNOG00000006972	**Zfp189**	zinc finger protein 189 (Zfp189), mRN	313219	5	69280868	69292223	11356	145,95	-0,54	4,88E-01
ENSRNOG00000013533	**Acin1**	apoptotic chromatin condensation indu	305884	15	37173169	37218052	44884	4750,74	0,31	4,88E-01
ENSRNOG00000013895	**Npdc1**	neural proliferation, differentiation and	296562	3	2606428	2612083	5656	5396,28	0,26	4,88E-01
ENSRNOG00000050206		SH3 and multiple ankyrin repeat doma	171093	1	224028791	224445824	417034	247,05	-0,36	4,88E-01
ENSRNOG00000003880	**Tph2**	tryptophan hydroxylase 2 (Tph2), mRN	317675	7	58053270	58157948	104679	35,69	-0,58	4,88E-01
ENSRNOG00000007692		intraflagellar transport protein 52 homo	362265	3	165588271	165605625	17355	433,63	-0,30	4,88E-01
ENSRNOG00000045696	**RGD13055**	similar to RIKEN cDNA 3110001I22 (R	363528	10	361770	368264	6495	44,41	-0,42	4,88E-01
ENSRNOG00000016960	**Polr3e**	polymerase (RNA) III (DNA directed) p	361640	1	197779117	197807240	28124	947,59	0,31	4,88E-01
ENSRNOG00000001265	**Mad1l1**	MAD1 mitotic arrest deficient-like 1 (ye	680006	12	18402692	18711591	308900	559,86	0,26	4,88E-01
ENSRNOG00000005470	**Atxn3**	ataxin 3 (Atxn3), mRNA [Source:RefSe	60331	6	135031498	135065319	33822	292,13	-0,39	4,88E-01
ENSRNOG00000010718	**Gpr153**	G protein-coupled receptor 153 (Gpr15	619550	5	173005832	173016052	10221	304,63	-0,55	4,88E-01
ENSRNOG00000003881	**Nit1**	nitrilase 1 (Nit1), transcript variant 2, n	289222	13	94349707	94353139	3433	1320,61	-0,36	4,89E-01
ENSRNOG00000004936	**Sdc2**	syndecan 2 (Sdc2), mRNA [Source:Re	25615	7	71743813	71857533	113721	1540,10	-0,41	4,89E-01
ENSRNOG00000034013		acetyl-CoA carboxylase 1 [Source:Re	60581	10	71459842	71630271	170430	3539,10	-0,33	4,89E-01
ENSRNOG00000046000	**Clasrp**	CLK4-associating serine/arginine rich	499390	1	81757541	81781715	24175	887,29	0,29	4,89E-01
ENSRNOG00000048848		coiled-coil domain containing 9 [Source:MGI Syr		1	79476529	79486019	9491	714,12	0,29	4,89E-01
ENSRNOG00000002361	**Prkg2**	protein kinase, cGMP-dependent, type	25523	14	12160807	12255994	95188	96,90	-0,57	4,89E-01
ENSRNOG00000001397	**Rbm19**	Protein Rbm19 [Source:UniProtKB/Tr	304512	12	43816223	43843543	27321	374,65	0,28	4,90E-01
ENSRNOG00000001517	**Pdk1**	pyruvate dehydrogenase kinase, isozy	116551	3	65019116	65046273	27158	871,74	-0,25	4,90E-01
ENSRNOG00000006908	**Pou3f2**	POU class 3 homeobox 2 (Pou3f2), m	29588	5	41323989	41325326	1338	596,63	0,29	4,90E-01
ENSRNOG00000012270	**Med26**	mediator complex subunit 26 (Med26)	306328	16	18863765	18913578	49814	425,07	0,31	4,90E-01
ENSRNOG00000049456	**Slc35f3**	Protein LOC679321 [Source:UniProtK	679321	19	69935371	69985675	50305	54,22	-0,54	4,90E-01
ENSRNOG00000000812	**RGD13029**	Uncharacterized protein C6orf136 hon	294231	20	5462332	5466442	4111	609,19	0,35	4,90E-01
ENSRNOG00000033522	**Prdm2**	PR domain containing 2, with ZNF dor	313678	5	165469844	165578324	108481	2567,08	0,30	4,90E-01
ENSRNOG00000010889	**Fbxw7**	Protein Fbxw7 [Source:UniProtKB/TrE	1E+08	2	203635360	203708054	72695	820,14	-0,25	4,90E-01

ENSRNOG00000001785	**Etv5**	ets variant 5 (Etv5), mRNA [Source:Re	303828	11	85282553	85339809	57257	2391,28	-0,34	4,90E-01
ENSRNOG00000010838	**Araf**	v-raf murine sarcoma 3611 viral oncog	64363	X	2194385	2206064	11680	1903,21	0,38	4,90E-01
ENSRNOG00000018461	**Pdgfrb**	platelet derived growth factor receptor	24629	18	55596758	55637692	40935	571,22	-0,50	4,90E-01
ENSRNOG00000043145				19	53267047	53267431	385	42,58	-0,47	4,90E-01
ENSRNOG00000002327	**Gabrb1**	gamma-aminobutyric acid (GABA) A receptor, be		14	38453156	38924075	470920	140,99	-0,52	4,91E-01
ENSRNOG00000011752	**Sh3d19**	Protein Sh3d19; Similar to SH3 domai	295171	2	204784166	204820405	36240	1171,57	-0,54	4,91E-01
ENSRNOG00000002635	**Dexi**	dexamethasone-induced transcript (De	497857	10	4022544	4035231	12688	505,62	0,30	4,91E-01
ENSRNOG00000008891	**Ift20**	intraflagellar transport 20 homolog (Ch	287541	10	65832269	65837760	5492	689,65	0,24	4,91E-01
ENSRNOG00000009388	**Sptssb**	serine palmitoyltransferase, small subi	1E+08	2	186020424	186049284	28861	48,04	0,56	4,91E-01
ENSRNOG00000016123	**Rnf144b**	ring finger protein 144B (Rnf144b), mF	364681	17	19987412	20121060	133649	67,16	0,52	4,91E-01
ENSRNOG00000018666	**Gpsm1**	G-protein signaling modulator 1 (Gpsn	246254	3	9127874	9155506	27633	4552,97	0,30	4,91E-01
ENSRNOG00000018767	**Rbm17**	RNA binding motif protein 17 (Rbm17)	291295	17	72289005	72305891	16887	2031,11	0,35	4,91E-01
ENSRNOG00000020068	**Ndufaf3**	NADH dehydrogenase (ubiquinone) co	56769	8	116696132	116698175	2044	832,92	0,26	4,91E-01
ENSRNOG00000032690	**Ube2e2**	ubiquitin-conjugating enzyme E2E 2 (Ube2e2), r		15	11938853	11995295	56443	913,06	-0,33	4,91E-01
ENSRNOG00000032788	**Dysf**	dysferlin (Dysf), mRNA [Source:RefSe	312492	4	180300911	180487010	186100	148,20	0,45	4,91E-01
ENSRNOG00000018650		adaptor-related protein complex 3, mu 2 subunit		16	73709144	73724608	15465	2599,90	0,40	4,91E-01
ENSRNOG00000049489		helicase ARIP4 [Source:RefSeq pepti	363135	8	113566122	113597907	31786	62,57	0,42	4,91E-01
ENSRNOG00000012204	**LOC10029**	expressed sequence AU040320 [Sour	1E+08	5	148550212	148642557	92346	2559,66	0,26	4,92E-01
ENSRNOG00000018977	**Ap3d1**	adaptor-related protein complex 3, del	314633	7	12006416	12041236	34821	8915,12	0,26	4,92E-01
ENSRNOG00000020032	**Impdh1**	IMP (inosine 5'-monophosphate) dehy	362329	4	56245546	56261085	15540	1944,09	0,34	4,92E-01
ENSRNOG00000037909	**Ppm1f**	protein phosphatase, Mg2+/Mn2+ dep	287931	11	91371435	91398731	27297	1169,55	0,34	4,92E-01
ENSRNOG00000001483	**Wbscr16**	Williams-Beuren syndrome chromosor	360796	12	27569603	27599906	30304	670,19	0,26	4,92E-01
ENSRNOG00000003953		RAB3 GTPase activating protein subunit 1 [Sour		13	49662343	49734484	72142	1577,70	0,31	4,92E-01
ENSRNOG00000011826	**Lzts1**	leucine zipper, putative tumor suppres	266711	16	22147127	22202135	55009	2385,12	0,39	4,92E-01
ENSRNOG00000015121	**N4bp1**	Protein N4bp1 [Source:UniProtKB/TrE	291921	19	32227867	32274348	46482	690,78	0,31	4,92E-01
ENSRNOG00000050784		Uncharacterized protein [Source:UniProtKB/TrE		3	140857469	140920456	62988	167,96	-0,37	4,92E-01
ENSRNOG00000011517	**Tnfrsf21**	tumor necrosis factor receptor superfa	316256	9	19419628	19494259	74632	1658,50	-0,39	4,92E-01
ENSRNOG00000009313	**Lrpap1**	low density lipoprotein receptor-relatec	116565	14	81600002	81613373	13372	4987,48	0,36	4,93E-01
ENSRNOG00000019442	**Josd2**	Josephin domain containing 2 (Josd2)	292876	1	101539030	101542738	3709	421,42	0,47	4,93E-01
ENSRNOG00000002055	**Noa1**	nitric oxide associated 1 (Noa1), mRN	289562	14	32901625	32918107	16483	935,73	0,29	4,93E-01
ENSRNOG00000034005	**Celsr3**	cadherin, EGF LAG seven-pass G-typ	83466	8	116964621	116991765	27145	4850,20	0,39	4,93E-01
ENSRNOG00000008142	**Brpf1**	bromodomain and PHD finger contain	679713	4	208561603	208578030	16428	920,90	0,32	4,94E-01
ENSRNOG00000016939	**Chmp7**	charged multivesicular body protein 7	364419	15	55114441	55128253	13813	2030,75	0,35	4,94E-01
ENSRNOG00000026109	**Prmt1**	protein arginine methyltransferase 1 (F	60421	1	102035576	102044782	9207	569,08	0,37	4,94E-01
ENSRNOG00000038933	**Spcs3**	signal peptidase complex subunit 3 hc	680782	16	39826990	39832408	5419	188,31	-0,52	4,94E-01
ENSRNOG00000049385	**Adamtsl4**	ADAMTS-like 4 (Adamtsl4), mRNA [So	310670	2	217293458	217305079	11622	37,53	-0,52	4,94E-01
ENSRNOG00000008749		collagen alpha-1(V) chain precursor [S	85490	3	11789103	11935751	146649	466,22	-0,28	4,95E-01
ENSRNOG00000009076	**Ttpal**	tocopherol (alpha) transfer protein-like	296349	3	166185907	166204117	18211	1494,45	0,36	4,95E-01

ENSRNOG00000019177	**Psd2**	pleckstrin and Sec7 domain containing	307500	18	28490974	28541901	50928	2314,76	-0,32	4,95E-01
ENSRNOG00000000729	**Tmlhe**	trimethyllysine hydroxylase, epsilon (T	170898	20	242193	279024	36832	123,97	-0,43	4,96E-01
ENSRNOG00000002280	**Sh3bgrl**	SH3 domain binding glutamic acid-rich	302363	X	80392505	80489959	97455	2552,24	-0,51	4,96E-01
ENSRNOG00000004287	**Wdr83**	WD repeat domain 83 (Wdr83), mRNA	288924	19	37164267	37169816	5550	428,89	0,41	4,96E-01
ENSRNOG00000018364	**Kat2a**	K(lysine) acetyltransferase 2A (Kat2a)	303539	10	88405799	88413750	7952	2675,15	0,30	4,96E-01
ENSRNOG00000018784	**Jph3**	junctophilin 3 (Jph3), mRNA [Source:F	307916	19	65267451	65327679	60229	2808,67	-0,40	4,96E-01
ENSRNOG00000019843	**St3gal3**	ST3 beta-galactoside alpha-2,3-sialylt	64445	5	140555257	140753537	198281	515,24	0,32	4,96E-01
ENSRNOG00000022176	**Gpr75**	G protein-coupled receptor 75 (Gpr75)	498434	14	114933397	114936006	2610	98,57	-0,58	4,96E-01
ENSRNOG00000022694	**Plekhg5**	pleckstrin homology domain containing	310999	5	172803041	172846760	43720	233,36	-0,43	4,96E-01
ENSRNOG00000022911	**Hjurp**	Holliday junction recognition protein (H	316602	9	95036301	95049229	12929	382,39	0,29	4,96E-01
ENSRNOG00000027797	**Nit2**	nitrilase family, member 2 (Nit2), mRN	288174	11	48655096	48665855	10760	1067,48	-0,42	4,96E-01
ENSRNOG00000021061	**Map4k2**	mitogen activated protein kinase kinas	293694	1	228699326	228713616	14291	243,50	0,47	4,96E-01
ENSRNOG00000010855	**Mrp63**	mitochondrial ribosomal protein 63 (M	691814	15	41937753	41938638	886	956,07	0,43	4,96E-01
ENSRNOG00000026941	**Tril**	TLR4 interactor with leucine-rich repea	362364	4	148632281	148634716	2436	4160,02	-0,47	4,97E-01
ENSRNOG00000011358	**Hipk3**	homeodomain interacting protein kinas	83617	3	100989148	101054811	65664	867,13	0,42	4,97E-01
ENSRNOG00000028870	**Acot1**	acyl-CoA thioesterase 1 (Acot1), mRN	50559	6	118051433	118059103	7671	96,04	-0,61	4,97E-01
ENSRNOG00000009733	**Praf2**	PRA1 domain family, member 2 (Praf2	367743	X	16399546	16402441	2896	1746,49	0,24	4,98E-01
ENSRNOG00000018487	**Slc3a2**	solute carrier family 3 (amino acid tran	50567	1	231843827	231858251	14425	9926,44	-0,40	4,98E-01
ENSRNOG00000033973	**Ccdc177**	Protein Ccdc177 [Source:UniProtKB/T	500686	6	112578662	112580770	2109	217,13	0,40	4,98E-01
ENSRNOG00000014061	**Dusp5**	dual specificity phosphatase 5 (Dusp5	171109	1	281658075	281671441	13367	120,07	-0,57	4,99E-01
ENSRNOG00000026962		Similar to oxysterol-binding protein-like protein 8		7	54031596	54036553	4958	218,93	-0,37	4,99E-01
ENSRNOG00000017823		fibronectin type III and SPRY domain c	313208	5	74616611	74664963	48353	176,28	-0,58	4,99E-01
ENSRNOG00000048164		acyl-Coenzyme A dehydrogenase fam	367196	8	28038469	28064606	26138	570,72	0,42	4,99E-01
ENSRNOG00000010934	**Spa17**	sperm autoantigenic protein 17 (Spa17	85244	8	40067946	40077019	9074	74,98	-0,46	4,99E-01
ENSRNOG00000012580	**Ccdc141**	Protein LOC311134 [Source:UniProtK	311134	3	70437253	70594245	156993	33,10	-0,60	4,99E-01
ENSRNOG00000018342	**Ctif**	CBP80/20-dependent translation initia	364900	18	70602149	70834596	232448	738,11	0,34	4,99E-01
ENSRNOG00000018951	**Col4a5**	Protein Col4a5 [Source:UniProtKB/TrEMBL;Acc		X	111376683	111436082	59400	496,14	-0,39	4,99E-01
ENSRNOG00000021268	**LOC10036**	RIKEN cDNA 1110034G24 gene [Sour	1E+08	3	131874304	131880404	6101	110,68	0,35	4,99E-01
ENSRNOG00000030391	**Ei24**	etoposide induced 2.4 (Ei24), mRNA [	300514	8	39257606	39269673	12068	3784,58	-0,24	4,99E-01
ENSRNOG00000043378	**Nkpd1**	Protein Nkpd1 [Source:UniProtKB/TrE	680440	1	81692939	81698896	5958	19,02	0,54	4,99E-01
ENSRNOG00000012017	**LOC64038**	sertolin (LOC64038), mRNA [Source:F	1E+08	2	99934603	99958197	23595	453,67	0,28	4,99E-01
ENSRNOG00000013409	**Gclm**	glutamate cysteine ligase, modifier sul	29739	2	243862903	243883273	20371	694,55	0,32	4,99E-01
ENSRNOG00000014270	**Rer1**	RER1 retention in endoplasmic reticul	298675	5	175932626	175944701	12076	2539,03	0,30	4,99E-01
ENSRNOG00000005286	**Coch**	cochlin (Coch), mRNA [Source:RefSeq	362735	6	81924200	81928193	3994	39,59	-0,52	4,99E-01
ENSRNOG00000025581	**Vwa2**	Protein Vwa2-ps1 [Source:UniProtKB	307988	1	285076089	285108016	31928	56,55	0,51	4,99E-01
ENSRNOG00000010789	**Dusp7**	dual specificity phosphatase 7 (Dusp7	300980	8	114436772	114442403	5632	589,85	-0,33	4,99E-01
ENSRNOG00000011930	**Rdh12**	retinol dehydrogenase 12 (all-trans/9-c	314264	6	115079502	115092424	12923	127,71	0,60	4,99E-01
ENSRNOG00000028910		RIKEN cDNA A430105I19 gene [Source:MGI Sy		3	117092046	117097427	5382	48,71	-0,50	4,99E-01

ENSRNOG00000002876	**Fam86a**	family with sequence similarity 86, me	302931	10	9300229	9310292	10064	53,05	-0,54	5,00E-01
ENSRNOG00000023095	**Npepps**	aminopeptidase puromycin sensitive (	50558	10	84930337	85012828	82492	7070,15	0,24	5,00E-01
ENSRNOG00000030034	**Sox11**	SRY (sex determining region Y)-box 1	84046	6	55343074	55345088	2015	2072,69	0,32	5,00E-01
ENSRNOG00000026277	**LOC10091(**	zinc finger CCCH type containing 6 (Z	311415	3	128463042	128513173	50132	285,76	-0,34	5,00E-01
ENSRNOG00000016502	**Dscaml1**	Down syndrome cell adhesion molecu	315615	8	48363433	48679606	316174	1150,62	-0,45	5,00E-01
ENSRNOG00000042690	**Zmat4**	zinc finger, matrin type 4 (Zmat4), mR	684961	16	72402519	72450584	48066	120,35	-0,47	5,00E-01
ENSRNOG00000048363		Protein Dnah5 [Source:UniProtKB/TrE	294854	2	100614638	100809390	194753	44,14	-0,52	5,00E-01
ENSRNOG00000025287	**RGD15656**	RGD1565611 (RGD1565611), mRNA	497957	10	63834295	63837955	3661	50,17	-0,60	5,00E-01
ENSRNOG00000019511	**Mrps18a**	mitochondrial ribosomal protein S18A	301249	9	16133559	16150048	16490	1405,07	0,27	5,01E-01
ENSRNOG00000036692		glucagon receptor (Gcgr), transcript va	24953	10	109301091	109309258	8168	20,19	0,53	5,01E-01
ENSRNOG00000018090	**Ppp6r1**	protein phosphatase 6, regulatory sub	361502	1	75750758	75777986	27229	4996,28	0,26	5,01E-01
ENSRNOG00000009956		WNK lysine deficient protein kinase 1	116477	4	219537467	219662816	125350	3199,57	-0,27	5,01E-01
ENSRNOG00000042838	**Junb**	jun B proto-oncogene (Junb), mRNA [S	24517	19	37069075	37070109	1035	352,55	0,43	5,01E-01
ENSRNOG00000011762	**Elf1**	E74-like factor 1 (Elf1), mRNA [Source	85424	15	65490135	65531085	40951	196,30	-0,29	5,01E-01
ENSRNOG00000020707	**Kat5**	K(lysine) acetyltransferase 5 (Kat5), m	192218	1	227898846	227906149	7304	1560,83	0,32	5,01E-01
ENSRNOG00000012134	**Scn4a**	sodium channel, voltage-gated, type I\	25722	10	94256236	94305849	49614	17,61	-0,57	5,02E-01
ENSRNOG00000011404	**Chkb**	choline kinase beta (Chkb), mRNA [So	29367	7	130089653	130093008	3356	1067,97	0,24	5,02E-01
ENSRNOG00000018645	**LOC10091(**	ribosomal protein SA (Rpsa), mRNA [S	29236	8	128006780	128010671	3892	17202,58	0,34	5,02E-01
ENSRNOG00000024617		Uncharacterized protein [Source:UniProtKB/TrE		4	142927550	142928740	1191	23,92	0,59	5,02E-01
ENSRNOG00000042201	**Efcab2**	EF-hand calcium binding domain 2 (Ef	289280	13	100744214	100849978	105765	90,16	-0,42	5,02E-01
ENSRNOG00000000982	**Zkscan5**	zinc finger with KRAB and SCAN dom	304275	12	13224021	13245216	21196	1134,01	0,36	5,03E-01
ENSRNOG00000005776		B-cell lymphoma/leukemia 11B isoforn	314423	6	141005579	141097519	91941	1701,20	0,41	5,03E-01
ENSRNOG00000023386	**Gorasp2**	golgi reassembly stacking protein 2 (G	113961	3	63767465	63782078	14614	2517,64	0,22	5,03E-01
ENSRNOG00000030314	**Golgb1**	golgin B1 (Golgb1), mRNA [Source:Re	192243	11	69853333	69909684	56352	1447,44	0,40	5,04E-01
ENSRNOG00000014702	**Elovl2**	ELOVL fatty acid elongase 2 (Elovl2),	498728	17	23366147	23405731	39585	3032,07	-0,32	5,04E-01
ENSRNOG00000016145	**Strn4**	striatin, calmodulin binding protein 4 (S	308392	1	79987125	80012887	25763	5087,24	0,33	5,04E-01
ENSRNOG00000025776	**Ccdc148**	coiled-coil domain containing 148 (Ccc	311051	3	50007273	50327062	319790	34,35	-0,47	5,04E-01
ENSRNOG00000049056		adenylate kinase 1 [Source:MGI Symbol;Acc:MG		3	16995039	17002961	7923	914,40	0,29	5,04E-01
ENSRNOG00000047118	**LOC68389**	RIKEN cDNA 1700021F05 gene [Sour	683897	20	49934857	49950906	16050	363,64	0,32	5,04E-01
ENSRNOG00000000539	**Zfand3**	zinc finger, AN1-type domain 3 (Zfand	361816	20	10942042	11081446	139405	3602,33	0,22	5,04E-01
ENSRNOG00000002618	**Ivns1abp**	influenza virus NS1A binding protein (I	289089	13	73676540	73683832	7293	2100,93	-0,41	5,04E-01
ENSRNOG00000003307	**Gcdh**	glutaryl-CoA dehydrogenase (Gcdh), r	364975	19	36976120	36982593	6474	1069,16	-0,30	5,04E-01
ENSRNOG00000004014	**Chmp6**	charged multivesicular body protein 6	287873	10	108673903	108679573	5671	777,93	0,32	5,04E-01
ENSRNOG00000006030	**Ptprz1**	protein tyrosine phosphatase, recepto	25613	4	49730552	49927326	196775	32495,77	-0,41	5,04E-01
ENSRNOG00000007420	**Hrh1**	histamine receptor H 1 (Hrh1), mRNA	24448	4	209749004	209750533	1530	92,63	-0,59	5,04E-01
ENSRNOG00000007793	**Pnrc1**	proline-rich nuclear receptor coactivat	286988	5	53083526	53086483	2958	937,02	0,49	5,04E-01
ENSRNOG00000008498	**Gabarapl1**	GABA(A) receptor-associated protein	689161	4	211937460	211946619	9160	10108,41	0,45	5,04E-01
ENSRNOG00000009887	**Arih1**	ariadne homolog, ubiquitin-conjugatin	300756	8	63928310	64030505	102196	2534,45	-0,28	5,04E-01

ENSRNOG00000013813	**Ubtd1**	ubiquitin domain containing 1 (Ubtd1),	309373	1	268681819	268733008	51190	531,47	0,43	5,04E-01
ENSRNOG00000014127	**Rnf151**	ring finger protein 151 (Rnf151), mRN.	302977	10	13899266	13901583	2318	21,39	0,50	5,04E-01
ENSRNOG00000014718	**Acsl3**	acyl-CoA synthetase long-chain family	114024	9	84327545	84351513	23969	2851,20	-0,37	5,04E-01
ENSRNOG00000015051	**Golga7b**	Protein Golga7b [Source:UniProtKB/T	309378	1	268961980	268966788	4809	579,97	0,35	5,04E-01
ENSRNOG00000018118	**Atad3a**	ATPase family, AAA domain containing	298682	5	176665202	176685381	20180	1720,24	0,26	5,04E-01
ENSRNOG00000019624	**Morc2**	MORC family CW-type zinc finger 2 (M	289736	14	84582010	84615107	33098	2212,73	-0,29	5,04E-01
ENSRNOG00000019990	**RGD15662**	similar to RIKEN cDNA 2810428I15 (F	306348	16	20527853	20530325	2473	889,16	0,39	5,04E-01
ENSRNOG00000020081	**As3mt**	arsenic (+3 oxidation state) methyltrar	140925	1	273913120	273945391	32272	38,68	-0,55	5,04E-01
ENSRNOG00000020514	**Sox6**	SRY (sex determining region Y)-box 6	293165	1	192644288	193151614	507327	865,16	-0,41	5,04E-01
ENSRNOG00000020812	**Gys1**	glycogen synthase 1, muscle (Gys1), i	690987	1	102506605	102526038	19434	985,21	0,24	5,04E-01
ENSRNOG00000021223	**Ptpra**	protein tyrosine phosphatase, recepto	25167	3	129475269	129582697	107429	5900,30	-0,33	5,04E-01
ENSRNOG00000022523	**Fkbp5**	FK506 binding protein 5 (Fkbp5), mRN	361810	20	10177067	10219361	42295	977,86	0,30	5,04E-01
ENSRNOG00000022607	**LOC683508**			X	64997194	64999757	2564	479,30	0,26	5,04E-01
ENSRNOG00000024374	**Sorl1**	sortilin-related receptor, LDLR class A	300652	8	44702383	44872500	170118	2247,35	-0,28	5,04E-01
ENSRNOG00000025001	**Pcolce**	procollagen C-endopeptidase enhance	29569	12	24171335	24177682	6348	25,20	-0,53	5,04E-01
ENSRNOG00000025059	**Nxph4**	neurexophilin 4 (Nxph4), mRNA [Sour	59316	7	71007644	71014940	7297	29,21	-0,55	5,04E-01
ENSRNOG00000025278		Protein Scai; RCG37693, isoform CRA	690538	3	28584731	28695956	111226	203,89	-0,45	5,04E-01
ENSRNOG00000028805	**RGD15641**	similar to FLJ46082 protein (RGD1564	499765	3	12858726	12980874	122149	51,55	-0,57	5,04E-01
ENSRNOG00000039197		collagen alpha-1(XV) chain precursor	298069	5	68988937	69056130	67194	20,17	0,51	5,04E-01
ENSRNOG00000047137		Erbb2 interacting protein [Source:MGI	365661	2	53011409	53080898	69490	1125,46	-0,55	5,04E-01
ENSRNOG00000048622	**Sin3b**	SIN3 transcription regulator homolog E	683381	16	18711540	18728708	17169	3747,35	0,26	5,04E-01
ENSRNOG00000017663	**Dolpp1**	dolichyldiphosphatase 1 (Dolpp1), mR	296624	3	14310907	14319391	8485	1035,21	0,28	5,04E-01
ENSRNOG00000042347	**Zfp53**	zinc finger protein 53 (Zfp53), mRNA [	308236	1	62424066	62450381	26316	33,65	-0,48	5,05E-01
ENSRNOG00000021206	**Pla2g16**	phospholipase A2, group XVI (Pla2g16	24913	1	229831424	229864255	32832	396,99	-0,52	5,05E-01
ENSRNOG00000028589		glutamate receptor, ionotropic, AMPA 2	29627	2	198991678	199112785	121108	3535,97	-0,44	5,06E-01
ENSRNOG00000004964	**Erbb3**	v-erb-b2 avian erythroblastic leukemia	29496	7	2964640	2983936	19297	64,66	-0,60	5,06E-01
ENSRNOG00000032705	**Bcl7b**	B-cell CLL/lymphoma 7B (Bcl7b), mRNA [Source		12	26541958	26554279	12322	289,68	0,27	5,06E-01
ENSRNOG00000024657	**Mfsd4**	major facilitator superfamily domain cc	498228	13	53877464	53918741	41278	113,97	-0,47	5,06E-01
ENSRNOG00000046128	**Gpr108**	G protein-coupled receptor 108 (Gpr10	316136	9	8711674	8722083	10410	585,57	0,24	5,06E-01
ENSRNOG00000000219	**Ano10**	Anoctamin [Source:UniProtKB/TrEMB	301111	8	130005122	130077030	71909	1094,77	0,41	5,06E-01
ENSRNOG00000032625		zinc finger protein 235 [Source:MGI Symbol;Acc		1	82234316	82239869	5554	137,69	-0,33	5,07E-01
ENSRNOG00000011696	**Lifr**	leukemia inhibitory factor receptor alph	81680	2	76191377	76227874	36498	360,47	-0,41	5,07E-01
ENSRNOG00000002512	**Gpatch2**	G patch domain containing 2 (Gpatch2	289362	13	110334296	110351724	17429	215,60	0,35	5,07E-01
ENSRNOG00000005291	**Slc38a1**	solute carrier family 38, member 1 (Slc	170567	7	137603305	137667386	64082	1726,71	-0,26	5,07E-01
ENSRNOG00000008247		IKBKB interacting protein (Ikbip), mRN	314730	7	31873293	31891984	18692	650,88	-0,36	5,07E-01
ENSRNOG00000009945	**Pls1**	plastin 1 (Pls1), mRNA [Source:RefSe	315926	8	103009507	103046804	37298	26,56	-0,54	5,07E-01
ENSRNOG00000012450	**Dynlrb2**	dynein light chain roadblock-type 2 (D	361415	19	59812610	59823939	11330	133,76	-0,60	5,07E-01
ENSRNOG00000014078	**Ndufb8**	NADH dehydrogenase (ubiquinone) 1	293991	1	271741825	271746891	5067	5992,52	0,42	5,07E-01

ENSRNOG00000025570	**Os9**	osteosarcoma amplified 9 (Os9), mRN	362891		7	70558019	70584816	26798	4724,99	0,32	5,07E-01
ENSRNOG00000039214	**RGD13054**	uncharacterized protein LOC288545		288545		12	21634746	21639034	4289	1546,58	0,33	5,07E-01
ENSRNOG00000027012	**Usp54**	ubiquitin specific peptidase 54 (Usp54	408223		15	8226916	8291196	64281	1594,62	-0,27	5,07E-01
ENSRNOG00000008785	**Klf5**	Kruppel-like factor 5 (Klf5), mRNA [So	84410		15	87216663	87231849	15187	75,15	-0,40	5,07E-01
ENSRNOG00000004150	**Slc9a7**	solute carrier family 9, subfamily A (NH	317170	X		3226549	3405152	178604	560,73	-0,32	5,08E-01
ENSRNOG00000014703	**Tonsl**	tonsoku-like, DNA repair protein (Tons	366953		7	117676480	117691180	14701	226,21	0,31	5,08E-01
ENSRNOG00000043348	**LOC10036**	ribosomal protein L39 (Rpl39), mRNA	25347	X		123841669	123844612	2944	131,16	-0,35	5,08E-01
ENSRNOG00000024435	**Wbscr17**	Williams-Beuren syndrome chromosome region			12	30671599	30922308	250710	304,61	-0,32	5,08E-01
ENSRNOG00000043128		armadillo repeat containing, X-linked 4	1E+08	X		105389526	105396644	7119	2850,89	0,33	5,09E-01
ENSRNOG00000016231	**Polr2d**	polymerase (RNA) II (DNA directed) p	364834		18	24287960	24295092	7133	884,69	0,27	5,09E-01
ENSRNOG00000017817	**Cox4i1**	cytochrome c oxidase subunit IV isofo	29445		19	64965335	64971575	6241	10387,81	0,33	5,09E-01
ENSRNOG00000039207	**Ccdc71l**	Protein Ccdc71l [Source:UniProtKB/T	500640		6	61298880	61299530	651	121,22	0,45	5,09E-01
ENSRNOG00000018123	**Ccny**	cyclin Y (Ccny), mRNA [Source:RefSe	361261		17	64106292	64238150	131859	2092,70	0,39	5,09E-01
ENSRNOG00000031041	**Rps4y2**	ribosomal protein S4, Y-linked 2 (Rps4	690845		4	246879802	246880735	934	34,66	0,47	5,09E-01
ENSRNOG00000004735	**Gzf1**	GDNF-inducible zinc finger protein 1 (	311508		3	149414356	149426464	12109	535,03	-0,34	5,10E-01
ENSRNOG00000001019		ATPase, H+ transporting, lysosomal V	116455		12	39234008	39296609	62602	1128,71	0,21	5,10E-01
ENSRNOG00000011622	**Echdc1**	enoyl CoA hydratase domain containir	361465		1	32308393	32350239	41847	456,63	-0,53	5,10E-01
ENSRNOG00000012168		F-box and leucine-rich repeat protein 21 [Source			17	10683643	10692612	8970	126,84	-0,37	5,10E-01
ENSRNOG00000012660	**Postn**	periostin, osteoblast specific factor (Po	361945		2	163331262	163362444	31183	33,34	-0,58	5,10E-01
ENSRNOG00000038818	**Zkscan4**	Protein Zfp307 [Source:UniProtKB/Tr	291164		17	58313742	58319451	5710	104,26	-0,31	5,10E-01
ENSRNOG00000011902	**Rdh11**	retinol dehydrogenase 11 (all-trans/9-c	362757		6	115043173	115059292	16120	1519,03	0,31	5,10E-01
ENSRNOG00000004733	**Wdr25**	WD repeat domain 25-like (Wdr25l), m	314443		6	141975870	142103153	127284	111,51	0,34	5,10E-01
ENSRNOG00000015869	**Pccb**	propionyl CoA carboxylase, beta polyp	24624		8	108785884	108836132	50249	1519,36	0,25	5,10E-01
ENSRNOG00000026114	**Ap5b1**	adaptor-related protein complex 5, bet	361709		1	227878862	227881595	2734	306,43	0,29	5,10E-01
ENSRNOG00000031269	**RGD13046**	similar to CG9646-PA (RGD1304694),	362974		7	125571736	125607106	35371	4520,98	0,33	5,10E-01
ENSRNOG00000043435	**RGD15663**	Protein RGD1566314 [Source:UniPro	288106		11	60808834	60882302	73469	53,79	0,43	5,10E-01
ENSRNOG00000048914	**Traf1**	TNF receptor-associated factor 1 (Tra	687813		3	19324027	19339773	15747	51,82	-0,59	5,10E-01
ENSRNOG00000025997	**Mrrf**	mitochondrial ribosome recycling facto	311903		3	20773165	20829830	56666	494,17	-0,30	5,10E-01
ENSRNOG00000004392	**Aatk**	apoptosis-associated tyrosine kinase (	690853		10	108786271	108822529	36259	3008,05	0,27	5,10E-01
ENSRNOG00000008288	**Akirin2**	akirin 2 (Akirin2), mRNA [Source:RefS	297968		5	54571138	54585493	14356	2733,95	0,29	5,10E-01
ENSRNOG00000010980	**Proser1**	proline and serine rich 1 (Proser1), mR	310417		2	162370018	162389653	19636	730,37	0,24	5,10E-01
ENSRNOG00000001616	**Senp7**	SUMO1/sentrin specific peptidase 7 (S	288167		11	50062235	50209557	147323	457,02	-0,48	5,11E-01
ENSRNOG00000019883	**Pak4**	p21 protein (Cdc42/Rac)-activated kin	292756		1	86408071	86417580	9510	648,18	0,41	5,11E-01
ENSRNOG00000029738	**Diras1**	DIRAS family, GTP-binding RAS-like 1	366826		7	11723556	11728420	4865	1536,94	0,28	5,11E-01
ENSRNOG00000009812	**RGD15594**	similar to SET binding factor 2 (RGD1	691042		1	181947530	182408115	460586	4348,92	-0,22	5,11E-01
ENSRNOG00000006004	**Phc2**	polyhomeotic homolog 2 (Drosophila)	313038		5	150620498	150668954	48457	3547,92	0,32	5,11E-01
ENSRNOG00000018271	**Scamp4**	secretory carrier membrane protein 4	65170		7	12137246	12149666	12421	1375,54	0,36	5,11E-01
ENSRNOG00000048274		Uncharacterized protein [Source:UniProtKB/TrE			7	140377089	140377364	276	58,69	-0,40	5,11E-01

ENSRNOG00000013717	**Bmp6**	bone morphogenetic protein 6 (Bmp6)	25644	17	28869728	29029564	159837	821,87	-0,55	5,11E-01
ENSRNOG00000001090	**Stard13**	StAR-related lipid transfer (START) dc	498130	12	1007827	1174378	166552	151,11	-0,37	5,11E-01
ENSRNOG00000004598	**Tsen54**	tRNA splicing endonuclease 54 homol	690308	10	103919116	103926625	7510	393,81	0,34	5,11E-01
ENSRNOG00000011290	**Tm2d3**	TM2 domain containing 3 (Tm2d3), ml	292995	1	127666050	127679266	13217	430,96	0,33	5,11E-01
ENSRNOG00000015049	**Scn5a**	sodium channel, voltage-gated, type V	25665	8	127375781	127471879	96099	33,09	-0,51	5,11E-01
ENSRNOG00000016896	**Rpl3**	ribosomal protein L3 (Rpl3), mRNA [S	300079	7	121287085	121292461	5377	13078,79	0,24	5,11E-01
ENSRNOG00000021110	**Mllt11**	myeloid/lymphoid or mixed-lineage leu	295264	2	215889656	215898937	9282	15927,93	0,34	5,11E-01
ENSRNOG00000028168	**Ankrd16**	ankyrin repeat domain 16 (Ankrd16), r	307102	17	72090953	72102223	11271	204,07	0,33	5,11E-01
ENSRNOG00000042758	**Tmem243**	transmembrane protein 243, mitochon	499990	4	21834167	21852015	17849	214,13	0,35	5,11E-01
ENSRNOG00000046085		uncharacterized protein LOC1002331	1E+08	6	135837722	135842402	4681	526,07	0,25	5,11E-01
ENSRNOG00000001729	**Xxylt1**	Protein Xxylt1 [Source:UniProtKB/TrE	363799	11	76271214	76402197	130984	469,59	-0,26	5,11E-01
ENSRNOG00000007578	**Zfp830**	zinc finger protein 830 (Zfp830), mRN	497967	10	69748958	69750421	1464	422,63	0,31	5,11E-01
ENSRNOG00000011282	**Ppp2r1a**	protein phosphatase 2, regulatory sub	117281	1	61883998	61903234	19237	19031,06	0,22	5,11E-01
ENSRNOG00000012337	**Pde1c**	phosphodiesterase 1C (Pde1c), mRN	81742	4	151012763	151485371	472609	100,52	-0,49	5,11E-01
ENSRNOG00000037165		Protein Nipal3 [Source:UniProtKB/TrE	502990	5	157571436	157606377	34942	359,35	0,49	5,11E-01
ENSRNOG00000028650		Uncharacterized protein [Source:UniProtKB/TrE		6	146172025	146187666	15642	354,42	-0,40	5,12E-01
ENSRNOG00000032254	**Sin3a**	SIN3 transcription regulator homolog A	363067	8	60332843	60373239	40397	1358,34	-0,21	5,12E-01
ENSRNOG00000002775	**Npl**	N-acetylneuraminate pyruvate lyase (N	304860	13	75903278	75946056	42779	255,71	-0,45	5,12E-01
ENSRNOG00000049666		Protein 1700086L19Rik; RCG62002 [Source:Ur		6	106657337	106657492	156	88,28	-0,50	5,12E-01
ENSRNOG00000047960	**Rab9b**	RAB9B, member RAS oncogene famil	367915	X	107414930	107426468	11539	647,46	-0,40	5,12E-01
ENSRNOG00000006761	**Sh3gl2**	SH3-domain GRB2-like 2 (Sh3gl2), ml	116743	5	107614520	107653528	39009	2362,32	0,30	5,13E-01
ENSRNOG00000006126	**Nup88**	nucleoporin 88 (Nup88), mRNA [Sourc	113929	10	57326777	57351383	24607	1390,40	0,34	5,13E-01
ENSRNOG00000001706		kalirin, RhoGEF kinase (Kalrn), mRNA	84009	11	72701013	73115328	414316	2971,54	-0,29	5,13E-01
ENSRNOG00000005323		Uncharacterized protein [Source:UniProtKB/TrE		6	108493028	108565605	72578	1045,54	0,29	5,13E-01
ENSRNOG00000002420	**Zc3h7a**	zinc finger CCCH type containing 7 A (	360466	10	3366355	3405540	39186	662,51	-0,33	5,13E-01
ENSRNOG00000002811	**Fam5c**	family with sequence similarity 5, mem	286901	13	68507843	68937974	430132	293,05	0,35	5,14E-01
ENSRNOG00000010544		large tumor suppressor 2 [Source:MGI Symbol;A		15	41788720	41828704	39985	1219,52	0,36	5,14E-01
ENSRNOG00000010728	**Stradb**	STE20-related kinase adaptor beta (S	501146	9	65570181	65589257	19077	2465,30	0,42	5,14E-01
ENSRNOG00000012786	**Pgrmc1**	progesterone receptor membrane component 1		X	123352162	123360154	7993	3037,86	-0,34	5,14E-01
ENSRNOG00000027690	**LOC10090**	eukaryotic translation initiation factor 3	299872	13	105112074	105144805	32732	293,03	-0,45	5,14E-01
ENSRNOG00000032441	**Pan2**	PAN2 polyA specific ribonuclease sub	408200	7	2697335	2714080	16746	551,66	0,25	5,14E-01
ENSRNOG00000033741	**Ankrd34a**	ankyrin repeat domain 34A (Ankrd34a	295283	2	218208658	218212088	3431	878,81	0,33	5,14E-01
ENSRNOG00000016692	**Hsdl2**	hydroxysteroid dehydrogenase like 2 (	313200	5	80949471	80982918	33448	579,05	-0,35	5,14E-01
ENSRNOG00000020571	**Mpg**	N-methylpurine-DNA glycosylase (Mpg	24561	10	15556935	15563138	6204	234,78	0,32	5,14E-01
ENSRNOG00000002810	**Gfpt2**	glutamine-fructose-6-phosphate transa	360518	10	35051132	35097280	46149	389,96	0,33	5,14E-01
ENSRNOG00000007821	**Dyrk2**	dual-specificity tyrosine-(Y)-phosphory	314862	7	61776788	61789208	12421	1504,40	-0,34	5,14E-01
ENSRNOG00000008614	**Zfyve1**	zinc finger, FYVE domain containing 1	299188	6	118461006	118509830	48825	1545,48	0,41	5,14E-01
ENSRNOG00000029871	**Slc8a3**	solute carrier family 8 (sodium/calcium	140448	6	113051975	113244454	192480	638,01	-0,27	5,14E-01

ENSRNOG00000002653	**Kcnk2**	potassium channel, subfamily K, mem	170899	13	112318475	112456560	138086	441,35	0,36	5,15E-01
ENSRNOG00000022196	**Bmpr2**	bone morphogenetic protein receptor,	140590	9	66371991	66479596	107606	1694,02	-0,36	5,15E-01
ENSRNOG00000008236	**Decr1**	2,4-dienoyl CoA reductase 1, mitochor	117543	5	34253236	34280749	27514	661,27	-0,48	5,15E-01
ENSRNOG00000008155	**Dus4l**	dihydrouridine synthase 4-like (S. cere	366593	6	59601170	59615497	14328	101,05	-0,39	5,16E-01
ENSRNOG00000008215	**Trim47**	tripartite motif-containing 47 (Trim47),	690374	10	103626183	103630654	4472	217,45	-0,54	5,16E-01
ENSRNOG00000014287	**Stk11**	serine/threonine kinase 11 (Stk11), mF	314621	7	12610856	12627616	16761	2531,43	0,23	5,16E-01
ENSRNOG00000015426	**Purg**	purine-rich element binding protein G	361162	16	62146553	62148455	1903	257,31	0,31	5,16E-01
ENSRNOG00000019613	**Syt9**	synaptotagmin IX (Syt9), mRNA [Sour	60564	1	178591098	178768239	177142	128,71	-0,39	5,16E-01
ENSRNOG00000023210	**Trappc10**	trafficking protein particle complex 10	309678	20	13338328	13398188	59861	3583,27	0,22	5,16E-01
ENSRNOG00000025701	**Mki67ip**	Mki67 (FHA domain) interacting nucle	246042	13	39397765	39407568	9804	1262,34	0,31	5,16E-01
ENSRNOG00000027498	**Engase**	endo-beta-N-acetylglucosaminidase (E	303702	10	107137511	107150161	12651	423,23	0,30	5,16E-01
ENSRNOG00000009071	**Gripap1**	GRIP1 associated protein 1 (Gripap1)	116493	X	16305164	16334935	29772	2093,47	0,31	5,16E-01
ENSRNOG00000002883	**Alg1**	ALG1, chitobiosyldiphosphodolichol be	360475	10	9309794	9320002	10209	365,11	0,26	5,16E-01
ENSRNOG00000015753	**Epn1**	Epsin 1 (Epn1), mRNA [Source:RefSe	117277	1	76226167	76242237	16071	7672,10	0,30	5,16E-01
ENSRNOG00000028426		MCF.2 cell line derived transforming se	117020	16	81326363	81465756	139394	6431,38	0,30	5,16E-01
ENSRNOG00000009824	**Irf2**	interferon regulatory factor 2 (Irf2), mF	290749	16	48295630	48404755	109126	362,18	0,33	5,16E-01
ENSRNOG00000016781	**Ppib**	peptidylprolyl isomerase B (Ppib), mR	64367	8	71388458	71394501	6044	4444,54	0,24	5,16E-01
ENSRNOG00000027722	**H1fx**	Protein H1fx [Source:UniProtKB/TrEM	500252	4	185137082	185137660	579	768,57	0,46	5,17E-01
ENSRNOG00000015161	**LOC688754**			18	50808403	50809253	851	264,35	0,36	5,17E-01
ENSRNOG00000001750	**Chrd**	chordin (Chrd), mRNA [Source:RefSec	117275	11	86930422	86939118	8697	107,53	-0,34	5,17E-01
ENSRNOG00000011417	**Pde3b**	phosphodiesterase 3B, cGMP-inhibite	29516	1	190865614	191050207	184594	423,12	-0,36	5,17E-01
ENSRNOG00000004322		SH3-domain kinase binding protein 1	84357	X	38095016	38505128	410113	1623,42	-0,26	5,17E-01
ENSRNOG00000017810	**Uros**	uroporphyrinogen III synthase (Uros),	309070	1	212705708	212726235	20528	240,72	0,44	5,18E-01
ENSRNOG00000000632	**Cdk1**	cyclin-dependent kinase 1 (Cdk1), mR	54237	20	22694920	22709859	14940	1164,41	0,40	5,18E-01
ENSRNOG00000003109	**Btbd17**	BTB (POZ) domain containing 17 (Btb	303660	10	102991771	102998271	6501	1146,75	0,36	5,18E-01
ENSRNOG00000015573	**Ctbs**	chitobiase, di-N-acetyl- (Ctbs), mRNA	81652	2	270832835	270847013	14179	153,58	-0,48	5,18E-01
ENSRNOG00000034303	**Spon1**	spondin 1, extracellular matrix protein	64456	1	185603830	185902796	298967	2861,99	-0,43	5,18E-01
ENSRNOG00000007331	**Rragd**	Ras-related GTP binding D (Rragd), m	297960	5	52808853	52844544	35692	778,89	-0,35	5,18E-01
ENSRNOG00000037514	**Qser1**	glutamine and serine rich 1 (Qser1), m	311266	3	101330796	101391977	61182	1100,83	-0,43	5,18E-01
ENSRNOG00000031475	**Col16a1**	collagen, type XVI, alpha 1 (Col16a1),	366474	5	151976658	152030103	53446	1477,41	-0,50	5,19E-01
ENSRNOG00000005185	**Nxph3**	neurexophilin 3 (Nxph3), mRNA [Sour	59315	10	83138261	83142939	4679	727,49	0,35	5,20E-01
ENSRNOG00000006174	**Grid2**	glutamate receptor, ionotropic, delta 2	79220	4	159485887	160229216	743330	228,01	-0,40	5,20E-01
ENSRNOG00000006570	**Plekhg3**	Protein Plekhg3 [Source:UniProtKB/T	314249	6	109036102	109053184	17083	197,88	-0,40	5,20E-01
ENSRNOG00000050539	**Fbln5**	fibulin 5 (Fbln5), mRNA [Source:RefSe	29158	6	134856700	134935853	79154	172,07	-0,56	5,20E-01
ENSRNOG00000008168	**Wnt5b**	wingless-type MMTV integration site fa	282582	4	217413755	217429687	15933	30,66	-0,55	5,20E-01
ENSRNOG00000001139		suppressor of defective silencing 3 ho	360819	12	47018975	47040897	21923	2880,43	0,24	5,20E-01
ENSRNOG00000008680	**Loxl1**	lysyl oxidase-like 1 (Loxl1), mRNA [So	315714	8	62843791	62868157	24367	161,82	-0,36	5,20E-01
ENSRNOG00000014765	**Vapa**	VAMP (vesicle-associated membrane	58857	9	112833702	112863559	29858	6607,20	0,23	5,20E-01

ENSRNOG00000018293	**Strip1**	Protein Fam40a [Source:UniProtKB/T	362012	2	229860322	229880245	19924	4472,74	0,31	5,20E-01
ENSRNOG00000020020	**Wbp1l**	WW domain binding protein 1-like (Wb	309456	1	273789070	273845332	56263	2195,15	0,26	5,20E-01
ENSRNOG00000037239	**Flna**	filamin A, alpha (Flna), mRNA [Source	293860	1	152204099	152227391	23293	5993,92	0,31	5,20E-01
ENSRNOG00000026177		maltase-glucoamylase [Source:MGI Symbol;Acc		4	133643837	133783434	139598	19,21	-0,58	5,20E-01
ENSRNOG00000003368	**Tspyl2**	TSPY-like 2 (Tspyl2), mRNA [Source:F	302612	X	64168075	64173685	5611	1353,94	0,42	5,20E-01
ENSRNOG00000000470	**Vps52**	vacuolar protein sorting 52 homolog (S	25218	20	7490618	7500200	9583	3239,80	0,22	5,20E-01
ENSRNOG00000047355		Uncharacterized protein [Source:UniProtKB/TrE		2	5010662	5032471	21810	26,97	-0,52	5,20E-01
ENSRNOG00000024291		Protein Tex9 [Source:UniProtKB/TrEM	300822	8	73967926	74003852	35927	74,85	-0,53	5,21E-01
ENSRNOG00000011011	**Nmb**	neuromedin B (Nmb), mRNA [Source:	499194	1	143673434	143676178	2745	51,58	-0,58	5,21E-01
ENSRNOG00000019724	**Ganab**	glucosidase, alpha; neutral AB (Ganab	293721	1	232034218	232053998	19781	6187,98	0,30	5,21E-01
ENSRNOG00000005729	**Palm3**	Protein Palm3; RCG51505, isoform Cl	1E+08	19	36120472	36128572	8101	369,99	0,48	5,21E-01
ENSRNOG00000009982		purine nucleoside phosphorylase [Sou	290029	15	31712198	31716288	4091	3446,75	0,25	5,21E-01
ENSRNOG00000011413	**Scp2**	sterol carrier protein 2 (Scp2), mRNA [	25541	5	131495400	131584319	88920	3966,44	-0,36	5,21E-01
ENSRNOG00000013136	**Oxsr1**	oxidative-stress responsive 1 (Oxsr1),	316064	8	127127110	127216712	89603	2674,33	0,44	5,21E-01
ENSRNOG00000017979		mRNA turnover protein 4 homolog [So	298586	5	161555067	161560580	5514	862,35	0,26	5,21E-01
ENSRNOG00000008113	**RGD15611**	similar to mKIAA1522 protein (RGD15	500552	5	151141119	151147606	6488	395,14	0,33	5,21E-01
ENSRNOG00000008942		cDNA sequence BC027072 [Source:M	313891	6	33204235	33211837	7603	18,12	-0,57	5,21E-01
ENSRNOG00000009222	**Epha2**	Eph receptor A2 (Epha2), mRNA [Sou	366492	5	163558779	163586206	27428	25,17	0,50	5,21E-01
ENSRNOG00000016243	**Casq2**	calsequestrin 2 (cardiac muscle) (Cas	29209	2	223945611	224001892	56282	17,76	-0,55	5,21E-01
ENSRNOG00000023333	**Aff2**	Protein Aff2 [Source:UniProtKB/TrEM	293922	1	149269845	149370503	100659	318,34	-0,30	5,21E-01
ENSRNOG00000029133	**Aes**	amino-terminal enhancer of split (Aes)	29466	7	11180239	11187085	6847	8956,35	0,33	5,21E-01
ENSRNOG00000047628	**Khsrp**	KH-type splicing regulatory protein (Kh	171137	9	9014580	9023574	8995	1370,39	0,25	5,21E-01
ENSRNOG00000002254	**Tmem33**	transmembrane protein 33 (Tmem33),	59303	14	42334624	42355562	20939	1017,18	-0,27	5,21E-01
ENSRNOG00000002580	**Trmt1l**	tRNA methyltransferase 1 homolog-lik	304851	13	73748227	73781248	33022	761,45	0,34	5,21E-01
ENSRNOG00000007926		mitogen activated protein kinase kinas	29568	8	67784869	68009803	224935	1044,81	0,33	5,21E-01
ENSRNOG00000001654	**Cpox**	coproporphyrinogen oxidase (Cpox), r	304024	11	47229585	47239564	9980	589,14	0,39	5,21E-01
ENSRNOG00000033729		zinc finger protein 719 [Source:MGI Symbol;Acc		1	100574928	100581740	6813	84,93	-0,40	5,21E-01
ENSRNOG00000015206	**Alad**	aminolevulinate dehydratase (Alad), m	25374	5	82488238	82498717	10480	612,05	0,26	5,21E-01
ENSRNOG00000019639	**Pgpep1**	pyroglutamyl-peptidase I (Pgpep1), ml	290648	16	20379057	20388077	9021	1160,01	0,25	5,21E-01
ENSRNOG00000008017	**Cdk5**	cyclin-dependent kinase 5 (Cdk5), mR	140908	4	7294309	7298788	4480	2545,97	0,27	5,21E-01
ENSRNOG00000011528	**Alg14**	ALG14, UDP-N-acetylglucosaminyltra	362031	2	242894212	242970804	76593	540,16	0,25	5,21E-01
ENSRNOG00000016731	**Tpm2**	tropomyosin 2, beta (Tpm2), mRNA [S	500450	5	63541287	63550225	8939	75,77	-0,48	5,21E-01
ENSRNOG00000020417	**Gsk3a**	glycogen synthase kinase 3 alpha (Gs	50686	1	83366057	83374707	8651	5903,18	0,32	5,21E-01
ENSRNOG00000006514	**Vezt**	vezatin, adherens junctions transmem	299738	7	34946146	35009764	63619	1826,83	0,37	5,22E-01
ENSRNOG00000000501	**Zfp523**	zinc finger protein 523 (Zfp523), mRN	361809	20	9964697	9985907	21211	1174,98	0,32	5,22E-01
ENSRNOG00000014475	**Slc31a1**	solute carrier family 31 (copper transp	171135	5	82342331	82369096	26766	1010,90	-0,42	5,22E-01
ENSRNOG00000013956	**Rnf38**	ring finger protein 38 (Rnf38), mRNA [	171501	5	64130111	64160870	30760	927,34	-0,22	5,23E-01
ENSRNOG00000016714	**Nrap**	nebulin-related anchoring protein (Nra	307982	1	284485970	284562973	77004	24,88	-0,48	5,23E-01

ENSRNOG00000017329	**LOC68817(**	transmembrane protein 129 (Tmem12	305458		14	83040119	83045463	5345	573,45	0,27	5,23E-01
ENSRNOG00000024345	**Pard3b**	par-3 partitioning defective 3 homolog	301455		9	68228908	69236542	1007635	157,40	-0,43	5,23E-01
ENSRNOG00000025644	**LOC49933'**	RIKEN cDNA D030056L22 gene [Sou	499331		1	241765683	241769891	4209	1028,21	0,30	5,23E-01
ENSRNOG00000030425		Uncharacterized protein [Source:UniProtKB/TrE			13	13486009	13487570	1562	26,62	0,52	5,23E-01
ENSRNOG00000047129	**Tcf19**	transcription factor 19 (Tcf19), mRNA [	406195		20	5831136	5834643	3508	106,53	0,42	5,23E-01
ENSRNOG00000006698	**Rab33a**	RAB33A, member RAS oncogene far	317580	X		135420269	135431674	11406	815,14	0,43	5,23E-01
ENSRNOG00000015797	**Neurl2**	neuralized homolog 2 (Drosophila) (Ne	311633		3	167481588	167484308	2721	114,84	0,36	5,23E-01
ENSRNOG00000017438	**Gfra1**	GDNF family receptor alpha 1 (Gfra1),	25454		1	286571283	286934801	363519	369,76	-0,33	5,23E-01
ENSRNOG00000048291	**Tbcc**	tubulin folding cofactor C (Tbcc), mRN	316221		9	15314178	15315200	1023	884,54	0,44	5,23E-01
ENSRNOG00000008277	**Bag1**	BCL2-associated athanogene (Bag1),	297994		5	61785751	61798332	12582	1700,54	0,23	5,23E-01
ENSRNOG00000007051	**Rraga**	Ras-related GTP binding A (Rraga), m	117044		5	108928889	108930465	1577	2569,13	0,32	5,23E-01
ENSRNOG00000003714	**Clk4**	CDC-like kinase 4 (Clk4), mRNA [Sou	287269		10	36487192	36504760	17569	246,43	-0,38	5,23E-01
ENSRNOG00000048009		Uncharacterized protein [Source:UniProtKB/TrE			9	73791854	73807324	15471	76,30	0,36	5,23E-01
ENSRNOG00000017706	**Pqlc2**	PQ loop repeat containing 2 (Pqlc2), n	362642		5	161489691	161497053	7363	176,22	0,32	5,24E-01
ENSRNOG00000021365	**Ybey**	ybeY metallopeptidase (Ybey), mRNA	361822		20	15075086	15084562	9477	204,96	0,27	5,24E-01
ENSRNOG00000047988	**LOC10036:**	Protein LOC100363408; RCG40058 [	502367		1	205099360	205099958	599	691,64	0,43	5,24E-01
ENSRNOG00000021637	**Ddx46**	DEAD (Asp-Glu-Ala-Asp) box polypep	245957		17	11582992	11626966	43975	1901,30	0,41	5,24E-01
ENSRNOG00000025034	**RGD13045**	similar to RIKEN cDNA A430005L14 (I	362671		5	174734521	174738965	4445	665,86	0,27	5,24E-01
ENSRNOG00000049931	**LOC28891:**	similar to LEYDIG CELL TUMOR 10 K	288913		19	36330664	36334796	4133	3417,96	0,33	5,24E-01
ENSRNOG00000023554		Uncharacterized protein [Source:UniProtKB/TrE			19	51995122	52176850	181729	29,54	-0,55	5,24E-01
ENSRNOG00000013851	**Spry4**	sprouty homolog 4 (Drosophila) (Spry4	291610		18	32272760	32287666	14907	738,25	-0,34	5,24E-01
ENSRNOG00000030027	**Fbxw17**	F-box and WD-40 domain protein 17 (	361219		17	17134537	17152331	17795	242,22	0,43	5,24E-01
ENSRNOG00000018936		Protein LOC100360638 [Source:UniP	1E+08		1	86096157	86112055	15899	100,71	-0,47	5,25E-01
ENSRNOG00000015699	**Lman2l**	lectin, mannose-binding 2-like (Lman2	301343		9	42675165	42676878	1714	219,53	0,31	5,25E-01
ENSRNOG00000009756		protein kinase C and casein kinase su	124461		7	124215247	124306978	91732	783,59	-0,32	5,25E-01
ENSRNOG00000011910	**Hnrnpr**	heterogeneous nuclear ribonucleoprot	319110		5	158435421	158467206	31786	182,25	-0,41	5,25E-01
ENSRNOG00000014265	**Tnfrsf19**	tumor necrosis factor receptor superfa	290300		15	45064736	45127279	62544	413,96	-0,28	5,25E-01
ENSRNOG00000007240	**Rrs1**	RRS1 ribosome biogenesis regulator I	297784		5	14230035	14231132	1098	611,56	0,25	5,25E-01
ENSRNOG00000017607	**Faf2**	Fas associated factor family member 2	291000		17	12613754	12653474	39721	1096,13	0,35	5,25E-01
ENSRNOG00000020743	**Cyp2s1**	cytochrome P450, family 2, subfamily	308445		1	83854677	83868967	14291	36,29	-0,50	5,25E-01
ENSRNOG00000045699		Uncharacterized protein [Source:UniProtKB/TrE			14	94106092	94107553	1462	119,45	-0,38	5,25E-01
ENSRNOG00000050233	**Cchcr1**	coiled-coil alpha-helical rod protein 1 (	406196		20	5817711	5826710	9000	302,28	0,29	5,25E-01
ENSRNOG00000002089	**Ccng2**	cyclin G2 (Ccng2), mRNA [Source:Ref	29157		14	16193744	16202239	8496	2552,87	0,47	5,26E-01
ENSRNOG00000005524	**Pno1**	partner of NOB1 homolog (S. cerevisia	289809		14	100166648	100175214	8567	811,64	0,33	5,26E-01
ENSRNOG00000012878	**Atp7b**	ATPase, Cu++ transporting, beta poly	24218		16	74495179	74574801	79623	178,36	-0,45	5,26E-01
ENSRNOG00000024437	**Blcap**	bladder cancer associated protein (Blc	171113		3	161557723	161559527	1805	3677,04	0,26	5,26E-01
ENSRNOG00000003732	**Flrt2**	fibronectin leucine rich transmembrane	299236		6	128739108	128828633	89526	565,79	-0,28	5,26E-01
ENSRNOG00000030954	**Fat1**	FAT atypical cadherin 1 (Fat1), mRNA	83720		16	50093094	50220784	127691	7273,33	0,27	5,26E-01

ENSRNOG00000010007	**Srsf4**	serine/arginine-rich splicing factor 4 (S	362612	5	153711721	153739487	27767	2241,80	0,22	5,27E-01
ENSRNOG00000006701	**Fgd6**	FYVE, RhoGEF and PH domain conta	500824	7	35009998	35131012	121015	237,40	-0,29	5,28E-01
ENSRNOG00000000142	**Plxdc2**	plexin domain containing 2 (Plxdc2), n	361282	17	84957327	85343868	386542	254,10	-0,34	5,28E-01
ENSRNOG00000008522	**Ogg1**	8-oxoguanine DNA glycosylase (Ogg1	81528	4	208580193	208586457	6265	261,29	-0,44	5,28E-01
ENSRNOG00000010636	**Selrc1**	Sel1 repeat containing 1 (Selrc1), mRI	298377	5	131772051	131782841	10791	235,02	0,31	5,28E-01
ENSRNOG00000019147	**Stom**	stomatin (Stom), mRNA [Source:RefS	296655	3	19833117	19855740	22624	724,54	-0,55	5,28E-01
ENSRNOG00000047363	**Kcnk13**	potassium channel, subfamily K, mem	64120	6	133202980	133305227	102248	40,03	-0,44	5,28E-01
ENSRNOG00000014456	**Coq10b**	coenzyme Q10 homolog B (S. cerevis	301416	9	61337179	61357089	19911	554,64	-0,26	5,28E-01
ENSRNOG00000030116	**Rbfa**	ribosome binding factor A (Rbfa), mRN	307235	18	72092362	72102513	10152	809,54	0,23	5,28E-01
ENSRNOG00000014980	**Mob4**	MOB family member 4, phocein (Mob4	171050	9	61403839	61427803	23965	1268,10	-0,30	5,28E-01
ENSRNOG00000012016	**Npc1**	Niemann-Pick disease, type C1 (Npc1	266732	18	3626015	3671642	45628	1449,86	-0,33	5,28E-01
ENSRNOG00000043366				6	61299592	61300029	438	240,75	0,34	5,28E-01
ENSRNOG00000010133	**Bpgm**	2,3-bisphosphoglycerate mutase (Bpg	296973	4	61650881	61679068	28188	2151,72	-0,22	5,28E-01
ENSRNOG00000001104	**Foxk1**	forkhead box K1 (Foxk1), mRNA [Sou	304298	12	16212041	16270069	58029	403,46	0,23	5,28E-01
ENSRNOG00000027739	**Cndp1**	carnosine dipeptidase 1 (metallopeptic	307212	18	80511689	80534735	23047	32,60	-0,51	5,28E-01
ENSRNOG00000020248	**Ina**	internexin neuronal intermediate filam	24503	1	274212015	274224817	12803	10229,30	0,37	5,29E-01
ENSRNOG00000018781	**Map1s**	microtubule-associated protein 1S (Ma	290640	16	20126207	20136394	10188	1896,89	0,28	5,29E-01
ENSRNOG00000015576	**Hsdl1**	hydroxysteroid dehydrogenase like 1 (	361418	19	62959341	62965942	6602	2067,37	0,34	5,29E-01
ENSRNOG00000002881	**Ddr2**	discoidin domain receptor tyrosine kin	685781	13	92942668	92987943	45276	304,49	-0,52	5,29E-01
ENSRNOG00000033134		myocyte enhancer factor 2C [Source:N	499497	2	11592404	11680469	88066	2144,23	-0,44	5,29E-01
ENSRNOG00000015678	**Spata1**	spermatogenesis associated 1 (Spata	362056	2	270841391	270886763	45373	27,24	-0,51	5,29E-01
ENSRNOG00000025730	**Armcx3**	armadillo repeat containing, X-linked 3	367902	X	105463207	105466731	3525	1701,67	0,27	5,30E-01
ENSRNOG00000011778	**Blvra**	biliverdin reductase A (Blvra), mRNA [	116599	3	126078456	126103260	24805	191,96	-0,41	5,30E-01
ENSRNOG00000020908	**LOC30356**	cDNA sequence BC030867 [Source:M	303566	10	89978419	90001064	22646	58,16	0,46	5,30E-01
ENSRNOG00000020164	**RGD15633**	Protein RGD1563319 [Source:UniPro	293632	1	222417535	222432332	14798	120,92	-0,50	5,30E-01
ENSRNOG00000020424	**Ppapdc1a**	phosphatidic acid phosphatase type 2	309014	1	209449940	209577459	127520	383,55	-0,54	5,30E-01
ENSRNOG00000010892	**Ythdf2**	YTH domain family, member 2 (Ythdf2	313053	5	154038417	154062397	23981	1844,30	0,23	5,30E-01
ENSRNOG00000020463	**Eif1ad**	eukaryotic translation initiation factor 1	293673	1	227675718	227680195	4478	740,83	0,24	5,30E-01
ENSRNOG00000021164	**Stip1**	stress-induced phosphoprotein 1 (Stip	192277	1	229264912	229291272	26361	4751,34	0,29	5,31E-01
ENSRNOG00000048733	**Nup62**	nucleoporin 62 (Nup62), mRNA [Sour	65274	1	101889514	101891781	2268	2308,57	0,22	5,31E-01
ENSRNOG00000018723	**Cops7b**	COP9 signalosome subunit 7B (Cops7	363273	9	93335413	93361000	25588	773,46	0,29	5,31E-01
ENSRNOG00000000811	**Pkib**	protein kinase (cAMP-dependent, cata	24678	20	42346937	42440588	93652	18,00	-0,57	5,32E-01
ENSRNOG00000001255	**Mlxip**	Protein Mlxip [Source:UniProtKB/TrEI	304479	12	40444472	40510059	65588	610,54	0,33	5,32E-01
ENSRNOG00000001733	**Ppp1r2**	protein phosphatase 1, regulatory (inh	192361	11	75823345	76106552	283208	1017,91	0,32	5,32E-01
ENSRNOG00000004553		COX20 cytochrome C oxidase asseml	289278	13	100661214	100666065	4852	664,68	-0,33	5,32E-01
ENSRNOG00000010146	**Ndn**	necdin, melanoma antigen (MAGE) fa	308690	1	124119141	124120755	1615	11601,97	0,31	5,32E-01
ENSRNOG00000015602	**Cdh2**	cadherin 2 (Cdh2), mRNA [Source:Re	83501	18	7997533	8212372	214840	8928,34	-0,21	5,32E-01
ENSRNOG00000015898		fer (fms/fps related) protein kinase, tes	301737	9	111141130	111404714	263585	492,18	-0,40	5,32E-01

ENSRNOG00000016103	**Nkd2**	naked cuticle homolog 2 (Drosophila)	308068	1	33482721	33509554	26834	434,31	-0,37	5,32E-01
ENSRNOG00000017586				10	14348352	14348831	480	250,71	0,29	5,32E-01
ENSRNOG00000019090	**Cct3**	chaperonin containing Tcp1, subunit 3	295230	2	207072068	207096552	24485	6579,75	0,31	5,32E-01
ENSRNOG00000022268	**Pnpla3**	patatin-like phospholipase domain cor	362972	7	125023373	125043275	19903	367,03	-0,35	5,32E-01
ENSRNOG00000023300		glutamate receptor, ionotropic, delta 1	79219	16	9920690	10248222	327533	838,68	-0,26	5,32E-01
ENSRNOG00000038909	**Pou3f3**	POU class 3 homeobox 3 (Pou3f3), m	192109	9	49151947	49153078	1132	1278,20	0,35	5,32E-01
ENSRNOG00000042245	**Dcaf7**	DDB1 and CUL4 associated factor 7 (	303602	10	93982501	94005038	22538	10241,64	0,33	5,32E-01
ENSRNOG00000043102		BAH domain and coiled-coil containing 1 [Source		10	109041495	109075317	33823	1414,29	0,29	5,32E-01
ENSRNOG00000048036		Neurocan core protein; Protein 10091	58982	16	20900031	20925162	25132	146,51	-0,44	5,32E-01
ENSRNOG00000049219	**Rnf187**	ring finger protein 187 (Rnf187), mRN	360533	10	45030946	45036845	5900	14094,68	0,34	5,32E-01
ENSRNOG00000048567	**Ankmy1**	Protein LOC684015 [Source:UniProtK	684015	9	99703793	99735597	31805	17,56	-0,56	5,32E-01
ENSRNOG00000009069	**Tnfaip1**	tumor necrosis factor, alpha-induced p	287543	10	65837927	65852617	14691	4360,09	0,35	5,32E-01
ENSRNOG00000048205	**Ube2h**	ubiquitin-conjugating enzyme E2H (Ub	296956	4	57272127	57387292	115166	9135,75	0,40	5,32E-01
ENSRNOG00000049965	**LOC10091**	DiGeorge syndrome critical region ger	360742	6	147343891	147394304	50414	463,51	0,23	5,32E-01
ENSRNOG00000012489	**Cacnb3**	calcium channel, voltage-dependent, b	25297	X	114818643	114828180	9538	4366,41	0,33	5,32E-01
ENSRNOG00000013510	**LOC10036**	transmembrane protein 60 (Tmem60),	296761	4	10810282	10815137	4856	361,69	-0,28	5,32E-01
ENSRNOG00000022983	**Trim17**	tripartite motif-containing 17 (Trim17),	64702	10	45063620	45071506	7887	44,04	0,47	5,32E-01
ENSRNOG00000018406	**Wipf1**	WAS/WASL interacting protein family,	117538	3	66633174	66649130	15957	499,48	-0,55	5,32E-01
ENSRNOG00000027693		methyltransferase like 21D [Source:MGI Symbol		6	101330637	101336068	5432	1012,98	0,28	5,32E-01
ENSRNOG00000028415	**Cdc20**	cell division cycle 20 (Cdc20), mRNA [	64515	5	141050192	141054395	4204	873,32	0,45	5,32E-01
ENSRNOG00000042980	**Adam19**	ADAM metallopeptidase domain 19 (A	303068	10	30966982	31059793	92812	1348,89	0,38	5,32E-01
ENSRNOG00000043085	**Clstn2**	calsyntenin 2 (Clstn2), mRNA [Source	171394	8	104768408	105478061	709654	785,93	-0,29	5,32E-01
ENSRNOG00000015321	**Moxd1**	Monooxygenase, DBH-like 1; Protein l	294119	1	23604655	23686657	82003	19,86	0,55	5,32E-01
ENSRNOG00000002803	**Cnot6**	CCR4-NOT transcription complex, sub	287249	10	34918194	34948524	30331	2078,70	-0,23	5,33E-01
ENSRNOG00000006412	**Zhx1**	zinc fingers and homeoboxes 1 (Zhx1)	171159	7	98521908	98550754	28847	1189,28	-0,49	5,33E-01
ENSRNOG00000008082		Regulator of G-protein signaling 6 [Sc	54295	6	114479664	114735956	256293	357,28	-0,41	5,33E-01
ENSRNOG00000011912	**Tmem38a**	transmembrane protein 38a (Tmem38	306327	16	18822745	18837486	14742	604,66	-0,27	5,33E-01
ENSRNOG00000012935	**Ppp1r8**	protein phosphatase 1, regulatory sub	313030	5	154679818	154696564	16747	1314,97	0,30	5,33E-01
ENSRNOG00000013005	**Rpa2**	replication protein A2 (Rpa2), mRNA [	59102	5	154624086	154634563	10478	503,71	0,29	5,33E-01
ENSRNOG00000014060	**Mras**	muscle RAS oncogene homolog (Mras	25482	8	107054728	107082507	27780	3713,25	0,32	5,33E-01
ENSRNOG00000014248	**Erbb4**	v-erb-b2 avian erythroblastic leukemia	59323	9	74804387	75550060	745674	1005,34	-0,39	5,33E-01
ENSRNOG00000014925	**Mesp2**	mesoderm posterior 2 homolog (mous	293046	1	142511695	142514301	2607	29,90	-0,54	5,33E-01
ENSRNOG00000017579	**Mylip**	myosin regulatory light chain interactir	306825	17	21703597	21725205	21609	227,47	-0,37	5,33E-01
ENSRNOG00000020358	**Ptov1**	prostate tumor overexpressed 1 (Ptov	292888	1	101923970	101930515	6546	8012,44	0,32	5,33E-01
ENSRNOG00000020577		phosphatidylinositol-4-phosphate 5-kir	314641	7	11433956	11461658	27703	9085,63	0,32	5,33E-01
ENSRNOG00000023338	**Tspan2**	tetraspanin 2 (Tspan2), mRNA [Source	64521	2	224590998	224633368	42371	1216,05	-0,38	5,33E-01
ENSRNOG00000025145	**Rmdn1**	regulator of microtubule dynamics 1 (F	500419	5	38235426	38258968	23543	167,17	-0,46	5,33E-01
ENSRNOG00000026085	**Rabl3**	RAB, member of RAS oncogene famil	360720	11	69141805	69170839	29035	241,57	0,45	5,33E-01

ENSRNOG00000031440		Neuro-oncological ventral antigen 1, isoform CR		6	76540824	76660498	119675	503,19	-0,28	5,33E-01
ENSRNOG00000032136	**Cdc42ep3**	CDC42 effector protein (Rho GTPase	313838	6	1912346	1932818	20473	769,38	-0,34	5,33E-01
ENSRNOG00000037476	**Galnt9**	UDP-N-acetyl-alpha-D-galactosamine	304571	12	53844763	53922179	77417	530,73	0,31	5,33E-01
ENSRNOG00000037500	**Pus1**	pseudouridylate synthase 1 (Pus1), m	304567	12	53675526	53684279	8754	453,78	0,23	5,33E-01
ENSRNOG00000046848	**PCOLCE2**	procollagen C-endopeptidase enhanc	684050	8	102910334	102934915	24582	17,44	0,54	5,33E-01
ENSRNOG00000050347		Uncharacterized protein [Source:UniProtKB/TrE		10	89133680	89136557	2878	32,70	0,45	5,33E-01
ENSRNOG00000014982	**Slc35f4**	Protein Slc35f4 [Source:UniProtKB/Tr	305865	15	30080358	30372054	291697	59,63	-0,50	5,33E-01
ENSRNOG00000018552	**Slc25a38**	solute carrier family 25, member 38 (S	301067	8	127991043	128003671	12629	707,18	0,30	5,33E-01
ENSRNOG00000019381	**Eri3**	Protein Eri3 [Source:UniProtKB/TrEM	313535	5	140195744	140322806	127063	4095,77	0,26	5,33E-01
ENSRNOG00000036980	**RGD13058**	Protein RGD1305834 [Source:UniPro	310880	2	251227779	251289021	61243	62,62	-0,45	5,33E-01
ENSRNOG00000022896	**Rrp7a**	ribosomal RNA processing 7 homolog	362967	7	123962950	123972838	9889	841,16	0,33	5,33E-01
ENSRNOG00000003889	**Tbc1d15**	Protein Tbc1d15; TBC1 domain family	366896	7	58174019	58228638	54620	432,31	-0,48	5,34E-01
ENSRNOG00000013256	**Ric8a**	resistance to inhibitors of cholinestera	293614	1	220531948	220538535	6588	2682,96	0,31	5,34E-01
ENSRNOG00000019626	**Slc27a5**	solute carrier family 27 (fatty acid tran	79111	1	66387809	66398417	10609	76,93	-0,54	5,34E-01
ENSRNOG00000031934	**Enah**	enabled homolog (Drosophila) (Enah),	360891	13	105280250	105385633	105384	1818,25	-0,31	5,34E-01
ENSRNOG00000009772	**Kirrel3**	kin of IRRE like 3 (Drosophila) (Kirrel3	315546	8	36148052	36273790	125739	1779,55	-0,33	5,34E-01
ENSRNOG00000014595	**Brf1**	BRF1, RNA polymerase III transcriptio	299347	6	146761304	146804485	43182	1080,63	0,25	5,34E-01
ENSRNOG00000022182		RAD50 interactor 1 [Source:MGI Symbol;Acc:M(		4	7860282	7893163	32882	440,09	-0,29	5,34E-01
ENSRNOG00000028411	**Pcnp**	PEST proteolytic signal containing nuc	288165	11	50243284	50250544	7261	1316,99	0,24	5,34E-01
ENSRNOG00000039200		Uncharacterized protein [Source:UniProtKB/TrE		8	44271901	44281224	9324	34,62	-0,42	5,34E-01
ENSRNOG00000004521	**Prpf39**	Protein Prpf39 [Source:UniProtKB/TrE	314171	6	96274790	96299787	24998	563,08	-0,45	5,35E-01
ENSRNOG00000024728	**Arhgap22**	Rho GTPase activating protein 22 (Arl	306279	16	11523404	11572152	48749	30,19	-0,46	5,35E-01
ENSRNOG00000028085		transmembrane protein 255A [Source:	313453	X	124488633	124550919	62287	31,61	-0,47	5,35E-01
ENSRNOG00000033842		Uncharacterized protein [Source:UniProtKB/TrE		1	105303305	105303933	629	18,53	0,51	5,35E-01
ENSRNOG00000000918	**Zbed5**	zinc finger, BED-type containing 5 (Zb	288622	12	32253311	32259362	6052	1118,51	0,34	5,36E-01
ENSRNOG00000015392	**Pole3**	polymerase (DNA directed), epsilon 3,	298098	5	82500187	82503419	3233	1136,23	0,38	5,36E-01
ENSRNOG00000018909	**Arv1**	ARV1 homolog (S. cerevisiae) (Arv1),	292097	19	68189521	68201340	11820	612,63	0,36	5,36E-01
ENSRNOG00000049560	**Glul**	glutamate-ammonia ligase (Glul), mRI	24957	13	76294459	76303626	9168	49426,50	-0,52	5,36E-01
ENSRNOG00000047962	**Taf3**	TAF3 RNA polymerase II, TATA box bi	1E+08	17	74052964	74076697	23734	86,12	0,34	5,36E-01
ENSRNOG00000000977	**Pnpla6**	Protein Pnpla6 [Source:UniProtKB/Trl	360753	12	4232147	4260092	27946	1318,82	0,33	5,36E-01
ENSRNOG00000001518		integrin, alpha 6 (Itga6), mRNA [Sourc	114517	3	64931857	65001002	69146	683,29	-0,37	5,36E-01
ENSRNOG00000002390	**Gnb2l1**	guanine nucleotide binding protein (G	83427	10	33920288	33939018	18731	11871,81	0,40	5,36E-01
ENSRNOG00000011853	**Mbd2**	methyl-CpG binding domain protein 2	680172	18	64990486	65060940	70455	741,37	0,26	5,36E-01
ENSRNOG00000021102	**Scn1b**	sodium channel, voltage-gated, type I,	29686	1	90705285	90715018	9734	402,46	0,33	5,36E-01
ENSRNOG00000021614		lysine (K)-specific methyltransferase 2E [Source		4	8218002	8261227	43226	938,75	-0,22	5,36E-01
ENSRNOG00000018630	**LOC68518(**	glyceraldehyde-3-phosphate dehydrog	24383	4	224693580	224697455	3876	651,67	0,25	5,37E-01
ENSRNOG00000007483	**Ccnf**	cyclin F (Ccnf), mRNA [Source:RefSe(	117524	10	13410718	13435965	25248	535,69	0,43	5,37E-01
ENSRNOG00000036576	**Zdhhc6**	zinc finger, DHHC-type containing 6 (Z	361771	1	283686772	283703450	16679	543,28	-0,32	5,37E-01

ENSRNOG00000009028	**Rnf126**	ring finger protein 126 (Rnf126), mRN	314613	7	12991016	12998304	7289	1102,25	0,36	5,37E-01
ENSRNOG00000007254	**Ttc9**	tetratricopeptide repeat domain 9 (Ttc	500689	6	113510909	113545657	34749	1445,90	0,33	5,38E-01
ENSRNOG00000006261	**Papolg**	poly(A) polymerase gamma (Papolg),	305586	14	108346030	108380042	34013	450,04	-0,32	5,38E-01
ENSRNOG00000046566	**Tub**	tubby homolog (mouse) (Tub), mRNA	25609	1	180597530	180615868	18339	1194,70	-0,29	5,38E-01
ENSRNOG00000007510	**Bbs12**	Protein Bbs12 [Source:UniProtKB/TrE	365770	2	143662813	143664933	2121	48,13	-0,41	5,38E-01
ENSRNOG00000021221	**Pced1a**	PC-esterase domain containing 1A (P	296158	3	129442747	129446650	3904	436,98	-0,28	5,38E-01
ENSRNOG00000033641		cGMP-dependent protein kinase 1 [S	54286	1	256221067	256702284	481218	17,41	-0,54	5,38E-01
ENSRNOG00000004050	**Snrk**	SNF related kinase (Snrk), mRNA [So	170837	8	129932817	129971319	38503	1610,54	0,33	5,38E-01
ENSRNOG00000010736	**Pbx4**	pre-B-cell leukemia homeobox 4 (Pbx	361131	16	21219921	21253490	33570	146,45	0,34	5,38E-01
ENSRNOG00000016156	**Nptxr**	neuronal pentraxin receptor (Nptxr), m	81005	7	121002639	121020713	18075	13392,92	-0,28	5,38E-01
ENSRNOG00000020510	**Sugp1**	SURP and G patch domain containing	290666	16	21016442	21047083	30642	569,15	0,21	5,38E-01
ENSRNOG00000025764	**Mt1a**	metallothionein 1a (Mt1a), mRNA [Sou	24567	19	11277133	11278149	1017	155,28	0,56	5,38E-01
ENSRNOG00000028161	**Enthd2**	ENTH domain containing 2 (Enthd2),	360673	10	108869255	108876525	7271	637,52	0,23	5,38E-01
ENSRNOG00000030877	**Htr2c**	5-hydroxytryptamine (serotonin) recep	25187	X	118339418	118460829	121412	86,37	-0,49	5,38E-01
ENSRNOG00000031849	**RGD13101**	similar to 3632451O06Rik protein (RG	361032	15	30512209	30604270	92062	647,08	0,37	5,38E-01
ENSRNOG00000042867		RIKEN cDNA 4932443I19 gene [Source:MGI Sy		16	80951548	80969299	17752	43,18	-0,56	5,38E-01
ENSRNOG00000006894	**Tor1a**	torsin family 1, member A (Tor1a), mR	266606	3	15158995	15165990	6996	1297,16	0,23	5,38E-01
ENSRNOG00000016353	**Nim1**	Protein Nim1 [Source:UniProtKB/TrE	310376	2	70812982	70828841	15860	516,75	-0,32	5,38E-01
ENSRNOG00000007990	**Adipor2**	adiponectin receptor 2 (Adipor2), mR	312670	4	217328527	217363832	35306	2203,51	0,35	5,38E-01
ENSRNOG00000017511	**Dcdc2**	doublecortin domain containing 2 (Dc	291130	17	43711172	43899062	187891	32,68	-0,51	5,38E-01
ENSRNOG00000018804	**Fam65b**	family with sequence similarity 65, me	306934	17	44192188	44289647	97460	1256,25	-0,35	5,38E-01
ENSRNOG00000024702	**Fam217b**	family with sequence similarity 217, m	311692	3	183130338	183135513	5176	497,93	0,30	5,38E-01
ENSRNOG00000048784				7	2088703	2093233	4531	46,58	-0,45	5,38E-01
ENSRNOG00000006756	**Maged1**	melanoma antigen, family D, 1 (Mage	84469	X	64712958	64719604	6647	19370,64	0,23	5,38E-01
ENSRNOG00000016623	**Tmeff2**	transmembrane protein with EGF-like	363228	9	55079810	55369414	289605	1163,31	-0,28	5,38E-01
ENSRNOG00000018729	**Rad9a**	Protein Rad9a; RCG47324, isoform C	1E+08	1	226314743	226321102	6360	287,95	0,28	5,38E-01
ENSRNOG00000005107	**Recql5**	RecQ protein-like 5 (Recql5), mRNA [	287834	10	103802523	103841703	39181	412,92	0,36	5,38E-01
ENSRNOG00000014493	**Golga1**	golgin A1 (Golga1), mRNA [Source:Re	311919	3	28531144	28575251	44108	399,61	-0,37	5,38E-01
ENSRNOG00000015733	**Myl12b**	myosin, light chain 12B, regulatory (M	50685	9	118761460	118775834	14375	4747,59	0,33	5,38E-01
ENSRNOG00000028137		antigen identified by monoclonal antibody Ki 67		1	214925928	214952235	26308	961,11	0,54	5,38E-01
ENSRNOG00000020062	**Pcdhb13**	Protein Pcdhb13; RCG49357 [Source	307489	18	30223157	30225547	2391	132,93	-0,35	5,39E-01
ENSRNOG00000026036	**Pdyn**	prodynorphin (Pdyn), mRNA [Source:F	29190	3	127809952	127822294	12343	40,79	0,51	5,39E-01
ENSRNOG00000005331	**Vapb**	VAMP (vesicle-associated membrane	60431	3	177890671	177927965	37295	3767,60	0,24	5,40E-01
ENSRNOG00000008154	**Ramp3**	receptor (G protein-coupled) activity m	56820	14	80555463	80572980	17518	41,39	-0,45	5,40E-01
ENSRNOG00000020415	**Ramp2**	receptor (G protein-coupled) activity m	58966	10	88965654	88967459	1806	296,67	0,28	5,40E-01
ENSRNOG00000020791	**Ube2q1**	ubiquitin-conjugating enzyme E2Q fan	295252	2	208528890	208537959	9070	2359,11	0,29	5,40E-01
ENSRNOG00000037264	**Hcfc1**	host cell factor C1 (Hcfc1), mRNA [So	363519	1	152560125	152585295	25171	2392,65	0,21	5,40E-01
ENSRNOG00000005963	**Rab2a**	RAB2A, member RAS oncogene famil	65158	5	26385975	26448949	62975	3911,00	0,29	5,40E-01

ENSRNOG00000001484	**Gatsl2**	GATS protein-like 2 (Gatsl2), mRNA [S	304410	12	27605972	27646215	40244	2141,63	0,33	5,40E-01
ENSRNOG00000019045		Uncharacterized protein [Source:UniProtKB/TrE		1	206257268	206270414	13147	1022,43	0,20	5,40E-01
ENSRNOG00000037661	**Tmsbl1**	thymosin beta-like protein 1 (Tmsbl1),	286978	X	107491614	107493728	2115	2972,82	-0,28	5,40E-01
ENSRNOG00000009713	**Oxa1l**	oxidase (cytochrome c) assembly 1-lik	691393	15	36884734	36893489	8756	2794,01	0,22	5,40E-01
ENSRNOG00000026753	**Mpzl3**	myelin protein zero-like 3 (Mpzl3), mR	363054	8	47981273	48001460	20188	27,16	0,47	5,40E-01
ENSRNOG00000008771	**Nlrx1**	NLR family member X1 (Nlrx1), mRNA	315599	8	47202129	47218106	15978	321,62	-0,41	5,40E-01
ENSRNOG00000027511				10	73799139	73799762	624	110,48	0,34	5,40E-01
ENSRNOG00000020024	**Taf7**	TAF7 RNA polymerase II, TATA box bi	307485	18	30475376	30477519	2144	384,12	-0,36	5,41E-01
ENSRNOG00000021379	**Txnl4b**	thioredoxin-like 4B (Txnl4b), mRNA [S	292008	19	52927031	52932730	5700	558,45	0,40	5,41E-01
ENSRNOG00000000417	**Numa1**	nuclear mitotic apparatus protein 1 (Nu	308870	1	173249830	173279637	29808	4351,80	0,24	5,41E-01
ENSRNOG00000019348	**Zc3h7b**	zinc finger CCCH-type containing 7B (	315158	7	122976510	123003695	27186	14603,82	0,23	5,41E-01
ENSRNOG00000018369		periaxin (Prx), mRNA [Source:RefSeq	78960	1	85526345	85541381	15037	64,38	-0,48	5,41E-01
ENSRNOG00000021492	**Rbbp4**	retinoblastoma binding protein 4 (Rbb	313048	5	151232122	151256539	24418	589,33	0,24	5,41E-01
ENSRNOG00000016912	**Btf3**	basic transcription factor 3 (Btf3), mRN	294680	2	47518639	47525723	7085	1050,36	0,36	5,41E-01
ENSRNOG00000049111	**Dchs2**	Protein Dchs2 [Source:UniProtKB/TrE	310550	2	201705946	201732964	27019	171,45	-0,43	5,41E-01
ENSRNOG00000000839	**Nfkbil1**	nuclear factor of kappa light polypeptic	361794	20	6904626	6919601	14976	583,97	0,24	5,41E-01
ENSRNOG00000004255	**Usf1**	upstream transcription factor 1 (Usf1),	83586	13	94424975	94433095	8121	2856,34	0,24	5,41E-01
ENSRNOG00000005565	**Traf3ip3**	TRAF3 interacting protein 3 (Traf3ip3)	360900	13	116447651	116472948	25298	25,17	-0,55	5,41E-01
ENSRNOG00000008103	**Mdh1**	malate dehydrogenase 1, NAD (solubl	24551	14	106449340	106463995	14656	9172,53	0,31	5,41E-01
ENSRNOG00000008676	**Emp1**	epithelial membrane protein 1 (Emp1)	25314	4	233415324	233449254	33931	129,90	-0,46	5,41E-01
ENSRNOG00000010997	**Ednrb**	endothelin receptor type B (Ednrb), ml	50672	15	91502602	91531979	29378	3698,88	-0,45	5,41E-01
ENSRNOG00000011454	**Tbck**	TBC1 domain containing kinase (Tbck	295446	2	256292173	256497625	205453	706,26	-0,23	5,41E-01
ENSRNOG00000011887	**RGD13079**	Protein RGD1307929 [Source:UniPro	303280	10	66084632	66113961	29330	6710,81	0,22	5,41E-01
ENSRNOG00000014034	**Olfml2a**	olfactomedin-like 2A (Olfml2a), mRNA	296708	3	28455740	28481133	25394	284,14	-0,51	5,41E-01
ENSRNOG00000014834	**Epc1**	Protein Epc1 [Source:UniProtKB/TrEI	1E+08	17	55333276	55360933	27658	1082,19	-0,36	5,41E-01
ENSRNOG00000017952	**Sept2**	septin 2 (Sept2), mRNA [Source:RefS	117515	9	100277909	100311079	33171	4738,93	-0,35	5,41E-01
ENSRNOG00000018459	**Xylt1**	Xylosyltransferase 1 [Source:UniProtI	64133	1	193935673	194215173	279501	1372,17	-0,34	5,41E-01
ENSRNOG00000019598	**Vegfa**	vascular endothelial growth factor A (V	83785	9	16232415	16247655	15241	1533,96	-0,35	5,41E-01
ENSRNOG00000020060	**Atf5**	activating transcription factor 5 (Atf5),	282840	1	101872584	101876673	4090	5976,39	-0,50	5,41E-01
ENSRNOG00000020349	**Rab3il1**	RAB3A interacting protein (rabin3)-like	171452	1	233022972	233030682	7711	56,37	-0,48	5,41E-01
ENSRNOG00000021004	**Rasip1**	Ras interacting protein 1 (Rasip1), mR	292912	1	102689553	102700426	10874	666,70	0,30	5,41E-01
ENSRNOG00000026455	**Gpd1l**	glycerol-3-phosphate dehydrogenase	363159	8	122268134	122300375	32242	3698,49	0,32	5,41E-01
ENSRNOG00000033883	**Stard8**	StAR-related lipid transfer (START) dc	312113	X	69645576	69659522	13947	130,74	-0,49	5,41E-01
ENSRNOG00000018980	**Tjap1**	tight junction associated protein 1 (Tja	316233	9	15983152	16007447	24296	742,62	0,24	5,41E-01
ENSRNOG00000021916	**Slc16a12**	solute carrier family 16, member 12 (S	309525	1	260199459	260222109	22651	25,19	-0,53	5,42E-01
ENSRNOG00000002372		sarcoglycan, delta (dystrophin-associated glycop		10	31878904	32255864	376961	29,05	-0,55	5,42E-01
ENSRNOG00000016446	**Gpr45**	G protein-coupled receptor 45 (Gpr45)	301372	9	49508442	49511911	3470	267,50	0,36	5,42E-01
ENSRNOG00000033932				MT	5010	5078	69	1525,75	-0,43	5,42E-01

ENSRNOG00000004941	**Gpn1**	GPN-loop GTPase 1 (Gpn1), mRNA [S	688393	6	36078633	36096305	17673	1232,02	0,24	5,42E-01
ENSRNOG00000011905	**Atp6v1e1**	ATPase, H+ transporting, lysosomal V	297566	4	220441645	220463709	22065	6205,31	0,28	5,42E-01
ENSRNOG00000018716	**Dennd2c**	DENN/MADD domain containing 2C (I	295333	2	225047710	225090476	42767	69,82	0,45	5,42E-01
ENSRNOG00000020573	**Efna1**	ephrin A1 (Efna1), mRNA [Source:Ref	94268	2	208002159	208009501	7343	31,88	0,43	5,42E-01
ENSRNOG00000037267	**Renbp**	renin binding protein (Renbp), mRNA [	81759	1	152602558	152611616	9059	249,67	-0,55	5,42E-01
ENSRNOG00000016204	**Lrrc24**	leucine rich repeat containing 24 (Lrrc	362945	7	117767712	117774850	7139	411,39	0,35	5,42E-01
ENSRNOG00000019492	**Pomgnt2**	glycosyltransferase-like domain contai	316091	8	129791153	129792895	1743	774,19	0,30	5,42E-01
ENSRNOG00000047873		SEC22 vesicle trafficking protein homolog C (S.		8	129507930	129520041	12112	391,09	0,36	5,42E-01
ENSRNOG00000020356	**Zfp846**	zinc finger protein 846 (Zfp846), mRN	363022	8	21667380	21674450	7071	557,56	-0,26	5,43E-01
ENSRNOG00000027711	**Usp32**	ubiquitin specific peptidase 32 (Usp32	303394	10	72206629	72323744	117116	1870,04	-0,31	5,43E-01
ENSRNOG00000042276	**Trappc2**	trafficking protein particle complex 2 (T	501550	X	29944166	29949901	5736	1098,02	-0,33	5,43E-01
ENSRNOG00000014424		Protein RGD1563354 [Source:UniPro	311592	3	160898555	160933737	35183	48,34	-0,52	5,43E-01
ENSRNOG00000005883	**Nek10**	Protein Nek10 [Source:UniProtKB/TrE	305710	15	15671472	15851264	179793	34,75	-0,43	5,43E-01
ENSRNOG00000006947	**Pdhx**	pyruvate dehydrogenase complex, co	311254	3	99552950	99576700	23751	1142,33	0,36	5,43E-01
ENSRNOG00000010609	**Abcf2**	ATP-binding cassette, subfamily F (GC	311959	4	7135433	7148121	12689	1240,48	0,30	5,43E-01
ENSRNOG00000018839	**Ntrk2**	neurotrophic tyrosine kinase, receptor,	25054	17	8156432	8464507	308076	9262,80	-0,40	5,43E-01
ENSRNOG00000022748	**Tmem126a**	transmembrane protein 126A (Tmem1	293113	1	162529250	162537179	7930	420,77	-0,29	5,43E-01
ENSRNOG00000040257	**Chmp2b**	Protein Chmp2b; Similar to CGI-84 pro	363720	11	2650917	2676654	25738	1277,52	0,28	5,43E-01
ENSRNOG00000006749	**Tmtc3**	transmembrane and tetratricopeptide r	314785	7	40209728	40256099	46372	334,68	-0,40	5,43E-01
ENSRNOG00000019907	**Nfkbie**	nuclear factor of kappa light polypeptic	316241	9	16717129	16723961	6833	342,16	0,25	5,43E-01
ENSRNOG00000002273	**Naaa**	N-acylethanolamine acid amidase (Na	497009	14	17199739	17218990	19252	36,95	-0,46	5,43E-01
ENSRNOG00000042042		SCO cytochrome oxidase deficient homolog 2 (y		7	130027705	130028478	774	1165,19	0,21	5,43E-01
ENSRNOG00000010176	**Map2k1**	mitogen activated protein kinase kinas	170851	8	68841356	69431569	590214	1723,47	0,27	5,44E-01
ENSRNOG00000005596	**Gdpd1**	glycerophosphodiester phosphodieste	303407	10	75724331	75767811	43481	2042,81	0,44	5,44E-01
ENSRNOG00000002040		BMP2 inducible kinase [Source:MGI Symbol;Acc		14	14073451	14135927	62477	556,74	-0,25	5,44E-01
ENSRNOG00000009271	**Smarca4**	SWI/SNF related, matrix associated, a	171379	8	22702277	22793519	91243	8227,26	0,23	5,44E-01
ENSRNOG00000010116	**Leo1**	Leo1, Paf1/RNA polymerase II comple	300837	8	81983992	82008425	24434	864,91	0,34	5,44E-01
ENSRNOG00000049326	**Tmem234**	Protein LOC682404; RCG30879, isofo	682404	5	151532921	151541216	8296	485,67	0,31	5,44E-01
ENSRNOG00000025428	**Rp2**	retinitis pigmentosa 2 homolog (huma	367714	X	2866100	2908347	42248	70,16	-0,48	5,44E-01
ENSRNOG00000032439	**Rsl1d1**	ribosomal L1 domain containing 1 (Rs	302898	10	3214338	3256101	41764	37,24	-0,43	5,44E-01
ENSRNOG00000001786	**Snx4**	sorting nexin 4 (Snx4), mRNA [Source	360725	11	73779997	73836594	56598	2197,95	0,27	5,44E-01
ENSRNOG00000006539	**Pex16**	peroxisomal biogenesis factor 16 (Pex	311203	3	87986605	87996037	9433	773,92	0,32	5,45E-01
ENSRNOG00000031709	**Ppfibp1**	PTPRF interacting protein, binding pro	312855	4	245465652	245578035	112384	828,77	-0,52	5,45E-01
ENSRNOG00000003463		sterol regulatory element binding trans	78968	10	46326907	46347948	21042	5779,67	-0,51	5,45E-01
ENSRNOG00000001378		IQ domain-containing protein D [Source:UniPro		12	43348902	43360033	11132	138,45	-0,40	5,46E-01
ENSRNOG00000007829	**Lactb2**	lactamase, beta 2 (Lactb2), mRNA [Sc	297768	5	4950859	4973620	22762	311,33	-0,30	5,46E-01
ENSRNOG00000048981	**Ahsa1**	AHA1, activator of heat shock 90kDa p	681996	6	120578428	120586235	7808	3039,67	0,36	5,46E-01
ENSRNOG00000006325	**Rps21**	ribosomal protein S21 (Rps21), mRNA	81775	3	181484785	181485814	1030	239,22	0,41	5,46E-01

ENSRNOG00000027320	**Ago1**	eukaryotic translation initiation factor 2	313594	5	148214328	148246407	32080	2237,01	-0,21	5,46E-01
ENSRNOG00000012363	**Peli2**	pellino E3 ubiquitin protein ligase fami	305835	15	28874964	29015649	140686	715,64	-0,35	5,46E-01
ENSRNOG00000016091	**Tmem169**	transmembrane protein 169 (Tmem16	690294	9	79403626	79427523	23898	615,15	-0,30	5,46E-01
ENSRNOG00000023786		Uncharacterized protein; Ybx1 protein	[Source:l	5	142136441	142152428	15988	672,13	-0,36	5,46E-01
ENSRNOG00000003302	**Flcn**	folliculin (Flcn), mRNA [Source:RefSe	303185	10	45909612	45928697	19086	1831,41	0,25	5,46E-01
ENSRNOG00000004438	**Cntn1**	contactin 1 (Cntn1), mRNA [Source:Re	117258	7	133085052	133270392	185341	4315,17	-0,48	5,46E-01
ENSRNOG00000005215	**Klhl4**	kelch-like family member 4 (Klhl4), mF	317196	X	86067141	86171225	104085	94,60	0,38	5,46E-01
ENSRNOG00000017737	**Dgkz**	diacylglycerol kinase zeta (Dgkz), mRl	81821	3	87542673	87572556	29884	2972,24	-0,37	5,46E-01
ENSRNOG00000018574	**Qsox2**	quiescin Q6 sulfhydryl oxidase 2 (Qso	681023	3	9024117	9053765	29649	883,78	0,32	5,46E-01
ENSRNOG00000018867	**Klhdc7a**	Protein Klhdc7a; RCG31274 [Source:	298590	5	162164870	162167191	2322	20,25	-0,51	5,46E-01
ENSRNOG00000021644	**Slc15a3**	solute carrier family 15 (oligopeptide tı	246239	1	233963402	233978192	14791	146,68	-0,32	5,46E-01
ENSRNOG00000027039	**Mrpl22**	mitochondrial ribosomal protein L22 (M	287302	10	43395542	43405875	10334	830,24	0,37	5,46E-01
ENSRNOG00000034022	**Taf2**	TAF2 RNA polymerase II, TATA box bi	170844	7	95339750	95396754	57005	1107,99	-0,32	5,46E-01
ENSRNOG00000004772	**Cytip**	cytohesin 1 interacting protein (Cytip),	311047	3	49270139	49296836	26698	27,96	0,52	5,46E-01
ENSRNOG00000036697	**Mafg**	v-maf avian musculoaponeurotic fibros	64188	10	109399211	109404375	5165	1046,23	0,25	5,47E-01
ENSRNOG00000022701		Protein Ceacam18-ps1 [Source:UniPı	502334	1	100630244	100644423	14180	23,77	0,54	5,47E-01
ENSRNOG00000026171	**Bbs4**	Bardet-Biedl syndrome 4 (Bbs4), mRN	300754	8	63883342	63916346	33005	713,61	-0,24	5,47E-01
ENSRNOG00000017250	**Gmpr**	guanosine monophosphate reductase	117533	17	21568732	21606386	37655	538,01	0,25	5,47E-01
ENSRNOG00000037428		Protein Zfp605 [Source:UniProtKB/TrEMBL;Acc		12	54344422	54385822	41401	34,33	-0,41	5,48E-01
ENSRNOG00000020821	**LOC10091**	CAP-GLY domain containing linker prc	308493	1	89906480	89921238	14759	187,20	0,31	5,48E-01
ENSRNOG00000002524	**Gpr37**	G protein-coupled receptor 37 (Gpr37	117549	4	51598203	51620342	22140	255,50	-0,38	5,48E-01
ENSRNOG00000050292		protocadherin alpha-7 precursor [Sou	393089	18	29690611	29692965	2355	165,73	-0,32	5,48E-01
ENSRNOG00000003253	**Qdpr**	quinoid dihydropteridine reductase (Q	64192	14	70207853	70221468	13616	3378,55	0,31	5,48E-01
ENSRNOG00000010947		matrix metallopeptidase 14 (membran	81707	15	36960273	36969507	9235	2698,31	-0,45	5,48E-01
ENSRNOG00000021434	**Usp51**	ubiquitin specific peptidase 51 (Usp51	317398	X	20035912	20038959	3048	33,99	-0,49	5,49E-01
ENSRNOG00000019132	**Fv1**	Friend virus susceptibility 1 (Fv1), mRl	308568	1	101289512	101291836	2325	4690,50	0,26	5,49E-01
ENSRNOG00000020843		Ferritin light chain 1 [Source:UniProtK	29292	1	102527141	102528775	1635	2004,11	0,36	5,49E-01
ENSRNOG00000048050		transmembrane protein 120B [Source:	690137	12	40794196	40814134	19939	186,95	0,32	5,49E-01
ENSRNOG00000002784	**Pou3f4**	POU class 3 homeobox 4 (Pou3f4), m	29589	X	82301606	82302855	1250	313,95	0,34	5,49E-01
ENSRNOG00000005697	**Slc6a11**	solute carrier family 6 (neurotransmitte	79213	4	209399630	209516970	117341	3479,18	-0,40	5,49E-01
ENSRNOG00000014828	**Avpi1**	arginine vasopressin-induced 1 (Avpi1	171386	1	268818253	268823980	5728	321,58	0,34	5,49E-01
ENSRNOG00000017993	**Abcb10**	ATP-binding cassette, subfamily B (MI	361439	19	67458448	67488456	30009	1073,94	0,32	5,49E-01
ENSRNOG00000020411	**Sec23ip**	SEC23 interacting protein (Sec23ip), r	309010	1	207214643	207257112	42470	2024,79	0,22	5,49E-01
ENSRNOG00000006832	**Zdhhc5**	zinc finger, DHHC-type containing 5 (Z	362156	3	78611962	78634327	22366	1245,89	0,33	5,49E-01
ENSRNOG00000012495	**Podxl**	podocalyxin-like (Podxl), mRNA [Sour	192181	4	58581515	58628079	46565	1176,24	0,31	5,49E-01
ENSRNOG00000016444				9	49466038	49466499	462	67,82	0,43	5,49E-01
ENSRNOG00000018164	**Spcs2**	signal peptidase complex subunit 2 hc	293142	1	171032500	171052353	19854	3997,69	0,33	5,49E-01
ENSRNOG00000050762	**Ttc30b**	tetratricopeptide repeat domain 30B (T	499814	3	69306229	69308223	1995	221,03	-0,34	5,49E-01

ENSRNOG00000016119	**Fzd7**	rCG22430-like (LOC100360552), mRI	1E+08	9	66112304	66114022	1719	112,62	0,38	5,50E-01
ENSRNOG00000007514	**Sox12**	SRY (sex determining region Y)-box 1	689988	3	154212620	154213564	945	970,30	0,37	5,50E-01
ENSRNOG00000015465	**Cep72**	Protein Cep72 [Source:UniProtKB/TrE	308064	1	33259590	33288992	29403	113,04	0,40	5,50E-01
ENSRNOG00000019356	**Lrfn4**	leucine rich repeat and fibronectin type	688721	1	226716517	226719809	3293	3078,65	0,36	5,50E-01
ENSRNOG00000027245	**Tdrp**	testis development related protein (Td	498662	16	80296809	80322591	25783	325,65	0,32	5,50E-01
ENSRNOG00000046635	**LOC10091**	cell division cycle associated 5 (Cdca5	684771	1	228384097	228394999	10903	48,36	0,41	5,50E-01
ENSRNOG00000049982	**Pex26**	peroxisomal biogenesis factor 26 (Pex	297570	4	220837560	220849661	12102	1053,04	0,34	5,50E-01
ENSRNOG00000010152	**RGD13062**	uncharacterized protein LOC296608 |	296608	3	12398276	12402626	4351	105,28	-0,50	5,51E-01
ENSRNOG00000004320	**Tgm4**	transglutaminase 4 (prostate) (Tgm4),	64679	8	131275399	131311091	35693	86,34	-0,41	5,51E-01
ENSRNOG00000007434		ubiquitin-conjugating enzyme E2J 1 [Source:MG		5	52858667	52874903	16237	1100,13	0,39	5,51E-01
ENSRNOG00000010608	**RGD13073**	Centrosomal protein of 162 kDa [Sou	300880	8	94370712	94427870	57159	146,07	0,37	5,51E-01
ENSRNOG00000013692		RIKEN cDNA 3110057O12 gene [Sou	499602	2	147374186	147429666	55481	188,01	-0,40	5,51E-01
ENSRNOG00000017349	**Tbc1d10b**	TBC1 domain family, member 10b (Tb	365372	1	205634749	205646405	11657	2511,65	0,23	5,51E-01
ENSRNOG00000027582	**Dpysl4**	dihydropyrimidinase-like 4 (Dpysl4), m	25417	1	218349771	218365280	15510	3299,48	0,23	5,51E-01
ENSRNOG00000028255	**Usp36**	ubiquitin specific peptidase 36 (Usp36	303700	10	106927771	106959353	31583	1104,18	0,28	5,51E-01
ENSRNOG00000000412	**Slc35f1**	solute carrier family 35, member F1 (S	502421	20	36017841	36174319	156479	3898,33	-0,29	5,51E-01
ENSRNOG00000001420		Mitochondrial fission 1 protein [Sourc	288584	12	24760056	24774734	14679	3840,23	0,39	5,51E-01
ENSRNOG00000013766	**Acaa2**	acetyl-CoA acyltransferase 2 (Acaa2),	170465	18	69873084	69901836	28753	2096,77	-0,39	5,51E-01
ENSRNOG00000017478	**Pcsk7**	proprotein convertase subtilisin/kexin t	29606	8	48824388	48847099	22712	781,04	0,34	5,51E-01
ENSRNOG00000008543	**Pdlim2**	PDZ and LIM domain 2 (Pdlim2), mRN	290354	15	55569718	55581479	11762	103,77	-0,54	5,51E-01
ENSRNOG00000018567	**Slc20a1**	solute carrier family 20 (phosphate tra	81826	3	128276543	128289618	13076	1607,23	-0,30	5,51E-01
ENSRNOG00000024497		U2 small nuclear RNA auxiliary factor	361542	1	90171104	90174276	3173	798,50	0,22	5,51E-01
ENSRNOG00000025115	**Exph5**	Protein Exph5 [Source:UniProtKB/TrE	315663	8	56463112	56541322	78211	61,91	-0,34	5,51E-01
ENSRNOG00000042032				5	175899997	175900661	665	21,53	-0,49	5,51E-01
ENSRNOG00000018338	**Vwa1**	von Willebrand factor A domain contai	298683	5	176692353	176697535	5183	334,90	0,32	5,52E-01
ENSRNOG00000019186	**Tmem53**	transmembrane protein 53 (Tmem53),	313529	5	139905905	139921464	15560	320,43	0,33	5,53E-01
ENSRNOG00000021027	**Dbp**	D site of albumin promoter (albumin D	24309	1	102766762	102771674	4913	733,13	-0,46	5,53E-01
ENSRNOG00000039488		Uncharacterized protein [Source:UniProtKB/TrE		12	17092501	17116350	23850	20,68	-0,49	5,53E-01
ENSRNOG00000001258	**Snx8**	sorting nexin 8 (Snx8), mRNA [Source	288504	12	18334035	18382248	48214	1071,91	0,28	5,53E-01
ENSRNOG00000004364	**Bre**	brain and reproductive organ-express	362704	6	35490054	35875542	385489	1174,37	0,34	5,53E-01
ENSRNOG00000005931	**Cpq**	carboxypeptidase Q (Cpq), mRNA [So	58952	7	71880824	72338526	457703	30,22	-0,54	5,53E-01
ENSRNOG00000006264	**Itfg2**	integrin alpha FG-GAP repeat containi	362441	4	226936182	226949437	13256	228,48	-0,26	5,53E-01
ENSRNOG00000009312	**LOC10091**	similar to 1500031N24Rik protein (RG	315088	7	117012150	117018916	6767	120,42	0,38	5,53E-01
ENSRNOG00000010199	**Glrb**	glycine receptor, beta (Glrb), mRNA [S	25456	2	199178597	199251847	73251	1445,89	-0,32	5,53E-01
ENSRNOG00000010822	**Dlx6**	Protein Dlx6 [Source:UniProtKB/TrEMBL;Acc:D		4	32242555	32244664	2110	25,95	-0,46	5,53E-01
ENSRNOG00000017078	**Sepn1**	Protein Sepn1 [Source:UniProtKB/TrE	362624	5	156535882	156547763	11882	1396,85	0,28	5,53E-01
ENSRNOG00000019623		zinc finger protein 446 [Source:MGI Symbol;Acc		1	66408336	66412079	3744	265,14	0,36	5,53E-01
ENSRNOG00000023172	**Dnajb3**	DnaJ (Hsp40) homolog, subfamily B, r	680216	9	94977349	94978074	726	69,23	0,43	5,53E-01

ENSRNOG00000026116	**Sall3**	sal-like 3 (Drosophila) (Sall3), mRNA [	364910	18	76676425	76682692	6268	1152,57	0,19	5,53E-01
ENSRNOG00000026466	**Lrrtm3**	leucine rich repeat transmembrane ne	294380	20	28060837	28233801	172965	420,23	-0,28	5,53E-01
ENSRNOG00000042421	**RGD15599**	RGD1559909 (RGD1559909), mRNA	362592	5	146614723	146615760	1038	2427,62	0,46	5,53E-01
ENSRNOG00000000274		phytanoyl-CoA 2-hydroxylase interacti	309901	20	21467184	21507653	40470	3131,19	-0,34	5,53E-01
ENSRNOG00000014162	**Plekhb2**	pleckstrin homology domain containin	301337	9	41017135	41049780	32646	9622,56	0,33	5,53E-01
ENSRNOG00000036676		casein kinase 1, delta (Csnk1d), mRN	64462	10	109736328	109769940	33613	6035,06	0,22	5,53E-01
ENSRNOG00000004479	**Aurka**	aurora kinase A (Aurka), mRNA [Sourc	261730	3	176439963	176453986	14024	372,50	0,42	5,53E-01
ENSRNOG00000001479		general transcription factor II I (Gtf2i),	353256	12	27420682	27497925	77244	8827,75	0,18	5,53E-01
ENSRNOG00000008507	**Cradd**	CASP2 and RIPK1 domain containing	314756	7	36457911	36470834	12924	56,19	-0,48	5,53E-01
ENSRNOG00000005730	**Pcmtd1**	protein-L-isoaspartate (D-aspartate) C	366300	5	16914995	16951164	36170	363,48	-0,29	5,54E-01
ENSRNOG00000010345	**Ssmem1**	serine-rich single-pass membrane pro	500068	4	57585320	57595615	10296	17,93	-0,51	5,54E-01
ENSRNOG00000010308	**Nr2f2**	nuclear receptor subfamily 2, group F,	113984	1	132487763	132493715	5953	651,12	-0,26	5,54E-01
ENSRNOG00000016866	**Fhl2**	four and a half LIM domains 2 (Fhl2),	63839	9	49591627	49620648	29022	235,20	-0,30	5,54E-01
ENSRNOG00000018827	**Htr7**	5-hydroxytryptamine (serotonin) recep	65032	1	261758785	261879737	120953	231,53	0,51	5,54E-01
ENSRNOG00000002556	**Tsr2**	TSR2, 20S rRNA accumulation, homo	317418	X	20968909	20977426	8518	271,32	0,44	5,55E-01
ENSRNOG00000005865	**Ssfa2**	sperm specific antigen 2 (Ssfa2), mRN	311146	3	73152918	73190192	37275	940,56	-0,41	5,55E-01
ENSRNOG00000013777	**Rnf166**	ring finger protein 166 (Rnf166), mRN	365022	19	65999963	66009803	9841	691,27	0,27	5,55E-01
ENSRNOG00000015189	**Bbs9**	Protein Bbs9 [Source:UniProtKB/TrEM	315484	8	23813100	24113782	300683	655,27	0,39	5,55E-01
ENSRNOG00000032776	**Srp54**	signal recognition particle 54A (Srp54)	116650	6	85532345	85572105	39761	829,54	-0,44	5,55E-01
ENSRNOG00000013773	**Secisbp2**	SECIS binding protein 2 (Secisbp2), m	79049	17	15630050	15661255	31206	472,91	0,29	5,55E-01
ENSRNOG00000021943	**RGD15601**	Protein RGD1560137 [Source:UniPro	290372	15	63952657	63962575	9919	17,88	-0,53	5,55E-01
ENSRNOG00000046139	**Syt14**	Protein LOC684293 [Source:UniProtK	684293	13	116187700	116311954	124255	201,84	-0,31	5,55E-01
ENSRNOG00000046996	**Pea15**	phosphoprotein enriched in astrocytes	364052	13	95100951	95110571	9621	28252,60	-0,32	5,56E-01
ENSRNOG00000008287	**Nle1**	notchless homolog 1 (Drosophila) (Nle	303372	10	69882124	69890695	8572	409,88	0,29	5,56E-01
ENSRNOG00000016380	**Necab3**	N-terminal EF-hand calcium binding p	311562	3	156420461	156434946	14486	124,70	-0,35	5,56E-01
ENSRNOG00000006726	**Zfp9**	zinc finger protein 9 (Zfp9), mRNA [So	1E+08	4	216439332	216446212	6881	397,29	-0,26	5,57E-01
ENSRNOG00000042587		Uncharacterized protein [Source:UniProtKB/TrE		4	242454081	242589655	135575	141,29	-0,28	5,57E-01
ENSRNOG00000012260	**Ddx25**	DEAD (Asp-Glu-Ala-Asp) box helicase	58856	8	36761173	36777206	16034	1454,08	0,34	5,57E-01
ENSRNOG00000050829		Uncharacterized protein [Source:UniProtKB/TrE		X	10689908	10879323	189416	343,82	-0,29	5,57E-01
ENSRNOG00000005621	**Gxylt2**	Protein Gxylt2 [Source:UniProtKB/TrE	688618	4	197660979	197744356	83378	31,68	-0,45	5,57E-01
ENSRNOG00000021031	**Grn**	granulin (Grn), transcript variant 1, mF	29143	10	90165634	90171736	6103	2620,01	0,35	5,57E-01
ENSRNOG00000015131	**Ube2c**	ubiquitin-conjugating enzyme E2C (Ub	296368	3	167422569	167424969	2401	361,92	0,47	5,57E-01
ENSRNOG00000001181	**Wdr4**	WD repeat domain 4 (Wdr4), mRNA [S	690032	20	12429884	12446081	16198	372,60	0,34	5,58E-01
ENSRNOG00000009029	**Nptn**	neuroplastin (Nptn), mRNA [Source:R	56064	8	63149055	63213749	64695	3613,48	-0,26	5,58E-01
ENSRNOG00000024651		gene regulated by estrogen in breast c	500633	6	51663027	51732012	68986	18,49	-0,51	5,58E-01
ENSRNOG00000033123	**Pcdhb20**	protocadherin beta 20 (Pcdhb20), mR	680203	18	30288725	30292100	3376	157,15	-0,35	5,58E-01
ENSRNOG00000038911		Uncharacterized protein [Source:UniProtKB/TrE		9	49104098	49121583	17486	200,04	0,29	5,58E-01
ENSRNOG00000048286		family with sequence similarity 220, member A [S		12	15110179	15110421	243	132,04	0,33	5,58E-01

ENSRNOG00000001285		ATPase, Ca++ transporting, cardiac m	29693	12	41435157	41482924	47768	12605,81	-0,27	5,58E-01
ENSRNOG00000030530	**Gzmm**	granzyme M (lymphocyte met-ase 1) (	29252	7	13062468	13068067	5600	50,98	0,48	5,58E-01
ENSRNOG00000011463	**Psmb2**	proteasome (prosome, macropain) sul	29675	5	148472364	148504751	32388	3540,22	0,32	5,58E-01
ENSRNOG00000003264	**Nat9**	N-acetyltransferase 9 (GCN5-related,	303669	10	104566822	104571582	4761	532,03	0,35	5,59E-01
ENSRNOG00000046523		adenosine deaminase-like [Source:MGI Symbol;		3	119633204	119634337	1134	26,13	-0,47	5,59E-01
ENSRNOG00000012157	**Snrnp200**	small nuclear ribonucleoprotein 200 (U	296126	3	126165542	126194857	29316	7151,22	0,24	5,59E-01
ENSRNOG00000047598	**Ctdsp2**	Protein LOC100365370 [Source:UniP	1E+08	7	70460050	70464817	4768	429,68	0,32	5,59E-01
ENSRNOG00000010448		polypyrimidine tract binding protein 1 (	29497	7	12833688	12843440	9753	2624,11	0,29	5,60E-01
ENSRNOG00000020905	**Hdac5**	histone deacetylase 5 (Hdac5), mRNA	84580	10	89927486	89938344	10859	4158,72	0,23	5,60E-01
ENSRNOG00000025695	**Tns3**	tensin 3 (Tns3), mRNA [Source:RefSe	360980	14	88471109	88645807	174699	2508,53	-0,45	5,60E-01
ENSRNOG00000028358	**Edem3**	ER degradation enhancer, mannosida	289085	13	74110016	74168822	58807	682,99	-0,32	5,60E-01
ENSRNOG00000001060	**Snrnp35**	small nuclear ribonucleoprotein 35 (U	360803	12	39401204	39408530	7327	625,76	0,34	5,60E-01
ENSRNOG00000002152	**Dcun1d4**	DCN1, defective in cullin neddylation	360928	14	37033185	37103735	70551	1437,91	-0,27	5,60E-01
ENSRNOG00000005043	**Cpeb2**	Protein Cpeb2 [Source:UniProtKB/TrE	360949	14	72360166	72408363	48198	866,86	0,34	5,60E-01
ENSRNOG00000015003	**Pex11a**	peroxisomal biogenesis factor 11 alph	85249	1	142435410	142442491	7082	305,20	-0,33	5,60E-01
ENSRNOG00000000567	**Unc5b**	unc-5 homolog B (C. elegans) (Unc5b	60630	20	32150986	32214979	63994	372,01	-0,40	5,60E-01
ENSRNOG00000046741		Protein Itsn2 [Source:UniProtKB/TrEM	313934	6	39701691	39789879	88189	475,86	-0,31	5,60E-01
ENSRNOG00000001450	**Nsun5**	NOP2/Sun domain family, member 5 (	288595	12	26339243	26344203	4961	766,96	0,28	5,60E-01
ENSRNOG00000006027	**Eif2ak4**	eukaryotic translation initiation factor 2	114859	3	116705333	116791037	85705	711,22	-0,24	5,60E-01
ENSRNOG00000012457		Cytochrome c-1 (Predicted), isoform CRA_b; Pr		7	117397227	117399580	2354	5404,52	0,38	5,60E-01
ENSRNOG00000016655	**Pex6**	peroxisomal biogenesis factor 6 (Pex6	117265	9	15475358	15487515	12158	938,96	0,29	5,60E-01
ENSRNOG00000016989	**Dolk**	dolichol kinase (Dolk), mRNA [Source:	311847	3	14203272	14204882	1611	539,02	0,24	5,60E-01
ENSRNOG00000017770		cell division cycle protein 123 homolog	116656	17	78066968	78109667	42700	1744,71	0,29	5,60E-01
ENSRNOG00000026319	**Akap9**	A kinase (PRKA) anchor protein (yotia	246150	4	27098572	27234808	136237	2419,31	0,48	5,60E-01
ENSRNOG00000032942	**Neu1**	neuraminidase 1 (Neu1), mRNA [Sour	24591	20	6690982	6695245	4264	696,19	0,29	5,60E-01
ENSRNOG00000047536		zinc finger protein 677 [Source:MGI Symbol;Acc		1	63749071	63755493	6423	34,41	-0,52	5,60E-01
ENSRNOG00000002203	**Lin54**	lin-54 homolog (C. elegans) (Lin54), m	305171	14	10733789	10775701	41913	681,44	0,28	5,61E-01
ENSRNOG00000002478	**Insig2**	insulin induced gene 2 (Insig2), mRNA	288985	13	42366079	42388541	22463	595,80	0,43	5,61E-01
ENSRNOG00000008487	**Amotl2**	angiomotin like 2 (Amotl2), mRNA [So	65157	8	110604450	110619840	15391	2889,08	0,24	5,61E-01
ENSRNOG00000017635	**Inpp5a**	inositol polyphosphate-5-phosphatase	365382	1	218656329	218844831	188503	913,26	0,26	5,61E-01
ENSRNOG00000020776	**Dhcr7**	7-dehydrocholesterol reductase (Dhcr	64191	1	223874935	223890913	15979	4029,22	0,39	5,61E-01
ENSRNOG00000022847		dopey family member 1 [Source:MGI S	315864	8	93665370	93737976	72607	930,87	-0,31	5,61E-01
ENSRNOG00000039832		G protein-coupled receptor 12 (Gpr12)	80840	12	12359656	12361832	2177	341,61	-0,28	5,61E-01
ENSRNOG00000009412	**Lrch1**	leucine-rich repeats and calponin hom	502020	15	60507567	60691597	184031	228,61	-0,29	5,62E-01
ENSRNOG00000005258	**Myef2**	myelin expression factor 2 (Myef2), ml	679712	3	123877348	123913267	35920	2415,75	-0,38	5,62E-01
ENSRNOG00000012235	**Ppp1r17**	protein phosphatase 1, regulatory sub	266705	4	150928122	150944836	16715	64,28	-0,48	5,62E-01
ENSRNOG00000001861	**Ydjc**	YdjC homolog (bacterial) (Ydjc), mRN	287938	11	91143087	91144893	1807	1581,16	0,43	5,62E-01
ENSRNOG00000005616		Nuclear receptor coactivator 3 [Sourc	84584	3	168933287	168958994	25708	1102,20	0,25	5,62E-01

ENSRNOG00000013720	**Aebp1**	AE binding protein 1 (Aebp1), mRNA [	305494	14	86792973	86802766	9794	34,96	-0,54	5,62E-01
ENSRNOG00000015734	**Ube3a**	ubiquitin protein ligase E3A (Ube3a), r	361585	1	117746227	117834011	87785	1217,25	-0,42	5,62E-01
ENSRNOG00000032447	**Zfp641**	zinc finger protein 641 (Zfp641), mRN.	300197	7	139044646	139049328	4683	68,34	-0,42	5,62E-01
ENSRNOG00000029501	**Hoga1**	4-hydroxy-2-oxoglutarate aldolase 1 (I	293949	1	268744342	268771456	27115	122,15	-0,50	5,62E-01
ENSRNOG00000019384	**Med11**	mediator complex subunit 11 (Med11),	287456	10	56814847	56816361	1515	283,97	0,37	5,62E-01
ENSRNOG00000020956	**Bcat2**	branched chain amino acid transamin	64203	1	102633422	102650872	17451	399,35	0,26	5,62E-01
ENSRNOG00000002218	**Stbd1**	starch binding domain 1 (Stbd1), mRN	305234	14	16889875	16893279	3405	105,51	-0,49	5,63E-01
ENSRNOG00000003400		flavin containing monooxygenase 4 (F	246247	13	85580300	85598234	17935	17,40	-0,54	5,63E-01
ENSRNOG00000009643	**Msl1**	male-specific lethal 1 homolog (Droso	303514	10	86507519	86517491	9973	4374,58	0,28	5,63E-01
ENSRNOG00000014422	**Gtf2e2**	general transcription factor IIE, polype	306516	16	61778124	61826935	48812	380,37	0,39	5,63E-01
ENSRNOG00000049876	**Prkar1a**	protein kinase, cAMP-dependent, regu	25725	10	97655261	97671804	16544	8151,19	0,27	5,63E-01
ENSRNOG00000002175	**LOC10091**	clock circadian regulator (Clock), mRN	60447	14	34242961	34282766	39806	152,40	-0,40	5,63E-01
ENSRNOG00000005551	**LOC50144**	derlin 1 (Derl1), mRNA [Source:RefSe	362912	7	98344208	98366882	22675	2895,93	0,31	5,63E-01
ENSRNOG00000016182	**Tgfa**	transforming growth factor alpha (Tgfa	24827	4	182534326	182616713	82388	580,16	-0,47	5,63E-01
ENSRNOG00000049653		ERBB receptor feedback inhibitor 1 [Source:Uni		5	171529356	171541439	12084	332,01	0,27	5,63E-01
ENSRNOG00000015622	**Fbxo4**	F-box protein 4 (Fbxo4), mRNA [Sourc	310363	2	72830881	72844061	13181	94,50	-0,42	5,63E-01
ENSRNOG00000014665	**Dhdds**	dehydrodolichyl diphosphate synthase	298541	5	155884756	155912508	27753	1350,88	0,20	5,63E-01
ENSRNOG00000005402				X	113721821	113723127	1307	20,45	0,48	5,63E-01
ENSRNOG00000010719		Protein LOC313707; RCG30986, isofc	313707	5	169031609	169054708	23100	1719,43	0,30	5,63E-01
ENSRNOG00000013945	**Itpkc**	inositol-trisphosphate 3-kinase C (Itpk	308451	1	85229798	85251619	21822	532,82	0,21	5,63E-01
ENSRNOG00000033643	**Exoc2**	exocyst complex component 2 (Exoc2	171455	17	38560758	38697270	136513	1878,16	-0,19	5,63E-01
ENSRNOG00000007661	**Zw10**	zw10 kinetochore protein (Zw10), mRI	363059	8	51980064	52005883	25820	596,57	0,31	5,63E-01
ENSRNOG00000045553	**Proser2**	Protein LOC683460; RCG55724 [Sou	683460	17	77749863	77777856	27994	49,39	-0,41	5,63E-01
ENSRNOG00000003214				10	48771452	48773235	1784	206,57	-0,31	5,63E-01
ENSRNOG00000006329	**Peli1**	pellino E3 ubiquitin protein ligase 1 (P	305549	14	106075061	106128271	53211	375,76	-0,25	5,63E-01
ENSRNOG00000006806	**Setmar**	SET domain without mariner transpos	500281	4	204560093	204571717	11625	72,32	0,46	5,63E-01
ENSRNOG00000007323	**Ric8b**	resistance to inhibitors of cholinestera	314681	7	26049680	26142157	92478	613,45	0,28	5,63E-01
ENSRNOG00000008472	**Rbm10**	RNA binding motif protein 10 (Rbm10)	64510	X	2548300	2580031	31732	1891,78	0,29	5,63E-01
ENSRNOG00000010974	**Ror2**	receptor tyrosine kinase-like orphan re	306782	17	14050995	14228982	177988	92,41	-0,46	5,63E-01
ENSRNOG00000016267	**Chst15**	carbohydrate (N-acetylgalactosamine	286974	1	211242707	211272037	29331	239,80	0,25	5,64E-01
ENSRNOG00000020191	**Tbc1d17**	TBC1 domain family, member 17 (Tbc	292886	1	101901664	101909886	8223	1647,93	0,36	5,64E-01
ENSRNOG00000026748	**Dennd2a**	Protein Dennd2a [Source:UniProtKB/	312257	4	67050512	67107852	57341	972,72	-0,34	5,64E-01
ENSRNOG00000049685		Uncharacterized protein [Source:UniProtKB/TrE		2	239910910	240089776	178867	21,35	-0,45	5,64E-01
ENSRNOG00000001440	**Mdh2**	malate dehydrogenase 2, NAD (mitocl	81829	12	25938703	25951658	12956	7448,42	0,28	5,64E-01
ENSRNOG00000001966	**Zbtb20**	zinc finger and BTB domain containin	288105	11	61147996	61583999	436004	508,99	-0,48	5,64E-01
ENSRNOG00000002191	**LOC49836**	RIKEN cDNA 0610040J01 gene [Sour	498368	14	45855972	45858411	2440	133,88	-0,33	5,64E-01
ENSRNOG00000003365	**Cadm3**	cell adhesion molecule 3 (Cadm3), mF	360882	13	96356701	96388464	31764	10703,33	-0,29	5,64E-01
ENSRNOG00000025715	**Dynlrb1**	dynein light chain roadblock-type 1 (D	170714	3	157129655	157150710	21056	8891,13	0,35	5,64E-01

ENSRNOG00000015835	**Cacna2d2**	calcium channel, voltage-dependent, a	300992	8	115510989	115640006	129018	597,81	-0,25	5,64E-01
ENSRNOG00000016778		COP9 signalosome subunit 7A (Cops7	312710	4	224497387	224504671	7285	1650,02	0,24	5,64E-01
ENSRNOG00000020889	**Nucb1**	nucleobindin 1 (Nucb1), mRNA [Sourc	84595	1	102559073	102579561	20489	3753,21	0,25	5,64E-01
ENSRNOG00000022466	**LOC10036**	coiled-coil-helix-coiled-coil-helix doma	312559	4	187427514	187436775	9262	231,88	0,36	5,64E-01
ENSRNOG00000049915		thrombospondin, type I, domain containing 4 [Sc		8	64574557	64669697	95141	40,94	-0,54	5,64E-01
ENSRNOG00000016629	**Zcchc6**	Protein Zcchc6 [Source:UniProtKB/Tr	501515	17	7452315	7504093	51779	1331,39	0,23	5,64E-01
ENSRNOG00000019682	**Timm13**	translocase of inner mitochondrial mer	252928	7	11844860	11845962	1103	3801,56	0,39	5,64E-01
ENSRNOG00000028628		zinc finger protein 865 [Source:MGI Symbol;Acc		1	76171248	76172483	1236	99,42	0,31	5,65E-01
ENSRNOG00000000818	**Nrm**	nurim (nuclear envelope membrane pr	361791	20	5494940	5498418	3479	224,37	0,31	5,65E-01
ENSRNOG00000028742		striatin interacting protein 2 [Source:MGI Symbo		4	56972105	57017628	45524	91,72	-0,51	5,65E-01
ENSRNOG00000007512	**Srp14**	signal recognition particle 14 (Srp14),	296076	3	116791591	116795282	3692	1636,04	0,23	5,65E-01
ENSRNOG00000009039	**Trappc12**	Protein Trappc12 [Source:UniProtKB/	314013	6	56656859	56722274	65416	1361,77	-0,30	5,65E-01
ENSRNOG00000009598	**Ncaph2**	non-SMC condensin II complex, subur	300149	7	130011951	130027604	15654	2559,25	0,20	5,65E-01
ENSRNOG00000013211	**Chchd3**	coiled-coil-helix-coiled-coil-helix doma	296966	4	59822342	60099177	276836	1633,49	-0,21	5,65E-01
ENSRNOG00000014072	**Txndc17**	thioredoxin domain containing 17 (Txn	287474	10	58517203	58520175	2973	538,60	0,29	5,65E-01
ENSRNOG00000017724	**Sf3b3**	splicing factor 3b, subunit 3 (Sf3b3), m	292019	19	54224944	54262543	37600	4116,24	0,28	5,65E-01
ENSRNOG00000030210	**Fndc1**	fibronectin type III domain containing 1	308099	1	48905802	48987223	81422	105,94	-0,49	5,65E-01
ENSRNOG00000048394	**Zfp560**	Protein LOC100359967; RCG31761, i	1E+08	8	21444670	21453959	9290	158,71	-0,30	5,65E-01
ENSRNOG00000019651	**Slc12a4**	solute carrier family 12 (potassium/chl	29501	19	48784045	48805888	21844	866,03	-0,36	5,66E-01
ENSRNOG00000017538	**Lrrc27**	leucine rich repeat containing 27 (Lrrc2	499281	1	218470409	218499898	29490	90,63	0,30	5,66E-01
ENSRNOG00000028323		dehydrogenase/reductase (SDR family	288525	12	20501893	20506182	4290	1105,25	0,40	5,66E-01
ENSRNOG00000016265		long-chain-fatty-acid--CoA ligase 5 [S	94340	1	283660224	283685366	25143	780,16	0,32	5,66E-01
ENSRNOG00000003785	**Usp43**	ubiquitin specific peptidase 43 (Usp43	1E+08	10	54152906	54213665	60760	83,51	-0,41	5,67E-01
ENSRNOG00000004400	**Avpr1a**	arginine vasopressin receptor 1A (Avp	25107	7	67528203	67532266	4064	20,23	-0,48	5,67E-01
ENSRNOG00000008572	**Ppcs**	phosphopantothenoylcysteine synthet	298490	5	142276594	142279846	3253	349,78	0,23	5,67E-01
ENSRNOG00000016217	**Gtpbp4**	GTP binding protein 4 (Gtpbp4), mRN	114300	17	64881147	64916648	35502	61,43	-0,41	5,67E-01
ENSRNOG00000017991	**Cdk20**	cyclin-dependent kinase 20 (Cdk20), r	364666	17	1924304	1931006	6703	710,03	0,25	5,67E-01
ENSRNOG00000049351	**Nat8l**	N-acetyltransferase 8-like (Nat8l), mR	289727	14	82725856	82732491	6636	2599,35	0,27	5,67E-01
ENSRNOG00000000999	**Smurf1**	SMAD specific E3 ubiquitin protein liga	690516	12	13499018	13591105	92088	1790,00	-0,25	5,67E-01
ENSRNOG00000005377		transmembrane protein 11 (Tmem11),	303196	10	46890611	46905318	14708	456,03	0,27	5,67E-01
ENSRNOG00000013264	**Pde12**	phosphodiesterase 12 (Pde12), mRNA	306231	16	2332374	2343389	11016	284,01	0,25	5,67E-01
ENSRNOG00000014170	**Dbn1**	drebrin 1 (Dbn1), mRNA [Source:RefS	81653	17	11788597	11802569	13973	12170,63	0,27	5,67E-01
ENSRNOG00000015337	**Phf10**	PHD finger protein 10 (Phf10), mRNA	292404	1	58134864	58191474	56611	1037,77	-0,21	5,67E-01
ENSRNOG00000019229	**Fbl**	fibrillarin (Fbl), mRNA [Source:RefSeq	292747	1	86319815	86328915	9101	1563,48	0,37	5,67E-01
ENSRNOG00000049244	**Sepsecs**	Sep (O-phosphoserine) tRNA:Sec (sel	679383	14	60774968	60804615	29648	293,55	-0,32	5,67E-01
ENSRNOG00000047950		Kinesin-like protein KIF22 [Source:Un	293502	1	205456384	205467610	11227	41,27	0,47	5,67E-01
ENSRNOG00000046245	**March9**	membrane-associated ring finger (C3H	679272	7	70520711	70522791	2081	1364,62	0,29	5,67E-01
ENSRNOG00000011821	**S100a4**	S100 calcium-binding protein A4 (S10	24615	2	209431370	209433679	2310	92,45	-0,45	5,67E-01

ENSRNOG00000020113	**Cnnm2**	cyclin M2 (Cnnm2), mRNA [Source:Re	294014	1	273961301	274079325	118025	435,30	0,29	5,67E-01
ENSRNOG00000033663	**P4ha2**	prolyl 4-hydroxylase, alpha polypeptid	360526	10	39214791	39243698	28908	80,49	-0,38	5,67E-01
ENSRNOG00000036866	**Acer3**	Protein Acer3; Similar to Alkaline phyt	499210	1	169371624	169469541	97918	78,46	-0,42	5,67E-01
ENSRNOG00000001559	**Mtx2**	metaxin 2 (Mtx2), mRNA [Source:RefS	288150	3	68216583	68279581	62999	848,29	0,31	5,67E-01
ENSRNOG00000005333	**Azin1**	antizyme inhibitor 1 (Azin1), mRNA [S	58961	7	77448675	77475344	26670	5390,15	-0,30	5,67E-01
ENSRNOG00000010985	**Zfp410**	zinc finger protein 410 (Zfp410), mRN	314310	6	117487588	117513541	25954	1134,43	0,38	5,67E-01
ENSRNOG00000049937	**Pdxk**	pyridoxal (pyridoxine, vitamin B6) kina	83578	20	13102325	13124153	21829	416,95	-0,26	5,67E-01
ENSRNOG00000050710		Uncharacterized protein [Source:UniProtKB/TrE		12	46583539	46731810	148272	91,41	-0,33	5,67E-01
ENSRNOG00000010150	**Strbp**	spermatid perinuclear RNA binding pr	84476	3	27069384	27141888	72505	1432,75	-0,37	5,67E-01
ENSRNOG00000001143		citron (rho-interacting, serine/threonin	83620	12	48135859	48294184	158326	799,04	-0,25	5,67E-01
ENSRNOG00000016591	**Utp15**	UTP15, U3 small nucleolar ribonucleo	310019	2	47453660	47472161	18502	980,61	-0,21	5,67E-01
ENSRNOG00000000036	**Klhdc8a**	kelch domain containing 8A (Klhdc8a)	305096	13	54143333	54151418	8086	253,98	-0,37	5,67E-01
ENSRNOG00000015095	**Spryd7**	SPRY domain containing 7 (Spryd7), r	290303	15	45673596	45693135	19540	758,78	-0,25	5,68E-01
ENSRNOG00000023262	**Cenpm**	centromere protein M (Cenpm), mRN	315164	7	123449521	123460809	11289	228,04	0,44	5,68E-01
ENSRNOG00000032579	**Zswim2**	zinc finger, SWIM-type containing 2 (Z	296455	3	77799742	77821010	21269	37,37	-0,38	5,68E-01
ENSRNOG00000007262	**Ccdc134**	coiled-coil domain containing 134 (Cc	500909	7	123347804	123359647	11844	312,03	0,24	5,68E-01
ENSRNOG00000012349	**Mesdc1**	mesoderm development candidate 1 (	308795	1	146957080	146958168	1089	400,50	0,36	5,69E-01
ENSRNOG00000049194		Protein LOC691414 [Source:UniProtK	501396	9	11415876	11555188	139313	58,95	-0,40	5,69E-01
ENSRNOG00000025910	**Hccs**	holocytochrome c synthase (Hccs), m	317444	X	26698858	26708253	9396	449,75	-0,32	5,69E-01
ENSRNOG00000003348	**Rasd1**	RAS, dexamethasone-induced 1 (Ras	64455	10	46087072	46088803	1732	28,41	-0,41	5,69E-01
ENSRNOG00000007486	**Atg7**	autophagy related 7 (Atg7), mRNA [S	312647	4	209889721	210066748	177028	431,17	0,27	5,69E-01
ENSRNOG00000008300	**Zbtb8a**	zinc finger and BTB domain containin	313049	5	151276506	151303640	27135	225,07	0,30	5,69E-01
ENSRNOG00000015800	**Elmo3**	engulfment and cell motility 3 (Elmo3)	291962	19	48126191	48130551	4361	36,70	-0,45	5,69E-01
ENSRNOG00000020302	**Klhl17**	kelch-like family member 17 (Klhl17),	246757	5	177128900	177133715	4816	299,95	0,37	5,69E-01
ENSRNOG00000045588		Protein RGD1561962 [Source:UniPro	304201	12	4753939	4763811	9873	374,97	0,28	5,69E-01
ENSRNOG00000018775	**Cystm1**	Protein Cystm1; RCG49325, isoform (	1E+08	18	28878687	28939398	60712	1350,77	0,36	5,69E-01
ENSRNOG00000033402	**LOC50111(**	similar to Glutathione S-transferase A	501110	9	26322566	26358469	35904	157,59	-0,41	5,69E-01
ENSRNOG00000007946		Bcl2-like 1 (Bcl2l1), transcript variant 2	24888	3	154662586	154716802	54217	1369,56	0,26	5,69E-01
ENSRNOG00000006711		Protein Zfp212; Zinc finger protein 212 [Source:		4	142261651	142273942	12292	651,84	0,30	5,69E-01
ENSRNOG00000020486	**Bcl2l12**	BCL2-like 12 (proline rich) (Bcl2l12), n	361567	1	102048990	102057872	8883	106,73	0,42	5,69E-01
ENSRNOG00000014992	**M6pr**	mannose-6-phosphate receptor, catio	312689	X	88235941	88245064	9124	472,23	0,34	5,70E-01
ENSRNOG00000012879	**Fabp3**	fatty acid binding protein 3, muscle an	79131	5	152246244	152253006	6763	1080,70	0,30	5,70E-01
ENSRNOG00000028505	**Rps18**	ribosomal protein S18 (Rps18), mRN	294282	20	7500487	7504165	3679	453,46	0,33	5,70E-01
ENSRNOG00000018262	**Ampd3**	adenosine monophosphate deaminas	25095	1	182574033	182618403	44371	447,02	-0,39	5,70E-01
ENSRNOG00000012616	**Ppt1**	palmitoyl-protein thioesterase 1 (Ppt1)	29411	5	144329251	144350133	20883	1989,74	-0,33	5,70E-01
ENSRNOG00000016608	**Zfp579**	zinc finger protein 579 (Zfp579), mRN	308339	1	76133750	76136920	3171	613,02	0,29	5,70E-01
ENSRNOG00000022252	**Kbtbd3**	kelch repeat and BTB (POZ) domain c	315394	8	1458405	1472634	14230	138,14	-0,43	5,70E-01
ENSRNOG00000001172	**Rnf10**	ring finger protein 10 (Rnf10), mRNA [	288710	12	48897002	48930784	33783	8001,51	0,26	5,70E-01

ENSRNOG00000003497	**Thoc6**	THO complex 6 homolog (Drosophila)	79227	10	12812180	12817540	5361	643,37	0,31	5,70E-01
ENSRNOG00000006523	**Atl2**	atlastin GTPase 2 (Atl2), mRNA [Sour	298757	6	2548708	2590119	41412	847,63	-0,36	5,70E-01
ENSRNOG00000009465	**Sfrp2**	secreted frizzled-related protein 2 (Sfr	310552	2	202136233	202143777	7545	601,75	-0,41	5,70E-01
ENSRNOG00000009971	**Plekha8**	pleckstrin homology domain containin	500132	4	149430315	149480827	50513	579,92	-0,34	5,70E-01
ENSRNOG00000010224	**Rab30**	RAB30, member RAS oncogene famil	308821	1	163889802	163905429	15628	509,11	-0,30	5,70E-01
ENSRNOG00000015144	**Ist1**	increased sodium tolerance 1 homolo	307833	19	52793724	52815862	22139	3707,17	0,37	5,70E-01
ENSRNOG00000019156	**Aurkaip1**	aurora kinase A interacting protein 1 (/	298687	5	176750389	176751784	1396	1320,51	0,21	5,70E-01
ENSRNOG00000020642	**Flad1**	flavin adenine dinucleotide synthetase	751787	2	208142452	208150771	8320	758,95	0,22	5,70E-01
ENSRNOG00000002050	**Igfbp7**	insulin-like growth factor binding prote	289560	14	32803851	32863713	59863	746,05	-0,50	5,70E-01
ENSRNOG00000006247	**Copa**	coatomer protein complex subunit alpl	304978	13	94990249	95030030	39782	10483,78	0,29	5,70E-01
ENSRNOG00000020269	**Sugp2**	SURP and G patch domain containing	361126	16	20760491	20789858	29368	3642,47	0,30	5,70E-01
ENSRNOG00000043225		zinc finger protein 771 [Source:MGI Symbol;Acc		1	205692999	205694000	1002	1181,22	0,44	5,70E-01
ENSRNOG00000015575		Protein LOC312502 [Source:UniProtK	312502	4	181695212	181731936	36725	2039,22	0,22	5,71E-01
ENSRNOG00000005715	**Lgr4**	leucine-rich repeat-containing G prote	286994	3	107652228	107752743	100516	1151,98	-0,40	5,71E-01
ENSRNOG00000017359	**Itm2c**	integral membrane protein 2C (Itm2c),	301575	9	92645770	92659580	13811	13713,89	0,27	5,71E-01
ENSRNOG00000028651	**RGD15663**	uncharacterized protein LOC304336		304336	12	20094026	20128202	34177	133,27	-0,36	5,71E-01
ENSRNOG00000037738	**LOC50025**	5-hydroxymethylcytosine (hmC) bindir	500251	4	185089595	185111626	22032	799,44	-0,22	5,71E-01
ENSRNOG00000023467	**Fam168b**	family with sequence similarity 168, m	690188	9	40978314	41000260	21947	1647,37	0,35	5,71E-01
ENSRNOG00000006062	**Hs1bp3**	Protein Hs1bp3 [Source:UniProtKB/Tı	313950	6	43302693	43333012	30320	792,00	0,29	5,72E-01
ENSRNOG00000009642	**Cct4**	chaperonin containing Tcp1, subunit 4	29374	14	107843411	107856260	12850	4934,83	0,33	5,72E-01
ENSRNOG00000010048	**Dctn1**	dynactin 1 (Dctn1), mRNA [Source:Re	29167	4	179465836	179485632	19797	12516,19	0,27	5,72E-01
ENSRNOG00000010409	**Nol6**	Neuroprotective protein 1; Protein Nol	313167	5	61976812	61981741	4930	1503,21	0,25	5,72E-01
ENSRNOG00000011509	**Agk**	acylglycerol kinase (Agk), mRNA [Sou	502749	4	133268922	133346790	77869	1222,13	0,33	5,72E-01
ENSRNOG00000014543	**Tbc1d2b**	TBC1 domain family, member 2B (Tbc	315880	8	97076275	97145782	69508	1171,43	-0,35	5,72E-01
ENSRNOG00000019103	**Cln3**	ceroid-lipofuscinosis, neuronal 3 (Cln3	293485	1	204965145	204976424	11280	369,00	0,36	5,72E-01
ENSRNOG00000020527	**Drap1**	Dr1 associated protein 1 (negative cof	293674	1	227733127	227735764	2638	2983,72	0,24	5,72E-01
ENSRNOG00000047625	**Atg4d**	autophagy related 4D, cysteine peptid	686505	8	22344081	22353967	9887	851,47	0,37	5,72E-01
ENSRNOG00000011503	**Shb**	Protein Shb [Source:UniProtKB/TrEM	362513	5	65479897	65586265	106369	490,69	0,23	5,72E-01
ENSRNOG00000032215	**Cdyl**	chromodomain protein, Y-like (Cdyl), n	361237	17	31569734	31795070	225337	251,95	0,23	5,72E-01
ENSRNOG00000000248	**Srsf2**	serine/arginine-rich splicing factor 2 (S	494445	10	105451332	105454511	3180	7059,55	0,25	5,72E-01
ENSRNOG00000000824	**Dse**	dermatan sulfate epimerase (Dse), mF	365583	20	29528501	29603844	75344	152,64	-0,27	5,72E-01
ENSRNOG00000001107	**Ap5z1**	adaptor-related protein complex 5, zet	641386	12	16198608	16204550	5943	170,45	0,28	5,72E-01
ENSRNOG00000004426	**LOC67997**	ribosomal protein S27a (Rps27a), mR	81777	14	113632952	113634784	1833	89,90	-0,30	5,72E-01
ENSRNOG00000012872	**RGD13118**	similar to RIKEN cDNA 2410127L17 (I	293871	1	241777572	241804669	27098	153,91	-0,43	5,72E-01
ENSRNOG00000016193	**RGD15621**	RGD1562114 (RGD1562114), mRNA	500795	7	12568305	12570015	1711	134,47	0,38	5,72E-01
ENSRNOG00000016867	**Zfp346**	zinc finger protein 346 (Zfp346), mRN	306765	17	12131051	12168912	37862	1376,89	0,21	5,72E-01
ENSRNOG00000013974	**Fahd2a**	fumarylacetoacetate hydrolase domai	296131	3	127321224	127329382	8159	612,22	0,28	5,72E-01
ENSRNOG00000002010	**Mrps6**	Protein LOC100360017; RCG58764 [	1E+08	11	35816151	35868614	52464	1086,51	-0,45	5,72E-01

ENSRNOG00000005577	**Desi1**	desumoylating isopeptidase 1 (Desi1),	315160	7	123223023	123244325	21303	1555,53	0,18	5,72E-01
ENSRNOG00000008443	**RGD13061**	uncharacterized protein LOC362455 |	362455	4	233247179	233290087	42909	3902,70	0,33	5,72E-01
ENSRNOG00000010533	**Arfip1**	ADP-ribosylation factor interacting pro	60382	2	202912659	202990882	78224	276,15	-0,40	5,73E-01
ENSRNOG00000001991	**Atp5o**	ATP synthase, H+ transporting, mitoch	192241	11	35685318	35691665	6348	5446,16	0,33	5,73E-01
ENSRNOG00000008950	**Adi1**	acireductone dioxygenase 1 (Adi1), m	298934	6	56642239	56649206	6968	302,43	-0,38	5,73E-01
ENSRNOG00000034272	**Pias1**	protein inhibitor of activated STAT, 1 (I	300772	8	67500467	67597766	97300	455,75	-0,30	5,73E-01
ENSRNOG00000014890	**Mrpl43**	mitochondrial ribosomal protein L43 (M	309440	1	272197964	272198767	804	1385,12	0,27	5,73E-01
ENSRNOG00000004904	**Pa2g4**	proliferation-associated 2G4 (Pa2g4),	288778	7	2953212	2960679	7468	4281,93	0,23	5,74E-01
ENSRNOG00000008382	**RGD15644**	Protein RGD1564405 [Source:UniPro	500386	5	3657788	3660120	2333	19,61	-0,50	5,74E-01
ENSRNOG00000010107	**Palld**	Protein LOC100360205 [Source:UniP	1E+08	16	31748095	31784275	36181	351,47	0,32	5,74E-01
ENSRNOG00000015495	**Slc25a37**	solute carrier family 25 (mitochondrial	306000	15	54858326	54898186	39861	48,60	-0,38	5,74E-01
ENSRNOG00000024800		diaphanous homolog 2 (Drosophila) [Source:MG		X	99734290	100216249	481960	37,21	-0,51	5,74E-01
ENSRNOG00000050111	**Cenpo**	Protein LOC684439 [Source:UniProtK	684439	6	38455912	38469073	13162	55,59	0,34	5,74E-01
ENSRNOG00000001316	**Anapc5**	anaphase-promoting complex subunit	288671	12	41102284	41134467	32184	11564,20	0,28	5,74E-01
ENSRNOG00000021552	**Uprt**	Protein Uprt [Source:UniProtKB/TrEM	317237	X	76185119	76209386	24268	188,38	-0,31	5,74E-01
ENSRNOG00000008212	**Wdr5**	WD repeat domain 5 (Wdr5), mRNA [S	362093	3	11423443	11441547	18105	1646,23	0,28	5,74E-01
ENSRNOG00000019207	**Shank1**	SH3 and multiple ankyrin repeat doma	78957	1	101362263	101409488	47226	8492,34	-0,32	5,74E-01
ENSRNOG00000008182	**Htra3**	HtrA serine peptidase 3 (Htra3), mRNA	360959	14	79875571	79902664	27094	75,66	-0,49	5,74E-01
ENSRNOG00000010834	**Mpped1**	metallophosphoesterase domain conta	362971	7	124671298	124740991	69694	6957,14	-0,30	5,74E-01
ENSRNOG00000040110	**Foxn2**	Protein Foxn2 [Source:UniProtKB/TrE	301676	6	22145314	22167316	22003	223,20	-0,31	5,74E-01
ENSRNOG00000007098	**Gtpbp10**	GTP-binding protein 10 (putative) (Gtp	312054	4	25542933	25560451	17519	164,63	-0,43	5,75E-01
ENSRNOG00000009565	**Pdk4**	pyruvate dehydrogenase kinase, isozy	89813	4	30451701	30463752	12052	59,54	-0,53	5,75E-01
ENSRNOG00000021399	**RGD13069**	similar to RIKEN cDNA 1110004E09 (I	288269	11	34705595	34714961	9367	993,51	0,26	5,75E-01
ENSRNOG00000049430	**Mpv17**	MPV17 mitochondrial membrane prote	360463	6	36403896	36413172	9277	210,35	-0,39	5,75E-01
ENSRNOG00000050735	**Cd99**	CD99 molecule (Cd99), mRNA [Sourc	652929	20	57779377	57783907	4531	1336,12	0,37	5,75E-01
ENSRNOG00000017260	**Cdr2**	cerebellar degeneration-related 2 (Cdr	308958	1	197815831	197840738	24908	326,75	0,25	5,76E-01
ENSRNOG00000003194				X	106603068	106603956	889	69,77	0,40	5,76E-01
ENSRNOG00000020456	**Nucb2**	nucleobindin 2 (Nucb2), mRNA [Sourc	59295	1	192078547	192114979	36433	447,92	-0,48	5,76E-01
ENSRNOG00000050180	**Zfpl1**	Protein LOC684755; RCG47159, isofo	684755	1	228380028	228383974	3947	822,98	0,27	5,76E-01
ENSRNOG00000003066	**Wnt9a**	wingless-type MMTV integration site fa	287357	10	45414117	45441637	27521	99,91	-0,37	5,76E-01
ENSRNOG00000000523	**Ppil1**	peptidylprolyl isomerase (cyclophilin)-l	309651	20	8760185	8761635	1451	920,68	0,24	5,76E-01
ENSRNOG00000005632	**Pdzrn3**	PDZ domain containing RING finger 3	312607	4	198195671	198429459	233789	969,91	-0,36	5,76E-01
ENSRNOG00000016138	**Crtc2**	CREB regulated transcription coactiva	310615	2	209048803	209058907	10105	767,07	0,22	5,76E-01
ENSRNOG00000022431	**Zfp867**	zinc finger protein 867 (Zfp867), mRN	1E+08	10	45566087	45568322	2236	68,36	-0,48	5,76E-01
ENSRNOG00000046710	**Pcdha8**	protocadherin alpha 8 (Pcdha8), mRN	116781	18	29696540	29924444	227905	70,99	-0,35	5,76E-01
ENSRNOG00000007979	**Phospho2**	phosphatase, orphan 2 (Phospho2), m	295663	3	62667716	62674876	7161	387,73	-0,27	5,76E-01
ENSRNOG00000004925		protein phosphatase 1, regulatory sub	116670	7	51419607	51527799	108193	1446,45	-0,34	5,76E-01
ENSRNOG00000002823	**Mapk9**	mitogen-activated protein kinase 9 (Ma	50658	10	35114480	35145312	30833	2542,02	-0,28	5,76E-01

ENSRNOG00000014633	**Vps28**	vacuolar protein sorting 28 homolog (S	300052	7	117672422	117676270	3849	2989,50	0,21	5,76E-01
ENSRNOG00000042213	**Zfp498**	zinc finger protein 498 (Zfp498), mRN.	363872	12	13837227	13847687	10461	373,68	0,26	5,76E-01
ENSRNOG00000038868	**Iqcb1**	IQ motif containing B1 (Iqcb1), mRNA	303915	11	69914670	69969805	55136	272,38	-0,41	5,76E-01
ENSRNOG00000049784	**Tsnax**	translin-associated factor X (Tsnax), m	64028	19	68481972	68495992	14021	3238,35	0,33	5,76E-01
ENSRNOG00000011922	**Stt3b**	STT3B, subunit of the oligosaccharyltr	363160	8	122604522	122668755	64234	1652,40	-0,22	5,76E-01
ENSRNOG00000014039	**Sucnr1**	succinate receptor 1 (Sucnr1), mRNA	408199	2	169643631	169644582	952	18,37	-0,52	5,76E-01
ENSRNOG00000017045	**Zdhhc1**	zinc finger, DHHC-type containing 1 (Z	291967	19	48294576	48306088	11513	235,25	-0,44	5,76E-01
ENSRNOG00000020753	**Bnip1**	BCL2/adenovirus E1B interacting prot	140932	10	16565648	16577894	12247	476,97	0,26	5,76E-01
ENSRNOG00000015033	**Mxd4**	Max dimerization protein 4 (Mxd4), mF	360961	14	82504737	82516133	11397	1270,66	0,28	5,76E-01
ENSRNOG00000019318	**Syt3**	synaptotagmin III (Syt3), mRNA [Sour	25731	1	101459652	101473304	13653	759,42	0,32	5,76E-01
ENSRNOG00000022800		nuclear body protein SP140 [Source:I	316580	9	92349344	92396014	46671	20,97	0,51	5,76E-01
ENSRNOG00000001149		paxillin (Pxn), mRNA [Source:RefSeq	360820	12	48595400	48642173	46774	953,25	0,22	5,77E-01
ENSRNOG00000011522	**Hcn1**	hyperpolarization-activated cyclic nucl	84390	2	68473431	68874494	401064	337,42	-0,27	5,77E-01
ENSRNOG00000003594	**Tmem183a**	transmembrane protein 183A (Tmem1	289034	13	56242447	56257679	15233	3065,43	0,34	5,77E-01
ENSRNOG00000004510	**Slc35b1**	solute carrier family 35, member B1 (S	287642	10	83010919	83017992	7074	2261,71	0,26	5,77E-01
ENSRNOG00000004629	**Fkbp3**	FK506 binding protein 3 (Fkbp3), mRN	299104	6	96299844	96311094	11251	2744,42	-0,25	5,77E-01
ENSRNOG00000007341	**Mroh8**	Protein Mroh8 [Source:UniProtKB/TrE	1E+08	3	158321285	158386288	65004	64,12	0,33	5,77E-01
ENSRNOG00000008584	**Rnaseh1**	ribonuclease H1 (Rnaseh1), mRNA [S	298933	6	56618249	56627607	9359	222,32	-0,29	5,77E-01
ENSRNOG00000028120				2	185686408	185688558	2151	119,77	-0,41	5,77E-01
ENSRNOG00000001010	**Tecpr1**	tectonin beta-propeller repeat containi	304285	12	14426445	14454186	27742	1509,36	0,22	5,77E-01
ENSRNOG00000026157	**RGD15622**	Protein RGD1562200 [Source:UniPro	363471	X	45741177	45744726	3550	62,47	0,40	5,77E-01
ENSRNOG00000018397	**Dnph1**	2'-deoxynucleoside 5'-phosphate N-hy	171047	9	15747109	15749854	2746	458,12	0,31	5,77E-01
ENSRNOG00000032948	**Tyk2**	tyrosine kinase 2 (Tyk2), mRNA [Sour	1E+08	8	22180760	22201861	21102	345,44	0,31	5,77E-01
ENSRNOG00000019752	**Slc29a1**	solute carrier family 29 (equilibrative n	63997	9	16673067	16687769	14703	35,50	-0,45	5,77E-01
ENSRNOG00000013598	**Melk**	maternal embryonic leucine zipper kin	362510	5	64307464	64367854	60391	209,40	0,40	5,77E-01
ENSRNOG00000017208	**Cspg4**	chondroitin sulfate proteoglycan 4 (Cs	81651	8	60101160	60136205	35046	441,31	-0,52	5,77E-01
ENSRNOG00000028062	**Arpc5**	actin related protein 2/3 complex, subu	360854	13	75146057	75154961	8905	3847,19	0,39	5,77E-01
ENSRNOG00000050205	**Afmid**	arylformamidase (Afmid), mRNA [Sou	688283	10	106468206	106483539	15334	120,52	-0,49	5,77E-01
ENSRNOG00000002569	**Mrps14**	mitochondrial ribosomal protein S14 (M	289143	13	82847625	82853368	5744	944,83	-0,28	5,77E-01
ENSRNOG00000023337	**Sema3a**	sema domain, immunoglobulin domair	29751	4	18141779	18354542	212764	121,63	0,49	5,77E-01
ENSRNOG00000015553	**Gatad2b**	GATA zinc finger domain containing 2E	310614	2	209087998	209163193	75196	793,24	-0,22	5,78E-01
ENSRNOG00000021256	**Adra1d**	adrenoceptor alpha 1D (Adra1d), mRN	29413	3	130621199	130637207	16009	17,62	-0,49	5,78E-01
ENSRNOG00000023577	**Gigyf2**	Protein LOC100362458 [Source:UniP	1E+08	9	94256279	94270201	13923	436,68	-0,26	5,78E-01
ENSRNOG00000020374	**Tmem8a**	transmembrane protein 8A (Tmem8a),	303004	10	15299605	15309077	9473	387,21	0,24	5,78E-01
ENSRNOG00000016015	**Dcp1a**	DCP1 decapping enzyme homolog A (	361109	16	6476320	6517715	41396	342,76	0,30	5,78E-01
ENSRNOG00000019128	**St8sia4**	ST8 alpha-N-acetyl-neuraminide alpha	116696	9	102730314	102820583	90270	95,25	-0,39	5,78E-01
ENSRNOG00000005308	**Tmx2**	thioredoxin-related transmembrane pr	295701	3	78594077	78601725	7649	3438,39	0,39	5,78E-01
ENSRNOG00000008080		histamine receptor H3 (Hrh3), transcri	85268	3	181639287	181644371	5085	191,07	-0,41	5,78E-01

ENSRNOG00000011294	**Usp33**	ubiquitin specific peptidase 33 (Usp33	310960	2	276246709	276280815	34107	2045,93	-0,27	5,78E-01
ENSRNOG00000021263	**Tmem230**	transmembrane protein 230 (Tmem23	681315	3	131364937	131374753	9817	1136,41	0,39	5,78E-01
ENSRNOG00000021355	**Car6**	carbonic anhydrase 6 (Car6), mRNA [	298657	5	170854181	170872721	18541	20,30	-0,49	5,78E-01
ENSRNOG00000022218		interferon-induced protein 44 [Source	310969	2	275575815	275593538	17724	225,74	0,46	5,78E-01
ENSRNOG00000049110	**Dpm2**	dolichyl-phosphate mannosyltransfera	29640	3	16938349	16940937	2589	2339,14	0,29	5,78E-01
ENSRNOG00000028837	**Myl6b**	Protein Myl6b; Similar to Myosin light	1E+08	7	2883129	2885907	2779	1087,47	0,26	5,78E-01
ENSRNOG00000013994	**Enpp1**	ectonucleotide pyrophosphatase/phos	85496	1	23228281	23291660	63380	18,67	-0,52	5,78E-01
ENSRNOG00000017105		dihydropyrimidine dehydrogenase [Source:MGI		2	240215937	240744042	528106	130,70	-0,31	5,78E-01
ENSRNOG00000025327	**Tert**	telomerase reverse transcriptase (Tert	301965	1	33676582	33697344	20763	38,36	-0,41	5,78E-01
ENSRNOG00000046975	**Sult4a1**	sulfotransferase family 4A, member 1	58953	7	124946961	124970976	24016	6697,77	0,31	5,78E-01
ENSRNOG00000017983	**Ubac1**	UBA domain containing 1 (Ubac1), mF	362087	3	8815483	8838290	22808	2373,69	0,23	5,78E-01
ENSRNOG00000012693	**Pmpcb**	peptidase (mitochondrial processing)	64198	4	9909977	9922768	12792	1648,59	0,24	5,79E-01
ENSRNOG00000018792	**Tekt4**	tektin 4 (Tekt4), mRNA [Source:RefSe	302991	10	14644898	14650074	5177	30,79	-0,52	5,79E-01
ENSRNOG00000021170	**Plekho1**	pleckstrin homology domain containin	310674	2	217601897	217609978	8082	1177,19	0,33	5,79E-01
ENSRNOG00000019176	**Kcne1l**	KCNE1-like (Kcne1l), mRNA [Source:	681190	X	112033905	112034336	432	830,26	-0,43	5,80E-01
ENSRNOG00000011195	**Prkra**	protein kinase, interferon inducible dou	311130	3	70062246	70081079	18834	1056,74	-0,23	5,80E-01
ENSRNOG00000003635	**Disp1**	dispatched homolog 1 (Drosophila) (D	289338	13	106384791	106527113	142323	381,38	0,30	5,80E-01
ENSRNOG00000032834	**Hspa13**	heat shock protein 70 family, member	29734	11	17802532	17816830	14299	603,52	-0,29	5,80E-01
ENSRNOG00000048497		Uncharacterized protein [Source:UniProtKB/TrE		1	28910368	29015405	105038	126,09	-0,35	5,80E-01
ENSRNOG00000024402		Protein Focad [Source:UniProtKB/TrE	313346	5	110426894	110709662	282769	1455,14	0,25	5,80E-01
ENSRNOG00000001680				11	46196256	46212064	15809	79,98	-0,46	5,80E-01
ENSRNOG00000024818	**Eva1b**	eva-1 homolog B (Eva1b), mRNA [Sou	362597	5	147928855	147930156	1302	26,56	-0,50	5,80E-01
ENSRNOG00000022153		transmembrane protein 134 (Tmem13	361695	1	226244711	226250208	5498	1440,19	0,42	5,80E-01
ENSRNOG00000011719	**Ngb**	neuroglobin (Ngb), mRNA [Source:Re	85382	6	120412242	120417308	5067	63,24	-0,44	5,80E-01
ENSRNOG00000012284	**Khdrbs2**	KH domain containing, RNA binding, s	170843	9	39209750	39679399	469650	201,75	-0,38	5,80E-01
ENSRNOG00000019120	**Hmgcs2**	3-hydroxy-3-methylglutaryl-CoA synth	24450	2	219928551	219954859	26309	416,52	-0,52	5,80E-01
ENSRNOG00000020188		Short transient receptor potential chan	64573	1	173347642	173381696	34055	348,42	0,32	5,80E-01
ENSRNOG00000031635		family with sequence similarity 47, me	686140	14	16905176	16933066	27891	28,11	-0,49	5,80E-01
ENSRNOG00000049221	**Kdm4b**	lysine (K)-specific demethylase 4B (K	301128	9	9647772	9725845	78074	3269,50	0,24	5,80E-01
ENSRNOG00000000534		TBC1 domain family, member 22B [Source:MGI		20	10434808	10477828	43021	1217,52	0,25	5,81E-01
ENSRNOG00000021250	**Rnf24**	ring finger protein 24 (Rnf24), mRNA [	362218	3	130344343	130345306	964	147,25	-0,30	5,81E-01
ENSRNOG00000028991	**Kcnb2**	potassium voltage gated channel, Sha	117105	5	2969360	2971917	2558	117,37	-0,37	5,81E-01
ENSRNOG00000004033		Protein Sema6a [Source:UniProtKB/T	361324	18	40975360	41030839	55480	2335,66	-0,31	5,81E-01
ENSRNOG00000025222	**Pcid2**	PCI domain containing 2 (Pcid2), mRN	361182	16	81242949	81268414	25466	911,48	0,32	5,81E-01
ENSRNOG00000019193	**Stx1b**	syntaxin 1B (Stx1b), mRNA [Source:R	24923	1	206274272	206293050	18779	5076,16	-0,23	5,81E-01
ENSRNOG00000013971	**Psat1**	phosphoserine aminotransferase 1 (P	293820	1	240241244	240263046	21803	2659,46	-0,40	5,81E-01
ENSRNOG00000014712	**Zfp39**	zinc finger protein 39 (Zfp39), mRNA [	303173	10	44977898	45001199	23302	140,93	-0,32	5,81E-01
ENSRNOG00000002612	**Ddx52**	DEAD (Asp-Glu-Ala-Asp) box polypep	85432	10	70864589	70887723	23135	147,83	-0,49	5,81E-01

ENSRNOG00000011458	**Zfp592**	zinc finger protein 592 (Zfp592), mRN.	293038	1	143779196	143815984	36789	1119,25	-0,20	5,81E-01
ENSRNOG00000011595	**Senp8**	SUMO/sentrin specific peptidase famil	315723	8	64321674	64335522	13849	57,90	-0,39	5,81E-01
ENSRNOG00000029418	**LOC49823**	LRRGT00144 (LOC498231), mRNA [S	498231	13	55296141	55315101	18961	253,71	0,30	5,81E-01
ENSRNOG00000003011	**Gadd45gip**	growth arrest and DNA-damage-induc	288916	19	36922599	36924670	2072	1621,22	0,36	5,82E-01
ENSRNOG00000007471	**Pigb**	phosphatidylinositol glycan anchor bio	315807	8	73373909	73397749	23841	54,35	-0,37	5,82E-01
ENSRNOG00000009629	**Car2**	carbonic anhydrase 2 (Car2), mRNA [S	54231	2	107871643	107886834	15192	170,91	-0,28	5,82E-01
ENSRNOG00000019063	**Fbxo38**	Protein Fbxo38 [Source:UniProtKB/Tr	307390	18	57054085	57092175	38091	994,36	0,29	5,82E-01
ENSRNOG00000007916	**Ptk2**	protein tyrosine kinase 2 (Ptk2), mRN/	25614	7	114371990	114525553	153564	3003,65	-0,25	5,82E-01
ENSRNOG00000000640	**Egr2**	early growth response 2 (Egr2), mRN/	114090	20	24551942	24556227	4286	59,75	-0,50	5,82E-01
ENSRNOG00000032246	**Acsm3**	acyl-CoA synthetase medium-chain fa	24763	1	196449105	196475587	26483	34,74	-0,49	5,82E-01
ENSRNOG00000008562		vacuolar protein sorting-associated pr	65081	12	40348553	40366395	17843	1034,75	0,26	5,83E-01
ENSRNOG00000011976	**Nudt7**	nudix (nucleoside diphosphate linked moiety X)-		19	57278776	57293046	14271	66,78	-0,50	5,83E-01
ENSRNOG00000014966		RIKEN cDNA 1600014C10 gene [Sou	690000	1	95670340	95676490	6151	102,56	0,36	5,83E-01
ENSRNOG00000025042	**Pde3a**	phosphodiesterase 3A, cGMP inhibite	50678	4	239659270	239921900	262631	279,22	-0,25	5,83E-01
ENSRNOG00000006787	**Dhcr24**	24-dehydrocholesterol reductase (Dhc	298298	5	130011403	130034921	23519	3436,73	0,44	5,83E-01
ENSRNOG00000025587	**Plagl1**	pleiomorphic adenoma gene-like 1 (Pl	25157	1	8857033	8894012	36980	778,22	0,41	5,83E-01
ENSRNOG00000012937	**Wwp2**	WW domain containing E3 ubiquitin pr	291999	19	50405611	50476926	71316	525,68	0,20	5,83E-01
ENSRNOG00000018747	**Elmo2**	engulfment and cell motility 2 (Elmo2),	362271	3	167997379	168185837	188459	1912,50	0,23	5,83E-01
ENSRNOG00000021145	**Gpr137**	G protein-coupled receptor 137 (Gpr1:	689984	1	229185120	229188106	2987	1668,26	0,28	5,83E-01
ENSRNOG00000026091	**Slc10a4**	solute carrier family 10, member 4 (Slc	305309	14	37575344	37580564	5221	191,92	-0,45	5,83E-01
ENSRNOG00000031643	**Dchs1**	dachsous 1 (Drosophila) (Dchs1), mR	308912	1	177600834	177620711	19878	5426,52	0,27	5,83E-01
ENSRNOG00000014975	**Zfp483**	zinc finger protein 483 (Zfp483), mRN.	170955	5	80236726	80247403	10678	181,51	0,34	5,83E-01
ENSRNOG00000042920		taste receptor, type 1, member 2 (Tas1	641315	5	161806691	161874412	67722	1236,92	0,25	5,84E-01
ENSRNOG00000025512	**Mettl17**	Protein Mettl17 [Source:UniProtKB/Tr	305845	15	32100248	32106730	6483	309,37	0,25	5,84E-01
ENSRNOG00000050414	**RGD1305704**			1	236363026	236363653	628	59,45	-0,47	5,84E-01
ENSRNOG00000003524	**Lrrc59**	leucine rich repeat containing 59 (Lrrc	287633	10	82171007	82185664	14658	5992,14	0,22	5,84E-01
ENSRNOG00000012196	**Asah2**	N-acylsphingosine amidohydrolase (no	114104	1	257793172	257864321	71150	21,90	-0,47	5,84E-01
ENSRNOG00000008523	**Faf1**	Fas (TNFRSF6) associated factor 1 (F	140657	5	133203185	133570250	367066	1689,55	0,32	5,85E-01
ENSRNOG00000008825	**Ssrp1**	structure specific recognition protein 1	81785	3	78958627	78968273	9647	5447,48	0,20	5,85E-01
ENSRNOG00000020519	**Olfm2**	olfactomedin 2 (Olfm2), mRNA [Sourc	313783	8	21740369	21799373	59005	3893,57	-0,26	5,85E-01
ENSRNOG00000013911	**Nagk**	N-acetylglucosamine kinase (Nagk), m	297393	4	180063236	180070557	7322	617,25	0,20	5,85E-01
ENSRNOG00000037710	**Calr4**	Protein Calr4 [Source:UniProtKB/TrEI	689537	5	132705288	132739231	33944	36,11	-0,49	5,85E-01
ENSRNOG00000008459	**Anapc13**	anaphase promoting complex subunit	685029	8	110505168	110513584	8417	633,96	-0,36	5,85E-01
ENSRNOG00000015860	**Gipr**	gastric inhibitory polypeptide receptor	25024	1	81329941	81340229	10289	139,35	-0,35	5,85E-01
ENSRNOG00000018390	**Pld3**	phospholipase D family, member 3 (Pl	361527	1	85556094	85567834	11741	6215,89	0,30	5,85E-01
ENSRNOG00000020985	**Atp4a**	ATPase, H+/K+ exchanging, alpha pol	24216	1	90317604	90330740	13137	32,77	-0,39	5,85E-01
ENSRNOG00000028557		3-hydroxyisobutyryl-CoA hydrolase (H	301384	9	53112588	53193104	80517	273,76	-0,34	5,85E-01
ENSRNOG00000017742	**Syf2**	SYF2 homolog, RNA splicing factor (S	170933	5	157038590	157046520	7931	948,13	0,20	5,85E-01

ENSRNOG00000018975	**Atg9a**	autophagy related 9A (Atg9a), mRNA	363254	9	82152061	82162643	10583	3879,18	0,25	5,85E-01
ENSRNOG00000036856				5	177039402	177040246	845	21,91	0,46	5,85E-01
ENSRNOG00000001058	**Timm44**	translocase of inner mitochondrial mer	29635	12	4669953	4686653	16701	1071,71	0,32	5,86E-01
ENSRNOG00000001348	**Erp29**	endoplasmic reticulum protein 29 (Erp	117030	12	42486494	42492998	6505	2447,33	0,20	5,86E-01
ENSRNOG00000007398	**Zfp691**	zinc finger protein 691 (Zfp691), mRN.	313548	5	142027715	142032377	4663	187,14	0,34	5,86E-01
ENSRNOG00000005698	**Ndufa5**	NADH dehydrogenase (ubiquinone) 1	25488	4	51368669	51377027	8359	1650,71	0,21	5,86E-01
ENSRNOG00000049198	**LOC68481**	Histone H3 [Source:UniProtKB/TrEMI	310678	17	45648645	45649595	951	22,65	0,48	5,86E-01
ENSRNOG00000008108	**Ubr7**	ubiquitin protein ligase E3 component	314399	6	135842580	135862537	19958	1700,61	-0,35	5,86E-01
ENSRNOG00000014887	**Homez**	homeobox and leucine zipper encodin	260325	15	37397308	37413881	16574	149,17	-0,28	5,87E-01
ENSRNOG00000004339	**Yy1**	YY1 transcription factor (Yy1), mRNA	24919	6	141872657	141896924	24268	1161,37	0,26	5,87E-01
ENSRNOG00000024852	**Tmx4**	thioredoxin-related transmembrane pr	296182	3	134016679	134057338	40660	4385,22	-0,37	5,87E-01
ENSRNOG00000003984	**Apln**	apelin (Apln), mRNA [Source:RefSeq	58812	X	134930886	134938569	7684	135,79	-0,46	5,87E-01
ENSRNOG00000012951	**Kctd9**	potassium channel tetramerization do	364410	15	47158099	47185440	27342	133,95	-0,29	5,87E-01
ENSRNOG00000000800	**Man1a1**	mannosidase, alpha, class 1A, membe	294410	20	37015823	37203609	187787	433,78	0,39	5,88E-01
ENSRNOG00000012603	**Sestd1**	SEC14 and spectrin domains 1 (Sestd	295678	3	70613829	70664787	50959	968,00	0,24	5,88E-01
ENSRNOG00000002813	**Dars2**	aspartyl-tRNA synthetase 2 (mitochon	304919	13	83752704	83780466	27763	284,39	-0,32	5,88E-01
ENSRNOG00000048371	**Fam185a**	family with sequence similarity 185, m	499979	4	10262501	10265769	3269	154,13	0,46	5,89E-01
ENSRNOG00000010649	**Ctnnd2**	catenin (cadherin-associated protein),	114028	2	103069802	103662662	592861	4885,34	-0,19	5,89E-01
ENSRNOG00000034078	**Mxi1**	MAX interactor 1 (Mxi1), mRNA [Sourc	25701	1	281463334	281503728	40395	1779,66	-0,40	5,89E-01
ENSRNOG00000034071	**Chmp4bl1**	chromatin modifying protein 4B-like 1	679886	4	66867607	66868281	675	900,93	0,23	5,89E-01
ENSRNOG00000013176	**Far1**	fatty acyl CoA reductase 1 (Far1), mR	293173	1	185332184	185377699	45516	288,70	-0,45	5,89E-01
ENSRNOG00000010060	**Panx1**	Pannexin 1 (Panx1), transcript variant	315435	8	13507955	13547151	39197	1773,06	0,30	5,89E-01
ENSRNOG00000003300	**Btg2**	BTG family, member 2 (Btg2), mRNA [	29619	13	55966299	55970058	3760	1743,70	0,36	5,89E-01
ENSRNOG00000006460	**Amdhd2**	amidohydrolase domain containing 2 (	302972	10	13344252	13352859	8608	543,68	0,35	5,89E-01
ENSRNOG00000020881	**Frmd8**	FERM domain containing 8 (Frmd8), r	309172	1	228146287	228166935	20649	2442,56	0,27	5,89E-01
ENSRNOG00000012060	**Gucy1b3**	guanylate cyclase 1, soluble, beta 3 (	25202	2	200384722	200433787	49066	1918,69	-0,32	5,89E-01
ENSRNOG00000021133	**Lsm14a**	LSM14A, SCD6 homolog A (S. cerevis	361554	1	91343533	91388619	45087	1739,10	0,18	5,90E-01
ENSRNOG00000022521	**RGD15596**	nitric oxide-inducible gene protein [Sc	499204	1	163919411	163943710	24300	39,59	0,49	5,90E-01
ENSRNOG00000050790		DDB1 and CUL4 associated factor 12	682812	5	62188735	62205330	16596	906,64	0,24	5,90E-01
ENSRNOG00000004041		inositol polyphosphate 5-phosphatase	287533	10	64209551	64228728	19178	592,84	-0,31	5,91E-01
ENSRNOG00000010941	**Tifa**	TRAF-interacting protein with forkhead	310877	2	251473156	251481591	8436	57,67	-0,45	5,91E-01
ENSRNOG00000012862	**Spsb4**	splA/ryanodine receptor domain and S	300950	8	104287499	104359356	71858	462,47	-0,33	5,91E-01
ENSRNOG00000026982	**LOC69192**	RIKEN cDNA 1190007I07 gene [Sourc	691921	7	27340344	27343437	3094	140,26	0,36	5,91E-01
ENSRNOG00000001163	**Srsf9**	serine/arginine-rich splicing factor 9 (S	288701	12	48833032	48839338	6307	739,57	0,31	5,91E-01
ENSRNOG00000005375	**RGD15640**	uncharacterized protein LOC500390 is	500390	5	11756424	12289040	532617	307,06	-0,34	5,91E-01
ENSRNOG00000019620	**Pmf1**	polyamine-modulated factor 1 (Pmf1),	681050	2	207154781	207174337	19557	329,11	0,40	5,91E-01
ENSRNOG00000004145	**Prorsd1**	prolyl-tRNA synthetase associated do	289864	14	113345111	113606671	261561	24,57	-0,48	5,92E-01
ENSRNOG00000020009	**Npas4**	neuronal PAS domain protein 4 (Npas	266734	1	227192031	227196795	4765	53,24	-0,51	5,92E-01

ENSRNOG00000003551	**B4galt3**	UDP-Gal:betaGlcNAc beta 1,4-galacto	494342	13	94270746	94276732	5987	1619,04	0,22	5,93E-01
ENSRNOG00000005049		T-box brain protein 1 [Source:RefSeq	680427	3	54349276	54356654	7379	1013,24	0,32	5,93E-01
ENSRNOG00000006534	**Crbn**	cereblon (Crbn), mRNA [Source:RefSe	297498	4	203337149	203355944	18796	413,14	-0,35	5,93E-01
ENSRNOG00000011070		guanine nucleotide-binding protein sul	294962	2	138634564	138658837	24274	1216,25	-0,31	5,93E-01
ENSRNOG00000018803	**Mkl1**	Protein Mkl1 [Source:UniProtKB/TrEM	315151	7	122218547	122313356	94810	814,29	0,26	5,93E-01
ENSRNOG00000028350	**Arse**	arylsulfatase E (chondrodysplasia pun	310326	2	142502844	142510517	7674	485,43	0,25	5,93E-01
ENSRNOG00000028584	**Alkbh2**	alkB, alkylation repair homolog 2 (E. c	304578	12	50039937	50044571	4635	203,56	0,30	5,93E-01
ENSRNOG00000048686	**LOC30174**	Protein LOC100360856 [Source:UniP	501223	9	7388103	7501715	113613	129,88	-0,30	5,93E-01
ENSRNOG00000015303	**Fbll1**	fibrillarin-like 1 (Fbll1), mRNA [Source:	363563	10	20494823	20495797	975	1861,94	0,31	5,93E-01
ENSRNOG00000000798	**Gnl1**	guanine nucleotide binding protein-like	309593	20	5388710	5397660	8951	2596,60	0,25	5,93E-01
ENSRNOG00000010733	**Vps11**	vacuolar protein sorting 11 homolog (S	315600	8	47296463	47311266	14804	2119,98	0,31	5,93E-01
ENSRNOG00000007622	**Arrdc1**	arrestin domain containing 1 (Arrdc1),	366001	3	2112659	2119716	7058	505,03	0,28	5,93E-01
ENSRNOG00000006952	**Prex1**	phosphatidylinositol-3,4,5-trisphospha	311647	3	169494331	169575983	81653	4119,60	-0,40	5,93E-01
ENSRNOG00000021447	**Prr7**	proline rich 7 (synaptic) (Prr7), mRNA	498704	17	11803152	11804276	1125	987,62	0,34	5,93E-01
ENSRNOG00000002835	**Luc7l3**	LUC7-like 3 (S. cerevisiae) (Luc7l3), n	360602	10	81823960	81859932	35973	1360,97	-0,24	5,93E-01
ENSRNOG00000037715		cDNA sequence BC024978 [Source:MGI Symbo		1	85220207	85226857	6651	140,85	-0,27	5,93E-01
ENSRNOG00000019575	**Rell2**	RELT-like 2 (Rell2), mRNA [Source:Re	361313	18	30783005	30786576	3572	442,16	0,41	5,94E-01
ENSRNOG00000011855		Uncharacterized protein [Source:UniProtKB/TrE		1	21819548	21820021	474	41,56	0,35	5,94E-01
ENSRNOG00000015179	**Tradd**	TNFRSF1A-associated via death dom	246756	19	48081092	48086780	5689	72,05	0,40	5,94E-01
ENSRNOG00000016327	**Mcee**	methylmalonyl CoA epimerase (Mcee)	293829	1	126338537	126361719	23183	441,32	-0,38	5,94E-01
ENSRNOG00000008945	**Nans**	N-acetylneuraminic acid synthase (Na	298071	5	66637340	66654050	16711	1920,71	0,19	5,94E-01
ENSRNOG00000023035		small integral membrane protein 8 (Sn	297971	5	54799587	54808380	8794	192,90	-0,24	5,94E-01
ENSRNOG00000009723	**Ptpmt1**	protein tyrosine phosphatase, mitocho	29390	3	86444081	86452749	8669	762,27	0,22	5,95E-01
ENSRNOG00000018477	**Otud4**	OTU domain containing 4 (Otud4), mF	307774	19	42803062	42846155	43094	1012,10	0,34	5,95E-01
ENSRNOG00000028105	**Hars**	histidyl-tRNA synthetase (Hars), mRN	307492	18	29315354	29332458	17105	2276,88	0,28	5,95E-01
ENSRNOG00000003975	**Pfn1**	profilin 1 (Pfn1), mRNA [Source:RefSe	64303	10	57018596	57021336	2741	5477,91	0,35	5,95E-01
ENSRNOG00000014816	**Slc1a1**	solute carrier family 1 (neuronal/epithe	25550	1	254195390	254275293	79904	1417,29	-0,29	5,95E-01
ENSRNOG00000015434	**Midn**	midnolin (Midn), mRNA [Source:RefSe	314623	7	12585958	12592656	6699	3793,03	0,34	5,95E-01
ENSRNOG00000038160	**Eif2b5**	eukaryotic translation initiation factor 2	192234	11	87151639	87161625	9987	1864,28	0,25	5,95E-01
ENSRNOG00000024455	**Gnptg**	N-acetylglucosamine-1-phosphate tra	287134	10	14410651	14415566	4916	1666,58	0,22	5,95E-01
ENSRNOG00000010846	**Ccdc22**	coiled-coil domain containing 22 (Ccd	317381	X	16534513	16546460	11948	483,13	0,22	5,95E-01
ENSRNOG00000008830		Protein Nfe2l1; RCG32665 [Source:UniProtKB/		10	84475360	84489905	14546	2570,22	0,28	5,96E-01
ENSRNOG00000010588		Protein Tenc1 [Source:UniProtKB/TrE	315326	7	141503820	141517146	13327	303,36	-0,50	5,96E-01
ENSRNOG00000013992	**Arfrp1**	ADP-ribosylation factor related protein	117051	3	180569335	180575252	5918	807,45	0,24	5,96E-01
ENSRNOG00000014037	**Dcun1d3**	DCN1, defective in cullin neddylation 1	309035	1	196562335	196600712	38378	136,77	0,34	5,96E-01
ENSRNOG00000017975	**Dnajb13**	DnaJ (Hsp40) homolog, subfamily B, r	308857	1	171716972	171731341	14370	141,98	-0,38	5,96E-01
ENSRNOG00000019509	**Fbxl15**	F-box and leucine-rich repeat protein 1	309453	1	273506723	273508964	2242	545,36	0,38	5,96E-01
ENSRNOG00000026644	**Glipr1**	GLI pathogenesis-related 1 (Glipr1), m	299783	7	54792116	54803129	11014	20,85	-0,44	5,96E-01

ENSRNOG00000026905	**Ppm1g**	protein phosphatase, Mg2+/Mn2+ dep	259229	6	36336359	36355553	19195	3825,31	0,26	5,96E-01
ENSRNOG00000037462	**Eml6**	Protein Eml6 [Source:UniProtKB/TrEMBL;Acc:D		14	113857590	114150219	292630	187,19	0,37	5,96E-01
ENSRNOG00000038597	**Dlg1**	discs, large homolog 1 (Drosophila) (D	25252	11	75239783	75454358	214576	707,06	-0,21	5,96E-01
ENSRNOG00000000931		Splicing factor, suppressor of white-apricot homo		12	32675288	32745272	69985	1584,52	0,30	5,96E-01
ENSRNOG00000001242	**Gstt3**	glutathione S-transferase, theta 3 (Gst	499422	20	16007776	16015160	7385	925,91	-0,42	5,96E-01
ENSRNOG00000004784	**Ndufaf5**	NADH dehydrogenase (ubiquinone) co	296190	3	139688332	139717715	29384	582,31	0,27	5,96E-01
ENSRNOG00000006200	**St18**	suppression of tumorigenicity 18 (St18	266680	5	17213494	17337689	124196	71,11	-0,40	5,96E-01
ENSRNOG00000006937	**Scn3b**	sodium channel, voltage-gated, type II	245956	8	61379752	61402266	22515	3216,89	-0,32	5,96E-01
ENSRNOG00000009689	**Api5**	apoptosis inhibitor 5 (Api5), mRNA [So	362170	3	90010501	90035577	25077	2088,09	-0,31	5,96E-01
ENSRNOG00000010624	**Ap3b1**	adaptor-related protein complex 3, bet	309969	2	43195234	43395094	199861	823,43	-0,28	5,96E-01
ENSRNOG00000012084	**Xpnpep1**	X-prolyl aminopeptidase (aminopeptid	170751	1	281121567	281171495	49929	4288,22	0,22	5,96E-01
ENSRNOG00000016687	**Ssc5d**	scavenger receptor cysteine rich doma	308341	1	76074530	76092319	17790	339,00	-0,45	5,96E-01
ENSRNOG00000017941		optineurin [Source:RefSeq peptide;Ac	246294	17	78833450	78884466	51017	694,08	-0,33	5,96E-01
ENSRNOG00000018818	**Txnl1**	thioredoxin-like 1 (Txnl1), mRNA [Sou	140922	18	58276629	58304098	27470	2810,05	-0,17	5,96E-01
ENSRNOG00000018824	**Slc7a5**	solute carrier family 7 (amino acid tran	50719	19	65412970	65441647	28678	8319,53	-0,34	5,96E-01
ENSRNOG00000020235		heterogeneous nuclear ribonucleoprot	80846	1	88176711	88187394	10684	9532,77	0,23	5,96E-01
ENSRNOG00000023589	**Gpr107**	G protein-coupled receptor 107 (Gpr1(	311857	3	15992527	16053874	61348	3515,78	0,38	5,96E-01
ENSRNOG00000028896	**LOC10091**	alpha-2-macroglobulin (A2m), mRNA [	24153	4	221393211	221442944	49734	25,78	-0,51	5,96E-01
ENSRNOG00000030515		neurofascin (Nfasc), transcript variant	116690	13	54410797	54597345	186549	6168,61	0,25	5,96E-01
ENSRNOG00000039415		FCH and double SH3 domains 1 (Fchs	307482	18	30784855	30797074	12220	860,76	0,30	5,96E-01
ENSRNOG00000043060	**Dnajc4**	DnaJ (Hsp40) homolog, subfamily C, r	361717	1	229233670	229237835	4166	343,29	0,30	5,96E-01
ENSRNOG00000030049	**Znhit6**	zinc finger, HIT-type containing 6 (Znh	292160	2	269981414	270012984	31571	782,10	0,32	5,96E-01
ENSRNOG00000019667	**Ppfibp2**	PTPRF interacting protein, binding pro	308918	1	178818985	178964741	145757	194,86	-0,47	5,97E-01
ENSRNOG00000017172		multivesicular body subunit 12B (Mvb1	362118	3	18111129	18275223	164095	7070,93	0,18	5,97E-01
ENSRNOG00000011627		Zinc finger RNA-binding protein [Sour	365703	2	82476890	82539304	62415	4539,63	-0,31	5,98E-01
ENSRNOG00000037707	**Armcx6**	armadillo repeat containing, X-linked 6	363496	X	105455045	105457641	2597	339,62	0,39	5,98E-01
ENSRNOG00000038883	**LOC29415**	DNA segment, Chr 17, Wayne State U	294154	20	9533345	9599034	65690	4273,25	0,18	5,98E-01
ENSRNOG00000045952		Eph receptor B4 [Source:MGI Symbol	686310	12	24408833	24432402	23570	234,58	0,29	5,98E-01
ENSRNOG00000003469	**MAST1**	microtubule associated serine/threonir	353118	19	37001666	37029385	27720	3302,15	0,27	5,98E-01
ENSRNOG00000008728	**Unc79**	unc-79 homolog (C. elegans) (Unc79)	314401	6	136066167	136268033	201867	2000,26	-0,21	5,98E-01
ENSRNOG00000010625	**Dnmt3b**	DNA (cytosine-5-)-methyltransferase 3	444985	3	155524090	155549356	25267	64,13	0,36	5,98E-01
ENSRNOG00000016910		Ral GEF with PH domain and SH3 bin	1E+08	3	17517035	17713947	196913	854,68	-0,21	5,98E-01
ENSRNOG00000020344		syntrophin, beta 2 [Source:RefSeq pe	689421	19	49990515	50072998	82484	53,44	-0,40	5,98E-01
ENSRNOG00000049676	**St6galnac5**	ST6 (alpha-N-acetyl-neuraminyl-2,3-be	365984	2	276690144	276717148	27005	374,62	-0,35	5,98E-01
ENSRNOG00000049771	**Gstt1**	glutathione S-transferase theta 1 (Gstt	25260	20	15989083	16006507	17425	54,65	-0,46	5,98E-01
ENSRNOG00000028113	**Whamm**	WAS protein homolog associated with	293057	1	144345711	144374081	28371	191,73	0,26	5,98E-01
ENSRNOG00000006130	**Rab5b**	RAB5B, member RAS oncogene famil	288779	7	3077243	3095997	18755	8526,41	0,31	5,98E-01
ENSRNOG00000032700	**Zbtb34**	Protein Zbtb34 [Source:UniProtKB/Tr	689174	3	17785513	17800154	14642	349,76	-0,25	5,99E-01

ENSRNOG00000048924	**Islr**	immunoglobulin superfamily containin	686539	8	62723487	62724773	1287	32,36	-0,49	5,99E-01
ENSRNOG00000003512	**Gabra1**	gamma-aminobutyric acid (GABA) A re	29705	10	27154359	27210126	55768	639,38	0,39	5,99E-01
ENSRNOG00000004208	**Crim1**	cysteine rich transmembrane BMP reg	298744	6	780568	952528	171961	1531,40	0,33	5,99E-01
ENSRNOG00000019850		SPEG complex locus (Speg), transcrip	363256	9	82340606	82397945	57340	1539,01	-0,24	5,99E-01
ENSRNOG00000008005	**Akna**	AT-hook transcription factor (Akna), m	362530	5	83298322	83336921	38600	674,90	-0,39	6,00E-01
ENSRNOG00000017463	**Bloc1s3**	Protein Bloc1s3 [Source:UniProtKB/T	680476	1	81681707	81682294	588	365,91	0,40	6,00E-01
ENSRNOG00000024501	**Rgs3**	regulator of G-protein signaling 3 (Rgs	54293	5	82606811	82690341	83531	717,36	-0,32	6,00E-01
ENSRNOG00000025823	**Rbm46**	RNA binding motif protein 46 (Rbm46)	310548	2	201245864	201288843	42980	42,53	-0,45	6,00E-01
ENSRNOG00000048506	**Inpp5b**	inositol polyphosphate-5-phosphatase	362590	5	146503106	146566949	63844	192,21	-0,32	6,00E-01
ENSRNOG00000004169	**Fzr1**	fizzy/cell division cycle 20 related 1 (D	314642	7	11368581	11380504	11924	1941,81	0,29	6,01E-01
ENSRNOG00000011296		centromere protein N (Cenpn), mRNA	361416	19	60132303	60270697	138395	163,82	0,35	6,01E-01
ENSRNOG00000009437		Uncharacterized protein [Source:UniProtKB/TrE		14	85999931	86028404	28474	23,08	0,42	6,01E-01
ENSRNOG00000001080	**Arl6ip4**	ADP-ribosylation-like factor 6 interacti	288656	12	39776742	39778884	2143	967,40	-0,32	6,01E-01
ENSRNOG00000001294	**Ift81**	intraflagellar transport 81 homolog (Ch	373066	12	41307847	41387638	79792	528,10	-0,36	6,01E-01
ENSRNOG00000004839	**B4galnt1**	beta-1,4-N-acetyl-galactosaminyl trans	64828	7	70630282	70637196	6915	1210,13	0,36	6,01E-01
ENSRNOG00000014661	**Zfp385d**	zinc finger protein 385D (Zfp385d), mR	305691	15	10266313	10653490	387178	120,43	-0,32	6,01E-01
ENSRNOG00000021092	**Scnm1**	sodium channel modifier 1 (Scnm1), m	310662	2	215794553	215798715	4163	240,81	0,23	6,01E-01
ENSRNOG00000000593	**Rev3l**	REV3-like, polymerase (DNA directed)	309812	20	46526299	46683995	157697	3563,47	0,37	6,02E-01
ENSRNOG00000039807	**Fam193b**	family with sequence similarity 193, m	498703	17	11705724	11737149	31426	1058,37	0,25	6,02E-01
ENSRNOG00000050318		fibronectin type III and SPRY domain-c	301506	9	10055588	10065298	9711	1283,13	0,24	6,02E-01
ENSRNOG00000020299	**Klc2**	Kinesin light chain 2 (Predicted), isofo	309159	1	227423230	227433026	9797	3612,65	0,34	6,02E-01
ENSRNOG00000012815	**Chst10**	carbohydrate sulfotransferase 10 (Chs	140568	9	45222143	45252322	30180	2344,06	0,29	6,03E-01
ENSRNOG00000015232	**Pip5k1b**	phosphatidylinositol-4-phosphate 5-kir	309419	1	249441815	249714561	272747	198,42	-0,28	6,03E-01
ENSRNOG00000009280	**Mkrn1**	makorin ring finger protein 1 (Mkrn1),	296988	4	66994932	67013389	18458	2139,08	0,31	6,03E-01
ENSRNOG00000010137	**Xpo4**	exportin 4 (Xpo4), mRNA [Source:Ref	290280	15	41680885	41771826	90942	420,50	-0,40	6,03E-01
ENSRNOG00000031396	**Grb14**	growth factor receptor bound protein 1	58844	3	57578232	57695025	116794	657,36	-0,29	6,03E-01
ENSRNOG00000050578	**Rheb**	Ras homolog enriched in brain (Rheb)	26954	4	6843843	6889004	45162	3856,41	0,31	6,03E-01
ENSRNOG00000009570	**Pdxp**	pyridoxal (pyridoxine, vitamin B6) phos	727679	7	120132151	120137599	5449	3279,33	0,33	6,03E-01
ENSRNOG00000005135	**RGD15621**	similar to chromosome X open reading	501559	X	38604610	38662385	57776	28,23	-0,45	6,03E-01
ENSRNOG00000005270	**Sycp3**	synaptonemal complex protein 3 (Syc	25561	7	29136815	29151274	14460	29,58	-0,50	6,03E-01
ENSRNOG00000012947	**Metap1**	methionyl aminopeptidase 1 (Metap1)	295500	2	262279056	262312345	33290	2124,71	0,33	6,03E-01
ENSRNOG00000018610	**Pde6d**	phosphodiesterase 6D, cGMP-specific	363272	9	93290037	93334964	44928	1521,14	0,21	6,03E-01
ENSRNOG00000010147	**Pdcd10**	programmed cell death 10 (Pdcd10), r	494345	2	193305005	193348014	43010	1366,33	-0,25	6,04E-01
ENSRNOG00000049505	**Nup43**	nucleoporin 43 (Nup43), mRNA [Sourc	683983	1	3468475	3478295	9821	255,75	0,22	6,04E-01
ENSRNOG00000008270	**Cntrob**	centrobin, centrosomal BRCA2 interac	303240	10	55644329	55666322	21994	224,03	0,27	6,04E-01
ENSRNOG00000003248	**Mpzl1**	myelin protein zero-like 1 (Mpzl1), mR	360871	13	88685412	88722184	36773	3085,50	0,31	6,04E-01
ENSRNOG00000011645	**Rrnad1**	ribosomal RNA adenine dimethylase d	361976	2	206697126	206705708	8583	311,04	0,33	6,04E-01
ENSRNOG00000017473	**Ttc25**	tetratricopeptide repeat domain 25 (Tt	303534	10	88252737	88281484	28748	39,36	-0,49	6,04E-01

ENSRNOG00000027935	**Lrrc34**	leucine rich repeat containing 34 (Lrrc	499589	2	136066289	136085758	19470	22,26	-0,50	6,04E-01
ENSRNOG00000047256		Uncharacterized protein [Source:UniProtKB/TrE		15	103739948	103770499	30552	97,42	-0,32	6,04E-01
ENSRNOG00000001828	**Stk38l**	serine/threonine kinase 38 like (Stk38l	691337	4	245208688	245245025	36338	472,84	0,27	6,04E-01
ENSRNOG00000004923	**Map4k5**	mitogen-activated protein kinase kinas	503027	6	101586288	101678374	92087	970,71	-0,22	6,04E-01
ENSRNOG00000005927	**Cpsf6**	cleavage and polyadenylation specific	299811	7	60387688	60418950	31263	4293,83	-0,33	6,04E-01
ENSRNOG00000013798	**Fnbp1l**	formin binding protein 1-like (Fnbp1l),	310839	2	246053113	246104570	51458	3337,45	-0,30	6,04E-01
ENSRNOG00000023127	**Pelo**	pelota homolog (Drosophila) (Pelo), m	294754	2	71808496	71810350	1855	622,72	0,19	6,04E-01
ENSRNOG00000010607	**Rnf208**	ring finger protein 208 (Rnf208), mRN	499748	3	2443879	2445203	1325	1380,67	0,27	6,04E-01
ENSRNOG00000013397	**Foxo1**	forkhead box O1 (Foxo1), mRNA [Sou	84482	2	161140308	161215605	75298	398,63	-0,31	6,04E-01
ENSRNOG00000019213	**Gpd1**	glycerol-3-phosphate dehydrogenase	60666	X	115875188	115882576	7389	121,22	-0,42	6,04E-01
ENSRNOG00000039476	**Pcdhb2**	protocadherin beta 2 (Pcdhb2), mRNA	498843	18	30083426	30085822	2397	118,02	-0,36	6,04E-01
ENSRNOG00000008307	**Nanp**	N-acetylneuraminic acid phosphatase	311530	3	153160645	153173615	12971	302,25	-0,23	6,05E-01
ENSRNOG00000008752	**Nrf1**	nuclear respiratory factor 1 (Nrf1), mR	312195	4	57139899	57198685	58787	373,87	0,22	6,05E-01
ENSRNOG00000048969		nucleolar protein with MIF4G domain 1 [Source:I		4	2392805	2408386	15582	708,75	0,27	6,05E-01
ENSRNOG00000012960	**Uap1l1**	UDP-N-acteylglucosamine pyrophospl	296560	3	2560671	2565936	5266	879,41	-0,46	6,05E-01
ENSRNOG00000033881	**Fam196a**	protein FAM196A [Source:RefSeq pe	1E+08	1	214053082	214108141	55060	253,50	-0,34	6,05E-01
ENSRNOG00000030633	**Lmf2**	lipase maturation factor 2 (Lmf2), mRN	315218	7	130007321	130011771	4451	897,34	0,29	6,05E-01
ENSRNOG00000008271	**Fam91a1**	family with sequence similarity 91, me	689997	7	98904337	98942732	38396	1807,81	-0,29	6,05E-01
ENSRNOG00000010587	**Crnkl1**	crooked neck pre-mRNA splicing facto	1E+08	3	146546963	146561830	14868	61,62	-0,35	6,05E-01
ENSRNOG00000012453	**Ddx6**	DEAD (Asp-Glu-Ala-Asp) box helicase	500988	8	47543903	47576345	32443	304,28	-0,26	6,05E-01
ENSRNOG00000012808	**Mbrl**	membralin (Mbrl), mRNA [Source:Ref	299608	7	12940961	12947623	6663	2795,86	0,26	6,05E-01
ENSRNOG00000017866	**Sirt5**	sirtuin 5 (Sirt5), mRNA [Source:RefSe	306840	17	25942005	25963431	21427	1867,55	0,23	6,05E-01
ENSRNOG00000018850	**Ttc9b**	tetratricopeptide repeat domain 9B (Tt	361528	1	85683938	85686120	2183	1660,08	0,36	6,05E-01
ENSRNOG00000020811	**Il6r**	interleukin 6 receptor (Il6r), mRNA [So	24499	2	208629066	208677779	48714	88,90	-0,46	6,05E-01
ENSRNOG00000024043	**Orc6**	origin recognition complex, subunit 6 (	291927	19	38423345	38430600	7256	352,67	0,29	6,05E-01
ENSRNOG00000049223	**Vipas39**	VPS33B interacting protein, apical-bas	681989	6	120553710	120577928	24219	1833,32	0,31	6,05E-01
ENSRNOG00000000169	**Spata5l1**	spermatogenesis associated 5-like 1 (	691729	3	121285518	121298517	13000	322,78	0,24	6,05E-01
ENSRNOG00000001174	**Pde9a**	phosphodiesterase 9A (Pde9a), mRN	191569	20	12328642	12405854	77213	287,07	-0,30	6,05E-01
ENSRNOG00000001686	**Hlcs**	Protein Hlcs [Source:UniProtKB/TrEM	288240	11	37947328	38086632	139305	191,45	0,33	6,05E-01
ENSRNOG00000009275	**Kpnb1**	karyopherin (importin) beta 1 (Kpnb1),	24917	10	84886482	84914608	28127	5669,07	0,23	6,05E-01
ENSRNOG00000009535	**Stoml2**	stomatin (Epb7.2)-like 2 (Stoml2), mR	298203	5	62998100	63001725	3626	1632,13	0,17	6,05E-01
ENSRNOG00000021390	**Meis3**	Meis homeobox 3 (Meis3), mRNA [So	361514	1	79335447	79346210	10764	1111,19	0,23	6,05E-01
ENSRNOG00000024945	**Dusp18**	dual specificity phosphatase 18 (Dusp	305477	14	84834651	84839036	4386	1059,04	-0,18	6,05E-01
ENSRNOG00000011663	**Rnf7**	ring finger protein 7 (Rnf7), mRNA [So	300948	8	103774031	103783041	9011	1142,01	0,38	6,05E-01
ENSRNOG00000019497	**Mrpl17**	mitochondrial ribosomal protein L17 (Mrpl17), m		1	177657945	177659630	1686	1904,00	0,18	6,05E-01
ENSRNOG00000006375	**LOC68306**	voltage-dependent anion channel 1 (V	83529	10	37498352	37525818	27467	1895,49	0,27	6,06E-01
ENSRNOG00000007710	**Usp20**	ubiquitin specific peptidase 20 (Usp20	311856	3	15181259	15214732	33474	2657,31	0,19	6,06E-01
ENSRNOG00000008786	**Ap1b1**	adaptor-related protein complex 1, bet	29663	14	85927663	85959251	31589	3295,91	0,23	6,06E-01

ENSRNOG00000014213	**Thap1**	THAP domain containing, apoptosis a	306547	16	70327540	70332022	4483	211,39	0,23	6,06E-01
ENSRNOG00000016394	**LOC10091**	WD repeat domain 77 (Wdr77), mRNA	310769	2	227998604	228008465	9862	48,57	0,33	6,06E-01
ENSRNOG00000017704	**Sema3f**	sema domain, immunoglobulin domain	315996	8	115795106	115824684	29579	454,87	0,23	6,06E-01
ENSRNOG00000020284	**Prkar2a**	protein kinase, cAMP dependent regul	29699	8	116830969	116890072	59104	1712,08	0,27	6,06E-01
ENSRNOG00000022039	**Zbtb40**	zinc finger and BTB domain containing	362635	5	159109614	159144251	34638	272,46	0,28	6,06E-01
ENSRNOG00000028756	**Ubc**	ubiquitin C (Ubc), mRNA [Source:RefS	50522	12	38515328	38520061	4734	1704,55	0,25	6,06E-01
ENSRNOG00000042929	**LOC10036**	predicted gene 7694 [Source:MGI Syr	1E+08	13	93262801	93267937	5137	39,92	-0,37	6,06E-01
ENSRNOG00000003122	**LOC10091**	histone deacetylase 8 (Hdac8), mRNA	363481	X	73005663	73211936	206274	281,72	0,36	6,06E-01
ENSRNOG00000011562	**Slitrk2**	SLIT and NTRK-like family, member 2	309349	X	151098816	151102256	3441	433,41	-0,27	6,06E-01
ENSRNOG00000018687	**Fbxw2**	F-box and WD repeat domain containi	311881	3	19191086	19216407	25322	2880,75	0,37	6,06E-01
ENSRNOG00000031266	**Siae**	sialic acid acetylesterase (Siae), mRN	363045	8	40079093	40114338	35246	414,65	0,40	6,06E-01
ENSRNOG00000004072	**Myo1c**	myosin IC (Myo1c), mRNA [Source:Re	65261	10	64185628	64201933	16306	710,65	0,25	6,06E-01
ENSRNOG00000005561	**Brinp1**	bone morphogenetic protein/retinoic a	140610	5	89013128	89158671	145544	1702,06	-0,23	6,06E-01
ENSRNOG00000008637	**Strada**	STE20-related kinase adaptor alpha (S	303605	10	94104054	94133270	29217	926,61	0,19	6,06E-01
ENSRNOG00000010257	**Cuedc1**	CUE domain containing 1 (Cuedc1), m	303419	10	74590109	74610568	20460	1261,85	0,33	6,06E-01
ENSRNOG00000010890	**Bmp1**	bone morphogenetic protein 1 (Bmp1)	83470	15	55889918	55934263	44346	1360,86	-0,29	6,06E-01
ENSRNOG00000012428	**Maf**	v-maf avian musculoaponeurotic fibros	54267	19	58998441	58999550	1110	348,31	0,29	6,06E-01
ENSRNOG00000012684	**Bloc1s2**	biogenesis of lysosomal organelles co	293938	1	271358891	271366128	7238	1158,68	0,25	6,06E-01
ENSRNOG00000013172	**Nr1h3**	nuclear receptor subfamily 1, group H,	58852	3	86712883	86721503	8621	21,83	-0,50	6,06E-01
ENSRNOG00000016210	**Micalcl**	MICAL C-terminal like (Micalcl), mRNA	293180	1	184172145	184211083	38939	24,16	-0,49	6,06E-01
ENSRNOG00000018077	**Agpat6**	1-acylglycerol-3-phosphate O-acyltran	290843	16	73249356	73282217	32862	3364,61	0,28	6,06E-01
ENSRNOG00000025274		beta-hexosaminidase subunit beta pre	294673	2	47094815	47114918	20104	1253,12	-0,39	6,06E-01
ENSRNOG00000029242	**Plekho2**	Protein Plekho2 [Source:UniProtKB/T	315764	8	70781065	70804767	23703	145,13	0,30	6,06E-01
ENSRNOG00000029956	**Zfp654**	Protein Zfp654 [Source:UniProtKB/Tr	288354	11	1815761	1817473	1713	92,04	-0,44	6,06E-01
ENSRNOG00000043141	**Ap3s2**	adaptor-related protein complex 3, sig	683402	1	142574435	142615225	40791	1185,40	0,18	6,06E-01
ENSRNOG00000000779	**Znrd1**	zinc ribbon domain containing 1 (Znrd	361784	20	4125232	4129150	3919	515,31	0,26	6,07E-01
ENSRNOG00000002519	**Magt1**	magnesium transporter 1 (Magt1), mR	116967	X	56145990	56184209	38220	196,93	-0,41	6,07E-01
ENSRNOG00000006285	**Tlk2**	tousled-like kinase 2 (Tlk2), mRNA [So	303592	10	93173881	93265759	91879	1211,62	-0,20	6,07E-01
ENSRNOG00000014916	**Lats1**	large tumor suppressor kinase 1 (Lats	308265	1	3492544	3510720	18177	1076,98	-0,21	6,07E-01
ENSRNOG00000018557	**Cdh22**	cadherin 22 (Cdh22), mRNA [Source:F	29182	3	168055444	168153121	97678	310,13	0,27	6,07E-01
ENSRNOG00000022877	**Poldip3**	polymerase (DNA-directed), delta inte	315170	7	123974866	124003513	28648	4176,60	0,25	6,07E-01
ENSRNOG00000003723	**Wdr26**	WD repeat domain 26 (Wdr26), mRNA	498301	13	104488124	104526797	38674	3903,59	-0,19	6,07E-01
ENSRNOG00000004476		Wnt inhibitory factor 1 [Source:UniPro	114557	7	63077688	63147645	69958	437,07	-0,37	6,07E-01
ENSRNOG00000004751	**Jkamp**	JNK1/MAPK8-associated membrane p	299127	6	104280504	104294443	13940	1156,52	0,41	6,07E-01
ENSRNOG00000019302	**Stx4**	syntaxin 4 (Stx4), mRNA [Source:RefS	81803	1	206310318	206317403	7086	595,83	-0,24	6,07E-01
ENSRNOG00000021155	**Ctsk**	cathepsin K (Ctsk), mRNA [Source:Re	29175	2	216154151	216164778	10628	22,05	-0,48	6,07E-01
ENSRNOG00000029148	**Pdgfd**	platelet derived growth factor D (Pdgfd	66018	8	4454510	4682165	227656	21,92	-0,47	6,07E-01
ENSRNOG00000045753	**LOC10091**	Histone H2B [Source:UniProtKB/TrEM	295274	2	217846757	217904831	58075	33,34	0,45	6,07E-01

ENSRNOG00000050648		Protein RGD1559979 [Source:UniPro	691658	8	72142803	72160730	17928	474,13	0,20	6,07E-01
ENSRNOG00000008533	**Ago2**	argonaute RISC catalytic component 2	59117	7	114274582	114312135	37554	584,80	0,24	6,07E-01
ENSRNOG00000012991	**Gpr124**	Protein LOC100363275; RCG43370 [	1E+08	16	68769891	68805960	36070	124,07	0,25	6,07E-01
ENSRNOG00000013446	**Nif3l1**	NIF3 NGG1 interacting factor 3-like 1 (	301431	9	65138584	65156220	17637	274,10	-0,28	6,07E-01
ENSRNOG00000021147		BCL2-associated agonist of cell death	64639	1	229189348	229198203	8856	1971,27	0,28	6,07E-01
ENSRNOG00000012274	**Ddi2**	DNA-damage inducible protein 2 (Ddi2	313668	5	163951352	163995207	43856	814,47	0,24	6,08E-01
ENSRNOG00000014847				1	184739197	184740620	1424	47,95	0,41	6,08E-01
ENSRNOG00000016029	**Rb1**	retinoblastoma 1 (Rb1), mRNA [Sourc	24708	15	58804732	58942062	137331	1670,57	-0,18	6,08E-01
ENSRNOG00000021513	**Rtn4rl2**	reticulon 4 receptor-like 2 (Rtn4rl2), m	311169	3	78793337	78809760	16424	570,33	-0,33	6,08E-01
ENSRNOG00000011057		mitofusin-1 [Source:RefSeq peptide;A	192647	2	138586943	138627370	40428	1190,01	-0,31	6,08E-01
ENSRNOG00000028156		phospholipase D1 (Pld1), mRNA [Sou	25096	2	133400609	133554180	153572	379,86	-0,39	6,08E-01
ENSRNOG00000021051	**Cyth2**	cytohesin 2 (Cyth2), mRNA [Source:R	116692	1	102875334	102882171	6838	2866,80	0,26	6,08E-01
ENSRNOG00000024625	**RGD15657**	uncharacterized protein LOC361541 [	361541	1	90149377	90161856	12480	513,03	0,23	6,08E-01
ENSRNOG00000007895	**Pdhb**	pyruvate dehydrogenase (lipoamide) b	289950	15	22507948	22513889	5942	3130,87	0,36	6,08E-01
ENSRNOG00000016971	**Zfp612**	zinc finger protein 612 (Zfp612), mRN	307839	19	52384704	52394810	10107	319,25	-0,26	6,08E-01
ENSRNOG00000037967	**Thap7**	THAP domain containing 7 (Thap7), m	287944	11	90450728	90453825	3098	422,93	0,26	6,08E-01
ENSRNOG00000001124	**Rnft2**	ring finger protein, transmembrane 2 (	304521	12	45773943	45839516	65574	2931,35	0,28	6,09E-01
ENSRNOG00000017824	**Ncoa5**	nuclear receptor coactivator 5 (Ncoa5)	296372	3	167650206	167683424	33219	2875,38	0,21	6,09E-01
ENSRNOG00000013507	**Selt**	selenoprotein T (Selt), mRNA [Source:	365802	2	168183559	168197314	13756	868,36	-0,41	6,09E-01
ENSRNOG00000032058	**Pogk**	pogo transposable element with KRAB	304941	13	89477377	89493293	15917	1881,64	-0,21	6,09E-01
ENSRNOG00000018481	**Zfp259**	zinc finger protein 259 (Zfp259), mRN	500989	8	49189009	49198583	9575	1230,01	0,30	6,09E-01
ENSRNOG00000050848	**Dtx1**	Protein LOC687424 [Source:UniProtK	687424	12	43244361	43273175	28815	1449,03	0,27	6,09E-01
ENSRNOG00000003466		IQ motif and Sec7 domain 2 (Iqsec2),	685244	X	64287088	64367249	80162	1553,47	-0,26	6,10E-01
ENSRNOG00000007504	**Actr10**	actin-related protein 10 homolog (S. ce	299121	6	102831244	102857628	26385	3543,78	0,20	6,10E-01
ENSRNOG00000007894	**Mpdz**	multiple PDZ domain protein (Mpdz), r	29365	5	103442809	103596543	153735	962,44	-0,23	6,10E-01
ENSRNOG00000042607	**LOC69013**	ras homolog gene family, member f [S	690130	12	40778915	40791466	12552	304,45	-0,29	6,10E-01
ENSRNOG00000009595	**Zbtb48**	zinc finger and BTB domain containing	362668	5	172750035	172758372	8338	282,41	0,24	6,10E-01
ENSRNOG00000021890	**Nob1**	NIN1/RPN12 binding protein 1 homolo	291996	19	49267151	49279617	12467	1753,02	0,28	6,10E-01
ENSRNOG00000028335	**Fat4**	FAT tumor suppressor homolog 4 (Dro	310341	2	145350319	145477455	127137	2040,37	-0,29	6,10E-01
ENSRNOG00000004159	**Flii**	flightless I homolog (Drosophila) (Flii),	287375	10	46728481	46742400	13920	2400,64	-0,18	6,11E-01
ENSRNOG00000014350	**Cyr61**	cysteine-rich, angiogenic inducer, 61 (	83476	2	270055954	270058912	2959	251,79	0,36	6,11E-01
ENSRNOG00000011491		Protein Dnajc13 [Source:UniProtKB/T	363127	8	112084846	112177085	92240	1540,68	0,30	6,11E-01
ENSRNOG00000013647	**Polm**	polymerase (DNA directed), mu (Polm	289757	14	86761193	86770768	9576	100,17	-0,36	6,11E-01
ENSRNOG00000020505	**Map4k1**	mitogen activated protein kinase kinas	292763	1	89114111	89135537	21427	61,70	-0,36	6,11E-01
ENSRNOG00000029598	**Robo2**	roundabout homolog 2 (Drosophila) (R	84409	11	14733231	14884133	150903	3184,82	0,36	6,11E-01
ENSRNOG00000014161	**Rbm15b**	Protein Rbm15b [Source:UniProtKB/T	315988	8	114977385	114980224	2840	1691,70	0,23	6,11E-01
ENSRNOG00000014644	**Zic1**	Zic family member 1 (Zic1), mRNA [Sc	64618	8	98226542	98229980	3439	158,27	-0,27	6,11E-01
ENSRNOG00000014957	**Tpgs2**	tubulin polyglutamylase complex subu	361301	18	17093464	17148510	55047	2006,82	0,34	6,11E-01

ENSRNOG00000016189		Protein Frmd5 [Source:UniProtKB/TrE	311362	3	120023279	120294028	270750	456,87	0,24	6,11E-01
ENSRNOG00000016897	**Rlbp1**	retinaldehyde binding protein 1 (Rlbp1	293049	1	142059883	142073463	13581	450,47	-0,39	6,11E-01
ENSRNOG00000019221	**Gstm4**	glutathione S-transferase mu 4 (Gstm4	499689	2	230302891	230308113	5223	1076,72	0,28	6,11E-01
ENSRNOG00000030625	**Srprb**	transferrin (Tf), mRNA [Source:RefSec	24825	8	111064527	111112746	48220	2049,49	0,45	6,11E-01
ENSRNOG00000037443	**Pgam5**	phosphoglycerate mutase family mem	288731	12	54202825	54210153	7329	1512,15	0,29	6,11E-01
ENSRNOG00000047439	**Pot1b**	Protein Pot1b [Source:UniProtKB/TrE	690237	9	6885290	6930692	45403	55,54	-0,39	6,11E-01
ENSRNOG00000020583	**Fcgrt**	Fc fragment of IgG, receptor, transpor	29558	1	102151857	102161645	9789	873,06	-0,46	6,11E-01
ENSRNOG00000003616	**Grem2**	gremlin 2 (Grem2), mRNA [Source:Re	289264	13	97359449	97453188	93740	449,98	-0,35	6,11E-01
ENSRNOG00000007865	**Ephb1**	Eph receptor B1 (Ephb1), mRNA [Sou	24338	8	109782848	110218328	435481	1777,84	-0,22	6,11E-01
ENSRNOG00000014588	**Dbr1**	debranching enzyme homolog 1 (S. ce	681234	8	107251356	107262731	11376	422,48	0,35	6,11E-01
ENSRNOG00000045928	**Myl6l**	myosin, light chain 6, alkali, smooth m	304447	7	2880878	2882604	1727	1181,46	0,26	6,11E-01
ENSRNOG00000033155	**Arf3**	ADP-ribosylation factor 3 (Arf3), mRN	140940	X	114922631	114927693	5063	30493,38	0,27	6,11E-01
ENSRNOG00000003359	**Ogt**	O-linked N-acetylglucosamine (GlcNA	26295	X	72390253	72435239	44987	3184,80	-0,41	6,12E-01
ENSRNOG00000006608	**Tmem70**	transmembrane protein 70 (Tmem70),	500384	5	2019520	2036983	17464	667,43	-0,25	6,12E-01
ENSRNOG00000016405	**Pcsk4**	proprotein convertase subtilisin/kexin t	171085	7	12417281	12425514	8234	40,60	-0,43	6,12E-01
ENSRNOG00000037310	**Riiad1**	regulatory subunit of type II PKA R-sul	1E+08	2	215183199	215191155	7957	82,20	-0,49	6,12E-01
ENSRNOG00000010164	**Mrps2**	mitochondrial ribosomal protein S2 (M	362094	3	12402368	12407371	5004	1703,51	-0,22	6,12E-01
ENSRNOG00000027867	**Rexo4**	REX4, RNA exonuclease 4 homolog (S	311826	3	10862784	10873113	10330	1814,85	0,21	6,12E-01
ENSRNOG00000010914	**Pigh**	phosphatidylinositol glycan anchor bio	362756	6	111632984	111642424	9441	276,21	-0,27	6,12E-01
ENSRNOG00000012735	**Kif24**	Protein Kif24 [Source:UniProtKB/TrEM	313170	5	62308159	62344056	35898	86,70	-0,39	6,12E-01
ENSRNOG00000016716		Protein Armc3 [Source:UniProtKB/TrE	1E+08	17	87632223	87725943	93721	18,70	-0,46	6,12E-01
ENSRNOG00000011062	**LOC10036**	general transcription factor IIA, 2 (Gtf2	83828	8	76640787	76653929	13143	640,18	-0,19	6,12E-01
ENSRNOG00000002104	**Scaf4**	SR-related CTD-associated factor 4 (S	245924	11	33991195	34047555	56361	1348,93	0,18	6,12E-01
ENSRNOG00000039284	**Haus4**	HAUS augmin-like complex, subunit 4	305882	15	37067372	37078759	11388	208,22	0,34	6,12E-01
ENSRNOG00000025970		UTP14, U3 small nucleolar ribonucleoprotein, hc		9	84307442	84310603	3162	137,95	-0,46	6,12E-01
ENSRNOG00000017053	**Fbxo36**	F-box protein 36 (Fbxo36), mRNA [So	363268	9	92168447	92231811	63365	29,77	-0,44	6,12E-01
ENSRNOG00000026821		Rho GTPase activating protein 42 [So	500943	8	7198985	7426476	227492	599,06	-0,39	6,12E-01
ENSRNOG00000026828	**Scfd2**	sec1 family domain containing 2 (Scfd	498353	14	36042335	36371465	329131	695,83	0,30	6,12E-01
ENSRNOG00000006582	**Aptx**	aprataxin (Aptx), mRNA [Source:RefS	259271	5	61522038	61542988	20951	497,78	0,34	6,13E-01
ENSRNOG00000007307	**Syde1**	synapse defective 1, Rho GTPase, ho	362842	7	14194129	14200115	5987	281,75	0,35	6,13E-01
ENSRNOG00000018373	**Tln2**	Protein Tln2 [Source:UniProtKB/TrEM	315776	8	77377033	77528139	151107	1558,21	-0,26	6,13E-01
ENSRNOG00000024562	**Tgfbrap1**	transforming growth factor, beta recep	301373	9	49523134	49573517	50384	1268,74	0,20	6,13E-01
ENSRNOG00000013428	**Atp6v0a4**	ATPase, H+ transporting, lysosomal V	296981	4	65554091	65635822	81732	105,95	-0,29	6,14E-01
ENSRNOG00000009063	**Dnajc15**	DnaJ (Hsp40) homolog, subfamily C, r	290370	15	63670037	63734653	64617	719,10	0,20	6,14E-01
ENSRNOG00000026636	**Urm1**	ubiquitin related modifier 1 (Urm1), mR	311840	3	13732444	13749414	16971	2261,52	0,38	6,14E-01
ENSRNOG00000027901	**Mrm1**	mitochondrial rRNA methyltransferase	363661	10	72043105	72049392	6288	336,66	-0,23	6,14E-01
ENSRNOG00000007506	**Ndufaf4**	NADH dehydrogenase (ubiquinone) cc	362495	5	43880679	43885850	5172	198,63	-0,41	6,14E-01
ENSRNOG00000046494	**LOC10036**	predicted gene, 17455 [Source:MGI S	1E+08	20	31763179	31763565	387	34,88	-0,49	6,14E-01

ENSRNOG00000009329		nuclear receptor subfamily 1, group D,	252917	10	86479868	86486598	6731	535,46	-0,29	6,14E-01
ENSRNOG00000050949	**MGC12523**	Tetratricopeptide repeat protein 39C [	686179	18	3955997	4072712	116716	546,55	-0,22	6,14E-01
ENSRNOG00000000618	**Mdga2**	MAM domain containing glycosylphos	314180	6	97931875	98773148	841274	336,63	-0,31	6,14E-01
ENSRNOG00000003763	**Prdx4**	peroxiredoxin 4 (Prdx4), mRNA [Sourc	85274	X	43801335	43816646	15312	1362,90	0,31	6,14E-01
ENSRNOG00000011871	**Pcaf**	Protein Pcaf [Source:UniProtKB/TrEM	301164	9	3479552	3527403	47852	221,58	-0,31	6,14E-01
ENSRNOG00000014232	**P2ry1**	purinergic receptor P2Y, G-protein cou	25265	2	170730012	170735513	5502	178,98	-0,28	6,14E-01
ENSRNOG00000003288	**Cacng5**	calcium channel, voltage-dependent, g	140726	10	95855859	95862665	6807	46,81	-0,46	6,15E-01
ENSRNOG00000021248	**Cdc25b**	cell division cycle 25B (Cdc25b), mRN	171103	3	130229228	130239028	9801	384,90	0,23	6,15E-01
ENSRNOG00000024428	**Kif20a**	kinesin family member 20A (Kif20a), m	361308	18	27137685	27146171	8487	700,94	0,32	6,15E-01
ENSRNOG00000033588	**Siglec15**	sialic acid binding Ig-like lectin 15 (Sig	498888	18	74076491	74082377	5887	17,39	-0,47	6,15E-01
ENSRNOG00000016148	**Gtse1**	G-2 and S-phase expressed 1 (Gtse1)	300126	7	126447562	126464313	16752	573,78	0,39	6,15E-01
ENSRNOG00000025130	**Ltk**	leukocyte receptor tyrosine kinase (Ltk	311337	3	118102135	118109011	6877	101,43	-0,36	6,15E-01
ENSRNOG00000023459	**Lurap1**	leucine rich adaptor protein 1 (Lurap1)	500527	5	138775907	138785616	9710	154,68	-0,37	6,15E-01
ENSRNOG00000010134	**Acot2**	acyl-CoA thioesterase 2 (Acot2), mRN	192272	6	118076658	118083477	6820	327,22	-0,38	6,15E-01
ENSRNOG00000020216	**Gmpr2**	guanosine monophosphate reductase	192357	15	38242891	38252027	9137	275,96	-0,28	6,15E-01
ENSRNOG00000004711	**Mta1**	metastasis associated 1 (Mta1), mRN/	64520	6	146918907	146944409	25503	2606,25	-0,30	6,15E-01
ENSRNOG00000020698	**Rnd2**	Rho family GTPase 2 (Rnd2), mRNA [	303553	10	89174534	89178109	3576	2001,94	-0,44	6,15E-01
ENSRNOG00000004423	**Zfp238**	zinc finger protein 238 (Zfp238), mRN/	64619	13	100036034	100037602	1569	4347,49	-0,30	6,15E-01
ENSRNOG00000013866	**Usp49**	ubiquitin specific peptidase 49 (Usp49	316211	9	14241623	14299667	58045	445,07	-0,30	6,15E-01
ENSRNOG00000020975	**Mrpl49**	mitochondrial ribosomal protein L49 (M	309176	1	228351856	228355562	3707	2269,68	0,35	6,15E-01
ENSRNOG00000031593	**Nanos3**	nanos homolog 3 (Drosophila) (Nanos	288909	19	36262009	36265435	3427	31,07	0,37	6,15E-01
ENSRNOG00000010812	**Osbpl6**	oxysterol binding protein-like 6 (Osbpl	311129	3	69952408	70024414	72007	676,72	-0,22	6,16E-01
ENSRNOG00000013201	**Tex264**	testis expressed 264 (Tex264), mRNA	300988	8	114720831	114748300	27470	2751,96	0,29	6,16E-01
ENSRNOG00000015212	**Hspa14**	heat shock protein 14 (Hspa14), mRN	307133	17	80379450	80400359	20910	778,90	-0,25	6,16E-01
ENSRNOG00000019601	**Mapk3**	mitogen activated protein kinase 3 (Ma	50689	1	205172641	205178843	6203	3319,82	0,20	6,16E-01
ENSRNOG00000005642	**Frs2**	fibroblast growth factor receptor subst	314850	7	60128597	60210278	81682	1801,90	-0,18	6,16E-01
ENSRNOG00000022402	**Luzp1**	leucine zipper protein 1 (Luzp1), mRN	79428	5	158664309	158673014	8706	936,89	0,26	6,16E-01
ENSRNOG00000023812	**Raver2**	ribonucleoprotein, PTB-binding 2 (Rav	362551	5	123786082	123863622	77541	534,49	-0,21	6,16E-01
ENSRNOG00000025443	**Map1lc3a**	microtubule-associated protein 1 light	362245	3	157169534	157171180	1647	6208,27	0,31	6,16E-01
ENSRNOG00000015059	**Mcfd2**	multiple coagulation factor deficiency 2	246117	6	20877155	20887267	10113	235,17	0,41	6,16E-01
ENSRNOG00000001709	**Ap2m1**	adaptor-related protein complex 2, mu	116563	11	87113489	87122152	8664	21984,06	0,25	6,17E-01
ENSRNOG00000008173	**Sesn3**	sestrin 3 (Sesn3), mRNA [Source:RefS	315427	8	12768653	12818203	49551	2127,62	-0,33	6,17E-01
ENSRNOG00000008569	**Ndufa6**	NADH dehydrogenase (ubiquinone) 1	315167	7	123567781	123571638	3858	2365,14	0,30	6,17E-01
ENSRNOG00000009184	**Foxp1**	forkhead box P1 (Foxp1), mRNA [Sou	297480	4	195858738	196089931	231194	533,19	-0,30	6,17E-01
ENSRNOG00000009360	**Sh3bp1**	SH3-domain binding protein 1 (Sh3bp	300067	7	120117015	120129416	12402	97,44	-0,42	6,17E-01
ENSRNOG00000014021	**Matn4**	matrilin 4 (Matn4), mRNA [Source:Ref	296358	3	167018918	167033936	15019	163,66	-0,42	6,17E-01
ENSRNOG00000016810	**RGD15653**	stathmin 1 (Stmn1), mRNA [Source:Re	29332	5	156466729	156472404	5676	19282,08	0,29	6,17E-01
ENSRNOG00000017747	**Snrnp27**	small nuclear ribonucleoprotein 27 (U4	362392	4	183046290	183057647	11358	1032,09	-0,22	6,17E-01

ENSRNOG00000007428	**Ypel4**	yippee-like 4 (Drosophila) (Ypel4), mR	502643	3	78655402	78659863	4462	284,08	0,46	6,17E-01
ENSRNOG00000000827	**Ier3**	immediate early response 3 (Ier3), mF	294235	20	5535617	5536820	1204	115,07	0,40	6,17E-01
ENSRNOG00000009599	**Rwdd2a**	RWD domain containing 2A (Rwdd2a)	363110	8	93756515	93759639	3125	69,59	-0,30	6,17E-01
ENSRNOG00000009724	**Tstd2**	thiosulfate sulfurtransferase (rhodanes	362514	5	66227997	66250472	22476	537,00	0,28	6,17E-01
ENSRNOG00000015418	**Klhl14**	kelch-like family member 14 (Klhl14), i	364823	18	12314920	12428674	113755	67,16	-0,35	6,17E-01
ENSRNOG00000016288	**Tcea2**	transcription elongation factor A (SII), :	29575	3	180896673	180904401	7729	1169,36	0,26	6,17E-01
ENSRNOG00000033794	**Sppl2b**	signal peptide peptidase-like 2B (Sppl:	362828	7	11879708	11892558	12851	2129,03	0,26	6,17E-01
ENSRNOG00000049336	**Lime1**	Lck interacting transmembrane adaptc	362289	3	180592159	180594482	2324	330,40	0,29	6,17E-01
ENSRNOG00000025742	**Lmnb2**	Protein Lmnb2 [Source:UniProtKB/TrI	299625	7	11828422	11844454	16033	1515,81	0,26	6,17E-01
ENSRNOG00000002095		Rho GTPase activating protein 24 (Arl	305156	14	8355352	8668071	312720	85,88	-0,32	6,17E-01
ENSRNOG00000001793		Protein Heg1 [Source:UniProtKB/TrEI	689710	11	73327373	73410395	83023	470,13	-0,33	6,17E-01
ENSRNOG00000006763	**Rbm18**	RNA binding motif protein 18 (Rbm18)	311902	3	20753255	20773093	19839	1556,20	-0,21	6,17E-01
ENSRNOG00000008604	**Nsfl1c**	NSFL1 (p97) cofactor (p47) (Nsfl1c), r	83809	3	153337781	153361981	24201	4248,71	0,30	6,17E-01
ENSRNOG00000012332		RAD51 homolog B [Source:MGI Symt	500679	6	115162984	115706613	543630	23,25	0,44	6,17E-01
ENSRNOG00000045931	**Sik3**	SIK family kinase 3 (Sik3), mRNA [Sou	684112	8	48934911	49144449	209539	1983,20	0,17	6,17E-01
ENSRNOG00000050197	**Pdia6**	protein disulfide isomerase family A, m	286906	6	52289504	52306885	17382	4516,40	0,21	6,17E-01
ENSRNOG00000002575	**Sap30l**	Protein Sap30l [Source:UniProtKB/TrI	360531	10	43023625	43030967	7343	595,63	0,30	6,17E-01
ENSRNOG00000011653	**Fam21c**	family with sequence similarity 21, me	297530	4	214084983	214143362	58380	3545,07	0,17	6,17E-01
ENSRNOG00000013194	**Rps6ka2**	ribosomal protein S6 kinase polypeptic	117269	1	54609625	54747731	138107	1558,10	-0,24	6,17E-01
ENSRNOG00000007418	**Ipo9**	importin 9 (Ipo9), mRNA [Source:RefS	304817	13	57258618	57305942	47325	3701,09	0,19	6,17E-01
ENSRNOG00000031523		RIKEN cDNA 1600029I14 gene [Sourc	315947	8	107125065	107142310	17246	18,73	-0,48	6,17E-01
ENSRNOG00000011424	**Cldn23**	claudin 23 (Cldn23), mRNA [Source:R	290789	16	59664248	59665971	1724	25,50	-0,45	6,18E-01
ENSRNOG00000014909	**Sh2d5**	Protein Sh2d5; RCG31450 [Source:U	366489	5	160369031	160374322	5292	73,99	-0,39	6,18E-01
ENSRNOG00000019346	**Tmco3**	transmembrane and coiled-coil domaii	306607	16	81037226	81075420	38195	535,82	0,35	6,18E-01
ENSRNOG00000029043	**Zfp84**	zinc finger protein 84 (Zfp84), mRNA [	308482	1	88824173	88839114	14942	471,43	-0,27	6,18E-01
ENSRNOG00000032917	**Zfand2a**	zinc finger, AN1-type domain 2A (Zfan	360772	12	19239057	19250441	11385	1983,37	0,29	6,18E-01
ENSRNOG00000048278		Uncharacterized protein [Source:UniProtKB/TrE		2	204775500	204783175	7676	138,80	-0,39	6,18E-01
ENSRNOG00000024111	**Cage1**	cancer antigen 1 (Cage1), mRNA [Sou	306872	17	29366867	29405080	38214	30,33	-0,41	6,18E-01
ENSRNOG00000017503		peroxisome proliferator-activated rece	291567	18	55857450	55959449	102000	64,79	-0,36	6,18E-01
ENSRNOG00000000500	**Scube3**	signal peptide, CUB domain, EGF-like	294297	20	9931818	9950181	18364	73,86	-0,29	6,19E-01
ENSRNOG00000019277		voltage-dependent anion channel 3 (V	83532	16	73924417	73940558	16142	4999,59	0,21	6,19E-01
ENSRNOG00000027271	**RGD13592**	Ribosomal_L22 domain containing prc	360649	10	95377290	95392627	15338	20,33	0,48	6,19E-01
ENSRNOG00000030983	**B3galt5**	UDP-Gal:betaGlcNAc beta 1,3-galactc	288161	11	40163670	40211572	47903	172,68	-0,38	6,19E-01
ENSRNOG00000032908		3-ketoacyl-CoA thiolase A, peroxisomi	24157	8	127234654	127242997	8344	1369,14	0,30	6,19E-01
ENSRNOG00000036842	**Smug1**	single-strand-selective monofunctiona	315344	7	142562646	142564771	2126	407,98	0,30	6,19E-01
ENSRNOG00000010771	**Pkd1**	polycystic kidney disease 1 homolog (	24650	10	13732199	13779017	46819	3931,45	0,26	6,19E-01
ENSRNOG00000022857	**Wdr92**	WD repeat domain 92 (Wdr92), mRNA	498418	14	100145268	100166279	21012	300,93	0,30	6,19E-01
ENSRNOG00000025791	**Dync1li2**	dynein, cytoplasmic 1 light intermediat	81655	19	739197	762114	22918	4551,43	-0,24	6,19E-01

ENSRNOG00000007992	**Srsf10**	serine/arginine-rich splicing factor 10 (	362630	5	157970240	157978639	8400	847,37	-0,19	6,19E-01
ENSRNOG00000008163	**Atp6v1g1**	ATPase, H transporting, lysosomal V1	298103	5	83481867	83488047	6181	6271,95	0,23	6,19E-01
ENSRNOG00000009711	**Hepacam2**	HEPACAM family member 2 (Hepacar	296846	4	28306779	28340882	34104	21,42	0,46	6,19E-01
ENSRNOG00000019653	**Csrnp2**	cysteine-serine-rich nuclear protein 2 (	315308	7	139923806	139939644	15839	1974,11	0,30	6,19E-01
ENSRNOG00000036686		anaphase-promoting complex subunit	498030	10	109367977	109376450	8474	2642,61	0,40	6,19E-01
ENSRNOG00000046496	**Gpr98**	Protein Gpr98 [Source:UniProtKB/TrE	1E+08	2	9258876	9289365	30490	80,43	-0,33	6,19E-01
ENSRNOG00000030520	**Sarnp**	SAP domain containing ribonucleoprot	362819	7	3217790	3271833	54044	573,65	0,29	6,20E-01
ENSRNOG00000004160	**LOC10091(**	phosphoribosyl pyrophosphate synthe	24689	X	28831387	28867879	36493	537,25	-0,26	6,20E-01
ENSRNOG00000011697		zinc finger protein 827 [Source:MGI Sy	291940	19	43282075	43457175	175101	681,22	0,23	6,20E-01
ENSRNOG00000014420	**Rap2b**	RAP2B, member of RAS oncogene fa	170923	2	171097071	171097622	552	384,80	0,24	6,20E-01
ENSRNOG00000015590	**Dnaaf1**	dynein, axonemal, assembly factor 1 (	361419	19	62973796	62994143	20348	46,55	-0,46	6,20E-01
ENSRNOG00000047905	**Pp3111**	PP3111 protein (Pp3111), mRNA [Sou	246185	10	109563527	109570585	7059	487,28	0,25	6,20E-01
ENSRNOG00000047880		Uncharacterized protein [Source:UniProtKB/TrE		12	20063825	20071699	7875	65,83	0,30	6,20E-01
ENSRNOG00000004148	**Cdk17**	cyclin-dependent kinase 17 (Cdk17), r	314743	7	34066669	34144779	78111	2926,13	-0,27	6,20E-01
ENSRNOG00000006939	**Ndufa7**	NADH dehydrogenase (ubiquinone) 1	299643	7	18861110	18873924	12815	2668,48	0,42	6,20E-01
ENSRNOG00000010299	**Eps15**	epidermal growth factor receptor pathv	313474	5	132763093	132861698	98606	1616,28	-0,34	6,20E-01
ENSRNOG00000010849	**Adprhl2**	ADP-ribosylhydrolase like 2 (Adprhl2),	362600	5	148103569	148108843	5275	2469,70	0,28	6,20E-01
ENSRNOG00000013933	**Med27**	mediator complex subunit 27 (Med27)	296612	3	13233711	13407329	173619	638,22	0,27	6,20E-01
ENSRNOG00000017408	**Fam160a2**	family with sequence similarity 160, m	293343	1	177223332	177245718	22387	1155,90	0,21	6,20E-01
ENSRNOG00000039494		aminoadipate-semialdehyde synthase [Source:M		4	49938570	49985323	46754	28,21	-0,40	6,20E-01
ENSRNOG00000004090				14	92831927	92832988	1062	33,21	0,37	6,20E-01
ENSRNOG00000030019	**Atp1a1**	ATPase, Na+/K+ transporting, alpha 1	24211	2	223440514	223469880	29367	7289,38	-0,18	6,21E-01
ENSRNOG00000007167	**Fhit**	fragile histidine triad (Fhit), mRNA [So	60398	15	20143551	20818336	674786	84,12	0,34	6,21E-01
ENSRNOG00000007508	**Lrtm2**	leucine-rich repeats and transmembra	680883	4	217290883	217305501	14619	293,05	-0,25	6,21E-01
ENSRNOG00000028699	**Sco1**	SCO1 cytochrome c oxidase assembly	497930	10	53346887	53359468	12582	404,80	0,29	6,21E-01
ENSRNOG00000005428		C-terminal binding protein 1 (Ctbp1), r	29382	14	83447906	83475121	27216	6208,70	-0,27	6,21E-01
ENSRNOG00000024012	**Pex10**	peroxisomal biogenesis factor 10 (Pex	680424	5	175925857	175931023	5167	361,59	0,21	6,21E-01
ENSRNOG00000011307	**Slmap**	sarcolemma associated protein (Slma	290533	16	2089451	2201800	112350	1281,06	0,25	6,21E-01
ENSRNOG00000011885	**Rhpn2**	rhophilin, Rho GTPase binding protein	308516	1	92723895	92784983	61089	234,80	-0,43	6,21E-01
ENSRNOG00000019249	**Ap3b2**	Protein Ap3b2 [Source:UniProtKB/TrE	308777	1	144226633	144257516	30884	5096,47	0,29	6,21E-01
ENSRNOG00000024620		Protein Mamdc2 [Source:UniProtKB/TrEMBL;A(		1	248356247	248440758	84512	91,48	-0,43	6,21E-01
ENSRNOG00000011387	**Tet3**	Protein Tet3 [Source:UniProtKB/TrEM	680576	4	179656564	179696869	40306	1419,70	0,20	6,21E-01
ENSRNOG00000017510		milk fat globule-EGF factor 8 protein (I	25277	1	141814240	141829651	15412	10633,66	-0,32	6,21E-01
ENSRNOG00000033658	**Kntc1**	kinetochore associated 1 (Kntc1), mRI	304477	12	40072618	40146547	73930	357,32	0,34	6,21E-01
ENSRNOG00000005355	**Ncstn**	nicastrin (Ncstn), mRNA [Source:RefS	289231	13	94974017	94990220	16204	3143,87	0,27	6,22E-01
ENSRNOG00000021589	**RGD15619**	Protein RGD1561931 [Source:UniPro	302396	X	75752491	75758257	5767	926,49	-0,47	6,22E-01
ENSRNOG00000026605	**Ifi27l2b**	interferon, alpha-inducible protein 27 l	299269	6	136556836	136558324	1489	365,72	0,40	6,22E-01
ENSRNOG00000000850	**Apom**	apolipoprotein M (Apom), mRNA [Sou	55939	20	7193732	7196332	2601	25,50	0,40	6,22E-01

ENSRNOG00000019080	**Hsd3b7**	hydroxy-delta-5-steroid dehydrogenas	246211	1	206270898	206274168	3271	214,74	-0,38	6,22E-01
ENSRNOG00000006877	**Efnb1**	ephrin B1 (Efnb1), mRNA [Source:Ref	25186	X	69764314	69777120	12807	1999,50	0,16	6,22E-01
ENSRNOG00000012830	**Paqr8**	progestin and adipoQ receptor family i	316275	9	25890194	25907174	16981	719,61	-0,32	6,22E-01
ENSRNOG00000024420	**Eme1**	essential meiotic structure-specific enc	287634	10	82185900	82191905	6006	38,42	0,42	6,22E-01
ENSRNOG00000048397	**Sephs2**	selenophosphate synthetase 2 (Sephs	308993	1	205712722	205714893	2172	1645,47	0,26	6,22E-01
ENSRNOG00000002292	**Hnrpd**	heterogeneous nuclear ribonucleoprot	79256	14	11200291	11218601	18311	6434,18	0,21	6,22E-01
ENSRNOG00000003614	**Mgat5**	mannosyl (alpha-1,6-)-glycoprotein be	65271	13	48946178	49250496	304319	859,21	-0,25	6,22E-01
ENSRNOG00000007869	**Wscd1**	WSC domain containing 1 (Wscd1), m	287466	10	58084069	58112877	28809	2683,17	-0,29	6,22E-01
ENSRNOG00000012266	**Zcchc17**	zinc finger, CCHC domain containing 1	500555	5	152252915	152294518	41604	1539,60	0,37	6,22E-01
ENSRNOG00000013874	**Rplp1**	ribosomal protein, large, P1 (Rplp1), n	140661	8	66594523	66595860	1338	6879,93	-0,43	6,22E-01
ENSRNOG00000015280	**Actr8**	ARP8 actin-related protein 8 homolog	361107	16	5979233	5993600	14368	1163,37	0,24	6,22E-01
ENSRNOG00000016214	**Agl**	amylo-alpha-1, 6-glucosidase, 4-alpha	362029	2	237376059	237430811	54753	1153,85	-0,31	6,22E-01
ENSRNOG00000018931	**Dis3l2**	DIS3 mitotic control homolog (S. cerev	367307	9	93387701	93886547	498847	792,02	0,27	6,22E-01
ENSRNOG00000019688		diaphanous homolog 1 (Drosophila) [S	307483	18	30665581	30734650	69070	1109,43	0,20	6,22E-01
ENSRNOG00000019807	**Sufu**	suppressor of fused homolog (Drosopl	361769	1	273574759	273669937	95179	394,08	0,21	6,22E-01
ENSRNOG00000021729	**Iqub**	IQ motif and ubiquitin domain containi	296936	4	51258446	51365176	106731	58,11	-0,40	6,22E-01
ENSRNOG00000022533	**Micall2**	Protein Micall2 [Source:UniProtKB/Tr	288515	12	18978841	19007460	28620	52,24	-0,41	6,22E-01
ENSRNOG00000042464		F-box protein 25 [Source:MGI Symbol;Acc:MGI:		16	80338195	80364514	26320	1826,43	0,28	6,22E-01
ENSRNOG00000049532				3	17086122	17086203	82	21,91	0,41	6,22E-01
ENSRNOG00000015277		small EDRK-rich factor 2 [Source:Ref	502663	3	119955927	119957588	1662	5150,29	0,38	6,22E-01
ENSRNOG00000002918	**FAM187A**	family with sequence similarity 187, m	287741	10	90761120	90762587	1468	20,92	-0,48	6,22E-01
ENSRNOG00000020373	**Dap3**	death associated protein 3 (Dap3), mF	295238	2	207624785	207652339	27555	1625,26	0,27	6,22E-01
ENSRNOG00000015857	**Ctsa**	cathepsin A (Ctsa), mRNA [Source:Re	296370	3	167484246	167489974	5729	3148,98	0,31	6,23E-01
ENSRNOG00000017604	**Fam65a**	family with sequence similarity 65, me	291974	19	48445032	48452707	7676	2483,28	-0,23	6,23E-01
ENSRNOG00000017871	**Sidt2**	SID1 transmembrane family, member	315617	8	48854808	48871125	16318	3012,49	-0,20	6,23E-01
ENSRNOG00000019024	**Trim67**	tripartite motif-containing 67 (Trim67),	307938	19	68269785	68305962	36178	766,68	-0,30	6,23E-01
ENSRNOG00000019838	**Gmfg**	glia maturation factor, gamma (Gmfg),	113940	1	86528123	86533107	4985	66,75	-0,36	6,23E-01
ENSRNOG00000023803		Protein Cmya5 [Source:UniProtKB/Tr	688915	2	41845822	41908429	62608	25,44	0,45	6,23E-01
ENSRNOG00000013431	**Rsbn1l**	round spermatid basic protein 1-like (F	311987	4	10741003	10801400	60398	351,34	0,30	6,23E-01
ENSRNOG00000002001	**Itsn1**	intersectin 1 (SH3 domain protein) (Its	29491	11	35547331	35635193	87863	2374,38	-0,20	6,23E-01
ENSRNOG00000020416		zinc finger RNA binding protein 2 [Source:MGI S		7	11503082	11521102	18021	777,23	0,22	6,23E-01
ENSRNOG00000024352	**Tyw1**	tRNA-yW synthesizing protein 1 homo	304423	12	31761916	31856112	94197	672,18	0,27	6,23E-01
ENSRNOG00000012073	**Abhd14b**	abhydrolase domain containing 14b (A	300983	8	114514600	114518645	4046	202,60	-0,42	6,23E-01
ENSRNOG00000038572		non-SMC condensin I complex, subun	305392	14	69941735	69962624	20890	167,99	-0,46	6,23E-01
ENSRNOG00000004258	**Chst7**	carbohydrate (N-acetylglucosamine 6-	302302	X	3413406	3449646	36241	557,88	-0,31	6,24E-01
ENSRNOG00000004585		transmembrane and TPR repeat-conta	299762	7	47197649	47603261	405613	285,32	-0,27	6,24E-01
ENSRNOG00000010964	**Akap13**	A kinase (PRKA) anchor protein 13 (A	293024	1	138008286	138312523	304238	629,99	-0,19	6,24E-01
ENSRNOG00000015416	**Nabp1**	nucleic acid binding protein 1 (Nabp1)	363227	9	54750053	54757517	7465	97,21	-0,36	6,24E-01

ENSRNOG00000038001	**Slc25a1**	solute carrier family 25 (mitochondrial	29743	11	90295813	90298828	3016	2227,94	0,33	6,24E-01
ENSRNOG00000001618	**Ripk4**	receptor-interacting serine-threonine k	304053	11	41762131	41784398	22268	77,42	0,35	6,24E-01
ENSRNOG00000003174	**Tapt1**	Protein Tapt1 [Source:UniProtKB/TrEI	305386	14	71467973	71492220	24248	544,02	-0,29	6,24E-01
ENSRNOG00000004471	**Polr3h**	polymerase (RNA) III (DNA directed) p	300088	7	123121661	123131987	10327	1040,70	0,27	6,24E-01
ENSRNOG00000005468	**Rbm28**	RNA binding motif protein 28 (Rbm28)	312182	4	56166274	56204840	38567	789,95	0,31	6,24E-01
ENSRNOG00000007393	**Ndrg1**	N-myc downstream regulated 1 (Ndrg	299923	7	107683925	107725300	41376	2954,27	-0,29	6,24E-01
ENSRNOG00000025317	**Gpatch11**	G patch domain containing 11 (Gpatch	362685	6	1401794	1411973	10180	582,10	0,27	6,24E-01
ENSRNOG00000027263	**RGD15625**	Protein RGD1562550 [Source:UniPro	499532	2	70726147	70729089	2943	34,55	-0,38	6,24E-01
ENSRNOG00000028956		methyl-CpG-binding domain protein 3	362834	7	12349402	12356298	6897	2284,11	0,32	6,24E-01
ENSRNOG00000029885	**Stag2**	stromal antigen 2 (Stag2), mRNA [Sou	313304	X	128583874	128712851	128978	824,07	-0,48	6,24E-01
ENSRNOG00000047230	**LOC68296**	zinc fingr protein 551 [Source:MGI Syr	682964	1	67682619	67689655	7037	79,69	-0,35	6,24E-01
ENSRNOG00000007599	**Afap1**	actin filament associated protein 1 (Afa	140935	14	79558760	79622351	63592	3310,82	0,23	6,25E-01
ENSRNOG00000029015	**LOC10090**	Fas apoptotic inhibitory molecule 2 (Fa	246274	X	115663276	115689093	25818	322,25	-0,31	6,25E-01
ENSRNOG00000011568	**Rspo3**	R-spondin 3 (Rspo3), mRNA [Source:I	498997	1	32115843	32198978	83136	143,53	-0,29	6,25E-01
ENSRNOG00000016316	**Mcm2**	minichromosome maintenance comple	312538	4	186066380	186080792	14413	1280,18	0,21	6,25E-01
ENSRNOG00000021087	**Lgi4**	leucine-rich repeat LGI family, membe	361549	1	90647194	90656994	9801	92,89	-0,45	6,25E-01
ENSRNOG00000008174	**Appl2**	adaptor protein, phosphotyrosine inter	362860	7	26387152	26436562	49411	1008,56	-0,42	6,25E-01
ENSRNOG00000021373	**Cox14**	Cytochrome c oxidase assembly prote	681219	X	115883242	115885365	2124	2261,63	0,36	6,25E-01
ENSRNOG00000023085	**Pmel**	premelanosome protein (Pmel), mRN/	362818	7	3105334	3116614	11281	144,47	-0,47	6,25E-01
ENSRNOG00000001547	**Agps**	alkylglycerone phosphate synthase (A	84114	3	69193349	69241491	48143	375,32	-0,31	6,25E-01
ENSRNOG00000016970	**Tcf3**	transcription factor 3 (Tcf3), transcript	171046	7	12316323	12338454	22132	2136,32	0,31	6,25E-01
ENSRNOG00000010821	**Ankrd54**	ankyrin repeat domain 54 (Ankrd54), r	362957	7	120275642	120286573	10932	553,84	0,26	6,25E-01
ENSRNOG00000008214	**Fbxo9**	f-box protein 9 (Fbxo9), mRNA [Sourc	300849	8	84953190	84978347	25158	1333,24	0,35	6,25E-01
ENSRNOG00000024433	**Fbxl7**	F-box and leucine-rich repeat protein 7	361907	2	98744396	98993396	249001	90,13	0,27	6,25E-01
ENSRNOG00000000477	**Daxx**	death-domain associated protein (Dax	140926	20	7538716	7544462	5747	1106,48	0,19	6,25E-01
ENSRNOG00000007379	**Iah1**	isoamyl acetate-hydrolyzing esterase	298917	6	60260794	60268037	7244	1597,99	0,28	6,25E-01
ENSRNOG00000013805	**Tnip2**	TNFAIP3 interacting protein 2 (Tnip2),	305451	14	82175496	82192493	16998	89,66	-0,38	6,25E-01
ENSRNOG00000014083	**Iqsec3**	IQ motif and Sec7 domain 3 (Iqsec3),	404781	4	221035990	221132460	96471	2267,86	0,28	6,25E-01
ENSRNOG00000012891	**Mdh1b**	malate dehydrogenase 1B, NAD (solu	316444	9	72123074	72169151	46078	20,94	-0,43	6,25E-01
ENSRNOG00000016097	**Ccbl1**	cysteine conjugate-beta lyase, cytopla	311844	3	14104787	14118929	14143	729,68	0,27	6,25E-01
ENSRNOG00000018884	**Ttc13**	tetratricopeptide repeat domain 13 (Tt	292095	19	68135050	68189367	54318	1865,49	0,33	6,25E-01
ENSRNOG00000020679	**Icam1**	intercellular adhesion molecule 1 (Icar	25464	8	22092135	22103927	11793	137,74	0,41	6,25E-01
ENSRNOG00000025373	**Polk**	polymerase (DNA directed) kappa (Po	171525	2	46418252	46477563	59312	61,12	-0,33	6,25E-01
ENSRNOG00000049722	**LOC10036**	tetratricopeptide repeat domain 30A1 (	311123	3	69348272	69350266	1995	33,54	-0,41	6,25E-01
ENSRNOG00000002975	**RGD15636**	Protein RGD1563606 [Source:UniPro	367749	X	17728416	17729024	609	20,10	0,40	6,25E-01
ENSRNOG00000004290	**Grb10**	growth factor receptor bound protein 1	498416	14	91818383	91914861	96479	840,81	-0,29	6,25E-01
ENSRNOG00000005093	**Lgr6**	Protein Lgr6 [Source:UniProtKB/TrEM	498233	13	56866583	56982331	115749	30,78	-0,40	6,25E-01
ENSRNOG00000008036		DENN/MADD domain containing 4C [Source:MG		5	109101564	109164418	62855	318,77	-0,39	6,25E-01

ENSRNOG00000008471	**Kif21b**	kinesin family member 21B (Kif21b), n	289397	13	58030724	58076237	45514	7603,75	-0,27	6,25E-01
ENSRNOG00000011156	**Clstn3**	calsyntenin 3 (Clstn3), mRNA [Source	171393	4	224064529	224097591	33063	6517,16	0,35	6,25E-01
ENSRNOG00000012146	**Pomt2**	protein-O-mannosyltransferase 2 (Por	688673	6	120423138	120461156	38019	939,54	0,24	6,25E-01
ENSRNOG00000013451		drosha, ribonuclease type III [Source:I	310159	2	81692861	81796037	103177	2267,04	-0,17	6,25E-01
ENSRNOG00000014628	**Pomk**	protein kinase-like protein SgK196 (Sg	306549	16	70523618	70533991	10374	1494,26	0,24	6,25E-01
ENSRNOG00000015063	**Dhodh**	dihydroorotate dehydrogenase (quinor	65156	19	52891893	52905966	14074	353,95	0,28	6,25E-01
ENSRNOG00000028111	**Zfyve26**	zinc finger, FYVE domain containing 2	314265	6	115096555	115158874	62320	946,51	-0,25	6,25E-01
ENSRNOG00000037295	**Poc1a**	POC1 centriolar protein homolog A (Cl	501048	8	114351523	114399635	48113	161,56	0,23	6,25E-01
ENSRNOG00000042777		zinc finger protein 28 [Source:MGI Symbol;Acc:I		1	72560203	72571773	11571	149,13	-0,26	6,25E-01
ENSRNOG00000047198		TatD DNase domain containing 1 [Source:MGI S		7	99382591	99403953	21363	104,42	-0,27	6,25E-01
ENSRNOG00000016636	**Lpin3**	lipin 3 (Lpin3), mRNA [Source:RefSeq	362261	3	163113545	163126527	12983	53,02	0,45	6,26E-01
ENSRNOG00000018163	**Ipcef1**	interaction protein for cytohesin excha	361474	1	44896239	44967362	71124	78,36	-0,29	6,26E-01
ENSRNOG00000026435	**Arid3a**	AT rich interactive domain 3A (Bright li	314616	7	12746095	12768092	21998	105,80	0,37	6,26E-01
ENSRNOG00000000923	**Cct6a**	chaperonin containing Tcp1, subunit 6	288620	12	32433338	32443781	10444	3490,07	0,29	6,26E-01
ENSRNOG00000001138	**Taok3**	TAO kinase 3 (Taok3), mRNA [Source	304530	12	46849166	47002369	153204	1309,88	0,27	6,26E-01
ENSRNOG00000001289	**LOC49815**	RIKEN cDNA 3110082I17 gene [Sourc	498154	12	19264599	19367357	102759	644,52	0,21	6,26E-01
ENSRNOG00000003495	**Prpf8**	pre-mRNA processing factor 8 (Prpf8)	287530	10	64348558	64371667	23110	12499,36	0,21	6,26E-01
ENSRNOG00000010078	**Spag1**	sperm associated antigen 1 (Spag1), r	315033	7	75143696	75202277	58582	70,46	-0,38	6,26E-01
ENSRNOG00000010580	**Acot7**	acyl-CoA thioesterase 7 (Acot7), trans	26759	5	172912096	173003553	91458	5774,35	0,33	6,26E-01
ENSRNOG00000011587	**Adcy6**	adenylate cyclase 6 (Adcy6), transcrip	25289	X	114774390	114794509	20120	2012,14	0,20	6,26E-01
ENSRNOG00000017841	**Nip30**	NEFA-interacting nuclear protein NIP3	307652	19	10821359	10850607	29249	1717,43	0,20	6,26E-01
ENSRNOG00000020615	**Nap1l4**	nucleosome assembly protein 1-like 4	361684	1	223575634	223611023	35390	6816,10	0,33	6,26E-01
ENSRNOG00000030160	**Zfp819**	zinc finger protein 819 (Zfp819), mRN	308561	1	100601819	100608478	6660	35,87	0,44	6,26E-01
ENSRNOG00000042978	**Ncald**	neurocalcin delta (Ncald), mRNA [Sou	553106	7	76320074	76414625	94552	4418,95	-0,26	6,26E-01
ENSRNOG00000002832	**Slc16a2**	solute carrier family 16, member 2 (thy	259248	X	75380006	75507522	127517	832,62	-0,20	6,26E-01
ENSRNOG00000003086	**Cenpv**	Protein Cenpv [Source:UniProtKB/TrE	501702	10	48632950	48645825	12876	956,41	0,32	6,26E-01
ENSRNOG00000003386	**Rbfox3**	RNA binding protein, fox-1 homolog (C	287847	10	107153676	107174397	20722	922,11	-0,25	6,26E-01
ENSRNOG00000004588	**LOC10091**	zinc finger, BED-type containing 4 (Zb	315211	7	129438426	129475805	37380	730,90	0,32	6,26E-01
ENSRNOG00000005538	**Psmd11**	proteasome (prosome, macropain) 26	303353	10	67465213	67510908	45696	3604,68	0,17	6,26E-01
ENSRNOG00000006617				4	141164125	141540241	376117	162,94	-0,31	6,26E-01
ENSRNOG00000010691	**Cmtm3**	CKLF-like MARVEL transmembrane d	291813	19	829213	836125	6913	285,42	0,34	6,26E-01
ENSRNOG00000011452	**Aldoc**	aldolase C, fructose-bisphosphate (Al	24191	10	66049072	66052680	3609	10482,81	-0,35	6,26E-01
ENSRNOG00000011788	**RGD15660**	Protein RGD1566029; Similar to mKIA	500913	7	63359409	63373749	14341	351,25	-0,32	6,26E-01
ENSRNOG00000012480	**Acpl2**	acid phosphatase-like 2 (Acpl2), mRN	315939	8	104171295	104237198	65904	818,91	0,28	6,26E-01
ENSRNOG00000012860	**Tmem184c**	transmembrane protein 184C (Tmem1	291946	19	44923193	44938607	15415	1374,58	-0,31	6,26E-01
ENSRNOG00000016484	**Gstk1**	glutathione S-transferase kappa 1 (Gs	297029	4	136427310	136431704	4395	408,59	-0,34	6,26E-01
ENSRNOG00000016872	**Lppr4**	lipid phosphate phosphatase-related p	295401	2	238365252	238405887	40636	4066,16	-0,32	6,26E-01
ENSRNOG00000020502	**LOC69046**	ribosomal protein S13 (Rps13), mRNA	161477	1	192299622	192302049	2428	134,11	0,27	6,26E-01

ENSRNOG00000020643		Protein Bub3 [Source:UniProtKB/TrEMBL;Acc:[		1	210535691	210567637	31947	1156,48	0,17	6,26E-01
ENSRNOG00000021638		eukaryotic translation elongation facto	300033	7	116820232	116835298	15067	2443,51	0,24	6,26E-01
ENSRNOG00000024677	**Arhgap33**	Protein Arhgap33 [Source:UniProtKB/	1E+08	1	90133412	90145522	12111	7898,27	-0,36	6,26E-01
ENSRNOG00000026604	**Cercam**	cerebral endothelial cell adhesion mol	296616	3	13774537	13788191	13655	56,45	-0,39	6,26E-01
ENSRNOG00000030517	**Zfp879**	zinc finger protein 879 (Zfp879), mRN	497896	10	36097634	36107629	9996	55,11	-0,30	6,26E-01
ENSRNOG00000031453	**Manbal**	mannosidase, beta A, lysosomal-like (	499934	3	158198825	158228410	29586	2090,15	0,26	6,26E-01
ENSRNOG00000039544	**Kcnd1**	potassium voltage-gated channel, Sha	116695	X	16288759	16301947	13189	158,03	-0,29	6,26E-01
ENSRNOG00000042478		Protein Adam22 [Source:UniProtKB/T	57033	4	22632878	22929264	296387	665,45	-0,23	6,26E-01
ENSRNOG00000047517	**Oxld1**	Protein LOC688310 [Source:UniProtK	688310	10	109222336	109223569	1234	207,90	0,22	6,26E-01
ENSRNOG00000015229	**Zc3h4**	Protein Zc3h4 [Source:UniProtKB/TrE	678741	1	79607132	79641214	34083	2035,84	0,17	6,26E-01
ENSRNOG00000018159	**Anxa4**	annexin A4 (Anxa4), mRNA [Source:R	79124	4	183108906	183164898	55993	102,13	-0,42	6,26E-01
ENSRNOG00000029342	**Scn7a**	sodium channel, voltage-gated, type V	64155	3	59396876	59469569	72694	95,90	-0,47	6,26E-01
ENSRNOG00000018937	**Gstm7**	glutathione S-transferase, mu 7 (Gstm	81869	2	230189594	230195105	5512	1347,58	-0,37	6,26E-01
ENSRNOG00000002453	**Taf9b**	TAF9B RNA polymerase II, TATA box	171152	X	56401296	56414163	12868	551,67	-0,30	6,26E-01
ENSRNOG00000006684	**Zfp317**	zinc finger protein 317 (Zfp317), mRN	500950	8	19964735	19983504	18770	270,40	-0,43	6,26E-01
ENSRNOG00000004503	**L3hypdh**	L-3-hydroxyproline dehydratase (trans	314214	6	104268771	104279567	10797	98,56	-0,33	6,26E-01
ENSRNOG00000023935	**Nrsn2**	neurensin 2 (Nrsn2), mRNA [Source:R	689978	3	154190944	154199640	8697	303,55	0,32	6,26E-01
ENSRNOG00000045765	**MGC94207**	similar to RIKEN cDNA C030006K11 (	362946	7	117774185	117776528	2344	939,51	0,27	6,26E-01
ENSRNOG00000004737	**Cd48**	Cd48 molecule (Cd48), mRNA [Source	245962	13	94737393	94760905	23513	178,41	-0,43	6,27E-01
ENSRNOG00000015750	**Wnt7b**	wingless-type MMTV integration site f	315196	7	126136632	126178957	42326	712,04	-0,38	6,27E-01
ENSRNOG00000030180	**Lrrc10b**	leucine rich repeat containing 10B (Lrr	309208	1	233448640	233449518	879	47,18	0,41	6,27E-01
ENSRNOG00000049281	**Gba**	glucosidase, beta, acid (Gba), mRNA	684536	2	207931042	207937061	6020	1526,97	0,33	6,27E-01
ENSRNOG00000009836		RNA-binding protein 26 [Source:RefS	306137	15	92760233	92831253	71021	1320,88	-0,24	6,27E-01
ENSRNOG00000034015	**Capn2**	calpain 2, (m/II) large subunit (Capn2)	29154	13	105814750	105864911	50162	3001,98	0,23	6,27E-01
ENSRNOG00000020867	**Numbl**	numb homolog (Drosophila)-like (Num	292732	1	85278881	85302554	23674	2235,62	0,18	6,27E-01
ENSRNOG00000046662		DEAD/H (Asp-Glu-Ala-Asp/His) box polypeptide		X	153466479	153506648	40170	48,15	-0,46	6,27E-01
ENSRNOG00000001439	**Srrm3**	serine/arginine repetitive matrix 3 (Srr	685890	12	25854467	25891680	37214	572,48	0,26	6,28E-01
ENSRNOG00000012961	**Klf7**	Kruppel-like factor 7 (ubiquitous) (Klf7), mRNA [S		9	71787156	71787933	778	331,06	-0,24	6,28E-01
ENSRNOG00000046851	**Cecr6**	Protein Cecr6 [Source:UniProtKB/TrE	500307	4	220114018	220115736	1719	207,68	-0,34	6,28E-01
ENSRNOG00000002041	**Boc**	Biregional cell adhesion molecule-rela	360715	11	65191760	65266747	74988	557,70	-0,35	6,29E-01
ENSRNOG00000008837	**Ass1**	argininosuccinate synthase 1 (Ass1),	25698	3	15686795	15728491	41697	31,41	-0,46	6,29E-01
ENSRNOG00000019500	**Cyp1a1**	cytochrome P450, family 1, subfamily	24296	8	62249046	62255076	6031	42,51	0,39	6,29E-01
ENSRNOG00000011199	**Parp6**	poly (ADP-ribose) polymerase family,	300759	8	64203227	64235456	32230	4293,20	0,22	6,29E-01
ENSRNOG00000011677	**Slc39a10**	Protein Slc39a10; Solute carrier family	363229	9	59726979	59755749	28771	1414,37	-0,22	6,29E-01
ENSRNOG00000048195	**Nudt13**	nudix (nucleoside diphosphate linked	682978	15	8534958	8555093	20136	186,92	0,30	6,29E-01
ENSRNOG00000001323	**Zfp157**	zinc finger protein 157 (Zfp157), mRN	360775	12	20449367	20471764	22398	336,54	-0,31	6,29E-01
ENSRNOG00000024553	**Cyb5d2**	cytochrome b5 domain containing 2 (C	303293	10	59082549	59098781	16233	156,94	0,35	6,29E-01
ENSRNOG00000000029	**Myh11**	myosin, heavy chain 11, smooth musc	24582	10	11444267	11538406	94140	67,61	-0,39	6,29E-01

ENSRNOG00000010712	**Terf2ip**	telomeric repeat binding factor 2, inter	307861	19	55037928	55044772	6845	2661,15	0,29	6,29E-01
ENSRNOG00000004637	**Fbxo7**	F-box protein 7 (Fbxo7), mRNA [Sourc	366854	7	23964802	23993030	28229	1746,89	0,19	6,29E-01
ENSRNOG00000005158	**Slc24a5**	solute carrier family 24 (sodium/potass	311387	3	123859070	123878338	19269	23,84	-0,40	6,29E-01
ENSRNOG00000008490	**Cwc15**	CWC15 spliceosome-associated prote	300361	8	12939501	12950303	10803	802,62	0,23	6,29E-01
ENSRNOG00000010975	**Adnp**	activity-dependent neuroprotector hom	64622	3	171077553	171087332	9780	5116,97	-0,18	6,29E-01
ENSRNOG00000013152	**Arhgap10**	Rho GTPase activating protein 10 (Arl	688429	19	45021146	45281603	260458	106,97	-0,25	6,29E-01
ENSRNOG00000016289	**Bmpr1b**	bone morphogenetic protein receptor,	310914	2	265932566	265965469	32904	148,15	-0,33	6,29E-01
ENSRNOG00000017337	**Lmbrd2**	LMBR1 domain containing 2 (Lmbrd2)	499539	2	77621904	77678202	56299	820,50	-0,24	6,29E-01
ENSRNOG00000019163	**Syt6**	synaptotagmin VI (Syt6), mRNA [Sour	60565	2	225492365	225553606	61242	73,94	-0,28	6,29E-01
ENSRNOG00000020857		protein tyrosine phosphatase, recepto	293645	1	224501043	224577428	76386	764,78	0,18	6,29E-01
ENSRNOG00000025053	**Lrp1**	low density lipoprotein receptor-relatec	299858	7	71018808	71099367	80560	16518,70	-0,22	6,29E-01
ENSRNOG00000003654	**Cldn9**	claudin 9 (Cldn9), mRNA [Source:RefS	287099	10	12826470	12827123	654	36,14	-0,47	6,29E-01
ENSRNOG00000010553	**Pnma1**	paraneoplastic Ma antigen 1 (Pnma1),	170636	6	117903766	117905380	1615	76,62	-0,30	6,29E-01
ENSRNOG00000020330	**Wdr47**	WD repeat domain 47 (Wdr47), mRNA	310785	2	230852232	230914750	62519	4274,80	-0,31	6,29E-01
ENSRNOG00000018051	**Farp2**	FERM, RhoGEF and pleckstrin domai	316639	9	100313423	100421287	107865	292,46	-0,22	6,30E-01
ENSRNOG00000019976		spermatogenesis associated 24 (Spat	291676	18	28159728	28167716	7989	89,10	-0,43	6,30E-01
ENSRNOG00000045920	**Mcu**	mitochondrial calcium uniporter (Mcu),	294560	20	30842417	31003860	161444	702,37	-0,26	6,30E-01
ENSRNOG00000003330	**Acsf2**	acyl-CoA synthetase family member 2	619561	10	82104326	82146073	41748	1093,81	-0,39	6,30E-01
ENSRNOG00000009722	**Chd3**	Protein Chd3 [Source:UniProtKB/TrEI	303241	10	55686400	55712312	25913	17318,86	0,29	6,30E-01
ENSRNOG00000012210	**Sptlc2**	serine palmitoyltransferase, long chair	366697	6	120620976	120699229	78254	807,99	0,23	6,31E-01
ENSRNOG00000012421		Protein Atp11b [Source:UniProtKB/TrEMBL;Acc		2	142002592	142080714	78123	636,85	-0,26	6,31E-01
ENSRNOG00000014000		kinesin heavy chain member 2A (Kif2a	84391	2	57241541	57302938	61398	2549,85	-0,22	6,31E-01
ENSRNOG00000034254	**Actb**	actin, beta (Actb), mRNA [Source:RefS	81822	12	15745854	15748827	2974	62896,40	0,24	6,31E-01
ENSRNOG00000013689	**Vps18**	vacuolar protein sorting 18 homolog (S	296083	3	117648071	117658924	10854	1325,84	0,24	6,32E-01
ENSRNOG00000016873	**Msrb2**	methionine sulfoxide reductase B2 (M	361286	17	87781867	87860120	78254	33,35	0,35	6,32E-01
ENSRNOG00000006962	**Stk32c**	serine/threonine kinase 32C (Stk32c),	365381	1	218367084	218446952	79869	1042,99	-0,24	6,32E-01
ENSRNOG00000005519	**Grm3**	glutamate receptor, metabotropic 3 (G	24416	4	21395468	21492586	97119	365,97	-0,44	6,32E-01
ENSRNOG00000001606	**Adamts5**	ADAM metallopeptidase with thrombo	304135	11	29034437	29081047	46611	77,67	-0,37	6,32E-01
ENSRNOG00000008105	**Dmrta2**	DMRT-like family A2 (Dmrta2), mRNA	313471	5	133586293	133592040	5748	307,67	0,29	6,33E-01
ENSRNOG00000024019	**Ccdc6**	Protein Ccdc6 [Source:UniProtKB/TrE	691155	20	21869694	21916820	47127	491,76	0,23	6,33E-01
ENSRNOG00000002721	**Ndufb4**	NADH dehydrogenase (ubiquinone) 1	288088	11	69052368	69058793	6426	917,42	0,24	6,33E-01
ENSRNOG00000005102	**Ccdc53**	coiled-coil domain containing 53 (Ccd	299707	7	28872399	28917928	45530	408,50	-0,34	6,33E-01
ENSRNOG00000010765	**Vcl**	vinculin (Vcl), mRNA [Source:RefSeq	305679	15	3433521	3522277	88757	1137,44	0,21	6,33E-01
ENSRNOG00000013763	**Erlin2**	ER lipid raft associated 2 (Erlin2), mRI	290823	16	68853256	68868395	15140	1464,63	-0,28	6,33E-01
ENSRNOG00000015196		Heat shock 70 kDa protein 14; RCG64128 [Sou		17	80383209	80384852	1644	42,60	0,44	6,33E-01
ENSRNOG00000017419	**Map3k4**	mitogen activated protein kinase kinas	308106	1	51043920	51129819	85900	1268,17	0,21	6,33E-01
ENSRNOG00000017552	**Mrpl34**	mitochondrial ribosomal protein L34 (M	290632	16	19735400	19736134	735	1087,61	0,34	6,33E-01
ENSRNOG00000019965	**Tgfb1i1**	transforming growth factor beta 1 indu	84574	1	206710120	206716908	6789	437,03	0,34	6,33E-01

ENSRNOG00000020148	**LOC10036**	interleukin 4 induced 1 [Source:MGI S	1E+08	1	101894734	101901347	6614	63,22	0,35	6,33E-01
ENSRNOG00000020923	**Tuft1**	tuftelin 1 (Tuft1), mRNA [Source:RefSe	365864	2	215340650	215386078	45429	122,08	-0,33	6,33E-01
ENSRNOG00000020957	**Igflr1**	IGF-like family receptor 1 (Igflr1), mRN	499126	1	90175156	90177034	1879	35,94	0,42	6,33E-01
ENSRNOG00000026023	**Taf4b**	Protein Taf4b [Source:UniProtKB/TrEI	291773	18	6169030	6241364	72335	63,70	0,33	6,33E-01
ENSRNOG00000031136	**Ntng1**	netrin G1 (Ntng1), mRNA [Source:Ref	295382	2	232164771	232320751	155981	283,98	0,25	6,33E-01
ENSRNOG00000046744	**Ankrd29**	ankyrin repeat domain 29 (Ankrd29), mRNA [So		18	3682901	3685174	2274	126,47	-0,34	6,33E-01
ENSRNOG00000046424	**Mfn2**	mitofusin 2 (Mfn2), mRNA [Source:Re	64476	5	168342974	168372608	29635	240,27	0,28	6,33E-01
ENSRNOG00000000488	**Hmga1**	high mobility group AT-hook 1 (Hmga1	117062	20	9338364	9341350	2987	810,53	0,26	6,33E-01
ENSRNOG00000002403	**Fam129a**	family with sequence similarity 129, m	63912	13	73927637	74076132	148496	163,13	0,45	6,33E-01
ENSRNOG00000005184	**Nop10**	NOP10 ribonucleoprotein (Nop10), mF	691534	3	110342516	110343601	1086	936,27	0,28	6,33E-01
ENSRNOG00000003925		hedgehog acyltransferase [Source:MGI Symbol;		13	115793877	116027857	233981	35,32	0,36	6,33E-01
ENSRNOG00000007447	**Pla2g4b**	phospholipase A2, group IVB (cytosoli	311341	3	118373799	118382163	8365	179,17	0,28	6,33E-01
ENSRNOG00000010050	**LOC69192**	START domain containing 9 [Source:M	691920	3	118974028	119202861	228834	326,76	-0,35	6,33E-01
ENSRNOG00000022391	**Zfp711**	Protein Zfp711 [Source:UniProtKB/Tr	302327	X	84015120	84043574	28455	187,20	-0,41	6,33E-01
ENSRNOG00000029373	**Naa10**	N(alpha)-acetyltransferase 10, NatA ca	363518	1	152611822	152617018	5197	719,49	0,27	6,33E-01
ENSRNOG00000006002	**Stk35**	serine/threonine kinase 35 (Stk35), ml	311419	3	127680155	127708598	28444	459,88	0,24	6,34E-01
ENSRNOG00000020526		Mau2 chromatid cohesion factor homo	290668	16	21047385	21075417	28033	2029,21	0,27	6,34E-01
ENSRNOG00000002985	**Nos1ap**	nitric oxide synthase 1 (neuronal) ada	192363	13	93279049	93562008	282960	341,99	-0,25	6,34E-01
ENSRNOG00000004688	**Rbfox2**	RNA binding protein, fox-1 homolog (C	362950	7	118149476	118388515	239040	3845,67	0,34	6,34E-01
ENSRNOG00000014003	**Fbxo16**	F-box protein 16 (Fbxo16), mRNA [So	305970	15	52415892	52465127	49236	284,34	0,27	6,34E-01
ENSRNOG00000014128	**Ecsit**	ECSIT signalling integrator (Ecsit), mF	300447	8	23140382	23153830	13449	570,71	0,19	6,34E-01
ENSRNOG00000020028	**Pcdhb22**	Protein Pcdhb22; RCG49585 [Source	307486	18	30299989	30302376	2388	258,35	-0,24	6,34E-01
ENSRNOG00000021611	**Clip2**	CAP-GLY domain containing linker pro	29264	12	27173529	27237324	63796	7007,91	0,22	6,34E-01
ENSRNOG00000027444	**Nubpl**	nucleotide binding protein-like (Nubpl)	299008	6	82453613	82709215	255603	228,63	0,25	6,34E-01
ENSRNOG00000036664	**Narf**	nuclear prelamin A recognition factor (	360681	10	110032455	110050385	17931	2190,62	0,34	6,34E-01
ENSRNOG00000000040	**RGD13046**	Protein RGD1304622 [Source:UniPro	305101	13	77796633	77815260	18628	26,72	-0,46	6,34E-01
ENSRNOG00000011138	**LOC69066**	ribosomal protein L29 (Rpl29), mRNA	29283	8	114495444	114497490	2047	795,98	0,28	6,34E-01
ENSRNOG00000013944	**Zc3hav1l**	Protein Zc3hav1l; RCG28359 [Source	362341	4	65805740	65816897	11158	43,12	-0,35	6,34E-01
ENSRNOG00000016433	**Prrc1**	proline-rich coiled-coil 1 (Prrc1), mRN	291444	18	51612007	51645426	33420	493,16	0,26	6,34E-01
ENSRNOG00000003822	**Slu7**	SLU7 splicing factor homolog (S. cere	303057	10	28871472	28886424	14953	571,14	-0,42	6,34E-01
ENSRNOG00000009542	**Kcnh5**	potassium voltage-gated channel, sub	171146	6	107294666	107578241	283576	28,15	-0,45	6,34E-01
ENSRNOG00000015971	**Slc12a2**	solute carrier family 12 (sodium/potas	83629	18	52104434	52172618	68185	1294,81	-0,26	6,34E-01
ENSRNOG00000020429		fuzzy planar cell polarity protein (Fuz),	308577	1	101956485	101961425	4941	635,96	0,27	6,34E-01
ENSRNOG00000038970	**Fgd1**	FYVE, RhoGEF and PH domain conta	363460	X	20846380	20888659	42280	560,00	-0,26	6,34E-01
ENSRNOG00000005923	**Mtmr2**	myotubularin related protein 2 (Mtmr2	315422	8	12233929	12284288	50360	1731,54	0,25	6,35E-01
ENSRNOG00000011112		Protein Fam161b; RCG20883 [Source:UniProtK		6	117470211	117485334	15124	337,78	0,26	6,35E-01
ENSRNOG00000012590	**Rbm41**	RNA binding motif protein 41 (Rbm41)	680581	3	53272291	53322770	50480	66,93	-0,34	6,35E-01
ENSRNOG00000018211	**Urod**	uroporphyrinogen decarboxylase (Uro	29421	5	139650565	139654649	4085	828,96	0,28	6,35E-01

ENSRNOG00000023781	**Plec**	plectin (Plec), transcript variant 1, mRl	64204	7	117215919	117275610	59692	1940,90	-0,35	6,35E-01
ENSRNOG00000013039	**Add1**	adducin 1 (alpha) (Add1), mRNA [Sou	24170	14	82054924	82113952	59029	9791,85	0,22	6,36E-01
ENSRNOG00000016559	**Tm2d2**	TM2 domain containing 2 (Tm2d2), ml	290833	16	71453136	71458658	5523	1847,54	0,24	6,36E-01
ENSRNOG00000048877		Uncharacterized protein [Source:UniProtKB/TrE		3	134212394	134414865	202472	78,67	-0,36	6,36E-01
ENSRNOG00000001516	**Rapgef4**	Rap guanine nucleotide exchange fac	252857	3	65349112	65408826	59715	534,84	-0,25	6,36E-01
ENSRNOG00000006328	**Wwp1**	WW domain containing E3 ubiquitin pr	297930	5	38263540	38318839	55300	519,42	-0,37	6,36E-01
ENSRNOG00000008224	**Jdp2**	Jun dimerization protein 2 (Jdp2), mRl	116674	6	118780172	118988281	208110	246,80	-0,33	6,36E-01
ENSRNOG00000008378	**Zfp414**	zinc finger protein 414 (Zfp414), mRN.	299647	7	18675411	18678017	2607	753,34	0,25	6,36E-01
ENSRNOG00000013027	**Rgl3**	ral guanine nucleotide dissociation stir	300444	8	23035692	23055269	19578	41,05	-0,38	6,36E-01
ENSRNOG00000017426	**Rragc**	Ras-related GTP binding C (Rragc), m	298514	5	145361045	145379290	18246	2260,50	0,29	6,36E-01
ENSRNOG00000019992	**Cnbd2**	cyclic nucleotide binding domain conta	296311	3	159226090	159288261	62172	1728,73	0,31	6,36E-01
ENSRNOG00000030308	**Zfp251**	zinc finger protein 251 (Zfp251), mRN.	366954	7	117899537	117924579	25043	988,07	0,31	6,36E-01
ENSRNOG00000030452	**Rimbp3**	Protein Rimbp3 [Source:UniProtKB/Tı	303785	11	91087427	91092265	4839	55,51	-0,30	6,36E-01
ENSRNOG00000032552	**Zfp799**	zinc finger protein 799 (Zfp799), mRN.	494339	7	15224533	15230625	6093	369,66	-0,28	6,36E-01
ENSRNOG00000040266	**Cdkl4**	Protein Cdkl4 [Source:UniProtKB/TrE	503009	6	3203271	3223505	20235	42,48	-0,36	6,36E-01
ENSRNOG00000043151	**Cntln**	centlein, centrosomal protein (Cntln), r	679640	5	107251787	107366588	114802	38,22	-0,44	6,36E-01
ENSRNOG00000046497	**Brap**	BRCA1 associated protein (Brap), mR	687346	12	42260102	42285527	25426	626,10	0,17	6,36E-01
ENSRNOG00000021139	**Esrra**	estrogen related receptor, alpha (Esrra	293701	1	229159584	229169579	9996	1223,41	0,33	6,36E-01
ENSRNOG00000004578	**Cthrc1**	collagen triple helix repeat containing	282836	7	78271770	78282052	10283	23,40	-0,45	6,36E-01
ENSRNOG00000018569	**Ahcyl1**	adenosylhomocysteinase-like 1 (Ahcyl	362013	2	229905782	229920833	15052	5402,58	-0,31	6,36E-01
ENSRNOG00000001302	**Adora2a**	adenosine A2a receptor (Adora2a), ml	25369	20	16449385	16466147	16763	134,65	0,31	6,36E-01
ENSRNOG00000002180		Protein Tbc1d1 [Source:UniProtKB/Tr	360937	14	45405698	45590253	184556	579,06	0,20	6,36E-01
ENSRNOG00000007613	**C1qtnf5**	C1q and tumor necrosis factor related	315598	8	47059596	47061466	1871	1035,57	-0,40	6,36E-01
ENSRNOG00000017447	**Eif2s2**	eukaryotic translation initiation factor 2	296302	3	156763138	156783857	20720	1844,04	-0,31	6,36E-01
ENSRNOG00000024809	**Ntmt1**	N-terminal Xaa-Pro-Lys N-methyltrans	362103	3	15002396	15018993	16598	1100,01	0,22	6,36E-01
ENSRNOG00000037638	**LOC68729**	translocase of inner mitochondrial mer	687295	1	86684450	86691548	7099	1283,78	0,28	6,36E-01
ENSRNOG00000014117	**Hmox1**	heme oxygenase (decycling) 1 (Hmox	24451	19	25622556	25629372	6817	5492,09	-0,45	6,37E-01
ENSRNOG00000002587	**LOC49826**	cDNA sequence BC055324 [Source:M	498265	13	87127496	87171448	43953	110,97	-0,34	6,37E-01
ENSRNOG00000002963	**C1ql1**	complement component 1, q subcomp	363686	10	90812551	90819723	7173	761,61	-0,43	6,37E-01
ENSRNOG00000007674	**Nacad**	NAC alpha domain containing (Nacad	289786	14	80494052	80502042	7991	2093,39	0,23	6,37E-01
ENSRNOG00000007706	**Prkaa2**	protein kinase, AMP-activated, alpha 2	78975	5	128436023	128503874	67852	662,41	0,21	6,37E-01
ENSRNOG00000018911	**Pfkfb3**	6-phosphofructo-2-kinase/fructose-2,6	117276	17	72386425	72413515	27091	1504,05	-0,30	6,37E-01
ENSRNOG00000043095	**Etv3**	ets variant 3 (Etv3), mRNA [Source:Re	295297	2	206278979	206288515	9537	196,15	0,36	6,37E-01
ENSRNOG00000019953		ubiquitin-conjugating enzyme E2F (pu	363284	9	98074824	98105362	30539	638,93	0,22	6,37E-01
ENSRNOG00000003985	**Tmem19**	transmembrane protein 19 (Tmem19),	299800	7	58325486	58352039	26554	459,23	0,36	6,37E-01
ENSRNOG00000006355	**Akap8l**	A kinase (PRKA) anchor protein 8-like	299569	7	14494266	14523993	29728	1233,21	0,29	6,37E-01
ENSRNOG00000016894	**Dnd1**	DND microRNA-mediated repression i	679841	18	29312395	29315019	2625	24,66	-0,42	6,37E-01
ENSRNOG00000017990	**Ubap2l**	Atpase, class I, type 8B, member 2 (A	361984	2	208766396	208832721	66326	5167,04	-0,17	6,37E-01

ENSRNOG00000020742	**Pcnxl3**	Protein Pcnxl3 [Source:UniProtKB/TrE	309167	1	227954413	227976723	22311	2666,62	0,17	6,37E-01
ENSRNOG00000032828	**Mapk8ip2**	Mitogen-activated protein kinase 8 inte	315220	7	130116132	130123675	7544	5423,10	0,21	6,37E-01
ENSRNOG00000007172	**Map4k3**	mitogen-activated protein kinase kinas	170920	6	3247187	3414716	167530	2441,25	-0,26	6,37E-01
ENSRNOG00000008054	**Igsf9**	immunoglobulin superfamily, member	304982	13	95389058	95405643	16586	911,79	0,29	6,37E-01
ENSRNOG00000010706	**Ccdc117**	coiled-coil domain containing 117 (Ccc	498404	14	86433500	86442865	9366	463,47	0,26	6,37E-01
ENSRNOG00000011873	**Atg14**	autophagy related 14 (Atg14), mRNA [	305831	15	28278546	28309904	31359	609,01	0,38	6,37E-01
ENSRNOG00000021555	**Mis18a**	MIS18 kinetochore protein homolog A	288272	11	34491738	34504737	13000	151,95	0,34	6,37E-01
ENSRNOG00000033479	**Pcdhb21**	protocadherin beta 21 (Pcdhb21), mR	307487	18	30295067	30297984	2918	54,16	-0,35	6,37E-01
ENSRNOG00000000786	**Rpp21**	ribonuclease P/MRP 21 subunit (Rpp2	406230	20	4545572	4547523	1952	1094,88	0,33	6,37E-01
ENSRNOG00000018087	**Vim**	vimentin (Vim), mRNA [Source:RefSec	81818	17	82500858	82509377	8520	30737,41	0,38	6,37E-01
ENSRNOG00000018649	**Slc35c2**	solute carrier family 35 (GDP-fucose tı	311637	3	167986132	167997206	11075	1614,99	0,28	6,37E-01
ENSRNOG00000000808	**Hsf2**	heat shock transcription factor 2 (Hsf2	64441	20	40673635	40701124	27490	987,13	-0,29	6,37E-01
ENSRNOG00000015160	**Gem**	GTP binding protein overexpressed in	297902	5	30058938	30062666	3729	106,69	-0,46	6,37E-01
ENSRNOG00000005006	**Ndufaf1**	NADH dehydrogenase (ubiquinone) cc	296086	3	118007327	118017688	10362	330,67	0,35	6,37E-01
ENSRNOG00000010350	**Rcan2**	regulator of calcineurin 2 (Rcan2), mR	140666	9	18397067	18626606	229540	1591,47	0,26	6,37E-01
ENSRNOG00000050266	**Hist1h2ao**	Histone H2A type 1-E [Source:UniPro	364723	17	58718819	58719211	393	30,27	0,42	6,38E-01
ENSRNOG00000013581	**Extl3**	exostosin-like glycosyltransferase 3 (E	56819	15	52169314	52192414	23101	1546,55	0,21	6,38E-01
ENSRNOG00000002135	**Sgcb**	sarcoglycan, beta (dystrophin-associa	680229	14	36936116	36951529	15414	1770,39	-0,24	6,38E-01
ENSRNOG00000007564	**Evc**	Ellis van Creveld syndrome (Evc), mR	289712	14	78187671	78227302	39632	494,87	-0,28	6,38E-01
ENSRNOG00000010514		VWFA and cache domain-containing p	298267	5	123487662	123714027	226366	1096,81	-0,25	6,38E-01
ENSRNOG00000013336	**Wdr70**	WD repeat domain 70 (Wdr70), mRNA	294783	2	76953968	77203824	249857	629,11	0,22	6,38E-01
ENSRNOG00000016779	**LOC68342(**	family with sequence similarity 120A (I	291019	17	18167847	18257857	90011	2988,22	-0,23	6,38E-01
ENSRNOG00000017959	**Ccdc37**	Protein Ccdc37 [Source:UniProtKB/Tı	297444	4	186855629	186870548	14920	27,34	-0,39	6,38E-01
ENSRNOG00000018939	**Rexo2**	RNA exonuclease 2 (Rexo2), mRNA [S	300689	8	51395457	51407172	11716	1706,75	0,19	6,38E-01
ENSRNOG00000019262	**Trak1**	trafficking protein, kinesin binding 1 (T	316085	8	129205675	129298643	92969	3035,63	-0,17	6,38E-01
ENSRNOG00000019222	**MGC11283**	similar to transcription factor (MGC112	361178	16	80578524	80615798	37275	3496,70	0,24	6,38E-01
ENSRNOG00000018419	**Zfp275**	zinc finger protein 275 (Zfp275), mRN,	293849	1	153203960	153220664	16705	1323,63	-0,22	6,38E-01
ENSRNOG00000012324		SOGA family member 3 [Source:MGI S	1E+08	1	32496076	32550554	54479	2872,33	0,26	6,39E-01
ENSRNOG00000025385	**Coa4**	cytochrome c oxidase assembly factor	499214	1	171750590	171753818	3229	314,48	-0,28	6,39E-01
ENSRNOG00000009620	**Cybrd1**	cytochrome b reductase 1 (Cybrd1), m	295669	3	64212933	64240161	27229	20,14	-0,44	6,39E-01
ENSRNOG00000013481	**Cdh11**	cadherin 11 (Cdh11), mRNA [Source:F	84407	19	2374108	2531571	157464	1855,41	-0,18	6,39E-01
ENSRNOG00000011814	**LOC10036(**	chromobox homolog 3 (Cbx3), mRNA	297093	4	145910667	145924081	13415	956,96	-0,22	6,40E-01
ENSRNOG00000022971		zinc finger protein 454 [Source:MGI Symbol;Acc		10	36136552	36146954	10403	95,00	-0,35	6,40E-01
ENSRNOG00000032019	**Bend4**	Protein Bend4 [Source:UniProtKB/TrE	681008	14	42198080	42227219	29140	124,51	0,27	6,40E-01
ENSRNOG00000016752	**Crispld2**	cysteine-rich secretory protein LCCL c	171547	19	63411526	63452565	41040	31,43	-0,38	6,40E-01
ENSRNOG00000002520	**Litaf**	lipopolysaccharide-induced TNF factor	65161	10	3548079	3589969	41891	851,83	-0,39	6,41E-01
ENSRNOG00000003397	**Adprm**	ADP-ribose/CDP-alcohol diphosphata:	287406	10	53334313	53346792	12480	479,46	0,27	6,41E-01
ENSRNOG00000006369	**Alg2**	ALG2, alpha-1,3/1,6-mannosyltransfer	313231	5	67707027	67711584	4558	7541,23	0,34	6,41E-01

ENSRNOG00000017846	**Slc44a1**	solute carrier family 44 (choline transp	85254	5	74448193	74543855	95663	910,25	-0,20	6,41E-01
ENSRNOG00000003846	**Pitpna**	phosphatidylinositol transfer protein, a	29525	10	64232947	64273229	40283	5363,26	0,24	6,41E-01
ENSRNOG00000004477	**Ublcp1**	ubiquitin-like domain containing CTD p	360514	10	29936811	29952077	15267	926,11	-0,30	6,41E-01
ENSRNOG00000009720	**Zcchc11**	zinc finger, CCHC domain containing 1	313481	5	131941061	132009237	68177	661,70	-0,24	6,41E-01
ENSRNOG00000009994	**Dlat**	dihydrolipoamide S-acetyltransferase	81654	8	53659922	53685238	25317	699,67	0,20	6,41E-01
ENSRNOG00000010021	**Isy1**	ISY1 splicing factor homolog (S. cerev	362394	4	184972995	184993684	20690	995,39	0,25	6,41E-01
ENSRNOG00000010593	**Ctnnal1**	catenin (cadherin associated protein),	298019	5	77719359	77778979	59621	427,03	-0,27	6,41E-01
ENSRNOG00000011648	**Aqp1**	aquaporin 1 (Aqp1), mRNA [Source:R	25240	4	150203461	150215641	12181	26,27	-0,45	6,41E-01
ENSRNOG00000015619	**Agfg1**	ArfGAP with FG repeats 1 (Agfg1), mF	363266	9	88354885	88410583	55699	1497,21	-0,29	6,41E-01
ENSRNOG00000021118	**Nomo1**	nodal modulator 1 (Nomo1), mRNA [S	361578	1	103100473	103152080	51608	2370,41	0,21	6,41E-01
ENSRNOG00000028526	**Mansc1**	MANSC domain containing 1 (Mansc1	690606	4	232645471	232657452	11982	94,52	-0,38	6,41E-01
ENSRNOG00000032575	**Ocel1**	occludin/ELL domain containing 1 (Oc	290628	16	19627386	19629155	1770	741,61	0,29	6,41E-01
ENSRNOG00000018099	**Itch**	itchy E3 ubiquitin protein ligase (Itch),	311567	3	157055848	157117955	62108	623,95	-0,32	6,41E-01
ENSRNOG00000003657	**Pkmyt1**	protein kinase, membrane associated	287101	10	12880557	12891739	11183	260,92	0,25	6,41E-01
ENSRNOG00000011918	**Vsx2**	visual system homeobox 2 (Vsx2), mF	171360	6	117237657	117260423	22767	17,66	-0,43	6,41E-01
ENSRNOG00000024456	**Radil**	Ras association and DIL domains (Ra	304299	12	16120532	16185309	64778	253,66	0,25	6,41E-01
ENSRNOG00000030688	**Lrrc2**	leucine rich repeat containing 2 (Lrrc2	301033	8	118567954	118602409	34456	43,35	-0,45	6,41E-01
ENSRNOG00000042576		T-complex protein 11-like protein 1 [S	499846	3	101247426	101278392	30967	1387,66	-0,26	6,41E-01
ENSRNOG00000013653	**Pdlim7**	PDZ and LIM domain 7 (Pdlim7), mRN	286908	17	11762295	11777443	15149	645,23	0,27	6,41E-01
ENSRNOG00000003626		Uncharacterized protein [Source:UniProtKB/TrE		10	104308188	104311283	3096	2818,82	0,29	6,42E-01
ENSRNOG00000001958	**Ift57**	intraflagellar transport 57 homolog (Ch	303968	11	56827640	56895355	67716	827,99	-0,19	6,42E-01
ENSRNOG00000002247	**Nup54**	nucleoporin 54 (Nup54), mRNA [Sourc	53372	14	17039623	17057923	18301	436,38	-0,20	6,42E-01
ENSRNOG00000015177	**Sun2**	Protein Sun2 [Source:UniProtKB/TrEI	315135	7	120941356	120958528	17173	2234,68	0,30	6,42E-01
ENSRNOG00000015308	**Pbk**	PDZ binding kinase (Pbk), mRNA [Sou	290326	15	49092280	49103281	11002	169,32	0,40	6,42E-01
ENSRNOG00000015732	**Bcl2l2**	Bcl2-like 2 (Bcl2l2), mRNA [Source:Re	60434	15	37431258	37436110	4853	1818,61	0,27	6,42E-01
ENSRNOG00000018859	**Pik3ip1**	phosphoinositide-3-kinase interacting	305472	14	84248813	84260745	11933	1550,76	0,40	6,42E-01
ENSRNOG00000019618		histone deacetylase 3 [Source:RefSe	84578	18	30764345	30782343	17999	2557,08	0,20	6,42E-01
ENSRNOG00000025384	**Slc2a10**	solute carrier family 2 (facilitated gluc	366251	3	168364925	168377379	12455	216,96	-0,35	6,42E-01
ENSRNOG00000033700	**Wbscr22**	Williams Beuren syndrome chromosor	368084	12	26669896	26681097	11202	455,54	0,33	6,42E-01
ENSRNOG00000042384	**Kctd7**	potassium channel tetramerization dor	688993	12	31961819	31970830	9012	313,36	0,26	6,42E-01
ENSRNOG00000014155	**Spg20**	spastic paraplegia 20 (Troyer syndrom	295053	2	163938864	163963320	24457	691,28	0,27	6,42E-01
ENSRNOG00000018359	**Smad7**	SMAD family member 7 (Smad7), mR	81516	18	70530559	70558925	28367	250,72	-0,23	6,42E-01
ENSRNOG00000013100	**Supt20**	suppressor of Ty 20 (Supt20), mRNA [	361946	2	160337768	160370670	32903	1077,63	-0,24	6,43E-01
ENSRNOG00000021222	**Vps16**	vacuolar protein sorting 16 homolog (S	296159	3	129448274	129469256	20983	1589,60	0,24	6,43E-01
ENSRNOG00000014969	**Lzts2**	leucine zipper, putative tumor suppres	365468	1	272213429	272218933	5505	891,58	0,25	6,43E-01
ENSRNOG00000016753	**Slc14a1**	solute carrier family 14 (urea transport	54301	18	74136473	74161863	25391	1013,40	-0,45	6,43E-01
ENSRNOG00000020466	**Apba3**	amyloid beta (A4) precursor protein-bi	83611	7	11481877	11486849	4973	614,80	0,30	6,43E-01
ENSRNOG00000024507				7	83817592	83819264	1673	156,54	-0,38	6,43E-01

ENSRNOG00000019456	**Kars**	lysyl-tRNA synthetase (Kars), mRNA [	292028	19	55018816	55037768	18953	3712,60	0,20	6,44E-01
ENSRNOG00000001205	**Agpat3**	1-acylglycerol-3-phosphate O-acyltran	294324	20	13285583	13314837	29255	8339,26	-0,21	6,44E-01
ENSRNOG00000017282	**Tesk2**	testis-specific kinase 2 (Tesk2), mRNA	170908	5	139370347	139456132	85786	99,67	0,30	6,44E-01
ENSRNOG00000000925	**Psph**	phosphoserine phosphatase (Psph), n	304429	12	32455767	32468317	12551	1221,48	-0,38	6,44E-01
ENSRNOG00000003100	**Dusp12**	dual specificity phosphatase 12 (Dusp	64014	13	93870680	93879772	9093	294,28	0,32	6,44E-01
ENSRNOG00000003724	**Mrpl27**	mitochondrial ribosomal protein L27 (M	287635	10	82194180	82199911	5732	1573,75	0,24	6,44E-01
ENSRNOG00000003835	**Slc43a2**	solute carrier family 43 (amino acid sy	287532	10	64285713	64327600	41888	1493,29	0,32	6,44E-01
ENSRNOG00000005486		PHD finger protein 20-like 1 (Phf20l1),	314964	7	107345691	107373740	28050	154,11	-0,31	6,44E-01
ENSRNOG00000006331	**Elovl5**	ELOVL fatty acid elongase 5 (Elovl5),	171400	8	84825602	84851400	25799	1678,35	0,23	6,44E-01
ENSRNOG00000006967	**Xiap**	X-linked inhibitor of apoptosis (Xiap), r	63879	X	128507449	128543727	36279	320,55	-0,34	6,44E-01
ENSRNOG00000007115	**Crebl2**	cAMP responsive element binding pro	362453	4	232891153	232893464	2312	59,43	-0,44	6,44E-01
ENSRNOG00000009116	**Itgb3bp**	integrin beta 3 binding protein (beta3-e	362548	5	122404718	122470214	65497	148,90	-0,31	6,44E-01
ENSRNOG00000010887	**RGD13095**	similar to RIKEN cDNA 4931406C07 (	363016	8	13778080	13800657	22578	430,88	-0,29	6,44E-01
ENSRNOG00000011817	**LOC68871**	ribosomal protein L22 like 1 (Rpl22l1),	361923	2	135060063	135061993	1931	681,30	-0,30	6,44E-01
ENSRNOG00000015866	**Hint2**	histidine triad nucleotide binding prote	313491	5	63675137	63677391	2255	746,95	0,34	6,44E-01
ENSRNOG00000017373	**Rhobtb2**	Rho-related BTB domain containing 2	306004	15	55195153	55213442	18290	1175,50	-0,18	6,44E-01
ENSRNOG00000018145	**Crat**	carnitine O-acetyltransferase (Crat), m	311849	3	14320765	14334358	13594	1479,80	-0,28	6,44E-01
ENSRNOG00000019113	**LOC10036**	heterogeneous nuclear ribonucleoprot	117282	17	8870031	8881027	10997	4412,61	-0,21	6,44E-01
ENSRNOG00000019643	**Pcyox1l**	prenylcysteine oxidase 1 like (Pcyox1l	307396	18	56249996	56259794	9799	1034,71	0,17	6,44E-01
ENSRNOG00000019645	**Osbp2**	oxysterol binding protein 2 (Osbp2), m	305475	14	84628870	84790722	161853	825,99	-0,19	6,44E-01
ENSRNOG00000019710	**Tm9sf1**	transmembrane 9 superfamily membe	361043	15	38204851	38212312	7462	915,57	0,21	6,44E-01
ENSRNOG00000020201	**Yif1a**	Yip1 interacting factor homolog A (S. c	171441	1	227401143	227405550	4408	455,40	0,22	6,44E-01
ENSRNOG00000021068	**Pip5k1a**	phosphatidylinositol-4-phosphate 5-kir	365865	2	215722371	215765253	42883	1275,56	0,26	6,44E-01
ENSRNOG00000022261	**Mlf1ip**	MLF1 interacting protein (Mlf1ip), mRN	306464	16	48613434	48636501	23068	48,95	0,42	6,44E-01
ENSRNOG00000022312	**Lrsam1**	leucine rich repeat and sterile alpha m	311866	3	17306364	17344203	37840	744,95	0,27	6,44E-01
ENSRNOG00000024998	**Trps1**	trichorhinophalangeal syndrome I (Trp	299897	7	90118884	90343325	224442	395,71	-0,35	6,44E-01
ENSRNOG00000025592	**Tspan31**	tetraspanin 31 (Tspan31), mRNA [Sou	362890	7	70530384	70533257	2874	1957,95	0,26	6,44E-01
ENSRNOG00000026857	**Kif7**	Protein Kif7 [Source:UniProtKB/TrEM	293047	1	142395563	142412012	16450	388,42	-0,28	6,44E-01
ENSRNOG00000030622		Cytosolic ovarian carcinoma antigen 1	302817	X	135988059	136139829	151771	65,01	0,35	6,44E-01
ENSRNOG00000036918	**Mettl20**	methyltransferase like 20 (Mettl20), m	316976	4	247781172	247790913	9742	36,99	-0,41	6,44E-01
ENSRNOG00000039091	**Pnpla8**	Protein Pnpla8; Similar to intracellular	314075	6	73841473	73876145	34673	1458,20	-0,41	6,44E-01
ENSRNOG00000045857		transmembrane emp24 domain containing 8 [So		6	120479116	120506780	27665	210,67	0,32	6,44E-01
ENSRNOG00000001645	**Filip1l**	Protein Filip1l [Source:UniProtKB/TrE	304020	11	48253095	48319928	66834	88,32	-0,37	6,44E-01
ENSRNOG00000011047	**Tmem185a**	transmembrane protein 185A (Tmem1	309357	8	69277216	69302074	24859	849,31	0,35	6,44E-01
ENSRNOG00000019438	**Rnf31**	ring finger protein 31 (Rnf31), mRNA [	364386	15	38160918	38172580	11663	544,45	0,25	6,44E-01
ENSRNOG00000019671	**Rsbn1**	round spermatid basic protein 1 (Rsbn	310749	2	225815982	225875478	59497	1015,03	-0,31	6,44E-01
ENSRNOG00000021359	**Mtfr1**	mitochondrial fission regulator 1 (Mtfr1	311403	2	123742968	123761211	18244	68,11	0,32	6,44E-01
ENSRNOG00000045822		Uncharacterized protein [Source:UniProtKB/TrE		18	78435262	78438593	3332	18,32	-0,40	6,44E-01

ENSRNOG00000046950	**Ewsr1**	EWS RNA-binding protein 1 (Ewsr1), ı	289752	4	24535689	24564164	28476	109,37	0,26	6,44E-01
ENSRNOG00000013553		neugrin, neurite outgrowth associated	499191	1	143003297	143009170	5874	1993,24	0,31	6,44E-01
ENSRNOG00000011241	**Tfdp2**	transcription factor Dp-2 (E2F dimeriza	300947	8	103555032	103624736	69705	236,40	0,30	6,45E-01
ENSRNOG00000036827	**Ppp1r1a**	protein phosphatase 1, regulatory (inh	58977	7	142925731	142933381	7651	1948,16	-0,29	6,45E-01
ENSRNOG00000004762	**Ak7**	adenylate kinase 7 (Ak7), mRNA [Sou	314416	6	138732026	138804714	72689	57,59	-0,44	6,45E-01
ENSRNOG00000002342	**Aldh3a2**	aldehyde dehydrogenase 3 family, me	65183	10	47300331	47320877	20547	1668,00	0,24	6,45E-01
ENSRNOG00000002415		ankyrin-repeat and fibronectin type III	1E+08	10	76797910	76834936	37027	19,04	-0,45	6,45E-01
ENSRNOG00000004719	**Pp2d1**	protein phosphatase 2C-like domain c	316157	14	72968271	72989251	20981	135,29	-0,28	6,45E-01
ENSRNOG00000016186	**Zfp709**	zinc finger protein 709 (Zfp709), mRN	266773	16	19491753	19500855	9103	252,52	-0,24	6,45E-01
ENSRNOG00000017102	**Cmtr2**	cap methyltransferase 2 (Cmtr2), mRN	292016	19	52255039	52261870	6832	459,78	0,29	6,45E-01
ENSRNOG00000000250	**Jmjd6**	jumonji domain containing 6 (Jmjd6), r	360665	10	105440306	105446356	6051	808,80	0,24	6,45E-01
ENSRNOG00000009639	**Zrsr1**	zinc finger (CCCH type), RNA binding	498425	14	107826181	107827911	1731	424,02	0,33	6,45E-01
ENSRNOG00000014186	**RGD13102**	Protein RGD1310257 [Source:UniPro	308381	1	76703515	76723402	19888	241,66	0,23	6,45E-01
ENSRNOG00000015525	**Zswim3**	zinc finger, SWIM-type containing 3 (Z	311630	3	167457690	167472950	15261	435,89	0,24	6,45E-01
ENSRNOG00000029821	**Zfp689**	zinc finger protein 689 (Zfp689), mRN	286996	1	205892549	205898917	6369	471,07	0,18	6,45E-01
ENSRNOG00000001487	**Fam120b**	family with sequence similarity 120B (I	308218	1	242134746	242181174	46429	2548,23	0,24	6,45E-01
ENSRNOG00000003853	**Scoc**	short coiled-coil protein (Scoc), mRNA	364981	19	35562898	35592442	29545	4413,05	0,24	6,46E-01
ENSRNOG00000004823		dedicator of cytokinesis 4 [Source:MG	366608	6	70153948	70369085	215138	1072,78	0,26	6,46E-01
ENSRNOG00000013238	**Tmem14a**	transmembrane protein 14A (Tmem14	363206	9	26187241	26204118	16878	195,71	0,26	6,46E-01
ENSRNOG00000008165	**Tpx2**	TPX2, microtubule-associated (Tpx2),	311546	3	154730957	154773179	42223	638,08	0,43	6,46E-01
ENSRNOG00000017600	**Ptpn9**	protein tyrosine phosphatase, non-rec	266611	8	60228924	60308849	79926	1417,22	0,23	6,46E-01
ENSRNOG00000005708	**Mmp16**	matrix metallopeptidase 16 (Mmp16),	65205	5	36239487	36479703	240217	882,16	-0,32	6,46E-01
ENSRNOG00000001441	**Tmem120a**	transmembrane protein 120A (Tmem1	288591	12	25986848	25995509	8662	1643,58	0,29	6,46E-01
ENSRNOG00000015962	**Nmnat1**	nicotinamide nucleotide adenylyltransf	298653	5	170057407	170076545	19139	170,81	0,22	6,46E-01
ENSRNOG00000018454	**Apoe**	apolipoprotein E (Apoe), transcript var	25728	1	81878373	81881182	2810	121774,88	-0,43	6,46E-01
ENSRNOG00000046132		Uncharacterized protein [Source:UniProtKB/TrE		16	43725	45840	2116	48,26	-0,34	6,46E-01
ENSRNOG00000004805	**Stac2**	SH3 and cysteine rich domain 2 (Stac	363674	10	85782664	85799680	17017	84,09	-0,36	6,46E-01
ENSRNOG00000007346	**Grasp**	GRP1 (general receptor for phosphoin	192254	7	140670666	140678432	7767	64,77	-0,38	6,46E-01
ENSRNOG00000010158	**Magel2**	Protein Magel2 [Source:UniProtKB/Tr	679875	1	124153364	124157140	3777	159,95	-0,30	6,46E-01
ENSRNOG00000012366	**Mesdc2**	mesoderm development candidate 2 (	308796	1	146966069	146971740	5672	2374,00	0,27	6,46E-01
ENSRNOG00000020763		small nuclear ribonucleoprotein 70 (U	361574	1	102431123	102468582	37460	7858,48	0,29	6,46E-01
ENSRNOG00000016679	**Tmc7**	transmembrane channel-like 7 (Tmc7)	499254	1	195142160	195191670	49511	100,87	-0,28	6,47E-01
ENSRNOG00000021184	**Mark2**	MAP/microtubule affinity-regulating kir	60328	1	229515236	229578049	62814	1253,04	0,22	6,47E-01
ENSRNOG00000020325	**Calhm2**	calcium homeostasis modulator 2 (Cal	294019	1	274338780	274344504	5725	29,50	-0,37	6,47E-01
ENSRNOG00000018415	**Acot13**	acyl-CoA thioesterase 13 (Acot13), mI	291135	17	44108940	44121410	12471	254,11	-0,26	6,47E-01
ENSRNOG00000003980	**Ttc1**	tetratricopeptide repeat domain 1 (Ttc	287208	10	29182729	29208110	25382	1059,79	0,32	6,48E-01
ENSRNOG00000006549	**Fndc9**	Protein Fndc9; RCG33426 [Source:U	1E+08	10	31143625	31144305	681	53,82	0,32	6,48E-01
ENSRNOG00000015552	**Ppil4**	peptidylprolyl isomerase (cyclophilin)-l	361449	1	3593965	3626926	32962	626,57	0,29	6,48E-01

ENSRNOG00000018651	**Agtpbp1**	ATP/GTP binding protein 1 (Agtpbp1),	290986	17	7735244	7837762	102519	4197,58	-0,30	6,48E-01
ENSRNOG00000028390	**Hhipl1**	Protein Hhipl1 [Source:UniProtKB/TrE	362781	6	141389361	141410629	21269	229,08	-0,28	6,49E-01
ENSRNOG00000018627	**Plekhb1**	pleckstrin homology domain containin	64471	1	171867146	171881413	14268	625,27	-0,39	6,49E-01
ENSRNOG00000033433	**Csrnp1**	cysteine-serine-rich nuclear protein 1 (	363165	8	127861112	127867216	6105	64,51	-0,36	6,49E-01
ENSRNOG00000011281				2	124725185	124729965	4781	28,02	0,35	6,49E-01
ENSRNOG00000048166		Protein LOC679082 [Source:UniProtK	679082	1	268471457	268504174	32718	144,90	0,36	6,49E-01
ENSRNOG00000008274	**Xpc**	xeroderma pigmentosum, complemen	312560	4	187383715	187411243	27529	1122,38	0,29	6,49E-01
ENSRNOG00000021129	**RGD13084**	similar to RIKEN cDNA 4931406P16 (	308509	1	91240941	91270158	29218	1186,01	0,21	6,49E-01
ENSRNOG00000024780	**MGC95210**	uncharacterized protein LOC287798		287798	10	101879212	101890139	10928	265,09	-0,21	6,49E-01
ENSRNOG00000029903	**Spock3**	sparc/osteonectin, cwcv and kazal-like	306404	16	28243878	28585574	341697	704,49	-0,23	6,49E-01
ENSRNOG00000050857	**Fut10**	fucosyltransferase 10 (Fut10), mRNA [Source:R		16	64290558	64361358	70801	205,55	-0,28	6,49E-01
ENSRNOG00000005032	**Ppp1r12b**	protein phosphatase 1, regulatory sub	304813	13	56637005	56836014	199010	2174,19	-0,20	6,49E-01
ENSRNOG00000014746	**Dzip1l**	DAZ interacting zinc finger protein 1-lil	315952	8	107305974	107339593	33620	604,39	0,20	6,49E-01
ENSRNOG00000031126	**Tecta**	tectorin alpha (Tecta), mRNA [Source:	300653	8	45076279	45150292	74014	82,26	0,37	6,49E-01
ENSRNOG00000003104	**Trpv2**	transient receptor potential cation cha	29465	10	48686568	48707994	21427	728,04	0,25	6,49E-01
ENSRNOG00000003689	**Nono**	non-POU domain containing, octamer	317259	X	72175057	72192917	17861	8071,94	0,28	6,49E-01
ENSRNOG00000009104	**Fnip1**	folliculin interacting protein 1 (Fnip1), r	1E+08	10	39559860	39640089	80230	524,16	-0,35	6,49E-01
ENSRNOG00000012414	**Rhobtb3**	Rho-related BTB domain containing 3	309922	2	2626102	2681752	55651	515,51	-0,26	6,49E-01
ENSRNOG00000014904	**Ppp1r3e**	protein phosphatase 1, regulatory sub	691447	15	37422932	37424092	1161	45,46	-0,38	6,49E-01
ENSRNOG00000020442	**Ubl5**	ubiquitin-like 5 (Ubl5), mRNA [Source:	500954	8	21719386	21721099	1714	4683,17	0,28	6,49E-01
ENSRNOG00000021106	**Gramd1a**	GRAM domain containing 1A (Gramd1	361550	1	90718529	90740144	21616	3206,03	0,21	6,49E-01
ENSRNOG00000042411	**Rps6ka1**	ribosomal protein S6 kinase polypeptid	81771	5	155760612	155805563	44952	259,03	0,22	6,49E-01
ENSRNOG00000005289	**Prkd3**	protein kinase D3 (Prkd3), mRNA [Sou	313834	6	1537626	1604551	66926	628,20	-0,33	6,49E-01
ENSRNOG00000042729	**Efhc1**	EF-hand domain (C-terminal) containi	301295	9	25924344	25962902	38559	152,63	-0,42	6,49E-01
ENSRNOG00000008055	**Ccne2**	cyclin E2 (Ccne2), mRNA [Source:Ref	362485	5	29160461	29171995	11535	184,05	-0,35	6,49E-01
ENSRNOG00000008784	**Tmem106c**	transmembrane protein 106C (Tmem1	315286	7	139636746	139642341	5596	466,03	-0,27	6,49E-01
ENSRNOG00000013852		mediator complex subunit 20 (Med20)	316209	9	14303845	14314475	10631	934,98	0,26	6,49E-01
ENSRNOG00000014179	**LOC10036**	ribosomal protein S2 (Rps2), mRNA [S	83789	10	13903888	13905746	1859	3257,29	0,38	6,49E-01
ENSRNOG00000019243	**Glb1l**	galactosidase, beta 1-like (Glb1l), mR	301525	9	82169830	82180165	10336	340,22	-0,32	6,49E-01
ENSRNOG00000043388	**Irf3**	interferon regulatory factor 3 (Irf3), mR	292892	1	102056872	102061803	4932	616,46	-0,24	6,49E-01
ENSRNOG00000021017	**Car11**	carbonic anhydrase 11 (Car11), mRN	308588	1	102761012	102766123	5112	1687,22	0,20	6,50E-01
ENSRNOG00000021581	**Rapgef2**	Rap guanine nucleotide exchange fac	310533	2	197171957	197285983	114027	3051,80	0,24	6,50E-01
ENSRNOG00000031852		limbic system-associated membrane p	29561	11	62956510	63592532	636023	1334,30	-0,16	6,50E-01
ENSRNOG00000008951	**Rasl10a**	RAS-like, family 10, member A (Rasl1	364190	14	85989454	85990832	1379	81,00	0,28	6,50E-01
ENSRNOG00000018207	**Dynlt1**	dynein light chain Tctex-type 1 (Dynlt1	83462	1	48510105	48516959	6855	4063,61	0,28	6,50E-01
ENSRNOG00000020710	**Raver1**	ribonucleoprotein, PTB-binding 1 (Rav	298705	8	22148766	22168037	19272	2061,06	0,29	6,50E-01
ENSRNOG00000042601		zinc finger, GATA-like protein 1 [Sourc	1E+08	8	22138372	22142767	4396	38,29	0,36	6,50E-01
ENSRNOG00000007330	**Capzb**	capping protein (actin filament) muscl	298584	5	161383344	161482714	99371	7711,15	0,21	6,50E-01

ENSRNOG00000018816	**LOC10036**	cytochrome c oxidase, subunit Va (Co	252934	8	62075284	62087001	11718	2883,78	0,31	6,50E-01
ENSRNOG00000019971	**Kxd1**	KxDL motif containing 1 (Kxd1), mRN/	498606	16	20512578	20522058	9481	775,51	0,25	6,50E-01
ENSRNOG00000047399		Uncharacterized protein [Source:UniProtKB/TrE		X	123647195	123649299	2105	184,68	-0,22	6,50E-01
ENSRNOG00000018655	**Adsl**	adenylosuccinate lyase (Adsl), mRNA	315150	7	122148984	122176665	27682	1399,09	0,25	6,50E-01
ENSRNOG00000036695	**Mrpl12**	mitochondrial ribosomal protein L12 (M	303746	10	109251300	109255333	4034	2375,34	0,29	6,50E-01
ENSRNOG00000001064		sno, strawberry notch homolog 1 (Drosophila) [S		12	39464772	39520024	55253	1677,17	-0,26	6,50E-01
ENSRNOG00000005275	**Shmt1**	serine hydroxymethyltransferase 1 (sc	287379	10	46803226	46831051	27826	234,73	0,29	6,50E-01
ENSRNOG00000008298	**Dock7**	dedicator of cytokinesis 7 (Dock7), mF	313388	5	121537643	121694484	156842	2898,94	-0,20	6,50E-01
ENSRNOG00000010178	**Cgrrf1**	cell growth regulator with ring finger d	116679	15	27562651	27583110	20460	452,20	0,24	6,50E-01
ENSRNOG00000017321	**Trub1**	TruB pseudouridine (psi) synthase hor	361775	1	285685221	285786577	101357	305,02	0,31	6,50E-01
ENSRNOG00000018343	**Isca1**	iron-sulfur cluster assembly 1 (Isca1),	290985	17	7507059	7519770	12712	3498,06	0,27	6,50E-01
ENSRNOG00000021182	**Sv2a**	synaptic vesicle glycoprotein 2a (Sv2a	117559	2	217811917	217823542	11626	7399,37	0,30	6,50E-01
ENSRNOG00000024793	**Kctd21**	potassium channel tetramerization doi	499209	1	168528833	168529713	881	97,13	0,25	6,50E-01
ENSRNOG00000031325	**Abi1**	abl-interactor 1 (Abi1), mRNA [Source	79249	17	91617157	91698733	81577	2729,01	-0,21	6,50E-01
ENSRNOG00000009310	**Nmd3**	NMD3 ribosome export adaptor (Nmd3	310512	2	185759080	185784445	25366	370,18	-0,34	6,50E-01
ENSRNOG00000020155		mitogen-activated protein kinase 8 [Source:MGI		16	11583877	11662858	78982	1473,26	-0,30	6,50E-01
ENSRNOG00000050856		protocadherin alpha-6 [Source:RefSe	393088	18	29684456	29686888	2433	178,40	-0,33	6,50E-01
ENSRNOG00000021108	**Slc22a12**	solute carrier family 22 (organic anion/	365398	1	228902089	228909194	7106	291,76	0,26	6,51E-01
ENSRNOG00000005695	**Mgp**	matrix Gla protein (Mgp), mRNA [Sour	25333	4	235113772	235117160	3389	21,27	-0,41	6,51E-01
ENSRNOG00000016412	**Fxyd6**	FXYD domain-containing ion transport	63847	8	48323326	48326136	2811	1724,07	-0,33	6,51E-01
ENSRNOG00000003195		CASK interacting protein 1 (Caskin1),	140722	10	13671324	13691239	19916	3881,10	-0,20	6,51E-01
ENSRNOG00000006941	**Thumpd3**	THUMP domain containing 3 (Thumpc	500288	4	208284225	208307188	22964	439,12	-0,31	6,51E-01
ENSRNOG00000013036	**Epha8**	Ephrin type-A receptor 8 [Source:UniI	60589	5	159057429	159078493	21065	166,81	-0,38	6,51E-01
ENSRNOG00000015213		frataxin, mitochondrial [Source:RefSe	499335	1	249400582	249426597	26016	470,56	-0,36	6,51E-01
ENSRNOG00000016249	**Cep85**	centrosomal protein 85kDa (Cep85), n	362622	5	156045929	156165672	119744	531,25	0,21	6,51E-01
ENSRNOG00000026700		myomesin-1 [Source:RefSeq peptide;	316740	9	118808061	118922531	114471	17,76	-0,39	6,51E-01
ENSRNOG00000000307	**Mical1**	microtubule associated monooxygena	294520	20	47892301	47904250	11950	132,45	0,33	6,51E-01
ENSRNOG00000002255	**Fam162a**	family with sequence similarity 162, m	360721	11	70706159	70733730	27572	876,98	-0,24	6,51E-01
ENSRNOG00000048057		zinc finger protein 106 [Source:MGI Symbol;Acc		3	118830516	118868033	37518	2417,38	-0,19	6,52E-01
ENSRNOG00000050714	**LOC68654**	immunoglobulin superfamily containin	686545	8	62760799	62763036	2238	3125,87	0,33	6,52E-01
ENSRNOG00000016589	**Mlf2**	myeloid leukemia factor 2 (Mlf2), mRN	312709	4	224470532	224475061	4530	14277,40	0,29	6,52E-01
ENSRNOG00000028908	**Eppin**	epididymal peptidase inhibitor (Eppin),	685161	3	167222870	167229659	6790	18,35	0,39	6,52E-01
ENSRNOG00000012959	**Nab1**	Ngfi-A binding protein 1 (Nab1), mRN/	64824	9	53571213	53610614	39402	764,94	0,19	6,52E-01
ENSRNOG00000019235	**Rbm22**	RNA binding motif protein 22 (Rbm22)	307410	18	55061638	55072446	10809	1388,53	0,16	6,53E-01
ENSRNOG00000008932	**Ncapd3**	non-SMC condensin II complex, subu	315508	8	28095382	28164187	68806	682,63	0,29	6,53E-01
ENSRNOG00000027355	**Wdr36**	Protein Wdr36 [Source:UniProtKB/TrE	688637	18	25352752	25380806	28055	751,26	-0,22	6,53E-01
ENSRNOG00000008461	**Smdt1**	Protein RGD1311310; Putative unchai	315166	7	123563597	123565558	1962	4351,13	0,36	6,53E-01
ENSRNOG00000020607	**Bckdha**	branched chain ketoacid dehydrogena	25244	1	83684797	83713570	28774	1464,47	0,24	6,53E-01

ENSRNOG00000024410	**LOC68146**	flavin reductase [Source:RefSeq pept	292737	1	85468868	85491182	22315	542,23	-0,26	6,53E-01
ENSRNOG00000004413	**Exosc7**	exosome component 7 (Exosc7), mRN	316098	8	131357731	131383561	25831	336,77	0,27	6,53E-01
ENSRNOG00000009742	**Rad52**	RAD52 homolog (S. cerevisiae) (Rad5	297561	4	219523669	219536494	12826	157,82	-0,28	6,53E-01
ENSRNOG00000042432	**Nek11**	NIMA-related kinase 11 (Nek11), mRN	315978	8	113173030	113322634	149605	99,15	0,29	6,53E-01
ENSRNOG00000049979		protocadherin alpha-5 precursor [Sou	393087	18	29677700	29680045	2346	147,10	-0,23	6,53E-01
ENSRNOG00000000549	**Tspyl1**	TSPY-like 1 (Tspyl1), mRNA [Source:F	29544	20	42813240	42815798	2559	4647,84	0,31	6,53E-01
ENSRNOG00000000145	**Pik3r3**	phosphoinositide-3-kinase, regulatory	60664	5	138859028	138926835	67808	684,24	0,35	6,53E-01
ENSRNOG00000002196	**Ociad2**	OCIA domain containing 2 (Ociad2), m	1E+08	14	37254040	37263149	9110	94,29	-0,36	6,53E-01
ENSRNOG00000007405		Protein Krba1 [Source:UniProtKB/TrE	362358	4	142688497	142709419	20923	841,99	0,20	6,53E-01
ENSRNOG00000010711	**Dicer1**	Protein Dicer1; RCG20888 [Source:U	299284	6	137590054	137632237	42184	1534,83	0,19	6,53E-01
ENSRNOG00000020044	**Mob2**	MOB kinase activator 2 (Mob2), mRN/	499288	1	221867617	221924073	56457	553,13	0,17	6,53E-01
ENSRNOG00000020995	**Fut1**	fucosyltransferase 1 (Fut1), mRNA [Sc	81919	1	102677798	102681216	3419	18,57	-0,39	6,53E-01
ENSRNOG00000024799	**Cd93**	CD93 molecule (Cd93), mRNA [Sourc	84398	3	149189170	149193959	4790	26,29	-0,40	6,53E-01
ENSRNOG00000026049	**Qrsl1**	glutaminyl-tRNA synthase (glutamine-l	309911	20	50498408	50523506	25099	209,58	0,29	6,53E-01
ENSRNOG00000046536	**LOC69070**	Protein LOC690700 [Source:UniProtKB/TrEMBl		14	94385563	94397132	11570	22,80	-0,37	6,53E-01
ENSRNOG00000050413	**Samd15**	sterile alpha motif domain containing 1	681908	6	120506948	120526577	19630	54,09	-0,31	6,53E-01
ENSRNOG00000050437		biorientation of chromosomes in cell d	207118	14	73758054	73804679	46626	1256,24	0,33	6,53E-01
ENSRNOG00000002652	**Rap1gap2**	RAP1 GTPase activating protein 2 (Ra	303298	10	60909166	60963561	54396	1414,26	-0,24	6,54E-01
ENSRNOG00000004814	**Tceb2**	transcription elongation factor B (SIII),	81807	10	12983021	12988206	5186	2279,00	0,34	6,54E-01
ENSRNOG00000017441	**Tpm3_v1**	tropomyosin 3 (Tpm3), transcript varia	117557	2	208863073	208884376	21304	6940,40	0,23	6,54E-01
ENSRNOG00000026124	**Id3**	inhibitor of DNA binding 3 (Id3), mRNA	25585	5	158263301	158264888	1588	3575,78	-0,43	6,54E-01
ENSRNOG00000042414	**Nat14**	N-acetyltransferase 14 (Nat14), mRNA	361500	1	76071585	76073672	2088	1310,39	0,30	6,54E-01
ENSRNOG00000020021		BR serine/threonine kinase 2 [Source:MGI Symb		1	221840644	221860773	20130	1709,77	0,29	6,54E-01
ENSRNOG00000024526	**Ttc34**	Protein Ttc34 [Source:UniProtKB/TrE	366517	5	175715172	175724866	9695	25,33	-0,38	6,54E-01
ENSRNOG00000050509	**Ebi3**	Epstein-Barr virus induced 3 (Ebi3), m	680609	9	10096038	10100007	3970	63,38	-0,35	6,54E-01
ENSRNOG00000050649	**Tvp23b**	trans-golgi network vesicle protein 23	687358	10	49077748	49089023	11276	1108,07	0,21	6,54E-01
ENSRNOG00000011480	**Tubgcp5**	tubulin, gamma complex associated p	308663	1	115192961	115228228	35268	354,64	-0,34	6,54E-01
ENSRNOG00000010463	**Cpa4**	carboxypeptidase A4 (Cpa4), mRNA [S	502736	4	57644770	57667613	22844	20,73	0,44	6,54E-01
ENSRNOG00000020513	**Becn1**	beclin 1, autophagy related (Becn1), tr	114558	10	89008976	89024328	15353	2550,88	0,20	6,54E-01
ENSRNOG00000021140	**Kcnk4**	potassium channel, subfamily K, mem	116489	1	229174130	229180458	6329	27,33	-0,35	6,54E-01
ENSRNOG00000014519	**Slc35g3**	solute carrier family 35, member G3 (S	691976	10	56112009	56113760	1752	37,44	-0,34	6,55E-01
ENSRNOG00000015226		mannosyl-oligosaccharide 1,2-alpha-n	295319	2	222338553	222489949	151397	1229,22	-0,22	6,55E-01
ENSRNOG00000027923	**Mynn**	myoneurin (Mynn), mRNA [Source:Re	361924	2	136092041	136107672	15632	382,47	-0,29	6,55E-01
ENSRNOG00000006777	**Mboat2**	membrane bound O-acyltransferase d	313997	6	60881777	61003918	122142	1655,58	-0,25	6,55E-01
ENSRNOG00000011096	**LOC68858**	high mobility group box 3 (Hmgb3), ml	305373	14	51617774	51622052	4279	35,40	-0,33	6,55E-01
ENSRNOG00000015473	**Phactr2**	phosphatase and actin regulator 2 (Ph	308291	1	8990542	9111369	120828	61,62	-0,32	6,55E-01
ENSRNOG00000032192	**RGD13073**	similar to RIKEN cDNA 4933411K20 (l	306469	16	48994780	49032961	38182	180,96	-0,40	6,55E-01
ENSRNOG00000037247	**Rras**	related RAS viral (r-ras) oncogene hor	361568	1	102077759	102081668	3910	474,40	0,36	6,55E-01

ENSRNOG00000019244	**Mxra8**	matrix-remodelling associated 8 (Mxra	313770	5	176764184	176768533	4350	591,51	-0,38	6,55E-01
ENSRNOG00000004891	**Arl14ep**	ADP-ribosylation factor-like 14 effecto	311279	3	104597234	104606970	9737	354,39	-0,22	6,55E-01
ENSRNOG00000011052	**Gars**	glycyl-tRNA synthetase (Gars), mRNA	297113	4	149892437	149933270	40834	5703,02	0,23	6,55E-01
ENSRNOG00000009957		solute carrier family 25 (mitochondrial	362322	4	31041996	31598338	556343	103,08	0,35	6,55E-01
ENSRNOG00000019638	**Lmna**	lamin A/C (Lmna), transcript variant 2, mRNA [S		2	207245239	207265869	20631	1573,74	0,25	6,55E-01
ENSRNOG00000038686	**Ap1s2**	adaptor-related protein complex 1, sig	302671	X	32691541	32717097	25557	892,18	-0,31	6,55E-01
ENSRNOG00000000519	**Stk38**	serine/threonine kinase 38 (Stk38), ml	361813	20	8472800	8506129	33330	1570,65	0,21	6,55E-01
ENSRNOG00000050404	**Pmepa1**	prostate transmembrane protein, andr	311676	3	177360059	177366886	6828	307,47	0,33	6,55E-01
ENSRNOG00000000321	**Cd24**	CD24 molecule (Cd24), mRNA [Sourc	25145	20	49989320	49994626	5307	7654,33	0,33	6,55E-01
ENSRNOG00000003612	**Axin2**	axin 2 (Axin2), mRNA [Source:RefSeq	29134	10	96933420	96959744	26325	288,46	0,19	6,55E-01
ENSRNOG00000008137	**Cdk18**	cyclin-dependent kinase 18 (Cdk18), r	289019	13	53964633	53972998	8366	27,73	-0,44	6,55E-01
ENSRNOG00000011285	**Zdhhc22**	zinc finger, DHHC-type containing 22 (	299211	6	120299219	120306482	7264	41,59	-0,37	6,55E-01
ENSRNOG00000017310	**Rnpc3**	RNA-binding region (RNP1, RRM) cor	691538	2	234574804	234603133	28330	308,72	-0,33	6,55E-01
ENSRNOG00000018604	**Tufm**	Tu translation elongation factor, mitocl	293481	1	204882342	204885949	3608	2262,73	0,19	6,55E-01
ENSRNOG00000050145		Protein Ahnak [Source:UniProtKB/TrEMBL;Acc:		1	232122559	232151281	28723	318,92	-0,42	6,55E-01
ENSRNOG00000003028		Protein Dnahc17 [Source:UniProtKB/	287845	10	106667437	106770733	103297	99,46	0,27	6,55E-01
ENSRNOG00000019980	**Tomm20**	translocase of outer mitochondrial mei	266601	19	70530583	70540402	9820	2764,64	0,22	6,55E-01
ENSRNOG00000021303	**Lrrc48**	leucine rich repeat containing 48 (Lrrc	287371	10	46477106	46517577	40472	271,32	-0,29	6,55E-01
ENSRNOG00000004433	**Kdm5b**	lysine (K)-specific demethylase 5B (Kc	304809	13	56438963	56509593	70631	5266,70	0,24	6,56E-01
ENSRNOG00000005123	**Emc2**	ER membrane protein complex subun	362905	7	82339014	82385900	46887	539,32	-0,40	6,56E-01
ENSRNOG00000013631	**Slc31a2**	solute carrier family 31 (copper transp	298091	5	82242402	82252907	10506	117,93	0,34	6,56E-01
ENSRNOG00000022343	**Alms1**	Alstrom syndrome 1 (Alms1), mRNA [S	297408	4	181947469	182048235	100767	507,32	0,32	6,56E-01
ENSRNOG00000032929	**Incenp**	inner centromere protein (Incenp), mR	293733	1	232874537	232900880	26344	799,15	0,27	6,56E-01
ENSRNOG00000012742	**Irx2**	iroquois homeobox 2 (Irx2), mRNA [Sc	306657	1	34682802	34688217	5416	34,84	0,40	6,56E-01
ENSRNOG00000027277	**Pcdhga8**	protocadherin gamma subfamily A, 8 (	364843	18	30569100	30571661	2562	267,20	-0,23	6,56E-01
ENSRNOG00000000576	**Anapc16**	Protein Anapc16; RCG22080, isoform	1E+08	20	31349977	31364636	14660	855,49	0,21	6,57E-01
ENSRNOG00000004794		Reticulon-1 [Source:UniProtKB/Swiss-Prot;Acc:		6	104395869	104615205	219337	39392,99	0,24	6,57E-01
ENSRNOG00000007419	**Pank3**	pantothenate kinase 3 (Pank3), mRNA	360511	10	20465020	20484497	19478	2887,35	-0,27	6,57E-01
ENSRNOG00000010396	**Eya3**	eyes absent homolog 3 (Drosophila) (I	313027	5	154501299	154581358	80060	1028,20	0,19	6,57E-01
ENSRNOG00000016360	**Gps2**	G protein pathway suppressor 2 (Gps2	497941	10	56269587	56272106	2520	626,77	0,17	6,57E-01
ENSRNOG00000016635	**LOC36164**	RIKEN cDNA D430042O09 gene [Sou	361646	1	204112871	204249196	136326	552,60	0,26	6,57E-01
ENSRNOG00000016823	**Skp2**	S-phase kinase-associated protein 2,	294790	2	77593581	77621639	28059	712,11	0,21	6,57E-01
ENSRNOG00000016948	**Nhlrc2**	NHL repeat containing 2 (Nhlrc2), mRI	307986	1	284736412	284796700	60289	491,84	-0,22	6,57E-01
ENSRNOG00000019544	**Zbtb2**	zinc finger and BTB domain containing	308126	1	42260208	42267107	6900	382,85	-0,21	6,57E-01
ENSRNOG00000031299	**Rps10l1**	40S ribosomal protein S10 [Source:U	81773	6	84968907	84969452	546	56,74	0,36	6,57E-01
ENSRNOG00000018648	**Mppe1**	metallophosphoesterase 1 (Mppe1), m	361344	18	62133743	62151276	17534	218,31	-0,26	6,57E-01
ENSRNOG00000017002	**Adrb1**	adrenoceptor beta 1 (Adrb1), mRNA [S	24925	1	284917872	284919272	1401	126,32	0,26	6,57E-01
ENSRNOG00000029726	**Gstm1**	glutathione S-transferase mu 1 (Gstm	24423	2	230272746	230278303	5558	4216,57	-0,40	6,57E-01

ENSRNOG00000003703	**Mcm6**	minichromosome maintenance comple	29685	13	50127493	50152683	25191	1577,59	0,18	6,57E-01
ENSRNOG00000008981	**Pdcd6ip**	programmed cell death 6 interacting p	501083	8	121231794	121286785	54992	2611,42	0,18	6,57E-01
ENSRNOG00000042044	**Stard3**	StAR-related lipid transfer (START) dc	363675	10	86110514	86133022	22509	1408,42	0,22	6,57E-01
ENSRNOG00000004932	**Sel1l3**	Protein Sel1l3 [Source:UniProtKB/TrE	360945	14	60243671	60348547	104877	506,30	-0,30	6,57E-01
ENSRNOG00000020207	**Has3**	hyaluronan synthase 3 (Has3), mRNA	266805	19	49928282	49937713	9432	264,43	-0,27	6,57E-01
ENSRNOG00000036701	**Potef**	actin, gamma 1 (Actg1), mRNA [Source:RefSeq		10	109113707	109116306	2600	28,69	0,39	6,57E-01
ENSRNOG00000010652	**Dock6**	dedicator of cytokinesis 6 (Dock6), mF	367039	8	22876566	22928936	52371	217,49	-0,35	6,58E-01
ENSRNOG00000010805	**Fabp4**	fatty acid binding protein 4, adipocyte	79451	2	113547680	113552343	4664	21,62	-0,43	6,58E-01
ENSRNOG00000014548	**Nedd9**	neural precursor cell expressed, devel	291044	17	25084201	25143750	59550	519,38	-0,26	6,58E-01
ENSRNOG00000019321	**Cck**	cholecystokinin (Cck), mRNA [Source:	25298	8	129312786	129319370	6585	2511,44	-0,33	6,58E-01
ENSRNOG00000021870	**Slco4a1**	solute carrier organic anion transporte	171144	3	181259389	181278378	18990	123,42	-0,31	6,58E-01
ENSRNOG00000029667	**Tbce**	tubulin folding cofactor E (Tbce), mRN	361255	17	51676788	51723129	46342	579,29	-0,26	6,58E-01
ENSRNOG00000029778	**Maob**	monoamine oxidase B (Maob), mRNA	25750	X	7249718	7352279	102562	238,75	-0,44	6,58E-01
ENSRNOG00000005450	**Lsm11**	LSM11, U7 small nuclear RNA associa	501688	10	30847784	30862999	15216	1547,41	-0,33	6,58E-01
ENSRNOG00000014786	**Ccne1**	cyclin E1 (Ccne1), mRNA [Source:Ref	25729	1	95575138	95584286	9149	395,78	0,30	6,58E-01
ENSRNOG00000011633	**Parvb**	parvin, beta (Parvb), mRNA [Source:R	362973	7	63181011	63267688	86678	1755,94	-0,37	6,59E-01
ENSRNOG00000008222		translocase of inner mitochondrial mer	171139	6	103015075	103027902	12828	629,10	-0,20	6,59E-01
ENSRNOG00000019573	**Lcat**	lecithin cholesterol acyltransferase (Lc	24530	19	48780369	48783847	3479	182,26	-0,43	6,59E-01
ENSRNOG00000020194	**Hes6**	hairy and enhancer of split 6 (Drosoph	316626	9	98225753	98227458	1706	6981,97	0,24	6,59E-01
ENSRNOG00000013393	**Eif2a**	eukaryotic translation initiation factor 2	502531	2	168140000	168173620	33621	502,70	-0,24	6,59E-01
ENSRNOG00000018591	**Rabepk**	Rab9 effector protein with kelch motifs	296649	3	19164931	19185412	20482	858,30	0,19	6,59E-01
ENSRNOG00000050410	**Snrpd3**	Protein LOC687711; RCG60635, isofc	687711	20	16266053	16279075	13023	1287,06	0,22	6,59E-01
ENSRNOG00000018129	**LOC68388**	acyl carrier protein, mitochondrial [So	293453	1	199110335	199120094	9760	2748,23	0,30	6,60E-01
ENSRNOG00000046056		Uncharacterized protein [Source:UniProtKB/TrE		1	64190273	64200165	9893	17,47	-0,43	6,60E-01
ENSRNOG00000001313	**Gnaz**	guanine nucleotide binding protein (G	25740	20	16776281	16801749	25469	1308,74	-0,23	6,60E-01
ENSRNOG00000011317	**Pkn2**	protein kinase N2 (Pkn2), mRNA [Sou	207122	2	267265825	267370861	105037	877,83	-0,26	6,60E-01
ENSRNOG00000012777	**Nudt19**	nudix (nucleoside diphosphate linked i	308518	1	92948067	92959663	11597	370,60	0,23	6,60E-01
ENSRNOG00000002914	**Trmt1**	tRNA methyltransferase 1 homolog (S	288914	19	36778905	36789062	10158	1358,98	0,18	6,60E-01
ENSRNOG00000008815	**Fbxo46**	F-box protein 46 (Fbxo46), mRNA [So	292686	1	81296298	81312721	16424	616,64	0,19	6,60E-01
ENSRNOG00000025890		optic atrophy 3 protein homolog [Sour	308409	1	81424764	81427718	2955	1589,39	0,27	6,60E-01
ENSRNOG00000005917	**Pawr**	PRKC, apoptosis, WT1, regulator (Pa\	64513	7	51286670	51365775	79106	222,03	0,23	6,60E-01
ENSRNOG00000012716	**Chd2**	chromodomain helicase DNA binding		308738	1	135785356	135895466	110111	1391,71	-0,18	6,60E-01
ENSRNOG00000039017	**Naa50**	N(alpha)-acetyltransferase 50, NatE c	288108	11	65569908	65637729	67822	279,20	0,21	6,60E-01
ENSRNOG00000006975	**Wasl**	Wiskott-Aldrich syndrome-like (Wasl),	682507	4	51455978	51504468	48491	1651,14	-0,27	6,61E-01
ENSRNOG00000020772	**Dhx8**	DEAH (Asp-Glu-Ala-His) box polypept	287727	10	89442421	89479672	37252	1334,91	0,21	6,61E-01
ENSRNOG00000020576	**Catsperg1**	cation channel, sperm-associated, gar	292767	1	89307167	89337459	30293	21,26	-0,43	6,61E-01
ENSRNOG00000000066	**Fbxo28**	F-box protein 28 (Fbxo28), mRNA [So	305105	13	105649130	105674598	25469	886,32	0,26	6,61E-01
ENSRNOG00000006642	**Yipf6**	Yip1 domain family, member 6 (Yipf6),	363476	X	69500967	69509223	8257	369,69	-0,23	6,61E-01

ENSRNOG00000009253	**Igsf9b**	Protein Igsf9b [Source:UniProtKB/TrE	315510	8	28371761	28406211	34451	624,71	-0,26	6,61E-01
ENSRNOG00000012089		cleavage stimulation factor subunit 3	362178	3	101210946	101228401	17456	355,01	-0,30	6,61E-01
ENSRNOG00000020994	**Slc25a39**	solute carrier family 25, member 39 (S	360636	10	90140265	90144607	4343	1780,32	0,25	6,61E-01
ENSRNOG00000029377	**LOC69046**	60S ribosomal protein L38 [Source:U	682793	8	62894575	62894787	213	24,47	0,39	6,61E-01
ENSRNOG00000047931	**LOC10091**	thymosin, beta 4, X chromosome (Tm	81814	X	28985069	28987071	2003	66551,43	0,34	6,61E-01
ENSRNOG00000047711		low density lipoprotein receptor-related protein 2		3	62271279	62359283	88005	524,19	-0,30	6,61E-01
ENSRNOG00000010379	**Celf1**	CUGBP, Elav-like family member 1 (C	362160	3	86477674	86553296	75623	5564,59	-0,19	6,62E-01
ENSRNOG00000024445		SLX4 structure-specific endonuclease	302953	10	10544331	10562666	18336	1489,09	0,25	6,62E-01
ENSRNOG00000003787		Protein Lcorl [Source:UniProtKB/TrE	498385	14	69844168	69909139	64972	142,18	-0,40	6,62E-01
ENSRNOG00000004200	**Sybu**	Protein Sybu [Source:UniProtKB/TrE	500865	7	83615448	83685901	70454	4988,89	-0,20	6,62E-01
ENSRNOG00000006479	**Lama5**	laminin, alpha 5 (Lama5), mRNA [Sou	140433	3	181517512	181565641	48130	1179,57	-0,34	6,62E-01
ENSRNOG00000009592	**Cyb5r3**	cytochrome b5 reductase 3 (Cyb5r3),	25035	7	124006942	124024519	17578	3580,01	-0,23	6,62E-01
ENSRNOG00000011584	**Gpatch1**	G patch domain containing 1 (Gpatch	292810	1	92659066	92707992	48927	394,85	0,23	6,62E-01
ENSRNOG00000012624	**Dnah5**	Protein Dnah5 [Source:UniProtKB/TrE	294854	2	100825559	101000916	175358	35,81	-0,36	6,62E-01
ENSRNOG00000014902				1	8676472	8678091	1620	32,82	0,41	6,62E-01
ENSRNOG00000016246	**Tshz1**	Protein Tshz1 [Source:UniProtKB/TrE	307217	18	79912225	79915437	3213	1623,33	-0,23	6,62E-01
ENSRNOG00000020407	**Atcay**	ataxia, cerebellar, Cayman type (Atca	362826	7	11523425	11547188	23764	6538,97	0,28	6,62E-01
ENSRNOG00000023480	**Nabp2**	nucleic acid binding protein 2 (Nabp2)	362813	7	2798471	2804072	5602	2947,36	0,21	6,62E-01
ENSRNOG00000030127	**Eml2**	echinoderm microtubule associated pr	192360	1	81353763	81386605	32843	1733,50	0,26	6,62E-01
ENSRNOG00000033609	**Irx1**	iroquois homeobox 1 (Irx1), mRNA [S	306659	1	35319895	35325724	5830	79,42	-0,44	6,62E-01
ENSRNOG00000048431		NYN domain and retroviral integrase containing		15	38408648	38428231	19584	672,11	0,30	6,62E-01
ENSRNOG00000048751		rno-mir-127 [Source:miRBase;Acc:MI0000899]		6	142873331	142879024	5694	34,06	0,44	6,62E-01
ENSRNOG00000049057	**Znrf2**	zinc and ring finger 2 (Znrf2), mRNA [	362367	4	149666569	149748158	81590	2492,19	-0,25	6,62E-01
ENSRNOG00000013583	**Tbc1d8**	TBC1 domain family, member 8 (Tbc1	680133	9	45778865	45893709	114845	349,45	-0,19	6,62E-01
ENSRNOG00000000095	**Gpr89b**	G protein-coupled receptor 89B (Gpr8	362003	2	218485504	218523850	38347	988,61	0,27	6,62E-01
ENSRNOG00000014521	**Armc8**	armadillo repeat containing 8 (Armc8),	315949	8	107148284	107243975	95692	1148,80	0,29	6,62E-01
ENSRNOG00000028348	**Lhx8**	LIM homeobox 8 (Lhx8), mRNA [Sour	365963	2	279235994	279258253	22260	22,88	0,41	6,62E-01
ENSRNOG00000010216		von Willebrand factor A domain contai	290381	15	64847113	65131503	284391	886,75	-0,31	6,62E-01
ENSRNOG00000047319		caprin family member 2 [Source:MGI S	686779	4	247436446	247503639	67194	179,83	0,31	6,62E-01
ENSRNOG00000050461	**Fam166b**	Protein LOC100365928 [Source:UniP	1E+08	5	63407888	63408724	837	19,39	-0,43	6,62E-01
ENSRNOG00000010536	**Nr2c2**	nuclear receptor subfamily 2, group C,	50659	4	189231911	189256038	24128	1393,49	-0,24	6,62E-01
ENSRNOG00000012853	**Tk2**	thymidine kinase 2, mitochondrial (Tk2	291824	19	918115	940133	22019	840,93	0,27	6,62E-01
ENSRNOG00000003696	**Prkx**	protein kinase, X-linked (Prkx), mRNA	501563	X	45378202	45420133	41932	1323,16	0,25	6,62E-01
ENSRNOG00000014506				18	13800603	13801131	529	65,85	0,34	6,62E-01
ENSRNOG00000008956	**Cdkn2c**	cyclin-dependent kinase inhibitor 2C (	54238	5	133181576	133186731	5156	224,10	0,24	6,63E-01
ENSRNOG00000000511		serine/threonine-protein kinase SRPK	361811	20	7867103	7900809	33707	1783,23	0,25	6,63E-01
ENSRNOG00000013434	**Exosc8**	exosome component 8 (Exosc8), mRN	295050	2	160331349	160337851	6503	132,22	-0,28	6,63E-01
ENSRNOG00000014844	**Kif21a**	Protein Kif21a [Source:UniProtKB/TrE	300158	7	131746524	131874044	127521	2092,83	0,15	6,63E-01

ENSRNOG00000024536	**Ccbe1**	Protein Ccbe1 [Source:UniProtKB/TrE	361341	18	60953849	60983423	29575	62,35	-0,35	6,63E-01
ENSRNOG00000002781	**Kdsr**	3-ketodihydrosphingosine reductase (I	360833	13	31929862	31962747	32886	742,55	0,37	6,63E-01
ENSRNOG00000006326	**Atraid**	all-trans retinoic acid-induced different	298841	6	36495621	36500202	4582	1423,51	0,19	6,63E-01
ENSRNOG00000016886	**Rnf25**	ring finger protein 25 (Rnf25), mRNA [	301515	9	81635676	81642487	6812	838,70	0,21	6,63E-01
ENSRNOG00000011747	**Tmem205**	transmembrane protein 205 (Tmem20	300441	8	22986957	22992693	5737	746,12	0,34	6,63E-01
ENSRNOG00000023778	**Gcnt2**	glucosaminyl (N-acetyl) transferase 2,	306860	17	23615673	23657985	42313	36,28	-0,41	6,63E-01
ENSRNOG00000024027	**Lemd3**	LEM domain containing 3 (Lemd3), ml	680066	7	62979066	63028458	49393	1032,16	0,33	6,63E-01
ENSRNOG00000050819	**Birc5**	baculoviral IAP repeat-containing 5 (B	64041	10	106494775	106503091	8317	446,81	0,32	6,63E-01
ENSRNOG00000020393	**Rhog**	ras homolog family member G (Rhog)	308875	1	173524755	173536314	11560	420,97	0,26	6,63E-01
ENSRNOG00000007037	**LOC67964**	WD repeat domain 65 [Source:MGI Sy	679641	5	141170381	141244598	74218	84,96	0,31	6,64E-01
ENSRNOG00000012490	**LOC10091**	amphiphysin (Amph), mRNA [Source:I	60668	17	46357604	46621763	264160	1165,46	-0,28	6,64E-01
ENSRNOG00000021006	**Tmem147**	transmembrane protein 147 (Tmem14	292792	1	90332928	90334759	1832	1796,84	0,24	6,64E-01
ENSRNOG00000021209	**Polr3gl**	polymerase (RNA) III (DNA directed) p	690254	2	218189916	218206136	16221	411,50	0,20	6,64E-01
ENSRNOG00000005659	**Aurkb**	aurora kinase B (Aurkb), mRNA [Sour	114592	10	55369628	55375183	5556	575,77	0,28	6,64E-01
ENSRNOG00000009761	**Tmod1**	tropomodulin 1 (Tmod1), mRNA [Sour	25566	5	66173406	66228499	55094	109,35	-0,24	6,64E-01
ENSRNOG00000010747	**Dap**	death-associated protein (Dap), mRN/	64322	2	103949819	104002894	53076	548,50	-0,30	6,64E-01
ENSRNOG00000046980	**LOC10091**	Coronin [Source:UniProtKB/TrEMBL;/	1E+08	10	10136001	10147417	11417	195,88	0,22	6,64E-01
ENSRNOG00000047295	**Prr22**	proline rich 22 (Prr22), mRNA [Source	680177	9	9284181	9286000	1820	44,44	0,33	6,65E-01
ENSRNOG00000002154	**Mepe**	matrix extracellular phosphoglycoprote	79110	14	6770084	6781630	11547	22,58	0,37	6,65E-01
ENSRNOG00000016358	**Apba2**	amyloid beta (A4) precursor protein-bi	83610	1	126475687	126655813	180127	4223,19	-0,21	6,65E-01
ENSRNOG00000011799		Coiled-coil domain-containing-like; Pro	300442	8	22992813	23001006	8194	85,20	-0,28	6,65E-01
ENSRNOG00000006766	**Laptm4b**	lysosomal protein transmembrane 4 b	315047	7	73094975	73138156	43182	1182,92	-0,27	6,66E-01
ENSRNOG00000008984		Interleukin-1 receptor-associated kinase 1 bindir		8	89871572	89885291	13720	222,84	0,23	6,66E-01
ENSRNOG00000015105	**Bspry**	B-box and SPRY domain containing (E	64027	5	82453655	82475831	22177	134,94	0,34	6,66E-01
ENSRNOG00000028403	**Ptcd2**	pentatricopeptide repeat domain 2 (Pt	310025	2	48732577	48760245	27669	314,65	0,33	6,66E-01
ENSRNOG00000038087		predicted gene 10676 [Source:MGI Symbol;Acc:		1	81159825	81160289	465	23,39	0,36	6,67E-01
ENSRNOG00000050321	**Zzz3**	zinc finger, ZZ-type containing 3 (Zzz3	310958	2	276312459	276347991	35533	545,67	-0,30	6,67E-01
ENSRNOG00000003259	**C1qtnf1**	C1q and tumor necrosis factor related	303701	10	107090742	107112156	21415	59,82	-0,39	6,67E-01
ENSRNOG00000010496	**Med17**	mediator complex subunit 17 (Med17)	300367	8	13759090	13774832	15743	617,14	0,29	6,67E-01
ENSRNOG00000017947		Uncharacterized protein [Source:UniProtKB/TrE		17	58827366	58862470	35105	204,39	0,30	6,67E-01
ENSRNOG00000023896	**Dusp6**	dual specificity phosphatase 6 (Dusp6	116663	7	41506397	41510626	4230	838,41	0,26	6,67E-01
ENSRNOG00000042620	**Marveld1**	MARVEL domain containing 1 (Marvel	309375	1	268843550	268845919	2370	49,80	-0,38	6,67E-01
ENSRNOG00000001057	**Ctxn1**	cortexin 1 (Ctxn1), mRNA [Source:Ref	29145	12	4687159	4688769	1611	7757,26	0,30	6,67E-01
ENSRNOG00000002373	**Akap1**	A kinase (PRKA) anchor protein 1 (Ak	114124	10	73940526	73959662	19137	1728,67	0,26	6,67E-01
ENSRNOG00000009134	**Mad2l2**	MAD2 mitotic arrest deficient-like 2 (ye	313702	5	168609827	168614293	4467	729,93	0,21	6,67E-01
ENSRNOG00000010659	**Gabpb1**	GA binding protein transcription factor,	499883	3	125617857	125645734	27878	207,12	-0,25	6,67E-01
ENSRNOG00000019424	**Aspdh**	aspartate dehydrogenase domain con	292875	1	101536453	101538714	2262	107,23	0,28	6,67E-01
ENSRNOG00000009614	**LOC300249**			7	141240894	141242157	1264	23,96	0,38	6,67E-01

ENSRNOG00000003018	**Olfml2b**	olfactomedin-like 2B (Olfml2b), mRNA	304960	13	93609913	93647084	37172	120,26	-0,37	6,68E-01
ENSRNOG00000003743	**Dars**	aspartyl-tRNA synthetase (Dars), mRN	116483	13	50158684	50212425	53742	1324,85	-0,32	6,68E-01
ENSRNOG00000000493	**LOC68856**	small nuclear ribonucleoprotein polype	361808	20	9611426	9629603	18178	239,55	0,24	6,68E-01
ENSRNOG00000015669	**Kctd11**	potassium channel tetramerization dor	363634	10	56235372	56236070	699	19,83	-0,40	6,68E-01
ENSRNOG00000001175	**Unc119b**	unc-119 homolog B (C. elegans) (Unc	288702	12	49034761	49047383	12623	1796,07	0,20	6,68E-01
ENSRNOG00000014713	**Csgalnact**	chondroitin sulfate N-acetylgalactosan	297554	4	216081812	216113012	31201	108,65	-0,33	6,68E-01
ENSRNOG00000036662	**Wdr45b**	WD repeat domain 45B (Wdr45b), mR	360682	10	110111643	110141197	29555	1363,64	0,25	6,68E-01
ENSRNOG00000023407	**Mrpl36**	mitochondrial ribosomal protein L36 (M	364656	1	32688645	32689707	1063	73,75	0,36	6,68E-01
ENSRNOG00000046049				6	84457699	84457949	251	167,81	-0,25	6,68E-01
ENSRNOG00000002610	**Carhsp1**	calcium regulated heat stable protein 1	260416	10	5842867	5856458	13592	4266,26	0,24	6,68E-01
ENSRNOG00000032630	**Mrps28**	mitochondrial ribosomal protein S28 (M	689025	2	114822876	114826124	3249	141,60	0,30	6,68E-01
ENSRNOG00000000392	**Supv3l1**	suppressor of var1, 3-like 1 (S. cerevis	294385	20	33844946	33864105	19160	850,04	0,22	6,69E-01
ENSRNOG00000004595	**Pou6f1**	POU class 6 homeobox 1 (Pou6f1), m	116545	7	140008707	140015389	6683	609,35	0,20	6,69E-01
ENSRNOG00000010883	**Pard6b**	par-6 (partitioning defective 6) homolo	362279	3	170968975	170990259	21285	313,22	0,24	6,69E-01
ENSRNOG00000016773	**Sap130**	Protein Sap130 [Source:UniProtKB/Tr	307527	18	24138859	24166083	27225	469,42	0,22	6,69E-01
ENSRNOG00000019743		transmembrane protein 63b [Source:M	363197	9	16593695	16613274	19580	3543,12	0,15	6,69E-01
ENSRNOG00000025589	**Jph4**	junctophilin 4 (Jph4), mRNA [Source:F	445271	15	37655680	37662194	6515	2328,11	-0,26	6,69E-01
ENSRNOG00000026115				1	206346140	206349456	3317	623,19	0,26	6,69E-01
ENSRNOG00000027944	**Pnmal1**	paraneoplastic Ma antigen family-like	361515	1	80140829	80143488	2660	435,96	-0,24	6,69E-01
ENSRNOG00000049032				10	34918584	34918848	265	177,87	-0,27	6,69E-01
ENSRNOG00000004681	**Eny2**	enhancer of yellow 2 homolog (Drosop	685258	7	83362801	83371599	8799	1541,91	-0,18	6,69E-01
ENSRNOG00000026295	**Rbpjl**	recombination signal binding protein fo	362268	3	167032450	167044820	12371	73,47	-0,35	6,69E-01
ENSRNOG00000027880		Protein Nbeal2-ps1 [Source:UniProtK	316014	8	118235182	118264997	29816	252,30	-0,25	6,69E-01
ENSRNOG00000017352	**Ctdnep1**	CTD nuclear envelope phosphatase 1	287447	10	56336411	56345351	8941	2071,76	0,19	6,69E-01
ENSRNOG00000028656	**Ccdc108**	Protein Ccdc108 [Source:UniProtKB/T	301521	9	81931773	81964983	33211	51,06	-0,43	6,69E-01
ENSRNOG00000031421	**Eif1a**	eukaryotic translation initiation factor 1	317163	18	40240997	40252362	11366	634,49	-0,33	6,69E-01
ENSRNOG00000012369	**Fam174b**	Protein Fam174b [Source:UniProtKB/	681398	1	136107322	136149232	41911	78,64	0,30	6,69E-01
ENSRNOG00000030683	**Vps26a**	vacuolar protein sorting 26 homolog A	361846	20	33875000	33903564	28565	1145,63	0,26	6,69E-01
ENSRNOG00000019179		N-acetyllactosaminide alpha-1,3-galac	246766	3	19925448	19957495	32048	70,81	-0,43	6,69E-01
ENSRNOG00000000697	**Coro1c**	coronin, actin binding protein 1C (Corc	501841	12	50264710	50337012	72303	6864,58	0,17	6,69E-01
ENSRNOG00000011484	**LOC68070**	ribosomal protein L10A [Source:MGI S	680700	5	143726192	143726968	777	18,02	-0,42	6,69E-01
ENSRNOG00000014624	**Selk**	selenoprotein K (Selk), mRNA [Source	290549	16	5967057	5975370	8314	1866,24	0,25	6,69E-01
ENSRNOG00000019932	**Ip6k1**	inositol hexakisphosphate kinase 1 (Ip	50560	8	116136333	116180472	44140	7844,02	0,26	6,69E-01
ENSRNOG00000009381	**Mapk6**	mitogen-activated protein kinase 6 (Ma	58840	8	81931190	81954196	23007	2747,61	-0,24	6,69E-01
ENSRNOG00000043197	**LOC68861**	RIKEN cDNA 1500009C09 gene [Sou	688613	7	123466996	123474760	7765	692,88	0,25	6,69E-01
ENSRNOG00000002319		translin [Source:MGI Symbol;Acc:MGI:109263]		13	39371412	39382208	10797	854,11	0,21	6,70E-01
ENSRNOG00000013459	**Ints9**	Protein Ints9 [Source:UniProtKB/TrEM	290322	15	52098924	52157914	58991	374,04	0,20	6,70E-01
ENSRNOG00000017847	**Rps19bp1**	ribosomal protein S19 binding protein	500907	7	121476268	121476890	623	634,89	0,30	6,70E-01

ENSRNOG00000002695		transcription factor B2, mitochondrial (	289307	13	102830291	102848026	17736	189,72	-0,29	6,71E-01
ENSRNOG00000009877	**Gar1**	GAR1 ribonucleoprotein (Gar1), mRN/	499709	2	74665990	74673267	7278	540,01	0,21	6,71E-01
ENSRNOG00000010776	**Atp13a1**	ATPase type 13A1 (Atp13a1), mRNA [	290673	16	21278077	21294131	16055	1814,09	0,17	6,71E-01
ENSRNOG00000029185	**Rasa1**	RAS p21 protein activator (GTPase ac	25676	2	13470580	13551782	81203	932,96	-0,27	6,71E-01
ENSRNOG00000007681	**Brd3**	bromodomain containing 3 (Brd3), mR	362092	3	11358266	11378548	20283	2397,24	-0,18	6,71E-01
ENSRNOG00000004012	**LOC688411**			13	78792709	78794553	1845	24,44	0,35	6,71E-01
ENSRNOG00000004820	**Sar1b**	SAR1 homolog B (S. cerevisiae) (Sar1	287276	10	36988854	37018530	29677	2821,86	-0,20	6,71E-01
ENSRNOG00000008725	**Slc37a3**	Protein Slc37a3; Solute carrier family	312255	4	66927271	66959185	31915	1238,58	0,32	6,71E-01
ENSRNOG00000010567	**Angel1**	angel homolog 1 (Drosophila) (Angel1	362765	6	119941590	119966988	25399	547,13	0,23	6,71E-01
ENSRNOG00000013017	**Arnt2**	aryl hydrocarbon receptor nuclear tran	25243	1	147327952	147484879	156928	9106,77	-0,19	6,71E-01
ENSRNOG00000013048	**Pde7a**	phosphodiesterase 7A (Pde7a), mRN/	81744	2	123767824	123856129	88306	533,05	-0,30	6,71E-01
ENSRNOG00000047656	**Gaa**	glucosidase, alpha, acid (Gaa), mRNA	367562	10	108007823	108024832	17010	3496,46	-0,32	6,71E-01
ENSRNOG00000022581	**Atp10a**	ATPase, class V, type 10A (Atp10a), m	365266	1	117145830	117311749	165920	33,94	-0,41	6,72E-01
ENSRNOG00000000166	**Apex2**	APEX nuclease (apurinic/apyrimidinic	317628	X	23566192	23586782	20591	195,89	0,20	6,72E-01
ENSRNOG00000001043	**Ddx55**	DEAD (Asp-Glu-Ala-Asp) box polypep	1E+08	12	39324516	39339745	15230	367,66	0,26	6,72E-01
ENSRNOG00000001500	**Rab4b**	RAB4B, member RAS oncogene famil	50866	1	85190236	85201526	11291	1672,59	0,31	6,72E-01
ENSRNOG00000002997	**Slc9a3r2**	solute carrier family 9, subfamily A (NH	116501	10	13820310	13830824	10515	358,42	-0,28	6,72E-01
ENSRNOG00000003211	**Srp9**	signal recognition particle 9 (Srp9), mF	690345	13	105486289	105494429	8141	1196,85	0,18	6,72E-01
ENSRNOG00000003402	**Stx6**	syntaxin 6 (Stx6), mRNA [Source:RefS	60562	13	77739763	77785250	45488	2882,49	0,27	6,72E-01
ENSRNOG00000003792	**Med14**	mediator complex subunit 14 (Med14)	317343	X	11762671	11879428	116758	1278,70	-0,19	6,72E-01
ENSRNOG00000004257	**Ctps2**	CTP synthase 2 (Ctps2), mRNA [Sour	619580	X	33731725	33870223	138499	1358,15	-0,20	6,72E-01
ENSRNOG00000008484	**Pdcl**	phosducin-like (Pdcl), mRNA [Source:	64013	3	26841046	26849027	7982	380,85	-0,27	6,72E-01
ENSRNOG00000011661	**Hnrnph2**	heterogeneous nuclear ribonucleoprot	308650	X	105308639	105312215	3577	1648,79	0,32	6,72E-01
ENSRNOG00000012044				16	68016007	68017062	1056	20,19	-0,36	6,72E-01
ENSRNOG00000012354	**Trim23**	tripartite motif-containing 23 (Trim23),	81002	2	53383587	53416925	33339	816,21	-0,35	6,72E-01
ENSRNOG00000013744	**Akip1**	A kinase (PRKA) interacting protein 1	361624	1	181316274	181324845	8572	194,45	-0,22	6,72E-01
ENSRNOG00000015807	**Pyroxd2**	pyridine nucleotide-disulphide oxidore	309381	1	269413550	269438930	25381	185,62	-0,41	6,72E-01
ENSRNOG00000018272	**Zfp511**	zinc finger protein 511 (Zfp511), mRN/	293586	1	219277464	219281869	4406	312,57	0,25	6,72E-01
ENSRNOG00000018662		alpha-methylacyl-CoA racemase (Ama	25284	2	83723121	83735159	12039	554,71	0,17	6,72E-01
ENSRNOG00000018746	**Slc30a5**	solute carrier family 30 (zinc transport	294698	2	49974659	50004756	30098	1036,79	-0,19	6,72E-01
ENSRNOG00000019937	**Kcnk1**	potassium channel, subfamily K, mem	59324	19	69519245	69555914	36670	388,27	-0,28	6,72E-01
ENSRNOG00000020736	**Nadsyn1**	NAD synthetase 1 (Nadsyn1), mRNA [	353255	1	223843327	223869684	26358	246,41	0,32	6,72E-01
ENSRNOG00000022227				1	78395178	78395462	285	70,65	-0,30	6,72E-01
ENSRNOG00000022392	**Hspb8**	heat shock protein B8 (Hspb8), mRNA	113906	12	47717762	47731882	14121	1269,91	-0,42	6,72E-01
ENSRNOG00000026651		inositol 1,4,5-trisphosphate receptor ty	25679	20	7711508	7774702	63195	24,79	-0,34	6,72E-01
ENSRNOG00000027422	**Fastkd3**	FAST kinase domains 3 (Fastkd3), mF	290946	1	39116027	39123314	7288	515,37	0,27	6,72E-01
ENSRNOG00000027762	**Nsun3**	Protein LOC100365826 [Source:UniP	1E+08	1	64371093	64414208	43116	20,85	-0,37	6,72E-01
ENSRNOG00000028904	**Vps9d1**	VPS9 domain containing 1 (Vps9d1), ı	307923	19	66745402	66759014	13613	868,66	0,17	6,72E-01

ENSRNOG00000031173	**Snd1**	staphylococcal nuclease and tudor do	64635	4	55519818	55919017	399200	4848,49	0,20	6,72E-01
ENSRNOG00000037449	**Pole**	polymerase (DNA directed), epsilon, c	304573	12	54138907	54187417	48511	450,73	0,22	6,72E-01
ENSRNOG00000042679	**Lcor**	Protein Lcor; Similar to DKFZP564P1	365462	1	268253436	268260098	6663	53,92	-0,32	6,72E-01
ENSRNOG00000042961		Protein Ormdl1 [Source:UniProtKB/Tr	1E+08	9	52775807	52782729	6923	26,68	0,34	6,72E-01
ENSRNOG00000034031		V-set and transmembrane domain containing 2-l		3	161177532	161206776	29245	746,74	-0,30	6,72E-01
ENSRNOG00000047761		zinc finger protein 326 [Source:MGI Symbol;Acc		14	4991243	5031697	40455	781,49	-0,19	6,72E-01
ENSRNOG00000019622	**Cxcr7**	chemokine (C-X-C motif) receptor 7 (C	84348	9	97046851	97058372	11522	355,12	-0,19	6,72E-01
ENSRNOG00000021713	**Kif18b**	kinesin family member 18B (Kif18b), n	303575	10	90780845	90800011	19167	531,70	0,29	6,72E-01
ENSRNOG00000015896	**Rbpms2**	RNA binding protein with multiple splic	503214	8	70852852	70883668	30817	83,61	0,35	6,72E-01
ENSRNOG00000017974				17	12537225	12539968	2744	6554,18	-0,21	6,73E-01
ENSRNOG00000018027	**Taf5l**	TAF5-like RNA polymerase II, p300/CI	367797	19	67497411	67516350	18940	103,13	-0,25	6,73E-01
ENSRNOG00000006901	**Cd320**	CD320 molecule (Cd320), mRNA [Sou	362851	7	18878075	18883844	5770	914,02	0,35	6,73E-01
ENSRNOG00000000524	**RGD73506**	similar to GI:13385412-like protein spl	294311	20	9109905	9143582	33678	2185,81	0,22	6,73E-01
ENSRNOG00000000561	**Pald1**	phosphatase domain containing, palac	294508	20	32729253	32765198	35946	191,68	-0,31	6,73E-01
ENSRNOG00000008258	**LOC10090**	formin binding protein 1 (Fnbp1), mRN	192348	3	15224331	15295979	71649	1598,14	-0,31	6,73E-01
ENSRNOG00000009705	**Lck**	lymphocyte-specific protein tyrosine ki	313050	5	151480223	151491490	11268	109,12	0,35	6,73E-01
ENSRNOG00000012985	**Pfdn5**	prefoldin subunit 5 (Pfdn5), mRNA [Sc	300257	7	141720703	141725439	4737	2965,49	0,24	6,73E-01
ENSRNOG00000031346	**Rabl5**	RAB, member RAS oncogene family-li	288585	12	24813507	24820156	6650	457,55	-0,20	6,73E-01
ENSRNOG00000017642	**Acbd5**	acyl-CoA binding domain containing 5	307170	17	91549892	91588835	38944	214,08	-0,26	6,73E-01
ENSRNOG00000000314	**Sec63**	SEC63 homolog (S. cerevisiae) (Sec6	309858	20	49156193	49225366	69174	2615,80	-0,20	6,73E-01
ENSRNOG00000017883	**Camsap1**	calmodulin regulated spectrin-associa	296580	3	8736053	8796109	60057	5542,24	0,21	6,73E-01
ENSRNOG00000049426	**Mmab**	Protein Mmab [Source:UniProtKB/TrE	687861	12	49711756	49722571	10816	148,33	0,21	6,73E-01
ENSRNOG00000003645	**LOC10091**	heterogeneous nuclear ribonucleoprot	83498	10	34728062	34733954	5893	222,17	0,27	6,74E-01
ENSRNOG00000013596	**Dimt1**	DIM1 dimethyladenosine transferase 1	294718	2	57227129	57239002	11874	406,54	-0,23	6,74E-01
ENSRNOG00000003964	**Nsmce2**	non-SMC element 2, MMS21 homolog	299957	7	100263600	100486902	223303	376,92	0,21	6,74E-01
ENSRNOG00000016503	**Smarcal1**	Swi/SNF related matrix associated, ac	316477	9	79716370	79762216	45847	327,44	0,18	6,74E-01
ENSRNOG00000009782	**Efcab7**	EF-hand calcium binding domain 7 (Ef	362549	5	122470265	122629531	159267	51,73	-0,42	6,75E-01
ENSRNOG00000005979	**Fam3c**	family with sequence similarity 3, mem	312159	4	49179398	49231971	52574	1464,61	0,19	6,75E-01
ENSRNOG00000034168		Protein Gemin7l1 [Source:UniProtKB/	685068	4	143643764	143644162	399	46,86	0,30	6,75E-01
ENSRNOG00000009970	**Dfna5**	deafness, autosomal dominant 5 (hum	353316	4	144606016	144619346	13331	177,20	-0,22	6,75E-01
ENSRNOG00000010240	**Fam46a**	family with sequence similarity 46, me	300870	8	92451691	92455379	3689	40,32	0,36	6,75E-01
ENSRNOG00000027906		ankyrin repeat domain 11 [Source:MG	365023	19	66410702	66443409	32708	5294,06	0,16	6,75E-01
ENSRNOG00000031022	**Rpl36al**	ribosomal protein L36a-like (Rpl36al),	81769	18	1551747	1553136	1390	797,18	0,18	6,75E-01
ENSRNOG00000013101		Protocadherin-8 [Source:UniProtKB/Swiss-Prot;		15	65857394	65861171	3778	3465,51	0,26	6,75E-01
ENSRNOG00000019960	**Ino80e**	INO80 complex subunit E (Ino80e), m	293494	1	205267670	205278256	10587	1159,91	0,18	6,75E-01
ENSRNOG00000021714	**Fam92b**	Protein Fam92b [Source:UniProtKB/T	361423	19	64472240	64483385	11146	82,76	-0,42	6,75E-01
ENSRNOG00000021131	**Fam63a**	family with sequence similarity 63, me	310665	2	215960035	215967611	7577	462,65	0,23	6,76E-01
ENSRNOG00000020500	**Clk2**	CDC-like kinase 2 (Clk2), mRNA [Sou	365842	2	207895502	207905133	9632	592,57	-0,20	6,76E-01

ENSRNOG00000049580	**Gpr6**	G protein-coupled receptor 6 (Gpr6), r	83683	20	47518790	47521561	2772	49,59	0,30	6,76E-01
ENSRNOG00000021130		ATP-binding cassette sub-family C me	25559	1	103194473	103275523	81051	279,15	0,31	6,76E-01
ENSRNOG00000004775	**Cstf1**	cleavage stimulation factor, 3' pre-RN/	311670	3	176456501	176467942	11442	1088,28	0,17	6,76E-01
ENSRNOG00000011949	**Ndufb5**	NADH dehydrogenase (ubiquinone) 1	294964	2	138791054	138804599	13546	2199,62	0,26	6,76E-01
ENSRNOG00000013991		cellular repressor of E1A-stimulated genes 2 [Sc		9	46047889	46088748	40860	75,01	-0,34	6,76E-01
ENSRNOG00000014291	**Elp3**	Protein Elp3 [Source:UniProtKB/TrEM	1E+08	15	52683591	52744094	60504	2885,30	0,24	6,76E-01
ENSRNOG00000019143	**Galnt2**	UDP-N-acetyl-alpha-D-galactosamine	292090	19	67774647	67824801	50155	5214,78	0,15	6,76E-01
ENSRNOG00000020435	**Terf2**	telomeric repeat binding factor 2 (Terf2	361403	19	50136826	50164563	27738	1000,68	0,16	6,76E-01
ENSRNOG00000027894	**Iqgap3**	IQ motif containing GTPase activating	310621	2	206850518	206892647	42130	311,15	0,21	6,76E-01
ENSRNOG00000014857	**RGD15600**	similar to RIKEN cDNA 2410004B18 (	499724	2	270343010	270349925	6916	1530,07	0,28	6,76E-01
ENSRNOG00000005558	**LOC10012**	RIKEN cDNA A130010J15 gene [Sour	1E+08	13	116445798	116448210	2413	135,25	-0,28	6,76E-01
ENSRNOG00000009243	**Oaf**	OAF homolog (Drosophila) (Oaf), mRI	315594	8	45980602	45998572	17971	891,88	-0,37	6,76E-01
ENSRNOG00000010524	**Cryab**	crystallin, alpha B (Cryab), mRNA [Soi	25420	8	53775525	53779781	4257	336,81	-0,39	6,76E-01
ENSRNOG00000025148	**Baz2b**	bromodomain adjacent to zinc finger d	317627	3	51081857	51200027	118171	811,57	0,24	6,76E-01
ENSRNOG00000026527	**Snx32**	Protein Snx32 [Source:UniProtKB/TrE	361708	1	227804038	227820073	16036	756,24	0,20	6,76E-01
ENSRNOG00000048110		Uncharacterized protein [Source:UniProtKB/TrE		17	55671837	55699822	27986	91,79	-0,30	6,76E-01
ENSRNOG00000001435	**Srcrb4d**	Protein Srcrb4d [Source:UniProtKB/T	304401	12	25751492	25761525	10034	88,67	0,39	6,76E-01
ENSRNOG00000003399	**Hnrnph1**	heterogeneous nuclear ribonucleoprot	140931	10	35642700	35651369	8670	3549,89	-0,34	6,76E-01
ENSRNOG00000010877	**Alg9**	alpha-1,2-mannosyltransferase (Alg9)	367083	8	53799792	53862451	62660	921,02	0,29	6,76E-01
ENSRNOG00000004519		autophagy related 2B [Source:MGI Symbol;Acc:		6	138645992	138712125	66134	2381,52	0,17	6,76E-01
ENSRNOG00000012534	**Mfsd10**	major facilitator superfamily domain cc	305449	14	82050489	82053316	2828	506,89	0,22	6,76E-01
ENSRNOG00000046313				2	97311486	97312150	665	11425,24	0,30	6,76E-01
ENSRNOG00000016975	**Pxmp4**	peroxisomal membrane protein 4 (Pxn	282634	3	156464197	156481427	17231	917,92	0,16	6,76E-01
ENSRNOG00000048577	**Zfp955a**	zinc finger protein 955A (Zfp955a), mF	314600	7	15957414	15961350	3937	584,28	-0,33	6,76E-01
ENSRNOG00000027152	**N4bp2**	Protein N4bp2 [Source:UniProtKB/TrE	305342	14	43850808	43892633	41826	662,81	-0,24	6,76E-01
ENSRNOG00000007249	**Cdkn1b**	cyclin-dependent kinase inhibitor 1B ((	83571	4	232962327	232967128	4802	3777,42	0,33	6,77E-01
ENSRNOG00000001238	**Rsrc2**	arginine/serine-rich coiled-coil 2 (Rsrc:	360807	12	40146860	40167591	20732	799,79	-0,38	6,77E-01
ENSRNOG00000002884	**Blzf1**	basic leucine zipper nuclear factor 1 (E	498266	13	87476065	87491192	15128	144,99	-0,31	6,77E-01
ENSRNOG00000005410	**Cdca7l**	cell division cycle associated 7 like (Cc	619566	6	154655200	154699230	44031	203,17	-0,22	6,77E-01
ENSRNOG00000007433	**Cyb561**	cytochrome b-561 (Cyb561), mRNA [S	303601	10	93889863	93896347	6485	804,96	0,30	6,77E-01
ENSRNOG00000017417	**Gabarap**	GABA(A) receptor-associated protein	58974	10	56346337	56349340	3004	8026,13	0,25	6,77E-01
ENSRNOG00000018395	**LOC10091**	ubiquitin specific peptidase 40 (Usp40	316599	9	94791220	94862200	70981	331,27	-0,24	6,77E-01
ENSRNOG00000000245	**Slc16a6**	solute carrier family 16, member 6 (Slc	303772	10	97459774	97480918	21145	182,61	-0,28	6,77E-01
ENSRNOG00000004094		Prostaglandin E2 receptor EP1 subtyp	25637	19	35779888	35782528	2641	424,02	0,22	6,77E-01
ENSRNOG00000005982	**LOC10091**	C1D nuclear receptor co-repressor (C	289810	14	100111604	100123763	12160	366,90	-0,26	6,77E-01
ENSRNOG00000006857	**RGD13110**	Protein RGD1311080 [Source:UniPro	312401	4	161341072	161348902	7831	190,58	-0,35	6,77E-01
ENSRNOG00000007066	**Htr5a**	5-hydroxytryptamine (serotonin) recep	25689	4	193112	202795	9684	26,80	-0,38	6,77E-01
ENSRNOG00000007834	**Cand1**	cullin-associated and neddylation-diss	117152	7	62113404	62151495	38092	5238,59	0,19	6,77E-01

ENSRNOG00000008943		Proenkephalin-A Synenkephalin Met-e	29237	5	21834414	21839739	5326	661,15	0,41	6,77E-01
ENSRNOG00000009121		Protein Ankrd36 [Source:UniProtKB/T	305491	14	86560037	86581295	21259	22,08	0,38	6,77E-01
ENSRNOG00000013743	**Sec61a1**	Sec61 alpha 1 subunit (S. cerevisiae)	80843	4	185693508	185707859	14352	6646,78	0,23	6,77E-01
ENSRNOG00000013828	**Sirt3**	sirtuin 3 (Sirt3), mRNA [Source:RefSe	293615	1	220539135	220561047	21913	1378,16	0,23	6,77E-01
ENSRNOG00000015376	**Npas1**	neuronal PAS domain protein 1 (Npas	308387	1	79667893	79687979	20087	322,34	0,21	6,77E-01
ENSRNOG00000016932		Protein LOC100361684 [Source:UniP	1E+08	17	12725166	12749263	24098	586,37	-0,19	6,77E-01
ENSRNOG00000019085	**Xpo5**	exportin 5 (Xpo5), mRNA [Source:Ref	363194	9	16021878	16059659	37782	2489,63	0,18	6,77E-01
ENSRNOG00000019799		protocadherin gamma-A12 [Source:R	498850	18	30608069	30660582	52514	5386,63	0,19	6,77E-01
ENSRNOG00000019908	**Lamtor2**	late endosomal/lysosomal adaptor, MA	295234	2	207313930	207317305	3376	1145,14	0,27	6,77E-01
ENSRNOG00000020263	**Atp1a3**	ATPase, Na+/K+ transporting, alpha 3	24213	1	83104725	83133845	29121	18622,01	-0,21	6,77E-01
ENSRNOG00000022256	**Cxcl10**	chemokine (C-X-C motif) ligand 10 (C	245920	14	17126851	17129068	2218	22,77	0,42	6,77E-01
ENSRNOG00000022727	**Tmem127**	transmembrane protein 127 (Tmem12	311405	3	126202793	126215584	12792	4092,91	0,27	6,77E-01
ENSRNOG00000024603	**Rps15**	ribosomal protein S15 (Rps15), mRNA	29285	7	12451930	12453368	1439	12123,79	0,37	6,77E-01
ENSRNOG00000024879	**Polrmt**	polymerase (RNA) mitochondrial (DNA	299604	7	13011142	13021357	10216	644,44	0,21	6,77E-01
ENSRNOG00000028892	**RGD15660**	Protein RGD1566084 [Source:UniPro	501795	12	4732781	4738127	5347	1572,05	0,21	6,77E-01
ENSRNOG00000030818	**RGD15610**	Protein RGD1561034 [Source:UniPro	308908	1	177219835	177223009	3175	127,79	-0,24	6,77E-01
ENSRNOG00000037251	**Zfp248**	Protein Znf248 [Source:UniProtKB/Tr	500304	4	216407426	216429195	21770	139,09	-0,24	6,77E-01
ENSRNOG00000046560				1	227765419	227765831	413	859,32	0,32	6,77E-01
ENSRNOG00000048907		mastermind like 3 (Drosophila) [Sourc	310405	2	160209608	160218015	8408	54,07	-0,32	6,77E-01
ENSRNOG00000050834	**Fam102a**	Protein LOC687750; RCG45756 [Sou	687750	3	16905536	16934339	28804	295,67	0,19	6,77E-01
ENSRNOG00000007518		nck-associated protein 1 [Source:Ref	58823	3	74284278	74346873	62596	7784,18	-0,32	6,77E-01
ENSRNOG00000015142	**Timm21**	translocase of inner mitochondrial mei	307210	18	80841881	80846335	4455	388,02	-0,26	6,77E-01
ENSRNOG00000031387	**Dazap1**	DAZ associated protein 1 (Dazap1), m	362836	7	12460102	12480849	20748	1136,51	0,22	6,77E-01
ENSRNOG00000020384	**Fam13b**	family with sequence similarity 13, me	291694	18	26936705	27002904	66200	3178,52	-0,21	6,77E-01
ENSRNOG00000003453	**Lypd1**	Ly6/Plaur domain containing 1 (Lypd1	360838	13	47327525	47368232	40708	633,32	-0,20	6,77E-01
ENSRNOG00000014243	**Pear1**	platelet endothelial aggregation recept	295293	2	206519129	206539048	19920	28,69	-0,38	6,78E-01
ENSRNOG00000018714	**Arl5b**	ADP-ribosylation factor-like 5B (Arl5b)	364788	17	83807999	83830811	22813	456,85	-0,19	6,78E-01
ENSRNOG00000019174	**Chtf18**	CTF18, chromosome transmission fide	287146	10	14903483	14911519	8037	229,93	0,30	6,78E-01
ENSRNOG00000002382	**Mfap4**	microfibrillar-associated protein 4 (Mfa	287382	10	47538040	47540977	2938	27,57	0,36	6,78E-01
ENSRNOG00000007921		heterogeneous nuclear ribonucleoprot	116655	7	18693856	18731848	37993	3890,29	0,26	6,78E-01
ENSRNOG00000019374	**Csk**	c-src tyrosine kinase (Csk), mRNA [Sc	315707	8	62183287	62187910	4624	2219,12	0,21	6,78E-01
ENSRNOG00000050519	**Rhoa**	ras homolog family member A (Rhoa),	117273	8	116431339	116464929	33591	9617,46	0,18	6,78E-01
ENSRNOG00000042504	**Zfp964**	Protein Zfp964 [Source:UniProtKB/Tr	1E+08	16	21386465	21396027	9563	56,53	-0,34	6,78E-01
ENSRNOG00000004574	**Ofd1**	oral-facial-digital syndrome 1 (Ofd1), r	302661	X	29955456	29996219	40764	208,79	0,29	6,78E-01
ENSRNOG00000028430	**LOC25765**	hippyragranin (LOC257650), mRNA [S	257650	10	86020708	86034258	13551	77,27	-0,31	6,78E-01
ENSRNOG00000007951	**Galnt14**	UDP-N-acetyl-alpha-D-galactosamine	313878	6	34618799	34832960	214162	464,30	-0,24	6,79E-01
ENSRNOG00000018220	**Pde4dip**	phosphodiesterase 4D interacting prot	64183	2	219345687	219479200	133514	4566,02	-0,20	6,79E-01
ENSRNOG00000003942	**Asnsd1**	asparagine synthetase domain contair	299507	9	52670315	52674870	4556	951,47	0,33	6,79E-01

ENSRNOG00000005900	**Slc2a6**	solute carrier family 2 (facilitated gluc	296600	3	10930061	10936876	6816	1291,15	0,28	6,79E-01
ENSRNOG00000011617	**Dguok**	deoxyguanosine kinase (Dguok), mRN	297389	4	179770727	179798683	27957	274,70	0,23	6,79E-01
ENSRNOG00000010701	**Depdc1b**	DEP domain containing 1B (Depdc1b)	310074	2	59075286	59147310	72025	126,44	0,36	6,79E-01
ENSRNOG00000014565	**Lcmt1**	leucine carboxyl methyltransferase 1 (	361643	1	200394431	200450381	55951	1696,10	0,26	6,79E-01
ENSRNOG00000003583	**Smyd2**	SET and MYND domain containing 2 (	289372	13	113261746	113302396	40651	1914,06	0,24	6,79E-01
ENSRNOG00000007327	**Pars2**	prolyl-tRNA synthetase 2, mitochondri	313429	5	130099179	130104237	5059	245,61	-0,19	6,79E-01
ENSRNOG00000020777	**Zfp382**	zinc finger protein 382 (Zfp382), mRN	246264	1	89737854	89763846	25993	289,51	-0,23	6,79E-01
ENSRNOG00000032407	**Npas3**	Protein Npas3 [Source:UniProtKB/TrE	299016	6	84015921	84456422	440502	919,30	-0,18	6,79E-01
ENSRNOG00000002907	**Plekha6**	Protein Plekha6 [Source:UniProtKB/T	360842	13	55612697	55632414	19718	405,55	-0,22	6,79E-01
ENSRNOG00000006557	**Cyfip2**	cytoplasmic FMR1 interacting protein	303073	10	31097979	31235818	137840	10867,20	-0,24	6,79E-01
ENSRNOG00000007584	**Ehd4**	EH-domain containing 4 (Ehd4), mRN	192204	3	118425620	118489608	63989	231,68	0,33	6,79E-01
ENSRNOG00000014011	**Dll4**	delta-like 4 (Drosophila) (Dll4), mRNA	311332	3	117685738	117695768	10031	61,99	-0,41	6,79E-01
ENSRNOG00000028662	**Zfp69**	Protein Zfp965 [Source:UniProtKB/Tr	1E+08	5	143651014	143656994	5981	21,41	-0,35	6,79E-01
ENSRNOG00000015911	**Lrp5**	low density lipoprotein receptor-relate	293649	1	225689356	225793030	103675	591,21	-0,27	6,80E-01
ENSRNOG00000011394	**Kif3c**	kinesin family member 3C (Kif3c), mR	85248	6	37579445	37625883	46439	7132,67	0,26	6,80E-01
ENSRNOG00000011488	**Sergef**	Protein Sergef [Source:UniProtKB/TrE	365243	1	103690972	103741937	50966	558,15	0,22	6,80E-01
ENSRNOG00000005856		solute carrier family 30 (zinc transport	298786	6	32084271	32113759	29489	349,12	-0,20	6,80E-01
ENSRNOG00000007304	**Herc3**	HECT and RLD domain containing E3	362377	4	153899767	153990202	90436	2136,68	-0,21	6,80E-01
ENSRNOG00000011063		DENN/MADD domain containing 1B [S	289051	13	61041188	61258169	216982	81,63	-0,33	6,80E-01
ENSRNOG00000012775	**Ccdc127**	coiled-coil domain containing 127 (Cc	308060	1	32965363	32971707	6345	291,14	-0,20	6,80E-01
ENSRNOG00000017340	**Wdr12**	WD repeat domain 12 (Wdr12), mRNA	363237	9	66665604	66691636	26033	539,01	0,26	6,80E-01
ENSRNOG00000018125	**Sephs1**	selenophosphate synthetase 1 (Sephs	291314	17	78978379	79006698	28320	1802,03	-0,21	6,80E-01
ENSRNOG00000021765	**LOC10091**	GLI family zinc finger 4 (Gli4), mRNA [	500893	7	116565906	116571116	5211	287,41	0,20	6,80E-01
ENSRNOG00000025332		CD109 antigen [Source:MGI Symbol;A	363104	8	85501109	85614760	113652	46,69	-0,33	6,80E-01
ENSRNOG00000046705	**Snx3**	sorting nexin 3 (Snx3), mRNA [Source:RefSeq n		20	48923087	48932813	9727	3918,08	0,17	6,80E-01
ENSRNOG00000048736	**Mllt1**	myeloid/lymphoid or mixed-lineage leu	301119	9	9110342	9154498	44157	3747,58	0,21	6,80E-01
ENSRNOG00000033397		a disintegrin-like and metallopeptidase (reprolysi		7	135127520	135291531	164012	57,25	-0,33	6,80E-01
ENSRNOG00000001288	**Gpr146**	G protein-coupled receptor 146 (Gpr1	498153	12	19332474	19345579	13106	226,27	-0,35	6,81E-01
ENSRNOG00000003855	**Dnaja3**	DnaJ (Hsp40) homolog, subfamily A, r	360481	10	9826850	9850500	23651	1199,61	0,26	6,81E-01
ENSRNOG00000007948	**Nf2**	neurofibromin 2 (merlin) (Nf2), mRNA	25744	14	85673149	85766300	93152	5287,25	-0,21	6,81E-01
ENSRNOG00000009014	**Slc35f2**	solute carrier family 35, member F2 (S	300713	8	56929088	56972651	43564	141,77	-0,35	6,81E-01
ENSRNOG00000011379	**Ccndbp1**	cyclin D-type binding-protein 1 (Ccndb	362201	3	119494100	119504496	10397	1405,62	0,18	6,81E-01
ENSRNOG00000012794	**Grhpr**	glyoxylate reductase/hydroxypyruvate	680021	5	65037813	65047242	9430	598,95	-0,37	6,81E-01
ENSRNOG00000025295	**Mavs**	mitochondrial antiviral signaling protei	311430	3	130273622	130287787	14166	301,39	-0,30	6,81E-01
ENSRNOG00000030328	**Fam98a**	family with sequence similarity 98, me	313873	6	30945057	30959716	14660	1256,07	0,26	6,81E-01
ENSRNOG00000045873		RCG29880; Uncharacterized protein [Source:U		4	211762499	211763248	750	217,40	-0,24	6,81E-01
ENSRNOG00000047799	**Gnb5**	guanine nucleotide binding protein (G protein), b		8	81861124	82010315	149192	560,12	0,27	6,81E-01
ENSRNOG00000000467	**Ring1**	ring finger protein 1 (Ring1), mRNA [S	309626	20	5891721	5895148	3428	797,60	0,21	6,81E-01

ENSRNOG00000011497	**Aldh1b1**	aldehyde dehydrogenase 1 family, me	298079	5	65898607	65903614	5008	285,62	0,25	6,81E-01
ENSRNOG00000027276	**Xkr4**	XK, Kell blood group complex subunit-	297801	5	20529390	20531948	2559	688,35	-0,33	6,81E-01
ENSRNOG00000046152		Uncharacterized protein [Source:UniProtKB/TrE		9	113466626	113474979	8354	74,26	-0,25	6,81E-01
ENSRNOG00000010658	**Cth**	cystathionase (cystathionine gamma-l	24962	2	282366121	282392112	25992	425,61	-0,32	6,81E-01
ENSRNOG00000019316	**Sh3bp4**	SH3-domain binding protein 4 (Sh3bp	64634	9	95977039	95993098	16060	2590,45	-0,41	6,81E-01
ENSRNOG00000001557	**Cxadr**	coxsackie virus and adenovirus recept	89843	11	20481046	20528209	47164	2092,70	-0,23	6,81E-01
ENSRNOG00000011357		sex hormone binding globulin (Shbg),	24775	10	55965504	55982854	17351	60,87	0,34	6,81E-01
ENSRNOG00000013504	**Prpf31**	pre-mRNA processing factor 31 (Prpf3	292536	1	63143068	63154743	11676	900,39	0,19	6,81E-01
ENSRNOG00000017692	**Ppp1r37**	protein phosphatase 1, regulatory sub	308398	1	81705739	81737548	31810	2725,05	0,21	6,81E-01
ENSRNOG00000009066	**Thra**	thyroid hormone receptor alpha (Thra)	81812	10	86465253	86480926	15674	19239,51	-0,18	6,82E-01
ENSRNOG00000037134	**Shc4**	SHC (Src homology 2 domain containi	679845	3	124421964	124514515	92552	51,82	-0,28	6,82E-01
ENSRNOG00000003745	**Atf3**	activating transcription factor 3 (Atf3),	25389	13	114411695	114443438	31744	91,92	-0,41	6,82E-01
ENSRNOG00000010716	**Atoh8**	atonal homolog 8 (Drosophila) (Atoh8)	500200	4	164837904	164870076	32173	120,28	-0,33	6,82E-01
ENSRNOG00000013517	**Phtf2**	putative homeodomain transcription fa	296762	4	10815516	10930367	114852	394,56	-0,26	6,82E-01
ENSRNOG00000014214	**RGD15599**	ribosomal protein L27a (Rpl27a), mRN	293418	1	181121468	181124486	3019	1052,87	0,30	6,82E-01
ENSRNOG00000019341	**Tmem179b**	transmembrane protein 179B (Tmem1	690263	1	231910394	231912376	1983	240,91	0,29	6,82E-01
ENSRNOG00000020247	**Cnih2**	cornichon family AMPA receptor auxili	361705	1	227405547	227411186	5640	5921,04	-0,33	6,82E-01
ENSRNOG00000036964		Ral GTPase activating protein, alpha subunit 2 (		3	146887749	147146568	258820	514,91	-0,24	6,82E-01
ENSRNOG00000039837		uncharacterized protein LOC299305 |	299305	X	2678420	2745615	67196	158,12	0,21	6,82E-01
ENSRNOG00000009462	**Ccdc90b**	coiled-coil domain containing 90B (Cc	308820	1	163647743	163661295	13553	389,26	-0,28	6,82E-01
ENSRNOG00000004180	**Fam19a2**	Protein Fam19a2 [Source:UniProtKB/	680647	7	66241454	66373683	132230	266,29	0,30	6,82E-01
ENSRNOG00000002353		RAB3 GTPase activating protein subu	289350	13	107643922	107901904	257983	1531,93	0,22	6,82E-01
ENSRNOG00000004295		ATP-binding cassette, sub-family A (Al	287788	10	98123261	98176991	53731	73,63	0,36	6,82E-01
ENSRNOG00000011592	**Lrp10**	low-density lipoprotein receptor-relate	305880	15	36993827	36999985	6159	1732,23	0,22	6,82E-01
ENSRNOG00000003730	**Aida**	axin interactor, dorsalization associate	682999	13	100906568	100933399	26832	1825,54	0,31	6,82E-01
ENSRNOG00000006655	**Plrg1**	pleiotropic regulator 1 (Plrg1), mRNA [	60376	2	201454938	201471292	16355	604,15	0,27	6,82E-01
ENSRNOG00000001161	**Gatc**	glutamyl-tRNA(Gln) amidotransferase,	360821	12	48824911	48832872	7962	935,18	0,16	6,83E-01
ENSRNOG00000008560	**Ccdc30**	Protein Ccdc30 [Source:UniProtKB/Tr	362580	5	142180663	142261056	80394	329,77	-0,23	6,83E-01
ENSRNOG00000009841		sterile alpha motif domain containing 9	500015	4	31134418	31206074	71657	101,13	0,41	6,83E-01
ENSRNOG00000020871	**Ltbp4**	latent transforming growth factor beta	292734	1	85328915	85361198	32284	1052,63	-0,23	6,83E-01
ENSRNOG00000011028		Uncharacterized protein [Source:UniProtKB/TrE		3	146566331	146786271	219941	85,74	-0,24	6,83E-01
ENSRNOG00000025239	**Nudt15**	nudix (nucleoside diphosphate linked r	290365	15	59141909	59178909	37001	52,64	0,31	6,83E-01
ENSRNOG00000038600	**Dnaaf3**	dynein, axonemal, assembly factor 3 (	1E+08	1	75669674	75677024	7351	98,92	0,26	6,83E-01
ENSRNOG00000018798	**LOC10091**	brevican (Bcan), transcript variant 1, n	25393	2	206762694	206775669	12976	10852,80	-0,31	6,83E-01
ENSRNOG00000026005	**Ints5**	integrator complex subunit 5 (Ints5), m	309200	1	232029232	232034010	4779	1299,93	0,20	6,83E-01
ENSRNOG00000037229	**Gmppb**	GDP-mannose pyrophosphorylase B (	363145	8	116181002	116210240	29239	403,96	0,18	6,83E-01
ENSRNOG00000022684	**Gpc3**	glypican 3 (Gpc3), mRNA [Source:Ref	25236	X	139625387	139993343	367957	101,88	-0,29	6,83E-01
ENSRNOG00000025881	**Rbms3**	Protein Rbms3 [Source:UniProtKB/Tr	680726	8	124104381	124353670	249290	57,88	0,27	6,83E-01

ENSRNOG00000024967	**Uqcrb**	ubiquinol-cytochrome c reductase binc	362897	7	71436514	71441869	5356	494,65	-0,20	6,83E-01
ENSRNOG00000010105	**S100a11**	S100 calcium binding protein A11 (S1(	445415	2	210998954	211004364	5411	111,63	-0,40	6,83E-01
ENSRNOG00000020452	**Nsmce4a**	non-SMC element 4 homolog A (S. cer	293528	1	208135294	208142382	7089	506,05	0,29	6,83E-01
ENSRNOG00000000432	**Fkbpl**	FK506 binding protein-like (Fkbpl), mF	406168	20	6491232	6492720	1489	465,19	0,23	6,84E-01
ENSRNOG00000002919	**Gfap**	glial fibrillary acidic protein (Gfap), mR	24387	10	90763149	90771847	8699	24583,96	-0,39	6,84E-01
ENSRNOG00000003809	**Sat1**	spermidine/spermine N1-acetyl transfe	302642	X	43930760	43934045	3286	1558,69	-0,31	6,84E-01
ENSRNOG00000005569	**Phospho1**	phosphatase, orphan 1 (Phospho1), m	287644	10	83440620	83447961	7342	41,04	0,38	6,84E-01
ENSRNOG00000008761	**Ncs1**	neuronal calcium sensor 1 (Ncs1), mR	65153	3	15914623	15960622	46000	2365,69	0,23	6,84E-01
ENSRNOG00000009992	**Rabggtb**	Rab geranylgeranyltransferase, beta s	25533	2	278774376	278779644	5269	1820,27	-0,18	6,84E-01
ENSRNOG00000012163	**Plekhm2**	pleckstrin homology domain containin(	313667	5	163897199	163934413	37215	1875,10	0,19	6,84E-01
ENSRNOG00000014531	**Hbs1l**	Hbs1-like (S. cerevisiae) (Hbs1l), mRN	293408	1	17974955	18051508	76554	2218,87	0,23	6,84E-01
ENSRNOG00000015227		Protein LOC100909685 [Source:UniP	1E+08	9	54267753	54414206	146454	211,39	-0,33	6,84E-01
ENSRNOG00000018768	**LOC10036**	ribosomal protein S8 (Rps8), mRNA [S	65136	5	139813899	139816616	2718	176,74	0,19	6,84E-01
ENSRNOG00000019094	**Gstm2**	glutathione S-transferase mu 2 (Gstm2	24424	2	230246914	230251686	4773	213,53	-0,41	6,84E-01
ENSRNOG00000025648	**Dhrs7**	dehydrogenase/reductase (SDR family	299131	6	104917830	104940633	22804	131,96	0,35	6,84E-01
ENSRNOG00000033791	**Apc2**	adenomatosis polyposis coli 2 (Apc2),	299611	7	12428263	12444598	16336	12115,56	0,25	6,84E-01
ENSRNOG00000013557	**Lancl1**	LanC lantibiotic synthetase componen	114515	9	73260452	73290356	29905	2714,17	0,24	6,84E-01
ENSRNOG00000031448	**Stpg1**	sperm-tail PG-rich repeat containing 1	500566	5	157611549	157654873	43325	109,18	-0,32	6,84E-01
ENSRNOG00000049724		UDP-GlcNAc:betaGal beta-1,3-N-acet	367384	10	110385026	110443571	58546	53,55	-0,27	6,84E-01
ENSRNOG00000004667	**Gen1**	GEN1 Holliday junction 5' flap endonu	298884	6	46660788	46690981	30194	42,20	0,34	6,84E-01
ENSRNOG00000019913	**Mta2**	metastasis associated 1 family, memb	361724	1	232078483	232087203	8721	3193,12	0,15	6,84E-01
ENSRNOG00000011791	**Zfand4**	zinc finger, AN1-type domain 4 (Zfand4	286998	4	214154261	214214383	60123	104,23	-0,27	6,84E-01
ENSRNOG00000048152	**Myo1b**	myosin Ib (Myo1b), mRNA [Source:Re	117057	9	54416222	54472391	56170	18,59	-0,36	6,84E-01
ENSRNOG00000020716	**Axl**	Axl receptor tyrosine kinase (Axl), tran	308444	1	83812118	83840542	28425	915,17	-0,30	6,84E-01
ENSRNOG00000016950	**Otud1**	Protein Otud1 [Source:UniProtKB/TrE	498803	17	88117971	88119338	1368	144,74	0,27	6,85E-01
ENSRNOG00000014614	**Cxxc1**	CXXC finger protein 1 (Cxxc1), mRNA	291440	18	69335505	69340557	5053	1651,15	0,17	6,85E-01
ENSRNOG00000005271	**Rapgef5**	Rap guanine nucleotide exchange fac	362799	6	154462518	154541111	78594	1386,19	0,23	6,85E-01
ENSRNOG00000006593	**Grpel1**	GrpE-like 1, mitochondrial (Grpel1), m	79563	14	79097609	79103770	6162	1491,29	0,19	6,85E-01
ENSRNOG00000017167		predicted gene 5070 [Source:MGI Symbol;Acc:M		11	43715836	43716721	886	35,69	0,40	6,85E-01
ENSRNOG00000017502	**Sike1**	suppressor of IKBKE 1 (Sike1), mRNA	362007	2	224918791	224926621	7831	2122,76	0,28	6,85E-01
ENSRNOG00000026286	**Nhlrc1**	NHL repeat containing 1 (Nhlrc1), mRI	364682	17	19876653	19877843	1191	202,86	-0,23	6,85E-01
ENSRNOG00000032490	**Cdh7**	cadherin 7, type 2 (Cdh7), mRNA [Sou	29162	13	36230292	36366642	136351	168,92	0,31	6,85E-01
ENSRNOG00000045892	**Cfl2**	cofilin 2, muscle (Cfl2), mRNA [Source	366624	6	85300272	85304317	4046	3331,89	-0,18	6,85E-01
ENSRNOG00000000436	**Egfl8**	EGF-like-domain, multiple 8 (Egfl8), tra	406166	20	6457567	6459956	2390	67,31	-0,34	6,85E-01
ENSRNOG00000037117	**Zcwpw2**	Protein Zcwpw2 [Source:UniProtKB/T	681381	8	125620683	125632560	11878	22,39	0,39	6,85E-01
ENSRNOG00000000921	**Ubl3**	ubiquitin-like 3 (Ubl3), mRNA [Source:	363869	12	9953322	9997647	44326	3440,44	0,24	6,85E-01
ENSRNOG00000007285	**Tp53rk**	TP53 regulating kinase (Tp53rk), mRN	362272	3	169715189	169718619	3431	188,04	0,29	6,85E-01
ENSRNOG00000012772	**Nqo1**	NAD(P)H dehydrogenase, quinone 1 (	24314	19	49291903	49306795	14893	2593,27	-0,36	6,85E-01

ENSRNOG00000021565	**LOC50289**	fructose-2,6-bisphosphatase TIGAR [	297610	4	231948824	231966866	18043	405,92	0,29	6,85E-01
ENSRNOG00000029837	**LOC68060**	RIKEN cDNA 4930485B16 gene [Sou	680606	7	33927709	33985085	57377	42,80	-0,33	6,85E-01
ENSRNOG00000034241	**Set**	SET nuclear oncogene (Set), mRNA [S	307947	19	69119722	69121240	1519	1957,11	0,19	6,85E-01
ENSRNOG00000010702	**Ube3c**	Protein Ube3c [Source:UniProtKB/TrE	362294	4	2558988	2658633	99646	4683,08	0,32	6,85E-01
ENSRNOG00000014008	**Mfsd2a**	major facilitator superfamily domain co	298504	5	144433450	144448324	14875	389,43	0,38	6,85E-01
ENSRNOG00000020804	**Smarcc1**	SWI/SNF related, matrix associated, a	301020	8	117561617	117675013	113397	3578,11	0,16	6,85E-01
ENSRNOG00000028711	**Dgat1**	diacylglycerol O-acyltransferase 1 (Dg	84497	7	117553999	117564368	10370	880,89	0,16	6,86E-01
ENSRNOG00000048190	**Rgmb**	Protein LOC681433 [Source:UniProtK	681433	1	58723856	58742185	18330	1547,04	0,27	6,86E-01
ENSRNOG00000006735	**Cdkn2b**	cyclin-dependent kinase inhibitor 2B (p	25164	5	111820712	111826334	5623	69,98	-0,32	6,86E-01
ENSRNOG00000012729	**Mfsd8**	major facilitator superfamily domain co	361939	2	147311814	147384522	72709	201,25	-0,31	6,86E-01
ENSRNOG00000014691	**Ric3**	resistance to inhibitors of cholinestera	687147	1	180623994	180672685	48692	247,33	0,24	6,86E-01
ENSRNOG00000015813	**Ubr2**	ubiquitin protein ligase E3 component	363188	9	14919790	14997035	77246	2034,91	0,27	6,86E-01
ENSRNOG00000046256	**Ralgapa1**	Ral GTPase activating protein, alpha s	56785	6	85920794	86131756	210963	1368,71	-0,30	6,86E-01
ENSRNOG00000008217	**Madcam1**	mucosal vascular addressin cell adhes	54266	7	13087425	13090827	3403	19,26	0,37	6,86E-01
ENSRNOG00000003424	**Elac2**	elaC ribonuclease Z 2 (Elac2), mRNA	282826	10	51236364	51259151	22788	915,89	0,18	6,87E-01
ENSRNOG00000017472	**Tcf12**	transcription factor 12 (Tcf12), mRNA	25720	8	74682708	74733231	50524	1593,33	-0,22	6,87E-01
ENSRNOG00000019414	**Tmem79**	transmembrane protein 79 (Tmem79),	310626	2	207104465	207109243	4779	47,16	-0,28	6,87E-01
ENSRNOG00000018063	**Rad1**	RAD1 homolog (S. pombe) (Rad1), m	294800	2	84204602	84212641	8040	288,46	-0,18	6,88E-01
ENSRNOG00000018510		cyclin-dependent kinase 7 [Source:MG	171150	2	49869831	49893977	24147	318,60	0,19	6,88E-01
ENSRNOG00000022485	**Chuk**	conserved helix-loop-helix ubiquitous	309361	1	271293822	271329346	35525	553,64	-0,32	6,88E-01
ENSRNOG00000045898	**LOC10091**	Protein LOC100911029 [Source:UniP	1E+08	14	87029165	87034391	5227	397,77	0,26	6,88E-01
ENSRNOG00000020848	**Adck4**	aarF domain containing kinase 4 (Adc	308453	1	85255491	85278009	22519	976,30	0,19	6,88E-01
ENSRNOG00000008512	**Btf3l4**	Protein Btf3l4; RCG50485, isoform CR	305284	5	132272426	132290204	17779	283,82	-0,18	6,88E-01
ENSRNOG00000011398	**Trmt61a**	tRNA methyltransferase 61 homolog A	314462	6	144259958	144265948	5991	715,39	0,31	6,88E-01
ENSRNOG00000004617		Protein Fmnl2 [Source:UniProtKB/TrE	499797	3	43498861	43772955	274095	1639,13	-0,17	6,88E-01
ENSRNOG00000048441		Protein LOC686295 [Source:UniProtK	686295	8	114231117	114247514	16398	1094,61	0,24	6,88E-01
ENSRNOG00000009830	**Fam204a**	family with sequence similarity 204, m	308004	1	288701239	288730895	29657	318,08	-0,24	6,89E-01
ENSRNOG00000010311		Protein Dzip1 [Source:UniProtKB/TrE	364475	15	107541022	107591804	50783	3265,18	0,20	6,89E-01
ENSRNOG00000010023	**Slc33a1**	solute carrier family 33 (acetyl-CoA tra	64018	2	173904053	173926434	22382	1093,30	0,28	6,89E-01
ENSRNOG00000008351	**RGD13081**	similar to 9930012K11Rik protein (RG	361066	15	55561684	55565495	3812	67,23	0,30	6,89E-01
ENSRNOG00000029875	**Mrpl41**	mitochondrial ribosomal protein L41 (M	296551	3	2162293	2163210	918	555,58	0,24	6,89E-01
ENSRNOG00000029980	**Zbtb16**	zinc finger and BTB domain containing	353227	8	51578440	51739786	161347	280,56	-0,36	6,89E-01
ENSRNOG00000028185	**Tstd3**	Protein Tstd3; Similar to CG12279-PA	500420	5	40549091	40558531	9441	239,67	-0,30	6,89E-01
ENSRNOG00000002039	**Evi5**	ecotropic viral integration site 5 (Evi5),	1E+08	14	2892968	2979616	86649	515,07	-0,29	6,89E-01
ENSRNOG00000002115	**Sod1**	superoxide dismutase 1, soluble (Sod	24786	11	33982764	33988340	5577	4522,95	0,21	6,89E-01
ENSRNOG00000000186	**Tst**	thiosulfate sulfurtransferase (Tst), mRI	25274	7	119606996	119613809	6814	3231,66	-0,37	6,89E-01
ENSRNOG00000003123	**Rpa1**	replication protein A1 (Rpa1), mRNA [S	287524	10	61855482	61897974	42493	1330,33	0,23	6,89E-01
ENSRNOG00000014006	**Neto1**	neuropilin (NRP) and tolloid (TLL)-like	307206	18	82532693	82645708	113016	814,72	-0,22	6,89E-01

ENSRNOG00000023919	**Gbas**	glioblastoma amplified sequence (Gba	498174	12	32476020	32506643	30624	992,49	0,19	6,89E-01
ENSRNOG00000029209	**Rnf13**	ring finger protein 13 (Rnf13), mRNA [	681578	2	167261840	167446313	184474	457,84	-0,32	6,89E-01
ENSRNOG00000000541	**Glo1**	glyoxalase 1 (Glo1), mRNA [Source:R	294320	20	11461658	11479676	18019	2486,29	0,21	6,89E-01
ENSRNOG00000027646	**Pop4**	processing of precursor 4, ribonucleas	292831	1	95761043	95769376	8334	458,31	0,28	6,90E-01
ENSRNOG00000030334	**Adck5**	aarF domain containing kinase 5 (Adc	362943	7	117631880	117649865	17986	393,98	-0,18	6,91E-01
ENSRNOG00000001101	**Rnf216**	ring finger protein 216 (Rnf216), mRN	304294	12	15547724	15668304	120581	2470,68	0,18	6,91E-01
ENSRNOG00000001415	**Ap1s1**	adaptor-related protein complex 1, sig	360785	12	24677439	24688360	10922	3938,21	0,20	6,91E-01
ENSRNOG00000002227	**Kit**	v-kit Hardy-Zuckerman 4 feline sarcon	64030	14	34903278	34979346	76069	1809,89	-0,31	6,91E-01
ENSRNOG00000004303	**Timp3**	TIMP metallopeptidase inhibitor 3 (Tim	25358	7	23694454	23744372	49919	1912,84	-0,26	6,91E-01
ENSRNOG00000009696	**Mdm4**	Mdm4 p53 binding protein homolog (m	304798	13	54863121	54899047	35927	393,15	-0,22	6,91E-01
ENSRNOG00000023789	**LOC10090**	Protein LOC100909434 [Source:UniP	1E+08	2	201528306	201668906	140601	71,26	-0,31	6,91E-01
ENSRNOG00000024874	**Prtfdc1**	phosphoribosyl transferase domain co	291355	17	89615538	89724742	109205	1090,16	0,22	6,91E-01
ENSRNOG00000005753	**Alg13**	asparagine-linked glycosylation 13 (Al	300284	X	114015104	114021925	6822	90,64	-0,29	6,91E-01
ENSRNOG00000005984	**Etv6**	ets variant 6 (Etv6), mRNA [Source:Re	312777	4	230278004	230512007	234004	129,39	-0,28	6,91E-01
ENSRNOG00000021680		Protein Setdb2 [Source:UniProtKB/Tr	1E+08	15	43533876	43566314	32439	121,93	-0,28	6,91E-01
ENSRNOG00000022234	**mrpl24**	mitochondrial ribosomal protein L24 (r	295224	2	206691661	206697673	6013	551,86	0,21	6,91E-01
ENSRNOG00000000639	**Ado**	2-aminoethanethiol (cysteamine) dioxy	309732	20	24545376	24546303	928	324,71	0,25	6,92E-01
ENSRNOG00000023348		TBC1 domain family, member 2 (Tbc1	313234	5	66739641	66785976	46336	39,00	-0,30	6,92E-01
ENSRNOG00000001815	**Eif4a2**	eukaryotic translation initiation factor 4	303831	11	84411366	84417958	6593	4682,72	-0,28	6,92E-01
ENSRNOG00000005196	**Slc12a6**	solute carrier family 12, member 6 (Slc	691209	3	110348100	110446527	98428	1731,14	-0,25	6,92E-01
ENSRNOG00000017491	**Carf**	calcium responsive transcription factor	301446	9	66702057	66739587	37531	154,68	-0,30	6,92E-01
ENSRNOG00000018019	**Hspa12a**	heat shock protein 12A (Hspa12a), mR	307997	1	287309702	287379109	69408	3246,91	-0,25	6,92E-01
ENSRNOG00000019868	**Psma5**	proteasome (prosome, macropain) sul	29672	2	230519705	230543036	23332	2280,34	0,19	6,92E-01
ENSRNOG00000020002	**Parp11**	Protein Parp11 [Source:UniProtKB/Tr	500323	4	231553882	231572522	18641	301,72	0,26	6,92E-01
ENSRNOG00000046257	**Ctdspl**	CTD (carboxy-terminal domain, RNA p	301056	8	126933361	126939913	6553	50,57	0,30	6,92E-01
ENSRNOG00000007493	**Xrcc2**	X-ray repair complementing defective	499966	4	5865957	5884492	18536	355,61	0,25	6,92E-01
ENSRNOG00000017911	**Fam115a**	Protein Fam115a; Similar to mKIAA07	362353	4	136915531	136965939	50409	3992,31	0,21	6,92E-01
ENSRNOG00000016892	**Nr2f6**	nuclear receptor subfamily 2, group F,	245980	16	19630090	19637536	7447	1407,58	0,36	6,92E-01
ENSRNOG00000009149	**Jam3**	junctional adhesion molecule 3 (Jam3	315509	8	28166276	28227631	61356	1677,66	0,23	6,92E-01
ENSRNOG00000031612	**Gls2**	glutaminase 2 (liver, mitochondrial) (G	192268	7	2584755	2600728	15974	179,16	0,27	6,92E-01
ENSRNOG00000045639	**RGD15608**	Protein RGD1560883 [Source:UniPro	294603	2	4250417	4442634	192218	104,82	-0,24	6,93E-01
ENSRNOG00000008159	**Msantd3**	Myb/SANT-like DNA-binding domain c	362516	5	68798535	68820976	22442	818,21	0,17	6,93E-01
ENSRNOG00000006938		mitogen-activated protein kinase 8 inte	116457	3	87998492	88016197	17706	11704,73	0,22	6,93E-01
ENSRNOG00000011153	**Il17ra**	interleukin 17 receptor A (Il17ra), mRN	312679	4	220083472	220106001	22530	365,16	0,24	6,93E-01
ENSRNOG00000016565				1	76158681	76159328	648	72,21	0,28	6,93E-01
ENSRNOG00000030352	**Pcdhga9**	protocadherin gamma subfamily A, 9 (	252895	18	30579832	30582436	2605	76,06	-0,25	6,93E-01
ENSRNOG00000032554		Protein Scd4 [Source:UniProtKB/TrEI	499358	1	271537000	271548263	11264	122,30	-0,30	6,93E-01
ENSRNOG00000033015	**LOC10091**	Ac2-032; Protein LOC100910150; RC	1E+08	18	51646959	51675579	28621	20,83	-0,34	6,93E-01

ENSRNOG00000013829	**Chrna3**	cholinergic receptor, nicotinic, alpha 3	25101	8	58175886	58189161	13276	41,46	-0,32	6,93E-01
ENSRNOG00000016906	**Lipt2**	lipoyl(octanoyl) transferase 2 (putative	365314	1	171371500	171373827	2328	159,09	-0,23	6,93E-01
ENSRNOG00000015636	**Wdr34**	Protein Wdr34; WD repeat domain 34	296618	3	13950972	13966415	15444	955,98	0,22	6,93E-01
ENSRNOG00000032941	**Mab21l1**	mab-21-like 1 (C. elegans) (Mab21l1),	688394	2	164587785	164590174	2390	19,30	-0,40	6,93E-01
ENSRNOG00000000130	**Dnajb5**	DnaJ (Hsp40) homolog, subfamily B, r	313811	5	62918616	62927261	8646	2855,52	0,19	6,94E-01
ENSRNOG00000003215	**Zfp287**	zinc finger protein 287 (Zfp287), mRN/	303212	10	48913568	48930648	17081	330,26	-0,28	6,94E-01
ENSRNOG00000015294	**RGD13062**	uncharacterized protein LOC296565 |	296565	3	2660403	2661945	1543	1186,00	0,24	6,94E-01
ENSRNOG00000017671	**Rasa3**	RAS p21 protein activator 3 (Rasa3), r	29372	16	80808492	80922641	114150	1463,34	-0,21	6,94E-01
ENSRNOG00000032492	**Usp22**	ubiquitin specific peptidase 22 (Usp22	303201	10	47203849	47227946	24098	9445,56	-0,23	6,94E-01
ENSRNOG00000032799				19	53265161	53265763	603	37,02	-0,33	6,94E-01
ENSRNOG00000024663		FK506 binding protein 15 [Source:MG	362528	5	82255154	82311236	56083	946,38	0,17	6,94E-01
ENSRNOG00000031127	**LOC10036**	small nuclear ribonucleoprotein polype	360844	8	128569282	128569560	279	434,86	0,18	6,94E-01
ENSRNOG00000025418	**Armc9**	armadillo repeat containing 9 (Armc9),	301579	9	92901738	93029061	127324	1596,73	0,19	6,94E-01
ENSRNOG00000007632	**Mss51**	MSS51 mitochondrial translational act	289904	15	8341460	8353329	11870	31,29	-0,38	6,94E-01
ENSRNOG00000010214	**Scrn2**	secernin 2 (Scrn2), mRNA [Source:Re	360612	10	84757537	84761506	3970	224,23	-0,29	6,94E-01
ENSRNOG00000010331	**Ctsb**	cathepsin B (Ctsb), mRNA [Source:Re	64529	15	50081535	50102406	20872	9836,83	-0,24	6,94E-01
ENSRNOG00000010415	**Atxn7l4**	Protein Atxn7l4 [Source:UniProtKB/Tr	362726	6	62253461	62294041	40581	219,06	-0,25	6,94E-01
ENSRNOG00000015936	**LOC10036**	guanine nucleotide binding protein (G	79218	2	270886941	270895438	8498	507,64	-0,32	6,94E-01
ENSRNOG00000032910		regulatory factor X 8 [Source:MGI Syn	501127	9	46101251	46161904	60654	42,18	0,36	6,94E-01
ENSRNOG00000042253		ecdysoneless homolog (Drosophila) [S	688968	15	8502035	8531649	29615	754,62	0,25	6,94E-01
ENSRNOG00000010254	**Htr1a**	5-hydroxytryptamine (serotonin) recep	24473	2	55362662	55363930	1269	24,18	-0,39	6,94E-01
ENSRNOG00000001833	**Mcm4**	minichromosome maintenance comple	29728	11	92565514	92579215	13702	583,87	0,27	6,94E-01
ENSRNOG00000004606	**Meis1**	Meis homeobox 1 (Meis1), mRNA [So	686117	14	102927228	103063968	136741	657,88	0,21	6,94E-01
ENSRNOG00000010104	**Pofut1**	protein O-fucosyltransferase 1 (Pofut1	311551	3	155352444	155374642	22199	393,01	0,25	6,94E-01
ENSRNOG00000017237	**Lama1**	laminin, alpha 1 (Lama1), mRNA [Sou	316758	9	115406905	115531632	124728	52,83	-0,34	6,94E-01
ENSRNOG00000020914	**Sptbn4**	spectrin, beta, non-erythrocytic 4 (Sptl	308458	1	85379607	85463656	84050	2142,04	0,23	6,94E-01
ENSRNOG00000022331		Protein Ccdc78; Uncharacterized prot	302996	10	14969085	14972980	3896	18,40	-0,39	6,94E-01
ENSRNOG00000015278	**Myl12a**	myosin, light chain 12A, regulatory, no	501203	9	118779626	118787509	7884	1159,58	0,27	6,95E-01
ENSRNOG00000038239	**RGD15615**	uncharacterized protein LOC292078 |	292078	19	66715538	66727297	11760	265,02	0,25	6,95E-01
ENSRNOG00000030449	**Gsta4**	glutathione S-transferase alpha 4 (Gst	300850	8	85067703	85078443	10741	306,89	0,27	6,95E-01
ENSRNOG00000002099	**Wdfy3**	WD repeat and FYVE domain containi	305164	14	9200912	9346382	145471	7140,13	-0,14	6,95E-01
ENSRNOG00000012980	**Hyls1**	Protein Hyls1 [Source:UniProtKB/TrE	680262	8	36779967	36780908	942	127,83	-0,24	6,95E-01
ENSRNOG00000021063	**Grin2d**	glutamate receptor, ionotropic, N-meth	24412	1	102899803	102935845	36043	251,45	-0,33	6,95E-01
ENSRNOG00000010428	**Bahd1**	Protein Bahd1; Similar to mKIAA0945	362194	3	117261470	117268034	6565	846,39	0,19	6,95E-01
ENSRNOG00000017036	**Sec11c**	SEC11 homolog C (S. cerevisiae) (Se	266758	18	60686314	60702656	16343	883,22	-0,28	6,95E-01
ENSRNOG00000004857	**L2hgdh**	L-2-hydroxyglutarate dehydrogenase (	314196	6	101466089	101507172	41084	177,89	-0,24	6,95E-01
ENSRNOG00000005686	**Suclg2**	succinate-CoA ligase, GDP-forming, b	362404	4	192061201	192333899	272699	1942,80	-0,31	6,95E-01
ENSRNOG00000036329	**Mir568**	rno-mir-568 [Source:miRBase;Acc:MI0012594]		11	61129098	61129193	96	23,21	-0,36	6,95E-01

ENSRNOG00000021628	**Wdr89**	WD repeat domain 89 (Wdr89), mRNA	314243	6	108017412	108023798	6387	77,29	-0,31	6,96E-01
ENSRNOG00000002905	**Ccdc103**	coiled-coil domain containing 103 (Ccd	498006	10	90756615	90760015	3401	33,59	-0,39	6,96E-01
ENSRNOG00000004395	**Fbxw11**	F-box and WD repeat domain containi	303024	10	17430246	17528590	98345	4275,11	0,23	6,96E-01
ENSRNOG00000014620	**Rhbdd1**	rhomboid domain containing 1 (Rhbdd	316557	9	87882639	87975590	92952	89,20	-0,32	6,96E-01
ENSRNOG00000016099	**Id4**	inhibitor of DNA binding 4 (Id4), mRNA	291023	17	18744511	18747080	2570	1298,91	-0,26	6,96E-01
ENSRNOG00000019514	**RGD13057**	similar to RIKEN cDNA 3110040N11 (I	293059	1	144544564	144551617	7054	165,69	0,29	6,96E-01
ENSRNOG00000047448		thyroid hormone receptor interactor 10 [Source:M		9	8696543	8709634	13092	278,56	0,23	6,96E-01
ENSRNOG00000047975				1	206299224	206299869	646	1188,42	0,31	6,96E-01
ENSRNOG00000014102	**Wdr38**	Protein Wdr38 [Source:UniProtKB/TrE	366035	3	28513535	28516643	3109	29,33	-0,38	6,96E-01
ENSRNOG00000002759	**Lias**	lipoic acid synthetase (Lias), mRNA [S	305348	14	44326599	44343665	17067	486,64	0,22	6,96E-01
ENSRNOG00000043245		Uncharacterized protein [Source:UniProtKB/TrE		1	58175407	58184738	9332	70,00	-0,34	6,96E-01
ENSRNOG00000037217	**Nudt17**	nudix (nucleoside diphosphate linked m	502584	2	218318480	218320972	2493	32,99	-0,36	6,97E-01
ENSRNOG00000000795	**RT1-N3**	RT1 class Ib, locus N3 (RT1-N3), mRN	24750	20	5265245	5269111	3867	251,30	0,30	6,97E-01
ENSRNOG00000001525	**Igsf11**	immunoglobulin superfamily, member	303926	11	67684930	67819746	134817	1977,62	-0,34	6,97E-01
ENSRNOG00000002545	**RGD13097**	similar to CG4768-PA (RGD1309748),	302913	10	5639257	5668196	28940	1867,08	-0,20	6,97E-01
ENSRNOG00000002654	**FAM120C**	Protein FAM120C [Source:UniProtKB	317423	X	22434579	22497841	63263	1926,85	-0,22	6,97E-01
ENSRNOG00000004059	**Ptrhd1**	peptidyl-tRNA hydrolase domain conta	298861	6	38469515	38472900	3386	445,76	0,33	6,97E-01
ENSRNOG00000004103	**Trappc6b**	trafficking protein particle complex 6B	299075	6	89667998	89679088	11091	910,37	0,19	6,97E-01
ENSRNOG00000004696	**Arhgap5**	Rho GTPase activating protein 5 (Arhg	299012	6	82918712	82926533	7822	1062,60	-0,38	6,97E-01
ENSRNOG00000008595	**Ttc12**	tetratricopeptide repeat domain 12 (Tt	300696	8	52390859	52436391	45533	44,57	-0,33	6,97E-01
ENSRNOG00000008917		EH domain binding protein 1 [Source:I	305556	14	106937303	107187814	250512	1464,71	-0,16	6,97E-01
ENSRNOG00000009196	**Rc3h2**	ring finger and CCCH-type domains 2	311909	3	26866072	26909177	43106	499,06	-0,28	6,97E-01
ENSRNOG00000011704	**Fbxo34**	F-box protein 34 (Fbxo34), mRNA [So	305830	15	28202792	28270018	67227	1005,82	0,21	6,97E-01
ENSRNOG00000012865	**Parp3**	poly (ADP-ribose) polymerase family, i	300985	8	114537740	114543478	5739	127,80	-0,35	6,97E-01
ENSRNOG00000014482	**Fam178a**	family with sequence similarity 178, m	499360	1	272113227	272175693	62467	762,00	-0,19	6,97E-01
ENSRNOG00000015124	**Gpam**	glycerol-3-phosphate acyltransferase,	29653	1	283249329	283278757	29429	2693,59	-0,20	6,97E-01
ENSRNOG00000017251		WD repeat-containing protein 55 [Sou	307494	18	29295418	29312145	16728	1336,76	0,25	6,97E-01
ENSRNOG00000017418	**RGD15608**	ribosomal protein S3 (Rps3), mRNA [S	140654	1	170637738	170643027	5290	14327,50	0,30	6,97E-01
ENSRNOG00000017794	**Fuk**	fucokinase (Fuk), mRNA [Source:RefS	307848	19	54167905	54190881	22977	352,80	0,18	6,97E-01
ENSRNOG00000019948	**Phf20**	PHD finger protein 20 (Phf20), mRNA	311575	3	159312084	159417512	105429	2059,02	-0,16	6,97E-01
ENSRNOG00000025185	**Nup188**	Protein LOC100911302 [Source:UniP	366016	3	14205291	14261380	56090	1248,11	0,15	6,97E-01
ENSRNOG00000025937	**Zfp26**	zinc finger protein 26 (Zfp26), mRNA [	367033	8	21503262	21515604	12343	445,69	-0,27	6,97E-01
ENSRNOG00000029146	**LOC10036**	similar to 1700123O20Rik protein (RG	361038	15	37218216	37222470	4255	2002,82	0,28	6,97E-01
ENSRNOG00000032364	**Tbcel**	tubulin folding cofactor E-like (Tbcel),	315591	8	45168898	45224997	56100	457,68	0,20	6,97E-01
ENSRNOG00000042647	**Rad51ap1**	RAD51 associated protein 1 (Rad51ap	689055	4	232130924	232143989	13066	232,49	0,23	6,97E-01
ENSRNOG00000046353		Protein LOC501406 [Source:UniProtK	501406	1	39953348	39957282	3935	32,02	-0,40	6,97E-01
ENSRNOG00000049517	**Tnfaip3**	Protein LOC683206 [Source:UniProtK	683206	1	15954045	15963939	9895	94,65	-0,30	6,97E-01
ENSRNOG00000050516		Uncharacterized protein [Source:UniProtKB/TrE		4	140627054	140627221	168	20,09	-0,34	6,97E-01

ENSRNOG00000019817	**Ddx28**	DEAD (Asp-Glu-Ala-Asp) box polypep	364995	19	48855707	48857329	1623	380,58	0,18	6,97E-01
ENSRNOG00000006118	**Klf10**	Kruppel-like factor 10 (Klf10), mRNA [S	81813	7	77258950	77265321	6372	1027,28	-0,32	6,97E-01
ENSRNOG00000010537	**Dis3l**	DIS3 mitotic control homolog (S. cerev	363077	8	69480088	69516482	36395	519,68	-0,15	6,97E-01
ENSRNOG00000010635	**Igfbp4**	insulin-like growth factor binding prote	360622	10	86747476	86759481	12006	587,77	-0,36	6,97E-01
ENSRNOG00000013574	**Appl1**	Protein Appl1 [Source:UniProtKB/TrE	290537	16	2537603	2576791	39189	937,03	-0,27	6,97E-01
ENSRNOG00000014673	**Eri2**	ERI1 exoribonuclease family member	691484	1	196474508	196484254	9747	165,72	-0,26	6,97E-01
ENSRNOG00000018057	**Mrpl2**	mitochondrial ribosomal protein L2 (Mi	301240	9	15550237	15554009	3773	539,41	0,21	6,97E-01
ENSRNOG00000022635		discs, large homolog 2 (Drosophila) (D	64053	1	162798344	163514250	715907	2149,45	-0,20	6,97E-01
ENSRNOG00000027615		Uncharacterized protein [Source:UniProtKB/TrE		10	93159138	93160489	1352	56,21	-0,26	6,97E-01
ENSRNOG00000027936				1	80181380	80183335	1956	206,84	-0,28	6,97E-01
ENSRNOG00000042406		protein N-terminal asparagine amidoh	360462	10	2096021	2112744	16724	784,53	0,17	6,97E-01
ENSRNOG00000017165	**Akr1e2**	aldo-keto reductase family 1, member	307091	17	71079204	71093695	14492	302,56	0,32	6,98E-01
ENSRNOG00000049802		Uncharacterized protein [Source:UniProtKB/TrE		18	17755066	17830235	75170	965,92	0,22	6,98E-01
ENSRNOG00000016573	**Dgat2**	diacylglycerol O-acyltransferase 2 (Dg	252900	1	170316041	170346306	30266	1247,41	-0,22	6,98E-01
ENSRNOG00000012513	**Pdk3**	pyruvate dehydrogenase kinase, isozy	296849	X	63428816	63496239	67424	606,66	0,24	6,98E-01
ENSRNOG00000007890	**Usp1**	ubiquitin specific peptidase 1 (Usp1), r	313387	5	121525009	121536377	11369	557,94	-0,31	6,98E-01
ENSRNOG00000022320	**Ccdc111**	Protein Ccdc111 [Source:UniProtKB/T	361147	16	48584457	48613173	28717	27,06	0,36	6,99E-01
ENSRNOG00000020624	**Acadsb**	acyl-CoA dehydrogenase, short/branc	25618	1	209013684	209048776	35093	268,58	-0,25	6,99E-01
ENSRNOG00000013089	**Kif13b**	kinesin family member 13B (Kif13b), m	305967	15	51779297	51936975	157679	512,57	0,18	6,99E-01
ENSRNOG00000000848	**Gpank1**	G patch domain and ankyrin repeats 1	415064	20	7186598	7189641	3044	596,30	0,20	6,99E-01
ENSRNOG00000013045	**Setd7**	SET domain containing (lysine methylt	689954	2	160051126	160093343	42218	464,29	0,25	6,99E-01
ENSRNOG00000015844	**Snrpd2**	small nuclear ribonucleoprotein D2 po	680309	1	81324033	81326882	2850	1544,07	-0,27	6,99E-01
ENSRNOG00000018417	**Naa35**	N(alpha)-acetyltransferase 35, NatC a	64472	17	7636176	7688566	52391	1640,35	0,18	6,99E-01
ENSRNOG00000028100	**Pex11g**	peroxisomal biogenesis factor 11 gam	288369	12	4163473	4170562	7090	78,80	0,34	6,99E-01
ENSRNOG00000046318	**LOC10091**	Protein LOC100910689; RCG35252 [	690300	10	78023828	78028723	4896	185,90	0,33	6,99E-01
ENSRNOG00000048817				X	64865963	64867861	1899	544,87	0,24	6,99E-01
ENSRNOG00000046870		Uncharacterized protein [Source:UniProtKB/TrE		12	31188210	31212714	24505	119,50	-0,28	6,99E-01
ENSRNOG00000019933	**Ubqln4**	ubiquilin 4 (Ubqln4), mRNA [Source:R	310633	2	207318101	207333300	15200	2547,06	-0,16	6,99E-01
ENSRNOG00000006778	**Mmp19**	matrix metallopeptidase 19 (Mmp19),	304608	7	3187862	3195640	7779	228,32	-0,39	6,99E-01
ENSRNOG00000007060	**Plin2**	perilipin 2 (Plin2), mRNA [Source:RefS	298199	5	108985563	108999161	13599	305,41	0,23	6,99E-01
ENSRNOG00000007359	**Gca**	grancalcin (Gca), mRNA [Source:RefS	295647	3	55291465	55324447	32983	578,35	-0,23	6,99E-01
ENSRNOG00000011231	**Cntnap4**	contactin associated protein-like 4 (Cntnap4), ml		19	55878687	55952179	73493	622,01	-0,31	6,99E-01
ENSRNOG00000016722	**LOC10091**	exportin 6 (Xpo6), mRNA [Source:RefS	293476	1	204484096	204573897	89802	3347,35	0,18	6,99E-01
ENSRNOG00000017404	**Pcmtd2**	protein-L-isoaspartate (D-aspartate) O	311726	3	181059923	181079779	19857	1375,42	-0,27	6,99E-01
ENSRNOG00000025462	**Wdr41**	WD repeat domain 41 (Wdr41), mRNA	361879	2	43827385	43876536	49152	1346,21	0,22	6,99E-01
ENSRNOG00000031834	**Nkain4**	Na+/K+ transporting ATPase interactir	296469	3	180154361	180174989	20629	913,80	-0,38	6,99E-01
ENSRNOG00000037647	**Ostm1**	osteopetrosis associated transmembra	499474	20	49062741	49092280	29540	1535,14	0,23	6,99E-01
ENSRNOG00000010534	**Ankrd50**	ankyrin repeat domain 50 (Ankrd50), r	294988	2	144894601	144925135	30535	1020,48	-0,20	7,00E-01

ENSRNOG00000025142	**Mex3b**	mex3 homolog B (C. elegans) (Mex3b	308790	1	145760227	145764462	4236	1886,03	0,26	7,00E-01
ENSRNOG00000020006		WD repeat domain 90 [Source:MGI Symbol;Acc:		10	15024903	15041178	16276	543,22	0,18	7,00E-01
ENSRNOG00000011492		zinc finger protein 866 [Source:MGI Symbol;Acc		16	21313542	21316847	3306	157,90	-0,21	7,00E-01
ENSRNOG00000009364	**Ndufb9**	NADH dehydrogenase (ubiquinone) 1	299954	7	99411522	99417939	6418	4138,34	0,28	7,00E-01
ENSRNOG00000021712	**Rcbtb1**	regulator of chromosome condensatio	361050	15	43439885	43481994	42110	662,55	0,19	7,00E-01
ENSRNOG00000031782		collagen, type VI, alpha 4 [Source:MGI Symbol;A		8	114039958	114137875	97918	31,25	-0,38	7,00E-01
ENSRNOG00000050611		Uncharacterized protein [Source:UniProtKB/TrE		8	124483148	124796061	312914	20,84	0,34	7,00E-01
ENSRNOG00000005535		IKAROS family zinc finger 4 [Source:M	1E+08	7	3033150	3047553	14404	289,52	0,26	7,01E-01
ENSRNOG00000010688	**Nrgn**	neurogranin (Nrgn), mRNA [Source:Re	64356	8	40015873	40024017	8145	1185,08	-0,30	7,01E-01
ENSRNOG00000018564	**Nup93**	nucleoporin 93 (Nup93), mRNA [Sourc	291874	19	11137505	11238898	101394	996,39	0,20	7,01E-01
ENSRNOG00000046969	**Ubxn7**	UBX domain protein 7 (Ubxn7), mRNA	303878	11	74746898	74779143	32246	54,69	0,29	7,01E-01
ENSRNOG00000005081	**Fbxl20**	F-box and leucine-rich repeat protein 2	64039	10	85847965	85896206	48242	270,61	0,27	7,01E-01
ENSRNOG00000042068	**Otud7b**	OTU domain containing 7B (Otud7b),	310677	2	217720895	217787184	66290	690,50	0,22	7,01E-01
ENSRNOG00000005857	**Lrrc3b**	Protein Lrrc3b; RCG42160, isoform CR	305705	15	15338715	15339494	780	102,31	-0,31	7,01E-01
ENSRNOG00000008941		v-ets avian erythroblastosis virus E26	24356	8	33858338	33921593	63256	702,36	-0,37	7,01E-01
ENSRNOG00000018144	**Depdc5**	DEP domain containing 5 (Depdc5), m	305464	14	83775510	83906306	130797	1331,43	0,20	7,01E-01
ENSRNOG00000022967	**Slc35d1**	solute carrier family 35 (UDP-GlcA/UD	298280	5	126567574	126612299	44726	163,80	-0,23	7,01E-01
ENSRNOG00000043114	**Tomm7**	translocase of outer mitochondrial mer	685620	4	7844007	7850847	6841	1770,87	-0,32	7,01E-01
ENSRNOG00000011543	**Fam118b**	family with sequence similarity 118, m	315549	8	36435180	36456913	21734	503,38	0,23	7,01E-01
ENSRNOG00000018906	**Ghdc**	GH3 domain containing (Ghdc), mRNA	303542	10	88467004	88471225	4222	392,63	0,28	7,01E-01
ENSRNOG00000017675	**Pard6g**	par-6 partitioning defective 6 homolog	307237	18	72178893	72246274	67382	177,36	0,19	7,02E-01
ENSRNOG00000026497	**Pigc**	phosphatidylinositol glycan anchor bio	364032	13	84780658	84783012	2355	900,85	0,24	7,02E-01
ENSRNOG00000046507		regulation of nuclear pre-mRNA domain containi		2	217398482	217426719	28238	210,46	-0,19	7,02E-01
ENSRNOG00000003825	**Wdr75**	WD repeat domain 75 (Wdr75), mRNA	314545	9	52359647	52383504	23858	326,16	-0,25	7,02E-01
ENSRNOG00000004183	**Ccdc59**	coiled-coil domain containing 59 (Ccdc	314799	7	47942423	47948975	6553	141,70	0,29	7,02E-01
ENSRNOG00000018681	**LOC10091**	nestin (Nes), mRNA [Source:RefSeq r	25491	2	206747073	206755585	8513	308,76	0,27	7,02E-01
ENSRNOG00000027341		teneurin-2 [Source:RefSeq peptide;Ac	117242	10	20716458	21085081	368624	4043,62	-0,26	7,02E-01
ENSRNOG00000005446	**Gna11**	guanine nucleotide binding protein, alp	81662	7	11200712	11214475	13764	1559,54	0,23	7,02E-01
ENSRNOG00000007489		zinc finger protein 41 [Source:MGI Symbol;Acc:M		7	116548746	116549342	597	237,05	0,23	7,02E-01
ENSRNOG00000009846	**Frg1**	Protein Frg1 [Source:UniProtKB/TrEMBL;Acc:D		16	53671702	53692373	20672	62,77	-0,28	7,02E-01
ENSRNOG00000012799	**Prkaa1**	protein kinase, AMP-activated, alpha 1	65248	2	73882080	73914818	32739	340,77	0,20	7,02E-01
ENSRNOG00000015428	**Mff**	mitochondrial fission factor (Mff), trans	301563	9	90620315	90648466	28152	129,34	0,23	7,02E-01
ENSRNOG00000020897	**Prpf19**	pre-mRNA processing factor 19 (Prpf1	246216	1	234012654	234023693	11040	10083,28	0,17	7,02E-01
ENSRNOG00000004863	**Mpped2**	metallophosphoesterase domain conta	362185	3	104336355	104510581	174227	1684,84	-0,20	7,02E-01
ENSRNOG00000037850	**Marc2**	mitochondrial amidoxime reducing com	171451	13	107401555	107432931	31377	689,42	0,27	7,02E-01
ENSRNOG00000049121	**Bysl**	bystin-like (Bysl), mRNA [Source:RefS	359727	9	14314823	14324573	9751	701,73	0,20	7,02E-01
ENSRNOG00000007901	**Tmed10**	transmembrane emp24-like trafficking	84599	6	116347918	116383215	35298	2649,62	-0,16	7,02E-01
ENSRNOG00000008039	**Cul5**	cullin 5 (Cul5), mRNA [Source:RefSeq	64624	8	56785487	56835567	50081	544,02	-0,34	7,02E-01

ENSRNOG00000019069	**Nxf1**	nuclear RNA export factor 1 (Nxf1), ml	59087	1	231895704	231908900	13197	2492,59	0,17	7,02E-01
ENSRNOG00000020095	**Pcdhb3**	protocadherin beta 3 (Pcdhb3), mRNA	291656	18	30088851	30091405	2555	247,34	-0,27	7,02E-01
ENSRNOG00000024461	**March11**	membrane-associated ring finger (C3H	499558	2	98502842	98603754	100913	90,97	-0,25	7,02E-01
ENSRNOG00000029304		angiogenic factor with G patch and FH	310005	2	44228102	44253809	25708	952,09	0,21	7,02E-01
ENSRNOG00000000909	**Uspl1**	ubiquitin specific peptidase like 1 (Usp	288447	12	9026185	9052902	26718	1388,68	0,33	7,03E-01
ENSRNOG00000004076	**Pms1**	postmeiotic segregation increased 1 (S	494322	9	52784772	52871008	86237	103,78	-0,33	7,03E-01
ENSRNOG00000014416	**Cacng7**	calcium channel, voltage-dependent, g	140728	1	63367661	63393913	26253	2234,90	0,22	7,03E-01
ENSRNOG00000031915	**Hdac10**	histone deacetylase 10 (Hdac10), mR	362981	7	129788136	129793226	5091	384,20	0,27	7,03E-01
ENSRNOG00000049511		scaffold attachment factor B2 [Source:MGI Symb		9	9476267	9489685	13419	1164,57	0,21	7,03E-01
ENSRNOG00000016338	**Fam92a1**	Protein Fam92a1 [Source:UniProtKB/	297903	5	30436531	30454713	18183	720,12	-0,33	7,03E-01
ENSRNOG00000027087	**Tmem186**	transmembrane protein 186 (Tmem18	497863	10	5878822	5882132	3311	709,80	0,25	7,03E-01
ENSRNOG00000046617		Uncharacterized protein [Source:UniProtKB/TrE		1	19126968	19149888	22921	19,60	0,35	7,03E-01
ENSRNOG00000005138	**Pip4k2c**	phosphatidylinositol-5-phosphate 4-kir	140607	7	70661195	70676538	15344	1093,04	0,22	7,03E-01
ENSRNOG00000009799	**Dpagt1**	dolichyl-phosphate (UDP-N-acetylgluc	300668	8	47277020	47283503	6484	1251,75	0,23	7,04E-01
ENSRNOG00000021012	**Rfx5**	regulatory factor X, 5 (influences HLA	310659	2	215615266	215622781	7516	1072,78	0,31	7,04E-01
ENSRNOG00000001034		Uncharacterized protein [Source:UniProtKB/TrE		12	4740789	4756602	15814	2048,96	0,22	7,04E-01
ENSRNOG00000005719	**Emc4**	ER membrane protein complex subun	296049	3	110445980	110450999	5020	2010,16	0,17	7,04E-01
ENSRNOG00000020296	**LOC10036**	up-regulated during skeletal muscle gr	171069	1	274290376	274297223	6848	722,22	0,17	7,04E-01
ENSRNOG00000048862	**Mrps36**	mitochondrial ribosomal protein S36 (M	294696	2	49899205	49906987	7783	489,49	-0,18	7,04E-01
ENSRNOG00000023385		ribosomal protein L37a (Rpl37a), trans	1E+08	9	79774879	79776319	1441	3183,82	0,23	7,04E-01
ENSRNOG00000042545		Uncharacterized protein [Source:UniProtKB/TrE		3	101156306	101174118	17813	91,34	-0,25	7,04E-01
ENSRNOG00000019977	**Ptprf**	protein tyrosine phosphatase, recepto	360406	5	140826095	140893372	67278	6878,55	-0,14	7,04E-01
ENSRNOG00000043465	**Arc**	activity-regulated cytoskeleton-associa	54323	7	115812499	115815943	3445	130,37	-0,33	7,04E-01
ENSRNOG00000012318	**Aspm**	asp (abnormal spindle) homolog, micr	289054	13	61564059	61609831	45773	631,73	0,37	7,04E-01
ENSRNOG00000016477	**Vangl1**	VANGL planar cell polarity protein 1 (V	690366	2	224011038	224058132	47095	303,98	-0,26	7,04E-01
ENSRNOG00000009568	**Hdac1**	histone deacetylase 1 (Hdac1), mRNA	297893	5	151445686	151472652	26967	526,45	0,20	7,05E-01
ENSRNOG00000015749	**Nme3**	NME/NM23 nucleoside diphosphate ki	85269	10	14074396	14075352	957	476,11	-0,25	7,05E-01
ENSRNOG00000017119	**Fam96a**	family with sequence similarity 96, me	300797	8	71455205	71467170	11966	607,13	-0,22	7,05E-01
ENSRNOG00000024025	**Edc4**	enhancer of mRNA decapping 4 (Edc4	361399	19	48719427	48731412	11986	573,41	0,21	7,05E-01
ENSRNOG00000029308		lymphoid-restricted membrane protein	500361	4	243582231	243622022	39792	86,28	-0,31	7,05E-01
ENSRNOG00000008411		Protein Sec23b; SEC23B (S. cerevisiae) (Predic		3	145145484	145186894	41411	1005,79	-0,23	7,05E-01
ENSRNOG00000015320	**LOC10036**	ATP synthase, H+ transporting, mitoch	171082	7	142056432	142064809	8378	6667,68	0,32	7,05E-01
ENSRNOG00000020436	**RGD13117**	similar to sid2057p (RGD1311703), ml	293160	1	192525001	192536714	11714	4497,79	0,28	7,05E-01
ENSRNOG00000022159	**Epg5**	Protein Epg5 [Source:UniProtKB/TrEI	364902	18	73973504	74068456	94953	1347,06	0,21	7,05E-01
ENSRNOG00000009103	**Chrac1**	chromatin accessibility complex 1 (Ch	315058	7	114258512	114261899	3388	541,11	0,31	7,05E-01
ENSRNOG00000020000	**Tmem219**	Protein Tmem219; Tmem219 protein	308986	1	205305037	205334839	29803	478,96	0,25	7,05E-01
ENSRNOG00000048989		la-related protein 7 [Source:RefSeq p	686883	2	251212713	251227583	14871	392,34	0,36	7,05E-01
ENSRNOG00000009015	**Nfx1**	nuclear transcription factor, X-box binc	313166	5	61822485	61879030	56546	1687,00	0,17	7,05E-01

ENSRNOG00000010153	**Raf1**	v-raf-leukemia viral oncogene 1 (Raf1)	24703	4	210819150	210836805	17656	3288,36	-0,15	7,05E-01
ENSRNOG00000011226		Protein Timm8a1 [Source:UniProtKB/	84383	X	105241980	105249390	7411	110,27	-0,33	7,05E-01
ENSRNOG00000014576	**Dock3**	Protein Dock3 [Source:UniProtKB/TrE	315992	8	114988609	115302046	313438	3393,41	-0,17	7,05E-01
ENSRNOG00000018774	**Rps14**	ribosomal protein S14 (Rps14), mRNA	29284	18	55278634	55281581	2948	23,38	0,35	7,05E-01
ENSRNOG00000045859				4	27977886	27978978	1093	37,97	-0,31	7,05E-01
ENSRNOG00000042182		maestro heat-like repeat family member 2A [Sou		9	94997059	95032615	35557	42,01	-0,30	7,05E-01
ENSRNOG00000004870	**Dyrk3**	dual-specificity tyrosine-(Y)-phosphory	304775	13	52989585	52998242	8658	226,44	0,23	7,06E-01
ENSRNOG00000015609	**Wdr27**	Protein Wdr27 [Source:UniProtKB/TrE	308222	1	58076105	58125719	49615	123,61	0,31	7,06E-01
ENSRNOG00000018230	**Gtf2h2**	general transcription factor IIH, polype	294693	2	49654325	49682092	27768	231,26	-0,24	7,06E-01
ENSRNOG00000004527	**LOC10091**	lin-7 homolog a (C. elegans) (Lin7a), r	85327	7	49443158	49666753	223596	20,84	-0,33	7,06E-01
ENSRNOG00000013963	**Il6st**	interleukin 6 signal transducer (Il6st), r	25205	2	63332284	63361404	29121	2684,73	-0,26	7,06E-01
ENSRNOG00000047635	**Tmem178b**	transmembrane protein 178B (Tmem178b), mRI		4	67816889	67864206	47318	7797,28	-0,25	7,06E-01
ENSRNOG00000025174	**Kat6a**	K(lysine) acetyltransferase 6A (Kat6a)	306571	16	73577853	73655378	77526	3092,02	0,13	7,06E-01
ENSRNOG00000020347	**Nup98**	nucleoporin 98 (Nup98), mRNA [Sourc	81738	1	173402761	173497459	94699	1889,80	-0,18	7,06E-01
ENSRNOG00000000568	**Slc29a3**	solute carrier family 29 (equilibrative n	353307	20	32099778	32135978	36201	348,33	0,27	7,06E-01
ENSRNOG00000001643	**Psmg1**	proteasome (prosome, macropain) ass	288236	11	39858073	39866253	8181	588,90	0,22	7,06E-01
ENSRNOG00000001847	**Ccdc91**	coiled-coil domain containing 91 (Ccd	312863	4	246022044	246189263	167220	445,24	0,24	7,06E-01
ENSRNOG00000003207	**Fmnl1**	formin-like 1 (Fmnl1), mRNA [Source:I	287746	10	91025570	91052384	26815	541,96	0,21	7,06E-01
ENSRNOG00000004668	**Slc25a35**	solute carrier family 25, member 35 (S	497933	10	55297129	55301122	3994	41,63	-0,28	7,06E-01
ENSRNOG00000008841	**Orc1**	origin recognition complex, subunit 1 (	313479	5	132031219	132050956	19738	70,96	0,25	7,06E-01
ENSRNOG00000009768	**Npy**	neuropeptide Y (Npy), mRNA [Source:	24604	4	144233780	144240952	7173	82,27	0,36	7,06E-01
ENSRNOG00000010353	**LOC69018**	DnaJ (Hsp40) homolog, subfamily B, r	362293	4	2761869	2825295	63427	1266,88	0,27	7,06E-01
ENSRNOG00000013916		neurofilament, medium polypeptide [Source:MG		15	46736822	46741141	4320	2295,00	0,34	7,06E-01
ENSRNOG00000016460	**Clu**	clusterin (Clu), mRNA [Source:RefSeq	24854	15	48926516	48939266	12751	8396,91	-0,37	7,06E-01
ENSRNOG00000016980	**Qprt**	quinolinate phosphoribosyltransferase	293504	1	205534751	205550041	15291	159,85	-0,38	7,06E-01
ENSRNOG00000018669	**Jak3**	Janus kinase 3 (Jak3), mRNA [Source	25326	16	19970616	19981122	10507	126,85	0,32	7,06E-01
ENSRNOG00000019328	**Phgdh**	phosphoglycerate dehydrogenase (Ph	58835	2	219959694	219988834	29141	9654,82	-0,34	7,06E-01
ENSRNOG00000020354	**Mrpl23**	mitochondrial ribosomal protein L23 (M	64360	1	222596334	222604103	7770	1232,83	0,31	7,06E-01
ENSRNOG00000021732	**Hsf1**	heat shock transcription factor 1 (Hsf1	79245	7	117526154	117553109	26956	577,99	0,22	7,06E-01
ENSRNOG00000028580	**Pcnxl2**	Protein Pcnxl2 [Source:UniProtKB/TrE	307949	19	69280871	69429521	148651	572,06	0,25	7,06E-01
ENSRNOG00000038766	**Atxn1l**	Protein Atxn1l [Source:UniProtKB/TrE	307970	19	52757281	52759320	2040	450,71	0,27	7,06E-01
ENSRNOG00000042650		arginine decarboxylase [Source:MGI Symbol;Ac		5	150852123	150877849	25727	169,13	-0,25	7,06E-01
ENSRNOG00000046204		uncharacterized protein LOC679462 |	679462	20	33606532	33648074	41543	414,91	-0,31	7,06E-01
ENSRNOG00000047860	**Plin5**	perilipin 5 (Plin5), mRNA [Source:RefS	501283	9	9943003	9948729	5727	39,22	-0,38	7,06E-01
ENSRNOG00000042422	**St6galnac3**	ST6 (alpha-N-acetyl-neuraminyl-2,3-beta-galact		2	278043623	278046871	3249	234,79	-0,22	7,07E-01
ENSRNOG00000032618	**Mst1r**	macrophage stimulating 1 receptor (c-	300999	8	116040918	116054449	13532	18,64	-0,37	7,07E-01
ENSRNOG00000016587	**Ninj1**	ninjurin 1 (Ninj1), mRNA [Source:RefS	25338	17	17930044	17945158	15115	659,37	-0,33	7,07E-01
ENSRNOG00000017040	**Wrnip1**	Werner helicase interacting protein 1 (	282835	17	34825602	34845847	20246	1504,38	0,22	7,07E-01

ENSRNOG00000023591	**Ppp2r3c**	protein phosphatase 2, regulatory sub	362739	6	85593213	85615398	22186	268,53	-0,25	7,07E-01
ENSRNOG00000025296	**Lrrc8a**	leucine rich repeat containing 8 family,	311846	3	14155802	14181483	25682	1976,82	-0,21	7,07E-01
ENSRNOG00000031167		Ab2-390; Uncharacterized protein [Source:UniP		3	153952796	153981628	28833	149,71	-0,28	7,07E-01
ENSRNOG00000020371		post-GPI attachment to proteins 2 (Pg	116675	1	173497659	173523297	25639	281,58	-0,25	7,08E-01
ENSRNOG00000049095		Uncharacterized protein [Source:UniProtKB/TrE		4	212194431	212195922	1492	32,51	-0,32	7,08E-01
ENSRNOG00000031743	**Gbp2**	guanylate binding protein 2, interferon	171164	2	266805234	266821133	15900	279,09	0,38	7,08E-01
ENSRNOG00000047575	**Dhx34**	DEAH (Asp-Glu-Ala-His) box polypept	684903	1	79355591	79375768	20178	351,21	0,16	7,08E-01
ENSRNOG00000000288	**Scarf2**	scavenger receptor class F, member 2	287949	11	90774764	90786143	11380	281,84	-0,31	7,08E-01
ENSRNOG00000001156	**Msi1**	musashi RNA-binding protein 1 (Msi1)	259272	12	48696700	48717413	20714	1301,19	0,19	7,08E-01
ENSRNOG00000002928	**Guk1**	guanylate kinase 1 (Guk1), mRNA [So	303179	10	45291712	45299982	8271	2845,43	0,18	7,08E-01
ENSRNOG00000010620	**Ndc1**	NDC1 transmembrane nucleoporin (N	362557	5	130824420	130868755	44336	582,51	0,23	7,08E-01
ENSRNOG00000012346	**Epb41l2**	Protein Epb4.1l2 [Source:UniProtKB/	309557	1	22362882	22443605	80724	2207,44	-0,30	7,08E-01
ENSRNOG00000038607	**Tmem86b**	transmembrane protein 86B (Tmem86	690610	1	75747873	75750239	2367	47,50	-0,28	7,08E-01
ENSRNOG00000042487		Protein Bcl7b; Similar to B-cell CLL/lyr	368001	12	26541276	26541602	327	132,19	0,22	7,08E-01
ENSRNOG00000002468		tenascin R [Source:MGI Symbol;Acc:MGI:99516		13	82511491	82587627	76137	1626,88	-0,33	7,08E-01
ENSRNOG00000001389	**Irs3**	insulin receptor substrate 3 (Irs3), mR	84021	12	24140329	24142467	2139	20,31	-0,35	7,08E-01
ENSRNOG00000017065	**Arhgap28**	Rho GTPase activating protein 28 (Arl	301709	9	115546748	115711949	165202	69,42	-0,27	7,08E-01
ENSRNOG00000020622	**Cilp2**	cartilage intermediate layer protein 2 (	306356	16	21204960	21211953	6994	278,49	-0,23	7,09E-01
ENSRNOG00000014135	**Rab11fip4**	RAB11 family interacting protein 4 (cla	303337	10	64601743	64705829	104087	3257,50	0,27	7,09E-01
ENSRNOG00000036971	**RGD15663**	RGD1566320 (RGD1566320), mRNA	296207	3	145194837	145196325	1489	1147,21	0,24	7,09E-01
ENSRNOG00000048353		Antisense paternally expressed gene 3; Putative		1	71626810	71627741	932	1004,62	-0,24	7,09E-01
ENSRNOG00000004676	**Pak3**	p21 protein (Cdc42/Rac)-activated kin	29433	X	113372070	113489867	117798	977,19	-0,28	7,09E-01
ENSRNOG00000005564	**Ubn2**	ubinuclein 2 (Ubn2), mRNA [Source:R	312248	4	65977908	66044679	66772	496,98	0,17	7,09E-01
ENSRNOG00000005739	**Asap1**	ArfGAP with SH3 domain, ankyrin rep	314961	7	91673052	91955738	282687	1847,83	-0,17	7,09E-01
ENSRNOG00000006934	**Acvr1b**	activin A receptor, type IB (Acvr1b), ml	29381	7	140617642	140659124	41483	2038,18	0,22	7,09E-01
ENSRNOG00000007432	**Polr3b**	polymerase (RNA) III (DNA directed) p	362858	7	24893473	24997174	103702	806,07	0,22	7,09E-01
ENSRNOG00000007864	**Vrk2**	vaccinia related kinase 2 (Vrk2), mRN	360991	14	110444503	110576584	132082	35,42	0,35	7,09E-01
ENSRNOG00000009977	**Thrap3**	thyroid hormone receptor associated p	313591	5	147947769	147989301	41533	5142,50	0,22	7,09E-01
ENSRNOG00000010543	**Srpr**	signal recognition particle receptor ('do	315548	8	36428866	36434332	5467	1917,09	-0,22	7,09E-01
ENSRNOG00000012817	**Lrrc17**	leucine rich repeat containing 17 (Lrrc	502715	4	10109708	10139147	29440	42,10	-0,36	7,09E-01
ENSRNOG00000017405	**Raly**	RALY heterogeneous nuclear ribonucl	296301	3	156695466	156756861	61396	3573,65	0,18	7,09E-01
ENSRNOG00000017613	**Papd7**	PAP associated domain containing 7 (	306672	1	37797465	37830260	32796	1668,43	0,21	7,09E-01
ENSRNOG00000020138	**Slc4a3**	solute carrier family 4 (anion exchange	24781	9	82512289	82524380	12092	1088,04	-0,17	7,09E-01
ENSRNOG00000020460	**Banf1**	barrier to autointegration factor 1 (Ban	114087	1	227673553	227675550	1998	3966,74	0,25	7,09E-01
ENSRNOG00000023389	**Ephx4**	epoxide hydrolase 4 (Ephx4), mRNA [	289440	14	3364193	3393612	29420	117,20	-0,27	7,09E-01
ENSRNOG00000024886	**Ext1**	exostosin glycosyltransferase 1 (Ext1)	299907	7	93246664	93521815	275152	1258,31	0,20	7,09E-01
ENSRNOG00000029784	**Pak1**	p21 protein (Cdc42/Rac)-activated kin	29431	1	168974734	169089649	114916	3158,13	0,23	7,09E-01
ENSRNOG00000005943	**Sp8**	Sp8 transcription factor (Sp8), mRNA	299499	6	155693431	155696034	2604	167,84	0,26	7,09E-01

ENSRNOG00000006978	**Mterfd3**	MTERF domain containing 3 (Mterfd3)	366856	7	24788439	24798713	10275	69,15	-0,23	7,09E-01
ENSRNOG00000011136	**Osr2**	odd-skipped related 2 (Drosophila) (O:	315039	7	74213951	74221075	7125	45,86	-0,38	7,09E-01
ENSRNOG00000012004	**Ubap1**	ubiquitin-associated protein 1 (Ubap1)	362502	5	62265514	62306294	40781	1060,35	-0,16	7,09E-01
ENSRNOG00000018326	**Pgls**	6-phosphogluconolactonase (Pgls), m	290636	16	19880872	19886358	5487	2019,93	0,32	7,09E-01
ENSRNOG00000018788	**Btbd2**	Protein Btbd2 [Source:UniProtKB/TrE	500793	7	12097176	12110126	12951	1555,38	0,18	7,09E-01
ENSRNOG00000021057	**Osbp**	oxysterol binding protein (Osbp), mRN	365410	1	235460266	235490067	29802	3005,71	0,23	7,09E-01
ENSRNOG00000027229	**Slc35d2**	solute carrier family 35 (UDP-GlcNAc/	290959	17	1608197	1638555	30359	18,62	-0,36	7,09E-01
ENSRNOG00000029447	**Epc2l1**	enhancer of polycomb homolog 2 (Drc	362132	3	38799078	38897923	98846	341,01	-0,17	7,09E-01
ENSRNOG00000030317		apoptosis-inducing, TAF9-like domain	25305	5	169588757	169780881	192125	19336,04	-0,15	7,09E-01
ENSRNOG00000039815	**Zfp512**	Protein Zfp512 [Source:UniProtKB/Trl	313906	6	36109571	36137715	28145	1853,51	0,25	7,09E-01
ENSRNOG00000015642	**Pabpc4**	poly(A) binding protein, cytoplasmic 4	298510	5	144769098	144784395	15298	1243,27	-0,16	7,10E-01
ENSRNOG00000001482		Protein Gtf2ird2 [Source:UniProtKB/TrEMBL;Ac		12	27518168	27553774	35607	498,22	0,19	7,10E-01
ENSRNOG00000013816	**Cmas**	cytidine monophosphate N-acetylneur	312826	4	241203361	241221675	18315	3722,47	0,23	7,10E-01
ENSRNOG00000024056		zinc finger protein 715 [Source:MGI Symbol;Acc		1	99693945	99708207	14263	158,58	0,30	7,10E-01
ENSRNOG00000029273	**E2f3**	E2F transcription factor 3 (E2f3), mRN	291105	17	37642240	37716592	74353	304,04	0,23	7,10E-01
ENSRNOG00000002625	**Ptpn4**	protein tyrosine phosphatase, non-rec	246116	13	40797330	40972784	175455	405,85	-0,36	7,11E-01
ENSRNOG00000006410	**Akap5**	A kinase (PRKA) anchor protein 5 (Ak:	171026	6	108768066	108773454	5389	288,60	-0,31	7,11E-01
ENSRNOG00000010642	**Lysmd2**	LysM, putative peptidoglycan-binding,	300839	8	82162170	82177806	15637	568,35	-0,22	7,11E-01
ENSRNOG00000016087	**Hars2**	histidyl-tRNA synthetase 2, mitochond	307491	18	29332602	29341926	9325	845,96	-0,17	7,11E-01
ENSRNOG00000008605		oligosaccharyltransferase 4 homolog (	1E+08	6	36651859	36653055	1197	3194,74	0,29	7,11E-01
ENSRNOG00000009778		similar to RIKEN cDNA 1110001M20 (	1E+08	5	130632416	130653535	21120	4709,96	0,24	7,11E-01
ENSRNOG00000003054	**Cask**	calcium/calmodulin-dependent serine	29647	X	10241012	10272189	31178	251,82	-0,22	7,11E-01
ENSRNOG00000007733	**Arhgef9**	Cdc42 guanine nucleotide exchange f:	66013	X	65173843	65329116	155274	771,77	-0,29	7,11E-01
ENSRNOG00000016188	**Mxd3**	Max dimerization protein 3 (Mxd3), mF	252915	17	11940006	11943732	3727	133,84	0,31	7,11E-01
ENSRNOG00000000113	**Elac1**	elaC ribonuclease Z 1 (Elac1), mRNA	307604	18	68851118	68868465	17348	287,44	0,29	7,11E-01
ENSRNOG00000005136	**Ldlrad3**	Protein Ldlrad3 [Source:UniProtKB/Tr	366138	3	98257809	98497316	239508	127,17	0,27	7,11E-01
ENSRNOG00000007400	**Srebf2**	sterol regulatory element binding trans	300095	7	123364495	123422294	57800	6427,50	0,22	7,11E-01
ENSRNOG00000007638	**Loxl3**	lysyl oxidase-like 3 (Loxl3), mRNA [So	312478	4	178552500	178568813	16314	212,70	-0,31	7,11E-01
ENSRNOG00000007830	**Apold1**	apolipoprotein L domain containing 1 (	444983	4	233025350	233028240	2891	110,01	-0,38	7,11E-01
ENSRNOG00000009224	**E4f1**	E4F transcription factor 1 (E4f1), mRN	681359	10	13632308	13643815	11508	467,74	0,19	7,11E-01
ENSRNOG00000016108	**Phlpp2**	PH domain and leucine rich repeat prc	498949	19	52555245	52621851	66607	904,40	-0,16	7,11E-01
ENSRNOG00000016917	**Clcn1**	chloride channel, voltage-sensitive 1 (	25688	4	136479780	136507216	27437	21,29	-0,37	7,11E-01
ENSRNOG00000023130	**Lyplal1**	lysophospholipase-like 1 (Lyplal1), mF	289357	13	108706787	108738098	31312	264,31	0,23	7,11E-01
ENSRNOG00000027707	**Exd2**	exonuclease 3'-5' domain containing 2	362759	6	112192710	112226140	33431	1668,48	0,19	7,11E-01
ENSRNOG00000001237	**Cabin1**	calcineurin binding protein 1 (Cabin1),	94165	20	16025715	16151225	125511	2367,07	0,13	7,11E-01
ENSRNOG00000005540	**Nin**	ninein (GSK3B interacting protein) (Ni	299117	6	101880766	101975096	94331	97,65	-0,23	7,12E-01
ENSRNOG00000016128	**Lrrc14**	leucine rich repeat containing 14 (Lrrc	500900	7	117761927	117767484	5558	585,47	0,23	7,12E-01
ENSRNOG00000009749	**Wdr45**	WD repeat domain 45 (Wdr45), mRNA	302559	X	16402699	16408333	5635	383,15	0,30	7,12E-01

ENSRNOG00000000611	**Ube2d1**	ubiquitin-conjugating enzyme E2D 1 (Ube2d1), r		20	20738031	20741952	3922	493,42	0,21	7,12E-01
ENSRNOG00000009354	**Nrarp**	Notch-regulated ankyrin repeat protein	499745	3	2377426	2379972	2547	677,22	0,36	7,12E-01
ENSRNOG00000015660	**Pex3**	peroxisomal biogenesis factor 3 (Pex3	83519	1	9313354	9354376	41023	487,74	-0,18	7,12E-01
ENSRNOG00000005703	**Arfgef1**	ADP-ribosylation factor guanine nucle	312915	5	13474693	13567432	92740	1692,11	0,28	7,12E-01
ENSRNOG00000007391	**Hes7**	hairy and enhancer of split 7 (Drosoph	287423	10	55450224	55452747	2524	100,79	0,21	7,12E-01
ENSRNOG00000011133		transient receptor potential cation chai	84494	2	163157119	163282064	124946	362,97	0,24	7,12E-01
ENSRNOG00000017462	**Spata5**	spermatogenesis associated 5 (Spata	361935	2	143758068	143950578	192511	298,67	-0,24	7,12E-01
ENSRNOG00000022702	**Fbxo22**	F-box protein 22 (Fbxo22), mRNA [So	300724	8	58351331	58367359	16029	1788,58	0,23	7,12E-01
ENSRNOG00000023799	**Pak1ip1**	PAK1 interacting protein 1 (Pak1ip1), i	361232	17	23562529	23573599	11071	409,46	0,22	7,12E-01
ENSRNOG00000023906	**Dyx1c1**	dyslexia susceptibility 1 candidate 1 (D	363096	8	73438098	73451286	13189	36,11	-0,32	7,12E-01
ENSRNOG00000025619	**Ap1g2**	Protein Ap1g2 [Source:UniProtKB/TrE	1E+08	15	37646285	37653522	7238	159,58	0,35	7,12E-01
ENSRNOG00000027259	**Faxdc2**	Protein LOC691221 [Source:UniProtK	691221	10	43298906	43308262	9357	120,30	0,22	7,12E-01
ENSRNOG00000028664		glucocorticoid receptor DNA binding factor 1 [So		16	27103740	27104747	1008	234,50	-0,21	7,12E-01
ENSRNOG00000039790				9	16832511	16835052	2542	58,08	-0,32	7,12E-01
ENSRNOG00000043419	**Hist3h2ba**	histone cluster 3, H2ba (Hist3h2ba), m	303175	10	45047288	45047917	630	1382,67	0,27	7,12E-01
ENSRNOG00000047348		family with sequence similarity 155, member A [S		16	85460698	85485992	25295	183,63	-0,25	7,12E-01
ENSRNOG00000037244	**Atp6ap1**	ATPase, H+ transporting, lysosomal a	83615	1	152148120	152155200	7081	9990,28	0,22	7,12E-01
ENSRNOG00000001764	**Vps8**	Protein Vps8 [Source:UniProtKB/TrEM	287990	11	86184488	86390249	205762	1674,20	-0,15	7,12E-01
ENSRNOG00000008470		leucine rich repeat containing 43 [Sou	288751	12	40388951	40405446	16496	21,57	-0,34	7,12E-01
ENSRNOG00000011411		G protein-coupled receptor 126 [Source:MGI Sy		1	10203643	10274931	71289	96,39	0,34	7,12E-01
ENSRNOG00000037765	**Lims1**	LIM and senescent cell antigen-like dc	499443	20	29774111	29824244	50134	1377,31	-0,22	7,12E-01
ENSRNOG00000028344	**Mmp11**	matrix metallopeptidase 11 (Mmp11), i	25481	20	15825614	15834396	8783	196,23	0,32	7,13E-01
ENSRNOG00000004509	**Eapp**	E2F-associated phosphoprotein (Eapp	299043	6	85107699	85129236	21538	387,07	-0,30	7,13E-01
ENSRNOG00000005391	**Prex2**	phosphatidylinositol-3,4,5-trisphospha	312912	5	12451146	12694003	242858	48,80	-0,34	7,13E-01
ENSRNOG00000010535	**Cdh18**	Protein Cdh18 [Source:UniProtKB/TrE	310174	2	94093007	94418627	325621	48,70	0,35	7,13E-01
ENSRNOG00000033654	**LOC50103**	Ab2-060 (LOC501038), mRNA [Sourc	501038	8	102801222	102809973	8752	18,29	-0,36	7,13E-01
ENSRNOG00000011402	**Isg20l2**	interferon stimulated exonuclease gen	361977	2	206705209	206714693	9485	113,17	0,27	7,13E-01
ENSRNOG00000003805	**Rnft1**	Protein Rnft1-ps1 [Source:UniProtKB/	360595	10	76280989	76290422	9434	221,53	-0,26	7,13E-01
ENSRNOG00000016697	**Lrrc56**	leucine rich repeat containing 56 (Lrrc	365389	1	221100369	221114723	14355	237,92	-0,33	7,13E-01
ENSRNOG00000009533	**LOC29552**	RIKEN cDNA 4922501L14 gene [Sour	295528	2	279661644	279694441	32798	73,60	-0,24	7,14E-01
ENSRNOG00000013847	**Nova2**	Protein Nova2 [Source:UniProtKB/TrE	292681	1	81109553	81126706	17154	1307,80	0,18	7,14E-01
ENSRNOG00000032232	**LOC68039**	Protein LOC100911683; RCG56468, i	680394	1	92715996	92716226	231	17,55	0,36	7,14E-01
ENSRNOG00000003538	**Adamts4**	ADAM metallopeptidase with thrombo	66015	13	94249862	94259351	9490	31,54	0,32	7,14E-01
ENSRNOG00000009397	**Kbtbd4**	kelch repeat and BTB (POZ) domain c	311185	3	86437916	86444958	7043	455,41	0,18	7,14E-01
ENSRNOG00000017863		zinc finger E-box binding homeobox 1	25705	17	52352978	52409521	56544	1147,16	-0,31	7,14E-01
ENSRNOG00000022807	**Pced1b**	PC-esterase domain containing 1B (P	315283	7	138330915	138468397	137483	162,17	0,23	7,14E-01
ENSRNOG00000024022	**Ndfip2**	Nedd4 family interacting protein 2 (Nd	361089	15	92899714	92951232	51519	2576,48	0,25	7,14E-01
ENSRNOG00000026243	**Ap2a1**	adaptor-related protein complex 2, alp	308578	1	101961565	101990733	29169	4505,36	-0,22	7,14E-01

ENSRNOG00000010550	**Trappc3**	trafficking protein particle complex 3 (T	362599	5	148049331	148062968	13638	4396,34	0,18	7,14E-01
ENSRNOG00000012438	**Larp6**	La ribonucleoprotein domain family, m	315731	8	65362299	65382759	20461	350,44	-0,20	7,14E-01
ENSRNOG00000014727	**Fahd1**	fumarylacetoacetate hydrolase domair	302980	10	14031088	14031753	666	266,69	0,24	7,14E-01
ENSRNOG00000004776	**Ftsj1**	Protein Ftsj1 [Source:UniProtKB/TrEN	363450	X	15778678	15784018	5341	168,22	0,23	7,15E-01
ENSRNOG00000028241	**Itfg3**	integrin alpha FG-GAP repeat containi	360502	10	15392902	15405995	13094	677,19	0,23	7,15E-01
ENSRNOG00000018353	**Rad17**	RAD17 homolog (S. pombe) (Rad17),	310034	2	49793991	49823799	29809	435,53	0,20	7,15E-01
ENSRNOG00000037658	**Gprasp2**	Protein Gprasp2 [Source:UniProtKB/T	317405	X	106757121	106759631	2511	1876,86	0,22	7,15E-01
ENSRNOG00000011260	**Cmbl**	carboxymethylenebutenolidase homol	310201	2	104317807	104339571	21765	57,74	-0,30	7,15E-01
ENSRNOG00000050869	**Cebpd**	CCAAT/enhancer binding protein (C/E	25695	11	92069789	92070898	1110	353,84	0,36	7,15E-01
ENSRNOG00000027098	**Sez6l2**	seizure related 6 homolog (mouse)-like	308988	1	205363570	205383913	20344	5881,61	0,27	7,15E-01
ENSRNOG00000003261	**Usp9x**	ubiquitin specific peptidase 9, X-linked	363445	X	11310179	11428084	117906	3944,31	-0,37	7,16E-01
ENSRNOG00000008810		NMDA receptor synaptonuclear signal	117536	3	2243540	2252243	8704	9428,02	-0,25	7,16E-01
ENSRNOG00000014511	**Alg10**	ALG10, alpha-1,2-glucosyltransferase	245960	7	131010627	131015654	5028	883,41	-0,24	7,16E-01
ENSRNOG00000001431		Protein Rasa4; RCG21933, isoform C	288589	12	25597617	25624562	26946	28,62	-0,31	7,16E-01
ENSRNOG00000016558	**Pllp**	plasmolipin (Pllp), mRNA [Source:RefS	64364	19	10725892	10746753	20862	91,55	0,34	7,16E-01
ENSRNOG00000015991	**Npr2**	natriuretic peptide receptor B/guanylat	116564	5	63653695	63672094	18400	283,56	-0,32	7,16E-01
ENSRNOG00000019365	**Ablim3**	actin binding LIM protein family, memb	307395	18	56356317	56442746	86430	1023,74	-0,19	7,16E-01
ENSRNOG00000036828	**Pde1b**	phosphodiesterase 1B, calmodulin-dep	29691	7	142898973	142925732	26760	1160,53	-0,22	7,16E-01
ENSRNOG00000015101	**Mak**	male germ cell-associated kinase (Ma	25677	17	23514574	23550277	35704	66,12	-0,26	7,16E-01
ENSRNOG00000001352	**RGD13097**	Protein RGD1309762 [Source:UniPro	304503	12	42581155	42685050	103896	6490,43	0,18	7,16E-01
ENSRNOG00000000869	**Arhgef6**	Rac/Cdc42 guanine nucleotide exchar	363509	X	154351133	154467649	116517	82,68	-0,31	7,16E-01
ENSRNOG00000004912		integrin alpha V [Source:MGI Symbol;Acc:MGI:9		3	77644078	77727811	83734	1202,33	-0,22	7,16E-01
ENSRNOG00000011470	**Tsc1**	tuberous sclerosis 1 (Tsc1), mRNA [So	60445	3	12588006	12615637	27632	1124,69	0,20	7,16E-01
ENSRNOG00000014343		Uncharacterized protein [Source:UniProtKB/TrE		8	23389798	23442950	53153	37,43	0,35	7,16E-01
ENSRNOG00000015603		protein kinase C epsilon type [Source	29340	6	9404742	9883579	478838	2564,38	-0,25	7,16E-01
ENSRNOG00000024475	**Fancd2**	Fanconi anemia, complementation grc	312641	4	208787164	208847877	60714	253,24	0,30	7,16E-01
ENSRNOG00000048682	**Zwint**	ZW10 interactor, kinetochore protein (.	257644	20	19258274	19274198	15925	35892,52	0,25	7,16E-01
ENSRNOG00000007416	**Clp1**	cleavage and polyadenylation factor I	311166	3	78645912	78649612	3701	246,95	0,20	7,16E-01
ENSRNOG00000013912	**Slc30a7**	solute carrier family 30 (zinc transporte	310801	2	236974683	237037073	62391	438,82	0,24	7,16E-01
ENSRNOG00000000891		Uncharacterized protein [Source:UniProtKB/TrE		12	6863267	6867447	4181	64,36	-0,30	7,16E-01
ENSRNOG00000004146	**Coro7**	coronin 7 (Coro7), mRNA [Source:Ref	192276	10	9866591	9909647	43057	1409,07	0,14	7,17E-01
ENSRNOG00000005140	**Prmt8**	protein arginine methyltransferase 8 (F	688502	4	231280525	231360197	79673	880,84	-0,22	7,17E-01
ENSRNOG00000014461	**Galns**	galactosamine (N-acetyl)-6-sulfate sul	292073	19	66100429	66131568	31140	145,82	0,23	7,17E-01
ENSRNOG00000022372	**Kdm1a**	lysine (K)-specific demethylase 1A (Kc	500569	5	158674383	158730522	56140	3240,91	0,15	7,17E-01
ENSRNOG00000015847		Uncharacterized protein [Source:UniProtKB/TrE		3	126960510	126961368	859	885,05	0,33	7,17E-01
ENSRNOG00000021828	**LOC10091(**	coronin, actin-binding protein, 1B (Cor	29474	1	226268590	226273903	5314	3358,75	0,23	7,17E-01
ENSRNOG00000009289	**Galk2**	galactokinase 2 (Galk2), mRNA [Sourc	296117	3	124669033	124793301	124269	477,18	0,28	7,17E-01
ENSRNOG00000019165	**Mrpl51**	mitochondrial ribosomal protein L51 (M	297601	4	224722975	224725933	2959	1131,39	0,22	7,17E-01

ENSRNOG00000024535		zinc finger, ZZ-type with EF hand dom	287476	10	59099083	59235227	136145	1665,37	0,21	7,17E-01
ENSRNOG00000039315	**LOC49978**	RIKEN cDNA 1700019L03 gene [Sour	499781	3	17150546	17157519	6974	84,30	-0,26	7,17E-01
ENSRNOG00000039457		Uncharacterized protein [Source:UniProtKB/TrE		18	30574247	30576643	2397	483,11	-0,19	7,17E-01
ENSRNOG00000000558	**Ppa1**	pyrophosphatase (inorganic) 1 (Ppa1)	294504	20	33048101	33089923	41823	1893,80	0,19	7,17E-01
ENSRNOG00000005074	**Arl6ip6**	ADP-ribosylation-like factor 6 interacti	499798	3	43839905	43867541	27637	585,82	-0,22	7,17E-01
ENSRNOG00000011527	**Bag5**	BCL2-associated athanogene 5 (Bag5	366734	6	144231051	144234393	3343	679,62	0,16	7,17E-01
ENSRNOG00000018515	**Det1**	de-etiolated homolog 1 (Arabidopsis) (	308775	1	141498061	141511032	12972	139,12	0,24	7,17E-01
ENSRNOG00000020185	**Wdr6**	WD repeat domain 6 (Wdr6), mRNA [S	301007	8	116702848	116709264	6417	15379,62	0,21	7,17E-01
ENSRNOG00000046023		exocyst complex component 4 [Sourc	116654	4	60287465	61081401	793937	1094,13	-0,25	7,17E-01
ENSRNOG00000048174	**Uqcrq**	ubiquinol-cytochrome c reductase, co	497902	10	38562435	38564716	2282	4614,88	0,32	7,17E-01
ENSRNOG00000050069		Protein LOC100909510; tRNA splicing	292534	1	63084821	63091558	6738	20,40	0,33	7,17E-01
ENSRNOG00000006419	**Aven**	apoptosis, caspase activation inhibitor	311299	3	110635712	110709919	74208	145,96	0,19	7,17E-01
ENSRNOG00000007292	**Spdl1**	spindle apparatus coiled-coil protein 1	303037	10	19504873	19529472	24600	70,41	0,33	7,17E-01
ENSRNOG00000009821	**Slitrk3**	SLIT and NTRK-like family, member 3	310519	2	189795359	189803411	8053	481,42	0,27	7,17E-01
ENSRNOG00000014347	**Slc4a2**	solute carrier family 4 (anion exchange	24780	4	7276047	7292587	16541	1465,83	0,21	7,17E-01
ENSRNOG00000014946	**Thumpd1**	THUMP domain containing 1 (Thumpd	309041	1	196394580	197295841	901262	834,56	0,25	7,17E-01
ENSRNOG00000020383	**Dapk3**	death-associated protein kinase 3 (Da	64391	7	11559843	11568211	8369	1193,73	0,23	7,17E-01
ENSRNOG00000033623	**Pigx**	phosphatidylinositol glycan anchor bio	288041	11	75013594	75031091	17498	1745,78	0,27	7,17E-01
ENSRNOG00000042503	**Ndufv2**	NADH dehydrogenase (ubiquinone) fla	81728	9	113404127	113424677	20551	3998,49	0,26	7,17E-01
ENSRNOG00000004310	**Caskin2**	cask-interacting protein 2 (Caskin2), m	303678	10	103927770	103941924	14155	755,56	-0,32	7,17E-01
ENSRNOG00000029339	**LOC10036**	NADH dehydrogenase (Ubiquinone) F	362588	2	215419281	215419757	477	563,28	0,23	7,18E-01
ENSRNOG00000020150	**Il18bp**	interleukin 18 binding protein (Il18bp),	84388	1	173280487	173281966	1480	70,25	-0,34	7,18E-01
ENSRNOG00000001223	**Pttg1ip**	pituitary tumor-transforming 1 interacti	365548	20	13912729	13930028	17300	3421,34	0,22	7,18E-01
ENSRNOG00000013780	**Nf1**	neurofibromin 1 (Nf1), mRNA [Source:	24592	10	64719521	64958999	239479	2064,43	-0,21	7,18E-01
ENSRNOG00000013802	**Slc25a36**	Protein Slc25a36 [Source:UniProtKB/	364991	8	104412321	104441922	29602	430,53	-0,30	7,18E-01
ENSRNOG00000014089	**Map3k2**	mitogen activated protein kinase kinas	171492	18	24676280	24740723	64444	193,82	-0,21	7,18E-01
ENSRNOG00000028097	**Slc7a14**	solute carrier family 7, member 14 (Slc	499587	2	135373151	135484821	111671	201,23	0,31	7,18E-01
ENSRNOG00000000922	**Sumf2**	sulfatase modifying factor 2 (Sumf2), r	360800	12	32407454	32432604	25151	410,56	0,23	7,18E-01
ENSRNOG00000001598	**Usp16**	ubiquitin specific peptidase 16 (Usp16	288306	11	30726675	30755789	29115	266,52	-0,31	7,18E-01
ENSRNOG00000002496	**Stxbp5l**	syntaxin binding protein 5-like (Stxbp5	288080	11	69337665	69661178	323514	162,66	-0,34	7,18E-01
ENSRNOG00000003206	**Actr3**	ARP3 actin-related protein 3 homolog	81732	13	46808188	46850658	42471	6486,18	-0,17	7,18E-01
ENSRNOG00000005147	**Ccdc157**	Protein Ccdc157 [Source:UniProtKB/	681091	14	85058453	85066554	8102	314,32	0,18	7,18E-01
ENSRNOG00000005478	**Fkbp9**	FK506 binding protein 9 (Fkbp9), mRN	297123	4	151819739	151885185	65447	1482,87	0,24	7,18E-01
ENSRNOG00000007705	**Kcnj10**	potassium inwardly-rectifying channel,	29718	13	95245046	95274535	29490	1076,29	0,21	7,18E-01
ENSRNOG00000009548	**Fpgt**	fucose-1-phosphate guanylyltransfera	310935	2	280067673	280074692	7020	70,79	-0,31	7,18E-01
ENSRNOG00000010750	**Twistnb**	TWIST neighbor (Twistnb), mRNA [So	362728	6	62372118	62389586	17469	286,87	-0,24	7,18E-01
ENSRNOG00000011014	**Copg2**	coatomer protein complex, subunit gamma 2 (Co		4	57822384	57951143	128760	1862,80	-0,22	7,18E-01
ENSRNOG00000017689	**Itih3**	inter-alpha trypsin inhibitor, heavy cha	50693	16	6918684	6933186	14503	411,46	0,36	7,18E-01

ENSRNOG00000022283	**Lhfpl5**	lipoma HMGIC fusion partner-like 5 (L	294303	20	7851367	7861665	10299	52,05	-0,30	7,18E-01
ENSRNOG00000025184	**Prss35**	protease, serine, 35 (Prss35), mRNA [	315866	8	93934402	93951097	16696	21,81	-0,37	7,18E-01
ENSRNOG00000026488	**Gltpd2**	glycolipid transfer protein domain cont	497943	10	56883712	56885374	1663	30,48	0,32	7,18E-01
ENSRNOG00000026616	**Ndufb2**	NADH dehydrogenase (ubiquinone) 1	362344	4	67185340	67192418	7079	4393,78	0,32	7,18E-01
ENSRNOG00000042502	**Smim17**	small integral membrane protein 17 (S	499067	1	72510939	72528650	17712	163,44	-0,29	7,18E-01
ENSRNOG00000042814	**Henmt1**	HEN1 methyltransferase homolog 1 (A	1E+08	2	231223367	231225173	1807	20,85	-0,32	7,18E-01
ENSRNOG00000049419	**Mcart1l**	mitochondrial carrier triple repeat 1-lik	679135	X	107500509	107502676	2168	223,89	-0,26	7,18E-01
ENSRNOG00000028063	**Tmem38b**	transmembrane protein 38B (Tmem38	362521	5	74816786	74852684	35899	375,81	-0,23	7,18E-01
ENSRNOG00000002454	**Rnf2**	ring finger protein 2 (Rnf2), mRNA [So	304850	13	73792852	73821013	28162	1242,29	-0,20	7,18E-01
ENSRNOG00000004289		lysine (K)-specific demethylase 6A [Sc	1E+08	X	5577376	5731431	154056	646,21	-0,26	7,18E-01
ENSRNOG00000023497	**Foxe1**	forkhead box E1 (thyroid transcription	192274	5	66467889	66470697	2809	52,01	0,32	7,18E-01
ENSRNOG00000014459	**Phax**	phosphorylated adaptor for RNA expo	286917	18	50857303	50873989	16687	1272,66	0,23	7,18E-01
ENSRNOG00000016203	**Efna2**	ephrin A2 (Efna2), mRNA [Source:Ref	84358	7	12552532	12563371	10840	360,16	0,20	7,18E-01
ENSRNOG00000037340	**Epha10**	EPH receptor A10 (Epha10), mRNA [S	298528	5	146646393	146678719	32327	121,69	-0,22	7,18E-01
ENSRNOG00000000799	**Abcf1**	ATP-binding cassette, subfamily F (GC	85493	20	5407475	5420356	12882	3568,08	0,18	7,19E-01
ENSRNOG00000003088	**Arhgap31**	Rho GTPase activating protein 31 (Arl	288093	11	67395537	67508274	112738	619,19	-0,35	7,19E-01
ENSRNOG00000005042	**Ranbp17**	RAN binding protein 17 (Ranbp17), m	303029	10	18024416	18328821	304406	101,98	-0,26	7,19E-01
ENSRNOG00000006913		RPA interacting protein (Rpain), mRNA	287463	10	57351862	57358959	7098	273,57	0,17	7,19E-01
ENSRNOG00000007485	**Arfgef2**	ADP-ribosylation factor guanine nucle	296380	3	169733008	169814500	81493	1422,38	0,22	7,19E-01
ENSRNOG00000008308		Histone deacetylase 7 [Source:UniPro	84582	7	139472968	139490046	17079	438,56	0,19	7,19E-01
ENSRNOG00000017144	**Mea1**	male-enhanced antigen 1 (Mea1), mR	685131	9	15516930	15518751	1822	4714,10	0,26	7,19E-01
ENSRNOG00000019450	**Etf1**	eukaryotic translation termination facto	307503	18	27409450	27436622	27173	1550,29	0,22	7,19E-01
ENSRNOG00000020464	**Mrpl54**	mitochondrial ribosomal protein L54 (M	299628	7	11488465	11490985	2521	1226,19	0,33	7,19E-01
ENSRNOG00000024957	**Dnali1**	dynein, axonemal, light intermediate c	298524	5	146821951	146830810	8860	97,93	-0,36	7,19E-01
ENSRNOG00000034177	**Efna5**	ephrin A5 (Efna5), mRNA [Source:Ref	116683	9	109627452	109897812	270361	67,61	-0,24	7,19E-01
ENSRNOG00000042118		RIKEN cDNA 2410089E03 gene [Sour	310137	2	77284221	77358709	74489	1063,84	-0,16	7,19E-01
ENSRNOG00000005836	**Fam49a**	family with sequence similarity 49, me	298890	6	50682248	50788037	105790	1281,88	-0,32	7,19E-01
ENSRNOG00000005515	**Rhbdl3**	rhomboid, veinlet-like 3 (Drosophila) (I	287556	10	67333796	67387701	53906	646,99	-0,29	7,19E-01
ENSRNOG00000022288	**Pafah2**	platelet-activating factor acetylhydrola	313611	5	156397032	156417572	20541	90,49	0,26	7,19E-01
ENSRNOG00000005279	**Ndufaf7**	NADH dehydrogenase (ubiquinone) co	298748	6	1524703	1535937	11235	438,29	0,19	7,20E-01
ENSRNOG00000013011	**Dnajb4**	DnaJ (Hsp40) homolog, subfamily B, r	295549	2	276072494	276101989	29496	982,24	0,29	7,20E-01
ENSRNOG00000014440	**Josd1**	Josephin domain containing 1 (Josd1)	315134	7	120895716	120910124	14409	1939,96	0,27	7,20E-01
ENSRNOG00000014589	**RGD13110**	uncharacterized protein LOC308765	308765	1	142624852	142632040	7189	55,69	-0,32	7,20E-01
ENSRNOG00000015625	**MGC11620**	Uncharacterized protein KIAA0895-like	688736	19	48103245	48110939	7695	3323,04	-0,26	7,20E-01
ENSRNOG00000015969	**Rpf1**	ribosome production factor 1 homolog	499725	2	270895282	270909803	14522	227,87	-0,31	7,20E-01
ENSRNOG00000022273	**Rfk**	riboflavin kinase (Rfk), mRNA [Source	311241	1	243798720	243806275	7556	515,13	0,27	7,20E-01
ENSRNOG00000028436	**Rprml**	reprimo-like (Rprml), mRNA [Source:R	685826	10	91476987	91477991	1005	483,67	-0,36	7,20E-01
ENSRNOG00000042072	**Arhgdig**	Rho GDP dissociation inhibitor (GDI) g	360500	10	15379685	15381848	2164	2047,88	-0,34	7,20E-01

ENSRNOG00000049235	**Arl16**	Protein LOC688311; RCG33173, isofc	688311	10	109230659	109232421	1763	996,95	0,21	7,20E-01
ENSRNOG00000004603	**Srgap1**	SLIT-ROBO Rho GTPase activating pı	314903	7	64196669	64472697	276029	1275,76	-0,17	7,20E-01
ENSRNOG00000019556	**Cd9**	CD9 molecule (Cd9), mRNA [Source:F	24936	4	224989642	225022649	33008	1301,62	-0,35	7,20E-01
ENSRNOG00000001272	**Mcm3ap**	minichromosome maintenance comple	294339	20	15036956	15075014	38059	2167,04	0,19	7,20E-01
ENSRNOG00000000955	**Lnx2**	ligand of numb-protein X 2 (Lnx2), mR	360761	12	11840785	11904808	64024	392,15	-0,19	7,20E-01
ENSRNOG00000003116	**Dph1**	DPH1 homolog (S. cerevisiae) (Dph1)	287523	10	61735049	61745330	10282	552,18	0,15	7,20E-01
ENSRNOG00000005151	**Dync2li1**	dynein cytoplasmic 2 light intermediate	298767	6	7837943	7869291	31349	390,92	0,20	7,20E-01
ENSRNOG00000014293	**Nkd1**	naked cuticle homolog 1 (Drosophila)	364952	19	30466493	30539435	72943	168,42	-0,28	7,20E-01
ENSRNOG00000018042		39S ribosomal protein L48, mitochond	293149	1	171765421	171807819	42399	1010,83	0,21	7,20E-01
ENSRNOG00000018231	**Nacc2**	NACC family member 2, BEN and BTI	296583	3	8870073	8903682	33610	4054,90	-0,25	7,20E-01
ENSRNOG00000019474	**Rsph9**	radial spoke head 9 homolog (Chlamy	316238	9	16120949	16140296	19348	517,13	-0,28	7,20E-01
ENSRNOG00000019832	**Bbs1**	Bardet-Biedl syndrome 1 (Bbs1), mRN	309156	1	227078590	227096525	17936	1157,32	-0,21	7,20E-01
ENSRNOG00000024563	**Ganc**	glucosidase, alpha; neutral C (Ganc),	24382	3	118721646	118773762	52117	273,86	-0,22	7,20E-01
ENSRNOG00000026044	**Prrg1**	proline rich Gla (G-carboxyglutamic ac	363472	X	45922647	46028688	106042	91,91	-0,32	7,20E-01
ENSRNOG00000022570		pseudouridylate synthase 7 homolog (	315264	7	135424411	135458044	33634	130,42	0,24	7,21E-01
ENSRNOG00000015523	**RGD13081**	similar to expressed sequence AW209	307008	17	50780397	50787033	6637	142,12	-0,25	7,21E-01
ENSRNOG00000030445	**Ormdl3**	ORM1-like 3 (S. cerevisiae) (Ormdl3),	360618	10	86343415	86350554	7140	1930,93	0,20	7,21E-01
ENSRNOG00000001724	**Atp13a3**	ATPase type 13A3 (Atp13a3), mRNA [	678704	11	76998192	77025060	26869	32,67	-0,31	7,21E-01
ENSRNOG00000000121	**Pigv**	phosphatidylinositol glycan anchor bio	366478	5	155574860	155586747	11888	308,10	0,23	7,21E-01
ENSRNOG00000026978	**Oscp1**	organic solute carrier partner 1 (Oscp1	362595	5	147837067	147867531	30465	511,62	0,18	7,21E-01
ENSRNOG00000001069	**Elavl1**	ELAV (embryonic lethal, abnormal visi	363854	12	4614833	4655379	40547	1136,95	-0,15	7,21E-01
ENSRNOG00000019075	**Stat5b**	signal transducer and activator of tran	25126	10	88480377	88506891	26515	449,43	0,21	7,21E-01
ENSRNOG00000003861	**Gorab**	golgin, RAB6-interacting (Gorab), mRI	304923	13	86562603	86579222	16620	288,56	-0,25	7,22E-01
ENSRNOG00000011155	**RGD1559519**			10	105227450	105228077	628	20,56	0,34	7,22E-01
ENSRNOG00000022356	**Zc2hc1a**	zinc finger, C2HC-type containing 1A (	310244	2	116224313	116261506	37194	1979,54	0,19	7,22E-01
ENSRNOG00000042776	**Lins**	Protein Lins [Source:UniProtKB/TrEM	308704	1	128695038	128700627	5590	245,55	0,23	7,22E-01
ENSRNOG00000010984	**Anxa11**	annexin A11 (Anxa11), mRNA [Source	290527	16	3833120	3877953	44834	599,54	0,24	7,22E-01
ENSRNOG00000012791	**Ajuba**	ajuba LIM protein (Ajuba), mRNA [Sou	85265	15	37092305	37102545	10241	305,52	0,24	7,22E-01
ENSRNOG00000013265	**Tgfbr2**	transforming growth factor, beta recep	81810	8	123585765	123671209	85445	52,93	-0,34	7,22E-01
ENSRNOG00000020239	**Capn15**	calpain 15 (Capn15), mRNA [Source:F	303000	10	15132749	15159349	26601	729,96	0,21	7,22E-01
ENSRNOG00000020381		POU domain, class 2, transcription fac	117058	1	83226783	83263163	36381	58,30	-0,26	7,22E-01
ENSRNOG00000030888	**Rela**	v-rel avian reticuloendotheliosis viral o	309165	1	227928092	227938571	10480	926,07	0,17	7,22E-01
ENSRNOG00000049655		RIKEN cDNA 1500009L16 gene [Sour	1E+08	7	25878012	25878803	792	22,74	-0,33	7,22E-01
ENSRNOG00000015925	**Zfp131**	zinc finger protein 131 (Zfp131), mRN	310375	2	70868110	70894880	26771	535,84	-0,29	7,22E-01
ENSRNOG00000015997	**Ccdc106**	Protein Ccdc106 [Source:UniProtKB/	499071	1	76201662	76204586	2925	718,29	0,16	7,22E-01
ENSRNOG00000015340	**Dnajc25**	DnaJ (Hsp40) homolog, subfamily C, ı	362526	5	80295287	80315200	19914	328,66	0,20	7,22E-01
ENSRNOG00000008372	**Vamp7**	vesicle-associated membrane protein	85491	12	20886479	21089729	203251	649,71	0,25	7,22E-01
ENSRNOG00000009972	**Rara**	retinoic acid receptor, alpha (Rara), mı	24705	10	86663849	86681847	17999	594,99	-0,28	7,22E-01

ENSRNOG00000028219	**Tmem209**	transmembrane protein 209 (Tmem20	312200	4	57558247	57585192	26946	413,21	0,17	7,22E-01
ENSRNOG00000032561		Solute carrier family 52, riboflavin tran	362942	7	117592708	117598130	5423	270,18	0,18	7,22E-01
ENSRNOG00000000306	**Smpd2**	sphingomyelin phosphodiesterase 2, r	83537	20	47904392	47907503	3112	285,21	0,22	7,22E-01
ENSRNOG00000007043	**RGD13050**	similar to 1110008L16Rik protein (RGI	299050	6	85615614	85708813	93200	285,54	0,22	7,22E-01
ENSRNOG00000007371	**Rbmx2**	RNA binding motif protein, X-linked 2 (	367930	X	135621454	135628587	7134	131,45	-0,27	7,22E-01
ENSRNOG00000007814	**Kdm3a**	lysine (K)-specific demethylase 3A (Kc	312440	4	164282438	164325750	43313	1099,51	-0,22	7,22E-01
ENSRNOG00000007874	**Tamm41**	TAM41, mitochondrial translocator ass	362419	4	210211579	210243653	32075	565,51	0,19	7,22E-01
ENSRNOG00000010911	**Tmem108**	Protein Tmem108 [Source:UniProtKB	300967	8	111361212	111485621	124410	642,85	-0,24	7,22E-01
ENSRNOG00000013668	**Capg**	capping protein (actin filament), gelsol	297339	4	165178009	165189797	11789	127,43	-0,36	7,22E-01
ENSRNOG00000017832	**Snx2**	sorting nexin 2 (Snx2), mRNA [Source	291464	18	47347481	47389036	41556	700,63	-0,35	7,22E-01
ENSRNOG00000000111	**Coprs**	coordinator of PRMT5, differentiation s	290925	16	80390902	80396012	5111	873,68	0,19	7,22E-01
ENSRNOG00000007503	**Adam17**	ADAM metallopeptidase domain 17 (A	57027	6	60268237	60315989	47753	514,28	-0,26	7,22E-01
ENSRNOG00000010434	**Dync1li1**	dynein cytoplasmic 1 light intermediate	252902	8	122055379	122088910	33532	2670,02	0,25	7,22E-01
ENSRNOG00000010650	**Plekhh1**	pleckstrin homology domain containing	314262	6	111585028	111633992	48965	174,77	-0,20	7,22E-01
ENSRNOG00000014453	**Anxa5**	annexin A5 (Anxa5), mRNA [Source:R	25673	2	142777627	142808368	30742	1374,46	0,28	7,22E-01
ENSRNOG00000018986	**Fbxl19**	F-box and leucine-rich repeat protein 1	308999	1	206219421	206238674	19254	3478,97	0,22	7,22E-01
ENSRNOG00000043503	**Ehd1**	EH-domain containing 1 (Ehd1), mRN	293692	1	228632629	228655001	22373	4719,58	0,22	7,22E-01
ENSRNOG00000002358	**Scpep1**	serine carboxypeptidase 1 (Scpep1), r	114861	10	73846394	73880404	34011	939,72	-0,30	7,23E-01
ENSRNOG00000019299	**Edem2**	ER degradation enhancer, mannosida	296304	3	157597581	157623398	25818	482,74	-0,23	7,23E-01
ENSRNOG00000000986	**Camsap3**	calmodulin regulated spectrin-associat	689074	12	4296404	4319735	23332	2241,25	0,20	7,23E-01
ENSRNOG00000011863	**Gins3**	GINS complex subunit 3 (Psf3 homolc	307639	19	9820191	9828727	8537	190,99	0,26	7,23E-01
ENSRNOG00000018536		phosphoenolpyruvate carboxykinase 2 (mitochoi		15	38106930	38114388	7459	149,04	-0,33	7,23E-01
ENSRNOG00000015077	**Acsf3**	Protein Acsf3; Similar to C50H11.1, isc	498962	19	66307158	66342053	34896	571,85	0,18	7,23E-01
ENSRNOG00000016258	**Zfp516**	zinc finger protein 516 (Zfp516), mRN	291406	18	78879100	78919207	40108	572,64	0,21	7,23E-01
ENSRNOG00000030871	**Calm2**	calmodulin 2 (Calm2), mRNA [Source:RefSeq m		15	69146646	69147756	1111	10092,25	-0,22	7,23E-01
ENSRNOG00000038200	**Rttn**	rotatin (Rttn), mRNA [Source:RefSeq r	291377	18	85154622	85286009	131388	135,98	0,27	7,23E-01
ENSRNOG00000049077				10	12981495	12981573	79	22,00	-0,31	7,23E-01
ENSRNOG00000001911	**Map6d1**	MAP6 domain containing 1 (Map6d1),	363823	11	87385908	87392137	6230	225,59	0,24	7,23E-01
ENSRNOG00000046852	**B3gat2**	beta-1,3-glucuronyltransferase 2 (gluc	64544	9	28816204	28899282	83079	736,08	-0,23	7,23E-01
ENSRNOG00000030591	**Arf1**	ADP-ribosylation factor 1 (Arf1), mRN	64310	10	45318171	45334628	16458	12557,95	0,17	7,23E-01
ENSRNOG00000003928	**Tmco1**	transmembrane and coiled-coil domai	289196	13	90109017	90131813	22797	376,68	0,24	7,24E-01
ENSRNOG00000019223		ATP synthase, H+ transporting, mitoch	116550	17	73895059	73944594	49536	5941,56	0,16	7,24E-01
ENSRNOG00000015641	**Exoc3l1**	exocyst complex component 3-like 1 (	291961	19	48110992	48116641	5650	83,50	-0,27	7,24E-01
ENSRNOG00000016284	**Dmrt3**	doublesex and mab-3 related transcrip	293976	1	250848350	250861669	13320	36,55	-0,28	7,24E-01
ENSRNOG00000019760	**Oxnad1**	oxidoreductase NAD-binding domain c	306270	16	8124248	8153317	29070	450,32	0,25	7,24E-01
ENSRNOG00000031216	**Zfp347**	zinc finger protein 347 (Zfp347), mRN	170902	7	10136669	10138411	1743	44,46	-0,32	7,25E-01
ENSRNOG00000037221	**Tipinl1**	timeless interacting protein (Tipin), mF	363076	5	173082	173912	831	86,64	-0,26	7,25E-01
ENSRNOG00000042449	**LOC10036**	CDC42 small effector 2 (Cdc42se2), n	691031	10	39827148	39895658	68511	2111,35	0,22	7,25E-01

ENSRNOG00000000024		heme-binding protein 1 [Source:RefS	362454	4	233174078	233203299	29222	710,20	-0,22	7,25E-01
ENSRNOG00000008994	**Arpc4**	actin related protein 2/3 complex, subu	297518	4	208627502	208638029	10528	4643,85	0,20	7,25E-01
ENSRNOG00000010825	**Snapc3**	small nuclear RNA activating complex	362537	5	105539242	105565938	26697	522,23	0,16	7,25E-01
ENSRNOG00000010951	**Cmtm6**	CKLF-like MARVEL transmembrane d	316035	8	122101557	122119645	18089	684,35	0,23	7,25E-01
ENSRNOG00000017443	**Tmem87b**	transmembrane protein 87B (Tmem87	362212	3	128621323	128654288	32966	393,37	-0,30	7,25E-01
ENSRNOG00000017505	**Npepo**	aminopeptidase O (Npepo), mRNA [S	290963	17	550168	814393	264226	83,08	-0,25	7,25E-01
ENSRNOG00000017843	**Polr3k**	polymerase (RNA) III (DNA directed) p	366277	3	181083089	181087319	4231	1911,28	0,27	7,25E-01
ENSRNOG00000018239	**Dhrs4**	dehydrogenase/reductase (SDR famil	266686	15	38045928	38057498	11571	126,63	-0,31	7,25E-01
ENSRNOG00000019236		Protein LOC500532 [Source:UniProtK	500532	5	139923825	140134648	210824	1596,82	0,21	7,25E-01
ENSRNOG00000038744	**Atp5h**	ATP synthase, H+ transporting, mitoch	306478	16	51540712	51541307	596	201,70	0,29	7,25E-01
ENSRNOG00000002711	**Nuf2**	NUF2, NDC80 kinetochore complex c	304951	13	92450022	92478789	28768	166,22	0,33	7,25E-01
ENSRNOG00000018322		phosphatidylinositol binding clathrin assembly pr		1	160681971	160752079	70109	2728,84	-0,20	7,25E-01
ENSRNOG00000004412	**Mtbp**	Mdm2, transformed 3T3 cell double m	500870	7	95931981	96009703	77723	159,33	0,26	7,25E-01
ENSRNOG00000017669		chromodomain helicase DNA binding	310707	2	219196513	219249842	53330	156,58	0,24	7,25E-01
ENSRNOG00000018048	**Dctn5**	dynactin 5 (p25) (Dctn5), mRNA [Sour	308961	1	199151318	199167998	16681	851,89	0,23	7,25E-01
ENSRNOG00000016472	**March4**	Protein March4 [Source:UniProtKB/Tr	367295	9	79555877	79670952	115076	750,16	-0,24	7,26E-01
ENSRNOG00000018404	**Aars**	alanyl-tRNA synthetase (Aars), mRNA	292023	19	54018027	54040573	22547	8498,55	0,13	7,26E-01
ENSRNOG00000049854		Uncharacterized protein [Source:UniProtKB/TrE		12	42689034	42694806	5773	110,41	0,22	7,26E-01
ENSRNOG00000029465	**Slc26a10**	solute carrier family 26, member 10 (S	366909	7	70636384	70645045	8662	49,82	0,31	7,26E-01
ENSRNOG00000000821	**Tubb5**	tubulin, beta 5 class I (Tubb5), mRNA	29214	20	5519551	5523476	3926	53800,30	0,23	7,26E-01
ENSRNOG00000001519	**Dlx2**	distal-less homeobox 2 (Dlx2), mRNA	296499	3	64665449	64667332	1884	673,15	0,19	7,26E-01
ENSRNOG00000007690	**Cmpk2**	cytidine monophosphate (UMP-CMP)	314004	6	54403782	54414584	10803	493,57	0,24	7,26E-01
ENSRNOG00000013286	**Pdcl3**	phosducin-like 3 (Pdcl3), mRNA [Sour	316348	9	45362541	45371009	8469	1137,33	0,26	7,26E-01
ENSRNOG00000015113	**Mocos**	molybdenum cofactor sulfurase (Mocc	361300	18	16349169	16395056	45888	33,20	0,30	7,26E-01
ENSRNOG00000015865	**Ap2s1**	adaptor-related protein complex 2, sig	65046	1	79918162	79929502	11341	6640,20	0,23	7,26E-01
ENSRNOG00000016221	**Scn2b**	sodium channel, voltage-gated, type II	25349	8	48045393	48053999	8607	384,20	0,29	7,26E-01
ENSRNOG00000020860	**Tdrkh**	tudor and KH domain containing (Tdrk	310652	2	215130624	215152162	21539	926,94	0,22	7,26E-01
ENSRNOG00000039265	**Psmg4**	proteasome (prosome, macropain) as	689623	17	33299002	33306344	7343	748,03	0,22	7,26E-01
ENSRNOG00000047171	**Rnf8**	ring finger protein 8, E3 ubiquitin prote	361815	20	10484895	10509126	24232	658,71	0,23	7,26E-01
ENSRNOG00000015195	**Hdhd3**	haloacid dehalogenase-like hydrolase	688746	5	82477554	82481021	3468	113,11	-0,26	7,26E-01
ENSRNOG00000009075	**Trim13**	tripartite motif-containing 13 (Trim13),	364398	15	45727569	45735914	8346	395,27	-0,20	7,26E-01
ENSRNOG00000003967	**Vmp1**	vacuole membrane protein 1 (Vmp1),	192129	10	76098589	76196545	97957	1543,83	0,20	7,26E-01
ENSRNOG00000005886	**Zscan20**	Protein Zscan20 [Source:UniProtKB/T	500549	5	150501908	150520675	18768	165,39	0,19	7,26E-01
ENSRNOG00000038951	**Cox17**	COX17 cytochrome c oxidase copper	89786	11	67113608	67119379	5772	494,40	0,20	7,26E-01
ENSRNOG00000001427	**Orai2**	ORAI calcium release-activated calciu	304592	12	25544833	25562556	17724	554,94	0,29	7,26E-01
ENSRNOG00000046338		RIKEN cDNA C030039L03 gene [Sou	1E+08	1	85760797	85774094	13298	61,99	-0,29	7,27E-01
ENSRNOG00000009244	**Abhd4**	abhydrolase domain containing 4 (Abh	364380	15	36705478	36717401	11924	12418,82	-0,29	7,27E-01
ENSRNOG00000020229	**Homer3**	homer homolog 3 (Drosophila) (Home	29548	16	20733291	20740680	7390	992,47	-0,27	7,27E-01

ENSRNOG00000025528	**Efr3a**	EFR3 homolog A (S. cerevisiae) (Efr3a	362923	7	106483576	106564947	81372	1439,28	-0,23	7,27E-01
ENSRNOG00000003041	**Smg6**	SMG6 nonsense mediated mRNA dec	287522	10	61493274	61555359	62086	52,58	0,26	7,27E-01
ENSRNOG00000011728	**Fto**	fat mass and obesity associated (Fto),	291905	19	27840262	28182203	341942	4460,50	0,15	7,27E-01
ENSRNOG00000012644	**Nupl1**	nucleoporin like 1 (Nupl1), mRNA [Sou	245922	15	44324078	44357949	33872	1166,98	-0,20	7,27E-01
ENSRNOG00000016706	**Fanca**	Fanconi anemia, complementation grc	361435	19	66772420	66822658	50239	198,59	0,22	7,27E-01
ENSRNOG00000043204	**Harbi1**	harbinger transposase derived 1 (Harb	690164	3	87314056	87321871	7816	238,50	0,19	7,27E-01
ENSRNOG00000008390	**Dcun1d5**	DCN1, defective in cullin neddylation 1	315405	8	5444079	5465187	21109	1717,98	0,22	7,27E-01
ENSRNOG00000001189	**Sik1**	salt-inducible kinase 1 (Sik1), mRNA [	59329	20	12845510	12855040	9531	297,62	0,17	7,27E-01
ENSRNOG00000007370	**Rnf144a**	ring finger protein 144A (Rnf144a), mR	500636	6	54234290	54348903	114614	704,82	-0,18	7,27E-01
ENSRNOG00000014087	**Kifc3**	kinesin family member C3 (Kifc3), mR	307644	19	10300709	10319285	18577	1883,65	-0,29	7,27E-01
ENSRNOG00000017243	**Bnip3**	BCL2/adenovirus E1B interacting prot	84480	1	218174697	218191883	17187	1089,75	-0,17	7,27E-01
ENSRNOG00000025689	**Abhd1**	abhydrolase domain containing 1 (Abh	313917	6	36603422	36608410	4989	305,66	-0,30	7,27E-01
ENSRNOG00000027228	**Mogat2**	monoacylglycerol O-acyltransferase 2	681211	1	170384257	170407542	23286	36,10	0,29	7,27E-01
ENSRNOG00000002036	**Paxbp1**	Protein Gcfc1 [Source:UniProtKB/TrE	681004	11	34796266	34824580	28315	729,04	-0,28	7,27E-01
ENSRNOG00000004149	**Mgat4c**	mannosyl (alpha-1,3-)-glycoprotein be	299756	7	43829446	44052611	223166	37,60	-0,30	7,27E-01
ENSRNOG00000009906	**Slfnl1**	schlafen-like 1 (Slfnl1), mRNA [Source	500540	5	143251801	143256272	4472	44,56	-0,29	7,27E-01
ENSRNOG00000009955	**Plch1**	phospholipase C, eta 1 (Plch1), mRNA	310463	2	173641410	173802758	161349	237,56	-0,28	7,27E-01
ENSRNOG00000018903	**Pik3r1**	phosphoinositide-3-kinase, regulatory	25513	2	50895264	50965217	69954	1509,86	-0,29	7,27E-01
ENSRNOG00000050224	**Stambpl1**	STAM binding protein-like 1 (Stambpl1	687696	1	259721133	259759242	38110	300,82	-0,24	7,27E-01
ENSRNOG00000000540	**Btbd9**	BTB (POZ) domain containing 9 (Btbd	294318	20	11090808	11437886	347079	922,08	0,20	7,27E-01
ENSRNOG00000003599	**Utp3**	UTP3, small subunit (SSU) processom	305258	14	21063887	21065299	1413	559,98	0,22	7,27E-01
ENSRNOG00000004022	**Klhdc9**	kelch domain containing 9 (Klhdc9), m	360878	13	94370325	94373418	3094	45,50	0,27	7,27E-01
ENSRNOG00000004572	**Zfp472**	zinc finger protein 472 (Zfp472), mRN	314587	7	15406064	15409287	3224	57,77	-0,36	7,27E-01
ENSRNOG00000005964	**Nr4a3**	nuclear receptor subfamily 4, group A,	58853	5	68298772	68339859	41088	3123,04	-0,30	7,27E-01
ENSRNOG00000012236	**Hddc3**	HD domain containing 3 (Hddc3), mRI	308758	1	143088853	143091138	2286	497,56	-0,23	7,27E-01
ENSRNOG00000013306	**Pcdh20**	protocadherin 20 (Pcdh20), mRNA [Sc	306081	15	75353079	75359335	6257	258,47	-0,29	7,27E-01
ENSRNOG00000016848	**Fzd4**	frizzled family receptor 4 (Fzd4), mRN	64558	1	159893043	159896963	3921	82,86	-0,23	7,27E-01
ENSRNOG00000039469	**Pcdhb19**	Protein Pcdhb19; RCG49392 [Source	548106	18	30281822	30284230	2409	301,32	-0,21	7,27E-01
ENSRNOG00000018893	**Sfrp4**	secreted frizzled-related protein 4 (Sfr	89803	17	56095160	56105248	10089	38,52	-0,30	7,27E-01
ENSRNOG00000007462	**Sept8**	septin 8 (Sept8), mRNA [Source:RefS	303135	10	38659245	38682851	23607	2693,81	-0,27	7,28E-01
ENSRNOG00000036798	**LOC10036**	dual specificity phosphatase 3 (Dusp3	498003	10	89697010	89708826	11817	2619,05	0,14	7,28E-01
ENSRNOG00000019298	**Dctn4**	dynactin 4 (p62) (Dctn4), mRNA [Sour	84428	18	55030628	55056365	25738	2016,24	-0,17	7,28E-01
ENSRNOG00000008927	**Hbp1**	HMG-box transcription factor 1 (Hbp1)	27080	6	59902129	59928239	26111	1051,12	-0,25	7,28E-01
ENSRNOG00000016532	**Ino80c**	INO80 complex subunit C (Ino80c), m	291737	18	15757173	15771806	14634	892,64	0,23	7,28E-01
ENSRNOG00000019504		growth hormone regulated TBC protei	290880	16	81104734	81173894	69161	166,83	-0,29	7,28E-01
ENSRNOG00000022321	**Hapln3**	hyaluronan and proteoglycan link prot	308773	1	141796056	141803912	7857	49,71	-0,32	7,28E-01
ENSRNOG00000028384	**Npepl1**	aminopeptidase-like 1 (Npepl1), mRN	311671	3	178247574	178259415	11842	418,51	-0,33	7,28E-01
ENSRNOG00000048082		Uncharacterized protein [Source:UniProtKB/TrE		2	274839167	274840283	1117	247,50	-0,20	7,28E-01

ENSRNOG00000005191	**Trim44**	tripartite motif-containing 44 (Trim44),	362172	3	98836267	98894480	58214	1271,76	-0,21	7,28E-01
ENSRNOG00000019983	**Mlx**	MLX, MAX dimerization protein (Mlx),	360631	10	88795895	88800648	4754	1206,19	0,18	7,28E-01
ENSRNOG00000021044	**Gpatch8**	Protein Gpatch8 [Source:UniProtKB/T	685233	10	90212055	90225094	13040	1358,90	0,23	7,28E-01
ENSRNOG00000011321	**Rftn1**	raftlin lipid raft linker 1 (Rftn1), mRNA	501095	9	11679882	11880237	200356	144,10	-0,28	7,28E-01
ENSRNOG00000013171	**Grm2**	glutamate receptor, metabotropic 2 (G	24415	8	114705123	114718170	13048	302,12	-0,34	7,28E-01
ENSRNOG00000043059	**Aplf**	aprataxin and PNKP like factor (Aplf),	500247	4	184767521	184819739	52219	163,90	0,29	7,29E-01
ENSRNOG00000037720	**RGD13067**	similar to RIKEN cDNA 1110008J03 (F	288683	12	43344177	43346986	2810	832,26	0,27	7,29E-01
ENSRNOG00000021433	**Arhgef39**	Rho guanine nucleotide exchange fac	298404	5	63522806	63526373	3568	369,19	0,16	7,29E-01
ENSRNOG00000014874	**Zfyve28**	zinc finger, FYVE domain containing 2	305454	14	82412194	82497078	84885	359,97	0,20	7,29E-01
ENSRNOG00000024557	**Cep112**	centrosomal protein 112kDa (Cep112)	287776	10	96387919	96868508	480590	23,85	-0,33	7,29E-01
ENSRNOG00000001108	**N4bp2l2**	NEDD4 binding protein 2-like 2 (N4bp	288416	12	613303	634435	21133	520,45	-0,18	7,29E-01
ENSRNOG00000010515	**Trim59**	tripartite motif-containing 59 (Trim59),	365813	2	184991028	185002731	11704	176,07	-0,32	7,29E-01
ENSRNOG00000019534	**Ap2a2**	adaptor-related protein complex 2, alp	81637	1	221451396	221523284	71889	8767,20	-0,16	7,29E-01
ENSRNOG00000012710	**Ubac2**	UBA domain containing 2 (Ubac2), mR	361094	15	111674945	111821085	146141	1504,67	0,16	7,30E-01
ENSRNOG00000000122	**RGD15621**	adenylate kinase 2 (Ak2), transcript va	24184	5	150910956	150929525	18570	617,18	0,20	7,30E-01
ENSRNOG00000002609	**Ero1lb**	ERO1-like beta (S. cerevisiae) (Ero1lb	364755	17	92372954	92415877	42924	131,76	-0,33	7,30E-01
ENSRNOG00000012868	**Uaca**	uveal autoantigen with coiled-coil dom	315732	8	65436328	65524224	87897	267,10	0,29	7,30E-01
ENSRNOG00000015916	**Ttc38**	tetratricopeptide repeat domain 38 (Tt	300125	7	126422523	126446119	23597	113,71	-0,27	7,30E-01
ENSRNOG00000017787	**Ripk1**	receptor (TNFRSF)-interacting serine-	306886	17	33430661	33462906	32246	429,40	-0,22	7,30E-01
ENSRNOG00000018809	**Psmd5**	proteasome (prosome, macropain) 26	296651	3	19235769	19253052	17284	1694,24	0,18	7,30E-01
ENSRNOG00000025702		lysine-specific demethylase 2B [Sourc	304495	12	40918245	41054955	136711	3092,48	-0,13	7,30E-01
ENSRNOG00000009891	**Pcf11**	Protein Pcf11 [Source:UniProtKB/TrE	361605	1	163712787	163737959	25173	705,97	-0,23	7,30E-01
ENSRNOG00000024661	**Hn1l**	hematological and neurological expres	360492	10	14120135	14139812	19678	834,24	0,14	7,30E-01
ENSRNOG00000009421	**Ivd**	isovaleryl-CoA dehydrogenase (Ivd), n	24513	3	117207297	117228098	20802	2527,09	0,18	7,30E-01
ENSRNOG00000021602	**Zmynd10**	zinc finger, MYND-type containing 10 (	363139	8	115658773	115663130	4358	186,63	-0,31	7,30E-01
ENSRNOG00000004307	**Tor3a**	torsin family 3, member A (Tor3a), mR	304884	13	79174048	79198607	24560	286,52	0,28	7,30E-01
ENSRNOG00000013503	**Cdh24**	Protein Cdh24; Similar to Cadherin-lik	498515	15	37163508	37170574	7067	481,89	-0,25	7,30E-01
ENSRNOG00000023888	**Ccdc42b**	Protein Ccdc42b [Source:UniProtKB/	304509	12	43322023	43328003	5981	31,15	-0,33	7,30E-01
ENSRNOG00000003901	**Ccdc104**	coiled-coil domain containing 104 (Cc	289859	14	113294068	113319546	25479	2103,47	0,19	7,31E-01
ENSRNOG00000004425		kelch domain-containing protein 1 [Sc	314190	6	100991376	101041810	50435	212,97	0,28	7,31E-01
ENSRNOG00000007302	**Fbn1**	fibrillin 1 (Fbn1), mRNA [Source:RefSe	83727	3	124095126	124289500	194375	391,32	-0,15	7,31E-01
ENSRNOG00000012443	**Cpt2**	carnitine palmitoyltransferase 2 (Cpt2)	25413	5	131353542	131370821	17280	614,21	-0,27	7,31E-01
ENSRNOG00000014331	**LOC10036**	Protein LOC100361503 [Source:UniP	1E+08	8	23330592	23337875	7284	262,01	-0,25	7,31E-01
ENSRNOG00000014779	**Pdcd4**	programmed cell death 4 (Pdcd4), mR	64031	1	282038264	282061732	23469	2680,24	0,20	7,31E-01
ENSRNOG00000020308	**Ech1**	enoyl CoA hydratase 1, peroxisomal (I	64526	1	88190403	88196621	6219	2707,38	0,30	7,31E-01
ENSRNOG00000021669	**Mybl1**	myeloblastosis oncogene-like 1 (Mybl	297783	5	14079943	14113762	33820	117,94	-0,33	7,31E-01
ENSRNOG00000022681	**RGD15611**	Protein RGD1561113 [Source:UniPro	499780	3	16756950	16759602	2653	1277,54	0,29	7,31E-01
ENSRNOG00000024450		POC1 centriolar protein homolog B [S	1E+08	7	41330541	41439226	108686	186,66	-0,17	7,31E-01

ENSRNOG00000029184	**Epha6**			11	45455271	45755803	300533	58,21	-0,28	7,31E-01
ENSRNOG00000031576	**Zfp455**	zinc finger protein 455 (Zfp455), mRN.	286979	2	106665557	106680474	14918	30,77	-0,36	7,31E-01
ENSRNOG00000042114	**Erich1**	glutamate-rich 1 (Erich1), mRNA [Sou	306622	16	80168261	80234542	66282	219,78	0,21	7,31E-01
ENSRNOG00000043426		cysteine conjugate-beta lyase 2 [Source:MGI Sy		2	267174663	267220549	45887	162,32	0,21	7,31E-01
ENSRNOG00000049541	**Alas1**	aminolevulinate, delta-, synthase 1 (Al	65155	8	114289621	114299701	10081	836,42	0,21	7,31E-01
ENSRNOG00000008445	**Dact1**	Protein Dact1 [Source:UniProtKB/TrE	500666	6	103201967	103211190	9224	898,83	-0,28	7,31E-01
ENSRNOG00000013035	**Rab33b**	RAB33B, member RAS oncogene fam	365793	2	160016557	160027158	10602	857,32	-0,18	7,31E-01
ENSRNOG00000000490	**Rps10**	ribosomal protein S10 (Rps10), mRNA	81773	20	9418122	9422687	4566	1045,66	0,24	7,31E-01
ENSRNOG00000001312	**Pdgfa**	platelet-derived growth factor alpha pc	25266	12	19727664	19748066	20403	719,01	-0,22	7,31E-01
ENSRNOG00000001623	**Zfp295**	zinc finger protein 295 (Zfp295), mRN.	304056	11	41954466	41967238	12773	626,30	0,21	7,31E-01
ENSRNOG00000004401	**Mrpl13**	mitochondrial ribosomal protein L13 (M	299938	7	95910454	95932053	21600	413,03	0,25	7,31E-01
ENSRNOG00000004544	**Ube2e3**	ubiquitin-conjugating enzyme E2E 3 (U	295686	3	72378562	72434857	56296	3359,82	-0,18	7,31E-01
ENSRNOG00000006148	**RGD13103**	similar to HTGN29 protein; keratinocyt	303122	10	37530138	37542768	12631	4319,56	0,25	7,31E-01
ENSRNOG00000006987	**RGD15633**	uncharacterized protein LOC362857 |	362857	7	24797541	24817759	20219	383,90	-0,22	7,31E-01
ENSRNOG00000007546	**Asns**	asparagine synthetase (glutamine-hyd	25612	4	33608632	33626579	17948	2122,12	-0,21	7,31E-01
ENSRNOG00000011355	**LOC10090**	ribosomal protein S9 (Rps9), mRNA [S	81772	1	63078979	63082298	3320	271,20	0,25	7,31E-01
ENSRNOG00000011586	**Ccnl1**	cyclin L1 (Ccnl1), mRNA [Source:RefS	114121	2	177110681	177122888	12208	616,53	-0,29	7,31E-01
ENSRNOG00000014516	**Hs6st1**	heparan sulfate 6-O-sulfotransferase 1	316325	9	42274321	42313302	38982	1921,11	0,20	7,31E-01
ENSRNOG00000016381	**Ust**	uronyl-2-sulfotransferase (Ust), mRNA	361450	1	4022062	4313796	291735	124,14	-0,21	7,31E-01
ENSRNOG00000024929	**Nudcd1**	NudC domain containing 1 (Nudcd1),	362906	7	83317601	83362537	44937	175,99	-0,28	7,31E-01
ENSRNOG00000025065	**RGD15604**	uncharacterized protein LOC500546 |	500546	5	146702472	146707778	5307	84,39	-0,23	7,31E-01
ENSRNOG00000025768	**Clk1**	CDC-like kinase 1 (Clk1), mRNA [Sou	301434	9	65104058	65115299	11242	1109,17	-0,21	7,31E-01
ENSRNOG00000027436		Protein Zfp324; RCG27445 [Source:U	365192	1	66417753	66423048	5296	407,07	0,20	7,31E-01
ENSRNOG00000028353	**Sox13**	SRY (sex determining region Y)-box 1	289026	13	55487561	55531977	44417	253,92	0,27	7,31E-01
ENSRNOG00000050473	**LOC10036**	ribosomal protein S27-like (Rps27l), m	681429	8	77076269	77078924	2656	1094,05	-0,27	7,31E-01
ENSRNOG00000042741		androglobin [Source:MGI Symbol;Acc:	292477	1	6109841	6231640	121800	25,01	-0,36	7,31E-01
ENSRNOG00000005106		coiled-coil and C2 domain containing 2	498386	14	71922811	71997340	74530	299,06	-0,20	7,31E-01
ENSRNOG00000005390	**Nup210**	nucleoporin 210 (Nup210), mRNA [So	58958	4	187804480	187901455	96976	1455,53	-0,14	7,31E-01
ENSRNOG00000006001	**Luc7l2**	LUC7-like 2 (S. cerevisiae) (Luc7l2), n	312251	4	66101838	66165880	64043	2353,90	-0,17	7,31E-01
ENSRNOG00000022939	**Gpkow**	G patch domain and KOW motifs (Gpk	679890	X	16418016	16433060	15045	1237,31	0,24	7,31E-01
ENSRNOG00000005625	**Zc4h2**	zinc finger, C4H2 domain containing (2	367838	X	68162944	68183409	20466	898,33	0,17	7,32E-01
ENSRNOG00000016166	**Pdlim1**	PDZ and LIM domain 1 (Pdlim1), mRN	54133	1	266752410	266800857	48448	159,37	0,28	7,32E-01
ENSRNOG00000024482		trinucleotide repeat-containing gene 1	304302	12	15837335	15939011	101677	8520,34	0,12	7,32E-01
ENSRNOG00000006400	**Tbc1d14**	TBC1 domain family, member 14 (Tbc	360956	14	78978024	79077774	99751	5455,34	0,24	7,32E-01
ENSRNOG00000026178	**Adpgk**	ADP-dependent glucokinase (Adpgk),	315722	8	63850863	63878782	27920	845,43	0,16	7,32E-01
ENSRNOG00000005905		phosphodiesterase 4B, cAMP specific	24626	5	125536302	126001150	464849	626,37	-0,22	7,32E-01
ENSRNOG00000010538	**Commd7**	COMM domain containing 7 (Commd7	296285	3	155480868	155495101	14234	1337,95	0,17	7,32E-01
ENSRNOG00000018680	**LOC69139**	ribosomal protein L17 (Rpl17), mRNA	291434	18	70108978	70112081	3104	1264,55	0,25	7,32E-01

ENSRNOG00000045637	**Rnf14**	ring finger protein 14 (Rnf14), mRNA [	619577	18	31124493	31147232	22740	9586,12	0,24	7,32E-01
ENSRNOG00000017602	**Il34**	interleukin 34 (Il34), mRNA [Source:Re	498951	19	51714803	51728622	13820	92,01	-0,35	7,32E-01
ENSRNOG00000048207				7	122667599	122667694	96	114,74	0,23	7,32E-01
ENSRNOG00000004415	**Fam179b**	Protein Fam179b [Source:UniProtKB/	314169	6	96205062	96268221	63160	714,50	-0,20	7,32E-01
ENSRNOG00000008226	**Zbtb8os**	Protein Zbtb8os-ps1 [Source:UniProtI	297885	5	151257317	151268014	10698	334,22	-0,20	7,32E-01
ENSRNOG00000014251	**Capn5**	calpain 5 (Capn5), mRNA [Source:Ref	171495	1	169278330	169336535	58206	1748,04	0,17	7,32E-01
ENSRNOG00000021088	**Tmod4**	tropomodulin 4 (Tmod4), transcript var	295261	2	215789411	215794215	4805	36,95	-0,26	7,32E-01
ENSRNOG00000024101	**Phkb**	phosphorylase kinase, beta (Phkb), m	361377	19	33045255	33289198	243944	563,37	-0,27	7,32E-01
ENSRNOG00000025594	**Scrt1**	scratch homolog 1, zinc finger protein	366951	7	117571084	117574734	3651	794,49	-0,23	7,32E-01
ENSRNOG00000029996	**LOC10036(**	Elongation factor 1-alpha [Source:Uni	1E+08	3	156526503	156528665	2163	24,06	0,32	7,32E-01
ENSRNOG00000004242	**Pole2**	polymerase (DNA directed), epsilon 2,	299112	6	100953300	100977903	24604	95,92	0,26	7,32E-01
ENSRNOG00000023587		dehydrogenase E1 and transketolase domain cc		17	77943929	78014754	70826	551,69	-0,17	7,32E-01
ENSRNOG00000024539	**Ndufb6**	NADH dehydrogenase (ubiquinone) 1	297990	5	61110999	61120566	9568	2783,28	0,22	7,32E-01
ENSRNOG00000025284				10	46835286	46835774	489	34,17	0,33	7,32E-01
ENSRNOG00000006644	**Kdm4c**	lysine (K)-specific demethylase 4C (Kc	298144	5	94876819	95110733	233915	285,90	0,27	7,32E-01
ENSRNOG00000020479	**Pik3c2a**	phosphatidylinositol-4-phosphate 3-kir	361632	1	192182647	192297034	114388	492,00	-0,33	7,33E-01
ENSRNOG00000003680	**Gabrb2**	gamma-aminobutyric acid (GABA) A re	25451	10	27818095	28026899	208805	171,21	-0,35	7,33E-01
ENSRNOG00000014035	**Arhgef4**	Protein Arhgef4; Similar to Rho guanir	301334	9	40964597	40972103	7507	1850,14	-0,17	7,33E-01
ENSRNOG00000015676	**Brd9**	bromodomain containing 9 (Brd9), mR	308067	1	33363870	33392304	28435	1433,37	0,21	7,33E-01
ENSRNOG00000001346	**Cops6**	COP9 signalosome subunit 6 (Cops6)	304343	12	21361017	21363856	2840	2368,49	0,15	7,33E-01
ENSRNOG00000011007	**Ube2o**	Protein Ube2o [Source:UniProtKB/TrE	303689	10	105170740	105216698	45959	3330,70	0,21	7,34E-01
ENSRNOG00000025691	**Pla2g7**	phospholipase A2, group VII (platelet-a	301265	9	18813230	18855030	41801	448,96	-0,31	7,34E-01
ENSRNOG00000000557	**Sar1a**	SAR1 homolog A (S. cerevisiae) (Sar1	361842	20	33095744	33108149	12406	5465,96	0,20	7,34E-01
ENSRNOG00000000866	**Rbmx**	RNA binding motif protein, X-linked (R	302855	X	154511687	154518145	6459	1595,50	-0,23	7,34E-01
ENSRNOG00000004508		peroxidasin homolog precursor [Sour	554172	6	57551669	57661402	109734	483,35	0,21	7,34E-01
ENSRNOG00000006747		coiled-coil and C2 domain containing 1	288908	19	36224013	36239011	14999	1691,90	0,17	7,34E-01
ENSRNOG00000007568	**Spata6**	spermatogenesis associated 6 (Spata	171413	5	135542967	135626738	83772	379,44	-0,26	7,34E-01
ENSRNOG00000030680	**Ddx5**	DEAD (Asp-Glu-Ala-Asp) box helicase	287765	10	94726628	94734161	7534	10992,66	0,22	7,34E-01
ENSRNOG00000017290		Zinc finger protein 335 [Source:UniPr	259270	3	167542097	167560575	18479	1041,45	0,13	7,34E-01
ENSRNOG00000024186	**Eef1b2**	eukaryotic translation elongation facto	363241	9	69762337	69765057	2721	5729,00	0,17	7,34E-01
ENSRNOG00000029389				MT	9383	9450	68	95,58	-0,24	7,34E-01
ENSRNOG00000048859	**Nudcd3**	NudC domain containing 3 (Nudcd3),	1E+08	14	87224340	87294823	70484	1090,42	0,19	7,34E-01
ENSRNOG00000014870	**Slc13a5**	solute carrier family 13 (sodium-deper	266998	10	58550465	58574301	23837	49,18	0,30	7,34E-01
ENSRNOG00000020701	**Brca1**	breast cancer 1, early onset (Brca1), r	497672	10	89193680	89252502	58823	162,22	0,31	7,34E-01
ENSRNOG00000027455	**RGD15648**	similar to chromosome 1 open reading	313551	5	142085034	142091328	6295	482,85	0,18	7,34E-01
ENSRNOG00000050645	**Tprkb**	Tp53rk binding protein (Tprkb), mRNA	297411	4	182164105	182177471	13367	22,50	-0,29	7,34E-01
ENSRNOG00000002667	**Lamc2**	laminin, gamma 2 (Lamc2), mRNA [Sc	192362	13	75532726	75592335	59610	41,23	-0,30	7,34E-01
ENSRNOG00000007128	**Nop56**	NOP56 ribonucleoprotein (Nop56), mF	362214	3	129303487	129308275	4789	2248,74	0,25	7,34E-01

ENSRNOG00000019895	**Nedd8**	neural precursor cell expressed, devel	25490	15	38230806	38242650	11845	5233,77	0,22	7,34E-01
ENSRNOG00000004181	**Sdccag8**	serologically defined colon cancer anti	305002	13	99341229	99528213	186985	426,29	0,16	7,34E-01
ENSRNOG00000005990	**Wdsub1**	WD repeat, sterile alpha motif and U-b	362137	3	51036940	51067275	30336	197,64	-0,22	7,34E-01
ENSRNOG00000010121	**Lef1**	lymphoid enhancer binding factor 1 (Le	161452	2	254782387	254876426	94040	36,86	-0,26	7,34E-01
ENSRNOG00000013251		transcription intermediary factor 1-alph	500084	4	65457120	65504094	46975	688,04	-0,15	7,34E-01
ENSRNOG00000016968	**Gramd4**	Protein Gramd4 [Source:UniProtKB/T	315203	7	126681301	126721804	40504	1111,10	-0,21	7,34E-01
ENSRNOG00000047970		Protein Lyrm4; RCG44066 [Source:UniProtKB/T		17	31335896	31449161	113266	205,20	0,18	7,34E-01
ENSRNOG00000012269	**Lpcat3**	lysophosphatidylcholine acyltransferas	362434	4	224199925	224240592	40668	1538,02	-0,15	7,34E-01
ENSRNOG00000016062	**Snta1**	syntrophin, alpha 1 (Snta1), mRNA [So	362242	3	156245201	156278466	33266	827,50	-0,28	7,35E-01
ENSRNOG00000010645	**Lgals3**	lectin, galactoside-binding, soluble, 3 (	83781	15	28094062	28106276	12215	334,70	0,35	7,35E-01
ENSRNOG00000012899	**Rbbp8**	retinoblastoma binding protein 8 (Rbbp	291787	18	3175473	3239957	64485	243,94	-0,31	7,35E-01
ENSRNOG00000016187	**Tmem67**	transmembrane protein 67 (Tmem67),	313067	5	30371964	30424943	52980	525,88	-0,28	7,35E-01
ENSRNOG00000018928		zinc finger protein 59 [Source:MGI Syn	1E+08	1	85951318	85965377	14060	50,53	-0,33	7,35E-01
ENSRNOG00000019657	**Mk1**	Mk1 protein (Mk1), mRNA [Source:Re	171436	16	20388727	20389104	378	371,99	0,18	7,35E-01
ENSRNOG00000019922	**Ntpcr**	nucleoside-triphosphatase, cancer-rela	361443	19	69262818	69276518	13701	630,33	0,18	7,35E-01
ENSRNOG00000025011	**Chd8**	chromodomain helicase DNA binding p	65027	15	32423199	32482730	59532	3356,82	0,13	7,35E-01
ENSRNOG00000026026	**Stam**	signal transducing adaptor molecule (S	498798	17	82954327	83002354	48028	2101,65	-0,15	7,35E-01
ENSRNOG00000026742	**Ypel5**	yippee-like 5 (Drosophila) (Ypel5), mR	298792	6	33915155	33935174	20020	1284,27	-0,19	7,35E-01
ENSRNOG00000033038		Mitochondrial intermembrane space in	312559	1	255649505	255649924	420	21,90	0,31	7,35E-01
ENSRNOG00000038212		suppressor of cytokine signaling 6 [So	307200	18	85006285	85007928	1644	342,99	0,18	7,35E-01
ENSRNOG00000019964		Serine/threonine-protein kinase TAO2 [Source:U		1	205281971	205300874	18904	5503,16	0,22	7,35E-01
ENSRNOG00000029165	**Stx1a**	syntaxin 1A (brain) (Stx1a), mRNA [So	116470	12	26682320	26710272	27953	4955,28	0,22	7,35E-01
ENSRNOG00000009143	**Lrrc57**	leucine rich repeat containing 57 (Lrrc57), mRNA		3	118912834	118918178	5345	335,41	-0,19	7,35E-01
ENSRNOG00000003350	**Mospd2**	motile sperm domain containing 2 (Mo	363463	X	31520482	31562128	41647	273,67	-0,31	7,35E-01
ENSRNOG00000002999	**Timmdc1**	translocase of inner mitochondrial mem	303922	11	67294276	67318850	24575	929,20	0,20	7,35E-01
ENSRNOG00000011267	**Zfp704**	Protein Zfp704 [Source:UniProtKB/Tr	310233	2	114131352	114304907	173556	690,10	-0,17	7,35E-01
ENSRNOG00000037508	**Hscb**	HscB iron-sulfur cluster co-chaperone	360826	12	53616659	53626996	10338	330,61	-0,19	7,35E-01
ENSRNOG00000026136	**Tnfaip8**	tumor necrosis factor, alpha-induced p	307428	18	43960149	44002489	42341	55,12	0,28	7,35E-01
ENSRNOG00000000112	**Champ1**	chromosome alignment maintaining ph	306647	16	80403595	80414561	10967	1788,44	0,16	7,36E-01
ENSRNOG00000004093	**Rhot1**	ras homolog gene family, member T1	303351	10	67216682	67297278	80597	1148,81	-0,19	7,36E-01
ENSRNOG00000007545	**Angptl4**	angiopoietin-like 4 (Angptl4), mRNA [S	362850	7	18805416	18811643	6228	524,88	-0,31	7,36E-01
ENSRNOG00000011501	**Atp1b3**	ATPase, Na+/K+ transporting, beta 3 p	25390	8	103638324	103669634	31311	1402,50	0,21	7,36E-01
ENSRNOG00000017777	**Ahcy**	adenosylhomocysteinase (Ahcy), mRN	29443	3	156957240	156972458	15219	1186,14	0,15	7,36E-01
ENSRNOG00000012415	**Mpc1**	mitochondrial pyruvate carrier 1 (Mpc1	171087	1	54275046	54286667	11622	3662,26	-0,17	7,36E-01
ENSRNOG00000019692	**Metrn**	meteorin, glial cell differentiation regul	287151	10	14977382	14979400	2019	3783,34	0,34	7,36E-01
ENSRNOG00000021966	**Il17rd**	interleukin 17 receptor D (Il17rd), mRN	498576	16	2643153	2704143	60991	144,88	0,19	7,36E-01
ENSRNOG00000025877	**Prr12**	Protein Prr12 [Source:UniProtKB/TrEl	361569	1	102088242	102110835	22594	2673,92	0,18	7,36E-01
ENSRNOG00000009899		SKI-like [Source:MGI Symbol;Acc:MG	114208	2	135562877	135584672	21796	447,92	0,26	7,37E-01

ENSRNOG00000008652		pleckstrin homology domain interactin	315843	8	89920884	90013180	92297	386,46	0,34	7,37E-01
ENSRNOG00000013111	**Mettl3**	methyltransferase-like 3 (Mettl3), mRN	361035	15	32520600	32531328	10729	1409,07	0,28	7,37E-01
ENSRNOG00000018969	**Gpatch4**	G patch domain containing 4 (Gpatch4	295228	2	206818279	206827062	8784	200,04	-0,27	7,37E-01
ENSRNOG00000019127	**Zfp606**	Protein Zfp606; RCG27439 [Source:U	292610	1	67027751	67050977	23227	154,54	-0,32	7,37E-01
ENSRNOG00000006236	**Dsn1**	DSN1, MIS12 kinetochore complex co	499933	3	158598784	158611010	12227	182,50	0,26	7,37E-01
ENSRNOG00000001878	**Klhl22**	kelch-like family member 22 (Klhl22),	303792	11	90730411	90771250	40840	2523,36	0,16	7,37E-01
ENSRNOG00000009615	**Mtor**	mechanistic target of rapamycin (serin	56718	5	168920401	169030568	110168	3876,39	0,17	7,37E-01
ENSRNOG00000011980		leucine rich repeat containing 7 (Lrrc7	117284	2	283550319	283934368	384050	711,25	-0,17	7,37E-01
ENSRNOG00000014584		outer dense fiber of sperm tails 2 (Odf	29479	3	13793731	13838706	44976	1693,09	0,15	7,37E-01
ENSRNOG00000022368	**Ddx49**	DEAD (Asp-Glu-Ala-Asp) box polypep	290660	16	20725435	20733081	7647	1675,48	0,19	7,37E-01
ENSRNOG00000042239		Protein Ube2l3; Ubiquitin-conjugating	363836	11	91139989	91140489	501	788,08	0,17	7,37E-01
ENSRNOG00000001388		serine dehydratase (Sds), mRNA [Sou	25044	12	43473326	43478300	4975	26,52	0,28	7,38E-01
ENSRNOG00000003982		tyrosine-protein phosphatase non-rece	171070	6	131870742	131920588	49847	276,36	-0,22	7,38E-01
ENSRNOG00000009863	**Prrt3**	Protein Prrt3 [Source:UniProtKB/TrEM	502873	4	208748160	208752133	3974	549,05	0,22	7,38E-01
ENSRNOG00000026239	**Arid4a**	AT rich interactive domain 4A (Rbp1 lil	314205	6	102917436	102998489	81054	424,91	-0,26	7,38E-01
ENSRNOG00000001956	**Dzip3**	Protein Dzip3 [Source:UniProtKB/TrE	303963	11	57580223	57635241	55019	545,58	-0,33	7,38E-01
ENSRNOG00000005539	**Thg1l**	tRNA-histidine guanylyltransferase 1-li	303067	10	30866224	30873429	7206	247,81	0,20	7,38E-01
ENSRNOG00000011104	**LOC10036**	ribosomal protein L22 (Rpl22), mRNA	81768	5	173055215	173066404	11190	738,24	0,23	7,38E-01
ENSRNOG00000048377		zinc finger protein 646 [Source:MGI Symbol;Acc		1	206349635	206351371	1737	165,80	0,22	7,38E-01
ENSRNOG00000001515		sterile alpha motif and leucine zipper c	311743	3	65449437	65598050	148614	353,71	-0,30	7,38E-01
ENSRNOG00000004263	**Ints7**	integrator complex subunit 7 (Ints7), m	289382	13	114821472	114873815	52344	1389,10	0,14	7,38E-01
ENSRNOG00000016684	**Wnk2**	WNK lysine deficient protein kinase 2	306811	17	17987437	18095251	107815	3668,58	0,17	7,38E-01
ENSRNOG00000043410	**LOC65448**	RIKEN cDNA 9230110C19 gene [Sour	654482	8	6217032	6234606	17575	157,48	-0,22	7,38E-01
ENSRNOG00000002177	**Gnpda2**	glucosamine-6-phosphate deaminase	289608	14	62731015	62749014	18000	172,14	-0,26	7,38E-01
ENSRNOG00000018602		calmodulin binding transcription activa	362665	5	171715302	171964129	248828	4310,08	-0,22	7,38E-01
ENSRNOG00000030213		vacuolar protein sorting 13C (yeast) [Source:MG		8	78012253	78174561	162309	904,54	0,25	7,38E-01
ENSRNOG00000046149		spectrin repeat containing, nuclear env	499010	1	43236641	43269644	33004	127,85	-0,21	7,39E-01
ENSRNOG00000012584	**Aamdc**	adipogenesis associated, Mth938 dom	361606	1	168726362	168740676	14315	241,39	-0,32	7,39E-01
ENSRNOG00000013927	**Jag2**	Delta-like protein [Source:UniProtKB/	29147	6	146708461	146729480	21020	677,61	0,21	7,39E-01
ENSRNOG00000014436	**Ppip5k1**	diphosphoinositol pentakisphosphate k	311355	3	119812648	119851674	39027	598,33	0,26	7,39E-01
ENSRNOG00000008347	**Slc43a3**	solute carrier family 43, member 3 (Slc	311170	3	78845160	78865582	20423	21,38	-0,30	7,39E-01
ENSRNOG00000005788	**Cdk5rap2**	CDK5 regulatory subunit associated p	286919	5	90474208	90641322	167115	957,76	0,28	7,39E-01
ENSRNOG00000008079	**Ugp2**	UDP-glucose pyrophosphorylase 2 (U	289827	14	106274950	106315312	40363	1437,85	-0,24	7,39E-01
ENSRNOG00000042359	**Pigk**	phosphatidylinositol glycan anchor bio	295543	2	276578101	276662884	84784	1391,21	0,17	7,39E-01
ENSRNOG00000009997	**Erap1**	endoplasmic reticulum aminopeptidas	80897	2	1381110	1419644	38535	449,82	-0,29	7,39E-01
ENSRNOG00000012343	**Pdp2**	pyruvate dehyrogenase phosphatase	246311	19	591632	595934	4303	225,02	-0,26	7,40E-01
ENSRNOG00000015349	**Six5**	Protein Six5 [Source:UniProtKB/TrEM	308406	1	81267940	81271004	3065	96,71	0,27	7,40E-01
ENSRNOG00000016226		ribonuclease P/MRP 40 subunit (Rpp4	291071	17	31534575	31543590	9016	117,38	-0,24	7,40E-01

ENSRNOG00000007224	**LOC50003**	RIKEN cDNA B630005N14 gene [Sou	500034		4	39890106	39963743	73638	496,63	-0,19	7,40E-01
ENSRNOG00000010912	**Mrps25**	mitochondrial ribosomal protein S25 (M	297459		4	189213384	189225146	11763	1248,26	0,14	7,40E-01
ENSRNOG00000004067		neuron-glia-CAM-related cell adhesion molecule			6	74371672	74446689	75018	4826,06	-0,17	7,40E-01
ENSRNOG00000014623	**Smurf2**	SMAD specific E3 ubiquitin protein liga	303614		10	94763750	94819535	55786	1508,96	-0,19	7,40E-01
ENSRNOG00000000636	**Rtkn2**	rhotekin 2 (Rtkn2), mRNA [Source:Ref	309729		20	24031447	24102848	71402	184,45	-0,28	7,41E-01
ENSRNOG00000002848	**Maoa**	monoamine oxidase A (Maoa), mRNA	29253	X		7373361	7439125	65765	446,45	-0,26	7,41E-01
ENSRNOG00000007583	**Pygb**	phosphorylase, glycogen; brain (Pygb	25739		3	152941739	152988415	46677	8887,50	-0,23	7,41E-01
ENSRNOG00000009245	**Exosc2**	exosome component 2 (Exosc2), mRN	366017		3	15511096	15521359	10264	883,81	0,15	7,41E-01
ENSRNOG00000012466	**Heca**	headcase homolog (Drosophila) (Heca	308624		1	14730556	14744291	13736	1196,75	-0,17	7,41E-01
ENSRNOG00000012882		ankyrin repeat domain 27 (VPS9 domain) [Sourc			1	92990234	93089746	99513	309,27	-0,16	7,41E-01
ENSRNOG00000022094	**Lacc1**	Protein Lacc1 [Source:UniProtKB/TrE	313790		15	62892256	62898184	5929	42,58	-0,33	7,41E-01
ENSRNOG00000023162	**Car14**	carbonic anhydrase 14 (Car14), mRNA	791259		2	217500706	217507185	6480	80,49	0,31	7,41E-01
ENSRNOG00000024414	**Ate1**	arginyltransferase 1 (Ate1), mRNA [Sc	293526		1	207845503	208113961	268459	635,85	0,22	7,41E-01
ENSRNOG00000048267		torsin A interacting protein 2 (Tor1aip2	304881		13	78633864	78645478	11615	1722,59	0,24	7,41E-01
ENSRNOG00000007270	**Il12rb2**	interleukin 12 receptor, beta 2 (Il12rb2	171334		4	162838651	162904433	65783	30,63	-0,30	7,41E-01
ENSRNOG00000014530	**Nav2**	neuron navigator 2 (Nav2), mRNA [So	171563		1	105631322	105994388	363067	3200,67	-0,21	7,41E-01
ENSRNOG00000000503	**Ppard**	peroxisome proliferator-activated rece	25682		20	10018464	10083772	65309	482,81	-0,25	7,41E-01
ENSRNOG00000020005	**Map2k2**	mitogen activated protein kinase kinas	58960		7	11626352	11645697	19346	2904,75	0,20	7,41E-01
ENSRNOG00000022524	**RGD15598**	Protein RGD1559841 [Source:UniPro	307816		19	49713671	49834380	120710	241,99	0,18	7,41E-01
ENSRNOG00000030750	**Zfp2**	zinc finger protein 2 (Zfp2), mRNA [So	497897		10	36160914	36175396	14483	267,17	-0,25	7,41E-01
ENSRNOG00000032429	**Tmprss9**	transmembrane protease, serine 9 (Tr	314636		7	11846111	11866968	20858	40,89	0,28	7,41E-01
ENSRNOG00000049308	**Sfxn2**	sideroflexin 2 (Sfxn2), mRNA [Source:	294011		1	273764160	273776423	12264	58,38	-0,23	7,41E-01
ENSRNOG00000003993	**Thap2**	Protein Thap2 [Source:UniProtKB/TrE	688019		7	58363484	58374215	10732	64,09	-0,31	7,41E-01
ENSRNOG00000007340	**Dcp1b**	Protein Dcp1b [Source:UniProtKB/TrE	500305		4	217165863	217200852	34990	392,33	0,17	7,41E-01
ENSRNOG00000001609	**Cep97**	centrosomal protein 97 (Cep97), mRN	304007		11	50327805	50355273	27469	228,90	0,21	7,41E-01
ENSRNOG00000002296	**Nfxl1**	nuclear transcription factor, X-box binc	289595		14	38003888	38044774	40887	512,17	-0,29	7,41E-01
ENSRNOG00000007230	**Kank3**	KN motif and ankyrin repeat domains	366848		7	18846292	18859539	13248	68,65	-0,29	7,41E-01
ENSRNOG00000008035	**Cinp**	cyclin-dependent kinase 2-interacting	299334		6	145109734	145123627	13894	1037,68	0,20	7,41E-01
ENSRNOG00000009052		Ab2-255; Protein Igf2bp3 [Source:UniProtKB/Tr			4	143435311	143447771	12461	87,75	0,24	7,41E-01
ENSRNOG00000010436	**Zbtb17**	zinc finger and BTB domain containing	313666		5	163713685	163734604	20920	995,37	0,17	7,41E-01
ENSRNOG00000013040	**Ddx31**	DEAD (Asp-Glu-Ala-Asp) box polypep	311835		3	12773312	12837612	64301	322,68	0,20	7,41E-01
ENSRNOG00000013300	**Atpif1**	ATPase inhibitory factor 1 (Atpif1), mR	25392		5	154387824	154391541	3718	3506,06	0,14	7,41E-01
ENSRNOG00000016536	**Pdik1l**	PDLIM1 interacting kinase 1 like (Pdik	313609		5	156253978	156265698	11721	396,76	-0,24	7,41E-01
ENSRNOG00000016870	**Pcif1**	PDX1 C-terminal inhibiting factor 1 (Pc	362269		3	167528503	167541435	12933	1690,47	0,18	7,41E-01
ENSRNOG00000019136	**Scamp2**	secretory carrier membrane protein 2	65168		8	62119185	62145786	26602	1421,03	0,21	7,41E-01
ENSRNOG00000019738	**Gpr61**	G protein-coupled receptor 61 (Gpr61)	310780		2	230406537	230412868	6332	348,59	-0,22	7,41E-01
ENSRNOG00000025003					1	142981854	142982647	794	70,56	0,23	7,41E-01
ENSRNOG00000030443	**Rab9a**	RAB9A, member RAS oncogene famil	84589	X		29918161	29940587	22427	316,62	-0,18	7,41E-01

ENSRNOG00000030880	**Hs6st2**	heparan sulfate 6-O-sulfotransferase 2	302489	X	138736166	139027990	291825	465,79	-0,15	7,41E-01
ENSRNOG00000032590	**Ttc28**	Protein Ttc28 [Source:UniProtKB/TrE	304558	12	53130431	53332482	202052	2660,86	0,13	7,41E-01
ENSRNOG00000039025				3	35062657	35063057	401	99,26	-0,27	7,41E-01
ENSRNOG00000047506	**LOC10036**	SREK1-interacting protein 1 (Srek1ip1	361888	7	122151954	122156372	4419	135,36	-0,22	7,41E-01
ENSRNOG00000048608		Uncharacterized protein [Source:UniProtKB/TrE		10	3313219	3324552	11334	49,32	0,33	7,41E-01
ENSRNOG00000049988	**Mbtd1**	Protein LOC688133 [Source:UniProtK	688133	10	81422273	81472427	50155	570,68	-0,16	7,41E-01
ENSRNOG00000018762	**Rsph3**	Protein Rsph3 [Source:UniProtKB/TrE	361476	1	48743738	48780631	36894	168,71	0,27	7,42E-01
ENSRNOG00000043162	**Herc1**	Protein Herc1 [Source:UniProtKB/TrE	315771	8	71775427	71862567	87141	3198,05	0,20	7,42E-01
ENSRNOG00000006789		DNA-damage inducible transcript 3 (D	29467	7	70754398	70759220	4823	538,54	0,28	7,42E-01
ENSRNOG00000013228	**Scrg1**	stimulator of chondrogenesis 1 (Scrg1	64458	16	35936797	35967145	30349	280,79	-0,34	7,42E-01
ENSRNOG00000016991	**Arl2bp**	ADP-ribosylation factor-like 2 binding p	498910	19	10747793	10757367	9575	7409,33	-0,14	7,42E-01
ENSRNOG00000015634		mothers against decapentaplegic hom	50554	18	68773453	68802426	28974	1491,29	0,21	7,42E-01
ENSRNOG00000016774	**Telo2**	Protein Telo2 [Source:UniProtKB/TrE	302986	10	14277469	14291593	14125	652,33	-0,21	7,43E-01
ENSRNOG00000004826	**Sos2**	son of sevenless homolog 2 (Drosoph	85384	6	101337035	101457492	120458	1533,98	-0,15	7,43E-01
ENSRNOG00000007160	**Csrp2bp**	CSRP2 binding protein (Csrp2bp), mR	362224	3	144939294	144979526	40233	476,19	0,19	7,43E-01
ENSRNOG00000011416	**Vegfc**	vascular endothelial growth factor C (V	114111	16	40216307	40331753	115447	63,70	0,24	7,43E-01
ENSRNOG00000012679	**RGD13082**	similar to chromosome 6 open reading	367214	9	13467179	13474021	6843	138,58	-0,22	7,43E-01
ENSRNOG00000021035	**Rpl18**	ribosomal protein L18 (Rpl18), mRNA	81766	1	102779721	102782381	2661	8831,54	0,26	7,43E-01
ENSRNOG00000000473	**Pfdn6**	prefoldin subunit 6 (Pfdn6), mRNA [Sc	309629	20	7514596	7516060	1465	1406,86	0,17	7,43E-01
ENSRNOG00000002433	**G3bp2**	GTPase activating protein (SH3 doma	305240	14	17409459	17438170	28712	2847,58	-0,19	7,43E-01
ENSRNOG00000004276		Integrin alpha 3 variant A; Protein Itga	360606	10	82667994	82697897	29904	691,51	0,24	7,43E-01
ENSRNOG00000012597	**RGD13118**	similar to RIKEN cDNA 2400010D15 (	291784	18	3606715	3626293	19579	846,77	0,24	7,43E-01
ENSRNOG00000013239		transducin-like enhancer of split 4 (E(sp1) homo		1	238531404	238666772	135369	1319,71	0,15	7,43E-01
ENSRNOG00000019145	**Kdm2a**	lysine (K)-specific demethylase 2A (Kc	361700	1	226436269	226508225	71957	3303,33	0,18	7,43E-01
ENSRNOG00000019778	**Ptrf**	polymerase I and transcript release fa	287710	10	88659595	88671569	11975	571,98	0,27	7,43E-01
ENSRNOG00000019840	**Mdp1**	magnesium-dependent phosphatase 1	290230	15	38226257	38229155	2899	488,68	0,18	7,43E-01
ENSRNOG00000024363	**Sertad1**	SERTA domain containing 1 (Sertad1)	361526	1	85510184	85513470	3287	143,10	0,23	7,43E-01
ENSRNOG00000031651		Ac1054; Ac2-193; RCG63365; Uncharacterized		9	43701732	43714993	13262	321,37	0,20	7,43E-01
ENSRNOG00000019528	**Plekhg6**	Protein Plekhg6 [Source:UniProtKB/T	1E+08	4	224894466	224910903	16438	77,44	-0,31	7,43E-01
ENSRNOG00000026236	**Morc3**	MORC family CW-type zinc finger 3 (M	304074	11	37641727	37683916	42190	580,31	-0,22	7,43E-01
ENSRNOG00000033844	**Bex1**	brain expressed, X-linked 1 (Bex1), m	501625	1	117724202	117725601	1400	1468,75	0,19	7,43E-01
ENSRNOG00000001409	**Gnb2**	guanine nucleotide binding protein (G	81667	12	24245847	24250864	5018	9019,66	0,22	7,43E-01
ENSRNOG00000008418	**Gtpbp5**	GTP binding protein 5 (Gtpbp5), mRN	296462	3	181654577	181661966	7390	803,51	0,14	7,43E-01
ENSRNOG00000013954	**Alpl**	alkaline phosphatase, liver/bone/kidne	25586	5	159841923	159897413	55491	162,14	-0,21	7,43E-01
ENSRNOG00000020315	**Taf13**	TAF13 RNA polymerase II, TATA box b	310784	2	230830656	230841317	10662	703,69	-0,29	7,43E-01
ENSRNOG00000006733	**Srgap2**	SLIT-ROBO Rho GTPase activating p	360840	13	53149845	53362638	212794	2463,24	-0,17	7,44E-01
ENSRNOG00000016825	**Cd3eap**	CD3e molecule, epsilon associated pr	680493	1	81533986	81537262	3277	370,06	0,14	7,44E-01
ENSRNOG00000046242		Kruppel-like factor 7 (Ubiquitous) (Pre	363243	9	71832274	71832558	285	222,34	-0,25	7,44E-01

ENSRNOG00000002899	**Akap10**	A kinase (PRKA) anchor protein 10 (A	360540	10	47943748	48001356	57609	487,29	-0,18	7,44E-01
ENSRNOG00000004367	**Elk3**	ELK3, member of ETS oncogene fami	362871	7	34151383	34186857	35475	392,26	0,21	7,44E-01
ENSRNOG00000008210	**Ky**	kyphoscoliosis peptidase (Ky), mRNA	315962	8	110380954	110420390	39437	27,85	-0,33	7,44E-01
ENSRNOG00000010421	**Wdr91**	WD repeat domain 91 (Wdr91), mRNA	312225	4	62130769	62166447	35679	855,09	-0,14	7,44E-01
ENSRNOG00000019027	**Habp4**	hyaluronan binding protein 4 (Habp4),	361196	17	1669173	1691084	21912	1029,87	0,23	7,44E-01
ENSRNOG00000025843	**Ccdc102a**	coiled-coil domain containing 102A (C	361363	19	10516256	10531535	15280	192,18	-0,22	7,44E-01
ENSRNOG00000032946	**Pdzd7**	PDZ domain containing 7 (Pdzd7), mF	293996	1	272218787	272237327	18541	58,09	0,31	7,44E-01
ENSRNOG00000048879		predicted gene, 20721 [Source:MGI Symbol;Acc		3	178489205	178489888	684	113,07	0,25	7,44E-01
ENSRNOG00000010882	**Sptlc1**	serine palmitoyltransferase, long chair	361213	17	13956140	13995224	39085	1123,85	0,28	7,44E-01
ENSRNOG00000005188	**Tspan11**	tetraspanin 11 (Tspan11), mRNA [Sou	312727	4	231093059	231159271	66213	1597,17	0,24	7,44E-01
ENSRNOG00000031939		Uncharacterized protein [Source:UniProtKB/TrE		9	7646711	7654388	7678	139,54	-0,20	7,44E-01
ENSRNOG00000038638	**LOC498426**			14	108015700	108017820	2121	522,00	0,24	7,44E-01
ENSRNOG00000005970	**Lrrc1**	leucine rich repeat containing 1 (Lrrc1	367113	8	84292834	84406002	113169	222,63	-0,26	7,44E-01
ENSRNOG00000025083		protein kinase C and casein kinase su	29704	20	9482180	9489048	6869	634,48	-0,16	7,44E-01
ENSRNOG00000021891	**Zdhhc8**	zinc finger, DHHC-type containing 8 (Z	303796	11	89997060	90009653	12594	976,78	0,16	7,44E-01
ENSRNOG00000043077	**Zfpm1**	zinc finger protein, multitype 1 (Zfpm1	691504	19	65810197	65865945	55749	192,95	-0,31	7,44E-01
ENSRNOG00000001214	**Pfkl**	phosphofructokinase, liver (Pfkl), mRN	25741	20	13563769	13585934	22166	2846,48	-0,14	7,45E-01
ENSRNOG00000014050	**Ptges2**	prostaglandin E synthase 2 (Ptges2), i	311865	3	16773065	16780249	7185	1061,68	0,19	7,45E-01
ENSRNOG00000014149	**Npy1r**	neuropeptide Y receptor Y1 (Npy1r), n	29358	16	24663045	24671859	8815	327,42	-0,24	7,45E-01
ENSRNOG00000009282	**Nub1**	negative regulator of ubiquitin-like prot	296731	4	6964481	6994708	30228	2972,34	0,23	7,45E-01
ENSRNOG00000013850	**Hes5**	hairy and enhancer of split 5 (Drosoph	79225	5	175820392	175821159	768	454,42	-0,33	7,45E-01
ENSRNOG00000048243	**LOC10090**	heparan sulfate 6-O-sulfotransferase 3	364476	15	108662014	108662727	714	165,32	-0,24	7,45E-01
ENSRNOG00000015255	**Haus3**	HAUS augmin-like complex, subunit 3	680477	14	82523103	82542098	18996	133,55	-0,28	7,45E-01
ENSRNOG00000025920	**Unc5a**	unc-5 homolog A (C. elegans) (Unc5a)	60629	17	12262674	12317433	54760	1048,63	-0,21	7,45E-01
ENSRNOG00000000546	**Nt5dc1**	5'-nucleotidase domain containing 1 (N	294456	20	42836806	42938666	101861	195,84	-0,18	7,45E-01
ENSRNOG00000000894		furry homolog (Drosophila) [Source:MGI Symbol		12	7565891	7876576	310686	4301,36	0,21	7,45E-01
ENSRNOG00000002303	**Kcnj12**	potassium inwardly-rectifying channel,	117052	10	47055582	47114620	59039	126,03	0,26	7,45E-01
ENSRNOG00000003025	**Nap1l2**	nucleosome assembly protein 1-like 2	317247	X	74802929	74804314	1386	841,35	0,19	7,45E-01
ENSRNOG00000004304	**Syap1**	synapse associated protein 1 (Syap1)	302678	X	33875314	33909005	33692	459,52	-0,25	7,45E-01
ENSRNOG00000006237	**Gpr37l1**	G protein-coupled receptor 37-like 1 ((	252939	13	57037410	57044257	6848	1616,04	-0,29	7,45E-01
ENSRNOG00000007924	**Mios**	missing oocyte, meiosis regulator, hon	362324	4	34098618	34124193	25576	292,81	-0,26	7,45E-01
ENSRNOG00000010930		U4/U6.U5 tri-snRNP-associated protei	297336	4	164952387	164984619	32233	1037,49	0,22	7,45E-01
ENSRNOG00000011278	**Stk3**	serine/threonine kinase 3 (Stk3), mRN	65189	7	73793742	74055204	261463	138,01	-0,23	7,45E-01
ENSRNOG00000012247	**Rab7a**	RAB7A, member RAS oncogene famil	29448	4	185158361	185205835	47475	7477,79	0,14	7,45E-01
ENSRNOG00000012803	**Wdr61**	WD repeat domain 61 (Wdr61), mRNA	363064	8	57877398	57894572	17175	1165,08	0,14	7,45E-01
ENSRNOG00000020361	**Ip6k2**	inositol hexakisphosphate kinase 2 (Ip	59268	8	116921872	116944749	22878	2126,02	0,21	7,45E-01
ENSRNOG00000021174	**Macrod1**	MACRO domain containing 1 (Macrod	246233	1	229301823	229442054	140232	399,01	0,21	7,45E-01
ENSRNOG00000024114		zinc finger, DBF-type containing 2 [So	501153	9	69879607	69894995	15389	202,47	0,28	7,45E-01

ENSRNOG00000024429	**Peg12**	paternally expressed 12 (Peg12), mRI	308692		1	124237097	124237921	825	44,22	0,24	7,45E-01
ENSRNOG00000025551	**Rgs22**	Protein Rgs22 [Source:UniProtKB/TrE	500853		7	74941968	75024694	82727	50,34	-0,34	7,45E-01
ENSRNOG00000026267	**Igbp1**	immunoglobulin (CD79A) binding prot	58845	X		71194717	71216954	22238	580,17	0,15	7,45E-01
ENSRNOG00000028382	**Rfxap**	regulatory factor X-associated protein	499617		2	163630968	163635150	4183	1405,77	-0,20	7,45E-01
ENSRNOG00000019712	**Cpsf3l**	cleavage and polyadenylation specific	298688		5	176794095	176812548	18454	1189,71	0,18	7,45E-01
ENSRNOG00000023936					17	12782625	12782915	291	26,42	0,31	7,45E-01
ENSRNOG00000005615	**Gadd45a**	growth arrest and DNA-damage-induc	25112		4	162568471	162570782	2312	285,53	0,22	7,45E-01
ENSRNOG00000037659		mitochondrial translation optimization 1 homolog			8	85357705	85381270	23566	462,78	0,18	7,46E-01
ENSRNOG00000010813	**Tspan14**	tetraspanin 14 (Tspan14), mRNA [Sou	306324		16	17421546	17478815	57270	2247,73	0,15	7,46E-01
ENSRNOG00000017220	**Tcirg1**	T-cell, immune regulator 1, ATPase, H	293650		1	226000345	226009795	9451	327,95	-0,29	7,46E-01
ENSRNOG00000046383					10	72190116	72190211	96	166,95	0,24	7,46E-01
ENSRNOG00000012271	**Cnot1**	CCR4-NOT transcription complex, sub	291841		19	9653369	9746683	93315	4576,30	0,13	7,46E-01
ENSRNOG00000030163	**Epb41l4b**	erythrocyte membrane protein band 4.	500464		5	78259812	78344997	85186	117,83	-0,23	7,46E-01
ENSRNOG00000004193	**Klhl12**	kelch-like family member 12 (Klhl12), r	266772		13	56340908	56370203	29296	547,97	-0,20	7,46E-01
ENSRNOG00000003537	**Spta1**	spectrin, alpha, erythrocytic 1 (elliptoc	289257		13	96764163	96859811	95649	194,24	-0,34	7,46E-01
ENSRNOG00000004195	**Dtl**	Protein Dtl [Source:UniProtKB/TrEMB	305073		13	114785011	114821077	36067	156,20	0,24	7,46E-01
ENSRNOG00000005277	**Ptprv**	protein tyrosine phosphatase, recepto	64576		13	56986605	57006799	20195	40,12	0,30	7,47E-01
ENSRNOG00000025198		Protein Gas2l3 [Source:UniProtKB/Tr	680280		7	30065374	30084852	19479	158,99	0,23	7,47E-01
ENSRNOG00000016352	**Cbfa2t2**	core-binding factor, runt domain, alpha	296293		3	156308253	156410892	102640	737,44	0,18	7,47E-01
ENSRNOG00000001613	**Zbtb11**	zinc finger and BTB domain containing	304010		11	50261124	50295883	34760	811,82	-0,13	7,47E-01
ENSRNOG00000010342	**Zfand1**	Protein Zfand1; Putative uncharacteriz	361917		2	113412452	113422260	9809	178,02	-0,22	7,47E-01
ENSRNOG00000011667	**Fastk**	Fas-activated serine/threonine kinase	296741		4	7271939	7275953	4015	1526,80	0,23	7,47E-01
ENSRNOG00000011932	**Snrpa1**	small nuclear ribonucleoprotein polype	1E+08		1	128047739	128060800	13062	567,52	0,26	7,47E-01
ENSRNOG00000016874	**Zfp521**	zinc finger protein 521 (Zfp521), mRN	307579		18	5006330	5284770	278441	506,84	-0,15	7,47E-01
ENSRNOG00000017133	**LOC30676**	Putative monooxygenase p33MONOX	306766		17	12708595	12724572	15978	3817,59	0,14	7,47E-01
ENSRNOG00000018901	**Rab14**	RAB14, member RAS oncogene famil	94197		3	19693766	19704635	10870	2023,92	0,18	7,47E-01
ENSRNOG00000017222	**Fam175b**	family with sequence similarity 175, m	293570		1	211854578	211880154	25577	594,72	-0,19	7,47E-01
ENSRNOG00000000551	**RGD13055**	similar to RIKEN cDNA 2010107G23 (	294499		20	33529902	33531720	1819	679,20	0,21	7,47E-01
ENSRNOG00000002896	**Prdx6**	peroxiredoxin 6 (Prdx6), mRNA [Sourc	94167		13	83972034	83983136	11103	5982,53	-0,31	7,47E-01
ENSRNOG00000012454	**Trmt10b**	tRNA methyltransferase 10 homolog E	298081		5	65359591	65373925	14335	358,53	0,24	7,47E-01
ENSRNOG00000024703	**Dock5**	Protein Dock5 [Source:UniProtKB/TrE	305987		15	46969737	47143689	173953	30,61	0,31	7,47E-01
ENSRNOG00000028188		Glutathione S-transferase theta-2 [So	29487		20	15913940	15950591	36652	268,20	0,20	7,47E-01
ENSRNOG00000002035	**Paqr3**	progestin and adipoQ receptor family r	305203		14	14051459	14068707	17249	1000,84	0,22	7,47E-01
ENSRNOG00000012405	**Tcf4**	transcription factor 4 (Tcf4), mRNA [Sc	84382		18	64471371	64694030	222660	2328,76	-0,14	7,47E-01
ENSRNOG00000000244	**Coil**	coilin (Coil), mRNA [Source:RefSeq m	50998		10	73778176	73791920	13745	498,08	0,23	7,48E-01
ENSRNOG00000003401		Uncharacterized protein [Source:UniProtKB/TrE			10	12692337	12693262	926	19,57	0,32	7,48E-01
ENSRNOG00000013479	**Stard7**	StAR-related lipid transfer (START) dc	296128		3	126224026	126251262	27237	2207,48	0,22	7,48E-01
ENSRNOG00000013587	**Fam135a**	family with sequence similarity 135, m	367235		9	29080802	29160296	79495	581,68	-0,25	7,48E-01

ENSRNOG00000013604		glutathione peroxidase 4 (Gpx4), trans	29328	7	12686678	12688775	2098	6341,16	0,26	7,48E-01
ENSRNOG00000021945	**MGC11446**	similar to expressed sequence AI8360	500925	7	138898271	138899670	1400	1009,18	0,17	7,48E-01
ENSRNOG00000016242	**Fzd1**	frizzled family receptor 1 (Fzd1), mRN	58868	4	26377857	26382008	4152	1254,86	-0,20	7,48E-01
ENSRNOG00000011407	**Pragmin**	pragma of Rnd2 (Pragmin), mRNA [Sc	306506	16	59345052	59397741	52690	1385,73	-0,23	7,48E-01
ENSRNOG00000016034	**Rqcd1**	Rcd1 required for cell differentiation1 I	301513	9	81547682	81573447	25766	2036,18	0,18	7,48E-01
ENSRNOG00000016454	**Nasp**	nuclear autoantigenic sperm protein (I	298441	5	139243239	139268227	24989	1487,26	-0,27	7,48E-01
ENSRNOG00000018673	**Sec22b**	SEC22 vesicle trafficking protein homo	310710	2	219551811	219573833	22023	3067,36	0,21	7,48E-01
ENSRNOG00000020274	**Taf5**	TAF5 RNA polymerase II, TATA box bi	294018	1	274274891	274290376	15486	177,20	-0,21	7,48E-01
ENSRNOG00000020864	**Kirrel2**	kin of IRRE like 2 (Drosophila) (Kirrel2	1E+08	1	90066397	90075012	8616	20,08	-0,29	7,48E-01
ENSRNOG00000023320	**Tspan1**	tetraspanin 1 (Tspan1), mRNA [Source	298436	5	138803952	138808976	5025	25,73	0,32	7,48E-01
ENSRNOG00000004608	**Pam16**	presequence translocase-associated r	679907	10	9911717	9919294	7578	1336,93	0,30	7,49E-01
ENSRNOG00000004841	**Akap6**	A kinase (PRKA) anchor protein 6 (Ak	64553	6	83111482	83545576	434095	4086,82	0,15	7,49E-01
ENSRNOG00000008334		ciliary rootlet coiled-coil, rootletin (Cro	313663	5	163253158	163288260	35103	429,50	-0,19	7,49E-01
ENSRNOG00000009350	**Sez6**	seizure related 6 homolog (mouse) (S	192247	10	66710425	66759039	48615	9282,53	-0,18	7,49E-01
ENSRNOG00000015503	**Otud7a**	Protein Otud7a [Source:UniProtKB/Tr	309252	1	125738333	125766168	27836	355,32	-0,18	7,49E-01
ENSRNOG00000016622	**LOC10091(**	ankyrin repeat family A protein 2 [Sou	294679	X	82382242	82392794	10553	387,73	0,22	7,49E-01
ENSRNOG00000019875	**Matr3**	matrin 3 (Matr3), mRNA [Source:RefS	29150	18	28074392	28103826	29435	241,56	-0,31	7,49E-01
ENSRNOG00000032436	**Tmod3**	tropomodulin 3 (Tmod3), mRNA [Sour	300838	8	82035312	82095422	60111	638,19	-0,19	7,49E-01
ENSRNOG00000041989	**LOC100912162**			2	140798914	140798992	79	144,89	0,19	7,49E-01
ENSRNOG00000003052	**Cox18**	cytochrome c oxidase assembly protei	289522	14	19341084	19352707	11624	378,24	0,19	7,49E-01
ENSRNOG00000007457	**Serping1**	serpin peptidase inhibitor, clade G (C1	295703	3	78681826	78691120	9295	124,29	0,34	7,49E-01
ENSRNOG00000009068	**Phlda3**	pleckstrin homology-like domain, famil	363989	13	57637217	57640301	3085	1368,40	0,29	7,49E-01
ENSRNOG00000011170	**RGD13596**	similar to RIKEN cDNA 1700088E04 (	315126	7	120366954	120374272	7319	1020,02	0,15	7,49E-01
ENSRNOG00000014205	**Klf2**	Kruppel-like factor 2 (lung) (Klf2), mRN	306330	16	19084196	19086146	1951	62,44	0,22	7,49E-01
ENSRNOG00000017170	**Med4**	mediator complex subunit 4 (Med4), m	306030	15	59128754	59139026	10273	576,62	0,20	7,49E-01
ENSRNOG00000024452	**RGD15623**	Protein RGD1562390 [Source:UniPro	307797	19	48519805	48531985	12181	837,34	0,18	7,49E-01
ENSRNOG00000026690	**Kin**	antigenic determinant of rec-A protein	689197	17	73884594	73894936	10343	119,11	-0,27	7,49E-01
ENSRNOG00000046002	**LOC30112**	uncharacterized protein LOC301124 [	301124	9	9443626	9446493	2868	4598,83	0,27	7,49E-01
ENSRNOG00000049507	**Sept10**	septin 10 (Sept10), mRNA [Source:Re	309891	20	30530111	30621223	91113	317,29	-0,24	7,49E-01
ENSRNOG00000050796		Uncharacterized protein [Source:UniProtKB/TrE		17	5950432	5950815	384	40,95	-0,27	7,49E-01
ENSRNOG00000010170	**Tubb4b**	tubulin, beta 4B class IVb (Tubb4b), m	296554	3	2422872	2425633	2762	5145,62	0,18	7,49E-01
ENSRNOG00000018279	**Sfxn1**	sideroflexin 1 (Sfxn1), mRNA [Source:	364678	17	13157817	13193253	35437	2956,86	-0,16	7,49E-01
ENSRNOG00000003341	**Med9**	mediator complex subunit 9 (Med9), m	497914	10	46071310	46086080	14771	656,45	-0,19	7,49E-01
ENSRNOG00000004727	**Nup37**	nucleoporin 37 (Nup37), mRNA [Sourc	299706	7	28825302	28853301	28000	232,85	-0,23	7,49E-01
ENSRNOG00000010316	**Magohb**	mago-nashi homolog B (Drosophila) (I	690303	4	228076684	228084406	7723	190,13	0,18	7,49E-01
ENSRNOG00000011420	**Mtmr7**	myotubularin related protein 7 (Mtmr7	306490	16	54469056	54560062	91007	897,32	0,22	7,49E-01
ENSRNOG00000015794	**Fam83d**	family with sequence similarity 83, me	311598	3	160322831	160342099	19269	300,44	0,23	7,49E-01
ENSRNOG00000021246	**Cenpb**		362217	3	130219464	130221882	2419	3005,44	0,21	7,49E-01

ENSRNOG00000013669		Phosphoinositide 3-kinase regulatory s	363131	8	113675822	113725967	50146	1148,11	0,20	7,49E-01
ENSRNOG00000004311	**Gpr182**	G protein-coupled receptor 182 (Gpr18	29307	7	71218212	71221052	2841	64,41	0,25	7,50E-01
ENSRNOG00000006873	**Dnase1**	deoxyribonuclease I (Dnase1), mRNA	25633	10	10512149	10515087	2939	20,40	0,29	7,50E-01
ENSRNOG00000009085	**Prkag2**	protein kinase, AMP-activated, gamma	373545	4	6770353	6816799	46447	1729,19	0,24	7,50E-01
ENSRNOG00000010258	**Vhl**	von Hippel-Lindau tumor suppressor, E	24874	4	208877266	208884234	6969	3303,32	0,22	7,50E-01
ENSRNOG00000014431				1	6889413	6889973	561	22,47	0,29	7,50E-01
ENSRNOG00000020333	**LOC28995**	cirrhosis, autosomal recessive 1A (cirh	291987	19	49952333	49980521	28189	875,05	0,19	7,50E-01
ENSRNOG00000007806	**Arf5**	ADP-ribosylation factor 5 (Arf5), mRN	79117	4	55463191	55466117	2927	8163,84	0,25	7,50E-01
ENSRNOG00000009344		Protein Syt16 [Source:UniProtKB/TrE	299142	6	106506534	106629182	122649	682,64	-0,18	7,50E-01
ENSRNOG00000015118	**Cpped1**	calcineurin-like phosphoesterase dom	302890	10	2523592	2643424	119833	127,61	-0,29	7,50E-01
ENSRNOG00000016698		mRNA cap guanine-N7 methyltransfer	291534	18	63299070	63322520	23451	1023,01	-0,25	7,50E-01
ENSRNOG00000018741		Protein Zfp318 [Source:UniProtKB/Tr	685082	9	15839403	15875058	35656	1921,73	-0,14	7,50E-01
ENSRNOG00000018836	**RGD15598**	similar to RIKEN cDNA 2310022B05 (	498967	19	68101910	68127030	25121	6227,70	-0,22	7,50E-01
ENSRNOG00000019766	**Exoc8**	exocyst complex component 8 (Exoc8	245709	19	68360729	68362879	2151	764,66	-0,17	7,50E-01
ENSRNOG00000003812	**Nlgn3**	neuroligin 3 (Nlgn3), mRNA [Source:R	171297	X	72051994	72074002	22009	4421,00	-0,15	7,50E-01
ENSRNOG00000050016	**Gtf3a**	general transcription factor III A (Gtf3a	246299	12	11968597	11976230	7634	871,05	0,16	7,50E-01
ENSRNOG00000049496	**Trim62**	Protein Trim62; Similar to tripartite mo	313045	5	150800021	150826717	26697	167,87	-0,24	7,51E-01
ENSRNOG00000003269	**Atp6v0e1**	ATPase, H+ transporting, lysosomal, V	94170	10	16661968	16685040	23073	951,70	0,23	7,51E-01
ENSRNOG00000020746	**Lin7b**	lin-7 homolog b (C. elegans) (Lin7b), r	60377	1	102422048	102424623	2576	282,62	-0,31	7,51E-01
ENSRNOG00000040279	**Ankrd32**	ankyrin repeat domain 32 (Ankrd32), r	294601	2	4092109	4193740	101632	272,21	-0,27	7,51E-01
ENSRNOG00000019943	**Slc7a6**	solute carrier family 7 (amino acid tran	307811	19	49029315	49045949	16635	898,24	0,23	7,52E-01
ENSRNOG00000002275	**Fip1l1**	factor interacting with PAPOLA and CF	289582	14	35976772	36034611	57840	2434,39	0,16	7,52E-01
ENSRNOG00000043105	**Lyrm2**	LYR motif containing 2 (Lyrm2), mRNA	690354	5	52635558	52637828	2271	683,94	0,17	7,52E-01
ENSRNOG00000003258	**Cdc73**	cell division cycle 73 (Cdc73), mRNA [	304832	13	65392579	65485175	92597	378,09	-0,16	7,52E-01
ENSRNOG00000007795				7	114865953	114867015	1063	49,59	-0,32	7,52E-01
ENSRNOG00000013794	**Rbp1**	retinol binding protein 1, cellular (Rbp1	25056	8	105891148	105912684	21537	97,37	0,27	7,52E-01
ENSRNOG00000006170	**Bach2**	similar to BTB and CNC homology 1, b	313125	5	52151470	52410900	259431	856,14	0,22	7,52E-01
ENSRNOG00000015913	**Tspan5**	tetraspanin 5 (Tspan5), mRNA [Source	362048	2	262587481	262753104	165624	3385,31	-0,16	7,52E-01
ENSRNOG00000001424		Homeobox protein cut-like 1 [Source:	116639	12	25151530	25469669	318140	1273,02	0,13	7,52E-01
ENSRNOG00000012149	**Gpsm2**	G-protein signaling modulator 2 (Gpsm	362021	2	230953071	230992673	39603	429,74	-0,21	7,52E-01
ENSRNOG00000012927	**Rrp9**	ribosomal RNA processing 9, small su	363134	8	114544186	114552838	8653	1090,15	0,21	7,52E-01
ENSRNOG00000050072	**Jmjd7**	jumonji domain containing 7 (Jmjd7), r	1E+08	3	118366787	118372848	6062	61,73	0,22	7,52E-01
ENSRNOG00000026180		LETM1 domain-containing protein LET	361169	16	70902112	70921166	19055	172,88	0,28	7,53E-01
ENSRNOG00000000082	**Hltf**	helicase-like transcription factor (Hltf),	295568	2	124574548	124634236	59689	307,88	-0,31	7,53E-01
ENSRNOG00000000833	**Vars2**	valyl-tRNA synthetase 2, mitochondria	309596	20	5685498	5696344	10847	671,79	0,13	7,53E-01
ENSRNOG00000001877	**Med15**	mediator complex subunit 15 (Med15)	360743	11	90606490	90681124	74635	1411,76	-0,15	7,53E-01
ENSRNOG00000010420	**Shfm1**	split hand/foot malformation (ectrodac	680532	4	31935396	31955552	20157	3346,29	0,23	7,53E-01
ENSRNOG00000017849		amyloid beta (A4) precursor protein-bi	117026	18	29202937	29210069	7133	802,12	0,22	7,53E-01

ENSRNOG00000018358	**Nt5dc2**	5'-nucleotidase domain containing 2 (N	290558	16	7141051	7149109	8059	1230,06	-0,26	7,53E-01
ENSRNOG00000019823	**LOC10091**	reactive oxygen species modulator 1 (	679572	3	158052831	158054431	1601	743,70	0,29	7,53E-01
ENSRNOG00000022260	**Senp1**	Protein Senp1 [Source:UniProtKB/TrE	300193	7	138765842	138811222	45381	567,33	-0,16	7,53E-01
ENSRNOG00000027742	**Adamtsl2**	Protein Adamtsl2 [Source:UniProtKB/	311827	3	10988786	11016680	27895	62,25	-0,26	7,53E-01
ENSRNOG00000029456	**Rp9**	retinitis pigmentosa 9 (human) (Rp9),	363032	8	23502288	23522452	20165	204,17	0,19	7,53E-01
ENSRNOG00000007850	**Mok**	MOK protein kinase (Mok), mRNA [So	362787	6	145154362	145185192	30831	308,93	0,19	7,53E-01
ENSRNOG00000008469	**Mettl5**	methyltransferase like 5 (Mettl5), mRN	502632	3	62736608	62747462	10855	341,95	-0,22	7,53E-01
ENSRNOG00000024207	**Fgfrl1**	fibroblast growth factor receptor-like 1	360903	14	2015651	2027710	12060	969,29	0,26	7,53E-01
ENSRNOG00000010743	**Fdxacb1**	ferredoxin-fold anticodon binding dom	315646	8	53793837	53798266	4430	102,33	-0,23	7,53E-01
ENSRNOG00000014970	**RGD15625**	Protein RGD1562511 [Source:UniPro	500571	5	160377365	160414628	37264	74,50	-0,24	7,53E-01
ENSRNOG00000016923	**Clptm1l**	CLPTM1-like (Clptm1l), mRNA [Sourc	316916	1	33706075	33722058	15984	2809,08	0,17	7,53E-01
ENSRNOG00000017967	**Asb13**	ankyrin repeat and SOCS box-contain	361268	17	71918385	71937087	18703	343,45	0,24	7,53E-01
ENSRNOG00000019935	**Armc5**	armadillo repeat containing 5 (Armc5),	361653	1	206701594	206708360	6767	1072,40	0,19	7,53E-01
ENSRNOG00000004442	**RGD13117**	uncharacterized protein LOC362769		362769	6	134008151	134091103	82953	162,30	-0,25	7,53E-01
ENSRNOG00000005190	**Nipal2**	NIPA-like domain containing 2 (Nipal2	362899	7	73513633	73624863	111231	48,89	0,29	7,53E-01
ENSRNOG00000005573	**Ntn4**	netrin 4 (Ntn4), mRNA [Source:RefSe	299737	7	34599083	34705852	106770	625,40	0,24	7,53E-01
ENSRNOG00000011310		phosphodiesterase 10A (Pde10a), mR	63885	1	53617430	53846125	228696	1540,28	0,24	7,53E-01
ENSRNOG00000011382	**Wdr33**	WD repeat domain 33 (Wdr33), mRNA	307524	18	24302098	24403929	101832	1886,02	0,17	7,53E-01
ENSRNOG00000014311	**Slc7a8**	solute carrier family 7 (amino acid tran	84551	15	37253915	37317161	63247	946,33	0,26	7,53E-01
ENSRNOG00000014832	**Mapkapk3**	mitogen-activated protein kinase-activ	315994	8	115368435	115402229	33795	590,37	-0,29	7,53E-01
ENSRNOG00000016952	**LOC68644**	ubiquinol-cytochrome c reductase, cor	686442	7	12341264	12345890	4627	5661,23	0,25	7,53E-01
ENSRNOG00000017523	**H6pd**	hexose-6-phosphate dehydrogenase (	298655	5	170640289	170671849	31561	172,86	-0,18	7,53E-01
ENSRNOG00000024605	**Fam220a**	family with sequence similarity 220, m	498145	12	15088874	15089653	780	820,88	0,16	7,53E-01
ENSRNOG00000031766		cytochrome b (mitochondrion) [Sourc	26192	MT	14136	15278	1143	227261,39	-0,18	7,53E-01
ENSRNOG00000033560	**Polr3c**	polymerase (RNA) III (DNA directed) p	310685	2	218323219	218339095	15877	703,17	0,18	7,53E-01
ENSRNOG00000033581		tenascin XB [Source:MGI Symbol;Acc:MGI:1932		20	6547861	6565849	17989	37,16	-0,33	7,53E-01
ENSRNOG00000037851	**Spidr**	scaffolding protein involved in DNA rep	498119	11	92071681	92313960	242280	124,41	-0,22	7,53E-01
ENSRNOG00000017879	**Gab1**	GRB2-associated binding protein 1 (G	361388	19	41699636	41808268	108633	1984,78	-0,21	7,53E-01
ENSRNOG00000002959	**Shroom4**	shroom family member 4 (Shroom4), r	317391	X	17504314	17711666	207353	62,17	0,20	7,54E-01
ENSRNOG00000011340	**Ankrd13c**	ankyrin repeat domain 13C (Ankrd13c	685374	2	283426167	283473209	47043	547,01	0,22	7,54E-01
ENSRNOG00000012726	**Zbtb5**	zinc finger and BTB domain containing	298084	5	65047763	65067167	19405	1551,78	0,20	7,54E-01
ENSRNOG00000000151	**Ldlrap1**	low density lipoprotein receptor adapto	500564	5	156742863	156765855	22993	29,05	-0,31	7,54E-01
ENSRNOG00000007271	**Map3k9**	mitogen-activated protein kinase kinas	500690	6	113606221	113664859	58639	1221,45	0,21	7,54E-01
ENSRNOG00000011797	**Mocs2**	molybdenum cofactor synthesis 2 (Mo	294753	2	71520174	71532081	11908	582,41	-0,19	7,54E-01
ENSRNOG00000012866	**Nek8**	NIMA-related kinase 8 (Nek8), mRNA	287473	10	66197474	66208846	11373	99,11	-0,21	7,54E-01
ENSRNOG00000012873	**Gfm1**	G elongation factor, mitochondrial 1 (G	114017	2	183950902	183995881	44980	972,49	-0,18	7,54E-01
ENSRNOG00000047963		Uncharacterized protein [Source:UniProtKB/TrE		8	95694193	95697728	3536	87,66	-0,25	7,54E-01
ENSRNOG00000050057		Abca3 protein; Uncharacterized protein [Source		10	13539869	13555079	15211	531,55	0,17	7,54E-01

ENSRNOG00000003515	**Ephx1**	epoxide hydrolase 1, microsomal (xen	25315	13	104268708	104343633	74926	1061,09	0,19	7,54E-01
ENSRNOG00000004282	**Arl4a**	ADP-ribosylation factor-like 4A (Arl4a)	29308	6	69536689	69538783	2095	643,94	-0,27	7,54E-01
ENSRNOG00000002565		TAF1 RNA polymerase II, TATA box bi	317256	X	72261638	72335791	74154	1284,71	0,25	7,54E-01
ENSRNOG00000004581	**Zdhhc9**	zinc finger, DHHC-type containing 9 (Z	302808	X	135081380	135116885	35506	2864,55	0,19	7,54E-01
ENSRNOG00000006639	**Scn9a**	sodium channel, voltage-gated, type I	78956	3	59207262	59286961	79700	48,24	-0,29	7,54E-01
ENSRNOG00000011222	**LOC10036**	dynein light chain LC8-type 1 (Dynll1),	58945	12	48867516	48869888	2373	4763,75	-0,22	7,54E-01
ENSRNOG00000013009	**Ldha**	lactate dehydrogenase A (Ldha), mRN	24533	1	103975207	103984632	9426	2355,75	0,17	7,54E-01
ENSRNOG00000016008		Uncharacterized protein [Source:UniProtKB/TrE		18	80216468	80255882	39415	36,09	0,26	7,54E-01
ENSRNOG00000020076	**RGD13117**	similar to RIKEN cDNA 2010012O05 (	294012	1	273881051	273894737	13687	1322,77	0,16	7,54E-01
ENSRNOG00000021780	**Rad51d**	RAD51 paralog D (Rad51d), mRNA [S	303375	10	69858340	69872417	14078	186,01	-0,16	7,54E-01
ENSRNOG00000000525	**Pi16**	peptidase inhibitor 16 (Pi16), mRNA [S	294312	20	9163205	9172499	9295	162,01	-0,27	7,55E-01
ENSRNOG00000002185		phosphoglucomutase-2 [Source:RefS	289632	14	45635119	45669788	34670	650,26	0,22	7,55E-01
ENSRNOG00000007227	**Mien1**	migration and invasion enhancer 1 (Mi	360617	10	86187295	86189073	1779	293,47	0,21	7,55E-01
ENSRNOG00000009219		hepatocyte cell adhesion molecule [Source:MGI		8	39849601	39866016	16416	1302,19	0,20	7,55E-01
ENSRNOG00000001710	**Abcf3**	ATP-binding cassette, subfamily F (GC	287982	11	87098519	87110250	11732	2164,10	0,17	7,55E-01
ENSRNOG00000000340	**RGD13047**	uncharacterized protein LOC302247		302247	15	8973717	8984672	10956	3333,37	0,22	7,55E-01
ENSRNOG00000002467	**Pgk1**	phosphoglycerate kinase 1 (Pgk1), mF	24644	X	56383382	56399430	16049	4399,97	0,18	7,55E-01
ENSRNOG00000007108	**Bmp7**	bone morphogenetic protein 7 (Bmp7)	85272	3	176945362	177020736	75375	270,91	-0,26	7,55E-01
ENSRNOG00000009112	**Pon2**	paraoxonase 2 (Pon2), mRNA [Source	296851	4	30251593	30287330	35738	2032,15	-0,27	7,55E-01
ENSRNOG00000009653		numb gene homolog (Drosophila) [Source:MGI S		6	118218525	118262951	44427	347,17	-0,17	7,55E-01
ENSRNOG00000013658	**Nefl**	neurofilament, light polypeptide (Nefl),	83613	15	46793526	46797403	3878	2461,46	0,28	7,55E-01
ENSRNOG00000014178	**Acad9**	acyl-CoA dehydrogenase family, mem	294973	2	142407990	142431623	23634	1433,72	0,18	7,55E-01
ENSRNOG00000018376		testis-specific gene 10 protein [Source	252923	9	44058082	44154707	96626	197,20	-0,27	7,55E-01
ENSRNOG00000020660	**LOC68843**	cofilin 1, non-muscle (Cfl1), mRNA [So	29271	1	227799060	227802655	3596	36280,05	0,21	7,55E-01
ENSRNOG00000023520	**Cs**	citrate synthase (Cs), mRNA [Source:I	170587	7	2731053	2757519	26467	7863,10	0,18	7,55E-01
ENSRNOG00000024178	**Cenpt**	centromere protein T (Cenpt), mRNA [	307805	19	48680056	48686495	6440	171,99	0,24	7,55E-01
ENSRNOG00000045649	**Arrdc3**	arrestin domain containing 3 (Arrdc3),	309945	2	8617992	8627696	9705	429,89	-0,31	7,55E-01
ENSRNOG00000049662	**LOC10090**	zinc finger protein 708 [Source:MGI Sy	1E+08	2	106142139	106147092	4954	44,12	-0,32	7,55E-01
ENSRNOG00000023991	**Rab20**	RAB20, member RAS oncogene famil	689377	16	82824911	82848168	23258	17,77	0,33	7,55E-01
ENSRNOG00000027596		methyl-CpG binding domain protein 5 [Source:M		3	38600058	38662936	62879	924,06	-0,18	7,55E-01
ENSRNOG00000042059	**Rccd1**	Protein Rccd1 [Source:UniProtKB/TrE	308760	1	143061512	143066894	5383	52,04	-0,22	7,55E-01
ENSRNOG00000001782	**Osbpl11**	oxysterol binding protein-like 11 (Osbp	303888	11	73855648	73918639	62992	1133,56	-0,18	7,55E-01
ENSRNOG00000006622	**Cry1**	cryptochrome 1 (photolyase-like) (Cry	299691	7	24683890	24784328	100439	505,21	-0,14	7,55E-01
ENSRNOG00000031222		Protein RGD1561327 [Source:UniPro	317593	X	144471634	144472890	1257	47,83	0,28	7,55E-01
ENSRNOG00000032070		dynein cytoplasmic 2 heavy chain 1 [Source:MG		8	5224356	5436622	212267	521,65	0,30	7,55E-01
ENSRNOG00000020059	**Pcdhb14**	Protein Pcdhb14 [Source:UniProtKB/	291647	18	30234900	30237293	2394	103,09	-0,19	7,56E-01
ENSRNOG00000009867	**Tgfb3**	transforming growth factor, beta 3 (Tgf	25717	6	119222052	119243828	21777	131,21	-0,22	7,56E-01
ENSRNOG00000012494	**Kctd14**	Protein Kctd14 [Source:UniProtKB/Tr	308836	1	168624638	168630653	6016	56,76	0,22	7,56E-01

ENSRNOG00000012782	**Tmem2**	transmembrane protein 2 (Tmem2), m	309400		1	246700907	246762954	62048	916,13	0,19	7,56E-01
ENSRNOG00000014526	**Cars2**	cysteinyl-tRNA synthetase 2, mitochor	361184		16	82756705	82792874	36170	545,81	0,14	7,56E-01
ENSRNOG00000016550	**Dclk2**	doublecortin-like kinase 2 (Dclk2), trar	310698		2	205516097	205643934	127838	4865,19	0,12	7,56E-01
ENSRNOG00000021289	**Rbbp5**	retinoblastoma binding protein 5 (Rbb|	304794		13	54325701	54352552	26852	1295,06	0,19	7,56E-01
ENSRNOG00000002751	**Zdhhc15**	zinc finger, DHHC-type containing 15 (	317235	X		76242145	76364606	122462	102,82	-0,23	7,56E-01
ENSRNOG00000000614	**Bicc1**	bicaudal C homolog 1 (Drosophila) (Bi	361832		20	20939898	21092147	152250	120,01	-0,27	7,56E-01
ENSRNOG00000032608	**Bcdin3d**	BCDIN3 domain containing (Bcdin3d),	363001	X		115635413	115639979	4567	214,47	0,23	7,56E-01
ENSRNOG00000045770	**LOC10091**	HIR histone cell cycle regulation defec	363849		11	89270162	89378396	108235	227,58	0,20	7,57E-01
ENSRNOG00000007912		chromogranin A [Source:MGI Symbol;Acc:MGI:8			6	135645155	135656039	10885	1407,88	-0,18	7,57E-01
ENSRNOG00000019178	**Taf10**	TAF10 RNA polymerase II, TATA box b	293345		1	177591012	177592280	1269	1103,96	0,29	7,57E-01
ENSRNOG00000003952	**Map3k19**	Protein Map3k19 [Source:UniProtKB/	289001		13	49609133	49633399	24267	27,58	-0,30	7,57E-01
ENSRNOG00000019394	**Mpv17l2**	MPV17 mitochondrial membrane prote	290645		16	20289807	20292193	2387	1255,40	0,25	7,57E-01
ENSRNOG00000019489	**RGD13052**	similar to RIKEN cDNA 1700052N19 (	292267		1	42325061	42348565	23505	474,11	0,17	7,57E-01
ENSRNOG00000021079	**Fxyd1**	FXYD domain-containing ion transport	58971		1	90639293	90642454	3162	331,29	-0,32	7,57E-01
ENSRNOG00000039666	**Srpx**	sushi-repeat-containing protein, X-link	64316	X		14932359	15003055	70697	39,86	0,24	7,57E-01
ENSRNOG00000046098		Uncharacterized protein [Source:UniProtKB/TrE			18	85110490	85151163	40674	38,21	0,24	7,57E-01
ENSRNOG00000018916	**Bcl7c**	B-cell CLL/lymphoma 7C (Bcl7c), mRN	293514		1	206178926	206182756	3831	852,02	0,26	7,57E-01
ENSRNOG00000010217	**Prrc2b**	Protein Prrc2b [Source:UniProtKB/TrE	296637		3	16556389	16597431	41043	12242,97	0,15	7,57E-01
ENSRNOG00000019053	**Bcap31**	B-cell receptor-associated protein 31 (	293852		1	152845255	152875711	30457	1038,06	0,16	7,57E-01
ENSRNOG00000037376	**Mterfd2**	MTERF domain containing 2 (Mterfd2)	363289		9	100096825	100101536	4712	183,68	0,20	7,57E-01
ENSRNOG00000032919	**Zfp105**	zinc finger protein 105 (Zfp105), mRN	316096		8	131156987	131164782	7796	215,74	-0,19	7,57E-01
ENSRNOG00000013767	**Ccdc61**	coiled-coil domain containing 61 (Ccd	292680		1	81057905	81077772	19868	248,29	0,18	7,57E-01
ENSRNOG00000020119	**Pcdhac1**	protocadherin alpha subfamily C, 1 (P	393091		18	29810769	29924444	113676	1117,55	-0,19	7,57E-01
ENSRNOG00000050675	**Myl4**	myosin, light chain 4 (Myl4), mRNA [S	688228		10	92390125	92398028	7904	38,98	0,23	7,57E-01
ENSRNOG00000017122	**Pacrg**	Park2 co-regulated (Pacrg), mRNA [S	499021		1	49266490	49625133	358644	88,44	-0,23	7,58E-01
ENSRNOG00000007720	**Rnf26**	ring finger protein 26 (Rnf26), mRNA [	300659		8	47063866	47065140	1275	615,63	-0,20	7,58E-01
ENSRNOG00000008829	**Sorbs3**	sorbin and SH3 domain containing 3 (	282843		15	55585616	55616959	31344	711,92	-0,27	7,58E-01
ENSRNOG00000026842	**Nnt**	nicotinamide nucleotide transhydroger	310378		2	70553345	70644277	90933	1608,06	-0,20	7,58E-01
ENSRNOG00000003443	**Klf1**	Kruppel-like factor 1 (erythroid) (Klf1),	304666		19	36992005	36995176	3172	53,09	-0,27	7,58E-01
ENSRNOG00000010100		tRNA-yW synthesizing protein 5 (Tyw5	301419		9	63883692	63901784	18093	134,09	-0,20	7,58E-01
ENSRNOG00000013683	**S1pr1**	sphingosine-1-phosphate receptor 1 (S	29733		2	236742193	236746400	4208	3404,49	-0,22	7,58E-01
ENSRNOG00000050960	**Atg4a**	autophagy related 4A, cysteine peptid	678769	X		110858648	110865113	6466	90,98	-0,25	7,58E-01
ENSRNOG00000015696	**Cdk5rap1**	CDK5 regulatory subunit associated p	252827		3	155983803	156241584	257782	367,75	0,20	7,58E-01
ENSRNOG00000000395	**LOC60629**	RIKEN cDNA 2510003E04 gene [Sou	606294		20	33976300	33995859	19560	3136,02	0,21	7,59E-01
ENSRNOG00000014659	**Ebpl**	emopamil binding protein-like (Ebpl), r	361054		15	45472249	45495637	23389	175,31	-0,28	7,59E-01
ENSRNOG00000014664	**RGD15599**	similar to mKIAA1429 protein (RGD15	313061		5	29771165	29841000	69836	1247,87	0,17	7,59E-01
ENSRNOG00000016670		phosphatidylinositol-5-phosphate 4-kinase, type			17	87260014	87463891	203878	851,72	-0,15	7,59E-01
ENSRNOG00000016791		choline kinase alpha (Chka), mRNA [S	29194		1	225950376	225998825	48450	2008,48	-0,13	7,59E-01

ENSRNOG00000020007	**LOC10091**	erythrocyte membrane protein band 4.	59317	3	159078047	159139766	61720	858,20	-0,16	7,59E-01
ENSRNOG00000020539	**Muc1**	mucin 1, cell surface associated (Muc	24571	2	207957655	207962391	4737	64,81	0,28	7,59E-01
ENSRNOG00000022686		Palmitoyltransferase ZDHHC2 [Sourc	246326	16	54635195	54704713	69519	1806,32	-0,17	7,59E-01
ENSRNOG00000026907	**Zbtb37**	zinc finger and BTB domain containing	304918	13	83731914	83747221	15308	55,13	-0,29	7,59E-01
ENSRNOG00000028215		nuclear pore complex protein Nup160	311182	3	86204592	86267431	62840	426,69	-0,27	7,59E-01
ENSRNOG00000029535	**Nrbp2**	nuclear receptor binding protein 2 (Nrt	680451	7	117131632	117137365	5734	3071,63	-0,20	7,59E-01
ENSRNOG00000037211		kinesin family member 14 [Source:MGI Symbol;/		13	58401865	58464821	62957	125,63	0,29	7,59E-01
ENSRNOG00000005149	**Sacm1l**	SAC1 suppressor of actin mutations 1	116482	8	131759866	131816202	56337	603,39	-0,22	7,59E-01
ENSRNOG00000015220	**Crtac1**	cartilage acidic protein 1 (Crtac1), mR	171438	1	268979488	269121995	142508	826,82	-0,18	7,59E-01
ENSRNOG00000000054	**Abhd8**	abhydrolase domain containing 8 (Abh	306338	16	19727394	19734131	6738	4428,18	0,26	7,59E-01
ENSRNOG00000010576		potassium voltage-gated channel, sub	298496	5	143423766	143472408	48643	142,11	-0,21	7,59E-01
ENSRNOG00000028549	**Capza2**	capping protein (actin filament) muscle	493810	4	45541352	45577239	35888	2720,90	-0,16	7,60E-01
ENSRNOG00000011475	**Srcin1**	SRC kinase signaling inhibitor 1 (Srcir	56029	10	85248782	85306184	57403	3633,44	0,15	7,60E-01
ENSRNOG00000014316		Rap guanine nucleotide exchange factor (GEF)		3	13580866	13648176	67311	4046,95	0,14	7,60E-01
ENSRNOG00000026813		Uncharacterized protein [Source:UniProtKB/TrE		20	28969826	28970344	519	38,30	-0,27	7,60E-01
ENSRNOG00000007025	**Evc2**	Ellis van Creveld syndrome 2 (Evc2), i	289711	14	78103484	78185967	82484	159,06	-0,26	7,60E-01
ENSRNOG00000008546	**Mrpl35**	mitochondrial ribosomal protein L35 (M	297334	4	164516933	164524701	7769	718,23	0,18	7,60E-01
ENSRNOG00000013177	**Map3k1**	mitogen activated protein kinase kinas	116667	2	62375910	62439605	63696	65,88	-0,21	7,60E-01
ENSRNOG00000026958	**LOC50007**	similar to RIKEN cDNA 3110062M04 (	500077	4	62123742	62128007	4266	154,92	0,21	7,60E-01
ENSRNOG00000004604	**Slc38a10**	Protein Slc38a10; Putative uncharacte	303740	10	108883444	108931638	48195	1714,48	0,19	7,60E-01
ENSRNOG00000007089	**Lgmn**	legumain (Lgmn), mRNA [Source:RefS	63865	6	135492944	135518919	25976	1137,45	-0,27	7,60E-01
ENSRNOG00000021517	**Tmem231**	transmembrane protein 231 (Tmem23	361410	19	54946388	54965717	19330	187,23	0,16	7,60E-01
ENSRNOG00000010643		KN motif and ankyrin repeat domains 1	1E+08	8	22848264	22872959	24696	347,33	-0,27	7,60E-01
ENSRNOG00000024061		retinoic acid receptor, beta (Rarb), mR	24706	15	13963828	14306790	342963	48,53	0,23	7,61E-01
ENSRNOG00000048487		solute carrier family 12 (potassium/chloride trans		12	24449475	24465532	16058	813,12	-0,24	7,61E-01
ENSRNOG00000001803	**Dnajb11**	DnaJ (Hsp40) homolog, subfamily B, r	360734	11	84829172	84845635	16464	1783,69	0,13	7,61E-01
ENSRNOG00000025371		sprouty homolog 1, antagonist of FGF	294981	2	144010431	144015083	4653	336,19	-0,15	7,61E-01
ENSRNOG00000000481		cutA divalent cation tolerance homolog	294288	20	7591037	7592613	1577	1629,71	0,22	7,61E-01
ENSRNOG00000001818		FYVE, RhoGEF and PH domain-conta	246174	11	91755139	91850043	94905	465,81	-0,28	7,61E-01
ENSRNOG00000002877		HECT, UBA and WWE domain containing 1 [Sou		X	21848516	21950685	102170	10725,54	0,12	7,61E-01
ENSRNOG00000011908		additional sex combs like 2 (Drosophil	313922	6	37646074	37728356	82283	349,11	0,17	7,61E-01
ENSRNOG00000012325	**Adk**	adenosine kinase (Adk), mRNA [Sourc	25368	15	3011936	3414198	402263	1026,60	-0,13	7,61E-01
ENSRNOG00000016163	**Slc1a3**	solute carrier family 1 (glial high affinity	29483	2	79348445	79422645	74201	30782,19	-0,20	7,61E-01
ENSRNOG00000038489	**Sumo3**	small ubiquitin-like modifier 3 (Sumo3)	499417	20	13892796	13903349	10554	3215,75	0,14	7,61E-01
ENSRNOG00000001201	**Cstb**	cystatin B (stefin B) (Cstb), mRNA [So	25308	20	13137897	13139938	2042	1943,49	0,22	7,61E-01
ENSRNOG00000015417	**Kansl3**	KAT8 regulatory NSL complex subunit	316328	9	42553537	42597031	43495	2701,97	0,18	7,61E-01
ENSRNOG00000017925		GTPase activating protein and VPS9 c	311880	3	19073588	19123578	49991	3188,56	-0,13	7,61E-01
ENSRNOG00000019387	**Ifi30**	interferon gamma inducible protein 30	290644	16	20283634	20287840	4207	346,74	-0,26	7,61E-01

ENSRNOG00000042126	**Lsm5**	LSM5 homolog, U6 small nuclear RNA	306222	4	151668265	151671481	3217	500,96	0,23	7,61E-01
ENSRNOG00000019693		caseinolytic peptidase B protein homo	65041	1	172929521	173055807	126287	986,22	-0,15	7,61E-01
ENSRNOG00000016675		potassium intermediate/small conduct	54262	18	38985930	39129190	143261	321,53	-0,25	7,62E-01
ENSRNOG00000016962	**Neu2**	sialidase 2 (Neu2), mRNA [Source:Re	29204	9	94440842	94444057	3216	30,66	0,25	7,62E-01
ENSRNOG00000028090	**Arhgef18**	rho/rac guanine nucleotide exchange f	304193	12	4056174	4161173	105000	1138,74	0,12	7,62E-01
ENSRNOG00000008689		Protein Cbx1; RCG35120, isoform CR	360609	10	84462829	84470096	7268	910,78	0,22	7,62E-01
ENSRNOG00000010477	**Pomt1**	protein-O-mannosyltransferase 1 (Por	84430	3	16602896	16620742	17847	569,57	0,17	7,62E-01
ENSRNOG00000017210	**Slc22a23**	solute carrier family 22, member 23 (S	64559	17	33132227	33298110	165884	3958,46	-0,13	7,62E-01
ENSRNOG00000026517	**Vrk3**	vaccinia related kinase 3 (Vrk3), mRN	361565	1	101820326	101847648	27323	920,41	0,18	7,62E-01
ENSRNOG00000003247	**Sde2**	SDE2 telomere maintenance homolog	289315	13	104135522	104151782	16261	1317,49	0,18	7,62E-01
ENSRNOG00000003821	**Rptor**	regulatory associated protein of MTOF	287871	10	108353697	108658916	305220	1857,51	0,16	7,62E-01
ENSRNOG00000015230		RGD1309374 protein; Uncharacterized protein		1	58165870	58171909	6040	58,31	-0,25	7,62E-01
ENSRNOG00000015987	**Rap1gds1**	RAP1, GTP-GDP dissociation stimulat	310909	2	262787596	262899803	112208	3709,20	-0,18	7,62E-01
ENSRNOG00000016829	**Isoc2b**	isochorismatase domain containing 2b	361501	1	75991121	76011769	20649	131,29	0,26	7,62E-01
ENSRNOG00000017702	**Gpld1**	glycosylphosphatidylinositol specific p	291132	17	43952825	43995477	42653	142,10	-0,28	7,62E-01
ENSRNOG00000047307	**Cntfr**	ciliary neurotrophic factor receptor (Cr	313173	5	62566649	62594369	27721	1680,49	-0,19	7,62E-01
ENSRNOG00000007048	**Rap1b**	RAP1B, member of RAS oncogene fa	171337	7	60851045	60860980	9936	1580,92	-0,25	7,62E-01
ENSRNOG00000014762	**Ppie**	peptidylprolyl isomerase E (cyclophilin	298508	5	144615833	144625430	9598	726,23	0,18	7,62E-01
ENSRNOG00000019372	**Pc**	pyruvate carboxylase (Pc), mRNA [So	25104	1	226626841	226726328	99488	1670,48	0,20	7,62E-01
ENSRNOG00000011830	**Plekha3**	pleckstrin homology domain-containin	295674	3	70109902	70131097	21196	563,92	0,14	7,62E-01
ENSRNOG00000017575	**Gins2**	GINS complex subunit 2 (Psf2 homolo	292058	19	64873928	64886488	12561	803,10	-0,19	7,62E-01
ENSRNOG00000019754	**Gdap2**	ganglioside-induced differentiation-ass	362004	2	221921976	221977531	55556	420,67	-0,21	7,62E-01
ENSRNOG00000028688	**Ctns**	cystinosin, lysosomal cystine transpor	287478	10	59488895	59503993	15099	483,63	-0,20	7,62E-01
ENSRNOG00000020942	**Plekha4**	pleckstrin homology domain containin	308584	1	102596674	102619582	22909	237,46	-0,29	7,63E-01
ENSRNOG00000006394	**Tbc1d10a**	TBC1 domain family, member 10a (Tb	360968	14	85098922	85127054	28133	663,39	0,23	7,63E-01
ENSRNOG00000007838	**Ddx47**	DEAD (Asp-Glu-Ala-Asp) box polypep	297685	4	233048048	233060507	12460	1242,97	0,22	7,63E-01
ENSRNOG00000014797	**Tmbim1**	transmembrane BAX inhibitor motif co	316516	9	81335843	81353016	17174	491,99	-0,27	7,63E-01
ENSRNOG00000001145	**Ccdc64**	coiled-coil domain containing 64 (Ccd	304537	12	48413869	48501896	88028	960,28	0,22	7,64E-01
ENSRNOG00000008465	**Tmem176b**	transmembrane protein 176B (Tmem1	171411	4	143138374	143145816	7443	298,36	0,32	7,64E-01
ENSRNOG00000027220	**Pcdhgb8**	protocadherin gamma subfamily B, 8 (	364845	18	30603545	30606131	2587	495,71	-0,16	7,64E-01
ENSRNOG00000039730	**Orai3**	ORAI calcium release-activated calciu	309000	1	206239787	206244645	4859	366,09	-0,18	7,64E-01
ENSRNOG00000045821	**Slc41a3**	solute carrier family 41, member 3 (Slc	641603	4	187052887	187078590	25704	349,84	0,24	7,64E-01
ENSRNOG00000046381		Uncharacterized protein [Source:UniProtKB/TrE		3	178473711	178481975	8265	3511,74	0,15	7,64E-01
ENSRNOG00000046698	**Gps1**	G protein pathway suppressor 1 (Gps1	117039	10	109558645	109563401	4757	3841,01	0,12	7,64E-01
ENSRNOG00000018524	**Ezr**	ezrin (Ezr), mRNA [Source:RefSeq mF	54319	1	48590973	48634511	43539	4648,89	-0,24	7,64E-01
ENSRNOG00000003395	**Cby3**	Protein Cby3 [Source:UniProtKB/TrEI	690544	10	35626296	35631272	4977	21,70	-0,27	7,64E-01
ENSRNOG00000038478	**RGD15636**	Protein RGD1563668 [Source:UniPro	497907	10	40824107	40825361	1255	135,18	0,20	7,64E-01
ENSRNOG00000026787	**Disp2**	dispatched homolog 2 (Drosophila) (D	311324	3	117115017	117130512	15496	5337,01	0,21	7,64E-01

ENSRNOG00000008314	**Orc3**	origin recognition complex, subunit 3 (	313138	5	54590014	54644408	54395	282,45	-0,23	7,64E-01
ENSRNOG00000009431	**Tbc1d4**	Protein RGD1561609 [Source:UniPro	306117	15	89697677	89871384	173708	85,80	-0,23	7,64E-01
ENSRNOG00000024336	**Senp6**	SUMO1/sentrin specific peptidase 6 (S	300860	8	87025318	87101909	76592	1889,08	-0,20	7,64E-01
ENSRNOG00000032516		Protein Cks1bp6 [Source:UniProtKB/T	499655	5	114776113	114776352	240	50,09	0,26	7,64E-01
ENSRNOG00000045531		rab11 family-interacting protein 1 isofo	498650	16	68742619	68753634	11016	64,94	-0,27	7,64E-01
ENSRNOG00000002434	**Tmem100**	transmembrane protein 100 (Tmem10	497979	10	77399483	77405339	5857	339,06	-0,21	7,64E-01
ENSRNOG00000003042	**Mettl13**	methyltransferase like 13 (Mettl13), m	289159	13	85319679	85333384	13706	680,33	0,16	7,64E-01
ENSRNOG00000013415	**Ptpn18**	protein tyrosine phosphatase, non-rec	301333	9	40642586	40659435	16850	58,94	-0,26	7,64E-01
ENSRNOG00000016298	**Lysmd3**	LysM, putative peptidoglycan-binding,	315923	2	9409771	9412384	2614	38,00	0,25	7,64E-01
ENSRNOG00000018040	**Gins4**	GINS complex subunit 4 (Sld5 homolo	290842	16	73218779	73230596	11818	286,87	0,16	7,64E-01
ENSRNOG00000021406				9	64502404	64502751	348	21,05	-0,27	7,64E-01
ENSRNOG00000042781	**Ropn1l**	rhophilin associated tail protein 1-like (	685646	2	104193712	104204276	10565	74,45	-0,30	7,64E-01
ENSRNOG00000046700	**RGD13077**	Williams Beuren syndrome chromosor	363995	12	26767621	26776161	8541	25,73	0,26	7,64E-01
ENSRNOG00000009459	**Slc35f6**	solute carrier family 35, member F6 (S	298851	6	36910714	36922551	11838	1397,00	0,20	7,64E-01
ENSRNOG00000050770		Uncharacterized protein [Source:UniProtKB/TrE		20	13230092	13230795	704	73,66	0,20	7,64E-01
ENSRNOG00000000661	**Hps4**	Hermansky-Pudlak syndrome 4 (Hps4	304555	12	52043359	52071054	27696	592,61	0,20	7,65E-01
ENSRNOG00000002778	**Aatf**	apoptosis antagonizing transcription fa	114512	10	71654755	71747166	92412	505,24	0,18	7,65E-01
ENSRNOG00000025518	**Lrrc16b**	leucine rich repeat containing 16B (Lrr	361041	15	38058379	38075410	17032	1383,66	-0,13	7,65E-01
ENSRNOG00000000983	**Zfp394**	zinc finger protein 394 (Zfp394), mRN	252860	12	13248537	13256461	7925	391,11	0,19	7,65E-01
ENSRNOG00000005406	**Clint1**	clathrin interactor 1 (Clint1), mRNA [So	360515	10	30656516	30825797	169282	700,79	-0,12	7,65E-01
ENSRNOG00000010594	**LOC50012**	RIKEN cDNA 4921507P07 gene [Sour	500124	4	145013636	145038535	24900	27,61	-0,26	7,65E-01
ENSRNOG00000013841	**Vprbp**	Vpr (HIV-1) binding protein (Vprbp), m	315987	8	114907024	114974112	67089	870,67	-0,13	7,65E-01
ENSRNOG00000020674	**Rpl27**	ribosomal protein L27 (Rpl27), mRNA	64306	10	89151134	89154564	3431	3130,73	0,24	7,65E-01
ENSRNOG00000022720	**Usp37**	Ubiquitin carboxyl-terminal hydrolase	1E+08	9	81486028	81537301	51274	327,44	-0,26	7,65E-01
ENSRNOG00000031129		coactivator-associated arginine methy	363026	8	22631820	22676514	44695	1910,85	0,13	7,65E-01
ENSRNOG00000043004	**LOC10036**	high mobility group nucleosome bindir	114637	16	33775173	33776354	1182	2327,93	-0,16	7,65E-01
ENSRNOG00000047476	**Wasf1**	WAS protein family, member 1 (Wasf1	294568	20	50107711	50155913	48203	7718,26	-0,21	7,65E-01
ENSRNOG00000018876	**RGD15622**	serologically defined colon cancer anti	306322	3	9188726	9195447	6722	233,41	-0,19	7,65E-01
ENSRNOG00000005763	**Arl1**	ADP-ribosylation factor-like 1 (Arl1), m	64187	7	29384709	29396455	11747	1661,67	0,21	7,65E-01
ENSRNOG00000008340	**RGD13097**	similar to ENSANGP00000021391 (R	363074	8	68035524	68040147	4624	150,73	0,21	7,65E-01
ENSRNOG00000015633	**Cul3**	cullin 3 (Cul3), mRNA [Source:RefSeq	301555	9	85794367	85853449	59083	3029,35	-0,15	7,65E-01
ENSRNOG00000023603	**Amer3**	Protein Fam123c [Source:UniProtKB/	501122	9	40766583	40768937	2355	679,01	0,22	7,65E-01
ENSRNOG00000025584	**Agap2**	ArfGAP with GTPase domain, ankyrin	65218	7	70542515	70555124	12610	11879,68	-0,19	7,65E-01
ENSRNOG00000050485	**Gas1**	Protein LOC683470 [Source:UniProtK	683470	17	7073440	7074591	1152	490,22	0,26	7,65E-01
ENSRNOG00000017857	**LOC10091**	Cullin-7 [Source:UniProtKB/Swiss-Pro	680835	9	15536182	15548930	12749	1905,21	0,18	7,65E-01
ENSRNOG00000036852		poly(rC)-binding protein 2 [Source:Re	363005	7	141870629	141894027	23399	3362,02	0,12	7,65E-01
ENSRNOG00000048986		RIKEN cDNA D430019H16 gene [Source:MGI S		6	138513542	138513793	252	389,39	-0,20	7,65E-01
ENSRNOG00000017087	**Man1c1**	mannosidase, alpha, class 1C, membe	362625	5	156559978	156703434	143457	570,21	-0,27	7,66E-01

ENSRNOG00000021347	**Mthfd2l**	methylenetetrahydrofolate dehydroger	305248	14	18548222	18629209	80988	80,89	-0,22	7,66E-01
ENSRNOG00000032180		coiled-coil domain containing 15 [Sour	367056	8	39763344	39831052	67709	61,17	-0,25	7,66E-01
ENSRNOG00000028747				4	183274692	183275077	386	219,78	0,25	7,66E-01
ENSRNOG00000010942		Protein Gk5 [Source:UniProtKB/TrEM	367146	8	103378039	103448596	70558	34,38	-0,26	7,66E-01
ENSRNOG00000015425	**Ssx2ip**	synovial sarcoma, X breakpoint 2 inter	308023	2	270790451	270822030	31580	1108,00	0,22	7,66E-01
ENSRNOG00000012608	**Tmem212**	transmembrane protein 212 (Tmem21	499586	2	133290901	133321820	30920	44,42	-0,30	7,67E-01
ENSRNOG00000028329	**Zfp438**	zinc finger protein 438 (Zfp438), mRN	307024	17	52693425	52814680	121256	72,71	-0,22	7,67E-01
ENSRNOG00000004589	**Galnt16**	UDP-N-acetyl-alpha-D-galactosamine	362760	6	112246063	112422114	176052	1930,88	-0,12	7,67E-01
ENSRNOG00000019519	**Idnk**	idnK, gluconokinase homolog (E. coli)	498695	17	9088387	9095099	6713	116,59	0,24	7,67E-01
ENSRNOG00000042547	**RGD15655**	60S ribosomal protein L18a [Source:U	499057	1	62626294	62626824	531	24,89	0,30	7,67E-01
ENSRNOG00000014611	**LOC10091**	atrophin 1 (Atn1), mRNA [Source:RefS	29515	4	224284992	224298835	13844	39,30	-0,27	7,67E-01
ENSRNOG00000042960	**LOC68357**	regulator of cell cycle (Rgcc), mRNA [S	117183	15	65214322	65227156	12835	787,52	-0,25	7,68E-01
ENSRNOG00000011384	**Aimp1**	aminoacyl tRNA synthetase complex-i	114632	2	256268302	256291846	23545	593,08	0,18	7,68E-01
ENSRNOG00000012594	**Sugt1**	SGT1, suppressor of G2 allele of SKP	290408	15	65536894	65577747	40854	975,13	0,25	7,68E-01
ENSRNOG00000010919		Protein Mbd4 [Source:UniProtKB/TrE	680915	4	211029615	211040005	10391	111,46	-0,18	7,68E-01
ENSRNOG00000004973	**Ppp2r5c**	protein phosphatase 2, regulatory sub	691318	6	143982521	144075381	92861	2916,00	0,18	7,68E-01
ENSRNOG00000005814	**Katnbl1**	katanin p80 subunit B-like 1 (Katnbl1),	691543	3	110490944	110499724	8781	121,84	-0,29	7,68E-01
ENSRNOG00000012175	**Nfatc2**	nuclear factor of activated T-cells, cytc	311658	3	171384481	171500358	115878	44,32	-0,29	7,68E-01
ENSRNOG00000049131		intraflagellar transport 172 [Source:MGI Symbol;		6	36210855	36304251	93397	995,57	0,15	7,68E-01
ENSRNOG00000009781	**Dync1i2**	dynein cytoplasmic 1 intermediate cha	116659	3	64314539	64364643	50105	5201,69	0,20	7,68E-01
ENSRNOG00000005003	**Ptprn2**	protein tyrosine phosphatase, recepto	29714	6	153312968	154056676	743709	3968,38	0,17	7,68E-01
ENSRNOG00000002632	**Naca**	nascent polypeptide-associated compl	288770	7	2438021	2450436	12416	7200,97	-0,14	7,69E-01
ENSRNOG00000018186	**Lamtor5**	late endosomal/lysosomal adaptor, MA	295357	2	229503095	229508451	5357	1577,81	0,18	7,69E-01
ENSRNOG00000019656	**Btd**	biotinidase (Btd), mRNA [Source:RefS	306262	16	7706028	7712859	6832	451,60	-0,18	7,69E-01
ENSRNOG00000025731		Prothymosin alpha Prothymosin alpha	29222	13	94396996	94501245	104250	147,75	-0,23	7,69E-01
ENSRNOG00000002024		EPH receptor A5 (Epha5), transcript va	79208	14	25418094	25783444	365351	3687,96	-0,15	7,69E-01
ENSRNOG00000049410	**Chrm3**	cholinergic receptor, muscarinic 3 (Chr	24260	17	65755434	65757203	1770	254,31	-0,22	7,69E-01
ENSRNOG00000049604		mevalonate kinase [Source:RefSeq p	81727	12	49695252	49710386	15135	1201,68	0,28	7,69E-01
ENSRNOG00000042855	**Ahdc1**	AT hook, DNA binding motif, containin	362617	5	154878646	154944310	65665	1898,58	-0,12	7,69E-01
ENSRNOG00000001408	**Actl6b**	actin-like 6B (Actl6b), mRNA [Source:I	288563	12	24211506	24227904	16399	2053,48	0,19	7,69E-01
ENSRNOG00000013480	**Ing2**	inhibitor of growth family, member 2 (In	290744	16	47437446	47445331	7886	404,81	0,22	7,69E-01
ENSRNOG00000009740	**Slco1c1**	solute carrier organic anion transporte	84511	4	239951726	239997943	46218	1187,49	-0,26	7,69E-01
ENSRNOG00000012531	**Ephb2**	Eph receptor B2 (Ephb2), mRNA [Sou	313633	5	158789192	158907250	118059	804,98	0,18	7,69E-01
ENSRNOG00000016987		proline-serine-threonine phosphatase-	307248	18	73912643	73961705	49063	84,82	-0,26	7,69E-01
ENSRNOG00000018999	**Cdc14b**	cell division cycle 14B (Cdc14b), mRN	361195	17	1699259	1786825	87567	54,63	-0,22	7,69E-01
ENSRNOG00000019122	**Sec16a**	SEC16 homolog A (S. cerevisiae) (Sec	1E+08	3	9218903	9248509	29607	2952,92	0,11	7,69E-01
ENSRNOG00000024120	**Rxfp1**	relaxin/insulin-like family peptide recep	295144	2	197738113	197930308	192196	25,77	-0,25	7,69E-01
ENSRNOG00000029416	**Zfp952**	Protein Zfp952 [Source:UniProtKB/TrE	1E+08	7	15437337	15441621	4285	237,74	-0,16	7,69E-01

ENSRNOG00000000060	**Cplx1**	complexin 1 (Cplx1), mRNA [Source:R	64832	14	2190908	2220991	30084	4384,72	-0,18	7,69E-01
ENSRNOG00000015781	**Ndst3**	N-deacetylase/N-sulfotransferase (hep	295430	2	247095402	247243643	148242	70,74	-0,27	7,69E-01
ENSRNOG00000001317	**Zfp68**	zinc finger protein 68 (Zfp68), transcrip	304337	12	20141441	20156299	14859	528,82	-0,29	7,69E-01
ENSRNOG00000001506	**Tmem35**	transmembrane protein 35 (Tmem35),	308134	X	105015270	105038196	22927	3867,68	0,22	7,69E-01
ENSRNOG00000011053	**Cyp2u1**	cytochrome P450, family 2, subfamily	310848	2	254963069	254980063	16995	230,88	0,16	7,69E-01
ENSRNOG00000012122		Clavesin-2 [Source:UniProtKB/Swiss-Prot;Acc:/		1	26631832	26756155	124324	245,93	-0,22	7,69E-01
ENSRNOG00000020552	**Fosl1**	fos-like antigen 1 (Fosl1), mRNA [Sou	25445	1	227755887	227764393	8507	209,90	-0,21	7,69E-01
ENSRNOG00000014599	**Dhx36**	DEAH (Asp-Glu-Ala-His) box polypept	310461	2	172178850	172217986	39137	1333,76	-0,25	7,70E-01
ENSRNOG00000006515	**LOC10091**	kelch-like family member 15 (Klhl15),	314111	X	63938977	63983954	44978	79,46	0,25	7,70E-01
ENSRNOG00000007134	**LOC10091**	serine/threonine kinase receptor asso	297699	4	235987501	236001739	14239	2700,19	0,17	7,70E-01
ENSRNOG00000007899	**Mterf**	mitochondrial transcription termination	85261	4	27268602	27275598	6997	61,15	-0,27	7,70E-01
ENSRNOG00000009613	**Sh3bgrl2**	SH3 domain binding glutamic acid-rich	501026	8	90589261	90680680	91420	125,29	0,21	7,70E-01
ENSRNOG00000010296	**Slc7a7**	solute carrier family 7 (amino acid tran	83509	15	36894261	36938138	43878	32,17	-0,31	7,70E-01
ENSRNOG00000015335	**LOC10036**	ribosomal protein L13 (Rpl13), mRNA	81765	19	66622803	66625369	2567	126,85	0,21	7,70E-01
ENSRNOG00000047229	**Trim68**	Protein LOC684189 [Source:UniProtK	684189	1	174021170	174027149	5980	116,14	0,27	7,70E-01
ENSRNOG00000020004	**Rbm34**	RNA binding motif protein 34 (Rbm34)	307956	19	70541743	70562213	20471	685,20	-0,17	7,70E-01
ENSRNOG00000026420	**Iqcg**	IQ motif containing G (Iqcg), mRNA [S	363796	11	74052400	74092525	40126	20,86	-0,31	7,70E-01
ENSRNOG00000017328	**Pter**	phosphotriesterase related (Pter), mR	63852	17	81874644	81936881	62238	71,99	-0,21	7,70E-01
ENSRNOG00000026432	**Syt7**	synaptotagmin VII (Syt7), mRNA [Sou	59267	1	233382666	233440845	58180	1558,72	-0,19	7,70E-01
ENSRNOG00000009661	**Tgds**	TDP-glucose 4,6-dehydratase (Tgds),	306164	15	106757794	106778765	20972	441,67	-0,28	7,70E-01
ENSRNOG00000011668	**Nfil3**	nuclear factor, interleukin 3 regulated	114519	17	14372306	14374095	1790	737,20	0,19	7,70E-01
ENSRNOG00000000485	**Bak1**	BCL2-antagonist/killer 1 (Bak1), mRN	116502	20	7675062	7683674	8613	1302,79	0,15	7,70E-01
ENSRNOG00000000881	**Mmgt1**	membrane magnesium transporter 1 (	302864	X	153601506	153610102	8597	187,59	0,16	7,70E-01
ENSRNOG00000008176	**Nppa**	natriuretic peptide A (Nppa), mRNA [S	24602	5	168466309	168467618	1310	17,70	-0,32	7,70E-01
ENSRNOG00000008510	**Abtb2**	ankyrin repeat and BTB (POZ) domair	171440	3	100136624	100289999	153376	364,10	-0,31	7,70E-01
ENSRNOG00000010489		protein Smaug homolog 1 [Source:Re	305826	15	27721129	27815275	94147	185,87	-0,19	7,70E-01
ENSRNOG00000013090	**Gadd45g**	growth arrest and DNA-damage-induc	291005	17	15469301	15471060	1760	762,04	0,27	7,70E-01
ENSRNOG00000013339		glucocorticoid modulatory element bin	83635	3	180464070	180501893	37824	341,16	0,15	7,70E-01
ENSRNOG00000014064	**Ctsh**	cathepsin H (Ctsh), mRNA [Source:Re	25425	8	96939705	96958827	19123	1065,29	-0,24	7,70E-01
ENSRNOG00000014520	**Ing1**	inhibitor of growth family, member 1 (I	306626	16	82745456	82746160	705	777,54	0,22	7,70E-01
ENSRNOG00000015713	**Parva**	parvin, alpha (Parva), mRNA [Source:	57341	1	184227712	184383031	155320	557,71	0,19	7,70E-01
ENSRNOG00000016313	**Ctnnbip1**	catenin, beta-interacting protein 1 (Ctr	503000	5	170131839	170159318	27480	1583,91	-0,18	7,70E-01
ENSRNOG00000017097	**Lhpp**	phospholysine phosphohistidine inorg	361663	1	211611521	211702233	90713	2121,32	0,12	7,70E-01
ENSRNOG00000018412	**Sfi1**	Sfi1 homolog, spindle assembly assoc	305467	14	84034648	84092093	57446	451,71	-0,21	7,70E-01
ENSRNOG00000023226	**S100a10**	S100 calcium binding protein A10 (S1	81778	2	210966310	210975004	8695	1506,78	-0,17	7,70E-01
ENSRNOG00000025757	**Myh6**	myosin, heavy chain 6, cardiac muscle	29556	15	37492599	37516191	23593	25,08	-0,28	7,70E-01
ENSRNOG00000041411				3	119957206	119957306	101	74,70	0,28	7,70E-01
ENSRNOG00000002713	**Zfp672**	zinc finger protein 672 (Zfp672), mRN	303165	10	43545159	43553389	8231	425,11	0,15	7,70E-01

ENSRNOG00000003218	**Zfp286a**	zinc finger protein 286A (Zfp286a), mF	497923	10	48966916	48977608	10693	242,68	-0,24	7,70E-01
ENSRNOG00000004493	**Tasp1**	taspase, threonine aspartase 1 (Tasp1	311468	3	139347887	139579995	232109	281,45	0,21	7,70E-01
ENSRNOG00000010784	**Scg3**	secretogranin III (Scg3), mRNA [Sourc	116635	8	82183497	82242336	58840	6232,86	0,14	7,70E-01
ENSRNOG00000016172	**RGD13100**	Protein RGD1310016 [Source:UniPro	309306	1	254939281	255032155	92875	713,68	-0,19	7,71E-01
ENSRNOG00000022421	**Crtc1**	CREB regulated transcription coactiva	684527	16	20593197	20644901	51705	1254,35	0,14	7,72E-01
ENSRNOG00000001489	**Tbp**	TATA box binding protein (Tbp), mRNA	117526	1	58420201	58438561	18361	547,29	-0,20	7,72E-01
ENSRNOG00000003232	**Slc9a3r1**	solute carrier family 9, subfamily A (NH	59114	10	104573316	104590543	17228	1513,16	-0,27	7,72E-01
ENSRNOG00000007256	**Necab1**	N-terminal EF-hand calcium binding pi	64169	5	33184793	33414046	229254	200,18	-0,31	7,72E-01
ENSRNOG00000010626	**Sphk1**	sphingosine kinase 1 (Sphk1), transcri	170897	10	105165074	105167961	2888	21,09	-0,31	7,72E-01
ENSRNOG00000013889	**Tmed3**	transmembrane emp24 protein transp	300888	8	96576976	96584888	7913	901,93	0,20	7,72E-01
ENSRNOG00000014524	**S1pr3**	sphingosine-1-phosphate receptor 3 (S	306792	17	15873396	15874535	1140	468,29	-0,28	7,72E-01
ENSRNOG00000015597	**LOC10012**	RIKEN cDNA 1600012H06 gene [Sou	1E+08	1	58133058	58135240	2183	643,39	0,20	7,72E-01
ENSRNOG00000018255	**Clptm1**	cleft lip and palate associated transme	292696	1	81814947	81846254	31308	6163,82	0,16	7,72E-01
ENSRNOG00000019052	**Ankzf1**	ankyrin repeat and zinc finger domain	363255	9	82162933	82169791	6859	913,40	0,22	7,72E-01
ENSRNOG00000048120		Protein LOC100909580; RCG56817, i	308309	1	63024114	63037747	13634	52,00	0,23	7,72E-01
ENSRNOG00000050277	**Uckl1**	uridine-cytidine kinase 1-like 1 (Uckl1)	499956	3	180775487	180788073	12587	448,88	0,14	7,72E-01
ENSRNOG00000012767	**Micu3**	mitochondrial calcium uptake family, m	364601	16	54746920	54835505	88586	911,67	-0,21	7,72E-01
ENSRNOG00000000801	**Msl3l2**	male-specific lethal 3-like 2 (Drosophil	309790	20	39332020	39333479	1460	170,19	0,18	7,72E-01
ENSRNOG00000006976	**Med6**	mediator complex subunit 6 (Med6), m	299180	6	113448725	113462191	13467	357,65	0,16	7,72E-01
ENSRNOG00000016719	**Zfp161**	zinc finger protein 161 (Zfp161), mRN	282825	9	117205246	117206593	1348	288,22	-0,14	7,72E-01
ENSRNOG00000004283	**Camta2**	calmodulin binding transcription activa	287462	10	57036786	57054892	18107	1924,35	0,14	7,72E-01
ENSRNOG00000006595	**Htr3a**	5-hydroxytryptamine (serotonin) recep	79246	8	51803897	51816207	12311	168,86	-0,26	7,72E-01
ENSRNOG00000008639	**Pabpc1**	poly(A) binding protein, cytoplasmic 1	171350	7	75557273	75570171	12899	10879,64	-0,16	7,72E-01
ENSRNOG00000018020	**Apbb1**	amyloid beta (A4) precursor protein-bi	29722	1	177393536	177409951	16416	8681,96	0,14	7,72E-01
ENSRNOG00000033148		zinc finger protein 790 [Source:MGI Symbol;Acc		1	88881496	88886634	5139	130,64	-0,17	7,72E-01
ENSRNOG00000049633		major facilitator superfamily domain cc	683955	13	114180965	114224409	43445	250,35	-0,18	7,72E-01
ENSRNOG00000002139		RIKEN cDNA C530008M17 gene [Sou	289568	14	33440563	33469053	28491	9941,86	-0,13	7,72E-01
ENSRNOG00000015977	**Zfp609**	zinc finger protein 609 (Zfp609), mRN	363412	8	70914209	71016530	102322	4161,58	0,12	7,72E-01
ENSRNOG00000026647	**Cxcl16**	chemokine (C-X-C motif) ligand 16 (C	497942	10	56817244	56821837	4594	150,55	-0,27	7,73E-01
ENSRNOG00000049556				8	115343614	115343701	88	29,96	0,26	7,73E-01
ENSRNOG00000020022	**Psmc3ip**	PSMC3 interacting protein (Psmc3ip),	140938	10	88800612	88803709	3098	162,40	-0,21	7,73E-01
ENSRNOG00000005358	**Pmm1**	phosphomannomutase 1 (Pmm1), mR	300089	7	123158887	123169142	10256	4516,60	0,14	7,73E-01
ENSRNOG00000011161	**Slc2a12**	solute carrier family 2 (facilitated gluc	308028	1	25467855	25520421	52567	76,45	-0,22	7,73E-01
ENSRNOG00000018740	**LOC10091**	UDP glucuronosyltransferase 1 family,	24861	9	94910377	94990037	79661	384,90	-0,30	7,73E-01
ENSRNOG00000021571	**Rhebl1**	Ras homolog enriched in brain like 1 (	359959	X	115063116	115066994	3879	275,64	0,22	7,73E-01
ENSRNOG00000046307	**Glyctk**	glycerate kinase (Glyctk), mRNA [Sou	684314	8	114213447	114218466	5020	267,84	-0,17	7,73E-01
ENSRNOG00000047697		gamma-glutamyltransferase 1 [Source:MGI Sym		20	16209475	16214423	4949	26,32	-0,28	7,73E-01
ENSRNOG00000004682	**Parpbp**	PARP1 binding protein (Parpbp), mRN	1E+08	7	28764494	28825206	60713	40,95	-0,26	7,73E-01

ENSRNOG00000010896	**Tprn**	taperin (Tprn), mRNA [Source:RefSeq	499749	3	2461645	2469118	7474	533,62	0,21	7,73E-01
ENSRNOG00000012934	**Arhgef7**	Rho guanine nucleotide exchange fac	114559	16	82479279	82557593	78315	6524,01	0,15	7,73E-01
ENSRNOG00000014333	**Vcam1**	vascular cell adhesion molecule 1 (Vc	25361	2	237158195	237177632	19438	670,63	-0,26	7,73E-01
ENSRNOG00000015618	**Wnt5a**	wingless-type MMTV integration site fa	64566	16	4414512	4432113	17602	249,57	0,22	7,73E-01
ENSRNOG00000016770	**Calm3**	calmodulin 3 (Calm3), mRNA [Source:	24242	1	80090638	80099277	8640	23992,76	-0,24	7,73E-01
ENSRNOG00000020948	**Pth1r**	parathyroid hormone 1 receptor (Pth1r	56813	8	118328864	118348383	19520	128,30	-0,25	7,73E-01
ENSRNOG00000042269	**Gpr21**	G protein-coupled receptor 21 (Gpr21)	689917	3	27002939	27005862	2924	28,25	-0,26	7,73E-01
ENSRNOG00000014483		putative DNA helicase INO80 complex	296084	3	117738630	117817221	78592	902,16	0,16	7,73E-01
ENSRNOG00000018726	**Elmo1**	engulfment and cell motility 1 (Elmo1),	361251	17	56598697	57133815	535119	1965,06	0,16	7,73E-01
ENSRNOG00000021776	**Cenpc**	centromere protein C (Cenpc), mRNA	305270	14	23515343	23571971	56629	366,87	-0,28	7,73E-01
ENSRNOG00000038004	**Zfp804a**	Protein Zfp804a [Source:UniProtKB/T	295695	3	76091997	76116669	24673	248,75	0,17	7,73E-01
ENSRNOG00000049780	**Sri**	sorcin (Sri), mRNA [Source:RefSeq ml	683667	4	22938728	22952058	13331	819,31	-0,15	7,73E-01
ENSRNOG00000000204	**Syncrip**	synaptotagmin binding, cytoplasmic R	363113	8	95602783	95628111	25329	1225,38	-0,19	7,73E-01
ENSRNOG00000019308	**Arrb2**	arrestin, beta 2 (Arrb2), mRNA [Sourc	25388	10	56797480	56805282	7803	551,10	0,14	7,73E-01
ENSRNOG00000011511	**Stk24**	serine/threonine kinase 24 (Stk24), ml	361092	15	110022517	110073160	50644	659,53	0,22	7,73E-01
ENSRNOG00000000718	**Cggbp1**	CGG triplet repeat binding protein 1 (C	288353	11	1876785	1893694	16910	1915,50	-0,15	7,73E-01
ENSRNOG00000000949		FL cytokine receptor precursor [Sourc	140635	12	11493007	11552299	59293	19,37	-0,29	7,73E-01
ENSRNOG00000013552	**Scd1**	stearoyl-Coenzyme A desaturase 1 (S	246074	1	271602887	271615702	12816	3458,88	-0,30	7,73E-01
ENSRNOG00000022871	**LOC69117(**	RIKEN cDNA 2610008E11 gene [Sour	691170	7	13517760	13533965	16206	172,73	-0,22	7,73E-01
ENSRNOG00000001717		optic atrophy 1 (Opa1), mRNA [Source	171116	11	77764210	77840586	76377	2369,47	0,17	7,73E-01
ENSRNOG00000018709		POZ (BTB) and AT hook containing zi	305471	14	84198496	84229550	31055	1889,70	0,12	7,73E-01
ENSRNOG00000034037	**Zfp266**	zinc finger protein 266 (Zfp266), mRN	367034	8	21569186	21580886	11701	3063,09	-0,21	7,73E-01
ENSRNOG00000028243		derlin-3 [Source:RefSeq peptide;Acc:	690315	20	15858311	15861768	3458	98,80	-0,25	7,74E-01
ENSRNOG00000000842	**Ddah2**	dimethylarginine dimethylaminohydrol	294239	20	7122435	7125498	3064	4086,71	0,20	7,74E-01
ENSRNOG00000007261	**Gli2**	GLI family zinc finger 2 (Gli2), mRNA [	304729	13	39955094	40011594	56501	122,87	-0,22	7,74E-01
ENSRNOG00000007920	**Ssbp3**	single stranded DNA binding protein 3	84354	5	130364179	130499466	135288	2234,65	-0,13	7,74E-01
ENSRNOG00000014153	**Lhfp**	lipoma HMGIC fusion partner (Lhfp), n	499615	2	161947947	162146115	198169	1231,69	-0,16	7,74E-01
ENSRNOG00000017940	**Rere**	arginine-glutamic acid dipeptide (RE) i	116665	5	170960573	171292512	331940	5236,60	0,14	7,74E-01
ENSRNOG00000010065		Protein Dgkh [Source:UniProtKB/TrEI	361076	15	64471270	64625227	153958	208,99	-0,27	7,74E-01
ENSRNOG00000015448	**Fam69c**	Protein Fam69c [Source:UniProtKB/T	498891	18	80614800	80635777	20978	286,11	0,19	7,74E-01
ENSRNOG00000016340	**Plcg1**	phospholipase C, gamma 1 (Plcg1), m	25738	3	162957126	162987842	30717	4636,39	0,14	7,74E-01
ENSRNOG00000017907	**Ube2i**	ubiquitin-conjugating enzyme E2I (Ube	25573	10	14437609	14453131	15523	356,33	0,15	7,74E-01
ENSRNOG00000018964	**Gss**	glutathione synthetase (Gss), mRNA [	25458	3	157443739	157474042	30304	523,19	0,18	7,74E-01
ENSRNOG00000019048	**Sod2**	superoxide dismutase 2, mitochondria	24787	1	51827380	51834230	6851	1652,64	-0,11	7,74E-01
ENSRNOG00000020140	**Pigq**	phosphatidylinositol glycan anchor bio	287159	10	15102511	15118524	16014	2218,66	0,16	7,74E-01
ENSRNOG00000020336	**Ezh1**	enhancer of zeste homolog 1 (Drosopl	303547	10	88902523	88928835	26313	867,53	0,24	7,74E-01
ENSRNOG00000024506	**LOC50047!**	RIKEN cDNA 4933430I17 gene [Sourc	500475	5	82503506	82531126	27621	43,00	0,22	7,74E-01
ENSRNOG00000032183	**Scmh1**	sex comb on midleg homolog 1 (Dros	362581	5	143168705	143249138	80434	744,42	-0,12	7,74E-01

ENSRNOG00000020142	**Gdf1**	ceramide synthase 1 (Cers1), mRNA [	306351	16	20698224	20699716	1493	1287,67	0,19	7,74E-01
ENSRNOG00000012604	**Swsap1**	SWIM-type zinc finger 7 associated pr	363029	8	23021536	23024064	2529	922,16	0,12	7,74E-01
ENSRNOG00000018932	**Ccdc124**	coiled-coil domain containing 124 (Cc	290642	16	20174990	20180809	5820	2546,28	0,20	7,74E-01
ENSRNOG00000027456	**Cdc42bpg**	CDC42 binding protein kinase gamma	293693	1	228661352	228681280	19929	88,32	-0,28	7,74E-01
ENSRNOG00000027503	**LOC69153**	ribosomal protein s25 (Rps25), mRNA	122799	8	47346312	47348692	2381	2672,96	0,18	7,74E-01
ENSRNOG00000004458	**Ston2**	stonin 2 (Ston2), mRNA [Source:RefS	314349	6	124654250	124765198	110949	405,83	-0,18	7,74E-01
ENSRNOG00000012763	**Cwf19l1**	CWF19-like 1, cell cycle control (S. po	365465	1	271332007	271355243	23237	925,53	0,24	7,74E-01
ENSRNOG00000017838	**Gmcl1**	germ cell-less, spermatogenesis asso	312516	4	183065019	183104409	39391	653,62	-0,17	7,74E-01
ENSRNOG00000018176	**Rab6a**	RAB6A, member RAS oncogene famil	84379	1	171826278	171865057	38780	11548,78	-0,21	7,74E-01
ENSRNOG00000020935	**Capn1**	calpain 1, (mu/I) large subunit (Capn1	29153	1	228280719	228304852	24134	26,31	0,29	7,74E-01
ENSRNOG00000021503		Protein LOC100912815 [Source:UniP	300225	X	115486141	115536581	50441	208,92	-0,18	7,74E-01
ENSRNOG00000024294				15	90889560	90890329	770	315,73	0,17	7,74E-01
ENSRNOG00000050317	**LOC10091**	UHRF1 binding protein 1-like (Uhrf1bp	363009	7	30505665	30553762	48098	1191,17	0,14	7,74E-01
ENSRNOG00000016920	**Spire2**	spire homolog 2 (Drosophila) (Spire2),	307925	19	66847908	66886394	38487	769,77	0,19	7,74E-01
ENSRNOG00000014031	**Esco1**	establishment of cohesion 1 homolog	307595	18	1823873	1862268	38396	174,18	-0,29	7,75E-01
ENSRNOG00000028198	**Sh2b3**	SH2B adaptor protein 3 (Sh2b3), mRN	58838	12	42132947	42136714	3768	344,03	-0,17	7,75E-01
ENSRNOG00000024309	**LOC68175**	similar to cytochrome c oxidase, subu	681754	1	90230830	90238903	8074	992,03	0,23	7,75E-01
ENSRNOG00000017828	**Egr3**	early growth response 3 (Egr3), mRN	25148	15	55482581	55485279	2699	145,85	-0,27	7,76E-01
ENSRNOG00000021041		family with sequence similarity 171, m	1E+08	10	90172344	90181848	9505	5664,19	0,14	7,76E-01
ENSRNOG00000025639	**Slc39a12**	solute carrier family 39 (zinc transport	291328	17	83212254	83288595	76342	547,77	-0,26	7,76E-01
ENSRNOG00000004206	**Glrx5**	glutaredoxin 5 (Glrx5), mRNA [Source	362776	6	137947875	137957960	10086	2756,73	0,26	7,76E-01
ENSRNOG00000011015	**Hivep2**	human immunodeficiency virus type I	29721	1	9930383	9953696	23314	5670,88	-0,18	7,76E-01
ENSRNOG00000036758	**Iqck**	Protein LOC100909537 [Source:UniP	1E+08	1	195514699	195603567	88869	87,81	-0,27	7,76E-01
ENSRNOG00000046891		hexokinase 1 [Source:MGI Symbol;Acc:MGI:961		20	33703472	33770509	67038	9395,23	-0,13	7,76E-01
ENSRNOG00000009045		dolichyl pyrophosphate Man9GlcNAc2	362547	5	122348215	122398919	50705	108,02	-0,21	7,76E-01
ENSRNOG00000023140	**Pnldc1**	poly(A)-specific ribonuclease (PARN)-	361478	1	51693999	51712435	18437	26,15	-0,28	7,77E-01
ENSRNOG00000033863	**RGD15604**	Protein RGD1560470 [Source:UniPro	362083	3	2780467	2786856	6390	1079,03	-0,15	7,77E-01
ENSRNOG00000001204	**Ankrd13a**	ankyrin repeat domain 13a (Ankrd13a	360823	12	49352116	49381860	29745	2025,04	-0,15	7,77E-01
ENSRNOG00000001657	**Cldnd1**	claudin domain containing 1 (Cldnd1),	288182	11	47176078	47182290	6213	2266,57	-0,21	7,77E-01
ENSRNOG00000007784	**Bloc1s1**	biogenesis of lysosomal organelles co	288785	7	3312814	3316434	3621	1622,66	0,24	7,77E-01
ENSRNOG00000008477	**Mrpl53**	mitochondrial ribosomal protein L53 (M	362388	4	178627009	178627896	888	1073,94	0,24	7,77E-01
ENSRNOG00000009499	**Hinfp**	Protein Hinfp [Source:UniProtKB/TrEI	300665	8	47246470	47252624	6155	392,71	0,20	7,77E-01
ENSRNOG00000014869	**Wdr31**	WD repeat domain 31 (Wdr31), mRNA	298096	5	82425258	82444010	18753	245,82	0,15	7,77E-01
ENSRNOG00000015756	**Nphp1**	nephronophthisis 1 (juvenile) (Nphp1),	296136	3	126969057	127017983	48927	521,59	0,21	7,77E-01
ENSRNOG00000016610	**Arhgap1**	Rho GTPase activating protein 1 (Arhg	311193	3	87254431	87275981	21551	3014,44	-0,14	7,77E-01
ENSRNOG00000046208	**Rsad1**	radical S-adenosyl methionine domain	688146	10	82098788	82099872	1085	52,68	0,22	7,77E-01
ENSRNOG00000015983	**MGC94335**	Uncharacterized protein C2orf47 hom	301418	9	63901497	63909426	7930	779,26	0,12	7,77E-01
ENSRNOG00000036911	**Bicd1**	bicaudal D homolog 1 (Drosophila) (Bi	362466	4	248136721	248283930	147210	312,18	-0,26	7,77E-01

ENSRNOG00000004040		ATP-binding cassette, sub-family A (ABC1), men		10	97950653	98008559	57907	107,57	-0,20	7,77E-01
ENSRNOG00000004118	**Frmpd4**	FERM and PDZ domain containing 4 (	302656	X	28470068	28599599	129532	883,11	0,14	7,77E-01
ENSRNOG00000004968	**Ncapg2**	Protein Ncapg2 [Source:UniProtKB/Tı	362798	6	153221967	153288508	66542	134,68	0,29	7,77E-01
ENSRNOG00000007061	**RGD15602**	Protein RGD1560224 [Source:UniPro	500608	6	2991537	3072513	80977	59,60	-0,27	7,77E-01
ENSRNOG00000031097	**Syce1l**	Protein Syce1l [Source:UniProtKB/TrE	1E+08	19	56771134	56781454	10321	50,44	0,21	7,77E-01
ENSRNOG00000000586	**Gtf3c6**	general transcription factor IIIC, polype	361858	20	47067523	47076603	9081	636,01	-0,18	7,77E-01
ENSRNOG00000008034	**Tmeff1**	transmembrane protein with EGF-like	63845	5	68842285	68926413	84129	3330,42	-0,17	7,77E-01
ENSRNOG00000011026	**Irf2bpl**	interferon regulatory factor 2 binding p	314329	6	120193879	120196227	2349	2541,41	0,13	7,77E-01
ENSRNOG00000011093	**Ssna1**	Sjogren syndrome nuclear autoantiger	311802	3	2471319	2471805	487	686,61	0,22	7,77E-01
ENSRNOG00000012840	**Sparc**	secreted protein, acidic, cysteine-rich	24791	10	40573518	40595276	21759	31929,01	-0,21	7,77E-01
ENSRNOG00000016940	**Ppp2r2d**	protein phosphatase 2, regulatory sub	246255	1	218132463	218166449	33987	779,63	0,23	7,77E-01
ENSRNOG00000018237	**Gstp1**	glutathione S-transferase pi 1 (Gstp1),	24426	1	226162330	226164798	2469	5583,91	-0,25	7,77E-01
ENSRNOG00000001270		hydrogen voltage-gated channel 1 [So	304485	12	41703158	41710333	7176	35,43	-0,31	7,77E-01
ENSRNOG00000015630	**Cct7**	chaperonin containing Tcp1, subunit 7	297406	4	181812638	181829880	17243	5608,21	0,14	7,77E-01
ENSRNOG00000026770	**Tspyl3**	TSPY-like 3 (Tspyl3), mRNA [Source:F	296280	3	155390626	155392698	2073	641,56	-0,17	7,78E-01
ENSRNOG00000008713	**Slc41a2**	solute carrier family 41 (magnesium tr	362861	7	26651682	26734269	82588	865,22	-0,18	7,78E-01
ENSRNOG00000003670		Uncharacterized protein [Source:UniProtKB/TrE		10	104184695	104194896	10202	1627,95	-0,17	7,78E-01
ENSRNOG00000004962	**Prdm4**	PR domain containing 4 (Prdm4), mRI	170820	7	24114241	24135565	21325	976,76	0,20	7,78E-01
ENSRNOG00000006052	**Sulf2**	sulfatase 2 (Sulf2), mRNA [Source:Re	311642	3	168962200	169044078	81879	3438,70	-0,26	7,78E-01
ENSRNOG00000012966	**Acadl**	acyl-CoA dehydrogenase, long chain (	25287	9	73434321	73472895	38575	1810,42	-0,26	7,78E-01
ENSRNOG00000014872	**Sec24d**	Protein Sec24d; Sec24d protein [Sou	310843	2	246849788	246919956	70169	667,46	-0,23	7,78E-01
ENSRNOG00000030487		Uncharacterized protein [Source:UniProtKB/TrE		3	62363998	62434837	70840	42,27	-0,26	7,78E-01
ENSRNOG00000025448	**Limd2**	LIM domain containing 2 (Limd2), mRI	360646	10	94099477	94101550	2074	1479,07	0,21	7,78E-01
ENSRNOG00000001053	**Tmed2**	transmembrane emp24 domain trafficl	65165	12	39342088	39351124	9037	5271,85	0,16	7,78E-01
ENSRNOG00000001278	**Gpn3**	GPN-loop GTPase 3 (Gpn3), mRNA [S	360810	12	41551262	41559994	8733	325,23	0,21	7,78E-01
ENSRNOG00000010126	**RGD13077**	similar to RIKEN cDNA 2410016O06 (	314300	6	118113409	118115202	1794	571,36	0,15	7,78E-01
ENSRNOG00000015598	**Zfp772**	zinc finger protein 978 (Zfp978), mRN	308318	1	71133884	71140536	6653	89,79	-0,23	7,78E-01
ENSRNOG00000019996	**Slc16a1**	solute carrier family 16 (monocarboxyl	25027	2	226529274	226550086	20813	1964,83	-0,23	7,78E-01
ENSRNOG00000000599	**Lama4**	Protein Lama4 [Source:UniProtKB/TrI	309816	20	45787184	45926467	139284	103,28	0,25	7,78E-01
ENSRNOG00000003419	**Bcas3**	breast carcinoma amplified sequence	363662	10	72660660	72944206	283547	505,04	0,15	7,78E-01
ENSRNOG00000006803	**Dnajc10**	DnaJ (Hsp40) homolog, subfamily C, r	295690	3	74098922	74138865	39944	2642,18	-0,19	7,78E-01
ENSRNOG00000018676	**RGD15629**	similar to cDNA sequence BC031181 (	498886	18	70114130	70122605	8476	1421,29	-0,18	7,78E-01
ENSRNOG00000019892	**Lrrfip1**	leucine rich repeat (in FLII) interacting	367314	9	97871992	97939937	67946	375,24	-0,19	7,78E-01
ENSRNOG00000026646	**Ndufs5**	NADH dehydrogenase (ubiquinone) Fe	362588	5	145178506	145195972	17467	735,99	0,22	7,78E-01
ENSRNOG00000039350	**Gng13**	guanine nucleotide binding protein (G	685451	10	14901656	14903560	1905	29,27	-0,29	7,78E-01
ENSRNOG00000049597		Uncharacterized protein [Source:UniProtKB/TrE		4	140727495	140908581	181087	41,34	-0,26	7,78E-01
ENSRNOG00000010440	**Gnal**	Guanine nucleotide-binding protein G(	24611	18	62036103	62131420	95318	800,08	-0,14	7,78E-01
ENSRNOG00000013993		Uncharacterized protein [Source:UniProtKB/TrE		1	283399380	283411892	12513	48,88	-0,28	7,78E-01

ENSRNOG00000046434	**Hist1h2ail1**	histone cluster 1, H2ai-like (Hist1h2ail	291159	17	45671789	45672199	411	18,49	0,30	7,78E-01
ENSRNOG00000006440	**Ift27**	intraflagellar transport 27 homolog (Ch	300062	7	119384971	119401297	16327	749,06	0,19	7,79E-01
ENSRNOG00000007561	**Glb1l2**	galactosidase, beta 1-like 2 (Glb1l2), r	503194	8	27827460	27872964	45505	49,16	-0,30	7,79E-01
ENSRNOG00000012055		alpha-mannosidase 2x [Source:RefSe	308757	1	143096200	143115693	19494	1679,74	-0,15	7,79E-01
ENSRNOG00000016836	**Casp7**	caspase 7 (Casp7), mRNA [Source:Re	64026	1	284591629	284623731	32103	360,94	0,21	7,79E-01
ENSRNOG00000020304	**Pdcd11**	programmed cell death 11 (Pdcd11), n	309458	1	274297342	274338453	41112	2084,24	-0,11	7,79E-01
ENSRNOG00000030862	**Atp6v1h**	ATPase, H+ transporting, lysosomal V	297797	5	19044666	19149940	105275	3454,40	0,22	7,79E-01
ENSRNOG00000036598	**LOC68593**	TBC1D12: TBC1 domain family, meml	685933	1	265008850	265089552	80703	226,73	-0,19	7,79E-01
ENSRNOG00000007286	**Mdm1**	Mdm1 nuclear protein homolog (mous	314859	7	61171143	61197122	25980	554,40	-0,22	7,79E-01
ENSRNOG00000009248	**Pnrc2**	proline-rich nuclear receptor coactivat	1E+08	5	157985391	157988374	2984	1078,21	-0,26	7,79E-01
ENSRNOG00000032162	**Atf1**	activating transcription factor 1 (Atf1),	315305	7	139692651	139735614	42964	367,37	0,18	7,79E-01
ENSRNOG00000021904	**Tecpr2**	Protein Tecpr2 [Source:UniProtKB/TrE	314456	6	145009992	145099887	89896	982,09	0,18	7,79E-01
ENSRNOG00000012434	**Zfp598**	zinc finger protein 598 (Zfp598), mRN	287119	10	13852129	13864076	11948	1280,13	0,15	7,80E-01
ENSRNOG00000012263	**Cnot7**	CCR4-NOT transcription complex, sub	306492	16	54602180	54621341	19162	1440,58	-0,16	7,80E-01
ENSRNOG00000013443	**Tm9sf3**	Protein Tm9sf3 [Source:UniProtKB/Tr	309475	1	267924051	267973774	49724	5250,51	-0,18	7,80E-01
ENSRNOG00000037254	**G6pd**	glucose-6-phosphate dehydrogenase	24377	1	152015532	152034676	19145	2443,14	0,12	7,80E-01
ENSRNOG00000043136		Uncharacterized protein [Source:UniProtKB/TrE		17	37764545	37769141	4597	37,73	-0,26	7,80E-01
ENSRNOG00000010008	**Polr3a**	DNA-directed RNA polymerase [Sour	361102	16	712736	750758	38023	1096,66	0,17	7,80E-01
ENSRNOG00000005868		tetratricopeptide repeat domain 21B [Source:MG		3	58917760	58990221	72462	255,05	-0,19	7,81E-01
ENSRNOG00000011237	**Zc3h13**	zinc finger CCCH type containing 13 (	305955	15	61051549	61116257	64709	1437,87	0,10	7,81E-01
ENSRNOG00000016963	**Trip12**	thyroid hormone receptor interactor 12	316575	9	92039803	92167938	128136	4359,42	-0,18	7,81E-01
ENSRNOG00000019220	**Rhod**	ras homolog family member D (Rhod),	293660	1	226536283	226550218	13936	63,87	0,24	7,81E-01
ENSRNOG00000019536	**Smim3**	small integral membrane protein 3 (Sn	286910	18	54978252	55005211	26960	418,27	0,16	7,81E-01
ENSRNOG00000038738	**LOC68520**	RIKEN cDNA 1700019D03 gene [Sou	685203	9	53106666	53106989	324	21,78	-0,27	7,81E-01
ENSRNOG00000042179	**Gtl3**	gene trap locus 3 (Gtl3), mRNA [Sourc	307642	19	10009274	10022984	13711	1685,57	-0,13	7,81E-01
ENSRNOG00000001274	**Vps29**	vacuolar protein sorting 29 homolog (S	288666	12	41570698	41579742	9045	2676,79	-0,15	7,81E-01
ENSRNOG00000006792	**Cep57**	centrosomal protein 57 (Cep57), mRN	315423	8	12285708	12305367	19660	441,17	-0,22	7,81E-01
ENSRNOG00000014240	**Trmt11**	tRNA methyltransferase 11 homolog (S	378794	1	30880325	30927014	46690	43,53	-0,25	7,81E-01
ENSRNOG00000014297	**Sdc4**	syndecan 4 (Sdc4), mRNA [Source:Re	24771	3	167052789	167071476	18688	1608,96	-0,26	7,81E-01
ENSRNOG00000027761	**Htatsf1**	HIV-1 Tat specific factor 1 (Htatsf1), m	317612	X	154143843	154158071	14229	919,80	0,24	7,81E-01
ENSRNOG00000000471	**B3galt4**	UDP-Gal:betaGlcNAc beta 1,3-galacto	171079	20	7504745	7506286	1542	100,83	0,17	7,81E-01
ENSRNOG00000018516	**Impa2**	inositol (myo)-1(or 4)-monophosphata	282636	18	62201776	62232717	30942	55,97	-0,19	7,81E-01
ENSRNOG00000003198	**RGD13057**	similar to RIKEN cDNA 2900011O08 (	360459	10	2286740	2443752	157013	5950,55	0,21	7,81E-01
ENSRNOG00000004685		metastasis associated 3 [Source:MGI Symbol;A		6	6899431	6981336	81906	674,45	0,14	7,81E-01
ENSRNOG00000010291	**Slc46a1**	solute carrier family 46 (folate transpo	303333	10	65901910	65909255	7346	1070,12	0,18	7,81E-01
ENSRNOG00000013321	**Dock11**	dedicator of cytokinesis 11 (Dock11), r	313438	X	122657242	122840107	182866	295,42	0,29	7,81E-01
ENSRNOG00000018089	**Sra1**	steroid receptor RNA activator 1 (Sra1	252891	18	29199079	29202306	3228	870,09	0,11	7,81E-01
ENSRNOG00000006827	**Tmem198b**	transmembrane protein 198b (Tmem1	500762	7	3196403	3200737	4335	321,72	0,16	7,82E-01

ENSRNOG00000007637	**Acer2**	alkaline ceramidase 2 (Acer2), mRNA	313339	5	109217448	109266599	49152	77,56	-0,23	7,82E-01
ENSRNOG00000008765	**Zfhx4**	zinc finger homeobox 4 (Zfhx4), mRN/	310250	2	118157860	118345227	187368	499,07	-0,16	7,82E-01
ENSRNOG00000009785		Cyclin-dependent kinase inhibitor 3 [Source:Uni		15	27496741	27508022	11282	228,34	0,27	7,82E-01
ENSRNOG00000022710	**Prrg4**	proline rich Gla (G-carboxyglutamic ac	499847	3	101408709	101433212	24504	27,82	0,30	7,82E-01
ENSRNOG00000008480	**Kcnab3**	potassium voltage-gated channel, sha	58981	10	55669298	55675728	6431	45,63	0,24	7,82E-01
ENSRNOG00000029663	**Il1rapl1**	interleukin 1 receptor accessory protei	317553	X	55629811	55687317	57507	24,83	0,29	7,82E-01
ENSRNOG00000006302	**Gclc**	glutamate-cysteine ligase, catalytic su	25283	8	84627768	84666185	38418	2756,68	-0,18	7,82E-01
ENSRNOG00000039949	**Rad54b**	RAD54 homolog B (S. cerevisiae) (Ra	313063	5	29863341	29928098	64758	31,15	0,24	7,82E-01
ENSRNOG00000002697	**Mtmr1**	myotubularin related protein 1 (Mtmr1)	317296	18	337401	370993	33593	1132,43	0,11	7,82E-01
ENSRNOG00000020277	**Cntnap1**	contactin associated protein 1 (Cntnap	84008	10	88888142	88902110	13969	286,40	0,15	7,82E-01
ENSRNOG00000004359	**Wars**	tryptophanyl-tRNA synthetase (Wars),	314442	6	141941104	141972286	31183	2574,78	0,17	7,82E-01
ENSRNOG00000010235	**Pkig**	protein kinase inhibitor, gamma (Pkig)	266709	3	166238136	166305338	67203	1633,48	0,17	7,82E-01
ENSRNOG00000021156	**Vegfb**	vascular endothelial growth factor B (\	89811	1	229229801	229233423	3623	682,51	-0,25	7,82E-01
ENSRNOG00000001891		Protein Gnb1l [Source:UniProtKB/TrE	680266	11	89677401	89701820	24420	146,16	0,15	7,82E-01
ENSRNOG00000002301	**Uso1**	USO1 vesicle transport factor (Uso1),	56042	14	17289140	17354576	65437	2769,31	-0,14	7,82E-01
ENSRNOG00000005882	**Tle1**	transducin-like enhancer of split 1 (E(s	362533	5	92618609	92701144	82536	1026,52	-0,18	7,82E-01
ENSRNOG00000019587	**Ptprn**	protein tyrosine phosphatase, recepto	116660	9	82215893	82231061	15169	4927,13	-0,20	7,82E-01
ENSRNOG00000027250		Protein Rbm27 [Source:UniProtKB/Tr	361317	18	36273350	36320859	47510	787,03	-0,13	7,82E-01
ENSRNOG00000002339	**Mark1**	MAP/microtubule affinity-regulating kir	117016	13	107485344	107589708	104365	3352,47	0,20	7,82E-01
ENSRNOG00000015420	**Stxbp1**	syntaxin binding protein 1 (Stxbp1), m	25558	3	17160754	17221831	61078	18276,08	-0,17	7,82E-01
ENSRNOG00000015703	**Hypk**	Protein Hypk [Source:UniProtKB/TrEM	311359	3	119963812	119964965	1154	1279,99	0,16	7,82E-01
ENSRNOG00000016137	**Rad23b**	RAD23 homolog B (S. cerevisiae) (Ra	298012	5	76267574	76305116	37543	11170,12	-0,17	7,82E-01
ENSRNOG00000017571	**Ndufa2**	NADH dehydrogenase (ubiquinone) 1	291660	18	29289478	29291567	2090	1523,60	0,18	7,82E-01
ENSRNOG00000017854	**Ucp2**	uncoupling protein 2 (mitochondrial, pı	54315	1	171707621	171713990	6370	45,77	-0,28	7,82E-01
ENSRNOG00000028822	**Ngfrap1**	nerve growth factor receptor (TNFRSF	117089	X	106309449	106311017	1569	3911,27	0,13	7,82E-01
ENSRNOG00000049019	**Tmem170a**	transmembrane protein 170A (Tmem1	498953	19	54896017	54901828	5812	144,28	0,19	7,82E-01
ENSRNOG00000050547		Synaptogyrin-2 [Source:UniProtKB/S\	89815	10	106451958	106456492	4535	423,86	0,25	7,82E-01
ENSRNOG00000003163	**Sdhc**	succinate dehydrogenase complex, su	289217	13	94125549	94146440	20892	6824,67	0,18	7,83E-01
ENSRNOG00000001493		kaptin [Source:RefSeq peptide;Acc:N	308107	1	79282539	79290168	7630	435,14	-0,17	7,83E-01
ENSRNOG00000002240	**Dirc2**	disrupted in renal carcinoma 2 (Dirc2),	303902	11	71288001	71359970	71970	3362,53	0,15	7,83E-01
ENSRNOG00000004657	**Sec23a**	Sec23 homolog A (S. cerevisiae) (Sec	58817	6	89586788	89633108	46321	2972,22	-0,11	7,83E-01
ENSRNOG00000006470	**Camk1g**	calcium/calmodulin-dependent protein	171358	13	116631306	116654952	23647	1170,19	0,23	7,83E-01
ENSRNOG00000007117	**Cluap1**	clusterin associated protein 1 (Cluap1	363544	10	10602017	10635054	33038	700,62	0,18	7,83E-01
ENSRNOG00000008827	**Cog5**	Protein Cog5 [Source:UniProtKB/TrEI	314030	6	59615559	59901353	285795	487,22	-0,18	7,83E-01
ENSRNOG00000014259	**Mycl**	v-myc avian myelocytomatosis viral or	298506	5	144483332	144487332	4001	583,47	-0,13	7,83E-01
ENSRNOG00000020139	**RGD15657**	Protein RGD1565775; Similar to RIKE	361980	2	207453978	207481697	27720	2002,12	0,23	7,83E-01
ENSRNOG00000022245	**Msantd4**	Myb/SANT-like DNA-binding domain c	500941	8	1497145	1507753	10609	683,91	-0,25	7,83E-01
ENSRNOG00000024629	**Hadha**	hydroxyacyl-CoA dehydrogenase/3-ke	170670	6	37400511	37439591	39081	4630,67	0,17	7,83E-01

ENSRNOG00000028844	**Slc9a5**	solute carrier family 9, subfamily A (NH	192215	19	48170660	48190747	20088	239,44	0,22	7,83E-01
ENSRNOG00000032840	**Sumo2l**	small ubiquitin-like modifier 2 (Sumo2)	682787	5	19767450	19768442	993	1424,37	-0,16	7,83E-01
ENSRNOG00000042916	**Rwdd1**	RWD domain containing 1 (Rwdd1), m	259218	20	29363288	29379915	16628	771,59	0,22	7,83E-01
ENSRNOG00000045644	**LOC68484**	Histone H3 [Source:UniProtKB/TrEMB	310678	17	45664146	45664556	411	19,36	0,30	7,83E-01
ENSRNOG00000045844	**Impact**	imprinted and ancient (Impact), mRNA	497198	18	4313599	4334407	20809	2226,89	-0,26	7,83E-01
ENSRNOG00000002515	**Atp7a**	ATPase, Cu++ transporting, alpha poly	24941	X	56243029	56310187	67159	170,70	-0,23	7,83E-01
ENSRNOG00000014562	**Hnrnpf**	heterogeneous nuclear ribonucleoprot	64200	4	215899353	215908011	8659	2235,57	0,16	7,83E-01
ENSRNOG00000006248	**Trim37**	tripartite motif-containing 37 (Trim37),	360592	10	75532170	75677779	145610	1628,13	-0,20	7,83E-01
ENSRNOG00000021203		Atlastin-3 [Source:UniProtKB/Swiss-P	309187	1	229733455	229775603	42149	300,26	-0,21	7,84E-01
ENSRNOG00000003649	**Qsox1**	quiescin Q6 sulfhydryl oxidase 1 (Qso	84491	13	78351223	78388725	37503	1370,81	0,18	7,84E-01
ENSRNOG00000005568		pecanex-like 4 (Drosophila) [Source:M	690217	6	104761251	104792348	31098	1214,90	-0,18	7,84E-01
ENSRNOG00000020873	**Nphs1**	nephrosis 1, congenital, Finnish type (	64563	1	90077824	90106424	28601	24,05	0,29	7,84E-01
ENSRNOG00000027240		kinase non-catalytic C-lobe domain (KIND) conta		1	219152239	219196635	44397	777,10	-0,16	7,84E-01
ENSRNOG00000003522	**Mr1**	major histocompatibility complex, class	25119	13	77707537	77725283	17747	24,46	-0,29	7,85E-01
ENSRNOG00000009636	**Scrn1**	secernin 1 (Scrn1), mRNA [Source:Re	502776	4	149342036	149400634	58599	7254,94	0,12	7,85E-01
ENSRNOG00000025815	**Cdr2l**	Protein Cdr2l; Similar to paraneoplasti	360656	10	104332243	104358042	25800	595,00	0,21	7,85E-01
ENSRNOG00000012148	**Trio**	Protein Trio [Source:UniProtKB/TrEM	310192	2	100139159	100435855	296697	5989,76	0,15	7,86E-01
ENSRNOG00000009318	**RGD15635**	Protein RGD1563583 [Source:UniPro	299208	6	119073228	119102361	29134	180,59	-0,15	7,86E-01
ENSRNOG00000007587	**Tcp11l2**	t-complex 11, testis-specific-like 2 (Tcp	314683	7	25004999	25031722	26724	715,28	-0,23	7,86E-01
ENSRNOG00000009265	**Kcnk9**	potassium channel, subfamily K, mem	84429	7	113841045	113875376	34332	424,84	0,20	7,86E-01
ENSRNOG00000009980	**Ppap2a**	phosphatidic acid phosphatase type 2	64369	2	63751336	63766877	15542	767,22	-0,16	7,86E-01
ENSRNOG00000010680	**Med12l**	mediator complex subunit 12-like (Med	690752	2	168632572	168735461	102890	234,06	0,16	7,86E-01
ENSRNOG00000010721	**Dlgap5**	discs, large (Drosophila) homolog-ass	289997	15	28108228	28138753	30526	204,35	0,28	7,86E-01
ENSRNOG00000016218	**Gtf3c1**	general transcription factor IIIC, polype	171063	1	204017341	204083109	65769	4066,33	0,13	7,86E-01
ENSRNOG00000021269	**Chgb**	chromogranin B (secretogranin 1) (Ch	24259	3	131912219	131925372	13154	3388,17	0,18	7,86E-01
ENSRNOG00000028630	**Ksr2**	Protein Ksr2 [Source:UniProtKB/TrEM	288691	12	46362374	46511934	149561	208,99	-0,18	7,86E-01
ENSRNOG00000033101		Protein Myo18a [Source:UniProtKB/TrEMBL;Ac		10	66559668	66646100	86433	5155,33	-0,12	7,86E-01
ENSRNOG00000038902	**RGD15656**	RGD1565641 (RGD1565641), mRNA	499567	2	108114927	108121186	6260	520,52	-0,25	7,86E-01
ENSRNOG00000022657	**Tmem97**	transmembrane protein 97 (Tmem97),	303330	10	65823082	65832164	9083	851,45	0,20	7,86E-01
ENSRNOG00000000924	**Slc7a1**	solute carrier family 7 (cationic amino	25648	12	10169916	10192908	22993	2345,53	-0,20	7,86E-01
ENSRNOG00000001773	**Senp2**	Sumo1/sentrin/SMT3 specific peptidas	78973	11	85718646	85755312	36667	972,93	0,18	7,86E-01
ENSRNOG00000008075	**Ift74**	intraflagellar transport 74 homolog (Ch	313365	5	117537609	117629815	92207	225,61	-0,30	7,86E-01
ENSRNOG00000008658	**Mitf**	microphthalmia-associated transcriptio	25094	4	194667979	194918737	250759	31,03	-0,23	7,86E-01
ENSRNOG00000009382		methyltransferase like 8 [Source:MGI	502633	3	64067594	64103342	35749	24,27	0,27	7,86E-01
ENSRNOG00000010833	**Mthfd2**	methylenetetrahydrofolate dehydroger	313410	4	179592814	179604331	11518	496,35	-0,17	7,86E-01
ENSRNOG00000012192		zinc finger protein 740 [Source:MGI Sy	685834	7	141611103	141614789	3687	339,80	0,16	7,86E-01
ENSRNOG00000017086		zinc finger protein 341 [Source:MGI Symbol;Acc		3	156507626	156525604	17979	151,51	0,16	7,86E-01
ENSRNOG00000017225	**Fam160b1**	LOC361774 (Predicted); Protein Fam1	361774	1	285574327	285597886	23560	751,23	0,13	7,86E-01

ENSRNOG00000018683	**LOC10090!**	dedicator of cyto-kinesis 1 (Dock1), m	309081	1	213838803	214349252	510450	1604,53	-0,20	7,86E-01
ENSRNOG00000020616	**Yif1b**	Yip1 interacting factor homolog B (S. c	292768	1	88361280	88371399	10120	1147,69	0,23	7,86E-01
ENSRNOG00000020731	**Ppfia3**	protein tyrosine phosphatase, recepto	140591	1	102393147	102420877	27731	3250,48	0,18	7,86E-01
ENSRNOG00000036813	**Ndrg3**	NDRG family member 3 (Ndrg3), mRNA [Source		3	158659369	158692285	32917	1357,31	0,12	7,86E-01
ENSRNOG00000049880	**Tmem200c**	transmembrane protein 200C (Tmem2	501201	9	116518447	116524285	5839	557,94	-0,15	7,86E-01
ENSRNOG00000050315	**Dcxr**	dicarbonyl L-xylulose reductase (Dcxr)	171408	10	109499391	109501278	1888	110,64	0,26	7,86E-01
ENSRNOG00000002286		Uncharacterized protein [Source:UniProtKB/TrE		10	11892969	11899527	6559	49,79	-0,20	7,86E-01
ENSRNOG00000008553	**Mthfr**	Methylenetetrahydrofolate reductase	362657	5	168504579	168518702	14124	188,63	0,21	7,86E-01
ENSRNOG00000009369	**Tor4a**	torsin family 4, member A (Tor4a), mR	311795	3	2389140	2392827	3688	25,07	0,28	7,86E-01
ENSRNOG00000016031		bicaudal D homolog 2 isoform 2 [Sou	306809	17	17731019	17775591	44573	1997,51	0,15	7,86E-01
ENSRNOG00000019822	**Gadd45b**	growth arrest and DNA-damage-induc	299626	7	11813536	11815565	2030	87,68	0,24	7,86E-01
ENSRNOG00000048324		Uncharacterized protein [Source:UniProtKB/TrE		2	205346424	205508257	161834	140,30	0,15	7,86E-01
ENSRNOG00000022870	**Smad5**	SMAD family member 5 (Smad5), mR	59328	17	10488652	10507888	19237	1348,77	-0,13	7,86E-01
ENSRNOG00000011315	**Med16**	mediator complex subunit 16 (Med16)	299607	7	12789464	12801962	12499	2244,54	0,18	7,86E-01
ENSRNOG00000007966	**B4galt1**	UDP-Gal:betaGlcNAc beta 1,4- galact	24390	5	61653278	61700179	46902	113,86	0,24	7,87E-01
ENSRNOG00000013562	**Ndfip1**	Nedd4 family interacting protein 1 (Nd	291609	18	31252998	31303451	50454	18477,02	0,18	7,87E-01
ENSRNOG00000006238	**Traf2**	Tnf receptor-associated factor 2 (Traf2	311786	3	2727488	2752033	24546	476,46	0,18	7,87E-01
ENSRNOG00000010189	**Rps24**	ribosomal protein S24 (Rps24), mRNA [Source:I		16	752366	757004	4639	1946,28	0,12	7,87E-01
ENSRNOG00000017917	**Cdhr2**	Protein Cdhr2 [Source:UniProtKB/TrE	291002	17	12541003	12565478	24476	254,32	0,23	7,87E-01
ENSRNOG00000036601	**Exoc6**	exocyst complex component 6 (Exoc6	50556	1	263708964	263850916	141953	325,86	-0,21	7,87E-01
ENSRNOG00000046366		dystrophin (Dmd), transcript variant Dp	24907	X	53540769	53697914	157146	446,75	-0,19	7,87E-01
ENSRNOG00000047028	**Mmp24**	matrix metallopeptidase 24 (Mmp24),	83513	3	157700503	157720612	20110	6113,85	-0,15	7,87E-01
ENSRNOG00000027057	**Gga3**	golgi associated, gamma adaptin ear c	360658	10	104124628	104143981	19354	1353,01	0,15	7,88E-01
ENSRNOG00000003781	**Atp10b**	Protein Atp10b [Source:UniProtKB/Tr	303056	10	28527558	28704605	177048	22,55	0,29	7,88E-01
ENSRNOG00000018191		opioid receptor, mu 1 (Oprm1), transcr	25601	1	44804261	44857206	52946	31,02	-0,23	7,88E-01
ENSRNOG00000025724	**Mtf1**	metal-regulatory transcription factor 1	362591	5	146568010	146612766	44757	91,79	0,20	7,88E-01
ENSRNOG00000045884				10	94728205	94728305	101	125,35	0,29	7,88E-01
ENSRNOG00000000014		Uncharacterized protein [Source:UniProtKB/TrE		3	158673563	158679000	5438	575,79	0,16	7,88E-01
ENSRNOG00000031031	**Zfp292**	zinc finger protein 292 (Zfp292), mRN	50552	5	54851038	54931298	80261	760,35	0,28	7,88E-01
ENSRNOG00000043068		Uncharacterized protein [Source:UniProtKB/TrE		8	69463020	69477177	14158	33,05	-0,22	7,88E-01
ENSRNOG00000032152		Protein Rab11fip3; Rab11fip3 protein	303002	10	15162863	15194666	31804	1715,13	0,15	7,88E-01
ENSRNOG00000003827	**Wipi1**	WD repeat domain, phosphoinositide i	303630	10	97578115	97614772	36658	1057,95	-0,19	7,88E-01
ENSRNOG00000007100		coiled-coil domain containing 136 [Sou	362331	4	56444662	56471661	27000	554,77	-0,15	7,89E-01
ENSRNOG00000012953	**Arsa**	arylsulfatase A (Arsa), mRNA [Source:	315222	7	130132039	130137458	5420	733,41	-0,18	7,89E-01
ENSRNOG00000014358	**Zic5**	Zic family member 5 (Zic5), mRNA [Sc	361095	15	112280174	112286885	6712	67,83	-0,18	7,89E-01
ENSRNOG00000037911	**LOC68022**	LRRGT00193 (LOC680227), mRNA [S	680227	X	75109508	75146839	37332	200,32	-0,24	7,89E-01
ENSRNOG00000005046	**Tspan13**	tetraspanin 13 (Tspan13), mRNA [Sou	366602	6	65111579	65138717	27139	6608,90	0,18	7,89E-01
ENSRNOG00000015181	**Snx21**		1E+08	3	167377588	167445774	68187	667,82	0,13	7,89E-01

ENSRNOG00000023346	**Rsg1**	REM2 and RAB-like small GTPase 1 (	500576	5	163450671	163451619	949	18,07	-0,26	7,89E-01
ENSRNOG00000045950		transmembrane channel-like gene family 6 [Sou		10	106405917	106420027	14111	133,17	-0,26	7,89E-01
ENSRNOG00000018372	**Cul9**	cullin 9 (Cul9), mRNA [Source:RefSeq	316228	9	15654761	15745377	90617	1317,25	0,16	7,89E-01
ENSRNOG00000012153	**Rbl2**	retinoblastoma-like 2 (Rbl2), mRNA [S	81758	19	28364201	28410920	46720	1272,99	-0,14	7,89E-01
ENSRNOG00000000075	**Mtf2**	metal response element binding trans	360905	14	2657176	2699986	42811	327,16	0,20	7,89E-01
ENSRNOG00000009406	**Tm9sf4**	transmembrane 9 superfamily protein	296279	3	155408354	155457981	49628	1481,91	0,20	7,89E-01
ENSRNOG00000016474	**Tspan3**	tetraspanin 3 (Tspan3), mRNA [Source	300733	8	59377470	59400900	23431	14255,17	0,19	7,89E-01
ENSRNOG00000042912	**Mycbpap**	Mycbp associated protein (Mycbpap),	494192	10	82051977	82072661	20685	17,95	-0,27	7,89E-01
ENSRNOG00000003721	**Paqr4**	progestin and adipoQ receptor family	302967	10	12891716	12895328	3613	1664,80	0,12	7,89E-01
ENSRNOG00000020876	**LOC68131**	Bcl2-associated X protein (Bax), mRN	24887	1	102530748	102536167	5420	1979,65	0,22	7,89E-01
ENSRNOG00000004532	**Fam69b**	family with sequence similarity 69, me	362090	3	9442976	9450728	7753	2721,26	0,17	7,90E-01
ENSRNOG00000009873		LRRGT00024; Uncharacterized protein [Source		15	49667494	49713060	45567	84,09	-0,18	7,90E-01
ENSRNOG00000000007	**Gad1**	glutamate decarboxylase 1 (Gad1), m	24379	3	63479963	63519877	39915	6638,46	0,23	7,90E-01
ENSRNOG00000002092	**Hunk**	hormonally upregulated Neu-associate	288275	11	34169334	34282445	113112	179,37	0,17	7,90E-01
ENSRNOG00000002178	**Mrps18c**	mitochondrial ribosomal protein S18C	289469	14	10388657	10394875	6219	702,34	-0,14	7,90E-01
ENSRNOG00000002821	**Phlpp1**	PH domain and leucine rich repeat pro	59265	13	31327712	31550366	222655	3692,47	-0,12	7,90E-01
ENSRNOG00000004720	**Kcnj2**	potassium inwardly-rectifying channel,	29712	10	99130687	99131970	1284	66,42	0,27	7,90E-01
ENSRNOG00000004893	**Atp5s**	ATP synthase, H+ transporting, mitoch	362749	6	101507336	101525541	18206	270,50	-0,20	7,90E-01
ENSRNOG00000009773	**Elovl4**	ELOVL fatty acid elongase 4 (Elovl4),	315851	8	90830571	90857120	26550	1377,00	0,22	7,90E-01
ENSRNOG00000010737	**Mbnl2**	muscleblind-like splicing regulator 2 (M	680445	15	109043305	109175502	132198	427,44	-0,18	7,90E-01
ENSRNOG00000010993	**Dpm1**	dolichyl-phosphate mannosyltransfera	296394	3	171105899	171125442	19544	552,94	0,14	7,90E-01
ENSRNOG00000011777	**Spag5**	sperm associated antigen 5 (Spag5),	252918	10	66053386	66071009	17624	386,40	0,20	7,90E-01
ENSRNOG00000012249	**Txlnb**	taxilin beta (Txlnb), mRNA [Source:Re	308622	1	14609470	14655258	45789	28,74	-0,27	7,90E-01
ENSRNOG00000013922	**Dok2**	docking protein 2 (Dok2), mRNA [Sou	290361	15	56176051	56179111	3061	47,10	0,27	7,90E-01
ENSRNOG00000014027	**RGD13047**	Protein RGD1304728 [Source:UniPro	360560	10	58469915	58512207	42293	486,77	0,18	7,90E-01
ENSRNOG00000016630	**Tln1**	talin 1 (Tln1), mRNA [Source:RefSeq	313494	5	63558230	63588186	29957	2286,99	0,17	7,90E-01
ENSRNOG00000019196	**Xpnpep3**	X-prolyl aminopeptidase (aminopeptid	685823	7	122612798	122661425	48628	401,93	-0,14	7,90E-01
ENSRNOG00000019734		mitochondrial ribosomal protein L14 (M	301250	9	16576229	16581660	5432	965,86	0,23	7,90E-01
ENSRNOG00000020225	**Dlgap4**	discs, large (Drosophila) homolog-ass	286930	3	158850471	158944119	93649	3472,52	0,14	7,90E-01
ENSRNOG00000023830	**Dnase2**	deoxyribonuclease II, lysosomal (Dnas	171575	19	36998478	37001146	2669	391,54	-0,25	7,90E-01
ENSRNOG00000030800	**Igdcc3**	Protein Igdcc3 [Source:UniProtKB/TrE	315759	8	70327832	70377787	49956	156,88	0,17	7,90E-01
ENSRNOG00000046979		Uncharacterized protein [Source:UniProtKB/TrE		1	91291632	91292162	531	127,29	0,17	7,90E-01
ENSRNOG00000047052	**Clpp**	ATP-dependent Clp protease proteoly	301117	9	9049368	9055012	5645	1419,51	0,18	7,90E-01
ENSRNOG00000047620	**Golph3l**	golgi phosphoprotein 3-like (Golph3l),	310669	2	217210324	217235987	25664	358,03	-0,23	7,90E-01
ENSRNOG00000005585	**Strn3**	striatin, calmodulin binding protein 3 (	114520	6	81940488	82026817	86330	1554,11	-0,15	7,91E-01
ENSRNOG00000016273	**Fam136a**	family with sequence similarity 136, m	297415	4	182731397	182737312	5916	596,69	0,16	7,91E-01
ENSRNOG00000016486	**Nop58**	NOP58 ribonucleoprotein (Nop58), mR	60373	9	66300036	66323764	23729	941,67	-0,24	7,91E-01
ENSRNOG00000018158	**Tmem181**	transmembrane protein 181 (Tmem18	502228	1	48465874	48505759	39886	1233,01	-0,20	7,91E-01

ENSRNOG00000020617	**Mus81**	MUS81 structure-specific endonucleas	293678	1	227791797	227797070	5274	425,56	0,14	7,91E-01
ENSRNOG00000024128	**Aco2**	aconitase 2, mitochondrial (Aco2), mR	79250	7	123077888	123120964	43077	13656,68	0,10	7,91E-01
ENSRNOG00000032112				MT	2665	2739	75	361,43	-0,19	7,91E-01
ENSRNOG00000037815	**Acad10**	acyl-CoA dehydrogenase family, mem	304500	12	42285817	42328188	42372	247,33	0,16	7,91E-01
ENSRNOG00000023549	**Samd5**	sterile alpha motif domain containing 5	365038	1	5388579	5437905	49327	195,68	-0,15	7,91E-01
ENSRNOG00000028586	**Mcph1**	Protein Mcph1 [Source:UniProtKB/TrE	306594	16	75509829	75715383	205555	410,28	-0,13	7,91E-01
ENSRNOG00000010062		Uncharacterized protein [Source:UniProtKB/TrE		5	138332170	138333222	1053	40,97	0,29	7,91E-01
ENSRNOG00000008098	**Lrrc4**	leucine rich repeat containing 4 (Lrrc4	641521	4	55859570	55861528	1959	1135,69	-0,11	7,91E-01
ENSRNOG00000009974	**Coq3**	coenzyme Q3 homolog, methyltransfe	29309	5	40679635	40710202	30568	428,69	0,15	7,91E-01
ENSRNOG00000016110	**Kcnk12**	potassium channel, subfamily K, mem	64119	6	21346993	21464940	117948	165,90	0,18	7,91E-01
ENSRNOG00000017781	**Dnajc7**	DnaJ (Hsp40) homolog, subfamily C, r	303536	10	88291502	88327520	36019	2375,89	0,17	7,91E-01
ENSRNOG00000023007	**Col6a6**	Protein Col6a6 [Source:UniProtKB/Tr	315979	8	113729107	113825010	95904	21,52	-0,25	7,91E-01
ENSRNOG00000037248	**Plxna3**	plexin A3 (Plxna3), mRNA [Source:Re	309280	1	152103540	152119323	15784	2719,98	-0,17	7,91E-01
ENSRNOG00000019873		echinoderm microtubule associated protein like 3		1	232066656	232120730	54075	389,53	-0,23	7,91E-01
ENSRNOG00000001436	**Ywhag**	tyrosine 3-monooxygenase/tryptophar	56010	12	25788286	25816531	28246	16282,27	0,18	7,91E-01
ENSRNOG00000005041	**Crip2**	cysteine-rich protein 2 (Crip2), mRNA	338401	6	146947874	146952833	4960	4409,47	0,15	7,91E-01
ENSRNOG00000008944	**Ext2**	exostosin glycosyltransferase 2 (Ext2)	311215	3	89303368	89435102	131735	1506,85	0,12	7,91E-01
ENSRNOG00000014460		human immunodeficiency virus type I enhancer I		17	24256199	24289488	33290	1526,16	-0,16	7,91E-01
ENSRNOG00000018782	**Gmnn**	geminin (Gmnn), mRNA [Source:RefS	291137	17	44170134	44178377	8244	195,22	0,26	7,91E-01
ENSRNOG00000020635	**Exosc5**	exosome component 5 (Exosc5), mRN	308441	1	83713977	83723114	9138	419,94	0,15	7,91E-01
ENSRNOG00000023373	**Sec24b**	SEC24 family, member B (S. cerevisia	295461	2	249637719	249707661	69943	2085,23	0,10	7,91E-01
ENSRNOG00000047340				10	78646951	78647046	96	137,64	0,19	7,91E-01
ENSRNOG00000028461	**Gnl3**	guanine nucleotide binding protein-like	290556	16	7026191	7032102	5912	578,35	-0,21	7,92E-01
ENSRNOG00000047768	**Lamb2**	laminin, beta 2 (Lamb2), mRNA [Sourc	25473	8	116613077	116625217	12141	1465,00	-0,23	7,92E-01
ENSRNOG00000042711		RGD1563782 protein; Uncharacterized protein		11	90011605	90014032	2428	27,64	0,27	7,92E-01
ENSRNOG00000001651	**Dcbld2**	discoidin, CUB and LCCL domain con	155696	11	47430604	47483838	53235	647,19	0,18	7,92E-01
ENSRNOG00000007166	**Gramd1b**	GRAM domain containing 1B (Gramd1	300644	8	61211947	61373438	161492	1040,45	-0,16	7,92E-01
ENSRNOG00000008798	**Pipox**	pipecolic acid oxidase (Pipox), mRNA	303272	10	66660854	66673403	12550	44,04	0,22	7,92E-01
ENSRNOG00000013333	**Maml3**	mastermind like 3 (Drosophila) (Maml3	310405	2	160716327	160717919	1593	68,34	-0,23	7,92E-01
ENSRNOG00000018783	**Bcas2**	breast carcinoma amplified sequence	295334	2	225092498	225100379	7882	1016,14	0,13	7,92E-01
ENSRNOG00000047053	**Thyn1**	thymocyte nuclear protein 1 (Thyn1), r	300470	8	28064823	28073704	8882	1134,57	0,15	7,92E-01
ENSRNOG00000010472	**Ccdc32**	coiled-coil domain containing 32 (Ccdc	296081	3	117353970	117364626	10657	2041,27	0,14	7,93E-01
ENSRNOG00000015538	**Abcd2**	ATP-binding cassette, subfamily D (AL	84356	7	131968473	132017044	48572	284,35	0,19	7,93E-01
ENSRNOG00000021142	**Pepd**	peptidase D (Pepd), mRNA [Source:R	292808	1	92392878	92416063	23186	845,65	0,12	7,93E-01
ENSRNOG00000045629	**Xrcc6bp1**	XRCC6 binding protein 1 (Xrcc6bp1),	299828	7	70360825	70375828	15004	222,43	-0,17	7,93E-01
ENSRNOG00000019152	**Trpc4ap**	transient receptor potential cation chai	362247	3	157518203	157588962	70760	5597,94	-0,10	7,93E-01
ENSRNOG00000000463	**Col11a2**	collagen, type XI, alpha 2 (Col11a2), n	294279	20	5909495	5938415	28921	208,25	-0,15	7,94E-01
ENSRNOG00000037087		Uncharacterized protein [Source:UniProtKB/TrE		7	126792542	126792820	279	254,21	0,17	7,94E-01

ENSRNOG00000000867	**Vars**	valyl-tRNA synthetase (Vars), mRNA [	25009	20	7066750	7081273	14524	2910,97	0,18	7,94E-01
ENSRNOG00000010732	**RGD15615**	Sin3-associated polypeptide 18 (Sap1	290284	15	41910103	41914431	4329	134,79	0,19	7,94E-01
ENSRNOG00000005389	**Ppp2ca**	protein phosphatase 2, catalytic subur	24672	10	37324233	37343983	19751	949,08	-0,17	7,94E-01
ENSRNOG00000007084	**March10**	membrane-associated ring finger (C3H	303596	10	93350039	93438439	88401	19,77	-0,25	7,94E-01
ENSRNOG00000024140	**Sft2d1**	SFT2 domain containing 1 (Sft2d1), m	292305	1	54248199	54264093	15895	1570,12	0,17	7,94E-01
ENSRNOG00000047526	**Zfp526**	Protein LOC687127; RCG53789 [Sou	687127	1	83361852	83363852	2001	135,81	0,16	7,94E-01
ENSRNOG00000001236		Protein Brat1 [Source:UniProtKB/TrEI	498150	12	18014033	18025424	11392	565,84	0,11	7,94E-01
ENSRNOG00000001548	**Nfe2l2**	nuclear factor, erythroid derived 2, like	83619	3	69041647	69069072	27426	678,13	-0,27	7,94E-01
ENSRNOG00000003849	**Stx8**	syntaxin 8 (Stx8), mRNA [Source:RefS	59074	10	54258230	54498841	240612	345,57	0,15	7,94E-01
ENSRNOG00000005907	**Rad18**	RAD18 homolog (S. cerevisiae) (Rad1	362412	4	207836648	207920030	83383	131,47	-0,27	7,94E-01
ENSRNOG00000006810	**Lancl2**	LanC lantibiotic synthetase componen	362375	4	153348313	153387979	39667	1948,17	0,18	7,94E-01
ENSRNOG00000007244	**LOC10091**	Der1-like domain family, member 2 [So	1E+08	10	57386773	57398527	11755	454,90	0,14	7,94E-01
ENSRNOG00000007837	**Acot11**	acyl-CoA thioesterase 11 (Acot11), mF	1E+08	5	130192237	130212638	20402	19,29	-0,28	7,94E-01
ENSRNOG00000008264	**Fndc8**	fibronectin type III domain containing 8	303376	10	69874015	69882153	8139	18,57	0,26	7,94E-01
ENSRNOG00000008612	**Agbl5**	ATP/GTP binding protein-like 5 (Agbl5	362710	6	36653651	36671181	17531	535,89	0,17	7,94E-01
ENSRNOG00000009181	**Dleu7**	deleted in lymphocytic leukemia, 7 (Dl	290308	15	49135932	49151896	15965	174,55	0,20	7,94E-01
ENSRNOG00000009754	**Nampt**	nicotinamide phosphoribosyltransferas	297508	6	61753125	61787574	34450	640,27	-0,15	7,94E-01
ENSRNOG00000012482		NDRG family member 4 (Ndrg4), trans	64457	19	9751518	9787015	35498	17360,14	0,18	7,94E-01
ENSRNOG00000013326		Protein Hmbox1 [Source:UniProtKB/T	305968	15	51947244	52031402	84159	498,91	0,17	7,94E-01
ENSRNOG00000014317	**Arpc5l**	actin related protein 2/3 complex, subu	296710	3	28523340	28530898	7559	336,77	0,19	7,94E-01
ENSRNOG00000014627	**Wnt1**	wingless-type MMTV integration site fa	24881	X	114975118	114979165	4048	21,19	0,26	7,94E-01
ENSRNOG00000014893	**Wdr63**	WD repeat domain 63 (Wdr63), mRNA	292165	2	270435973	270494382	58410	61,98	-0,29	7,94E-01
ENSRNOG00000015097	**Kcmf1**	potassium channel modulatory factor 1	684322	4	165526163	165549472	23310	1754,49	-0,12	7,94E-01
ENSRNOG00000017108	**Syngr1**	synaptogyrin 1 (Syngr1), mRNA [Sour	29205	7	121301106	121324532	23427	1343,94	0,18	7,94E-01
ENSRNOG00000021469		coiled-coil domain containing 14 [Sour	288054	11	72374164	72407312	33149	91,52	-0,25	7,94E-01
ENSRNOG00000026704	**Drp2**	dystrophin related protein 2 (Drp2), mI	66027	X	105131004	105176943	45940	287,60	0,20	7,94E-01
ENSRNOG00000028176	**LOC10091**	ELMO/CED-12 domain containing 2 (E	688581	19	35445968	35465400	19433	29,61	0,23	7,94E-01
ENSRNOG00000028512	**Ilvbl**	ilvB (bacterial acetolactate synthase)-l	362843	7	14201069	14211122	10054	509,10	0,19	7,94E-01
ENSRNOG00000029321	**Ccser1**	coiled-coil serine-rich protein 1 (Ccser	500153	4	156183029	156605322	422294	129,66	-0,18	7,94E-01
ENSRNOG00000030355	**Mroh1**	maestro heat-like repeat family memb	1E+08	7	117432821	117459811	26991	171,52	-0,24	7,94E-01
ENSRNOG00000032034		suppressor of Ty 5 [Source:MGI Symb	308472	1	86651826	86680238	28413	4832,65	0,14	7,94E-01
ENSRNOG00000032660	**Lphn2**	latrophilin 2 (Lphn2), transcript variant	171447	2	277462665	277666602	203938	2983,36	-0,14	7,94E-01
ENSRNOG00000033498	**Cib1**	calcium and integrin binding 1 (calmyr	81823	1	142969357	142974856	5500	675,78	-0,23	7,94E-01
ENSRNOG00000033772	**Serpinb9**	serpin peptidase inhibitor, clade B (ova	361241	17	34542627	34554071	11445	167,74	-0,27	7,94E-01
ENSRNOG00000038480	**Ppp1r36**	protein phosphatase 1, regulatory sub	299153	6	108854061	108874065	20005	63,15	-0,24	7,94E-01
ENSRNOG00000039357	**Fam129c**	family with sequence similarity 129, m	498604	16	19887776	19899604	11829	52,53	-0,24	7,94E-01
ENSRNOG00000039985	**Ube2a**	ubiquitin-conjugating enzyme E2A (Ub	298317	X	123632116	123642663	10548	1607,16	0,12	7,94E-01
ENSRNOG00000042826	**Zfp52**	zinc finger protein 52 (Zfp52), mRNA [	361487	1	62456477	62500679	44203	32,10	-0,24	7,94E-01

ENSRNOG00000043487	**RGD15601**	Protein RGD1560171 [Source:UniPro	367949	X	143708537	143709355	819	132,84	-0,21	7,94E-01
ENSRNOG00000047111		Nol6 protein; Uncharacterized protein [Source:U		5	61976173	61985660	9488	1209,74	0,15	7,94E-01
ENSRNOG00000049020	**Tceal3**	Protein LOC679974; Protein Tceal3 [S	501628	X	107206874	107208817	1944	302,88	0,12	7,94E-01
ENSRNOG00000050424	**Decr2**	2,4-dienoyl CoA reductase 2, peroxiso	64461	10	15265552	15273597	8046	95,81	-0,18	7,94E-01
ENSRNOG00000001514	**Cdca7**	cell division cycle associated 7 (Cdca7	311742	3	65630826	65641459	10634	1018,44	0,19	7,94E-01
ENSRNOG00000001876	**Ccdc74a**	Protein Ccdc74a; RCG36565, isoform	680752	11	90601613	90605826	4214	40,96	-0,25	7,94E-01
ENSRNOG00000004342	**Ahr**	aryl hydrocarbon receptor (Ahr), mRN.	25690	6	64588008	64624107	36100	170,49	-0,24	7,94E-01
ENSRNOG00000009593	**Dtwd1**	DTW domain containing 1 (Dtwd1), ml	296119	3	124955129	124966716	11588	143,23	-0,18	7,94E-01
ENSRNOG00000020420		pyruvate kinase, liver and RBC (Pklr),	24651	2	207865576	207874393	8818	97,11	-0,19	7,94E-01
ENSRNOG00000021026	**Zfp687**	Protein Zfp687 [Source:UniProtKB/Tr	1E+08	2	215666721	215672448	5728	1015,18	-0,15	7,94E-01
ENSRNOG00000028749		Protein Atxn7l2 [Source:UniProtKB/Tr	310781	2	230462305	230470500	8196	341,55	0,14	7,94E-01
ENSRNOG00000030237	**LOC10036**	cytochrome c oxidase, subunit VIIc (C	1E+08	2	14553406	14555427	2022	6564,20	0,14	7,94E-01
ENSRNOG00000039463	**Pcdhga1**	protocadherin gamma subfamily A, 1 (	553129	18	30502814	30505234	2421	92,95	-0,29	7,94E-01
ENSRNOG00000045918	**Jarid2**	Protein LOC681740; RCG44016, isofo	681740	17	22195199	22367244	172046	1291,37	-0,12	7,94E-01
ENSRNOG00000033056	**LOC10036**	zinc finger protein 81 [Source:MGI Symbol;Acc:I		7	15991402	15992292	891	105,55	-0,21	7,95E-01
ENSRNOG00000014059	**Odf2l**	outer dense fiber of sperm tails 2-like (	685425	2	269522749	269560126	37378	125,36	-0,24	7,95E-01
ENSRNOG00000016571	**Ngf**	nerve growth factor (beta polypeptide)	310738	2	224316241	224369656	53416	33,73	-0,27	7,95E-01
ENSRNOG00000026931	**Ranbp6**	RAN binding protein 6 (Ranbp6), mRN	309326	1	255232607	255235924	3318	401,97	0,27	7,95E-01
ENSRNOG00000005936	**Foxm1**	forkhead box M1 (Foxm1), mRNA [Sou	58921	4	226891344	226902363	11020	548,41	0,18	7,95E-01
ENSRNOG00000006364	**Dld**	dihydrolipoamide dehydrogenase (Dld	298942	6	59273260	59293928	20669	1162,82	-0,26	7,95E-01
ENSRNOG00000009205	**Lmo4**	LIM domain only 4 (Lmo4), mRNA [So	362051	2	268752257	268775408	23152	4009,14	-0,17	7,95E-01
ENSRNOG00000010697	**Hadh**	hydroxyacyl-CoA dehydrogenase (Had	113965	2	254902140	254944001	41862	664,03	0,16	7,95E-01
ENSRNOG00000011311		bifunctional 3'-phosphoadenosine 5'-p	295443	2	255150454	255224840	74387	4088,13	0,09	7,95E-01
ENSRNOG00000015055	**Scg2**	secretogranin II (Scg2), mRNA [Sourc	24765	9	84991744	84997049	5306	1752,06	-0,15	7,95E-01
ENSRNOG00000042374	**Tgif2**	TGFB-induced factor homeobox 2 (Tg	499929	3	158772767	158787955	15189	113,80	-0,20	7,95E-01
ENSRNOG00000043212	**Dip2a**	DIP2 disco-interacting protein 2 homol	690211	20	15201216	15285083	83868	1813,52	0,12	7,95E-01
ENSRNOG00000011140	**Fam213a**	similar to RIKEN cDNA 5730469M10 (	361118	16	17376841	17396620	19780	1161,00	-0,19	7,96E-01
ENSRNOG00000012237		radixin [Source:RefSeq peptide;Acc:N	315655	8	55167300	55194096	26797	2448,79	0,16	7,96E-01
ENSRNOG00000017118	**P4ha3**	prolyl 4-hydroxylase, alpha polypeptid	361612	1	171496702	171531029	34328	117,02	-0,21	7,96E-01
ENSRNOG00000022712	**Bend7**	BEN domain containing 7 (Bend7), mF	361275	17	79104805	79183747	78943	78,56	-0,17	7,96E-01
ENSRNOG00000029893	**Rwdd3**	RWD domain containing 3 (Rwdd3), m	65026	2	242756616	242769365	12750	57,67	-0,25	7,96E-01
ENSRNOG00000019520	**Mettl6**	methyltransferase like 6 (Mettl6), mRN	290564	16	7507483	7523713	16231	373,33	0,14	7,96E-01
ENSRNOG00000027278	**Pramef8**	PRAME family member 8 (Pramef8), r	502994	5	165877921	165891643	13723	232,49	-0,22	7,96E-01
ENSRNOG00000007367		septin-4 [Source:RefSeq peptide;Acc:	287606	10	75217843	75227838	9996	144,27	-0,15	7,96E-01
ENSRNOG00000008664		Uncharacterized protein [Source:UniProtKB/TrE		15	8232279	8234013	1735	30,82	-0,26	7,96E-01
ENSRNOG00000016496	**Ctsc**	cathepsin C (Ctsc), mRNA [Source:Re	25423	1	158231466	158263672	32207	195,84	-0,23	7,96E-01
ENSRNOG00000006082	**Rmdn2**	regulator of microtubule dynamics 2 (F	313840	6	2200625	2264456	63832	20,91	-0,25	7,96E-01
ENSRNOG00000018349	**Polr1b**	polymerase (RNA) I polypeptide B (Pc	83582	3	128358264	128382727	24464	634,39	0,14	7,96E-01

ENSRNOG00000046143	**Perld1**	per1-like domain containing 1 (Perld1)	688174	10	86138993	86150849	11857	325,35	0,14	7,97E-01
ENSRNOG00000007875	**Actr6**	ARP6 actin-related protein 6 homolog	314718	7	30474273	30494826	20554	473,03	-0,18	7,97E-01
ENSRNOG00000023533	**Fam69a**	family with sequence similarity 69, me	360906	14	2789913	2860548	70636	674,74	-0,15	7,97E-01
ENSRNOG00000009022	**Rtkn**	rhotekin (Rtkn), mRNA [Source:RefSe	297383	4	179424134	179439771	15638	559,13	-0,27	7,97E-01
ENSRNOG00000022742	**Zfp954**	zinc finger protein 772 (Zfp772), mRN	308320	1	71250634	71256311	5678	205,66	0,16	7,97E-01
ENSRNOG00000002926	**Uap1**	UDP-N-acteylglucosamine pyrophospl	304954	13	93089458	93123827	34370	386,56	-0,17	7,97E-01
ENSRNOG00000006094	**Cd44**	Cd44 molecule (Cd44), mRNA [Source	25406	3	99339455	99426032	86578	764,14	-0,24	7,97E-01
ENSRNOG00000007751	**Gemin4**	gem (nuclear organelle) associated pr	497958	10	63637708	63645490	7783	334,67	0,14	7,97E-01
ENSRNOG00000019195	**Polh**	polymerase (DNA directed), eta (Polh)	316235	9	16059843	16093621	33779	248,28	0,15	7,97E-01
ENSRNOG00000033341	**MGC11619**	similar to RIKEN cDNA 1700001E04 (	367620	9	8132823	8147367	14545	54,66	-0,25	7,97E-01
ENSRNOG00000014276	**Plce1**	phospholipase C, epsilon 1 (Plce1), m	114633	1	264652842	264956663	303822	511,70	-0,19	7,97E-01
ENSRNOG00000030118	**Msn**	moesin (Msn), mRNA [Source:RefSeq	81521	X	66113012	66137455	24444	2897,06	0,19	7,97E-01
ENSRNOG00000001314	**Fam20c**	family with sequence similarity 20, me	304334	12	19906233	19965797	59565	2210,78	0,16	7,98E-01
ENSRNOG00000005601	**Pex13**	peroxisomal biogenesis factor 13 (Pex	305581	14	105229694	105246870	17177	428,93	0,17	7,98E-01
ENSRNOG00000010361	**Kif3b**	kinesin family member 3B (Kif3b), mR	296284	3	155284616	155324361	39746	4734,46	0,20	7,98E-01
ENSRNOG00000010595	**Rbm45**	RNA binding motif protein 45 (Rbm45)	266631	3	69794037	69809015	14979	677,22	0,18	7,98E-01
ENSRNOG00000013340	**LOC10091**	twisted gastrulation homolog 1 (Droso	363294	9	113230657	113263829	33173	1718,71	-0,17	7,98E-01
ENSRNOG00000015233	**Etfa**	electron-transfer-flavoprotein, alpha po	300726	8	58608433	58665406	56974	905,03	-0,22	7,98E-01
ENSRNOG00000020205		agrin (Agrn), mRNA [Source:RefSeq m	25592	5	177063801	177085091	21291	7554,17	-0,12	7,98E-01
ENSRNOG00000000826	**Flot1**	flotillin 1 (Flot1), mRNA [Source:RefSe	64665	20	5524710	5535207	10498	1942,17	0,11	7,98E-01
ENSRNOG00000006392	**Xrcc6**	X-ray repair complementing defective	25019	7	123244581	123265278	20698	771,85	0,15	7,98E-01
ENSRNOG00000007831	**Ufl1**	Ufm1-specific ligase 1 (Ufl1), mRNA [S	313115	5	44245076	44277549	32474	312,54	-0,25	7,98E-01
ENSRNOG00000010735	**Nfyc**	nuclear transcription factor-Y gamma (	25337	5	143482583	143542254	59672	1145,94	-0,13	7,98E-01
ENSRNOG00000037356		diphthamine biosynthesis 6 [Source:MGI Symbo		3	112579630	112708153	128524	138,21	-0,15	7,98E-01
ENSRNOG00000042458	**Stau2**	staufen double-stranded RNA binding	171500	5	2343373	2389279	45907	1826,12	-0,16	7,98E-01
ENSRNOG00000006000		cyclin-dependent kinase 12 (Cdk12), t	192350	10	85954655	86008029	53375	1095,95	0,11	7,98E-01
ENSRNOG00000020097	**Inha**	inhibin alpha (Inha), mRNA [Source:Re	24504	9	82469743	82472643	2901	177,67	0,15	7,98E-01
ENSRNOG00000023494	**Tti2**	TELO2 interacting protein 2 (Tti2), mR	290811	16	64394792	64402657	7866	508,17	0,18	7,98E-01
ENSRNOG00000026754	**Usp47**	ubiquitin specific peptidase 47 (Usp47	308896	1	183834046	183917156	83111	2700,63	0,23	7,98E-01
ENSRNOG00000048433	**Tshz2**	Protein LOC100911757 [Source:UniP	1E+08	3	172859967	172863020	3054	744,53	0,17	7,98E-01
ENSRNOG00000008824	**Vax1**	ventral anterior homeobox 1 (Vax1), m	64571	1	287695646	287699562	3917	73,04	-0,24	7,98E-01
ENSRNOG00000012362	**Cacng3**	calcium channel, voltage-dependent, g	140724	1	199676988	199771889	94902	159,09	-0,16	7,98E-01
ENSRNOG00000018228	**Cog2**	component of oligomeric golgi comple	690961	19	67991564	68023917	32354	636,22	-0,15	7,98E-01
ENSRNOG00000003219	**Trim16**	tripartite motif-containing 16 (Trim16),	303214	10	49011305	49033990	22686	179,37	0,19	7,98E-01
ENSRNOG00000013380	**Rhov**	ras homolog family member V (Rhov),	171581	3	117629288	117637987	8700	675,66	0,13	7,98E-01
ENSRNOG00000024136	**Fam151b**	family with sequence similarity 151, m	499507	2	41226510	41262309	35800	386,32	-0,12	7,98E-01
ENSRNOG00000034161		Cytochrome c oxidase subunit 6B1; Pr	681754	2	230806987	230807459	473	1101,42	0,14	7,98E-01
ENSRNOG00000012602	**Recql**	RecQ protein-like (DNA helicase Q1-lil	312824	4	240795680	240820165	24486	362,83	-0,15	7,99E-01

ENSRNOG00000016303		zinc finger protein 236 [Source:MGI Symbol;Acc		18	78517629	78608816	91188	580,62	0,17	8,00E-01
ENSRNOG00000003950	**Aqpep**	Protein Aqpep [Source:UniProtKB/TrE	502160	18	40527841	40601218	73378	40,97	-0,26	8,00E-01
ENSRNOG00000004402	**Lpgat1**	lysophosphatidylglycerol acyltransfera	679692	13	114962169	115028639	66471	1568,27	-0,16	8,00E-01
ENSRNOG00000005126	**Pqlc3**	PQ loop repeat containing 3 (Pqlc3), n	298906	6	52010004	52021578	11575	68,59	0,23	8,00E-01
ENSRNOG00000005849	**Aco1**	aconitase 1, soluble (Aco1), mRNA [S	50655	5	60972004	61027705	55702	3313,90	-0,11	8,00E-01
ENSRNOG00000012162	**Mpdu1**	mannose-P-dolichol utilization defect 1	303244	10	56007275	56012851	5577	1775,51	0,18	8,00E-01
ENSRNOG00000014365	**Gne**	glucosamine (UDP-N-acetyl)-2-epimer	114711	5	64035390	64065617	30228	864,23	0,18	8,00E-01
ENSRNOG00000018356	**Trim3**	tripartite motif-containing 3 (Trim3), m	83616	1	177447514	177470193	22680	2322,38	0,15	8,00E-01
ENSRNOG00000019550	**Slc11a2**	solute carrier family 11 (proton-couple	25715	7	139837654	139870639	32986	2798,15	0,18	8,00E-01
ENSRNOG00000019982	**Ethe1**	ethylmalonic encephalopathy 1 (Ethe1	292710	1	82716410	82731463	15054	370,44	0,16	8,00E-01
ENSRNOG00000043341		RIKEN cDNA 3110052M02 gene [Source:MGI S		1	62601559	62605710	4152	40,83	-0,23	8,00E-01
ENSRNOG00000043513	**Cyp4x1**	cytochrome P450, family 4, subfamily	246767	5	137721033	137750571	29539	299,40	0,24	8,00E-01
ENSRNOG00000049088	**Ttc32**	Protein LOC684830 [Source:UniProtK	684830	6	43935897	43942418	6522	102,50	0,16	8,00E-01
ENSRNOG00000019282	**Ubqln1**	ubiquilin 1 (Ubqln1), mRNA [Source:R	114590	17	9044249	9080134	35886	4453,28	0,10	8,00E-01
ENSRNOG00000027257	**Gmip**	Gem-interacting protein (Gmip), mRN	306357	16	21263641	21276944	13304	302,37	0,15	8,00E-01
ENSRNOG00000050419	**Avil**	advillin (Avil), mRNA [Source:RefSeq	79253	7	70468814	70486801	17988	110,35	0,22	8,00E-01
ENSRNOG00000020651	**Cars**	cysteinyl-tRNA synthetase (Cars), mR	293638	1	223619594	223661823	42230	3194,97	0,13	8,00E-01
ENSRNOG00000037225	**Tyms**	thymidylate synthetase (Tyms), mRNA	29261	9	121369214	121381507	12294	410,61	-0,19	8,00E-01
ENSRNOG00000046283	**Tmem41a**	transmembrane protein 41a (Tmem41	681708	11	85817837	85824489	6653	242,59	0,13	8,00E-01
ENSRNOG00000000380	**Sirt1**	Protein Sirt1 [Source:UniProtKB/TrEM	309757	20	8141451	8159506	18056	266,93	-0,17	8,00E-01
ENSRNOG00000011276	**Tmem254**	family with sequence similarity 213, m	290529	16	3783384	3787309	3926	1077,55	-0,22	8,00E-01
ENSRNOG00000016180	**Pdp1**	pyruvate dehyrogenase phosphatase	54705	5	30284176	30290060	5885	693,56	-0,19	8,00E-01
ENSRNOG00000021440	**Pptc7**	PTC7 protein phosphatase homolog (S	304488	12	41612148	41650388	38241	979,34	0,16	8,00E-01
ENSRNOG00000012705	**Pcgf2**	polycomb group ring finger 2 (Pcgf2),	287662	10	85420166	85424249	4084	642,89	0,13	8,00E-01
ENSRNOG00000014362	**Nudt14**	nudix (nucleoside diphosphate linked	299346	6	146734337	146741325	6989	479,49	0,22	8,01E-01
ENSRNOG00000007725	**Mis12**	MIS12 kinetochore complex compone	501706	10	57405280	57405900	621	191,95	-0,22	8,01E-01
ENSRNOG00000019240	**Ampd2**	adenosine monophosphate deaminas	362015	2	230330496	230341895	11400	2411,00	-0,12	8,01E-01
ENSRNOG00000023404	**Slc9b2**	solute carrier family 9, subfamily B (N	679958	2	259014874	259040106	25233	76,98	0,26	8,01E-01
ENSRNOG00000024243	**Cadm4**	cell adhesion molecule 4 (Cadm4), m	365216	1	82625738	82630379	4642	10368,44	0,15	8,01E-01
ENSRNOG00000001474	**Wbscr28**	Protein Wbscr28 [Source:UniProtKB/	288604	12	26803998	26808539	4542	57,94	-0,21	8,01E-01
ENSRNOG00000003285	**Mpv17l**	Mpv17 transgene, kidney disease mut	1E+08	10	2215802	2234250	18449	70,44	-0,24	8,01E-01
ENSRNOG00000005733	**Cpsf2**	cleavage and polyadenylation specific	299256	6	135081852	135109466	27615	2134,02	-0,15	8,01E-01
ENSRNOG00000010824	**Parp8**	Protein Parp8 [Source:UniProtKB/TrE	294762	2	67464107	67546909	82803	755,18	-0,14	8,01E-01
ENSRNOG00000011059	**Ttbk2**	tau tubulin kinase 2 (Ttbk2), mRNA [S	311349	3	119227873	119332743	104871	878,93	0,10	8,01E-01
ENSRNOG00000015511	**LOC10091**	5-aminoimidazole-4-carboxamide ribo	81643	9	78636339	78656386	20048	1482,06	0,14	8,01E-01
ENSRNOG00000030351	**LOC68552**	Protein LOC685520 [Source:UniProtK	25459	1	250371993	250373468	1476	60,04	-0,20	8,01E-01
ENSRNOG00000015720	**Polr2c**	polymerase (RNA) II (DNA directed) p	361365	19	10569862	10576683	6822	1004,39	-0,11	8,01E-01
ENSRNOG00000010629	**Nod1**	nucleotide-binding oligomerization dor	500133	4	149781209	149832175	50967	239,71	-0,24	8,01E-01

ENSRNOG00000007907	**Tmem178a**	transmembrane protein 178A (Tmem1	362691	6	3625175	3683554	58380	898,12	0,18	8,01E-01
ENSRNOG00000016545	**Ift140**	Protein Ift140 [Source:UniProtKB/TrE	1E+08	10	14192092	14276393	84302	975,15	-0,16	8,01E-01
ENSRNOG00000002574	**Nubp1**	nucleotide binding protein 1 (Nubp1),	287042	10	4155444	4166034	10591	445,98	0,20	8,01E-01
ENSRNOG00000011751	**Ncdn**	neurochondrin (Ncdn), mRNA [Source	89791	5	148539983	148549743	9761	12809,97	0,18	8,01E-01
ENSRNOG00000015440	**Wrn**	Protein Wrn [Source:UniProtKB/TrEM	290805	16	62174295	62279989	105695	126,94	-0,27	8,01E-01
ENSRNOG00000018841	**Sox8**	SRY (sex determining region Y)-box 8	302993	10	14745882	14750870	4989	1954,40	-0,27	8,01E-01
ENSRNOG00000003256	**Ccng1**	cyclin G1 (Ccng1), mRNA [Source:Ref	25405	10	25753833	25760534	6702	6407,31	0,17	8,01E-01
ENSRNOG00000033568	**Mapk8ip3**	mitogen-activated protein kinase 8 inte	302983	10	14076803	14115183	38381	5986,35	0,14	8,01E-01
ENSRNOG00000015999	**Cirbp**	cold inducible RNA binding protein (Ci	81825	7	12571310	12575127	3818	2217,63	0,20	8,02E-01
ENSRNOG00000018456	**LOC49875**	cDNA sequence BC005537 [Source:M	498750	17	44124794	44134878	10085	1560,65	0,16	8,02E-01
ENSRNOG00000004420	**Rad21**	RAD21 homolog (S. pombe) (Rad21),	314949	7	92155547	92182749	27203	5510,45	0,13	8,02E-01
ENSRNOG00000007023	**Galm**	galactose mutarotase (aldose 1-epime	313843	6	2785889	2837634	51746	323,04	-0,25	8,02E-01
ENSRNOG00000018848	**Eif1b**	eukaryotic translation initiation factor 1	301068	8	128356683	128359302	2620	3928,24	0,12	8,02E-01
ENSRNOG00000020351		vacuolar protein sorting 4 homolog A (	246772	19	50093779	50106670	12892	3726,74	0,16	8,02E-01
ENSRNOG00000018233	**Gas6**	growth arrest specific 6 (Gas6), mRNA	58935	16	80701561	80732410	30850	6518,76	-0,22	8,02E-01
ENSRNOG00000026252	**E2f7**	E2F transcription factor 7 (E2f7), mRN	314818	7	53286862	53326929	40068	148,51	0,22	8,02E-01
ENSRNOG00000043031	**RGD13056**	uncharacterized protein LOC314467		314467	6	145557161	145559139	1979	19,09	-0,27	8,02E-01
ENSRNOG00000016279	**Zfp865**	zinc finger protein 865 (Zfp865), mRN	308337	1	76174719	76179197	4479	263,07	-0,15	8,02E-01
ENSRNOG00000022323	**LOC49845**	transcription elongation factor A (SII) 1	362479	5	19293486	19330732	37247	303,75	-0,17	8,02E-01
ENSRNOG00000048708	**Exosc1**	Protein LOC679140 [Source:UniProtK	679140	1	268622701	268632262	9562	422,14	-0,14	8,02E-01
ENSRNOG00000015655	**Ptgfrn**	prostaglandin F2 receptor inhibitor (Pt	29602	2	222873519	222931395	57877	3209,46	-0,09	8,02E-01
ENSRNOG00000020925	**Ccdc86**	coiled-coil domain containing 86 (Ccd	293738	1	234061778	234067753	5976	506,23	0,14	8,02E-01
ENSRNOG00000029966	**Xrcc4**	X-ray repair complementing defective	309995	2	18553028	18803145	250118	146,63	-0,22	8,02E-01
ENSRNOG00000019629	**Lamp1**	lysosomal-associated membrane prote	25328	16	81175815	81192800	16986	13723,90	-0,17	8,02E-01
ENSRNOG00000000937	**Slc46a3**	solute carrier family 46, member 3 (Sl	288454	12	10842213	10858620	16408	103,91	0,17	8,02E-01
ENSRNOG00000002491				X	20664796	20665374	579	117,86	0,15	8,02E-01
ENSRNOG00000007333	**Wdr20**	WD repeat domain 20 (Wdr20), mRNA	314453	6	145187523	145256218	68696	609,97	0,15	8,02E-01
ENSRNOG00000013536	**Cdc42**	cell division cycle 42 (GTP binding pro	64465	5	159447307	159485241	37935	6212,33	-0,13	8,02E-01
ENSRNOG00000013770	**Folh1**	folate hydrolase 1 (Folh1), mRNA [Sou	85309	1	156631301	156702948	71648	194,31	-0,26	8,02E-01
ENSRNOG00000014264		phosphatase and actin regulator 1 [S	306844	17	25210472	25713154	502683	1950,71	0,13	8,02E-01
ENSRNOG00000015480	**Prkcz**	protein kinase C, zeta (Prkcz), mRNA	25522	5	176118465	176228206	109742	3048,84	0,18	8,02E-01
ENSRNOG00000015888	**Larp4b**	La ribonucleoprotein domain family, m	307070	17	65036457	65085258	48802	3684,03	-0,13	8,02E-01
ENSRNOG00000029582	**Lrch2**	Protein Lrch2 [Source:UniProtKB/TrE	680591	X	118678923	118759461	80539	551,83	-0,22	8,02E-01
ENSRNOG00000038622	**Zfp628**	Protein Zfp628 [Source:UniProtKB/Tr	1E+08	1	76067424	76070543	3120	387,46	0,15	8,02E-01
ENSRNOG00000048824	**Rnf165**	ring finger protein 165 (Rnf165), mRN	307251	18	73505861	73553878	48018	4207,83	0,13	8,02E-01
ENSRNOG00000000985	**Cpsf4**	cleavage and polyadenylation specific	304277	12	13269002	13284752	15751	327,56	0,16	8,03E-01
ENSRNOG00000018765	**Pold4**	polymerase (DNA-directed), delta 4, a	361698	1	226351249	226352915	1667	69,87	0,23	8,03E-01
ENSRNOG00000005796	**Ctnna1**	catenin (cadherin-associated protein),	307505	18	27633009	27766063	133055	2951,53	0,16	8,03E-01

ENSRNOG00000011847	**Grk4**	G protein-coupled receptor kinase 4 ((	59077	14	81953240	82026972	73733	150,28	0,17	8,03E-01
ENSRNOG00000017383		Protein Hikeshi [Source:UniProtKB/Sv	293103	1	160451593	160474922	23330	1249,87	0,11	8,03E-01
ENSRNOG00000030245	**Tango2**	transport and golgi organization 2 hon	360738	11	89887931	89934492	46562	846,60	0,16	8,03E-01
ENSRNOG00000015974	**Tmem208**	transmembrane protein 208 (Tmem20	291963	19	48147514	48150214	2701	2964,10	0,22	8,03E-01
ENSRNOG00000010922	**Ppp2r1b**	protein phosphatase 2, regulatory sub	315648	8	53876377	53897403	21027	1231,38	0,15	8,03E-01
ENSRNOG00000006112		zinc finger protein 120 [Source:MGI Symbol;Acc		3	152003144	152005643	2500	29,44	-0,28	8,03E-01
ENSRNOG00000013055	**Zfyve16**	zinc finger, FYVE domain containing 1	499508	2	41265679	41308896	43218	288,79	-0,23	8,03E-01
ENSRNOG00000026807		cDNA sequence BC030307 [Source:M	362863	7	27360100	27428178	68079	35,08	0,24	8,03E-01
ENSRNOG00000007727	**Lhfpl4**	lipoma HMGIC fusion partner-like 4 (L	353230	4	208415890	208445642	29753	941,92	0,15	8,03E-01
ENSRNOG00000017954	**Slmo1**	slowmo homolog 1 (Drosophila) (Slmo	690253	18	62388335	62401682	13348	335,36	0,16	8,03E-01
ENSRNOG00000006435	**Tor1b**	torsin family 1, member B (Tor1b), mR	311854	3	15151511	15157600	6090	680,82	0,18	8,03E-01
ENSRNOG00000008645	**Igfbp3**	insulin-like growth factor binding prote	24484	14	81091987	81099928	7942	1455,03	0,17	8,03E-01
ENSRNOG00000018204	**LOC69196(**	Protein LOC691960 [Source:UniProtK	691960	3	121177530	121185650	8121	17,56	-0,27	8,03E-01
ENSRNOG00000022922		solute carrier family 25 (mitochondrial carrier, Ar		3	64378093	64485534	107442	1721,09	0,17	8,03E-01
ENSRNOG00000031019	**RGD15643**	sprouty homolog 3 (Drosophila) (Spry3	498159	12	20930800	20931768	969	25,27	0,23	8,03E-01
ENSRNOG00000042160	**LOC10036(**	transmembrane protein 167B (Tmem1	499690	2	230818557	230822325	3769	114,49	0,23	8,03E-01
ENSRNOG00000043216	**Pemt**	phosphatidylethanolamine N-methyltra	25511	10	46096652	46161547	64896	76,53	0,24	8,03E-01
ENSRNOG00000007853	**Prdm10**	Protein Prdm10 [Source:UniProtKB/T	500964	8	32477808	32548005	70198	298,40	0,15	8,03E-01
ENSRNOG00000004437	**Map2k6**	mitogen-activated protein kinase kinas	114495	10	98413927	98527709	113783	536,66	0,17	8,03E-01
ENSRNOG00000014550	**Plcxd3**	phosphatidylinositol-specific phosphol	310358	2	73220198	73389440	169243	117,84	-0,24	8,03E-01
ENSRNOG00000004864	**Prpf40a**	PRP40 pre-mRNA processing factor 4	295607	3	43779312	43838952	59641	1482,42	-0,22	8,03E-01
ENSRNOG00000008686	**Tigd5**	tigger transposable element derived 5	300034	7	116835628	116838147	2520	255,82	0,15	8,03E-01
ENSRNOG00000011329	**Pkm**	pyruvate kinase, muscle (Pkm), mRN/	25630	8	64244179	64265969	21791	977,10	0,19	8,03E-01
ENSRNOG00000023993		Protein Kif1a [Source:UniProtKB/TrEI	363288	9	99830371	99893950	63580	22626,12	-0,12	8,03E-01
ENSRNOG00000007454	**Aloxe3**	arachidonate lipoxygenase 3 (Aloxe3)	287424	10	55455906	55478970	23065	136,16	0,27	8,03E-01
ENSRNOG00000022938	**Ap4e1**	adaptor-related protein complex 4, eps	311404	3	126010551	126074320	63770	366,95	-0,17	8,03E-01
ENSRNOG00000009958	**LOC10015**	uncharacterized protein LOC1001517(	1E+08	8	53637061	53647516	10456	2034,56	0,19	8,03E-01
ENSRNOG00000001448	**Hip1**	huntingtin interacting protein 1 (Hip1),	192154	12	26178240	26312676	134437	3092,37	-0,15	8,04E-01
ENSRNOG00000006519	**Tmem107**	transmembrane protein 107 (Tmem10	691750	10	55397006	55399330	2325	646,30	0,14	8,04E-01
ENSRNOG00000019434	**Adat1**	adenosine deaminase, tRNA-specific	690810	19	54994610	55017852	23243	155,94	0,17	8,04E-01
ENSRNOG00000023233		zinc finger protein 658 [Source:MGI Symbol;Acc		1	100555504	100565596	10093	209,49	-0,15	8,04E-01
ENSRNOG00000041608				1	85228074	85228192	119	28,52	-0,23	8,04E-01
ENSRNOG00000019684	**Fam173a**	family with sequence similarity 173, m	287150	10	14972980	14974838	1859	1185,20	0,19	8,04E-01
ENSRNOG00000046874		sorting nexin 9 [Source:RefSeq peptic	683687	1	48084884	48130893	46010	409,69	0,14	8,04E-01
ENSRNOG00000037227	**Yes1**	Yamaguchi sarcoma viral (v-yes) onco	24884	9	121252196	121327338	75143	267,61	-0,26	8,05E-01
ENSRNOG00000002122	**Lrrc8c**	leucine rich repeat containing 8 family,	289443	14	5278309	5369415	91107	371,52	-0,11	8,05E-01
ENSRNOG00000009264	**Erc1**	ELKS/RAB6-interacting/CAST family r	266806	4	219179492	219465108	285617	2164,88	-0,15	8,05E-01
ENSRNOG00000020770	**Arl4d**	ADP-ribosylation factor-like 4D (Arl4d)	303559	10	89375528	89377684	2157	464,70	-0,16	8,05E-01

ENSRNOG00000029986	**Insr**	insulin receptor (Insr), mRNA [Source:	24954	12	3852577	3989125	136549	1241,09	0,19	8,05E-01
ENSRNOG00000049975	**Zfp46**	zinc finger protein 46 (Zfp46), mRNA [	298558	5	158417871	158422371	4501	1848,86	0,17	8,05E-01
ENSRNOG00000049316		Protein Hs3st4 [Source:UniProtKB/TrEMBL;Acc		1	201294785	201303219	8435	56,38	-0,20	8,05E-01
ENSRNOG00000027697	**Nmnat2**	nicotinamide nucleotide adenylyltransf	289095	13	75346746	75525374	178629	1702,61	0,18	8,05E-01
ENSRNOG00000022781		coiled-coil serine rich 2 [Source:MGI S	306306	16	13981317	14070911	89595	1204,44	-0,19	8,05E-01
ENSRNOG00000006930	**Casq1**	calsequestrin 1 (fast-twitch, skeletal m	686019	13	95113786	95123307	9522	92,28	-0,18	8,06E-01
ENSRNOG00000048495		ribosomal RNA processing 12 homolo	679127	1	268559595	268592751	33157	832,05	0,16	8,06E-01
ENSRNOG00000024695	**Dtwd2**	DTW domain containing 2 (Dtwd2), ml	361326	14	46072253	46156847	84595	164,89	0,18	8,06E-01
ENSRNOG00000050828	**Vkorc1**	vitamin K epoxide reductase complex,	309004	1	206361382	206363943	2562	754,63	0,18	8,06E-01
ENSRNOG00000003220	**LOC10036**	Histone H3.3 [Source:UniProtKB/Swis	117056	13	104089839	104101388	11550	850,70	-0,10	8,06E-01
ENSRNOG00000011251	**Hcrtr2**	hypocretin (orexin) receptor 2 (Hcrtr2)	25605	8	82883206	82998507	115302	61,56	-0,21	8,06E-01
ENSRNOG00000019494	**Psmb10**	proteasome (prosome, macropain) sul	291983	19	48776577	48779068	2492	779,22	0,24	8,06E-01
ENSRNOG00000019507	**Hnrnpul2**	Protein Hnrnpul2 [Source:UniProtKB/	309197	1	231953988	231965766	11779	4689,60	0,11	8,06E-01
ENSRNOG00000000230	**Lmf1**	Protein Lmf1 [Source:UniProtKB/TrEM	360495	10	14758811	14844833	86023	411,93	0,14	8,06E-01
ENSRNOG00000016547	**Rgs19**	regulator of G-protein signaling 19 (Rg	59293	3	180905218	180910056	4839	447,17	-0,16	8,06E-01
ENSRNOG00000003073	**Gpr161**	Protein Gpr161 [Source:UniProtKB/Tr	289180	13	88434397	88450766	16370	480,21	0,19	8,06E-01
ENSRNOG00000016725	**Ikbkap**	inhibitor of kappa light polypeptide ger	140934	5	77661316	77710707	49392	3575,51	0,14	8,07E-01
ENSRNOG00000003050	**Tbc1d9b**	TBC1 domain family, member 9B (with	360520	10	35397655	35436143	38489	2234,76	-0,12	8,07E-01
ENSRNOG00000005176	**Map7d2**	Protein Map7d2 [Source:UniProtKB/T	317508	X	38706790	38777103	70314	824,52	-0,17	8,07E-01
ENSRNOG00000009199	**Accs**	1-aminocyclopropane-1-carboxylate sy	311218	3	89443360	89457535	14176	53,27	-0,19	8,07E-01
ENSRNOG00000000264	**Def8**	differentially expressed in FDCP 8 hor	307973	19	66954015	66969849	15835	1186,31	-0,11	8,07E-01
ENSRNOG00000042419	**Acyp2**	acylphosphatase 2, muscle type (Acyp	364224	14	114552149	114712779	160631	568,32	-0,16	8,07E-01
ENSRNOG00000022326	**Ccdc142**	Protein Ccdc142 [Source:UniProtKB/	297380	4	178620221	178626252	6032	343,45	0,16	8,07E-01
ENSRNOG00000009857	**Ftsj3**	FtsJ homolog 3 (E. coli) (Ftsj3), mRNA	303608	10	94190363	94196805	6443	956,83	0,16	8,08E-01
ENSRNOG00000036960		ATP-binding cassette sub-family C member 9 [S		4	241021683	241139051	117369	29,18	0,21	8,08E-01
ENSRNOG00000005038	**Ube2t**	ubiquitin-conjugating enzyme E2T (pu	360847	13	56842245	56852747	10503	132,33	0,21	8,08E-01
ENSRNOG00000014529		Uncharacterized protein [Source:UniProtKB/TrE		8	58292998	58348188	55191	480,40	-0,14	8,08E-01
ENSRNOG00000018653	**Pfdn1**	prefoldin subunit 1 (Pfdn1), mRNA [Sc	361310	18	28940034	28997301	57268	2184,40	-0,10	8,08E-01
ENSRNOG00000005958		ribosome binding protein 1 [Source:M	311483	3	144493295	144533705	40411	2714,90	-0,14	8,08E-01
ENSRNOG00000006228	**Pdia4**	protein disulfide isomerase family A, m	116598	4	142140946	142159845	18900	2279,33	0,21	8,08E-01
ENSRNOG00000008648	**Mogs**	glucosidase 1 (Gcs1), mRNA [Source:	78947	4	178633399	178636808	3410	915,38	0,11	8,08E-01
ENSRNOG00000007044	**L3mbtl1**	Lethal(3)malignant brain tumor-like pro	311613	3	165517651	165544071	26421	53,34	-0,23	8,08E-01
ENSRNOG00000002186	**Nudt9**	nudix (nucleoside diphosphate linked r	305149	14	7027806	7045536	17731	1145,77	0,12	8,08E-01
ENSRNOG00000008037	**Tmed1**	transmembrane emp24 protein transp	315461	8	22594535	22597120	2586	823,32	0,22	8,08E-01
ENSRNOG00000012290	**Gchfr**	GTP cyclohydrolase I feedback regula	171128	3	117526535	117530595	4061	31,60	-0,26	8,08E-01
ENSRNOG00000018012	**Tulp4**	tubby like protein 4 (Tulp4), mRNA [Sc	499016	1	48334822	48430485	95664	3519,26	0,16	8,08E-01
ENSRNOG00000037273	**Arhgap4**	Rho GTPase activating protein 4 (Arhg	246249	1	152621949	152636830	14882	75,38	0,23	8,09E-01
ENSRNOG00000018069	**Ngdn**	neuroguidin, EIF4E binding protein (N	305887	15	37564770	37571979	7210	620,74	0,21	8,09E-01

ENSRNOG00000021808	**RGD15600**	trans-2,3-enoyl-CoA reductase (Tecr),	191576	19	35684511	35711343	26833	7702,38	0,15	8,09E-01
ENSRNOG00000002171		pleckstrin homology-like domain, family B, meml		11	60566708	60644335	77628	171,65	-0,23	8,09E-01
ENSRNOG00000027286	**Fam211a**	Protein Fam211a [Source:UniProtKB/	691777	10	48713373	48727258	13886	146,69	-0,16	8,09E-01
ENSRNOG00000001863	**Hic2**	hypermethylated in cancer 2 (Hic2), m	287940	11	91042179	91071088	28910	291,86	0,20	8,09E-01
ENSRNOG00000001971	**LOC10091**	bobby sox homolog (Drosophila) (Bbx	303970	11	56250760	56397254	146495	129,20	-0,21	8,09E-01
ENSRNOG00000005618	**Fmc1**	formation of mitochondrial complexes	500087	4	66088284	66096317	8034	434,23	0,22	8,09E-01
ENSRNOG00000012778	**Magoh**	mago-nashi homolog, proliferation-ass	298385	5	131328664	131335510	6847	574,31	-0,16	8,09E-01
ENSRNOG00000012920		collagen alpha-1(IX) chain [Source:Re	305104	9	29243190	29325623	82434	26,74	0,26	8,09E-01
ENSRNOG00000018878	**Ssh3**	slingshot protein phosphatase 3 (Ssh3	365396	1	226382006	226389511	7506	287,31	-0,23	8,09E-01
ENSRNOG00000024545	**Ccdc18**	Protein Ccdc18 [Source:UniProtKB/Tr	305628	14	2509530	2609972	100443	25,24	-0,25	8,09E-01
ENSRNOG00000025155	**Lmtk2**	lemur tyrosine kinase 2 (Lmtk2), mRN	304286	12	14461531	14592856	131326	4119,06	-0,13	8,09E-01
ENSRNOG00000000442	**Notch4**	notch 4 (Notch4), mRNA [Source:RefS	406162	20	6409150	6433141	23992	371,67	0,17	8,09E-01
ENSRNOG00000020297	**Gon4l**	gon-4-like (C. elegans) (Gon4l), mRN	499653	2	207538842	207612074	73233	1868,14	0,13	8,09E-01
ENSRNOG00000022438	**Jmjd4**	jumonji domain containing 4 (Jmjd4), r	287359	10	45554645	45559869	5225	152,72	-0,13	8,09E-01
ENSRNOG00000012950	**Efr3b**	Protein Efr3b [Source:UniProtKB/TrEI	313928	6	38203470	38235681	32212	2106,25	-0,11	8,09E-01
ENSRNOG00000007213	**Yars**	tyrosyl-tRNA synthetase (Yars), mRNA	313047	5	151109967	151138472	28506	4028,30	0,12	8,10E-01
ENSRNOG00000018218	**Dusp22**	dual specificity phosphatase 22 (Dusp	361242	17	38367181	38417679	50499	126,55	0,19	8,10E-01
ENSRNOG00000019975	**Cdc5l**	cell division cycle 5-like (Cdc5l), mRN	85434	9	16838171	16876916	38746	1757,23	0,18	8,10E-01
ENSRNOG00000027249	**Fancl**	Fanconi anemia, complementation grc	305600	14	110369260	110433487	64228	136,08	-0,18	8,10E-01
ENSRNOG00000007159	**Ccl2**	chemokine (C-C motif) ligand 2 (Ccl2)	24770	10	69047091	69048889	1799	29,36	-0,26	8,10E-01
ENSRNOG00000010790	**Bnip2**	BCL2/adenovirus E1B interacting prot	300811	8	76662499	76675895	13397	607,74	0,21	8,10E-01
ENSRNOG00000023863	**Gpr139**	G protein-coupled receptor 139 (Gpr1	293545	1	195796295	195837147	40853	38,43	-0,27	8,10E-01
ENSRNOG00000036684	**Pcyt2**	phosphate cytidylyltransferase 2, etha	89841	10	109381452	109388764	7313	1807,84	0,19	8,10E-01
ENSRNOG00000007883	**Timm10**	translocase of inner mitochondrial mei	64464	3	78747542	78751013	3472	1470,99	0,19	8,10E-01
ENSRNOG00000009208	**Pibf1**	Protein Pibf1 [Source:UniProtKB/TrEN	306104	15	87005567	87327709	322143	150,38	-0,27	8,10E-01
ENSRNOG00000037437	**Golga3**	golgin A3 (Golga3), mRNA [Source:Re	312077	12	54248805	54295058	46254	195,19	0,16	8,10E-01
ENSRNOG00000002097	**Rasl11b**	RAS-like family 11 member B (Rasl11l	305302	14	36378166	36382381	4216	542,43	-0,18	8,10E-01
ENSRNOG00000009110	**Psen1**	presenilin 1 (Psen1), mRNA [Source:F	29192	6	118325068	118373047	47980	2557,00	0,19	8,10E-01
ENSRNOG00000011129	**Arhgef40**	Rho guanine nucleotide exchange fac	361034	15	32193215	32213812	20598	1738,45	0,13	8,10E-01
ENSRNOG00000014092	**Paip2b**	Protein Paip2b [Source:UniProtKB/Trl	312490	4	180076627	180082198	5572	318,09	0,19	8,10E-01
ENSRNOG00000019091		Wilms tumor 1 associated protein (Wt	499020	1	51782341	51806765	24425	936,73	-0,14	8,10E-01
ENSRNOG00000037595		Vasculin-like protein 1 [Source:UniPrc	313519	5	139209461	139235050	25590	70,05	0,22	8,10E-01
ENSRNOG00000028254	**Tmem141**	transmembrane protein 141 (Tmem14	499755	3	2825048	2827006	1959	165,23	0,19	8,10E-01
ENSRNOG00000038375	**LOC100360145**			16	71095553	71096359	807	287,49	0,14	8,10E-01
ENSRNOG00000002181	**Helq**	helicase, POLQ-like (Helq), mRNA [Sc	360912	14	10394947	10439008	44062	280,96	0,16	8,10E-01
ENSRNOG00000011262	**Gltscr1**	glioma tumor suppressor candidate re	292622	1	79133679	79158168	24490	943,91	0,12	8,11E-01
ENSRNOG00000002969		inositol-trisphosphate 3-kinase B [Sou	54260	13	103624308	103715736	91429	697,66	0,14	8,11E-01
ENSRNOG00000009930		phosphatidylinositol glycan anchor bio	313341	5	62987512	62994935	7424	381,32	0,16	8,11E-01

ENSRNOG00000017137	**Mettl10**	methyltransferase like 10 (Mettl10), m	361664	1	211824330	211848437	24108	348,77	-0,12	8,11E-01
ENSRNOG00000030024		zinc finger protein 36, C3H1 type-like	29344	6	115988971	115992268	3298	702,44	0,14	8,11E-01
ENSRNOG00000012686	**Pomc**	proopiomelanocortin (Pomc), mRNA [S	24664	6	38191989	38197809	5821	29,76	-0,27	8,11E-01
ENSRNOG00000006997	**App**	amyloid beta (A4) precursor protein (A	54226	11	28049096	28265240	216145	23171,72	0,17	8,11E-01
ENSRNOG00000004883	**Erh**	enhancer of rudimentary homolog (Dro	681415	6	112452339	112461605	9267	4523,35	0,09	8,11E-01
ENSRNOG00000016847	**Bace1**	beta-site APP cleaving enzyme 1 (Bac	29392	8	48766005	48788272	22268	1918,38	0,14	8,12E-01
ENSRNOG00000007925	**Pak6**	p21 protein (Cdc42/Rac)-activated kin	296078	3	117007787	117026619	18833	3057,51	-0,15	8,12E-01
ENSRNOG00000016408	**Kirrel**	kin of IRRE like (Drosophila) (Kirrel), n	310695	2	205830116	205884761	54646	306,21	0,14	8,12E-01
ENSRNOG00000018246	**Tdp2**	tyrosyl-DNA phosphodiesterase 2 (Tdp	498749	17	44097468	44108824	11357	281,10	0,17	8,12E-01
ENSRNOG00000013836		Rho GTPase activating protein 17 (Arh	63994	1	200297804	200387135	89332	443,51	-0,21	8,12E-01
ENSRNOG00000049128	**Nktr**	Protein LOC100364165 [Source:UniP	1E+08	8	129548839	129572815	23977	1256,98	-0,13	8,12E-01
ENSRNOG00000012881	**Fgl2**	fibrinogen-like 2 (Fgl2), mRNA [Source	84586	4	10315675	10321309	5635	89,71	-0,27	8,12E-01
ENSRNOG00000017254	**Nsun2**	NOP2/Sun RNA methyltransferase fam	361191	1	37692672	37716918	24247	1813,12	0,11	8,12E-01
ENSRNOG00000005673	**Runx1t1**	runt-related transcription factor 1; trans	362489	5	32018815	32131170	112356	1318,03	0,20	8,12E-01
ENSRNOG00000013611	**Krt32**	keratin 32 (Krt32), mRNA [Source:Ref	450230	10	87787319	87792744	5426	52,77	0,24	8,12E-01
ENSRNOG00000023764	**Kctd2**	Protein Kctd2 [Source:UniProtKB/TrE	498024	10	104295843	104304213	8371	356,31	0,14	8,13E-01
ENSRNOG00000028924		SET and MYND domain containing 3 [Source:M		13	102350017	102588971	238955	282,47	0,16	8,13E-01
ENSRNOG00000000974	**Zfp358**	zinc finger protein 358 (Zfp358), mRN	360754	12	4209518	4213611	4094	1385,89	0,16	8,13E-01
ENSRNOG00000009297	**Chchd1**	coiled-coil-helix-coiled-coil-helix doma	361005	15	8126681	8127855	1175	1148,72	0,11	8,13E-01
ENSRNOG00000010303	**Trim32**	tripartite motif-containing 32 (Trim32),	313264	5	85530389	85541239	10851	3172,30	-0,13	8,13E-01
ENSRNOG00000011526	**Pcsk6**	proprotein convertase subtilisin/kexin t	25507	1	127884080	128033742	149663	348,33	-0,20	8,13E-01
ENSRNOG00000019804	**B3gat3**	beta-1,3-glucuronyltransferase 3 (gluc	293722	1	232057681	232064199	6519	4792,69	0,22	8,13E-01
ENSRNOG00000026783	**Smyd4**	SET and MYND domain containing 4 (	287525	10	61904769	61951108	46340	274,71	-0,16	8,13E-01
ENSRNOG00000029958				18	14526050	14526694	645	92,09	-0,19	8,13E-01
ENSRNOG00000032328	**Diras2**	DIRAS family, GTP-binding RAS-like 2	291006	17	14854091	14855291	1201	789,27	-0,17	8,13E-01
ENSRNOG00000021232	**Ddrgk1**	DDRGK domain containing 1 (Ddrgk1)	296162	3	129693708	129704956	11249	1144,31	-0,14	8,13E-01
ENSRNOG00000039596		proline rich basic protein 1 [Source:MG	689207	18	28154937	28157990	3054	19,05	-0,25	8,13E-01
ENSRNOG00000046445		REST corepressor 3 [Source:RefSeq	684192	13	115381202	115421941	40740	242,46	0,15	8,13E-01
ENSRNOG00000033445		Uncharacterized protein [Source:UniProtKB/TrE		5	105772478	105809828	37351	21,62	-0,26	8,13E-01
ENSRNOG00000010523	**Naa20**	N(alpha)-acetyltransferase 20, NatB ca	362228	3	146527794	146545208	17415	1537,19	-0,13	8,13E-01
ENSRNOG00000001783	**Tra2b**	transformer 2 beta homolog (Drosophi	117259	11	85462041	85480254	18214	1351,97	-0,17	8,13E-01
ENSRNOG00000010024	**Cyb5r4**	cytochrome b5 reductase 4 (Cyb5r4),	171015	8	94201539	94266045	64507	733,30	0,18	8,13E-01
ENSRNOG00000036663	**Foxk2**	forkhead box K2 (Foxk2), mRNA [Sour	303753	10	110055948	110104742	48795	3443,55	-0,16	8,14E-01
ENSRNOG00000046731		voltage-dependent P/Q-type calcium c	25398	19	36502533	36725231	222699	2498,24	-0,15	8,14E-01
ENSRNOG00000043182	**Sept6**	septin 6 (Sept6), mRNA [Source:RefSe	691335	X	123673433	123745926	72494	1029,15	-0,14	8,14E-01
ENSRNOG00000002205	**Ociad1**	OCIA domain containing 1 (Ociad1), m	289590	14	37276461	37293850	17390	5623,93	0,19	8,14E-01
ENSRNOG00000016064	**Tkt**	transketolase (Tkt), mRNA [Source:Re	64524	16	6543527	6568461	24935	8023,08	0,13	8,14E-01
ENSRNOG00000001416	**LOC10090**	VGF nerve growth factor inducible (Vg	29461	12	24689888	24692911	3024	723,97	-0,23	8,14E-01

ENSRNOG00000002394	**Tpr**	translocated promoter region, nuclear	304862	13	72576529	72637648	61120	2185,33	0,23	8,14E-01
ENSRNOG00000002588	**Clec16a**	Protein Clec16a [Source:UniProtKB/T	287044	10	3829933	3987938	158006	877,78	0,12	8,14E-01
ENSRNOG00000002748	**Ccdc43**	coiled-coil domain containing 43 (Ccdc	360637	10	90434924	90447114	12191	743,31	0,12	8,14E-01
ENSRNOG00000004842	**Dnajc24**	DnaJ (Hsp40) homolog, subfamily C, r	362184	3	102647266	102687956	40691	185,75	-0,15	8,14E-01
ENSRNOG00000004917	**Rasal2**	RAS protein activator like 2 (Rasal2), r	304893	13	79688897	79840482	151586	369,68	0,11	8,14E-01
ENSRNOG00000006898	**Mrps16**	mitochondrial ribosomal protein S16 (M	688912	15	8452790	8455192	2403	920,80	0,18	8,14E-01
ENSRNOG00000007036	**Tgfbr1**	transforming growth factor, beta recep	29591	5	67575722	67639194	63473	448,18	-0,14	8,14E-01
ENSRNOG00000007923	**Cgref1**	cell growth regulator with EF hand dor	245918	6	36612741	36624744	12004	589,34	-0,17	8,14E-01
ENSRNOG00000008051	**Itpk1**	inositol-tetrakisphosphate 1-kinase (Itp	500709	6	135660943	135790645	129703	1073,96	-0,15	8,14E-01
ENSRNOG00000011502	**LOC10091(**	collagen, type IX, alpha 2 (Col9a2), m	362584	5	143756118	143773305	17188	100,08	0,16	8,14E-01
ENSRNOG00000015182	**Ppp2cb**	protein phosphatase 2, catalytic subur	24673	16	61933984	61955477	21494	5008,48	0,12	8,14E-01
ENSRNOG00000016390	**Eef1e1**	eukaryotic translation elongation facto	291057	17	28701069	28713201	12133	1259,42	-0,14	8,14E-01
ENSRNOG00000018748		solute carrier family 16, member 11 [S	287450	10	56567392	56571115	3724	50,31	-0,22	8,14E-01
ENSRNOG00000019430	**Coro1a**	coronin, actin binding protein 1A (Corc	155151	1	205102883	205107856	4974	3121,43	0,20	8,14E-01
ENSRNOG00000020054	**Pcdhb15**	Protein Pcdhb15 [Source:UniProtKB/T	291646	18	30258072	30260432	2361	145,26	-0,14	8,14E-01
ENSRNOG00000022555	**LOC49789!**	cDNA sequence BC049762 [Source:M	497899	10	36480645	36488292	7648	55,80	0,18	8,14E-01
ENSRNOG00000024849	**Tor1aip2**	torsin A interacting protein 2 (Tor1aip2	304881	13	78633864	78660388	26525	589,02	-0,15	8,14E-01
ENSRNOG00000010286	**Cast**	calpastatin (Cast), transcript variant 1,	25403	2	1422027	1530718	108692	429,78	-0,21	8,15E-01
ENSRNOG00000014241	**Ece1**	endothelin converting enzyme 1 (Ece1	94204	5	159968569	160070310	101742	971,10	0,15	8,15E-01
ENSRNOG00000000454	**RT1-DOb**	RT1 class II, locus DOb (RT1-DOb), m	365542	20	6099931	6105533	5603	41,61	0,24	8,15E-01
ENSRNOG00000012929	**Wsb1**	WD repeat and SOCS box-containing	303336	10	65046088	65062151	16064	2798,83	-0,25	8,15E-01
ENSRNOG00000008846	**Plag1**	pleiomorphic adenoma gene 1 (Plag1)	297804	5	21569212	21577465	8254	31,16	0,26	8,15E-01
ENSRNOG00000016924	**Acly**	ATP citrate lyase (Acly), transcript vari	24159	10	88185495	88236092	50598	12111,08	0,16	8,15E-01
ENSRNOG00000001190	**Git2**	G protein-coupled receptor kinase inte	304546	12	49383689	49424283	40595	1219,93	0,10	8,15E-01
ENSRNOG00000007982	**Slc9a1**	solute carrier family 9, subfamily A (NH	24782	5	155237482	155290406	52925	1157,76	0,13	8,15E-01
ENSRNOG00000012469	**Vwa9**	von Willebrand factor A domain contai	300782	8	70146546	70170129	23584	971,76	0,16	8,16E-01
ENSRNOG00000012502	**Stk17b**	serine/threonine kinase 17b (Stk17b),	170904	9	60085947	60115674	29728	217,67	-0,20	8,16E-01
ENSRNOG00000027888	**Cmss1**	cms1 ribosomal small subunit homolog	288176	11	48227871	48525826	297956	221,20	0,20	8,16E-01
ENSRNOG00000038068	**Pcdh9**	protocadherin 9 (Pcdh9), mRNA [Sour	306091	15	81283365	81286488	3124	780,48	-0,14	8,16E-01
ENSRNOG00000045941		RIKEN cDNA 4933426M11 gene [Sou	678893	6	112646965	112698358	51394	338,89	0,15	8,16E-01
ENSRNOG00000048838		Gap junction gamma-1 protein [Sourc	266706	10	90573583	90576548	2966	618,27	-0,11	8,16E-01
ENSRNOG00000001243	**Zcchc8**	zinc finger, CCHC domain containing 8	288661	12	40191351	40200212	8862	626,30	-0,11	8,16E-01
ENSRNOG00000018659	**Csf1**	colony stimulating factor 1 (macrophag	78965	2	229989435	230017945	28511	592,70	-0,22	8,16E-01
ENSRNOG00000012795	**Cables1**	Cdk5 and Abl enzyme substrate 1 (Ca	307585	18	3328555	3432388	103834	475,50	0,16	8,16E-01
ENSRNOG00000015594	**Rftn2**	Protein Rftn2 [Source:UniProtKB/TrEI	363231	9	61439705	61493694	53990	650,63	-0,22	8,16E-01
ENSRNOG00000003590	**Tom1l2**	target of myb1-like 2 (chicken) (Tom1l2	360537	10	46367588	46475907	108320	1858,34	-0,14	8,16E-01
ENSRNOG00000013485	**Alg5**	ALG5, dolichyl-phosphate beta-glucos	295051	2	160317035	160330762	13728	433,45	0,12	8,16E-01
ENSRNOG00000020153	**Supt3h**	Protein Supt3h [Source:UniProtKB/Tr	685697	9	17205632	17478325	272694	280,28	0,18	8,16E-01

ENSRNOG00000002833	**Gsk3b**	glycogen synthase kinase 3 beta (Gsk	84027	11	66873996	67016493	142498	2991,52	0,13	8,16E-01
ENSRNOG00000003576	**Ints2**	integrator complex subunit 2 (Ints2), m	360589	10	73559143	73589442	30300	250,54	0,21	8,16E-01
ENSRNOG00000005624	**Zfx**	zinc finger protein X-linked (Zfx), mRN	367832	X	63750387	63796284	45898	178,61	-0,20	8,16E-01
ENSRNOG00000007127	**Bbs5**	Bardet-Biedl syndrome 5 (Bbs5), mRN	362142	3	62498548	62519537	20990	173,71	-0,16	8,16E-01
ENSRNOG00000009739	**RGD15644**	Protein RGD1564420 [Source:UniPro	362919	7	100210512	100254422	43911	1973,40	-0,15	8,16E-01
ENSRNOG00000010330	**Mark3**	MAP/microtubule affinity-regulating kir	170577	6	144287323	144374797	87475	4174,57	0,13	8,16E-01
ENSRNOG00000010447	**Tmod2**	tropomodulin 2 (Tmod2), mRNA [Sour	58814	8	82109739	82135088	25350	2153,45	-0,16	8,16E-01
ENSRNOG00000012932	**Ccdc146**	coiled-coil domain containing 146 (Ccc	499980	4	10360967	10414944	53978	18,52	0,23	8,16E-01
ENSRNOG00000013148		RAD54 like (S. cerevisiae) (Rad54l), n	298429	5	138732243	138762035	29793	116,27	0,18	8,16E-01
ENSRNOG00000013521	**Dhfr**	dihydrofolate reductase (Dhfr), mRNA	24312	2	41137857	41163224	25368	206,51	0,19	8,16E-01
ENSRNOG00000013721	**RGD13114**	uncharacterized protein LOC303948		303948	11	65032121	65044284	12164	91,18	-0,16	8,16E-01
ENSRNOG00000021093	**Syngr4**	synaptogyrin 4 (Syngr4), mRNA [Sour	292916	1	102951507	102962708	11202	18,53	0,26	8,16E-01
ENSRNOG00000023053	**RGD13097**	Protein RGD1309747 [Source:UniPro	306665	1	36680380	36718654	38275	2891,83	-0,13	8,16E-01
ENSRNOG00000025310	**Pop7**	processing of precursor 7, ribonucleas	288564	12	24276481	24277522	1042	858,83	-0,15	8,16E-01
ENSRNOG00000033261	**Fam107a**	family with sequence similarity 107, m	361018	15	22284696	22387948	103253	607,20	-0,25	8,16E-01
ENSRNOG00000002229	**Adcy5**	adenylate cyclase 5 (Adcy5), mRNA [S	64532	11	71787104	71933840	146737	3010,96	0,16	8,16E-01
ENSRNOG00000006575	**Ccdc96**	Protein Ccdc96 [Source:UniProtKB/Tr	289716	14	79082553	79084481	1929	69,70	-0,19	8,16E-01
ENSRNOG00000008180	**Lyn**	v-yes-1 Yamaguchi sarcoma viral relat	81515	5	21307089	21422838	115750	138,03	-0,22	8,16E-01
ENSRNOG00000011435	**Osbpl10**	Oxysterol-binding protein [Source:Uni	316039	8	122531508	122593466	61959	284,73	0,15	8,16E-01
ENSRNOG00000025793	**Usp31**	ubiquitin specific peptidase 31 (Usp31	308959	1	198532743	198568863	36121	863,16	-0,15	8,16E-01
ENSRNOG00000031040	**LOC10091**	trophinin associated protein (Troap), m	300219	X	115263537	115271228	7692	304,08	0,21	8,16E-01
ENSRNOG00000003049	**Hsd17b10**	hydroxysteroid (17-beta) dehydrogena	63864	X	21747939	21750384	2446	2083,92	0,19	8,17E-01
ENSRNOG00000004309	**Actn1**	actinin, alpha 1 (Actn1), mRNA [Sourc	81634	6	116056248	116149475	93228	1502,13	0,11	8,17E-01
ENSRNOG00000014666	**Tfeb**	transcription factor EB (Tfeb), mRNA [	316214	9	14132622	14139303	6682	110,03	-0,25	8,17E-01
ENSRNOG00000015852		Rho GTPase activating protein 35 (Arl	306400	1	79753058	79769240	16183	5462,18	-0,14	8,17E-01
ENSRNOG00000046109		PL-5283 protein isoform 1 precursor [	689574	4	62503531	62513357	9827	1113,35	0,18	8,17E-01
ENSRNOG00000008329	**Ndufb11**	NADH dehydrogenase (ubiquinone) 1	299310	X	2580319	2582577	2259	4310,37	0,20	8,17E-01
ENSRNOG00000016638	**Gnb1**	guanine nucleotide binding protein (G	24400	5	176410631	176455109	44479	7769,03	0,16	8,17E-01
ENSRNOG00000017901	**Acy3**	aspartoacylase (aminocyclase) 3 (Acy	293653	1	226104425	226107749	3325	181,32	-0,23	8,17E-01
ENSRNOG00000000703	**Ficd**	FIC domain containing (Ficd), mRNA [	288741	12	50441769	50444594	2826	299,43	-0,16	8,17E-01
ENSRNOG00000001182	**Ndufv3**	NADH dehydrogenase (ubiquinone) fla	64539	20	12455139	12464611	9473	1429,33	0,12	8,17E-01
ENSRNOG00000002970	**Zswim7**	zinc finger, SWIM-type containing 7 (Z	287388	10	48375624	48387713	12090	40,19	-0,21	8,17E-01
ENSRNOG00000005700	**Nsg1**	neuron specific gene family member 1	25247	14	77362124	77383868	21745	21023,09	0,15	8,17E-01
ENSRNOG00000011599	**Gldc**	glycine dehydrogenase (decarboxylati	309312	1	255544864	255624575	79712	1119,65	-0,19	8,17E-01
ENSRNOG00000016509	**Usp10**	ubiquitin specific peptidase 10 (Usp10	307905	19	63314211	63356421	42211	2296,62	0,13	8,17E-01
ENSRNOG00000037402	**Myeov2**	myeloma overexpressed 2 (Myeov2),	681389	9	99478497	99483427	4931	3825,74	0,17	8,17E-01
ENSRNOG00000000692		uracil-DNA glycosylase [Source:RefS	304577	12	50028792	50037073	8282	330,49	0,18	8,18E-01
ENSRNOG00000016021	**Lims2**	LIM and senescent cell antigen like dc	361303	18	24422408	24460482	38075	174,09	-0,19	8,18E-01

ENSRNOG00000017532	**Tesk1**	testis-specific kinase 1 (Tesk1), mRNA	29460	5	63463575	63469304	5730	1710,22	0,15	8,18E-01
ENSRNOG00000039654	**LOC100911361**			X	65620518	65621681	1164	136,79	0,21	8,18E-01
ENSRNOG00000037456	**P2rx2**	purinergic receptor P2X, ligand-gated	114115	12	54133185	54136379	3195	29,07	-0,22	8,18E-01
ENSRNOG00000002021		Son DNA binding protein (Son), transc	304092	11	35415351	35446496	31146	4899,55	-0,16	8,18E-01
ENSRNOG00000009042	**Sec24c**	SEC24 family, member C (S. cerevisia	685144	15	8136414	8158375	21962	4423,77	0,14	8,18E-01
ENSRNOG00000021569		T cell lymphoma invasion and metastasis 1 [Sou		11	33551577	33671896	120320	1231,25	0,15	8,18E-01
ENSRNOG00000037282	**Pdzd4**	PDZ domain containing 4 (Pdzd4), mR	293856	1	152711938	152741657	29720	4690,65	-0,14	8,18E-01
ENSRNOG00000048695		Uncharacterized protein [Source:UniProtKB/TrE		6	119175318	119187901	12584	40,77	-0,20	8,18E-01
ENSRNOG00000003146	**Fopnl**	FGFR1OP N-terminal like (Fopnl), mR	360461	10	11412943	11433757	20815	557,44	-0,11	8,19E-01
ENSRNOG00000008775	**Lrguk**	leucine-rich repeats and guanylate kin	296968	4	61143344	61248921	105578	33,11	-0,23	8,19E-01
ENSRNOG00000021931	**Uba6**	ubiquitin-like modifier activating enzym	305268	14	23412097	23475036	62940	381,40	-0,21	8,19E-01
ENSRNOG00000043107	**Abhd10**	abhydrolase domain containing 10 (Ab	303953	11	60649543	60661662	12120	333,08	0,20	8,19E-01
ENSRNOG00000050849	**Leprot**	leptin receptor overlapping transcript (	56766	5	124375762	124388209	12448	1596,66	0,21	8,19E-01
ENSRNOG00000002072	**Eva1c**	Protein Fam176c [Source:UniProtKB/	360695	11	34614323	34687047	72725	20,95	0,26	8,19E-01
ENSRNOG00000005371	**Klhl29**	kelch-like family member 29 (Klhl29), i	298867	6	40403632	40706610	302979	693,40	-0,17	8,19E-01
ENSRNOG00000011162	**Smco4**	single-pass membrane protein with co	363020	8	14139544	14159122	19579	59,74	-0,19	8,19E-01
ENSRNOG00000012109	**Fam105a**	family with sequence similarity 105, m	310190	2	100047744	100070970	23227	49,47	-0,23	8,19E-01
ENSRNOG00000012665	**Nmrk1**	nicotinamide riboside kinase 1 (Nmrk1	499330	1	241821771	241849122	27352	115,99	-0,21	8,19E-01
ENSRNOG00000014305	**Fbxo42**	F-box protein 42 (Fbxo42), mRNA [So	362646	5	163382899	163441506	58608	879,45	0,11	8,19E-01
ENSRNOG00000017564	**Mib2**	mindbomb E3 ubiquitin protein ligase 2	474147	5	176558731	176565084	6354	2021,16	0,11	8,19E-01
ENSRNOG00000018857	**Abcd1**	ATP-binding cassette, subfamily D (AL	363516	1	152823436	152844829	21394	376,59	-0,13	8,19E-01
ENSRNOG00000019020	**Bbs2**	Bardet-Biedl syndrome 2 (Bbs2), mRN	113948	19	11360527	11395931	35405	656,23	0,17	8,19E-01
ENSRNOG00000019447	**Ecel1**	endothelin converting enzyme-like 1 (E	60417	9	93966150	93975953	9804	135,82	-0,25	8,19E-01
ENSRNOG00000031679	**Zkscan3**	zinc finger with KRAB and SCAN dom	306977	17	58204679	58220497	15819	962,23	-0,14	8,19E-01
ENSRNOG00000033837	**Cdh9**	cadherin 9 (Cdh9), mRNA [Source:Re	29163	2	86724081	86810246	86166	69,39	-0,23	8,19E-01
ENSRNOG00000033970	**Moap1**	modulator of apoptosis 1 (Moap1), mR	299261	6	135826374	135828304	1931	1327,15	0,17	8,19E-01
ENSRNOG00000046862	**Armcx1**	armadillo repeat containing, X-linked 1	501619	X	105798563	105803308	4746	26,29	0,20	8,19E-01
ENSRNOG00000014313		myeloblastosis oncogene [Source:MGI Symbol;A		1	17818418	17848370	29953	58,64	-0,26	8,19E-01
ENSRNOG00000017496	**Cnp**	2',3'-cyclic nucleotide 3' phosphodieste	25275	10	88284045	88290603	6559	5174,44	0,25	8,19E-01
ENSRNOG00000012386	**Zbtb38**	zinc finger and BTB domain containing	315936	8	104036362	104039973	3612	495,59	0,13	8,19E-01
ENSRNOG00000027300		tetratricopeptide repeat protein 7B [So	362768	6	133541064	133653972	112909	1203,21	-0,11	8,19E-01
ENSRNOG00000005388	**Rassf3**	Ras association (RalGDS/AF-6) doma	362886	7	63737800	63801753	63954	348,04	0,16	8,20E-01
ENSRNOG00000011984	**Cxcl14**	chemokine (C-X-C motif) ligand 14 (C:	306748	17	11271210	11279229	8020	1759,92	-0,22	8,20E-01
ENSRNOG00000028623	**Agpat5**	1-acylglycerol-3-phosphate O-acyltran	306582	16	75417609	75460357	42749	1181,26	0,19	8,20E-01
ENSRNOG00000004540	**LOC10091**	C-type lectin domain family 3, membe	1E+08	8	131394766	131400452	5687	31,12	-0,26	8,20E-01
ENSRNOG00000006005	**Pus10**	pseudouridylate synthase 10 (Pus10),	305583	14	105247077	105284030	36954	95,82	0,18	8,20E-01
ENSRNOG00000039776	**Exoc3**	exocyst complex component 3 (Exoc3	252881	1	33126575	33156980	30406	1903,43	0,17	8,20E-01
ENSRNOG00000007194	**Cpsf3**	cleavage and polyadenylation specific	298916	6	60231498	60259421	27924	1196,28	0,11	8,20E-01

ENSRNOG00000016334	**Ptbp3**	polypyrimidine tract binding protein 3 (	83515	5	80811877	80893823	81947	296,30	-0,18	8,20E-01
ENSRNOG00000018677	**Akt2**	v-akt murine thymoma viral oncogene	25233	1	85627946	85666362	38417	1538,59	0,13	8,20E-01
ENSRNOG00000039777	**Ttf1**	Homeobox protein Nkx-2.1 [Source:U	499766	3	12988356	13006428	18073	179,50	-0,15	8,20E-01
ENSRNOG00000039856	**Lrrc73**	leucine rich repeat containing 73 (Lrrc	501101	9	16007854	16010854	3001	1267,37	0,18	8,20E-01
ENSRNOG00000047192	**Polr2m**	polymerase (RNA) II (DNA directed) p	192147	19	300201	437406	137206	141,07	0,15	8,20E-01
ENSRNOG00000047427	**Cdan1**	Protein Cdan1 [Source:UniProtKB/TrE	311348	3	119207104	119220303	13200	492,84	0,11	8,20E-01
ENSRNOG00000027126	**Dpy30**	dpy-30 homolog (C. elegans) (Dpy30)	286897	6	32188946	32203027	14082	1466,93	0,12	8,20E-01
ENSRNOG00000013727	**Ndc80**	NDC80 kinetochore complex compone	301701	9	119293740	119324796	31057	213,74	0,25	8,21E-01
ENSRNOG00000018711	**Ppcdc**	phosphopantothenoylcysteine decarbo	363069	8	60649506	60673125	23620	228,84	0,12	8,21E-01
ENSRNOG00000008181	**Chek1**	checkpoint kinase 1 (Chek1), mRNA [S	140583	8	39184021	39204447	20427	188,49	-0,16	8,21E-01
ENSRNOG00000006615	**Mtap**	methylthioadenosine phosphorylase (M	298227	5	111679661	111746208	66548	139,96	-0,14	8,21E-01
ENSRNOG00000002480	**Gpr137b**	G protein-coupled receptor 137B (Gpr	289287	17	92351953	92362368	10416	231,03	0,16	8,21E-01
ENSRNOG00000006683	**Nedd4**	neural precursor cell expressed, devel	25489	8	73679021	73716822	37802	26718,32	0,11	8,21E-01
ENSRNOG00000008209	**St3gal1**	ST3 beta-galactoside alpha-2,3-sialylt	362924	7	107847816	107854483	6668	409,26	-0,16	8,21E-01
ENSRNOG00000013660	**Cog6**	component of oligomeric golgi comple	310411	2	161883500	161920986	37487	666,89	0,20	8,21E-01
ENSRNOG00000014647	**Cbfb**	core-binding factor, beta subunit (Cbfb	361391	19	47993674	48037234	43561	1124,38	0,17	8,21E-01
ENSRNOG00000015735	**Zswim1**	Protein Zswim1; RCG32422, isoform (	311631	3	167475675	167477042	1368	307,00	0,13	8,21E-01
ENSRNOG00000020433	**Actn4**	actinin alpha 4 (Actn4), mRNA [Source	63836	1	88258638	88327926	69289	4722,70	0,09	8,21E-01
ENSRNOG00000025028	**Prkdc**	Protein Prkdc [Source:UniProtKB/TrE	360748	11	92347523	92565000	217478	346,35	0,24	8,21E-01
ENSRNOG00000036700	**Fscn2**	fascin homolog 2, actin-bundling prote	303741	10	109128055	109134594	6540	68,45	0,17	8,21E-01
ENSRNOG00000001611	**Rpl24**	ribosomal protein L24 (Rpl24), mRNA	64307	11	50298776	50304145	5370	4960,34	0,17	8,22E-01
ENSRNOG00000004013		glutamate receptor interacting protein	84016	7	64890318	65081385	191068	1274,84	-0,12	8,22E-01
ENSRNOG00000006545		septin 7 (Sept7), transcript variant 1, n	64551	8	26435007	26498715	63709	909,93	-0,25	8,22E-01
ENSRNOG00000025286	**Pank2**	pantothenate kinase 2 (Pank2), mRNA	296167	3	130303289	130337378	34090	611,78	0,12	8,22E-01
ENSRNOG00000028075	**LOC36047**	RIKEN cDNA 4930451G09 gene [Sou	360479	10	9562157	9575513	13357	20,60	-0,22	8,22E-01
ENSRNOG00000042492	**Rfwd2**	ring finger and WD repeat domain 2, E	360860	13	81891069	81986189	95121	1671,58	0,13	8,22E-01
ENSRNOG00000009097	**Immt**	inner membrane protein, mitochondria	312444	4	164531449	164567175	35727	1672,89	0,22	8,22E-01
ENSRNOG00000009302		Protein Zfp623; RCG60016 [Source:UniProtKB/		7	116873069	116874568	1500	454,68	0,18	8,22E-01
ENSRNOG00000009949	**Pcdh18**	protocadherin 18 (Pcdh18), mRNA [So	295027	2	158301955	158315635	13681	525,35	-0,19	8,22E-01
ENSRNOG00000016680	**Nsd1**	nuclear receptor binding SET domain	306764	17	11953813	12062583	108771	3496,78	-0,12	8,22E-01
ENSRNOG00000017960	**Deaf1**	DEAF1 transcription factor (Deaf1), m	83632	1	221201338	221234579	33242	2569,06	0,17	8,22E-01
ENSRNOG00000018153	**Rbm5**	RNA binding motif protein 5 (Rbm5), n	300996	8	115857799	115886421	28623	2722,20	-0,18	8,22E-01
ENSRNOG00000020385	**Fads3**	fatty acid desaturase 3 (Fads3), mRN	286922	1	233037626	233054184	16559	1062,28	0,12	8,22E-01
ENSRNOG00000026944	**Gpr150**	G protein-coupled receptor 150 (Gpr1	499486	2	2811666	2812949	1284	37,74	-0,19	8,22E-01
ENSRNOG00000045926	**Lrrc45**	leucine rich repeat containing 45 (Lrrc	688318	10	109485832	109493737	7906	610,58	-0,10	8,22E-01
ENSRNOG00000010967	**Cdc37l1**	cell division cycle 37-like 1 (Cdc37l1),	293886	1	254349958	254376747	26790	1303,21	0,17	8,22E-01
ENSRNOG00000008027	**Murc**	muscle-related coiled-coil protein (Mu	313225	5	68926895	68934739	7845	35,14	-0,20	8,23E-01
ENSRNOG00000003887	**Lgi2**	leucine-rich repeat LGI family, membe	305417	14	60876468	60903945	27478	359,29	0,16	8,23E-01

ENSRNOG00000008106	**Shmt2**	serine hydroxymethyltransferase 2 (mi	299857	7	70998104	71003174	5071	1955,63	-0,22	8,23E-01
ENSRNOG00000011566	**Cecr2**	cat eye syndrome chromosome regior	500308	4	220306982	220412138	105157	238,94	0,15	8,23E-01
ENSRNOG00000016416	**LOC10036**	ribonuclease/angiogenin inhibitor 1 [S	1E+08	1	221068622	221076550	7929	1622,09	0,19	8,23E-01
ENSRNOG00000016890	**Tppp3**	tubulin polymerization-promoting prote	291966	19	48289941	48293620	3680	333,47	-0,24	8,23E-01
ENSRNOG00000017731	**Agpat4**	1-acylglycerol-3-phosphate O-acyltran	170919	1	50930058	51035498	105441	2062,38	0,17	8,23E-01
ENSRNOG00000022146	**Hist1h2af**	histone cluster 1, H2af (Hist1h2af), mF	498753	17	45481622	45482014	393	20,86	-0,24	8,23E-01
ENSRNOG00000023579		Protein Tet2 [Source:UniProtKB/TrEM	310859	2	257255362	257279069	23708	927,88	0,14	8,23E-01
ENSRNOG00000023765		neuronal tyrosine-phophorylated phosphoinositic		9	86726986	86863468	136483	214,99	0,19	8,23E-01
ENSRNOG00000019140	**Banp**	Btg3 associated nuclear protein (Banp	292064	19	65485733	65559757	74025	664,36	0,13	8,23E-01
ENSRNOG00000006822	**Cep290**	centrosomal protein 290 (Cep290), mF	314787	7	40256312	40344956	88645	118,57	0,24	8,23E-01
ENSRNOG00000006921	**Rbl1**	retinoblastoma-like 1 (p107) (Rbl1), m	680111	3	158398177	158452482	54306	158,24	0,20	8,23E-01
ENSRNOG00000045703		Eph receptor A10 [Source:MGI Symbol;Acc:MGI		11	45068264	45248899	180636	47,70	-0,17	8,23E-01
ENSRNOG00000009946	**Ldlr**	low density lipoprotein receptor (Ldlr),	300438	8	22804325	22827199	22875	714,34	0,24	8,23E-01
ENSRNOG00000027736	**Cnn1**	calponin 1, basic, smooth muscle (Cnı	65204	8	23167863	23176532	8670	83,35	-0,24	8,23E-01
ENSRNOG00000029698	**Pim3**	pim-3 oncogene (Pim3), mRNA [Sourc	64534	7	129544794	129547908	3115	985,98	0,21	8,24E-01
ENSRNOG00000014571	**Dbndd2**	dysbindin (dystrobrevin binding proteir	499941	3	167115995	167118753	2759	779,65	0,20	8,24E-01
ENSRNOG00000018143	**Hpdl**	4-hydroxyphenylpyruvate dioxygenase	313521	5	139472196	139473311	1116	30,28	-0,25	8,24E-01
ENSRNOG00000019150	**Polb**	polymerase (DNA directed), beta (Polk	29240	16	73869833	73895208	25376	1402,86	0,16	8,24E-01
ENSRNOG00000026132	**Trdmt1**	tRNA aspartic acid methyltransferase	291324	17	82445133	82477970	32838	230,94	-0,25	8,24E-01
ENSRNOG00000005556	**Snrpf**	small nuclear ribonucleoprotein polype	680737	7	34543253	34549956	6704	1033,88	0,17	8,24E-01
ENSRNOG00000030199	**Tmem192**	transmembrane protein 192 (Tmem19	361137	16	26503238	26515689	12452	359,03	0,17	8,24E-01
ENSRNOG00000008360	**Pcgf1**	polycomb group ring finger 1 (Pcgf1),	312480	4	178596101	178598690	2590	212,99	0,18	8,24E-01
ENSRNOG00000009936	**Dido1**	Protein Dido1 [Source:UniProtKB/TrE	362286	3	179864373	179894768	30396	1903,91	-0,10	8,24E-01
ENSRNOG00000000504	**Fance**	Fanconi anemia, complementation grc	309643	20	10095446	10106503	11058	752,06	0,15	8,24E-01
ENSRNOG00000000900		tyrosylprotein sulfotransferase 1 (Tpst	288617	12	31983442	32043407	59966	850,53	-0,14	8,24E-01
ENSRNOG00000007628	**Ptp4a3**	protein tyrosine phosphatase type IVA	362930	7	114918557	114924152	5596	660,16	0,14	8,24E-01
ENSRNOG00000007671	**Smu1**	smu-1 suppressor of mec-8 and unc-5	117541	5	61576564	61595134	18571	1300,38	0,13	8,24E-01
ENSRNOG00000009888	**Timm8b**	translocase of inner mitochondrial mei	64372	8	53635064	53636450	1387	1962,96	0,14	8,24E-01
ENSRNOG00000011544	**Zfp219**	zinc finger protein 219 (Zfp219), mRN.	305848	15	32213649	32217278	3630	1828,43	0,16	8,24E-01
ENSRNOG00000014066	**Phf17**	PHD finger protein 17 (Phf17), mRNA	310352	2	148061395	148114462	53068	538,59	-0,11	8,24E-01
ENSRNOG00000015318	**Heyl**	hairy/enhancer-of-split related with YR	313575	5	144713817	144730808	16992	30,35	-0,25	8,24E-01
ENSRNOG00000018242	**Camkk1**	calcium/calmodulin-dependent protein	60341	10	59324561	59347636	23076	784,04	-0,16	8,24E-01
ENSRNOG00000031661	**Zfp839**	Protein Zfp839 [Source:UniProtKB/Tr	500723	6	145126873	145143286	16414	145,22	0,17	8,24E-01
ENSRNOG00000037509	**Chek2**	checkpoint kinase 2 (Chek2), mRNA [	114212	12	53584455	53615983	31529	112,96	0,16	8,24E-01
ENSRNOG00000041449				7	122793216	122793269	54	27,01	0,20	8,24E-01
ENSRNOG00000000657	**Nek7**	NIMA-related kinase 7 (Nek7), mRNA	360850	13	60518085	60586290	68206	228,79	-0,13	8,24E-01
ENSRNOG00000001375	**Gal3st4**	galactose-3-O-sulfotransferase 4 (Gal	498166	12	21639959	21648595	8637	224,78	-0,19	8,24E-01
ENSRNOG00000001875	**Smpd4**	sphingomyelin phosphodiesterase 4, r	303790	11	90575599	90599309	23711	1099,97	0,09	8,24E-01

ENSRNOG00000005342	**Rassf5**	Ras association (RalGDS/AF-6) doma	54355	13	53032716	53098045	65330	182,31	0,15	8,24E-01
ENSRNOG00000008586		aldehyde dehydrogenase 1 family, member L2 [S		7	26506321	26555206	48886	286,25	-0,16	8,24E-01
ENSRNOG00000012447	**Setd6**	SET domain containing 6 (Setd6), mR	291844	19	9747567	9750562	2996	251,87	0,15	8,24E-01
ENSRNOG00000013121		mesoderm induction early response pr	310086	2	62316534	62339547	23014	323,38	-0,19	8,24E-01
ENSRNOG00000018561	**Pprc1**	peroxisome proliferator-activated rece	294007	1	273241077	273257483	16407	1193,93	0,15	8,24E-01
ENSRNOG00000019245		SAGA-associated factor 29 homolog	293488	1	205046259	205079492	33234	361,08	0,12	8,24E-01
ENSRNOG00000021480	**Rad9b**	RAD9 homolog B (S. pombe) (Rad9b)	363924	12	41579810	41610543	30734	74,40	-0,20	8,24E-01
ENSRNOG00000043288	**LOC69080**	Small integral membrane protein 13 [S	690806	17	23323000	23337383	14384	102,98	0,15	8,24E-01
ENSRNOG00000043439	**Zfp707**	zinc finger protein 707 (Zfp707), mRN	362936	7	116983080	116993715	10636	31,46	0,20	8,24E-01
ENSRNOG00000049302		chromodomain helicase DNA binding protein 9 [S		19	28495974	28508536	12563	93,42	0,18	8,24E-01
ENSRNOG00000050122				2	123892553	123893209	657	42,31	0,22	8,24E-01
ENSRNOG00000051011	**LOC68165**	Protein LOC681658 [Source:UniProtK	681658	4	145618483	145620206	1724	18,72	-0,22	8,24E-01
ENSRNOG00000003105		kinesin family member 19A [Source:MGI Symbo		10	102966408	102991701	25294	147,21	0,20	8,25E-01
ENSRNOG00000014450	**Shprh**	SNF2 histone linker PHD RING helica	308282	1	6891253	6965104	73852	2127,51	0,11	8,25E-01
ENSRNOG00000003150	**Mpc2**	mitochondrial pyruvate carrier 2 (Mpc2	1E+08	13	88560644	88579857	19214	3114,65	0,13	8,25E-01
ENSRNOG00000005234	**Gxylt1**	glucoside xylosyltransferase 1 (Gxylt1	300173	7	134118984	134166045	47062	585,24	0,14	8,25E-01
ENSRNOG00000006204	**Slc30a3**	solute carrier family 30 (zinc transporte	366568	6	36457851	36464874	7024	366,07	-0,17	8,25E-01
ENSRNOG00000007318	**Tmx1**	thioredoxin-related transmembrane pr	362751	6	102311253	102321357	10105	3929,95	0,19	8,25E-01
ENSRNOG00000009673	**RGD13055**	Protein RGD1305508 [Source:UniPro	303275	10	66299994	66302981	2988	349,42	-0,17	8,25E-01
ENSRNOG00000009845	**Acadm**	acyl-CoA dehydrogenase, C-4 to C-12	24158	2	278788485	278812656	24172	281,85	-0,19	8,25E-01
ENSRNOG00000010418		NIMA (never in mitosis gene a)-relatec	290705	16	32155914	32274325	118412	213,32	-0,22	8,25E-01
ENSRNOG00000012828	**Emg1**	EMG1 N1-specific pseudouridine meth	312706	4	224239981	224248243	8263	1146,29	0,14	8,25E-01
ENSRNOG00000014646		heparan-alpha-glucosaminide N-acetyltransferas		16	70540588	70573814	33227	1104,69	-0,10	8,25E-01
ENSRNOG00000016551		BCL2-like 11 (apoptosis facilitator) (Bc	64547	3	126571721	126601829	30109	67,35	-0,17	8,25E-01
ENSRNOG00000019453		ribosomal protein S5 (Rps5), transcrip	25538	1	66471610	66475879	4270	9439,29	0,17	8,25E-01
ENSRNOG00000042592	**Rgs10**	regulator of G-protein signaling 10 (Rg	54290	1	206829266	206870538	41273	1230,32	-0,19	8,25E-01
ENSRNOG00000046958	**Abo**	ABO blood group (transferase A, alpha	1E+08	3	9729919	9749616	19698	81,49	0,20	8,25E-01
ENSRNOG00000050504		nucleolar protein 11 [Source:MGI Symbol;Acc:M		10	95128388	95146244	17857	678,40	0,14	8,25E-01
ENSRNOG00000013314	**Avl9**	Protein Avl9; RCG52383 [Source:Unil	312371	4	151679026	151718420	39395	587,24	0,16	8,25E-01
ENSRNOG00000017471	**Rspry1**	ring finger and SPRY domain containi	689249	19	10764919	10820883	55965	642,52	-0,16	8,25E-01
ENSRNOG00000024705	**Rarres2**	retinoic acid receptor responder (tazar	297073	4	142869496	142872500	3005	64,55	-0,25	8,25E-01
ENSRNOG00000045545	**Eid2b**	Protein Eid2b [Source:UniProtKB/TrE	499112	1	86720613	86721255	643	189,60	0,19	8,25E-01
ENSRNOG00000045712				3	158611530	158612194	665	53,50	0,20	8,25E-01
ENSRNOG00000016566	**Arhgap39**	Rho GTPase activating protein 39 (Arl	500901	7	117776694	117869344	92651	1819,23	0,13	8,25E-01
ENSRNOG00000003081	**Ribc1**	RIB43A domain with coiled-coils 1 (Rib	317431	X	21736681	21747812	11132	147,25	-0,18	8,25E-01
ENSRNOG00000003426		lysine (K)-specific demethylase 5C [Sc	317432	X	64231964	64277128	45165	2870,52	0,09	8,25E-01
ENSRNOG00000014573	**Ckmt1b**	creatine kinase, mitochondrial 1B (Ckr	29593	3	119859003	119864004	5002	638,33	0,14	8,25E-01
ENSRNOG00000018766	**Rrp8**	ribosomal RNA processing 8, methyltr	308911	1	177580524	177584545	4022	188,79	0,14	8,25E-01

ENSRNOG00000020678	**Ifi35**	interferon-induced protein 35 (Ifi35), m	287719	10	89156392	89164642	8251	339,86	0,24	8,25E-01
ENSRNOG00000043210		protein phosphatase 3, regulatory sub	29748	14	100178534	100227295	48762	8549,33	0,18	8,25E-01
ENSRNOG00000001699	**Setd4**	SET domain containing 4 (Setd4), mR	245975	11	37374248	37394377	20130	625,85	0,17	8,25E-01
ENSRNOG00000031092		Rho-associated coiled-coil containing	81762	18	1292383	1432455	140073	771,08	-0,18	8,25E-01
ENSRNOG00000047356	**Abl1**	c-abl oncogene 1, non-receptor tyrosir	311860	3	15401182	15504438	103257	379,22	-0,11	8,25E-01
ENSRNOG00000032327	**Pdia5**	protein disulfide isomerase family A, m	360722	11	71580311	71666271	85961	47,09	0,21	8,26E-01
ENSRNOG00000027091	**Kctd18**	potassium channel tetramerization dor	301436	9	64649839	64714190	64352	1033,84	-0,16	8,26E-01
ENSRNOG00000005145		uncharacterized protein LOC299909		299909	7	91517912	91581982	64071	3745,96	-0,15	8,26E-01
ENSRNOG00000005307		solute carrier family 4, sodium bicarbo	295645	3	54662473	54932969	270497	548,57	0,17	8,26E-01
ENSRNOG00000010313		astrotactin 2 (Astn2), mRNA [Source:F	1E+08	5	85643128	86266662	623535	291,98	0,13	8,26E-01
ENSRNOG00000012258	**Rras2**	related RAS viral (r-ras) oncogene hor	365355	1	185908264	185978450	70187	1031,15	-0,17	8,26E-01
ENSRNOG00000019018	**Plat**	plasminogen activator, tissue (Plat), m	25692	16	73730256	73754851	24596	1641,42	-0,19	8,26E-01
ENSRNOG00000030183	**Plod2**	procollagen lysine, 2-oxoglutarate 5-di	300901	8	99462925	99545578	82654	477,88	-0,15	8,26E-01
ENSRNOG00000045705	**LOC10091**	RAS-like, family 10, member B (Rasl1	303382	10	71089431	71099354	9924	30,17	0,22	8,26E-01
ENSRNOG00000048978	**Nphp3**	nephronophthisis 3 (adolescent) (Nph	363126	8	111913834	111963488	49655	125,66	-0,16	8,26E-01
ENSRNOG00000027030	**Adm**	adrenomedullin (Adm), mRNA [Source	25026	1	182433109	182435280	2172	64,60	-0,25	8,26E-01
ENSRNOG00000005220		Uncharacterized protein [Source:UniProtKB/TrE		6	108326951	108353338	26388	70,77	0,18	8,26E-01
ENSRNOG00000008577		Fas (TNFRSF6) binding factor 1 [Sour	287836	10	103567053	103592829	25777	675,52	0,11	8,26E-01
ENSRNOG00000019228	**Pik3r2**	phosphoinositide-3-kinase, regulatory	29741	16	20273500	20282048	8549	5661,51	0,14	8,26E-01
ENSRNOG00000030266	**Plekhg2**	pleckstrin homology domain containin	292750	1	86601601	86614808	13208	627,79	0,16	8,26E-01
ENSRNOG00000033192	**Osmr**	oncostatin M receptor (Osmr), mRNA	310132	2	75851664	75892056	40393	45,51	-0,25	8,26E-01
ENSRNOG00000047984	**Mrps22**	mitochondrial ribosomal protein S22 (I	683519	8	106047831	106060998	13168	490,11	-0,13	8,26E-01
ENSRNOG00000014368	**Eif4g3**	eukaryotic translation initiation factor 4	298573	5	160146094	160313421	167328	5112,51	-0,13	8,27E-01
ENSRNOG00000000796	**LOC10091**	RAN binding protein 2 (Ranbp2), mRN	294429	20	29846539	29896149	49611	1391,77	0,25	8,27E-01
ENSRNOG00000006508	**Thnsl2**	threonine synthase-like 2 (S. cerevisia	297332	4	163753104	163771684	18581	161,31	-0,22	8,27E-01
ENSRNOG00000005609	**Neurod1**	neuronal differentiation 1 (Neurod1), m	29458	3	72974863	72978179	3317	295,70	-0,25	8,27E-01
ENSRNOG00000019429	**LOC10091**	Protein Dip2b [Source:UniProtKB/TrE	300231	X	116297431	116379696	82266	2748,57	0,11	8,28E-01
ENSRNOG00000005589		dehydrogenase/reductase (SDR family) membe		6	104960581	104979170	18590	848,51	0,20	8,28E-01
ENSRNOG00000016090	**Mtmr10**	myotubularin related protein 10 (Mtmr	309255	1	126233793	126284907	51115	1259,35	-0,17	8,28E-01
ENSRNOG00000025811	**Cfp**	complement factor properdin (Cfp), ml	299314	X	2126107	2131703	5597	73,16	-0,24	8,28E-01
ENSRNOG00000007944	**Edem1**	Protein Edem1 [Source:UniProtKB/Tr	297504	4	217499573	217528646	29074	670,59	0,17	8,28E-01
ENSRNOG00000012106	**Dnaja4**	DnaJ (Hsp40) homolog, subfamily A, r	300721	8	57859314	57875055	15742	413,80	0,18	8,28E-01
ENSRNOG00000012757		NIMA (never in mitosis gene a)-related expresse		16	74416297	74436641	20345	127,38	-0,15	8,28E-01
ENSRNOG00000014705	**Rbm20**	RNA binding motif protein 20 (Rbm20)	309544	1	281801481	282003053	201573	41,81	-0,24	8,28E-01
ENSRNOG00000016790	**Suv420h1**	suppressor of variegation 4-20 homolc	361688	1	225874424	225923708	49285	1996,89	-0,13	8,28E-01
ENSRNOG00000000622	**LOC68442**	histidine triad nucleotide binding prote	684427	10	39980470	39984241	3772	2556,42	0,19	8,28E-01
ENSRNOG00000012126	**Stard3nl**	STARD3 N-terminal like (Stard3nl), ml	291182	17	55791552	55825490	33939	2524,08	0,11	8,28E-01
ENSRNOG00000016379	**Jtb**	jumping translocation breakpoint (Jtb),	29439	2	209024504	209028715	4212	992,51	0,11	8,28E-01

ENSRNOG00000025885		Protein RGD1305422 [Source:UniPro	303885	11	74236038	74282108	46071	2350,28	0,13	8,28E-01
ENSRNOG00000043357	**Zfp407**	Protein Zfp407 [Source:UniProtKB/Trl	307213	18	80335599	80473948	138350	256,62	0,15	8,28E-01
ENSRNOG00000046535		Protein LOC686298 [Source:UniProtk	686298	8	114252464	114256314	3851	119,47	-0,16	8,28E-01
ENSRNOG00000014785	**Ykt6**	YKT6 v-SNARE homolog (S. cerevisia	64351	14	86880204	86889524	9321	2212,40	0,09	8,28E-01
ENSRNOG00000002449	**Maged2**	melanoma antigen, family D, 2 (Magec	113947	5	37832723	37840896	8174	3257,34	-0,11	8,29E-01
ENSRNOG00000003281	**RGD15605**	DNA helicase MCM9 [Source:UniProt	499437	20	36606873	36687699	80827	224,29	-0,12	8,29E-01
ENSRNOG00000006063	**Phf21a**	Protein Phf21a [Source:UniProtKB/Tr	362166	3	87933979	87975301	41323	859,42	-0,12	8,29E-01
ENSRNOG00000010141	**Ttll1**	tubulin tyrosine ligase-like family, mem	362969	7	124327306	124356545	29240	1112,89	-0,13	8,29E-01
ENSRNOG00000012477	**Eef1a2**	eukaryotic translation elongation facto	24799	3	180367139	180376314	9176	4716,40	0,15	8,29E-01
ENSRNOG00000013654	**Cbln2**	cerebellin 2 precursor (Cbln2), mRNA	291388	18	82838798	82844063	5266	765,15	0,23	8,29E-01
ENSRNOG00000014747		neurobeachin [Source:MGI Symbol;Ac	361948	2	164425380	164825633	400254	4548,57	0,12	8,29E-01
ENSRNOG00000019367	**Dclre1b**	DNA cross-link repair 1B (Dclre1b), ml	310745	2	225708799	225717309	8511	595,95	-0,10	8,29E-01
ENSRNOG00000019584	**Dlk1**	delta-like 1 homolog (Drosophila) (Dlk	114587	6	142742285	142749166	6882	49,06	-0,23	8,29E-01
ENSRNOG00000020457		transforming, acidic coiled-coil contain	309025	1	208087506	208360445	272940	4444,42	0,13	8,29E-01
ENSRNOG00000020584	**Efna3**	Protein Efna3 [Source:UniProtKB/TrE	170901	2	208048648	208055648	7001	636,34	-0,16	8,29E-01
ENSRNOG00000024008	**Cdc25c**	cell division cycle 25C (Cdc25c), mRN	307511	18	27243054	27263496	20443	180,49	0,19	8,29E-01
ENSRNOG00000049957	**Ddx17**	DEAD (Asp-Glu-Ala-Asp) box helicase	315133	7	120754843	120758011	3169	4790,14	-0,19	8,29E-01
ENSRNOG00000005021	**Orc4**	origin recognition complex, subunit 4 (	295596	3	38201916	38240567	38652	356,81	-0,24	8,29E-01
ENSRNOG00000020946	**Ccdc12**	coiled-coil domain containing 12 (Ccdc	363151	8	118267351	118317922	50572	652,12	0,13	8,29E-01
ENSRNOG00000008305	**Sc5d**	sterol-C5-desaturase (Sc5d), mRNA [S	114100	8	45001503	45010434	8932	2554,74	-0,18	8,29E-01
ENSRNOG00000013718	**Herc2**	HECT and RLD domain containing E3	308669	1	115459547	115658973	199427	6077,01	0,15	8,29E-01
ENSRNOG00000009802	**Fam124a**	Protein Fam124a [Source:UniProtKB/	691938	15	49481137	49546175	65039	725,99	-0,14	8,29E-01
ENSRNOG00000011346	**Ehd2**	EH-domain containing 2 (Ehd2), mRN	361512	1	79112280	79130529	18250	206,99	-0,23	8,29E-01
ENSRNOG00000012410	**S100a1**	S100 calcium binding protein A1 (S10	295214	2	209331131	209333683	2553	612,69	-0,24	8,29E-01
ENSRNOG00000013364	**Hey2**	hairy/enhancer-of-split related with YR	155430	1	30633675	30643762	10088	219,79	-0,16	8,29E-01
ENSRNOG00000015018	**Pdia3**	protein disulfide isomerase family A, m	29468	3	119916837	119941739	24903	8467,32	0,10	8,29E-01
ENSRNOG00000015088		Protein prune homolog 2 [Source:Uni	293823	1	243982264	244061511	79248	561,79	0,22	8,29E-01
ENSRNOG00000015269	**Atf7**	activating transcription factor 7 (Atf7),	315333	7	141928320	142016018	87699	281,78	-0,14	8,29E-01
ENSRNOG00000020517	**Prrg2**	proline rich Gla (G-carboxyglutamic ac	361570	1	102112490	102119928	7439	26,81	-0,22	8,29E-01
ENSRNOG00000042732		Uncharacterized protein [Source:UniProtKB/TrE		4	205769022	205769540	519	198,55	0,19	8,29E-01
ENSRNOG00000012760	**Chtop**	similar to DKFZP547E1010 protein (C	361990	2	209318321	209329789	11469	3981,03	-0,14	8,29E-01
ENSRNOG00000016888	**Pask**	PAS domain containing serine/threonii	301617	9	100106951	100194898	87948	315,31	0,18	8,29E-01
ENSRNOG00000000397	**Ccar1**	cell division cycle and apoptosis regul	361849	20	34157349	34199950	42602	735,30	-0,18	8,29E-01
ENSRNOG00000006565	**Fstl4**	follistatin-like 4 (Fstl4), mRNA [Source	303130	10	37877767	38313673	435907	123,10	-0,15	8,29E-01
ENSRNOG00000008244		RIKEN cDNA 2700049A03 gene [Sou	690035	6	103029034	103132204	103171	351,11	-0,11	8,29E-01
ENSRNOG00000010989	**Ipo5**	Protein Ipo5 [Source:UniProtKB/TrEM	306182	15	109653549	109688633	35085	5693,65	-0,11	8,29E-01
ENSRNOG00000012393	**S100a13**	S100 calcium binding protein A13 (S1	295213	2	209336486	209342980	6495	837,91	-0,23	8,29E-01
ENSRNOG00000016411	**LOC68948**	ribosomal protein S12 (Rps12), mRNA	65139	1	24229986	24232144	2159	110,03	0,15	8,29E-01

ENSRNOG00000019508	**Wars2**	tryptophanyl tRNA synthetase 2 (mitoc	690654	2	220640807	220721461	80655	148,07	0,17	8,29E-01
ENSRNOG00000019834	**LOC10090**	heat shock protein 90 alpha (cytosolic	301252	9	16706486	16712003	5518	27319,96	0,09	8,29E-01
ENSRNOG00000020788		EH domain binding protein 1-like 1 (Eh	309169	1	227997206	228017354	20149	908,46	-0,13	8,29E-01
ENSRNOG00000024641	**Nyap1**	neuronal tyrosine-phosphorylated pho	304376	12	24043357	24054629	11273	1329,91	0,14	8,29E-01
ENSRNOG00000047734	**Chst2**	Protein Chst2 [Source:UniProtKB/TrE	367145	8	102648882	102650474	1593	3167,97	-0,11	8,29E-01
ENSRNOG00000049137		Uncharacterized protein [Source:UniProtKB/TrE		18	15662254	15663001	748	27,56	-0,24	8,29E-01
ENSRNOG00000005165	**Smcr8**	Smith-Magenis syndrome chromosom	497918	10	46791502	46798641	7140	645,92	0,14	8,29E-01
ENSRNOG00000010895	**Tmem30a**	transmembrane protein 30A (Tmem30	300857	8	86767828	86789807	21980	1881,36	-0,11	8,29E-01
ENSRNOG00000005987	**Suox**	sulfite oxidase (Suox), mRNA [Source	81805	7	3070930	3073497	2568	888,06	0,14	8,29E-01
ENSRNOG00000007610	**Gdf11**	growth differentiation factor 11 (Gdf11)	29454	7	3280685	3286883	6199	338,47	-0,14	8,29E-01
ENSRNOG00000010516	**Plau**	plasminogen activator, urokinase (Plau	25619	15	3621553	3627467	5915	51,87	-0,23	8,29E-01
ENSRNOG00000014080	**Kif23**	kinesin family member 23 (Kif23), mRI	315740	8	66598467	66624304	25838	332,59	0,18	8,29E-01
ENSRNOG00000047296		Falz protein; Protein Bptf [Source:UniProtKB/Tr		10	94990564	95002294	11731	527,39	0,12	8,29E-01
ENSRNOG00000012473		CASP8 and FADD-like apoptosis regu	117279	9	65342448	65389220	46773	236,83	-0,17	8,29E-01
ENSRNOG00000018032	**Pafah1b2**	platelet-activating factor acetylhydrola	64189	8	48884455	48902445	17991	2814,92	0,16	8,29E-01
ENSRNOG00000010817	**Celf6**	CUGBP, Elav-like family member 6 (C	300758	8	64126553	64191454	64902	833,84	0,14	8,30E-01
ENSRNOG00000025923	**Zmiz2**	zinc finger, MIZ-type containing 2 (Zmi	289783	14	87476042	87492529	16488	5732,97	-0,13	8,30E-01
ENSRNOG00000031950	**Izumo4**	Protein Izumo4 [Source:UniProtKB/Tr	362832	7	12043098	12045489	2392	78,54	-0,15	8,30E-01
ENSRNOG00000049681	**Mapre2**	microtubule-associated protein, RP/EB family, m		18	15548085	15549586	1502	7100,11	0,12	8,30E-01
ENSRNOG00000011798	**Mapre1**	microtubule-associated protein, RP/EE	114764	3	155599601	155627491	27891	9680,21	0,14	8,30E-01
ENSRNOG00000000613	**Tfam**	transcription factor A, mitochondrial (T	83474	20	20760387	20772300	11914	1138,37	0,14	8,30E-01
ENSRNOG00000002253	**Wdr5b**	WD repeat domain 5B (Wdr5b), mRN/	303907	11	70733911	70734897	987	56,22	-0,16	8,30E-01
ENSRNOG00000004512	**Apool**	apolipoprotein O-like (Apool), mRNA [	317191	X	83639376	83708626	69251	66,58	-0,22	8,30E-01
ENSRNOG00000005496	**Ing3**	inhibitor of growth family, member 3 (I	312154	4	48812271	48837948	25678	316,73	-0,13	8,30E-01
ENSRNOG00000014048		ubiquitin carboxyl-terminal hydrolase (	312937	19	30298400	30348647	50248	128,65	-0,18	8,30E-01
ENSRNOG00000014515	**Cdc14a**	cell division cycle 14A (Cdc14a), trans	310806	2	237760156	237913699	153544	95,79	-0,17	8,30E-01
ENSRNOG00000016863	**Pnmal2**	paraneoplastic Ma antigen family-like	308393	1	80123763	80126337	2575	5068,67	0,13	8,30E-01
ENSRNOG00000027326	**Tada2b**	transcriptional adaptor 2B (Tada2b), m	289717	14	79085067	79096335	11269	1633,51	0,13	8,30E-01
ENSRNOG00000042536		phosphodiesterase 4D, cAMP-specific	24627	2	59859784	60521357	661574	693,79	-0,13	8,30E-01
ENSRNOG00000045752	**Rrm1**	ribonucleotide reductase M1 (Rrm1), r	685579	1	173726994	173751294	24301	1576,34	0,18	8,30E-01
ENSRNOG00000046391		Protein Fam177a1 [Source:UniProtKE	1E+08	6	85578014	85590447	12434	273,22	-0,13	8,30E-01
ENSRNOG00000046640		Uncharacterized protein [Source:UniProtKB/TrE		4	241402620	241402850	231	32,09	0,20	8,30E-01
ENSRNOG00000001158	**Abcg1**	ATP-binding cassette, subfamily G (W	85264	20	11971328	12020646	49319	733,79	0,22	8,30E-01
ENSRNOG00000046845	**LOC67956**	Protein LOC100910957 [Source:UniP	679565	17	91544959	91589401	44443	49,25	0,18	8,30E-01
ENSRNOG00000006554	**Fam149b1**	family with sequence similarity 149, m	289900	15	8463360	8500617	37258	864,68	0,13	8,30E-01
ENSRNOG00000005195	**Cst3**	cystatin C (Cst3), mRNA [Source:RefS	25307	3	149628698	149632565	3868	41613,77	0,22	8,30E-01
ENSRNOG00000013981		tyrosine-protein phosphatase non-rece	29644	1	104263143	104320930	57788	2054,65	0,13	8,30E-01
ENSRNOG00000030416	**Zfp870**	zinc finger protein 870 (Zfp870), mRN	362845	7	15317710	15322754	5045	148,03	-0,14	8,30E-01

ENSRNOG00000032042	**RGD15601**	similar to RIKEN cDNA 2700081O15 (	499309	1	229659747	229664618	4872	5835,75	0,14	8,30E-01
ENSRNOG00000048651	**Nrtn**	neurturin (Nrtn), mRNA [Source:RefSe	84423	9	9302550	9303677	1128	21,28	-0,24	8,30E-01
ENSRNOG00000011341	**Rictor**	Protein Rictor [Source:UniProtKB/TrE	310131	2	75754852	75842492	87641	321,14	0,24	8,30E-01
ENSRNOG00000016013	**Gprc5b**	G protein-coupled receptor, family C, g	293546	1	195631489	195656026	24538	2183,19	-0,22	8,31E-01
ENSRNOG00000028996	**Krt1**	keratin 1 (Krt1), mRNA [Source:RefSe	300250	7	141245848	141251072	5225	38,89	0,22	8,31E-01
ENSRNOG00000043295	**RGD15650**	uncharacterized protein LOC498014 [	498014	10	94983256	94985409	2154	186,94	-0,17	8,31E-01
ENSRNOG00000033100				3	150210241	150210980	740	67,00	0,22	8,31E-01
ENSRNOG00000018097	**Emc1**	ER membrane protein complex subun	362643	5	161560620	161585938	25319	2067,30	-0,10	8,31E-01
ENSRNOG00000006816		ubiquitin protein ligase E3 component n-recogni		7	76906409	76975003	68595	5323,10	-0,13	8,31E-01
ENSRNOG00000010392		neuregulin 1 (Nrg1), transcript variant	112400	16	63540981	63718742	177762	144,92	-0,13	8,31E-01
ENSRNOG00000007665	**Cse1l**	CSE1 chromosome segregation 1-like	362273	3	169825002	169862567	37566	1684,73	-0,17	8,31E-01
ENSRNOG00000009338	**Kras**	Kirsten rat sarcoma viral oncogene (K	24525	4	243667401	243693788	26388	838,56	-0,14	8,31E-01
ENSRNOG00000010473		cullin-associated and neddylation-diss	192226	4	210970266	210998144	27879	618,02	-0,15	8,31E-01
ENSRNOG00000013589		chemokine (C-X-C motif) ligand 12 (C	24772	4	215195689	215203554	7866	593,94	-0,13	8,31E-01
ENSRNOG00000028451	**Cox7b**	cytochrome c oxidase subunit VIIb (Co	303393	X	56188258	56194536	6279	1791,11	-0,18	8,31E-01
ENSRNOG00000046090		Uncharacterized protein [Source:UniProtKB/TrE		6	124017212	124049097	31886	67,79	-0,22	8,32E-01
ENSRNOG00000010284	**St3gal5**	ST3 beta-galactoside alpha-2,3-sialylt	83505	4	164713253	164769522	56270	2176,19	-0,12	8,32E-01
ENSRNOG00000017592		Uncharacterized protein [Source:UniProtKB/TrE		8	71719378	71720238	861	208,88	0,19	8,32E-01
ENSRNOG00000018425	**Dym**	dymeclin (Dym), mRNA [Source:RefSe	291433	18	70132075	70449686	317612	1608,35	0,10	8,32E-01
ENSRNOG00000050343		junction-mediating and regulatory protein [Sourc		2	42238034	42292484	54451	519,96	-0,11	8,32E-01
ENSRNOG00000014862	**Gabrg3**	gamma-aminobutyric acid (GABA) A re	79211	1	113167703	113438056	270354	76,94	0,17	8,32E-01
ENSRNOG00000015491		RNA terminal phosphate cyclase-like 1	309301	1	254471098	254544969	73872	470,00	0,10	8,32E-01
ENSRNOG00000001858	**Ypel1**	Protein yippee-like [Source:UniProtKE	1E+08	11	91226555	91233207	6653	287,53	0,19	8,32E-01
ENSRNOG00000005933	**Yap1**	yes-associated protein 1 (Yap1), mRN	363014	8	6138909	6202218	63310	344,29	-0,17	8,32E-01
ENSRNOG00000008907	**Fggy**	FGGY carbohydrate kinase domain co	298250	5	118461635	118848789	387155	100,71	0,18	8,32E-01
ENSRNOG00000012568	**Madd**	MAP-kinase activating death domain (	94193	3	86669054	86711776	42723	2889,25	-0,12	8,33E-01
ENSRNOG00000001597	**Atf2**	activating transcription factor 2 (Atf2),	81647	3	67199878	67274694	74817	1250,89	0,13	8,33E-01
ENSRNOG00000003117	**Psmd12**	proteasome (prosome, macropain) 26	287772	10	95441589	95460233	18645	2101,82	0,18	8,33E-01
ENSRNOG00000003241	**Gabrg2**	gamma-aminobutyric acid (GABA) A re	29709	10	26937103	27023929	86827	805,48	0,13	8,33E-01
ENSRNOG00000006996	**Mms22l**	MMS22-like, DNA repair protein (Mms	313108	5	43473598	43590298	116701	253,39	0,17	8,33E-01
ENSRNOG00000010186	**Cdv3**	carnitine deficiency-associated gene e	315970	8	111231870	111242236	10367	609,92	-0,15	8,33E-01
ENSRNOG00000010301	**Eif4e3**	eukaryotic translation initiation factor 4	297481	4	196560131	196602330	42200	226,95	0,18	8,33E-01
ENSRNOG00000018009	**Rab8b**	RAB8B, member RAS oncogene famil	266688	8	76975470	77047522	72053	434,31	-0,12	8,33E-01
ENSRNOG00000022066	**Fam117b**	family with sequence similarity 117, m	363236	9	66520071	66596356	76286	3999,37	0,15	8,33E-01
ENSRNOG00000025523	**RGD15648**	Protein RGD1564887 [Source:UniPro	499363	1	273006527	273170809	164283	709,55	0,14	8,33E-01
ENSRNOG00000031197	**F8**	coagulation factor VIII, procoagulant component		18	413495	444411	30917	441,19	0,21	8,33E-01
ENSRNOG00000039551	**Rdh14**	retinol dehydrogenase 14 (all-trans/9-c	500629	6	45868730	45873873	5144	444,36	-0,16	8,33E-01
ENSRNOG00000001804		inositol 1,4,5-trisphosphate receptor ty	81678	4	244585712	244960183	374472	969,30	-0,19	8,33E-01

ENSRNOG00000013135	**Ptpn12**	protein tyrosine phosphatase, non-rec	117255	4	10623859	10695124	71266	1138,56	-0,12	8,33E-01
ENSRNOG00000014328	**Frs3**	fibroblast growth factor receptor subst	316213	9	14222004	14228827	6824	357,87	0,11	8,33E-01
ENSRNOG00000020285	**Gprin2**	Protein Gprin2 [Source:UniProtKB/TrE	306284	16	8846316	8847683	1368	227,86	0,16	8,33E-01
ENSRNOG00000030568		regulator of G-protein signaling 12 (Rg	54292	14	81671045	81742380	71336	2061,95	-0,13	8,33E-01
ENSRNOG00000050108		Protein LOC100911319 [Source:UniP	1E+08	7	2329849	2331791	1943	18,78	-0,23	8,33E-01
ENSRNOG00000007455	**Tmem132e**	transmembrane protein 132E (Tmem1	287564	10	69370778	69428365	57588	649,17	-0,18	8,33E-01
ENSRNOG00000009790	**Kcnk3**	potassium channel, subfamily K, mem	29553	6	36969854	37005778	35925	206,13	0,16	8,33E-01
ENSRNOG00000012738	**Eif3m**	eukaryotic translation initiation factor 3	295975	3	101614557	101632161	17605	1588,07	-0,19	8,33E-01
ENSRNOG00000015217	**Ltv1**	LTV1 homolog (S. cerevisiae) (Ltv1), r	361452	1	8958295	8971014	12720	478,29	-0,18	8,33E-01
ENSRNOG00000016475	**Nt5c3b**	5'-nucleotidase, cytosolic IIIB (Nt5c3b)	360629	10	88135607	88149785	14179	1108,62	0,10	8,33E-01
ENSRNOG00000018778	**Cadm1**	cell adhesion molecule 1 (Cadm1), mF	363058	8	50460915	50788814	327900	2859,00	-0,14	8,33E-01
ENSRNOG00000019369	**Slx1b**	SLX1 structure-specific endonuclease	293489	1	205093520	205099060	5541	600,90	0,12	8,33E-01
ENSRNOG00000048111		Uncharacterized protein [Source:UniProtKB/TrE		2	9126057	9182713	56657	149,03	-0,17	8,33E-01
ENSRNOG00000003385		Glutaredoxin-2, mitochondrial [Source	114022	13	65518240	65528569	10330	1269,80	-0,11	8,33E-01
ENSRNOG00000006485	**Topors**	topoisomerase I binding, arginine/serir	362501	5	61098862	61110393	11532	1043,25	0,11	8,33E-01
ENSRNOG00000010600	**RGD15608**	similar to RIKEN cDNA 2310002J15 (F	499747	3	2439578	2440668	1091	30,35	0,22	8,33E-01
ENSRNOG00000013271	**Ireb2**	iron responsive element binding protei	64831	8	58039175	58083277	44103	1662,92	-0,16	8,33E-01
ENSRNOG00000004312	**Tmbim4**	transmembrane BAX inhibitor motif co	362884	7	65206720	65221307	14588	1191,00	0,15	8,34E-01
ENSRNOG00000004591	**Alg12**	asparagine-linked glycosylation 12, alp	315212	7	129487375	129501100	13726	487,64	0,15	8,34E-01
ENSRNOG00000010512	**Yipf1**	Yip1 domain family, member 1 (Yipf1),	298312	5	130781795	130817034	35240	1440,45	0,14	8,34E-01
ENSRNOG00000012762	**Zfp64**	zinc finger protein 64 (Zfp64), mRNA [	311661	3	171846883	171875427	28545	614,67	0,10	8,34E-01
ENSRNOG00000009820	**Bnip3l**	BCL2/adenovirus E1B interacting prot	140923	15	47927967	47951099	23133	3480,00	-0,12	8,34E-01
ENSRNOG00000014171	**Tnfsf13**	tumor necrosis factor (ligand) superfar	287437	10	56032611	56035590	2980	43,76	-0,24	8,34E-01
ENSRNOG00000014813	**Prkag1**	protein kinase, AMP-activated, gamma	25520	X	114999624	115017043	17420	1839,84	0,17	8,34E-01
ENSRNOG00000028311	**Cnpy1**	Protein LOC685001 [Source:UniProtK	685001	4	470884	477469	6586	38,68	-0,20	8,34E-01
ENSRNOG00000013898	**Cdca4**	cell division cycle associated 4 (Cdca4	500727	6	146400543	146401256	714	197,51	0,11	8,34E-01
ENSRNOG00000001331	**Rnf34**	ring finger protein 34, E3 ubiquitin prot	282845	12	41057289	41078167	20879	1777,76	-0,13	8,34E-01
ENSRNOG00000005064	**Ube2b**	ubiquitin-conjugating enzyme E2B (Ub	81816	10	37213059	37227635	14577	3633,51	0,19	8,34E-01
ENSRNOG00000009332	**RGD13118**	uncharacterized protein LOC300751 [	300751	8	63215248	63301532	86285	48,86	-0,21	8,34E-01
ENSRNOG00000010839	**RGD15603**	Protein RGD1560383; RCG34143 [Sc	497994	10	86940348	86949914	9567	22,16	-0,21	8,34E-01
ENSRNOG00000015488	**Tead1**	TEA domain family member 1 (Tead1)	361630	1	184473372	184683039	209668	413,91	-0,16	8,34E-01
ENSRNOG00000023605	**Ncbp1**	nuclear cap binding protein subunit 1 (	298075	5	66250579	66282943	32365	1951,71	0,16	8,34E-01
ENSRNOG00000042376				18	30530558	30532961	2404	288,04	-0,14	8,34E-01
ENSRNOG00000042467	**Ttc4**	tetratricopeptide repeat domain 4 (Ttc4	362556	5	130118034	130127367	9334	366,61	0,15	8,34E-01
ENSRNOG00000047329	**Mtif3**	mitochondrial translational initiation fac	684274	12	11958153	11969303	11151	364,59	0,13	8,34E-01
ENSRNOG00000015382	**Arid5a**	AT rich interactive domain 5A (Mrf1 lik	316327	9	42534321	42538959	4639	296,95	-0,17	8,34E-01
ENSRNOG00000013254	**Dctn6**	dynactin 6 (Dctn6), mRNA [Source:Re	290798	16	61461230	61480355	19126	1631,41	0,11	8,34E-01
ENSRNOG00000015558	**Zfp512b**	zinc finger protein 512B (Zfp512b), mF	311721	3	180788171	180798662	10492	3139,18	-0,10	8,34E-01

ENSRNOG00000017956	**LOC10036**	golgin A7 (Golga7), mRNA [Source:Re	361171	16	73198501	73213640	15140	5073,54	0,14	8,34E-01
ENSRNOG00000028517		RAD9, HUS1, RAD1-interacting nuclear orphan		4	226885736	226891055	5320	218,08	0,12	8,34E-01
ENSRNOG00000045974		TAF4A RNA polymerase II, TATA box binding pro		3	181753108	181816579	63472	719,18	-0,11	8,34E-01
ENSRNOG00000006391	**Smarcad1**	SWI/SNF-related, matrix-associated a	312398	4	160672979	160733484	60506	993,86	-0,20	8,35E-01
ENSRNOG00000011720	**L3mbtl3**	Protein L3mbtl3 [Source:UniProtKB/T	309550	1	21510156	21584250	74095	816,67	-0,13	8,35E-01
ENSRNOG00000010196	**Glb1**	galactosidase, beta 1 (Glb1), mRNA [S	316033	8	121752182	121824793	72612	650,03	0,13	8,35E-01
ENSRNOG00000012639	**Rsrc1**	arginine/serine-rich coiled-coil 1 (Rsrc	361956	2	183491965	183881122	389158	195,25	-0,14	8,35E-01
ENSRNOG00000009902	**Lrrc46**	leucine rich repeat containing 46 (Lrrc	287653	10	84762161	84766655	4495	177,10	0,16	8,35E-01
ENSRNOG00000016462		Protein Zfp87; RCG42042, isoform CRA_c [Sou		1	39309508	39319406	9899	106,46	-0,22	8,35E-01
ENSRNOG00000038012	**Commd6**	COMM domain containing 6 (Commd6	498559	15	89902294	89908870	6577	621,31	0,12	8,35E-01
ENSRNOG00000031938	**LOC31414**	ribose-phosphate pyrophosphokinase	314140	6	87689010	87697170	8161	254,46	0,16	8,35E-01
ENSRNOG00000022164	**Zscan2**	Protein LOC683603; RCG24561 [Sou	683603	1	143633720	143646167	12448	255,04	0,18	8,35E-01
ENSRNOG00000010087	**Zfp143**	zinc finger protein 143 (Zfp143), mRN	361627	1	181702291	181738044	35754	213,55	-0,17	8,35E-01
ENSRNOG00000002710	**Cnst**	Protein Cnst [Source:UniProtKB/TrEM	498297	13	102879477	102922898	43422	282,03	0,18	8,36E-01
ENSRNOG00000004052	**Ppp1r9b**	protein phosphatase 1, regulatory sub	84686	10	82616848	82632352	15505	8819,04	0,12	8,36E-01
ENSRNOG00000019791	**Sipa1l2**	signal-induced proliferation-associatec	361442	19	68936975	69098880	161906	1977,21	0,09	8,36E-01
ENSRNOG00000020118	**Nme5**	Protein LOC688903 [Source:UniProtK	688903	18	27071255	27087561	16307	59,60	-0,18	8,36E-01
ENSRNOG00000036699	**RGD13069**	Protein RGD1306926 [Source:UniPro	303742	10	109136955	109145928	8974	1144,96	0,14	8,36E-01
ENSRNOG00000000638	**Zfp365**	zinc finger protein 365 (Zfp365), mRN	499425	20	24159323	24183057	23735	574,54	-0,14	8,36E-01
ENSRNOG00000024077	**Fbxo5**	F-box protein 5 (Fbxo5), mRNA [Sourc	292263	1	43791187	43797556	6370	158,55	-0,13	8,36E-01
ENSRNOG00000025940	**Mettl9**	methyltransferase like 9 (Mettl9), mRN	1E+08	1	197891178	197936219	45042	2211,45	-0,12	8,36E-01
ENSRNOG00000008064	**Naga**	N-acetyl galactosaminidase, alpha (Na	315165	7	123547945	123556139	8195	1208,77	0,13	8,36E-01
ENSRNOG00000008455		Protein Tmem87a [Source:UniProtKB	366170	3	118677063	118721810	44748	349,38	0,13	8,36E-01
ENSRNOG00000009373				8	33096207	33097816	1610	86,65	0,18	8,36E-01
ENSRNOG00000011245	**Arhgap18**	Rho GTPase activating protein 18 (Ar	293947	1	20730432	20879837	149406	99,18	0,23	8,36E-01
ENSRNOG00000013728	**Polg2**	polymerase (DNA directed), gamma 2	303612	10	94714852	94724570	9719	201,27	0,14	8,36E-01
ENSRNOG00000016419	**Pdlim5**	PDZ and LIM domain 5 (Pdlim5), mRN	64353	2	266348751	266518443	169693	1226,34	-0,16	8,36E-01
ENSRNOG00000021056	**Kcnj14**	potassium inwardly-rectifying channel,	276720	1	102884516	102888529	4014	243,26	0,19	8,36E-01
ENSRNOG00000037567		RIKEN cDNA B230118H07 gene [Source:MGI S		3	97747219	97833544	86326	146,04	-0,22	8,36E-01
ENSRNOG00000005576	**Rpia**	ribose 5-phosphate isomerase A (Rpia	362383	4	163347716	163373352	25637	414,43	0,16	8,37E-01
ENSRNOG00000009186	**Stmn4**	stathmin-like 4 (Stmn4), transcript vari	79423	15	48564801	48582956	18156	11330,86	0,17	8,37E-01
ENSRNOG00000010427	**Ipo7**	importin 7 (Ipo7), mRNA [Source:RefS	308939	1	181641921	181696091	54171	1990,18	-0,21	8,37E-01
ENSRNOG00000000168	**Gatm**	glycine amidinotransferase (L-arginine	81660	3	121249708	121266292	16585	5012,98	0,10	8,37E-01
ENSRNOG00000013907	**Sall1**	sal-like 1 (Drosophila) (Sall1), mRNA [	307740	19	34379544	34395131	15588	1894,67	0,15	8,37E-01
ENSRNOG00000027376	**Il17rc**	interleukin 17 receptor C (Il17rc), mRN	297520	4	208724712	208737142	12431	213,33	0,21	8,37E-01
ENSRNOG00000027857	**Pigw**	phosphatidylinositol glycan anchor bio	378774	10	72101788	72103296	1509	96,10	-0,15	8,37E-01
ENSRNOG00000043061	**Siah3**	siah E3 ubiquitin protein ligase family	692004	15	61215305	61278376	63072	103,85	-0,16	8,37E-01
ENSRNOG00000005129	**Dtx3**	deltex homolog 3 (Drosophila) (Dtx3),	500847	7	70654776	70659437	4662	3837,41	0,12	8,37E-01

ENSRNOG00000006600		unkempt homolog (Drosophila) [Sourc	360663	10	103673535	103702359	28825	623,84	-0,12	8,37E-01
ENSRNOG00000031328	**Zfp110**	zinc finger protein 110 (Zfp110), mRN/	308362	1	66553227	66567666	14440	361,07	-0,16	8,37E-01
ENSRNOG00000003479	**Rnf150**	ring finger protein 150 (Rnf150), mRN.	364983	19	35023410	35242919	219510	163,74	0,17	8,38E-01
ENSRNOG00000003837	**Mif4gd**	MIF4G domain containing (Mif4gd), m	360659	10	104117570	104122031	4462	235,11	-0,18	8,38E-01
ENSRNOG00000003858	**Atp6ap2**	ATPase, H+ transporting, lysosomal a	302526	X	11935610	11963424	27815	3584,95	0,12	8,38E-01
ENSRNOG00000007064	**Zfp746**	zinc finger protein 746 (Zfp746), mRN.	312303	4	142392264	142416151	23888	1155,54	0,11	8,38E-01
ENSRNOG00000012364	**Prickle2**	prickle homolog 2 (Drosophila) (Prickle	312563	4	188925956	189025114	99159	3326,91	-0,13	8,38E-01
ENSRNOG00000014837	**Emilin2**	Protein Emilin2 [Source:UniProtKB/Tr	316736	9	119048148	119106488	58341	21,76	-0,23	8,38E-01
ENSRNOG00000015986	**Rassf8**	Ras association (RalGDS/AF-6) doma	312846	4	244235300	244304646	69347	151,90	0,12	8,38E-01
ENSRNOG00000019352	**Emc6**	ER membrane protein complex subun	287477	10	59481630	59481962	333	1145,10	0,21	8,38E-01
ENSRNOG00000019819	**Dus2**	dihydrouridine synthase 2 (Dus2), mR	291978	19	48857360	48900338	42979	601,35	0,16	8,38E-01
ENSRNOG00000021685	**Cdk5r1**	cyclin-dependent kinase 5, regulatory	116671	10	67515764	67516965	1202	2739,47	0,14	8,38E-01
ENSRNOG00000027533	**Ns5atp4**	NS5A (hepatitis C virus) transactivatec	311934	3	40659571	40660326	756	59,00	0,19	8,38E-01
ENSRNOG00000037783	**Fam155a**	Protein Fam155a [Source:UniProtKB/	498667	16	84967014	84967958	945	328,41	-0,16	8,38E-01
ENSRNOG00000020506	**Khnyn**	Protein Khnyn-ps1 [Source:UniProtKE	498517	15	38440708	38450025	9318	161,74	-0,17	8,38E-01
ENSRNOG00000006795	**Apmap**	adipocyte plasma membrane associat	366227	3	152738538	152758056	19519	1238,27	0,17	8,38E-01
ENSRNOG00000011148		signal sequence receptor, gamma [Source:MGI :		2	175399773	175410993	11221	5013,83	-0,17	8,38E-01
ENSRNOG00000014354	**Cep95**	centrosomal protein 95kDa (Cep95), n	287766	10	94734062	94762044	27983	499,36	-0,16	8,38E-01
ENSRNOG00000017090	**RGD13087**	similar to RIKEN cDNA 4921524J17 (F	291925	19	33717642	33744546	26905	684,45	0,15	8,38E-01
ENSRNOG00000043144		6.8 kDa mitochondrial proteolipid [Sou	1E+08	6	145534566	145536613	2048	127,64	0,12	8,38E-01
ENSRNOG00000016957	**Igfbp2**	insulin-like growth factor binding prote	25662	9	79888614	79915188	26575	10667,68	0,21	8,38E-01
ENSRNOG00000048812	**Gpx1**	glutathione peroxidase 1 (Gpx1), mRN	24404	8	116466150	116467210	1061	2724,25	0,21	8,38E-01
ENSRNOG00000002875	**Klhl20**	kelch-like family member 20 (Klhl20), i	304920	13	83806860	83851817	44958	646,13	-0,17	8,38E-01
ENSRNOG00000004360	**Gemin2**	gem (nuclear organelle) associated pr	84404	6	89634623	89648253	13631	252,31	-0,14	8,38E-01
ENSRNOG00000006335	**Klhl9**	kelch-like family member 9 (Klhl9), mF	313348	5	111299137	111303302	4166	2471,43	-0,14	8,38E-01
ENSRNOG00000006751	**Psmd6**	proteasome (prosome, macropain) 26	289924	15	16438805	16447424	8620	1678,10	0,16	8,38E-01
ENSRNOG00000009878		Uncharacterized protein [Source:UniProtKB/TrE		8	121695471	121703895	8425	129,93	-0,18	8,38E-01
ENSRNOG00000010461	**Gpx8**	glutathione peroxidase 8 (Gpx8), mRN	294744	2	63940890	63944583	3694	570,65	0,15	8,38E-01
ENSRNOG00000019232	**RGD13113**	similar to CG9752-PA (RGD1311345),	361201	17	8887525	8895096	7572	623,83	0,17	8,38E-01
ENSRNOG00000027282	**Cep68**	Protein Cep68 [Source:UniProtKB/TrE	289822	14	104240524	104251658	11135	1067,01	0,09	8,38E-01
ENSRNOG00000031333				MT	5185	5252	68	1859,08	-0,21	8,38E-01
ENSRNOG00000032055	**Spats2**	spermatogenesis associated, serine-ri	300221	X	115333557	115384244	50688	1608,16	0,11	8,38E-01
ENSRNOG00000032307	**Dsel**	Protein Dsel; RCG24512 [Source:Uni	297865	13	1936646	1940272	3627	275,36	-0,15	8,38E-01
ENSRNOG00000013498	**Pex14**	peroxisomal biogenesis factor 14 (Pex	64460	5	169433225	169568626	135402	1131,70	0,10	8,38E-01
ENSRNOG00000016913	**Stk36**	serine/threonine kinase 36 (Stk36), ml	301516	9	81642557	81670395	27839	408,88	0,12	8,38E-01
ENSRNOG00000017030		AT rich interactive domain 1B (SWI-like) [Source		1	47239545	47548532	308988	2892,76	0,11	8,38E-01
ENSRNOG00000015133		lysine (K)-specific methyltransferase 2	315606	8	47733498	47777433	43936	3489,91	0,12	8,38E-01
ENSRNOG00000016467	**Kctd1**	potassium channel tetramerization doi	291772	18	6337410	6378501	41092	340,31	0,13	8,39E-01

ENSRNOG00000002142	**Cds1**	CDP-diacylglycerol synthase 1 (Cds1)	81925	14	9356230	9418464	62235	1250,86	-0,12	8,39E-01
ENSRNOG00000002537	**Wnk3**	WNK lysine deficient protein kinase 3	317420	X	21096338	21198949	102612	302,04	0,22	8,39E-01
ENSRNOG00000003882		centrosomal protein 350 [Source:MGI	246304	13	78427849	78547242	119394	830,05	0,20	8,39E-01
ENSRNOG00000013876	**Mipep**	mitochondrial intermediate peptidase (	81684	15	44896328	45002041	105714	731,55	0,13	8,39E-01
ENSRNOG00000026280	**Mettl4**	methyltransferase like 4 (Mettl4), mRN	316731	9	119326955	119350223	23269	36,72	-0,19	8,39E-01
ENSRNOG00000003936	**Pwwp2a**	PWWP domain containing 2A (Pwwp2	303060	10	29129861	29174706	44846	685,73	0,15	8,39E-01
ENSRNOG00000006444	**Fkbp4**	FK506 binding protein 4 (Fkbp4), mRN	260321	4	226954534	226962988	8455	5528,00	0,13	8,39E-01
ENSRNOG00000008929	**Aebp2**	AE binding protein 2 (Aebp2), mRNA [	297705	4	239043565	239094663	51099	334,81	-0,11	8,39E-01
ENSRNOG00000011334	**Tmem63c**	transmembrane protein 63c (Tmem63	314332	6	120339455	120404855	65401	327,04	-0,15	8,39E-01
ENSRNOG00000014097	**Psmd7**	proteasome (prosome, macropain) 26	307821	19	50501208	50508494	7287	3148,29	0,10	8,39E-01
ENSRNOG00000017889	**Serac1**	Protein Serac1; Similar to serine activ	499015	1	48241866	48275927	34062	263,08	-0,11	8,39E-01
ENSRNOG00000031993	**Prim1**	primase, DNA, polypeptide 1 (Prim1),	246327	7	2416149	2430936	14788	816,93	-0,11	8,39E-01
ENSRNOG00000008297	**Oser1**	oxidative stress responsive serine-rich	296346	3	165976713	165995482	18770	596,03	0,12	8,39E-01
ENSRNOG00000005344	**Nol10**	nucleolar protein 10 (Nol10), mRNA [S	313981	6	52376044	52460900	84857	412,34	-0,17	8,39E-01
ENSRNOG00000043033		lactation elevated 1 [Source:MGI Symbol;Acc:M		20	48714680	48828329	113650	113,53	0,18	8,39E-01
ENSRNOG00000047374	**Gnas**	GNAS complex locus (Gnas), transcript variant 3		3	178489204	178490283	1080	8068,02	-0,12	8,39E-01
ENSRNOG00000004403	**Slc25a32**	solute carrier family 25 (mitochondrial	315023	7	78244520	78262013	17494	209,53	0,16	8,40E-01
ENSRNOG00000019039	**Inpp5e**	inositol polyphosphate-5-phosphatase E (Inpp5e		3	9204538	9216619	12082	468,53	0,12	8,40E-01
ENSRNOG00000021249	**Ap5s1**	adaptor-related protein complex 5, sig	499893	3	130251743	130255035	3293	184,43	0,15	8,40E-01
ENSRNOG00000012085	**Isca2-ps1**	iron-sulfur cluster assembly 2 (Isca2),	364178	6	117058978	117060567	1590	319,79	0,15	8,40E-01
ENSRNOG00000048795		topoisomerase (DNA) II beta [Source:MGI Symb		15	14334848	14350228	15381	1578,01	-0,18	8,40E-01
ENSRNOG00000026880	**Usp38**	Ubiquitin carboxyl-terminal hydrolase	307764	19	41576637	41606593	29957	805,30	0,16	8,40E-01
ENSRNOG00000000565	**Sgpl1**	sphingosine-1-phosphate lyase 1 (Sgp	286896	20	32497280	32543194	45915	1426,33	0,15	8,40E-01
ENSRNOG00000000836	**Ltb**	lymphotoxin beta (TNF superfamily, m	361795	20	6941754	6943598	1845	24,10	-0,22	8,40E-01
ENSRNOG00000010958	**Prdx3**	peroxiredoxin 3 (Prdx3), mRNA [Sourc	64371	1	289577331	289589744	12414	1702,37	0,12	8,40E-01
ENSRNOG00000017469	**Anxa1**	annexin A1 (Anxa1), mRNA [Source:R	25380	1	245192877	245208910	16034	257,12	-0,23	8,40E-01
ENSRNOG00000020147		Longevity assurance-like protein 1; Pr	290658	16	20700117	20712536	12420	1276,77	0,13	8,40E-01
ENSRNOG00000025643		coiled-coil domain containing 13 [Sour	686091	8	129616116	129648663	32548	70,55	0,18	8,40E-01
ENSRNOG00000031100	**Klhl1**	kelch-like family member 1 (Klhl1), mR	290426	15	83811191	84250457	439267	132,79	0,24	8,40E-01
ENSRNOG00000042512				3	52290069	52290929	861	211,88	-0,14	8,40E-01
ENSRNOG00000050204	**Naif1**	Protein LOC687739; RCG45484 [Sou	687739	3	16826054	16828861	2808	59,53	0,15	8,40E-01
ENSRNOG00000003650	**Nt5c**	5', 3'-nucleotidase, cytosolic (Nt5c), m	1E+08	10	104226190	104227156	967	51,68	0,23	8,41E-01
ENSRNOG00000042070	**Ticam2**	toll-like receptor adaptor molecule 2 (T	364867	18	40209745	40226880	17136	46,14	-0,17	8,41E-01
ENSRNOG00000047174		Protocadherin alpha-4 [Source:UniPro	116741	18	29670058	29672442	2385	341,47	-0,14	8,41E-01
ENSRNOG00000014744		Protein Pacs2 [Source:UniProtKB/TrE	691631	6	146820376	146881865	61490	3624,49	-0,11	8,41E-01
ENSRNOG00000036678		alveolar soft part sarcoma chromosom	691026	10	109444698	109480501	35804	1134,74	0,14	8,41E-01
ENSRNOG00000009702	**Lmo7**	LIM domain 7 (Lmo7), mRNA [Source:	361084	15	87780478	87996342	215865	472,85	-0,16	8,41E-01
ENSRNOG00000001708	**Dvl3**	dishevelled segment polarity protein 3	303811	11	87125789	87139453	13665	2719,31	0,11	8,41E-01

ENSRNOG00000004602	**Rnf145**	ring finger protein 145 (Rnf145), mRN.	287212	10	30002561	30040956	38396	3022,06	-0,18	8,41E-01
ENSRNOG00000007294	**Sntg1**	syntrophin, gamma 1 (Sntg1), mRNA [	500394	5	14977623	15286329	308707	63,97	-0,20	8,41E-01
ENSRNOG00000007722	**Fam57a**	Protein Fam57a; RCG33700, isoform	1E+08	10	63647911	63653810	5900	184,44	0,19	8,41E-01
ENSRNOG00000007785	**Trmt5**	tRNA methyltransferase 5 homolog (S	362754	6	105596366	105601887	5522	253,68	0,17	8,41E-01
ENSRNOG00000007955	**Timp4**	tissue inhibitor of metalloproteinase 4	680130	4	210445694	210452213	6520	211,58	0,17	8,41E-01
ENSRNOG00000011146	**Gyg1**	glycogenin 1 (Gyg1), mRNA [Source:F	81675	2	124636898	124676137	39240	926,83	-0,13	8,41E-01
ENSRNOG00000015810	**Trip13**	thyroid hormone receptor interactor 13	292206	1	33392427	33437364	44938	231,96	0,14	8,41E-01
ENSRNOG00000018229	**Slc45a1**	solute carrier family 45, member 1 (Slc	246258	5	171300141	171322391	22251	1133,25	0,11	8,41E-01
ENSRNOG00000018830	**Aff3**	AF4/FMR2 family, member 3 (Aff3), m	363220	9	44535656	44901101	365446	1260,04	-0,13	8,41E-01
ENSRNOG00000021241	**Gfra4**	GDNF family receptor alpha 4 (Gfra4),	66023	3	130078342	130080863	2522	59,54	0,20	8,41E-01
ENSRNOG00000002469	**Trim7**	Protein Trim7 [Source:UniProtKB/TrE	303089	10	33969595	33982976	13382	69,65	-0,14	8,41E-01
ENSRNOG00000003682	**Hook2**	hook homolog 2 (Drosophila) (Hook2),	304669	19	37082461	37094975	12515	551,15	0,14	8,41E-01
ENSRNOG00000018647	**Mrpl20**	mitochondrial ribosomal protein L20 (M	680747	5	176723859	176728389	4531	2418,77	-0,20	8,41E-01
ENSRNOG00000046345	**Rpn1**	ribophorin I (Rpn1), mRNA [Source:Re	25596	4	185243186	185264600	21415	13324,15	0,14	8,41E-01
ENSRNOG00000046398		proteasome (prosome, macropain) 26S subunit,		3	52274008	52279029	5022	204,63	0,15	8,41E-01
ENSRNOG00000018532	**Pcgf5**	polycomb group ring finger 5 (Pcgf5),	681178	1	262240468	262351833	111366	107,13	0,18	8,42E-01
ENSRNOG00000001259		cut-like homeobox 2 [Source:MGI Syn	288665	12	42062599	42089880	27282	1064,83	-0,16	8,42E-01
ENSRNOG00000002644	**Utp18**	UTP18, small subunit (SSU) processo	303456	10	81390897	81417858	26962	946,05	0,17	8,42E-01
ENSRNOG00000033076	**Thnsl1**	threonine synthase-like 1 (S. cerevisia	498805	17	89783177	89789609	6433	300,60	0,17	8,42E-01
ENSRNOG00000034157	**Cyp4f6**	cytochrome P450, family 4, subfamily	266689	7	15357407	15384091	26685	175,70	-0,20	8,42E-01
ENSRNOG00000038183		predicted gene, 17673 [Source:MGI S	500692	6	118090629	118120708	30080	51,45	-0,16	8,42E-01
ENSRNOG00000042333	**Dnal1**	dynein, axonemal, light chain 1 (Dnal1	685664	6	117911562	117933171	21610	1669,29	0,11	8,42E-01
ENSRNOG00000001050	**Eif2ak1**	eukaryotic translation initiation factor 2	27137	12	14792378	14825317	32940	1867,95	-0,12	8,42E-01
ENSRNOG00000030667		protein phosphatase, Mg2+/Mn2+ dep	24667	6	8152847	8212461	59615	1339,89	-0,11	8,42E-01
ENSRNOG00000017746	**Pard6a**	par-6 (partitioning defective 6,) homolo	307799	19	48535376	48537153	1778	379,82	0,12	8,42E-01
ENSRNOG00000018689	**RGD13054**	similar to human chromosome 15 ope	315702	8	60482444	60488730	6287	300,23	-0,19	8,42E-01
ENSRNOG00000021954	**Spg11**	spastic paraplegia 11 (autosomal rece	311372	3	120539692	120604601	64910	1128,11	-0,11	8,42E-01
ENSRNOG00000024779	**Polr2b**	polymerase (RNA) II (DNA directed) p	289561	14	32863627	32901277	37651	1698,24	0,18	8,42E-01
ENSRNOG00000031459		zinc finger protein 128 [Source:MGI Symbol;Acc		1	66504503	66516392	11890	374,73	-0,15	8,42E-01
ENSRNOG00000037162	**Slbp**	stem-loop binding protein (Slbp), mRN	681062	14	83046301	83056019	9719	1006,38	0,14	8,42E-01
ENSRNOG00000042322	**Pcdhga4**	Protein Pcdhga4 [Source:UniProtKB/T	252894	18	30525592	30528009	2418	283,15	0,13	8,42E-01
ENSRNOG00000019295	**Rab12**	RAB12, member RAS oncogene famil	25530	9	114187499	114211152	23654	916,59	0,14	8,43E-01
ENSRNOG00000030031		Uncharacterized protein [Source:UniProtKB/TrE		12	29411909	29616480	204572	47,10	-0,16	8,43E-01
ENSRNOG00000011625	**Tnks**	tankyrase, TRF1-interacting ankyrin-re	290794	16	60596310	60737794	141485	3743,97	0,12	8,43E-01
ENSRNOG00000019533	**Klhl3**	Kelch-like protein 3 [Source:UniProtK	498697	17	9127611	9226414	98804	157,32	0,14	8,43E-01
ENSRNOG00000008030	**Shc2**	SHC (Src homology 2 domain containi	314612	7	13117670	13139889	22220	1199,60	-0,12	8,43E-01
ENSRNOG00000048897	**Vmac**	vimentin-type intermediate filament as	363327	9	9336155	9337409	1255	149,45	0,16	8,43E-01
ENSRNOG00000000433	**Prrt1**	proline-rich transmembrane protein 1 (	406167	20	6471982	6475627	3646	1206,30	-0,13	8,43E-01

ENSRNOG00000002461	**Nid1**	Nidogen-1 [Source:UniProtKB/Swiss-	25494	17	92216094	92287843	71750	1170,88	-0,17	8,43E-01
ENSRNOG00000002572	**Cacybp**	calcyclin binding protein (Cacybp), mF	289144	13	82855951	82866235	10285	1869,16	-0,17	8,43E-01
ENSRNOG00000003326	**LOC10091**	RRN3 RNA polymerase I transcription	304714	10	2057048	2092163	35116	567,19	0,12	8,43E-01
ENSRNOG00000004773	**Yaf2**	YY1 associated factor 2 (Yaf2), mRNA	690262	7	134175947	134178051	2105	1785,08	0,09	8,43E-01
ENSRNOG00000005141	**Hus1**	HUS1 checkpoint homolog (S. pombe	498411	14	88991468	89006201	14734	472,67	-0,12	8,43E-01
ENSRNOG00000005713	**Ccdc82**	coiled-coil domain containing 82 (Ccd	300359	8	11846685	11885449	38765	119,54	-0,16	8,43E-01
ENSRNOG00000005807	**Ptpn7**	protein tyrosine phosphatase, non-rec	246781	13	57010236	57022149	11914	66,67	-0,20	8,43E-01
ENSRNOG00000006509	**Srgap3**	SLIT-ROBO Rho GTPase activating p	500287	4	207938831	208167482	228652	4465,42	-0,11	8,43E-01
ENSRNOG00000006705	**Mkks**	McKusick-Kaufman syndrome (Mkks),	311456	3	136347550	136365793	18244	667,30	-0,12	8,43E-01
ENSRNOG00000007253	**Cbll1**	Cbl proto-oncogene-like 1, E3 ubiquiti	314028	6	59443728	59457590	13863	1178,98	-0,14	8,43E-01
ENSRNOG00000008052	**Atp13a2**	ATPase type 13A2 (Atp13a2), mRNA [	362645	5	163224537	163243882	19346	5531,50	0,12	8,43E-01
ENSRNOG00000009341	**Hivep3**	human immunodeficiency virus type I	313557	5	142797806	142865048	67243	1299,32	-0,13	8,43E-01
ENSRNOG00000011202	**Chrna4**	cholinergic receptor, nicotinic, alpha 4	25590	3	180243038	180258028	14991	780,49	-0,12	8,43E-01
ENSRNOG00000011391	**Zfyve20**	zinc finger, FYVE domain containing 2	312562	4	189180786	189209501	28716	1643,09	0,13	8,43E-01
ENSRNOG00000011623	**Rab3c**	RAB3C, member RAS oncogene famil	171058	2	60628055	60843345	215291	282,88	-0,18	8,43E-01
ENSRNOG00000011989	**Vat1l**	Protein LOC361414 [Source:UniProtK	361414	19	57347972	57515078	167107	1540,52	0,15	8,43E-01
ENSRNOG00000012678		cyclin B2 [Source:MGI Symbol;Acc:MGI:88311]		8	76221576	76233721	12146	32,79	0,19	8,43E-01
ENSRNOG00000012858	**Odf3**	outer dense fiber of sperm tails 3 (Odf	365387	1	220522827	220526034	3208	27,14	0,19	8,43E-01
ENSRNOG00000013223		fumarylacetoacetase [Source:RefSeq	29383	1	147640320	147662820	22501	46,80	-0,22	8,43E-01
ENSRNOG00000013422	**Med23**	mediator complex subunit 23 (Med23)	309565	1	23019613	23067284	47672	1170,25	0,09	8,43E-01
ENSRNOG00000014503	**Celf4**	CUGBP, Elav-like family member 4 (C	307540	18	17451658	17470521	18864	2082,67	0,11	8,43E-01
ENSRNOG00000016434	**Prkd2**	protein kinase D2 (Prkd2), mRNA [Sou	292658	1	80015015	80043543	28529	195,29	0,11	8,43E-01
ENSRNOG00000016690	**Idi1**	isopentenyl-diphosphate delta isomera	89784	17	59783326	59789305	5980	654,01	-0,22	8,43E-01
ENSRNOG00000017309	**RGD13595**	similar to chromosome 1 open reading	362626	5	157029893	157033632	3740	3100,30	0,21	8,43E-01
ENSRNOG00000017900	**rnf141**	ring finger protein 141 (rnf141), mRNA	308900	1	182622487	182645144	22658	958,28	0,13	8,43E-01
ENSRNOG00000018531	**Mrps11**	mitochondrial ribosomal protein S11 (M	499185	1	141453419	141462799	9381	1012,88	0,11	8,43E-01
ENSRNOG00000019077	**Lipa**	lipase A, lysosomal acid, cholesterol e	25055	1	260038182	260071430	33249	882,71	0,16	8,43E-01
ENSRNOG00000020300		lymphocyte specific 1 [Source:RefSe	361680	1	222524162	222557294	33133	21,70	-0,23	8,43E-01
ENSRNOG00000020621		Protein Dcst1 [Source:UniProtKB/TrE	295246	2	208082807	208106097	23291	19,44	-0,21	8,43E-01
ENSRNOG00000023064	**Catsper2**	cation channel, sperm associated 2 (C	366174	3	119896998	119916540	19543	90,65	-0,14	8,43E-01
ENSRNOG00000023197	**Zfp608**	zinc finger protein 608 (Zfp608), mRN	307296	18	49033842	49138505	104664	1792,78	0,14	8,43E-01
ENSRNOG00000023910	**Man2b1**	mannosidase, alpha, class 2B, membe	361378	19	37172460	37191890	19431	946,65	-0,17	8,43E-01
ENSRNOG00000027028	**Lamtor4**	late endosomal/lysosomal adaptor, MA	360776	12	21630758	21634473	3716	2032,80	0,21	8,43E-01
ENSRNOG00000028357	**Lrrc14b**	leucine rich repeat containing 14B (Lrr	502225	1	32957232	32961194	3963	35,36	-0,19	8,43E-01
ENSRNOG00000029740	**Slc35g1**	solute carrier family 35, member G1 (S	294072	1	264556961	264563395	6435	18,63	0,23	8,43E-01
ENSRNOG00000032329	**Fam196b**	Protein Fam196b [Source:UniProtKB/	1E+08	10	19244625	19262447	17823	64,59	-0,17	8,43E-01
ENSRNOG00000033280	**Pam**	peptidylglycine alpha-amidating mono	25508	9	110584151	110737325	153175	6649,98	0,21	8,43E-01
ENSRNOG00000037207		ubiquitin protein ligase E3 component	499877	3	119343521	119453073	109553	1050,93	-0,12	8,43E-01

ENSRNOG00000038789		centromere protein L (Cenpl), transcrip	289150	13	83781165	83795518	14354	223,75	0,12	8,43E-01
ENSRNOG00000046001				10	104947556	104948325	770	477,48	-0,23	8,43E-01
ENSRNOG00000046194				9	92023361	92023437	77	48,06	0,19	8,43E-01
ENSRNOG00000046493	**Pnpo**	pyridoxamine 5'-phosphate oxidase (P	64533	10	84665836	84672099	6264	740,64	0,13	8,43E-01
ENSRNOG00000047003	**LOC10091**	Protein LOC100912852 [Source:UniP	1E+08	9	11366446	11368305	1860	71,57	-0,18	8,43E-01
ENSRNOG00000005387	**Rbm3**	RNA binding motif (RNP1, RRM) prote	114488	X	15882065	15885501	3437	6489,91	0,16	8,43E-01
ENSRNOG00000014137	**Fbln1**	fibulin 1 (Fbln1), mRNA [Source:RefSe	315191	7	125810696	125890371	79676	255,86	0,16	8,43E-01
ENSRNOG00000024142	**Arglu1**	arginine and glutamate rich 1 (Arglu1)	290912	16	86014418	86038236	23819	2438,03	-0,16	8,43E-01
ENSRNOG00000025504	**Ankrd46**	ankyrin repeat domain 46 (Ankrd46), r	299982	7	75427580	75436478	8899	5302,98	0,15	8,43E-01
ENSRNOG00000009729	**Ubp1**	upstream binding protein 1 (LBP-1a) (	301038	8	121510557	121553887	43331	1685,74	0,17	8,43E-01
ENSRNOG00000043394	**LOC10036**	Protein LOC100360426; RCG54790, i	680080	5	65164582	65167406	2825	1162,18	0,12	8,43E-01
ENSRNOG00000012844	**Tox4**	TOX high mobility group box family me	286990	15	32506570	32520158	13589	2765,60	0,13	8,43E-01
ENSRNOG00000002369	**Rgs8**	regulator of G-protein signaling 8 (Rgs	54297	13	76072717	76104924	32208	709,32	-0,14	8,44E-01
ENSRNOG00000003191	**Bhlhb9**	basic helix-loop-helix domain containir	317407	X	106727764	106729383	1620	747,48	-0,16	8,44E-01
ENSRNOG00000013387	**Tpcn2**	two pore segment channel 2 (Tpcn2),	309139	1	225286482	225316676	30195	105,25	0,13	8,44E-01
ENSRNOG00000011714	**Sat2**	spermidine/spermine N1-acetyltransfe	360547	10	55973620	55975433	1814	608,73	-0,18	8,44E-01
ENSRNOG00000007478	**Cry2**	cryptochrome 2 (photolyase-like) (Cry2	170917	3	88018205	88048159	29955	3307,52	0,11	8,44E-01
ENSRNOG00000007719	**Ccnc**	cyclin C (Ccnc), mRNA [Source:RefSe	114839	5	40521921	40537695	15775	693,34	-0,14	8,44E-01
ENSRNOG00000015809		peroxisomal trans-2-enoyl-CoA reduct	113956	9	79375180	79403569	28390	552,34	0,13	8,44E-01
ENSRNOG00000017567	**Ano8**	Anoctamin [Source:UniProtKB/TrEMB	306340	16	19747154	19755987	8834	1888,22	-0,09	8,44E-01
ENSRNOG00000020318	**Pnkp**	polynucleotide kinase 3'-phosphatase	308576	1	101918528	101923538	5011	478,41	0,15	8,44E-01
ENSRNOG00000023453		lipopolysaccharide-responsive and bei	361975	2	204990264	205322437	332174	429,14	0,12	8,44E-01
ENSRNOG00000025394	**Tanc1**	tetratricopeptide repeat, ankyrin repea	311055	3	50829732	51028013	198282	524,84	-0,16	8,44E-01
ENSRNOG00000043866				MT	1094	2664	1571	26905,95	-0,17	8,44E-01
ENSRNOG00000048427				16	18258854	18259933	1080	26,21	-0,18	8,44E-01
ENSRNOG00000048849	**Pdss1**	Protein LOC100364990; RCG40823, i	1E+08	17	91698846	91736232	37387	306,93	0,15	8,44E-01
ENSRNOG00000005801	**Stx17**	syntaxin 17 (Stx17), mRNA [Source:R	252853	5	68383379	68440085	56707	168,83	-0,15	8,44E-01
ENSRNOG00000020634	**Pih1d1**	PIH1 domain containing 1 (Pih1d1), m	292898	1	102217680	102221752	4073	388,05	0,10	8,45E-01
ENSRNOG00000009826	**Bche**	butyrylcholinesterase (Bche), mRNA [S	65036	2	190443044	190536106	93063	20,26	-0,22	8,45E-01
ENSRNOG00000019773	**Egln1**	Egl nine homolog 1 [Source:UniProtK	308913	19	68375019	68412725	37707	608,62	0,13	8,45E-01
ENSRNOG00000000320	**Timm10b**	translocase of inner mitochondrial membrane 10		20	49963289	49963955	667	17,70	0,21	8,45E-01
ENSRNOG00000000888	**Sbds**	Shwachman-Bodian-Diamond syndror	288615	12	31858315	31867472	9158	880,97	-0,14	8,45E-01
ENSRNOG00000000981	**Scarb1**	scavenger receptor class B, member 1	25073	12	38572168	38638463	66296	942,82	-0,16	8,45E-01
ENSRNOG00000001044	**Aimp2**	aminoacyl tRNA synthetase complex-i	288480	12	14782144	14791697	9554	960,34	0,13	8,45E-01
ENSRNOG00000001304	**Bcr**	Protein Bcr [Source:UniProtKB/TrEMB	309696	20	16604841	16664083	59243	1706,32	0,09	8,45E-01
ENSRNOG00000001573	**Usp25**	ubiquitin specific peptidase 25 (Usp25	304150	11	19095389	19215605	120217	492,51	-0,14	8,45E-01
ENSRNOG00000003536	**Rufy1**	RUN and FYVE domain containing 1 (	360521	10	35653747	35698367	44621	745,80	0,10	8,45E-01
ENSRNOG00000003773	**Hmox2**	heme oxygenase (decycling) 2 (Hmox	79239	10	9756667	9772409	15743	1146,84	0,13	8,45E-01

ENSRNOG00000004091	**Cwc25**	CWC25 spliceosome-associated prote	360613	10	85475897	85499557	23661	590,00	0,14	8,45E-01
ENSRNOG00000004152	**Lrp12**	low density lipoprotein receptor-relatec	314941	7	78885444	78956523	71080	1720,62	0,15	8,45E-01
ENSRNOG00000004189		helicase (DNA) B [Source:MGI Symbc	500837	7	65079036	65104144	25109	281,54	0,11	8,45E-01
ENSRNOG00000004549	**Dhx40**	DEAH (Asp-Glu-Ala-His) box polypept	287595	10	75981100	76019044	37945	669,72	-0,19	8,45E-01
ENSRNOG00000005174	**Tmem121**	Protein Tmem121 [Source:UniProtKB	691678	6	146991739	146992695	957	849,89	0,19	8,45E-01
ENSRNOG00000005292	**Trip11**	thyroid hormone receptor interactor 11	314393	6	134956635	135024177	67543	649,39	0,20	8,45E-01
ENSRNOG00000006699	**Mlh3**	mutL homolog 3 (E. coli) (Mlh3), mRN.	314320	6	116461531	116497654	36124	243,44	0,14	8,45E-01
ENSRNOG00000008319	**Ccdc130**	coiled-coil domain containing 130 (Ccc	304656	19	36343741	36351556	7816	325,45	0,13	8,45E-01
ENSRNOG00000010334		CCR4-NOT transcription complex sub	316034	8	121945850	122001591	55742	1130,51	0,15	8,45E-01
ENSRNOG00000011421	**Smap2**	small ArfGAP2 (Smap2), mRNA [Sour	298500	5	143678526	143724570	46045	3802,76	0,11	8,45E-01
ENSRNOG00000011560	**Mtmr9**	myotubularin related protein 9 (Mtmr9)	282584	15	50459294	50481325	22032	1666,84	0,11	8,45E-01
ENSRNOG00000011936	**Abhd14a**	abhydrolase domain containing 14A (A	300982	8	114505677	114513516	7840	236,49	0,15	8,45E-01
ENSRNOG00000012802	**Tenm3**	odz, odd Oz/ten-m homolog 3 (Drosop	306451	16	46462637	46657495	194859	5842,52	0,13	8,45E-01
ENSRNOG00000013253	**Armc1**	armadillo repeat containing 1 (Armc1),	294948	2	123653834	123701646	47813	1082,97	0,15	8,45E-01
ENSRNOG00000015948		solute carrier family 1 (neutral amino a	292657	1	79957648	79971750	14103	122,57	0,19	8,45E-01
ENSRNOG00000016266	**Mphosph1**	M-phase phosphoprotein 10 (U3 small	293828	1	126321919	126338110	16192	271,85	-0,21	8,45E-01
ENSRNOG00000016768	**Oprl1**	opiate receptor-like 1 (Oprl1), mRNA [	29256	3	180934268	180940194	5927	583,06	-0,15	8,45E-01
ENSRNOG00000017072	**Slc16a14**	solute carrier family 16, member 14 (S	316578	9	92237485	92261950	24466	379,82	0,14	8,45E-01
ENSRNOG00000017786	**Acta1**	actin, alpha 1, skeletal muscle (Acta1)	29437	19	67389901	67392928	3028	74,14	0,15	8,45E-01
ENSRNOG00000019555		Protein Arap1 [Source:UniProtKB/TrE	361617	1	172649120	172714995	65876	881,94	-0,18	8,45E-01
ENSRNOG00000020432	**Cic**	capicua transcriptional repressor (Cic)	308435	1	83415622	83427167	11546	5628,12	0,13	8,45E-01
ENSRNOG00000020903	**Oraov1**	oral cancer overexpressed 1 (Oraov1)	309136	1	224945387	224952084	6698	886,91	0,14	8,45E-01
ENSRNOG00000021237	**RGD15656**	RGD1565616 (RGD1565616), mRNA	499891	3	129743622	129876576	132955	3795,45	0,10	8,45E-01
ENSRNOG00000021316	**Tmem98**	transmembrane protein 98 (Tmem98),	303356	10	67842260	67853166	10907	522,53	-0,19	8,45E-01
ENSRNOG00000022143	**Dusp23**	dual specificity phosphatase 23 (Dusp	360881	13	95537373	95538565	1193	77,64	0,19	8,45E-01
ENSRNOG00000022745	**RGD13065**	uncharacterized protein LOC310158 |	310158	2	81805197	81821374	16178	672,00	0,18	8,45E-01
ENSRNOG00000025349		mannosidase, endo-alpha-like [Source	366466	5	146622170	146627751	5582	482,17	-0,10	8,45E-01
ENSRNOG00000028625	**Eif4ebp3**	eukaryotic translation initiation factor 4	1E+08	18	29196055	29197801	1747	75,79	-0,17	8,45E-01
ENSRNOG00000030216	**Abcg3l1**	ATP-binding cassette, subfamily G (W	289453	14	6468120	6520631	52512	21,02	-0,23	8,45E-01
ENSRNOG00000033765	**LOC69187**	eukaryotic translation initiation factor 1	287703	10	88020702	88022793	2092	4512,40	-0,09	8,45E-01
ENSRNOG00000046644	**Slmo2**	slowmo homolog 2 (Drosophila) (Slmo2), mRNA		3	178613098	178614956	1859	566,12	-0,09	8,45E-01
ENSRNOG00000046730		Uncharacterized protein [Source:UniProtKB/TrE		8	95752882	95762389	9508	26,78	0,18	8,45E-01
ENSRNOG00000047106	**Mtrf1**	Protein LOC686234; RCG37077, isofc	686234	15	65330214	65354186	23973	114,79	-0,21	8,45E-01
ENSRNOG00000047366		Uncharacterized protein [Source:UniProtKB/TrE		1	255228321	255228914	594	58,94	-0,16	8,45E-01
ENSRNOG00000049970		Protein LOC100909742 [Source:UniP	1E+08	20	13061351	13084547	23197	41,13	-0,18	8,45E-01
ENSRNOG00000050983	**Hpcal4**	hippocalcin-like 4 (Hpcal4), mRNA [Sc	50872	5	144660374	144672320	11947	2844,07	0,16	8,45E-01
ENSRNOG00000023202	**Usp15**	ubiquitin specific peptidase 15 (Usp15	171329	7	66793581	66884024	90444	657,70	-0,19	8,46E-01
ENSRNOG00000010646	**Tmem229b**	transmembrane protein 229B (Tmem2	503035	6	111524858	111565742	40885	3944,21	-0,13	8,46E-01

ENSRNOG00000009577	**Ndst4**	N-deacetylase/N-sulfotransferase (hep	362035	2	248551066	248852063	300998	19,18	0,23	8,47E-01
ENSRNOG00000010208	**Timp1**	TIMP metallopeptidase inhibitor 1 (Tim	116510	X	2179963	2184589	4627	690,23	0,21	8,47E-01
ENSRNOG00000014937	**Lrrc71**	leucine rich repeat containing 71 (Lrrc	310689	2	206506261	206518913	12653	338,76	-0,11	8,47E-01
ENSRNOG00000017428	**Map1b**	microtubule-associated protein 1B (Ma	29456	2	48837124	48930483	93360	79385,85	-0,14	8,47E-01
ENSRNOG00000021046	**Sult2b1**	sulfotransferase family, cytosolic, 2B,	292915	1	102791087	102853054	61968	516,76	0,15	8,47E-01
ENSRNOG00000018345	**Abce1**	ATP-binding cassette, subfamily E (OA	361390	19	42771956	42796838	24883	1879,22	-0,20	8,47E-01
ENSRNOG00000025140	**Zfat**	zinc finger and AT hook domain contai	362925	7	108980617	109147542	166926	251,79	0,12	8,47E-01
ENSRNOG00000009594	**Snai1**	snail family zinc finger 1 (Snai1), mRN	116490	3	170425898	170430382	4485	35,64	0,21	8,47E-01
ENSRNOG00000015644	**Ugcg**	UDP-glucose ceramide glucosyltransf	83626	5	80530140	80562612	32473	3151,00	-0,16	8,47E-01
ENSRNOG00000007120	**Mtmr3**	myotubularin related protein 3 (Mtmr3)	305482	14	85381833	85500445	118613	4309,21	0,12	8,47E-01
ENSRNOG00000009125	**Dis3**	DIS3 mitotic control homolog (S. cerev	306103	15	86977711	87005406	27696	380,43	-0,19	8,47E-01
ENSRNOG00000004351	**Slc25a29**	solute carrier family 25 (mitochondrial	314441	6	141907050	141917838	10789	378,28	0,15	8,47E-01
ENSRNOG00000007527	**Tm2d1**	TM2 domain containing 1 (Tm2d1), ml	362545	5	120939559	120981789	42231	1082,50	0,10	8,47E-01
ENSRNOG00000008256	**Mrpl38**	mitochondrial ribosomal protein L38 (M	303685	10	103596443	103603247	6805	696,27	0,16	8,47E-01
ENSRNOG00000012021	**Ctnnbl1**	catenin, beta like 1 (Ctnnbl1), mRNA [	296320	3	161228794	161389518	160725	1304,18	0,11	8,47E-01
ENSRNOG00000017939	**Chn1**	chimerin (chimaerin) 1 (Chn1), mRNA	84030	3	66992186	67035054	42869	733,65	-0,13	8,47E-01
ENSRNOG00000018987		CREB/ATF bZIP transcription factor [S	293112	1	162537394	162542285	4892	1251,13	0,17	8,47E-01
ENSRNOG00000023837	**Mtx3**	Protein LOC688905 [Source:UniProtK	688905	2	41632738	41640357	7620	96,89	-0,15	8,47E-01
ENSRNOG00000045846	**Rfx2**	regulatory factor X, 2 (influences HLA	301121	9	9214191	9280395	66205	219,10	-0,20	8,47E-01
ENSRNOG00000005826		Protein RGD1562420 [Source:UniPro	314532	6	155306094	155306663	570	48,60	0,22	8,48E-01
ENSRNOG00000001844	**Klhl42**	kelch-like family, member 42 (Klhl42),	500367	4	245631025	245652794	21770	499,62	0,10	8,48E-01
ENSRNOG00000012215		brain-specific angiogenesis inhibitor 1-	685357	7	120486375	120511795	25421	19,61	0,21	8,48E-01
ENSRNOG00000007763	**Plod1**	procollagen-lysine, 2-oxoglutarate 5-di	116552	5	168379097	168405535	26439	801,56	-0,16	8,48E-01
ENSRNOG00000008596	**Ttll9**	tubulin tyrosine ligase-like family, mem	311548	3	154841931	154888352	46422	46,85	0,20	8,48E-01
ENSRNOG00000013190	**Rnaset2**	ribonuclease T2 (Rnaset2), mRNA [Sc	292306	1	54425133	54442302	17170	404,42	0,16	8,48E-01
ENSRNOG00000014218	**Tmem25**	transmembrane protein 25 (Tmem25),	689172	8	47719267	47724685	5419	223,07	-0,15	8,48E-01
ENSRNOG00000005667		astrotactin 1 precursor [Source:RefSe	304900	13	80970701	81285187	314487	7286,71	-0,12	8,48E-01
ENSRNOG00000010526	**Dennd1a**	DENN/MADD domain containing 1A (D	311913	3	27296835	27729383	432549	1561,54	0,09	8,48E-01
ENSRNOG00000024247	**Srsf12**	serine/arginine-rich splicing factor 12 (	297962	5	53038883	53066076	27194	143,83	0,15	8,49E-01
ENSRNOG00000008964	**Tep1**	telomerase associated protein 1 (Tep1	64523	15	31583613	31631251	47639	275,81	-0,20	8,49E-01
ENSRNOG00000003997	**Pld5**	phospholipase D family, member 5 (Pl	289270	13	98486317	98812030	325714	44,53	-0,21	8,49E-01
ENSRNOG00000028659	**Szt2**	Protein Szt2 [Source:UniProtKB/TrEM	362573	5	140980270	141025836	45567	1011,08	-0,09	8,49E-01
ENSRNOG00000003472		Protein Atp11c [Source:UniProtKB/TrE	317599	X	143369611	143482681	113071	355,43	-0,14	8,49E-01
ENSRNOG00000001033	**Nufip1**	nuclear fragile X mental retardation pr	364430	15	61872647	61903369	30723	740,62	0,11	8,49E-01
ENSRNOG00000008978	**Trmt12**	tRNA methyltransferase 12 homolog (	314999	7	99348971	99350595	1625	177,59	0,14	8,49E-01
ENSRNOG00000011678	**Wbp4**	WW domain binding protein 4 (Wbp4),	114765	15	65410542	65436878	26337	205,64	-0,14	8,49E-01
ENSRNOG00000014004		Protein Ncoa7; Similar to nuclear rece	498995	1	30738090	30828265	90176	1049,00	0,14	8,49E-01
ENSRNOG00000016705	**Prpf4b**	PRP4 pre-mRNA processing factor 4 h	291078	17	32583633	32614446	30814	1440,17	-0,18	8,49E-01

ENSRNOG00000021218	**Pias3**	protein inhibitor of activated STAT, 3 (I	83614	2	218310301	218318466	8166	780,80	-0,11	8,49E-01
ENSRNOG00000001928		interleukin 1 receptor accessory protei	25466	11	83123891	83253144	129254	218,37	0,10	8,49E-01
ENSRNOG00000001355		Transcription initiation factor TFIID subunit 6 [S		12	21378048	21386267	8220	1462,58	0,09	8,50E-01
ENSRNOG00000011967	**Nphp4**	nephronophthisis 4 (Nphp4), mRNA [S	313749	5	173212951	173298420	85470	451,77	0,09	8,50E-01
ENSRNOG00000013884	**Psd3**	Protein Psd3 [Source:UniProtKB/TrEI	306380	16	23327925	23666165	338241	957,15	-0,15	8,50E-01
ENSRNOG00000042482	**Tatdn2**	TatD DNase domain containing 2 (Tat	500295	4	208950001	208965742	15742	1832,41	0,14	8,50E-01
ENSRNOG00000013059	**Aste1**	asteroid homolog 1 (Drosophila) (Aste	363130	8	113322528	113333049	10522	28,85	0,20	8,50E-01
ENSRNOG00000046471	**Fmn2**	Protein LOC100360457 [Source:UniP	1E+08	13	97036274	97135362	99089	830,00	0,12	8,50E-01
ENSRNOG00000007764	**Frmd4b**	Protein Frmd4b [Source:UniProtKB/T	252858	4	194159317	194342877	183561	262,84	-0,17	8,50E-01
ENSRNOG00000017294	**Zranb1**	Protein LOC100360606; RCG48022, i	1E+08	1	211953388	211990331	36944	774,82	-0,15	8,50E-01
ENSRNOG00000029879	**Mcrs1**	microspherule protein 1 (Mcrs1), mRN	300222	X	115414777	115423588	8812	2429,74	0,12	8,50E-01
ENSRNOG00000001106		density-regulated protein [Source:MGI Symbol;A		12	39966924	39987019	20096	755,23	0,14	8,50E-01
ENSRNOG00000004624	**Rnd3**	Rho family GTPase 3 (Rnd3), mRNA [	295588	3	41756399	41774581	18183	756,17	-0,19	8,50E-01
ENSRNOG00000006450		v-erb-b2 avian erythroblastic leukemia	24337	10	86163531	86187663	24133	102,92	0,17	8,50E-01
ENSRNOG00000010109	**Nol9**	Nucleolar protein 9; Protein Nol9 [Sou	313744	5	172771378	172789897	18520	1528,27	0,10	8,50E-01
ENSRNOG00000015353	**Prss12**	protease, serine, 12 neurotrypsin (mot	85266	2	247015139	247074636	59498	233,62	0,19	8,50E-01
ENSRNOG00000015383		additional sex combs like 3 (Drosophila) [Source		18	13136947	13274883	137937	535,65	-0,12	8,50E-01
ENSRNOG00000015577	**Lpar6**	lysophosphatidic acid receptor 6 (Lpar	691774	15	58850070	58851878	1809	39,01	-0,22	8,50E-01
ENSRNOG00000021158	**Nudt22**	nudix (nucleoside diphosphate linked i	293703	1	229238281	229241759	3479	242,92	0,16	8,50E-01
ENSRNOG00000000774		gamma-aminobutyric acid (GABA) B r	81657	20	3996333	4025427	29095	22993,37	-0,10	8,50E-01
ENSRNOG00000016357	**Casc4**	cancer susceptibility candidate 4 (Cas	362204	3	120358106	120424427	66322	1617,98	-0,13	8,50E-01
ENSRNOG00000002192	**Rell1**	RELT-like 1 (Rell1), mRNA [Source:Re	289635	14	45787243	45835906	48664	49,87	-0,17	8,50E-01
ENSRNOG00000004269		myelin transcription factor 1-like protei	116668	6	57146288	57529924	383637	3122,52	-0,12	8,50E-01
ENSRNOG00000005434	**Sptbn1**	spectrin, beta, non-erythrocytic 1 (Sptl	305614	14	114188059	114356233	168175	22950,58	-0,09	8,50E-01
ENSRNOG00000006548	**Mrc2**	mannose receptor, C type 2 (Mrc2), m	498011	10	93278918	93339315	60398	220,92	-0,13	8,50E-01
ENSRNOG00000007576	**Zfp597**	zinc finger protein 597 (Zfp597), mRN	266774	10	10668906	10674261	5356	316,60	-0,13	8,50E-01
ENSRNOG00000009298		F-box only protein 44 [Source:RefSec	500587	5	168620959	168644538	23580	1322,03	0,09	8,50E-01
ENSRNOG00000017494	**Acp6**	acid phosphatase 6, lysophosphatidic	295305	2	218767629	218789268	21640	279,73	0,11	8,50E-01
ENSRNOG00000030264	**Fam83h**	family with sequence similarity 83, me	362937	7	117048588	117056776	8189	157,30	0,15	8,50E-01
ENSRNOG00000014698	**Chrnb1**	cholinergic receptor, nicotinic, beta 1 (	24261	10	56135720	56148237	12518	56,81	0,18	8,50E-01
ENSRNOG00000022736	**Cdkn2aip**	CDKN2A interacting protein (Cdkn2aip	306455	16	47385309	47388586	3278	528,20	-0,13	8,50E-01
ENSRNOG00000012124	**Trappc13**	trafficking protein particle complex 13	294709	2	53350386	53383214	32829	905,01	0,15	8,50E-01
ENSRNOG00000001953	**Bace2**	beta-site APP-cleaving enzyme 2 (Bac	288227	11	41305970	41388420	82451	188,59	-0,13	8,51E-01
ENSRNOG00000047895	**Mettl1**	Protein LOC679091 [Source:UniProtK	679091	7	70506428	70509927	3500	128,28	0,17	8,51E-01
ENSRNOG00000008551	**LOC10036**	ribosomal protein S7 (Rps7), mRNA [S	29258	6	56601565	56606473	4909	2978,51	0,13	8,51E-01
ENSRNOG00000000190		E1A-binding protein; Protein Ep300 [Source:Un		7	122793713	122821002	27290	319,39	-0,11	8,51E-01
ENSRNOG00000047406	**RGD13085**	LOC361192 (Predicted), isoform CRA	361192	1	39083932	39107584	23653	258,52	0,13	8,51E-01
ENSRNOG00000003840	**Slit2**	slit homolog 2 (Drosophila) (Slit2), mR	360272	14	66864065	67201603	337539	1002,02	-0,13	8,51E-01

ENSRNOG00000021314	**Fdft1**	farnesyl diphosphate farnesyl transfer	29580	15	50104043	50132096	28054	5260,01	0,18	8,51E-01
ENSRNOG00000023411		ZV-set and immunoglobulin domain cc	308556	1	99538138	99544067	5930	85,11	-0,17	8,51E-01
ENSRNOG00000015808	**Prkrir**	protein-kinase, interferon-inducible do	308845	1	169866610	169882330	15721	1752,80	-0,11	8,51E-01
ENSRNOG00000011796		complement C1r subcomponent precu	312705	4	224144564	224154807	10244	573,51	0,20	8,51E-01
ENSRNOG00000027089	**Ell2**	Protein Ell2 [Source:UniProtKB/TrEM	309918	2	2450088	2518045	67958	287,76	-0,13	8,51E-01
ENSRNOG00000006668				6	32343030	32343379	350	20,07	-0,22	8,51E-01
ENSRNOG00000011954	**Kiss1r**	KISS1 receptor (Kiss1r), mRNA [Sour	78976	7	12776069	12779590	3522	31,19	0,18	8,51E-01
ENSRNOG00000026212	**Micall1**	Protein Micall1 [Source:UniProtKB/Tr	362958	7	120339357	120364917	25561	790,44	-0,12	8,51E-01
ENSRNOG00000028627		hemicentin-1 precursor [Source:RefS	289094	13	72887378	73321707	434330	37,88	-0,17	8,51E-01
ENSRNOG00000039191	**Yjefn3**	Protein Yjefn3 [Source:UniProtKB/TrE	498608	16	21202779	21204490	1712	37,25	-0,20	8,51E-01
ENSRNOG00000011459	**Rhbdf2**	rhomboid 5 homolog 2 (Drosophila) (R	303690	10	105236676	105243042	6367	36,51	0,19	8,51E-01
ENSRNOG00000026403	**Naf1**	nuclear assembly factor 1 ribonucleop	306387	16	24466738	24496546	29809	366,09	0,13	8,51E-01
ENSRNOG00000049758	**Tbc1d16**	Protein Tbc1d16 [Source:UniProtKB/T	303734	10	107871279	107934334	63056	2201,58	0,16	8,51E-01
ENSRNOG00000016133	**Sumo1**	small ubiquitin-like modifier 1 (Sumo1)	301442	9	66258863	66288103	29241	1817,74	-0,15	8,52E-01
ENSRNOG00000019921	**Rhbdl1**	rhomboid, veinlet-like 1 (Drosophila) (I	117025	10	15014446	15017086	2641	829,85	-0,17	8,52E-01
ENSRNOG00000018621		leukocyte receptor cluster (LRC) mem	1E+08	1	74825506	74827023	1518	107,60	0,13	8,52E-01
ENSRNOG00000029402	**LOC49940**	LRRGT00097 (LOC499407), mRNA [S	499407	20	9532258	9563211	30954	32,07	-0,20	8,52E-01
ENSRNOG00000047757		Epc1 protein; Uncharacterized protein [Source:U		17	55393325	55395236	1912	106,62	0,13	8,52E-01
ENSRNOG00000011076		ankyrin 2, brain [Source:MGI Symbol;Acc:MGI:8		2	250581158	250869034	287877	19714,74	-0,11	8,52E-01
ENSRNOG00000001694		Uncharacterized protein [Source:UniProtKB/TrE		11	45701160	45702169	1010	66,36	0,21	8,53E-01
ENSRNOG00000011365	**Emb**	embigin (Emb), mRNA [Source:RefSe	114511	2	68054520	68107903	53384	154,85	0,15	8,53E-01
ENSRNOG00000033351	**Zfp869**	Protein Zfp869; RCG38640, isoform C	1E+08	16	21358627	21362836	4210	172,35	-0,13	8,53E-01
ENSRNOG00000048553	**Iqcc**	Protein LOC682431 [Source:UniProtK	682431	5	151548619	151552255	3637	146,49	0,15	8,53E-01
ENSRNOG00000050231	**Gng12**	guanine nucleotide binding protein (G	114120	4	162543862	162545626	1765	126,39	0,15	8,53E-01
ENSRNOG00000029571	**Coq10a**	coenzyme Q10 homolog A (S. cerevisi	362810	7	2759394	2765320	5927	832,28	0,13	8,53E-01
ENSRNOG00000004774	**Ccdc175**	coiled-coil domain containing 175 (Cc	500668	6	104294725	104377617	82893	71,73	0,20	8,53E-01
ENSRNOG00000047679	**Dusp28**	Protein LOC684024 [Source:UniProtK	684024	9	99742099	99742740	642	31,25	0,17	8,53E-01
ENSRNOG00000004760	**Lars2**	leucyl-tRNA synthetase 2, mitochondri	363172	8	131594014	131689033	95020	538,16	-0,08	8,53E-01
ENSRNOG00000008788	**Mpp5**	membrane protein, palmitoylated 5 (M	314259	6	111299906	111403406	103501	349,94	-0,20	8,53E-01
ENSRNOG00000011319	**Zbtb10**	zinc finger and BTB domain containing	80338	2	114442396	114479723	37328	290,19	0,12	8,53E-01
ENSRNOG00000024125	**Slain1**	SLAIN motif family, member 1 (Slain1)	361087	15	91364574	91401697	37124	918,32	0,11	8,53E-01
ENSRNOG00000033318	**Rfxank**	regulatory factor X-associated ankyrin	306353	16	20879467	20886949	7483	183,94	0,16	8,53E-01
ENSRNOG00000048723	**Pros1**	protein S (alpha) (Pros1), mRNA [Sou	81750	7	1199864	1279990	80127	203,30	0,15	8,53E-01
ENSRNOG00000007707	**Zfp467**	zinc finger protein 467 (Zfp467), mRN	500110	4	142732565	142739034	6470	363,77	-0,13	8,53E-01
ENSRNOG00000008104	**LOC68114**	tyrosine 3-monooxygenase/tryptophar	25577	6	60331003	60361242	30240	6760,44	0,12	8,53E-01
ENSRNOG00000011427	**Hr**	hair growth associated (Hr), mRNA [S	60563	15	55968992	55984793	15802	276,57	0,21	8,53E-01
ENSRNOG00000015004		Fibrinogen silencer-binding protein [Source:Unl		5	29883352	29887996	4645	30,04	0,21	8,53E-01
ENSRNOG00000016816	**Phf2**	PHD finger protein 2 (Phf2), mRNA [S	306814	17	18301177	18332952	31776	2204,78	-0,10	8,53E-01

ENSRNOG00000021173	**Vps45**	vacuolar protein sorting 45 homolog (S	64516	2	217613524	217674281	60758	1244,35	0,16	8,53E-01
ENSRNOG00000032748	**Abcc1**	ATP-binding cassette, subfamily C (CF	24565	10	11232233	11354931	122699	496,74	0,17	8,53E-01
ENSRNOG00000001870	**Lztr1**	leucine-zipper-like transcription regula	360745	11	90433248	90448806	15559	1794,42	-0,09	8,54E-01
ENSRNOG00000002112	**Zfp644**	zinc finger protein 644 (Zfp644), mRN.	305127	14	4120819	4195995	75177	1043,36	-0,15	8,54E-01
ENSRNOG00000002994	**Popdc2**	popeye domain containing 2 (Popdc2)	360718	11	67129358	67149796	20439	47,06	-0,16	8,54E-01
ENSRNOG00000005574	**Adamts8**	Protein Adamts8 [Source:UniProtKB/T	300475	8	32044798	32063464	18667	106,45	-0,13	8,54E-01
ENSRNOG00000011541	**Cygb**	cytoglobin (Cygb), mRNA [Source:Ref	170520	10	105280905	105290670	9766	436,49	-0,11	8,54E-01
ENSRNOG00000003815	**Slc25a11**	solute carrier family 25 (mitochondrial	64201	10	57010935	57013640	2706	2559,76	0,07	8,54E-01
ENSRNOG00000021013	**Stx3**	syntaxin 3 (Stx3), mRNA [Source:RefS	81802	1	235218151	235262711	44561	253,22	0,21	8,54E-01
ENSRNOG00000000604	**Hdac2**	histone deacetylase 2 (Hdac2), mRNA	84577	20	44811967	44835337	23371	3905,12	0,12	8,54E-01
ENSRNOG00000007474		suppression of tumorigenicity 7 (ST7),	296911	4	45604814	45851998	247185	934,39	0,17	8,54E-01
ENSRNOG00000015540		protein phosphatase 6, regulatory sub	309144	1	225564228	225682638	118411	3064,84	0,10	8,54E-01
ENSRNOG00000012852	**Bin1**	bridging integrator 1 (Bin1), mRNA [Sc	117028	18	24878722	24937708	58987	3560,20	0,11	8,54E-01
ENSRNOG00000002463	**Snn**	stannin (Snn), mRNA [Source:RefSeq	29140	10	3467738	3468004	267	1102,52	0,18	8,54E-01
ENSRNOG00000000814	**Fabp7**	fatty acid binding protein 7, brain (Fab	80841	20	42501163	42504695	3533	22354,29	-0,19	8,54E-01
ENSRNOG00000002105	**Cdc7**	cell division cycle 7 (Cdc7), mRNA [Sc	360908	14	3823737	3843969	20233	281,67	0,15	8,54E-01
ENSRNOG00000004903	**Ebp**	emopamil binding protein (sterol isome	117278	X	15832773	15839092	6320	178,00	0,12	8,54E-01
ENSRNOG00000005989		sodium channel protein type 1 subunit	81574	3	59009807	59089000	79194	271,40	0,11	8,54E-01
ENSRNOG00000012199	**Sox2**	SRY (sex determining region Y)-box 2	499593	2	140810593	140813001	2409	5450,05	-0,13	8,54E-01
ENSRNOG00000013013	**Tle3**	transducin-like enhancer of split 3 (E(s	84424	8	66038798	66084091	45294	3265,21	-0,09	8,54E-01
ENSRNOG00000015304	**Tmem160**	transmembrane protein 160 (Tmem16	292654	1	79664960	79667668	2709	1137,19	0,17	8,54E-01
ENSRNOG00000017456	**Vstm2b**	V-set and transmembrane domain con	361560	1	98716586	98744919	28334	568,78	-0,15	8,54E-01
ENSRNOG00000018082	**Slc26a2**	solute carrier family 26 (anion exchang	117267	18	55751800	55765475	13676	118,00	-0,13	8,54E-01
ENSRNOG00000018198	**Dapk1**	death associated protein kinase 1 (Da	306722	17	6525247	6683920	158674	2976,88	0,12	8,54E-01
ENSRNOG00000024924	**Cep19**	centrosomal protein 19 (Cep19), mRN	192229	11	75004786	75013404	8619	709,61	0,15	8,54E-01
ENSRNOG00000038616	**Cox6b2**	cytochrome c oxidase subunit VIb poly	654441	1	75873174	75874397	1224	30,09	-0,20	8,54E-01
ENSRNOG00000032882				MT	6865	6933	69	2262,56	-0,15	8,54E-01
ENSRNOG00000009057	**Sec62**	SEC62 homolog (S. cerevisiae) (Sec6	294912	2	135885423	135914374	28952	855,53	-0,17	8,55E-01
ENSRNOG00000012015	**Lrrc49**	leucine rich repeat containing 49 (Lrrc	300763	8	65233821	65339662	105842	1751,72	-0,09	8,55E-01
ENSRNOG00000010230	**Fam45a**	family with sequence similarity 45, me	308009	1	289526637	289550131	23495	885,26	-0,10	8,55E-01
ENSRNOG00000013691		Uncharacterized protein [Source:UniProtKB/TrE		7	120758999	120772909	13911	4975,53	-0,15	8,55E-01
ENSRNOG00000050398				7	113659666	113676676	17011	191,11	0,12	8,55E-01
ENSRNOG00000002516	**Mtm1**	myotubularin 1 (Mtm1), mRNA [Source	288762	6	28722724	28736837	14114	655,36	0,12	8,55E-01
ENSRNOG00000008519		uncharacterized protein LOC315891 |	315891	8	101571619	101642463	70845	248,05	0,13	8,55E-01
ENSRNOG00000020792	**Etv4**	ets variant 4 (Etv4), mRNA [Source:Re	360635	10	89481284	89496509	15226	444,21	0,17	8,55E-01
ENSRNOG00000036572	**Sfxn4**	sideroflexin 4 (Sfxn4), mRNA [Source:	361778	1	289552169	289574442	22274	108,05	-0,12	8,55E-01
ENSRNOG00000007779	**Kank4**	KN motif and ankyrin repeat domains 4	313385	5	121318387	121381170	62784	41,65	-0,20	8,55E-01
ENSRNOG00000011476	**Nars2**	asparaginyl-tRNA synthetase 2 (mitoc	293128	1	168135529	168218290	82762	254,72	-0,12	8,55E-01

ENSRNOG00000011884	**Golga2**	golgin A2 (Golga2), mRNA [Source:Re	64528	3	16666198	16686435	20238	2538,46	0,10	8,55E-01
ENSRNOG00000012322	**Pex7**	peroxisomal biogenesis factor 7 (Pex7	308718	1	16860050	16923047	62998	230,19	-0,11	8,55E-01
ENSRNOG00000016251	**Dnaja2**	DnaJ (Hsp40) homolog, subfamily A, r	84026	19	33576436	33594574	18139	3170,50	0,12	8,55E-01
ENSRNOG00000016794	**Phyhd1**	phytanoyl-CoA dioxygenase domain c	296621	3	14185690	14199073	13384	368,41	0,19	8,55E-01
ENSRNOG00000017748	**Nkx6-2**	NK6 homeobox 2 (Nkx6-2), mRNA [Sc	309095	1	218846384	218847924	1541	25,13	-0,22	8,55E-01
ENSRNOG00000019564	**Sptbn2**	spectrin, beta, non-erythrocytic 2 (Sptl	29211	1	226828798	226869726	40929	14838,77	-0,16	8,55E-01
ENSRNOG00000019768	**Ncoa4**	nuclear receptor coactivator 4 (Ncoa4	619385	16	8219983	8240323	20341	3113,47	-0,13	8,55E-01
ENSRNOG00000032768	**Stxbp4**	syntaxin binding protein 4 (Stxbp4), m	303443	10	77871153	78023722	152570	219,87	-0,13	8,55E-01
ENSRNOG00000037518	**Hyi**	Hydroxypyruvate isomerase [Source:l	500536	5	140977015	140979672	2658	104,49	0,19	8,55E-01
ENSRNOG00000013714	**Snrpd1**	small nuclear ribonucleoprotein D1 (Sı	291794	18	1898841	1909374	10534	1256,15	-0,10	8,55E-01
ENSRNOG00000005374	**Lyar**	Ly1 antibody reactive (Lyar), mRNA [S	289707	14	77290080	77303645	13566	266,24	0,17	8,55E-01
ENSRNOG00000011376	**Ccdc176**	coiled-coil domain containing 176 (Ccı	500693	6	117396790	117422542	25753	76,19	-0,15	8,55E-01
ENSRNOG00000013156	**Cnot3**	CCR4-NOT transcription complex, sub	308311	1	63123105	63139348	16244	1411,11	0,10	8,55E-01
ENSRNOG00000017647	**Zc3h8**	zinc finger CCCH type containing 8 (Z	311414	3	128523840	128540532	16693	612,67	-0,15	8,55E-01
ENSRNOG00000020264	**Dhrs1**	dehydrogenase/reductase (SDR family	290234	15	38313184	38320467	7284	676,82	0,11	8,55E-01
ENSRNOG00000021962	**Fzd2**	frizzled family receptor 2 (Fzd2), mRN	64512	10	90343133	90345043	1911	899,61	0,15	8,55E-01
ENSRNOG00000047504		Protein Astn2 [Source:UniProtKB/TrEMBL;Acc:M		5	85464437	85573482	109046	111,21	0,12	8,55E-01
ENSRNOG00000013961	**Ghitm**	growth hormone inducible transmembı	290596	16	14273917	14285152	11236	6242,51	0,11	8,55E-01
ENSRNOG00000017536	**Cdc16**	cell division cycle 16 (Cdc16), mRNA [	290875	16	80981550	81004585	23036	2180,87	0,16	8,56E-01
ENSRNOG00000018996	**Phf7**	PHD finger protein 7 (Phf7), mRNA [S	364510	16	7251919	7264620	12702	189,67	0,14	8,56E-01
ENSRNOG00000003653	**Fh**	fumarate hydratase (Fh), mRNA [Sour	24368	13	98116501	98142647	26147	992,36	0,17	8,56E-01
ENSRNOG00000007131	**Paxip1**	PAX interacting (with transcription-acti	311944	4	4000253	4047988	47736	1328,27	0,11	8,56E-01
ENSRNOG00000008061	**Nuak1**	NUAK family, SNF1-like kinase, 1 (Nu	299694	7	25184054	25253032	68979	476,96	-0,14	8,56E-01
ENSRNOG00000011621	**LOC10091**	heterogeneous nuclear ribonucleoprot	290046	15	32297190	32327569	30380	43,05	0,18	8,56E-01
ENSRNOG00000013783	**Efhd2**	EF-hand domain family, member D2 (I	298609	5	164119687	164135720	16034	3750,23	0,15	8,56E-01
ENSRNOG00000015493	**Emx1**	Protein Emx1 [Source:UniProtKB/TrE	500235	4	181547461	181563885	16425	482,99	0,17	8,56E-01
ENSRNOG00000033256	**LOC69114**	cDNA sequence AB124611 [Source:M	691141	8	22613207	22630263	17057	47,38	-0,17	8,56E-01
ENSRNOG00000037137	**Svip**	small VCP/p97-interacting protein (Svi	499157	1	108412833	108415318	2486	178,60	0,13	8,56E-01
ENSRNOG00000037291	**Plxnb3**	plexin B3 (Plxnb3), mRNA [Source:Re	363517	1	152764192	152777321	13130	204,93	-0,22	8,56E-01
ENSRNOG00000043150	**Arfgap1**	ADP-ribosylation factor GTPase activa	246310	3	180185720	180199752	14033	1810,83	0,11	8,56E-01
ENSRNOG00000003712	**Smek2**	SMEK homolog 2, suppressor of mek1	360993	14	113245254	113293242	47989	1029,39	-0,19	8,56E-01
ENSRNOG00000005691		ring finger protein 32 (Rnf32), mRNA [	311936	4	2108922	2122183	13262	32,47	-0,18	8,56E-01
ENSRNOG00000029798	**Lrrc4c**	leucine rich repeat containing 4C (Lrrc	311236	3	93303511	93502229	198719	948,97	-0,13	8,56E-01
ENSRNOG00000003546	**Tnfrsf12a**	tumor necrosis factor receptor superfa	302965	10	12818949	12820979	2031	307,19	0,19	8,56E-01
ENSRNOG00000020288	**Slc25a20**	solute carrier family 25 (carnitine/acylc	117035	8	116800146	116821558	21413	1932,82	0,12	8,56E-01
ENSRNOG00000026898	**Zbtb8b**	zinc finger and BTB domain containing	500553	5	151348210	151355100	6891	230,65	-0,14	8,56E-01
ENSRNOG00000030410	**Zfp958**	zinc finger protein 958 (Zfp958), mRN	1E+08	12	7595233	7600265	5033	30,11	-0,19	8,56E-01
ENSRNOG00000001351	**Trafd1**	TRAF type zinc finger domain containi	114635	12	42562635	42576553	13919	3658,91	0,11	8,56E-01

ENSRNOG00000009478	**RGD13591**	similar to RIKEN cDNA 3110043O21 (	313155	5	55229460	55254965	25506	1254,05	-0,18	8,56E-01
ENSRNOG00000031778	**Mef2d**	myocyte enhancer factor 2D (Mef2d),	81518	2	206915181	206941043	25863	1323,09	0,09	8,56E-01
ENSRNOG00000000809	**Atat1**	alpha tubulin acetyltransferase 1 (Atat	361789	20	5448826	5462080	13255	3064,61	0,09	8,56E-01
ENSRNOG00000042501	**RGD15643**	RGD1564379 (RGD1564379), mRNA	499758	3	8952576	8956268	3693	1354,84	0,13	8,56E-01
ENSRNOG00000013946	**Rnf149**	Protein Rnf149 [Source:UniProtKB/Tr	363222	9	45974158	45998057	23900	709,91	0,17	8,57E-01
ENSRNOG00000016834		WD repeat-containing protein 37 [Sou	307075	17	59811420	59855683	44264	1992,97	0,15	8,57E-01
ENSRNOG00000018029	**Doc2g**	double C2-like domains, gamma (Doc2	293654	1	226120916	226124439	3524	46,49	0,18	8,57E-01
ENSRNOG00000014585	**Ccdc11**	Protein Ccdc11 [Source:UniProtKB/Tr	364899	18	69405189	69471842	66654	26,00	0,17	8,57E-01
ENSRNOG00000016889	**Fancc**	Fanconi anemia, complementation grc	24361	17	826322	913387	87066	135,87	0,14	8,57E-01
ENSRNOG00000029941	**Grina**	glutamate receptor, ionotropic, N-meth	266668	7	117290445	117293619	3175	9300,78	-0,14	8,57E-01
ENSRNOG00000018877	**Zfp629**	zinc finger protein 629 (Zfp629), mRN	308998	1	206078605	206084317	5713	1463,10	0,12	8,57E-01
ENSRNOG00000002272	**Lnx1**	ligand of numb-protein X 1, E3 ubiquit	360926	14	35873425	35976075	102651	751,16	0,10	8,57E-01
ENSRNOG00000007939	**Naprt1**	nicotinate phosphoribosyltransferase c	315085	7	116814964	116818403	3440	760,98	-0,19	8,57E-01
ENSRNOG00000015385	**Pink1**	PTEN induced putative kinase 1 (Pink	298575	5	160426051	160437821	11771	1564,83	0,13	8,57E-01
ENSRNOG00000010161	**Myo10**	myosin X (Myo10), mRNA [Source:Re	310178	2	97589017	97789261	200245	4159,72	-0,16	8,57E-01
ENSRNOG00000024503	**LOC69007**	neuroblastoma amplified sequence [S	690073	6	47240439	47541152	300714	1148,84	0,10	8,57E-01
ENSRNOG00000039658	**G4**	G4 protein (G4), mRNA [Source:RefSe	406868	20	7191265	7193591	2327	330,23	-0,13	8,57E-01
ENSRNOG00000028816		bromodomain adjacent to zinc finger d	304601	7	2507667	2526280	18614	1117,46	-0,09	8,57E-01
ENSRNOG00000004186	**Snx13**	sorting nexin 13 (Snx13), mRNA [Sour	362731	6	64112186	64212889	100704	649,18	-0,18	8,57E-01
ENSRNOG00000004377	**Lpin1**	lipin 1 (Lpin1), mRNA [Source:RefSeq	313977	6	51534870	51602407	67538	514,17	0,13	8,57E-01
ENSRNOG00000007042	**Purb**	purine rich element binding protein B (	498407	14	80372896	80373849	954	433,21	-0,13	8,57E-01
ENSRNOG00000008859	**Tank**	TRAF family member-associated NFK	252961	3	54111817	54239689	127873	123,47	-0,17	8,57E-01
ENSRNOG00000016260	**Polr3g**	polymerase (RNA) III (DNA directed) p	685465	2	9417665	9458293	40629	217,11	-0,13	8,57E-01
ENSRNOG00000017113	**Ccnt2**	cyclin T2 (Ccnt2), mRNA [Source:RefS	304758	13	49560748	49600517	39770	1262,85	-0,19	8,57E-01
ENSRNOG00000019779	**Disc1**	disrupted in schizophrenia 1 (Disc1), r	307940	19	68529791	68769050	239260	76,04	-0,16	8,57E-01
ENSRNOG00000028359	**Exoc6b**	exocyst complex component 6B (Exoc	500233	4	180900780	181368585	467806	1953,68	0,09	8,57E-01
ENSRNOG00000029826		centrosomal protein 164 [Source:MGI	363055	8	48694831	48751727	56897	482,04	0,11	8,57E-01
ENSRNOG00000042657	**Mob1a**	MOB kinase activator 1A (Mob1a), mR	297387	4	179612702	179631467	18766	737,98	-0,14	8,57E-01
ENSRNOG00000008987	**Rnf139**	ring finger protein 139 (Rnf139), mRN	315000	7	99371053	99381956	10904	875,60	0,11	8,58E-01
ENSRNOG00000012564	**RGD15645**	uncharacterized protein LOC313433		313433	X	123605221	123631755	26535	582,23	0,15	8,58E-01
ENSRNOG00000043178	**Zfp655**	zinc finger protein 655 (Zfp655), mRN	360764	12	13197574	13213969	16396	58,58	0,15	8,58E-01
ENSRNOG00000048320	**Ndufa11**	NADH dehydrogenase (ubiquinone) 1	301123	9	9331184	9335243	4060	2940,06	0,16	8,58E-01
ENSRNOG00000003029	**Calr**	calreticulin (Calr), mRNA [Source:RefS	64202	19	36931420	36937294	5875	13069,25	0,10	8,58E-01
ENSRNOG00000004481	**Adss**	adenylosuccinate synthase (Adss), mR	289276	13	100355979	100386314	30336	1222,48	-0,10	8,58E-01
ENSRNOG00000009789	**Topbp1**	Protein Topbp1 [Source:UniProtKB/Tr	315969	8	111183198	111226187	42990	661,25	0,15	8,58E-01
ENSRNOG00000011279		Protein Cmc2; RCG51296 [Source:Ur	1E+08	19	60224644	60248690	24047	314,27	0,16	8,58E-01
ENSRNOG00000013255	**Dnajc8**	DnaJ (Hsp40) homolog, subfamily C, r	313035	5	154393926	154411983	18058	2547,92	0,16	8,58E-01
ENSRNOG00000015292	**Cul2**	cullin 2 (Cul2), mRNA [Source:RefSeq	361258	17	54986452	55026463	40012	1230,60	-0,15	8,58E-01

ENSRNOG00000017420	**Nudt6**	nudix (nucleoside diphosphate linked r	207120	2	143742244	143757825	15582	165,15	-0,13	8,58E-01
ENSRNOG00000019465	**Gnai3**	guanine nucleotide binding protein (G	25643	2	230365905	230404045	38141	2249,13	0,15	8,58E-01
ENSRNOG00000019649	**Cul4a**	cullin 4A (Cul4a), mRNA [Source:RefS	361181	16	81204992	81242020	37029	2037,85	0,13	8,58E-01
ENSRNOG00000026718				4	211475002	211476581	1580	18,84	0,19	8,58E-01
ENSRNOG00000037793	**Cdk5r2**	cyclin-dependent kinase 5, regulatory	501164	9	81888640	81890108	1469	2632,95	0,18	8,58E-01
ENSRNOG00000005281	**Stx16**	syntaxin 16 (Stx16), mRNA [Source:R	362283	3	178207252	178234585	27334	1047,63	0,16	8,58E-01
ENSRNOG00000002012	**Donson**	downstream neighbor of SON (Donso	288257	11	35446572	35456223	9652	416,97	-0,16	8,59E-01
ENSRNOG00000015895	**B4galt6**	UDP-Gal:betaGlcNAc beta 1,4-galacto	65196	18	15258330	15300179	41850	2862,72	-0,16	8,59E-01
ENSRNOG00000014274	**Trit1**	tRNA isopentenyltransferase 1 (Trit1),	362586	5	144503063	144546565	43503	529,35	0,17	8,59E-01
ENSRNOG00000017791	**Arhgap12**	Rho GTPase activating protein 12 (Ar	307016	17	51975229	52089012	113784	925,01	-0,18	8,59E-01
ENSRNOG00000030572	**Smc5**	structural maintenance of chromosom	293967	1	248263018	248331369	68352	550,37	-0,19	8,59E-01
ENSRNOG00000017720	**Mxd1**	max dimerization protein 1 (Mxd1), mF	362391	4	183021767	183042167	20401	847,69	0,15	8,59E-01
ENSRNOG00000002838	**Pdzd11**	PDZ domain containing 11 (Pdzd11), r	302422	X	71330221	71333253	3033	470,92	-0,10	8,59E-01
ENSRNOG00000007917	**Cd46**	CD46 molecule, complement regulato	29333	13	118334939	118366003	31065	64,64	-0,19	8,59E-01
ENSRNOG00000015989	**Mrpl32**	mitochondrial ribosomal protein L32 (M	291206	17	50802293	50805159	2867	393,16	-0,09	8,59E-01
ENSRNOG00000014863	**Reck**	reversion-inducing-cysteine-rich protei	313488	5	63871878	63938183	66306	330,42	-0,18	8,59E-01
ENSRNOG00000003970	**Tnpo2**	transportin 2 (Tnpo2), mRNA [Source:	304670	19	37127523	37147431	19909	8076,96	0,10	8,59E-01
ENSRNOG00000006077		Sorting nexin-5 [Source:UniProtKB/Sv	296199	3	144812724	144831878	19155	1576,95	-0,13	8,59E-01
ENSRNOG00000018467	**Mitd1**	MIT, microtubule interacting and trans	363219	9	44169745	44181035	11291	188,26	0,13	8,59E-01
ENSRNOG00000020603	**Angptl6**	angiopoietin-like 6 (Angptl6), mRNA [S	298698	8	21951839	21958144	6306	37,64	-0,17	8,59E-01
ENSRNOG00000038347	**Usp21**	ubiquitin specific peptidase 21 (Usp21	688466	13	94281930	94288016	6087	1060,77	0,11	8,59E-01
ENSRNOG00000049484		ATPase, class II, type 9A [Source:MGI Symbol;A		3	171545974	171615105	69132	14200,65	0,12	8,59E-01
ENSRNOG00000010746	**LOC68868**	ribosomal protein L32 (Rpl32), mRNA	28298	4	210999039	211002616	3578	70,87	-0,17	8,59E-01
ENSRNOG00000017263		centrosomal protein 192 [Source:MGI Symbol;Ac		18	62807103	62835427	28325	126,30	-0,16	8,59E-01
ENSRNOG00000022999	**Ppp2r3a**	protein phosphatase 2, regulatory sub	363122	8	108900011	108993750	93740	660,11	-0,16	8,59E-01
ENSRNOG00000025745	**Gpr17**	G protein-coupled receptor 17 (Gpr17)	767613	18	24448653	24449672	1020	536,71	-0,15	8,59E-01
ENSRNOG00000005303	**Tbc1d25**	TBC1 domain family, member 25 (Tbc	302552	X	15847845	15871454	23610	562,22	0,10	8,59E-01
ENSRNOG00000009630	**Hsd17b12**	hydroxysteroid (17-beta) dehydrogena	84013	3	89626177	89749430	123254	2496,69	0,10	8,59E-01
ENSRNOG00000037446	**Pxmp2**	peroxisomal membrane protein 2 (Pxn	29533	12	54187729	54198030	10302	235,16	-0,16	8,59E-01
ENSRNOG00000042254		FMS-like tyrosine kinase 3 ligand [Source:MGI S		1	102191516	102196921	5406	47,25	0,17	8,59E-01
ENSRNOG00000047367		caspase recruitment domain family, member 14		10	108050242	108074296	24055	44,65	-0,18	8,59E-01
ENSRNOG00000001982	**Cblb**	Cbl proto-oncogene B, E3 ubiquitin pr	171136	11	54221084	54383336	162253	706,45	0,11	8,60E-01
ENSRNOG00000024577	**Gamt**	guanidinoacetate N-methyltransferase	25257	7	12484550	12487284	2735	975,01	-0,15	8,60E-01
ENSRNOG00000010777	**Tox**	thymocyte selection-associated high m	362481	5	24669152	24965951	296800	852,23	-0,12	8,60E-01
ENSRNOG00000001199	**Pop5**	processing of precursor 5, ribonuclease P/MRP		12	48932062	48936411	4350	423,18	0,12	8,60E-01
ENSRNOG00000050655	**P4ha1**	prolyl 4-hydroxylase, alpha polypeptid	64475	20	30726604	30776292	49689	917,55	-0,15	8,60E-01
ENSRNOG00000003816	**Tada1**	transcriptional adaptor 1 (Tada1), mRN	360874	13	89462838	89476792	13955	847,43	0,15	8,60E-01
ENSRNOG00000009151	**Mipol1**	Protein Mipol1 [Source:UniProtKB/TrE	1E+08	6	87786212	88024568	238357	51,84	-0,18	8,60E-01

ENSRNOG00000020362	**Zfp574**	zinc finger protein 574 (Zfp574), mRN	308434	1	83212053	83217314	5262	994,77	0,10	8,60E-01
ENSRNOG00000037249	**Lage3**	L antigen family, member 3 (Lage3), m	293863	1	152095511	152096934	1424	3332,40	0,17	8,60E-01
ENSRNOG00000000988	**Xab2**	XPA binding protein 2 (Xab2), mRNA [	245976	12	4320723	4332729	12007	1157,62	0,08	8,60E-01
ENSRNOG00000021795	**Fam227a**	uncharacterized protein LOC300074		300074	7	120839737	120872765	33029	146,77	-0,21	8,60E-01
ENSRNOG00000001059	**Usp42**	ubiquitin specific peptidase 42 (Usp42	288482	12	14904019	14922553	18535	1037,14	-0,11	8,60E-01
ENSRNOG00000003234	**Mgrn1**	mahogunin ring finger 1, E3 ubiquitin p	302938	10	9600449	9648907	48459	4831,43	0,07	8,60E-01
ENSRNOG00000003918	**LOC10091**	solute carrier family 25 (mitochondrial	303676	10	104101681	104114606	12926	221,67	0,11	8,60E-01
ENSRNOG00000009446	**Rxra**	retinoid X receptor alpha (Rxra), mRN	25271	3	11570610	11654513	83904	588,59	-0,16	8,60E-01
ENSRNOG00000043387		carboxypeptidase E [Source:MGI Symbol;Acc:M		16	26785730	26888884	103155	33000,59	-0,15	8,60E-01
ENSRNOG00000009552	**Serinc3**	serine incorporator 3 (Serinc3), mRNA	296350	3	166209489	166228921	19433	3964,08	-0,12	8,61E-01
ENSRNOG00000015085		dystrophia myotonica-protein kinase [S	308405	1	81257166	81267129	9964	737,84	-0,15	8,61E-01
ENSRNOG00000016422	**Klhl36**	kelch-like family member 36 (Klhl36), i	498957	19	63259151	63280344	21194	359,99	-0,14	8,61E-01
ENSRNOG00000010450	**Elovl7**	ELOVL fatty acid elongase 7 (Elovl7),	361895	2	59446064	59459522	13459	24,84	0,19	8,61E-01
ENSRNOG00000011882	**Gab2**	GRB2-associated binding protein 2 (G	84477	1	168294282	168486053	191772	562,06	-0,10	8,61E-01
ENSRNOG00000007314	**Slc26a4**	solute carrier family 26 (anion exchang	29440	6	59479855	59518321	38467	20,20	-0,21	8,61E-01
ENSRNOG00000014654	**LOC69180**	RIKEN cDNA 1810037I17 gene [Sourc	691807	2	246456867	246458626	1760	2334,90	-0,14	8,61E-01
ENSRNOG00000019451	**Smtn**	smoothelin (Smtn), mRNA [Source:Re	289734	14	84443089	84464810	21722	114,89	0,15	8,61E-01
ENSRNOG00000019944	**Fkbp8**	FK506 binding protein 8 (Fkbp8), mRN	290652	16	20501666	20508369	6704	6858,95	0,15	8,61E-01
ENSRNOG00000028178	**Smim4**	LOC361111 (Predicted), isoform CRA_	361111	16	7135209	7138892	3684	206,44	0,18	8,61E-01
ENSRNOG00000037853	**Rarres1**	retinoic acid receptor responder (tazar	310486	2	184000125	184034599	34475	54,97	-0,18	8,61E-01
ENSRNOG00000020590		ADAM metallopeptidase domain 15 (A	57025	2	208071876	208082511	10636	1799,98	-0,13	8,61E-01
ENSRNOG00000047891	**Foxg1**	forkhead box G1 (Foxg1), mRNA [Sou	24370	6	79532855	79535665	2811	6681,02	-0,11	8,61E-01
ENSRNOG00000004496	**Rock2**	Rho-associated coiled-coil containing	25537	6	51952278	52006294	54017	720,25	-0,20	8,61E-01
ENSRNOG00000011349		LON peptidase N-terminal domain and	306505	16	59243274	59272339	29066	845,78	-0,12	8,61E-01
ENSRNOG00000013260	**Calr3**	calreticulin 3 (Calr3), mRNA [Source:F	364529	16	18960142	18985150	25009	64,23	-0,14	8,61E-01
ENSRNOG00000001813		dynamin 1-like (Dnm1l), mRNA [Sourc	114114	11	91885380	91935165	49786	2097,27	-0,17	8,62E-01
ENSRNOG00000002232	**Aff1**	AF4/FMR2 family, member 1 (Aff1), m	305152	14	7216503	7314729	98227	237,62	0,12	8,62E-01
ENSRNOG00000015658		sorbin and SH3 domain containing 1 [S	686098	1	266820767	266928403	107637	2673,05	-0,13	8,62E-01
ENSRNOG00000016093	**Hyal3**	hyaluronoglucosaminidase 3 (Hyal3), i	300993	8	115692772	115698406	5635	576,64	0,10	8,62E-01
ENSRNOG00000017601	**Srd5a1**	steroid-5-alpha-reductase, alpha polyp	24950	1	37717525	37751715	34191	753,06	0,10	8,62E-01
ENSRNOG00000018110		supervillin [Source:MGI Symbol;Acc:M	361256	17	52916420	52984969	68550	438,31	-0,13	8,62E-01
ENSRNOG00000026643	**Chordc1**	cysteine and histidine-rich domain (CH	315447	8	17494921	17518882	23962	693,02	-0,16	8,62E-01
ENSRNOG00000028641	**Brpf3**	bromodomain and PHD finger containi	309647	20	8307922	8337553	29632	525,21	0,10	8,62E-01
ENSRNOG00000043186	**Ppil6**	Protein Ppil6 [Source:UniProtKB/TrEM	685567	20	47907699	47936062	28364	241,86	-0,14	8,62E-01
ENSRNOG00000004382	**Arel1**	apoptosis resistant E3 ubiquitin protein	299197	6	116893001	116917829	24829	2244,56	-0,11	8,62E-01
ENSRNOG00000017396	**March5**	membrane-associated ring finger (C3H	294079	1	263317720	263338103	20384	2053,51	0,13	8,62E-01
ENSRNOG00000004162		6-phosphofructo-2-kinase/fructose-2,6	24640	13	52504015	52529346	25332	975,13	-0,14	8,62E-01
ENSRNOG00000007351	**Ggh**	gamma-glutamyl hydrolase (conjugase	25455	5	38693591	38716694	23104	566,65	-0,15	8,62E-01

ENSRNOG00000009296	**Snapc1**	small nuclear RNA activating complex	314228	6	106302977	106324704	21728	311,09	-0,15	8,62E-01
ENSRNOG00000031579		40S ribosomal protein S24 [Source:U	81776	10	78838574	78838975	402	57,80	0,15	8,62E-01
ENSRNOG00000011494	**Rpl36a**	ribosomal protein L36a (Rpl36a), mRN	81769	X	105292221	105294934	2714	188,37	0,15	8,62E-01
ENSRNOG00000027024	**Rgs16**	regulator of G-protein signaling 16 (Rg	360857	13	76145330	76148774	3445	190,25	0,18	8,62E-01
ENSRNOG00000018752	**Clcf1**	cardiotrophin-like cytokine factor 1 (Cl	365395	1	226338540	226342117	3578	18,97	-0,21	8,62E-01
ENSRNOG00000025040	**Gng10**	guanine nucleotide binding protein (G	114119	5	80325905	80332784	6880	3654,27	-0,09	8,62E-01
ENSRNOG00000004000	**Tefm**	transcription elongation factor, mitoch	287554	10	67134520	67137359	2840	114,10	-0,13	8,62E-01
ENSRNOG00000011320	**Igfbpl1**	insulin-like growth factor binding prote	366366	5	65911664	65927068	15405	452,56	-0,11	8,62E-01
ENSRNOG00000009370	**Tbkbp1**	TBK1 binding protein 1 (Tbkbp1), mRN	266764	10	84861028	84874814	13787	1356,14	0,09	8,62E-01
ENSRNOG00000008861	**RGD1565647**			5	19868622	19871856	3235	36,89	-0,17	8,63E-01
ENSRNOG00000001004	**Ncor2**	nuclear receptor co-repressor 2 (Ncor2	360801	12	38748528	38909933	161406	4249,30	-0,13	8,63E-01
ENSRNOG00000006864	**Scaper**	Protein Scaper [Source:UniProtKB/Tr	117521	8	58788796	59138175	349380	661,50	-0,13	8,63E-01
ENSRNOG00000009538	**Etfdh**	electron-transferring-flavoprotein dehy	295143	2	197702709	197725069	22361	787,50	-0,14	8,63E-01
ENSRNOG00000009771	**Cdh10**	cadherin 10 (Cdh10), mRNA [Source:F	29181	2	89140758	89362817	222060	685,99	-0,11	8,63E-01
ENSRNOG00000011968	**Dnajc6**	DnaJ (Hsp40) homolog, subfamily C, r	313409	5	124221010	124370645	149636	5406,04	0,13	8,63E-01
ENSRNOG00000013624	**Uevld**	Protein Uevld [Source:UniProtKB/TrE	691172	1	104054415	104082541	28127	26,33	0,19	8,63E-01
ENSRNOG00000024632	**Atf6**	activating transcription factor 6 (Atf6),	304962	13	93670582	93854827	184246	1547,62	0,10	8,63E-01
ENSRNOG00000006399	**Synj2bp**	synaptojanin 2 binding protein (Synj2b	64531	6	113374941	113407704	32764	276,50	0,13	8,63E-01
ENSRNOG00000049920	**Psma2**	proteasome (prosome, macropain) sul	29669	17	50789084	50802098	13015	3514,72	0,10	8,63E-01
ENSRNOG00000007443	**Jag1**	jagged 1 (Jag1), mRNA [Source:RefSe	29146	3	136558688	136594097	35410	163,99	-0,12	8,63E-01
ENSRNOG00000047306	**Stap2**	signal transducing adaptor family mem	363334	9	10049873	10055402	5530	31,32	0,20	8,63E-01
ENSRNOG00000004218	**Klhl28**	kelch-like family member 28 (Klhl28), r	299103	6	96177047	96204629	27583	359,92	0,17	8,63E-01
ENSRNOG00000008619	**Agtrap**	angiotensin II receptor-associated pro	298646	5	168546220	168556520	10301	393,62	-0,17	8,64E-01
ENSRNOG00000013149	**Vps33b**	vacuolar protein sorting 33 homolog B	64060	1	143013798	143036705	22908	667,36	0,13	8,64E-01
ENSRNOG00000012641		Swi5-dependent recombination DNA repair prote		1	274916100	274942306	26207	873,45	-0,19	8,64E-01
ENSRNOG00000046258	**B3gnt5**	UDP-GlcNAc:betaGal beta-1,3-N-acet	116740	11	87978737	87990865	12129	81,33	0,16	8,64E-01
ENSRNOG00000018886	**Aaed1**	AhpC/TSA antioxidant enzyme domair	498685	17	1787661	1819338	31678	41,71	-0,17	8,65E-01
ENSRNOG00000002368	**Iars2**	Protein Iars2 [Source:UniProtKB/TrEM	364070	13	107928811	107937935	9125	524,04	0,09	8,65E-01
ENSRNOG00000012392	**Dnajc2**	DnaJ (Hsp40) homolog, subfamily C, r	116456	4	9884085	9909846	25762	846,98	-0,15	8,65E-01
ENSRNOG00000030330	**Pcdh11x**	protocadherin 11 X-linked (Pcdh11x), r	317204	X	92557746	92590961	33216	439,18	-0,16	8,65E-01
ENSRNOG00000046983		adenosine deaminase, tRNA-specific 2 [Source:		1	9354667	9371486	16820	170,12	0,14	8,65E-01
ENSRNOG00000005934		muscular LMNA-interacting protein [Source:MGI		8	84006332	84203965	197634	148,04	0,12	8,65E-01
ENSRNOG00000042944	**Cenpw**	centromere protein W (Cenpw), mRNA	689399	1	31347827	31352722	4896	161,84	0,14	8,65E-01
ENSRNOG00000014419	**Brd7**	bromodomain containing 7 (Brd7), mR	361374	19	30698186	30726814	28629	2173,55	0,09	8,65E-01
ENSRNOG00000008862	**Abcg4**	ATP-binding cassette, subfamily G (W	300664	8	47222818	47238385	15568	1351,92	0,11	8,65E-01
ENSRNOG00000000044	**Tmem175**	transmembrane protein 175 (Tmem17	305623	14	2079289	2095394	16106	879,16	0,11	8,65E-01
ENSRNOG00000002182	**Sept11**	septin 11 (Sept11), mRNA [Source:Re	305227	14	16295536	16378882	83347	3790,49	-0,10	8,65E-01
ENSRNOG00000003772	**Csrp2**	cysteine and glycine-rich protein 2 (Cs	29317	7	53641861	53660495	18635	893,09	-0,18	8,65E-01

ENSRNOG00000014462	**Fnta**	farnesyltransferase, CAAX box, alpha	25318	16	70499606	70519062	19457	1578,21	0,10	8,65E-01
ENSRNOG00000008629	**Secisbp2l**	SECIS binding protein 2-like (Secisbp2	296115	3	124532094	124575388	43295	2518,46	0,09	8,66E-01
ENSRNOG00000026400		predicted gene 4793 [Source:MGI Symbol;Acc:M		13	57462765	57463247	483	24,56	-0,20	8,66E-01
ENSRNOG00000045838		Hermansky-Pudlak syndrome 1 homolog (Huma		1	269464006	269466179	2174	23,62	0,20	8,66E-01
ENSRNOG00000014202	**Snx20**	sorting nexin 20 (Snx20), mRNA [Sour	307742	19	30428393	30436776	8384	31,97	-0,16	8,66E-01
ENSRNOG00000027628	**Elp5**	elongator acetyltransferase complex s	287446	10	56324104	56335657	11554	1574,58	0,11	8,66E-01
ENSRNOG00000017154	**Atp11a**	ATPase, class VI, type 11A (Atp11a), r	306600	16	81476069	81586090	110022	1147,69	-0,11	8,66E-01
ENSRNOG00000020670	**LOC10091**	ATR interacting protein (Atrip), mRNA	301014	8	117149699	117163734	14036	153,03	0,17	8,66E-01
ENSRNOG00000043099	**Ddx21**	DEAD (Asp-Glu-Ala-Asp) box helicase	317399	20	33997712	34017196	19485	585,96	-0,14	8,66E-01
ENSRNOG00000020929	**Lin37**	lin-37 homolog (C. elegans) (Lin37), m	292787	1	90165072	90169004	3933	384,32	0,10	8,66E-01
ENSRNOG00000012940	**Vps41**	vacuolar protein sorting 41 homolog (S	306991	17	46706104	46866332	160229	891,00	0,17	8,66E-01
ENSRNOG00000025302	**Cdca2**	cell division cycle associated 2 (Cdca2	305984	15	47186191	47231339	45149	217,71	0,17	8,66E-01
ENSRNOG00000032936	**Shank3**	SH3 and multiple ankyrin repeat doma	59312	7	130159261	130217900	58640	2345,26	-0,12	8,66E-01
ENSRNOG00000046116		Uncharacterized protein [Source:UniProtKB/TrE		1	64183522	64184837	1316	77,83	0,13	8,66E-01
ENSRNOG00000048391		Uncharacterized protein [Source:UniProtKB/TrE		8	74423476	74618938	195463	251,64	-0,09	8,66E-01
ENSRNOG00000029549	**Eci3**	enoyl-Coenzyme A delta isomerase 3	291076	17	32517375	32539054	21680	51,88	-0,20	8,67E-01
ENSRNOG00000000387	**Slc25a16**	solute carrier family 25 (mitochondrial	361836	20	29124028	29147931	23904	672,13	0,13	8,67E-01
ENSRNOG00000001407	**Tfr2**	transferrin receptor 2 (Tfr2), mRNA [S	288562	12	24194288	24211206	16919	42,92	0,18	8,67E-01
ENSRNOG00000004004	**Nrde2**	Protein Nrde2 [Source:UniProtKB/TrE	314381	6	133365357	133404493	39137	340,87	0,13	8,67E-01
ENSRNOG00000021773	**Bop1**	block of proliferation 1 (Bop1), mRNA	300050	7	117502164	117525973	23810	1765,35	0,12	8,67E-01
ENSRNOG00000003603	**Arhgap44**	Protein Arhgap44 [Source:UniProtKB/	303222	10	51259534	51425613	166080	1607,07	0,10	8,67E-01
ENSRNOG00000008452	**Eid1**	EP300 interacting inhibitor of differenti	499882	3	124462790	124464446	1657	13450,55	-0,12	8,67E-01
ENSRNOG00000021053	**Lsr**	lipolysis stimulated lipoprotein recepto	64355	1	90539046	90554568	15523	172,69	0,15	8,67E-01
ENSRNOG00000001753		F-box protein 45 [Source:MGI Symbol	288042	11	74880642	74892625	11984	347,31	-0,11	8,67E-01
ENSRNOG00000003367		Protein Tmem220; Similar to RIKEN c	287405	10	53322013	53330915	8903	24,64	0,20	8,67E-01
ENSRNOG00000008794	**Ube2e1**	Protein LOC100366017 [Source:UniP	1E+08	15	12188506	12240285	51780	2319,72	-0,13	8,67E-01
ENSRNOG00000011929	**Abcd3**	ATP-binding cassette, subfamily D (AL	25270	2	243374199	243409607	35409	2854,39	-0,10	8,67E-01
ENSRNOG00000020596	**Atp5sl**	ATP5S-like (Atp5sl), mRNA [Source:R	361520	1	83674728	83680655	5928	713,88	0,10	8,67E-01
ENSRNOG00000001889	**Comt**	catechol-O-methyltransferase (Comt),	24267	11	89824622	89829518	4897	2152,32	0,11	8,68E-01
ENSRNOG00000007336	**Churc1**	churchill domain containing 1 (Churc1	299154	6	109213899	109228029	14131	625,25	0,09	8,68E-01
ENSRNOG00000012827	**Mlf1**	myeloid leukemia factor 1 (Mlf1), mRN	310483	2	183898817	183932144	33328	64,59	-0,20	8,68E-01
ENSRNOG00000013577	**Kdelr3**	KDEL (Lys-Asp-Glu-Leu) endoplasmic	315131	7	120748515	120753160	4646	273,13	0,13	8,68E-01
ENSRNOG00000014018		alpha-N-acetylneuraminide alpha-2,8-	25280	4	241274172	241351774	77603	111,49	-0,14	8,68E-01
ENSRNOG00000024846	**Ier5l**	immediate early response 5-like (Ier5l)	499772	3	14389636	14390865	1230	201,03	-0,18	8,68E-01
ENSRNOG00000011585	**Fat3**	FAT atypical cadherin 3 (Fat3), mRNA	191571	8	14504021	14593187	89167	906,35	0,08	8,68E-01
ENSRNOG00000000155	**Cog3**	component of oligomeric golgi comple	361073	15	61462682	61512608	49927	595,51	-0,12	8,68E-01
ENSRNOG00000002207	**Guf1**	GUF1 GTPase homolog (S. cerevisiae	305317	14	62712388	62731766	19379	321,51	-0,16	8,68E-01
ENSRNOG00000004083		zinc finger CCCH type containing 14 [Source:M(		6	131951525	131988793	37269	1496,88	-0,11	8,68E-01

ENSRNOG00000006652	**Ddx1**	DEAD (Asp-Glu-Ala-Asp) box helicase	84474	6	47188699	47219567	30869	2066,05	0,18	8,68E-01
ENSRNOG00000012329	**Tmem66**	transmembrane protein 66 (Tmem66),	290796	16	61400998	61419223	18226	5198,67	0,13	8,68E-01
ENSRNOG00000015430	**Nlgn2**	neuroligin 2 (Nlgn2), mRNA [Source:R	117096	10	56177613	56189966	12354	4826,88	0,10	8,68E-01
ENSRNOG00000017069	**Mark4**	MAP/microtubule affinity-regulating kir	680407	1	81601798	81633585	31788	2793,79	0,12	8,68E-01
ENSRNOG00000020470	**Slc25a24**	solute carrier family 25 (mitochondrial	310791	2	231401404	231438472	37069	165,00	0,16	8,68E-01
ENSRNOG00000021718	**Cspp1**	centrosome and spindle pole associat	362472	5	13569515	13672474	102960	390,76	-0,11	8,68E-01
ENSRNOG00000007174	**Utp11l**	UTP11-like, U3 small nucleolar ribonu	313581	5	146437400	146452289	14890	746,14	0,15	8,68E-01
ENSRNOG00000027550	**Mzf1**	myeloid zinc finger 1 (Mzf1), mRNA [S	361508	1	66333351	66344445	11095	798,95	0,16	8,68E-01
ENSRNOG00000005869				15	15688668	15690224	1557	96,54	-0,11	8,68E-01
ENSRNOG00000008144	**Irf1**	interferon regulatory factor 1 (Irf1), mF	24508	10	38891468	38898469	7002	317,07	-0,17	8,68E-01
ENSRNOG00000014139	**Sclt1**	sodium channel and clathrin linker 1 (S	266809	2	148123775	148271208	147434	136,77	0,18	8,68E-01
ENSRNOG00000018013	**Park2**	parkinson protein 2, E3 ubiquitin prote	56816	1	49909479	50841981	932503	131,94	-0,11	8,68E-01
ENSRNOG00000018183	**Ubr4**	ubiquitin protein ligase E3 component	313658	5	161587918	161695703	107786	8044,64	0,08	8,68E-01
ENSRNOG00000019192		Rho guanine nucleotide exchange factor (GEF)		1	172109215	172164079	54865	1493,76	0,08	8,68E-01
ENSRNOG00000022664		K(lysine) acetyltransferase 7 [Source:MGI Symb		10	82905361	82935808	30448	2334,12	0,09	8,68E-01
ENSRNOG00000028652	**Map10**	microtubule-associated protein 10 (Ma	307948	19	69203373	69206039	2667	89,19	-0,15	8,68E-01
ENSRNOG00000006703	**Zfp398**	zinc finger protein 398 (Zfp398), mRN	500108	4	142188819	142215380	26562	480,74	0,11	8,68E-01
ENSRNOG00000011512	**Ccnt1**	cyclin T1 (Ccnt1), mRNA [Source:RefS	315291	X	114723350	114749903	26554	373,88	0,12	8,68E-01
ENSRNOG00000011654	**Plk4**	polo-like kinase 4 (Plk4), mRNA [Sour	310344	2	147290039	147308583	18545	175,01	0,19	8,68E-01
ENSRNOG00000023484	**Rpap2**	RNA polymerase II associated protein	305120	14	3126078	3205387	79310	404,13	0,10	8,68E-01
ENSRNOG00000050090	**Slc6a17**	solute carrier family 6 (neutral amino a	613226	2	229723069	229753554	30486	5103,57	-0,12	8,68E-01
ENSRNOG00000000041	**Slc26a1**	solute carrier family 26 (anion exchang	64076	14	2046024	2051630	5607	110,11	0,13	8,68E-01
ENSRNOG00000018668	**Glg1**	golgi glycoprotein 1 (Glg1), mRNA [So	29476	19	53811076	53912971	101896	4730,53	-0,11	8,68E-01
ENSRNOG00000012100	**Ssbp1**	single-stranded DNA binding protein 1	54304	4	133421333	133430764	9432	182,86	-0,13	8,68E-01
ENSRNOG00000012630	**Rhoc**	ras homolog family member C (Rhoc),	295342	2	226691039	226697174	6136	2582,96	0,16	8,68E-01
ENSRNOG00000001854	**Tmtc1**	Protein Tmtc1 [Source:UniProtKB/TrE	362465	4	246760019	246960998	200980	66,95	-0,13	8,68E-01
ENSRNOG00000003291	**Creg1**	cellular repressor of E1A-stimulated ge	289185	13	88852202	88864665	12464	870,17	-0,12	8,68E-01
ENSRNOG00000003910	**RGD15643**	Protein RGD1564387 [Source:UniPro	498269	13	101283662	101284531	870	192,97	-0,14	8,68E-01
ENSRNOG00000005193	**Pfas**	phosphoribosylformylglycinamidine sy	287420	10	55316450	55328118	11669	1545,87	-0,10	8,68E-01
ENSRNOG00000025804				3	129652794	129655212	2419	176,28	0,12	8,69E-01
ENSRNOG00000001056	**Snapc2**	small nuclear RNA activating complex	304204	12	4690243	4693261	3019	2001,55	0,15	8,69E-01
ENSRNOG00000005938		general transcription factor 3C polype	313914	6	36383331	36401257	17927	1736,47	0,07	8,69E-01
ENSRNOG00000008869	**Ppp1r9a**	protein phosphatase 1, regulatory sub	84685	4	29885036	30148874	263839	1177,25	0,08	8,69E-01
ENSRNOG00000020350	**Pacs1**	phosphofurin acidic cluster sorting pro	171444	1	227445874	227574749	128876	3297,38	0,09	8,69E-01
ENSRNOG00000021259	**Prnp**	prion protein (Prnp), mRNA [Source:R	24686	3	131010028	131025366	15339	8681,26	0,13	8,69E-01
ENSRNOG00000003687	**Rgs2**	regulator of G-protein signaling 2 (Rgs	84583	13	65830461	65834020	3560	459,12	-0,15	8,70E-01
ENSRNOG00000005411		Protein Aftph [Source:UniProtKB/TrEI	305544	14	104652712	104691189	38478	1516,42	0,13	8,70E-01
ENSRNOG00000011530		family with sequence similarity 188, m	312365	4	150080080	150184008	103929	244,51	-0,11	8,70E-01

ENSRNOG00000036585	**Wdr96**	Protein Wdr96 [Source:UniProtKB/TrE	365476		1	274944315	275031132	86818	81,25	-0,17	8,70E-01
ENSRNOG00000010549	**Tspo**	translocator protein (Tspo), mRNA [So	24230		7	124449359	124459089	9731	65,86	-0,20	8,70E-01
ENSRNOG00000018623	**Rev1**	REV1, polymerase (DNA directed) (Re	316344		9	44410206	44457118	46913	1185,77	-0,11	8,70E-01
ENSRNOG00000047236		phosphodiesterase 4D, cAMP specific [Source:N			2	60524304	60524492	189	52,80	-0,18	8,70E-01
ENSRNOG00000010774	**Scamp1**	secretory carrier membrane protein 1 (	29521		2	43029911	43112171	82261	6366,21	-0,12	8,70E-01
ENSRNOG00000049565		LRRGT00040; Uncharacterized protein [Source			10	61679950	61702356	22407	62,88	0,14	8,70E-01
ENSRNOG00000038483		tumor necrosis factor receptor superfamily, mem			15	55161838	55192095	30258	23,07	-0,17	8,70E-01
ENSRNOG00000003226	**Mprip**	myosin phosphatase Rho interacting p	116504		10	45775202	45890063	114862	6122,75	0,08	8,70E-01
ENSRNOG00000016220		60S ribosomal protein L12 [Source:Ui	499782		3	17346299	17348585	2287	223,50	-0,18	8,70E-01
ENSRNOG00000047244		Uncharacterized protein [Source:UniProtKB/TrE			3	14588194	14597174	8981	191,85	-0,10	8,70E-01
ENSRNOG00000049985	**Gprasp1**	G protein-coupled receptor associated	171407	X		106806370	106813428	7059	4462,90	0,09	8,70E-01
ENSRNOG00000001811	**Fgfr1op2**	FGFR1 oncogene partner 2 (Fgfr1op2	362463		4	245046889	245067828	20940	940,69	0,14	8,71E-01
ENSRNOG00000015643	**Prph**	peripherin (Prph), mRNA [Source:RefS	24688	X		115247359	115251138	3780	143,33	0,17	8,71E-01
ENSRNOG00000003213	**Helz**	helicase with zinc finger (Helz), mRNA	287773		10	95504935	95641663	136729	917,02	-0,11	8,71E-01
ENSRNOG00000003667		Dystrophin [Source:UniProtKB/Swiss-Prot;Acc:F		X		51878333	52510293	631961	20,61	0,20	8,71E-01
ENSRNOG00000007858	**Stil**	Protein Stil [Source:UniProtKB/TrEME	313506		5	137593818	137641722	47905	175,11	0,17	8,71E-01
ENSRNOG00000023065	**Zfp637**	zinc finger protein 637 (Zfp637), mRN.	362425		4	215807099	215812034	4936	1476,47	0,08	8,71E-01
ENSRNOG00000050323	**Srsf1**	serine/arginine-rich splicing factor 1 (S	689890		10	74759412	74765463	6052	7755,50	-0,13	8,71E-01
ENSRNOG00000012379	**Wdr18**	WD repeat domain 18 (Wdr18), mRNA	314617		7	12731050	12738845	7796	1436,46	0,14	8,71E-01
ENSRNOG00000017146		nuclear factor of activated T-cells, cytc	1E+08		18	76340752	76426095	85344	154,81	-0,14	8,71E-01
ENSRNOG00000019108	**Rmi1**	Protein Rmi1 [Source:UniProtKB/TrEI	306734		17	8862959	8864812	1854	313,61	-0,13	8,71E-01
ENSRNOG00000023098		RGD1307897 protein; Uncharacterized protein			6	96348000	96362690	14691	127,13	-0,14	8,71E-01
ENSRNOG00000021324	**RGD15650**	uncharacterized protein C3orf33 homc	499630		2	173869856	173892479	22624	43,35	-0,18	8,72E-01
ENSRNOG00000019428	**Higd1a**	HIG1 hypoxia inducible domain family,	140937		8	129660260	129669650	9391	2141,08	-0,10	8,72E-01
ENSRNOG00000001774		Protein Lrch3 [Source:UniProtKB/TrE	303883		11	74096967	74193090	96124	858,55	0,08	8,72E-01
ENSRNOG00000004421	**Tmem5**	transmembrane protein 5 (Tmem5), m	299841		7	64550353	64562127	11775	768,83	0,09	8,72E-01
ENSRNOG00000005678		laminin B1 [Source:MGI Symbol;Acc:N	298941		6	59204204	59271185	66982	957,74	-0,14	8,72E-01
ENSRNOG00000007997	**Kmt2c**	Protein LOC502710 [Source:UniProtK	502710		4	6271095	6313413	42319	1203,75	0,10	8,72E-01
ENSRNOG00000012209		cytidine and dCMP deaminase domair	361052		15	43686834	43715716	28883	689,05	0,15	8,72E-01
ENSRNOG00000016224	**Tldc1**	Protein RGD1308461 [Source:UniPro	307901		19	63155131	63168351	13221	260,09	0,13	8,72E-01
ENSRNOG00000019460		Uncharacterized protein [Source:UniProtKB/TrE			2	144075179	144075935	757	18,43	0,18	8,72E-01
ENSRNOG00000020554	**Slc50a1**	solute carrier family 50 (sugar efflux tr	295245		2	207998463	208000843	2381	132,65	-0,15	8,72E-01
ENSRNOG00000022419	**Dok7**	docking protein 7 (Dok7), mRNA [Sou	305448		14	81615342	81647361	32020	29,98	-0,16	8,72E-01
ENSRNOG00000047250	**Gmfb**	glia maturation factor, beta (Gmfb), mF	81661		15	27541339	27554439	13101	6319,93	-0,13	8,72E-01
ENSRNOG00000047809		UDP-N-acetyl-alpha-D-galactosamine:polypeptic			16	34815067	34937326	122260	24,72	-0,17	8,72E-01
ENSRNOG00000050562	**Zfp449**	Protein LOC684901; RCG44245 [Sou	684901	X		74213334	74228388	15055	86,76	0,19	8,72E-01
ENSRNOG00000012918	**Mtmr6**	myotubularin related protein 6 (Mtmr6)	305935		15	44371075	44409232	38158	2488,06	0,12	8,72E-01
ENSRNOG00000008374	**Nipsnap1**	nipsnap homolog 1 (C. elegans) (Nips	360971		14	85791355	85815289	23935	3677,08	0,12	8,72E-01

ENSRNOG00000013476		mannosidase, beta A, lysosomal (Man	310864		2	259210951	259317116	106166	124,08	-0,18	8,72E-01
ENSRNOG00000014951	**Mesp1**	mesoderm posterior 1 homolog (mous	308766		1	142493452	142494970	1519	17,62	0,19	8,72E-01
ENSRNOG00000010099	**Asb8**	ankyrin repeat and SOCS box-contain	315287		7	138859490	138867223	7734	1802,98	0,11	8,73E-01
ENSRNOG00000003062	**LOC67996(**	kelch domain containing 2 (Klhdc2), m	679960	X		73290437	73292013	1577	67,25	0,12	8,73E-01
ENSRNOG00000012045	**Bai3**	brain-specific angiogenesis inhibitor 3	301309		9	30096276	30829729	733454	2804,08	-0,12	8,73E-01
ENSRNOG00000020353	**Sh3pxd2a**	SH3 and PX domains 2A (Sh3pxd2a),	309460		1	274477469	274636784	159316	547,02	0,11	8,73E-01
ENSRNOG00000027724	**Plekhf1**	pleckstrin homology domain containin	308543		1	95700788	95701627	840	38,17	-0,19	8,73E-01
ENSRNOG00000008872	**Tmem68**	transmembrane protein 68 (Tmem68),	312946		5	21163802	21194571	30770	1056,78	0,14	8,73E-01
ENSRNOG00000002393	**Eprs**	glutamyl-prolyl-tRNA synthetase (Eprs	289352		13	107973816	108044104	70289	4767,35	-0,15	8,73E-01
ENSRNOG00000005144	**Tmem18**	transmembrane protein 18 (Tmem18),	362722		6	58209471	58215201	5731	465,31	0,13	8,73E-01
ENSRNOG00000016399	**RGD13591**	similar to RIKEN cDNA 2310011J03 (F	299612		7	12426182	12428308	2127	576,56	0,13	8,73E-01
ENSRNOG00000020559	**Cdkn1c**	cyclin-dependent kinase inhibitor 1C ((	246060		1	223521111	223523972	2862	438,31	-0,17	8,73E-01
ENSRNOG00000002860	**Ccdc181**	coiled-coil domain containing 181 (Ccc	360867		13	87458873	87472530	13658	665,59	0,17	8,74E-01
ENSRNOG00000047002	**Fstl5**	follistatin-like 5 (Fstl5), mRNA [Source	365823		2	194400119	194576586	176468	333,88	-0,13	8,74E-01
ENSRNOG00000000418	**Zbtb12**	zinc finger and BTB domain containing	309613		20	6652087	6654932	2846	897,53	-0,09	8,74E-01
ENSRNOG00000008237		unc-13 homolog B (C. elegans) (Unc1	64830		5	63075168	63240229	165062	631,40	0,09	8,74E-01
ENSRNOG00000010482	**Impa1**	Inositol (myo)-1(or 4)-monophosphata	83523		2	113430723	113451415	20693	2093,03	0,16	8,74E-01
ENSRNOG00000023951		TBC1 domain family, member 30 [Sou	299824		7	63585586	63631131	45546	780,86	-0,13	8,74E-01
ENSRNOG00000049932		Uncharacterized protein [Source:UniProtKB/TrE		X		53236352	53462545	226194	23,50	-0,20	8,74E-01
ENSRNOG00000008362	**Zfp775**	zinc finger protein 775 (Zfp775), mRN/	312309		4	142904069	142922229	18161	501,25	0,09	8,74E-01
ENSRNOG00000013234		coiled-coil domain-containing protein 1	363213		9	37462255	37466909	4655	1336,99	0,11	8,74E-01
ENSRNOG00000015582	**Me2**	malic enzyme 2, NAD(+)-dependent, r	307270		18	68882367	68927780	45414	699,21	-0,12	8,74E-01
ENSRNOG00000020357	**Msto1**	misato homolog 1 (Drosophila) (Msto1	295237		2	207611385	207615656	4272	863,28	0,10	8,74E-01
ENSRNOG00000027728		inhibitor of Bruton agammaglobulinemia tyrosine			8	92839911	92905660	65750	882,93	-0,09	8,74E-01
ENSRNOG00000047257	**Tmem185b**	transmembrane protein 185B (Tmem1	304731		13	40669220	40672564	3345	769,19	0,11	8,74E-01
ENSRNOG00000019968	**Trim8**	tripartite motif-containing 8 (Trim8), ml	688785		1	273686719	273701616	14898	3379,04	0,10	8,74E-01
ENSRNOG00000036876	**RGD13089**	LOC362678 (RGD1308923), mRNA [S	362678		5	176107841	176114290	6450	252,31	0,13	8,74E-01
ENSRNOG00000050298	**Rfng**	RFNG O-fucosylpeptide 3-beta-N-ace	60433		10	109555217	109558316	3100	1628,59	0,11	8,74E-01
ENSRNOG00000022844	**Pdzrn4**	PDZ domain containing RING finger 4	315250		7	133539221	133687546	148326	303,82	-0,15	8,74E-01
ENSRNOG00000012842	**Rnf111**	ring finger protein 111 (Rnf111), mRNA	300813		8	76142477	76218329	75853	1489,60	0,10	8,74E-01
ENSRNOG00000021615	**RGD13071**	similar to CG18661-PA (RGD1307155	302998		10	15054533	15065356	10824	196,22	0,10	8,74E-01
ENSRNOG00000033787	**Adamtsl5**	ADAMTS-like 5 (Adamtsl5), mRNA [So	314626		7	12401337	12404316	2980	20,24	-0,20	8,74E-01
ENSRNOG00000045638					15	90890439	90890522	84	23,31	0,16	8,74E-01
ENSRNOG00000024149	**Prr18**	proline rich 18 (Prr18), mRNA [Source	361481		1	54240541	54242506	1966	371,90	0,12	8,74E-01
ENSRNOG00000040297	**Ttc37**	Protein Ttc37 [Source:UniProtKB/TrE	294595		2	2906498	2990393	83896	360,37	0,17	8,74E-01
ENSRNOG00000042939		Protein Nrip2 [Source:UniProtKB/TrEl	689619		4	226926042	226942929	16888	52,20	-0,18	8,74E-01
ENSRNOG00000005203	**LOC10090!**	SEC61, gamma subunit (Sec61g), mR	689134		14	99067709	99074084	6376	1278,89	0,16	8,74E-01
ENSRNOG00000021900		zinc finger protein 873 [Source:MGI Symbol;Acc			7	10940588	10943347	2760	38,49	-0,19	8,74E-01

ENSRNOG00000043223	**Zfp949**	zinc finger protein 949 (Zfp949), mRN	1E+08	8	95849787	95874469	24683	141,48	-0,14	8,75E-01
ENSRNOG00000027860		gametogenetin binding protein 2 (Ggn	360584	10	72063589	72095167	31579	1051,87	-0,14	8,75E-01
ENSRNOG00000003237	**Tp53bp2**	Protein Tp53bp2 [Source:UniProtKB/T	305025	13	105773785	105809219	35435	1281,81	-0,10	8,75E-01
ENSRNOG00000006206	**Tmem106b**	transmembrane protein 106B (Tmem1	312132	4	39352537	39369348	16812	1069,22	-0,11	8,75E-01
ENSRNOG00000015122	**Tpd52l2**	tumor protein D52-like 2 (Tpd52l2), ml	296480	3	180699955	180718702	18748	1448,71	0,08	8,75E-01
ENSRNOG00000020626	**Snrnp25**	Protein Snrnp25; Similar to chromosor	287170	10	15581365	15584287	2923	526,43	0,14	8,75E-01
ENSRNOG00000023238	**Dgkd**	Protein Dgkd [Source:UniProtKB/TrEI	368088	9	94730643	94790627	59985	3995,89	-0,10	8,75E-01
ENSRNOG00000028129	**Fktn**	fukutin (Fktn), mRNA [Source:RefSeq	362520	5	74697863	74751515	53653	898,99	0,11	8,75E-01
ENSRNOG00000043007	**Rcn3**	reticulocalbin 3, EF-hand calcium bind	494125	1	102141920	102151199	9280	203,96	0,17	8,75E-01
ENSRNOG00000043386	**Commd2**	COMM domain containing 2 (Commd2	688478	2	167183080	167187326	4247	994,25	0,08	8,75E-01
ENSRNOG00000043345	**Rnf168**	ring finger protein 168, E3 ubiquitin pr	690043	11	74805908	74827533	21626	476,17	0,11	8,75E-01
ENSRNOG00000004806	**Strn**	striatin, calmodulin binding protein (St	29149	6	1228267	1309977	81711	1166,86	0,11	8,75E-01
ENSRNOG00000009176	**Mkrn2**	makorin, ring finger protein, 2 (Mkrn2)	297525	4	210801433	210819192	17760	1224,81	-0,11	8,75E-01
ENSRNOG00000010794	**Dennd3**	Protein Dennd3 [Source:UniProtKB/T	315055	7	114658386	114735074	76689	144,89	-0,13	8,75E-01
ENSRNOG00000016611	**LOC36785**	Harvey rat sarcoma virus oncogene (H	293621	1	221095946	221099225	3280	1195,51	0,13	8,75E-01
ENSRNOG00000024454	**Ccdc149**	Protein LOC688939 [Source:UniProtK	688939	14	60971605	61064616	93012	293,81	-0,12	8,75E-01
ENSRNOG00000048315	**Eif2ak2**	eukaryotic translation initiation factor 2	54287	6	1420771	1444923	24153	229,90	0,20	8,75E-01
ENSRNOG00000048580	**Trip6**	thyroid hormone receptor interactor 6	686323	12	24466257	24470664	4408	579,46	0,15	8,75E-01
ENSRNOG00000017201	**Cd2bp2**	Cd2 (cytoplasmic tail) binding protein 2	293505	1	205629178	205632675	3498	3592,10	0,10	8,75E-01
ENSRNOG00000029529		Uncharacterized protein [Source:UniProtKB/TrE		11	87050350	87057691	7342	137,01	0,16	8,75E-01
ENSRNOG00000008156	**Tgs1**	trimethylguanosine synthase 1 (Tgs1),	312947	5	21194620	21228145	33526	519,82	0,13	8,75E-01
ENSRNOG00000000312	**Fig4**	FIG4 homolog, SAC1 lipid phosphatas	309855	20	47625556	47747494	121939	1134,46	0,08	8,76E-01
ENSRNOG00000001239	**Ddt**	D-dopachrome tautomerase (Ddt), mF	29318	20	16016818	16019307	2490	1850,25	0,13	8,76E-01
ENSRNOG00000002100	**LOC10091**	signal recognition particle 72 (Srp72),	498351	14	33237745	33351416	113672	490,93	-0,14	8,76E-01
ENSRNOG00000002251	**Sec31a**	SEC31 homolog A (S. cerevisiae) (Sec	93646	14	10801889	10856886	54998	2798,32	0,11	8,76E-01
ENSRNOG00000002458	**Cox11**	cytochrome c oxidase assembly homolog 11 (ye		10	77969326	78029697	60372	308,18	0,13	8,76E-01
ENSRNOG00000002642	**Ptges3**	prostaglandin E synthase 3 (cytosolic)	362809	7	2459082	2476400	17319	3189,81	0,10	8,76E-01
ENSRNOG00000003019	**Ubn1**	ubinuclein 1 (Ubn1), mRNA [Source:R	302935	10	9457239	9492668	35430	1753,80	-0,08	8,76E-01
ENSRNOG00000004474		Kelch domain-containing protein 2 [S	299113	6	101054620	101068438	13819	2373,47	0,10	8,76E-01
ENSRNOG00000004908	**Smc6**	structural maintenance of chromosom	313961	6	46691346	46744578	53233	586,14	0,18	8,76E-01
ENSRNOG00000005015	**Rabep1**	rabaptin, RAB GTPase binding effecto	54190	10	57207873	57324847	116975	2096,51	-0,14	8,76E-01
ENSRNOG00000005531	**Tyro3**	TYRO3 protein tyrosine kinase (Tyro3	25232	3	118146800	118165787	18988	2494,48	-0,11	8,76E-01
ENSRNOG00000007360		regulatory factor X, 4 (influences HLA class II ex		7	25940667	26037662	96996	1059,47	-0,15	8,76E-01
ENSRNOG00000008857		Protein Adamts10 [Source:UniProtKB/TrEMBL;/		7	18586778	18603292	16515	158,32	-0,12	8,76E-01
ENSRNOG00000009402	**Rabgap1**	RAB GTPase activating protein 1 (Rab	311911	3	26937207	27053941	116735	2911,35	0,10	8,76E-01
ENSRNOG00000010775	**Arrdc4**	arrestin domain containing 4 (Arrdc4),	293019	1	130820199	130829248	9050	600,50	0,15	8,76E-01
ENSRNOG00000011105		Protein Arl15 [Source:UniProtKB/TrEI	689079	2	64824710	65081235	256526	219,60	0,13	8,76E-01
ENSRNOG00000011519	**Orc5**	origin recognition complex, subunit 5 (	362304	4	9162486	9226334	63849	397,25	-0,14	8,76E-01

ENSRNOG00000011893	**LOC10091**	ribosomal protein S3a (Rps3a), mRNA	29288	2	204831067	204835622	4556	455,71	-0,17	8,76E-01
ENSRNOG00000012651	**Cwc22**	Protein Cwc22 [Source:UniProtKB/Trl	362153	3	71298090	71346652	48563	137,52	-0,17	8,76E-01
ENSRNOG00000013734	**Dnai1**	dynein, axonemal, intermediate chain	500442	5	62474510	62544534	70025	50,29	-0,16	8,76E-01
ENSRNOG00000013949	**Idh2**	isocitrate dehydrogenase 2 (NADP+),	361596	1	142830044	142849360	19317	1897,73	-0,13	8,76E-01
ENSRNOG00000014341	**Btbd10**	BTB (POZ) domain containing 10 (Btb	308890	1	185106509	185164263	57755	1449,90	0,10	8,76E-01
ENSRNOG00000015546	**MGC11612**	similar to RIKEN cDNA 2700062C07 (	498830	18	16197063	16204477	7415	275,80	0,13	8,76E-01
ENSRNOG00000016200	**Lzic**	leucine zipper and CTNNBIP1 domain	366507	5	170076691	170088575	11885	1924,40	0,12	8,76E-01
ENSRNOG00000016250	**Ammecr1l**	AMME chromosomal region gene 1-lik	307526	18	24257857	24280774	22918	1545,34	-0,10	8,76E-01
ENSRNOG00000016459	**LOC10091**	eukaryotic translation initiation factor 3	691947	3	120516661	120538895	22235	684,80	-0,14	8,76E-01
ENSRNOG00000016838	**Pla2g5**	phospholipase A2, group V (Pla2g5), r	29354	5	160988969	161010285	21317	159,77	-0,16	8,76E-01
ENSRNOG00000018105	**Thtpa**	thiamine triphosphatase (Thtpa), mRN	305889	15	37642563	37646200	3638	2332,62	0,09	8,76E-01
ENSRNOG00000018384	**Adam12**	Protein Adam12; RCG47941, isoform	679837	1	212904287	213226787	322501	358,81	-0,13	8,76E-01
ENSRNOG00000018631	**Enkur**	enkurin, TRPC channel interacting pro	291354	17	89759584	89782791	23208	180,89	-0,17	8,76E-01
ENSRNOG00000019203	**Eya2**	eyes absent homolog 2 (Drosophila) (l	156826	3	168537541	168647321	109781	62,66	-0,17	8,76E-01
ENSRNOG00000019701	**LOC69034**	RIKEN cDNA 1810009A15 gene [Sour	690349	1	232022942	232024908	1967	450,33	-0,13	8,76E-01
ENSRNOG00000021231	**Lzts3**	leucine zipper, putative tumor suppres	280670	3	129673988	129677772	3785	965,54	0,12	8,76E-01
ENSRNOG00000023008	**Fam131c**	Protein Fam131c [Source:UniProtKB/	690880	5	163642831	163647134	4304	37,79	-0,18	8,76E-01
ENSRNOG00000024272	**Ino80d**	INO80 complex subunit D (Ino80d), m	316440	9	69639480	69677228	37749	232,59	-0,10	8,76E-01
ENSRNOG00000026408	**Rnf169**	Protein Rnf169 [Source:UniProtKB/Tr	685009	1	171124367	171179920	55554	230,33	-0,10	8,76E-01
ENSRNOG00000027891	**Dhrs11**	dehydrogenase/reductase (SDR family	360583	10	72050901	72060234	9334	265,06	-0,14	8,76E-01
ENSRNOG00000032813	**Mdc1**	mediator of DNA damage checkpoint 1	309595	20	5502121	5516934	14814	858,35	0,09	8,76E-01
ENSRNOG00000033719	**Pcdhb12**	protocadherin beta 12 (Pcdhb12), mR	25133	18	30216588	30219606	3019	136,67	-0,11	8,76E-01
ENSRNOG00000036660	**Fn3krp**	fructosamine-3-kinase-related protein	303755	10	110200529	110209224	8696	1284,75	-0,10	8,76E-01
ENSRNOG00000037274	**L1cam**	L1 cell adhesion molecule (L1cam), m	50687	1	152659413	152676120	16708	10631,37	0,13	8,76E-01
ENSRNOG00000045542				5	156466664	156466754	91	106,96	-0,12	8,76E-01
ENSRNOG00000048136	**LOC69025**	Protein LOC690251 [Source:UniProtK	303874	11	75965739	75989510	23772	19,24	-0,18	8,76E-01
ENSRNOG00000050131		heterogeneous nuclear ribonucleoprotein D-like		9	5521601	5522507	907	95,55	0,13	8,76E-01
ENSRNOG00000050553		ATPase, H+ transporting, lysosomal V1 subunit		6	52307213	52349242	42030	18,96	-0,17	8,76E-01
ENSRNOG00000043171	**Hdhd2**	haloacid dehalogenase-like hydrolase	361351	18	72904753	72955270	50518	1078,64	0,10	8,76E-01
ENSRNOG00000014191	**Zfp395**	zinc finger protein 395 (Zfp395), mRN	305972	15	52531127	52542971	11845	189,39	-0,12	8,76E-01
ENSRNOG00000014973	**Tekt1**	tektin 1 (Tekt1), mRNA [Source:RefSe	85270	10	58636236	58663145	26910	125,76	0,12	8,76E-01
ENSRNOG00000019246		proteasome activator complex subunit	29614	15	38155752	38160046	4295	134,89	0,13	8,76E-01
ENSRNOG00000007430	**Slx4ip**	SLX4 interacting protein (Slx4ip), mRN	499895	3	136488328	136545079	56752	91,80	-0,13	8,76E-01
ENSRNOG00000009832	**Slc39a14**	solute carrier family 39 (zinc transport	306009	15	55707607	55752619	45013	630,58	0,12	8,76E-01
ENSRNOG00000012323	**Cherp**	calcium homeostasis endoplasmic reti	290614	16	18931534	18944588	13055	2513,53	0,08	8,76E-01
ENSRNOG00000018429		Protein Zscan12 [Source:UniProtKB/T	266716	17	58187516	58188211	696	67,33	0,16	8,76E-01
ENSRNOG00000020093		40S ribosomal protein S12 [Source:U	1E+08	19	49487602	49488236	635	31,95	0,16	8,76E-01
ENSRNOG00000006919	**Clvs1**	clavesin 1 (Clvs1), mRNA [Source:Ref	366311	5	27115709	27312485	196777	711,27	0,09	8,76E-01

ENSRNOG00000008316	**Vps39**	vacuolar protein sorting 39 homolog (S	362199	3	118634996	118673025	38030	2515,25	-0,10	8,76E-01
ENSRNOG00000008580	**Nbn**	nibrin (Nbn), mRNA [Source:RefSeq m	85482	5	34300932	34335368	34437	296,85	-0,18	8,76E-01
ENSRNOG00000012232	**Saa4**	Hermansky-Pudlak syndrome 5 (Hps5	308598	1	103850710	103923493	72784	446,16	0,13	8,76E-01
ENSRNOG00000012906	**Bcas1**	breast carcinoma amplified sequence	246755	3	174061542	174136769	75228	2459,97	-0,17	8,76E-01
ENSRNOG00000014098	**Pold2**	polymerase (DNA directed), delta 2, a	289758	14	86802862	86809022	6161	952,75	0,10	8,76E-01
ENSRNOG00000016701	**Gng8**	guanine nucleotide binding protein (G	245986	1	80067667	80069629	1963	21,86	-0,18	8,76E-01
ENSRNOG00000017166	**Mycbp**	Myc binding protein (Mycbp), mRNA [S	1E+08	5	145348772	145354755	5984	205,17	0,12	8,76E-01
ENSRNOG00000017684	**Fbxl22**	F-box and leucine-rich repeat protein 2	363083	8	71865760	71872447	6688	127,08	-0,12	8,76E-01
ENSRNOG00000023366	**Styxl1**	serine/threonine/tyrosine interacting-lik	360792	12	25952012	25984353	32342	65,57	0,15	8,76E-01
ENSRNOG00000027593		NADH dehydrogenase (ubiquinone) fla	64539	13	56353959	56354285	327	275,77	0,17	8,76E-01
ENSRNOG00000048088	**Mest**	mesoderm specific transcript (Mest), n	58827	4	57816009	57821886	5878	1343,80	0,10	8,76E-01
ENSRNOG00000004120	**RGD15658**	similar to RIKEN cDNA 1700045I19 (R	317486	X	32856976	32858174	1199	37,25	-0,15	8,76E-01
ENSRNOG00000008145	**Traf3**	Tnf receptor-associated factor 3 (Traf3	362788	6	144695911	144803845	107935	1385,60	0,07	8,76E-01
ENSRNOG00000001549	**Gabpa**	GA binding protein transcription factor,	363735	11	27889614	27949403	59790	867,64	-0,09	8,76E-01
ENSRNOG00000006358	**Itgb1bp1**	integrin beta 1 binding protein 1 (Itgb1	298914	6	60216649	60231041	14393	358,34	-0,11	8,76E-01
ENSRNOG00000010865	**Vps13b**	vacuolar protein sorting 13 homolog B	315036	7	74283881	74879193	595313	960,58	-0,13	8,76E-01
ENSRNOG00000045913	**Prdm16**	Protein LOC100366024 [Source:UniP	1E+08	5	175138207	175180209	42003	409,94	-0,16	8,76E-01
ENSRNOG00000000068	**Ppp2r5a**	protein phosphatase 2, regulatory sub	312754	13	114599131	114643527	44397	882,91	-0,10	8,76E-01
ENSRNOG00000008427	**Necap2**	NECAP endocytosis associated 2 (Necap2), mR		5	163302214	163314566	12353	1545,37	0,14	8,76E-01
ENSRNOG00000010298		X-box binding protein 1 (Xbp1), transc	289754	14	86423858	86428922	5065	2711,70	0,11	8,76E-01
ENSRNOG00000015984	**Mfap1a**	microfibrillar-associated protein 1A (M	499878	3	119968974	119985554	16581	554,32	0,15	8,76E-01
ENSRNOG00000016121	**Trip4**	thyroid hormone receptor interactor 4 (	315769	8	71051695	71205615	153921	319,19	0,13	8,76E-01
ENSRNOG00000020345	**Slc25a42**	solute carrier family 25, member 42 (S	689414	16	20826789	20835169	8381	923,37	-0,11	8,76E-01
ENSRNOG00000032048	**Zfp462**	Protein Zfp462 [Source:UniProtKB/Tr	362522	5	75910137	75985296	75160	2251,43	-0,12	8,76E-01
ENSRNOG00000039504	**LOC36091**	RIKEN cDNA 5830473C10 gene [Sou	360919	14	18938045	18982052	44008	25,79	0,18	8,76E-01
ENSRNOG00000050214		aminomethyltransferase, mitochondria	306586	8	116421431	116427436	6006	1247,69	0,12	8,76E-01
ENSRNOG00000014961	**Pdpn**	podoplanin (Pdpn), mRNA [Source:Re	54320	5	165646114	165679595	33482	2225,41	-0,16	8,76E-01
ENSRNOG00000002773	**Rgs4**	regulator of G-protein signaling 4 (Rgs	29480	13	92682956	92689247	6292	2086,77	-0,12	8,77E-01
ENSRNOG00000010365	**Syn1**	synapsin I (Syn1), transcript variant 1,	24949	X	2136354	2193641	57288	4801,06	-0,10	8,77E-01
ENSRNOG00000020657	**Shc1**	SHC (Src homology 2 domain containi	85385	2	208159786	208171348	11563	568,66	0,13	8,77E-01
ENSRNOG00000000108	**Aga**	aspartylglucosaminidase (Aga), mRNA	290923	16	40992085	41003942	11858	532,30	-0,14	8,77E-01
ENSRNOG00000001452	**Fzd9**	frizzled family receptor 9 (Fzd9), mRN	266608	12	26471284	26473598	2315	273,89	0,11	8,77E-01
ENSRNOG00000002079		mitogen activated protein kinase 10 (M	25272	14	8053730	8340390	286661	2065,84	0,12	8,77E-01
ENSRNOG00000003876	**Exo1**	exonuclease 1 (Exo1), mRNA [Source	305000	13	98403058	98427400	24343	101,96	0,15	8,77E-01
ENSRNOG00000004049		BAI1-associated protein 2 (Baiap2), m	117542	10	108707318	108785277	77960	733,39	-0,12	8,77E-01
ENSRNOG00000009180	**Xkr7**	XK, Kell blood group complex subunit-	311549	3	154913771	154936396	22626	381,71	0,12	8,77E-01
ENSRNOG00000012062	**Npc2**	Niemann-Pick disease, type C2 (Npc2	286898	6	117060860	117081692	20833	4288,95	0,12	8,77E-01
ENSRNOG00000018107	**Zfand5**	zinc finger, AN1-type domain 5 (Zfand	293960	1	246131910	246141138	9229	4023,42	-0,12	8,77E-01

ENSRNOG00000027264	**Dagla**	diacylglycerol lipase, alpha (Dagla), m	309207	1	233241963	233298558	56596	1734,49	-0,10	8,77E-01
ENSRNOG00000032735	**Srek1**	splicing regulatory glutamine/lysine-ric	56763	2	52939245	52957927	18683	502,58	-0,12	8,77E-01
ENSRNOG00000001006	**Nptx2**	neuronal pentraxin II (Nptx2), mRNA [S	288475	12	14073308	14084217	10910	410,95	-0,16	8,77E-01
ENSRNOG00000008150		uncharacterized protein LOC300836 [	300836	8	81535953	81559190	23238	1030,62	0,15	8,77E-01
ENSRNOG00000011077	**Tjp1**	tight junction protein 1 (Tjp1), mRNA [S	292994	1	127243076	127307192	64117	2985,19	-0,09	8,77E-01
ENSRNOG00000003129	**Zfp496**	zinc finger protein 496 (Zfp496), mRNA	287361	10	45590717	45608593	17877	755,07	0,09	8,77E-01
ENSRNOG00000000505	**LOC10091**	ribosomal protein L10A (Rpl10a), mRN	81729	20	10108670	10111228	2559	221,27	0,13	8,78E-01
ENSRNOG00000001380	**Tpcn1**	two pore segment channel 1 (Tpcn1),	246215	12	43398893	43421373	22481	1213,53	0,09	8,78E-01
ENSRNOG00000004035	**Krr1**	KRR1, small subunit (SSU) processon	314830	7	54780329	54791757	11429	231,86	-0,16	8,78E-01
ENSRNOG00000010332	**Nipsnap3b**	nipsnap homolog 3B (C. elegans) (Nip	313211	5	73997164	74012019	14856	1383,17	0,10	8,78E-01
ENSRNOG00000011709	**Pum1**	pumilio homolog 1 (Drosophila) (Pum1	362609	5	152491280	152616529	125250	3535,93	-0,09	8,78E-01
ENSRNOG00000012816	**Leng1**	leukocyte receptor cluster (LRC) mem	292535	1	63119164	63124096	4933	308,59	0,09	8,78E-01
ENSRNOG00000029235	**Sfmbt2**	Scm-like with four mbt domains 2 (Sfm	307106	17	73413305	73589644	176340	61,16	0,14	8,78E-01
ENSRNOG00000046155	**LOC49867**	RIKEN cDNA 1700102P08 gene [Sour	498675	8	116518983	116523598	4616	81,30	-0,11	8,78E-01
ENSRNOG00000003554	**Piga**	phosphatidylinositol glycan anchor bio	363464	X	32160734	32170419	9686	64,79	-0,16	8,78E-01
ENSRNOG00000003076	**Rbms2**	RNA binding motif, single stranded inte	288771	7	2533526	2567969	34444	334,25	-0,12	8,78E-01
ENSRNOG00000020835	**Hipk4**	homeodomain interacting protein kinas	308449	1	85544935	85555304	10370	61,94	0,15	8,78E-01
ENSRNOG00000026662	**Plekhf2**	pleckstrin homology domain containing	362484	5	28967292	28982656	15365	819,04	-0,10	8,78E-01
ENSRNOG00000047450		LIM domain only 3 [Source:MGI Symb	497798	4	237202548	237256241	53694	1599,97	0,12	8,78E-01
ENSRNOG00000004168	**Slc35e4**	solute carrier family 35, member E4 (S	266687	14	84847697	84854560	6864	1288,63	-0,12	8,78E-01
ENSRNOG00000015346		obscurin-like 1 [Source:MGI Symbol;Acc:MGI:21		9	82450335	82468812	18478	351,09	0,10	8,78E-01
ENSRNOG00000002094	**Atg3**	autophagy related 3 (Atg3), mRNA [So	171415	11	64687322	64715848	28527	1302,07	0,11	8,78E-01
ENSRNOG00000007014		connector enhancer of kinase suppres	59322	X	40020771	40261531	240761	1462,24	-0,12	8,78E-01
ENSRNOG00000049378		DENN/MADD domain containing 5B [S	1E+08	4	247608223	247671190	62968	733,77	-0,09	8,78E-01
ENSRNOG00000024543	**RGD15599**	Protein RGD1559971 [Source:UniPro	499462	20	46719966	46723072	3107	59,97	0,15	8,79E-01
ENSRNOG00000009274	**Fut11**	fucosyltransferase 11 (alpha (1,3) fucc	286971	15	8132712	8136310	3599	356,41	0,11	8,79E-01
ENSRNOG00000010265	**Ada**	adenosine deaminase (Ada), mRNA [S	24165	3	166306003	166330118	24116	54,51	-0,17	8,79E-01
ENSRNOG00000017598		Protein RGD1565247 [Source:UniPro	302987	10	14362585	14410684	48100	1517,85	0,14	8,79E-01
ENSRNOG00000004621	**Rtn4**	reticulon 4 (Rtn4), mRNA [Source:Ref	83765	14	113792443	113839935	47493	21448,69	-0,08	8,79E-01
ENSRNOG00000017581	**RGD13062**	similar to 4833420G17Rik protein (RG	310377	2	70696812	70718962	22151	184,16	0,15	8,79E-01
ENSRNOG00000040214		RIKEN cDNA 5031425E22 gene [Source:MGI Sy		4	8262324	8262788	465	68,04	0,15	8,79E-01
ENSRNOG00000008989	**Cryl1**	crystallin, lambda 1 (Cryl1), mRNA [So	290277	15	41389716	41509443	119728	227,28	-0,14	8,79E-01
ENSRNOG00000011899	**Tmem150a**	transmembrane protein 150A (Tmem1	245966	4	164989421	164992889	3469	766,11	-0,16	8,79E-01
ENSRNOG00000018483	**Smad1**	SMAD family member 1 (Smad1), mR	25671	19	43085029	43145286	60258	1583,01	0,09	8,79E-01
ENSRNOG00000007560	**Zfp706**	zinc finger protein 706 (Zfp706), mRNA	500855	7	75948335	75948499	165	812,56	-0,09	8,79E-01
ENSRNOG00000011933	**Csnk2a2**	casein kinase 2, alpha prime polypepti	307641	19	9956493	9996387	39895	1274,51	0,09	8,79E-01
ENSRNOG00000014981	**Usp14**	ubiquitin specific peptidase 14 (tRNA-g	291796	18	1221707	1259299	37593	3975,71	0,13	8,79E-01
ENSRNOG00000020307	**RGD13091**	similar to CG5435-PA (RGD1309139),	362020	2	230789966	230802122	12157	20,89	0,16	8,79E-01

ENSRNOG00000038761	**Ptplb**	Protein Ptplb [Source:UniProtKB/TrEM	288058	11	71985569	72080891	95323	418,48	-0,12	8,79E-01
ENSRNOG00000043215	**LOC10090**	retbindin (Rtbdn), mRNA [Source:RefS	304667	19	37041750	37048817	7068	34,69	0,18	8,79E-01
ENSRNOG00000002314	**Marf1**	meiosis arrest female 1 (Marf1), mRN	170946	10	28243	72905	44663	60,60	-0,12	8,79E-01
ENSRNOG00000013468	**Fam213b**	family with sequence similarity 213, m	362676	5	175760762	175763365	2604	635,16	0,13	8,79E-01
ENSRNOG00000019708	**Ctsf**	cathepsin F (Ctsf), mRNA [Source:Ref	361704	1	227045196	227050974	5779	1931,69	0,15	8,79E-01
ENSRNOG00000022499	**Sgol1**	Protein Sgol1 [Source:UniProtKB/TrE	363174	9	3460288	3473013	12726	78,88	0,16	8,79E-01
ENSRNOG00000042773		neuron navigator 1 [Source:MGI Symbol;Acc:MC		13	57484770	57485465	696	89,19	-0,17	8,79E-01
ENSRNOG00000008919		cAMP-regulated phosphoprotein 21 (A	363153	8	119663718	119785747	122030	1470,22	-0,13	8,80E-01
ENSRNOG00000010452	**LOC69067**	cytochrome c, somatic (Cycs), mRNA	25309	4	145002164	145004305	2142	228,57	-0,14	8,80E-01
ENSRNOG00000002450	**Rrp15**	ribosomal RNA processing 15 homolo	360895	13	109807390	109830733	23344	410,88	0,15	8,80E-01
ENSRNOG00000003403	**Slc35f5**	solute carrier family 35, member F5 (S	288993	13	46994593	47034006	39414	1013,94	0,16	8,80E-01
ENSRNOG00000006670	**Rai2**	retinoic acid induced 2 (Rai2), mRNA		501555	X	35074629	35135655	61027	242,45	-0,10	8,80E-01
ENSRNOG00000007412	**Dok1**	docking protein 1 (Dok1), mRNA [Sou	312477	4	178549317	178551776	2460	48,59	0,17	8,80E-01
ENSRNOG00000007645	**Kcnj9**	potassium inwardly-rectifying channel,	116560	13	95224238	95231331	7094	326,38	-0,12	8,80E-01
ENSRNOG00000014504	**Il1r1**	interleukin 1 receptor, type I (Il1r1), mF	25663	9	46681412	46720622	39211	90,31	-0,15	8,80E-01
ENSRNOG00000026502	**Dscc1**	Protein Dscc1 [Source:UniProtKB/TrE	299933	7	95399973	95415357	15385	107,64	0,15	8,80E-01
ENSRNOG00000026528	**Mrps33**	mitochondrial ribosomal protein S33 (I	296995	4	67414413	67417850	3438	1063,24	-0,08	8,80E-01
ENSRNOG00000028636	**Xkr5**	XK, Kell blood group complex subunit-	497083	16	75349398	75367303	17906	81,19	0,11	8,80E-01
ENSRNOG00000043219	**Fbn2**	fibrillin 2 (Fbn2), mRNA [Source:RefSe	689008	18	52284517	52414950	130434	303,45	0,13	8,80E-01
ENSRNOG00000027234	**Zfp367**	zinc finger protein 367 (Zfp367), mRN	306695	17	1642451	1661577	19127	283,97	-0,13	8,80E-01
ENSRNOG00000015215	**Cript**	cysteine-rich PDZ-binding protein (Cri	56725	6	20582635	20590644	8010	1285,60	-0,10	8,80E-01
ENSRNOG00000006143	**Ngly1**	N-glycanase 1 (Ngly1), mRNA [Source	361014	15	14450836	14501356	50521	542,05	-0,11	8,80E-01
ENSRNOG00000009389	**Ripk2**	receptor-interacting serine-threonine k	362491	5	34512875	34543807	30933	226,21	0,12	8,80E-01
ENSRNOG00000014212		methyltransferase like 21A [Source:MGI Symbol		9	71262621	71271320	8700	106,22	0,10	8,80E-01
ENSRNOG00000019264	**Ltbr**	lymphotoxin beta receptor (TNFR sup	297604	4	224839883	224846284	6402	724,05	-0,17	8,80E-01
ENSRNOG00000012528	**Zfyve19**	zinc finger, FYVE domain containing 1	499871	3	117564491	117572476	7986	286,51	-0,10	8,80E-01
ENSRNOG00000013673	**Msh3**	mutS homolog 3 (E. coli) (Msh3), mRN	499505	2	40996035	41137564	141530	300,65	-0,14	8,80E-01
ENSRNOG00000015878	**Pif1**	PIF1 5'-to-3' DNA helicase (Pif1), mRN	367645	8	70811353	70818568	7216	38,96	0,16	8,80E-01
ENSRNOG00000021815	**Lig3**	ligase III, DNA, ATP-dependent (Lig3),	303369	10	69765475	69786585	21111	1026,95	0,09	8,80E-01
ENSRNOG00000002734				X	147600093	147602602	2510	250,00	-0,14	8,80E-01
ENSRNOG00000007744	**Ehd3**	EH-domain containing 3 (Ehd3), mRN	192249	6	34923815	34948298	24484	1582,81	0,11	8,80E-01
ENSRNOG00000011213	**Blm**	Protein Blm [Source:UniProtKB/TrEM	308755	1	143198805	143264208	65404	227,88	-0,11	8,80E-01
ENSRNOG00000014029	**Klhl13**	Kelch-like 13 (Drosophila); Protein Klh	313445	X	121716857	121821667	104811	1585,35	-0,16	8,80E-01
ENSRNOG00000021748		LAS1-like (S. cerevisiae) [Source:MGI	296865	X	65923645	65944397	20753	974,83	0,10	8,80E-01
ENSRNOG00000024089	**Fndc3b**	fibronectin type III domain containing 3	294925	2	132830966	133061245	230280	575,19	0,09	8,80E-01
ENSRNOG00000026474	**Cad**	carbamoyl-phosphate synthetase 2, a	24240	6	36472498	36495450	22953	1665,95	-0,08	8,80E-01
ENSRNOG00000029876	**Gucy1a2**	guanylate cyclase 1, soluble, alpha 2 (	66012	8	417940	903205	485266	54,77	-0,18	8,80E-01
ENSRNOG00000043006	**Epm2aip1**	EPM2A (laforin) interacting protein 1 (I	316021	8	118865756	118868983	3228	1584,25	-0,12	8,80E-01

ENSRNOG00000015912	**Ptdss2**	phosphatidylserine synthase 2 (Ptdss2	293620	1	221040330	221067014	26685	2408,89	-0,08	8,80E-01
ENSRNOG00000002141	**Cd200**	Cd200 molecule (Cd200), mRNA [Sou	24560	11	64474114	64501029	26916	7091,20	0,14	8,81E-01
ENSRNOG00000006103	**Tbc1d31**	TBC1 domain family, member 31 (Tbc	299949	7	98385290	98446226	60937	226,15	-0,15	8,81E-01
ENSRNOG00000008320	**Lypla1**	lysophospholipase I (Lypla1), mRNA [S	25514	5	19341836	19370923	29088	1113,18	-0,13	8,81E-01
ENSRNOG00000013368	**Plcl2**	phospholipase C-like 2 (Plcl2), mRNA	301173	9	1740384	1972816	232433	1180,56	0,12	8,81E-01
ENSRNOG00000013790	**Mfsd1**	major facilitator superfamily domain cc	361957	2	184095497	184116407	20911	3148,99	-0,12	8,81E-01
ENSRNOG00000015932	**Plgrkt**	plasminogen receptor, C-terminal lysir	293888	1	254750915	254763185	12271	443,29	0,12	8,81E-01
ENSRNOG00000017116	**Zfp532**	zinc finger protein 532 (Zfp532), mRN	307362	18	60458275	60565220	106946	2382,55	0,09	8,81E-01
ENSRNOG00000049259	**LOC50140**	zinc finger protein 748 [Source:MGI Sy	501406	1	39329955	39347172	17218	110,62	-0,18	8,81E-01
ENSRNOG00000050794	**Pdlim4**	PDZ and LIM domain 4 (Pdlim4), mRN	24915	10	39171368	39185480	14113	1186,15	-0,18	8,81E-01
ENSRNOG00000002724	**Prpsap2**	phosphoribosyl pyrophosphate synthe	117272	10	47801905	47837190	35286	2187,37	0,13	8,81E-01
ENSRNOG00000031171	**Wdr46**	WD repeat domain 46 (Wdr46), mRNA	309628	20	7506474	7514244	7771	860,51	0,10	8,81E-01
ENSRNOG00000019041	**Psme1**	proteasome (prosome, macropain) act	29630	15	38146266	38149140	2875	1522,22	0,13	8,81E-01
ENSRNOG00000037406	**RGD15641**	RGD1564171 (RGD1564171), mRNA	499656	2	208847060	208850038	2979	32,11	-0,16	8,81E-01
ENSRNOG00000007528	**Kcnh7**	potassium voltage-gated channel, sub	170739	3	55334508	55822719	488212	126,17	-0,14	8,81E-01
ENSRNOG00000021468	**Grm8**	glutamate receptor, metabotropic 8 (Grm8), mRN		4	54229072	54397900	168829	34,64	0,15	8,81E-01
ENSRNOG00000001037	**RGD13072**	similar to RIKEN cDNA 1200011I18 (R	298712	15	61855088	61872506	17419	374,13	0,12	8,81E-01
ENSRNOG00000002839	**Slc19a2**	solute carrier family 19 (thiamine trans	289175	13	87436626	87450478	13853	499,58	0,16	8,81E-01
ENSRNOG00000004882	**Capn6**	calpain 6 (Capn6), mRNA [Source:Ref	83685	X	113502312	113527023	24712	38,94	-0,18	8,81E-01
ENSRNOG00000006338	**Lrp6**	low density lipoprotein receptor-relatec	312781	4	232477066	232579512	102447	943,72	-0,08	8,81E-01
ENSRNOG00000006770	**Brd4**	bromodomain containing 4 (Brd4), mR	362844	7	14378187	14457537	79351	4649,40	-0,08	8,81E-01
ENSRNOG00000009449	**Trim35**	tripartite motif-containing 35 (Trim35),	498538	15	48617501	48633962	16462	9269,97	0,09	8,81E-01
ENSRNOG00000010227	**Gpatch2l**	G patch domain containing 2-like (Gpa	314325	6	119400117	119527025	126909	813,65	0,12	8,81E-01
ENSRNOG00000012002	**Iqgap1**	IQ motif containing GTPase activating	361598	1	143476887	143567395	90509	709,25	0,08	8,81E-01
ENSRNOG00000014742	**Ankrd13b**	Protein Ankrd13b [Source:UniProtKB/	360575	10	62372014	62388298	16285	2925,37	0,12	8,81E-01
ENSRNOG00000015843	**Itfg1**	integrin alpha FG-GAP repeat containi	171083	19	33289326	33410841	121516	4557,75	-0,12	8,81E-01
ENSRNOG00000023874	**Tmem62**	Protein Tmem62 [Source:UniProtKB/T	311350	3	119459037	119493418	34382	635,58	0,14	8,81E-01
ENSRNOG00000028677	**LOC36134**	similar to chromosome 18 open readir	361346	18	68106831	68129952	23122	194,89	-0,16	8,81E-01
ENSRNOG00000029610	**Kcnd2**	potassium voltage-gated channel, Sha	65180	4	48100008	48607643	507636	629,97	0,13	8,81E-01
ENSRNOG00000039859	**Dnmt1**	DNA (cytosine-5-)-methyltransferase 1 (Dnmt1),		8	21978830	22024656	45827	3348,86	0,07	8,81E-01
ENSRNOG00000028047	**Mecr**	mitochondrial trans-2-enoyl-CoA reduc	29470	5	153680464	153705789	25326	414,25	-0,12	8,82E-01
ENSRNOG00000005650	**Pgf**	placental growth factor (Pgf), mRNA [S	94203	6	116552660	116563240	10581	21,20	-0,17	8,82E-01
ENSRNOG00000051006		UFM1-specific peptidase 1 [Source:MGI Symbol		12	24486665	24487318	654	156,04	0,13	8,82E-01
ENSRNOG00000008996	**LOC10091**	dihydropyrimidinase-like 5 (Dpysl5), m	65208	6	36756090	36840626	84537	3370,11	0,11	8,82E-01
ENSRNOG00000009536	**Pgp**	phosphoglycolate phosphatase (Pgp),	287115	10	13653091	13655717	2627	1630,32	0,12	8,82E-01
ENSRNOG00000021010	**Arl2**	ADP-ribosylation factor-like 2 (Arl2), m	65142	1	228438709	228451114	12406	1146,29	0,11	8,82E-01
ENSRNOG00000021918	**Cep76**	centrosomal protein 76 (Cep76), mRN	291540	18	62550817	62579728	28912	305,43	-0,11	8,82E-01
ENSRNOG00000027514		NEDD8-conjugating enzyme Ubc12 [S	361509	1	66348960	66351941	2982	3881,92	0,12	8,82E-01

ENSRNOG00000031667				MT	11665	11735	71	101,21	0,15	8,82E-01
ENSRNOG00000016225		FYVE, RhoGEF and PH domain-conta	361223	17	17820436	17855077	34642	506,94	-0,09	8,83E-01
ENSRNOG00000000520	**LOC10091(**	serine/arginine-rich splicing factor 3 (S	361814	20	8534915	8544847	9933	2702,77	-0,09	8,83E-01
ENSRNOG00000003948	**Llgl1**	lethal giant larvae homolog 1 (Drosopl	54265	10	46714014	46727304	13291	4045,61	0,12	8,83E-01
ENSRNOG00000020696	**Pmvk**	phosphomevalonate kinase (Pmvk), m	310645	2	208198108	208207798	9691	1576,04	0,11	8,83E-01
ENSRNOG00000025454		ribonucleoside-diphosphate reductase	299976	7	76865859	76885636	19778	120,70	-0,10	8,83E-01
ENSRNOG00000048004	**Gareml**	GRB2 associated, regulator of MAPK1	362801	6	37442035	37453747	11713	720,03	0,11	8,83E-01
ENSRNOG00000002241	**Hspbap1**	Hspb associated protein 1 (Hspbap1),	171460	11	71232155	71287075	54921	259,63	0,09	8,83E-01
ENSRNOG00000015273	**Ak3**	adenylate kinase 3 (Ak3), mRNA [Sou	26956	1	254383052	254408286	25235	849,59	-0,11	8,83E-01
ENSRNOG00000018137	**Tubgcp2**	tubulin, gamma complex associated p	309098	1	219251912	219272444	20533	1672,23	-0,08	8,83E-01
ENSRNOG00000039241		YTH domain containing 2 [Source:MG	307446	1	189058204	189111184	52981	170,74	-0,15	8,83E-01
ENSRNOG00000045742	**Cyb5d1**	cytochrome b5 domain containing 1 (C	363629	10	55735829	55739764	3936	635,82	0,12	8,83E-01
ENSRNOG00000001350	**Naa25**	N(alpha)-acetyltransferase 25, NatB a	360811	12	42495120	42547489	52370	880,50	-0,09	8,83E-01
ENSRNOG00000000277		tet methylcytosine dioxygenase 1 [Sou	309902	20	29201150	29267359	66210	206,16	-0,17	8,83E-01
ENSRNOG00000016371	**Slc18b1**	Protein Slc18b1 [Source:UniProtKB/T	309570	1	24179175	24220597	41423	310,58	0,14	8,83E-01
ENSRNOG00000022297	**Kcnmb4**	potassium large conductance calcium-	66016	7	59472015	59526626	54612	1228,59	-0,16	8,83E-01
ENSRNOG00000027469	**Pcdhb4**	protocadherin beta 4 (Pcdhb4), mRNA	291655	18	30095466	30098005	2540	99,96	-0,13	8,83E-01
ENSRNOG00000046547	**Rbm24**	RNA binding motif protein 24 (Rbm24)	690139	17	19261277	19269603	8327	146,23	-0,14	8,83E-01
ENSRNOG00000028638	**Il10rb**	interleukin 10 receptor, beta (Il10rb), n	304091	11	35169509	35190491	20983	87,44	-0,14	8,83E-01
ENSRNOG00000011955	**LOC10036(**	60S acidic ribosomal protein P1 [Sour	140661	17	11392283	11393688	1406	35,51	-0,18	8,83E-01
ENSRNOG00000017459	**C1ql3**	complement component 1, q subcomp	680404	17	81939086	81945581	6496	165,88	0,14	8,83E-01
ENSRNOG00000023746	**Tmem39b**	transmembrane protein 39b (Tmem39	362608	5	151617692	151634824	17133	375,88	0,09	8,83E-01
ENSRNOG00000004104	**Zfp167**	zinc finger protein 167 (Zfp167), mRN	363170	8	131088429	131105818	17390	186,59	-0,13	8,83E-01
ENSRNOG00000009290	**Nup35**	nucleoporin 35 (Nup35), mRNA [Sourc	295692	3	74426913	74452803	25891	753,22	0,11	8,83E-01
ENSRNOG00000009889	**Pgm1**	phosphoglucomutase 1 (Pgm1), mRN	24645	5	122648579	122708212	59634	2283,07	-0,13	8,83E-01
ENSRNOG00000010502	**Fbxo8**	F-box protein 8 (Fbxo8), mRNA [Sourc	306436	16	36936326	36981671	45346	562,81	-0,13	8,83E-01
ENSRNOG00000012764	**Agap3**	ArfGAP with GTPase domain, ankyrin	362300	4	7213697	7240039	26343	6309,86	0,11	8,83E-01
ENSRNOG00000013215		dCMP deaminase (Dctd), transcript va	290741	16	46903570	46933233	29664	103,35	-0,10	8,83E-01
ENSRNOG00000013436	**Pde7b**	phosphodiesterase 7B (Pde7b), mRN	140929	1	17438266	17650684	212419	114,02	-0,10	8,83E-01
ENSRNOG00000018576	**Rcan3**	RCAN family member 3 (Rcan3), mRN	362627	5	157542942	157551147	8206	254,49	0,08	8,83E-01
ENSRNOG00000018616	**Cdc42ep5**	CDC42 effector protein (Rho GTPase	361505	1	74828671	74829132	462	97,86	0,16	8,83E-01
ENSRNOG00000024533	**Eogt**	EGF domain-specific O-linked N-acety	494219	4	193983250	194019171	35922	293,67	0,12	8,83E-01
ENSRNOG00000037919	**LOC10091**	Protein Chic1 [Source:UniProtKB/TrE	363484	X	75012969	75050446	37478	41,84	-0,16	8,83E-01
ENSRNOG00000045775	**Rnpepl1**	arginyl aminopeptidase (aminopeptida	684035	9	99743849	99752173	8325	594,96	0,12	8,83E-01
ENSRNOG00000050348		isochorismatase domain containing 2a	684270	1	76040561	76044554	3994	320,87	0,15	8,83E-01
ENSRNOG00000011815		serum/glucocorticoid regulated kinase	29517	1	25652755	25660738	7984	725,25	0,10	8,83E-01
ENSRNOG00000021086	**Dtx4**	deltex homolog 4 (Drosophila) (Dtx4),	293774	1	235895198	235915304	20107	832,80	0,12	8,84E-01
ENSRNOG00000010027	**Atr**	Protein Atr [Source:UniProtKB/TrEMB	685055	8	103124135	103220823	96689	158,06	0,17	8,84E-01

ENSRNOG00000001252	**Chst12**	carbohydrate (chondroitin 4) sulfotrans	304322	12	18193614	18194873	1260	351,16	0,08	8,84E-01
ENSRNOG00000004593		Uncharacterized protein [Source:UniProtKB/TrE		7	64548576	64550004	1429	41,16	-0,13	8,84E-01
ENSRNOG00000002722	**Sec14l1**	SEC14-like 1 (S. cerevisiae) (Sec14l1	360668	10	105718075	105765280	47206	4902,80	-0,08	8,84E-01
ENSRNOG00000003177	**Mat2b**	methionine adenosyltransferase II, bet	683630	10	25686298	25699413	13116	915,02	0,12	8,84E-01
ENSRNOG00000004205	**Pkdcc**	protein kinase domain containing, cytc	313860	6	6384829	6394146	9318	500,02	-0,16	8,84E-01
ENSRNOG00000017545	**Mrs2**	MRS2 magnesium homeostasis factor	79032	17	43932173	43950097	17925	469,75	0,15	8,84E-01
ENSRNOG00000018863	**Abcc10**	ATP-binding cassette, subfamily C (CF	316231	9	15938028	15957940	19913	283,58	0,11	8,84E-01
ENSRNOG00000047185		eukaryotic translation initiation factor 3, subunit I		5	151525930	151532743	6814	1900,96	0,10	8,84E-01
ENSRNOG00000050997	**Ifrd1**	interferon-related developmental regul	29596	6	69720076	69739472	19397	1313,50	0,10	8,84E-01
ENSRNOG00000000246	**Amz2**	archaelysin family metallopeptidase 2	360650	10	97435124	97445659	10536	6180,24	0,11	8,84E-01
ENSRNOG00000005918	**RGD15639**	uncharacterized protein LOC500993 |	500993	8	51422579	51422993	415	51,70	0,17	8,84E-01
ENSRNOG00000016592	**Gnai2**	guanine nucleotide binding protein (G	81664	8	115726576	115746724	20149	16494,79	0,10	8,84E-01
ENSRNOG00000017298	**Cutc**	cutC copper transporter (Cutc), mRNA	361760	1	270956594	270971364	14771	180,52	0,13	8,84E-01
ENSRNOG00000018475	**LOC10091**	eukaryotic translation initiation factor 4	305468	14	84100708	84143194	42487	941,74	-0,07	8,84E-01
ENSRNOG00000023313	**Arhgef19**	Rho guanine nucleotide exchange fac	362648	5	163483734	163494854	11121	81,02	-0,16	8,84E-01
ENSRNOG00000001300	**P2rx4**	purinergic receptor P2X, ligand-gated	29659	12	41196780	41214369	17590	353,47	0,09	8,84E-01
ENSRNOG00000002882		zinc finger, CCHC domain containing 2	304695	13	31126927	31175425	48499	543,70	-0,08	8,84E-01
ENSRNOG00000011561	**Nln**	neurolysin (metallopeptidase M3 famil	117041	2	53216605	53313982	97378	2593,23	-0,09	8,84E-01
ENSRNOG00000013162	**Zcchc12**	zinc finger, CCHC domain containing 1	313436	X	122958362	122961546	3185	4308,03	-0,14	8,84E-01
ENSRNOG00000016361		phospholipase C, delta 4 (Plcd4), mRI	140693	9	81581514	81608512	26999	695,87	-0,16	8,84E-01
ENSRNOG00000012788	**Clns1a**	chloride channel, nucleotide-sensitive,	65160	1	168883053	168903321	20269	1880,89	0,11	8,84E-01
ENSRNOG00000005261	**Fbxl5**	F-box and leucine-rich repeat protein 5	305424	14	71880738	71918708	37971	1010,90	-0,14	8,84E-01
ENSRNOG00000014963		G-protein coupled receptor 56 precurs	260326	19	10403029	10442272	39244	11284,96	0,11	8,84E-01
ENSRNOG00000037620	**Mob3c**	MOB kinase activator 3C (Mob3c), mF	313511	5	138464738	138471205	6468	31,01	-0,15	8,84E-01
ENSRNOG00000012105	**RGD15630**	Protein RGD1563084 [Source:UniPro	315912	3	53611063	53697823	86761	32,85	-0,18	8,85E-01
ENSRNOG00000005028	**Rab5a**	RAB5A, member RAS oncogene famil	64633	14	72908161	72908809	649	544,46	0,11	8,85E-01
ENSRNOG00000005996	**Lhx6**	LIM homeobox 6 (Lhx6), mRNA [Sour	311901	3	20721151	20742464	21314	167,54	0,11	8,85E-01
ENSRNOG00000029572	**Apeh**	acylaminoacyl-peptide hydrolase (Ape	24206	8	116216748	116225863	9116	1487,73	-0,12	8,85E-01
ENSRNOG00000004482	**Ccdc88c**	Protein Ccdc88c [Source:UniProtKB/T	362770	6	134128636	134247012	118377	1018,52	-0,07	8,85E-01
ENSRNOG00000030302	**Csde1**	cold shock domain containing E1, RN/	117180	2	224955792	224983578	27787	275,40	-0,09	8,85E-01
ENSRNOG00000003664	**Brox**	BRO1 domain and CAAX motif contair	305031	13	100886374	100906156	19783	2420,03	0,09	8,85E-01
ENSRNOG00000003795	**Angel2**	angel homolog 2 (Drosophila) (Angel2	305035	13	114107705	114121845	14141	1077,12	-0,14	8,85E-01
ENSRNOG00000005063	**Atl1**	atlastin GTPase 1 (Atl1), mRNA [Sour	362750	6	101678899	101819541	140643	2055,47	-0,10	8,85E-01
ENSRNOG00000014190		histidine triad nucleotide binding prote	246769	1	30852989	30862850	9862	312,62	-0,10	8,85E-01
ENSRNOG00000016143	**Gpr162**	G protein-coupled receptor 162 (Gpr1	362436	4	224392962	224398883	5922	2735,98	-0,08	8,85E-01
ENSRNOG00000022173	**Gatad2a**	GATA zinc finger domain containing 2/	290669	16	21093916	21184110	90195	2153,42	0,11	8,85E-01
ENSRNOG00000029644		C-type lectin domain family 2 member	296985	4	66216161	66232497	16337	869,12	-0,17	8,85E-01
ENSRNOG00000043377		farnesyl diphosphate synthase (Fdps),	83791	2	207805217	207826348	21132	8885,32	0,15	8,85E-01

ENSRNOG00000013505	**Vdac2**	voltage-dependent anion channel 2 (V	83531	15	2615637	2629307	13671	5919,66	0,08	8,85E-01
ENSRNOG00000013548	**Sepw1**	selenoprotein W, 1 (Sepw1), mRNA [S	25545	1	78801628	78804176	2549	6142,21	0,13	8,85E-01
ENSRNOG00000017489	**Gse1**	Protein Gse1 [Source:UniProtKB/TrEI	307913	19	64849291	64873898	24608	1036,57	-0,10	8,85E-01
ENSRNOG00000016032	**Cnnm3**	cyclin M3 (Cnnm3), mRNA [Source:Re	301345	9	42747044	42762392	15349	1464,38	-0,10	8,85E-01
ENSRNOG00000010128		solute carrier family 27 (fatty acid transporter), m		3	125543564	125580197	36634	45,60	0,17	8,85E-01
ENSRNOG00000032740	**LOC69013**	Protein LOC100363048 [Source:UniP	499317	9	61233464	61233703	240	22,35	0,15	8,85E-01
ENSRNOG00000007062	**Rin3**	Ras and Rab interactor 3 (Rin3), mRN	314397	6	135381785	135490155	108371	18,17	-0,17	8,86E-01
ENSRNOG00000005433	**Shq1**	SHQ1, H/ACA ribonucleoprotein asser	297483	4	197520939	197616582	95644	93,18	-0,10	8,86E-01
ENSRNOG00000020244	**RGD13049**	similar to RIKEN cDNA 2310042D19 (	313776	5	177114820	177120113	5294	18,54	-0,16	8,86E-01
ENSRNOG00000032320				MT	9800	9867	68	50,81	0,14	8,86E-01
ENSRNOG00000003694	**Prox1**	prospero homeobox 1 (Prox1), mRNA	305066	13	113004597	113060843	56247	367,62	-0,15	8,86E-01
ENSRNOG00000003428		RUN and FYVE domain containing 3 (	360921	14	20980420	21038332	57913	4119,64	0,11	8,86E-01
ENSRNOG00000004229	**Tac3**	tachykinin 3 (Tac3), mRNA [Source:Re	29191	7	71196645	71203251	6607	27,08	0,18	8,86E-01
ENSRNOG00000009886	**LOC10091**	FK506 binding protein 14 (Fkbp14), m	362366	4	149413605	149428266	14662	249,40	-0,11	8,86E-01
ENSRNOG00000037522				14	108635688	108636078	391	89,40	-0,15	8,86E-01
ENSRNOG00000042152		PDZ domain-containing protein GIPC2	365960	2	275974533	276052941	78409	31,91	0,14	8,86E-01
ENSRNOG00000018119	**Dhx32**	DEAH (Asp-Glu-Ala-His) box polypept	361667	1	212738509	212790818	52310	1853,86	-0,12	8,86E-01
ENSRNOG00000020072	**Pcdhb9**	protocadherin beta 9 (Pcdhb9), mRNA	680047	18	30182142	30185312	3171	271,95	-0,10	8,86E-01
ENSRNOG00000004430	**Azi1**	5-azacytidine induced 1 (Azi1), mRNA	360672	10	108841399	108865764	24366	1431,33	-0,07	8,86E-01
ENSRNOG00000027290	**Pcdhga7**	protocadherin gamma subfamily A, 7 (	291635	18	30558239	30560872	2634	929,79	-0,12	8,87E-01
ENSRNOG00000034075	**Ube2ql1**	ubiquitin-conjugating enzyme E2Q fan	679949	1	37582312	37622672	40361	3707,62	0,09	8,87E-01
ENSRNOG00000000399	**Kpna1**	karyopherin alpha 1 (Kpna1), mRNA [S	288064	11	70740054	70791248	51195	2026,77	-0,12	8,87E-01
ENSRNOG00000039850		phosphatidylinositol N-acetylglucosam	288238	11	38179879	38185851	5973	362,73	-0,10	8,87E-01
ENSRNOG00000023278	**Sec61a2**	Sec61 alpha 2 subunit (S. cerevisiae)	361273	17	78015736	78041913	26178	4057,30	0,14	8,87E-01
ENSRNOG00000031916	**Timeless**	timeless circadian clock (Timeless), m	83508	7	2622164	2646197	24034	420,13	-0,11	8,87E-01
ENSRNOG00000019400	**Dag1**	dystroglycan 1 (dystrophin-associated	114489	8	116336544	116346738	10195	6087,23	-0,10	8,87E-01
ENSRNOG00000001453	**Baz1b**	bromodomain adjacent to zinc finger d	368002	12	26476185	26534154	57970	3729,61	0,07	8,88E-01
ENSRNOG00000002946	**Socs3**	suppressor of cytokine signaling 3 (So	89829	10	106612942	106613619	678	55,32	-0,15	8,88E-01
ENSRNOG00000003782	**Acot9**	acyl-CoA thioesterase 9 (Acot9), mRN	302640	X	43846065	43895730	49666	403,61	-0,13	8,88E-01
ENSRNOG00000003971	**Gosr1**	golgi SNAP receptor complex member	94189	10	63104268	63261223	156956	655,31	0,13	8,88E-01
ENSRNOG00000004048	**Lrrk2**	leucine-rich repeat kinase 2 (Lrrk2), m	300160	7	132531605	132693763	162159	158,36	0,12	8,88E-01
ENSRNOG00000004500	**Myc**	myelocytomatosis oncogene (Myc), m	24577	7	103157452	103162376	4925	1421,79	-0,13	8,88E-01
ENSRNOG00000007106	**Sos1**	Son of sevenless homolog 1 (Drosoph	313845	6	3082456	3159445	76990	2223,66	-0,08	8,88E-01
ENSRNOG00000008741	**Camsap2**	calmodulin regulated spectrin-associa	289400	13	58197543	58276568	79026	4560,74	-0,09	8,88E-01
ENSRNOG00000008806	**Clip4**	CAP-GLY domain containing linker pro	298801	6	33101207	33150094	48888	193,61	-0,13	8,88E-01
ENSRNOG00000013180	**Ercc3**	excision repair cross-complementing r	291703	18	24752960	24783859	30900	1121,38	0,09	8,88E-01
ENSRNOG00000014107	**Omg**	oligodendrocyte-myelin glycoprotein ((	450224	10	64800318	64803052	2735	294,03	-0,14	8,88E-01
ENSRNOG00000015544	**LOC10090**	tubulin, alpha 1C (Tuba1c), mRNA [Sc	300218	2	218679908	218687509	7602	199,71	0,12	8,88E-01

ENSRNOG00000018160	**Zswim5**	zinc finger, SWIM-type containing 5 (Z	313524	5	139532215	139647709	115495	632,37	0,08	8,88E-01
ENSRNOG00000023023	**Trpt1**	tRNA phosphotransferase 1 (Trpt1), m	293704	1	229241774	229246773	5000	90,95	-0,11	8,88E-01
ENSRNOG00000025554	**Zfp445**	zinc finger protein 445 (Zfp445), mRN.	301076	8	131040161	131054065	13905	873,78	-0,16	8,88E-01
ENSRNOG00000027179	**Zdhhc23**	zinc finger, DHHC-type containing 23 (	363783	11	60791711	60807079	15369	275,65	-0,13	8,88E-01
ENSRNOG00000031163	**Nfkbiz**	nuclear factor of kappa light polypeptic	304005	11	50420563	50447282	26720	35,48	-0,17	8,88E-01
ENSRNOG00000004346	**Notch3**	notch 3 (Notch3), mRNA [Source:RefS	56761	7	14294587	14345774	51188	2166,05	-0,15	8,88E-01
ENSRNOG00000008926		Guanine nucleotide-binding protein subunit beta		8	81880465	81889635	9171	1190,88	0,11	8,88E-01
ENSRNOG00000011471	**Fgf9**	fibroblast growth factor 9 (Fgf9), mRN.	25444	15	42176762	42217711	40950	128,36	-0,14	8,88E-01
ENSRNOG00000000308	**Zbtb24**	zinc finger and BTB domain containing	365590	20	47865623	47882126	16504	340,78	-0,10	8,88E-01
ENSRNOG00000017745	**Cog4**	component of oligomeric golgi comple	361407	19	54191116	54224687	33572	1754,24	0,12	8,88E-01
ENSRNOG00000020601	**Hmg20b**	high mobility group 20 B (Hmg20b), m	362825	7	11405510	11410190	4681	352,21	0,14	8,88E-01
ENSRNOG00000001817	**Tm7sf3**	transmembrane 7 superfamily membe	297725	4	245073413	245110560	37148	1076,39	0,09	8,88E-01
ENSRNOG00000002543		THAP domain containing 6 [Source:R	305244	14	17523509	17533559	10051	85,62	0,11	8,88E-01
ENSRNOG00000006199	**Ispd**	isoprenoid synthase domain containin	493574	6	65492817	65769833	277017	124,11	0,12	8,88E-01
ENSRNOG00000011831	**Nudt18**	nudix (nucleoside diphosphate linked i	361068	15	55989072	55991602	2531	215,87	-0,11	8,88E-01
ENSRNOG00000017114		synaptojanin 2 (Synj2), transcript varia	84018	1	48140615	48236028	95414	139,00	0,12	8,88E-01
ENSRNOG00000020501	**Tjp3**	tight junction protein 3 (Tjp3), mRNA [	314640	7	11469366	11481943	12578	60,51	-0,16	8,88E-01
ENSRNOG00000025189	**Mettl18**	methyltransferase like 18 (Mettl18), m	304928	13	87171578	87173701	2124	75,80	0,14	8,88E-01
ENSRNOG00000031544	**Larp1**	Protein Larp1 [Source:UniProtKB/TrE	303158	10	43275068	43293843	18776	2705,29	0,07	8,88E-01
ENSRNOG00000009835	**Tram1l1**	translocation associated membrane pi	310846	2	247895258	247897439	2182	1276,47	0,13	8,88E-01
ENSRNOG00000001303	**Specc1l**	sperm antigen with calponin homology	361828	20	16472453	16577173	104721	1794,86	0,09	8,88E-01
ENSRNOG00000004787		Uncharacterized protein [Source:UniProtKB/TrE		3	102353885	102575106	221222	269,15	0,08	8,88E-01
ENSRNOG00000006727	**Dtd2**	D-tyrosyl-tRNA deacylase 2 (putative)	366619	6	82342355	82348642	6288	316,53	0,09	8,88E-01
ENSRNOG00000007892	**Tram1**	translocation associated membrane pi	312903	5	10062171	10157544	95374	2020,28	-0,10	8,88E-01
ENSRNOG00000016183	**Ipp**	intracisternal A particle-promoted poly	298439	5	139150941	139184255	33315	560,44	-0,10	8,88E-01
ENSRNOG00000016807	**Oat**	ornithine aminotransferase (Oat), mRN	64313	1	211555927	211575708	19782	3980,40	-0,09	8,88E-01
ENSRNOG00000016961	**LOC10091**	ribosomal protein S27 (Rps27), mRNA	94266	2	209004975	209006077	1103	4466,01	0,14	8,88E-01
ENSRNOG00000023923	**Fastkd2**	FAST kinase domains 2 (Fastkd2), mF	301463	9	72102979	72121685	18707	99,01	-0,14	8,88E-01
ENSRNOG00000025459	**Mars**	methionyl-tRNA synthetase (Mars), ml	299851	7	70759159	70776456	17298	3397,55	0,07	8,88E-01
ENSRNOG00000039234	**Zscan21**	zinc finger and SCAN domain containi	304342	12	21321340	21335678	14339	1003,41	0,10	8,88E-01
ENSRNOG00000020962	**Pogz**	pogo transposable element with ZNF (	310658	2	215507130	215534824	27695	2069,43	-0,08	8,89E-01
ENSRNOG00000001531	**Chac2**	ChaC, cation transport regulator homc	360994	14	115040363	115047436	7074	173,35	-0,11	8,89E-01
ENSRNOG00000000895	**Rabgef1**	RAB guanine nucleotide exchange fac	360797	12	31898729	31926568	27840	880,44	0,11	8,89E-01
ENSRNOG00000010574	**Ptpn1**	protein tyrosine phosphatase, non-rec	24697	3	170816920	170866072	49153	1857,31	-0,08	8,89E-01
ENSRNOG00000014201		Mesencephalic astrocyte-derived neur	315989	8	114982002	114985213	3212	2838,38	0,09	8,89E-01
ENSRNOG00000015035	**Myo7b**	myosin VIIb (Myo7b), mRNA [Source:I	498834	18	24456652	24538352	81701	22,29	-0,11	8,89E-01
ENSRNOG00000016560	**RGD13060**	similar to RIKEN cDNA 1110007C09 (I	361224	17	17916365	17922567	6203	96,86	0,15	8,89E-01
ENSRNOG00000016715	**Kif11**	kinesin family member 11 (Kif11), mRI	171304	1	263519768	263570293	50526	375,74	0,17	8,89E-01

ENSRNOG00000020455	**Cst6**	cystatin E/M (Cst6), mRNA [Source:Re	171096	1	227656559	227658267	1709	429,91	0,13	8,89E-01
ENSRNOG00000024201		Uncharacterized protein [Source:UniProtKB/TrE		9	42599347	42651825	52479	47,94	-0,15	8,89E-01
ENSRNOG00000032639	**Foxo6**	Protein Foxo6 [Source:UniProtKB/TrE	313558	5	142994406	143013378	18973	804,22	-0,09	8,89E-01
ENSRNOG00000045960		protocadherin alpha-2 precursor [Sou	393086	18	29657322	29659709	2388	316,57	-0,11	8,89E-01
ENSRNOG00000046227		tumor protein p63 regulated 1-like (Tp	687090	5	174938793	174941982	3190	3337,87	0,08	8,89E-01
ENSRNOG00000015667	**Epcam**	epithelial cell adhesion molecule (Epca	171577	6	21264138	21280102	15965	41,02	0,17	8,90E-01
ENSRNOG00000007446	**Apip**	APAF1 interacting protein (Apip), mRN	295961	3	99612153	99638003	25851	144,05	-0,11	8,90E-01
ENSRNOG00000009113	**Marcksl1**	MARCKS-like 1 (Marcksl1), mRNA [Sc	81520	1	86018382	86035603	17222	1309,98	0,11	8,90E-01
ENSRNOG00000009258	**Cdk6**	cyclin-dependent kinase 6 (Cdk6), mR	114483	4	27688961	27869534	180574	136,69	0,12	8,90E-01
ENSRNOG00000010673		GTPase Era, mitochondrial [Source:R	363646	10	66341064	66356725	15662	628,97	0,09	8,90E-01
ENSRNOG00000012206	**Dennd5a**	DENN/MADD domain containing 5A (D	308942	1	181508456	181572938	64483	8219,68	-0,07	8,90E-01
ENSRNOG00000017120	**Abhd2**	abhydrolase domain containing 2 (Abh	293050	1	141965576	142049687	84112	1803,12	0,09	8,90E-01
ENSRNOG00000019142	**Fas**	Fas cell surface death receptor (Fas),	246097	1	259812248	259844583	32336	127,70	0,14	8,90E-01
ENSRNOG00000019934	**Paip2**	poly(A) binding protein interacting prot	361309	18	28123065	28129020	5956	1521,99	0,08	8,90E-01
ENSRNOG00000019986	**Pot1**	protection of telomeres 1 (Pot1), mRN	500054	4	51664674	51719559	54886	178,93	-0,13	8,90E-01
ENSRNOG00000021168	**Anp32e**	acidic (leucine-rich) nuclear phosphop	361999	2	217530775	217545154	14380	3365,40	0,09	8,90E-01
ENSRNOG00000024922	**Fbxo24**	F-box protein 24 (Fbxo24), mRNA [So	304374	12	24159896	24170247	10352	27,62	-0,14	8,90E-01
ENSRNOG00000028196	**Mgme1**	mitochondrial genome maintenance ex	296200	3	144832022	144840677	8656	76,47	0,12	8,90E-01
ENSRNOG00000036659	**Fn3k**	fructosamine 3 kinase (Fn3k), mRNA [	498034	10	110212812	110227791	14980	79,27	0,16	8,90E-01
ENSRNOG00000013169	**Traf4**	Tnf receptor associated factor 4 (Traf4	303285	10	66209815	66215917	6103	3729,19	0,15	8,90E-01
ENSRNOG00000017728	**RGD13068**	similar to erythroid differentiation-relat	309069	1	212655637	212692701	37065	633,32	-0,10	8,90E-01
ENSRNOG00000031824	**Slc44a2**	solute carrier family 44 (choline transp	363024	8	22422933	22439983	17051	1861,42	0,10	8,90E-01
ENSRNOG00000009482	**Emx2**	empty spiracles homeobox 2 (Emx2),	499380	1	287993648	288000656	7009	437,84	-0,08	8,90E-01
ENSRNOG00000014275		protein Hook homolog 3 [Source:RefS	306548	16	70407095	70495812	88718	209,84	-0,15	8,90E-01
ENSRNOG00000028092	**Cpa2**	carboxypeptidase A2 (pancreatic) (Cpa	296959	4	57616943	57640152	23210	82,68	0,14	8,90E-01
ENSRNOG00000002607	**Sox9**	Protein Sox9; RCG33659 [Source:Un	140586	10	100965137	100968386	3250	946,82	-0,13	8,90E-01
ENSRNOG00000023546	**Hspb1**	heat shock protein 1 (Hspb1), mRNA [	24471	12	25837084	25838661	1578	160,62	-0,17	8,90E-01
ENSRNOG00000005210		guanine nucleotide binding protein (G protein), a		4	13388237	13470279	82043	3046,51	-0,12	8,90E-01
ENSRNOG00000017129	**Ttll4**	Protein Ttll4 [Source:UniProtKB/TrEM	690512	9	81703012	81717068	14057	455,00	-0,08	8,90E-01
ENSRNOG00000045541	**LOC68739**	RIKEN cDNA 1700030J22 gene [Sour	687399	19	60299655	60309967	10313	102,44	-0,09	8,90E-01
ENSRNOG00000015975	**LOC49794**	RIKEN cDNA 2810408A11 gene [Sour	497940	10	56252672	56256702	4031	270,61	0,12	8,90E-01
ENSRNOG00000012752	**Cklf**	chemokine-like factor (Cklf), mRNA [S	245978	19	907067	915792	8726	39,24	-0,16	8,90E-01
ENSRNOG00000020088	**Klhl26**	kelch-like family member 26 (Klhl26), i	290657	16	20557872	20582253	24382	876,61	0,07	8,91E-01
ENSRNOG00000006116	**Hk2**	hexokinase 2 (Hk2), mRNA [Source:R	25059	4	178239350	178288251	48902	185,88	-0,11	8,91E-01
ENSRNOG00000020189	**Tinf2**	TERF1 (TRF1)-interacting nuclear fac	290232	15	38250951	38254058	3108	287,66	0,13	8,91E-01
ENSRNOG00000001070	**Cdk2ap1**	cyclin-dependent kinase 2 associated	360804	12	39538931	39549784	10854	3714,88	0,13	8,91E-01
ENSRNOG00000011726		glutamate receptor, ionotropic, N-meth	24408	3	2489166	2515963	26798	4166,74	0,12	8,91E-01
ENSRNOG00000025676		Protein Fam198a [Source:UniProtKB/	316090	8	129772970	129788337	15368	77,99	-0,14	8,91E-01

ENSRNOG00000014630	**Iws1**	IWS1 homolog (S. cerevisiae) (Iws1),	291705	18	24564414	24595079	30666	611,30	0,12	8,91E-01
ENSRNOG00000011739		DENN/MADD domain containing 4A [Source:MG		8	70023053	70104119	81067	612,49	-0,15	8,91E-01
ENSRNOG00000009635	**Tmem74b**	Protein Tmem74b; RCG37457; RCG3	499917	3	153578609	153579307	699	132,44	-0,09	8,91E-01
ENSRNOG00000002028	**Tmem50b**	transmembrane protein 50B (Tmem50	360698	11	35327977	35360982	33006	3597,97	-0,12	8,91E-01
ENSRNOG00000025126	**Palb2**	partner and localizer of BRCA2 (Palb2	293452	1	199127042	199151114	24073	80,69	-0,12	8,91E-01
ENSRNOG00000002210	**Hsd17b11**	hydroxysteroid (17-beta) dehydrogena	289456	14	7064323	7095461	31139	349,57	0,14	8,91E-01
ENSRNOG00000005619	**RGD13102**	rno-mir-1199 [Source:miRBase;Acc:M	304650	19	36108972	36110933	1962	23,67	-0,15	8,91E-01
ENSRNOG00000008249	**Brms1l**	breast cancer metastasis-suppressor	299053	6	86207478	86240310	32833	416,08	0,10	8,91E-01
ENSRNOG00000009206	**Fezf2**	Fez family zinc finger 2 (Fezf2), mRN/	305719	15	16854926	16858715	3790	755,73	0,10	8,91E-01
ENSRNOG00000009563	**Krt2**	keratin 2 (Krt2), mRNA [Source:RefSe	406228	7	141211466	141218154	6689	35,51	-0,15	8,91E-01
ENSRNOG00000009664	**Ankrd42**	ankyrin repeat domain 42 (Ankrd42), r	293117	1	163663271	163705736	42466	353,67	0,14	8,91E-01
ENSRNOG00000011785	**Xrn2**	5'-3' exoribonuclease 2 (Xrn2), mRNA	362229	3	147642886	147716287	73402	2596,98	-0,10	8,91E-01
ENSRNOG00000012552	**Ints4**	integrator complex subunit 4 (Ints4), m	308837	1	168654458	168718199	63742	1140,53	0,10	8,91E-01
ENSRNOG00000013676		1-phosphatidylinositol 4,5-bisphospha	29337	19	60828451	60942301	113851	23,40	0,14	8,91E-01
ENSRNOG00000013968	**Comtd1**	catechol-O-methyltransferase domain	305685	15	2612542	2615653	3112	580,79	-0,12	8,91E-01
ENSRNOG00000014829	**Prmt3**	protein arginine methyltransferase 3 (I	89820	1	106163844	106250508	86665	1192,45	0,11	8,91E-01
ENSRNOG00000016448	**Eef2k**	eukaryotic elongation factor-2 kinase (	25435	1	197704704	197768158	63455	2591,69	-0,10	8,91E-01
ENSRNOG00000017783	**Sfrp1**	secreted frizzled-related protein 1 (Sfr	84402	16	73009689	73045509	35821	880,59	-0,12	8,91E-01
ENSRNOG00000018267	**B3gnt7**	UDP-GlcNAc:betaGal beta-1,3-N-acet	316583	9	93055878	93059828	3951	321,57	-0,10	8,91E-01
ENSRNOG00000018593	**Txndc9**	thioredoxin domain containing 9 (Txnd	280671	9	44344165	44353711	9547	983,33	0,11	8,91E-01
ENSRNOG00000021270	**Trmt6**	tRNA methyltransferase 6 homolog (S	311441	3	131942470	131954191	11722	243,27	-0,11	8,91E-01
ENSRNOG00000023021	**Msl2**	Male-specific lethal 2-like 1 (Drosophil	315959	8	108873511	108895784	22274	630,52	-0,08	8,91E-01
ENSRNOG00000023639	**Rpusd1**	RNA pseudouridylate synthase domai	287148	10	14911834	14915688	3855	415,15	0,09	8,91E-01
ENSRNOG00000025635				6	61214769	61215174	406	223,35	-0,11	8,91E-01
ENSRNOG00000027299	**Pcdhga5**	protocadherin gamma subfamily A, 5 (	291637	18	30534927	30537518	2592	229,56	0,12	8,91E-01
ENSRNOG00000030630	**Ehmt2**	euchromatic histone lysine N-methyltr	361798	20	6656521	6672961	16441	4527,03	-0,10	8,91E-01
ENSRNOG00000031478	**Zfp663**	Protein Zfp663 [Source:UniProtKB/Tr	1E+08	3	168210633	168217863	7231	87,68	0,11	8,91E-01
ENSRNOG00000032487	**Sts**	steroid sulfatase (microsomal), isozym	24800	X	45646096	45654126	8031	164,37	0,15	8,91E-01
ENSRNOG00000038218	**Ndufc1**	Protein Ndufc1; RCG49984, isoform C	689938	2	159935705	159939478	3774	1941,56	-0,13	8,91E-01
ENSRNOG00000038436		Uncharacterized protein [Source:UniProtKB/TrE		2	143191113	143317876	126764	1478,92	0,15	8,91E-01
ENSRNOG00000042888	**Cdyl2**	chromodomain protein, Y-like 2 (Cdyl2	292044	19	59888243	59956167	67925	209,20	-0,09	8,91E-01
ENSRNOG00000047531		coiled-coil domain containing 57 [Source:MGI Sy		10	109599919	109688163	88245	166,20	-0,08	8,91E-01
ENSRNOG00000048282	**Mpnd**	MPN domain containing (Mpnd), mRN	681944	9	10040660	10046879	6220	1308,65	0,12	8,91E-01
ENSRNOG00000049414	**Mia3**	Protein LOC683007 [Source:UniProtK	683007	13	100936411	100964461	28051	2013,33	0,06	8,91E-01
ENSRNOG00000049730				1	185299044	185300070	1027	31,03	0,14	8,91E-01
ENSRNOG00000008190	**Pnpla7**	patatin-like phospholipase domain cor	246246	3	2164205	2243126	78922	302,22	-0,16	8,91E-01
ENSRNOG00000021687				3	89556578	89557305	728	656,93	-0,15	8,91E-01
ENSRNOG00000013186	**G3bp1**	GTPase activating protein (SH3 doma	171092	10	40644334	40678457	34124	5598,11	0,07	8,92E-01

ENSRNOG00000014936	**Ifitm2**	interferon induced transmembrane prc	114709	1	220671382	220672562	1181	132,73	-0,16	8,92E-01
ENSRNOG00000015411	**Apobec1**	apolipoprotein B mRNA editing enzym	25383	4	222411176	222425679	14504	61,06	-0,17	8,92E-01
ENSRNOG00000017569	**Mastl**	microtubule associated serine/threonir	307169	17	91504460	91539143	34684	49,50	0,17	8,92E-01
ENSRNOG00000017876	**Dnajc21**	DnaJ (Hsp40) homolog, subfamily C, r	192210	2	84235133	84250946	15814	147,90	0,10	8,92E-01
ENSRNOG00000018433	**Hps6**	Hermansky-Pudlak syndrome 6 (Hps6	309446	1	273191755	273194363	2609	157,85	0,13	8,92E-01
ENSRNOG00000018910	**Abhd11**	abhydrolase domain containing 11 (Ab	360831	12	26722553	26725258	2706	596,66	-0,09	8,92E-01
ENSRNOG00000031203	**Scfd1**	sec1 family domain containing 1 (Scfd	54350	6	81688281	81766841	78561	499,89	-0,15	8,92E-01
ENSRNOG00000001296	**P2rx7**	purinergic receptor P2X, ligand-gated	29665	12	41242368	41284797	42430	56,67	-0,11	8,92E-01
ENSRNOG00000038035	**Kif4a**	Protein Kif4a [Source:UniProtKB/TrEI	84393	X	71333718	71432169	98452	175,05	0,17	8,92E-01
ENSRNOG00000022796	**Fgfbp3**	fibroblast growth factor binding protein	499349	1	263030909	263032914	2006	1205,68	-0,15	8,92E-01
ENSRNOG00000005705	**RGD15622**	similar to Glutaminyl-peptide cyclotran	313837	6	1647589	1679763	32175	257,82	-0,09	8,92E-01
ENSRNOG00000012843	**Aspg**	asparaginase homolog (S. cerevisiae)	246266	6	145692075	145711786	19712	34,59	0,17	8,92E-01
ENSRNOG00000021758	**Tnpo3**	transportin 3 (Tnpo3), mRNA [Source:	296954	4	56587184	56664471	77288	2456,51	-0,09	8,92E-01
ENSRNOG00000008524	**LOC29688**	glucocorticoid induced transcript 1 (Gl	296884	4	34142803	34470794	327992	952,19	-0,11	8,92E-01
ENSRNOG00000012722	**Ppdpf**	pancreatic progenitor cell differentiatio	296470	3	180401475	180402473	999	1917,74	0,10	8,92E-01
ENSRNOG00000012806	**Rbbp6**	Protein Rbbp6 [Source:UniProtKB/TrE	308968	1	199993669	200024682	31014	1531,09	0,07	8,92E-01
ENSRNOG00000016206	**Uvrag**	UV radiation resistance associated ge	308846	1	170067082	170304150	237069	378,56	0,08	8,92E-01
ENSRNOG00000016280	**Btrc**	beta-transducin repeat containing E3 u	361765	1	272580308	272705419	125112	1509,02	0,11	8,92E-01
ENSRNOG00000016733	**Rab13**	RAB13, member RAS oncogene famil	81756	2	209014516	209019148	4633	781,09	-0,14	8,92E-01
ENSRNOG00000017558	**Tubb2a**	tubulin, beta 2A class IIa (Tubb2a), mF	498736	17	33386331	33390011	3681	6284,38	-0,12	8,92E-01
ENSRNOG00000019970	**mrpl11**	mitochondrial ribosomal protein L11 (n	293666	1	227174538	227177359	2822	868,88	0,07	8,92E-01
ENSRNOG00000020167	**Reep5**	receptor accessory protein 5 (Reep5),	364838	18	26842003	26873146	31144	9865,14	0,07	8,92E-01
ENSRNOG00000027552	**Tapbpl**	TAP binding protein-like (Tapbpl), mRI	297602	4	224752544	224759995	7452	428,63	0,15	8,92E-01
ENSRNOG00000027818	**Znhit3**	Protein Znhit3; Similar to thyroid horm	497975	10	72134570	72142765	8196	367,92	0,10	8,92E-01
ENSRNOG00000028834	**Polr2a**	DNA-directed RNA polymerase [Sour	363633	10	56085087	56110288	25202	5593,37	0,07	8,92E-01
ENSRNOG00000039829		Protein Rpp14 [Source:UniProtKB/TrE	361020	15	22593558	22598956	5399	414,91	0,09	8,92E-01
ENSRNOG00000049393	**Cebpb**	CCAAT/enhancer binding protein (C/E	24253	3	170577777	170578880	1104	160,40	-0,16	8,92E-01
ENSRNOG00000007607	**Nr4a1**	nuclear receptor subfamily 4, group A,	79240	7	140713176	140721070	7895	329,67	-0,11	8,92E-01
ENSRNOG00000012514	**Abhd17b**	abhydrolase domain containing 17B (A	309399	1	246554183	246587553	33371	989,00	-0,08	8,92E-01
ENSRNOG00000015020	**Idh1**	isocitrate dehydrogenase 1 (NADP+),	24479	9	70668272	70689957	21686	4175,22	-0,09	8,92E-01
ENSRNOG00000030247	**Ankhd1**	ankyrin repeat and KH domain contair	1E+08	18	29093401	29190681	97281	1063,67	0,11	8,92E-01
ENSRNOG00000043390	**Samd12**	sterile alpha motif domain containing 1	362910	7	93636142	93930039	293898	96,96	-0,15	8,92E-01
ENSRNOG00000014160	**Tcp1**	t-complex 1 (Tcp1), mRNA [Source:Re	24818	1	51719055	51726734	7680	899,05	0,11	8,92E-01
ENSRNOG00000016383	**Commd3**	COMM domain containing 3 (Commd3	291339	17	87079392	87083183	3792	2322,44	0,08	8,92E-01
ENSRNOG00000032888		M-phase phosphoprotein 8 (Mphosph8	290270	15	40732115	40799437	67323	753,40	-0,12	8,92E-01
ENSRNOG00000039461	**Pcdhga3**	protocadherin gamma subfamily A, 3 (	498847	18	30514378	30516962	2585	680,57	0,12	8,92E-01
ENSRNOG00000047605	**Ptprk**	protein tyrosine phosphatase, recepto	360302	1	18602550	18714240	111691	1554,93	-0,10	8,92E-01
ENSRNOG00000002305	**Slc15a2**	solute carrier family 15 (oligopeptide tr	60577	11	70022984	70052072	29089	1539,37	-0,09	8,92E-01

ENSRNOG00000003998	**Sgca**	sarcoglycan, alpha (dystrophin-associ	303468	10	82588479	82602338	13860	23,39	-0,15	8,92E-01
ENSRNOG00000005299	**Kif5a**	kinesin family member 5A (Kif5a), mR	314906	7	70690905	70727087	36183	22948,87	0,10	8,92E-01
ENSRNOG00000009515	**Pgm3**	phosphoglucomutase 3 (Pgm3), mRN	363109	8	93739288	93756358	17071	737,93	-0,08	8,92E-01
ENSRNOG00000011381	**Acsbg1**	acyl-CoA synthetase bubblegum famil	171410	8	57764975	57820724	55750	8182,30	-0,13	8,92E-01
ENSRNOG00000013195	**Ruvbl1**	RuvB-like 1 (E. coli) (Ruvbl1), mRNA [	65137	4	185652325	185687382	35058	853,86	0,10	8,92E-01
ENSRNOG00000014766	**Galt**	galactose-1-phosphate uridylyltransfer	298003	5	62669071	62672295	3225	178,46	-0,11	8,92E-01
ENSRNOG00000015691	**Fam212b**	family with sequence similarity 212, m	310764	2	227591605	227606918	15314	1047,44	-0,09	8,92E-01
ENSRNOG00000016134	**Msh6**	Protein Msh6 [Source:UniProtKB/TrEI	1E+08	6	21616774	21634251	17478	980,41	0,09	8,92E-01
ENSRNOG00000017380	**RGD13067**	uncharacterized protein LOC312511 [	312511	4	182814382	182843018	28637	341,49	0,13	8,92E-01
ENSRNOG00000020027	**Arhgef2**	rho/rac guanine nucleotide exchange f	310635	2	207396432	207423791	27360	2071,12	-0,09	8,92E-01
ENSRNOG00000027204	**Map6**	microtubule-associated protein 6 (Map	29457	1	170429900	170494770	64871	1342,86	-0,10	8,92E-01
ENSRNOG00000027938	**RGD15620**	Protein RGD1562037 [Source:UniPro	498764	17	53353516	53378127	24612	254,39	-0,09	8,92E-01
ENSRNOG00000030232	**LOC10036**	protein tyrosine phosphatase-like A do	300783	8	70170452	70207419	36968	7948,45	-0,10	8,92E-01
ENSRNOG00000031135		Protein Smarcc2 [Source:UniProtKB/T	685179	7	2852070	2879092	27023	6195,33	0,08	8,92E-01
ENSRNOG00000005614	**Txn2**	thioredoxin 2 (Txn2), mRNA [Source:F	79462	7	119138949	119152970	14022	2627,20	0,12	8,93E-01
ENSRNOG00000011763	**Serp1**	stress-associated endoplasmic reticulu	80881	2	168137271	168139485	2215	1472,26	-0,08	8,93E-01
ENSRNOG00000013969	**Ahi1**	Abelson helper integration site 1 (Ahi1	308923	1	18958623	19080397	121775	1311,31	0,06	8,93E-01
ENSRNOG00000017253	**Ccdc107**	Protein Ccdc107 [Source:UniProtKB/T	313496	5	63519844	63522846	3003	1045,41	-0,08	8,93E-01
ENSRNOG00000002826	**Hsd17b7**	hydroxysteroid (17-beta) dehydrogena	29540	13	92917716	92934194	16479	292,72	-0,14	8,93E-01
ENSRNOG00000017481	**Sucla2**	succinate-CoA ligase, ADP-forming, be	361071	15	59183906	59238106	54201	934,76	0,15	8,93E-01
ENSRNOG00000010891	**Lrrcc1**	leucine rich repeat and coiled-coil cen	266808	2	108155535	108184411	28877	142,25	-0,13	8,93E-01
ENSRNOG00000003977	**Dusp1**	dual specificity phosphatase 1 (Dusp1	114856	10	16867370	16870162	2793	552,14	-0,09	8,94E-01
ENSRNOG00000010125	**Skiv2l2**	superkiller viralicidic activity 2-like 2 (S	365668	2	63766778	63816210	49433	764,59	0,15	8,94E-01
ENSRNOG00000017610	**Nedd4l**	neural precursor cell expressed, devel	291553	18	59776172	59921504	145333	2860,98	-0,08	8,94E-01
ENSRNOG00000031004	**Cyp2j4**	cytochrome P450, family 2, subfamily	65210	5	123437432	123455817	18386	62,14	-0,16	8,94E-01
ENSRNOG00000003988	**Dnajc27**	DnaJ (Hsp40) homolog, subfamily C, r	298859	6	38322002	38351682	29681	2039,36	0,12	8,94E-01
ENSRNOG00000018193	**Poc5**	POC5 centriolar protein (Poc5), mRNA	294667	2	46127645	46152889	25245	315,78	0,09	8,94E-01
ENSRNOG00000029152	**Tmem69**	transmembrane protein 69 (Tmem69),	619582	5	139190201	139195856	5656	459,98	-0,07	8,94E-01
ENSRNOG00000020594	**Rhbdf1**	rhomboid 5 homolog 1 (Drosophila) (R	303008	10	15567525	15580427	12903	709,34	0,10	8,94E-01
ENSRNOG00000025946	**Igf2bp2**	insulin-like growth factor 2 mRNA bind	303824	11	85681571	85711438	29868	204,56	-0,10	8,94E-01
ENSRNOG00000007687	**Sema7a**	semaphorin 7A, GPI membrane ancho	315711	8	62499077	62521157	22081	1571,19	0,14	8,94E-01
ENSRNOG00000000398	**Zufsp**	zinc finger with UFM1-specific peptida	294390	20	34255577	34285599	30023	236,32	-0,12	8,94E-01
ENSRNOG00000000979	**Bri3bp**	Bri3 binding protein (Bri3bp), mRNA [S	498176	12	38445352	38457171	11820	388,76	-0,09	8,94E-01
ENSRNOG00000001376	**Mettl7a**	methyltransferase like 7A (Mettl7a), m	315306	7	139783755	139790601	6847	488,32	-0,16	8,94E-01
ENSRNOG00000002248		Protein Fryl [Source:UniProtKB/TrEM	364144	14	37424298	37566066	141769	1374,22	-0,08	8,94E-01
ENSRNOG00000003823	**Efcab11**	EF-hand calcium binding domain 11 (E	500705	6	132968496	133119919	151424	38,28	0,13	8,94E-01
ENSRNOG00000006970	**Ntn3**	netrin 3 (Ntn3), mRNA [Source:RefSec	114524	10	13393760	13396856	3097	139,64	-0,10	8,94E-01
ENSRNOG00000008986	**Diaph3**	Protein Diaph3 [Source:UniProtKB/Tr	290396	15	73786132	73958786	172655	76,96	0,15	8,94E-01

ENSRNOG00000009195	**Tnks1bp1**	Protein Tnks1bp1 [Source:UniProtKB,	295707	3	78972444	78993394	20951	1998,21	-0,08	8,94E-01
ENSRNOG00000013052		BCL2-associated transcription factor 1 [Source:M		1	17328181	17404737	76557	1597,45	0,15	8,94E-01
ENSRNOG00000014795	**Nr2f1**	nuclear receptor subfamily 2, group F,	81808	2	5553707	5562941	9235	2299,10	0,07	8,94E-01
ENSRNOG00000019339		Protein Ttll11; Ttll11 protein [Source:U	689746	3	20395616	20588216	192601	108,17	-0,11	8,94E-01
ENSRNOG00000023708	**Tmem176a**	transmembrane protein 176A (Tmem1	297077	4	143146281	143150058	3778	87,36	-0,17	8,94E-01
ENSRNOG00000023861	**Snap91**	synaptosomal-associated protein 91 (S	65178	8	93963290	94074083	110794	7175,22	0,10	8,94E-01
ENSRNOG00000046264		Uncharacterized protein [Source:UniProtKB/TrE		18	30123334	30134092	10759	114,51	0,11	8,94E-01
ENSRNOG00000029799	**Tomm34**	translocase of outer mitochondrial mei	311621	3	166632891	166649736	16846	1108,73	-0,10	8,94E-01
ENSRNOG00000039717		importin 11 [Source:MGI Symbol;Acc:I	310056	2	57050932	57205300	154369	464,33	0,13	8,94E-01
ENSRNOG00000047499	**Rbm15**	Protein LOC684233 [Source:UniProtK	684233	2	229554811	229557675	2865	183,91	0,11	8,94E-01
ENSRNOG00000011348	**Snx14**	sorting nexin 14 (Snx14), mRNA [Soui	315871	8	95519167	95583948	64782	425,20	-0,10	8,94E-01
ENSRNOG00000015029	**Dbt**	dihydrolipoamide branched chain trans	29611	2	237625212	237654081	28870	587,66	-0,10	8,94E-01
ENSRNOG00000021091	**Trank1**	tetratricopeptide repeat and ankyrin re	316022	8	118916156	118993803	77648	2050,27	-0,15	8,94E-01
ENSRNOG00000028640	**Siva1**	SIVA1, apoptosis-inducing factor (Siva	362791	6	146217298	146221553	4256	688,29	0,16	8,94E-01
ENSRNOG00000008659	**Arhgap21**	Rho GTPase activating protein 21 (Arl	307178	17	89415087	89486545	71459	5865,68	-0,06	8,94E-01
ENSRNOG00000000660	**Asphd2**	aspartate beta-hydroxylase domain cc	364948	12	52030649	52037039	6391	812,61	-0,11	8,94E-01
ENSRNOG00000014163		Protein Zfp536 [Source:UniProtKB/Trl	292820	1	94800255	95054443	254189	449,82	0,11	8,94E-01
ENSRNOG00000017758	**LOC36198**	similar to NICE-3 (LOC361985), mRN	361985	2	208833076	208846654	13579	2795,23	0,11	8,94E-01
ENSRNOG00000001795		integrin, beta 5 (Itgb5), mRNA [Source	257645	11	73146148	73261215	115068	1160,06	-0,11	8,94E-01
ENSRNOG00000013167	**LOC10091**	high mobility group box 2 (Hmgb2), ml	29395	16	35886764	35889338	2575	55,29	-0,10	8,94E-01
ENSRNOG00000028701	**C8g**	complement component 8, gamma po	296545	3	2706037	2708861	2825	35,11	0,13	8,94E-01
ENSRNOG00000050518	**LOC10091**	Protein LOC100910427; Protein RGD	498179	12	39559199	39572337	13139	25,69	0,17	8,94E-01
ENSRNOG00000004353	**Ppfia2**	protein tyrosine phosphatase, recepto	362876	7	48882184	49058116	175933	1426,42	0,12	8,94E-01
ENSRNOG00000008331	**RGD13099**	Protein RGD1309995 [Source:UniPro	314690	7	26439714	26491588	51875	766,12	0,15	8,94E-01
ENSRNOG00000016619	**Use1**	unconventional SNARE in the ER 1 hc	290627	16	19623234	19625901	2668	1815,76	0,14	8,94E-01
ENSRNOG00000002538		erythrocyte membrane protein band 4.	304733	13	40694351	40787937	93587	424,31	-0,09	8,94E-01
ENSRNOG00000001277	**Mafk**	v-maf avian musculoaponeurotic fibros	246760	12	18914797	18925513	10717	405,86	0,07	8,95E-01
ENSRNOG00000013892		Protein chibby homolog 1 [Source:Un	246768	7	120884400	120888649	4250	1719,31	-0,08	8,95E-01
ENSRNOG00000016744	**Chd6**	chromodomain helicase DNA binding	311607	3	163164938	163324764	159827	2327,02	0,08	8,95E-01
ENSRNOG00000004288	**Ptrh2**	peptidyl-tRNA hydrolase 2 (Ptrh2), mR	287593	10	76088309	76098333	10025	600,84	0,08	8,95E-01
ENSRNOG00000005122	**LOC10091**	UV-stimulated scaffold protein A (Uvss	314061	14	83308371	83344207	35837	127,86	-0,12	8,95E-01
ENSRNOG00000011400	**Ralyl**	RALY RNA binding protein-like (Ralyl)	294883	2	108813893	109080615	266723	516,63	-0,12	8,95E-01
ENSRNOG00000012798		tubulin, gamma complex associated pi	362203	3	119671767	119699292	27526	1066,30	-0,10	8,95E-01
ENSRNOG00000023521	**Map3k10**	mitogen activated protein kinase kinas	308463	1	85686570	85704588	18019	3943,14	0,12	8,95E-01
ENSRNOG00000013057	**Prc1**	protein regulator of cytokinesis 1 (Prc1	308761	1	143039960	143061006	21047	961,39	0,13	8,95E-01
ENSRNOG00000032293	**Polg**	polymerase (DNA directed), gamma (F	85472	1	142134505	142149608	15104	1406,80	0,11	8,95E-01
ENSRNOG00000010184	**Brk1**	BRICK1, SCAR/WAVE actin-nucleatin	679934	4	208855604	208870836	15233	4742,30	0,07	8,95E-01
ENSRNOG00000030120	**Ormdl2**	ORM1-like 2 (S. cerevisiae) (Ormdl2),	288783	7	3214561	3217641	3081	518,95	-0,09	8,95E-01

ENSRNOG00000000415	**Asf1a**	anti-silencing function 1A histone chap	294408	20	36651864	36666710	14847	866,31	-0,10	8,95E-01
ENSRNOG00000003749	**Xk**	X-linked Kx blood group (McLeod sync	497078	X	15278809	15315163	36355	106,82	-0,10	8,95E-01
ENSRNOG00000018950	**Fa2h**	fatty acid 2-hydroxylase (Fa2h), mRN/	307855	19	54355772	54407097	51326	83,99	-0,12	8,95E-01
ENSRNOG00000016427	**Letm1**	leucine zipper-EF-hand containing trar	305457	14	82917692	82956467	38776	2267,30	0,08	8,95E-01
ENSRNOG00000019388		Epidermal growth factor-like protein 7 [Source:U		3	9395191	9404525	9335	38,06	0,14	8,95E-01
ENSRNOG00000030775	**Adarb2**	adenosine deaminase, RNA-specific, I	117088	17	59905474	60096380	190907	498,65	-0,11	8,95E-01
ENSRNOG00000001653	**St3gal6**	ST3 beta-galactoside alpha-2,3-sialylt	304023	11	47382433	47426593	44161	58,33	-0,12	8,95E-01
ENSRNOG00000001727	**Lsg1**	large subunit GTPase 1 homolog (S. c	288029	11	76764254	76793848	29595	756,84	0,08	8,95E-01
ENSRNOG00000007426	**LOC68917**	transmembrane protein 64 [Source:M(	689176	5	33529654	33545931	16278	179,85	0,10	8,95E-01
ENSRNOG00000016342	**Atg10**	autophagy related 10 (Atg10), mRNA [	688555	2	19729784	20026448	296665	45,03	-0,14	8,95E-01
ENSRNOG00000017156	**Ankle1**	Protein Ankle1 [Source:UniProtKB/TrE	361122	16	19660584	19663917	3334	64,56	0,12	8,95E-01
ENSRNOG00000017673	**Brsk1**	BR serine/threonine kinase 1 (Brsk1),	499073	1	75808536	75833928	25393	7006,67	-0,08	8,95E-01
ENSRNOG00000027360	**Srsf7**	serine/arginine-rich splicing factor 7 (S	362687	6	2857412	2863365	5954	1792,22	-0,09	8,95E-01
ENSRNOG00000008332	**Smo**	smoothened, frizzled family receptor (	25273	4	56787102	56808940	21839	1426,67	0,12	8,95E-01
ENSRNOG00000016414	**Slc22a17**	solute carrier family 22, member 17 (S	305886	15	37456778	37463273	6496	16865,81	0,08	8,95E-01
ENSRNOG00000016417		zinc finger protein 882 [Source:MGI Symbol;Acc		18	794768	796608	1841	59,62	-0,15	8,95E-01
ENSRNOG00000019462	**Pcdh1**	Protein Pcdh1 [Source:UniProtKB/TrE	307481	18	31017455	31023632	6178	3343,13	0,08	8,95E-01
ENSRNOG00000021201	**Txnip**	thioredoxin interacting protein (Txnip),	117514	2	218170012	218173900	3889	4613,80	-0,14	8,95E-01
ENSRNOG00000004300	**Gtf2a1**	general transcription factor IIA, 1 (Gtf2	83830	6	124579899	124607638	27740	438,21	0,10	8,95E-01
ENSRNOG00000014900		cAMP responsive element modulator (	25620	17	55055679	55121385	65707	204,00	-0,10	8,95E-01
ENSRNOG00000000177	**Ppap2c**	phosphatidic acid phosphatase type 2	246115	7	13225606	13233682	8077	43,17	-0,16	8,95E-01
ENSRNOG00000002844		ubiquitin specific peptidase 27, X chro	302566	X	16773235	16774848	1614	312,68	-0,10	8,95E-01
ENSRNOG00000009675	**Cdc42bpb**	CDC42 binding protein kinase beta (D	113960	6	144585640	144667686	82047	6912,39	0,08	8,95E-01
ENSRNOG00000019858	**Rom1**	retinal outer segment membrane prote	309201	1	232064354	232066360	2007	145,30	-0,13	8,95E-01
ENSRNOG00000000802		TBC1 domain family, member 32 [Sou	1E+08	20	39211898	39445181	233284	137,54	-0,10	8,95E-01
ENSRNOG00000015389	**Stag1**	stromal antigen 1 (Stag1), mRNA [Sou	315958	8	108373849	108760291	386443	688,97	-0,13	8,95E-01
ENSRNOG00000020295	**Plekhn1**	pleckstrin homology domain containin	298694	5	177120235	177127945	7711	81,51	-0,14	8,95E-01
ENSRNOG00000024468	**Traf3ip1**	TNF receptor-associated factor 3 inter	363286	9	98297553	98332869	35317	291,74	-0,10	8,95E-01
ENSRNOG00000024671	**Dmxl1**	Protein Dmxl1 [Source:UniProtKB/TrE	307429	18	43689414	43865045	175632	472,39	-0,15	8,95E-01
ENSRNOG00000047686				5	146414652	146417633	2982	908,32	0,13	8,95E-01
ENSRNOG00000006949	**C1qbp**	complement component 1, q subcomp	29681	10	57359094	57363789	4696	2854,94	0,09	8,96E-01
ENSRNOG00000003276	**Myo1d**	myosin ID (Myo1d), mRNA [Source:Re	25485	10	67520650	67811744	291095	93,73	-0,09	8,96E-01
ENSRNOG00000018929	**Kif20b**	kinesin family member 20B (Kif20b), n	309523	1	260440354	260494019	53666	80,53	0,16	8,96E-01
ENSRNOG00000020687		Zinc finger protein neuro-d4 [Source:U	50545	1	88427539	88437120	9582	2886,24	0,10	8,96E-01
ENSRNOG00000030654	**Man2c1**	mannosidase, alpha, class 2C, membe	246136	8	60375817	60386940	11124	1811,59	0,06	8,96E-01
ENSRNOG00000000096	**Pdzk1**	PDZ domain containing 1 (Pdzk1), mF	65144	2	218452670	218484881	32212	226,72	-0,10	8,96E-01
ENSRNOG00000001425	**Sh2b2**	SH2B adaptor protein 2 (Sh2b2), mRN	114203	12	25473620	25501386	27767	509,46	0,09	8,96E-01
ENSRNOG00000008616		Protein Ubr3 [Source:UniProtKB/TrEN	311115	3	62759980	62914683	154704	2561,41	-0,10	8,96E-01

ENSRNOG00000009432	**Eif2s1**	eukaryotic translation initiation factor 2	54318	6	111422340	111447066	24727	1084,47	-0,11	8,96E-01
ENSRNOG00000018305	**St8sia3**	ST8 alpha-N-acetyl-neuraminide alpha	25547	18	58962843	58969336	6494	768,76	-0,08	8,96E-01
ENSRNOG00000019985	**Asic4**	acid-sensing (proton-gated) ion chann	63882	9	82416332	82438181	21850	520,71	-0,12	8,96E-01
ENSRNOG00000021793	**Tmem136**	transmembrane protein 136 (Tmem13	315593	8	45867546	45874816	7271	707,02	0,12	8,96E-01
ENSRNOG00000025244	**Trrap**	transformation/transcription domain-as	288471	12	13602590	13692085	89496	5183,50	0,06	8,96E-01
ENSRNOG00000000304	**Cd164**	CD164 molecule, sialomucin (Cd164),	83689	20	47942770	47954348	11579	2516,52	0,09	8,96E-01
ENSRNOG00000001151	**Sirt4**	sirtuin 4 (Sirt4), mRNA [Source:RefSe	304539	12	48665591	48673711	8121	108,59	-0,09	8,96E-01
ENSRNOG00000006925	**Zfp786**	zinc finger protein 786 (Zfp786), mRN	1E+08	4	142166950	142179995	13046	341,38	0,12	8,96E-01
ENSRNOG00000014777	**Prpf4**	PRP4 pre-mRNA processing factor 4 h	298095	5	82386980	82400904	13925	828,00	-0,07	8,96E-01
ENSRNOG00000015894	**Dock8**	dedicator of cytokinesis 8 (Dock8), mR	499337	1	250225062	250417768	192707	50,26	-0,15	8,96E-01
ENSRNOG00000017644	**Usp6nl**	USP6 N-terminal like (Usp6nl), mRNA	291309	17	77417426	77541567	124142	1166,31	0,09	8,96E-01
ENSRNOG00000018375	**Eif2b3**	eukaryotic translation initiation factor 2	171145	5	139677837	139744382	66546	360,44	0,12	8,96E-01
ENSRNOG00000018789	**Asic1**	acid-sensing (proton-gated) ion chann	79123	X	115830969	115859585	28617	2470,81	0,08	8,96E-01
ENSRNOG00000020291	**Fam35a**	family with sequence similarity 35, me	364514	16	8888936	8979969	91034	161,77	-0,11	8,96E-01
ENSRNOG00000025909	**Mnf1**	mitochondrial nucleoid factor 1 (Mnf1)	361805	20	7775855	7787605	11751	949,79	0,15	8,96E-01
ENSRNOG00000030109	**Kpna5**	karyopherin alpha 5 (importin alpha 6)	294392	20	34297579	34353838	56260	154,76	-0,12	8,96E-01
ENSRNOG00000037632	**LOC68855**	predicted gene 11992 [Source:MGI Sy	688553	14	89050478	89069657	19180	19,23	-0,16	8,96E-01
ENSRNOG00000016135	**Fars2**	phenylalanyl-tRNA synthetase 2, mito	306879	17	30910292	31335168	424877	531,76	-0,06	8,96E-01
ENSRNOG00000046871	**LOC68136**	RIKEN cDNA 2310039H08 gene [Sou	681367	9	15426094	15426438	345	266,49	0,14	8,96E-01
ENSRNOG00000002544		kinesin-associated protein 3 [Source:I	289168	13	86953394	87089644	136251	6217,12	0,10	8,96E-01
ENSRNOG00000002824	**LOC10091**	ring finger protein, LIM domain interac	317241	X	74567732	74583626	15895	24,64	-0,14	8,96E-01
ENSRNOG00000003289	**Lap3**	leucine aminopeptidase 3 (Lap3), mR	289668	14	70145277	70164260	18984	1805,64	-0,11	8,96E-01
ENSRNOG00000004234	**Mgat2**	mannosyl (alpha-1,6-)-glycoprotein be	94273	6	100935605	100938090	2486	1461,60	0,10	8,96E-01
ENSRNOG00000005318	**Ahsa2**	AHA1, activator of heat shock protein	305577	14	105098652	105109823	11172	704,73	0,08	8,96E-01
ENSRNOG00000006576		BMS1 homolog, ribosome assembly protein (yea		4	216359881	216395631	35751	746,74	0,13	8,96E-01
ENSRNOG00000011283		ACN9 homolog (S. cerevisiae) (Acn9),	362323	4	32359888	32421681	61794	90,26	-0,11	8,96E-01
ENSRNOG00000012019	**B3galnt1**	beta-1,3-N-acetylgalactosaminyltransf	310508	2	185583757	185613045	29289	2671,32	0,13	8,96E-01
ENSRNOG00000012081	**Txn1**	thioredoxin 1 (Txn1), mRNA [Source:R	116484	5	79201252	79209403	8152	4790,60	-0,11	8,96E-01
ENSRNOG00000012562	**Grin3b**	glutamate receptor, ionotropic, N-meth	170796	7	12934715	12941031	6317	247,32	-0,09	8,96E-01
ENSRNOG00000014617	**Tgoln2**	trans-golgi network protein 2 (Tgoln2),	192152	4	165245799	165253551	7753	2171,84	0,13	8,96E-01
ENSRNOG00000014674	**Exosc9**	exosome component 9 (Exosc9), mRN	294975	2	142879824	142890726	10903	545,14	-0,11	8,96E-01
ENSRNOG00000014986	**Ube4b**	ubiquitination factor E4B (Ube4b), mR	298652	5	169804688	169890811	86124	2926,71	0,06	8,96E-01
ENSRNOG00000015928	**Dhx35**	DEAH (Asp-Glu-Ala-His) box polypept	362260	3	160256785	160313877	57093	351,83	0,10	8,96E-01
ENSRNOG00000016728	**Tiam2**	Protein Tiam2 [Source:UniProtKB/TrE	1E+08	1	45659696	45762842	103147	1871,65	0,08	8,96E-01
ENSRNOG00000016878	**Lppr5**	lipid phosphate phosphatase-related p	310812	2	237965738	238169325	203588	1567,30	0,12	8,96E-01
ENSRNOG00000017955	**Nudt8**	Protein Nudt8; RCG47285, isoform CR	361692	1	226118370	226119800	1431	207,55	0,13	8,96E-01
ENSRNOG00000019093		neuregulin 2 (Nrg2), mRNA [Source:R	432361	18	28545126	28724922	179797	115,36	-0,10	8,96E-01
ENSRNOG00000023683	**Nog**	noggin (Nog), mRNA [Source:RefSeq	25495	10	76678292	76678990	699	130,61	-0,13	8,96E-01

ENSRNOG00000033508	**Mink1**	misshapen-like kinase 1 (Mink1), mRN	303259	10	56963630	56984070	20441	3277,74	-0,08	8,96E-01
ENSRNOG00000034240	**Bcor**	BCL6 co-repressor (Bcor), mRNA [Sou	317346	X	12446667	12488777	42111	1319,87	0,08	8,96E-01
ENSRNOG00000045665	**Amigo1**	adhesion molecule with Ig like domain	295365	2	230446904	230451545	4642	1191,53	-0,12	8,96E-01
ENSRNOG00000050636		Rho guanine nucleotide exchange fac	684811	5	162726802	162827928	101127	1928,27	0,09	8,96E-01
ENSRNOG00000010390	**Hmbs**	hydroxymethylbilane synthase (Hmbs)	25709	8	47286249	47293618	7370	700,49	0,08	8,97E-01
ENSRNOG00000024813	**Mzt1**	Protein Mzt1 [Source:UniProtKB/TrEM	692032	15	86948035	86953141	5107	148,78	-0,12	8,97E-01
ENSRNOG00000001347				12	42400868	42403856	2989	278,02	-0,12	8,97E-01
ENSRNOG00000026425		serine/threonine-protein phosphatase	300146	7	129912709	129945225	32517	3015,78	-0,07	8,97E-01
ENSRNOG00000045570	**LOC28897**	RIKEN cDNA 3110009E18 gene [Sour	288978	13	41264900	41329152	64253	50,86	0,11	8,97E-01
ENSRNOG00000011588	**Rnf146**	ring finger protein 146 (Rnf146), mRN	308051	1	32297192	32307783	10592	701,36	-0,12	8,97E-01
ENSRNOG00000042149	**LOC10036**	predicted gene 9047 [Source:MGI Syr	1E+08	4	56515853	56517639	1787	18,82	-0,15	8,98E-01
ENSRNOG00000004217	**Stk10**	serine/threonine kinase 10 (Stk10), ml	29398	10	17316681	17409678	92998	297,94	0,08	8,98E-01
ENSRNOG00000004207		echinoderm microtubule associated pr	444982	6	131991613	132118730	127118	222,80	-0,09	8,98E-01
ENSRNOG00000015188	**LOC10090**	zinc finger protein 661 [Source:MGI Sy	1E+08	3	127172411	127176402	3992	41,44	-0,12	8,98E-01
ENSRNOG00000005033	**Acvr1**	activin A receptor, type I (Acvr1), mRN	79558	3	49551414	49641517	90104	1033,64	-0,08	8,98E-01
ENSRNOG00000001582	**LOC10091**	BTB and CNC homology 1, basic leuc	304127	11	30989417	31023028	33612	413,99	-0,09	8,99E-01
ENSRNOG00000003139		structural maintenance of chromosomes 1A [Sou		X	21691772	21736383	44612	1629,47	0,15	8,99E-01
ENSRNOG00000024954	**Mgat5b**	mannosyl (alpha-1,6-)-glycoprotein be	303693	10	105520095	105589480	69386	2205,59	-0,08	8,99E-01
ENSRNOG00000010156	**Snapc5**	small nuclear RNA activating complex	691501	8	68833944	68837775	3832	428,65	-0,11	8,99E-01
ENSRNOG00000008816	**Gpnmb**	glycoprotein (transmembrane) nmb (G	113955	4	143383026	143404102	21077	1137,08	-0,14	8,99E-01
ENSRNOG00000047934		Protein B230219D22Rik; RCG24243	498701	17	11561581	11571465	9885	4334,32	-0,11	8,99E-01
ENSRNOG00000001309		calcium/calmodulin-dependent protein	83506	12	41155843	41191356	35514	723,95	-0,11	8,99E-01
ENSRNOG00000016965	**Anapc1**	anaphase promoting complex subunit	311412	3	128786183	128864613	78431	4115,91	-0,07	8,99E-01
ENSRNOG00000017631	**Cyp20a1**	cytochrome P450, family 20, subfamily	316435	9	66943519	66993517	49999	721,60	-0,07	8,99E-01
ENSRNOG00000017887	**Mutyh**	mutY homolog (E. coli) (Mutyh), mRN/	170841	5	139459661	139471463	11803	222,83	0,09	8,99E-01
ENSRNOG00000030126	**Clk3**	CDC-like kinase 3 (Clk3), mRNA [Sou	171305	8	62305184	62318918	13735	1244,58	0,06	8,99E-01
ENSRNOG00000030339				MT	15280	15346	67	87,25	-0,11	8,99E-01
ENSRNOG00000020310	**Grik5**	glutamate receptor, ionotropic, kainate	24407	1	83137817	83207039	69223	4068,18	0,07	8,99E-01
ENSRNOG00000021448	**Tmem57**	transmembrane protein 57 (Tmem57),	313618	5	156896212	156958442	62231	2671,76	-0,10	9,00E-01
ENSRNOG00000028493	**Ppp1r15b**	protein phosphatase 1, regulatory sub	304799	13	55001947	55009303	7357	2212,18	-0,09	9,00E-01
ENSRNOG00000017093	**Pxdc1**	PX domain containing 1 (Pxdc1), mRN	361238	17	32865487	32893955	28469	428,34	-0,12	9,00E-01
ENSRNOG00000001770	**Ehhadh**	enoyl-CoA, hydratase/3-hydroxyacyl C	171142	11	86024803	86057811	33009	18,66	0,14	9,00E-01
ENSRNOG00000022249	**Mllt10**	myeloid/lymphoid or mixed-lineage leu	361285	17	86605397	86705156	99760	1066,37	0,11	9,00E-01
ENSRNOG00000001766	**Tfrc**	transferrin receptor (Tfrc), mRNA [Sou	64678	11	74479841	74502561	22721	1916,36	-0,12	9,00E-01
ENSRNOG00000003981	**Zfp18**	zinc finger protein 18 (Zfp18), mRNA [	303226	10	52085992	52100911	14920	362,24	0,10	9,00E-01
ENSRNOG00000004112	**RGD13087**	similar to RIKEN cDNA 4921536K21 (	305476	14	84825689	84834332	8644	46,59	0,11	9,00E-01
ENSRNOG00000004478	**Srrm2**	serine/arginine repetitive matrix 2 (Srr	302969	10	12965077	12982440	17364	14906,23	-0,07	9,00E-01
ENSRNOG00000005602	**Mthfd1**	methylenetetrahydrofolate dehydroger	64300	6	108694405	108761891	67487	1070,76	0,08	9,00E-01

ENSRNOG00000005823	**Utp20**	UTP20, small subunit (SSU) processo	314713	7	29399170	29479994	80825	780,15	0,10	9,00E-01
ENSRNOG00000008578		cyclin-dependent kinase 16 (Cdk16), t	81741	X	2500720	2512068	11349	5715,48	-0,07	9,00E-01
ENSRNOG00000009928	**Bckdhb**	branched chain keto acid dehydrogena	29711	8	90982519	91173704	191186	924,85	0,07	9,00E-01
ENSRNOG00000011293	**RGD13117**	similar to 2700029M09Rik protein (RG	290706	16	32358969	32375073	16105	741,25	0,11	9,00E-01
ENSRNOG00000012419	**RGD15591**	Protein RGD1559150 [Source:UniPro	311353	3	119661131	119670600	9470	129,96	0,13	9,00E-01
ENSRNOG00000012880	**Tti1**	TELO2 interacting protein 1 (Tti1), mR	499935	3	161113415	161140919	27505	901,70	0,09	9,00E-01
ENSRNOG00000017291	**Sord**	sorbitol dehydrogenase (Sord), mRNA	24788	3	120716970	120746949	29980	1459,78	-0,11	9,00E-01
ENSRNOG00000019490	**Slc20a2**	solute carrier family 20 (phosphate tra	29502	16	73949887	74042911	93025	1169,00	0,12	9,00E-01
ENSRNOG00000021023		myelin-associated glycoprotein (Mag),	29409	1	90500839	90516262	15424	40,98	-0,15	9,00E-01
ENSRNOG00000022812		Excision repair cross-complementing rodent repa		9	50592370	50637767	45398	293,99	-0,08	9,00E-01
ENSRNOG00000025716	**Lsm10**	LSM10, U7 small nuclear RNA associa	366468	5	147876338	147878244	1907	261,87	0,10	9,00E-01
ENSRNOG00000043037	**Zfp770**	Protein Zfp770 [Source:UniProtKB/Tr	691610	3	112234699	112236810	2112	130,80	-0,16	9,00E-01
ENSRNOG00000048847	**Wdr17**	Protein LOC364653 [Source:UniProtK	364653	16	39602548	39713667	111120	294,51	-0,11	9,00E-01
ENSRNOG00000005096	**Bzw2**	basic leucine zipper and W2 domains	171439	6	65200722	65261870	61149	4179,93	0,10	9,00E-01
ENSRNOG00000001383	**Slc24a6**	solute carrier family 24 (sodium/lithium	498185	12	43421507	43444143	22637	68,45	-0,14	9,00E-01
ENSRNOG00000003185	**Acbd3**	acyl-CoA binding domain containing 3	289312	13	104006998	104035795	28798	1186,88	0,09	9,00E-01
ENSRNOG00000007472	**Setd5**	SET domain containing 5 (Setd5), mR	297514	4	208315965	208393602	77638	4683,91	0,08	9,00E-01
ENSRNOG00000010633	**Acsl1**	acyl-CoA synthetase long-chain family	25288	16	48653023	48697521	44499	1426,31	-0,06	9,00E-01
ENSRNOG00000010807	**LOC10036**	cytochrome c oxidase, subunit VIc (Co	54322	7	74880039	74892526	12488	6199,97	0,07	9,00E-01
ENSRNOG00000012262	**Depdc7**	DEP domain containing 7 (Depdc7), m	295971	3	101290429	101311699	21271	84,60	-0,14	9,00E-01
ENSRNOG00000023931	**Ggn**	gametogenetin (Ggn), mRNA [Source:	292765	1	89288946	89292049	3104	60,38	0,12	9,00E-01
ENSRNOG00000028708	**Ntsr1**	neurotensin receptor 1 (Ntsr1), mRNA	366274	3	179681394	179747034	65641	638,05	-0,09	9,00E-01
ENSRNOG00000031406	**Hps3**	Hermansky-Pudlak syndrome 3 homol	310288	2	124523995	124551506	27512	273,83	0,13	9,00E-01
ENSRNOG00000000987	**Ptcd1**	pentatricopeptide repeat domain 1 (Pt	304278	12	13284989	13302661	17673	893,27	0,08	9,00E-01
ENSRNOG00000004686	**Spop**	speckle-type POZ protein (Spop), mRI	287643	10	83040649	83121496	80848	1917,07	0,06	9,00E-01
ENSRNOG00000004812	**Sema6d**	sema domain, transmembrane domair	311384	3	123424951	123482782	57832	3340,56	0,07	9,00E-01
ENSRNOG00000005009	**Zfp3**	zinc finger protein 3 (Zfp3), mRNA [So	497944	10	57132328	57140571	8244	206,55	-0,08	9,00E-01
ENSRNOG00000007822	**Vgll4**	vestigial like 4 (Drosophila) (Vgll4), mF	297523	4	210069605	210129654	60050	1478,00	-0,09	9,00E-01
ENSRNOG00000012396	**Rgr**	retinal G protein coupled receptor (Rg	306307	16	14188718	14203346	14629	29,58	-0,14	9,00E-01
ENSRNOG00000018835	**Notch2**	notch 2 (Notch2), mRNA [Source:RefS	29492	2	219662511	219793306	130796	2292,39	-0,11	9,00E-01
ENSRNOG00000019744	**Prr16**	proline rich 16 (Prr16), mRNA [Source	361327	18	45166704	45370225	203522	26,26	-0,12	9,00E-01
ENSRNOG00000028703	**Slc39a6**	solute carrier family 39 (zinc transport	291733	18	16282423	16302964	20542	2895,92	-0,10	9,00E-01
ENSRNOG00000043113	**Fam43b**	Protein Fam43b [Source:UniProtKB/T	313650	5	160558871	160559863	993	157,91	0,11	9,00E-01
ENSRNOG00000047817		Protein Enox1; RCG36864 [Source:U	306038	15	63013560	63564417	550858	805,80	-0,08	9,00E-01
ENSRNOG00000000579	**Marcks**	myristoylated alanine rich protein kina	25603	20	44693601	44696898	3298	7379,77	0,07	9,00E-01
ENSRNOG00000003223	**Degs1**	delta(4)-desaturase, sphingolipid 1 (De	58970	13	105599916	105606572	6657	1933,23	0,07	9,00E-01
ENSRNOG00000003938	**Tubd1**	tubulin, delta 1 (Tubd1), mRNA [Sourc	287592	10	76212408	76233751	21344	138,99	0,10	9,00E-01
ENSRNOG00000007808	**Nap1l5**	nucleosome assembly protein 1-like 5	688843	4	153970373	153972240	1868	1469,89	0,09	9,00E-01

ENSRNOG00000008613	**Gatad1**	Protein Gatad1 [Source:UniProtKB/Tr	500005	4	27550561	27561759	11199	2150,86	-0,09	9,00E-01
ENSRNOG00000011383	**Ndufs4**	NADH dehydrogenase (ubiquinone) F	499529	2	65403183	65515260	112078	1475,87	-0,11	9,00E-01
ENSRNOG00000016241		FH1/FH2 domain-containing protein 1	291964	19	48150419	48169181	18763	89,28	-0,11	9,00E-01
ENSRNOG00000017997	**Nek4**	NIMA-related kinase 4 (Nek4), mRNA	306252	16	6961230	7004141	42912	299,73	-0,09	9,00E-01
ENSRNOG00000025792	**Crk**	v-crk avian sarcoma virus CT10 oncog	54245	10	64149258	64175516	26259	4665,74	-0,11	9,00E-01
ENSRNOG00000037604	**Ascc3**	activating signal cointegrator 1 complex subunit		20	57025333	57156837	131505	150,21	0,15	9,00E-01
ENSRNOG00000038211	**RGD15597**	Protein RGD1559751 [Source:UniPro	689153	18	85031735	85033111	1377	25,06	0,13	9,00E-01
ENSRNOG00000047213	**Gnpda1**	glucosamine-6-phosphate deaminase	683570	18	31157709	31166267	8559	701,02	0,11	9,00E-01
ENSRNOG00000005290	**Ywhae**	tyrosine 3-monooxygenase/tryptophar	29753	10	64084523	64121823	37301	40426,79	-0,06	9,01E-01
ENSRNOG00000024065	**Zfp575**	zinc finger protein 575 (Zfp575), mRN	308430	1	82707389	82709496	2108	326,65	-0,07	9,01E-01
ENSRNOG00000003578	**Fem1c**	fem-1 homolog c (C. elegans) (Fem1c	302288	18	40148636	40173809	25174	958,10	-0,08	9,01E-01
ENSRNOG00000008733	**Lsm3**	LSM3 homolog, U6 small nuclear RNA	297455	4	187377370	187383614	6245	692,77	0,11	9,01E-01
ENSRNOG00000009266	**Anp32b**	acidic (leucine-rich) nuclear phosphop	170724	5	66591103	66624323	33221	2454,43	0,10	9,01E-01
ENSRNOG00000008056	**Ankrd9**	ankyrin repeat domain 9 (Ankrd9), mR	314457	6	145000298	145002580	2283	104,14	-0,13	9,01E-01
ENSRNOG00000006308	**Mycn**	v-myc avian myelocytomatosis viral or	298894	6	51348170	51353994	5825	1798,05	0,07	9,01E-01
ENSRNOG00000023335		additional sex combs like 1 [Source:MGI Symbo		3	155204644	155263216	58573	1614,80	0,08	9,01E-01
ENSRNOG00000009032	**Ttll3**	tubulin tyrosine ligase-like family, mem	362415	4	208639199	208662542	23344	48,84	0,12	9,01E-01
ENSRNOG00000026248	**Nmt2**	N-myristoyltransferase 2 (Nmt2), mRN	291318	17	80562797	80583124	20328	1010,42	0,08	9,01E-01
ENSRNOG00000048476	**RGD15624**	ATP synthase-coupling factor 6, mitoc	500560	5	155473746	155474447	702	445,56	0,07	9,01E-01
ENSRNOG00000001827	**Masp1**	mannan-binding lectin serine peptidas	64023	11	79916067	79998571	82505	1287,86	0,07	9,01E-01
ENSRNOG00000016117	**Myof**	myoferlin (Myof), mRNA [Source:RefS	309499	1	264066287	264215424	149138	173,91	-0,13	9,01E-01
ENSRNOG00000020498	**Megf8**	multiple EGF-like-domains 8 (Megf8),	114029	1	83449539	83497902	48364	4964,74	0,06	9,01E-01
ENSRNOG00000046667	**Fosb**	FBJ osteosarcoma oncogene B (Fosb	1E+08	1	81481174	81487900	6727	38,19	-0,15	9,01E-01
ENSRNOG00000032374	**Paqr9**	progestin and adipoQ receptor family	315904	8	102795907	102797028	1122	98,25	0,11	9,01E-01
ENSRNOG00000033921	**Als2cl**	Protein Als2cl [Source:UniProtKB/TrE	316017	8	118499106	118513205	14100	269,72	-0,13	9,01E-01
ENSRNOG00000000763	**RT1-M3-1**	RT1 class Ib, locus M3, gene 1 (RT1-M	24747	20	3856748	3860887	4140	232,12	0,14	9,01E-01
ENSRNOG00000018845		small nuclear RNA activating complex	362088	3	9170635	9185512	14878	389,34	0,10	9,01E-01
ENSRNOG00000046530	**Zdhhc16**	zinc finger, DHHC-type containing 16 (	654495	1	268632479	268643839	11361	1110,79	0,08	9,01E-01
ENSRNOG00000000635		AT-rich interactive domain-containing	309728	20	23939869	23980214	40346	678,63	0,06	9,02E-01
ENSRNOG00000000917	**Nupr1l**	nuclear protein, transcriptional regulat	360799	12	32248567	32248878	312	290,97	0,13	9,02E-01
ENSRNOG00000001767	**RGD15623**	Protein RGD1562339 [Source:UniPro	498107	11	86128083	86173597	45515	425,05	0,09	9,02E-01
ENSRNOG00000004844	**Med30**	mediator complex subunit 30 (Med30)	299905	7	92876548	92898051	21504	660,97	-0,10	9,02E-01
ENSRNOG00000009299	**Adra2c**	adrenoceptor alpha 2C (Adra2c), mRN	24175	14	81422367	81423743	1377	124,10	-0,13	9,02E-01
ENSRNOG00000010682	**Clcn3**	chloride channel, voltage-sensitive 3 (	84360	16	32284020	32353988	69969	3953,43	-0,10	9,02E-01
ENSRNOG00000016833	**Ide**	insulin degrading enzyme (Ide), mRNA	25700	1	263390397	263488576	98180	2368,19	-0,08	9,02E-01
ENSRNOG00000018791		DNL-type zinc finger (Dnlz), mRNA [S	296587	3	9157628	9159374	1747	1108,38	0,13	9,02E-01
ENSRNOG00000022921	**Dact2**	dapper, antagonist of beta-catenin, ho	308212	1	57354081	57362996	8916	129,99	-0,09	9,02E-01
ENSRNOG00000023213	**Dmrtb1**	DMRT-like family B with proline-rich C	313484	5	131114908	131121740	6833	206,57	-0,13	9,02E-01

ENSRNOG00000024934	**Npat**	nuclear protein, ataxia-telangiectasia l	315666	8	56703321	56740923	37603	402,74	-0,12	9,02E-01
ENSRNOG00000047653		crystallin, beta B1 (Crybb1), mRNA [S	25421	12	52147269	52165088	17820	17,62	-0,15	9,02E-01
ENSRNOG00000002749	**Spag9**	sperm associated antigen 9 (Spag9), r	360600	10	81527102	81660894	133793	9153,46	0,08	9,02E-01
ENSRNOG00000007202	**Sema3d**	sema domain, immunoglobulin domair	246262	4	19169069	19359164	190096	29,33	-0,15	9,02E-01
ENSRNOG00000008711	**Med24**	mediator complex subunit 24 (Med24)	619436	10	86416484	86441676	25193	1735,93	0,07	9,02E-01
ENSRNOG00000012420	**Bcl9l**	B-cell CLL/lymphoma 9-like (Bcl9l), ml	300673	8	47424070	47452698	28629	3392,35	-0,06	9,02E-01
ENSRNOG00000016552	**Hmgcs1**	3-hydroxy-3-methylglutaryl-CoA synth	29637	2	70800442	70809191	8750	21709,30	-0,14	9,02E-01
ENSRNOG00000019612		hydroxyacylglutathione hydrolase-like [Source:M		10	14965681	14968346	2666	382,08	0,09	9,02E-01
ENSRNOG00000036910	**LOC10036**	Protein LOC100362344 [Source:UniP	362428	4	248214033	248215196	1164	90,50	-0,12	9,02E-01
ENSRNOG00000049569				1	21819063	21819643	581	45,83	0,12	9,02E-01
ENSRNOG00000006740	**Gatsl3**	GATS protein-like 3 (Gatsl3), mRNA [S	360969	14	85129586	85133998	4413	20,20	-0,15	9,02E-01
ENSRNOG00000007422	**Vav2**	vav 2 guanine nucleotide exchange fa	296603	3	11173752	11337325	163574	1601,57	-0,07	9,02E-01
ENSRNOG00000026048	**Gbf1**	golgi brefeldin A resistant guanine nuc	309451	1	273335046	273464048	129003	4450,83	0,06	9,02E-01
ENSRNOG00000040145	**Abracl**	ABRA C-terminal like (Abracl), mRNA	685045	1	14859231	14869483	10253	524,41	0,09	9,02E-01
ENSRNOG00000042951	**Xrn1**	Protein LOC100911537 [Source:UniP	300944	8	103225041	103329228	104188	428,25	-0,10	9,02E-01
ENSRNOG00000008747	**Plekha5**	pleckstrin homology domain containin	246237	4	238838538	239007298	168761	885,43	0,11	9,02E-01
ENSRNOG00000006646	**Vopp1**	vesicular, overexpressed in cancer, pr	362374	4	153266271	153337776	71506	4257,64	0,10	9,02E-01
ENSRNOG00000020083	**Scly**	selenocysteine lyase (Scly), mRNA [S	363285	9	98115050	98135542	20493	316,31	-0,09	9,02E-01
ENSRNOG00000019650	**Rbm4b**	RNA binding motif protein 4B (Rbm4b)	474154	1	226874271	226884602	10332	696,03	0,08	9,03E-01
ENSRNOG00000009484	**Ptcd3**	Pentatricopeptide repeat domain 3 (Pt	500199	4	164573542	164600825	27284	618,28	-0,10	9,03E-01
ENSRNOG00000019416	**Zfp94**	zinc finger protein 94 (Zfp94), mRNA [	499095	1	82400963	82415836	14874	316,57	0,12	9,03E-01
ENSRNOG00000020546	**Lipe**	lipase, hormone sensitive (Lipe), mRN	25330	1	83511519	83530200	18682	135,96	-0,10	9,03E-01
ENSRNOG00000001992	**Atp6v1a**	ATPase, H+ transporting, lysosomal V	685232	11	65638470	65691322	52853	6447,04	0,10	9,03E-01
ENSRNOG00000005362	**Rab3ip**	RAB3A interacting protein (Rab3ip), m	29885	7	59933272	59962078	28807	555,44	-0,11	9,03E-01
ENSRNOG00000050554	**LOC10036**	RIKEN cDNA 9430038I01 gene [Sour	1E+08	1	216635512	216673147	37636	65,57	-0,13	9,03E-01
ENSRNOG00000001210	**Pwp2**	PWP2 periodic tryptophan protein hon	690297	20	13398455	13412219	13765	1009,97	0,07	9,03E-01
ENSRNOG00000006309	**Soga1**	Protein Soga1 [Source:UniProtKB/TrE	311578	3	158525115	158589194	64080	4057,99	0,07	9,03E-01
ENSRNOG00000014602	**Mtfmt**	mitochondrial methionyl-tRNA formyltr	315763	8	70644131	70674636	30506	387,19	-0,08	9,03E-01
ENSRNOG00000046484	**Ccdc160**	Protein LOC683891; RCG53195, isofo	683891	X	140224652	140225620	969	21,93	-0,15	9,03E-01
ENSRNOG00000004332	**Egfr**	epidermal growth factor receptor (Egfr	24329	14	100438987	100617315	178329	1332,69	-0,13	9,04E-01
ENSRNOG00000005417	**Zhx2**	zinc fingers and homeoboxes 2 (Zhx2)	314988	7	98300087	98302597	2511	540,51	-0,09	9,04E-01
ENSRNOG00000007981	**Klhl23**	kelch-like family member 23 (Klhl23), r	311114	3	62692399	62705970	13572	1515,55	-0,08	9,04E-01
ENSRNOG00000009217	**Fbxo6**	F-box protein 6 (Fbxo6), mRNA [Sourc	192351	5	168614323	168617886	3564	826,16	0,09	9,04E-01
ENSRNOG00000039390	**Slc37a2**	solute carrier family 37 (glucose-6-pho	500973	8	39713122	39734767	21646	30,91	-0,14	9,04E-01
ENSRNOG00000004781	**Crmp1**	collapsin response mediator protein 1	25415	14	78240967	78282969	42003	34197,94	-0,10	9,04E-01
ENSRNOG00000009088	**Txnrd1**	thioredoxin reductase 1 (Txnrd1), mRI	58819	7	27065820	27104095	38276	4304,98	-0,05	9,04E-01
ENSRNOG00000022199	**Zmynd12**	zinc finger, MYND-type containing 12	313552	5	142280330	142312575	32246	34,49	-0,14	9,04E-01
ENSRNOG00000024870	**Anks1b**	ankyrin repeat and sterile alpha motif	314721	7	30922186	31772758	850573	1348,15	-0,08	9,04E-01

ENSRNOG00000047708	**Gstz1**	glutathione S-transferase zeta 1 (Gstz	681913	6	120460963	120471409	10447	596,72	-0,07	9,04E-01
ENSRNOG00000012748	**Vamp8**	vesicle-associated membrane protein	83730	4	165020594	165023049	2456	60,95	-0,14	9,04E-01
ENSRNOG00000012957	**Sh3glb1**	SH3-domain GRB2-like endophilin B1	292156	2	269242689	269271017	28329	2158,78	-0,05	9,04E-01
ENSRNOG00000005710		RNA binding motif protein 7 [Source:MGI Symbo		8	51415941	51421071	5131	388,76	-0,07	9,04E-01
ENSRNOG00000018162	**Nhej1**	nonhomologous end-joining factor 1 (N	363251	9	81999493	82096795	97303	59,22	-0,13	9,04E-01
ENSRNOG00000032519				18	53729188	53730131	944	79,51	-0,13	9,04E-01
ENSRNOG00000005802	**Usp24**	ubiquitin specific peptidase 24 (Usp24	313427	5	129744412	129876157	131746	2428,47	0,11	9,04E-01
ENSRNOG00000030055	**Vamp3**	vesicle-associated membrane protein	29528	5	171703254	171713723	10470	2180,75	-0,08	9,04E-01
ENSRNOG00000005718	**LOC100911956**			6	135075500	135076578	1079	42,27	-0,14	9,05E-01
ENSRNOG00000015207		Uncharacterized protein [Source:UniProtKB/TrE		17	53566763	53567862	1100	217,81	-0,08	9,05E-01
ENSRNOG00000016813	**Tia1**	TIA1 cytotoxic granule-associated RN	312510	4	182779170	182806468	27299	1048,36	-0,13	9,05E-01
ENSRNOG00000022307	**Acap3**	ArfGAP with coiled-coil, ankyrin repea	313772	5	176815678	176830373	14696	4020,35	0,09	9,05E-01
ENSRNOG00000032410	**RGD15625**	similar to mKIAA0774 protein (RGD15	498136	12	10197626	10240087	42462	211,60	-0,11	9,05E-01
ENSRNOG00000000775	**Mog**	myelin oligodendrocyte glycoprotein (M	24558	20	4044677	4054910	10234	385,58	0,09	9,05E-01
ENSRNOG00000002980	**Tsr1**	Protein Tsr1; RCG34104, isoform CRA	1E+08	10	61467842	61479252	11411	909,98	0,10	9,05E-01
ENSRNOG00000007126	**Gpr19**	G protein-coupled receptor 19 (Gpr19)	312787	4	232913212	232929837	16626	678,02	0,10	9,05E-01
ENSRNOG00000007753	**Plaa**	phospholipase A2, activating protein (I	116645	5	117491919	117523679	31761	2027,13	-0,09	9,05E-01
ENSRNOG00000010843	**Nhlrc3**	Protein Nhlrc3 [Source:UniProtKB/TrE	310416	2	162358223	162369485	11263	294,94	0,10	9,05E-01
ENSRNOG00000011513	**Gla**	galactosidase, alpha (Gla), mRNA [So	363494	X	105295029	105306686	11658	1067,49	0,11	9,05E-01
ENSRNOG00000014960	**Bard1**	BRCA1 associated RING domain 1 (B	64557	9	78074908	78145962	71055	210,35	0,09	9,05E-01
ENSRNOG00000015621	**Whsc1l1**	Wolf-Hirschhorn syndrome candidate	290831	16	70791573	70897651	106079	1643,41	0,07	9,05E-01
ENSRNOG00000019869	**Lrfn1**	leucine rich repeat and fibronectin type	365222	1	86507668	86515938	8271	1452,29	0,09	9,05E-01
ENSRNOG00000026080	**LOC68839**	RIKEN cDNA 9030612E09 gene [Sour	688390	20	49571819	49573769	1951	43,44	0,12	9,05E-01
ENSRNOG00000046727	**Abcc2**	ATP-binding cassette, subfamily C (CF	25303	1	270999832	271057750	57919	99,67	-0,11	9,05E-01
ENSRNOG00000048782	**Wnt7a**	wingless-type MMTV integration site fa	114850	4	187505391	187551604	46214	2121,50	-0,08	9,05E-01
ENSRNOG00000049686	**Mrpl40**	mitochondrial ribosomal protein L40 (M	287962	11	89378582	89384417	5836	141,61	-0,10	9,05E-01
ENSRNOG00000008415	**Nab2**	Ngfi-A binding protein 2 (Nab2), mRNA	314910	7	71135886	71142278	6393	556,57	0,11	9,05E-01
ENSRNOG00000005544		KAT8 regulatory NSL complex subunit	360642	10	92150853	92236188	85336	1524,60	-0,08	9,05E-01
ENSRNOG00000038574	**Rassf9**	Ras association (RalGDS/AF-6) doma	65053	7	44205842	44207104	1263	29,05	0,12	9,05E-01
ENSRNOG00000022615	**Tp53i13**	tumor protein p53 inducible protein 13	287550	10	62347593	62352210	4618	202,46	-0,12	9,05E-01
ENSRNOG00000000942		Protein Pan3 [Source:UniProtKB/TrEI	360760	12	11316059	11449762	133704	558,20	-0,10	9,06E-01
ENSRNOG00000001192	**Gltp**	glycolipid transfer protein (Gltp), mRN	288707	12	49460373	49480640	20268	1498,81	0,12	9,06E-01
ENSRNOG00000013465	**Tepp**	testis, prostate and placenta expresse	291850	19	10113530	10121992	8463	24,81	-0,12	9,06E-01
ENSRNOG00000017798		ATPase, Ca++ transporting, plasma m	29599	1	152935958	153056722	120765	890,79	-0,10	9,06E-01
ENSRNOG00000049939		Uncharacterized protein [Source:UniProtKB/TrE		9	11296596	11299073	2478	29,68	-0,12	9,06E-01
ENSRNOG00000019750	**Kcna1**	potassium voltage-gated channel, sha	24520	4	226189075	226190562	1488	66,99	-0,13	9,06E-01
ENSRNOG00000020102	**Sirt2**	sirtuin 2 (Sirt2), mRNA [Source:RefSe	361532	1	88130545	88153084	22540	5932,89	0,11	9,06E-01
ENSRNOG00000001797	**Umps**	uridine monophosphate synthetase (U	288051	11	73123827	73134236	10410	1069,90	0,08	9,06E-01

ENSRNOG00000015498		interleukin 17 receptor B (Il17rb), mRN	306247	16	5995418	6009258	13841	48,40	0,11	9,06E-01
ENSRNOG00000031827	**Arih2**	ariadne homolog 2 (Drosophila) (Arih2	316005	8	116731513	116790857	59345	1657,69	-0,07	9,06E-01
ENSRNOG00000049824		KRI1 homolog (S. cerevisiae) [Source	687183	8	22354134	22368139	14006	584,06	0,06	9,06E-01
ENSRNOG00000003068	**RGD15640**	similar to RIKEN cDNA 3010026O09 (	497895	10	35439501	35463124	23624	408,45	-0,08	9,06E-01
ENSRNOG00000045647		HCLS1-associated protein X-1 [Sourc	291202	2	208762955	208765294	2340	847,39	-0,08	9,06E-01
ENSRNOG00000047005	**Kcnk5**	potassium channel, subfamily K, mem	364241	15	8654578	8696346	41769	30,51	-0,13	9,06E-01
ENSRNOG00000016165	**RGD15619**	Protein RGD1561963 [Source:UniPro	301556	9	86061951	86318505	256555	701,33	-0,15	9,06E-01
ENSRNOG00000001336	**Orai1**	ORAI calcium release-activated calciu	304496	12	40878320	40879082	763	255,49	0,10	9,06E-01
ENSRNOG00000007246	**Atxn7**	Protein Atxn7 [Source:UniProtKB/TrE	361015	15	16456718	16543614	86897	124,15	-0,09	9,06E-01
ENSRNOG00000022455	**Gltpd1**	glycolipid transfer protein domain cont	313771	5	176789844	176793929	4086	524,64	-0,08	9,07E-01
ENSRNOG00000020822		Protein Atp8b2 [Source:UniProtKB/Tr	685152	2	208710296	208729604	19309	726,35	0,10	9,07E-01
ENSRNOG00000001344		aldehyde dehydrogenase, mitochondri	29539	12	42334147	42365988	31842	1684,45	0,08	9,07E-01
ENSRNOG00000002216		steroid 5 alpha-reductase 3 (Srd5a3),	305291	14	34388669	34402835	14167	349,43	0,07	9,07E-01
ENSRNOG00000002940	**Ankrd17**	ankyrin repeat domain 17 (Ankrd17), r	289521	14	19224896	19337844	112949	5053,87	-0,05	9,07E-01
ENSRNOG00000014615	**Grk6**	G protein-coupled receptor kinase 6 ((	59076	17	11815973	11830526	14554	1475,75	0,09	9,07E-01
ENSRNOG00000017719		Uncharacterized protein [Source:UniProtKB/TrE		8	75085452	75088146	2695	480,04	0,08	9,07E-01
ENSRNOG00000037214	**Sgk494**	Protein Sgk494 [Source:UniProtKB/Tr	685208	10	66081541	66084340	2800	50,12	-0,12	9,07E-01
ENSRNOG00000004918	**Kcna4**	potassium voltage-gated channel, sha	25469	3	104901966	104905092	3127	214,90	0,11	9,07E-01
ENSRNOG00000030243	**Lrp1b**	low density lipoprotein receptor-relate	311926	3	30668625	31634018	965394	92,91	0,13	9,07E-01
ENSRNOG00000045735		Uncharacterized protein [Source:UniProtKB/TrE	X		53872613	53876226	3614	24,14	-0,13	9,07E-01
ENSRNOG00000010077	**Smarcd3**	SWI/SNF related, matrix associated, a	296732	4	7089270	7120843	31574	2079,06	0,08	9,07E-01
ENSRNOG00000011573	**Csad**	cysteine sulfinic acid decarboxylase ((	60356	7	141579062	141607052	27991	405,28	-0,10	9,07E-01
ENSRNOG00000011841		microtubule-associated protein 2 (Map	25595	9	73841103	73922677	81575	14227,21	-0,10	9,07E-01
ENSRNOG00000005890		Plasminogen activator inhibitor 1 RNA	246303	4	162813203	162830522	17320	7343,20	-0,08	9,07E-01
ENSRNOG00000018847	**Stx5**	syntaxin 5 (Stx5), mRNA [Source:RefS	65134	1	231876955	231893829	16875	1571,22	0,09	9,07E-01
ENSRNOG00000028319	**Cryz**	crystallin, zeta (quinone reductase) (C	362061	2	279546017	279573472	27456	508,28	0,09	9,07E-01
ENSRNOG00000046834	**C3**	complement component 3 (C3), mRN/	24232	9	8728465	8754412	25948	122,56	0,12	9,07E-01
ENSRNOG00000015520	**Ticrr**	Protein Ticrr [Source:UniProtKB/TrEM	308768	1	142352621	142393849	41229	220,55	0,12	9,08E-01
ENSRNOG00000001048	**Ankrd61**	ankyrin repeat domain 61 (Ankrd61), r	689907	12	14799684	14804040	4357	23,82	-0,15	9,08E-01
ENSRNOG00000001599	**Rwdd2b**	RWD domain containing 2B (Rwdd2b)	304132	11	30711159	30719812	8654	347,35	0,10	9,08E-01
ENSRNOG00000000422	**Dxo**	DOM-3 homolog Z (C. elegans) (Dom3	361799	20	6597106	6599188	2083	287,15	0,11	9,08E-01
ENSRNOG00000010492	**Sp2**	Sp2 transcription factor (Sp2), mRNA		303499	10	84682783	84711848	29066	377,59	0,08	9,08E-01
ENSRNOG00000012455	**Tardbp**	TAR DNA binding protein (Tardbp), mF	298648	5	169088696	169098491	9796	1443,15	-0,11	9,08E-01
ENSRNOG00000002871		RNA binding motif protein 25 [Source	366693	10	9231938	9233329	1392	479,07	-0,10	9,09E-01
ENSRNOG00000046533				19	39162885	39165281	2397	45,60	-0,14	9,09E-01
ENSRNOG00000018914	**Napg**	N-ethylmaleimide-sensitive factor atta	307382	18	57548236	57566987	18752	2276,33	0,13	9,09E-01
ENSRNOG00000004135	**RGD13117**	similar to RIKEN cDNA 1110059G10 (I	301079	8	131184119	131187503	3385	212,07	-0,08	9,09E-01
ENSRNOG00000004407	**LOC688551**			13	94450397	94451625	1229	25,83	0,12	9,09E-01

ENSRNOG00000007164	**Cln6**	ceroid-lipofuscinosis, neuronal 6, late i	315746	8	67461962	67476917	14956	299,00	0,11	9,09E-01
ENSRNOG00000008708	**Pycrl**	pyrroline-5-carboxylate reductase-like	300035	7	116842455	116847953	5499	1368,78	0,09	9,09E-01
ENSRNOG00000011189	**Acy1**	aminoacylase 1 (Acy1), mRNA [Sourc	300981	8	114498901	114503595	4695	429,26	-0,10	9,09E-01
ENSRNOG00000012138				19	43975806	43976912	1107	906,74	0,07	9,09E-01
ENSRNOG00000014288		Fibronectin [Source:UniProtKB/TrEMBL;Acc:F1		9	78674439	78743407	68969	3219,41	-0,11	9,09E-01
ENSRNOG00000019332	**Gtpbp2**	GTP binding protein 2 (Gtpbp2), mRN	363195	9	16095446	16103483	8038	1108,82	0,07	9,09E-01
ENSRNOG00000020941	**Psenen**	presenilin enhancer 2 homolog (C. ele	292788	1	90170911	90172105	1195	1995,36	0,10	9,09E-01
ENSRNOG00000023985	**Fan1**	FANCD2/FANCI-associated nuclease	309256	1	126292302	126318211	25910	303,32	-0,08	9,09E-01
ENSRNOG00000024636	**Gpr85**	G protein-coupled receptor 85 (Gpr85)	64020	4	43716135	43719803	3669	2429,75	-0,12	9,09E-01
ENSRNOG00000027513	**Rtel1**	regulator of telomere elongation helica	362288	3	180531778	180566698	34921	732,90	-0,09	9,09E-01
ENSRNOG00000033545				MT	3761	3831	71	61,50	0,11	9,09E-01
ENSRNOG00000042426	**Cyp4v3**	cytochrome P450, family 4, subfamily	266761	16	49834760	49859697	24938	220,23	-0,13	9,09E-01
ENSRNOG00000010435	**Nupl2**	nucleoporin like 2 (Nupl2), mRNA [Sou	499974	4	7524012	7540636	16625	442,55	-0,08	9,09E-01
ENSRNOG00000019785	**Phtf1**	putative homeodomain transcription fa	252962	2	225878015	225916843	38829	794,96	0,10	9,09E-01
ENSRNOG00000050200		Protein LOC682469 [Source:UniProtK	682469	18	27289264	27342331	53068	2736,84	0,05	9,09E-01
ENSRNOG00000003228	**Mid1ip1**	MID1 interacting protein 1 (Mid1ip1), r	404280	X	13905532	13907710	2179	2866,93	0,10	9,09E-01
ENSRNOG00000046505	**Bend3**	Protein LOC683923; RCG58541, isofo	683923	20	49908063	49926273	18211	134,01	-0,07	9,09E-01
ENSRNOG00000023995	**Ankrd39**	ankyrin repeat domain 39 (Ankrd39), r	367251	9	42773452	42781822	8371	134,80	-0,09	9,09E-01
ENSRNOG00000005332	**Csdc2**	cold shock domain containing C2, RNA	266600	7	123144206	123158716	14511	5565,02	0,08	9,09E-01
ENSRNOG00000017801	**Atf4**	activating transcription factor 4 (Atf4),	79255	7	121468540	121470594	2055	8488,88	-0,07	9,09E-01
ENSRNOG00000000885		autism susceptibility candidate 2 [Sou	1E+08	12	30173028	30212512	39485	739,60	-0,08	9,09E-01
ENSRNOG00000013236	**Samd8**	sterile alpha motif domain containing 8	305684	15	2670863	2712120	41258	1650,39	-0,07	9,09E-01
ENSRNOG00000014693	**Btbd6**	BTB (POZ) domain containing 6 (Btbd	690367	6	146782164	146784616	2453	1180,93	-0,06	9,09E-01
ENSRNOG00000021264	**Pcna**	proliferating cell nuclear antigen (Pcna	25737	3	131376228	131380189	3962	2316,73	-0,08	9,09E-01
ENSRNOG00000049306				6	116131605	116132088	484	25,49	-0,12	9,09E-01
ENSRNOG00000002159	**Agpat9**	1-acylglycerol-3-phosphate O-acyltran	305166	14	10293028	10343730	50703	115,87	-0,13	9,09E-01
ENSRNOG00000016767	**Ggps1**	geranylgeranyl diphosphate synthase	291211	17	51649823	51662765	12943	584,16	-0,11	9,09E-01
ENSRNOG00000047447		Uncharacterized protein [Source:UniProtKB/TrE		4	224392096	224392730	635	35,59	0,11	9,09E-01
ENSRNOG00000014463	**Uri1**	URI1, prefoldin-like chaperone (Uri1),	308537	1	95435846	95493474	57629	1599,97	0,12	9,10E-01
ENSRNOG00000029087	**Nap1l3**	nucleosome assembly protein 1-like 3	170914	X	94947141	94949936	2796	1354,46	0,08	9,10E-01
ENSRNOG00000012470	**Zc3h12c**	zinc finger CCCH type containing 12C	315658	8	55222440	55277181	54742	245,08	-0,10	9,10E-01
ENSRNOG00000006028	**Tubgcp6**	tubulin, gamma complex associated p	362980	7	129766841	129787647	20807	1112,97	-0,06	9,10E-01
ENSRNOG00000012538	**LOC10036**	ribosomal protein L37 (Rpl37), mRNA	81770	2	73857477	73859430	1954	21,76	-0,12	9,10E-01
ENSRNOG00000013752	**Elavl3**	ELAV (embryonic lethal, abnormal visi	282824	8	23085036	23117799	32764	3447,45	0,08	9,10E-01
ENSRNOG00000015679	**Dok4**	docking protein 4 (Dok4), mRNA [Sou	361364	19	10558225	10568895	10671	1768,91	0,11	9,10E-01
ENSRNOG00000017938	**Btaf1**	BTAF1 RNA polymerase II, B-TFIID tra	368042	1	263055333	263130789	75457	816,81	-0,12	9,10E-01
ENSRNOG00000026921	**Rpl32-ps1**			3	124034166	124034675	510	23,30	0,12	9,10E-01
ENSRNOG00000028422	**Rmnd5a**	Protein Rmnd5a [Source:UniProtKB/T	312439	4	164031739	164084227	52489	738,62	-0,09	9,10E-01

ENSRNOG00000000952	**Gsx1**	GS homeobox 1 (Gsx1), mRNA [Sourc	288457	12	11718789	11720084	1296	189,26	-0,14	9,10E-01
ENSRNOG00000012472	**Pyroxd1**	pyridine nucleotide-disulphide oxidore	297708	4	240779516	240795751	16236	100,28	-0,09	9,11E-01
ENSRNOG00000023393	**Nup214**	nucleoporin 214 (Nup214), mRNA [So	296634	3	16351779	16423232	71454	1243,83	0,06	9,11E-01
ENSRNOG00000009333	**Osgep**	O-sialoglycoprotein endopeptidase (O	290028	15	31674874	31682291	7418	600,63	0,07	9,11E-01
ENSRNOG00000008607	**U2surp**	Protein U2surp [Source:UniProtKB/Tr	315903	8	102704851	102757330	52480	541,64	-0,13	9,11E-01
ENSRNOG00000011453	**Kif6**	Protein Kif6 [Source:UniProtKB/TrEM	171291	9	11958961	12245887	286927	24,90	-0,12	9,12E-01
ENSRNOG00000028504	**Socs5**	suppressor of cytokine signaling 5 (So	500616	6	20665943	20696549	30607	1294,76	0,10	9,12E-01
ENSRNOG00000013141	**LOC10091**	enolase 2, gamma, neuronal (Eno2), r	24334	4	224302789	224311633	8845	141,10	0,10	9,12E-01
ENSRNOG00000018273	**Ncl**	nucleolin (Ncl), mRNA [Source:RefSec	25135	9	93097881	93106405	8525	2423,98	0,12	9,12E-01
ENSRNOG00000024578	**Ttyh2**	Protein Ttyh2 [Source:UniProtKB/TrE	287803	10	102865904	102908421	42518	545,73	0,10	9,12E-01
ENSRNOG00000050843	**Mut**	Protein Mut; RCG43751 [Source:UniF	688517	9	22184294	22208962	24669	265,00	-0,08	9,12E-01
ENSRNOG00000042627	**Camta1**	calmodulin binding transcription activa	362665	5	172507291	172577540	70250	106,58	-0,11	9,12E-01
ENSRNOG00000010373		TRIO and F-actin-binding protein [Sou	362956	7	120168221	120222100	53880	1829,49	0,07	9,12E-01
ENSRNOG00000008301	**Tagln2**	transgelin 2 (Tagln2), mRNA [Source:F	304983	13	95407052	95413998	6947	831,64	0,13	9,13E-01
ENSRNOG00000014146		propionyl-CoA carboxylase alpha chai	687008	15	112346187	112687786	341600	295,45	-0,08	9,13E-01
ENSRNOG00000018000	**Ranbp10**	RAN binding protein 10 (Ranbp10), m	361396	19	48600923	48661834	60912	1389,93	-0,08	9,13E-01
ENSRNOG00000017780	**Akr7a2**	aldo-keto reductase family 7, member	171445	5	161499565	161508610	9046	802,99	0,10	9,13E-01
ENSRNOG00000017826		5-methyltetrahydrofolate-homocystein	290947	1	39124771	39155639	30869	297,07	0,11	9,13E-01
ENSRNOG00000023150	**Gpi**	glucose-6-phosphate isomerase (Gpi)	292804	1	91207017	91234890	27874	8938,33	-0,06	9,13E-01
ENSRNOG00000001442	**Por**	P450 (cytochrome) oxidoreductase (P	29441	12	25995663	26044117	48455	2544,52	0,06	9,13E-01
ENSRNOG00000010209	**Nol12**	nucleolar protein 12 (Nol12), mRNA [S	362955	7	120152881	120158542	5662	294,53	0,07	9,13E-01
ENSRNOG00000013391	**Sorbs2**	sorbin and SH3 domain containing 2 (	114901	16	49355585	49545433	189849	2703,98	0,07	9,13E-01
ENSRNOG00000018730	**Pvrl2**	poliovirus receptor-related 2 (Pvrl2), m	308417	1	81896928	81931943	35016	706,54	0,07	9,13E-01
ENSRNOG00000001851		Protein Far2 [Source:UniProtKB/TrEM	500368	4	246666586	246698934	32349	215,50	0,07	9,13E-01
ENSRNOG00000002750		RING CCCH (C3H) domains 1 [Source:MGI Syn		13	83648578	83682162	33585	546,37	-0,07	9,13E-01
ENSRNOG00000002898	**Nme7**	NME/NM23 family member 7 (Nme7),	171566	13	87510979	87621629	110651	251,43	-0,08	9,13E-01
ENSRNOG00000008632	**Invs**	inversin (Invs), mRNA [Source:RefSec	313228	5	68566901	68726568	159668	143,57	-0,09	9,13E-01
ENSRNOG00000011308	**Uhrf2**	ubiquitin-like with PHD and ring finger	309331	1	255476029	255539517	63489	1747,99	-0,11	9,13E-01
ENSRNOG00000013178	**Cmip**	c-Maf-inducing protein (Cmip), mRNA	292051	19	60579252	60790153	210902	6156,14	0,06	9,13E-01
ENSRNOG00000013454	**Rala**	v-ral simian leukemia viral oncogene h	81757	17	47770966	47784077	13112	1528,11	-0,07	9,13E-01
ENSRNOG00000020851		amyloid beta (A4) precursor-like protei	502317	1	90053391	90063141	9751	15352,72	0,07	9,13E-01
ENSRNOG00000022507	**Twf1**	twinfilin actin-binding protein 1 (Twf1),	315265	7	135495110	135507004	11895	468,90	0,10	9,13E-01
ENSRNOG00000029070				MT	7693	7756	64	54,54	0,11	9,13E-01
ENSRNOG00000043480		Uncharacterized protein [Source:UniProtKB/TrE		8	114871505	114872457	953	82,22	-0,12	9,13E-01
ENSRNOG00000050626	**Ube2n**	ubiquitin-conjugating enzyme E2N (Ube2n), mRI		4	22460062	22461311	1250	150,00	0,09	9,13E-01
ENSRNOG00000011134	**Lama2**	Protein Lama2 [Source:UniProtKB/Trl	309368	1	20155194	20597569	442376	48,28	0,10	9,13E-01
ENSRNOG00000013747	**Sh3bp2**	SH3-domain binding protein 2 (Sh3bp:	305450	14	82122933	82160436	37504	325,56	0,08	9,13E-01
ENSRNOG00000018967	**Wdr59**	Protein Wdr59 [Source:UniProtKB/TrE	690751	19	54464897	54523339	58443	633,19	0,09	9,13E-01

ENSRNOG00000020751	**Rdm1**	RAD52 motif 1 (Rdm1), mRNA [Sourc	287726	10	89337811	89348066	10256	77,27	0,12	9,13E-01
ENSRNOG00000024760	**B4galnt4**	beta-1,4-N-acetyl-galactosaminyl trans	309105	1	220789704	220800586	10883	2277,36	0,08	9,13E-01
ENSRNOG00000029301				MT	3695	3763	69	18,03	-0,13	9,13E-01
ENSRNOG00000003038	**Sft2d2**	SFT2 domain containing 2 (Sft2d2), m	360868	13	88322945	88331583	8639	101,42	-0,13	9,13E-01
ENSRNOG00000005310	**Cul1**	cullin 1 (Cul1), mRNA [Source:RefSeq	362356	4	141883675	141951628	67954	2268,24	-0,10	9,13E-01
ENSRNOG00000008246	**Emilin1**	elastin microfibril interfacer 1 (Emilin1)	298845	6	36636870	36644587	7718	476,41	-0,09	9,13E-01
ENSRNOG00000015253	**Hsf4**	heat shock transcription factor 4 (Hsf4	291960	19	48091910	48097634	5725	32,30	0,12	9,14E-01
ENSRNOG00000016831	**Serpinh1**	serpin peptidase inhibitor, clade H (he	29345	1	170503596	170510775	7180	4236,46	-0,11	9,14E-01
ENSRNOG00000022015	**Cntrl**	Protein Cntrl [Source:UniProtKB/TrEM	311886	3	19623108	19689507	66400	245,96	0,11	9,14E-01
ENSRNOG00000001845	**Top3b**	topoisomerase (DNA) III beta (Top3b),	287930	11	91401397	91429763	28367	865,86	0,06	9,14E-01
ENSRNOG00000001115	**Slc29a4**	solute carrier family 29 (equilibrative n	288499	12	15942167	15952584	10418	3532,26	-0,10	9,14E-01
ENSRNOG00000005306	**Lmbr1**	limb development membrane protein 1	362295	4	2172459	2330391	157933	1336,64	-0,09	9,14E-01
ENSRNOG00000006689		chromodomain helicase DNA binding	312974	5	26581851	26700143	118293	1762,44	-0,07	9,14E-01
ENSRNOG00000007755	**Pm20d2**	peptidase M20 domain containing 2 (F	313130	5	53002968	53019197	16230	228,96	0,08	9,14E-01
ENSRNOG00000007862	**Acat1**	acetyl-CoA acetyltransferase 1 (Acat1	25014	8	56750515	56778323	27809	3757,10	0,10	9,14E-01
ENSRNOG00000008673	**Arpc3**	actin related protein 2/3 complex, subu	288669	12	41533346	41548335	14990	4417,72	-0,08	9,14E-01
ENSRNOG00000009303		zwilch kinetochore protein [Source:MGI Symbol;		8	68798171	68827407	29237	239,69	0,12	9,14E-01
ENSRNOG00000012561		Protein RGD1565043 [Source:UniPro	306618	16	79303507	79377402	73896	523,32	-0,08	9,14E-01
ENSRNOG00000012593	**Mmel1**	membrane metallo-endopeptidase-like	313755	5	175729188	175759867	30680	19,78	-0,12	9,14E-01
ENSRNOG00000012655		a disintegrin-like and metallopeptidase	361886	2	53503157	53713513	210357	45,36	-0,10	9,14E-01
ENSRNOG00000013579		Protein Gm7964 [Source:UniProtKB/T	1E+08	5	64434354	64435904	1551	226,66	-0,08	9,14E-01
ENSRNOG00000013862	**Dusp2**	dual specificity phosphatase 2 (Dusp2	311406	3	126303199	126305373	2175	212,80	-0,08	9,14E-01
ENSRNOG00000015401	**Mapk4**	mitogen-activated protein kinase 4 (Ma	54268	18	69040280	69087394	47115	455,49	0,09	9,14E-01
ENSRNOG00000017962	**Serpinb6**	serpin peptidase inhibitor, clade B (ova	291085	17	34084123	34102912	18790	123,96	-0,11	9,14E-01
ENSRNOG00000018021	**Brix1**	BRX1, biogenesis of ribosomes, homo	294799	2	84212803	84223679	10877	457,53	-0,13	9,14E-01
ENSRNOG00000047309	**LOC10036**	RIKEN cDNA 2700089E24 gene [Sou	1E+08	4	229747953	229749357	1405	1433,13	0,07	9,14E-01
ENSRNOG00000002423		family with sequence similarity 114, member A2		10	42702545	42733587	31043	638,77	0,07	9,15E-01
ENSRNOG00000005175	**Sgpp1**	Sphingosine-1-phosphate phosphatas	81536	6	108094887	108114210	19324	509,76	0,09	9,15E-01
ENSRNOG00000010601	**Srpk2**	SRSF protein kinase 2 (Srpk2), mRNA	296753	4	8073530	8191643	118114	3427,16	-0,07	9,15E-01
ENSRNOG00000011274		cadherin-8 precursor [Source:RefSeq	84408	19	6024414	6417023	392610	871,27	-0,11	9,15E-01
ENSRNOG00000011756	**Phf3**	PHD finger protein 3 (Phf3), mRNA [S	363210	9	35878437	35948938	70502	2136,53	0,06	9,15E-01
ENSRNOG00000012550	**Uqcrh**	ubiquinol-cytochrome c reductase hing	366448	5	138702848	138711012	8165	2452,48	0,09	9,15E-01
ENSRNOG00000020279	**Syt11**	synaptotagmin XI (Syt11), mRNA [Sou	60568	2	207513048	207537284	24237	15833,10	0,08	9,15E-01
ENSRNOG00000020468	**Stard4**	StAR-related lipid transfer (START) do	291699	18	25698888	25712288	13401	417,58	-0,13	9,15E-01
ENSRNOG00000023661	**Celf2**	CUGBP, Elav-like family member 2 (C	29428	17	76914823	77312284	397462	4546,69	0,07	9,15E-01
ENSRNOG00000042932	**Ttc27**	tetratricopeptide repeat domain 27 (Tt	298782	6	31624976	31768786	143811	546,80	0,06	9,15E-01
ENSRNOG00000046769	**Mms19l**	MMS19-like (MET18 homolog, S. cere	171124	1	268644120	268681496	37377	987,34	0,05	9,15E-01
ENSRNOG00000011193	**Hmgcll1**	3-hydroxymethyl-3-methylglutaryl-CoA	367112	8	82645962	82780148	134187	309,89	-0,10	9,15E-01

ENSRNOG00000012728	**Nkx2-2**	NK2 homeobox 2 (Nkx2-2), mRNA [Sc	366214	3	147827035	147829407	2373	221,79	-0,12	9,15E-01
ENSRNOG00000018225		tumor protein p53 inducible nuclear pr	362246	3	157278565	157283521	4957	1202,17	-0,11	9,15E-01
ENSRNOG00000022030	**Aunip**	Aurora kinase A and ninein-interacting	689656	5	156507651	156520014	12364	55,30	0,09	9,15E-01
ENSRNOG00000023129	**Mfsd9**	major facilitator superfamily domain cc	316356	9	47180040	47199516	19477	591,08	0,10	9,15E-01
ENSRNOG00000024335	**Fastkd1**	FAST kinase domains 1 (Fastkd1), mF	311112	3	62537420	62563261	25842	308,85	-0,10	9,15E-01
ENSRNOG00000028594		interferon (alpha, beta and omega) rec	288264	11	35248575	35272229	23655	3980,58	0,10	9,15E-01
ENSRNOG00000031801	**Ephb3**	Eph receptor B3 (Ephb3), mRNA [Sou	287989	11	86602390	86621102	18713	668,13	-0,10	9,15E-01
ENSRNOG00000032825	**LOC100910870**			10	37797860	37798207	348	458,52	-0,08	9,15E-01
ENSRNOG00000002731		Uncharacterized protein [Source:UniProtKB/TrE		13	83228271	83229277	1007	20,15	0,13	9,15E-01
ENSRNOG00000004659	**Creld2**	cysteine-rich with EGF-like domains 2	362978	7	129501501	129508346	6846	570,59	-0,07	9,15E-01
ENSRNOG00000007797	**Rbpjl2**	Protein Rbpsuh [Source:UniProtKB/Tr	297767	5	4846867	4848587	1721	21,38	-0,13	9,15E-01
ENSRNOG00000011624		potassium voltage-gated channel subf	170848	3	180299959	180354983	55025	3035,71	0,09	9,15E-01
ENSRNOG00000011745	**Psma1**	proteasome (prosome, macropain) sul	29668	1	190708745	190719764	11020	1871,77	0,11	9,15E-01
ENSRNOG00000012512		nexilin (F actin binding protein) (Nexn)	246172	2	276130293	276151480	21188	64,86	0,13	9,15E-01
ENSRNOG00000013313	**Nceh1**	neutral cholesterol ester hydrolase 1 (	294930	2	132589874	132649579	59706	718,97	0,09	9,15E-01
ENSRNOG00000014373	**Trim66**	tripartite motif-containing 66 (Trim66),	361623	1	181055797	181108418	52622	111,65	0,13	9,15E-01
ENSRNOG00000015038	**Adam10**	ADAM metallopeptidase domain 10 (A	29650	8	75843846	75915593	71748	1226,89	-0,10	9,15E-01
ENSRNOG00000016299	**Klf4**	Kruppel-like factor 4 (gut) (Klf4), mRN	114505	5	76447645	76452001	4357	58,93	-0,12	9,15E-01
ENSRNOG00000017318	**Zfp322a**	zinc finger protein 322a (Zfp322a), mF	680201	17	45878662	45892679	14018	218,21	-0,10	9,15E-01
ENSRNOG00000021047	**Lrrfip2**	leucine rich repeat (in FLII) interacting	301035	8	118723098	118824560	101463	586,38	0,06	9,15E-01
ENSRNOG00000025181	**Pigu**	phosphatidylinositol glycan anchor bio	353304	3	157171342	157253270	81929	1030,76	0,08	9,15E-01
ENSRNOG00000025497	**Fbxl6**	F-box and leucine-rich repeat protein 6	362941	7	117589688	117592585	2898	600,10	0,09	9,15E-01
ENSRNOG00000028783	**Wdr35**	WD repeat domain 35-like (Wdr35l), m	503018	6	43875471	43932450	56980	678,53	-0,11	9,15E-01
ENSRNOG00000029592	**Srsf11**	serine/arginine-rich splicing factor 11 (	502603	2	283477397	283496845	19449	2497,09	0,08	9,15E-01
ENSRNOG00000043451	**Spp1**	secreted phosphoprotein 1 (Spp1), mF	25353	14	6653093	6658953	5861	58,86	-0,14	9,15E-01
ENSRNOG00000046168	**Ppm1l**	protein phosphatase, Mg2+/Mn2+ dep	310506	2	185498460	185567985	69526	254,99	0,06	9,15E-01
ENSRNOG00000049689		small integral membrane protein 18 [Source:MG		16	61816367	61816654	288	193,47	0,10	9,15E-01
ENSRNOG00000009985	**Tdrd7**	tudor domain containing 7 (Tdrd7), mF	85425	5	66079898	66147306	67409	1154,87	0,06	9,15E-01
ENSRNOG00000014277	**Zdhhc13**	zinc finger, DHHC-type containing 13 (	365252	1	105165499	105204298	38800	581,75	-0,10	9,15E-01
ENSRNOG00000020915	**Setd2**	SET domain containing 2 (Setd2), mR	316013	8	118163197	118229411	66215	2281,09	0,09	9,15E-01
ENSRNOG00000003759	**Galc**	galactosylceramidase (Galc), mRNA [S	314360	6	131392303	131454533	62231	406,38	0,10	9,15E-01
ENSRNOG00000032395	**Tfcp2**	transcription factor CP2 (Tfcp2), mRN	315309	7	139946315	139988265	41951	847,15	0,06	9,15E-01
ENSRNOG00000011964	**Abcd4**	ATP-binding cassette, subfamily D (AL	299196	6	117216244	117231218	14975	224,30	-0,07	9,15E-01
ENSRNOG00000014879	**Ttc7a**	tetratricopeptide repeat domain 7A (Tt	362696	6	20900469	21002055	101587	220,47	0,07	9,16E-01
ENSRNOG00000003706	**Ufc1**	ubiquitin-fold modifier conjugating enz	445268	13	94288628	94295639	7012	1537,21	-0,05	9,16E-01
ENSRNOG00000004616	**Npm1**	nucleophosmin (nucleolar phosphoprc	25498	10	17965978	17975978	10001	1581,99	-0,13	9,16E-01
ENSRNOG00000006888	**Gcfc2**	GC-rich sequence DNA-binding factor	312474	4	177350363	177387465	37103	211,30	-0,10	9,16E-01
ENSRNOG00000012338	**Ddx11**	Protein Ddx11 [Source:UniProtKB/TrE	316767	9	113624115	113648909	24795	110,09	0,08	9,16E-01

ENSRNOG00000018927	**Sprn**	shadow of prion protein homolog (zebi	541462	1	219536513	219536956	444	207,41	0,09	9,16E-01
ENSRNOG00000026415		collagen alpha-1(XIV) chain precursor	314981	7	95678308	95895351	217044	63,46	-0,12	9,16E-01
ENSRNOG00000048509	**Ubxn6**	UBX domain protein 6 (Ubxn6), mRNA	363332	9	9982495	9987589	5095	1528,14	0,07	9,16E-01
ENSRNOG00000050490				10	94727751	94727817	67	40,55	0,14	9,16E-01
ENSRNOG00000001859	**Sdf2l1**	stromal cell-derived factor 2-like 1 (Sd	680945	11	91174846	91177088	2243	396,16	-0,13	9,16E-01
ENSRNOG00000002757	**Tada2a**	transcriptional adaptor 2A (Tada2a), m	360581	10	71307415	71354773	47359	760,32	-0,09	9,16E-01
ENSRNOG00000003464	**Hid1**	Protein Hid1 [Source:UniProtKB/TrEN	287822	10	104372280	104391897	19618	2050,82	0,06	9,16E-01
ENSRNOG00000004464	**Sel1l**	sel-1 suppressor of lin-12-like (C. eleg	314352	6	124826559	124868279	41721	3074,86	0,08	9,16E-01
ENSRNOG00000004967	**Snrpb2**	small nuclear ribonucleoprotein polype	362223	3	143580422	143589939	9518	658,47	-0,09	9,16E-01
ENSRNOG00000007739	**Rars**	arginyl-tRNA synthetase (Rars), mRNA	287191	10	20505808	20530334	24527	702,53	-0,09	9,16E-01
ENSRNOG00000011636	**Dennd6a**	family with sequence similarity 116, m	306229	16	2252208	2302165	49958	1087,84	-0,10	9,16E-01
ENSRNOG00000012394	**Bcl2l13**	BCL2-like 13 (apoptosis facilitator) (Bc	312682	4	220475338	220525622	50285	534,98	0,06	9,16E-01
ENSRNOG00000012440		methionine sulfoxide reductase A (Msr	29447	15	51325823	51550758	224936	274,64	-0,07	9,16E-01
ENSRNOG00000013832	**Fam193a**	Protein Fam193a [Source:UniProtKB/	305452	14	82204090	82286026	81937	1185,19	-0,06	9,16E-01
ENSRNOG00000013934	**St5**	suppression of tumorigenicity 5 (St5),	308944	1	181125790	181275226	149437	1294,84	-0,08	9,16E-01
ENSRNOG00000014791		paternally expressed 3 [Source:MGI Symbol;Acc		1	71615728	71626512	10785	5002,30	-0,09	9,16E-01
ENSRNOG00000016033	**Endog**	endonuclease G (Endog), mRNA [Sou	362100	3	14094225	14096950	2726	191,42	0,11	9,16E-01
ENSRNOG00000018149	**Smarca5**	SWI/SNF related, matrix associated, a	307766	19	41841149	41873900	32752	690,83	0,10	9,16E-01
ENSRNOG00000018152	**Ptprh**	protein tyrosine phosphatase, recepto	171125	1	75692060	75720081	28022	54,91	-0,10	9,16E-01
ENSRNOG00000019139	**Ctnnb1**	catenin (cadherin associated protein),	84353	8	128798559	128825828	27270	21801,08	-0,08	9,16E-01
ENSRNOG00000026059	**Paqr6**	progestin and adipoQ receptor family i	681021	2	207140710	207144108	3399	29,66	-0,14	9,16E-01
ENSRNOG00000029366		proline-rich transmembrane protein 2 (	361651	1	205431761	205435296	3536	1639,83	-0,08	9,16E-01
ENSRNOG00000032578				MT	11606	11665	60	72,59	-0,10	9,16E-01
ENSRNOG00000038184	**Camk2n2**	calcium/calmodulin-dependent protein	59314	11	87047758	87048885	1128	600,38	0,08	9,16E-01
ENSRNOG00000042915		matrix-remodelling associated 7 [Sour	690599	10	105406417	105430525	24109	138,72	-0,11	9,16E-01
ENSRNOG00000007076		zinc finger protein 101 [Source:MGI Sy	691422	7	16020784	16036263	15480	99,09	-0,12	9,16E-01
ENSRNOG00000015458	**Hemk1**	HemK methyltransferase family memb	300989	8	115438339	115448894	10556	244,25	-0,07	9,16E-01
ENSRNOG00000025075	**Relt**	RELT tumor necrosis factor receptor (I	361615	1	172081904	172089124	7221	127,91	0,11	9,16E-01
ENSRNOG00000027919	**Rdh13**	retinol dehydrogenase 13 (all-trans/9-(	361504	1	75563122	75590916	27795	395,19	-0,08	9,16E-01
ENSRNOG00000043436	**Micu1**	mitochondrial calcium uptake 1 (Micu1	365567	20	31089817	31235816	146000	3135,87	-0,07	9,16E-01
ENSRNOG00000031233	**Mapk12**	mitogen-activated protein kinase 12 (M	60352	7	129795269	129805662	10394	93,21	0,13	9,16E-01
ENSRNOG00000006718		RNA binding motif protein 33 [Source:	362297	4	587764	688265	100502	1263,13	0,09	9,16E-01
ENSRNOG00000019000	**Limk2**	LIM domain kinase 2 (Limk2), mRNA [	29524	14	84262200	84330097	67898	2858,48	-0,06	9,16E-01
ENSRNOG00000006992	**Rpl7**	ribosomal protein L7 (Rpl7), mRNA [S	297755	5	2621855	2624860	3006	3443,62	0,12	9,16E-01
ENSRNOG00000004131	**Pkn1**	protein kinase N1 (Pkn1), mRNA [Sou	29355	19	35782679	35810655	27977	1634,14	0,06	9,17E-01
ENSRNOG00000011582	**Rab3d**	RAB3D, member RAS oncogene famil	140665	8	22975024	22984052	9029	658,63	-0,08	9,17E-01
ENSRNOG00000013756	**Snrnp48**	small nuclear ribonucleoprotein 48 (U1	291060	17	29159013	29194311	35299	266,86	-0,09	9,17E-01
ENSRNOG00000030911		Uncharacterized protein [Source:UniProtKB/TrE		6	62083390	62181811	98422	466,07	0,06	9,17E-01

ENSRNOG00000048705				17	77009722	77009785	64	32,19	-0,12	9,17E-01
ENSRNOG00000005228	**Gnptab**	Protein Gnptab [Source:UniProtKB/Tr	362865	7	29063508	29128693	65186	1895,27	0,08	9,17E-01
ENSRNOG00000013694	**Ntng2**	netrin G2 (Ntng2), mRNA [Source:Ref	311836	3	13091083	13144474	53392	1158,28	0,07	9,17E-01
ENSRNOG00000017311	**Me3**	malic enzyme 3, NADP(+)-dependent,	361602	1	160166289	160360861	194573	100,45	-0,12	9,17E-01
ENSRNOG00000009117	**Otub2**	OTU domain, ubiquitin aldehyde bindi	314405	6	136503051	136521614	18564	525,17	0,08	9,17E-01
ENSRNOG00000000466	**Hsd17b8**	hydroxysteroid (17-beta) dehydrogena	361802	20	5896544	5898539	1996	665,56	-0,10	9,17E-01
ENSRNOG00000000609	**Ipmk**	inositol polyphosphate multikinase (Ip	171458	20	20623399	20655922	32524	1006,44	-0,07	9,17E-01
ENSRNOG00000002051		synaptojanin-1 [Source:RefSeq peptic	85238	11	34717258	34791435	74178	4472,72	-0,06	9,17E-01
ENSRNOG00000004556	**Dcaf5**	DDB1 and CUL4 associated factor 5 (	314273	6	111691590	111780260	88671	2524,00	-0,06	9,17E-01
ENSRNOG00000007570	**Dera**	Protein Dera [Source:UniProtKB/TrEM	690945	4	236026964	236082651	55688	291,22	-0,10	9,17E-01
ENSRNOG00000007748	**Tex15**	testis expressed 15 (Tex15), mRNA [S	290803	16	61973992	61988059	14068	40,28	0,14	9,17E-01
ENSRNOG00000009433	**Ccdc109b**	Protein Ccdc109b [Source:UniProtKB	295462	2	249552961	249573851	20891	17,82	-0,13	9,17E-01
ENSRNOG00000009963	**Ctps1**	CTP synthase 1 (Ctps1), mRNA [Sour	313560	5	143259189	143288320	29132	2107,31	-0,10	9,17E-01
ENSRNOG00000018958	**Mt3**	metallothionein 3 (Mt3), mRNA [Sourc	117038	19	11300015	11301422	1408	15115,60	-0,12	9,17E-01
ENSRNOG00000022144	**LOC10036**	Protein LOC100361993 [Source:UniP	1E+08	13	112209407	112210163	757	204,52	0,09	9,17E-01
ENSRNOG00000036667	**Hexdc**	hexosaminidase (glycosyl hydrolase fa	1E+08	10	109993108	110011700	18593	764,04	0,08	9,17E-01
ENSRNOG00000046053	**Nudt11**	Protein LOC100912928; Protein Nudt	367747	X	17951888	17952382	495	29,14	-0,12	9,17E-01
ENSRNOG00000049912	**Atp5e**	ATP synthase, H+ transporting, mitoch	245958	3	178609114	178612055	2942	5989,78	0,12	9,17E-01
ENSRNOG00000008187	**Ubash3b**	ubiquitin associated and SH3 domain	315579	8	43705720	43853558	147839	713,15	-0,07	9,17E-01
ENSRNOG00000011944	**Snx10**	sorting nexin 10 (Snx10), mRNA [Sour	297096	4	145977454	146040122	62669	2302,66	0,06	9,17E-01
ENSRNOG00000017557	**LOC10036**	RIKEN cDNA 2810459M11 gene [Sou	1E+08	9	92791692	92797922	6231	192,45	-0,11	9,17E-01
ENSRNOG00000025679	**Stk40**	serine/threonine kinase 40 (Stk40), m	360230	5	147883548	147920182	36635	1649,00	0,07	9,17E-01
ENSRNOG00000037673	**RGD15651**	Protein RGD1565183 [Source:UniPro	1E+08	1	85803624	85804087	464	49,92	0,13	9,17E-01
ENSRNOG00000016465		tRNA 5-methylaminomethyl-2-thiourid	362976	7	126467263	126483589	16327	330,47	0,08	9,18E-01
ENSRNOG00000022893	**Rimbp2**	RIMS binding protein 2 (Rimbp2), mR	266780	12	33319996	33529398	209403	1754,22	-0,08	9,18E-01
ENSRNOG00000008278	**Nfrkb**	nuclear factor related to kappa B bindi	315523	8	32555212	32586755	31544	1266,15	0,08	9,18E-01
ENSRNOG00000047966	**Hspa1l**	heat shock protein 1-like (Hspa1l), mR	24963	20	7035968	7038223	2256	53,06	0,11	9,18E-01
ENSRNOG00000004226	**Irak3**	interleukin-1 receptor-associated kinas	314870	7	65142553	65201088	58536	38,73	0,13	9,18E-01
ENSRNOG00000004740	**Mmadhc**	methylmalonic aciduria (cobalamin de	362134	3	40897290	40915185	17896	689,16	-0,08	9,18E-01
ENSRNOG00000024364	**Enkd1**	enkurin domain containing 1 (Enkd1),	291975	19	48537328	48541781	4454	166,46	-0,10	9,18E-01
ENSRNOG00000042140	**Eomes**	Protein Eomes [Source:UniProtKB/Tr	316052	8	126369794	126374108	4315	17,76	0,13	9,18E-01
ENSRNOG00000019426	**Fam103a1**	family with sequence similarity 103, m	293058	1	144527225	144533106	5882	1122,92	-0,07	9,19E-01
ENSRNOG00000029178	**Abcc5**	ATP-binding cassette, subfamily C (C	116721	11	87460863	87554517	93655	1423,54	0,11	9,19E-01
ENSRNOG00000013623				15	44476884	44478899	2016	514,14	-0,07	9,19E-01
ENSRNOG00000004789	**Diexf**	digestive organ expansion factor hom	305076	13	116383169	116404439	21271	355,83	-0,07	9,19E-01
ENSRNOG00000007315	**Thoc2**	THO complex 2 (Thoc2), mRNA [Sour	313308	X	128246550	128361038	114489	768,07	-0,12	9,19E-01
ENSRNOG00000009575	**Ubiad1**	UbiA prenyltransferase domain contai	313706	5	168904231	168915829	11599	716,10	0,06	9,19E-01
ENSRNOG00000010205	**MGC95152**	similar to B230212L03Rik protein (MG	297109	4	149514309	149531680	17372	3009,51	0,09	9,19E-01

ENSRNOG00000012582	**Eif4ebp1**	eukaryotic translation initiation factor 4	116636	16	68580280	68593651	13372	769,40	-0,13	9,19E-01
ENSRNOG00000018400		golgi membrane protein 1 [Source:MG	680692	17	7606702	7633706	27005	370,52	0,08	9,19E-01
ENSRNOG00000019957	**Lrig2**	leucine-rich repeats and immunoglobu	310753	2	226353985	226419472	65488	806,79	-0,10	9,19E-01
ENSRNOG00000036729	**LOC69046**	ribosomal protein L38 (Rpl38), mRNA	682793	10	102858164	102861799	3636	1119,70	0,09	9,19E-01
ENSRNOG00000008767	**Atg4c**	autophagy related 4C, cysteine peptid	313391	5	121820649	121868749	48101	468,79	-0,11	9,19E-01
ENSRNOG00000024071	**Mtcp1**	Protein Mtcp1 [Source:UniProtKB/TrE	498814	18	456454	457099	646	91,01	0,11	9,19E-01
ENSRNOG00000003387	**Fdxr**	ferredoxin reductase (Fdxr), mRNA [S	79122	10	104468583	104477286	8704	339,40	0,08	9,19E-01
ENSRNOG00000011448	**Eri1**	exoribonuclease 1 (Eri1), mRNA [Sour	361159	16	59982385	60002473	20089	354,75	-0,10	9,19E-01
ENSRNOG00000011725		ubiquitin-associated protein 2 [Source	313169	5	62064695	62156277	91583	2464,27	-0,07	9,19E-01
ENSRNOG00000013659	**Tex2**	Protein Tex2; Testis expressed gene 2	303611	10	94446007	94508324	62318	1718,13	-0,07	9,19E-01
ENSRNOG00000014013	**Map4k4**	mitogen-activated protein kinase kinas	301363	9	46343468	46467917	124450	16618,90	-0,05	9,19E-01
ENSRNOG00000015011	**Fam189a2**	RGD1307524 protein [Source:UniPro	309415	1	249102133	249155546	53414	393,44	0,09	9,19E-01
ENSRNOG00000021015		suprabasin (Sbsn), transcript variant 2	292793	1	90357212	90361688	4477	54,27	0,09	9,19E-01
ENSRNOG00000024530		LYR motif-containing protein 1 [Sourc	365361	1	196605007	196652808	47802	325,77	0,09	9,19E-01
ENSRNOG00000025895	**Sdpr**	serum deprivation response (Sdpr), m	316384	9	54940738	54952755	12018	47,79	-0,11	9,19E-01
ENSRNOG00000026407	**Fam184a**	Protein Fam184a-ps1 [Source:UniPrc	361853	20	36692972	36756728	63757	379,71	-0,07	9,19E-01
ENSRNOG00000028386		predicted gene 867 [Source:MGI Symbol;Acc:M		20	15817930	15820632	2703	343,27	-0,12	9,19E-01
ENSRNOG00000042041	**Gal3st1**	galactose-3-O-sulfotransferase 1 (Gal	683713	14	84915364	84929663	14300	150,12	0,13	9,19E-01
ENSRNOG00000038190	**Dok6**	docking protein 6 (Dok6), mRNA [Sou	498898	18	85459997	85911591	451595	211,46	-0,12	9,19E-01
ENSRNOG00000010903	**Crb1**	crumbs homolog 1 (Drosophila) (Crb1	304825	13	61292273	61480872	188600	20,27	-0,12	9,19E-01
ENSRNOG00000020894	**Lsm12**	LSM12 homolog (S. cerevisiae) (Lsm1	287731	10	89892925	89914903	21979	2814,91	0,05	9,19E-01
ENSRNOG00000022598	**Trerf1**	transcriptional regulating factor 1 (Trer	316219	9	14576728	14621302	44575	356,77	-0,07	9,19E-01
ENSRNOG00000015455	**Spr**	sepiapterin reductase (7,8-dihydrobiop	29270	4	181492883	181496612	3730	180,85	0,10	9,19E-01
ENSRNOG00000025157	**LOC31017**	Protein LOC310177 [Source:UniProtK	310177	2	97504433	97505107	675	19,78	0,13	9,20E-01
ENSRNOG00000010037		erythrocyte protein band 4.1 [Source:M	313052	5	153762345	153894181	131837	1731,39	-0,10	9,20E-01
ENSRNOG00000005975	**LOC10036**	ribosomal protein L30 (Rpl30), mRNA	64640	7	73381072	73383965	2894	380,32	0,12	9,20E-01
ENSRNOG00000007241	**Fnbp4**	formin binding protein 4 (Fnbp4), mRN	311183	3	86277504	86317615	40112	1309,39	0,08	9,20E-01
ENSRNOG00000014865		Uncharacterized protein [Source:UniProtKB/TrE		10	58525071	58525478	408	80,45	0,09	9,20E-01
ENSRNOG00000015848	**Lyrm5**	Protein Lyrm5; RCG29836, isoform CI	1E+08	4	243658583	243663698	5116	195,58	-0,09	9,20E-01
ENSRNOG00000007392	**Atp6v1f**	ATPase, H transporting, lysosomal V1	116664	4	56511722	56514817	3096	3825,70	0,07	9,20E-01
ENSRNOG00000016707	**Casp2**	caspase 2 (Casp2), mRNA [Source:Re	64314	4	136454793	136474624	19832	1016,61	0,10	9,20E-01
ENSRNOG00000015476	**Ubxn11**	UBX domain protein 11 (Ubxn11), mRI	192207	5	156019009	156042873	23865	304,08	-0,09	9,20E-01
ENSRNOG00000034126	**Pcdhga2**	protocadherin gamma subfamily A, 2 (	498846	18	30509184	30511774	2591	833,78	-0,07	9,20E-01
ENSRNOG00000014598	**Abhd13**	abhydrolase domain containing 13 (Ab	306630	16	84756698	84769135	12438	575,41	-0,07	9,20E-01
ENSRNOG00000004956	**Phf15**	PHD finger protein 15 (Phf15), mRNA	303113	10	37043431	37084794	41364	113,56	0,10	9,20E-01
ENSRNOG00000001141		serine/arginine repetitive matrix 4 [Source:MGI S		12	47542290	47695044	152755	551,13	0,07	9,20E-01
ENSRNOG00000002134		Protein LOC498435 [Source:UniProtK	305139	14	5910568	5927152	16585	45,04	-0,13	9,20E-01
ENSRNOG00000002514	**Ccdc93**	coiled-coil domain containing 93 (Ccd	304743	13	42558446	42626672	68227	367,69	0,07	9,20E-01

ENSRNOG00000005424	**Odc1**	ornithine decarboxylase 1 (Odc1), mR	24609	6	52565188	52571771	6584	4398,83	0,08	9,20E-01
ENSRNOG00000006958	**Zfp12**	Protein Zfp12 [Source:UniProtKB/TrE	288486	12	15413267	15423624	10358	440,57	0,08	9,20E-01
ENSRNOG00000007046	**Ttc18**	Protein Ttc18 [Source:UniProtKB/TrE	361006	15	8389847	8452066	62220	100,14	-0,09	9,20E-01
ENSRNOG00000009588		Syntaphilin [Source:UniProtKB/Swiss	296267	3	153457050	153496985	39936	1755,95	0,08	9,20E-01
ENSRNOG00000017365	**Klhl35**	Protein Klhl35 [Source:UniProtKB/TrE	308850	1	170627335	170632794	5460	103,13	-0,10	9,20E-01
ENSRNOG00000018972	**Rab18**	RAB18, member RAS oncogene famil	307039	17	61632853	61664236	31384	1178,06	-0,05	9,20E-01
ENSRNOG00000019219	**Vamp1**	vesicle-associated membrane protein	25624	4	224743851	224750439	6589	339,54	-0,09	9,20E-01
ENSRNOG00000020497	**Plekha1**	pleckstrin homology domain containin	361659	1	208462231	208513823	51593	1619,44	-0,10	9,20E-01
ENSRNOG00000024737	**Tnrc6a**	trinucleotide repeat containing 6a (Tnr	308971	1	200136262	200204255	67994	1532,70	-0,06	9,20E-01
ENSRNOG00000025948	**RGD13118**	uncharacterized protein LOC289399		289399	13	58140417	58160691	20275	92,58	-0,11	9,20E-01
ENSRNOG00000031814	**Sort1**	sortilin 1 (Sort1), mRNA [Source:RefS	83576	2	230547671	230637210	89540	2220,46	0,06	9,20E-01
ENSRNOG00000042289	**Plcxd2**	phosphatidylinositol-specific phosphol	363781	11	60371804	60423719	51916	2298,58	-0,11	9,20E-01
ENSRNOG00000042340	**LOC67989**	cDNA sequence BC005561 [Source:M	679894	14	6561520	6566289	4770	104,09	-0,12	9,20E-01
ENSRNOG00000046486	**Usp4**	ubiquitin specific peptidase 4 (proto-or	290864	8	116474847	116518209	43363	2633,00	0,06	9,20E-01
ENSRNOG00000050814				9	81627948	81628282	335	38,66	0,10	9,20E-01
ENSRNOG00000000531	**Ccdc167**	coiled-coil domain containing 167 (Cc	689755	20	10569147	10583904	14758	96,00	-0,09	9,21E-01
ENSRNOG00000000571	**Psap**	prosaposin (Psap), transcript variant 2	25524	20	31629576	31668604	39029	42816,76	-0,09	9,21E-01
ENSRNOG00000001825		Protein Pkp2 [Source:UniProtKB/TrEMBL;Acc:F		11	91966047	92013360	47314	28,24	-0,10	9,21E-01
ENSRNOG00000002852	**Myo19**	myosin XIX (Myo19), mRNA [Source:F	497974	10	72105466	72134751	29286	286,67	-0,09	9,21E-01
ENSRNOG00000004179	**Nts**	neurotensin (Nts), mRNA [Source:Ref	299757	7	44140236	44150124	9889	319,25	-0,09	9,21E-01
ENSRNOG00000006661	**Rad51c**	RAD51 paralog C (Rad51c), mRNA [S	497976	10	75376954	75403188	26235	88,50	-0,09	9,21E-01
ENSRNOG00000008663		N-acetyltransferase 10 (GCN5-related	311257	3	100298714	100338733	40020	725,29	0,06	9,21E-01
ENSRNOG00000009983	**Lrrc42**	leucine rich repeat containing 42 (Lrrc	298309	5	130684407	130705517	21111	1708,93	0,05	9,21E-01
ENSRNOG00000011119	**LOC10036**	spindlin 1 (Spin1), mRNA [Source:Ref	361217	16	879502	925738	46237	99,46	-0,09	9,21E-01
ENSRNOG00000012001	**Vps37a**	vacuolar protein sorting 37 homolog A	290775	16	54564705	54602075	37371	477,85	-0,06	9,21E-01
ENSRNOG00000015459	**Heatr3**	Protein Heatr3 [Source:UniProtKB/Tr	361375	19	30884411	30921078	36668	617,91	0,08	9,21E-01
ENSRNOG00000016071	**Leprel2**	leprecan-like 2 (Leprel2), mRNA [Sour	297595	4	224377008	224390639	13632	969,14	0,07	9,21E-01
ENSRNOG00000016875	**Cbx7**	chromobox homolog 7 (Cbx7), mRNA	362962	7	121126405	121143721	17317	107,26	0,11	9,21E-01
ENSRNOG00000018838	**Paox**	polyamine oxidase (exo-N4-amino) (P	293589	1	219493099	219518141	25043	237,38	-0,09	9,21E-01
ENSRNOG00000023509	**Irs2**	insulin receptor substrate 2 (Irs2), mR	29376	16	83287185	83311239	24055	2518,13	0,06	9,21E-01
ENSRNOG00000047143	**Fkbp1b**	FK506 binding protein 1B (Fkbp1b), m	58950	6	39899738	39908165	8428	326,91	0,08	9,21E-01
ENSRNOG00000050729		Uncharacterized protein [Source:UniProtKB/TrE		3	152165990	152171823	5834	18,63	-0,12	9,21E-01
ENSRNOG00000011425	**Ptpn3**	Tyrosine-protein phosphatase non-rec	362524	5	78401874	78516924	115051	82,29	-0,08	9,21E-01
ENSRNOG00000001455	**Kif13a**	kinesin family member 13A (Kif13a), m	308173	17	19600217	19782859	182643	1760,73	0,07	9,21E-01
ENSRNOG00000007604	**Igsf8**	immunoglobulin superfamily, member	304979	13	95213847	95221979	8133	2005,91	0,05	9,21E-01
ENSRNOG00000024918	**Fam229b**	family with sequence similarity 229, m	1E+08	20	45931980	45947726	15747	63,41	-0,11	9,21E-01
ENSRNOG00000020440	**Fads2**	fatty acid desaturase 2 (Fads2), mRN	83512	1	232857120	233097150	240031	7809,25	-0,06	9,21E-01
ENSRNOG00000006700	**Wdyhv1**	WDYHV motif containing 1 (Wdyhv1),	362914	7	98562311	98582049	19739	132,13	-0,07	9,21E-01

ENSRNOG00000004831	**Arid2**	Protein Arid2 [Source:UniProtKB/TrEI	366980	7	137312722	137427659	114938	2063,52	-0,06	9,22E-01
ENSRNOG00000034246	**LOC67997**	Ubiquitin-40S ribosomal protein S27a	81777	5	142431197	142431667	471	17,78	-0,11	9,22E-01
ENSRNOG00000000147	**Mast2**	microtubule associated serine/threonir	313819	5	138933880	139079273	145394	3624,05	0,05	9,22E-01
ENSRNOG00000008620		Mothers against decapentaplegic hom	25631	8	68274137	68397727	123591	323,59	0,09	9,22E-01
ENSRNOG00000015719	**Egr4**	early growth response 4 (Egr4), mRN/	25129	4	181870041	181872503	2463	56,00	-0,13	9,22E-01
ENSRNOG00000019073	**Ikbkb**	inhibitor of kappa light polypeptide ger	84351	16	73809853	73863635	53783	696,40	0,07	9,22E-01
ENSRNOG00000026979		Protein RGD1561444 [Source:UniPro	315473	8	23468894	23498785	29892	274,97	0,10	9,22E-01
ENSRNOG00000002474	**Tom1l1**	target of myb1 (chicken)-like 1 (Tom1l	287622	10	78029169	78072505	43337	229,80	-0,07	9,22E-01
ENSRNOG00000024923		neuronatin (Nnat), transcript variant 1,	94270	3	161553362	161555777	2416	47009,50	-0,08	9,22E-01
ENSRNOG00000025498	**Crb2**	crumbs homolog 2 (Drosophila) (Crb2	366031	3	27274066	27294580	20515	402,59	0,09	9,22E-01
ENSRNOG00000019290	**Pskh1**	protein serine kinase H1 (Pskh1), mRI	364993	19	48740283	48772656	32374	187,74	-0,08	9,22E-01
ENSRNOG00000016776	**Extl1**	exostosin-like glycosyltransferase 1 (E	313610	5	156370680	156385881	15202	178,30	0,08	9,22E-01
ENSRNOG00000029912		septin 5 (Sept5), mRNA [Source:RefS	116728	11	89612789	89619907	7119	6338,12	-0,08	9,22E-01
ENSRNOG00000018081	**Lactb**	lactamase, beta (Lactb), mRNA [Sourc	300803	8	77083026	77099150	16125	515,25	0,07	9,22E-01
ENSRNOG00000005298	**Zfp513**	zinc finger protein 513 (Zfp513), mRN.	313913	6	36355970	36359311	3342	1097,92	0,06	9,22E-01
ENSRNOG00000018549	**Fbxo18**	F-box protein, helicase, 18 (Fbxo18), r	291293	17	72103236	72141153	37918	3046,79	0,07	9,22E-01
ENSRNOG00000002682		zinc finger protein 692 [Source:RefSe	303164	10	43537190	43544617	7428	329,25	0,09	9,22E-01
ENSRNOG00000003272				X	64162930	64164050	1121	117,96	0,08	9,22E-01
ENSRNOG00000008656	**Snca**	synuclein, alpha (non A4 component c	29219	4	155592411	155690420	98010	2311,68	-0,11	9,23E-01
ENSRNOG00000009325	**Fuca1**	fucosidase, alpha-L- 1, tissue (Fuca1)	24375	5	158034147	158051396	17250	1554,91	0,08	9,23E-01
ENSRNOG00000009503	**Depdc1**	Protein Depdc1; RCG28484, isoform (	295538	2	283374022	283406639	32618	46,19	-0,13	9,23E-01
ENSRNOG00000009837	**LOC67981**	RIKEN cDNA D930015E06 gene [Sou	679811	2	202310349	202443572	133224	2062,89	-0,08	9,23E-01
ENSRNOG00000012521	**Shisa2**	shisa family member 2 (Shisa2), mRN	498528	15	43763011	43767309	4299	40,59	-0,11	9,23E-01
ENSRNOG00000030721	**Fbrsl1**	Protein Fbrsl1 [Source:UniProtKB/TrE	304572	12	54025375	54112849	87475	1389,69	0,06	9,23E-01
ENSRNOG00000032471		ankyrin repeat and SOCS box protein	364227	14	115009338	115105125	95788	379,96	-0,07	9,23E-01
ENSRNOG00000032659	**Plcl1**	phospholipase C-like 1 (Plcl1), mRNA	84587	9	61825029	62156595	331567	459,14	0,06	9,23E-01
ENSRNOG00000003592	**Med28**	mediator complex subunit 28 (Med28)	305391	14	70131688	70140484	8797	1567,03	0,06	9,23E-01
ENSRNOG00000004107	**Rpl23**	ribosomal protein L23 (Rpl23), mRNA	29282	10	85510910	85514900	3991	6429,12	0,08	9,23E-01
ENSRNOG00000007625	**B3galt1**	UDP-Gal:betaGlcNAc beta 1,3-galactc	366064	3	60870422	60883765	13344	519,73	-0,09	9,23E-01
ENSRNOG00000029064	**Zcchc4**	zinc finger, CCHC domain containing 4	360946	14	60629291	60670784	41494	118,42	-0,09	9,23E-01
ENSRNOG00000012087	**Ufsp2**	UFM1-specific peptidase 2 (Ufsp2), m	361151	16	49192532	49211670	19139	474,81	0,07	9,23E-01
ENSRNOG00000016229	**Gltscr1l**	GLTSCR1-like (Gltscr1l), mRNA [Sour	301235	9	15372871	15401348	28478	729,92	0,09	9,23E-01
ENSRNOG00000003243	**Wdr81**	WD repeat domain 81 (Wdr81), mRNA	303312	10	61988536	62001860	13325	1111,73	0,07	9,24E-01
ENSRNOG00000006785	**Dcaf8**	DDB1 and CUL4 associated factor 8 (	364050	13	95053335	95099779	46445	1810,55	0,08	9,24E-01
ENSRNOG00000008635	**Mks1**	Meckel syndrome, type 1 (Mks1), mRI	287612	10	74942158	74952824	10667	198,98	-0,07	9,24E-01
ENSRNOG00000009473	**Osbpl7**	oxysterol binding protein-like 7 (Osbpl	303497	10	84776869	84794423	17555	541,21	0,05	9,24E-01
ENSRNOG00000009481	**Ddhd1**	DDHD domain containing 1 (Ddhd1), r	305816	15	23929560	23996564	67005	1155,33	0,07	9,24E-01
ENSRNOG00000009609	**LOC299312**			X	2409626	2411526	1901	28,00	-0,12	9,24E-01

ENSRNOG00000010068	**Ppapdc3**	phosphatidic acid phosphatase type 2	296635	3	16467145	16481233	14089	479,99	-0,07	9,24E-01
ENSRNOG00000010071	**RGD15600**	RGD1560010 (RGD1560010), mRNA	310537	2	197725440	197728572	3133	59,06	-0,09	9,24E-01
ENSRNOG00000011603	**Cab39l**	calcium binding protein 39-like (Cab39	290291	15	43580647	43669195	88549	269,18	-0,07	9,24E-01
ENSRNOG00000011881	**Slc25a15**	solute carrier family 25 (mitochondrial	306574	16	74169024	74189301	20278	107,93	-0,08	9,24E-01
ENSRNOG00000012874	**Rgma**	RGM domain family, member A (Rgma	308739	1	135725212	135768408	43197	6096,75	-0,07	9,24E-01
ENSRNOG00000015589	**Smyd5**	SMYD family member 5 (Smyd5), mR	312503	4	181792679	181807488	14810	903,72	-0,07	9,24E-01
ENSRNOG00000017674	**Ctcf**	CCCTC-binding factor (zinc finger pro	83726	19	48467521	48516377	48857	1997,53	-0,06	9,24E-01
ENSRNOG00000018052	**Cnksr3**	Cnksr family member 3 (Cnksr3), mRN	308113	1	45120906	45213960	93055	232,66	-0,08	9,24E-01
ENSRNOG00000018517	**Trim21**	tripartite motif-containing 21 (Trim21),	308901	1	173874145	173885518	11374	66,32	-0,12	9,24E-01
ENSRNOG00000019995	**Dnajc18**	DnaJ (Hsp40) homolog, subfamily C, r	291677	18	28179461	28206342	26882	1023,89	0,09	9,24E-01
ENSRNOG00000020828		phosphodiesterase 4A, cAMP-specific	25638	8	22242476	22289441	46966	422,95	0,06	9,24E-01
ENSRNOG00000022719	**Abcb1b**	ATP-binding cassette, subfamily B (MI	24646	4	22161935	22244635	82701	194,54	-0,10	9,24E-01
ENSRNOG00000024492	**Ap1ar**	adaptor-related protein complex 1 ass	362037	2	251491339	251523419	32081	1263,75	0,08	9,24E-01
ENSRNOG00000026638	**Scamp5**	secretory carrier membrane protein 5	65171	8	61999367	62006968	7602	2064,75	0,06	9,24E-01
ENSRNOG00000028362	**LOC68999**	Protein LOC689991 [Source:UniProtK	689991	9	73619817	73692047	72231	279,47	0,09	9,24E-01
ENSRNOG00000028984	**RGD15633**	Protein RGD1563307 [Source:UniPro	308503	1	90799762	90800583	822	41,71	0,09	9,24E-01
ENSRNOG00000029808	**LOC10036**	LRRGT00053-like (LOC100360244), r	1E+08	15	65536300	65537142	843	17,46	0,12	9,24E-01
ENSRNOG00000030131	**Zscan30**	Protein Zscan30 [Source:UniProtKB/T	364827	18	15638854	15639531	678	19,34	0,12	9,24E-01
ENSRNOG00000032404	**Top2a**	topoisomerase (DNA) II alpha (Top2a)	360243	10	86699277	86735462	36186	1060,25	0,12	9,24E-01
ENSRNOG00000032459		Uncharacterized protein [Source:UniProtKB/TrE		5	165623810	165625284	1475	206,34	0,08	9,24E-01
ENSRNOG00000041744	**LOC100910332**			6	142824619	142824698	80	139,51	0,13	9,24E-01
ENSRNOG00000045670	**Tnfsf12**	tumor necrosis factor ligand superfami	360548	10	56036418	56045715	9298	84,12	-0,12	9,24E-01
ENSRNOG00000049349	**Fam19a5**	family with sequence similarity 19 (che	500915	7	128196443	128419725	223283	2986,71	-0,06	9,24E-01
ENSRNOG00000050087	**Mphosph6**	M phase phosphoprotein 6 (Mphosph6	686999	19	61135818	61145641	9824	233,52	-0,12	9,24E-01
ENSRNOG00000001256	**Atxn2**	Protein Atxn2 [Source:UniProtKB/TrE	288663	12	42137231	42203348	66118	2587,12	-0,04	9,24E-01
ENSRNOG00000003312	**Chi3l1**	chitinase 3-like 1 (cartilage glycoprote	89824	13	56075046	56086477	11432	160,04	-0,13	9,24E-01
ENSRNOG00000011784	**Mocs1**	molybdenum cofactor synthesis 1 (Mo	301221	9	12426598	12446849	20252	304,46	0,08	9,24E-01
ENSRNOG00000013061	**Tusc3**	tumor suppressor candidate 3 (Tusc3)	290783	16	55949792	56097480	147689	6146,58	0,08	9,24E-01
ENSRNOG00000019926	**Ramp1**	receptor (G protein-coupled) activity m	58965	9	97995669	98041718	46050	17,69	0,12	9,24E-01
ENSRNOG00000000098	**Rnf115**	ring finger protein 115 (Rnf115), mRN	362002	2	218339347	218406934	67588	913,72	0,05	9,24E-01
ENSRNOG00000004132	**Lasp1**	LIM and SH3 protein 1 (Lasp1), mRN	29278	10	85533431	85573897	40467	7539,38	-0,05	9,24E-01
ENSRNOG00000014552		zinc finger protein 239 [Source:MGI Symbol;Acc		4	215837491	215838096	606	81,32	0,08	9,24E-01
ENSRNOG00000016855	**B3galnt2**	beta-1,3-N-acetylgalactosaminyltransf	291212	17	51721961	51764786	42826	312,90	0,10	9,24E-01
ENSRNOG00000018179	**Glt8d1**	glycosyltransferase 8 domain containi	306253	16	7011033	7025985	14953	690,91	-0,10	9,24E-01
ENSRNOG00000018333	**Rbm6**	RNA binding motif protein 6 (Rbm6), n	315997	8	115890040	115989941	99902	1133,49	0,06	9,24E-01
ENSRNOG00000032130	**LOC10036**	6.8 kDa mitochondrial proteolipid [Sou	1E+08	1	190738267	190738449	183	90,05	-0,09	9,24E-01
ENSRNOG00000039877	**Smim11**	small integral membrane protein 11 (S	1E+08	11	36054414	36064298	9885	525,84	-0,09	9,24E-01
ENSRNOG00000000299	**Foxo3**	forkhead box O3 (Foxo3), mRNA [Sou	294515	20	48112118	48202608	90491	719,41	0,07	9,24E-01

ENSRNOG00000000585	**Amd1**	adenosylmethionine decarboxylase 1 (	81640	20	47103762	47119440	15679	2396,23	-0,07	9,24E-01
ENSRNOG00000008124	**RGD13105**	Protein RGD1310592 [Source:UniPro	297823	5	29171674	29219259	47586	643,90	-0,09	9,24E-01
ENSRNOG00000017092	**Zfp169**	Protein Zfp169 [Source:UniProtKB/Tr	306816	17	18503758	18526760	23003	203,23	0,08	9,24E-01
ENSRNOG00000017965	**Afg3l2**	AFG3 ATPase family member 3-like 2	307350	18	62325697	62369789	44093	1617,04	0,06	9,24E-01
ENSRNOG00000022593	**Pdpr**	pyruvate dehydrogenase phosphatase	307852	19	53934957	53967868	32912	827,70	0,06	9,24E-01
ENSRNOG00000006663	**Usp2**	ubiquitin specific peptidase 2 (Usp2), r	115771	8	47022640	47046713	24074	276,79	-0,08	9,24E-01
ENSRNOG00000020542	**Krtcap2**	keratinocyte associated protein 2 (Krtc	295243	2	207975076	207979072	3997	1101,48	-0,08	9,24E-01
ENSRNOG00000027520	**Pls3**	plastin 3 (Pls3), mRNA [Source:RefSe	81748	X	119173343	119266228	92886	689,44	-0,12	9,24E-01
ENSRNOG00000029995	**Herpud2**	HERPUD family member 2 (Herpud2),	300463	8	26290107	26335056	44950	1448,92	0,07	9,24E-01
ENSRNOG00000000480	**Phf1**	PHD finger protein 1 (Phf1), mRNA [S	294287	20	7585974	7590952	4979	655,61	0,06	9,24E-01
ENSRNOG00000021151	**Ppp1r14b**	protein phosphatase 1, regulatory (inh	259225	1	229220688	229222797	2110	3313,64	0,12	9,24E-01
ENSRNOG00000017230	**LOC10091**	cytochrome c oxidase assembly homo	309391	1	270939907	270956562	16656	495,49	-0,05	9,24E-01
ENSRNOG00000007678	**Zfp263**	zinc finger protein 263 (Zfp263), mRN	287076	10	10781419	10788226	6808	437,34	-0,09	9,24E-01
ENSRNOG00000030478				MT	68	1025	958	16251,02	-0,09	9,24E-01
ENSRNOG00000020524	**Thbs3**	Protein LOC681309 [Source:UniProtK	681309	2	207943473	207955131	11659	254,47	-0,09	9,24E-01
ENSRNOG00000038459	**Ulk3**	unc-51 like kinase 3 (Ulk3), mRNA [Sc	691171	8	62146623	62151819	5197	84,79	-0,09	9,24E-01
ENSRNOG00000049983	**Shd**	src homology 2 domain-containing trai	316507	9	10075316	10081904	6589	529,18	0,09	9,25E-01
ENSRNOG00000005904	**Cdc27**	cell division cycle 27 (Cdc27), mRNA [	360643	10	92316648	92364397	47750	705,52	0,11	9,25E-01
ENSRNOG00000011422	**Atmin**	ATM interactor (Atmin), mRNA [Source	315037	19	60273787	60290987	17201	207,16	0,07	9,25E-01
ENSRNOG00000003691	**Tmed7**	transmembrane emp24 protein transp	252889	18	40233821	40240942	7122	3182,82	-0,09	9,25E-01
ENSRNOG00000008475	**Fut9**	fucosyltransferase 9 (alpha (1,3) fucos	84597	5	44660497	44863523	203027	65,87	-0,12	9,25E-01
ENSRNOG00000028752	**Tmem81**	transmembrane protein 81 (Tmem81),	498229	13	54353122	54355551	2430	24,91	0,10	9,25E-01
ENSRNOG00000019419	**Taf6l**	TAF6-like RNA polymerase II, p300/CI	309194	1	231912413	231922611	10199	444,15	-0,07	9,25E-01
ENSRNOG00000017654	**Emc8**	ER membrane protein complex subun	361425	19	64955328	64965075	9748	1076,16	0,07	9,25E-01
ENSRNOG00000017852		asparaginyl-tRNA synthetase (Nars), t	291556	18	59196512	59212728	16217	7321,83	-0,06	9,25E-01
ENSRNOG00000021177	**Cox8a**	cytochrome c oxidase subunit VIIIa (C	171335	1	229457140	229459465	2326	7152,34	0,11	9,25E-01
ENSRNOG00000037540	**LOC10091**	transmembrane protein 256 (Tmem25	287442	10	56191141	56192248	1108	426,20	0,10	9,25E-01
ENSRNOG00000008756		orthodenticle homolog 1 (Drosophila) [Source:M		14	106926625	106929875	3251	117,41	-0,07	9,25E-01
ENSRNOG00000011618	**Mmaa**	methylmalonic aciduria (cobalamin de	291939	19	43204957	43222204	17248	263,38	0,09	9,25E-01
ENSRNOG00000015421	**Slc27a3**	solute carrier family 27 (fatty acid trans	295219	2	209191620	209196288	4669	1137,53	0,07	9,25E-01
ENSRNOG00000001874	**Slc7a4**	solute carrier family 7, member 4 (Slc7	303787	11	90497556	90501226	3671	368,40	-0,08	9,25E-01
ENSRNOG00000014041	**Cep78**	centrosomal protein 78 (Cep78), mRN	60347	1	240303398	240331196	27799	413,07	0,09	9,25E-01
ENSRNOG00000021153	**Fkbp2**	FK506 binding protein 2 (Fkbp2), trans	293702	1	229223204	229225877	2674	1116,87	0,09	9,25E-01
ENSRNOG00000012709	**Bag2**	Bcl2-associated athanogene 2 (Bag2)	690038	9	38145393	38147902	2510	628,79	-0,07	9,26E-01
ENSRNOG00000009851	**Slc38a9**	solute carrier family 38, member 9 (Slc	310091	2	63565645	63631032	65388	130,98	-0,09	9,26E-01
ENSRNOG00000010510		Collagen alpha-1(XII) chain [Source:U	25683	8	86584556	86698440	113885	834,93	-0,09	9,26E-01
ENSRNOG00000020922	**Hspb6**	heat shock protein, alpha-crystallin-rel	192245	1	90162903	90165073	2171	325,03	0,08	9,26E-01
ENSRNOG00000010558	**Ppif**	peptidylprolyl isomerase F (Ppif), mRN	282819	16	1955697	1962447	6751	644,49	0,06	9,26E-01

ENSRNOG00000019723	**Rbm12**	RNA binding motif protein 12 (Rbm12)	652928	3	158005713	158023035	17323	26,05	-0,10	9,26E-01
ENSRNOG00000023107	**Yeats2**	YEATS domain containing 2 (Yeats2),	498112	11	87394894	87645690	250797	1946,47	0,06	9,26E-01
ENSRNOG00000002128	**Ppat**	phosphoribosyl pyrophosphate amidot	117544	14	33371454	33405832	34379	1103,64	-0,07	9,26E-01
ENSRNOG00000001715	**Ece2**	endothelin-converting enzyme 2 (Ece2	408243	11	87020944	87036302	15359	195,61	-0,07	9,26E-01
ENSRNOG00000004279		G protein-coupled receptor 125 [Source:MGI Sy		14	64778550	64878260	99711	1308,36	0,05	9,26E-01
ENSRNOG00000004777	**Esf1**	ESF1, nucleolar pre-rRNA processing	366203	3	139635099	139688205	53107	212,22	0,11	9,26E-01
ENSRNOG00000010362	**Anxa2**	annexin A2 (Anxa2), mRNA [Source:R	56611	8	80014371	80050842	36472	486,60	0,09	9,26E-01
ENSRNOG00000011647	**S100a6**	S100 calcium binding protein A6 (S10	85247	2	209441323	209442604	1282	19,14	-0,12	9,26E-01
ENSRNOG00000016578	**Mtpap**	mitochondrial poly(A) polymerase (Mtp	307050	17	54083470	54105086	21617	600,76	-0,05	9,26E-01
ENSRNOG00000016612	**Trim36**	tripartite motif-containing 36 (Trim36),	291597	18	39737213	39789086	51874	286,82	0,09	9,26E-01
ENSRNOG00000019382	**Zbtb47**	Protein Zfp651 [Source:UniProtKB/Tr	316087	8	129581305	129586826	5522	2031,66	-0,07	9,26E-01
ENSRNOG00000020039	**Slc5a2**	solute carrier family 5 (sodium/glucose	64522	1	206728635	206734756	6122	23,40	-0,11	9,26E-01
ENSRNOG00000020695	**Tead2**	TEA domain family member 2 (Tead2)	308582	1	102279567	102295451	15885	534,50	0,10	9,26E-01
ENSRNOG00000021113	**Uba2**	ubiquitin-like modifier activating enzym	308508	1	91155863	91179053	23191	314,53	0,06	9,26E-01
ENSRNOG00000026985	**Phldb1**	pleckstrin homology-like domain, famil	171434	8	47615943	47656917	40975	3133,74	-0,10	9,26E-01
ENSRNOG00000027035	**Sgol2**	shugoshin-like 2 (S. pombe) (Sgol2), r	316425	9	64698066	64714965	16900	30,28	0,12	9,26E-01
ENSRNOG00000035499	**Mir124-2**	rno-mir-124-2 [Source:miRBase;Acc:MI0000894		2	122049028	122049136	109	18,42	0,12	9,26E-01
ENSRNOG00000012698	**Chsy1**	chondroitin sulfate synthase 1 (Chsy1)	292999	1	128095353	128156041	60689	1355,84	-0,06	9,26E-01
ENSRNOG00000014996	**Katna1**	katanin p60 (ATPase-containing) subu	292464	1	3522927	3564343	41417	225,78	0,08	9,26E-01
ENSRNOG00000030285	**Epha3**	Eph receptor A3 (Epha3), mRNA [Sou	29210	11	423018	782262	359245	363,44	0,10	9,26E-01
ENSRNOG00000018452		unc-13 homolog A (C. elegans) [Source:MGI Sy		16	19917585	19958035	40451	2947,40	-0,07	9,27E-01
ENSRNOG00000021183	**LOC10091**	REST corepressor 2 (Rcor2), mRNA [	305811	15	23568509	23574620	6112	646,31	-0,11	9,27E-01
ENSRNOG00000023375	**Rab34**	RAB34, member RAS oncogene famil	360571	10	66182985	66185961	2977	437,63	-0,09	9,27E-01
ENSRNOG00000002137	**Aasdh**	Protein Aasdh [Source:UniProtKB/TrE	364136	14	33415303	33438093	22791	397,04	0,10	9,27E-01
ENSRNOG00000009170	**Dmxl2**	Protein Dmxl2-ps1 [Source:UniProtKE	315676	8	57509282	57571056	61775	1704,22	0,09	9,27E-01
ENSRNOG00000011302	**Rab11a**	RAB11a, member RAS oncogene fam	81830	8	69896587	69919171	22585	4365,18	-0,04	9,27E-01
ENSRNOG00000015685	**Mfsd3**	major facilitator superfamily domain cc	500899	7	117751879	117753881	2003	321,82	0,06	9,27E-01
ENSRNOG00000018367	**Taldo1**	transaldolase 1 (Taldo1), mRNA [Sour	83688	1	221292657	221303008	10352	4553,40	0,06	9,27E-01
ENSRNOG00000018718	**Rpp30**	ribonuclease P/MRP subunit (Rpp30),	685332	1	262053598	262076260	22663	458,96	0,06	9,27E-01
ENSRNOG00000032871	**Mlc1**	megalencephalic leukoencephalopath	315215	7	129636392	129656289	19898	15480,69	-0,09	9,27E-01
ENSRNOG00000016596	**Hspa4**	heat shock protein 4 (Hspa4), mRNA [	266759	10	38383101	38423972	40872	8434,70	-0,07	9,27E-01
ENSRNOG00000019383	**Tef**	thyrotrophic embryonic factor (Tef), mF	29362	7	123019282	123034383	15102	3789,62	0,05	9,27E-01
ENSRNOG00000028841	**Mt1m**	Metallothionein [Source:UniProtKB/Tr	1E+08	20	5766751	5766936	186	24,37	0,12	9,27E-01
ENSRNOG00000025545		trans-golgi network vesicle protein 23	360467	10	4123748	4158380	34633	1522,60	0,08	9,27E-01
ENSRNOG00000011060	**Unc119**	UNC-119 homolog (C. elegans) (Unc1	29402	10	66026834	66032267	5434	1288,70	-0,10	9,27E-01
ENSRNOG00000002767	**Dlg3**	discs, large homolog 3 (Drosophila) (D	58948	X	71468334	71519054	50721	2884,37	0,08	9,27E-01
ENSRNOG00000007663	**LOC10091**	40S ribosomal protein S6 [Source:Un	29304	5	109196750	109199600	2851	420,84	0,11	9,28E-01
ENSRNOG00000007629	**Sf3a3**	splicing factor 3a, subunit 3 (Sf3a3), m	313583	5	146473837	146493458	19622	670,96	0,08	9,28E-01

ENSRNOG00000007654		Lrig3 protein; Protein Lrig3 [Source:UniProtKB/T		7	69404288	69454370	50083	177,64	-0,09	9,28E-01
ENSRNOG00000010966	**Itgb1**	integrin, beta 1 (Itgb1), mRNA [Source	24511	19	72324612	72372323	47712	5937,82	0,08	9,28E-01
ENSRNOG00000012054	**Zmpste24**	zinc metallopeptidase STE24 (Zmpste	313564	5	143777121	143808982	31862	896,25	0,09	9,28E-01
ENSRNOG00000023077	**Cpne9**	copine family member IX (Cpne9), mR	297516	4	208536106	208559592	23487	18,94	-0,12	9,28E-01
ENSRNOG00000012536	**Sgms1**	sphingomyelin synthase 1 (Sgms1), m	353229	1	257923301	258182226	258926	353,87	-0,07	9,28E-01
ENSRNOG00000014215	**Klf9**	Kruppel-like factor 9 (Klf9), mRNA [So	117560	1	248193534	248218136	24603	411,23	-0,07	9,28E-01
ENSRNOG00000015109	**Ubxn8**	UBX domain protein 8 (Ubxn8), mRNA	290802	16	61911951	61933546	21596	369,31	-0,08	9,28E-01
ENSRNOG00000017047	**Inip**	Protein Inip; Similar to HSPC043 prote	298032	5	81152939	81162816	9878	92,68	-0,07	9,28E-01
ENSRNOG00000021537	**Rai1**	retinoic acid induced 1 (Rai1), mRNA [	303188	10	46311520	46328065	16546	2274,26	-0,06	9,28E-01
ENSRNOG00000038025	**Rpp38**	ribonuclease P/MRP 38 subunit (Rpp3	291317	17	80556409	80559948	3540	204,45	-0,06	9,28E-01
ENSRNOG00000004284	**Btg1**	B-cell translocation gene 1, anti-prolife	29618	7	37858321	37860578	2258	2781,58	0,09	9,28E-01
ENSRNOG00000009894	**Ccdc132**	coiled-coil domain containing 132 (Cc	312083	4	28344519	28455450	110932	332,67	0,11	9,28E-01
ENSRNOG00000017099	**Nsdhl**	NAD(P) dependent steroid dehydroge	309262	1	148661899	148692767	30869	935,45	-0,11	9,28E-01
ENSRNOG00000042286	**Nsl1**	NSL1, MIS12 kinetochore complex co	498310	13	114264896	114292134	27239	182,09	-0,06	9,28E-01
ENSRNOG00000006980	**Vcpip1**	valosin containing protein (p97)/p47 c	286761	5	14031200	14059036	27837	1261,64	-0,08	9,28E-01
ENSRNOG00000009470	**Flnb**	filamin B, beta (Flnb), mRNA [Source:	306204	15	22716610	22849363	132754	793,35	0,08	9,28E-01
ENSRNOG00000012383	**Ndufc2**	NADH dehydrogenase (ubiquinone) 1,	293130	1	168576229	168582496	6268	3819,86	0,08	9,28E-01
ENSRNOG00000020813	**Ltbp3**	latent transforming growth factor beta	83838	1	228032961	228049059	16099	1480,26	0,07	9,28E-01
ENSRNOG00000030790	**Ctnnd1**	catenin (cadherin associated protein),	311163	3	78522966	78573636	50671	4984,44	-0,06	9,28E-01
ENSRNOG00000026853	**Urb2**	URB2 ribosome biogenesis 2 homolog	292087	19	67518507	67541817	23311	680,49	-0,07	9,28E-01
ENSRNOG00000033235		avian reticuloendotheliosis viral (v-rel)	1E+08	1	81783432	81810721	27290	174,48	-0,10	9,28E-01
ENSRNOG00000042399		Uncharacterized protein [Source:UniProtKB/TrE		5	52599509	52601175	1667	47,61	-0,09	9,28E-01
ENSRNOG00000018045				2	45222756	45223178	423	46,21	0,10	9,28E-01
ENSRNOG00000008628	**Ica1**	islet cell autoantigen 1 (Ica1), mRNA [	81024	4	34493597	34639858	146262	895,21	0,08	9,28E-01
ENSRNOG00000006720	**Rnpep**	arginyl aminopeptidase (aminopeptida	81761	13	57144705	57163169	18465	3035,21	0,05	9,29E-01
ENSRNOG00000016534		family with sequence similarity 171, m	680259	17	80669766	80784669	114904	970,49	0,07	9,29E-01
ENSRNOG00000018327	**Fank1**	fibronectin type 3 and ankyrin repeat c	309071	1	212790893	212897407	106515	73,36	-0,09	9,29E-01
ENSRNOG00000026458	**Tbccd1**	TBCC domain containing 1 (Tbccd1),	303830	11	84857007	84882195	25189	290,59	-0,06	9,29E-01
ENSRNOG00000029651		retinol dehydrogenase 16 (all-trans) (F	299511	7	71230580	71245358	14779	95,63	-0,08	9,29E-01
ENSRNOG00000031435				15	106077528	106254924	177397	84,79	0,08	9,29E-01
ENSRNOG00000042561	**Cks1l**	CDC28 protein kinase regulatory subunit 1B (Ck		2	208154853	208159427	4575	226,23	-0,06	9,29E-01
ENSRNOG00000004314	**Ppm1h**	protein phosphatase, Mg2+/Mn2+ dep	314897	7	67042623	67310367	267745	1390,80	0,07	9,29E-01
ENSRNOG00000015801	**Spns2**	spinster homolog 2 (Spns2), mRNA [S	1E+08	10	58749467	58788522	39056	538,49	-0,07	9,29E-01
ENSRNOG00000010370	**Tnip1**	TNFAIP3 interacting protein 1 (Tnip1),	363599	10	40028044	40075239	47196	671,36	0,07	9,29E-01
ENSRNOG00000018387		WD repeat domain 7 [Source:MGI Symbol;Acc:M		18	58326473	58612197	285725	2188,63	-0,05	9,30E-01
ENSRNOG00000001169	**Slc37a1**	solute carrier family 37 (glucose-6-pho	294321	20	12216552	12278026	61475	41,41	0,10	9,30E-01
ENSRNOG00000002032	**Ifngr2**	interferon gamma receptor 2 (Ifngr2),	360697	11	35302873	35321145	18273	1547,13	-0,06	9,30E-01
ENSRNOG00000002276		Protein Phf6; RCG47045, isoform CR	1E+08	X	153328379	153362187	33809	118,95	-0,09	9,30E-01

ENSRNOG00000002800	**Gdpd2**	glycerophosphodiester phosphodieste	302421	X	71435127	71443913	8787	622,77	0,07	9,30E-01
ENSRNOG00000003407	**Ptpn14**	protein tyrosine phosphatase, non-rec	305064	13	113312814	113456734	143921	322,12	-0,10	9,30E-01
ENSRNOG00000005877	**Lrpprc**	leucine-rich pentatricopeptide repeat c	313867	6	7919929	8001949	82021	2800,17	-0,09	9,30E-01
ENSRNOG00000006349	**LOC10035**	Histone H3 [Source:UniProtKB/TrEMI	1E+08	6	70571776	70573263	1488	22,54	0,10	9,30E-01
ENSRNOG00000010480	**Vstm5**	V-set and transmembrane domain con	500947	8	13735551	13755647	20097	38,15	-0,11	9,30E-01
ENSRNOG00000010653	**Elmsan1**	similar to transcriptional regulating pro	314306	6	117858477	117899444	40968	543,93	-0,08	9,30E-01
ENSRNOG00000011690	**Rmdn3**	regulator of microtubule dynamics 3 (F	311328	3	117494482	117515087	20606	264,82	0,07	9,30E-01
ENSRNOG00000011856	**LOC10091**	Protein LOC100910805 [Source:UniP	498823	14	109244900	109274018	29119	41,48	-0,09	9,30E-01
ENSRNOG00000012995	**Gpr165**	G protein-coupled receptor 165 (Gpr1	296866	X	66627493	66633278	5786	34,70	-0,09	9,30E-01
ENSRNOG00000023356	**Eif5b**	eukaryotic translation initiation factor 5	308306	9	44354656	44409587	54932	104,96	-0,11	9,30E-01
ENSRNOG00000037284	**Idh3g**	isocitrate dehydrogenase 3 (NAD), ga	25179	1	152747876	152757618	9743	2780,53	0,05	9,30E-01
ENSRNOG00000006136	**Pdpk1**	3-phosphoinositide dependent protein	81745	10	13267428	13330747	63320	962,52	0,07	9,30E-01
ENSRNOG00000002419	**Plp1**	proteolipid protein 1 (Plp1), mRNA [Sc	24943	X	107379831	107394874	15044	687,45	0,12	9,30E-01
ENSRNOG00000008283	**B4galt5**	UDP-Gal:betaGlcNAc beta 1,4-galactc	362275	3	170196325	170211612	15288	1272,79	-0,09	9,30E-01
ENSRNOG00000004111	**Soat1**	sterol O-acyltransferase 1 (Soat1), mF	81782	13	78956528	78999029	42502	515,05	-0,09	9,30E-01
ENSRNOG00000009658	**Rnf19a**	ring finger protein 19A, E3 ubiquitin pr	362900	7	75206742	75246247	39506	1308,78	0,07	9,30E-01
ENSRNOG00000015173	**Mbtps1**	membrane-bound transcription factor	89842	19	62895290	62934742	39453	2076,48	0,05	9,30E-01
ENSRNOG00000018526	**Dlg4**	discs, large homolog 4 (Drosophila) (D	29495	10	56372666	56398718	26053	9524,91	-0,06	9,30E-01
ENSRNOG00000024808	**Stk39**	serine threonine kinase 39 (Stk39), ml	54348	3	60973443	61243989	270547	965,50	-0,07	9,30E-01
ENSRNOG00000000720	**MGC95208**	similar to 4930453N24Rik protein (MG	304176	11	1794871	1799518	4648	47,10	0,09	9,30E-01
ENSRNOG00000022155	**RGD15602**	similar to chromosome 3 open reading	500258	4	189423813	189460874	37062	34,08	-0,11	9,30E-01
ENSRNOG00000008437	**Ss18l1**	synovial sarcoma translocation gene c	192352	3	181673460	181691930	18471	779,53	0,07	9,30E-01
ENSRNOG00000005447	**LOC10036**	LOC360590 (RGD1311564), mRNA [S	360590	10	75804187	75858297	54111	286,18	0,10	9,30E-01
ENSRNOG00000047456	**Ccdc94**	coiled-coil domain containing 94 (Ccd	501285	9	10084088	10093905	9818	359,60	0,08	9,30E-01
ENSRNOG00000006341		SAYSVFN motif domain containing 1 [Source:M		15	8756256	8761704	5449	146,39	0,09	9,31E-01
ENSRNOG00000013602	**LOC10091**	ubiquitin specific peptidase 48 (Usp48	362636	5	159691521	159758705	67185	972,22	0,06	9,31E-01
ENSRNOG00000014490	**Bdh2**	3-hydroxybutyrate dehydrogenase, typ	295458	2	258981008	259001648	20641	467,74	-0,10	9,31E-01
ENSRNOG00000021024	**Pi4kb**	phosphatidylinositol 4-kinase, catalytic	81747	2	215634441	215666468	32028	2217,54	0,05	9,31E-01
ENSRNOG00000047261	**LOC10091**	Protein LOC100911918 [Source:UniP	679221	18	14687027	14746311	59285	225,13	0,06	9,31E-01
ENSRNOG00000049552	**Sgsh**	Protein LOC688293 [Source:UniProtk	688293	10	108075842	108079088	3247	48,24	0,09	9,31E-01
ENSRNOG00000003346	**Fancb**	Fanconi anemia, complementation grc	501552	X	31503735	31518179	14445	30,86	0,11	9,31E-01
ENSRNOG00000003581	**Pou2f1**	POU class 2 homeobox 1 (Pou2f1), m	171068	13	88963389	89095110	131722	122,32	-0,08	9,31E-01
ENSRNOG00000007701	**Thoc7**	THO complex 7 homolog (Drosophila)	305714	15	16606452	16611280	4829	758,60	0,08	9,31E-01
ENSRNOG00000009276	**Schip1**	schwannomin interacting protein 1 (Sc	295105	2	191602850	192287061	684212	2262,84	-0,05	9,31E-01
ENSRNOG00000009430	**Gnl2**	guanine nucleotide binding protein-like	362593	5	146796615	146821993	25379	576,07	0,08	9,31E-01
ENSRNOG00000011377	**Socs4**	suppressor of cytokine signaling 4 (So	305828	15	27997125	28011599	14475	223,07	-0,09	9,31E-01
ENSRNOG00000011619	**Myo9a**	myosin IXA (Myo9a), mRNA [Source:F	171296	8	64373611	64534630	161020	828,64	0,11	9,31E-01
ENSRNOG00000011962	**Gin1**	gypsy retrotransposon integrase 1 (Gi	316687	9	110750668	110770901	20234	85,70	-0,07	9,31E-01

ENSRNOG00000012067	**Fam111a**	family with sequence similarity 111, me	499322	1	236163691	236179257	15567	115,73	0,12	9,31E-01
ENSRNOG00000012702	**Nfya**	nuclear transcription factor-Y alpha (N	29508	9	13474260	13497400	23141	707,37	0,08	9,31E-01
ENSRNOG00000037562	**Zbtb42**	zinc finger and BTB domain containing	691556	6	146250223	146252352	2130	38,44	0,10	9,31E-01
ENSRNOG00000009746	**Akap17b**	Protein Akap17b [Source:UniProtKB/T	313434	X	123364608	123379436	14829	175,96	-0,08	9,31E-01
ENSRNOG00000012622	**Mmp15**	matrix metallopeptidase 15 (Mmp15),	291848	19	10064056	10085300	21245	3341,93	-0,09	9,31E-01
ENSRNOG00000024112	**Dgkq**	diacylglycerol kinase, theta (Dgkq), ml	1E+08	14	2064952	2078913	13962	733,79	0,07	9,31E-01
ENSRNOG00000017466	**Kif5b**	kinesin family member 5B (Kif5b), mR	117550	17	51876234	51906466	30233	1705,63	-0,11	9,31E-01
ENSRNOG00000020317	**RGD13077**	similar to RIKEN cDNA 1110008F13 (F	296315	3	158753367	158763385	10019	1609,74	0,08	9,31E-01
ENSRNOG00000003208	**Ercc6l**	excision repair cross-complementing r	317252	X	72862788	72878439	15652	53,65	0,12	9,31E-01
ENSRNOG00000004003	**Dusp10**	dual specificity phosphatase 10 (Dusp	63995	13	108932784	108969913	37130	129,42	0,10	9,31E-01
ENSRNOG00000005908		cyclin D binding myb-like transcription	114485	4	21793677	21834126	40450	446,45	-0,10	9,31E-01
ENSRNOG00000009114	**Fam110b**	family with sequence similarity 110, me	500400	5	23688479	23690112	1634	1459,74	-0,06	9,31E-01
ENSRNOG00000013442	**Ciz1**	CDKN1A interacting zinc finger protein	296639	3	16741661	16759565	17905	1060,62	0,05	9,31E-01
ENSRNOG00000014883	**Git1**	G protein-coupled receptor kinase inte	83709	10	62352883	62362209	9327	5225,53	-0,07	9,31E-01
ENSRNOG00000017029	**Snx1**	sorting nexin 1 (Snx1), mRNA [Source	84471	8	71414978	71453464	38487	376,56	0,08	9,31E-01
ENSRNOG00000018184		tropomyosin 1, alpha (Tpm1), transcri	24851	8	77147583	77174392	26810	3722,82	-0,06	9,31E-01
ENSRNOG00000021670	**Frem2**	Fras1 related extracellular matrix prote	310418	2	162430795	162568910	138116	311,70	0,05	9,31E-01
ENSRNOG00000024795	**Zfp862**	Protein Zfp862 [Source:UniProtKB/Tr	681217	4	142804180	142826031	21852	137,94	0,09	9,31E-01
ENSRNOG00000003108	**Acbd4**	acyl-CoA binding domain containing 4	303577	10	90959528	90968101	8574	340,52	0,08	9,31E-01
ENSRNOG00000015381	**Ttf2**	transcription termination factor, RNA p	295324	2	222765601	222799844	34244	348,55	0,06	9,31E-01
ENSRNOG00000032723	**Eftud1**	elongation factor Tu GTP binding dom	308789	1	145530403	145668711	138309	449,14	0,06	9,31E-01
ENSRNOG00000012864	**Ern1**	endoplasmic reticulum to nucleus sign	498013	10	94340405	94400743	60339	194,63	0,06	9,31E-01
ENSRNOG00000014650	**Sema4g**	sema domain, immunoglobulin domain	361764	1	272184299	272195785	11487	1489,01	0,06	9,31E-01
ENSRNOG00000022732	**Tmem126b**	transmembrane protein 126B (Tmem1	293114	1	162508341	162520322	11982	523,65	-0,05	9,31E-01
ENSRNOG00000025926	**Heatr5b**	HEAT repeat containing 5B (Heatr5b),	362683	6	1321469	1401571	80103	2566,21	0,06	9,31E-01
ENSRNOG00000017422	**Ambra1**	autophagy/beclin 1 regulator 1 (Ambra	59319	3	87368296	87528736	160441	1621,15	-0,05	9,32E-01
ENSRNOG00000009225	**Copz2**	coatomer protein complex, subunit zet	360611	10	84511733	84524391	12659	73,20	-0,10	9,32E-01
ENSRNOG00000048295		Uncharacterized protein [Source:UniProtKB/TrE		X	73488585	73492110	3526	63,80	0,09	9,32E-01
ENSRNOG00000015052	**Star**	steroidogenic acute regulatory protein	25557	16	70698593	70705478	6886	51,79	-0,08	9,32E-01
ENSRNOG00000000595	**Traf3ip2**	Traf3 interacting protein 2 (Traf3ip2), r	361857	20	46401898	46445444	43547	60,98	0,09	9,33E-01
ENSRNOG00000012166	**Adal**	adenosine deaminase-like (Adal), mRI	311352	3	119641987	119647770	5784	72,28	-0,09	9,33E-01
ENSRNOG00000050113				10	62400915	62401054	140	43,97	-0,08	9,33E-01
ENSRNOG00000025407	**Kif15**	kinesin family member 15 (Kif15), mRI	353302	8	131187617	131258348	70732	195,43	0,11	9,33E-01
ENSRNOG00000015322		zinc finger protein 78 [Source:MGI Symbol;Acc:M		1	72576020	72583272	7253	33,12	-0,09	9,33E-01
ENSRNOG00000020587		EGF-containing fibulin-like extracellula	293677	1	227783786	227790739	6954	558,50	0,09	9,33E-01
ENSRNOG00000025629	**Prpf3**	pre-mRNA processing factor 3 (Prpf3)	361995	2	217438359	217462273	23915	757,18	0,07	9,33E-01
ENSRNOG00000031495	**Tmem170b**	transmembrane protein 170B (Tmem1	361230	17	24806010	24837335	31326	308,14	-0,07	9,33E-01
ENSRNOG00000026965		Transmembrane protein 140 [Source:	362334	4	62112723	62123502	10780	29,51	0,11	9,33E-01

ENSRNOG00000040287	**Cyp1b1**	cytochrome P450, family 1, subfamily	25426	6	2298849	2300644	1796	42,48	-0,11	9,33E-01
ENSRNOG00000009917	**LOC50022**	RIKEN cDNA 1700003E16 gene [Sou	500227	4	179443528	179449747	6220	224,03	0,06	9,33E-01
ENSRNOG00000004557	**Zfp763**	zinc finger protein 763 (Zfp763), mRN	314586	7	15455707	15460067	4361	176,76	-0,08	9,33E-01
ENSRNOG00000000648	**Jmjd1c**	jumonji domain containing 1C (Jmjd1c	171120	20	24842479	24972937	130459	2803,27	-0,06	9,33E-01
ENSRNOG00000002828	**Tob1**	transducer of ErbB-2.1 (Tob1), mRNA	170842	10	81746522	81748549	2028	716,61	-0,05	9,33E-01
ENSRNOG00000003078		Protein Dcaf6; Similar to IQ motif and WD repea		13	88458601	88559585	100985	1884,59	0,06	9,33E-01
ENSRNOG00000005316		Uncharacterized protein [Source:UniProtKB/TrE		7	59647425	59648698	1274	35,66	0,09	9,33E-01
ENSRNOG00000015184	**Clic3**	chloride intracellular channel 3 (Clic3),	296566	3	2657749	2659658	1910	40,73	-0,09	9,33E-01
ENSRNOG00000017209	**Tubb3**	tubulin, beta 3 class III (Tubb3), mRN	246118	19	66931873	66940864	8992	30208,27	0,08	9,33E-01
ENSRNOG00000017258	**Ica1l**	islet cell autoantigen 1-like (Ica1l), mR	316432	9	66602089	66639739	37651	185,84	-0,06	9,33E-01
ENSRNOG00000031085	**Ergic3**	ERGIC and golgi 3 (Ergic3), mRNA [S	296306	3	157918210	157927936	9727	3471,56	-0,05	9,33E-01
ENSRNOG00000050706		Protein LOC687064 [Source:UniProtK	687064	2	254151921	254267154	115234	90,67	-0,09	9,33E-01
ENSRNOG00000001337		SET domain containing 1B [Source:MGI Symbol		12	40746333	40767105	20773	1376,51	0,05	9,33E-01
ENSRNOG00000001843	**Bcl6**	B-cell CLL/lymphoma 6 (Bcl6), mRNA	303836	11	80454014	80477192	23179	378,51	-0,07	9,33E-01
ENSRNOG00000011849	**Ndufs1**	NADH dehydrogenase (ubiquinone) F	301458	9	69728736	69762079	33344	2102,39	-0,07	9,33E-01
ENSRNOG00000016497	**Bloc1s5**	biogenesis of lysosomal organelles co	306868	17	28724370	28749287	24918	332,47	-0,06	9,33E-01
ENSRNOG00000020394	**RGD13067**	similar to cDNA sequence BC025641	309009	1	207165765	207210643	44879	1783,19	0,04	9,33E-01
ENSRNOG00000028771	**LOC69190**	predicted gene 166 [Source:MGI Sym	691909	1	206054803	206059914	5112	66,21	-0,08	9,33E-01
ENSRNOG00000006999	**Wdr78**	WD repeat domain 78 (Wdr78), mRN	313417	5	126419824	126507661	87838	196,01	-0,09	9,34E-01
ENSRNOG00000000275		family with sequence similarity 13, me	294565	20	21507803	21625228	117426	946,30	-0,05	9,34E-01
ENSRNOG00000001520	**Dlx1**	distal-less homeobox 1 (Dlx1), mRNA	296500	3	64650292	64654882	4591	2313,31	-0,08	9,34E-01
ENSRNOG00000001701	**Cbr3**	carbonyl reductase 3 (Cbr3), mRNA [S	304078	11	37500661	37508918	8258	458,75	-0,10	9,34E-01
ENSRNOG00000005526	**Man2b2**	mannosidase, alpha, class 2B, membe	360955	14	87135572	87159745	24174	927,10	0,07	9,34E-01
ENSRNOG00000008088	**Btbd3**	BTB (POZ) domain containing 3 (Btbd	311462	3	137825258	137831600	6343	608,86	0,08	9,34E-01
ENSRNOG00000008682	**Mtch2**	mitochondrial carrier 2 (Mtch2), mRN	295922	3	86385047	86404835	19789	3633,53	0,07	9,34E-01
ENSRNOG00000010861	**LOC10091**	ATPase family, AAA domain containin	309532	1	258466603	258609714	143112	2092,17	0,06	9,34E-01
ENSRNOG00000012708	**Ptchd4**	Protein Ptchd4 [Source:UniProtKB/Tr	301271	9	20002092	20202925	200834	20,93	0,11	9,34E-01
ENSRNOG00000014476	**Evl**	Enah/Vasp-like (Evl), mRNA [Source:F	79115	6	141721470	141760317	38848	7522,77	-0,06	9,34E-01
ENSRNOG00000015551	**Fuca2**	fucosidase, alpha-L- 2, plasma (Fuca2	292485	1	9287057	9301800	14744	238,67	-0,09	9,34E-01
ENSRNOG00000015777	**Mex3c**	mex-3 homolog C (C. elegans) (Mex3	307271	18	68695439	68717238	21800	1168,53	-0,06	9,34E-01
ENSRNOG00000016269	**Rpl7l1**	Protein Rpl7l1 [Source:UniProtKB/TrE	317275	9	15416069	15420414	4346	1385,28	0,06	9,34E-01
ENSRNOG00000017612	**Vps35**	vacuolar protein sorting 35 homolog (	25479	19	38430733	38466263	35531	2456,25	-0,11	9,34E-01
ENSRNOG00000018988		inhibitor of growth family, member 5 (I	363292	9	100585744	100603189	17446	370,16	0,07	9,34E-01
ENSRNOG00000019592		Protein Iqca1 [Source:UniProtKB/TrE	316616	9	96873940	96982269	108330	34,62	-0,10	9,34E-01
ENSRNOG00000020073	**Pcdhb8**	protocadherin beta 8 (Pcdhb8), mRNA	680020	18	30142319	30144658	2340	139,34	0,10	9,34E-01
ENSRNOG00000020084	**Pcdhb5**	protocadherin beta 5 (Pcdhb5), mRNA	291654	18	30105642	30108224	2583	444,62	-0,07	9,34E-01
ENSRNOG00000027191	**Birc6**	baculoviral IAP repeat-containing 6 (B	313876	6	31524882	31980891	456010	2025,38	0,10	9,34E-01
ENSRNOG00000031669	**Lpp**	LIM domain containing preferred trans	288010	11	81026494	81519788	493295	45,37	0,09	9,34E-01

ENSRNOG00000037262	**Mecp2**	methyl CpG binding protein 2 (Mecp2)	29386	1	152450808	152452884	2077	602,00	-0,05	9,34E-01
ENSRNOG00000047339	**LOC68307**	praja1, RING-H2 motif containing [Sou	683077	X	70086047	70090909	4863	5322,17	0,07	9,34E-01
ENSRNOG00000050550	**Nr2e1**	nuclear receptor subfamily 2, group E,	684085	20	48957316	48976460	19145	686,77	-0,08	9,34E-01
ENSRNOG00000002664	**Emp2**	epithelial membrane protein 2 (Emp2)	360468	10	4254720	4288862	34143	114,01	-0,09	9,34E-01
ENSRNOG00000004837	**Limd1**	LIM domains containing 1 (Limd1), mF	316101	8	131705918	131751522	45605	545,39	-0,07	9,34E-01
ENSRNOG00000005841	**Erp44**	endoplasmic reticulum protein 44 (Erp	298066	5	68454074	68548279	94206	1306,81	-0,06	9,34E-01
ENSRNOG00000005981	**Nt5c3a**	5'-nucleotidase, cytosolic IIIA (Nt5c3a)	312373	4	151889319	151932147	42829	1085,96	-0,05	9,34E-01
ENSRNOG00000011451	**Lrp3**	low density lipoprotein receptor-relatec	89787	1	92579059	92593301	14243	2824,82	0,06	9,34E-01
ENSRNOG00000012385	**Naa15**	N(alpha)-acetyltransferase 15, NatA a	310399	2	159946628	160009197	62570	1502,12	0,10	9,34E-01
ENSRNOG00000012692	**Riok2**	RIO kinase 2 (Riok2), mRNA [Source:	308201	1	60057339	60073786	16448	284,62	-0,07	9,34E-01
ENSRNOG00000018615	**Cenph**	Protein Cenph [Source:UniProtKB/TrE	681185	2	49924881	49937755	12875	73,91	0,10	9,34E-01
ENSRNOG00000020703	**Sipa1l3**	signal-induced proliferation-associatec	292771	1	88439887	88477653	37767	620,38	-0,05	9,34E-01
ENSRNOG00000025071	**Qrich1**	glutamine-rich 1 (Qrich1), mRNA [Sou	301004	8	116652041	116691239	39199	1935,14	0,06	9,34E-01
ENSRNOG00000025981	**LOC69196**	spindle and kinetochore associated cc	287598	10	75668741	75686674	17934	50,71	-0,09	9,34E-01
ENSRNOG00000027015	**Ythdf1**	YTH domain family, member 1 (Ythdf1	296467	3	180125834	180141343	15510	2512,26	-0,05	9,34E-01
ENSRNOG00000038176	**Ufm1**	ubiquitin-fold modifier 1 (Ufm1), mRNA	365797	2	162779819	162787960	8142	1015,84	0,05	9,34E-01
ENSRNOG00000011854	**RGD15639**	Protein LCHN [Source:UniProtKB/Sw	312270	4	133355168	133382858	27691	593,82	0,05	9,34E-01
ENSRNOG00000023679	**Prdm5**	Protein Prdm5 [Source:UniProtKB/TrE	689788	4	161443647	161572269	128623	36,26	0,09	9,34E-01
ENSRNOG00000005045	**Ppp2r5e**	protein phosphatase 2, regulatory sub	299147	6	107836104	107985205	149102	3379,51	0,06	9,34E-01
ENSRNOG00000006067	**Aifm1**	apoptosis-inducing factor, mitochondri	83533	X	135375533	135414546	39014	460,11	0,07	9,34E-01
ENSRNOG00000008440	**Cttnbp2**	cortactin binding protein 2 (Cttnbp2), r	282587	4	42516939	42680592	163654	1628,31	0,07	9,34E-01
ENSRNOG00000021781	**Camk1**	calcium/calmodulin-dependent protein	171503	4	208586463	208596796	10334	1522,19	-0,06	9,34E-01
ENSRNOG00000014886	**Fam107b**	family with sequence similarity 107, m	498796	17	80136084	80208479	72396	1097,16	0,09	9,34E-01
ENSRNOG00000016549	**Tfb1m**	transcription factor B1, mitochondrial (	308140	1	45762960	45804760	41801	421,80	-0,06	9,34E-01
ENSRNOG00000013069	**Sapcd2**	suppressor APC domain containing 2	680531	3	2572744	2577996	5253	1138,18	0,11	9,34E-01
ENSRNOG00000001816	**Rfc4**	replication factor C (activator 1) 4 (Rfc	288003	11	84396735	84411361	14627	255,03	-0,10	9,34E-01
ENSRNOG00000005851	**Tmf1**	TATA element modulatory factor 1 (Tm	114206	4	194025430	194047939	22510	463,23	-0,10	9,34E-01
ENSRNOG00000014891	**Ptar1**	protein prenyltransferase alpha subun	286972	1	248783205	248838996	55792	156,29	-0,08	9,35E-01
ENSRNOG00000015030	**Tjp2**	tight junction protein 2 (Tjp2), mRNA [	115769	1	249224596	249358699	134104	1532,36	0,08	9,35E-01
ENSRNOG00000017605	**Gmds**	GDP-mannose 4, 6-dehydratase (Gm	291095	17	35298853	35824291	525439	716,06	0,05	9,35E-01
ENSRNOG00000018825		DDB1 and CUL4 associated factor 11	305895	15	38124950	38134699	9750	1745,74	0,05	9,35E-01
ENSRNOG00000019413	**Atg16l2**	autophagy related 16-like 2 (S. cerevis	308865	1	172590904	172603337	12434	116,45	-0,10	9,35E-01
ENSRNOG00000050951	**Rnf135**	ring finger protein 135 (Rnf135), mRN	303350	10	67189251	67207113	17863	86,89	-0,08	9,35E-01
ENSRNOG00000001524	**Hat1**	histone acetyltransferase 1 (Hat1), mF	296501	3	64510903	64558567	47665	403,60	-0,10	9,35E-01
ENSRNOG00000022894		Protein Bivm; RCG22223 [Source:UniProtKB/Tr		9	50559566	50586752	27187	354,18	-0,06	9,35E-01
ENSRNOG00000042496	**Cyp4f17**	cytochrome P450, family 4, subfamily	500801	7	14682232	14697943	15712	60,52	0,08	9,35E-01
ENSRNOG00000006178		dynein cytoplasmic 1 heavy chain 1 (D	29489	6	144094996	144159980	64985	20957,42	0,06	9,35E-01
ENSRNOG00000008901	**Coro2a**	coronin, actin binding protein 2A (Corc	313235	5	66684828	66714854	30027	317,94	0,08	9,35E-01

ENSRNOG00000021000	**Naaladl1**	N-acetylated alpha-linked acidic dipep	83568	1	228405386	228418544	13159	118,47	-0,06	9,35E-01
ENSRNOG00000007203	**Psmc6**	proteasome (prosome, macropain) 26	289990	15	23631640	23653070	21431	1260,69	-0,11	9,35E-01
ENSRNOG00000030397	**Zbtb1**	zinc finger and BTB domain containing	314246	6	108825981	108828652	2672	339,64	0,06	9,35E-01
ENSRNOG00000031113		zinc finger and SCAN domain containing 22 [Sou		1	66488759	66492578	3820	159,38	-0,07	9,35E-01
ENSRNOG00000039862	**Cldn12**	claudin 12 (Cldn12), mRNA [Source:R	500000	4	25593560	25599715	6156	1057,11	0,06	9,35E-01
ENSRNOG00000043002	**Lcmt2**	leucine carboxyl methyltransferase 2 (	296098	3	119629556	119631616	2061	199,02	-0,05	9,35E-01
ENSRNOG00000002541	**Pds5a**	PDS5, regulator of cohesion maintena	305343	14	44003226	44099515	96290	753,68	-0,10	9,35E-01
ENSRNOG00000002797	**Gpr156**	G protein-coupled receptor 156 (Gpr1	260430	11	66732875	66797798	64924	67,14	-0,10	9,35E-01
ENSRNOG00000008167	**Abhd6**	abhydrolase domain containing 6 (Abh	305795	15	22614365	22661344	46980	915,85	-0,07	9,35E-01
ENSRNOG00000008399	**Dcaf4**	DDB1 and CUL4 associated factor 4 (	362762	6	118513786	118539582	25797	735,08	0,05	9,35E-01
ENSRNOG00000010971	**Snx18**	sorting nexin 18 (Snx18), mRNA [Sour	310097	2	64486855	64512619	25765	1177,08	0,06	9,35E-01
ENSRNOG00000014509	**Sacs**	Protein Sacs [Source:UniProtKB/TrEM	305940	15	45295350	45374159	78810	1269,64	0,10	9,35E-01
ENSRNOG00000020124	**Lrrc51**	leucine rich repeat containing 51 (Lrrc	293156	1	173188730	173207946	19217	36,95	-0,09	9,35E-01
ENSRNOG00000022778	**Pphln1**	periphilin 1 (Pphln1), mRNA [Source:F	366975	7	134270162	134361035	90874	1164,09	0,06	9,35E-01
ENSRNOG00000000216	**Klf11**	Kruppel-like factor 11 (Klf11), mRNA [S	313994	6	60701428	60713134	11707	972,06	-0,08	9,35E-01
ENSRNOG00000002352	**Tsen15**	tRNA splicing endonuclease 15 homol	289083	13	74737179	74753103	15925	593,44	0,08	9,35E-01
ENSRNOG00000002376		Uncharacterized protein [Source:UniProtKB/TrE		13	39530430	39713144	182715	3321,35	-0,05	9,35E-01
ENSRNOG00000007743	**Mgst1**	microsomal glutathione S-transferase	171341	4	236372197	236387443	15247	410,44	-0,10	9,35E-01
ENSRNOG00000009192	**Rnaseh2b**	ribonuclease H2, subunit B (Rnaseh2b	361056	15	49197430	49240305	42876	306,03	0,07	9,35E-01
ENSRNOG00000010252	**Hexa**	hexosaminidase A (Hexa), mRNA [Sou	300757	8	64087247	64112585	25339	1222,53	0,07	9,35E-01
ENSRNOG00000010729	**Usp8**	ubiquitin specific peptidase 8 (Usp8), r	296121	3	125699400	125747040	47641	1461,61	0,05	9,35E-01
ENSRNOG00000011380	**Kcnc1**	potassium voltage gated channel, Sha	25327	1	103500850	103541476	40627	405,10	-0,06	9,35E-01
ENSRNOG00000012314	**Slirp**	SRA stem-loop interacting RNA bindin	688717	6	120757482	120766753	9272	648,11	-0,06	9,35E-01
ENSRNOG00000014188	**Tma16**	translation machinery associated 16 h	290686	16	24862193	24878384	16192	61,68	-0,08	9,35E-01
ENSRNOG00000015068	**Il11ra1**	interleukin 11 receptor, alpha chain 1 (	245983	5	62676351	62683438	7088	789,73	-0,10	9,35E-01
ENSRNOG00000016321	**Entpd4**	ectonucleoside triphosphate diphosph	361063	15	54952393	54980226	27834	1251,79	0,08	9,35E-01
ENSRNOG00000016949	**Slc25a33**	solute carrier family 25 (pyrimidine nuc	691431	5	170345879	170371208	25330	927,58	-0,04	9,35E-01
ENSRNOG00000019981	**Sdf4**	stromal cell derived factor 4 (Sdf4), ml	155173	5	176901518	176918493	16976	2233,47	0,05	9,35E-01
ENSRNOG00000020451	**Cd81**	Cd81 molecule (Cd81), mRNA [Source	25621	1	223099329	223115089	15761	19566,40	-0,08	9,35E-01
ENSRNOG00000024845		28S ribosomal protein S21, mitochond	689432	2	217465593	217473727	8135	524,83	-0,05	9,35E-01
ENSRNOG00000025060	**Nanos1**	Protein Nanos1 [Source:UniProtKB/Tr	365494	1	289473532	289474323	792	93,23	-0,07	9,35E-01
ENSRNOG00000025502	**Arhgef37**	Rho guanine nucleotide exchange fac	307398	18	56048375	56099677	51303	78,29	0,10	9,35E-01
ENSRNOG00000027026	**Jazf1**	Protein Jazf1 [Source:UniProtKB/TrEI	685879	4	147501616	147547058	45443	454,12	-0,07	9,35E-01
ENSRNOG00000028858	**Rbmxrtl**	RNA binding motif protein, X chromos	307779	4	41774128	41775805	1678	315,71	0,05	9,35E-01
ENSRNOG00000028946	**Cdc42ep4**	CDC42 effector protein (Rho GTPase	303653	10	101923477	101947679	24203	2386,63	-0,06	9,35E-01
ENSRNOG00000029055	**Ttk**	Ttk protein kinase (Ttk), mRNA [Sourc	315852	8	90886762	90924325	37564	119,89	0,11	9,35E-01
ENSRNOG00000031032		high mobility group nucleosomal bindi	113990	8	90151453	90187798	36346	1062,19	0,07	9,35E-01
ENSRNOG00000032846	**RGD13060**	similar to 2210021J22Rik protein (RGI	362975	7	126398401	126402228	3828	177,24	-0,07	9,35E-01

ENSRNOG00000033411	**Usp12**	ubiquitin specific peptidase 12 (Usp12	360763	12	12145472	12195185	49714	77,93	-0,09	9,35E-01
ENSRNOG00000042869	**LOC10036(**	Protein LOC100360002 [Source:UniP	1E+08	10	43262511	43262819	309	72,52	0,08	9,35E-01
ENSRNOG00000046460		TatD DNase domain containing 3 [Sou	289378	13	114250219	114264743	14525	153,85	0,07	9,35E-01
ENSRNOG00000017752	**Mccc2**	methylcrotonoyl-CoA carboxylase 2 (b	361884	2	49333902	49404647	70746	1257,04	0,05	9,35E-01
ENSRNOG00000020478	**Camk4**	calcium/calmodulin-dependent protein	25050	18	25464155	25680622	216468	285,77	0,09	9,35E-01
ENSRNOG00000013076	**Csnk1e**	casein kinase 1, epsilon (Csnk1e), mF	58822	7	120646909	120665180	18272	3056,27	0,04	9,35E-01
ENSRNOG00000013493	**Psma4**	proteasome (prosome, macropain) sul	29671	8	58114599	58122076	7478	1563,32	-0,09	9,35E-01
ENSRNOG00000001996	**Ythdc1**	YTH domain containing 1 (Ythdc1), ml	170956	14	22869119	22899609	30491	1666,84	-0,05	9,35E-01
ENSRNOG00000003103		lamin B receptor [Source:MGI Symbol;Acc:MGI::		13	106629770	106648432	18663	477,22	-0,06	9,35E-01
ENSRNOG00000010479		MYC binding protein 2 [Source:MGI S	290447	15	97165491	97355788	190298	4907,86	0,05	9,35E-01
ENSRNOG00000013657		stathmin-3 [Source:RefSeq peptide;A	29246	3	180518143	180526259	8117	24471,84	0,09	9,35E-01
ENSRNOG00000018039	**Sncb**	synuclein, beta (Sncb), mRNA [Source	113893	17	12510521	12518727	8207	4472,51	0,06	9,35E-01
ENSRNOG00000020086		Protein Aar2; RGD1311066 protein; Si	296312	3	159044400	159067330	22931	1891,92	0,04	9,35E-01
ENSRNOG00000000123	**Rnf19b**	ring finger protein 19B (Rnf19b), mRN	313806	5	150992439	151005653	13215	1097,94	0,06	9,35E-01
ENSRNOG00000006604	**Thy1**	Thy-1 cell surface antigen (Thy1), mR	24832	8	46998623	47003437	4815	896,73	-0,06	9,35E-01
ENSRNOG00000014723	**Cbfa2t3**	core-binding factor, runt domain, alpha	361431	19	66150087	66218763	68677	356,75	-0,06	9,35E-01
ENSRNOG00000005243	**Pop1**	processing of precursor 1, ribonucleas	315045	7	73436519	73473073	36555	384,97	-0,06	9,35E-01
ENSRNOG00000003946	**Tor1aip1**	torsin A interacting protein 1 (Tor1aip1	246314	13	78595698	78629717	34020	494,87	0,06	9,36E-01
ENSRNOG00000004158	**Hdac9**	histone deacetylase 9 (Hdac9), mRNA	687001	6	63111204	63679614	568411	703,38	0,07	9,36E-01
ENSRNOG00000007034	**Hipk2**	homeodomain interacting protein kinas	362342	4	66255897	66435382	179486	2295,68	0,04	9,36E-01
ENSRNOG00000007158	**Qtrt1**	queuine tRNA-ribosyltransferase 1 (Qt	64016	8	22501169	22508439	7271	416,49	0,05	9,36E-01
ENSRNOG00000010417	**RGD15635**	Protein RGD1563510 [Source:UniPro	362240	3	155172218	155195270	23053	630,23	-0,06	9,36E-01
ENSRNOG00000014808	**Srfbp1**	serum response factor binding protein	291469	18	46669390	46690468	21079	331,90	0,09	9,36E-01
ENSRNOG00000014911		Protein RGD1310081; RGD1310081 p	310137	2	78935591	78957358	21768	325,21	-0,06	9,36E-01
ENSRNOG00000029244	**Pcdhb10**	protocadherin beta 10 (Pcdhb10), mR	291649	18	30194692	30197239	2548	149,69	0,08	9,36E-01
ENSRNOG00000020372	**Hdac4**	histone deacetylase 4 (Hdac4), mRNA	363287	9	98731874	98973785	241912	768,89	0,05	9,36E-01
ENSRNOG00000028341	**Alkbh5**	alkB, alkylation repair homolog 5 (E. c	303193	10	46679920	46700727	20808	539,75	0,05	9,36E-01
ENSRNOG00000002091	**Gtf3c3**	general transcription factor IIIC, polype	316810	9	60683825	60717610	33786	575,20	0,06	9,36E-01
ENSRNOG00000006690		transmembrane protein 168 [Source:I	312135	4	39840557	39863946	23390	534,27	-0,07	9,36E-01
ENSRNOG00000016828	**Cmtm5**	CKLF-like MARVEL transmembrane domain cor		15	37486998	37489784	2787	803,03	0,09	9,36E-01
ENSRNOG00000004345	**Daam1**	dishevelled associated activator of mo	314212	6	104080527	104183583	103057	4142,05	-0,05	9,36E-01
ENSRNOG00000005854	**Angpt1**	angiopoietin 1 (Angpt1), mRNA [Sourc	89807	7	81368937	81618369	249433	149,41	-0,11	9,36E-01
ENSRNOG00000006839		ADP-ribosylation factor-like protein 5A	117050	3	43024477	43046967	22491	1744,97	0,07	9,36E-01
ENSRNOG00000015568	**Ppapdc1b**	phosphatidic acid phosphatase type 2	680466	16	70782080	70786221	4142	400,93	-0,06	9,36E-01
ENSRNOG00000019476	**Agap1**	ArfGAP with GTPase domain, ankyrin	316611	9	96438897	96719918	281022	4081,90	0,05	9,36E-01
ENSRNOG00000025025		deoxynucleotidyltransferase, terminal,	1E+08	2	243890081	243901563	11483	281,51	-0,10	9,36E-01
ENSRNOG00000025680		Nipped-B homolog (Drosophila) [Sour	294787	2	78994110	79078582	84473	1165,95	0,10	9,36E-01
ENSRNOG00000001620		Protein Prdm15 [Source:UniProtKB/T	304054	11	41806821	41860803	53983	373,66	0,06	9,36E-01

ENSRNOG00000004185	**Mon2**	MON2 homolog (S. cerevisiae) (Mon2	314894	7	66915094	66990456	75363	1667,09	-0,05	9,36E-01
ENSRNOG00000008117	**Galnt11**	UDP-N-acetyl-alpha-D-galactosamine	311952	4	6318189	6351691	33503	2661,73	0,08	9,36E-01
ENSRNOG00000009474	**Ddx42**	DEAD (Asp-Glu-Ala-Asp) box helicase	303607	10	94158861	94190354	31494	3168,20	-0,04	9,36E-01
ENSRNOG00000012287	**Slc35e1**	solute carrier family 35, member E1 (S	498599	16	18915079	18927347	12269	2081,85	0,05	9,36E-01
ENSRNOG00000012640	**Dpp7**	dipeptidylpeptidase 7 (Dpp7), mRNA [:	83799	3	2550548	2554800	4253	4435,61	-0,08	9,36E-01
ENSRNOG00000016935	**Mapk1ip1**	mitogen-activated protein kinase 1 inte	499280	1	218122520	218123311	792	262,23	-0,08	9,36E-01
ENSRNOG00000018874	**Phf19**	PHD finger protein 19 (Phf19), mRNA	296653	3	19276705	19297793	21089	164,27	0,06	9,36E-01
ENSRNOG00000019673	**Zfp36**	zinc finger protein 36 (Zfp36), mRNA [	79426	1	86595418	86597898	2481	191,38	-0,11	9,36E-01
ENSRNOG00000025634	**Zfhx2**	zinc finger homeobox 2 (Zfhx2), mRN/	305888	15	37607644	37639750	32107	501,70	-0,10	9,36E-01
ENSRNOG00000027502	**Nmi**	N-myc (and STAT) interactor (Nmi), m	311021	3	42562212	42584322	22111	48,14	0,10	9,36E-01
ENSRNOG00000029490		zinc finger protein 768 [Source:MGI Symbol;Acc		1	205791203	205793534	2332	732,69	0,06	9,36E-01
ENSRNOG00000033176	**Zrsr2**	Protein Zrsr2 [Source:UniProtKB/TrEI	302670	X	32667263	32689259	21997	557,83	-0,06	9,36E-01
ENSRNOG00000042988	**Hist3h2a**	histone cluster 3, H2a (Hist3h2a), mRI	64646	10	45054233	45055784	1552	1359,84	0,07	9,36E-01
ENSRNOG00000009330	**Slc17a5**	solute carrier family 17 (acidic sugar tr	363103	8	85440757	85475700	34944	588,13	0,08	9,36E-01
ENSRNOG00000004786	**Cyp4f1**	cytochrome P450, family 4, subfamily	56266	7	15338348	15349540	11193	74,34	-0,09	9,36E-01
ENSRNOG00000003832	**Vash2**	vasohibin 2 (Vash2), mRNA [Source:R	498309	13	114122604	114154107	31504	2089,12	0,08	9,36E-01
ENSRNOG00000013469	**Txndc5**	thioredoxin domain containing 5 (endc	1E+08	17	28841101	28867877	26777	1904,21	-0,07	9,36E-01
ENSRNOG00000010483		Protein Fstl5 [Source:UniProtKB/TrEN	365823	2	193937492	194332727	395236	185,19	-0,07	9,36E-01
ENSRNOG00000001551	**Atp5j**	ATP synthase, H+ transporting, mitoch	94271	11	27914800	27918237	3438	1803,62	0,06	9,37E-01
ENSRNOG00000001866	**Pi4ka**	phosphatidylinositol 4-kinase, catalytic	64161	11	90914592	91028242	113651	4799,63	-0,05	9,37E-01
ENSRNOG00000004577		Fasciculation and elongation protein zeta-2; Fez		6	956475	995426	38952	483,52	0,06	9,37E-01
ENSRNOG00000006217	**Wiz**	widely-interspaced zinc finger motifs (\	314598	7	14528651	14556359	27709	2408,35	-0,06	9,37E-01
ENSRNOG00000011094	**Efcab6**	Protein Efcab6 [Source:UniProtKB/Tr	315179	7	124748217	124910667	162451	106,27	0,08	9,37E-01
ENSRNOG00000014945	**Kpna3**	karyopherin alpha 3 (Kpna3), mRNA [S	361055	15	45502394	45574246	71853	1281,87	-0,08	9,37E-01
ENSRNOG00000018691		Cyclin-L2 [Source:UniProtKB/Swiss-F	298686	5	176732433	176743690	11258	1997,03	0,10	9,37E-01
ENSRNOG00000019661	**Gdf15**	growth differentiation factor 15 (Gdf15	29455	16	20411167	20413750	2584	105,94	-0,10	9,37E-01
ENSRNOG00000021255	**RGD15644**	spermine oxidase (Smox), mRNA [Sou	308652	3	130580816	130594899	14084	422,59	0,06	9,37E-01
ENSRNOG00000023431	**RGD15612**	Protein RGD1561277 [Source:UniPro	691468	10	44964718	44965590	873	35,92	0,08	9,37E-01
ENSRNOG00000027451	**Mettl25**	methyltransferase like 25 (Mettl25), m	314798	7	47862494	47942719	80226	159,18	-0,06	9,37E-01
ENSRNOG00000049402	**Nbl1**	neuroblastoma 1, DAN family BMP an	50594	5	161263767	161274897	11131	4797,20	-0,09	9,37E-01
ENSRNOG00000004559	**LOC10091(**	tubulin, alpha 1A (Tuba1a), mRNA [So	64158	X	115143855	115147552	3698	32699,50	0,07	9,37E-01
ENSRNOG00000019311	**Nfkb2**	nuclear factor of kappa light polypeptic	309452	1	273483943	273490197	6255	211,66	0,08	9,37E-01
ENSRNOG00000000420	**Nelfe**	negative elongation factor complex me	294258	20	6610221	6615882	5662	815,67	-0,05	9,37E-01
ENSRNOG00000002855	**Rfc1**	replication factor C (activator 1) 1 (Rfc	89809	14	44397927	44516383	118457	644,11	-0,09	9,37E-01
ENSRNOG00000008674		PR domain containing 11 [Source:MGI Symbol;A		3	88574911	88613187	38277	91,15	-0,07	9,37E-01
ENSRNOG00000010131	**Rin2**	Ras and Rab interactor 2 (Rin2), mRN	311494	3	146317465	146510342	192878	571,70	-0,07	9,37E-01
ENSRNOG00000011559	**Cnn3**	calponin 3, acidic (Cnn3), mRNA [Sou	54321	2	243044080	243075190	31111	9469,36	-0,06	9,37E-01
ENSRNOG00000017914	**Prkcdbp**	protein kinase C, delta binding protein	85332	1	177323467	177325064	1598	925,57	0,10	9,37E-01

ENSRNOG00000018697	**Abcb6**	ATP-binding cassette, subfamily B (MI	140669	9	82143209	82151515	8307	1250,31	0,04	9,37E-01
ENSRNOG00000020232	**Rit1**	Ras-like without CAAX 1 (Rit1), mRNA	499652	2	207487476	207498920	11445	1173,41	-0,06	9,37E-01
ENSRNOG00000046420		Uncharacterized protein [Source:UniProtKB/TrE		1	242690781	242723675	32895	113,35	0,10	9,37E-01
ENSRNOG00000049391		Protein Cdkal1 [Source:UniProtKB/Trl	361243	17	37843673	37934819	91147	31,47	-0,09	9,37E-01
ENSRNOG00000005548	**RGD13051**	Uncharacterized protein KIAA1841 ho	305579	14	105141275	105211491	70217	773,76	-0,08	9,37E-01
ENSRNOG00000007185		leiomodin-1 [Source:RefSeq peptide;/	304816	13	57197927	57237507	39581	66,63	-0,10	9,37E-01
ENSRNOG00000007338		Fibulin 2, isoform CRA_b; Protein Fblr	282583	4	187651235	187690477	39243	2050,97	-0,09	9,37E-01
ENSRNOG00000008151	**Plscr4**	phospholipid scramblase 4 (Plscr4), m	300900	8	99364963	99403085	38123	69,11	-0,10	9,37E-01
ENSRNOG00000012653	**Ppp1r13b**	protein phosphatase 1, regulatory sub	314465	6	145405635	145444597	38963	1677,78	-0,06	9,37E-01
ENSRNOG00000005350	**Pwp1**	PWP1 homolog (S. cerevisiae) (Pwp1	362856	7	24138856	24154854	15999	852,00	0,07	9,37E-01
ENSRNOG00000033737		Protein Pkd1l3 [Source:UniProtKB/Trl	1E+08	19	52814669	52879420	64752	24,74	0,10	9,37E-01
ENSRNOG00000001073	**RGD15634**	uncharacterized protein LOC498179 |	498179	12	39552271	39555685	3415	60,07	-0,08	9,37E-01
ENSRNOG00000005795	**Aasdhppt**	aminoadipate-semialdehyde dehydrog	300328	8	1433781	1444562	10782	806,85	0,05	9,37E-01
ENSRNOG00000014410		mortality factor 4 like 1 (Morf4l1), mRN	300891	8	96974730	96995688	20959	1317,79	0,05	9,37E-01
ENSRNOG00000015585	**Suv39h2**	suppressor of variegation 3-9 homolog	364785	17	80406940	80425965	19026	284,72	0,08	9,37E-01
ENSRNOG00000017506	**Cltb**	clathrin, light chain B (Cltb), mRNA [Sc	116561	17	12665098	12682747	17650	4339,46	0,06	9,37E-01
ENSRNOG00000019730		inositol polyphosphate phosphatase-like 1 [Sour		1	173089239	173101408	12170	1525,01	0,06	9,37E-01
ENSRNOG00000000303	**Cep57l1**	centrosomal protein 57-like 1 (Cep57l	294519	20	48291080	48346877	55798	176,33	-0,07	9,37E-01
ENSRNOG00000001391		serine dehydratase-like [Source:MGI Symbol;Ac		12	43487966	43497281	9316	109,20	-0,09	9,37E-01
ENSRNOG00000002735	**Dhx9**	DEAH (Asp-Glu-Ala-His) box helicase	304859	13	75851157	75887898	36742	4570,39	-0,07	9,37E-01
ENSRNOG00000003131	**Traf7**	TNF receptor-associated factor 7, E3 ι	360491	10	13691946	13701566	9621	1804,97	0,05	9,37E-01
ENSRNOG00000003845	**Wnt3**	wingless-type MMTV integration site fa	24882	10	91597106	91641213	44108	28,01	-0,09	9,37E-01
ENSRNOG00000031003		RCG40489; Uncharacterized protein [Source:U		1	205835438	205837482	2045	116,63	-0,07	9,37E-01
ENSRNOG00000015686	**Col4a3bp**	collagen, type IV, alpha 3 (Goodpastur	365652	2	46477805	46582436	104632	1302,87	-0,06	9,37E-01
ENSRNOG00000000158	**Cdo1**	cysteine dioxygenase type 1 (Cdo1), r	81718	18	40348061	40362704	14644	1630,43	-0,05	9,37E-01
ENSRNOG00000005276	**Csnk2a1**	casein kinase 2, alpha 1 polypeptide (	116549	3	154062024	154105941	43918	3505,01	0,07	9,37E-01
ENSRNOG00000008393		Tax1 (human T-cell leukemia virus typ	246244	4	147442105	147499135	57031	1375,62	-0,10	9,37E-01
ENSRNOG00000010753	**Aig1**	androgen-induced 1 (Aig1), mRNA [Sc	292486	1	9445190	9665307	220118	1188,55	0,05	9,37E-01
ENSRNOG00000018785	**Slc16a13**	solute carrier family 16, member 13 (S	287451	10	56571908	56575557	3650	313,66	0,06	9,37E-01
ENSRNOG00000022171	**Dhx37**	DEAH (Asp-Glu-Ala-His) box polypept	288647	12	38472183	38492290	20108	852,69	-0,05	9,37E-01
ENSRNOG00000024580	**Mamstr**	Protein Mamstr [Source:UniProtKB/Tr	691041	1	102702088	102706389	4302	23,96	0,09	9,37E-01
ENSRNOG00000049123		Uncharacterized protein [Source:UniProtKB/TrE		9	8931868	8936411	4544	18,42	0,09	9,37E-01
ENSRNOG00000009719	**C2cd2l**	C2CD2-like (C2cd2l), mRNA [Source:I	300666	8	47259704	47269967	10264	2759,78	-0,06	9,37E-01
ENSRNOG00000013183	**Eif4e**	eukaryotic translation initiation factor 4	117045	2	262355381	262387511	32131	1205,77	0,06	9,37E-01
ENSRNOG00000001592	**Cct8**	chaperonin containing Tcp1, subunit 8	288305	11	30755620	30767329	11710	2934,72	0,09	9,38E-01
ENSRNOG00000027491	**Vldlr**	very low density lipoprotein receptor (\	25696	1	252487250	252518719	31470	926,39	0,07	9,38E-01
ENSRNOG00000007133	**Plxnb2**	plexin B2 (Plxnb2), mRNA [Source:Re	315217	7	129821099	129836927	15829	6282,24	0,05	9,38E-01
ENSRNOG00000018885		zinc finger protein 60 [Source:MGI Syr	1E+08	1	85810963	85824370	13408	25,79	-0,10	9,38E-01

ENSRNOG00000042851				7	15288822	15289202	381	27,22	0,08	9,38E-01
ENSRNOG00000009950		Myb/SANT-like DNA-binding domain containing		8	39961366	39994454	33089	543,35	0,08	9,38E-01
ENSRNOG00000017593	**Mtr**	5-methyltetrahydrofolate-homocystein	81522	17	67958262	68041430	83169	338,53	0,05	9,38E-01
ENSRNOG00000012014	**Fam160b2**	family with sequence similarity 160, m	306015	15	55995013	56012410	17398	700,73	-0,04	9,38E-01
ENSRNOG00000031213	**Slc39a11**	solute carrier family 39, member 11 (S	287796	10	101430090	101762931	332842	455,65	-0,07	9,38E-01
ENSRNOG00000033343	**Prss36**	protease, serine, 36 (Prss36), mRNA [	497040	1	206402346	206418143	15798	69,72	-0,07	9,39E-01
ENSRNOG00000009545	**Polr1a**	polymerase (RNA) I polypeptide A (Po	83581	4	164601148	164665028	63881	1063,60	0,05	9,39E-01
ENSRNOG00000029260	**Pitpnm2**	phosphatidylinositol transfer protein, m	304474	12	39751082	39776218	25137	3439,50	-0,04	9,39E-01
ENSRNOG00000048367	**LOC692035**			10	18552726	18553291	566	75,36	0,07	9,39E-01
ENSRNOG00000024460	**Tarsl2**	threonyl-tRNA synthetase-like 2 (Tarsl	308701	1	127615729	127651843	36115	428,04	-0,05	9,39E-01
ENSRNOG00000026293	**Jun**	jun proto-oncogene (Jun), mRNA [Sou	24516	5	117958961	117959965	1005	1239,88	-0,05	9,39E-01
ENSRNOG00000039819		cyclin-dependent kinase 8 [Source:Re	498140	12	12597402	12663828	66427	1030,33	-0,06	9,39E-01
ENSRNOG00000016386	**Ptms**	parathymosin (Ptms), mRNA [Source:l	83801	4	224453141	224457317	4177	16609,04	0,07	9,39E-01
ENSRNOG00000026040	**Prss53**	protease, serine, 53 (Prss53), mRNA [	499270	1	206354407	206358841	4435	47,67	-0,09	9,39E-01
ENSRNOG00000042427	**Gskip**	GSK3B interacting protein (Gskip), mF	362778	6	138712664	138728642	15979	463,26	0,07	9,39E-01
ENSRNOG00000003494	**Ppfia4**	protein tyrosine phosphatase, recepto	140592	13	56191119	56239610	48492	818,22	-0,06	9,39E-01
ENSRNOG00000018140	**Smad2**	SMAD family member 2 (Smad2), tran	29357	18	71672969	71734982	62014	1021,32	0,05	9,39E-01
ENSRNOG00000027622	**Slc35e3**	solute carrier family 35, member E3 (S	362883	7	60765042	60778112	13071	1738,84	0,05	9,39E-01
ENSRNOG00000001267		lanosterol synthase [Source:MGI Symbol;Acc:M		20	15001945	15027496	25552	2474,30	-0,08	9,39E-01
ENSRNOG00000019808	**RGD13111**	uncharacterized protein LOC297607 |	297607	4	232097436	232130662	33227	454,03	-0,04	9,39E-01
ENSRNOG00000024650	**LOC69117**	cytoskeleton associated protein 2 (Cka	306575	16	74383677	74410206	26530	232,55	-0,06	9,39E-01
ENSRNOG00000032391		extended synaptotagmin-like protein 2	299488	6	153092828	153181968	89141	1161,12	-0,08	9,39E-01
ENSRNOG00000009694	**Bmp4**	bone morphogenetic protein 4 (Bmp4)	25296	15	24735177	24738725	3549	141,09	-0,06	9,39E-01
ENSRNOG00000010475	**Casp3**	caspase 3 (Casp3), mRNA [Source:Re	25402	16	48560091	48578283	18193	1276,16	0,05	9,39E-01
ENSRNOG00000042653	**LOC69087**	RIKEN cDNA 1810043H04 gene [Sou	690871	10	108876795	108878946	2152	622,36	0,09	9,39E-01
ENSRNOG00000000322	**Atg5**	autophagy related 5 (Atg5), mRNA [Source:RefS		20	50951130	51026118	74989	883,83	0,05	9,40E-01
ENSRNOG00000002061	**Ptpn13**	protein tyrosine phosphatase, non-rec	498331	14	7662574	7837443	174870	602,25	-0,06	9,40E-01
ENSRNOG00000003890	**Nap1l1**	nucleosome assembly protein 1-like 1	89825	7	54238052	54261360	23309	4478,40	-0,05	9,40E-01
ENSRNOG00000008234	**Lrrc61**	leucine rich repeat containing 61 (Lrrc	500111	4	142850801	142870502	19702	746,15	-0,05	9,40E-01
ENSRNOG00000010853	**Chrna7**	cholinergic receptor, nicotinic, alpha 7	25302	1	125028876	125158170	129295	492,27	0,08	9,40E-01
ENSRNOG00000019484	**Slc6a9**	solute carrier family 6 (neurotransmitte	116509	5	140458723	140492746	34024	1578,17	-0,07	9,40E-01
ENSRNOG00000021338	**Tmem132a**	transmembrane protein 132A (Tmem1	338474	1	233978360	233989649	11290	10581,83	-0,05	9,40E-01
ENSRNOG00000014928	**Apba1**	amyloid beta (A4) precursor protein-bi	83589	1	248875910	249076645	200736	5354,22	-0,05	9,40E-01
ENSRNOG00000008843	**Eci1**	enoyl-CoA delta isomerase 1 (Eci1), m	29740	10	13614548	13627899	13352	1140,11	0,08	9,40E-01
ENSRNOG00000014670		zinc finger, MYND-type containing 11 (	291259	17	65590225	65642353	52129	4992,44	-0,05	9,40E-01
ENSRNOG00000031174	**Arnt**	aryl hydrocarbon receptor nuclear tran	25242	2	216092641	216149878	57238	389,43	-0,05	9,40E-01
ENSRNOG00000025338	**Msi2**	Protein Msi2 [Source:UniProtKB/TrEM	360596	10	74071857	74427908	356052	859,27	-0,07	9,40E-01
ENSRNOG00000004700	**Nxt1**	NTF2-like export factor 1 (Nxt1), mRN	296219	3	149404160	149407137	2978	334,88	0,07	9,40E-01

ENSRNOG00000016645	**Sfmbt1**	Scm-like with four mbt domains 1 (Sfm	58967	16	6708903	6821329	112427	772,02	0,06	9,40E-01
ENSRNOG00000023573	**Ccpg1**	cell cycle progression 1 (Ccpg1), mRN	363098	8	73397117	73429325	32209	1108,52	-0,06	9,40E-01
ENSRNOG00000008309	**Rpa3**	replication protein A3 (Rpa3), mRNA [	296883	4	34139498	34142529	3032	359,41	-0,06	9,40E-01
ENSRNOG00000020182	**Mvp**	major vault protein (Mvp), mRNA [Sou	64681	1	205401191	205428861	27671	354,76	0,09	9,41E-01
ENSRNOG00000030830	**Mex3d**	Protein Mex3d [Source:UniProtKB/TrE	299613	7	12361697	12367764	6068	1516,82	0,07	9,41E-01
ENSRNOG00000016751	**Slc25a28**	solute carrier family 25 (mitochondrial	688811	1	270865549	270876783	11235	627,07	0,05	9,41E-01
ENSRNOG00000028328	**Tyw3**	Protein Tyw3; Similar to RIKEN cDNA	499731	2	279523743	279545826	22084	117,70	0,05	9,41E-01
ENSRNOG00000005053	**Egln3**	EGL nine homolog 3 (C. elegans) (Egl	54702	6	84592894	84618360	25467	1180,53	0,08	9,41E-01
ENSRNOG00000004011	**Nedd1**	neural precursor cell expressed, devel	299730	7	33615235	33655544	40310	264,77	-0,06	9,41E-01
ENSRNOG00000016665	**Tpra1**	transmembrane protein, adipocyte ass	85494	4	186084105	186095214	11110	505,09	0,05	9,41E-01
ENSRNOG00000018755	**Acss2**	acyl-CoA synthetase short-chain famil	311569	3	157400421	157443341	42921	1196,60	-0,06	9,41E-01
ENSRNOG00000012982	**Map9**	microtubule-associated protein 9 (Map	310544	2	200743299	200775030	31732	1658,60	-0,05	9,41E-01
ENSRNOG00000001720	**Hes1**	hairy and enhancer of split 1 (Drosoph	29577	11	77360110	77362537	2428	364,09	-0,07	9,42E-01
ENSRNOG00000008672	**Chmp5**	charged multivesicular body protein 5	297995	5	61798614	61815784	17171	1471,29	0,05	9,42E-01
ENSRNOG00000010306	**H2afz**	H2A histone family, member Z (H2afz)	58940	2	261653914	261655196	1283	696,29	0,07	9,42E-01
ENSRNOG00000019212	**Tpp1**	tripeptidyl peptidase I (Tpp1), mRNA [	83534	1	177593887	177600020	6134	2099,59	-0,07	9,42E-01
ENSRNOG00000003048	**Tiprl**	TIP41, TOR signaling pathway regulat	360869	13	88358553	88384034	25482	1714,82	0,06	9,42E-01
ENSRNOG00000015670	**LOC10091**	syntaxin 7 (Stx7), mRNA [Source:RefS	60466	1	23721818	23761945	40128	1265,56	-0,06	9,42E-01
ENSRNOG00000018694	**Lipg**	lipase, endothelial (Lipg), mRNA [Sour	291437	18	70043855	70064759	20905	119,92	-0,08	9,42E-01
ENSRNOG00000000785	**Trim39**	tripartite motif-containing 39 (Trim39),	309591	20	4530743	4544598	13856	428,70	0,06	9,42E-01
ENSRNOG00000005492	**Hpcal1**	hippocalcin-like 1 (Hpcal1), mRNA [Sc	50871	6	52713426	52818871	105446	847,63	-0,07	9,42E-01
ENSRNOG00000008734		zinc finger, MYM-type 2 [Source:MGI Symbol;Ac		15	41020722	41075984	55263	1258,48	-0,08	9,42E-01
ENSRNOG00000009856	**Sypl1**	synaptophysin-like 1 (Sypl1), mRNA [S	366595	6	61891964	61910838	18875	706,01	-0,05	9,42E-01
ENSRNOG00000018410	**Nfu1**	NFU1 iron-sulfur cluster scaffold homo	297416	4	183383769	183404239	20471	1002,49	-0,05	9,42E-01
ENSRNOG00000018960		spectrin repeat containing, nuclear env	499010	1	42947845	43100865	153021	1032,51	-0,04	9,42E-01
ENSRNOG00000025388	**Mrpl33**	Brain and reproductive organ-express	1E+08	6	35954540	35962710	8171	821,32	0,07	9,42E-01
ENSRNOG00000008704	**Nlk**	nemo like kinase (Nlk), mRNA [Source	497961	10	65679688	65803568	123881	398,01	0,07	9,42E-01
ENSRNOG00000007272	**Rnf103**	ring finger protein 103 (Rnf103), mRN	84508	4	164177797	164194495	16699	1651,67	0,07	9,42E-01
ENSRNOG00000028774	**Vgll3**	Protein Vgll3 [Source:UniProtKB/TrEM	498038	11	3097621	3142253	44633	42,01	0,08	9,42E-01
ENSRNOG00000048430	**Myo18b**	Protein Myo18b [Source:UniProtKB/T	304551	12	51538331	51737732	199402	19,10	-0,09	9,42E-01
ENSRNOG00000049768	**Adcy9**	adenylate cyclase 9 (Adcy9), mRNA [S	302950	10	10152945	10164223	11279	128,28	0,07	9,42E-01
ENSRNOG00000012311	**Slc35d3**	solute carrier family 35, member D3 (S	308717	1	16846809	16850281	3473	68,68	0,10	9,42E-01
ENSRNOG00000010495	**Ttc17**	tetratricopeptide repeat domain 17 (Tt	311224	3	89898709	90006207	107499	1316,88	-0,06	9,42E-01
ENSRNOG00000030102	**Mboat4**	membrane bound O-acyltransferase d	306515	16	61448506	61456800	8295	21,85	-0,09	9,42E-01
ENSRNOG00000002208	**Shroom3**	shroom family member 3 (Shroom3), r	305230	14	16547964	16816352	268389	976,09	0,07	9,43E-01
ENSRNOG00000005369	**Kcnj3**	potassium inwardly-rectifying channel,	50599	3	46103575	46266134	162560	418,63	-0,06	9,43E-01
ENSRNOG00000011253	**RGD13048**	similar to 6430573F11Rik protein (RG	306504	16	58998370	59047495	49126	110,65	-0,05	9,43E-01
ENSRNOG00000015906	**Tgif1**	TGFB-induced factor homeobox 1 (Tg	316742	9	118636412	118646122	9711	246,29	-0,08	9,43E-01

ENSRNOG00000017295		SH3-domain GRB2-like endophilin B2 [Source:M		3	14262005	14276806	14802	2644,03	0,06	9,43E-01
ENSRNOG00000017930	**Lpcat1**	lysophosphatidylcholine acyltransfera:	361467	1	33802633	33853118	50486	3486,61	-0,08	9,43E-01
ENSRNOG00000018296	**Anapc10**	anaphase promoting complex subunit	361389	19	42719406	42771825	52420	721,25	-0,06	9,43E-01
ENSRNOG00000027955	**Zfp133**	zinc finger protein 133 (Zfp133), mRN.	499900	3	145027552	145046045	18494	176,84	-0,07	9,43E-01
ENSRNOG00000033515	**LOC10036**	Da1-10-like (LOC100366054), mRNA	1E+08	13	92913183	92937227	24045	30,47	-0,09	9,43E-01
ENSRNOG00000004440	**Fignl1**	fidgetin-like 1 (Fignl1), mRNA [Source	289777	14	91679884	91688408	8525	37,88	-0,08	9,43E-01
ENSRNOG00000030170	**Krt10**	keratin 10 (Krt10), mRNA [Source:Ref	450225	10	87092253	87096248	3996	91,42	-0,07	9,43E-01
ENSRNOG00000004100	**Trib1**	tribbles homolog 1 (Drosophila) (Trib1	78969	7	100534391	100540925	6535	184,55	0,06	9,43E-01
ENSRNOG00000029642	**LOC100909892**			1	88809379	88809766	388	780,67	0,04	9,43E-01
ENSRNOG00000000633	**Rhobtb1**	Rho-related BTB domain containing 1	309722	20	22756929	22831478	74550	390,97	0,06	9,43E-01
ENSRNOG00000004077		potassium voltage gated channel, Sha	246153	7	54999433	55177445	178013	243,24	0,08	9,43E-01
ENSRNOG00000004219	**Dhps**	deoxyhypusine synthase (Dhps), mRN	288923	19	37160274	37164382	4109	1635,60	-0,06	9,43E-01
ENSRNOG00000004695	**Celf5**	CUGBP, Elav-like family member 5 (C	314647	7	11262657	11287130	24474	6660,92	-0,05	9,43E-01
ENSRNOG00000005426	**Snupn**	snurportin 1 (Snupn), mRNA [Source:I	316108	8	60185056	60217869	32814	290,07	-0,06	9,43E-01
ENSRNOG00000007964	**Tp53inp1**	tumor protein p53 inducible nuclear pr	297822	5	29136810	29142800	5991	224,34	0,07	9,43E-01
ENSRNOG00000009209	**Slitrk1**	SLIT and NTRK-like family, member 1	306147	15	96793133	96797171	4039	1897,97	0,07	9,43E-01
ENSRNOG00000010005	**Adck2**	aarF domain containing kinase 2 (Adc	312258	4	67166683	67179568	12886	383,54	0,07	9,43E-01
ENSRNOG00000010910	**Gmeb1**	glucocorticoid modulatory element bin	500558	5	154078313	154108562	30250	403,03	0,05	9,43E-01
ENSRNOG00000010986	**Cmtm7**	CKLF-like MARVEL transmembrane d	501065	8	122126648	122153564	26917	23,68	0,10	9,43E-01
ENSRNOG00000011921	**Dusp4**	dual specificity phosphatase 4 (Dusp4	60587	16	60750785	60760965	10181	561,67	0,08	9,43E-01
ENSRNOG00000012659	**Vamp5**	vesicle-associated membrane protein	89818	4	165002077	165013304	11228	32,02	0,09	9,43E-01
ENSRNOG00000014268	**Abca2**	ATP-binding cassette, subfamily A (AE	79248	3	2630278	2650182	19905	3318,78	-0,04	9,43E-01
ENSRNOG00000014452	**Zfhx3**	Protein Zfhx3 [Source:UniProtKB/TrE	307829	19	53585376	53608565	23190	200,36	0,05	9,43E-01
ENSRNOG00000014927	**Zcchc9**	zinc finger, CCHC domain containing !	309986	2	48035517	48044197	8681	546,76	0,07	9,43E-01
ENSRNOG00000015508	**RGD13072**	similar to RIKEN cDNA 2310035C23 (	309053	13	30820433	30915037	94605	2336,77	-0,06	9,43E-01
ENSRNOG00000016511	**Pitrm1**	pitrilysin metallopeptidase 1 (Pitrm1), i	307081	17	70194249	70225893	31645	1124,31	-0,05	9,43E-01
ENSRNOG00000018166	**Prkab2**	protein kinase, AMP-activated, beta 2	64562	2	219310175	219322360	12186	387,24	0,06	9,43E-01
ENSRNOG00000019012	**D2hgdh**	D-2-hydroxyglutarate dehydrogenase	301624	9	100609556	100626136	16581	386,35	0,05	9,43E-01
ENSRNOG00000020714	**Trpm4**	transient receptor potential cation chai	171143	1	102357043	102386710	29668	377,36	0,05	9,43E-01
ENSRNOG00000024640	**Oacyl**	O-acyltransferase like (Oacyl), mRNA	291549	18	60573908	60629730	55823	21,28	0,09	9,43E-01
ENSRNOG00000027040	**Ggact**	gamma-glutamylamine cyclotransfera:	290500	15	112688814	112712357	23544	116,51	-0,08	9,43E-01
ENSRNOG00000028273	**Tmem150b**	transmembrane protein 150B (Tmem1	308346	1	75833876	75842777	8902	336,57	-0,06	9,43E-01
ENSRNOG00000030748	**LOC10036:**	Protein LOC100362827 [Source:UniP	1E+08	2	79213431	79213694	264	66,83	0,07	9,43E-01
ENSRNOG00000032895	**Cyp4f4**	cytochrome P450, family 4, subfamily	286904	7	15045084	15061271	16188	59,01	0,06	9,43E-01
ENSRNOG00000017017	**Zbtb43**	zinc finger and BTB domain containing	311872	3	17825499	17834966	9468	261,68	0,06	9,43E-01
ENSRNOG00000021521	**Chst5**	carbohydrate (N-acetylglucosamine 6-	307859	19	54922197	54942511	20315	84,64	-0,07	9,43E-01
ENSRNOG00000000989	**Bud31**	BUD31 homolog (S. cerevisiae) (Bud3	89819	12	13302069	13309503	7435	717,44	0,07	9,43E-01
ENSRNOG00000010802	**Ube3d**	ubiquitin protein ligase E3D (Ube3d), i	315863	8	93463078	93633601	170524	155,78	0,06	9,43E-01

ENSRNOG00000019525	**Hspa9**	heat shock protein 9 (Hspa9), mRNA [	291671	18	27441855	27459272	17418	2501,78	-0,08	9,43E-01
ENSRNOG00000016443	**Vps13d**	vacuolar protein sorting 13 homolog D	313825	5	166572596	166798221	225626	2572,72	0,04	9,43E-01
ENSRNOG00000022784				12	33681223	33683082	1860	40,51	-0,09	9,43E-01
ENSRNOG00000046654	**Crym**	crystallin, mu (Crym), mRNA [Source:I	117024	1	196874276	196889820	15545	3524,76	-0,07	9,43E-01
ENSRNOG00000010596		glycine C-acetyltransferase (2-amino-3	1E+08	7	120254440	120260146	5707	271,95	-0,07	9,44E-01
ENSRNOG00000012939	**Abca7**	ATP-binding cassette, subfamily A (AE	299609	7	12912237	12930983	18747	164,70	0,06	9,44E-01
ENSRNOG00000001792	**Slc12a8**	solute carrier family 12, member 8 (Slc	266733	11	73432331	73585559	153229	20,77	-0,09	9,44E-01
ENSRNOG00000006226	**Cacng2**	calcium channel, voltage-dependent, g	84347	7	119222819	119343985	121167	338,93	-0,07	9,44E-01
ENSRNOG00000006278	**Tspyl5**	Protein Tspyl5 [Source:UniProtKB/TrE	314555	7	72497606	72498838	1233	503,78	-0,06	9,44E-01
ENSRNOG00000010944	**Hyou1**	hypoxia up-regulated 1 (Hyou1), trans	192235	8	47318957	47330880	11924	3706,61	0,05	9,44E-01
ENSRNOG00000011614	**Tmcc1**	Protein LOC100912698 [Source:UniP	312654	4	211205163	211332130	126968	1052,61	-0,06	9,44E-01
ENSRNOG00000013449	**RGD15637**	Protein RGD1563753 [Source:UniProtKB/TrEM		3	52858678	52863881	5204	37,63	-0,08	9,44E-01
ENSRNOG00000014605	**Lig4**	ligase IV, DNA, ATP-dependent (Lig4),	290907	16	84770909	84776812	5904	187,98	0,08	9,44E-01
ENSRNOG00000018391	**Cdk2ap2**	cyclin-dependent kinase 2 associated	688405	1	226216692	226218360	1669	830,52	-0,07	9,44E-01
ENSRNOG00000018686	**Atxn2l**	ataxin 2-like (Atxn2l), mRNA [Source:F	361649	1	204886854	204898240	11387	4726,09	0,04	9,44E-01
ENSRNOG00000020673	**Pbxip1**	pre-B-cell leukemia homeobox interac	310644	2	208180635	208190539	9905	2245,80	-0,07	9,44E-01
ENSRNOG00000021302	**Tmem91**	Protein Tmem91 [Source:UniProtKB/T	1E+08	1	83725532	83729572	4041	166,27	-0,07	9,44E-01
ENSRNOG00000026963	**Hsp90b1**	heat shock protein 90, beta, member 1	362862	7	27345730	27359757	14028	5839,92	-0,07	9,44E-01
ENSRNOG00000029049	**Zdhhc14**	zinc finger, DHHC-type containing 14 (	499014	1	47692788	47953571	260784	448,04	-0,06	9,44E-01
ENSRNOG00000047010		Uncharacterized protein [Source:UniProtKB/TrE		2	168917166	168964460	47295	110,49	-0,06	9,44E-01
ENSRNOG00000049076	**Vwc2**	von Willebrand factor C domain contai	501231	14	90987662	91137489	149828	135,24	-0,06	9,44E-01
ENSRNOG00000020878	**Keap1**	Kelch-like ECH-associated protein 1 (I	117519	8	22306484	22313655	7172	1589,16	0,04	9,44E-01
ENSRNOG00000031247	**Lekr1**	leucine, glutamate and lysine rich 1 (L	361953	2	176821695	176987915	166221	36,07	-0,08	9,44E-01
ENSRNOG00000002660	**Magee1**	melanoma antigen, family E, 1 (Magee	317232	X	76881860	76885407	3548	2497,62	0,05	9,44E-01
ENSRNOG00000027841	**Mical3**	microtubule associated monoxygenase	362427	4	220573991	220682835	108845	1826,40	0,04	9,44E-01
ENSRNOG00000004063		Protein RGD1309926 [Source:UniPro	303021	10	17104828	17187475	82648	1190,89	-0,06	9,44E-01
ENSRNOG00000016841		glutamic pyruvate transaminase (alani	307759	19	33596261	33638298	42038	1222,61	0,05	9,44E-01
ENSRNOG00000017354	**Zyx**	zyxin (Zyx), mRNA [Source:RefSeq m	114636	4	136544930	136553784	8855	1210,63	0,05	9,44E-01
ENSRNOG00000029077	**LOC10091**	dolichyl-phosphate mannosyltransfera	502017	2	207997009	207997524	516	28,86	0,09	9,44E-01
ENSRNOG00000048043	**F2r**	coagulation factor II (thrombin) recepto	25439	2	45256548	45257759	1212	927,58	-0,06	9,44E-01
ENSRNOG00000008534		dual specificity phosphatase 15 (Dusp	362238	3	154820985	154831486	10502	640,70	-0,07	9,44E-01
ENSRNOG00000013977	**Bzw1**	basic leucine zipper and W2 domains	363232	9	65086973	65098942	11970	2629,42	-0,08	9,44E-01
ENSRNOG00000009049	**Usp11**	ubiquitin specific peptidase 11 (Usp11	408217	X	2481248	2497422	16175	4943,97	0,05	9,45E-01
ENSRNOG00000011194	**Tpp2**	tripeptidyl peptidase II (Tpp2), mRNA		81815	9	50328789	50409442	80654	1303,57	-0,06	9,45E-01
ENSRNOG00000011375	**Tsc2**	tuberous sclerosis 2 (Tsc2), mRNA [So	24855	10	13778991	13813550	34560	2171,27	0,04	9,45E-01
ENSRNOG00000011857	**Mtpn**	myotrophin (Mtpn), mRNA [Source:Re	79215	4	62731234	62760270	29037	11253,31	0,06	9,45E-01
ENSRNOG00000013118	**Atox1**	antioxidant 1 copper chaperone (Atox1	84355	10	40622328	40637386	15059	1149,86	-0,08	9,45E-01
ENSRNOG00000001353	**Ap4m1**	adaptor-related protein complex 4, mu	304344	12	21371823	21377987	6165	389,67	0,06	9,45E-01

ENSRNOG00000010637	**Tbc1d5**	TBC1 domain family, member 5 (Tbc1	501088	9	1106437	1704245	597809	849,31	0,05	9,45E-01
ENSRNOG00000010760	**E2f5**	Transcription factor E2F5 [Source:Un	116651	2	108121173	108136514	15342	479,31	-0,05	9,45E-01
ENSRNOG00000014130	**LOC10036:**	CDC28 protein kinase regulatory subu	498709	17	15662366	15667466	5101	225,23	0,04	9,45E-01
ENSRNOG00000023946	**RGD13091**	similar to RIKEN cDNA 3110001D03 (I	298147	5	96088255	96089351	1097	544,06	-0,04	9,45E-01
ENSRNOG00000047957		TNF receptor-associated factor 5 [Source:MGI S		13	115317421	115342128	24708	26,09	-0,07	9,45E-01
ENSRNOG00000014071	**Ddb2**	damage specific DNA binding protein :	1E+08	3	86739191	86761706	22516	176,22	-0,07	9,46E-01
ENSRNOG00000016562	**Zfp191**	zinc finger protein 191 (Zfp191), mRN.	360204	18	15680091	15687290	7200	646,88	-0,06	9,46E-01
ENSRNOG00000015903		adducin 2 (beta) (Add2), transcript var	24171	4	182317207	182454565	137359	3545,36	0,05	9,46E-01
ENSRNOG00000018719	**Cir1**	corepressor interacting with RBPJ, 1 (	362149	3	66477815	66507826	30012	729,99	0,05	9,46E-01
ENSRNOG00000008565		NF-kappa-B inhibitor-interacting Ras-li	305751	15	12240425	12245726	5302	630,66	-0,07	9,46E-01
ENSRNOG00000016769	**Rab38**	RAB38, member RAS oncogene famil	252916	1	158385878	158466622	80745	18,93	0,08	9,46E-01
ENSRNOG00000020298	**Bag3**	Bcl2-associated athanogene 3 (Bag3)	293524	1	206989244	207013176	23933	330,29	-0,08	9,46E-01
ENSRNOG00000049127		small ArfGAP 1 [Source:MGI Symbol;/	684800	9	28900107	28991030	90924	1950,47	-0,04	9,46E-01
ENSRNOG00000000605	**Hs3st5**	heparan sulfate (glucosamine) 3-O-su	294449	20	41969878	42279750	309873	223,98	0,07	9,46E-01
ENSRNOG00000000975	**Mcoln1**	mucolipin 1 (Mcoln1), mRNA [Source:I	288371	12	4216868	4230870	14003	1030,67	0,03	9,46E-01
ENSRNOG00000002075	**Cnot6l**	CCR4-NOT transcription complex, sub	360917	14	15000864	15087397	86534	705,97	0,05	9,46E-01
ENSRNOG00000003844	**LOC10091(**	DEAH (Asp-Glu-Ala-His) box helicase	289693	14	61248771	61286905	38135	2117,37	-0,07	9,46E-01
ENSRNOG00000004921	**Nusap1**	nucleolar and spindle associated prote	311336	3	117970749	118001101	30353	437,15	0,07	9,46E-01
ENSRNOG00000012711	**Kcnh8**	potassium voltage-gated channel, sub	246325	9	36863082	37426999	563918	38,04	0,08	9,46E-01
ENSRNOG00000012850		K(lysine) acetyltransferase 6B [Source:MGI Sym		15	2794421	2945373	150953	1176,48	-0,04	9,46E-01
ENSRNOG00000013804	**Exoc5**	exocyst complex component 5 (Exoc5	60627	15	29811329	29855876	44548	1133,79	0,07	9,46E-01
ENSRNOG00000014052	**Ccna1**	cyclin A1 (Ccna1), mRNA [Source:Ref	295052	2	163876264	163886211	9948	62,41	-0,07	9,46E-01
ENSRNOG00000014076	**Mbnl1**	muscleblind-like splicing regulator 1 (N	282635	2	170175585	170287163	111579	202,59	-0,06	9,46E-01
ENSRNOG00000016800	**Ss18**	synovial sarcoma translocation, Chron	361295	18	5987818	6050274	62457	1340,79	-0,04	9,46E-01
ENSRNOG00000017568	**Rit2**	Ras-like without CAAX 2 (Rit2), mRN/	291713	18	23424107	23780916	356810	928,29	-0,06	9,46E-01
ENSRNOG00000019068	**Ctrb1**	chymotrypsinogen B1 (Ctrb1), mRNA	24291	19	54713958	54718713	4756	42,90	0,07	9,46E-01
ENSRNOG00000019271		Protein Ulk4 [Source:UniProtKB/TrEM	363167	8	128827983	129119610	291628	284,24	0,05	9,46E-01
ENSRNOG00000020029	**Fam195a**	family with sequence similarity 195, m	685545	10	15043744	15048683	4940	243,77	0,08	9,46E-01
ENSRNOG00000023434	**Tomt**	O-methyltransferase domain containin	308868	1	173176767	173178816	2050	60,33	0,08	9,46E-01
ENSRNOG00000023541	**Ahctf1**	AT hook containing transcription factor	360886	13	103034524	103084625	50102	718,62	0,07	9,46E-01
ENSRNOG00000026793	**Nt5dc3**	5'-nucleotidase domain containing 3 (N	691922	7	27430564	27482415	51852	1003,66	0,05	9,46E-01
ENSRNOG00000028247	**Ankrd13d**	ankyrin repeat domain 13 family, mem	361699	1	226390565	226402786	12222	1201,53	-0,05	9,46E-01
ENSRNOG00000029212		versican (Vcan), transcript variant 1, m	114122	2	18363890	18441931	78042	2622,17	-0,07	9,46E-01
ENSRNOG00000036592	**Zfp518a**	zinc finger protein 518A (Zfp518a), mF	309478	1	267596841	267621543	24703	102,19	-0,08	9,46E-01
ENSRNOG00000043301		sterile alpha motif domain containing 1 [Source:I		19	36097608	36099114	1507	755,26	0,07	9,46E-01
ENSRNOG00000049908	**Tmem29**	Protein LOC100359832 [Source:UniP	1E+08	X	21561686	21573679	11994	240,02	-0,04	9,46E-01
ENSRNOG00000004672	**Sec14l2**	SEC14-like 2 (S. cerevisiae) (Sec14l2	116486	14	85017262	85037193	19932	847,26	-0,06	9,46E-01
ENSRNOG00000005509	**Pak7**	p21 protein (Cdc42/Rac)-activated kin	311450	3	135530902	135841101	310200	795,74	-0,07	9,46E-01

ENSRNOG00000011042		RIKEN cDNA 2610034M16 gene [Source:MGI S			9	105131606	105256934	125329	20,50	0,09	9,46E-01
ENSRNOG00000012000	**Copb1**	coatomer protein complex, subunit bet	114023		1	190670321	190704747	34427	1872,40	-0,07	9,46E-01
ENSRNOG00000016146	**LOC36583**	similar to elongation protein 4 homolog	365839		2	206033597	206034385	789	40,89	0,06	9,46E-01
ENSRNOG00000016708	**E2f1**	E2F transcription factor 1 (E2f1), mRN	399489		3	156435530	156446390	10861	523,80	-0,05	9,46E-01
ENSRNOG00000023280	**Als2**	amyotrophic lateral sclerosis 2 (juvenil	363235		9	65769813	65823755	53943	1839,66	0,04	9,46E-01
ENSRNOG00000037768	**Rab43**	RAB43, member RAS oncogene famil	500249		4	184946341	184965637	19297	114,46	0,06	9,46E-01
ENSRNOG00000043441	**Lin52**	Protein Lin52; Similar to CG15929-PA	362763		6	117298279	117378681	80403	154,92	-0,05	9,46E-01
ENSRNOG00000033335	**Cenpi**	centromere protein I (Cenpi), mRNA [S	25448	X		105040385	105090803	50419	110,82	-0,09	9,46E-01
ENSRNOG00000033378		ring finger protein 123 (Rnf123), mRN	1E+08		8	116183556	116210629	27074	1598,17	-0,03	9,46E-01
ENSRNOG00000009198	**Rab6b**	RAB6B, member RAS oncogene famil	363123		8	111018332	111059791	41460	20273,27	-0,05	9,46E-01
ENSRNOG00000025167	**Sema4b**	sema domain, immunoglobulin domair	293042		1	142941088	142963935	22848	672,70	0,05	9,46E-01
ENSRNOG00000027489	**Mn1**	meningioma 1 (Mn1), mRNA [Source:I	498194		12	52955948	52991986	36039	2410,12	-0,04	9,46E-01
ENSRNOG00000008260		centrosomal protein 152 [Source:MGI	311391		3	124350158	124417461	67304	96,22	-0,06	9,46E-01
ENSRNOG00000005513	**Srsf5**	serine/arginine-rich splicing factor 5 (S	29667		6	112776230	112780506	4277	3971,78	-0,06	9,46E-01
ENSRNOG00000009580	**Lca5**	Leber congenital amaurosis 5 (Lca5),	300866		8	90445556	90503560	58005	197,41	-0,06	9,46E-01
ENSRNOG00000026217	**Armc2**	Protein Armc2 [Source:UniProtKB/TrE	499470		20	48446099	48543912	97814	51,34	0,06	9,46E-01
ENSRNOG00000028087		RIKEN cDNA 1110058L19 gene [Sour	685888		9	29068247	29076025	7779	248,38	-0,05	9,46E-01
ENSRNOG00000049208					3	161366789	161367079	291	65,80	0,07	9,46E-01
ENSRNOG00000000583	**Cdk19**	cyclin-dependent kinase 19 (Cdk19), r	309804		20	47178053	47318293	140241	3958,07	-0,05	9,47E-01
ENSRNOG00000001173		calcium binding protein 1 (Cabp1), trai	171051		12	48980781	49004881	24101	275,20	-0,08	9,47E-01
ENSRNOG00000001747	**Pak2**	p21 protein (Cdc42/Rac)-activated kin	29432		11	75034488	75092727	58240	91,45	-0,05	9,47E-01
ENSRNOG00000002790		ATP-binding cassette sub-family B me	302395	X		75954688	76094049	139362	166,06	0,09	9,47E-01
ENSRNOG00000003600	**Pnpt1**	polyribonucleotide nucleotidyltransfera	360992		14	113205574	113236749	31176	285,68	-0,07	9,47E-01
ENSRNOG00000003800	**Rgs9**	regulator of G-protein signaling 9 (Rgs	29481		10	97225541	97298645	73105	81,10	-0,07	9,47E-01
ENSRNOG00000009152	**Caprin1**	cell cycle associated protein 1 (Caprin	362173		3	100343084	100374879	31796	8972,80	-0,05	9,47E-01
ENSRNOG00000009550	**Sqle**	squalene epoxidase (Sqle), mRNA [Sc	29230		7	100196963	100211948	14986	4778,23	0,07	9,47E-01
ENSRNOG00000009884	**Lgals1**	lectin, galactoside-binding, soluble, 1 (	56646		7	120144848	120147980	3133	531,57	0,08	9,47E-01
ENSRNOG00000010188	**Satb2**	SATB homeobox 2 (Satb2), mRNA [Sc	501145		9	63264787	63441506	176720	2917,57	-0,05	9,47E-01
ENSRNOG00000012186	**Golph3**	golgi phosphoprotein 3 (coat-protein) (	78961		2	82299654	82327662	28009	2174,99	0,06	9,47E-01
ENSRNOG00000015441	**Il4r**	interleukin 4 receptor (Il4r), mRNA [So	25084		1	203940373	203952928	12556	108,74	-0,07	9,47E-01
ENSRNOG00000015740		E3 ubiquitin-protein ligase RNF125 [S	361296		18	14972266	15004357	32092	23,70	0,09	9,47E-01
ENSRNOG00000017988	**Mcur1**	Protein Ccdc90a; Putative uncharacte	291034		17	26114737	26131882	17146	170,42	-0,04	9,47E-01
ENSRNOG00000018053	**Fech**	ferrochelatase (Fech), mRNA [Source:	361338		18	59152151	59185351	33201	1078,22	0,05	9,47E-01
ENSRNOG00000018450	**LOC10091**	solute carrier family 25 (mitochondrial	309111		1	220890718	220898559	7842	18,15	0,09	9,47E-01
ENSRNOG00000021682	**Dcakd**	dephospho-CoA kinase domain contai	360639		10	90867138	90889424	22287	3978,09	0,06	9,47E-01
ENSRNOG00000022595	**LOC10036**	SNRPN upstream reading frame prote	113938		17	63105712	63105927	216	42,06	0,07	9,47E-01
ENSRNOG00000032208	**Eda**	Protein Eda [Source:UniProtKB/TrEM	302424	X		71080496	71092240	11745	37,55	-0,07	9,47E-01
ENSRNOG00000033330	**Skida1**	Protein LOC680770 [Source:UniProtK	680770		17	86556268	86557647	1380	173,34	-0,05	9,47E-01

ENSRNOG00000033697	**Casp4**	caspase 4, apoptosis-related cysteine	114555	8	2658133	2673885	15753	112,81	-0,09	9,47E-01
ENSRNOG00000046794	**Khdrbs1**	KH domain containing, RNA binding, s	117268	5	151655537	151680547	25011	6736,19	-0,03	9,47E-01
ENSRNOG00000019059	**Zfp1**	Protein Zfp1 [Source:UniProtKB/TrEM	498952	19	54689936	54693524	3589	254,85	-0,06	9,47E-01
ENSRNOG00000006432	**LOC10091**	tRNA nucleotidyl transferase, CCA-ad	312616	4	203326023	203339605	13583	154,18	-0,08	9,47E-01
ENSRNOG00000016398	**Clstn1**	calsyntenin 1 (Clstn1), mRNA [Source	313717	5	170179472	170247833	68362	19298,67	0,04	9,47E-01
ENSRNOG00000010381	**Mknk1**	MAP kinase-interacting serine/threonir	500526	5	138476361	138513933	37573	451,14	-0,06	9,47E-01
ENSRNOG00000017446	**Ndufs8**	NADH dehydrogenase (ubiquinone) Fe	293652	1	226013897	226017215	3319	2156,04	0,08	9,47E-01
ENSRNOG00000010630	**Prcp**	prolylcarboxypeptidase (angiotensinas	293118	1	163944735	163993907	49173	579,08	0,06	9,48E-01
ENSRNOG00000027654	**Appbp2**	amyloid beta precursor protein (cytopl	303396	10	72408561	72450355	41795	3969,91	0,05	9,48E-01
ENSRNOG00000021678	**Alkbh3**	alkB, alkylation repair homolog 3 (E. c	362169	3	89575454	89608081	32628	709,07	0,03	9,48E-01
ENSRNOG00000009000	**Foxj2**	forkhead box J2 (Foxj2), mRNA [Sour	502886	4	222674597	222700672	26076	1147,11	-0,04	9,48E-01
ENSRNOG00000025710	**Ces5a**	carboxylesterase 5A (Ces5a), mRNA [	307660	19	23219490	23250277	30788	17,73	-0,08	9,48E-01
ENSRNOG00000002080	**Urb1**	URB1 ribosome biogenesis 1 homolog	304104	11	34528215	34588898	60684	726,43	-0,05	9,49E-01
ENSRNOG00000006050	**Med19**	mediator complex subunit 19 (Med19)	311165	3	78601276	78611797	10522	917,38	0,04	9,49E-01
ENSRNOG00000022691	**St8sia5**	ST8 alpha-N-acetyl-neuraminide alpha	364901	18	73242668	73302825	60158	38,63	-0,07	9,49E-01
ENSRNOG00000020525	**Col5a3**	collagen, type V, alpha 3 (Col5a3), mF	60379	8	21842413	21887757	45345	871,92	-0,06	9,49E-01
ENSRNOG00000004542	**Ttc8**	tetratricopeptide repeat domain 8 (Ttc	299246	6	132140830	132207106	66277	309,65	-0,06	9,49E-01
ENSRNOG00000031335	**Ankrd37**	ankyrin repeat domain 37 (Ankrd37), r	361149	16	49189582	49192471	2890	28,30	0,08	9,49E-01
ENSRNOG00000047138				X	153600222	153600284	63	29,43	0,07	9,49E-01
ENSRNOG00000005359	**Csrnp3**	cysteine-serine-rich nuclear protein 3 (	311093	3	58735636	58752630	16995	223,75	-0,07	9,49E-01
ENSRNOG00000007881	**Mtmr14**	myotubularin related protein 14 (Mtmr	312634	4	208492229	208535256	43028	772,96	0,04	9,49E-01
ENSRNOG00000004639	**Traf6**	TNF receptor-associated factor 6, E3 u	311245	3	97912483	97931261	18779	254,71	0,04	9,49E-01
ENSRNOG00000005512	**Ndufa4**	NADH dehydrogenase (ubiquinone) 1	681024	4	38070978	38078251	7274	7100,33	-0,04	9,49E-01
ENSRNOG00000043267	**Ftsj2**	FtsJ RNA methyltransferase homolog	304323	12	18391358	18396265	4908	234,07	0,04	9,49E-01
ENSRNOG00000002568	**Socs1**	suppressor of cytokine signaling 1 (So	252971	10	3782936	3783574	639	52,10	0,08	9,50E-01
ENSRNOG00000028274	**Myrf**	myelin regulatory factor (Myrf), mRNA	293736	1	233205242	233237427	32186	512,45	-0,08	9,50E-01
ENSRNOG00000015528	**Pja2**	praja ring finger 2, E3 ubiquitin protein	192256	9	111541401	111568364	26964	5025,08	-0,06	9,51E-01
ENSRNOG00000030367	**Fam19a1**	Protein Fam19a1 [Source:UniProtKB/	500266	4	193332283	193442038	109756	26,32	-0,08	9,51E-01
ENSRNOG00000049641	**Rab7l1**	RAB7, member RAS oncogene family-	171122	13	53717939	53722976	5038	281,32	-0,06	9,51E-01
ENSRNOG00000000236	**Zfp207**	zinc finger protein 207 (Zfp207), mRN	303763	10	67418703	67430464	11762	3272,34	0,05	9,51E-01
ENSRNOG00000020179	**Nt5c2**	Protein Nt5c2 [Source:UniProtKB/TrE	1E+08	1	274089443	274123321	33879	1320,93	-0,04	9,51E-01
ENSRNOG00000042029	**Efs**	embryonal Fyn-associated substrate (	290212	15	37466899	37476240	9342	704,57	-0,08	9,51E-01
ENSRNOG00000007732	**Slc35c1**	solute carrier family 35 (GDP-fucose tı	311204	3	88065146	88071681	6536	1047,22	-0,03	9,51E-01
ENSRNOG00000001602	**Ltn1**	listerin E3 ubiquitin protein ligase 1 (Lt	288308	11	30648567	30706023	57457	822,50	-0,08	9,51E-01
ENSRNOG00000001834	**Mzt2b**	mitotic spindle organizing protein 2B (I	287929	11	92330667	92336557	5891	1081,74	0,05	9,51E-01
ENSRNOG00000007524	**Phactr3**	phosphatase and actin regulator 3 (Ph	362284	3	183262099	183304794	42696	875,55	0,06	9,51E-01
ENSRNOG00000010566	**Cep44**	centrosomal protein 44 (Cep44), mRN	290722	16	36982024	37001397	19374	226,88	0,06	9,51E-01
ENSRNOG00000013737	**Bcar3**	breast cancer anti-estrogen resistance	310838	2	245929016	246041116	112101	554,29	-0,06	9,51E-01

ENSRNOG00000023968	**Fancf**	Protein Fancf [Source:UniProtKB/TrE	499155	1	108273110	108274114	1005	129,32	-0,06	9,51E-01
ENSRNOG00000026162	**Cetn4**	centrin 4 (Cetn4), mRNA [Source:RefS	688611	2	143653652	143655150	1499	21,92	-0,08	9,51E-01
ENSRNOG00000028356	**Chchd10**	coiled-coil-helix-coiled-coil-helix doma	361824	20	15820607	15822447	1841	547,73	-0,08	9,51E-01
ENSRNOG00000042983		Cleavage and polyadenylation specific	291877	19	11425193	11442077	16885	2192,99	0,04	9,51E-01
ENSRNOG00000045728	**LOC10036**	Protein LOC100360726 [Source:UniP	1E+08	11	46086259	46133478	47220	45,52	-0,07	9,51E-01
ENSRNOG00000014049	**Riok1**	RIO kinase 1 (Riok1), mRNA [Source:	291061	17	29343651	29366597	22947	332,84	0,05	9,51E-01
ENSRNOG00000014783	**Athl1**	Protein Athl1 [Source:UniProtKB/TrEM	309103	1	220658976	220663578	4603	95,28	-0,08	9,51E-01
ENSRNOG00000015939	**Ccdc25**	coiled-coil domain containing 25 (Ccd	361059	15	49038971	49070547	31577	545,23	-0,06	9,51E-01
ENSRNOG00000031364	**Kif1c**	kinesin family member 1C (Kif1c), mR	113886	10	57067764	57097459	29696	2848,74	-0,06	9,51E-01
ENSRNOG00000003278	**Map3k14**	mitogen-activated protein kinase kinas	360640	10	91074396	91121436	47041	493,84	0,05	9,51E-01
ENSRNOG00000015235	**Fra10ac1**	fragile site, folic acid type, rare, fra(10	365458	1	264362189	264394030	31842	215,93	-0,06	9,51E-01
ENSRNOG00000042686		tripartite motif-containing 34 [Source:I	1E+08	1	175577162	175583070	5909	29,15	0,08	9,51E-01
ENSRNOG00000045854	**Zfp277**	Protein Zfp277 [Source:UniProtKB/Tr	298977	6	69835901	69962618	126718	177,07	-0,07	9,51E-01
ENSRNOG00000001065	**Cyth3**	cytohesin 3 (Cyth3), mRNA [Source:R	116693	12	14921915	14953446	31532	1439,54	-0,05	9,51E-01
ENSRNOG00000013555	**Ankrd55**	ankyrin repeat domain 55 (Ankrd55), r	361898	2	63169817	63238388	68572	145,01	-0,07	9,51E-01
ENSRNOG00000003767	**Rps6kc1**	ribosomal protein S6 kinase, polypepti	289342	13	113940375	114084551	144177	1315,56	0,05	9,51E-01
ENSRNOG00000004361		poly (ADP-ribose) polymerase family, member 1		7	117277297	117286365	9069	226,88	0,08	9,51E-01
ENSRNOG00000008090	**Txndc12**	thioredoxin domain containing 12 (end	298370	5	132291196	132316221	25026	1580,82	0,05	9,51E-01
ENSRNOG00000009358	**Osgin2**	Protein Osgin2; Similar to Protein C8o	313085	5	34342398	34360423	18026	206,98	0,05	9,51E-01
ENSRNOG00000010959	**Klhl25**	kelch-like family member 25 (Klhl25),	293023	1	138329800	138354991	25192	2193,98	-0,04	9,51E-01
ENSRNOG00000015976	**LOC68868**	SNRPN upstream reading frame (Snu	81781	1	202123032	202145397	22366	6594,02	-0,05	9,51E-01
ENSRNOG00000020668	**Cpsf7**	cleavage and polyadenylation specific	365407	1	233519360	233542386	23027	1916,53	-0,04	9,51E-01
ENSRNOG00000021117	**Rps6ka4**	ribosomal protein S6 kinase, polypepti	361715	1	229077912	229088867	10956	1003,96	-0,06	9,51E-01
ENSRNOG00000024754				19	36157945	36158416	472	46,57	0,06	9,51E-01
ENSRNOG00000003831	**Tdp1**	tyrosyl-DNA phosphodiesterase 1 (Td	314380	6	133127134	133187079	59946	224,86	-0,04	9,52E-01
ENSRNOG00000007441	**Klhl32**	Protein Klhl32 [Source:UniProtKB/TrE	313111	5	43602094	43787137	185044	307,78	-0,05	9,52E-01
ENSRNOG00000010239	**Cnbp**	CCHC-type zinc finger, nucleic acid bi	64530	4	184999246	185008099	8854	4518,40	-0,05	9,52E-01
ENSRNOG00000018568	**Rab5c**	RAB5C, member RAS oncogene famil	287709	10	88416138	88439534	23397	6791,75	-0,04	9,52E-01
ENSRNOG00000028685	**Shf**	Protein Shf; Similar to Shb-like adapte	362205	3	120827512	120837367	9856	460,45	-0,04	9,52E-01
ENSRNOG00000038916	**Dram1**	DNA-damage regulated autophagy mo	679937	7	29007048	29040615	33568	24,33	-0,08	9,52E-01
ENSRNOG00000025604	**Atad2**	ATPase family, AAA domain containin	314993	7	98589668	98631755	42088	229,56	-0,08	9,52E-01
ENSRNOG00000015218	**Nsmce1**	non-SMC element 1 homolog (S. cere	361645	1	203844089	203869649	25561	879,79	-0,05	9,52E-01
ENSRNOG00000000913		glucuronidase, beta (Gusb), mRNA [S	24434	12	32136118	32164707	28590	307,38	0,06	9,52E-01
ENSRNOG00000017890	**Crhbp**	corticotropin releasing hormone bindin	29625	2	44299507	44311225	11719	60,65	0,06	9,52E-01
ENSRNOG00000032778	**Bub1**	BUB1 mitotic checkpoint serine/threon	296137	3	126929123	126960429	31307	178,04	-0,08	9,52E-01
ENSRNOG00000016528	**Ppp1r21**	protein phosphatase 1, regulatory sub	362697	6	22219236	22281276	62041	1108,44	-0,04	9,52E-01
ENSRNOG00000010940	**Acad11**	acyl-CoA dehydrogenase family, mem	315973	8	111982416	112062756	80341	331,82	-0,07	9,53E-01
ENSRNOG00000010063	**Htr2a**	5-hydroxytryptamine (serotonin) recep	29595	15	60388152	60456545	68394	119,87	-0,06	9,53E-01

ENSRNOG00000010004	**Ripply2**	Protein Ripply2 [Source:UniProtKB/Tr	363111	8	94195165	94199268	4104	21,14	0,08	9,53E-01
ENSRNOG00000013322		DNA polymerase alpha catalytic subur	85241	X	62976410	63286475	310066	177,32	-0,08	9,53E-01
ENSRNOG00000014192	**Naa30**	N(alpha)-acetyltransferase 30, NatC c	498489	15	29962873	29982034	19162	1617,90	-0,05	9,53E-01
ENSRNOG00000019681	**Pold1**	polymerase (DNA directed), delta 1, ca	59294	1	101603485	101614451	10967	358,35	0,06	9,53E-01
ENSRNOG00000047736	**Kcna6**	potassium voltage gated channel, sha	64358	4	232324385	232357644	33260	1493,23	-0,06	9,53E-01
ENSRNOG00000050483	**Pradc1**	Protein LOC686393; RCG56502, isofc	686393	4	181808148	181812369	4222	301,49	-0,04	9,53E-01
ENSRNOG00000012213	**Nthl1**	nth (endonuclease III)-like 1 (E.coli) (N	29541	10	13813645	13819807	6163	148,08	-0,05	9,53E-01
ENSRNOG00000002246	**Slc30a9**	solute carrier family 30 (zinc transport	498358	14	42260672	42315461	54790	3451,14	0,06	9,53E-01
ENSRNOG00000002630	**Cnot8**	CCR4-NOT transcription complex, sub	363603	10	43336562	43345136	8575	2047,06	0,06	9,53E-01
ENSRNOG00000004141	**Vasn**	vasorin (Vasn), mRNA [Source:RefSe	679921	10	9886399	9896906	10508	229,06	0,05	9,53E-01
ENSRNOG00000006833	**Rb1cc1**	RB1-inducible coiled-coil 1 (Rb1cc1),	312927	5	17805493	17868916	63424	1016,69	0,08	9,53E-01
ENSRNOG00000011137	**Zbtb41**	zinc finger and BTB domain containing	289052	13	61532188	61554983	22796	291,36	-0,08	9,53E-01
ENSRNOG00000011172	**Ccdc67**	coiled-coil domain containing 67 (Ccdc	315438	8	14190848	14252439	61592	27,30	-0,08	9,53E-01
ENSRNOG00000011441	**Tpd52**	tumor protein D52 (Tpd52), mRNA [Sc	294900	2	114752028	114782627	30600	569,79	0,05	9,53E-01
ENSRNOG00000012944	**Casp9**	caspase 9, apoptosis-related cysteine	58918	5	164067768	164085301	17534	816,22	0,04	9,53E-01
ENSRNOG00000013030	**Pip4k2b**	phosphatidylinositol-5-phosphate 4-kir	89812	10	85449155	85475137	25983	3459,77	0,05	9,53E-01
ENSRNOG00000014867	**Synpo2**	synaptopodin 2 (Synpo2), mRNA [Sou	499702	2	246617297	246771857	154561	139,15	0,08	9,53E-01
ENSRNOG00000015162		lon peptidase 2, peroxisomal (Lonp2),	291922	19	32410041	32501857	91817	2619,91	0,05	9,53E-01
ENSRNOG00000015180	**Kdm8**	lysine (K)-specific demethylase 8 (Kdr	308976	1	203825919	203840778	14860	352,76	0,04	9,53E-01
ENSRNOG00000016306		Uncharacterized protein [Source:UniProtKB/TrE		2	9326677	9384140	57464	112,80	0,06	9,53E-01
ENSRNOG00000016936	**Nadk**	NAD kinase (Nadk), mRNA [Source:R	1E+08	5	176461415	176490616	29202	1960,49	0,05	9,53E-01
ENSRNOG00000017035	**Cep120**	centrosomal protein 120 (Cep120), mI	307302	18	47868772	47930749	61978	1441,05	0,05	9,53E-01
ENSRNOG00000017066	**Zfp384**	zinc finger protein 384 (Zfp384), trans	171018	4	224541608	224570434	28827	1233,10	-0,04	9,53E-01
ENSRNOG00000017577	**Bphl**	biphenyl hydrolase-like (serine hydrola	361239	17	33392069	33428242	36174	346,99	-0,05	9,53E-01
ENSRNOG00000017808	**Klf15**	Kruppel-like factor 15 (Klf15), mRNA [	85497	4	186906050	186918645	12596	552,44	0,05	9,53E-01
ENSRNOG00000019022	**Fam89a**	family with sequence similarity 89, me	361441	19	68206966	68219573	12608	318,76	-0,08	9,53E-01
ENSRNOG00000026840	**Thap3**	THAP domain containing, apoptosis a	362667	5	172713824	172719572	5749	619,86	0,06	9,53E-01
ENSRNOG00000033593	**Osbpl9**	oxysterol binding protein-like 9 (Osbpl	298369	5	132534676	132679332	144657	508,73	-0,07	9,53E-01
ENSRNOG00000033653	**Mpp3**	membrane protein, palmitoylated 3 (M	114202	10	89722766	89751677	28912	685,01	-0,05	9,53E-01
ENSRNOG00000039976		PTK7 protein tyrosine kinase 7 [Source:MGI Syr		9	15569249	15634049	64801	1220,83	0,04	9,53E-01
ENSRNOG00000039994	**Upf3b**	UPF3 regulator of nonsense transcript	313449	X	123849755	123867683	17929	333,66	-0,07	9,53E-01
ENSRNOG00000046492		transmembrane protein 194A [Source	679504	7	71158749	71168143	9395	51,20	0,06	9,53E-01
ENSRNOG00000048337		Uncharacterized protein [Source:UniProtKB/TrE		1	229473685	229475427	1743	27,56	0,07	9,53E-01
ENSRNOG00000031038	**Gtdc1**	glycosyltransferase-like domain contai	362129	3	34615976	34911411	295436	491,60	0,05	9,53E-01
ENSRNOG00000017202				8	74980451	75067085	86635	41,83	0,07	9,53E-01
ENSRNOG00000050476		Uncharacterized protein [Source:UniProtKB/TrE		1	71078544	71089740	11197	20,95	-0,07	9,53E-01
ENSRNOG00000025052	**Stard5**	StAR-related lipid transfer (START) dc	502348	1	146699180	146709472	10293	120,61	-0,06	9,53E-01
ENSRNOG00000003290		proline-rich coiled-coil 2C [Source:MGI Symbol;/		13	85439057	85489378	50322	988,50	-0,04	9,53E-01

ENSRNOG00000000034	**Nuak2**	NUAK family, SNF1-like kinase, 2 (Nu	289419	13	54160995	54177739	16745	427,36	0,06	9,54E-01
ENSRNOG00000000262		zinc finger protein 821 [Source:MGI Symbol;Acc		19	52765875	52784091	18217	1387,45	0,04	9,54E-01
ENSRNOG00000000902	**Hsph1**	heat shock 105/110 protein 1 (Hsph1),	288444	12	8423548	8442481	18934	3603,01	-0,06	9,54E-01
ENSRNOG00000006205	**Rel**	Protein Rel [Source:UniProtKB/TrEMI	305584	14	108212989	108231038	18050	26,16	-0,07	9,54E-01
ENSRNOG00000008225				6	86193465	86193876	412	30,00	0,07	9,54E-01
ENSRNOG00000027032		Rhomboid domain-containing protein	289753	14	86028976	86034641	5666	1142,63	0,05	9,54E-01
ENSRNOG00000040040	**Ndufaf6**	NADH dehydrogenase (ubiquinone) c	297821	5	29024405	29048252	23848	271,24	0,05	9,54E-01
ENSRNOG00000018943	**Tnnc1**	troponin C type 1 (slow) (Tnnc1), mRN	290561	16	7220777	7223907	3131	64,72	0,07	9,54E-01
ENSRNOG00000018396	**Prpf18**	PRP18 pre-mRNA processing factor 1	171552	17	79256708	79287301	30594	1537,49	0,06	9,54E-01
ENSRNOG00000048250	**Tmem159**	transmembrane protein 159 (Tmem15	378467	1	196800449	196819294	18846	81,02	-0,06	9,54E-01
ENSRNOG00000001215	**RGD13095**	uncharacterized protein LOC309681		309681	20	13587480	13594340	6861	865,43	-0,05	9,54E-01
ENSRNOG00000008885	**Chst11**	carbohydrate (chondroitin 4) sulfotrans	314694	7	26770955	26981219	210265	591,23	0,04	9,54E-01
ENSRNOG00000018991	**Gsn**	gelsolin (Gsn), mRNA [Source:RefSec	296654	3	19799929	19826452	26524	872,98	-0,07	9,54E-01
ENSRNOG00000045871	**Copb2**	coatomer protein complex, subunit bet	60384	8	106034675	106045348	10674	2342,27	0,06	9,54E-01
ENSRNOG00000001430	**Polr2j**	polymerase (RNA) II (DNA directed) p	288588	12	25587572	25593124	5553	1315,48	-0,05	9,54E-01
ENSRNOG00000003296	**Dck**	deoxycytidine kinase (Dck), mRNA [S	79127	14	20843808	20863311	19504	892,27	-0,04	9,54E-01
ENSRNOG00000009965	**Pih1d2**	PIH1 domain containing 2 (Pih1d2), m	315645	8	53647606	53655845	8240	101,49	-0,07	9,54E-01
ENSRNOG00000013843	**Arcn1**	archain 1 (Arcn1), mRNA [Source:Ref	300674	8	47670185	47694935	24751	5799,36	-0,04	9,54E-01
ENSRNOG00000016724		erythrocyte membrane protein band 4.	116724	9	117003894	117131765	127872	1755,81	-0,04	9,54E-01
ENSRNOG00000023322	**Ostc**	oligosaccharyltransferase complex sul	362040	2	254367954	254383277	15324	1625,81	-0,05	9,54E-01
ENSRNOG00000009745		Protein Ppp3cc [Source:UniProtKB/TrEMBL;Acc		15	55622790	55692739	69950	569,99	0,05	9,54E-01
ENSRNOG00000014187	**Igf1r**	insulin-like growth factor 1 receptor (Ig	25718	1	129985804	130265820	280017	627,46	0,07	9,54E-01
ENSRNOG00000020893		sorting nexin family member 27 (Snx2	260323	2	215217244	215300226	82983	7509,45	0,03	9,54E-01
ENSRNOG00000027049	**Atp5j2**	ATP synthase, H+ transporting, mitoch	690441	12	13260912	13267470	6559	2948,56	-0,03	9,54E-01
ENSRNOG00000042731	**Slc25a18**	solute carrier family 25 (glutamate car	681896	4	220420268	220440655	20388	672,62	-0,07	9,54E-01
ENSRNOG00000048980		guanine nucleotide binding protein (G protein), g		15	8934677	8941164	6488	1790,01	0,04	9,54E-01
ENSRNOG00000016688	**Ston1**	stonin 1 (Ston1), mRNA [Source:RefS	360202	6	22325769	22342414	16646	317,59	-0,06	9,54E-01
ENSRNOG00000010783	**Mak16**	MAK16 homolog (S. cerevisiae) (Mak	306526	16	64386704	64395256	8553	402,50	0,04	9,54E-01
ENSRNOG00000013293	**Mccc1**	methylcrotonoyl-CoA carboxylase 1 (a	294972	2	142186429	142317803	131375	270,12	0,06	9,54E-01
ENSRNOG00000004506	**Taf1b**	TATA box binding protein (Tbp)-associ	690450	6	60512955	60589982	77028	172,69	-0,05	9,54E-01
ENSRNOG00000018553	**Pitpnm1**	phosphatidylinositol transfer protein, n	361694	1	226219372	226232745	13374	3366,35	0,04	9,54E-01
ENSRNOG00000010592	**Slc25a27**	solute carrier family 25, member 27 (S	85262	9	18758260	18783966	25707	531,00	-0,07	9,55E-01
ENSRNOG00000017678				1	38348364	38351171	2808	19,87	-0,07	9,55E-01
ENSRNOG00000016174	**Slc35b3**	Protein Slc35b3 [Source:UniProtKB/T	306866	17	28399913	28425662	25750	160,83	-0,05	9,55E-01
ENSRNOG00000033790	**Hnrnpu**	heterogeneous nuclear ribonucleoprot	117280	13	100670813	100679599	8787	13706,12	-0,04	9,55E-01
ENSRNOG00000005366		family with sequence similarity 98, member B [S		3	115501131	115533474	32344	32,37	-0,06	9,55E-01
ENSRNOG00000016377	**Cep55**	centrosomal protein 55 (Cep55), mRN	294074	1	264226027	264241499	15473	78,02	0,07	9,55E-01
ENSRNOG00000047874	**Nelfcd**	negative elongation factor complex me	679203	3	178573032	178574252	1221	448,45	0,03	9,55E-01

ENSRNOG00000033051	**Slc22a15**	solute carrier family 22, member 15 (S	310732	2	223715478	223775516	60039	262,41	0,06	9,55E-01
ENSRNOG00000009934	**Emc3**	ER membrane protein complex subun	312640	4	208768761	208784670	15910	3227,61	0,06	9,55E-01
ENSRNOG00000005924	**Dstn**	destrin (Dstn), mRNA [Source:RefSeq	296197	3	144462864	144489654	26791	1009,33	-0,04	9,55E-01
ENSRNOG00000015675	**Nell1**	NEL-like 1 (chicken) (Nell1), mRNA [S	81733	1	106540585	107260907	720323	617,64	0,06	9,55E-01
ENSRNOG00000009690	**Rnf219**	ring finger protein 219 (Rnf219), mRN	361088	15	92127751	92165948	38198	471,23	0,04	9,55E-01
ENSRNOG00000019031	**Neu4**	sialidase 4 (Neu4), mRNA [Source:Re	316642	9	100655816	100661472	5657	70,98	0,07	9,55E-01
ENSRNOG00000021750	**Id1**	inhibitor of DNA binding 1 (Id1), mRN/	25261	3	154620033	154621183	1151	935,80	-0,07	9,55E-01
ENSRNOG00000046607	**Cited4**	Cbp/p300-interacting transactivator, w	114491	5	143379104	143379995	892	34,24	-0,07	9,55E-01
ENSRNOG00000048187	**LOC69115**	RIKEN cDNA E130012A19 gene [Sou	691153	10	85361304	85362419	1116	227,63	0,06	9,55E-01
ENSRNOG00000024904	**Pla2g4e**	Protein Pla2g4e [Source:UniProtKB/T	296091	3	118501873	118532419	30547	124,07	-0,06	9,56E-01
ENSRNOG00000029938	**Pik3c2b**	phosphatidylinositol-4-phosphate 3-kir	289021	13	54919438	54977002	57565	1066,77	0,04	9,56E-01
ENSRNOG00000017850	**Dctpp1**	dCTP pyrophosphatase 1 (Dctpp1), m	192252	1	205696307	205699698	3392	346,61	0,08	9,56E-01
ENSRNOG00000049052	**Sgk3**	Serine/threonine-protein kinase Sgk3	171498	5	13849631	13894844	45214	63,89	-0,06	9,56E-01
ENSRNOG00000027988	**Zhx3**	zinc fingers and homeoboxes 3 (Zhx3)	311604	3	162994286	163008101	13816	2378,04	-0,05	9,56E-01
ENSRNOG00000045605	**Uxs1**	UDP-glucuronate decarboxylase 1 (U›	246232	9	10150139	10226865	76727	1347,01	0,05	9,56E-01
ENSRNOG00000019211	**Olfml3**	olfactomedin-like 3 (Olfml3), mRNA [S	310743	2	225643129	225645958	2830	171,48	-0,05	9,56E-01
ENSRNOG00000001299	**Sun1**	Sad1 and UNC84 domain containing 1	360773	12	19476257	19509115	32859	1050,42	0,04	9,56E-01
ENSRNOG00000002378	**Bpnt1**	3'(2'), 5'-bisphosphate nucleotidase 1	64473	13	107941117	107965559	24443	2436,30	0,05	9,56E-01
ENSRNOG00000019070	**St13**	suppression of tumorigenicity 13 (St13	81800	7	122561393	122612277	50885	529,89	-0,04	9,56E-01
ENSRNOG00000027329	**Dhx57**	DEAH (Asp-Glu-Ala-Asp/His) box poly	366532	6	2898233	2941135	42903	3006,69	0,05	9,56E-01
ENSRNOG00000000506	**Tead3**	TEA domain family member 3 (Tead3)	294299	20	10111923	10131306	19384	181,68	-0,06	9,56E-01
ENSRNOG00000000903	**Asl**	argininosuccinate lyase (Asl), mRNA [	59085	12	32102491	32115103	12613	448,54	-0,05	9,56E-01
ENSRNOG00000002101	**Paics**	phosphoribosylaminoimidazole carbox	140946	14	33354799	33371479	16681	400,42	0,05	9,56E-01
ENSRNOG00000005082	**Irf6**	interferon regulatory factor 6 (Irf6), mF	364081	13	116425025	116444232	19208	43,20	0,06	9,56E-01
ENSRNOG00000005393	**Sertad2**	SERTA domain containing 2 (Sertad2)	498423	14	104552934	104616957	64024	199,25	0,05	9,56E-01
ENSRNOG00000005804	**Eif3d**	eukaryotic translation initiation factor 3	362952	7	119182951	119195340	12390	3295,08	0,03	9,56E-01
ENSRNOG00000007189	**Ttc22**	tetratricopeptide repeat domain 22 (Tt	298300	5	130071415	130088219	16805	30,32	0,06	9,56E-01
ENSRNOG00000007214	**Oma1**	zinc metallopeptidase homolog (S. cer	298282	5	126714757	126767291	52535	89,64	0,07	9,56E-01
ENSRNOG00000007688	**Sms**	spermine synthase (Sms), mRNA [Sou	363469	X	40674244	40725965	51722	711,07	0,05	9,56E-01
ENSRNOG00000007769	**March2**	membrane-associated ring finger (C3H	362849	7	18736909	18754963	18055	2549,72	0,05	9,56E-01
ENSRNOG00000007788	**Glod4**	glyoxalase domain containing 4 (Glod4	363644	10	63616109	63633601	17493	1044,48	-0,03	9,56E-01
ENSRNOG00000008405		translational activator of mitochondrially encodec		10	94011140	94018653	7514	59,19	-0,06	9,56E-01
ENSRNOG00000009034		Tudor domain-containing protein 3 [S	306066	15	74335410	74462431	127022	448,13	-0,04	9,56E-01
ENSRNOG00000009250	**Pts**	6-pyruvoyl-tetrahydropterin synthase (	29498	8	53551584	53558594	7011	189,23	-0,04	9,56E-01
ENSRNOG00000009451	**Prpf38a**	PRP38 pre-mRNA processing factor 3	298374	5	132015346	132027043	11698	642,85	-0,06	9,56E-01
ENSRNOG00000010584	**Tmem123**	transmembrane protein 123 (Tmem12	363013	8	5995680	5999126	3447	302,48	0,04	9,56E-01
ENSRNOG00000013154	**Sfpq**	splicing factor proline/glutamine-rich (S	252855	5	148850564	148858900	8337	7372,84	-0,04	9,56E-01
ENSRNOG00000013328		RNA binding protein with multiple splic	498642	16	61619193	61767832	148640	27,97	0,06	9,56E-01

ENSRNOG00000013529	**Stk4**	serine/threonine kinase 4 (Stk4), mRN	311622	3	166652145	166731210	79066	1252,61	-0,03	9,56E-01
ENSRNOG00000013878	**Hmgxb4**	HMG box domain containing 4 (Hmgxl	307667	19	25465206	25503029	37824	510,69	-0,05	9,56E-01
ENSRNOG00000014093	**Tom1**	target of myb1 (chicken) (Tom1), mRN	361370	19	25505008	25539012	34005	1163,59	0,05	9,56E-01
ENSRNOG00000014525	**Hspd1**	heat shock protein 1 (chaperonin) (Hs	63868	9	61361746	61372172	10427	1211,10	0,05	9,56E-01
ENSRNOG00000015107		lysine (K)-specific methyltransferase 2	362996	X	115020064	115052804	32741	3330,91	0,03	9,56E-01
ENSRNOG00000015347	**Trim45**	tripartite motif-containing 45 (Trim45),	295323	2	222749091	222757785	8695	141,93	-0,06	9,56E-01
ENSRNOG00000015772	**LOC681022**			18	50416993	50418933	1941	38,01	0,07	9,56E-01
ENSRNOG00000017306	**Tmem238**	Protein Tmem238 [Source:UniProtKB/TrEMBL;/		1	75903410	75910074	6665	105,63	0,07	9,56E-01
ENSRNOG00000017429	**Lat**	linker for activation of T cells (Lat), mF	81511	1	204747720	204752745	5026	18,81	-0,07	9,56E-01
ENSRNOG00000017488	**Snx24**	sorting nexin 24 (Snx24), mRNA [Sour	361328	18	47416795	47569094	152300	847,42	0,04	9,56E-01
ENSRNOG00000017493	**Pias2**	protein inhibitor of activated STAT, 2 (F	83422	18	73147272	73203959	56688	882,13	0,03	9,56E-01
ENSRNOG00000021202	**Rtn3**	reticulon 3 (Rtn3), transcript variant 1,	140945	1	229664611	229721430	56820	33797,34	0,04	9,56E-01
ENSRNOG00000022054	**Paqr7**	progestin and adipoQ receptor family r	313615	5	156503752	156504789	1038	476,45	-0,05	9,56E-01
ENSRNOG00000023412	**Tmem241**	Protein Tmem241 [Source:UniProtKB	680558	18	3456528	3565125	108598	66,94	-0,05	9,56E-01
ENSRNOG00000024479	**Klhl34**	Protein Klhl34 [Source:UniProtKB/TrE	302703	X	40264786	40266723	1938	136,81	0,05	9,56E-01
ENSRNOG00000025100	**Ikbke**	inhibitor of kappa light polypeptide ger	363984	13	53107178	53132162	24985	27,33	-0,07	9,56E-01
ENSRNOG00000027099	**Fbxl2**	Protein Fbxl2 [Source:UniProtKB/TrEl	363156	8	121556744	121594358	37615	380,16	-0,05	9,56E-01
ENSRNOG00000028884	**LOC68002(**	ATP synthase, H+ transporting, mitoch	300677	8	47838601	47845456	6856	8066,65	0,06	9,56E-01
ENSRNOG00000031653	**Ccdc58**	coiled-coil domain containing 58 (Ccd	288065	11	70685896	70706181	20286	220,80	0,05	9,56E-01
ENSRNOG00000032517	**Cntn6**	contactin 6 (Cntn6), mRNA [Source:Re	27256	4	201062012	201373876	311865	136,98	0,07	9,56E-01
ENSRNOG00000013288	**Xpo7**	exportin 7 (Xpo7), mRNA [Source:Ref	361070	15	56082710	56122236	39527	2414,98	0,03	9,56E-01
ENSRNOG00000009628	**Pfkm**	phosphofructokinase, muscle (Pfkm),	65152	7	138833721	138857275	23555	6823,83	0,04	9,56E-01
ENSRNOG00000018291	**LOC10090**	vitamin K epoxide reductase complex,	399684	8	16361561	16407860	46300	73,46	0,06	9,56E-01
ENSRNOG00000031536	**Syndig1**	synapse differentiation inducing 1 (Syr	362235	3	151405855	151571800	165946	440,23	0,05	9,56E-01
ENSRNOG00000013661	**Kif26a**	kinesin family member 26A (Kif26a), m	314473	6	145729938	145764118	34181	946,91	0,06	9,56E-01
ENSRNOG00000014997	**Igf2r**	insulin-like growth factor 2 receptor (Ig	25151	1	51488769	51577141	88373	2979,64	-0,05	9,56E-01
ENSRNOG00000018381	**Cdkal1**	Protein Cdkal1 [Source:UniProtKB/Trl	361243	17	38824446	38993624	169179	51,62	0,07	9,56E-01
ENSRNOG00000004710	**Rbks**	ribokinase (Rbks), mRNA [Source:Ref	362706	6	35875927	35953018	77092	234,18	-0,05	9,56E-01
ENSRNOG00000005988	**Arl8a**	ADP-ribosylation factor-like 8A (Arl8a)	498234	13	57024684	57033686	9003	10540,08	-0,04	9,56E-01
ENSRNOG00000007541	**Fhl3**	four and a half LIM domains 3 (Fhl3),	313582	5	146460220	146466971	6752	73,70	0,06	9,56E-01
ENSRNOG00000009322	**LOC10036**	coiled-coil domain containing 126 (Ccc	500117	4	143671032	143692061	21030	187,57	0,06	9,56E-01
ENSRNOG00000010695	**Pdgfc**	platelet derived growth factor C (Pdgfc	79429	2	199362291	199537365	175075	156,39	0,06	9,56E-01
ENSRNOG00000013559		tubulin tyrosine ligase-like family, mem	308762	1	142989465	143002432	12968	89,81	-0,07	9,56E-01
ENSRNOG00000014209	**Utp6**	Protein Utp6 [Source:UniProtKB/TrEMBL;Acc:F		10	66914440	66944884	30445	637,88	0,06	9,56E-01
ENSRNOG00000020688	**Ccdc51**	coiled-coil domain containing 51 (Ccd	316008	8	117172283	117182587	10305	362,73	-0,03	9,56E-01
ENSRNOG00000023455	**Pomgnt1**	protein O-linked mannose N-acetylglu	362567	5	138791235	138801110	9876	1799,92	0,03	9,56E-01
ENSRNOG00000025509	**Apbb2**	Protein Apbb2 [Source:UniProtKB/TrE	305338	14	43166629	43317056	150428	1363,77	-0,05	9,56E-01
ENSRNOG00000047302	**Btbd8**	Protein LOC100364240 [Source:UniP	1E+08	14	3307623	3355037	47415	36,29	0,06	9,56E-01

ENSRNOG00000048024		Protein Tmem178b [Source:UniProtK	679651	4	67476292	67606749	130458	299,42	0,05	9,56E-01
ENSRNOG00000000172	**Sqrdl**	sulfide quinone reductase-like (yeast)	691966	3	121438451	121477299	38849	176,84	0,05	9,56E-01
ENSRNOG00000014564	**Yipf5**	Yip1 domain family, member 5 (Yipf5),	361315	18	33075378	33109160	33783	1547,96	0,04	9,56E-01
ENSRNOG00000020585	**Tbxa2r**	thromboxane A2 receptor (Tbxa2r), ml	24816	7	11419918	11424715	4798	34,54	-0,06	9,56E-01
ENSRNOG00000010011	**Osbpl3**	Oxysterol-binding protein [Source:Uni	362360	4	144700488	144778606	78119	87,42	-0,06	9,57E-01
ENSRNOG00000016689	**Fanci**	Protein Fanci [Source:UniProtKB/TrEI	1E+08	1	142082254	142121329	39076	154,28	0,06	9,57E-01
ENSRNOG00000025625	**Rnase4**	ribonuclease, RNase A family 4 (Rnas	56759	15	31854894	31866407	11514	272,15	-0,06	9,57E-01
ENSRNOG00000003717	**Cnih4**	cornichon homolog 4 (Drosophila) (Cn	289324	13	104468780	104484267	15488	520,26	-0,04	9,57E-01
ENSRNOG00000012718	**Zfp451**	zinc finger protein 451 (Zfp451), mRN.	316312	9	38077911	38133115	55205	481,47	-0,04	9,57E-01
ENSRNOG00000046480		Uncharacterized protein [Source:UniProtKB/TrE		1	205828271	205830240	1970	119,74	0,05	9,57E-01
ENSRNOG00000012787	**Tmem164**	transmembrane protein 164 (Tmem16	367763	X	112397269	112556696	159428	2186,17	0,03	9,57E-01
ENSRNOG00000026913	**Bbs10**	Bardet-Biedl syndrome 10 (Bbs10), ml	500832	7	54041088	54044111	3024	228,77	0,06	9,57E-01
ENSRNOG00000032039	**Atp9b**	ATPase, class II, type 9B (Atp9b), mR	291411	18	76446514	76638326	191813	1190,16	0,05	9,57E-01
ENSRNOG00000014720		Protein Srbd1 [Source:UniProtKB/TrE	301665	6	9187408	9376698	189291	523,14	-0,05	9,58E-01
ENSRNOG00000023647	**Aldoa**	aldolase A, fructose-bisphosphate (Alc	24189	1	205208255	205212211	3957	8592,28	-0,05	9,58E-01
ENSRNOG00000000381	**Herc4**	HECT and RLD domain containing E3	309758	20	8063831	8138835	75005	634,58	0,05	9,58E-01
ENSRNOG00000001120	**Med13l**	Protein Med13l [Source:UniProtKB/Tr	360817	12	45260303	45412655	152353	2558,41	0,03	9,58E-01
ENSRNOG00000001377	**Ddx54**	DEAD (Asp-Glu-Ala-Asp) box polypep	360815	12	43328542	43342983	14442	1716,76	0,05	9,58E-01
ENSRNOG00000002669	**Cluh**	Clustered mitochondria protein homol	303300	10	61161917	61182201	20285	2535,74	-0,04	9,58E-01
ENSRNOG00000005376	**Mad2l1**	MAD2 mitotic arrest deficient-like 1 (ye	297176	4	162316426	162322429	6004	472,57	0,03	9,58E-01
ENSRNOG00000006627	**Cys1**	cystin 1 (Cys1), mRNA [Source:RefSe	690489	6	60716420	60733664	17245	68,33	-0,05	9,58E-01
ENSRNOG00000007242	**Ehmt1**	euchromatic histone-lysine N-methyltr	362078	3	1952113	2043561	91449	2341,04	0,03	9,58E-01
ENSRNOG00000008063	**Hibadh**	3-hydroxyisobutyrate dehydrogenase	63938	4	147270737	147368793	98057	1387,88	0,05	9,58E-01
ENSRNOG00000009539	**Khdrbs3**	KH domain containing, RNA binding, s	64015	7	109965262	110111223	145962	4729,72	0,04	9,58E-01
ENSRNOG00000010952	**Ift122**	Intraflagellar transport 122 homolog (C	312651	4	211040406	211095866	55461	684,18	0,04	9,58E-01
ENSRNOG00000011975	**Crtc3**	Protein Crtc3 [Source:UniProtKB/TrEI	365297	1	143353060	143452208	99149	153,80	-0,04	9,58E-01
ENSRNOG00000012629	**Rab23**	RAB23, member RAS oncogene famil	367242	9	38152129	38175620	23492	933,83	-0,04	9,58E-01
ENSRNOG00000012784	**Gtf3c4**	general transcription factor IIIC, polype	685539	3	12755272	12773005	17734	1458,83	-0,03	9,58E-01
ENSRNOG00000019406	**Zfp61**	zinc finger protein 61 (Zfp61), mRNA [	499094	1	82386242	82390511	4270	378,08	0,05	9,58E-01
ENSRNOG00000020339	**Neurl1a**	neuralized homolog 1A (Drosophila) (N	309459	1	274383497	274451430	67934	207,67	-0,05	9,58E-01
ENSRNOG00000021380	**Fads6**	fatty acid desaturase 6 (Fads6), mRN	303671	10	104447218	104462044	14827	338,26	-0,04	9,58E-01
ENSRNOG00000023753		afadin [Source:RefSeq peptide;Acc:N	26955	1	54957455	55043458	86004	2431,01	-0,03	9,58E-01
ENSRNOG00000028839		cytochrome b-561 domain containing	362017	2	230456544	230460353	3810	100,99	0,07	9,58E-01
ENSRNOG00000047089	**Alkbh7**	alkB, alkylation repair homolog 7 (E. c	679944	9	9046025	9048157	2133	1172,05	0,06	9,58E-01
ENSRNOG00000007136	**Anxa7**	annexin A7 (Anxa7), mRNA [Source:R	155423	15	8355756	8382542	26787	1035,05	0,06	9,58E-01
ENSRNOG00000015774	**Mreg**	melanoregulin (Mreg), mRNA [Source:	501162	9	79264980	79318250	53271	62,61	-0,06	9,58E-01
ENSRNOG00000013379	**Zfp422**	zinc finger protein 422 (Zfp422), mRN	360389	4	214696098	214700282	4185	1596,39	0,05	9,58E-01
ENSRNOG00000035551	**Mir9-1**	rno-mir-9b-1 [Source:miRBase;Acc:MI0015430]		2	206985807	206985914	108	22,08	0,07	9,58E-01

ENSRNOG00000016050		Fibroblast growth factor receptor 1 (Fg	79114	16	70925574	70978004	52431	4632,90	-0,05	9,58E-01
ENSRNOG00000019338	**Dll3**	delta-like 3 (Drosophila) (Dll3), mRNA	114125	1	86697088	86704825	7738	547,06	0,06	9,58E-01
ENSRNOG00000009434	**RGD13105**	similar to RIKEN cDNA 1300017J02 (I	315963	8	111134566	111154326	19761	19,88	-0,07	9,58E-01
ENSRNOG00000024372	**Cwf19l2**	CWF19-like 2, cell cycle control (S. po	362804	6	28868716	28935276	66561	279,20	0,07	9,58E-01
ENSRNOG00000015541	**Gnb3**	guanine nucleotide binding protein (G	60449	4	224370235	224375938	5704	44,89	-0,06	9,58E-01
ENSRNOG00000020891	**Kif9**	kinesin family member 9 (Kif9), mRNA	501059	8	118086487	118136590	50104	60,13	-0,05	9,58E-01
ENSRNOG00000005637	**Usp44**	ubiquitin specific peptidase 44 (Usp44	314746	7	34803452	34818325	14874	22,88	-0,06	9,58E-01
ENSRNOG00000016038	**Mgmt**	O-6-methylguanine-DNA methyltransfe	25332	1	216161435	216388310	226876	623,89	0,07	9,58E-01
ENSRNOG00000003013		Rpgr protein; Uncharacterized protein [Source:U	X		15053410	15113670	60261	122,60	-0,04	9,58E-01
ENSRNOG00000008015	**Fos**	FBJ osteosarcoma oncogene (Fos), m	314322	6	116249742	116252608	2867	348,10	-0,07	9,58E-01
ENSRNOG00000008643	**Rars2**	arginyl-tRNA synthetase 2, mitochondi	297969	5	54644487	54685987	41501	183,40	0,06	9,58E-01
ENSRNOG00000013287	**Sall2**	sal-like 2 (Drosophila) (Sall2), mRNA [	305854	15	32538672	32556550	17879	2674,31	-0,03	9,58E-01
ENSRNOG00000042170		Uncharacterized protein [Source:UniProtKB/TrE		4	183369282	183370346	1065	140,72	-0,05	9,58E-01
ENSRNOG00000048961	**Bhlhe41**	Protein Bhlhe41 [Source:UniProtKB/T	117095	4	244395779	244398201	2423	176,42	-0,05	9,58E-01
ENSRNOG00000011658	**Tex30**	testis expressed 30 (Tex30), mRNA [S	301381	9	50525698	50535199	9502	199,02	0,04	9,59E-01
ENSRNOG00000014645	**Aldh7a1**	aldehyde dehydrogenase 7 family, me	291450	18	50814400	50846386	31987	2367,56	0,04	9,59E-01
ENSRNOG00000028592		Uncharacterized protein [Source:UniProtKB/TrE		12	6052650	6055814	3165	22,74	-0,06	9,59E-01
ENSRNOG00000015825	**Aurkc**	aurora kinase C (Aurkc), mRNA [Sour	292554	1	71352904	71355297	2394	86,02	-0,06	9,59E-01
ENSRNOG00000016356	**Got1**	glutamic-oxaloacetic transaminase 1, :	24401	1	270691432	270714931	23500	2208,08	0,04	9,59E-01
ENSRNOG00000019057	**Prkcq**	protein kinase C, theta (Prkcq), mRNA	85420	17	72670750	72747139	76390	129,63	-0,07	9,59E-01
ENSRNOG00000019978	**Parg**	poly (ADP-ribose) glycohydrolase (Pa	83507	16	8267358	8375027	107670	1212,28	0,05	9,59E-01
ENSRNOG00000020952		Protein Cgn [Source:UniProtKB/TrEM	310655	2	215388585	215407679	19095	79,78	-0,06	9,59E-01
ENSRNOG00000028279	**LOC10036**	RIKEN cDNA 4933413G19 gene [Sou	1E+08	4	226902964	226911231	8268	18,77	-0,07	9,59E-01
ENSRNOG00000050289	**Cstf2t**	cleavage stimulation factor, 3' pre-RN	309338	1	256813774	256817290	3517	3663,40	0,05	9,59E-01
ENSRNOG00000002504	**Spata17**	Protein Spata17 [Source:UniProtKB/T	498305	13	110184471	110286162	101692	20,04	-0,06	9,59E-01
ENSRNOG00000002956	**Stim2**	stromal interaction molecule 2 (Stim2)	117087	14	59324133	59450997	126865	1172,12	-0,05	9,59E-01
ENSRNOG00000003455	**Zfp13**	zinc finger protein 13 (Zfp13), mRNA [	287095	10	12729940	12738081	8142	731,57	0,03	9,59E-01
ENSRNOG00000008292	**Hif1a**	hypoxia-inducible factor 1, alpha subu	29560	6	106242730	106287704	44975	2581,58	-0,04	9,59E-01
ENSRNOG00000009099	**Zfp281**	zinc finger protein 281 (Zfp281), mRN	305083	13	58574471	58577421	2951	691,07	-0,04	9,59E-01
ENSRNOG00000010454	**Ccno**	cyclin O (Ccno), mRNA [Source:RefSe	499528	2	63896130	63899197	3068	124,23	-0,06	9,59E-01
ENSRNOG00000012785	**Armc10**	armadillo repeat containing 10 (Armc1	296758	4	10006007	10021181	15175	1760,27	-0,03	9,59E-01
ENSRNOG00000012911	**Erlin1**	ER lipid raft associated 1 (Erlin1), mR	293939	1	271255433	271290754	35322	879,54	-0,04	9,59E-01
ENSRNOG00000025892	**Pkn3**	protein kinase N3 (Pkn3), mRNA [Sou	296619	3	13979910	14008349	28440	164,37	0,04	9,59E-01
ENSRNOG00000026008	**Gtf2e1**	general transcription factor IIE, polype	303918	11	69170988	69203018	32031	910,39	-0,04	9,59E-01
ENSRNOG00000018566	**Ctsl1**	cathepsin L1 (Ctsl1), mRNA [Source:F	25697	17	1861404	1867575	6172	2725,65	0,04	9,59E-01
ENSRNOG00000000184	**Tmprss6**	transmembrane protease, serine 6 (Tr	315388	7	119649832	119680257	30426	20,54	0,06	9,59E-01
ENSRNOG00000005623	**Ankmy2**	ankyrin repeat and MYND domain con	314046	6	65262213	65303138	40926	2500,67	0,04	9,59E-01
ENSRNOG00000015211	**Sass6**	spindle assembly 6 homolog (C. elega	310807	2	237565447	237588679	23233	37,49	0,07	9,59E-01

ENSRNOG00000019780	**Sypl2**	synaptophysin-like 2 (Sypl2), mRNA [S	362018	2	230472384	230487115	14732	26,56	-0,07	9,59E-01
ENSRNOG00000032609				MT	5081	5152	72	1420,74	-0,06	9,59E-01
ENSRNOG00000042682	**Grpel2**	GrpE-like 2, mitochondrial (Grpel2), m	688777	18	56266330	56278569	12240	620,82	-0,04	9,59E-01
ENSRNOG00000009531	**Col9a3**	Protein Col9a3 [Source:UniProtKB/Tr	362285	3	179803040	179824998	21959	129,23	0,05	9,59E-01
ENSRNOG00000002886	**Myh10**	myosin, heavy chain 10, non-muscle (	79433	10	55017314	55148294	130981	9468,07	-0,03	9,59E-01
ENSRNOG00000008482	**Rbms1**	RNA binding motif, single stranded int	362138	3	51911276	52132833	221558	155,12	0,05	9,59E-01
ENSRNOG00000023309	**Anks6**	ankyrin repeat and sterile alpha motif	362515	5	67163440	67204853	41414	305,90	0,04	9,59E-01
ENSRNOG00000024523	**Dnai2**	dynein, axonemal, intermediate chain	360654	10	102925918	102959578	33661	96,47	-0,06	9,59E-01
ENSRNOG00000004172		pyruvate dehydrogenase kinase, isoenzyme 2 [S		10	82650223	82663773	13551	750,30	-0,05	9,59E-01
ENSRNOG00000020438	**Prpf38b**	PRP38 pre-mRNA processing factor 3	499691	2	231180384	231189409	9026	1722,81	0,04	9,59E-01
ENSRNOG00000027158	**Tsen2**	tRNA splicing endonuclease 2 homolo	312649	4	210742791	210778835	36045	263,37	0,03	9,59E-01
ENSRNOG00000018603	**RGD13082**	Protein RGD1308234 [Source:UniPro	309150	1	226285566	226292919	7354	18,23	-0,06	9,59E-01
ENSRNOG00000002074	**Rest**	RE1-silencing transcription factor (Res	83618	14	32929210	32944266	15057	277,94	-0,04	9,60E-01
ENSRNOG00000003893	**Ccnjl**	cyclin J-like (Ccnjl), mRNA [Source:Re	303059	10	28961987	29020105	58119	163,09	-0,05	9,60E-01
ENSRNOG00000003949	**Phka2**	phosphorylase kinase, alpha 2 (Phka2	678739	X	37169606	37247279	77674	59,02	-0,05	9,60E-01
ENSRNOG00000012697	**Rsb66**	Rsb-66 protein (Rsb66), mRNA [Sourc	296610	3	12620543	12629448	8906	49,40	0,07	9,60E-01
ENSRNOG00000018962	**Ctf1**	cardiotrophin 1 (Ctf1), mRNA [Source:	29201	1	206186717	206191927	5211	155,33	-0,07	9,60E-01
ENSRNOG00000001049	**RGD15657**	tumor protein, translationally-controlle	116646	15	61598728	61601614	2887	7572,41	-0,03	9,60E-01
ENSRNOG00000025406	**Iqgap2**	Protein LOC100360623 [Source:UniP	1E+08	2	45296359	45569036	272678	406,18	-0,06	9,60E-01
ENSRNOG00000005183	**Rtf1**	Rtf1, Paf1/RNA polymerase II complex	366169	3	118026883	118086227	59345	2867,19	-0,04	9,60E-01
ENSRNOG00000010800	**Hadhb**	hydroxyacyl-CoA dehydrogenase/3-ke	171155	6	37366083	37393666	27584	3145,94	0,05	9,60E-01
ENSRNOG00000011175	**LOC10036**	heterogeneous nuclear ribonucleoprot	362361	4	145901254	145909847	8594	3793,49	0,06	9,60E-01
ENSRNOG00000012886	**Maff**	v-maf avian musculoaponeurotic fibros	366960	7	120573573	120584925	11353	96,12	-0,06	9,60E-01
ENSRNOG00000015202	**Dnajc5**	DnaJ (Hsp40) homolog, subfamily C, r	79130	3	180723887	180754185	30299	2480,73	-0,04	9,60E-01
ENSRNOG00000019353	**Ctrl**	chymotrypsin-like (Ctrl), mRNA [Sourc	117184	19	48772845	48774756	1912	34,89	-0,07	9,60E-01
ENSRNOG00000021244	**Hspa12b**	heat shock protein 12B (Hspa12b), mF	311427	3	130168347	130187026	18680	108,72	-0,05	9,60E-01
ENSRNOG00000043069	**Sec22a**	SEC22 vesicle trafficking protein homo	117513	11	71720717	71777813	57097	405,55	0,04	9,60E-01
ENSRNOG00000047783	**Tmem200a**	transmembrane protein 200A (Tmem2	498987	1	21895332	21896789	1458	212,66	0,04	9,60E-01
ENSRNOG00000008481	**Reep1**	receptor accessory protein 1 (Reep1),	362384	4	164396528	164513460	116933	9593,52	-0,04	9,60E-01
ENSRNOG00000009687	**Ccdc120**	coiled-coil domain containing 120 (Cc	317377	X	16388333	16398891	10559	793,30	0,03	9,60E-01
ENSRNOG00000038002	**Ccdc62**	coiled-coil domain containing 62 (Ccd	689909	12	39928538	39964256	35719	96,87	-0,06	9,60E-01
ENSRNOG00000002397	**Slc30a10**	solute carrier family 30, member 10 (S	289353	13	108069778	108080242	10465	565,95	0,05	9,60E-01
ENSRNOG00000003147		sequestosome 1 (Sqstm1), transcript	113894	10	35463172	35474738	11567	17031,36	0,04	9,60E-01
ENSRNOG00000004280	**Tcn2**	transcobalamin 2 (Tcn2), mRNA [Sour	64365	14	84857866	84872462	14597	944,54	-0,05	9,60E-01
ENSRNOG00000004291	**Cltc**	clathrin, heavy chain (Hc) (Cltc), mRN	54241	10	76030220	76085922	55703	14723,43	0,05	9,60E-01
ENSRNOG00000005130	**Ogdh**	oxoglutarate (alpha-ketoglutarate) deh	360975	14	87376186	87442861	66676	2997,67	-0,03	9,60E-01
ENSRNOG00000006587	**LOC10091**	Protein LOC100910833 [Source:UniP	299295	6	141193723	141246255	52533	1815,62	-0,03	9,60E-01
ENSRNOG00000007597	**Rhpn1**	Protein Rhpn1 [Source:UniProtKB/TrE	300030	7	116631700	116641078	9379	545,00	0,06	9,60E-01

ENSRNOG00000010819	**Hspa4l**	heat shock protein 4-like (Hspa4l), mR	294993	2	147228804	147280566	51763	1136,86	-0,05	9,60E-01
ENSRNOG00000011130		calcitonin-related polypeptide alpha (C	24241	1	191158063	191162954	4892	19,15	-0,06	9,60E-01
ENSRNOG00000016227	**Glrx3**	glutaredoxin 3 (Glrx3), mRNA [Source	58815	1	216692884	216724091	31208	3243,88	0,04	9,60E-01
ENSRNOG00000016346	**Prkcd**	protein kinase C, delta (Prkcd), mRNA	170538	16	6588979	6608725	19747	55,20	0,06	9,60E-01
ENSRNOG00000017117		Protein Ybx2 [Source:UniProtKB/TrEN	303250	10	56291867	56297098	5232	65,42	-0,05	9,60E-01
ENSRNOG00000018812	**Rpp25**	ribonuclease P/MRP 25 subunit (Rpp2	315705	8	62060880	62062260	1381	228,23	-0,05	9,60E-01
ENSRNOG00000019812	**Nr1h2**	nuclear receptor subfamily 1, group H,	58851	1	101619967	101625287	5321	1335,97	0,04	9,60E-01
ENSRNOG00000020650		solute carrier family 17 (vesicular gluta	116638	1	102226199	102238038	11840	7418,43	0,04	9,60E-01
ENSRNOG00000023176	**Kidins220**	kinase D-interacting substrate 220 (Ki	116478	6	52955625	53048254	92630	14033,66	0,03	9,60E-01
ENSRNOG00000025651	**Ikzf5**	IKAROS family zinc finger 5 (Ikzf5), m	309031	1	208995435	209013481	18047	661,47	-0,04	9,60E-01
ENSRNOG00000033064		Protein LOC688400 [Source:UniProtK	688400	2	17606363	17875057	268695	360,89	-0,05	9,60E-01
ENSRNOG00000034102	**Plk5**	polo-like kinase 5 (Plk5), mRNA [Sour	314627	7	12382706	12391125	8420	152,63	-0,06	9,60E-01
ENSRNOG00000037252	**Slc10a3**	solute carrier family 10, member 3 (Slc	501665	1	152076587	152080391	3805	163,25	0,05	9,60E-01
ENSRNOG00000042747	**Fam72a**	family with sequence similarity 72, me	681249	13	53363989	53374272	10284	31,01	-0,05	9,60E-01
ENSRNOG00000047972	**Rpp14**	ribonuclease P/MRP 14 subunit (Rpp14), transc		15	22592972	22593448	477	609,85	0,03	9,60E-01
ENSRNOG00000000316	**Sobp**	sine oculis-binding protein homolog (D	309860	20	49411760	49571386	159627	2151,32	0,04	9,60E-01
ENSRNOG00000011305	**Sox10**	SRY (sex determining region Y)-box 1	29361	7	120387071	120397340	10270	967,27	-0,06	9,60E-01
ENSRNOG00000011700	**Ptprm**	protein tyrosine phosphatase, recepto	29616	9	114341760	115046974	705215	1218,24	-0,04	9,60E-01
ENSRNOG00000011886		unconventional myosin-Ie [Source:Re	25484	8	76241676	76323566	81891	527,21	-0,05	9,60E-01
ENSRNOG00000018294	**Hspa5**	heat shock protein 5 (Hspa5), mRNA [	25617	3	19157874	19162332	4459	8650,83	0,04	9,60E-01
ENSRNOG00000042691	**Armc7**	armadillo repeat containing 7 (Armc7),	287827	10	104227119	104243604	16486	300,13	-0,04	9,60E-01
ENSRNOG00000050701				2	11747905	11748199	295	341,69	-0,06	9,60E-01
ENSRNOG00000006075	**Fez1**	fasciculation and elongation protein ze	81730	8	39307654	39353312	45659	13320,04	0,03	9,60E-01
ENSRNOG00000011971	**C1s**	complement component 1, s subcomp	192262	4	224162096	224174113	12018	322,12	-0,07	9,60E-01
ENSRNOG00000021105	**Gabpb2**	Protein Gabpb2 [Source:UniProtKB/T	295263	2	215855910	215874322	18413	38,28	-0,05	9,60E-01
ENSRNOG00000028380		coiled-coil domain containing 66 [Source:MGI Sy		16	3075032	3105389	30358	171,50	-0,06	9,61E-01
ENSRNOG00000022060	**Magi1**	membrane associated guanylate kinas	500261	4	190207466	190470946	263481	1389,49	-0,04	9,61E-01
ENSRNOG00000031312	**Tnfrsf1a**	tumor necrosis factor receptor superfa	25625	4	224881445	224894088	12644	1518,47	-0,06	9,61E-01
ENSRNOG00000019670	**Wdr3**	WD repeat domain 3 (Wdr3), mRNA [S	310720	2	221899195	221921857	22663	618,65	0,05	9,61E-01
ENSRNOG00000027253	**Pcdhga10**	protocadherin gamma subfamily A, 10	498849	18	30589911	30592472	2562	382,39	-0,04	9,61E-01
ENSRNOG00000007324	**Plxna2**	plexin A2 (Plxna2), mRNA [Source:Re	289392	13	117922569	118118393	195825	5935,93	0,04	9,61E-01
ENSRNOG00000004723	**Fcf1**	FCF1 small subunit (SSU) processom	299198	6	116748859	116760725	11867	459,25	0,04	9,62E-01
ENSRNOG00000007596		E3 ubiquitin-protein ligase rififylin [Sou	282844	10	69788337	69851152	62816	330,16	0,03	9,62E-01
ENSRNOG00000004328	**Deptor**	Protein Deptor [Source:UniProtKB/TrE	314979	7	95432383	95620730	188348	60,31	-0,04	9,62E-01
ENSRNOG00000011504	**Akap2**	A kinase (PRKA) anchor protein 2 (Aka	298024	5	79083351	79123669	40319	725,18	-0,03	9,62E-01
ENSRNOG00000020271	**Tial1**	Tia1 cytotoxic granule-associated RNA	361655	1	206892011	206913052	21042	1492,55	-0,05	9,62E-01
ENSRNOG00000028238		SH3 domain binding glutamic acid-rich	498066	11	40086028	40106740	20713	18,44	-0,07	9,62E-01
ENSRNOG00000046984	**St6galnac6**	ST6 (alpha-N-acetyl-neuraminyl-2,3-be	407765	3	16979690	16987558	7869	2055,34	0,04	9,62E-01

ENSRNOG00000049437	**Gpc1**	glypican 1 (Gpc1), mRNA [Source:Ref	58920	9	99664180	99692723	28544	4130,34	0,04	9,62E-01
ENSRNOG00000009506	**Mre11a**	MRE11 meiotic recombination 11 hom	64046	8	13245885	13289958	44074	335,31	0,04	9,62E-01
ENSRNOG00000009708	**Tas1r1**	taste receptor, type 1, member 1 (Tas1	29407	5	172758655	172770376	11722	153,06	-0,06	9,62E-01
ENSRNOG00000012486		primase, DNA, polypeptide 2 (Prim2),	301323	9	38217427	38424414	206988	252,79	0,04	9,62E-01
ENSRNOG00000016257	**Cotl1**	coactosin-like 1 (Dictyostelium) (Cotl1	361422	19	63210065	63243545	33481	1974,02	0,04	9,62E-01
ENSRNOG00000046697	**RGD15608**	Protein RGD1560833 [Source:UniPro	1E+08	14	30188746	30202158	13413	625,07	0,03	9,62E-01
ENSRNOG00000022395		trinucleotide repeat containing 6C [Sou	303774	10	106337152	106400601	63450	2449,44	-0,03	9,62E-01
ENSRNOG00000012662		lysosomal trafficking regulator [Source:MGI Sym		17	91981718	92183054	201337	501,97	-0,06	9,62E-01
ENSRNOG00000006069	**Eif2ak3**	eukaryotic translation initiation factor 2	29702	4	163428182	163488913	60732	1023,45	-0,04	9,62E-01
ENSRNOG00000047220		Uncharacterized protein [Source:UniProtKB/TrE		3	156103697	156104661	965	17,56	0,07	9,62E-01
ENSRNOG00000005662	**Grm7**	glutamate receptor, metabotropic 7 (G	81672	4	206090084	206680107	590024	1014,37	-0,05	9,62E-01
ENSRNOG00000048095	**RGD15648**	Protein RGD1564801 [Source:UniPro	499084	1	76520725	76522170	1446	22,71	0,07	9,62E-01
ENSRNOG00000014917	**Nck1**	NCK adaptor protein 1 (Nck1), mRNA	300955	8	108204373	108207897	3525	184,23	-0,06	9,62E-01
ENSRNOG00000018254	**Sncaip**	synuclein, alpha interacting protein (Si	307309	18	46954706	47092054	137349	1510,68	-0,04	9,62E-01
ENSRNOG00000019189	**Acat2**	acetyl-CoA acetyltransferase 2 (Acat2	308100	1	51757962	51776935	18974	1681,49	0,06	9,62E-01
ENSRNOG00000027921	**Rlf**	Insulin-like 3 [Source:UniProtKB/TrEM	313566	5	143900361	143938190	37830	773,43	0,05	9,62E-01
ENSRNOG00000046878		Shc SH2-domain binding protein 1 [Sc	364648	16	89996176	90026610	30435	139,14	-0,06	9,62E-01
ENSRNOG00000000028	**Nde1**	nudE neurodevelopment protein 1 (Nd	83836	10	11540861	11576271	35411	178,05	0,05	9,62E-01
ENSRNOG00000012297	**March8**	membrane-associated ring finger (C3H	312656	4	214232834	214345405	112572	3108,22	-0,04	9,62E-01
ENSRNOG00000014090	**Retsat**	retinol saturase (all trans retinol 13,14	246298	4	165235748	165244511	8764	1640,76	0,06	9,62E-01
ENSRNOG00000026034		Ubiquitin carboxyl-terminal hydrolase [Source:U		14	104962650	105100491	137842	2665,14	-0,05	9,62E-01
ENSRNOG00000047752	**Pfn3**	profilin 3 (Pfn3), mRNA [Source:RefSe	685936	17	11855665	11856192	528	23,04	-0,06	9,62E-01
ENSRNOG00000017212	**Spsb1**	splA/ryanodine receptor domain and S	313722	5	170557219	170568525	11307	158,70	-0,04	9,62E-01
ENSRNOG00000019351		chitinase domain containing 1 (Chid1)	293628	1	220956823	220991829	35007	99,32	0,04	9,62E-01
ENSRNOG00000010149	**Cmc1**	COX assembly mitochondrial protein 1	363162	8	125679760	125737762	58003	350,96	-0,04	9,63E-01
ENSRNOG00000014319	**Smchd1**	Protein Smchd1 [Source:UniProtKB/T	316732	9	119128952	119271724	142773	929,86	0,06	9,63E-01
ENSRNOG00000017583	**Mapkap1**	mitogen-activated protein kinase asso	296648	3	18809022	19021959	212938	1497,45	0,03	9,63E-01
ENSRNOG00000026472	**Kctd8**	potassium channel tetramerization dor	364148	14	62015922	62261874	245953	56,32	-0,05	9,63E-01
ENSRNOG00000047491		trophinin [Source:MGI Symbol;Acc:MG	1E+08	X	23502765	23511033	8269	1394,52	0,04	9,63E-01
ENSRNOG00000013877	**Ttc23**	tetratricopeptide repeat domain 23 (Ttc	308708	1	129702818	129753636	50819	94,83	0,04	9,63E-01
ENSRNOG00000031896	**Stt3a**	STT3A, subunit of the oligosaccharyltr	500972	8	39207076	39246422	39347	2454,27	-0,05	9,63E-01
ENSRNOG00000000527	**Mtch1**	mitochondrial carrier 1 (Mtch1), mRNA	294313	20	9177064	9200859	23796	14128,89	0,04	9,63E-01
ENSRNOG00000050650		tetratricopeptide repeat domain 16 [Source:MGI		3	17146832	17148228	1397	20,93	0,06	9,63E-01
ENSRNOG00000001099	**Rbak**	RB-associated KRAB zinc finger (Rba	288489	12	15470256	15482463	12208	201,66	-0,03	9,63E-01
ENSRNOG00000014852	**Fbxo30**	F-box protein 30 (Fbxo30), mRNA [So	308283	1	7018429	7039842	21414	297,48	-0,05	9,63E-01
ENSRNOG00000022247	**Nr2c2ap**	nuclear receptor 2C2-associated prote	361128	16	20886520	20888715	2196	1018,19	-0,05	9,63E-01
ENSRNOG00000010194	**Ift43**	intraflagellar transport 43 homolog (Ch	299209	6	119247640	119323931	76292	265,94	0,04	9,64E-01
ENSRNOG00000004563		SEC24 family, member A (S. cerevisia	287275	10	36913611	36988825	75215	753,00	0,03	9,64E-01

ENSRNOG00000015456	**Zfp787**	zinc finger protein 787 (Zfp787), mRN	365176	1	72827317	72840114	12798	571,52	0,04	9,64E-01
ENSRNOG00000019609	**B4galt2**	UDP-Gal:betaGlcNAc beta 1,4- galact	313536	5	140496559	140505030	8472	3932,45	0,05	9,64E-01
ENSRNOG00000006304	**Mdm2**	p53 E3 ubiquitin protein ligase (Mdm2	314856	7	60719029	60743291	24263	1370,59	-0,04	9,64E-01
ENSRNOG00000015787	**Leprel4**	leprecan-like 4 (Leprel4), mRNA [Sour	59101	10	88112407	88118469	6063	341,49	-0,05	9,64E-01
ENSRNOG00000012151	**Itpripl1**	inositol 1,4,5-trisphosphate receptor in	499885	3	126142197	126145117	2921	38,19	0,05	9,64E-01
ENSRNOG00000000967	**Aacs**	acetoacetyl-CoA synthetase (Aacs), m	65984	12	38381589	38425067	43479	2723,73	0,04	9,64E-01
ENSRNOG00000004130	**Anapc4**	anaphase promoting complex subunit	305420	14	60589356	60621597	32242	844,01	-0,04	9,64E-01
ENSRNOG00000007001	**Nek9**	NIMA-related kinase 9 (Nek9), mRNA	299204	6	116390547	116435517	44971	2965,44	0,03	9,64E-01
ENSRNOG00000013388	**Pgap1**	post-GPI attachment to proteins 1 (Pg	316400	9	60749693	60818301	68609	1129,40	-0,04	9,64E-01
ENSRNOG00000022347	**Fam173b**	family with sequence similarity 173, m	499561	2	104351684	104366724	15041	315,91	-0,03	9,64E-01
ENSRNOG00000042886	**LOC68980**	ribosomal protein S28 (Rps28), mRNA	689805	7	18859671	18861040	1370	19,78	-0,06	9,64E-01
ENSRNOG00000030149		latrophilin 3 (Lphn3), mRNA [Source:R	170641	14	28214945	28671248	456304	1728,53	0,03	9,64E-01
ENSRNOG00000003063	**Phka1**	phosphorylase kinase, alpha 1 (Phka1	64561	X	73220952	73355698	134747	552,96	-0,03	9,64E-01
ENSRNOG00000003114	**B4galt4**	UDP-Gal:betaGlcNAc beta 1,4-galacto	303923	11	67525273	67550867	25595	454,70	0,05	9,64E-01
ENSRNOG00000003127	**Spryd4**	SPRY domain containing 4 (Spryd4), r	288772	7	2600837	2602657	1821	541,90	-0,03	9,64E-01
ENSRNOG00000005993	**Oxsm**	3-oxoacyl-ACP synthase, mitochondria	289934	15	14508023	14511770	3748	368,19	0,03	9,64E-01
ENSRNOG00000006527	**Slc6a1**	solute carrier family 6 (neurotransmitte	79212	4	209570471	209585792	15322	13605,79	-0,04	9,64E-01
ENSRNOG00000007065		RNA polymerase II associated protein 3 [Source		7	139359860	139386634	26775	608,11	0,04	9,64E-01
ENSRNOG00000007364	**Rab15**	RAB15, member RAS oncogene famil	299156	6	109242428	109266332	23905	2442,30	0,03	9,64E-01
ENSRNOG00000014387	**Chac1**	ChaC, cation transport regulator homo	362196	3	117711150	117714352	3203	611,80	0,05	9,64E-01
ENSRNOG00000015036	**Ctgf**	connective tissue growth factor (Ctgf),	64032	1	23331547	23334660	3114	294,21	0,05	9,64E-01
ENSRNOG00000017231	**Adam9**	ADAM metallopeptidase domain 9 (Ad	290834	16	71459144	71536608	77465	1534,44	0,04	9,64E-01
ENSRNOG00000019099	**Zfp334**	Protein Zfp334 [Source:UniProtKB/Tr	685574	3	168239929	168243006	3078	476,72	0,03	9,64E-01
ENSRNOG00000050839				2	83834741	83835296	556	27,37	-0,06	9,64E-01
ENSRNOG00000011524	**Mkln1**	muskelin 1, intracellular mediator cont	83536	4	58448153	58570396	122244	648,27	-0,04	9,64E-01
ENSRNOG00000005633	**Zbtb49**	zinc finger and BTB domain containing	305428	14	77303902	77325596	21695	217,54	-0,04	9,64E-01
ENSRNOG00000017434	**Mgat3**	mannosyl (beta-1,4-)-glycoprotein beta	29582	7	121398823	121425021	26199	2062,91	-0,03	9,64E-01
ENSRNOG00000019036	**Ldhd**	lactate dehydrogenase D (Ldhd), mRN	307858	19	54634157	54656104	21948	55,56	-0,06	9,64E-01
ENSRNOG00000033206	**Entpd5**	ectonucleoside triphosphate diphosph	314312	6	117425815	117455726	29912	389,12	-0,04	9,64E-01
ENSRNOG00000033694	**Klf16**	Kruppel-like factor 16 (Klf16), mRNA [	690820	7	12174235	12184049	9815	1444,31	-0,05	9,64E-01
ENSRNOG00000034234		cytochrome c oxidase subunit I (mitoc	26195	MT	5323	6867	1545	446098,72	-0,02	9,64E-01
ENSRNOG00000035628	**Mir9-2**	rno-mir-9b-2 [Source:miRBase;Acc:MI0015439]		2	11746809	11746926	118	113,74	-0,05	9,64E-01
ENSRNOG00000028611				X	16736366	16737916	1551	33,18	0,05	9,64E-01
ENSRNOG00000000596	**Fyn**	FYN oncogene related to SRC, FGR,	25150	20	46161060	46352027	190968	5866,59	-0,03	9,64E-01
ENSRNOG00000015442	**Sfxn3**	sideroflexin 3 (Sfxn3), mRNA [Source:	65042	1	272239201	272247546	8346	3243,53	0,04	9,64E-01
ENSRNOG00000000529	**Pim1**	pim-1 oncogene (Pim1), mRNA [Sourc	24649	20	10364285	10545832	181548	88,13	-0,04	9,65E-01
ENSRNOG00000001849	**Mapk1**	mitogen activated protein kinase 1 (Ma	116590	11	91262522	91325572	63051	8280,77	0,04	9,65E-01
ENSRNOG00000002271	**Slain2**	SLAIN motif family, member 2 (Slain2)	305310	14	37612584	37681311	68728	2037,00	0,04	9,65E-01

ENSRNOG00000003545	**Uchl5**	ubiquitin carboxyl-terminal hydrolase L	360853	13	65556707	65592648	35942	250,65	-0,05	9,65E-01
ENSRNOG00000004294	**Ascl1**	achaete-scute complex homolog 1 (Dr	64186	7	28152425	28154267	1843	1237,85	-0,04	9,65E-01
ENSRNOG00000005348	**Pamr1**	peptidase domain containing associate	311252	3	99054239	99145372	91134	60,93	-0,05	9,65E-01
ENSRNOG00000005357	**Ctc1**	CTS telomere maintenance complex c	303238	10	55339019	55359673	20655	1591,04	-0,03	9,65E-01
ENSRNOG00000006231	**Ptpro**	protein tyrosine phosphatase, recepto	50677	4	235508260	235718672	210413	3497,74	-0,04	9,65E-01
ENSRNOG00000006796	**Zfp146**	Protein LOC687516 [Source:UniProtK	687516	10	75331264	75332142	879	355,12	-0,04	9,65E-01
ENSRNOG00000007757	**Ppp3cb**	protein phosphatase 3, catalytic subur	24675	15	8293592	8338152	44561	6761,70	-0,04	9,65E-01
ENSRNOG00000016468	**Tpmt**	thiopurine S-methyltransferase (Tpmt)	690050	17	19889511	19907982	18472	115,76	-0,04	9,65E-01
ENSRNOG00000017382	**Snx33**	sorting nexin 33 (Snx33), mRNA [Sour	315696	8	60154183	60164559	10377	534,94	0,04	9,65E-01
ENSRNOG00000018998	**Yipf3**	Yip1 domain family, member 3 (Yipf3),	301245	9	16011992	16017295	5304	2227,36	0,03	9,65E-01
ENSRNOG00000028227		polybromo 1 [Source:MGI Symbol;Acc	306254	16	7037836	7128727	90892	1807,04	0,03	9,65E-01
ENSRNOG00000031855	**Actr3b**	ARP3 actin-related protein 3 homolog B (yeast)		4	5668135	5696597	28463	812,13	-0,04	9,65E-01
ENSRNOG00000046285	**Pcdha9**	protocadherin alpha 9 (Pcdha9), mRN	393090	18	29701815	29924444	222630	81,41	-0,05	9,65E-01
ENSRNOG00000050312	**Ism2**	Protein LOC681936 [Source:UniProtK	681936	6	120592068	120611725	19658	27,42	-0,05	9,65E-01
ENSRNOG00000010041	**Ube2g1**	ubiquitin-conjugating enzyme E2G 1 (l	64631	10	58911569	58992363	80795	2094,58	0,04	9,65E-01
ENSRNOG00000013217	**Ttc33**	tetratricopeptide repeat domain 33 (Tt	294774	2	73921832	73962356	40525	357,35	0,04	9,65E-01
ENSRNOG00000038319	**Akr1c19**	aldo-keto reductase family 1, member	307096	17	71363870	71384879	21010	37,51	0,05	9,65E-01
ENSRNOG00000021681	**Eea1**	early endosome antigen 1 (Eea1), mR	314764	7	37144349	37233282	88934	463,45	-0,06	9,65E-01
ENSRNOG00000010694	**Tpbg**	trophoblast glycoprotein (Tpbg), mRN/	83684	8	93006542	93009911	3370	561,68	-0,03	9,65E-01
ENSRNOG00000013119		Fgfr1 oncogene partner [Source:MGI S	683722	1	54371830	54403026	31197	144,05	0,05	9,65E-01
ENSRNOG00000016047		microtubule-actin crosslinking factor 1 [Source:M		5	144830885	145151309	320425	8798,80	0,04	9,65E-01
ENSRNOG00000016507	**LOC68767!**	small nuclear ribonucleoprotein polype	680394	4	182738544	182746092	7549	372,21	-0,05	9,65E-01
ENSRNOG00000018170	**Slc27a1**	solute carrier family 27 (fatty acid trans	94172	16	19859540	19876618	17079	3329,35	-0,05	9,65E-01
ENSRNOG00000019340		centrosomal protein 250 [Source:MGI	311573	3	157872143	157908796	36654	725,85	-0,03	9,65E-01
ENSRNOG00000020066	**Pcdhb11**	Protein Pcdhb11 [Source:UniProtKB/T	291648	18	30203513	30205831	2319	147,71	-0,04	9,65E-01
ENSRNOG00000022006		RCG37698, isoform CRA_b; Uncharacterized pr		3	35194081	35196003	1923	124,64	0,04	9,65E-01
ENSRNOG00000024631		chondroadherin-like [Source:MGI Sym	1E+08	7	122895768	122900832	5065	185,78	-0,06	9,65E-01
ENSRNOG00000027096	**Ctsw**	cathepsin W (Ctsw), mRNA [Source:R	293676	1	227773906	227777294	3389	19,37	0,06	9,65E-01
ENSRNOG00000028330				9	53285314	53285877	564	19,06	-0,06	9,65E-01
ENSRNOG00000028498	**Wdr1**	WD repeat domain 1 (Wdr1), mRNA [S	360950	14	76974171	77007900	33730	8727,19	0,02	9,65E-01
ENSRNOG00000029264	**Kcnn1**	potassium intermediate/small conduct	54261	16	20194361	20205054	10694	325,96	-0,06	9,65E-01
ENSRNOG00000042189	**Rab31**	RAB31, member RAS oncogene famil	246324	9	112902313	113037023	134711	10190,92	0,03	9,65E-01
ENSRNOG00000050407	**Hiat1**	hippocampus abundant gene transcrip	1E+08	1	71185529	71217901	32373	33,22	0,06	9,65E-01
ENSRNOG00000001642	**Tbc1d23**	Protein Tbc1d23; TBC1 domain family	304019	11	48594603	48651071	56469	820,09	-0,03	9,65E-01
ENSRNOG00000002294	**Snx29**	sorting nexin 29 (Snx29), mRNA [Sour	689142	10	2710405	3115786	405382	638,12	-0,04	9,65E-01
ENSRNOG00000015859	**Chdh**	choline dehydrogenase (Chdh), mRN/	290551	16	6009402	6042912	33511	126,46	0,05	9,65E-01
ENSRNOG00000037243	**Taz**	tafazzin (Taz), mRNA [Source:RefSeq	363521	1	152161156	152168740	7585	707,29	0,03	9,65E-01
ENSRNOG00000012611	**Lpxn**	leupaxin (Lpxn), mRNA [Source:RefSe	293783	1	236795798	236827489	31692	140,45	-0,04	9,65E-01

ENSRNOG00000003834	**Map2k4**	mitogen activated protein kinase kinas	287398	10	51951707	52022942	71236	738,92	-0,04	9,65E-01
ENSRNOG00000012030	**Wwox**	WW domain-containing oxidoreductas	292041	19	57582357	57870662	288306	120,51	0,04	9,65E-01
ENSRNOG00000037160	**Bloc1s6**	biogenesis of lysosomal organelles co	317630	3	121407586	121418323	10738	1117,58	0,04	9,65E-01
ENSRNOG00000011431	**Mfhas1**	malignant fibrous histiocytoma amplifie	306508	16	59803007	59882577	79571	1246,72	0,03	9,65E-01
ENSRNOG00000007984	**Amer1**	APC membrane recruitment protein 1	501584	X	65585784	65601668	15885	581,89	0,03	9,65E-01
ENSRNOG00000010096		5-azacytidine-induced protein 2 [Sour	316051	8	125653644	125677999	24356	1178,07	-0,03	9,65E-01
ENSRNOG00000012012	**Pinx1**	PIN2/TERF1 interacting, telomerase ir	305963	15	50970604	51029711	59108	250,30	-0,03	9,65E-01
ENSRNOG00000012962	**Nudt16**	nudix (nucleoside diphosphate linked r	363129	8	113059261	113061297	2037	537,13	-0,02	9,65E-01
ENSRNOG00000014566	**Fkbp11**	FK506 binding protein 11 (Fkbp11), ml	300211	X	114908415	114911848	3434	133,54	0,04	9,65E-01
ENSRNOG00000029441	**Klhl2**		290692	16	26635307	26729968	94662	1846,37	-0,04	9,65E-01
ENSRNOG00000036816	**Wls**	wntless homolog (Drosophila) (Wls), tr	362065	2	284950842	285064162	113321	1988,69	0,03	9,65E-01
ENSRNOG00000037263	**Irak1**	interleukin-1 receptor-associated kinas	363520	1	152464438	152474011	9574	2906,29	-0,03	9,65E-01
ENSRNOG00000039656	**LOC10091:**	ATP-binding cassette, subfamily A (AE	287112	10	13446127	13533395	87269	34,66	0,06	9,65E-01
ENSRNOG00000040190	**RGD1563847**			14	1440025	1441601	1577	22,00	0,06	9,65E-01
ENSRNOG00000003674	**Pir**	pirin (iron-binding nuclear protein) (Pir	363465	X	32226074	32341358	115285	337,78	-0,05	9,65E-01
ENSRNOG00000009951	**Aif1l**	allograft inflammatory factor 1-like (Aif	362107	3	14598261	14622945	24685	796,76	-0,03	9,65E-01
ENSRNOG00000015201	**RGD15620**	similar to expressed sequence AI1180	501002	8	58480570	58593993	113424	322,07	0,03	9,65E-01
ENSRNOG00000043391	**Pecam1**	platelet/endothelial cell adhesion mole	29583	10	94598952	94632587	33636	37,78	0,06	9,65E-01
ENSRNOG00000018997	**Myh7b**	myosin, heavy chain 7B, cardiac musc	311570	3	157494398	157518151	23754	55,59	0,05	9,65E-01
ENSRNOG00000004680		kinesin heavy chain isoform 5C [Sour	311024	3	40279088	40366547	87460	8935,83	0,03	9,65E-01
ENSRNOG00000013508	**LOC30261:**	ribosomal protein L31 (Rpl31), mRNA	64298	9	45774873	45778902	4030	505,94	0,04	9,66E-01
ENSRNOG00000018692	**Mc4r**	melanocortin 4 receptor (Mc4r), mRN/	25635	18	61799647	61800645	999	30,23	-0,06	9,66E-01
ENSRNOG00000021660	**Nprl2**	nitrogen permease regulator-like 2 (S.	363138	8	115654210	115657382	3173	549,94	-0,03	9,66E-01
ENSRNOG00000024306				2	242965052	242965619	568	19,99	0,05	9,66E-01
ENSRNOG00000026119	**Ywhah**	tyrosine 3-monooxygenase/tryptophar	25576	14	83739010	83748417	9408	13654,68	0,04	9,66E-01
ENSRNOG00000048831	**Sowahc**	sosondowah ankyrin repeat domain fa	503306	20	30621651	30626117	4467	708,37	-0,03	9,66E-01
ENSRNOG00000049152	**Nme4**	non-metastatic cells 4, protein express	685679	10	15275267	15279122	3856	60,84	0,06	9,66E-01
ENSRNOG00000003026	**Rad23a**	RAD23 homolog A (S. cerevisiae) (Ra	361381	19	36925113	36930994	5882	917,77	0,04	9,66E-01
ENSRNOG00000000441	**Gpsm3**	G-protein signaling modulator 3 (Gpsn	406163	20	6434602	6436463	1862	68,81	-0,04	9,66E-01
ENSRNOG00000001091	**Hip1r**	huntingtin interacting protein 1 related	81917	12	39895516	39924082	28567	1463,32	-0,03	9,66E-01
ENSRNOG00000001232	**Slc19a1**	solute carrier family 19 (folate transpo	29723	20	14497682	14514760	17079	205,53	0,03	9,66E-01
ENSRNOG00000002488	**Galnt10**	UDP-N-acetyl-alpha-D-galactosamine	170501	10	42867364	43008297	140934	669,29	-0,05	9,66E-01
ENSRNOG00000002536	**C1galt1c1**	C1GALT1-specific chaperone 1 (C1ga	302499	X	125006189	125010692	4504	360,45	-0,05	9,66E-01
ENSRNOG00000003929	**Pcdh19**	protocadherin 19 (Pcdh19), mRNA [Sc	317183	X	104235047	104335006	99960	1340,92	0,04	9,66E-01
ENSRNOG00000004245	**Mgst3**	microsomal glutathione S-transferase	289197	13	90244300	90265035	20736	2751,83	-0,05	9,66E-01
ENSRNOG00000004753	**Napb**	N-ethylmaleimide-sensitive factor atta	499903	3	149431212	149473190	41979	754,69	-0,04	9,66E-01
ENSRNOG00000004874	**Flrt3**	fibronectin leucine rich transmembran	366205	3	141134897	141147030	12134	638,75	-0,04	9,66E-01
ENSRNOG00000005263	**Scaf11**	SR-related CTD-associated factor 11 (	312030	7	137439150	137471025	31876	646,91	0,04	9,66E-01

ENSRNOG00000005600	**Nr4a2**	nuclear receptor subfamily 4, group A,	54278	3	48183787	48189254	5468	399,75	-0,04	9,66E-01
ENSRNOG00000006441	**Zbtb25**	zinc finger and BTB domain containing	314245	6	108791852	108800583	8732	235,50	-0,03	9,66E-01
ENSRNOG00000006697	**Tmem55a**	transmembrane protein 55A (Tmem55	362490	5	33079533	33126619	47087	1951,66	-0,03	9,66E-01
ENSRNOG00000007234		Lanosterol 14-alpha demethylase [So	25427	4	27078469	27096431	17963	6183,35	-0,04	9,66E-01
ENSRNOG00000007752	**Zmynd19**	zinc finger, MYND-type containing 19	311791	3	2140257	2149618	9362	618,19	0,04	9,66E-01
ENSRNOG00000007998	**Ssb**	Sjogren syndrome antigen B (Ssb), ml	81783	3	62727509	62737009	9501	1270,35	0,05	9,66E-01
ENSRNOG00000008364	**Cat**	catalase (Cat), mRNA [Source:RefSec	24248	3	100021859	100053940	32082	2524,49	-0,04	9,66E-01
ENSRNOG00000008489	**Ppih**	Peptidyl-prolyl cis-trans isomerase [S	366461	5	142169207	142177993	8787	465,89	0,03	9,66E-01
ENSRNOG00000009161	**Clasp2**	cytoplasmic linker associated protein 2	114514	8	121390289	121499025	108737	10037,36	-0,04	9,66E-01
ENSRNOG00000009450	**Hcn4**	hyperpolarization activated cyclic nucl	59266	8	63367893	63405326	37434	701,49	-0,04	9,66E-01
ENSRNOG00000009893	**Uxt**	ubiquitously-expressed, prefoldin-like	299313	X	2090451	2102800	12350	532,25	0,04	9,66E-01
ENSRNOG00000011111	**RGD13094**	similar to mKIAA1737 protein (RGD13	314330	6	120260096	120281691	21596	2058,71	0,03	9,66E-01
ENSRNOG00000011167	**Wdhd1**	WD repeat and HMG-box DNA binding	305827	15	27962416	27996815	34400	308,31	-0,04	9,66E-01
ENSRNOG00000011987	**Cd2ap**	CD2-associated protein (Cd2ap), mRN	316258	9	19659500	19720953	61454	104,05	0,05	9,66E-01
ENSRNOG00000012975	**Ggcx**	gamma-glutamyl carboxylase (Ggcx),	81716	4	165047672	165063176	15505	404,92	0,04	9,66E-01
ENSRNOG00000013636	**Ppil3**	peptidylprolyl isomerase (cyclophilin)-l	301432	9	65122704	65136360	13657	264,44	0,04	9,66E-01
ENSRNOG00000014838	**Glipr2**	Protein Glipr2; RCG54881 [Source:U	679819	5	63950141	63968973	18833	134,98	-0,05	9,66E-01
ENSRNOG00000015158		Protein Pikfyve; Similar to phosphatidy	316457	9	70568996	70656647	87652	1117,46	-0,03	9,66E-01
ENSRNOG00000015419	**Spem1**	spermatid maturation 1 (Spem1), mRN	691981	10	56173581	56174867	1287	85,08	-0,05	9,66E-01
ENSRNOG00000016519	**Kdm1b**	lysine (K)-specific demethylase 1B (K	306819	17	19908417	19948376	39960	664,54	-0,03	9,66E-01
ENSRNOG00000016530	**Nsa2**	NSA2 ribosome biogenesis homolog (	171456	2	47053384	47060005	6622	322,39	0,05	9,66E-01
ENSRNOG00000016561	**Ns5atp9**	NS5A (hepatitis C virus) transactivated	300795	8	71186635	71199152	12518	649,20	0,03	9,66E-01
ENSRNOG00000016740	**Fam210a**	family with sequence similarity 210, m	307343	18	63227537	63298831	71295	166,71	0,04	9,66E-01
ENSRNOG00000016905	**Hmg20a**	high mobility group 20A (Hmg20a), mF	315689	8	59650251	59727759	77509	2835,72	0,03	9,66E-01
ENSRNOG00000018935				1	48887512	48888429	918	86,87	-0,04	9,66E-01
ENSRNOG00000019023	**Tars**	threonyl-tRNA synthetase (Tars), mRN	294810	2	83279780	83298533	18754	1697,69	-0,04	9,66E-01
ENSRNOG00000019159		janus kinase and microtubule-interacti	307479	18	37525249	37743590	218342	966,93	0,05	9,66E-01
ENSRNOG00000019380	**Cers5**	ceramide synthase 5 (Cers5), mRNA [	366984	X	115891039	115929134	38096	2630,92	0,03	9,66E-01
ENSRNOG00000020605	**RGD15643**	similar to phosphoseryl-tRNA kinase (l	361661	1	208982377	208992787	10411	218,82	-0,04	9,66E-01
ENSRNOG00000020782	**Pspc1**	paraspeckle component 1 (Pspc1), mF	305910	15	40822487	40882550	60064	1567,15	-0,03	9,66E-01
ENSRNOG00000021725	**Mlec**	malectin (Mlec), mRNA [Source:RefSe	304543	12	49014186	49024661	10476	1592,18	-0,03	9,66E-01
ENSRNOG00000022354	**Pusl1**	pseudouridylate synthase-like 1 (Pusl1	362681	5	176811652	176815508	3857	429,06	-0,02	9,66E-01
ENSRNOG00000023093	**Mis18bp1**	MIS18 binding protein 1 (Mis18bp1), n	689296	6	96368090	96413264	45175	49,77	-0,06	9,66E-01
ENSRNOG00000023687	**Rap2a**	RAS related protein 2a (Rap2a), mRN	114560	15	109253818	109254308	491	161,74	0,03	9,66E-01
ENSRNOG00000025252	**Yars2**	tyrosyl-tRNA synthetase 2 (mitochond	287924	11	91936943	91942704	5762	244,07	0,04	9,66E-01
ENSRNOG00000026316	**Wdr43**	WD repeat domain 43 (Wdr43), mRNA	362703	6	33299540	33339177	39638	1236,84	-0,03	9,66E-01
ENSRNOG00000027002		Protein Mast4 [Source:UniProtKB/TrE	310040	2	51998511	52583349	584839	21,70	-0,05	9,66E-01
ENSRNOG00000027149	**RGD15638**	ribosomal protein L10 (Rpl10), mRNA	81764	1	152178398	152180608	2211	6502,44	0,03	9,66E-01

ENSRNOG00000027213	**Asphd1**	Protein RGD1306636 [Source:UniPro	293498	1	205359580	205362551	2972	360,26	-0,04	9,66E-01
ENSRNOG00000028753	**B9d2**	B9 protein domain 2 (B9d2), mRNA [S	308443	1	83735614	83741208	5595	476,39	-0,05	9,66E-01
ENSRNOG00000006609	**Psme4**	proteasome (prosome, macropain) act	498433	14	114823668	114926542	102875	1754,05	0,05	9,66E-01
ENSRNOG00000013719	**Dph5**	diphthamide biosynthesis 5 (Dph5), m	295394	2	236920286	236953013	32728	174,65	-0,03	9,66E-01
ENSRNOG00000014119	**Farsb**	phenylalanyl-tRNA synthetase, beta su	301544	9	84083346	84142523	59178	952,10	-0,03	9,66E-01
ENSRNOG00000015396	**Sptan1**	spectrin, alpha, non-erythrocytic 1 (Sp	64159	3	13878433	13950970	72538	30007,90	-0,03	9,66E-01
ENSRNOG00000011445	**Nkain1**	Protein Nkain1 [Source:UniProtKB/Tr	500557	5	152374954	152381791	6838	892,20	-0,04	9,67E-01
ENSRNOG00000018635	**Ccnb1**	cyclin B1 (Ccnb1), mRNA [Source:Ref	25203	2	49941586	49948770	7185	420,29	0,04	9,67E-01
ENSRNOG00000018808	**Vip**	vasoactive intestinal peptide (Vip), mR	117064	1	43498384	43506462	8079	241,28	-0,06	9,67E-01
ENSRNOG00000001633	**Tfg**	Trk-fused gene (Tfg), mRNA [Source:R	360709	11	49365948	49392422	26475	2278,74	-0,02	9,67E-01
ENSRNOG00000005326	**Cnrip1**	cannabinoid receptor interacting prote	364208	14	100291061	100320330	29270	5959,21	-0,03	9,67E-01
ENSRNOG00000013565	**LOC10091**	zinc finger protein 507 (Zfp507), mRN	292816	1	93220458	93250626	30169	324,30	-0,04	9,67E-01
ENSRNOG00000023230		predicted gene 527 [Source:MGI Symbol;Acc:M		6	96145979	96154792	8814	85,23	-0,04	9,67E-01
ENSRNOG00000036534				8	76400651	76400745	95	46,20	-0,04	9,67E-01
ENSRNOG00000049343		Morc4 protein; Uncharacterized protein [Source		3	53403188	53417392	14205	40,81	-0,04	9,67E-01
ENSRNOG00000002912	**Parn**	poly(A)-specific ribonuclease (Parn), n	360464	10	207050	358235	151186	584,10	0,03	9,67E-01
ENSRNOG00000003070		N-acetylglucosaminyl-phosphatidylino	192263	10	48560057	48618520	58464	209,32	0,04	9,67E-01
ENSRNOG00000009649	**Slitrk5**	SLIT and NTRK-like family, member 5	306152	15	100299987	100304808	4822	1647,47	-0,04	9,67E-01
ENSRNOG00000012928	**Prmt10**	protein arginine methyltransferase 10	291947	19	44952070	44987000	34931	298,94	0,04	9,67E-01
ENSRNOG00000017500	**Mtss1l**	metastasis suppressor 1-like (Mtss1l),	307845	19	51731405	51745922	14518	5580,41	-0,03	9,67E-01
ENSRNOG00000016628	**Scgn**	secretagogin, EF-hand calcium bindin	306942	17	45214779	45256404	41626	27,99	-0,05	9,67E-01
ENSRNOG00000006336	**Fyco1**	FYVE and coiled-coil domain containir	301085	8	131995143	132061908	66766	256,37	0,03	9,67E-01
ENSRNOG00000029954				MT	5256	5321	66	424,30	0,05	9,67E-01
ENSRNOG00000020623	**Aldh16a1**	aldehyde dehydrogenase 16 family, m	361571	1	102203197	102216266	13070	643,78	-0,04	9,67E-01
ENSRNOG00000014182	**Tns1**	tensin 1 (Tns1), mRNA [Source:RefSe	301509	9	80960143	81166699	206557	771,87	0,04	9,67E-01
ENSRNOG00000049091		La ribonucleoprotein domain family, member 4 [S		X	116149230	116180301	31072	175,84	-0,04	9,67E-01
ENSRNOG00000000043	**Idua**	alpha-L-iduronidase (Idua), mRNA [Sc	360904	14	2037952	2052303	14352	476,16	-0,04	9,67E-01
ENSRNOG00000014833	**LOC10036**	survival motor neuron domain containi	287768	1	281510232	281520160	9929	196,10	-0,03	9,67E-01
ENSRNOG00000043026		Protein Gm4775 [Source:UniProtKB/TrEMBL;Ac		7	2935067	2935501	435	61,81	0,05	9,67E-01
ENSRNOG00000037998	**Snw1**	SNW domain containing 1 (Snw1), mF	500695	6	120764419	120788496	24078	670,11	0,04	9,67E-01
ENSRNOG00000000563	**Adamts14**	ADAM metallopeptidase with thrombo	309837	20	32605126	32679770	74645	26,32	0,05	9,68E-01
ENSRNOG00000001736	**Bdh1**	3-hydroxybutyrate dehydrogenase, typ	117099	11	75653264	75688263	35000	1649,75	-0,04	9,68E-01
ENSRNOG00000004133	**Slc7a3**	solute carrier family 7 (cationic amino	29485	X	71824808	71829659	4852	848,76	0,05	9,68E-01
ENSRNOG00000006800	**Tmem246**	transmembrane protein 246 (Tmem24	362518	5	69342965	69344176	1212	1194,77	0,03	9,68E-01
ENSRNOG00000003559	**Ccdc112**	coiled-coil domain containing 112 (Ccc	498858	18	39874113	39908103	33991	397,10	0,03	9,68E-01
ENSRNOG00000000439	**Ager**	advanced glycosylation end product-s	81722	20	6442508	6445435	2928	34,84	0,04	9,68E-01
ENSRNOG00000002044	**Gtpbp8**	GTP-binding protein 8 (putative) (Gtpb	360714	11	65019537	65029484	9948	248,56	0,03	9,68E-01
ENSRNOG00000011909	**Rasa2**	RAS p21 protein activator 2 (Rasa2), r	25597	8	103860860	103988460	127601	234,81	-0,04	9,68E-01

ENSRNOG00000012156	**Ostf1**	osteoclast stimulating factor 1 (Ostf1),	259275	1	241874541	241895663	21123	411,09	0,05	9,68E-01
ENSRNOG00000017191	**Trim5**	tripartite motif-containing 5 (Trim5), ml	308906	1	175586845	175603800	16956	153,51	0,05	9,68E-01
ENSRNOG00000019458	**Shpk**	sedoheptulokinase (Shpk), mRNA [So	287479	10	59504502	59528848	24347	246,31	0,04	9,68E-01
ENSRNOG00000021143	**LOC10091**	SET domain, bifurcated 1 (Setdb1), m	689883	2	215994816	216025705	30890	1866,60	-0,03	9,68E-01
ENSRNOG00000039183	**RGD13103**	Protein RGD1310335 [Source:UniPro	360711	11	57538666	57565904	27239	84,56	-0,06	9,68E-01
ENSRNOG00000000926	**Mrps17**	mitochondrial ribosomal protein S17 (M	288621	12	32511014	32514191	3178	807,06	-0,03	9,68E-01
ENSRNOG00000015701	**Rreb1**	ras responsive element binding protei	306873	17	29453342	29579484	126143	343,91	-0,04	9,68E-01
ENSRNOG00000025166	**Prps1**	phosphoribosyl pyrophosphate synthe	29562	3	52779466	52801499	22034	2805,41	-0,03	9,68E-01
ENSRNOG00000009947	**Kremen1**	kringle containing transmembrane pro	114107	14	86114543	86175895	61353	100,36	0,04	9,68E-01
ENSRNOG00000042308	**LOC68020**	similar to zinc finger protein 455 (LOC	680200	1	39184216	39200503	16288	27,38	-0,05	9,68E-01
ENSRNOG00000007387	**Per1**	period circadian clock 1 (Per1), mRNA	287422	10	55430184	55439080	8897	974,93	0,03	9,68E-01
ENSRNOG00000010881	**Trak2**	trafficking protein, kinesin binding 2 (T	171086	9	65505455	65570017	64563	3164,11	-0,03	9,68E-01
ENSRNOG00000011439	**Grk5**	G protein-coupled receptor kinase 5 ((	59075	1	289603945	289797677	193733	304,99	0,03	9,68E-01
ENSRNOG00000020989	**Tm7sf2**	transmembrane 7 superfamily membe	293688	1	228361453	228365792	4340	730,54	-0,05	9,68E-01
ENSRNOG00000021437		perlecan (heparan sulfate proteoglycan 2) [Sour		5	159568698	159668805	100108	726,70	-0,04	9,68E-01
ENSRNOG00000026589	**Dpy19l1**	dpy-19-like 1 (C. elegans) (Dpy19l1), r	315496	8	25615337	25700570	85234	1315,94	-0,04	9,68E-01
ENSRNOG00000029785	**Cdc26**	cell division cycle 26 (Cdc26), transcri	366381	5	82373129	82386784	13656	890,95	0,02	9,68E-01
ENSRNOG00000036913	**RGD13096**	Protein RGD1309621 [Source:UniPro	316982	4	248014738	248023483	8746	213,83	-0,05	9,68E-01
ENSRNOG00000047563		mitochondrial ribosomal protein L39 [Source:MG		11	27815367	27830369	15003	321,46	0,03	9,68E-01
ENSRNOG00000008178	**Nxn**	nucleoredoxin (Nxn), mRNA [Source:F	360577	10	63464227	63601539	137313	1170,45	-0,04	9,68E-01
ENSRNOG00000021442	**Hddc2**	HD domain containing 2 (Hddc2), mRI	361462	1	30034693	30054904	20212	206,98	0,03	9,68E-01
ENSRNOG00000000861	**Zic3**	Zic family member 3 (Zic3), mRNA [Sc	367944	X	140921368	140932307	10940	45,90	-0,04	9,68E-01
ENSRNOG00000016344	**Slc39a1**	solute carrier family 39 (zinc transport	361986	2	209042655	209048221	5567	3767,98	0,04	9,68E-01
ENSRNOG00000017453		tyrosine-protein phosphatase non-rece	117063	18	62607293	62671878	64586	306,22	-0,03	9,68E-01
ENSRNOG00000014289	**Arpc2**	actin related protein 2/3 complex, subu	301511	9	81285098	81320273	35176	4321,57	0,03	9,68E-01
ENSRNOG00000019361	**Inpp5j**	inositol polyphosphate-5-phosphatase	171088	14	84418928	84430035	11108	325,88	-0,03	9,68E-01
ENSRNOG00000027296		Uncharacterized protein [Source:UniProtKB/TrE		18	30551074	30553488	2415	291,83	0,03	9,68E-01
ENSRNOG00000039455		Uncharacterized protein [Source:UniProtKB/TrE		18	30585096	30587513	2418	186,40	-0,03	9,68E-01
ENSRNOG00000004959	**Actr2**	ARP2 actin-related protein 2 homolog	289820	14	104074912	104109758	34847	3821,00	-0,03	9,69E-01
ENSRNOG00000007445		Asph protein; Uncharacterized protein [Source:l		5	27476277	27500471	24195	317,14	0,04	9,69E-01
ENSRNOG00000038140	**Whsc1**	Wolf-Hirschhorn syndrome candidate	680537	14	82803450	82855797	52348	2398,82	-0,03	9,69E-01
ENSRNOG00000042163	**LOC10036**	BTB (POZ) domain containing 19 [Sou	1E+08	5	139782809	139787901	5093	122,72	-0,04	9,69E-01
ENSRNOG00000004489	**Cd97**	CD97 molecule (Cd97), mRNA [Sourc	361383	19	35834144	35853867	19724	48,52	0,05	9,69E-01
ENSRNOG00000006469	**Cdk2**	cyclin dependent kinase 2 (Cdk2), mR	362817	7	3097654	3105134	7481	667,49	0,03	9,69E-01
ENSRNOG00000010572	**Pus7**	pseudouridylate synthase 7 homolog (	296751	4	7897731	7938568	40838	497,04	-0,03	9,69E-01
ENSRNOG00000015257	**Coro2b**	Coronin [Source:UniProtKB/TrEMBL;/	300768	8	67107334	67177186	69853	3067,72	-0,03	9,69E-01
ENSRNOG00000018642	**Leng8**	leukocyte receptor cluster (LRC) mem	361506	1	74814349	74826049	11701	1662,78	0,04	9,69E-01
ENSRNOG00000028648	**Olig1**	oligodendrocyte transcription factor 1 (	60394	11	35037423	35039563	2141	9057,00	-0,05	9,69E-01

ENSRNOG00000042740	**Mrpl42**	mitochondrial ribosomal protein L42 (M	299743	7	36638838	36661936	23099	215,91	0,05	9,69E-01
ENSRNOG00000042940				8	60173484	60173947	464	254,82	0,03	9,69E-01
ENSRNOG00000045771		cell adhesion molecule with homology to L1CAM		4	135060114	135120550	60437	3390,10	-0,04	9,69E-01
ENSRNOG00000049713		Uncharacterized protein [Source:UniProtKB/TrE		10	6155044	6156005	962	23,52	-0,05	9,69E-01
ENSRNOG00000018091	**Gdi2**	GDP dissociation inhibitor 2 (Gdi2), ml	29662	17	72003434	72030115	26682	5438,46	-0,03	9,69E-01
ENSRNOG00000004006	**Dnajb9**	DnaJ (Hsp40) homolog, subfamily B, r	24908	6	73754561	73758774	4214	851,40	-0,04	9,69E-01
ENSRNOG00000007843	**Wnt2**	Protein Wnt [Source:UniProtKB/TrEM	114487	4	45896571	45936384	39814	18,07	-0,05	9,69E-01
ENSRNOG00000012123	**Fdx1**	ferredoxin 1 (Fdx1), mRNA [Source:Re	29189	8	54958315	54977222	18908	396,67	-0,04	9,70E-01
ENSRNOG00000022707	**Pmp2**	peripheral myelin protein 2 (Pmp2), ml	688790	2	113582541	113586204	3664	59,64	0,05	9,70E-01
ENSRNOG00000009345	**Ugt8**	UDP glycosyltransferase 8 (Ugt8), mR	50555	2	248998871	249072741	73871	267,51	-0,05	9,70E-01
ENSRNOG00000042123	**RGD15608**	similar to myosin tail domain-containin	502957	5	105651512	105735254	83743	31,75	0,05	9,70E-01
ENSRNOG00000015591	**Cndp2**	CNDP dipeptidase 2 (metallopeptidase	291394	18	80566929	80584026	17098	1480,15	0,02	9,70E-01
ENSRNOG00000018007	**LOC68905**	general transcription factor IIH, polype	502227	1	48280968	48287542	6575	1924,25	0,02	9,70E-01
ENSRNOG00000000302	**Sesn1**	sestrin 1 (Sesn1), mRNA [Source:RefS	294518	20	48347551	48437686	90136	993,22	0,04	9,70E-01
ENSRNOG00000003151	**Bfar**	bifunctional apoptosis regulator (Bfar),	304709	10	361895	394572	32678	1267,65	0,04	9,70E-01
ENSRNOG00000004571	**RGD15636**	Protein RGD1563680 [Source:UniPro	499864	3	113709868	113813616	103749	84,37	-0,03	9,70E-01
ENSRNOG00000008868	**Dusp19**	dual specificity phosphatase 19 (Dusp	311151	3	74406311	74418820	12510	422,73	0,03	9,70E-01
ENSRNOG00000012761	**Ak8**	adenylate kinase 8 (Ak8), mRNA [Sou	311833	3	12630119	12742333	112215	50,13	0,04	9,70E-01
ENSRNOG00000015840	**Hsd17b4**	hydroxysteroid (17-beta) dehydrogena	79244	18	44032347	44119545	87199	1840,36	-0,03	9,70E-01
ENSRNOG00000033119		phospholipase C, beta 4 (Plcb4), mRN	25031	3	135243862	135458277	214416	288,08	-0,04	9,70E-01
ENSRNOG00000018637	**Srcap**	Protein Srcap [Source:UniProtKB/TrE	361652	1	205962862	205981323	18462	343,72	-0,02	9,70E-01
ENSRNOG00000001177	**Acads**	acyl-CoA dehydrogenase, C-2 to C-3 s	64304	12	49049580	49059163	9584	688,63	-0,04	9,70E-01
ENSRNOG00000001184	**Pknox1**	PBX/knotted 1 homeobox 1 (Pknox1),	294322	20	12522385	12544160	21776	371,03	-0,02	9,70E-01
ENSRNOG00000002320	**Commd8**	COMM domain containing 8 (Commd8	289603	14	38422179	38432351	10173	1175,37	0,03	9,70E-01
ENSRNOG00000002977	**Ttc19**	tetratricopeptide repeat domain 19 (Tt	691506	10	48387826	48415743	27918	900,97	0,04	9,70E-01
ENSRNOG00000004677	**Zeb2**	zinc finger E-box binding homeobox 2	311071	3	35063909	35185403	121495	2723,25	-0,02	9,70E-01
ENSRNOG00000004978	**Prkacb**	protein kinase, cAMP dependent, cata	293508	2	271132406	271218985	86580	977,90	0,03	9,70E-01
ENSRNOG00000005965	**Irak4**	interleukin-1 receptor-associated kinas	300177	7	135461296	135487468	26173	129,18	0,04	9,70E-01
ENSRNOG00000015275	**Ska1**	spindle and kinetochore associated cc	291441	18	69317015	69327048	10034	82,37	-0,04	9,70E-01
ENSRNOG00000015718	**RGD13074**	uncharacterized protein LOC300990		300990	8	115451318	115461002	9685	169,46	-0,03	9,70E-01
ENSRNOG00000016937	**Mtfr1l**	mitochondrial fission regulator 1-like (M	298549	5	156522183	156532186	10004	3959,93	0,03	9,70E-01
ENSRNOG00000017945	**Serf1**	Protein Serf1 [Source:UniProtKB/TrEI	502503	2	49506158	49511082	4925	833,88	-0,03	9,70E-01
ENSRNOG00000018411	**Pold3**	polymerase (DNA-directed), delta 3, a	293144	1	171284865	171323242	38378	724,90	0,03	9,70E-01
ENSRNOG00000029939	**Gypc**	glycophorin C (Gerbich blood group) (	364837	18	25028316	25060325	32010	57,30	-0,05	9,70E-01
ENSRNOG00000050051	**Pin4**	Protein LOC684441; RCG36372, isofo	684441	X	72849383	72856079	6697	338,24	0,03	9,70E-01
ENSRNOG00000037366		zinc finger protein 939 [Source:MGI Symbol;Acc		1	96489118	96500809	11692	28,24	0,04	9,70E-01
ENSRNOG00000025076		expressed sequence AI314180 [Source:MGI Syr		5	80076567	80161883	85317	2273,40	-0,04	9,70E-01
ENSRNOG00000047023		aldehyde dehydrogenase 1 family, member L1 [S		4	186990816	187030720	39905	834,54	0,04	9,70E-01

ENSRNOG00000002225	**Scarb2**	scavenger receptor class B, member 2	117106	14	16980270	17031712	51443	5615,40	-0,04	9,70E-01
ENSRNOG00000004027	**Aldh9a1**	aldehyde dehydrogenase 9 family, me	64040	13	90223961	90240182	16222	1155,34	0,03	9,70E-01
ENSRNOG00000005775		PHD finger protein 14 (Phf14), mRNA	500030	4	38078121	38177520	99400	1637,27	-0,04	9,70E-01
ENSRNOG00000007682		glutamate receptor, ionotropic, AMPA 3	29628	X	127656584	127922234	265651	732,93	0,05	9,70E-01
ENSRNOG00000013147		calcium channel, voltage-dependent, L	29716	16	6045673	6339138	293466	997,12	0,04	9,70E-01
ENSRNOG00000014836	**Ralgapb**	Ral GTPase activating protein, beta su	362257	3	160627361	160699898	72538	2113,98	0,02	9,70E-01
ENSRNOG00000015019	**Spg21**	spastic paraplegia 21 homolog (humai	300791	8	70682806	70710180	27375	2400,45	-0,03	9,70E-01
ENSRNOG00000015190		Protein Gabpb1l [Source:UniProtKB/T	364738	17	53581541	53582571	1031	67,53	0,05	9,70E-01
ENSRNOG00000023700	**RGD13118**	similar to 1700030K09Rik protein (RG	290615	16	18944084	18960080	15997	493,86	-0,03	9,70E-01
ENSRNOG00000000547	**Tspyl4**	TSPY-like 4 (Tspyl4), mRNA [Source:F	309828	20	42829886	42831871	1986	5366,39	0,03	9,71E-01
ENSRNOG00000002807		septin-9 isoform 1 [Source:RefSeq pe	83788	10	105893974	105995152	101179	2170,51	0,03	9,71E-01
ENSRNOG00000004650	**Begain**	brain-enriched guanylate kinase-assoc	79146	6	142107881	142129457	21577	1516,49	-0,03	9,71E-01
ENSRNOG00000008980	**Rbm48**	RNA binding motif protein 48 (Rbm48)	296840	4	27601633	27611886	10254	123,27	-0,04	9,71E-01
ENSRNOG00000011859	**Eif5a2**	eukaryotic translation initiation factor 5	310261	2	135035420	135047177	11758	61,26	0,04	9,71E-01
ENSRNOG00000012576	**Vimp**	VCP-interacting membrane protein (Vi	286900	1	128065919	128075694	9776	2319,36	0,03	9,71E-01
ENSRNOG00000013014	**Cyba**	cytochrome b-245, alpha polypeptide (	79129	19	65959818	65967224	7407	283,58	-0,05	9,71E-01
ENSRNOG00000016544	**Arhgef28**	Rho guanine nucleotide exchange fac	361882	2	44892452	45152594	260143	697,26	-0,02	9,71E-01
ENSRNOG00000024854		HAUS augmin-like complex, subunit 6 [Source:M		5	108934526	108969204	34679	60,72	0,05	9,71E-01
ENSRNOG00000047386		SMG1 homolog, phosphatidylinositol 3-kinase-re		1	194739745	194836241	96497	1773,84	0,04	9,71E-01
ENSRNOG00000020721	**Nme6**	NME/NM23 nucleoside diphosphate ki	58964	8	117273929	117280642	6714	230,77	0,04	9,71E-01
ENSRNOG00000016447	**RGD13105**	similar to expressed sequence AI5974	301374	9	49573659	49584318	10660	1091,64	0,04	9,71E-01
ENSRNOG00000008239	**Repin1**	replication initiator 1 (Repin1), mRNA	445541	4	142898415	142900127	1713	774,69	0,03	9,71E-01
ENSRNOG00000047179		amyloid beta (A4) precursor-like protei	64312	8	32323517	32353901	30385	12917,69	-0,04	9,71E-01
ENSRNOG00000019698	**Ssbp4**	single stranded DNA binding protein 4	364534	16	20433840	20444999	11160	2349,69	0,04	9,71E-01
ENSRNOG00000020653	**S1pr2**	sphingosine-1-phosphate receptor 2 (S	29415	8	22041267	22052351	11085	149,78	-0,03	9,71E-01
ENSRNOG00000027317		YLP motif containing 1 [Source:MGI Symbol;Acc		6	116659702	116735987	76286	2668,84	0,02	9,71E-01
ENSRNOG00000013353	**Tmem260**	similar to Protein C14orf101 homolog	361030	15	29220349	29287363	67015	457,23	0,04	9,71E-01
ENSRNOG00000017623	**Paip1**	poly(A) binding protein interacting prot	365684	2	70663730	70691621	27892	2185,55	0,02	9,71E-01
ENSRNOG00000023901				1	248564008	248564407	400	106,64	0,04	9,71E-01
ENSRNOG00000025318	**Scyl3**	SCY1-like 3 (S. cerevisiae) (Scyl3), m	360866	13	87103921	87127294	23374	238,19	-0,03	9,71E-01
ENSRNOG00000038169	**Spin4**	Protein Spin4 [Source:UniProtKB/TrE	299038	X	65121072	65121821	750	34,25	-0,04	9,71E-01
ENSRNOG00000019423	**Dvl1**	dishevelled segment polarity protein 1	83721	5	176771886	176783561	11676	2055,12	0,02	9,71E-01
ENSRNOG00000050767	**Nrn1**	neuritin 1 (Nrn1), mRNA [Source:RefS	83834	17	29834676	29877125	42450	377,81	0,04	9,71E-01
ENSRNOG00000000064	**Atp5i**	ATP synthase, H+ transporting, mitoch	140608	14	2323662	2324790	1129	3830,72	-0,03	9,71E-01
ENSRNOG00000004492	**Mterfd1**	MTERF domain containing 1 (Mterfd1)	299514	7	71447418	71465651	18234	316,25	-0,04	9,71E-01
ENSRNOG00000030920	**Rtn4r**	reticulon 4 receptor (Rtn4r), mRNA [So	113912	11	90085449	90086849	1401	753,62	0,03	9,71E-01
ENSRNOG00000048747	**Cdk5rap3**	CDK5 regulatory subunit associated p	80278	10	84571933	84581074	9142	1398,91	0,03	9,71E-01
ENSRNOG00000000237	**RGD13104**	similar to Protein Njmu-R1 (RGD1310	303764	10	67394162	67406916	12755	884,26	-0,03	9,72E-01

ENSRNOG00000001593	**Lnp**	limb and neural patterns (Lnp), mRNA	362151	3	67901786	67962502	60717	537,60	0,03	9,72E-01
ENSRNOG00000001758	**Tctex1d2**	Tctex1 domain containing 2 (Tctex1d2	498095	11	74678025	74686385	8361	834,41	0,02	9,72E-01
ENSRNOG00000002851	**Fam104a**	family with sequence similarity 104, m	619573	10	101859027	101879775	20749	1142,28	0,02	9,72E-01
ENSRNOG00000010950	**Cep41**	centrosomal protein 41 (Cep41), mRN	500069	4	57728637	57743407	14771	272,35	0,02	9,72E-01
ENSRNOG00000014819	**Hap1**	huntingtin-associated protein 1 (Hap1)	29430	10	88051271	88059466	8196	2744,14	-0,03	9,72E-01
ENSRNOG00000015796	**Msh2**	mutS homolog 2 (E. coli) (Msh2), mRN	81709	6	21198374	21256854	58481	534,12	0,03	9,72E-01
ENSRNOG00000017667	**Adnp2**	ADNP homeobox 2 (Adnp2), mRNA [S	307236	18	72154072	72169317	15246	1086,70	0,02	9,72E-01
ENSRNOG00000017977	**Smpd1**	sphingomyelin phosphodiesterase 1, a	308909	1	177389588	177393430	3843	2101,40	0,02	9,72E-01
ENSRNOG00000018351	**Thap4**	THAP domain containing 4 (Thap4), m	363291	9	100501662	100541256	39595	551,99	0,02	9,72E-01
ENSRNOG00000018904	**Dtymk**	deoxythymidylate kinase (thymidylate	301622	9	100574720	100583554	8835	1098,77	-0,03	9,72E-01
ENSRNOG00000020423	**Apc**	adenomatous polyposis coli (Apc), mR	24205	18	26761212	26818872	57661	12103,99	0,04	9,72E-01
ENSRNOG00000002972	**RGD15652**	similar to novel protein (RGD1565283	498274	13	93270446	93274071	3626	20,06	0,04	9,72E-01
ENSRNOG00000003794	**Nmral1**	NmrA-like family domain containing 1	287063	10	9811579	9820512	8934	284,30	0,03	9,72E-01
ENSRNOG00000003833	**Nenf**	neudesin neurotrophic factor (Nenf), m	289380	13	114555781	114562973	7193	220,14	0,05	9,72E-01
ENSRNOG00000005039	**Tfpi**	tissue factor pathway inhibitor (lipopro	29436	3	78373814	78414596	40783	65,07	-0,04	9,72E-01
ENSRNOG00000007551		InaD-like (Drosophila) [Source:MGI Sy	140581	5	120995203	121279827	284625	123,99	-0,04	9,72E-01
ENSRNOG00000010897		Serine/threonine-protein kinase Nek6	360161	3	28080322	28113347	33026	1672,34	0,03	9,72E-01
ENSRNOG00000012559	**Man1b1**	mannosidase, alpha, class 1B, membe	499751	3	2529399	2550462	21064	4006,15	0,02	9,72E-01
ENSRNOG00000016012	**Spats2l**	spermatogenesis associated, serine-ri	316426	9	64543200	64641802	98603	902,40	-0,02	9,72E-01
ENSRNOG00000016617	**Wwtr1**	WW domain containing transcription re	295062	2	166985078	167101346	116269	282,65	-0,03	9,72E-01
ENSRNOG00000016846	**Pik3cd**	phosphatidylinositol-4,5-bisphosphate	366508	5	170248202	170274177	25976	756,28	-0,02	9,72E-01
ENSRNOG00000017123	**B2m**	beta-2 microglobulin (B2m), mRNA [So	24223	3	120627499	120633521	6023	9500,74	0,04	9,72E-01
ENSRNOG00000019776	**Sh3gl3**	SH3-domain GRB2-like 3 (Sh3gl3), ml	81921	1	145001325	145131589	130265	925,03	0,03	9,72E-01
ENSRNOG00000021402		ankyrin repeat domain 53 [Source:MGI Symbol;A		4	180029358	180035420	6063	261,34	0,04	9,72E-01
ENSRNOG00000021805	**Rft1**	RFT1 homolog (S. cerevisiae) (Rft1), r	290552	16	6645368	6679675	34308	431,59	0,04	9,72E-01
ENSRNOG00000009252	**Poldip2**	polymerase (DNA-directed), delta inte	287544	10	65852787	65861626	8840	2707,13	0,02	9,72E-01
ENSRNOG00000012429	**Ints10**	integrator complex subunit 10 (Ints10)	290679	16	22511881	22542494	30614	1342,14	0,03	9,72E-01
ENSRNOG00000027193	**Ppp1r26**	Protein Ppp1r26 [Source:UniProtKB/T	685398	3	12386168	12389893	3726	1540,48	0,03	9,72E-01
ENSRNOG00000017412		Uncharacterized protein [Source:UniProtKB/TrE		1	51190321	51190872	552	19,19	0,05	9,72E-01
ENSRNOG00000017864		Uncharacterized protein [Source:UniProtKB/TrE		2	49425843	49447799	21957	135,88	0,04	9,72E-01
ENSRNOG00000023296	**Wtip**	Protein Wtip [Source:UniProtKB/TrEM	361552	1	91112725	91137956	25232	269,90	0,04	9,72E-01
ENSRNOG00000017095		Uncharacterized protein [Source:UniProtKB/TrE		1	37265515	37268822	3308	28,75	0,04	9,72E-01
ENSRNOG00000007390	**Nfkbia**	nuclear factor of kappa light polypeptid	25493	6	85803991	85807223	3233	677,02	0,04	9,72E-01
ENSRNOG00000008432	**Slc22a5**	solute carrier family 22 (organic cation	29726	10	38982888	39009871	26984	500,85	0,04	9,72E-01
ENSRNOG00000020067	**Rab40c**	Rab40c, member RAS oncogene fami	359728	10	15060857	15095926	35070	1184,86	-0,03	9,72E-01
ENSRNOG00000026143	**Ckap2l**	Protein Ckap2l [Source:UniProtKB/Tr	680515	3	128193419	128219403	25985	167,59	0,04	9,72E-01
ENSRNOG00000000081		anthrax toxin receptor 2 [Source:MGI Symbol;Ac		14	13133992	13216815	82824	128,81	-0,04	9,72E-01
ENSRNOG00000001335	**Zkscan1**	zinc finger with KRAB and SCAN dom	498160	12	21289593	21305888	16296	578,73	-0,04	9,72E-01

ENSRNOG00000011461	**Ap3m1**	adaptor-related protein complex 3, mu	171126	15	3414671	3432589	17919	1538,49	0,03	9,72E-01
ENSRNOG00000021960	**LOC100910850**			13	98024859	98025799	941	252,34	-0,02	9,72E-01
ENSRNOG00000030319		MORN repeat containing 2 (Morn2), tr	500606	6	2948609	2953694	5086	80,28	-0,03	9,72E-01
ENSRNOG00000001429	**Lrwd1**	uncharacterized protein LOC304396 |	304396	12	25574639	25587606	12968	438,09	0,03	9,72E-01
ENSRNOG00000003075	**Tmem39a**	transmembrane protein 39a (Tmem39	288092	11	67357137	67386470	29334	327,23	-0,03	9,72E-01
ENSRNOG00000003329	**Ppm1d**	protein phosphatase, Mg2+/Mn2+ dep	287585	10	72813803	72849854	36052	778,70	0,03	9,72E-01
ENSRNOG00000014806		paroxysmal nonkinesigenic dyskinesia	1E+08	9	81332621	81400719	68099	3710,83	0,03	9,72E-01
ENSRNOG00000031061	**LOC30607**	similar to RIKEN cDNA 3100001N19 (	306079	15	66175916	66187138	11223	41,11	0,04	9,72E-01
ENSRNOG00000045615	**LOC68828**	threonine aldolase 1 [Source:MGI Syn	688286	10	106516763	106521627	4865	85,12	-0,03	9,72E-01
ENSRNOG00000020450	**Slc26a6**	solute carrier family 26 (anion exchan	301010	8	116993106	117003173	10068	275,07	0,02	9,72E-01
ENSRNOG00000001311	**Rab36**	RAB36, member RAS oncogene famil	690407	20	16740316	16755556	15241	539,59	-0,03	9,72E-01
ENSRNOG00000006418		SAM domain and HD domain, 1 [Sour	311580	3	158468734	158503809	35076	193,72	-0,03	9,72E-01
ENSRNOG00000012053	**S100a16**	S100 calcium binding protein A16 (S1	361991	2	209356215	209361933	5719	4069,14	-0,04	9,72E-01
ENSRNOG00000011204	**Socs7**	Protein Socs7 [Source:UniProtKB/TrE	287659	10	85087048	85118959	31912	909,18	-0,02	9,72E-01
ENSRNOG00000012942	**Satb1**	SATB homeobox 1 (Satb1), mRNA [Sc	316164	9	2150780	2222014	71235	1855,36	0,02	9,72E-01
ENSRNOG00000021881	**Metap2**	methionyl aminopeptidase 2 (Metap2)	64370	7	34821204	34853497	32294	306,64	-0,04	9,72E-01
ENSRNOG00000004305	**Abl2**	c-abl oncogene 2, non-receptor tyrosir	304883	13	79075163	79253783	178621	605,72	0,02	9,72E-01
ENSRNOG00000007699	**Golga5**	golgin A5 (Golga5), mRNA [Source:Re	299258	6	135563809	135589329	25521	345,08	-0,03	9,72E-01
ENSRNOG00000008622	**Creb5**	cAMP responsive element binding pro	500131	4	148056324	148499530	443207	169,55	0,03	9,72E-01
ENSRNOG00000005429	**Foxp2**	forkhead box P2 (Foxp2), mRNA [Sou	500037	4	41461965	41525487	63523	65,78	0,03	9,73E-01
ENSRNOG00000022622	**Slc38a6**	solute carrier family 38, member 6 (Sl	299139	6	105603543	105670544	67002	99,94	0,04	9,73E-01
ENSRNOG00000014775	**Nol4**	nucleolar protein 4 (Nol4), mRNA [Sou	307553	18	13443296	13799530	356235	599,64	0,03	9,73E-01
ENSRNOG00000050151		coiled-coil domain-containing protein 4	287867	10	107952498	108004475	51978	154,22	-0,03	9,73E-01
ENSRNOG00000009988	**LOC10091**	adaptor-related protein complex 2, bet	140670	10	70154033	70255794	101762	1803,72	-0,02	9,73E-01
ENSRNOG00000008831	**Hcn2**	hyperpolarization activated cyclic nucl	114244	7	13021934	13040277	18344	1149,98	-0,02	9,73E-01
ENSRNOG00000013674	**Megf10**	multiple EGF-like domains 10 (Megf10	291445	18	51448631	51551513	102883	2384,54	-0,03	9,73E-01
ENSRNOG00000047902	**LOC10090**	fragile X mental retardation gene 1, au	1E+08	2	140165790	140167400	1611	269,05	0,04	9,73E-01
ENSRNOG00000020883	**Dhdh**	Protein Dhdh [Source:UniProtKB/TrEI	691002	1	102545589	102555779	10191	58,77	-0,04	9,73E-01
ENSRNOG00000015437		transmembrane protein 14C (Tmem14	171432	17	23554045	23560166	6122	1159,80	0,03	9,73E-01
ENSRNOG00000001989	**Alcam**	activated leukocyte cell adhesion mole	79559	11	53964952	54165636	200685	2465,10	-0,02	9,73E-01
ENSRNOG00000002198		Uncharacterized protein [Source:UniProtKB/TrE		14	47906674	47907345	672	123,18	-0,04	9,73E-01
ENSRNOG00000002548	**Tnn**	tenascin N (Tnn), mRNA [Source:RefS	304913	13	82737832	82805226	67395	20,71	-0,04	9,73E-01
ENSRNOG00000013618		ankyrin repeat domain 10 (Ankrd10), r	361183	16	82674223	82697364	23142	1860,94	0,03	9,73E-01
ENSRNOG00000006334	**Txndc16**	Protein Txndc16 [Source:UniProtKB/T	361025	15	23438880	23507088	68209	823,10	0,04	9,73E-01
ENSRNOG00000011945	**Cyfip1**	cytoplasmic FMR1 interacting protein	308666	1	115265170	115353514	88345	2315,55	-0,02	9,73E-01
ENSRNOG00000033624	**Zfp426**	zinc finger protein 426 (Zfp426), mRN	690895	8	21534980	21550452	15473	258,01	-0,03	9,73E-01
ENSRNOG00000019354	**Ptch1**	patched 1 (Ptch1), mRNA [Source:Ref	89830	17	1025796	1079561	53766	880,03	0,02	9,74E-01
ENSRNOG00000033556		similar to Msx2-interacting protein (SP	690911	5	163736425	163790133	53709	3643,15	-0,02	9,74E-01

ENSRNOG00000002948	**Abcc3**	ATP-binding cassette, subfamily C (CF	140668	10	81880042	81938202	58161	19,81	0,05	9,74E-01
ENSRNOG00000004947		zinc finger protein 871 [Source:MGI Symbol;Acc		7	15181626	15184752	3127	243,10	-0,04	9,74E-01
ENSRNOG00000028668	**Slc28a2**	solute carrier family 28 (sodium-couple	60423	3	120897877	120920739	22863	22,41	-0,05	9,74E-01
ENSRNOG00000022502	**Nsrp1**	nuclear speckle splicing regulatory pro	303346	10	62796501	62829730	33230	186,95	0,04	9,74E-01
ENSRNOG00000002144	**Exoc1**	exocyst complex component 1 (Exoc1	305287	14	33923765	33954581	30817	766,26	0,03	9,75E-01
ENSRNOG00000002746		follistatin-like 1 [Source:MGI Symbol;Acc:MGI:1(		11	68876239	68907277	31039	629,08	-0,03	9,75E-01
ENSRNOG00000006717	**Ankrd26**	Protein Ankrd26 [Source:UniProtKB/T	312667	4	216472889	216540555	67667	205,89	-0,04	9,75E-01
ENSRNOG00000009447	**Fam221a**	family with sequence similarity 221, m	500118	4	143710942	143732809	21868	137,55	-0,04	9,75E-01
ENSRNOG00000011713	**LOC69111**	RIKEN cDNA 0610030E20 gene [Sour	691113	4	164980910	164982685	1776	52,78	0,03	9,75E-01
ENSRNOG00000011937	**Sgtb**	small glutamine-rich tetratricopeptide r	294708	2	53314248	53350721	36474	1354,92	-0,03	9,75E-01
ENSRNOG00000011956	**Aktip**	AKT interacting protein (Aktip), mRNA	291906	19	28354290	28364162	9873	2265,18	0,02	9,75E-01
ENSRNOG00000014230	**Map1a**	microtubule-associated protein 1A (Ma	25152	3	119797486	119811427	13942	11652,48	0,02	9,75E-01
ENSRNOG00000017195	**Ctr9**	CTR9, Paf1/RNA polymerase II compl	293184	1	182823942	182853969	30028	3274,76	0,02	9,75E-01
ENSRNOG00000039630	**Wapal**	Protein Wapal [Source:UniProtKB/TrE	290577	16	9357510	9432068	74559	816,96	0,03	9,75E-01
ENSRNOG00000047796	**Pcbd2**	Protein Pcbd2 [Source:UniProtKB/TrE	685729	17	11094484	11145120	50637	339,36	-0,03	9,75E-01
ENSRNOG00000020175	**Rnf121**	ring finger protein 121 (Rnf121), mRN	308871	1	173285052	173347260	62209	634,83	0,02	9,75E-01
ENSRNOG00000009008	**Rab39a**	RAB39A, member RAS oncogene fam	315668	8	56857848	56874859	17012	194,71	0,03	9,75E-01
ENSRNOG00000011168	**Micu2**	mitochondrial calcium uptake 2 (Micu2	171433	15	42032272	42113082	80811	2140,11	0,02	9,75E-01
ENSRNOG00000013516	**Sf3b1**	splicing factor 3b, subunit 1 (Sf3b1), m	84486	9	61275836	61315726	39891	5306,53	0,03	9,75E-01
ENSRNOG00000016212	**Ankfy1**	ankyrin repeat and FYVE domain cont	303292	10	59017118	59065842	48725	2386,43	-0,03	9,75E-01
ENSRNOG00000018773	**Mtrf1l**	mitochondrial translational release fac	361473	1	43805702	43815909	10208	189,12	0,03	9,75E-01
ENSRNOG00000012751	**Tm9sf2**	transmembrane 9 superfamily membe	306197	15	111913765	111967206	53442	4313,37	-0,03	9,75E-01
ENSRNOG00000028266	**Lrrc55**	leucine rich repeat containing 55 (Lrrc	311171	3	79101424	79114130	12707	226,87	-0,03	9,75E-01
ENSRNOG00000010160	**Narg2**	NMDA receptor regulated 2 (Narg2), n	691379	8	79953562	79993266	39705	82,04	0,04	9,76E-01
ENSRNOG00000013252	**Cwc27**	CWC27 spliceosome-associated prote	361887	2	53846102	54048509	202408	184,65	0,03	9,76E-01
ENSRNOG00000042962	**Pdss2**	prenyl (decaprenyl) diphosphate synth	365592	20	49627299	49851499	224201	412,20	0,02	9,76E-01
ENSRNOG00000050624	**Lysmd4**	Protein LOC681647; RCG24927, isofo	681647	1	129266339	129269152	2814	195,14	-0,02	9,76E-01
ENSRNOG00000034287		autism susceptibility candidate 2 [Source:MGI Sy		12	29129143	29129451	309	25,25	0,04	9,76E-01
ENSRNOG00000008851	**Slc35b4**	solute carrier family 35 (UDP-xylose/U	296969	4	61271551	61293559	22009	1967,22	0,03	9,76E-01
ENSRNOG00000045999	**Tnfaip8l1**	Protein Tnfaip8l1 [Source:UniProtKB/	301131	9	9885791	9886351	561	64,55	0,04	9,76E-01
ENSRNOG00000006137	**Arid1a**	AT rich interactive domain 1A (SWI-like	297867	5	155592619	155635649	43031	5084,46	0,02	9,76E-01
ENSRNOG00000009401	**Lmo2**	LIM domain only 2 (Lmo2), transcript v	362176	3	100560598	100572022	11425	127,89	0,04	9,76E-01
ENSRNOG00000013018	**Eda2r**	Protein Eda2r [Source:UniProtKB/TrE	296872	X	67391484	67415596	24113	33,59	0,04	9,76E-01
ENSRNOG00000015461	**Serpine2**	serpin peptidase inhibitor, clade E (ne:	29366	9	85312143	85375551	63409	9190,94	-0,02	9,76E-01
ENSRNOG00000016479	**Plekhg4**	pleckstrin homology domain containin	307796	19	48196258	48202946	6689	18,03	0,04	9,76E-01
ENSRNOG00000018520	**Nsun6**	NOP2/Sun domain family, member 6 (	307148	17	83764477	83802110	37634	223,68	-0,03	9,76E-01
ENSRNOG00000021103	**Nrxn2**	neurexin 2 (Nrxn2), mRNA [Source:Re	116595	1	228789462	228899342	109881	17210,98	0,02	9,76E-01
ENSRNOG00000006738	**Fbxo32**	F-box protein 32 (Fbxo32), mRNA [So	171043	7	98669144	98701551	32408	67,48	-0,04	9,77E-01

ENSRNOG00000019574	**Cuedc2**	CUE domain containing 2 (Cuedc2), m	294009	1	273509049	273517240	8192	416,13	0,03	9,77E-01
ENSRNOG00000027006	**LOC10036**	heterogeneous nuclear ribonucleoprot	362152	10	80795019	80796402	1384	240,54	0,03	9,77E-01
ENSRNOG00000019376	**Zfp329**	zinc finger protein 329 (Zfp329), mRN	308361	1	66592685	66611630	18946	343,88	-0,04	9,77E-01
ENSRNOG00000042320	**Slc41a1**	solute carrier family 41 (magnesium tr	363985	13	53681648	53701895	20248	2076,37	0,02	9,77E-01
ENSRNOG00000005087	**Cebpz**	CCAAT/enhancer binding protein zeta	362686	6	1506913	1524597	17685	622,15	0,04	9,77E-01
ENSRNOG00000006380	**Srsf6**	serine/arginine-rich splicing factor 6 (S	362264	3	165502385	165507708	5324	4721,20	0,03	9,77E-01
ENSRNOG00000015239	**Ginm1**	Protein Ginm1; Similar to cDNA seque	361448	1	3566601	3582540	15940	346,39	-0,03	9,77E-01
ENSRNOG00000019491	**Stard10**	StAR-related lipid transfer (START) dc	293150	1	172622995	172647498	24504	486,55	0,03	9,77E-01
ENSRNOG00000013825		Protein Rap1gap; RAP1, GTPase activating prot		5	159782996	159829864	46869	3440,92	0,03	9,77E-01
ENSRNOG00000049407	**Shisa8**	shisa family member 8 (Shisa8), mRN	315163	7	123423854	123429304	5451	74,63	-0,04	9,77E-01
ENSRNOG00000000566	**Pcbd1**	pterin-4 alpha-carbinolamine dehydrat	29700	20	32402034	32491710	89677	25,74	-0,04	9,77E-01
ENSRNOG00000011025	**Trmt10a**	tRNA methyltransferase 10 homolog A	295496	2	261963880	261977434	13555	300,70	-0,02	9,77E-01
ENSRNOG00000003956	**Arhgap6**	Protein Arhgap6 [Source:UniProtKB/T	1E+08	X	26720670	26781259	60590	21,89	-0,04	9,77E-01
ENSRNOG00000016213	**Ssbp2**	Protein Ssbp2 [Source:UniProtKB/TrE	361877	2	20379437	20574043	194607	3272,55	0,02	9,77E-01
ENSRNOG00000016151	**Ankrd23**	ankyrin repeat domain 23 (Ankrd23), r	316330	9	42764892	42771491	6600	19,08	0,04	9,77E-01
ENSRNOG00000010102	**Fbxl3**	F-box and leucine-rich repeat protein 3	306129	15	97135296	97155065	19770	937,09	-0,02	9,77E-01
ENSRNOG00000019162	**Emc9**	ER membrane protein complex subun	290224	15	38149137	38153351	4215	213,07	-0,03	9,78E-01
ENSRNOG00000038366	**Larp1b**	La ribonucleoprotein domain family, m	310348	2	147446540	147478242	31703	329,40	0,02	9,78E-01
ENSRNOG00000019461	**Zbtb3**	zinc finger and BTB domain containing	499313	1	231936345	231938662	2318	132,92	0,02	9,78E-01
ENSRNOG00000018440	**Arfip2**	ADP-ribosylation factor interacting pro	293344	1	177472247	177476928	4682	2207,59	0,02	9,78E-01
ENSRNOG00000016754	**Bcs1l**	BC1 (ubiquinol-cytochrome c reductas	301514	9	81630647	81634579	3933	473,54	0,02	9,78E-01
ENSRNOG00000018920	**Slc25a17**	solute carrier family 25 (mitochondrial	300083	7	122515166	122557399	42234	844,86	-0,02	9,78E-01
ENSRNOG00000026564	**Ptpdc1**	protein tyrosine phosphatase domain c	291022	17	18389453	18436686	47234	394,37	0,02	9,78E-01
ENSRNOG00000011825	**Ndufb3**	NADH dehydrogenase (ubiquinone) 1	301427	9	65285887	65296093	10207	307,70	-0,02	9,78E-01
ENSRNOG00000020426	**Erf**	Ets2 repressor factor (Erf), mRNA [So	292721	1	83378953	83382939	3987	1630,98	-0,03	9,78E-01
ENSRNOG00000027784	**Tsku**	tsukushi, small leucine rich proteoglyc	308843	1	169524021	169535753	11733	596,94	0,03	9,79E-01
ENSRNOG00000028658	**Olig2**	oligodendrocyte lineage transcription f	304103	11	34998542	35001918	3377	4900,47	-0,04	9,79E-01
ENSRNOG00000029570	**Fam222b**	family with sequence similarity 222, m	497960	10	66217748	66283043	65296	1170,86	0,02	9,79E-01
ENSRNOG00000014478	**Fndc3a**	fibronectin type III domain containing 3	306022	15	58102193	58290602	188410	1824,37	0,02	9,79E-01
ENSRNOG00000016343	**Dkk3**	dickkopf WNT signaling pathway inhib	171548	1	183922626	183961795	39170	975,24	-0,03	9,79E-01
ENSRNOG00000020238	**Plekhh3**	pleckstrin homology domain containing	360634	10	88874968	88883381	8414	519,00	0,03	9,79E-01
ENSRNOG00000007029	**Dnaja1**	DnaJ (Hsp40) homolog, subfamily A, r	65028	5	61562288	61573199	10912	5053,95	0,03	9,79E-01
ENSRNOG00000004927	**Supt7l**	suppressor of Ty 7 (S. cerevisiae)-like	313905	6	36065222	36075359	10138	523,31	0,02	9,79E-01
ENSRNOG00000008441	**Map3k3**	mitogen activated protein kinase kinas	303604	10	94029306	94098038	68733	537,97	-0,03	9,79E-01
ENSRNOG00000010358	**Sh3rf1**	SH3 domain containing ring finger 1 (S	306417	16	31900567	32060681	160115	1059,61	0,02	9,79E-01
ENSRNOG00000017690	**Ccsap**	centriole, cilia and spindle-associated	307926	19	67325675	67343393	17719	964,97	0,02	9,79E-01
ENSRNOG00000001126	**Fbxw8**	F-box and WD repeat domain containi	304522	12	45933952	45966317	32366	812,29	-0,02	9,79E-01
ENSRNOG00000016974	**Ppp1r7**	protein phosphatase 1, regulatory sub	301618	9	100146206	100160432	14227	1525,15	0,02	9,79E-01

ENSRNOG00000017932	**St3gal2**	ST3 beta-galactoside alpha-2,3-sialylt	64442	19	54102746	54118616	15871	1664,29	0,02	9,79E-01
ENSRNOG00000019009	**Arrdc2**	arrestin domain containing 2 (Arrdc2),	306344	16	20209578	20213710	4133	203,44	0,03	9,79E-01
ENSRNOG00000017151		ring finger protein 214 [Source:MGI Sy	363056	8	48792588	48822370	29783	933,66	-0,02	9,79E-01
ENSRNOG00000039848		TAF9 RNA polymerase II, TATA box bi	373541	2	49824043	49836090	12048	450,63	0,02	9,79E-01
ENSRNOG00000021120	**Prune**	prune exopolyphosphatase (Prune), m	310664	2	215925757	215953662	27906	1106,84	0,02	9,79E-01
ENSRNOG00000014002	**St7l**	suppression of tumorigenicity 7-like (S	295344	2	226767139	226835168	68030	464,44	0,02	9,79E-01
ENSRNOG00000024145		tripartite motif-containing protein 65 [S	303684	10	103604566	103610661	6096	204,17	-0,02	9,79E-01
ENSRNOG00000006340	**Memo1**	mediator of cell motility 1 (Memo1), ml	298787	6	32248023	32295274	47252	1117,46	-0,02	9,79E-01
ENSRNOG00000002764		methyltransferase like 16 [Source:MGI Symbol;A		10	61348499	61393511	45013	382,66	0,02	9,79E-01
ENSRNOG00000003728	**Xylt2**	xylosyltransferase II (Xylt2), mRNA [So	64134	10	82204622	82218102	13481	818,62	0,02	9,79E-01
ENSRNOG00000007283	**Erlec1**	endoplasmic reticulum lectin 1 (Erlec1	289874	14	114972393	115009184	36792	1234,22	-0,02	9,79E-01
ENSRNOG00000008736	**Slamf8**	SLAM family member 8 (Slamf8), mRN	289237	13	95487465	95496444	8980	30,56	0,04	9,79E-01
ENSRNOG00000039076	**LOC68845**	RIKEN cDNA 4930546H06 gene [Sou	688452	1	60624656	60635421	10766	48,41	-0,03	9,79E-01
ENSRNOG00000012505	**Ppwd1**	peptidylprolyl isomerase domain and V	294711	2	53418985	53442093	23109	138,38	-0,03	9,79E-01
ENSRNOG00000009020		tissue specific transplantation antigen	300036	7	116851238	116856095	4858	1469,94	0,03	9,80E-01
ENSRNOG00000015137	**RGD13100**	Spermatogenesis associated 6-like pro	361747	1	254286410	254327754	41345	41,34	-0,03	9,80E-01
ENSRNOG00000035620	**Mir22**	rno-mir-22 [Source:miRBase;Acc:MI0000851]		10	62013642	62013736	95	62,25	-0,03	9,80E-01
ENSRNOG00000037886				15	107871179	107872025	847	123,16	0,02	9,80E-01
ENSRNOG00000049819	**RGD15602**	similar to homolog of yeast TIM14 isof	502525	2	140149972	140174575	24604	714,46	-0,02	9,80E-01
ENSRNOG00000010752	**Tmem41b**	transmembrane protein 41B (Tmem41	361626	1	181591182	181605545	14364	1179,59	-0,03	9,80E-01
ENSRNOG00000019444		autocrine motility factor receptor [Sour	361367	19	11447974	11482482	34509	5277,19	-0,02	9,80E-01
ENSRNOG00000017726	**Vom1r90**	vomeronasal 1 receptor 90 (Vom1r90)	266771	4	184297337	184306351	9015	66,79	0,03	9,80E-01
ENSRNOG00000018378	**Cacnb2**	calcium channel, voltage-dependent, b	116600	17	83540713	83761206	220494	251,79	0,03	9,80E-01
ENSRNOG00000019310	**Eid2**	Protein Eid2 [Source:UniProtKB/TrEM	685691	1	86727767	86728474	708	537,33	0,02	9,80E-01
ENSRNOG00000020599	**Tssk6**	testis-specific serine kinase 6 (Tssk6),	290670	16	21189471	21190772	1302	73,03	0,03	9,80E-01
ENSRNOG00000032398	**RGD15621**	similar to D1Ertd622e protein (RGD15	501195	9	110878815	110898589	19775	2054,87	0,02	9,80E-01
ENSRNOG00000043192	**Ptpla**	protein tyrosine phosphatase-like (pro	680115	17	82918314	82932322	14009	169,01	-0,02	9,80E-01
ENSRNOG00000003625	**Ubxn4**	UBX domain protein 4 (Ubxn4), mRNA	304766	13	50042178	50074671	32494	3695,97	0,02	9,80E-01
ENSRNOG00000013741		ubiquitin-conjugating enzyme E2 D3 [	81920	2	259159895	259175013	15119	4827,58	0,02	9,80E-01
ENSRNOG00000022741	**Ilf3**	interleukin enhancer binding factor 3 (	84472	8	22478044	22494771	16728	3234,78	0,02	9,80E-01
ENSRNOG00000005533	**LOC68601**	RIKEN cDNA 1700067K01 gene [Sou	686013	19	36100966	36103834	2869	29,60	-0,03	9,80E-01
ENSRNOG00000012695	**Golt1b**	golgi transport 1B (Golt1b), mRNA [So	362460	4	240820452	240831667	11216	579,20	-0,03	9,80E-01
ENSRNOG00000002791	**Bcl2**	B-cell CLL/lymphoma 2 (Bcl2), mRNA	24224	13	31758213	31919850	161638	94,26	0,02	9,80E-01
ENSRNOG00000016150	**Ifrd2**	interferon-related developmental regul	300994	8	115699356	115704574	5219	219,35	-0,02	9,80E-01
ENSRNOG00000030225	**Clpx**	ClpX caseinolytic peptidase X homolo	300786	8	70480810	70535360	54551	562,01	0,02	9,80E-01
ENSRNOG00000015295	**Ctdsp1**	CTD (carboxy-terminal domain, RNA p	363249	9	81313441	81442524	129084	1571,31	-0,03	9,80E-01
ENSRNOG00000016255	**Podxl2**	podocalyxin-like 2 (Podxl2), mRNA [So	297433	4	186026104	186057984	31881	5751,88	-0,02	9,80E-01
ENSRNOG00000014434		chromodomain-helicase-DNA-binding	308215	1	58619223	58682200	62978	772,74	0,02	9,81E-01

ENSRNOG00000016505	**Fam169a**	Protein Fam169a [Source:UniProtKB/	310013	2	47016424	47049414	32991	500,72	-0,02	9,81E-01
ENSRNOG00000008666	**Etl4**	Protein Etl4 [Source:UniProtKB/TrEM	291356	17	88907370	89370265	462896	267,89	-0,02	9,81E-01
ENSRNOG00000025430	**Ddx18**	DEAD (Asp-Glu-Ala-Asp) box polypep	308490	13	42680397	42693902	13506	496,04	0,02	9,81E-01
ENSRNOG00000000587	**Rpf2**	ribosome production factor 2 homolog	294436	20	47041320	47062618	21299	347,51	0,04	9,81E-01
ENSRNOG00000008410		centrosomal protein 63 (Cep63), trans	300963	8	110462282	110513824	51543	213,03	0,02	9,81E-01
ENSRNOG00000012730	**Lrrk1**	leucine-rich repeat kinase 1 (Lrrk1), m	308703	1	128248473	128372456	123984	79,08	0,03	9,81E-01
ENSRNOG00000022189	**Htatip2**	HIV-1 Tat interactive protein 2 (Htatip2	292935	1	106145091	106159700	14610	31,53	0,03	9,81E-01
ENSRNOG00000023972		Protein Col4a2 [Source:UniProtKB/TrEMBL;Acc		16	82853206	82987193	133988	1825,29	0,02	9,81E-01
ENSRNOG00000007440		Protein RGD1311344 [Source:UniPro	311486	3	145054603	145112674	58072	396,34	-0,02	9,81E-01
ENSRNOG00000029360	**Serinc1**	serine incorporator 1 (Serinc1), mRNA	294421	20	40703831	40722882	19052	11098,53	-0,02	9,81E-01
ENSRNOG00000001297	**Prmt2**	protein arginine methyltransferase 2 (I	499420	20	15309078	15366940	57863	2475,37	0,02	9,81E-01
ENSRNOG00000045998	**Sema6b**	sema domain, transmembrane domair	84609	9	9928560	9938199	9640	2936,21	-0,02	9,81E-01
ENSRNOG00000010655	**Mttp**	microsomal triglyceride transfer protei	310900	2	261907825	261948693	40869	88,06	0,03	9,81E-01
ENSRNOG00000004716		serologically defined colon cancer anti	1E+08	6	101068662	101132662	64001	1607,85	-0,02	9,81E-01
ENSRNOG00000006719		zinc finger protein 618 [Source:MGI S	313253	5	83013558	83084797	71240	206,12	-0,02	9,81E-01
ENSRNOG00000008797	**Tmpo**	thymopoietin (Tmpo), mRNA [Source:I	25359	7	31934412	31952695	18284	1745,15	0,02	9,81E-01
ENSRNOG00000011675	**Zfp40**	zinc finger protein 40 (Zfp40), mRNA [	690661	1	65342107	65361955	19849	152,71	-0,03	9,81E-01
ENSRNOG00000040384	**Mir770**	rno-mir-770 [Source:miRBase;Acc:MI0006165]		6	142845041	142845134	94	55,26	-0,04	9,81E-01
ENSRNOG00000049814	**LOC10091(**	RNA binding motif protein 39 (Rbm39)	362251	3	159431614	159464754	33141	47,66	-0,03	9,81E-01
ENSRNOG00000050727	**LOC100361083**			5	144887273	144890597	3325	390,00	0,03	9,81E-01
ENSRNOG00000000901	**Crcp**	CGRP receptor component (Crcp), mF	114205	12	32061700	32088178	26479	446,31	0,02	9,82E-01
ENSRNOG00000001030		TSC22 domain family, member 1 (Tsc2	498545	15	62249980	62348598	98619	13047,96	-0,02	9,82E-01
ENSRNOG00000003033	**Plcd3**	phospholipase C, delta 3 (Plcd3), mRI	287745	10	90937143	90957990	20848	136,08	0,03	9,82E-01
ENSRNOG00000003508	**Ergic1**	Protein Ergic1 [Source:UniProtKB/TrE	287177	10	16719603	16763481	43879	1006,67	0,02	9,82E-01
ENSRNOG00000004706	**Vit**	Protein Vit [Source:UniProtKB/TrEMB	313831	6	1127877	1199126	71250	21,39	-0,03	9,82E-01
ENSRNOG00000005227	**Tfap4**	transcription factor AP-4 (Tfap4), mRN	360482	10	9969937	9986048	16112	256,67	0,02	9,82E-01
ENSRNOG00000006462		ERO1-like protein alpha precursor [S	171562	15	23583076	23620342	37267	1189,83	0,03	9,82E-01
ENSRNOG00000006681	**Rdh10**	retinol dehydrogenase 10 (all-trans) (F	353252	5	2593904	2620243	26340	205,65	0,02	9,82E-01
ENSRNOG00000006916	**Sardh**	sarcosine dehydrogenase (Sardh), mF	114123	3	11101704	11162650	60947	350,92	-0,03	9,82E-01
ENSRNOG00000007882	**Ablim2**	actin binding LIM protein family, memb	360958	14	79676533	79799069	122537	464,77	0,02	9,82E-01
ENSRNOG00000008593	**Ryk**	receptor-like tyrosine kinase (Ryk), mF	140585	8	110718666	110790096	71431	1302,65	-0,02	9,82E-01
ENSRNOG00000010058	**Spry2**	sprouty homolog 2 (Drosophila) (Spry2	306141	15	93662947	93665780	2834	2245,78	0,02	9,82E-01
ENSRNOG00000010326	**Col20a1**	Protein Col20a1 [Source:UniProtKB/T	311716	3	180204604	180234584	29981	37,74	0,03	9,82E-01
ENSRNOG00000014199	**Zfp703**	zinc finger protein 703 (Zfp703), mRN	680717	16	68911230	68915401	4172	1183,01	-0,02	9,82E-01
ENSRNOG00000014322		Kazrin [Source:UniProtKB/Swiss-Prot	313672	5	164350465	164739758	389294	824,88	-0,02	9,82E-01
ENSRNOG00000014405	**Aprt**	adenine phosphoribosyl transferase (A	292072	19	66095570	66097799	2230	1757,98	-0,03	9,82E-01
ENSRNOG00000015145	**Ppp6c**	protein phosphatase 6, catalytic subur	171121	3	28702128	28732821	30694	1109,48	-0,02	9,82E-01
ENSRNOG00000015780	**Rcn2**	reticulocalbin 2, EF-hand calcium bind	29218	8	59280470	59297530	17061	4555,60	-0,02	9,82E-01

ENSRNOG00000016058	**Kazald1**	Kazal-type serine peptidase inhibitor d	293997	1	272270128	272273540	3413	35,71	0,03	9,82E-01
ENSRNOG00000017085	**Tubgcp3**	tubulin, gamma complex associated p	306599	16	81670485	81729860	59376	1090,55	-0,02	9,82E-01
ENSRNOG00000017106		casein kinase 1, alpha 1 (Csnk1a1), m	113927	18	56116409	56147273	30865	7072,69	0,02	9,82E-01
ENSRNOG00000018092	**Cd83**	CD83 molecule (Cd83), mRNA [Sourc	361226	17	26364944	26384002	19059	104,71	-0,03	9,82E-01
ENSRNOG00000018371	**Tubb6**	tubulin, beta 6 class V (Tubb6), mRNA	307351	18	62314821	62324460	9640	1256,30	-0,03	9,82E-01
ENSRNOG00000018485	**Gpr155**	G protein-coupled receptor 155 (Gpr1	311730	3	66535459	66588943	53485	644,25	-0,02	9,82E-01
ENSRNOG00000019047	**Ola1**	Obg-like ATPase 1 (Ola1), mRNA [Sou	296488	3	66275428	66395046	119619	2019,59	0,02	9,82E-01
ENSRNOG00000020342		Protein Samd11; RCG31097, isoform CRA_a [S		5	177146850	177154330	7481	125,19	-0,03	9,82E-01
ENSRNOG00000021067	**Fxyd7**	FXYD domain-containing ion transport	63848	1	90630083	90639081	8999	196,91	-0,03	9,82E-01
ENSRNOG00000021234	**Slc4a11**	solute carrier family 4, sodium borate t	311423	3	129722370	129725935	3566	25,01	-0,03	9,82E-01
ENSRNOG00000022837	**Slc25a40**	solute carrier family 25, member 40 (S	296813	4	22508236	22593670	85435	65,15	-0,03	9,82E-01
ENSRNOG00000023013	**March3**	membrane-associated ring finger (C3H	364878	18	51040248	51192562	152315	449,71	-0,02	9,82E-01
ENSRNOG00000026579		predicted gene 960 [Source:MGI Symbol;Acc:M		1	226730593	226778314	47722	25,90	-0,03	9,82E-01
ENSRNOG00000029434		Uncharacterized protein [Source:UniProtKB/TrE		5	114681712	114683652	1941	45,65	-0,03	9,82E-01
ENSRNOG00000032238	**Plcd1**	phospholipase C, delta 1 (Plcd1), mRN	24655	8	126970655	126991422	20768	621,36	0,03	9,82E-01
ENSRNOG00000033517	**LOC10036**	Protein LOC100360791 [Source:UniP	1E+08	1	21603257	21604069	813	1097,82	0,02	9,82E-01
ENSRNOG00000039459		Uncharacterized protein [Source:UniProtKB/TrE		18	30563855	30566233	2379	228,26	0,02	9,82E-01
ENSRNOG00000042309	**RGD13100**	uncharacterized LOC292199 (Rgd131	292199	1	32469423	32484734	15312	3764,08	0,02	9,82E-01
ENSRNOG00000042519	**RGD13120**	similar to RIKEN cDNA C230081A13 (	315686	8	59437967	59649674	211708	729,10	0,02	9,82E-01
ENSRNOG00000042602		Uncharacterized protein [Source:UniProtKB/TrE		2	109268083	109268647	565	81,17	0,03	9,82E-01
ENSRNOG00000043498		salt inducible kinase 2 [Source:MGI Symbol;Acc		8	53905730	54005174	99445	2323,66	-0,02	9,82E-01
ENSRNOG00000045668		interferon (alpha and beta) receptor 2	686326	11	35148029	35167632	19604	343,31	-0,02	9,82E-01
ENSRNOG00000046981	**Gp1bb**	glycoprotein Ib (platelet), beta polypep	116727	11	89621049	89622160	1112	593,94	0,03	9,82E-01
ENSRNOG00000005153		RIKEN cDNA 4930427A07 gene [Sou	690422	6	146963710	146970406	6697	400,05	0,03	9,82E-01
ENSRNOG00000020850	**Mpp2**	membrane protein, palmitoylated 2 (M	85275	10	89789505	89819927	30423	995,67	0,02	9,82E-01
ENSRNOG00000001629	**Wrb**	tryptophan rich basic protein (Wrb), m	288233	11	40028142	40042073	13932	3609,13	0,02	9,82E-01
ENSRNOG00000046660				15	106497423	106607464	110042	120,36	-0,02	9,82E-01
ENSRNOG00000049856	**Zfp961**	Protein Zfp961 [Source:UniProtKB/Tr	1E+08	16	19398387	19417115	18729	50,88	-0,03	9,82E-01
ENSRNOG00000016585	**Bmi1**	Bmi1 polycomb ring finger oncogene (	307151	17	87087687	87091415	3729	861,26	0,02	9,82E-01
ENSRNOG00000020488	**Luc7l**	LUC7-like (S. cerevisiae) (Luc7l), mRN	360503	10	15435862	15462627	26766	1124,44	0,02	9,82E-01
ENSRNOG00000000389	**Dna2**	DNA replication ATP-dependent helica	309762	20	29094517	29122979	28463	133,04	-0,03	9,82E-01
ENSRNOG00000003099	**Ubqln2**	ubiquilin 2 (Ubqln2), mRNA [Source:R	317396	X	19487982	19491373	3392	6871,41	0,01	9,82E-01
ENSRNOG00000019273	**Capn7**	calpain 7 (Capn7), mRNA [Source:Ref	306260	16	7371493	7407436	35944	1022,41	0,02	9,82E-01
ENSRNOG00000027747	**Ninl**	Protein Ninl [Source:UniProtKB/TrEM	311529	3	153080769	153124873	44105	281,82	-0,02	9,82E-01
ENSRNOG00000033887	**Acot3**	acyl-CoA thioesterase 3 (Acot3), mRN	314304	6	118006796	118012735	5940	446,48	-0,02	9,82E-01
ENSRNOG00000037091		hypothetical protein, MNCb-2457 [Source:MGI S		5	160559204	160559988	785	24,22	-0,03	9,82E-01
ENSRNOG00000039454	**Pcdhgb7**	protocadherin gamma subfamily B, 7 (	364844	18	30594347	30596938	2592	380,29	-0,03	9,82E-01
ENSRNOG00000047258	**Prss55**	Protein LOC683415 [Source:UniProtK	683415	15	51192538	51200853	8316	30,65	0,03	9,82E-01

ENSRNOG00000048660	**Gsg2**	Protein LOC687827; RCG34364 [Sou	687827	10	59435380	59437635	2256	183,32	0,02	9,82E-01
ENSRNOG00000001404	**Agfg2**	ArfGAP with FG repeats 2 (Agfg2), mF	304375	12	24099333	24135596	36264	595,03	0,02	9,83E-01
ENSRNOG00000005879	**Maml2**	Protein Maml2 [Source:UniProtKB/TrE	689844	8	12115651	12201338	85688	583,36	-0,02	9,83E-01
ENSRNOG00000012771	**LOC68880**	DEAD (Asp-Glu-Ala-Asp) box polypep	314336	17	11740661	11746052	5392	633,03	0,02	9,83E-01
ENSRNOG00000016152	**Dek**	DEK oncogene (Dek), mRNA [Source:	306817	17	19948514	19970468	21955	1218,85	-0,03	9,83E-01
ENSRNOG00000018309	**Chd4**	Protein Chd4 [Source:UniProtKB/TrEI	117535	4	224630792	224662765	31974	7510,67	-0,01	9,83E-01
ENSRNOG00000021903	**Atad5**	Protein Atad5 [Source:UniProtKB/TrE	303348	10	67086230	67131964	45735	281,50	-0,03	9,83E-01
ENSRNOG00000037441	**Ankle2**	ankyrin repeat and LEM domain conta	360829	12	54212226	54240448	28223	614,69	-0,02	9,83E-01
ENSRNOG00000001478	**Gtf2ird1**	GTF2I repeat domain containing 1 (Gt	246770	12	27273682	27380402	106721	74,71	-0,02	9,83E-01
ENSRNOG00000001681	**Dscr3**	Down syndrome critical region gene 3	360703	11	38355950	38383907	27958	1082,24	0,02	9,83E-01
ENSRNOG00000002238	**Sema5b**	sema domain, seven thrombospondin	303901	11	71394318	71405876	11559	759,36	0,02	9,83E-01
ENSRNOG00000005036	**Atad2b**	ATPase family, AAA domain containing	500625	6	40825203	40889050	63848	139,30	0,03	9,83E-01
ENSRNOG00000005052	**Slc39a9**	solute carrier family 39, member 9 (Sl	314275	6	112463008	112498488	35481	864,68	0,02	9,83E-01
ENSRNOG00000006003	**Ctnna2**	catenin (cadherin associated protein),	297357	4	172588005	173519137	931133	2223,92	0,02	9,83E-01
ENSRNOG00000006131	**Mettl2b**	methyltransferase like 2B (Mettl2b), m	363687	10	93112426	93126251	13826	360,73	0,02	9,83E-01
ENSRNOG00000006532	**H3f3b**	H3 histone, family 3B (H3f3b), mRNA	117056	10	103709482	103711708	2227	11483,04	0,02	9,83E-01
ENSRNOG00000008504	**Naa38**	N(alpha)-acetyltransferase 38, NatC a	296913	4	43016688	43022554	5867	1066,31	-0,02	9,83E-01
ENSRNOG00000008517	**Cdc42ep1**	CDC42 effector protein (Rho GTPase	315121	7	120058685	120062286	3602	191,90	0,03	9,83E-01
ENSRNOG00000010090	**Ccdc174**	coiled-coil domain containing 174 (Cc	297458	4	189474031	189501549	27519	304,78	-0,02	9,83E-01
ENSRNOG00000013634	**Myd88**	myeloid differentiation primary respons	301059	8	127229273	127233342	4070	249,39	0,03	9,83E-01
ENSRNOG00000015858	**Hyal1**	hyaluronoglucosaminidase 1 (Hyal1),	367166	8	115689184	115691497	2314	161,63	0,03	9,83E-01
ENSRNOG00000016640	**Dner**	Protein Dner [Source:UniProtKB/TrEM	316573	9	91700913	91800040	99128	4815,94	-0,02	9,83E-01
ENSRNOG00000017333	**Syt4**	synaptotagmin IV (Syt4), mRNA [Sour	64440	18	23893019	23902428	9410	9154,16	-0,02	9,83E-01
ENSRNOG00000017561	**Toe1**	target of EGR1, member 1 (nuclear) (	298443	5	139456024	139459589	3566	301,22	0,03	9,83E-01
ENSRNOG00000017729	**Psmg2**	proteasome (prosome, macropain) as	291539	18	62579926	62594539	14614	389,77	-0,02	9,83E-01
ENSRNOG00000017915	**Dvl2**	dishevelled 2 (Dvl2), mRNA [Source:R	303251	10	56355170	56364388	9219	792,49	0,01	9,83E-01
ENSRNOG00000018448	**LOC10091**	cell cycle exit and neuronal differentiat	361675	1	220887403	220890392	2990	51,45	0,03	9,83E-01
ENSRNOG00000020640	**Zbtb7b**	zinc finger and BTB domain containing	295248	2	208119628	208121619	1992	200,84	-0,03	9,83E-01
ENSRNOG00000020880	**Klhl18**	Protein Klhl18 [Source:UniProtKB/TrE	316012	8	118015204	118079834	64631	885,41	0,02	9,83E-01
ENSRNOG00000028394	**Vpreb3**	pre-B lymphocyte 3 (Vpreb3), mRNA [	365550	20	15812246	15813556	1311	25,38	-0,03	9,83E-01
ENSRNOG00000037253	**Fam3a**	family with sequence similarity 3, mem	501664	1	152059824	152068446	8623	719,63	0,02	9,83E-01
ENSRNOG00000037432	**Exo5**	exonuclease 5 (Exo5), mRNA [Source	313563	5	143643134	143646451	3318	165,94	0,02	9,83E-01
ENSRNOG00000038403	**Ptges3l1**	prostaglandin E synthase 3-like 1 (Ptg	367808	X	45636274	45645196	8923	70,34	-0,02	9,83E-01
ENSRNOG00000042258	**RGD15611**	RGD1561157 (RGD1561157), mRNA	360487	10	12903766	12904690	925	23,63	-0,03	9,83E-01
ENSRNOG00000045891	**LOC69184**	predicted gene 5918 [Source:MGI Syr	691849	8	73429543	73434009	4467	190,38	-0,03	9,83E-01
ENSRNOG00000047235	**Vps26b**	vacuolar protein sorting 26 homolog B	300472	8	28074137	28095244	21108	4572,62	-0,02	9,83E-01
ENSRNOG00000047867	**Klhdc8b**	kelch domain containing 8B (Klhdc8b)	306589	8	116576640	116581366	4727	1144,19	-0,02	9,83E-01
ENSRNOG00000050932	**Pfdn4**	Protein LOC100366237; RCG32197, i	1E+08	3	174227574	174235341	7768	1093,96	-0,02	9,83E-01

ENSRNOG00000013751	**Prosc**	proline synthetase co-transcribed hom	306544	16	68838027	68849618	11592	618,91	-0,02	9,83E-01
ENSRNOG00000022652	**LOC10036**	proteasome subunit alpha type 3-like (	1E+08	10	65807627	65808523	897	24,87	-0,03	9,83E-01
ENSRNOG00000030840	**Cadm2**	cell adhesion molecule 2 (Cadm2), mF	360687	11	7871257	8090083	218827	834,43	0,02	9,83E-01
ENSRNOG00000004757	**Tmem158**	transmembrane protein 158 (Tmem15	117582	8	131497635	131498492	858	533,26	0,02	9,83E-01
ENSRNOG00000028523	**Tctn1**	tectonic family member 1 (Tctn1), mRI	304486	12	41671666	41704377	32712	726,07	-0,02	9,83E-01
ENSRNOG00000009267	**B3gnt2**	UDP-GlcNAc:betaGal beta-1,3-N-acet	305571	14	107652782	107677809	25028	159,56	-0,02	9,83E-01
ENSRNOG00000025423	**Map3k13**	mitogen-activated protein kinase kinas	303823	11	85830850	86018056	187207	377,36	0,02	9,83E-01
ENSRNOG00000000502	**Def6**	differentially expressed in FDCP 6 hor	309642	20	9988008	10009186	21179	35,15	0,03	9,83E-01
ENSRNOG00000000858	**Sapcd1**	suppressor APC domain containing 1 (	406170	20	7091299	7093067	1769	45,78	-0,03	9,83E-01
ENSRNOG00000001325	**Il3ra**	interleukin 3 receptor, alpha (Il3ra), mF	246144	12	20519721	20525025	5305	596,97	0,02	9,83E-01
ENSRNOG00000001769	**Tnk2**	tyrosine kinase, non-receptor, 2 (Tnk2	303882	11	74431231	74470437	39207	3208,90	0,02	9,83E-01
ENSRNOG00000002941	**RGD15618**	U2AF homology motif (UHM) kinase 1	246332	13	93147551	93162493	14943	524,42	-0,02	9,83E-01
ENSRNOG00000003315	**Cops3**	COP9 signalosome subunit 3 (Cops3)	287367	10	45938611	45962012	23402	1996,72	-0,01	9,83E-01
ENSRNOG00000005285	**Fbxo33**	F-box protein 33 (Fbxo33), mRNA [So	314157	6	89828089	89860308	32220	425,15	0,02	9,83E-01
ENSRNOG00000005884	**RGD13050**	UPF0480 protein C15orf24 homolog [	296050	3	110539195	110549807	10613	1673,81	0,02	9,83E-01
ENSRNOG00000006350	**RGD15615**	Protein RGD1561530 [Source:UniPro	299637	7	11133941	11155091	21151	98,12	-0,03	9,83E-01
ENSRNOG00000007691	**Osbpl2**	oxysterol binding protein-like 2 (Osbpl	296461	3	181579720	181624984	45265	2048,28	0,02	9,83E-01
ENSRNOG00000007937	**Krit1**	KRIT1, ankyrin repeat containing (Krit	362317	4	27341835	27376376	34542	562,67	-0,02	9,83E-01
ENSRNOG00000008463	**Rundc3b**	RUN domain containing 3B (Rundc3b)	688590	4	22380551	22518349	137799	1365,06	0,02	9,83E-01
ENSRNOG00000010369	**Dhx29**	Protein Dhx29 [Source:UniProtKB/TrE	1E+08	2	63827375	63877478	50104	772,63	0,02	9,83E-01
ENSRNOG00000011800	**F3**	coagulation factor III (thromboplastin,	25584	2	243348430	243360438	12009	854,48	-0,02	9,83E-01
ENSRNOG00000012273	**Dmtn**	dematin actin binding protein (Dmtn), i	361069	15	56016282	56043902	27621	2020,97	-0,02	9,83E-01
ENSRNOG00000012663	**Mfsd6**	major facilitator superfamily domain cc	301388	9	53294987	53378134	83148	1285,88	-0,02	9,83E-01
ENSRNOG00000015080	**Wdfy1**	WD repeat and FYVE domain containi	301549	9	85231523	85280641	49119	1058,71	-0,02	9,83E-01
ENSRNOG00000015268	**Ppapdc2**	phosphatidic acid phosphatase type 2	619549	1	254323970	254326819	2850	553,82	0,02	9,83E-01
ENSRNOG00000015744	**Spata25**	spermatogenesis associated 25 (Spat	499943	3	167477457	167479409	1953	60,68	-0,02	9,83E-01
ENSRNOG00000015980	**Dclre1c**	DNA cross-link repair 1C (Dclre1c), ml	259171	17	80427568	80455878	28311	70,60	-0,02	9,83E-01
ENSRNOG00000016686	**Tmem167a**	Protein Tmem167a [Source:UniProtK	1E+08	2	18803359	18820412	17054	794,50	0,02	9,83E-01
ENSRNOG00000018507	**Gfpt1**	glutamine fructose-6-phosphate transa	297417	4	183421403	183467041	45639	1815,98	-0,01	9,83E-01
ENSRNOG00000020014	**Myh14**	myosin, heavy chain 14, non-muscle (	308572	1	101674362	101737053	62692	957,63	0,02	9,83E-01
ENSRNOG00000020272		RIKEN cDNA 5330417C22 gene [Source:MGI S		2	230716011	230792265	76255	238,78	0,03	9,83E-01
ENSRNOG00000022572	**LOC10091**	peroxisomal biogenesis factor 19 (Pex	289233	18	70740144	70749094	8951	89,31	-0,02	9,83E-01
ENSRNOG00000024241	**Cdc23**	cell division cycle 23 (Cdc23), mRNA [	291689	18	27146157	27165911	19755	1794,84	0,02	9,83E-01
ENSRNOG00000024798	**Bora**	bora, aurora kinase A activator (Bora),	306102	15	86953494	86975023	21530	115,93	0,02	9,83E-01
ENSRNOG00000026238	**RGD15626**	UPF0583 protein C15orf59 homolog [	501007	8	63057200	63067429	10230	1392,12	0,01	9,83E-01
ENSRNOG00000037237	**Ikbkg**	inhibitor of kappa light polypeptide ger	309295	1	151997874	152011467	13594	130,31	-0,02	9,83E-01
ENSRNOG00000043201	**Adck3**	aarF domain containing kinase 3 (Adc	360887	13	103459496	103486271	26776	530,56	0,03	9,83E-01
ENSRNOG00000049286		Uncharacterized protein [Source:UniProtKB/TrE		6	96312761	96335514	22754	93,52	0,02	9,83E-01

ENSRNOG00000050378		protocadherin alpha-3 precursor [Sou	116780	18	29663495	29665864	2370	427,44	0,02	9,83E-01
ENSRNOG00000002506		cyclin-dependent kinase-like 2 (CDC2-	305242	14	17448776	17480554	31779	1241,65	0,02	9,84E-01
ENSRNOG00000003747	**Asna1**	arsA arsenite transporter, ATP-binding	288919	19	37108650	37116734	8085	3198,14	0,02	9,84E-01
ENSRNOG00000008062	**Dqx1**	DEAQ box RNA-dependent ATPase 1	680434	4	178575609	178584448	8840	22,40	-0,03	9,84E-01
ENSRNOG00000010165	**Tnfaip2**	tumor necrosis factor, alpha-induced p	299339	6	144512073	144524703	12631	99,59	-0,03	9,84E-01
ENSRNOG00000000961	**Glt1d1**	Protein Glt1d1 [Source:UniProtKB/TrE	304445	12	34575934	34647621	71688	95,44	-0,02	9,84E-01
ENSRNOG00000001522	**Metap1d**	methionyl aminopeptidase type 1D (m	311748	3	64572489	64646083	73595	173,42	0,02	9,84E-01
ENSRNOG00000002593	**Esrrg**	estrogen-related receptor gamma (Esı	360896	13	111109757	111326800	217044	89,87	0,02	9,84E-01
ENSRNOG00000016351		ferric-chelate reductase 1 [Source:MGI Symbol;/		2	238494314	238531241	36928	32,37	-0,03	9,84E-01
ENSRNOG00000019464	**Ttc9c**	tetratricopeptide repeat domain 9C (Tt	309196	1	231942033	231952730	10698	1949,49	0,02	9,84E-01
ENSRNOG00000021233	**Itpa**	inosine triphosphatase (nucleoside trip	311422	3	129707739	129719394	11656	1960,22	-0,02	9,84E-01
ENSRNOG00000021525		neurobeachin like 1 [Source:MGI Symbol;Acc:M		9	66766280	66923953	157674	106,65	-0,03	9,84E-01
ENSRNOG00000032300	**Rpl39l**	ribosomal protein L39-like (Rpl39l), mF	497860	10	4350271	4354377	4107	54,54	-0,02	9,84E-01
ENSRNOG00000036942	**Tnfrsf9**	tumor necrosis factor receptor superfa	500590	5	171587145	171613565	26421	34,68	0,03	9,84E-01
ENSRNOG00000047115	**Cox16**	COX16 cytochrome c oxidase assemb	1E+08	6	113347562	113360496	12935	370,15	-0,02	9,84E-01
ENSRNOG00000047466	**Bdnf**	brain-derived neurotrophic factor (Bdn	24225	3	107390648	107421863	31216	180,25	0,03	9,84E-01
ENSRNOG00000014984		Protein Dmwd [Source:UniProtKB/TrE	680252	1	81250759	81255534	4776	1722,68	0,02	9,84E-01
ENSRNOG00000010815	**Elf2**	E74-like factor 2 (Elf2), transcript varia	361944	2	159783380	159860729	77350	1145,08	-0,02	9,84E-01
ENSRNOG00000019065	**Zfp385b**	zinc finger protein 385B (Zfp385b), mF	311137	3	70838018	71110119	272102	376,11	-0,02	9,84E-01
ENSRNOG00000006378		Protein Mga [Source:UniProtKB/TrEM	499874	3	118247721	118308351	60631	995,22	0,02	9,84E-01
ENSRNOG00000011642	**Tfap2d**	transcription factor AP-2 delta (Tfap2d	301284	9	24167386	24227618	60233	25,75	-0,03	9,84E-01
ENSRNOG00000020485	**Vav3**	vav 3 guanine nucleotide exchange fa	295378	2	231716228	232053965	337738	348,92	-0,02	9,84E-01
ENSRNOG00000002627	**Kctd3**	potassium channel tetramerization dor	305055	13	112063807	112101984	38178	2721,52	-0,01	9,84E-01
ENSRNOG00000002932	**Wdr19**	WD repeat domain 19 (Wdr19), mRNA	305349	14	44519192	44580950	61759	715,97	0,02	9,84E-01
ENSRNOG00000008192	**Slc48a1**	solute carrier family 48 (heme transpo	300191	7	139463280	139471081	7802	2652,96	0,02	9,84E-01
ENSRNOG00000013595	**Gls**	glutaminase (Gls), nuclear gene encod	24398	9	53923145	53969091	45947	1933,89	0,02	9,84E-01
ENSRNOG00000016387	**Rpl34**	ribosomal protein L34 (Rpl34), mRNA	362041	2	254401908	254405650	3743	1806,70	-0,02	9,84E-01
ENSRNOG00000019946	**Gmppa**	GDP-mannose pyrophosphorylase A (	501167	9	82401645	82409071	7427	474,78	-0,02	9,84E-01
ENSRNOG00000026483	**Trnp1**	Protein Trnp1 [Source:UniProtKB/TrE	688990	5	155371380	155372027	648	285,69	0,03	9,84E-01
ENSRNOG00000031721	**Dnajc30**	DnaJ (Hsp40) homolog, subfamily C, r	368190	12	26669070	26669729	660	217,50	0,01	9,84E-01
ENSRNOG00000033087		cadherin 23 (otocadherin) [Source:MGI Symbol;/		20	31669366	31817711	148346	65,85	0,02	9,84E-01
ENSRNOG00000033912	**Fcho1**	FCH domain only 1 (Fcho1), mRNA [S	290639	16	19995969	20008828	12860	1145,59	-0,01	9,84E-01
ENSRNOG00000017904	**Nol7**	Protein Nol7-ps1 [Source:UniProtKB/	498727	17	25966535	25969643	3109	1140,63	-0,01	9,84E-01
ENSRNOG00000015026	**Arhgef11**	Rho guanine nucleotide exchange fac	78966	2	206385941	206506479	120539	1874,28	0,02	9,84E-01
ENSRNOG00000004214		60S ribosomal protein L26 [Source:R	287417	10	55234796	55237915	3120	551,72	-0,02	9,85E-01
ENSRNOG00000011953	**Supt16h**	suppressor of Ty 16 homolog (S. cere	305851	15	32385107	32422226	37120	1979,96	0,03	9,85E-01
ENSRNOG00000014513	**Exnef**	exonuclease NEF-sp (Exnef), mRNA [	309036	1	196484323	196554085	69763	85,87	-0,02	9,85E-01
ENSRNOG00000017445	**Tubb2b**	tubulin, beta 2B class IIb (Tubb2b), mF	291081	17	33336065	33339110	3046	52453,42	0,02	9,85E-01

ENSRNOG00000019831	**Samd4b**	sterile alpha motif domain containing 4	308473	1	86553119	86577571	24453	2095,89	-0,01	9,85E-01
ENSRNOG00000020459	**Rgs11**	regulator of G-protein signaling 11 (Rg	54291	10	15383438	15391694	8257	26,89	0,03	9,85E-01
ENSRNOG00000046986		Uncharacterized protein [Source:UniProtKB/TrE		2	143352589	143356746	4158	133,72	-0,02	9,85E-01
ENSRNOG00000048911		Uncharacterized protein [Source:UniProtKB/TrE		1	129686743	129693757	7015	41,22	0,02	9,85E-01
ENSRNOG00000022482	**Trappc11**	trafficking protein particle complex 11 (	290746	16	47594590	47640419	45830	1607,74	0,02	9,85E-01
ENSRNOG00000029082	**RGD15661**	Ferritin [Source:UniProtKB/TrEMBL;A	499244	1	183063225	183064130	906	18,81	-0,03	9,85E-01
ENSRNOG00000033613	**RGD15654**	Protein RGD1565459 [Source:UniPro	287782	10	97220705	97221073	369	22,60	0,03	9,85E-01
ENSRNOG00000033865		MRNA stabilizer 2b1 splice variant; Uncharacteri		10	39078693	39105980	27288	36,31	0,03	9,85E-01
ENSRNOG00000010519	**Cyp39a1**	cytochrome P450, family 39, subfamily	301264	9	18681949	18758013	76065	24,22	-0,03	9,85E-01
ENSRNOG00000019422	**Egr1**	early growth response 1 (Egr1), mRNA	24330	18	27369166	27372951	3786	1120,66	-0,02	9,85E-01
ENSRNOG00000020273	**Maz**	MYC-associated zinc finger protein (pu	691842	1	205437135	205440514	3380	3625,11	0,02	9,85E-01
ENSRNOG00000004673	**Rbm43**	RNA binding motif protein 43 (Rbm43)	311020	3	42539334	42549835	10502	81,62	0,02	9,85E-01
ENSRNOG00000033957				MT	11538	11605	68	101,24	0,02	9,85E-01
ENSRNOG00000024824		collagen, type XXII, alpha 1 [Source:M	315071	7	112952964	113165457	212494	236,74	0,02	9,85E-01
ENSRNOG00000007851	**Psma3**	proteasome (prosome, macropain) sul	29670	6	102868534	102898750	30217	2123,86	-0,02	9,86E-01
ENSRNOG00000010309	**Nfyb**	nuclear transcription factor-Y beta (Nfy	25336	7	27200204	27216014	15811	1124,81	0,02	9,86E-01
ENSRNOG00000010657	**LOC10036**	LRRGT00022-like (LOC100363289), r	1E+08	8	86749196	86757691	8496	35,18	-0,02	9,86E-01
ENSRNOG00000017509	**Eed**	embryonic ectoderm development (Ee	293104	1	160493754	160520834	27081	413,35	-0,02	9,86E-01
ENSRNOG00000026705	**Dgki**	diacylglycerol kinase, iota (Dgki), mRN	688705	4	64199211	64653936	454726	745,17	-0,01	9,86E-01
ENSRNOG00000032381	**Naglu**	Protein Naglu; RCG33377, isoform CF	360630	10	88777919	88785165	7247	587,76	-0,02	9,86E-01
ENSRNOG00000001728	**LOC10090**	family with sequence similarity 43, me	288031	11	76749153	76750445	1293	103,29	-0,02	9,86E-01
ENSRNOG00000019830	**Hsd17b1**	hydroxysteroid (17-beta) dehydrogena	25322	10	88786054	88788253	2200	24,93	-0,02	9,86E-01
ENSRNOG00000022772	**Prickle1**	prickle homolog 1 (Drosophila) (Prickle	315259	7	134370195	134389446	19252	943,11	0,02	9,86E-01
ENSRNOG00000024245	**Zkscan2**	Protein Zkscan2 [Source:UniProtKB/T	368120	1	200499515	200517858	18344	228,99	-0,02	9,86E-01
ENSRNOG00000023258	**Nfkb1**	nuclear factor of kappa light polypeptic	81736	2	259330384	259421280	90897	686,48	0,02	9,86E-01
ENSRNOG00000003908		Uncharacterized protein [Source:UniProtKB/TrE		6	124275260	124393300	118041	104,36	-0,03	9,86E-01
ENSRNOG00000011844	**Ints12**	integrator complex subunit 12 (Ints12)	295448	2	256790319	256807886	17568	290,25	0,02	9,86E-01
ENSRNOG00000018075	**Spcs1**	signal peptidase complex subunit 1 hc	290555	16	7008973	7010540	1568	2033,18	0,01	9,86E-01
ENSRNOG00000019553	**Ipo4**	importin 4 (Ipo4), mRNA [Source:RefS	290228	15	38194620	38204540	9921	2732,43	-0,02	9,86E-01
ENSRNOG00000019704	**Resp18**	regulated endocrine-specific protein 18	50561	9	82240055	82246397	6343	320,83	0,02	9,86E-01
ENSRNOG00000036839		heterogeneous nuclear ribonucleoprot	29578	7	142647376	142652966	5591	330,10	0,02	9,86E-01
ENSRNOG00000017730	**Psmd1**	proteasome (prosome, macropain) 26	83806	9	92810407	92884819	74413	4687,78	-0,01	9,86E-01
ENSRNOG00000006186	**Eif2s3**	eukaryotic translation initiation factor 2	299027	X	63800221	63884009	83789	3422,22	-0,02	9,87E-01
ENSRNOG00000013411	**Nup155**	nucleoporin 155 (Nup155), mRNA [So	117021	2	77213963	77265544	51582	757,37	-0,02	9,87E-01
ENSRNOG00000042580	**Zfp866**	Protein Zfp866 [Source:UniProtKB/Tr	1E+08	16	21335976	21337730	1755	39,04	-0,02	9,87E-01
ENSRNOG00000007519	**Tmem43**	transmembrane protein 43 (Tmem43),	362401	4	187412093	187427232	15140	1402,12	0,02	9,87E-01
ENSRNOG00000037220		Protein Rpl5l1 [Source:UniProtKB/TrE	501206	10	112140976	112141869	894	31,69	0,02	9,87E-01
ENSRNOG00000001110	**N4bp2l1**	NEDD4 binding protein 2-like 1 (N4bp	498131	12	533776	563436	29661	111,26	0,02	9,87E-01

ENSRNOG00000006048	**Ezh2**	enhancer of zeste homolog 2 (Drosop	312299	4	141955864	142018471	62608	769,35	0,02	9,87E-01
ENSRNOG00000010945	**Ywhab**	tyrosine 3-monooxygenase/tryptophar	56011	3	166577976	166586801	8826	21158,39	0,02	9,87E-01
ENSRNOG00000013213	**Epha4**	Eph receptor A4 (Epha4), mRNA [Sou	316539	9	82880599	83022444	141846	2801,05	-0,02	9,87E-01
ENSRNOG00000018141	**RGD13074**	similar to mKIAA0319 protein (RGD13	361244	17	44033616	44094176	60561	496,24	0,02	9,87E-01
ENSRNOG00000027183	**Zfyve9**	zinc finger, FYVE domain containing 9	313477	5	132073607	132233186	159580	1648,69	-0,02	9,87E-01
ENSRNOG00000025527		Protein Soga2 [Source:UniProtKB/TrE	316764	9	113998705	114125108	126404	733,47	0,01	9,87E-01
ENSRNOG00000003755	**Clgn**	calmegin (Clgn), mRNA [Source:RefSe	685504	19	35523738	35555259	31522	59,99	0,02	9,87E-01
ENSRNOG00000006731	**Spc25**	SPC25, NDC80 kinetochore complex	295661	3	62050002	62063264	13263	138,18	0,02	9,87E-01
ENSRNOG00000016643	**Lpcat2**	lysophosphatidylcholine acyltransferas	1E+08	19	26567387	26627661	60275	79,29	-0,02	9,87E-01
ENSRNOG00000018944	**Pank1**	pantothenate kinase 1 (Pank1), mRNA	294088	1	260338636	260404074	65439	404,16	-0,01	9,87E-01
ENSRNOG00000023187	**RGD15656**	similar to RIKEN cDNA 1810030O07 (	317344	X	11882874	11904138	21265	282,34	-0,01	9,87E-01
ENSRNOG00000023529	**LOC10090**	ribosomal protein L5 (Rpl5), mRNA [S	81763	14	2861180	2867612	6433	2014,95	0,02	9,87E-01
ENSRNOG00000037640		Uncharacterized protein [Source:UniProtKB/TrE		7	87398222	87640017	241796	19,91	0,02	9,87E-01
ENSRNOG00000049989		cDNA sequence BC068281 [Source:M	500623	6	39922666	39927993	5328	245,35	-0,01	9,87E-01
ENSRNOG00000009508	**Casp6**	caspase 6 (Casp6), transcript variant	83584	2	249539675	249551881	12207	108,93	0,02	9,87E-01
ENSRNOG00000000991	**Arpc1b**	actin related protein 2/3 complex, sub	54227	12	13321403	13334951	13549	407,63	0,02	9,88E-01
ENSRNOG00000000577	**Ddit4**	DNA-damage-inducible transcript 4 (D	140942	20	31315030	31317123	2094	3822,27	0,02	9,88E-01
ENSRNOG00000002387	**Tceal1**	transcription elongation factor A (SII)-li	302593	X	107254190	107256097	1908	559,62	0,01	9,88E-01
ENSRNOG00000006235	**Nell2**	NEL-like 2 (chicken) (Nell2), mRNA [S	81734	7	136173239	136494641	321403	11472,04	0,02	9,88E-01
ENSRNOG00000007849	**Zmat5**	Protein Zmat5 [Source:UniProtKB/TrE	501926	14	85614859	85645517	30659	321,31	0,01	9,88E-01
ENSRNOG00000014204	**Pacsin3**	protein kinase C and casein kinase su	311187	3	86781242	86789804	8563	824,38	-0,02	9,88E-01
ENSRNOG00000021420	**Tirap**	Protein Tirap [Source:UniProtKB/TrEM	680127	8	36396804	36406884	10081	24,05	-0,02	9,88E-01
ENSRNOG00000050742		dynamin binding protein [Source:MGI	309362	1	271071975	271134597	62623	448,17	-0,01	9,88E-01
ENSRNOG00000013110	**Nudt2**	nudix (nucleoside diphosphate linked	297998	5	62373679	62388693	15015	711,44	-0,01	9,88E-01
ENSRNOG00000003178	**Mageh1**	melanoma antigen, family H, 1 (Mageh	367767	X	20009490	20010738	1249	2141,94	0,02	9,88E-01
ENSRNOG00000024365		epithelial cell transforming sequence 2	361921	2	132490052	132546997	56946	344,87	0,02	9,88E-01
ENSRNOG00000025216	**LOC10091**	alkB, alkylation repair homolog 6 (E. c	292780	1	89922890	89928260	5371	21,15	0,02	9,88E-01
ENSRNOG00000029147	**Rbm11**	RNA binding motif protein 11 (Rbm11)	288321	11	17664410	17674391	9982	94,67	-0,02	9,88E-01
ENSRNOG00000039300		putative adenosylhomocysteinase 3 [	312192	4	56817402	56967031	149630	1570,89	-0,02	9,88E-01
ENSRNOG00000019674	**RGD13093**	similar to RIKEN cDNA 4930538D17 (	309454	1	273536065	273553299	17235	502,32	0,01	9,88E-01
ENSRNOG00000014868	**Hspe1**	heat shock protein 1 (chaperonin 10) (	25462	9	61372462	61375650	3189	2491,43	-0,01	9,88E-01
ENSRNOG00000017401	**Tmco4**	transmembrane and coiled-coil domai	500573	5	161163107	161240392	77286	118,35	0,02	9,88E-01
ENSRNOG00000003778	**Opn3**	opsin 3 (Opn3), mRNA [Source:RefSe	498289	13	98180840	98211263	30424	59,17	-0,02	9,88E-01
ENSRNOG00000022932	**Serhl2**	serine hydrolase-like 2 (Serhl2), mRN	500911	7	123946390	123972802	26413	49,64	-0,02	9,88E-01
ENSRNOG00000028083	**Zmym6nb**	ZMYM6 neighbor (Zmym6nb), mRNA	1E+08	5	148958175	148961378	3204	100,59	-0,02	9,88E-01
ENSRNOG00000043300	**LOC10091**	energy homeostasis associated [Sour	1E+08	5	62545002	62545232	231	751,41	0,02	9,88E-01
ENSRNOG00000003014	**Poglut1**	protein O-glucosyltransferase 1 (Poglu	288091	11	67321442	67348158	26717	1257,79	0,02	9,88E-01
ENSRNOG00000008748	**Pex2**	peroxisomal biogenesis factor 2 (Pex2	29534	2	117991484	118007743	16260	595,44	-0,01	9,88E-01

ENSRNOG00000001098	**Pds5b**	PDS5, regulator of cohesion maintena	304218	12	687516	777801	90286	3174,69	-0,01	9,88E-01
ENSRNOG00000013376	**Mvd**	mevalonate (diphospho) decarboxylas	81726	19	65918097	65978333	60237	1886,18	-0,02	9,89E-01
ENSRNOG00000000327	**Hace1**	HECT domain and ankyrin repeat cont	361866	20	52261205	52394868	133664	730,12	0,02	9,89E-01
ENSRNOG00000000572	**Chst3**	carbohydrate (chondroitin 6) sulfotrans	84468	20	31536785	31539327	2543	188,98	0,01	9,89E-01
ENSRNOG00000014382	**C2cd5**	C2 calcium-dependent domain contair	362461	4	241450858	241536419	85562	1540,80	0,02	9,89E-01
ENSRNOG00000002452	**Txndc11**	thioredoxin domain containing 11 (Txn	302899	10	3408243	3465319	57077	1009,37	-0,01	9,89E-01
ENSRNOG00000010233	**Cald1**	caldesmon 1 (Cald1), mRNA [Source:I	25687	4	61952324	62021827	69504	734,76	0,02	9,89E-01
ENSRNOG00000012375	**Inpp1**	inositol polyphosphate-1-phosphatase	316376	9	53229087	53240761	11675	315,76	-0,01	9,89E-01
ENSRNOG00000019918	**Coasy**	CoA synthase (Coasy), mRNA [Source	287711	10	88790983	88795172	4190	1126,10	0,01	9,89E-01
ENSRNOG00000029022	**Zfp112**	zinc finger protein 112 (Zfp112), mRN/	308420	1	82218532	82224354	5823	150,66	-0,01	9,89E-01
ENSRNOG00000000713				11	2766902	2768191	1290	26,95	0,02	9,89E-01
ENSRNOG00000001227	**Adarb1**	adenosine deaminase, RNA-specific, I	25367	20	14137260	14264944	127685	2439,06	-0,02	9,89E-01
ENSRNOG00000002270	**Hnrnpdl**	heterogeneous nuclear ribonucleoprot	305178	14	11143239	11146504	3266	3041,28	0,01	9,89E-01
ENSRNOG00000009293		Uncharacterized protein [Source:UniProtKB/TrE		2	185674828	185676668	1841	27,81	0,02	9,89E-01
ENSRNOG00000009411	**Chn2**	chimerin 2 (Chn2), mRNA [Source:Ref	84031	4	149084636	149113928	29293	391,15	-0,01	9,89E-01
ENSRNOG00000011114	**Tbpl1**	TBP-like 1 (Tbpl1), mRNA [Source:Re	689030	1	25441900	25466736	24837	774,93	0,01	9,89E-01
ENSRNOG00000012141		X-ray repair complementing defective repair in C		6	145379532	145387325	7794	36,97	0,02	9,89E-01
ENSRNOG00000013628	**Cela2a**	chymotrypsin-like elastase family, mer	24332	5	164085554	164095305	9752	26,72	0,02	9,89E-01
ENSRNOG00000013809	**Kbtbd2**	kelch repeat and BTB (POZ) domain c	312372	4	151741021	151763287	22267	1197,50	-0,02	9,89E-01
ENSRNOG00000022631	**Fgf5**	fibroblast growth factor 5 (Fgf5), mRN.	60662	14	12917742	12938405	20664	21,81	-0,02	9,89E-01
ENSRNOG00000029543	**Cish**	cytokine inducible SH2-containing prot	83681	8	115411027	115415970	4944	90,09	-0,02	9,89E-01
ENSRNOG00000029922	**Tpk1**	thiamin pyrophosphokinase 1 (Tpk1),	680668	4	137495338	137869961	374624	128,29	-0,02	9,89E-01
ENSRNOG00000042560	**Bag4**	BCL2-associated athanogene 4 (Bag4	361167	16	70733989	70737834	3846	583,75	0,02	9,89E-01
ENSRNOG00000011214	**Polr2f**	polymerase (RNA) II (DNA directed) p	83503	7	120374383	120386016	11634	2149,55	-0,02	9,89E-01
ENSRNOG00000015631	**Ippk**	inositol 1,3,4,5,6-pentakisphosphate 2	306808	17	17663712	17701633	37922	189,63	-0,02	9,89E-01
ENSRNOG00000009605	**Tfe3**	transcription factor binding to IGHM er	317376	X	16357167	16368638	11472	619,16	-0,01	9,89E-01
ENSRNOG00000015605		Uncharacterized protein [Source:UniProtKB/TrE		1	19260652	19574883	314232	592,78	-0,01	9,89E-01
ENSRNOG00000024411		carbonyl reductase family member 4		359725	16	31794875	31810284	15410	165,64	-0,01	9,89E-01
ENSRNOG00000048172	**Rac3**	Protein LOC688319 [Source:UniProtK	688319	10	109496517	109497867	1351	3502,10	-0,02	9,89E-01
ENSRNOG00000046252	**Tbl2**	Protein LOC686610; RCG21429, isofo	686610	12	26566310	26576309	10000	476,02	-0,01	9,89E-01
ENSRNOG00000006841	**Ano4**	anoctamin 4 (Ano4), mRNA [Source:R	299714	7	29593754	29804859	211106	157,87	-0,02	9,89E-01
ENSRNOG00000043098	**Mt2A**	metallothionein 2A (Mt2A), mRNA [Sou	689415	19	11283094	11283867	774	669,67	-0,02	9,89E-01
ENSRNOG00000015886	**Cnnm4**	cyclin M4 (Cnnm4), mRNA [Source:Re	363216	9	42703466	42740316	36851	345,32	0,01	9,89E-01
ENSRNOG00000004736	**Ralgps2**	Ral GEF with PH domain and SH3 bin	304887	13	79366283	79434660	68378	341,12	-0,01	9,90E-01
ENSRNOG00000029614	**Robo1**	roundabout, axon guidance receptor, I	58946	11	13298875	13807531	508657	3263,23	-0,01	9,90E-01
ENSRNOG00000019485	**Bckdk**	branched chain ketoacid dehydrogena	29603	1	206374937	206379462	4526	1021,70	0,01	9,90E-01
ENSRNOG00000031681		predicted gene 5607 [Source:MGI Symbol;Acc:N		16	81923060	81923819	760	118,73	0,02	9,90E-01
ENSRNOG00000001011	**Fam101a**	family with sequence similarity 101, m	689711	12	38914965	38923860	8896	18,35	0,02	9,90E-01

ENSRNOG00000050169	**Pet100**	PET100 homolog (S. cerevisiae) (Pet1	688786	12	4332962	4335583	2622	924,49	0,01	9,90E-01
ENSRNOG00000050282		Protein Srcap [Source:UniProtKB/TrEMBL;Acc:I		1	206013298	206015031	1734	978,52	-0,01	9,90E-01
ENSRNOG00000004937	**Csmd3**		314942	7	86716233	87187949	471717	215,02	0,02	9,90E-01
ENSRNOG00000012178	**Lmbrd1**	LMBR1 domain containing 1 (Lmbrd1)	246046	9	29762573	29854651	92079	998,90	-0,01	9,91E-01
ENSRNOG00000012500	**Ddx10**	DEAD (Asp-Glu-Ala-Asp) box polypep	300710	8	56244977	56407666	162690	295,25	0,02	9,91E-01
ENSRNOG00000024312	**Xkr8**	XK, Kell blood group complex subunit-	313033	5	154586204	154592456	6253	125,56	0,01	9,91E-01
ENSRNOG00000001647	**Ets2**	v-ets avian erythroblastosis virus E26	304063	11	39606999	39623785	16787	717,95	-0,01	9,91E-01
ENSRNOG00000010017	**Wee1**	wee 1 homolog (S. pombe) (Wee1), m	308937	1	181767890	181786292	18403	570,36	0,01	9,91E-01
ENSRNOG00000011369	**Kcns2**	potassium voltage-gated channel, dela	66022	7	73763134	73765356	2223	105,13	-0,02	9,91E-01
ENSRNOG00000012360	**Gtf2h1**	general transcription factor IIH, polype	361580	1	103923952	103951992	28041	1242,00	0,01	9,91E-01
ENSRNOG00000012654	**Vps36**	vacuolar protein sorting 36 homolog (S	290851	16	74355188	74378996	23809	520,12	0,01	9,91E-01
ENSRNOG00000014556	**Cdh20**	cadherin 20 (Cdh20), mRNA [Source:F	363948	13	30018338	30085298	66961	525,64	0,01	9,91E-01
ENSRNOG00000016877	**Shisa7**	shisa family member 7 (Shisa7), mRN	691429	1	75971937	75990854	18918	2980,31	-0,01	9,91E-01
ENSRNOG00000016891	**Uimc1**	ubiquitin interaction motif containing 1	290997	17	12173068	12239748	66681	552,24	0,01	9,91E-01
ENSRNOG00000017595		Ras suppressor protein 1 [Source:MGI Symbol;A		17	82010120	82197615	187496	429,51	-0,01	9,91E-01
ENSRNOG00000020937	**Ubtf**	upstream binding transcription factor, I	25574	10	90038146	90051350	13205	3921,15	-0,01	9,91E-01
ENSRNOG00000021265	**Cds2**	CDP-diacylglycerol synthase (phospha	114101	3	131392178	131430664	38487	1225,14	0,01	9,91E-01
ENSRNOG00000027125	**Mboat1**	membrane bound O-acyltransferase d	498741	17	37374238	37486374	112137	53,86	-0,01	9,91E-01
ENSRNOG00000036894	**Cisd3**	CDGSH iron sulfur domain 3 (Cisd3),	287661	10	85415958	85418989	3032	165,78	-0,02	9,91E-01
ENSRNOG00000037645	**Tceal7**	transcription elongation factor A (SII)-li	680319	X	106353272	106355368	2097	31,40	0,02	9,91E-01
ENSRNOG00000039980	**Slc25a5**	solute carrier family 25 (mitochondrial	25176	X	123549683	123552808	3126	2450,13	0,01	9,91E-01
ENSRNOG00000041826				3	100988231	100988310	80	31,38	-0,02	9,91E-01
ENSRNOG00000048273	**Apod**	apolipoprotein D (Apod), mRNA [Sour	25239	11	75781495	75804020	22526	45,65	-0,02	9,91E-01
ENSRNOG00000049384		Protein Uggt2 [Source:UniProtKB/TrEMBL;Acc:I		15	107668389	107670932	2544	19,24	-0,02	9,91E-01
ENSRNOG00000014935	**Peo1**	progressive external ophthalmoplegia	309441	1	272198895	272205288	6394	687,71	-0,01	9,91E-01
ENSRNOG00000018320	**Rps15a**	ribosomal protein S15a (Rps15a), mR	117053	1	194713998	194720803	6806	198,17	-0,01	9,91E-01
ENSRNOG00000046905	**Sgce**	sarcoglycan, epsilon (Sgce), mRNA [S	432360	4	29634751	29678921	44171	864,97	0,01	9,91E-01
ENSRNOG00000050792	**Tnfaip6**	tumor necrosis factor alpha induced pr	84397	3	42649266	42663185	13920	55,68	-0,02	9,91E-01
ENSRNOG00000024955	**Scyl2**	SCY1-like 2 (S. cerevisiae) (Scyl2), m	314717	7	30387991	30441330	53340	1423,16	0,01	9,91E-01
ENSRNOG00000012724	**RGD15597**	similar to RIKEN cDNA 0610037L13 (I	298384	5	131337314	131345189	7876	703,15	-0,01	9,91E-01
ENSRNOG00000034269		argonaute RISC catalytic subunit 3 [Source:MGI		5	148131830	148191172	59343	123,66	-0,01	9,91E-01
ENSRNOG00000048342	**Tbca**	tubulin folding cofactor A (Tbca), mRNA [Source		2	43661375	43671792	10418	1751,78	-0,01	9,91E-01
ENSRNOG00000001608	**Nxpe3**	neurexophilin and PC-esterase domai	681096	11	50366320	50415914	49595	405,70	0,01	9,91E-01
ENSRNOG00000001756	**LOC10091**	Protein LOC100911374 [Source:UniP	1E+08	11	75862557	75890080	27524	100,90	-0,01	9,91E-01
ENSRNOG00000005157	**Rbbp7**	retinoblastoma binding protein 7 (Rbb	83712	X	33995673	34012812	17140	2016,75	-0,01	9,91E-01
ENSRNOG00000007219	**LOC10036**	heat shock protein 90, alpha (cytosolic	299331	6	144182014	144187540	5527	1170,06	-0,02	9,91E-01
ENSRNOG00000011550	**Kcnab2**	potassium voltage-gated channel, sha	29738	5	173126375	173212638	86264	567,19	-0,01	9,91E-01
ENSRNOG00000012546	**Frmpd1**	Protein Frmpd1 [Source:UniProtKB/Tr	313244	5	65288586	65346162	57577	925,06	0,01	9,91E-01

ENSRNOG00000013390	**Kbtbd8**	kelch repeat and BTB (POZ) domain c	500262	4	191675891	191687447	11557	92,63	0,02	9,91E-01
ENSRNOG00000015501		Protein Ddhd2 [Source:UniProtKB/TrE	680971	16	70751370	70779434	28065	528,58	0,02	9,91E-01
ENSRNOG00000017660	**Inpp4a**	inositol polyphosphate-4-phosphatase	80849	9	43533507	43706166	172660	3242,26	0,01	9,91E-01
ENSRNOG00000018151	**Isg20**	interferon stimulated exonuclease gen	293052	1	141576506	141582788	6283	57,33	0,01	9,91E-01
ENSRNOG00000018350	**RGD13115**	similar to 4930506M07Rik protein (RG	292139	1	287509273	287581588	72316	2347,81	-0,01	9,91E-01
ENSRNOG00000019322	**Notch1**	notch 1 (Notch1), mRNA [Source:RefS	25496	3	9266672	9311575	44904	3462,24	0,01	9,91E-01
ENSRNOG00000019418	**Lrrc4b**	leucine rich repeat containing 4B (Lrrc	308571	1	101516007	101533767	17761	6880,45	0,01	9,91E-01
ENSRNOG00000019475	**Sh3bp5**	SH3-domain binding protein 5 (BTK-as	117186	16	7408999	7472907	63909	1739,03	-0,01	9,91E-01
ENSRNOG00000019950	**Peli3**	pellino E3 ubiquitin protein ligase fami	309157	1	227124426	227135055	10630	123,37	-0,01	9,91E-01
ENSRNOG00000020839	**Lrfn3**	leucine rich repeat and fibronectin type	308495	1	89975457	89980935	5479	587,61	-0,01	9,91E-01
ENSRNOG00000023360	**Fus**	fused in sarcoma (Fus), mRNA [Sourc	317385	1	206435552	206449420	13869	4798,65	0,01	9,91E-01
ENSRNOG00000024085	**Tmem237**	transmembrane protein 237 (Tmem23	316412	9	65691943	65724365	32423	748,32	-0,01	9,91E-01
ENSRNOG00000025484	**Tmem177**	transmembrane protein 177 (Tmem17	304735	13	41042438	41045862	3425	205,92	-0,01	9,91E-01
ENSRNOG00000026120	**Fam8a1**	Protein Fam8a1 [Source:UniProtKB/T	291031	17	19513325	19519557	6233	952,39	-0,01	9,91E-01
ENSRNOG00000032027	**Tmem42**	transmembrane protein 42 (Tmem42),	363171	8	131260785	131263282	2498	543,10	0,01	9,91E-01
ENSRNOG00000034198	**Wbp5**	WW domain binding protein 5 (Wbp5),	680354	X	106336505	106338637	2133	1143,86	0,01	9,91E-01
ENSRNOG00000047436	**Cabyr**	calcium binding tyrosine-(Y)-phosphor	686189	18	4076915	4088623	11709	28,86	-0,02	9,91E-01
ENSRNOG00000047472		suppressor of zeste 12 homolog (Drosophila) [S		10	66984133	67029188	45056	616,85	-0,01	9,91E-01
ENSRNOG00000048593		Uncharacterized protein [Source:UniProtKB/TrE		5	85276708	85455932	179225	118,05	0,01	9,91E-01
ENSRNOG00000049075	**Fabp5**	fatty acid binding protein 5, epidermal	140868	2	113735166	113738835	3670	17139,01	0,01	9,91E-01
ENSRNOG00000006846	**En2**	engrailed homeobox 2 (En2), mRNA [S	499964	4	433481	439314	5834	37,02	-0,01	9,92E-01
ENSRNOG00000013445	**Aaas**	achalasia, adrenocortical insufficiency,	300259	7	141733241	141751976	18736	991,19	-0,01	9,92E-01
ENSRNOG00000048610				X	151809495	151810803	1309	41,82	0,01	9,92E-01
ENSRNOG00000050552		synaptosomal-associated protein 23 [Source:MG		3	118893523	118908112	14590	42,88	-0,01	9,92E-01
ENSRNOG00000004106		zinc finger, C3H1-type containing [Source:MGI S		7	58375094	58436158	61065	760,80	0,01	9,92E-01
ENSRNOG00000012574	**LOC49933**	Pumilio domain-containing protein KIA	499339	1	252723283	252761006	37724	125,60	-0,01	9,92E-01
ENSRNOG00000043370	**Fubp1**	far upstream element (FUSE) binding	654496	2	276102476	276125397	22922	2692,35	-0,01	9,92E-01
ENSRNOG00000007407	**Ndufa12**	NADH dehydrogenase (ubiquinone) 1	299739	7	35193809	35229886	36078	857,19	-0,01	9,92E-01
ENSRNOG00000038225	**Fbxo15**	F-box protein 15 (Fbxo15), mRNA [So	361354	18	80860288	80873947	13660	31,52	0,02	9,92E-01
ENSRNOG00000002312		Uncharacterized protein [Source:UniProtKB/TrE		14	38288165	38385637	97473	126,34	0,01	9,92E-01
ENSRNOG00000026542	**Suco**	SUN domain containing ossification fa	360863	13	84631122	84694399	63278	739,19	-0,01	9,92E-01
ENSRNOG00000004628	**Dazap2**	DAZ associated protein 2 (Dazap2), m	300235	7	140043812	140048948	5137	6380,53	-0,01	9,92E-01
ENSRNOG00000014846		Uncharacterized protein [Source:UniProtKB/TrE		8	67058674	67066371	7698	41,27	0,01	9,92E-01
ENSRNOG00000043329	**Rnf180**	ring finger protein 180 (Rnf180), mRNA [Source:		2	54840703	54989258	148556	1248,18	0,01	9,92E-01
ENSRNOG00000001178	**Sppl3**	Protein Sppl3-ps1 [Source:UniProtKB	360822	12	49073449	49107696	34248	1772,22	-0,01	9,92E-01
ENSRNOG00000021176	**Mtmr11**	myotubularin related protein 11 (Mtmr1	689613	2	217790151	217798768	8618	354,30	0,02	9,92E-01
ENSRNOG00000004730	**Meis2**	Meis homeobox 2 (Meis2), mRNA [So	311311	3	114124747	114324924	200178	3066,75	-0,01	9,92E-01
ENSRNOG00000001341	**Zfp113**	Protein LOC100912096 [Source:UniP	1E+08	12	21336802	21352518	15717	172,20	0,01	9,92E-01

ENSRNOG00000004372	**Cbln4**	cerebellin 4 precursor (Cbln4), mRNA	499947	3	176112391	176120178	7788	394,67	-0,02	9,92E-01
ENSRNOG00000014258	**Rab32**	RAB32, member RAS oncogene famil	365042	1	6299755	6314539	14785	155,39	-0,01	9,92E-01
ENSRNOG00000030142	**Hist1h4b**	histone cluster 1, H4b (Hist1h4b), mRI	64627	17	58943975	58948418	4444	454,20	-0,01	9,92E-01
ENSRNOG00000000573	**Spock2**	sparc/osteonectin, cwcv and kazal-like	361840	20	31456801	31479643	22843	3261,78	-0,01	9,92E-01
ENSRNOG00000001074	**Mphosph9**	M-phase phosphoprotein 9 (Mphosph9	288654	12	39583052	39639208	56157	156,54	-0,01	9,92E-01
ENSRNOG00000001886	**Dgcr8**	DiGeorge syndrome critical region ger	287954	11	89946649	89979162	32514	1564,91	0,01	9,92E-01
ENSRNOG00000005641		F-box/LRR-repeat protein 4 [Source:F	313101	5	41196942	41270900	73959	289,62	0,01	9,92E-01
ENSRNOG00000006185	**LOC316820**	Ewing tumor-associated antigen 1 (Eta	316820	14	102045074	102047692	2619	19,03	0,02	9,92E-01
ENSRNOG00000006695		ranBP-type and C3HC4-type zinc finge	60383	3	154141158	154157799	16642	1928,78	-0,01	9,92E-01
ENSRNOG00000008754		feline leukemia virus subgroup C rece	314323	6	118926357	118989790	63434	35,33	0,01	9,92E-01
ENSRNOG00000010094	**Kcnmb2**	potassium large conductance calcium-	294961	2	138218940	138256373	37434	39,33	0,02	9,92E-01
ENSRNOG00000010552	**Lamtor3**	late endosomal/lysosomal adaptor, MA	362045	2	261708666	261721119	12454	1369,23	0,01	9,92E-01
ENSRNOG00000010852	**Nup205**	nucleoporin 205 (Nup205), mRNA [So	362335	4	62430061	62496858	66798	1205,80	0,01	9,92E-01
ENSRNOG00000011669	**Gstcd**	glutathione S-transferase, C-terminal c	310855	2	256699240	256790462	91223	60,46	0,01	9,92E-01
ENSRNOG00000011839	**Mrps31**	mitochondrial ribosomal protein S31 (M	290850	16	74102247	74133103	30857	313,37	-0,01	9,92E-01
ENSRNOG00000014183	**Gnaq**	guanine nucleotide binding protein (G	81666	1	240497178	240736864	239687	2387,89	0,01	9,92E-01
ENSRNOG00000016271	**Itm2b**	integral membrane protein 2B (Itm2b),	290364	15	58977919	59000926	23008	16357,03	-0,01	9,92E-01
ENSRNOG00000018214	**Bok**	BCL2-related ovarian killer (Bok), mRN	29884	9	100482657	100493562	10906	1231,36	-0,01	9,92E-01
ENSRNOG00000018304	**Lars**	leucyl-tRNA synthetase (Lars), mRNA	291624	18	36191060	36244275	53216	1581,69	-0,01	9,92E-01
ENSRNOG00000018399	**Cpne6**	copine VI (neuronal) (Cpne6), mRNA [	691478	15	38077840	38084718	6879	520,22	0,01	9,92E-01
ENSRNOG00000020853	**Scap**	SREBF chaperone (Scap), mRNA [So	301024	8	117914034	117972289	58256	3533,23	-0,01	9,92E-01
ENSRNOG00000021166	**Ecm1**	extracellular matrix protein 1 (Ecm1), r	116662	2	217345269	217350435	5167	28,45	-0,01	9,92E-01
ENSRNOG00000024549	**Mum1**	melanoma associated antigen (mutate	362838	7	12501116	12516175	15060	722,59	-0,01	9,92E-01
ENSRNOG00000026799	**Zbtb9**	zinc finger and BTB domain containing	294289	20	7625764	7628014	2251	307,86	0,01	9,92E-01
ENSRNOG00000037242	**Dnase1l1**	deoxyribonuclease 1-like 1 (Dnase1l1)	363522	1	152169732	152178213	8482	179,41	0,01	9,92E-01
ENSRNOG00000042264		Uncharacterized protein [Source:UniProtKB/TrE		18	30520487	30522868	2382	23,24	-0,01	9,92E-01
ENSRNOG00000046327	**Rbpj**	recombination signal binding protein fo	679028	14	59785053	59859229	74177	1087,02	0,01	9,92E-01
ENSRNOG00000048109		40S ribosomal protein S3a [Source:U	1E+08	11	81181713	81182507	795	33,44	0,01	9,92E-01
ENSRNOG00000018340	**Marveld2**	MARVEL domain containing 2 (Marvel	365657	2	49772747	49793880	21134	18,57	0,01	9,92E-01
ENSRNOG00000001789	**Zfp148**	zinc finger protein 148 (Zfp148), mRN	58820	11	73602071	73704363	102293	925,76	0,01	9,92E-01
ENSRNOG00000004387	**Utp23**	UTP23, small subunit (SSU) processo	299900	7	92028550	92033826	5277	192,05	0,01	9,92E-01
ENSRNOG00000004819	**Porcn**	porcupine homolog (Drosophila) (Porc	317368	X	15819273	15831751	12479	919,73	-0,01	9,92E-01
ENSRNOG00000004998	**Jakmip1**	janus kinase and microtubule interacti	305434	14	78398572	78443092	44521	1112,96	-0,01	9,92E-01
ENSRNOG00000006359	**Galk1**	galactokinase 1 (Galk1), mRNA [Sourc	287835	10	103720846	103725036	4191	1035,67	0,01	9,92E-01
ENSRNOG00000019326	**Cfdp1**	craniofacial development protein 1 (Cf	292027	19	54794746	54885270	90525	1660,06	-0,01	9,92E-01
ENSRNOG00000021272	**Mcm8**	minichromosome maintenance comple	296178	3	131954329	131984573	30245	71,95	0,01	9,92E-01
ENSRNOG00000028534	**Zfp518b**	Protein Zfp518b; RCG36096 [Source:	498390	14	76850725	76853958	3234	841,44	0,01	9,92E-01
ENSRNOG00000000823	**Gcc2**	GRIP and coiled-coil domain containin	309798	20	29653463	29699347	45885	459,44	-0,01	9,93E-01

ENSRNOG00000001528	**Gap43**	growth associated protein 43 (Gap43)	29423	11	62777623	62870996	93374	32798,43	0,01	9,93E-01
ENSRNOG00000007538	**Lepre1**	leucine proline-enriched proteoglycan	114200	5	142091634	142106916	15283	467,52	0,01	9,93E-01
ENSRNOG00000010999		RIKEN cDNA 5830418K08 gene [Source:MGI S		8	13810931	13846024	35094	219,98	0,01	9,93E-01
ENSRNOG00000011572	**Klc1**	kinesin light chain 1 (Klc1), transcript v	171041	6	145338402	145370499	32098	14960,31	0,01	9,93E-01
ENSRNOG00000012924	**Tmem194b**	transmembrane protein 194B (Tmem1	503257	9	53380915	53400175	19261	262,81	0,01	9,93E-01
ENSRNOG00000013232		Protein Nr6a1 [Source:UniProtKB/TrE	362125	3	28258302	28400493	142192	22,74	-0,01	9,93E-01
ENSRNOG00000014007	**Gfod1**	glucose-fructose oxidoreductase doma	306842	17	25775676	25873301	97626	266,74	0,01	9,93E-01
ENSRNOG00000014648	**Efnb2**	ephrin B2 (Efnb2), mRNA [Source:Ref	306636	16	86044304	86085652	41349	950,03	0,01	9,93E-01
ENSRNOG00000017074		ras-related protein Rab-28 [Source:R	117049	14	73894215	73970534	76320	1346,25	0,01	9,93E-01
ENSRNOG00000012207		Protein Dst [Source:UniProtKB/TrEMI	316313	9	37587752	37808725	220974	10465,06	0,01	9,93E-01
ENSRNOG00000026660	**RGD13071**	similar to RIKEN cDNA D630029K19 (	294978	2	143374909	143380387	5479	251,15	-0,01	9,93E-01
ENSRNOG00000021030	**Usf2**	upstream transcription factor 2, c-fos i	81817	1	90527311	90538049	10739	1966,48	0,01	9,93E-01
ENSRNOG00000009131	**Zc3h12a**	zinc finger CCCH type containing 12A	313587	5	146878973	146887760	8788	38,04	0,01	9,93E-01
ENSRNOG00000015949	**Aqp9**	aquaporin 9 (Aqp9), mRNA [Source:R	65054	8	75483006	75523420	40415	18,36	0,01	9,93E-01
ENSRNOG00000017431	**RGD13048**	similar to RIKEN cDNA 6430548M08 (	307907	19	64452679	64469186	16508	2297,82	-0,01	9,93E-01
ENSRNOG00000015191	**Phc1**	polyhomeotic homolog 1 (Drosophila)	312690	4	222121225	222143340	22116	2098,31	-0,00	9,93E-01
ENSRNOG00000016122	**Hmgcr**	3-hydroxy-3-methylglutaryl-CoA reduc	25675	2	46592883	46614276	21394	9631,29	0,01	9,93E-01
ENSRNOG00000016627		eyes absent homolog 4 [Source:RefS	292172	1	24878801	25079460	200660	22,54	-0,01	9,93E-01
ENSRNOG00000017897	**Adam8**	Protein Adam8 [Source:UniProtKB/Tr	499285	1	219236991	219248402	11412	28,90	-0,01	9,93E-01
ENSRNOG00000021578	**Wfikkn1**	WAP, follistatin/kazal, immunoglobulin	363555	10	15056372	15058700	2329	50,72	-0,01	9,93E-01
ENSRNOG00000027569		trafficking protein particle complex 9 (T	315059	7	113923438	114244503	321066	1155,24	-0,01	9,93E-01
ENSRNOG00000033529	**Amy1a**	amylase, alpha 1A (salivary) (Amy1a),	24203	2	234523262	234538403	15142	39,25	0,01	9,93E-01
ENSRNOG00000001040	**Pms2**	postmeiotic segregation increased 2 (S	288479	12	14757747	14781994	24248	636,59	0,01	9,93E-01
ENSRNOG00000003125	**Rogdi**	rogdi homolog (Drosophila) (Rogdi), m	287061	10	9528490	9533108	4619	1623,21	-0,01	9,93E-01
ENSRNOG00000003762	**Smarca1**	SWI/SNF related, matrix associated, a	317575	X	134721150	134791365	70216	507,56	-0,01	9,93E-01
ENSRNOG00000007267	**Mnat1**	MNAT CDK-activating kinase assembl	266713	6	105442513	105587332	144820	279,58	-0,01	9,93E-01
ENSRNOG00000007334	**Ly6h**	lymphocyte antigen 6 complex, locus H	300025	7	116499353	116501205	1853	5505,17	0,01	9,93E-01
ENSRNOG00000015534	**Cnep1r1**	CTD nuclear envelope phosphatase 1	291914	19	30924101	30938400	14300	785,94	-0,01	9,93E-01
ENSRNOG00000016256	**Myo9b**	myosin IXb (Myo9b), transcript variant	25486	16	19529581	19614175	84595	1933,50	0,01	9,93E-01
ENSRNOG00000020730	**Nbr1**	neighbor of Brca1 gene 1 (Nbr1), mRN	303554	10	89257664	89281799	24136	4992,56	-0,01	9,93E-01
ENSRNOG00000026874	**Zfp956**	zinc finger protein 956 (Zfp956), mRN	312301	4	142293173	142304997	11825	355,06	0,01	9,93E-01
ENSRNOG00000026814		Protein Qtrtd1 [Source:UniProtKB/TrE	288364	11	60895383	60911712	16330	127,56	0,01	9,93E-01
ENSRNOG00000000512	**Slc26a8**	Protein Slc26a8 [Source:UniProtKB/T	309646	20	7916763	7962295	45533	151,49	0,01	9,93E-01
ENSRNOG00000001082	**Abcb9**	ATP-binding cassette, subfamily B (MI	63886	12	39796337	39829020	32684	464,24	-0,01	9,93E-01
ENSRNOG00000001621	**C2cd2**	C2 calcium-dependent domain contair	304055	11	41867287	41929895	62609	432,27	-0,01	9,93E-01
ENSRNOG00000013708		RGD1307526 protein; Uncharacterized protein		8	76059827	76065681	5855	597,04	-0,01	9,93E-01
ENSRNOG00000007529	**Bmf**	Bcl2 modifying factor (Bmf), mRNA [S	246142	3	116851722	116869739	18018	152,96	0,01	9,93E-01
ENSRNOG00000004951	**Kif16b**	kinesin family member 16B (Kif16b), m	311478	3	143103729	143381497	277769	123,67	-0,01	9,94E-01

ENSRNOG00000014168	**Xylb**	xylulokinase homolog (H. influenzae) (	316067	8	127250936	127283699	32764	557,71	0,01	9,94E-01
ENSRNOG00000028017	**Tmem109**	transmembrane protein 109 (Tmem10	361732	1	233990666	234001285	10620	911,25	0,01	9,94E-01
ENSRNOG00000031479	**Hdlbp**	high density lipoprotein binding proteir	64474	9	100209420	100277671	68252	4882,97	-0,00	9,94E-01
ENSRNOG00000005133		microtubule-associated protein tau (M	29477	10	92050859	92144937	94079	14054,06	0,01	9,94E-01
ENSRNOG00000006009	**Mns1**	meiosis-specific nuclear structural 1 (I	363093	8	74012279	74033156	20878	77,88	0,01	9,94E-01
ENSRNOG00000006559	**Akap8**	A kinase (PRKA) anchor protein 8 (Ak	116633	7	14477686	14493949	16264	1753,79	0,01	9,94E-01
ENSRNOG00000010234	**Nsmaf**	neutral sphingomyelinase (N-SMase)	353233	5	24284202	24330127	45926	456,76	0,01	9,94E-01
ENSRNOG00000012664	**Polr1e**	polymerase (RNA) I polypeptide E (Pc	313245	5	65081629	65097153	15525	467,67	-0,01	9,94E-01
ENSRNOG00000019272	**Gkap1**	G kinase anchoring protein 1 (Gkap1),	361202	17	8989135	9028899	39765	959,58	-0,01	9,94E-01
ENSRNOG00000024011		ankyrin 3, node of Ranvier (ankyrin G)	361833	20	22243056	22609934	366879	5135,91	-0,01	9,94E-01
ENSRNOG00000027903		tripartite motif-containing 46 [Source:MGI Symbo		2	207963162	207974768	11607	1843,52	-0,01	9,94E-01
ENSRNOG00000028481	**Ten1**	TEN1 telomerase capping complex su	360664	10	104741402	104840569	99168	248,12	-0,01	9,94E-01
ENSRNOG00000000860	**Vwa7**	von Willebrand factor A domain contai	309611	20	7081524	7092056	10533	18,15	-0,01	9,94E-01
ENSRNOG00000003461	**Zfp330**	zinc finger protein 330 (Zfp330), mRN	361387	19	34916045	34926423	10379	767,54	0,01	9,94E-01
ENSRNOG00000003606	**RGD15613**	Protein RGD1561381 [Source:UniPro	498340	14	21073667	21074125	459	64,51	-0,01	9,94E-01
ENSRNOG00000005264	**Sav1**	salvador homolog 1 (Drosophila) (Sav	299116	6	101825584	101846408	20825	614,30	-0,01	9,94E-01
ENSRNOG00000016252	**Mblac2**	metallo-beta-lactamase domain contai	365627	2	9458243	9501517	43275	710,58	-0,01	9,94E-01
ENSRNOG00000019439	**Polr2g**	polymerase (RNA) II (DNA directed) p	117017	1	231929424	231932948	3525	2464,12	0,01	9,94E-01
ENSRNOG00000020392	**Stxbp3**	syntaxin binding protein 3 (Stxbp3), m	114095	2	231068919	231112841	43923	395,16	0,01	9,94E-01
ENSRNOG00000024213	**Golim4**	golgi integral membrane protein 4 (Go	310526	2	193658083	193749106	91024	593,88	-0,01	9,94E-01
ENSRNOG00000029813		Uncharacterized protein [Source:UniProtKB/TrE		12	9168919	9180693	11775	21,00	0,01	9,94E-01
ENSRNOG00000030017	**Ercc6**	excision repair cross-complementing r	306274	16	10703928	10770565	66638	807,30	-0,01	9,94E-01
ENSRNOG00000039379	**RGD15629**	similar to chromosome 6 open reading	498729	17	23581994	23595492	13499	34,13	0,01	9,94E-01
ENSRNOG00000049370	**Lsm6**	LSM6 homolog, U6 small nuclear RN	498934	19	43672737	43679724	6988	187,20	-0,01	9,94E-01
ENSRNOG00000000954	**Rpo1-3**	RNA polymerase 1-3 (Rpo1-3), mRNA	360762	12	11806333	11839715	33383	1636,55	-0,01	9,94E-01
ENSRNOG00000000664	**Tpst2**	tyrosylprotein sulfotransferase 2 (Tpst	288719	12	52099687	52139825	40139	475,24	0,01	9,94E-01
ENSRNOG00000001193	**Hsf2bp**	heat shock transcription factor 2 bindir	499413	20	12930027	13014181	84155	37,34	0,01	9,94E-01
ENSRNOG00000002267	**Chic2**	cysteine-rich hydrophobic domain 2 (C	83835	14	35512033	35546381	34349	519,04	-0,01	9,94E-01
ENSRNOG00000002407		pyridoxal-dependent decarboxylase d	304721	10	2110310	2198387	88078	208,33	0,01	9,94E-01
ENSRNOG00000003931	**Arsg**	arylsulfatase G (Arsg), mRNA [Source	303631	10	97497927	97577194	79268	160,72	-0,01	9,94E-01
ENSRNOG00000004344	**LOC68319**	zinc finger, DHHC-type containing 3 (Z	301081	8	131320550	131342691	22142	1513,19	-0,00	9,94E-01
ENSRNOG00000004888	**Spred2**	sprouty-related, EVH1 domain contain	305539	14	103926069	104024317	98249	868,25	0,01	9,94E-01
ENSRNOG00000007099	**S100pbp**	S100P binding protein (S100pbp), mR	500551	5	151069683	151109626	39944	948,67	-0,01	9,94E-01
ENSRNOG00000007726	**Mcam**	melanoma cell adhesion molecule (Mc	78967	8	47091209	47099626	8418	261,88	-0,01	9,94E-01
ENSRNOG00000007735	**Pigm**	phosphatidylinositol glycan anchor bio	79112	13	95281187	95284884	3698	725,68	0,01	9,94E-01
ENSRNOG00000007867	**Rngtt**	RNA guanylyltransferase and 5'-phosp	313131	5	53143346	53362337	218992	1222,93	0,01	9,94E-01
ENSRNOG00000009660		ectonucleotide pyrophosphatase/phos	306460	16	48028067	48150740	122674	25,63	-0,00	9,94E-01
ENSRNOG00000010438	**Cpt1b**	carnitine palmitoyltransferase 1b, mus	25756	7	130080033	130089083	9051	90,33	0,01	9,94E-01

ENSRNOG00000014285	**Ssh2**	slingshot protein phosphatase 2 (Ssh2	303342		10	62402504	62512012	109509	1788,32	-0,00	9,94E-01
ENSRNOG00000015645	**Rnf138**	ring finger protein 138, E3 ubiquitin protein ligas			18	14930045	14956940	26896	915,81	-0,01	9,94E-01
ENSRNOG00000016016	**Zmat2**	zinc finger, matrin type 2 (Zmat2), mR	679898		18	29343457	29347545	4089	1614,91	-0,01	9,94E-01
ENSRNOG00000017744	**Dram2**	DNA-damage regulated autophagy mc	362011		2	228569725	228597446	27722	277,86	-0,01	9,94E-01
ENSRNOG00000018982	**Entpd3**	ectonucleoside triphosphate diphosph	316077		8	128400253	128430331	30079	94,25	-0,01	9,94E-01
ENSRNOG00000018992	**Dpysl3**	dihydropyrimidinase-like 3 (Dpysl3), m	25418		18	37372519	37474987	102469	20759,08	0,01	9,94E-01
ENSRNOG00000019884		RIKEN cDNA 9130023H24 gene [Source:MGI S			1	206700081	206701094	1014	46,89	0,01	9,94E-01
ENSRNOG00000022166	**Ammecr1**	Protein Ammecr1 [Source:UniProtKB/	501539	X		112577876	112679212	101337	40,40	0,01	9,94E-01
ENSRNOG00000022202	**Trappc8**	trafficking protein particle complex 8 (T	291750		18	15077991	15154044	76054	1144,97	-0,01	9,94E-01
ENSRNOG00000022619	**LOC68913**	ferritin, heavy polypeptide 1 (Fth1), mF	25319		1	232979054	232981410	2357	21521,16	0,01	9,94E-01
ENSRNOG00000024230	**Tnfaip8l3**	Protein Tnfaip8l3; Tumor necrosis fact	315673		8	57183192	57226987	43796	51,90	-0,01	9,94E-01
ENSRNOG00000025704	**Yod1**	YOD1 deubiquitinase (Yod1), mRNA [S	363982		13	52529467	52531852	2386	209,28	0,01	9,94E-01
ENSRNOG00000027232	**Pcdhga11**	protocadherin gamma subfamily A, 11	252897		18	30598591	30601151	2561	121,77	0,01	9,94E-01
ENSRNOG00000027630	**Casc1**	cancer susceptibility candidate 1 (Cas	297720		4	243622215	243658236	36022	106,03	-0,01	9,94E-01
ENSRNOG00000007459	**Pcnx**	pecanex homolog (Drosophila) (Pcnx)	314288		6	113754112	113886060	131949	4156,86	0,01	9,94E-01
ENSRNOG00000048473		Uncharacterized protein [Source:UniProtKB/TrE			20	23665733	23889075	223343	89,15	0,01	9,94E-01
ENSRNOG00000001276		pericentrin (kendrin) [Source:MGI Syr	309692		20	15116797	15194771	77975	545,40	-0,00	9,94E-01
ENSRNOG00000016620	**Csnk1g1**	casein kinase 1, gamma 1 (Csnk1g1),	64086		8	71219632	71347691	128060	141,77	-0,01	9,94E-01
ENSRNOG00000016942	**Wdr48**	WD repeat domain 48 (Wdr48), mRNA	363164		8	127778763	127811970	33208	2279,65	0,01	9,95E-01
ENSRNOG00000008949	**Synpo2l**	Protein Synpo2l [Source:UniProtKB/T	305675		15	8165924	8173853	7930	19,51	-0,01	9,95E-01
ENSRNOG00000040005	**Ndufa1**	NADH dehydrogenase (ubiquinone) 1	363441	X		124598917	124602556	3640	1869,98	-0,01	9,95E-01
ENSRNOG00000005272	**Tspan12**	tetraspanin 12 (Tspan12), mRNA [Sou	362326		4	48641397	48715044	73648	1845,80	0,01	9,95E-01
ENSRNOG00000013538	**Capza1**	capping protein (actin filament) muscle	691149		2	226722919	226767300	44382	1208,22	0,01	9,95E-01
ENSRNOG00000017725	**Pnlip**	pancreatic lipase (Pnlip), mRNA [Sour	25702		1	287159727	287172912	13186	57,92	0,01	9,95E-01
ENSRNOG00000028629	**Akt1**	v-akt murine thymoma viral oncogene	24185		6	146226687	146243773	17087	5054,55	0,01	9,95E-01
ENSRNOG00000012488	**Rab2b**	RAB2B, member RAS oncogene famil	305853		15	32487246	32506322	19077	774,69	-0,01	9,95E-01
ENSRNOG00000004679	**Fign**	fidgetin (Fign), mRNA [Source:RefSeq	295649		3	56637401	56764164	126764	48,04	0,01	9,95E-01
ENSRNOG00000011363	**Napepld**	N-acyl phosphatidylethanolamine pho	296757		4	9983118	10003406	20289	67,17	0,01	9,95E-01
ENSRNOG00000015205	**Cyb5a**	cytochrome b5 type A (microsomal) (C	64001		18	80739205	80770883	31679	1184,06	-0,01	9,95E-01
ENSRNOG00000017297	**Cab39**	calcium binding protein 39 (Cab39), m	301574		9	92563873	92625079	61207	3508,73	0,01	9,95E-01
ENSRNOG00000018066	**Bccip**	BRCA2 and CDKN1A interacting prote	361666		1	212726542	212738954	12413	368,15	-0,01	9,95E-01
ENSRNOG00000018873	**Fam168a**	family with sequence similarity 168, m	361614		1	171925496	172076149	150654	7107,33	-0,01	9,95E-01
ENSRNOG00000047751		Uncharacterized protein [Source:UniProtKB/TrE			6	82946933	82948496	1564	61,27	-0,01	9,95E-01
ENSRNOG00000016100	**LOC10036**	Protein LOC100361193 [Source:UniP	366995		7	142294230	142294553	324	433,66	-0,00	9,95E-01
ENSRNOG00000006865	**Laptm4a**	lysosomal protein transmembrane 4 al	298875		6	43822827	43841070	18244	13625,16	0,00	9,95E-01
ENSRNOG00000003769	**Tmem163**	transmembrane protein 163 (Tmem16	360839		13	49258672	49433018	174347	583,68	-0,01	9,95E-01
ENSRNOG00000004301	**Dcaf13**	DDB1 and CUL4 associated factor 13	362902		7	78209281	78244263	34983	509,24	0,01	9,95E-01
ENSRNOG00000005012		U3 small nucleolar RNA-associated pr	317579	X		135163467	135188944	25478	245,48	0,01	9,95E-01

ENSRNOG00000007280	**Naa60**	N(alpha)-acetyltransferase 60, NatF ca	363545	10	10638001	10658047	20047	2350,85	0,00	9,95E-01
ENSRNOG00000009751	**Gpx7**	glutathione peroxidase 7 (Gpx7), mRN	298376	5	131847356	131855200	7845	49,97	-0,01	9,95E-01
ENSRNOG00000010602	**Birc2**	baculoviral IAP repeat-containing 2 (B	60371	8	6015753	6033822	18070	896,68	-0,01	9,95E-01
ENSRNOG00000013046	**LOC10036**	translocating chain-associating membr	1E+08	9	25973255	26047245	73991	43,62	-0,01	9,95E-01
ENSRNOG00000014678				9	71120245	71122004	1760	76,83	0,01	9,95E-01
ENSRNOG00000018584	**LOC10035**	prothymosin alpha (Ptma), mRNA [Sou	29222	9	93273313	93277374	4062	3628,39	0,00	9,95E-01
ENSRNOG00000023312	**Lonrf2**	Protein Lonrf2 [Source:UniProtKB/TrE	301361	9	45165340	45186609	21270	2187,16	-0,00	9,95E-01
ENSRNOG00000033608	**Cd276**	Cd276 molecule (Cd276), mRNA [Sou	315716	8	63090340	63120668	30329	1422,52	-0,01	9,95E-01
ENSRNOG00000042211	**Tmem240**	Protein Tmem240 [Source:UniProtKB	1E+08	5	176659600	176664534	4935	241,23	0,01	9,95E-01
ENSRNOG00000001061	**Rilpl2**	Rab interacting lysosomal protein-like	288652	12	39415025	39426666	11642	812,92	-0,00	9,96E-01
ENSRNOG00000001269		protein phosphatase 1, catalytic subur	24669	12	41744539	41761986	17448	4470,70	0,00	9,96E-01
ENSRNOG00000002266	**Gsx2**	GS homeobox 2 (Gsx2), mRNA [Sourc	364140	14	35479361	35481085	1725	34,92	-0,01	9,96E-01
ENSRNOG00000002643	**Ugdh**	UDP-glucose 6-dehydrogenase (Ugdh	83472	14	44299226	44322226	23001	1085,51	-0,00	9,96E-01
ENSRNOG00000002671	**Nme2**	NME/NM23 nucleoside diphosphate ki	83782	10	81478178	81483679	5502	7719,66	-0,01	9,96E-01
ENSRNOG00000003434	**Trove2**	TROVE domain family, member 2 (Trc	304833	13	65535453	65556443	20991	663,96	-0,01	9,96E-01
ENSRNOG00000004337	**Fam20b**	family with sequence similarity 20, me	304885	13	79201816	79251753	49938	2193,21	0,00	9,96E-01
ENSRNOG00000005528	**Tbk1**	TANK-binding kinase 1 (Tbk1), mRNA	299827	7	63877567	63910298	32732	478,05	0,00	9,96E-01
ENSRNOG00000007053		mediator complex subunit 7 [Source:M	1E+08	10	31347710	31351847	4138	306,10	0,01	9,96E-01
ENSRNOG00000007957	**Bzrap1**	Peripheral-type benzodiazepine recep	287609	10	75025954	75047368	21415	1459,61	0,00	9,96E-01
ENSRNOG00000008195		14-3-3 protein zeta/delta [Source:Unil	25578	7	75721286	75743714	22429	33291,91	0,01	9,96E-01
ENSRNOG00000008618	**Tex10**	testis expressed 10 (Tex10), mRNA [S	298065	5	68695431	68742028	46598	824,31	0,00	9,96E-01
ENSRNOG00000009872	**Kcnh2**	potassium voltage-gated channel, sub	117018	4	7366964	7398603	31640	482,94	-0,00	9,96E-01
ENSRNOG00000011639	**Mrpl47**	mitochondrial ribosomal protein L47 (M	294963	2	138779651	138790789	11139	238,98	-0,01	9,96E-01
ENSRNOG00000011740		KDEL motif-containing protein 1 precu	316370	9	50539118	50551705	12588	413,12	-0,01	9,96E-01
ENSRNOG00000013938	**Tshz3**	teashirt zinc finger homeobox 3 (Tshz3	308523	1	93825019	93896294	71276	884,64	-0,00	9,96E-01
ENSRNOG00000014582	**Mrpl18**	mitochondrial ribosomal protein L18 (M	292244	1	51713678	51718630	4953	1052,99	-0,00	9,96E-01
ENSRNOG00000019175	**Ercc6l2**	Protein Ercc6l2 [Source:UniProtKB/Tr	361197	17	1318565	1414152	95588	351,33	0,01	9,96E-01
ENSRNOG00000019393	**Lima1**	LIM domain and actin binding 1 (Lima1	300228	X	115932904	115999574	66671	2373,32	0,01	9,96E-01
ENSRNOG00000020386	**Ash1l**	ash1 (absent, small, or homeotic)-like	310638	2	207652622	207799105	146484	2874,33	0,00	9,96E-01
ENSRNOG00000021052	**Patl1**	protein associated with topoisomerase	361736	1	235401989	235433951	31963	1508,37	0,00	9,96E-01
ENSRNOG00000022414	**Zfp142**	zinc finger protein 142 (Zfp142), mRN	316524	9	81608286	81630468	22183	1526,29	0,00	9,96E-01
ENSRNOG00000026716	**LOC10036**	ribosomal protein L9 (Rpl9), mRNA [S	29257	14	44343832	44347025	3194	58,53	-0,00	9,96E-01
ENSRNOG00000027088	**Ube2k**	ubiquitin-conjugating enzyme E2K (Ub	289623	14	44108859	44168631	59773	4768,69	0,01	9,96E-01
ENSRNOG00000027136	**Spast**	spastin (Spast), mRNA [Source:RefSe	362700	6	32118321	32168698	50378	3448,96	-0,00	9,96E-01
ENSRNOG00000027408	**Ppid**	peptidylprolyl isomerase D (Ppid), mR	361967	2	197690280	197702613	12334	334,87	-0,01	9,96E-01
ENSRNOG00000028155	**Dnaaf2**	dynein, axonemal, assembly factor 2 (	362746	6	100940356	100949091	8736	377,82	0,00	9,96E-01
ENSRNOG00000031974		Protein Pcdh10 [Source:UniProtKB/TrEMBL;Acc		2	156482756	156503613	20858	3309,17	0,00	9,96E-01
ENSRNOG00000033906		zinc finger protein 667 [Source:MGI Symbol;Acc		1	72665080	72666501	1422	45,10	-0,01	9,96E-01

ENSRNOG00000043123	**Rnf128**	ring finger protein 128, E3 ubiquitin pr	315911	3	53714662	53773426	58765	357,52	-0,01	9,96E-01
ENSRNOG00000046603		small integral membrane protein 20 [Source:MG		14	60072827	60190437	117611	566,30	-0,01	9,96E-01
ENSRNOG00000003958	**Commd10**	COMM domain containing 10 (Commc	361323	18	40666687	40798787	132101	558,27	0,01	9,96E-01
ENSRNOG00000004061	**Pnn**	pinin, desmosome associated protein	368070	6	89685236	89693549	8314	967,54	0,00	9,96E-01
ENSRNOG00000042451	**Ddx59**	DEAD (Asp-Glu-Ala-Asp) box polypep	289402	13	58350167	58375119	24953	347,02	0,00	9,96E-01
ENSRNOG00000018106	**Neu3**	sialidase 3 (membrane sialidase) (Neu	117185	1	171006740	171017255	10516	32,73	0,01	9,96E-01
ENSRNOG00000021273	**Crls1**	cardiolipin synthase 1 (Crls1), mRNA [	366196	3	131987548	132005390	17843	390,73	0,00	9,96E-01
ENSRNOG00000016239	**Zadh2**	zinc binding alcohol dehydrogenase, c	291403	18	79987672	79997023	9352	536,93	-0,00	9,96E-01
ENSRNOG00000001123	**RGD15623**	uncharacterized protein LOC498188		498188	12	45753578	45773812	20235	666,53	-0,00	9,96E-01
ENSRNOG00000019257	**Kif27**	kinesin family member 27 (Kif27), mRI	246209	17	8905688	8975053	69366	136,84	0,00	9,96E-01
ENSRNOG00000025496	**Usp7**	ubiquitin specific peptidase 7 (herpes	360471	10	5776625	5821051	44427	3282,55	0,00	9,96E-01
ENSRNOG00000028746	**Gsto1**	glutathione S-transferase omega 1 (G	114846	1	275040049	275050032	9984	2273,60	-0,01	9,96E-01
ENSRNOG00000050046				15	51713251	51713339	89	28,53	-0,01	9,96E-01
ENSRNOG00000002153	**Cep135**	Protein Cep135 [Source:UniProtKB/T	305288	14	33837370	33892880	55511	181,20	-0,00	9,96E-01
ENSRNOG00000001964	**Cd47**	Cd47 molecule (Cd47), mRNA [Source	29364	11	56693298	56738925	45628	1290,00	-0,00	9,96E-01
ENSRNOG00000003979		zinc finger, RAN-binding domain conta	304761	13	49746797	49846410	99614	206,26	-0,00	9,96E-01
ENSRNOG00000007102	**Acss1**	acyl-CoA synthetase short-chain famil	296259	3	152779621	152829324	49704	2280,65	0,01	9,96E-01
ENSRNOG00000007183	**LOC10036**	Peptidyl-prolyl cis-trans isomerase A F	25518	14	22609711	22610447	737	19,22	-0,01	9,96E-01
ENSRNOG00000010408		polymerase (RNA) II (DNA directed) p	1E+08	7	75138464	75141444	2981	599,89	-0,00	9,96E-01
ENSRNOG00000012176	**Rab27b**	RAB27B, member RAS oncogene fam	84590	18	67642827	67703841	61015	30,82	0,01	9,96E-01
ENSRNOG00000014876	**Lpin2**	lipin 2 (Lpin2), mRNA [Source:RefSeq	316737	9	118970173	119045052	74880	1760,96	-0,00	9,96E-01
ENSRNOG00000016208	**Setbp1**	Protein Setbp1 [Source:UniProtKB/Tr	291423	18	74772393	75106921	334529	1183,44	0,00	9,96E-01
ENSRNOG00000017059	**Cetn2**	Centrin 2, isoform CRA_a; Protein Ce	84593	1	148656627	148661622	4996	844,24	0,00	9,96E-01
ENSRNOG00000017132	**Snx30**	sorting nexin family member 30 (Snx3	298033	5	81185823	81292332	106510	806,34	-0,00	9,96E-01
ENSRNOG00000018796	**Herpud1**	homocysteine-inducible, endoplasmic	85430	19	11027635	11037998	10364	1333,04	0,00	9,96E-01
ENSRNOG00000022162	**Pbx3**	pre-B-cell leukemia homeobox 3 (Pbx	311876	3	18569629	18763387	193759	1171,34	0,00	9,96E-01
ENSRNOG00000024733	**Mnd1**	Protein Mnd1 [Source:UniProtKB/TrE	295160	2	202485557	202541302	55746	26,85	0,01	9,96E-01
ENSRNOG00000030101	**Traip**	TRAF-interacting protein (Traip), mRN	367167	8	116085076	116104850	19775	189,72	0,00	9,96E-01
ENSRNOG00000033748	**LOC100359613**			4	149872503	149873230	728	278,65	-0,00	9,96E-01
ENSRNOG00000048121		Protein Ercc4 [Source:UniProtKB/TrE	304719	10	898678	925921	27244	84,64	-0,00	9,96E-01
ENSRNOG00000049327		jumonji C domain-containing histone c	684297	4	66726642	66756026	29385	105,27	0,00	9,96E-01
ENSRNOG00000004689	**Ptdss1**	phosphatidylserine synthase 1 (Ptdss	314553	7	71466006	71525416	59411	2213,31	-0,00	9,97E-01
ENSRNOG00000007319	**Trib3**	tribbles homolog 3 (Drosophila) (Trib3	246273	3	154162213	154167791	5579	96,69	-0,00	9,97E-01
ENSRNOG00000007650	**Cd63**	Cd63 molecule (Cd63), mRNA [Source	29186	7	3304375	3305798	1424	6318,40	-0,00	9,97E-01
ENSRNOG00000010042	**Wdfy2**	WD repeat and FYVE domain containi	305956	15	49774557	49900008	125452	663,83	0,00	9,97E-01
ENSRNOG00000011593	**Hey1**	hairy/enhancer-of-split related with YR	155437	2	115062450	115064660	2211	253,10	0,00	9,97E-01
ENSRNOG00000011758	**Fkbp7**	FK506 binding protein 7 (Fkbp7), mRN	295672	3	70095653	70107822	12170	95,78	0,00	9,97E-01
ENSRNOG00000011804	**Stx12**	syntaxin 12 (Stx12), mRNA [Source:R	65033	5	154712006	154740248	28243	3494,58	-0,00	9,97E-01

ENSRNOG00000012137	**Ccdc84**	Protein Ccdc84 [Source:UniProtKB/Tr	689046	8	47348806	47355477	6672	195,42	0,00	9,97E-01
ENSRNOG00000012733	**Ankrd12**	ankyrin repeat domain 12 (Ankrd12), r	316775	9	113137358	113214157	76800	1732,99	0,00	9,97E-01
ENSRNOG00000013690	**RGD13073**	LOC362793 (RGD1307315), mRNA [S	362793	6	146389788	146396583	6796	374,66	-0,00	9,97E-01
ENSRNOG00000014415	**Hsbp1**	heat shock factor binding protein 1 (Hs	286899	19	62733736	62738068	4333	9984,74	-0,00	9,97E-01
ENSRNOG00000015274	**Ddx20**	DEAD (Asp-Glu-Ala-Asp) box polypep	84473	2	227565095	227573086	7992	653,05	0,00	9,97E-01
ENSRNOG00000015614	**Ppp1r16b**	protein phosphatase 1, regulatory sub	680616	3	160348036	160448839	100804	740,95	-0,00	9,97E-01
ENSRNOG00000016818	**Fgfr3**	fibroblast growth factor receptor 3 (Fg	84489	14	82962626	82976486	13861	2209,00	0,00	9,97E-01
ENSRNOG00000020567	**Fibp**	fibroblast growth factor (acidic) intrace	282837	1	227769414	227773733	4320	758,48	0,00	9,97E-01
ENSRNOG00000022325	**Smc2**	structural maintenance of chromosom	362519	5	73152087	73198318	46232	447,96	0,01	9,97E-01
ENSRNOG00000030639	**Usp13**	ubiquitin specific peptidase 13 (Usp13	310306	2	138848099	138965318	117220	815,51	0,00	9,97E-01
ENSRNOG00000008746	**Dtd1**	D-tyrosyl-tRNA deacylase 1 (Dtd1), ml	362227	3	145200425	145361899	161475	2008,95	0,00	9,97E-01
ENSRNOG00000018114	**Acadvl**	acyl-CoA dehydrogenase, very long ch	25363	10	56364442	56369608	5167	919,02	0,00	9,97E-01
ENSRNOG00000020250	**Pcgf6**	polycomb group ring finger 6 (Pcgf6),	309457	1	274241831	274261387	19557	312,48	0,00	9,97E-01
ENSRNOG00000005998	**Smoc1**	SPARC related modular calcium bindi	314280	6	112880630	113039729	159100	1557,75	-0,00	9,97E-01
ENSRNOG00000016919	**Scaf8**	SR-related CTD-associated factor 8 (S	245926	1	45348476	45451204	102729	2750,41	-0,00	9,97E-01
ENSRNOG00000010241	**Hspb11**	heat shock protein family B (small), me	685284	5	130705939	130732192	26254	161,24	-0,00	9,97E-01
ENSRNOG00000002546	**Rchy1**	ring finger and CHY zinc finger domain	289508	14	17533357	17548693	15337	832,94	0,00	9,97E-01
ENSRNOG00000000408	**Gopc**	golgi associated PDZ and coiled-coil m	309774	20	35250250	35299383	49134	1790,81	-0,00	9,97E-01
ENSRNOG00000005916	**Ppm1a**	protein phosphatase, Mg2+/Mn2+ dep	24666	6	103918826	103929711	10886	1872,33	-0,00	9,97E-01
ENSRNOG00000007114	**Psma6**	proteasome (prosome, macropain) sul	29673	6	85725314	85742471	17158	2414,36	-0,00	9,97E-01
ENSRNOG00000007325	**Usp28**	ubiquitin specific peptidase 28 (Usp28	315639	8	51909370	51969623	60254	999,21	-0,00	9,97E-01
ENSRNOG00000012565	**Cln8**	ceroid-lipofuscinosis, neuronal 8 (epile	306619	16	79403688	79413576	9889	495,21	0,00	9,97E-01
ENSRNOG00000013594	**Acp2**	acid phosphatase 2, lysosomal (Acp2)	24162	3	86730222	86739116	8895	1349,89	0,00	9,97E-01
ENSRNOG00000019955	**Ogdhl**	oxoglutarate dehydrogenase-like (Ogc	290566	16	8417213	8439689	22477	955,06	-0,00	9,97E-01
ENSRNOG00000022229	**Sdad1**	SDA1 domain containing 1 (Sdad1), m	289504	14	17163928	17190707	26780	398,83	-0,00	9,97E-01
ENSRNOG00000024595	**Cers6**	Protein Cers6 [Source:UniProtKB/TrE	366065	3	61708611	61906524	197914	314,75	0,00	9,97E-01
ENSRNOG00000027447	**Stam2**	signal transducing adaptor molecule (S	311030	3	43331361	43378116	46756	527,02	-0,00	9,97E-01
ENSRNOG00000036802	**Snhg11**	small nucleolar RNA host gene 11 (no	362256	3	160736978	160743135	6158	174,26	0,00	9,97E-01
ENSRNOG00000000778	**Znrd1as1**	ZNRD1 antisense RNA 1 (Znrd1as1),	499401	20	4118986	4120402	1417	19,58	0,00	9,97E-01
ENSRNOG00000001245	**Pcbp3**	poly(rC) binding protein 3 (Pcbp3), mF	294336	20	14590227	14789565	199339	1534,60	0,00	9,97E-01
ENSRNOG00000011734	**Vti1b**	Vesicle transport through interaction w	1E+08	6	115026970	115040094	13125	1419,50	-0,00	9,97E-01
ENSRNOG00000011913	**Cp**	ceruloplasmin (ferroxidase) (Cp), trans	24268	2	124467580	124510450	42871	299,52	0,00	9,97E-01
ENSRNOG00000013707	**Spata13**	spermatogenesis associated 13 (Spat	305938	15	44749846	44875232	125387	286,46	0,00	9,97E-01
ENSRNOG00000015271	**LOC69113**	zinc finger protein 418 (Zfp418), mRN	292548	1	71012442	71026967	14526	47,40	0,00	9,97E-01
ENSRNOG00000018217	**Syt5**	synaptotagmin V (Syt5), mRNA [Sourc	54309	1	75683291	75691003	7713	4470,71	-0,00	9,97E-01
ENSRNOG00000024470		protein ERGIC-53 precursor [Source:	116666	18	60880943	60901867	20925	781,37	0,00	9,97E-01
ENSRNOG00000030537		solute carrier family 26, member 11 [S	360670	10	108086788	108110278	23491	233,10	-0,00	9,97E-01
ENSRNOG00000003613	**Mid1**	midline 1 (Opitz/BBB syndrome) (Mid1	54252	X	25873594	26001241	127648	149,00	0,00	9,97E-01

ENSRNOG00000047513	**Mdn1**	Protein Mdn1 [Source:UniProtKB/TrE	362498	5	52561738	52570313	8576	109,67	0,00	9,97E-01
ENSRNOG00000001689		ADP-ribosylation factor-like protein 6	363760	11	46049116	46069551	20436	379,50	-0,00	9,97E-01
ENSRNOG00000004926	**RGD13049**	similar to RIKEN cDNA 0610027B03 (	295985	3	106819557	106965873	146317	33,70	-0,00	9,98E-01
ENSRNOG00000016105	**Xrcc5**	X-ray repair complementing defective	363247	9	79432205	79520611	88407	541,66	0,00	9,98E-01
ENSRNOG00000020829	**Them4**	thioesterase superfamily member 4 (T	361992	2	215034646	215055404	20759	770,30	-0,00	9,98E-01
ENSRNOG00000006905		HECT domain containing 1 [Source:M	362736	6	82073589	82157549	83961	2806,37	-0,00	9,98E-01
ENSRNOG00000000233	**Grm6**	glutamate receptor, metabotropic 6 (G	24419	10	36116940	36133439	16500	48,74	-0,00	9,98E-01
ENSRNOG00000033202		F-box protein 41 [Source:MGI Symbol;Acc:MGI:		4	181834385	181844668	10284	1185,25	-0,00	9,98E-01
ENSRNOG00000015941	**Fkbp10**	FK506 binding protein 10 (Fkbp10), m	360627	10	88119584	88131443	11860	821,57	0,00	9,98E-01
ENSRNOG00000001111	**Brca2**	breast cancer 2, early onset (Brca2), r	360254	12	491025	531772	40748	159,40	0,00	9,98E-01
ENSRNOG00000006929	**Hnrnpll**	Protein Hnrpll [Source:UniProtKB/TrE	313842	6	2678572	2708005	29434	1776,73	0,00	9,98E-01
ENSRNOG00000006144	**Cntn3**	contactin 3 (plasmacytoma associated	54279	4	199262482	199546616	284135	262,87	-0,00	9,98E-01
ENSRNOG00000006345	**Sec61b**	Sec61 beta subunit (Sec61b), mRNA [	298068	5	67711881	67720091	8211	2011,17	-0,00	9,98E-01
ENSRNOG00000006589	**Mif**	macrophage migration inhibitory factor	81683	20	15885248	15886113	866	2803,83	0,00	9,98E-01
ENSRNOG00000007515	**Kif3a**	kinesin family member 3a (Kif3a), mRI	84392	10	38700476	38733694	33219	3777,31	0,00	9,98E-01
ENSRNOG00000012185	**Zfyve21**	zinc finger, FYVE domain containing 2	362789	6	145388967	145408787	19821	697,80	0,00	9,98E-01
ENSRNOG00000037206	**Ccdc77**	coiled-coil domain containing 77 (Ccdc	312677	4	219949770	219972305	22536	167,10	-0,00	9,98E-01
ENSRNOG00000048814		Uncharacterized protein [Source:UniProtKB/TrE		9	26253133	26254365	1233	104,94	-0,00	9,98E-01
ENSRNOG00000009006	**Ccdc47**	coiled-coil domain containing 47 (Ccdc	303606	10	94139732	94158251	18520	3275,75	-0,00	9,99E-01
ENSRNOG00000014753	**Tcf7l1**	transcription factor 7-like 1 (T-cell spec	312451	4	165263183	165426980	163798	479,80	0,00	9,99E-01
ENSRNOG00000018205	**Ttl**	tubulin tyrosine ligase (Ttl), mRNA [So	171572	3	128391316	128417936	26621	1228,30	0,00	9,99E-01
ENSRNOG00000019281	**RGD13031**	kidney predominant protein NCU-G1 (	295231	2	207100471	207104060	3590	1221,63	-0,00	9,99E-01
ENSRNOG00000019333		homeodomain interacting protein kinase 1 [Sour		2	225647725	225697789	50065	4136,24	-0,00	9,99E-01
ENSRNOG00000031748	**Kifc2**	kinesin family member C2 (Kifc2), mR	300053	7	117711349	117718783	7435	3614,87	-0,00	9,99E-01
ENSRNOG00000010453	**Klhl7**	kelch-like family member 7 (Klhl7), mF	362303	4	7544414	7593562	49149	2629,70	-0,00	9,99E-01
ENSRNOG00000022582	**Fam58b**	family with sequence similarity 58, me	303321	10	65623147	65624309	1163	223,21	0,00	9,99E-01
ENSRNOG00000031638	**Zcchc16**	Protein Zcchc16 [Source:UniProtKB/T	680778	X	116540436	116541341	906	18,47	-0,00	9,99E-01
ENSRNOG00000049782	**Csf2ra**	colony stimulating factor 2 receptor, al	652957	14	1465139	1469169	4031	110,59	0,00	9,99E-01
ENSRNOG00000022636	**Alpk1**	Protein Alpk1 [Source:UniProtKB/TrEl	310879	2	251345862	251401138	55277	89,03	0,00	9,99E-01
ENSRNOG00000010232	**Enpp5**	ectonucleotide pyrophosphatase/phos	316249	9	18348413	18355062	6650	3239,82	-0,00	9,99E-01
ENSRNOG00000027617	**Ttc26**	tetratricopeptide repeat domain 26 (Tt	500086	4	65904583	65960064	55482	374,50	0,00	9,99E-01
ENSRNOG00000000407	**Dcbld1**	Protein Dcbld1; RCG22100 [Source:U	309773	20	35139175	35232665	93491	305,75	0,00	9,99E-01
ENSRNOG00000022014	**Rab35**	RAB35, member RAS oncogene famil	288700	12	48502884	48516964	14081	2870,13	0,00	9,99E-01
ENSRNOG00000004019	**Phlda1**	pleckstrin homology-like domain, famil	29380	7	54272090	54274294	2205	730,35	0,00	9,99E-01
ENSRNOG00000010484	**Zdhhc21**	zinc finger, DHHC-type containing 21 (	298184	5	104956427	104999765	43339	174,98	0,00	9,99E-01
ENSRNOG00000002323	**RGD15662**	similar to RIKEN cDNA 2610002M06 (	363487	X	79066119	79106756	40638	262,12	-0,00	9,99E-01
ENSRNOG00000022730	**Zfp57**	zinc finger protein 57 (Zfp57), mRNA [	361783	20	4054824	4061113	6290	692,45	-0,00	9,99E-01
ENSRNOG00000017751	**Arl6ip1**	ADP-ribosylation factor-like 6 interacti	293551	1	194724270	194733783	9514	7419,25	0,00	9,99E-01

ENSRNOG00000049034		Protein Ptrh1; RCG45678, isoform CR	362113	3	17148932	17162610	13679	199,55	0,00	9,99E-01
ENSRNOG00000011171	**Rims3**	regulating synaptic membrane exocyto	65025	5	143583599	143612220	28622	358,23	0,00	9,99E-01
ENSRNOG00000006357		SH3-containing GRB2-like protein 3-in	313413	5	126257868	126358529	100662	1453,42	-0,00	9,99E-01
ENSRNOG00000011072		host cell factor 2 [Source:RefSeq pep	314704	7	27226472	27255036	28565	535,78	0,00	9,99E-01
ENSRNOG00000014618	**Med31**	mediator complex subunit 31 (Med31)	287475	10	58521398	58524780	3383	484,25	0,00	9,99E-01
ENSRNOG00000039057	**Klf8**	Protein Klf8-ps1 [Source:UniProtKB/T	302582	X	19603538	19636499	32962	103,00	-0,00	9,99E-01
ENSRNOG00000048870	**St6galnac4**	Protein siat7D [Source:UniProtKB/TrE	407764	3	16954184	16964482	10299	572,92	-0,00	9,99E-01
ENSRNOG00000001055	**Rilpl1**	Rab interacting lysosomal protein-like	304469	12	39361410	39398925	37516	878,00	-0,00	9,99E-01
ENSRNOG00000017001	**Zfp652**	zinc finger protein 652 (Zfp652), mRN/	497984	10	83360955	83376165	15211	533,19	-0,00	9,99E-01
ENSRNOG00000019727	**Dph3**	diphthamide biosynthesis 3 (Dph3), m	680594	16	8121974	8124449	2476	317,61	0,00	9,99E-01
ENSRNOG00000006859	**Insig1**	insulin induced gene 1 (Insig1), mRNA	64194	4	337380	345601	8222	1713,64	-0,00	1,00E+00
ENSRNOG00000014964		haptoglobin (Hp), mRNA [Source:RefS	24464	19	52921330	52925933	4604	35,96	0,00	1,00E+00
ENSRNOG00000021144	**Cebpg**	CCAAT/enhancer binding protein (C/E	25301	1	92418852	92427591	8740	1403,07	-0,00	1,00E+00
ENSRNOG00000021247	**RGD13112**	sperm flagellar protein 1 [Source:RefS	311429	3	130212953	130216821	3869	435,48	-0,00	1,00E+00
ENSRNOG00000022044	**Cabp4**	calcium binding protein 4 (Cabp4), mF	365394	1	226254103	226258660	4558	64,93	0,00	1,00E+00
ENSRNOG00000000830	**Ddr1**	discoidin domain receptor tyrosine kin	25678	20	5536686	5671944	135259	4173,53	-0,00	1,00E+00
ENSRNOG00000000001	**Arsj**	arylsulfatase family, member J (Arsj), r	311013	2	249516525	250013503	496979	8,18	0,45	
ENSRNOG00000000008	**Alx4**	ALX homeobox 4 (Alx4), mRNA [Sour	296511	3	89249578	89286113	36536	1,77	-0,26	
ENSRNOG00000000009	**Tmco5b**	Protein Tmco5b [Source:UniProtKB/T	366158	3	111335116	111350242	15127	5,06	-0,41	
ENSRNOG00000000012	**Tcf15**	transcription factor 15 (Tcf15), mRNA	296272	3	153992077	153998331	6255	13,30	-0,03	
ENSRNOG00000000017	**Steap1**	six transmembrane epithelial antigen c	297738	4	25343969	25354487	10519	13,88	-0,97	
ENSRNOG00000000035	**Tmed11**	Protein Tmed11 [Source:UniProtKB/T	689712	14	1932659	1953305	20647	0,00		
ENSRNOG00000000039				5	23302351	23306655	4305	0,00		
ENSRNOG00000000053	**Crp**	C-reactive protein, pentraxin-related ((	25419	13	95599335	95601216	1882	0,27	-0,03	
ENSRNOG00000000055		cell surface glycoprotein gp42 precurs	305103	13	95506496	95511808	5313	0,28	-0,06	
ENSRNOG00000000065	**Pde6b**	phosphodiesterase 6B, cGMP-specific	289878	14	2327104	2370811	43708	0,19	0,04	
ENSRNOG00000000070	**LOC10091**	down-regulator of transcription 1 (Dr1)	289881	14	1616257	1627609	11353	0,00		
ENSRNOG00000000093				2	129183810	129184177	368	0,16	0,04	
ENSRNOG00000000097	**Cd160**	Protein Cd160 [Source:UniProtKB/TrE	502585	2	218423045	218429666	6622	1,57	0,04	
ENSRNOG00000000109	**Fgf20**	fibroblast growth factor 20 (Fgf20), mF	66017	16	54855241	54861845	6605	0,00		
ENSRNOG00000000124		Uncharacterized protein [Source:UniProtKB/TrE		9	104087	104326	240	0,00		
ENSRNOG00000000137	**Ly86**	lymphocyte antigen 86 (Ly86), mRNA	291359	17	30007745	30080876	73132	3,94	-0,57	
ENSRNOG00000000146		Uncharacterized protein [Source:UniProtKB/TrE		17	85915693	85979867	64175	14,16	-0,55	
ENSRNOG00000000156	**Megf6**	multiple EGF-like-domains 6 (Megf6),	65049	5	174960465	175048912	88448	13,62	-0,85	
ENSRNOG00000000165		6-phosphofructo-2-kinase/fructose-2,6	24638	X	23512648	23564313	51666	6,61	0,17	
ENSRNOG00000000167	**Alas2**	aminolevulinate, delta-, synthase 2 (Alas2), mRN		X	23587683	23597195	9513	1,38	0,09	
ENSRNOG00000000187	**Csf2rb**	colony stimulating factor 2 receptor, be	171081	7	119544873	119558539	13667	1,58	-0,22	
ENSRNOG00000000195		von Willebrand factor A domain-contai	301097	8	42434018	42452423	18406	0,14	-0,04	

ENSRNOG00000000196	**Cyp19a1**	cytochrome P450, family 19, subfamily	25147	8	57321085	57348101	27017	7,42	0,13	
ENSRNOG00000000199	**Dppa5**	Protein Dppa5; Protein RGD1559995;	299829	8	85264716	85265568	853	0,00		
ENSRNOG00000000201	**Gsta5**	glutathione S-transferase alpha 2 (Gst	24422	8	85111434	85122897	11464	0,19	0,04	
ENSRNOG00000000207	**RGD15657**	Protein RGD1565766 [Source:UniPro	500622	6	38176259	38178435	2177	15,29	0,21	
ENSRNOG00000000217	**LOC10090**	Glutathione S-transferase [Source:Un	1E+08	8	85078647	85079315	669	15,97	0,27	
ENSRNOG00000000221	**Abhd5**	abhydrolase domain containing 5 (Abh	316122	8	130136517	130163114	26598	363,98	0,22	
ENSRNOG00000000239	**Ccl7**	chemokine (C-C motif) ligand 7 (Ccl7)	287561	10	69058109	69059959	1851	11,84	-0,23	
ENSRNOG00000000251	**St6galnac1**	ST6 (alpha-N-acetyl-neuraminyl-2,3-b	287920	10	105374445	105384844	10400	0,43	-0,09	
ENSRNOG00000000252	**LOC68842**	Protein LOC688424 [Source:UniProtK	688424	19	3591392	3591658	267	0,00		
ENSRNOG00000000282	**Gsc2**	goosecoid homeobox 2 (Gsc2), mRNA	363831	11	90312181	90314169	1989	0,72	0,04	
ENSRNOG00000000296	**Aqp6**	aquaporin 6, kidney specific (Aqp6), m	29170	X	115763284	115766221	2938	0,42	-0,15	
ENSRNOG00000000297	**Aqp2**	aquaporin 2 (collecting duct) (Aqp2), n	25386	X	115742312	115747347	5036	0,41	-0,06	
ENSRNOG00000000311				20	47770834	47772319	1486	3,68	-0,38	
ENSRNOG00000000315				20	49256771	49257158	388	0,00		
ENSRNOG00000000323	**Prdm1**	PR domain containing 1, with ZNF dor	309871	20	51096616	51118240	21625	7,59	0,08	
ENSRNOG00000000335	**Ermap**	Protein Ermap [Source:UniProtKB/TrE	298485	5	142037663	142049164	11502	1,08	0,22	
ENSRNOG00000000345	**LOC10090**	Protein LOC100909961 [Source:UniProtKB/TrE		6	111984163	112115715	131553	0,00		
ENSRNOG00000000373	**Ctnna3**	Protein Ctnna3 [Source:UniProtKB/Tr	361839	20	27189005	27896704	707700	0,63	0,06	
ENSRNOG00000000376		Protein LOC100360884; Protein LOC1	499690	20	28597911	28598135	225	2,37	-0,09	
ENSRNOG00000000378		RGD1562230 protein; Uncharacterized protein		20	28580212	28654253	74042	0,79	-0,09	
ENSRNOG00000000383	**Mypn**	myopalladin (Mypn), mRNA [Source:R	309760	20	28840499	28914635	74137	4,95	-0,32	
ENSRNOG00000000384	**Atoh7**	atonal homolog 7 (Drosophila) (Atoh7)	365564	20	28923499	28923948	450	3,05	0,04	
ENSRNOG00000000386	**Pbld1**	phenazine biosynthesis-like protein dc	171564	20	28943839	28958143	14305	3,64	-0,21	
ENSRNOG00000000390	**LOC10091**	RUN and FYVE domain containing 2 (	690777	20	29061217	29090619	29403	8,99	-0,43	
ENSRNOG00000000394	**Srgn**	serglycin (Srgn), mRNA [Source:RefSe	56782	20	33908574	33921685	13112	1,47	-0,08	
ENSRNOG00000000401		G protein-coupled receptor, family C, g	294394	20	34391557	34412835	21279	0,00		
ENSRNOG00000000402				20	34627658	34628142	485	0,16	0,04	
ENSRNOG00000000403				20	34814754	34816365	1612	0,14	-0,04	
ENSRNOG00000000404		Uncharacterized protein [Source:UniProtKB/TrE		20	34830239	34847800	17562	1,01	0,10	
ENSRNOG00000000405	**Vgll2**	vestigial like 2 (Drosophila) (Vgll2), mF	309772	20	34858272	34864128	5857	2,22	0,15	
ENSRNOG00000000406	**Ros1**	c-ros oncogene 1 , receptor tyrosine k	25346	20	34881357	35027503	146147	1,99	-0,24	
ENSRNOG00000000410	**Nepn**	nephrocan (Nepn), mRNA [Source:Re	309775	20	35306004	35321920	15917	1,21	-0,02	
ENSRNOG00000000413	**Pln**	phospholamban (Pln), mRNA [Source:	64672	20	36390886	36398875	7990	0,67	0,11	
ENSRNOG00000000428	**Cyp21a1**	cytochrome P450, family 21, subfamily	24298	20	6565575	6568637	3063	1,00	0,12	
ENSRNOG00000000443	**LOC10091**	Protein LOC100909666 [Source:UniP	1E+08	20	6575264	6587996	12733	6,02	0,49	
ENSRNOG00000000446	**Btnl7**	butyrophilin-like 7 (Btnl7), mRNA [Sou	406159	20	5007213	5017445	10233	0,00		
ENSRNOG00000000451	**RT1-Ba**	RT1 class II, locus Ba (RT1-Ba), mRN	309621	20	6146271	6150864	4594	0,89	0,21	
ENSRNOG00000000455	**Tap2**	transporter 2, ATP-binding cassette, su	24812	20	6075092	6089133	14042	6,56	0,05	

ENSRNOG00000000461	**LOC10090**	bromodomain containing 2 (Brd2), mR	294276	20	7323527	7330397	6871	2,43	0,38	
ENSRNOG00000000478				20	7549885	7550097	213	0,00		
ENSRNOG00000000479	**Kifc1**	kinesin family member C1 (Kifc1), mR	294286	20	7567671	7585186	17516	788,56	0,37	
ENSRNOG00000000486	**Ggnbp1**	gametogenetin binding protein 1 (Ggn	494520	20	7684426	7690732	6307	5,60	-0,28	
ENSRNOG00000000491	**Spdef**	SAM pointed domain containing ets tra	689210	20	9495176	9509626	14451	11,41	-1,08	
ENSRNOG00000000499	**Tcp11**	t-complex protein 11 (Tcp11), mRNA [S	309641	20	9848951	9858712	9762	5,72	0,27	
ENSRNOG00000000507	**Tulp1**	tubby like protein 1 (Tulp1), mRNA [Sc	309900	20	10132031	10143932	11902	0,19	0,04	
ENSRNOG00000000508	**Armc12**	armadillo repeat containing 12 (Armc1	294301	20	10308966	10319455	10490	0,14	-0,04	
ENSRNOG00000000510	**Clps**	colipase, pancreatic (Clps), mRNA [Sc	25680	20	7838977	7841137	2161	0,00		
ENSRNOG00000000515	**Mapk13**	mitogen activated protein kinase 13 (M	29513	20	8272098	8280990	8893	0,44	-0,06	
ENSRNOG00000000516	**Pxt1**	Protein LOC100360640 [Source:UniP	1E+08	20	8441861	8442921	1061	0,16	0,04	
ENSRNOG00000000528	**Fgd2**	FYVE, RhoGEF and PH domain conta	309653	20	9212091	9228716	16626	7,10	-0,55	
ENSRNOG00000000542		dynein, axonemal, heavy chain 8 [Source:MGI S		20	11501454	11743556	242103	11,42	0,28	
ENSRNOG00000000543	**Frk**	fyn-related kinase (Frk), mRNA [Sourc	79209	20	42996503	43114433	117931	0,67	-0,11	
ENSRNOG00000000545		collagen, type X, alpha 1 [Source:MGI	25681	20	42913374	42918535	5162	1,29	-0,22	
ENSRNOG00000000552	**Col13a1**	collagen, type XIII, alpha 1 (Col13a1),	499431	20	33254962	33395041	140080	3,07	-0,69	
ENSRNOG00000000554				20	32870174	32870753	580	2,82	-0,02	
ENSRNOG00000000556	**Nodal**	nodal growth differentiation factor (No	294503	20	32822880	32831247	8368	0,25	0,05	
ENSRNOG00000000559	**Npffr1**	neuropeptide FF receptor 1 (Npffr1), n	64107	20	33016962	33028398	11437	2,51	-0,30	
ENSRNOG00000000562	**Prf1**	perforin 1 (pore forming protein) (Prf1)	50669	20	32706313	32711803	5491	0,00		
ENSRNOG00000000564		Protein Tbata [Source:UniProtKB/TrE	499429	20	32581738	32595910	14173	0,14	-0,04	
ENSRNOG00000000574		Uncharacterized protein [Source:UniProtKB/TrE		20	31383225	31385285	2061	3,61	0,21	
ENSRNOG00000000580	**Fam26d**	Protein Fam26d [Source:UniProtKB/T	499445	20	29402840	29405551	2712	7,25	-0,02	
ENSRNOG00000000588	**Slc16a10**	solute carrier family 16 (aromatic amin	170566	20	46863306	46979589	116284	7,76	-0,56	
ENSRNOG00000000589		Uncharacterized protein [Source:UniProtKB/TrE		20	46841630	46845915	4286	6,88	-0,40	
ENSRNOG00000000590	**Naglt1**	Na+ dependent glucose transporter 1	337920	20	46815193	46827493	12301	0,14	-0,04	
ENSRNOG00000000597	**Wisp3**	WNT1 inducible signaling pathway prc	499461	20	45964515	45980204	15690	5,08	-0,32	
ENSRNOG00000000607	**RGD15599**	similar to RIKEN cDNA 1700049L16 (I	502418	20	19931569	19932366	798	0,00		
ENSRNOG00000000620		Uncharacterized protein [Source:UniProtKB/TrE		X	118884826	118908105	23280	0,00		
ENSRNOG00000000621				X	119317628	119318152	525	0,00		
ENSRNOG00000000626				X	119687431	119687918	488	0,00		
ENSRNOG00000000630		Uncharacterized protein [Source:UniProtKB/TrE		20	22641378	22642712	1335	0,00		
ENSRNOG00000000634	**RGD13067**	similar to RIKEN cDNA 1700040L02 (I	361834	20	23418565	23526792	108228	8,58	-0,56	
ENSRNOG00000000650		Uncharacterized protein [Source:UniProtKB/TrE		20	25235694	25236336	643	0,00		
ENSRNOG00000000651		Uncharacterized protein [Source:UniProtKB/TrE		20	25645596	25646786	1191	0,00		
ENSRNOG00000000653	**Nr5a2**	nuclear receptor subfamily 5, group A,	60349	13	58794163	58910799	116637	3,00	-0,48	
ENSRNOG00000000655	**Ptprc**	protein tyrosine phosphatase, recepto	24699	13	60094039	60205338	111300	2,26	-0,70	
ENSRNOG00000000656		Uncharacterized protein [Source:UniProtKB/TrE		13	60488188	60489862	1675	1,44	0,16	

ENSRNOG00000000691	**Foxn4**	forkhead box N4 (Foxn4), mRNA [Sou	288736	12	49887751	49907292	19542	9,76	-0,08	
ENSRNOG00000000694	**Dao**	D-amino-acid oxidase (Dao), mRNA [S	114027	12	50135334	50157417	22084	15,92	0,45	
ENSRNOG00000000699	**Selplg**	selectin P ligand (Selplg), mRNA [Sou	363930	12	50359789	50361080	1292	2,26	-0,67	
ENSRNOG00000000700	**Tmem119**	transmembrane protein 119 (Tmem11!	304581	12	50380531	50387588	7058	5,70	-0,61	
ENSRNOG00000000704	**Cmklr1**	chemokine-like receptor 1 (Cmklr1), m	60669	12	50577124	50581377	4254	3,87	-0,34	
ENSRNOG00000000715		Pituitary-specific positive transcription factor 1 [S		11	2630254	2647082	16829	0,78	-0,05	
ENSRNOG00000000716	**Htr1f**	5-hydroxytryptamine (serotonin) recep	60448	11	1960467	1961567	1101	2,54	-0,09	
ENSRNOG00000000726	**Olr1671**	olfactory receptor 1671 (Olr1671), mR	294138	20	384602	385649	1048	0,00		
ENSRNOG00000000728	**Clic2**	chloride intracellular channel 2 (Clic2),	294141	20	289420	304438	15019	13,19	-0,26	
ENSRNOG00000000741				20	1002565	1003200	636	0,00		
ENSRNOG00000000746	**Olr1695**	olfactory receptor 1695 (Olr1695), mR	294159	20	844754	845719	966	0,00		
ENSRNOG00000000747	**Olr1694**	olfactory receptor 1694 (Olr1694), mR	406008	20	831646	832590	945	0,00		
ENSRNOG00000000748				20	829357	829854	498	1,10	0,18	
ENSRNOG00000000749	**Olr1693**	olfactory receptor 1693 (Olr1693), mR	365523	20	814959	815921	963	0,00		
ENSRNOG00000000759	**Olr1869**	olfactory receptor 1869 (Olr1869), mR	294187	20	3692456	3693409	954	0,00		
ENSRNOG00000000765	**Olr1743**	olfactory receptor 1743 (Olr1743), mR	294197	20	3886817	3887758	942	0,00		
ENSRNOG00000000767				20	3915872	3916910	1039	0,91	0,12	
ENSRNOG00000000768	**Ubd**	ubiquitin D (Ubd), mRNA [Source:RefS	29168	20	3917783	3919728	1946	2,09	0,21	
ENSRNOG00000000782	**Trim10**	tripartite motif-containing 10 (Trim10),	294210	20	4219706	4228396	8691	0,85	0,35	
ENSRNOG00000000783	**Trim40**	tripartite motif-containing 40 (Trim40),	365528	20	4206663	4215227	8565	0,14	-0,04	
ENSRNOG00000000787		Uncharacterized protein [Source:UniProtKB/TrE		20	5344905	5347354	2450	4,12	0,50	
ENSRNOG00000000790		Uncharacterized protein [Source:UniProtKB/TrE		20	4669962	4672771	2810	0,16	0,04	
ENSRNOG00000000825	**Fam26e**	family with sequence similarity 26, me	294431	20	29476309	29482652	6344	6,11	-0,35	
ENSRNOG00000000837	**Tnf**	tumor necrosis factor (Tnf), mRNA [Sc	24835	20	6936229	6938839	2611	0,76	-0,13	
ENSRNOG00000000838	**Lta**	lymphotoxin alpha (Lta), mRNA [Sourc	25008	20	6933071	6935077	2007	2,76	0,11	
ENSRNOG00000000843	**Ly6g6c**	lymphocyte antigen 6 complex, locus (	294241	20	7130631	7133328	2698	7,06	-0,22	
ENSRNOG00000000844	**Ly6g6d**	lymphocyte antigen 6 complex, locus (	415062	20	7134779	7137362	2584	0,19	0,04	
ENSRNOG00000000846	**Ly6g5c**	lymphocyte antigen 6 complex, locus (	294245	20	7173674	7177753	4080	0,00		
ENSRNOG00000000853	**Aif1**	allograft inflammatory factor 1 (Aif1), n	29427	20	7234611	7236508	1898	14,51	-0,98	
ENSRNOG00000000854	**Ncr3**	natural cytotoxicity triggering receptor	294251	20	7243480	7249038	5559	0,58	-0,10	
ENSRNOG00000000855	**Lst1**	leukocyte specific transcript 1 (Lst1), r	64569	20	7249282	7252477	3196	1,14	-0,01	
ENSRNOG00000000857	**Msh5**	mutS homolog 5 (E. coli) (Msh5), mRN	294252	20	7093711	7110018	16308	15,52	-0,07	
ENSRNOG00000000868				X	154402193	154403000	808	1,32	0,10	
ENSRNOG00000000871	**Cd40lg**	CD40 ligand (Cd40lg), mRNA [Source	84349	X	154330893	154341958	11066	0,00		
ENSRNOG00000000872				20	7047182	7047804	623	0,70	0,16	
ENSRNOG00000000873	**Brs3**	bombesin-like receptor 3 (Brs3), mRN	260319	X	154135022	154139400	4379	0,14	-0,04	
ENSRNOG00000000878	**Slc44a4**	solute carrier family 44, member 4 (Slc	294255	20	6673376	6689628	16253	14,37	-0,53	
ENSRNOG00000000887				12	6758233	6758976	744	4,99	-0,10	

ENSRNOG00000000889	**LOC30423**	Protein LOC304239 [Source:UniProtK	304239	2	176396082	176435667	39586	1,45	-0,22	
ENSRNOG00000000896		Uncharacterized protein [Source:UniProtKB/TrE		8	89832413	89832919	507	0,61	-0,07	
ENSRNOG00000000897	**Rxfp2**	relaxin/insulin-like family peptide recep	363866	12	8119972	8181214	61243	4,90	-0,18	
ENSRNOG00000000904		WD40 repeat domain 95 [Source:MGI Symbol;A		12	8367747	8417809	50063	1,63	0,14	
ENSRNOG00000000905	**Tex26**	testis expressed 26 (Tex26), mRNA [S	498133	12	8769516	8800588	31073	4,05	-0,37	
ENSRNOG00000000906	**Medag**	Protein Medag [Source:UniProtKB/Tr	360757	12	8809780	8827873	18094	5,95	-0,73	
ENSRNOG00000000907	**Alox5ap**	arachidonate 5-lipoxygenase activatin	29624	12	8950728	8974708	23981	3,76	-0,52	
ENSRNOG00000000916	**LOC10091**	katanin p60 subunit A-like 1 (Katnal1),	288449	12	9305265	9358427	53163	4,74	0,35	
ENSRNOG00000000940	**LOC10091**	FMS-related tyrosine kinase 1 (Flt1), r	54251	12	11155072	11322925	167854	2,29	-0,02	
ENSRNOG00000000943	**LOC68927**	Protein LOC689271 [Source:UniProtK	689271	12	33185002	33185268	267	0,14	-0,04	
ENSRNOG00000000947				12	11470821	11471333	513	0,16	0,04	
ENSRNOG00000000958				7	16320409	16321523	1115	0,19	0,04	
ENSRNOG00000000964	**Frg1l1**	Protein Frg1l1; RCG63649 [Source:U	686103	12	35899612	35900388	777	11,51	0,11	
ENSRNOG00000000969	**Cyp3a18**	cytochrome P450, family 3, subfamily	252931	12	12738271	12787786	49516	0,00		
ENSRNOG00000000973	**RGD15634**	similar to FLJ35784 protein (RGD156	498129	12	4185567	4196186	10620	0,00		
ENSRNOG00000000978		Uncharacterized protein [Source:UniProtKB/TrE		12	13727979	13740677	12699	0,00		
ENSRNOG00000000993	**Pcp2**	Purkinje cell protein 2 (Pcp2), mRNA [	304195	12	4334710	4336463	1754	0,00		
ENSRNOG00000001001	**Retn**	resistin (Retn), mRNA [Source:RefSeq	246250	12	4364007	4365746	1740	0,00		
ENSRNOG00000001005		Fc fragment of IgE, low affinity II, rece	171075	12	4395823	4407330	11508	1,59	-0,08	
ENSRNOG00000001007	**Baiap2l1**	BAI1-associated protein 2-like 1 (Baia	304282	12	14285463	14373559	88097	15,28	-0,71	
ENSRNOG00000001017	**Clec4m**	C-type lectin domain family 4, membe	363856	12	4560987	4567014	6028	0,14	-0,04	
ENSRNOG00000001023	**LOC68885**	Protein LOC688858 [Source:UniProtK	688858	12	5809207	5814195	4989	0,00		
ENSRNOG00000001027		Uncharacterized protein [Source:UniProtKB/TrE		12	4869120	4872081	2962	0,00		
ENSRNOG00000001031	**Ocm2**	oncomodulin 2 (Ocm2), mRNA [Sourc	25503	12	14673089	14678847	5759	8,41	0,36	
ENSRNOG00000001066		inter-alpha trypsin inhibitor, heavy chain 2 [Sour		17	73846179	73882795	36617	1,62	0,39	
ENSRNOG00000001071	**Higd2al1**	Protein Higd2al1 [Source:UniProtKB/	688606	12	6674350	6674670	321	2,47	-0,15	
ENSRNOG00000001092	**Kl**	Klotho (Kl), mRNA [Source:RefSeq mF	83504	12	929158	972144	42987	1,02	-0,18	
ENSRNOG00000001095		Uncharacterized protein [Source:UniProtKB/TrE		12	15432751	15433621	871	0,00		
ENSRNOG00000001152	**Glp1r**	glucagon-like peptide 1 receptor (Glp1	25051	20	11768514	11808416	39903	0,14	-0,04	
ENSRNOG00000001153	**Pla2g1b**	phospholipase A2, group IB, pancreas	29526	12	48676472	48685929	9458	1,61	-0,17	
ENSRNOG00000001157		uromodulin-like 1 [Source:MGI Symbol;Acc:MGI		20	11825676	11880997	55322	4,85	-0,03	
ENSRNOG00000001159	**Tff3**	trefoil factor 3, intestinal (Tff3), mRNA	25563	20	12030960	12035618	4659	14,09	-0,39	
ENSRNOG00000001160	**LOC10091**	TP53 regulated inhibitor of apoptosis	691278	12	48781114	48783573	2460	0,00		
ENSRNOG00000001162	**Tff2**	trefoil factor 2 (Tff2), mRNA [Source:R	116592	20	12052826	12055719	2894	0,00		
ENSRNOG00000001164	**Tff1**	trefoil factor 1 (Tff1), mRNA [Source:R	117270	20	12071835	12075695	3861	0,00		
ENSRNOG00000001165	**Tmprss3**	transmembrane protease, serine 3 (Tr	309665	20	12090233	12109416	19184	0,84	-0,29	
ENSRNOG00000001167	**Ubash3a**	ubiquitin associated and SH3 domain	309666	20	12127337	12164639	37303	0,45	0,05	
ENSRNOG00000001183	**Hnf1a**	HNF1 homeobox A (Hnf1a), mRNA [S	24817	12	49201979	49226746	24768	0,77	0,22	

ENSRNOG00000001195	**Trpv4**	transient receptor potential cation chai	66026	12	49492070	49529956	37887	6,96	-0,15	
ENSRNOG00000001212	**Dnmt3l**	DNA (cytosine-5-)-methyltransferase 3	309680	20	13514654	13528928	14275	0,79	-0,09	
ENSRNOG00000001213	**Aire**	autoimmune regulator (Aire), mRNA [S	294328	20	13535776	13550481	14706	0,28	0,12	
ENSRNOG00000001218	**LOC69043**	similar to chromosome 21 open readir	365547	20	13727387	13738309	10923	0,00		
ENSRNOG00000001219		thrombospondin type laminin G domai	365546	20	13671813	13694360	22548	0,86	-0,09	
ENSRNOG00000001220	**LOC10036**	predicted gene 10142 [Source:MGI Sy	1E+08	20	13792626	13792958	333	0,00		
ENSRNOG00000001224	**Itgb2**	integrin, beta 2 (Itgb2), mRNA [Source	309684	20	13944200	13966161	21962	9,63	-0,74	
ENSRNOG00000001226		Nucleoside diphosphate kinase [Source:UniProt		20	14095197	14095667	471	0,40	-0,07	
ENSRNOG00000001251	**Grifin**	galectin-related inter-fiber protein (Grif	117130	12	18164011	18165993	1983	6,16	-0,13	
ENSRNOG00000001260	**Nudt1**	nudix (nucleoside diphosphate linked i	117260	12	18384509	18387638	3130	196,42	0,14	
ENSRNOG00000001261	**Ftcd**	formimidoyltransferase cyclodeaminas	89833	20	14965125	14978653	13529	0,31	0,12	
ENSRNOG00000001262	**Spatc1l**	spermatogenesis and centriole associ	499418	20	15318496	15327599	9104	1,42	-0,25	
ENSRNOG00000001271	**Card6**	caspase recruitment domain family, m	294770	2	73837349	73848917	11569	12,21	0,29	
ENSRNOG00000001275	**Tmem184a**	transmembrane protein 184A (Tmem1	304325	12	18904350	18912233	7884	0,14	-0,04	
ENSRNOG00000001284	**Uncx**	UNC homeobox (Uncx), mRNA [Sourc	29375	12	19169972	19174488	4517	1,53	0,20	
ENSRNOG00000001287	**Gper1**	G protein-coupled estrogen receptor 1	171104	12	19296904	19298031	1128	14,06	0,11	
ENSRNOG00000001298		Protein Slc5a4b [Source:UniProtKB/T	294342	20	15403024	15457768	54745	0,16	0,04	
ENSRNOG00000001315	**Rtdr1**	rhabdoid tumor deletion region gene 1	365552	20	16760642	16769831	9190	12,30	-0,23	
ENSRNOG00000001329	**Gjc3**	Gap junction protein [Source:UniProtk	288529	12	21225451	21226227	777	4,86	0,09	
ENSRNOG00000001333	**Azgp1**	alpha-2-glycoprotein 1, zinc-binding (A	25294	12	21255662	21263727	8066	2,04	-0,42	
ENSRNOG00000001338	**Hpd**	4-hydroxyphenylpyruvate dioxygenase	29531	12	40729052	40740668	11617	0,88	-0,08	
ENSRNOG00000001370		2 ' -5 ' oligoadenylate synthetase 1H ((	494200	12	43068941	43086182	17242	3,04	0,70	
ENSRNOG00000001379	**Cyp3a62**	cytochrome P450, family 3, subfamily	170509	12	20667012	20696614	29603	15,21	-0,11	
ENSRNOG00000001387	**Mepce**	methylphosphate capping enzyme (Me	304361	12	23998803	24003521	4719	0,14	-0,04	
ENSRNOG00000001392	**Lhx5**	LIM homeobox 5 (Lhx5), mRNA [Sour	124451	12	43513076	43521513	8438	14,26	0,05	
ENSRNOG00000001399	**Tbx5**	T-box 5 (Tbx5), mRNA [Source:RefSe	304514	12	43949166	43996736	47571	0,00		
ENSRNOG00000001412	**Epo**	erythropoietin (Epo), mRNA [Source:R	24335	12	24291481	24294919	3439	0,47	-0,03	
ENSRNOG00000001419	**Cldn15**	claudin 15 (Cldn15), mRNA [Source:R	304388	12	24751108	24758228	7121	10,75	0,31	
ENSRNOG00000001422		uncharacterized protein LOC685612 p	685612	12	24844139	24982182	138044	15,57	-1,17	
ENSRNOG00000001434	**Zp3**	zona pellucida glycoprotein 3 (sperm r	114639	12	25733871	25740838	6968	0,72	0,09	
ENSRNOG00000001444	**Ccl26**	chemokine (C-C motif) ligand 26 (Ccl2	685958	12	26156069	26159172	3104	0,28	-0,06	
ENSRNOG00000001451	**Fkbp6**	FK506 binding protein 6 (Fkbp6), mRN	288597	12	26364514	26435296	70783	0,49	0,05	
ENSRNOG00000001460	**LOC10091**	vacuolar protein sorting 37 homolog C	308178	1	233687419	233712492	25074	0,26	-0,01	
ENSRNOG00000001466	**Cyp2c24**	cytochrome P450, family 2, subfamily	499353	1	265662098	265704202	42105	0,00		
ENSRNOG00000001476	**Cldn4**	claudin 4 (Cldn4), mRNA [Source:Ref	304407	12	26761480	26763275	1796	0,49	0,05	
ENSRNOG00000001480	**Ncf1**	neutrophil cytosolic factor 1 (Ncf1), mF	114553	12	27505793	27514988	9196	12,26	-0,57	
ENSRNOG00000001499	**Mia**	melanoma inhibitory activity (Mia), mR	81510	1	85202541	85204208	1668	8,68	0,14	
ENSRNOG00000001510				3	66061785	66062242	458	1,56	-0,07	

ENSRNOG00000001511		Enolase [Source:UniProtKB/TrEMBL;Acc:D4A0		3	77473917	77475216	1300	0,00		
ENSRNOG00000001512		Enolase [Source:UniProtKB/TrEMBL;Acc:F1M9		10	44417849	44418922	1074	0,12	0,03	
ENSRNOG00000001527	**Cd80**	Cd80 molecule (Cd80), mRNA [Source	25408	11	67256686	67281236	24551	7,91	0,33	
ENSRNOG00000001544	**Cyyr1**	cysteine/tyrosine-rich 1 (Cyyr1), mRN/	304138	11	28596076	28702011	105936	0,00		
ENSRNOG00000001556		Uncharacterized protein [Source:UniProtKB/TrE		3	68939816	68961602	21787	16,98	0,00	
ENSRNOG00000001566		Protein Krtap19-9a [Source:UniProtKB/TrEMBL		11	32489128	32489295	168	0,00		
ENSRNOG00000001567		keratin associated protein 14 [Source:MGI Symb		11	32396247	32396462	216	0,00		
ENSRNOG00000001570	**LOC10036**	RIKEN cDNA 2310061N02 gene [Sou	1E+08	11	32292510	32293037	528	0,00		
ENSRNOG00000001571	**Cldn8**	claudin 8 (Cldn8), mRNA [Source:RefS	304124	11	32146891	32147568	678	0,14	-0,04	
ENSRNOG00000001572	**Hoxd1**	homeo box D1 (Hoxd1), mRNA [Sourc	288151	3	68151156	68153295	2140	0,00		
ENSRNOG00000001577		homeobox D3 [Source:MGI Symbol;A	288152	3	68131906	68134972	3067	0,00		
ENSRNOG00000001578	**Hoxd4**	homeo box D4 (Hoxd4), mRNA [Sourc	288153	3	68099689	68116740	17052	0,00		
ENSRNOG00000001580	**Hoxd9**	homeo box D9 (Hoxd9), mRNA [Sourc	688999	3	68086882	68088227	1346	0,27	-0,03	
ENSRNOG00000001581	**Hoxd10**	homeo box D10 (Hoxd10), mRNA [So	303991	3	68080469	68083678	3210	0,12	0,03	
ENSRNOG00000001584	**LOC30413**	Map3k7 C-terminal like [Source:MGI S	304131	11	30834012	30877227	43216	5,92	0,01	
ENSRNOG00000001587	**Hoxd12**	homeo box D12 (Hoxd12), mRNA [So	366082	3	68063896	68065017	1122	0,00		
ENSRNOG00000001588	**Hoxd13**	homeo box D13 (Hoxd13), mRNA [So	288154	3	68056713	68060029	3317	0,00		
ENSRNOG00000001589	**Evx2**	Protein Evx2 [Source:UniProtKB/TrEM	288155	3	68044676	68048012	3337	0,00		
ENSRNOG00000001591		Uncharacterized protein [Source:UniProtKB/TrE		14	44423928	44424653	726	1,61	0,10	
ENSRNOG00000001595		Protein LOC688891 [Source:UniProtK	688891	3	67837381	67838709	1329	1,38	0,15	
ENSRNOG00000001601		Uncharacterized protein [Source:UniProtKB/TrE		11	50973836	50974132	297	0,00		
ENSRNOG00000001622	**Impg2**	interphotoreceptor matrix proteoglycar	245919	11	49797496	49875529	78034	7,68	0,17	
ENSRNOG00000001627	**Abi3bp**	Protein Abi3bp [Source:UniProtKB/Trl	363767	11	49393776	49607902	214127	10,17	-0,56	
ENSRNOG00000001634	**LOC10091**	G protein-coupled receptor 128 (Gpr1	304014	11	49975766	50038280	62515	0,00		
ENSRNOG00000001644	**Tmem30c**	transmembrane protein 30C (Tmem30	288175	11	48534906	48552064	17159	1,62	-0,33	
ENSRNOG00000001652	**Erg**	v-ets avian erythroblastosis virus E26	170909	11	39232849	39336456	103608	0,77	-0,01	
ENSRNOG00000001656	**Kcnj15**	potassium inwardly-rectifying channel,	170847	11	39154504	39171712	17209	4,28	-0,55	
ENSRNOG00000001675				11	48836880	48837795	916	0,00		
ENSRNOG00000001679	**Gabrr3**	gamma-aminobutyric acid (GABA) A re	192258	11	46236525	46289103	52579	3,90	-0,72	
ENSRNOG00000001684	**Ripply3**	ripply transcriptional repressor 3 (Ripp	288239	11	38145428	38153525	8098	2,21	0,10	
ENSRNOG00000001688	**Sim2**	single-minded homolog 2 (Drosophila)	304071	11	37902514	37942468	39955	2,45	0,03	
ENSRNOG00000001691	**Cldn14**	claudin 14 (Cldn14), mRNA [Source:R	304073	11	37721801	37731973	10173	0,63	0,02	
ENSRNOG00000001704	**Runx1**	runt-related transcription factor 1 (Run	50662	11	36371333	36460969	89637	6,56	0,24	
ENSRNOG00000001711	**Hrasls**	HRAS-like suppressor (Hrasls), mRNA	288025	11	78170405	78188507	18103	9,10	-0,19	
ENSRNOG00000001713				11	77910919	77912199	1281	0,14	-0,04	
ENSRNOG00000001714	**Atp13a4**	ATPase type 13A4 (Atp13a4), mRNA [	288026	11	77880133	78013596	133464	13,58	-0,10	
ENSRNOG00000001732				11	76094251	76094690	440	0,14	-0,04	
ENSRNOG00000001742	**LOC10091**	chloride channel, voltage-sensitive 2 (	29232	11	86955812	86969312	13501	0,00		

ENSRNOG00000001746	**Ncbp2**	phosphatidylinositol glycan anchor bio	689116	11	75147391	75156228	8838	0,33	0,01	
ENSRNOG00000001748	**Polr2h**	polymerase (RNA) II (DNA directed) p	291795	11	85935622	85940070	4449	0,00		
ENSRNOG00000001752	**Nrros**	negative regulator of reactive oxygen s	303875	11	74956756	74973813	17058	7,11	-0,66	
ENSRNOG00000001757	**Tm4sf19**	transmembrane 4 L six family member	288044	11	74692458	74703220	10763	11,48	0,02	
ENSRNOG00000001765	**Slc51a**	solute carrier family 51, alpha subunit	303879	11	74617803	74632201	14399	2,01	-0,21	
ENSRNOG00000001776	**Muc20**	mucin 20, cell surface associated (Mu	303886	11	74307694	74321026	13333	0,30	0,01	
ENSRNOG00000001777		mucin 4 [Source:MGI Symbol;Acc:MGI:2153525		11	74327777	74369743	41967	1,46	-0,22	
ENSRNOG00000001794		mucin 13, epithelial transmembrane [S	207126	11	73275007	73296286	21280	2,85	0,40	
ENSRNOG00000001806	**Fetub**	fetuin B (Fetub), mRNA [Source:RefSe	83928	11	84745603	84756917	11315	0,14	-0,04	
ENSRNOG00000001809	**Hrg**	histidine-rich glycoprotein (Hrg), mRN/	171016	11	84718230	84736638	18409	0,00		
ENSRNOG00000001821	**Adipoq**	adiponectin, C1Q and collagen domai	246253	11	84363940	84382663	18724	0,86	-0,19	
ENSRNOG00000001830		Uncharacterized protein [Source:UniProtKB/TrE		4	245261269	245310513	49245	5,52	-0,07	
ENSRNOG00000001839	**Vpreb2**	pre-B lymphocyte gene 2 (Vpreb2), ml	363830	11	90249249	90249959	711	0,12	0,03	
ENSRNOG00000001840	**Rep15**	RAB15 effector protein (Rep15), mRN	500366	4	245578542	245579615	1074	0,14	-0,04	
ENSRNOG00000001860	**Ccdc116**	coiled-coil domain containing 116 (Ccc	287936	11	91147684	91150766	3083	15,81	-0,26	
ENSRNOG00000001865	**Serpind1**	serpin peptidase inhibitor, clade D (he	79224	11	90969518	90980515	10998	0,12	0,03	
ENSRNOG00000001869	**Rab44**	Protein Rab44 [Source:UniProtKB/TrE	309649	20	8642480	8655402	12923	0,27	-0,03	
ENSRNOG00000001892	**Tbx1**	T-box 1 (Tbx1), mRNA [Source:RefSe	360737	11	89651955	89660875	8921	1,63	-0,25	
ENSRNOG00000001893				11	89521819	89522884	1066	10,80	-0,16	
ENSRNOG00000001908	**Klhl6**	kelch-like family member 6 (Klhl6), mF	287974	11	87808385	87847157	38773	1,43	-0,11	
ENSRNOG00000001912	**Tecrl**	trans-2,3-enoyl-CoA reductase-like (Te	364134	14	26494055	26567265	73211	0,00		
ENSRNOG00000001916	**Tmprss15**	transmembrane protease, serine 15 (T	288291	11	21207441	21331294	123854	5,41	-0,17	
ENSRNOG00000001918				11	26153569	26153917	349	0,16	0,04	
ENSRNOG00000001923	**Tprg1**	tumor protein p63 regulated 1 (Tprg1),	360731	11	81863279	81991056	127778	0,00		
ENSRNOG00000001924		tumor protein p63 (Tp63), transcript va	246334	11	82262249	82492158	229910	3,01	0,21	
ENSRNOG00000001925	**Leprel1**	leprecan-like 1 (Leprel1), mRNA [Sour	288016	11	82549652	82690068	140417	15,77	0,25	
ENSRNOG00000001926	**Cldn1**	claudin 1 (Cldn1), mRNA [Source:RefS	65129	11	82888894	82904046	15153	10,89	-0,80	
ENSRNOG00000001932				11	78352562	78353559	998	1,36	-0,02	
ENSRNOG00000001942	**Vcsa1**	variable coding sequence A1 (Vcsa1),	24867	14	21398248	21398637	390	0,45	0,05	
ENSRNOG00000001943	**Retnlg**	resistin-like gamma (Retnlg), mRNA [S	288135	11	57674751	57676090	1340	0,00		
ENSRNOG00000001945		variable coding sequence A2 precursc	289526	14	21467157	21467453	297	0,00		
ENSRNOG00000001948		Uncharacterized protein [Source:UniProtKB/TrE		14	21516593	21524162	7570	0,14	-0,04	
ENSRNOG00000001949		submaxillary gland androgen-regulate	498341	14	21613121	21616915	3795	0,00		
ENSRNOG00000001950	**Cabs1**	calcium-binding protein, spermatid-spe	64029	14	21656185	21658246	2062	0,00		
ENSRNOG00000001951	**Csn3**	casein kappa (Csn3), mRNA [Source:I	29188	14	21713823	21720378	6556	0,00		
ENSRNOG00000001955	**Retnla**	resistin like alpha (Retnla), mRNA [So	81712	11	57705933	57707432	1500	0,00		
ENSRNOG00000001957	**ste2**	estrogen sulfotransferase (ste2), mRN	25355	14	21986910	22004134	17225	0,00		
ENSRNOG00000001960	**Sult1d1**	sulfotransferase family 1D, member 1	60393	14	22022302	22040897	18596	10,09	-1,06	

ENSRNOG00000001962	**Fam3b**	family with sequence similarity 3, mem	304050	11	41430624	41503454	72831	0,00		
ENSRNOG00000001967	**Sult1b1**	sulfotransferase family, cytosolic, 1B, i	64305	14	22057243	22070527	13285	0,00		
ENSRNOG00000001973		UDP glucuronosyltransferase 2 family, polypepti		14	22107068	22131223	24156	0,00		
ENSRNOG00000001974	**LOC10091**	GRAM domain containing 1C (Gramd1	360717	11	60714570	60776082	61513	10,41	-0,21	
ENSRNOG00000001975	**Ugt2a3**	UDP glucuronosyltransferase 2 family,	289533	14	22165755	22351508	185754	0,00		
ENSRNOG00000001976	**Tmprss2**	transmembrane protease, serine 2 (Tr	156435	11	41574416	41613792	39377	13,23	0,64	
ENSRNOG00000001980	**Ugt2b35**	UDP glucuronosyltransferase 2 family,	83808	14	22455325	22493180	37856	0,56	-0,21	
ENSRNOG00000001984	**Kcne1**	potassium voltage-gated channel, Isk-	25471	11	36101919	36112078	10160	0,56	-0,10	
ENSRNOG00000001986	**LOC49806**	RIKEN cDNA 4930563D23 gene [Sou	498063	11	36073085	36073840	756	0,12	0,03	
ENSRNOG00000001990	**Ugt2b17**	UDP glucuronosyltransferase 2 family,	286954	14	22625222	22636971	11750	1,51	-0,39	
ENSRNOG00000002002	**Tmprss11b**	transmembrane protease, serine 11b (	365265	14	23940743	23960116	19374	0,00		
ENSRNOG00000002005	**Tmprss11f**	Protein Tmprss11f [Source:UniProtKE	498345	14	23817779	23854617	36839	0,59	0,19	
ENSRNOG00000002006	**Tmprss11c**	transmembrane protease, serine 11d (	64565	14	23218327	23289172	70846	0,30	-0,02	
ENSRNOG00000002011	**Gnrhr**	gonadotropin releasing hormone recep	81668	14	23384861	23402815	17955	0,43	-0,09	
ENSRNOG00000002014	**Stap1**	signal transducing adaptor family mem	305269	14	23479675	23508138	28464	2,71	-0,18	
ENSRNOG00000002029				14	1371545	1372535	991	9,25	-0,08	
ENSRNOG00000002031	**Naa11**	N(alpha)-acetyltransferase 11, NatA ca	289482	14	13771578	13781613	10036	0,35	0,13	
ENSRNOG00000002034		Importin subunit alpha [Source:UniProtKB/TrEM		18	33725201	33726789	1589	0,14	-0,04	
ENSRNOG00000002042	**Gfi1**	growth factor independent 1 transcript	24388	14	3060444	3069821	9378	0,00		
ENSRNOG00000002045		annexin A3 (Anxa3), mRNA [Source:R	25291	14	14305733	14351478	45746	8,70	-0,69	
ENSRNOG00000002046		Protein Cd200r1l [Source:UniProtKB/	501779	11	64918042	64932901	14860	1,25	-0,07	
ENSRNOG00000002057	**Slc10a6**	solute carrier family 10 (sodium/bile ac	289459	14	7592605	7656400	63796	4,79	0,27	
ENSRNOG00000002070		Uncharacterized protein [Source:UniProtKB/TrE		14	14893494	14949596	56103	1,80	0,05	
ENSRNOG00000002073		bromodomain, testis-specific (Brdt), m	305123	14	3441091	3465773	24683	0,00		
ENSRNOG00000002084				9	3089553	3090213	661	0,00		
ENSRNOG00000002085	**LOC49835**	similar to testicular haploid expressed	498350	14	33180111	33221418	41308	1,57	0,40	
ENSRNOG00000002110	**Fam114a1l**	Protein Fam114a1l1 [Source:UniProtK	316729	9	119555072	119556772	1701	7,68	-0,52	
ENSRNOG00000002116	**Rplp2**	ribosomal protein, large P2 (Rplp2), m	140662	1	220908603	220910873	2271	0,00		
ENSRNOG00000002117	**Barhl2**	BarH-like homeobox 2 (Barhl2), mRN/	65050	14	4356650	4361538	4889	5,35	-0,40	
ENSRNOG00000002127				14	33387837	33388272	436	0,97	0,07	
ENSRNOG00000002130		Uncharacterized protein [Source:UniProtKB/TrE		14	5830062	5841454	11393	0,77	0,04	
ENSRNOG00000002145	**Tmprss7**	transmembrane protease, serine 7 (Tr	288118	11	64202921	64238347	35427	6,02	-0,05	
ENSRNOG00000002149	**Nkx6-1**	NK6 homeobox 1 (Nkx6-1), mRNA [Sc	65193	14	9516942	9523867	6926	6,25	-0,25	
ENSRNOG00000002156		Uncharacterized protein [Source:UniProtKB/TrE		14	34041127	34042683	1557	0,74	0,21	
ENSRNOG00000002158	**Ibsp**	integrin-binding sialoprotein (Ibsp), mF	24477	14	6789273	6802236	12964	7,38	0,35	
ENSRNOG00000002161	**Tlr6**	toll-like receptor 6 (Tlr6), mRNA [Sour	305353	14	44848509	44850998	2490	11,63	-0,05	
ENSRNOG00000002164	**Nmu**	neuromedin U (Nmu), mRNA [Source:	63887	14	34140350	34167675	27326	0,39	-0,00	
ENSRNOG00000002167	**Dmp1**	dentin matrix acidic phosphoprotein 1	25312	14	6878109	6885158	7050	4,11	-0,35	

ENSRNOG00000002168		dentin sialophosphoprotein (Dspp), ml	25254	14	6914452	6920492	6041	0,00		
ENSRNOG00000002169	**Pdcl2**	Protein Pdcl2 [Source:UniProtKB/TrE	498352	14	34176028	34190891	14864	0,84	-0,29	
ENSRNOG00000002174	**Cwh43**	cell wall biogenesis 43 C-terminal hom	305307	14	37134059	37180298	46240	0,98	-0,33	
ENSRNOG00000002187	**Ropn1**	rhophilin associated tail protein 1 (Rop	288053	11	72417281	72446282	29002	0,41	-0,06	
ENSRNOG00000002188		heparanase [Source:MGI Symbol;Acc:MGI:1343		14	10483181	10523889	40709	52,35	-0,64	
ENSRNOG00000002195		Glyceraldehyde-3-phosphate dehydrogenase [S		14	47023948	47024961	1014	1,89	0,11	
ENSRNOG00000002199	**LOC10091**	transmembrane protein 165 (Tmem16	364137	14	34290288	34315304	25017	0,00		
ENSRNOG00000002200				14	10571469	10572332	864	0,12	0,03	
ENSRNOG00000002201		Uncharacterized protein [Source:UniProtKB/TrE		14	48780281	48781151	871	4,95	-0,73	
ENSRNOG00000002202	**Sowahb**	Protein Sowahb [Source:UniProtKB/T	289501	14	16417565	16419868	2304	2,32	-0,04	
ENSRNOG00000002204	**Ccdc158**	Protein Ccdc158 [Source:UniProtKB/	289502	14	16838813	16887877	49065	13,64	-0,85	
ENSRNOG00000002211		Uncharacterized protein [Source:UniProtKB/TrE		8	85054455	85055187	733	2,79	-0,19	
ENSRNOG00000002212	**Hsd17b13**	hydroxysteroid (17-beta) dehydrogena	305150	14	7103692	7118366	14675	4,89	-0,52	
ENSRNOG00000002217	**Plac8**	placenta-specific 8 (Plac8), mRNA [Sc	360914	14	10640286	10661811	21526	0,14	-0,04	
ENSRNOG00000002223				2	218078619	218078867	249	1,84	-0,19	
ENSRNOG00000002224	**Yipf7**	Yip1 domain family, member 7 (Yipf7),	364147	14	62670745	62698718	27974	3,04	-0,14	
ENSRNOG00000002233				14	41391394	41393101	1708	0,12	0,03	
ENSRNOG00000002243				14	42079628	42080633	1006	0,12	0,03	
ENSRNOG00000002256	**Art3**	ADP-ribosyltransferase 3 (Art3), mRN	305235	14	17059132	17087206	28075	13,76	0,23	
ENSRNOG00000002265	**Casr**	calcium-sensing receptor (Casr), mRN	24247	11	70279170	70348597	69428	6,20	-0,46	
ENSRNOG00000002282	**Ppef2**	protein phosphatase, EF hand calcium	305246	14	17220015	17254101	34087	3,55	0,34	
ENSRNOG00000002285	**LOC10090**	Protein Fam122b; Protein LOC10090	501647	X	152479632	152502537	22906	0,00		
ENSRNOG00000002289	**Ildr1**	immunoglobulin-like domain containing	303914	11	70094409	70127482	33074	0,56	-0,12	
ENSRNOG00000002291		bromodomain and WD repeat domain	317213	X	79999818	80086731	86914	167,28	0,15	
ENSRNOG00000002299		Protein Phox2b [Source:UniProtKB/Tr	364152	14	42510775	42513503	2729	0,00		
ENSRNOG00000002302	**Corin**	corin, serine peptidase (Corin), mRNA	289596	14	38058862	38284566	225705	0,28	0,12	
ENSRNOG00000002304	**Tnfrsf13b**	Protein Tnfrsf13b [Source:UniProtKB/	691719	10	47184940	47193309	8370	0,14	-0,04	
ENSRNOG00000002316	**LOC10091**	complement component 1, q subcomp	300220	X	115272678	115275588	2911	1,10	0,03	
ENSRNOG00000002321				X	79670595	79671062	468	0,42	-0,15	
ENSRNOG00000002331	**Aldh3a1**	aldehyde dehydrogenase 3 family, me	25375	10	47264967	47274685	9719	15,11	-0,52	
ENSRNOG00000002332	**Mospd1**	motile sperm domain containing 1 (Mo	317312	X	152382193	152397168	14976	16,09	-0,06	
ENSRNOG00000002333	**Olr982**	olfactory receptor 982 (Olr982), mRNA	404929	7	4205494	4206429	936	0,00		
ENSRNOG00000002335	**Gspt1**	G1 to S phase transition 1 (Gspt1), mI	24420	10	3277695	3312136	34442	0,00		
ENSRNOG00000002338	**Dgke**	diacylglycerol kinase, epsilon 64kDa (I	497978	10	73703727	73725817	22091	619,30	0,08	
ENSRNOG00000002340	**LOC10036**	Protein LOC100361079 [Source:UniP	1E+08	1	90414056	90414508	453	1,70	-0,32	
ENSRNOG00000002346	**Tbx22**	T-box 22 (Tbx22), mRNA [Source:Ref	302369	X	78966939	78980062	13124	1,69	-0,09	
ENSRNOG00000002350	**Eaf2**	ELL associated factor 2 (Eaf2), mRNA	266787	11	69969912	70013412	43501	2,11	0,03	
ENSRNOG00000002355	**Slc47a1**	solute carrier family 47 (multidrug and	360539	10	47383154	47440136	56983	2,17	0,30	

ENSRNOG00000002379	**Plac1**	placenta-specific 1 (Plac1), mRNA [Sc	317316	X	152678178	152758811	80634	0,35	0,13	
ENSRNOG00000002385	**Prg4**	proteoglycan 4, (megakaryocyte stimu	289104	13	72637409	72653723	16315	8,66	-0,18	
ENSRNOG00000002391	**Glra4**	glycine receptor, alpha 4 (Glra4), mRN	367912	X	107306116	107326637	20522	0,96	-0,25	
ENSRNOG00000002396	**Serpinb8**	serpin peptidase inhibitor, clade B (ova	288937	13	33035661	33058451	22791	0,33	0,01	
ENSRNOG00000002402				13	108210335	108211117	783	1,47	-0,04	
ENSRNOG00000002408	**Rbm47**	RNA binding motif protein 47 (Rbm47)	305340	14	43597574	43625113	27540	2,30	-0,27	
ENSRNOG00000002412	**Mapk7**	mitogen-activated protein kinase 7 (Ma	114509	10	47541088	47546199	5112	0,00		
ENSRNOG00000002417	**Serpinb10**	serpin peptidase inhibitor, clade B (ova	266775	13	32615862	32633549	17688	0,00		
ENSRNOG00000002425	**Pctp**	phosphatidylcholine transfer protein (F	29510	10	77353604	77374000	20397	11,38	-0,28	
ENSRNOG00000002429		zinc finger, CCHC domain containing 5 [Source:I		X	78150794	78152662	1869	2,82	-0,23	
ENSRNOG00000002451	**Fndc3c1**	fibronectin type III domain containing 3	317224	X	77628482	77754338	125857	11,90	-0,01	
ENSRNOG00000002459	**Fbxo40**	Protein Fbxo40 [Source:UniProtKB/Tr	363790	11	69805391	69822494	17104	0,00		
ENSRNOG00000002460	**Serpinb2**	serpin peptidase inhibitor, clade B (ova	60325	13	32603846	32612854	9009	0,88	-0,07	
ENSRNOG00000002462	**LOC10091**	B9 protein domain 1 (B9d1), mRNA [S	287383	10	48805171	48812716	7546	0,16	0,04	
ENSRNOG00000002470	**Ifi47**	interferon gamma inducible protein 47	246208	10	34059639	34067269	7631	17,37	0,74	
ENSRNOG00000002475				7	138517314	138517832	519	13,30	0,44	
ENSRNOG00000002484	**Chrna9**	cholinergic receptor, nicotinic, alpha 9	65024	14	43670533	43677246	6714	0,76	-0,08	
ENSRNOG00000002486				10	34368918	34369855	938	0,16	0,04	
ENSRNOG00000002487	**Mbnl3**	muscleblind-like splicing regulator 3 (M	302492	X	138438371	138488850	50480	4,59	-0,37	
ENSRNOG00000002495				6	28607037	28607816	780	0,00		
ENSRNOG00000002497	**LOC10091**	Protein LOC100912616 [Source:UniP	1E+08	10	70623541	70624388	848	0,00		
ENSRNOG00000002501	**RGD13095**	similar to probable ATP-dependent RN	364073	13	110058379	110061548	3170	3,21	0,08	
ENSRNOG00000002505	**Itih6**	Protein LOC100912775 [Source:UniP	1E+08	5	37815093	37828006	12914	0,58	-0,12	
ENSRNOG00000002511	**Flt4**	fms-related tyrosine kinase 4 (Flt4), m	114110	10	34850707	34892141	41435	2,09	0,02	
ENSRNOG00000002517	**Pdc**	phosducin (Pdc), mRNA [Source:RefS	25343	13	72510241	72523360	13120	3,62	-0,39	
ENSRNOG00000002525	**Ptgs2**	prostaglandin-endoperoxide synthase	29527	13	72316370	72323056	6687	11,33	-1,00	
ENSRNOG00000002531				AABR0	86	455	370	0,00		
ENSRNOG00000002532	**Prm1**	protamine 1 (Prm1), mRNA [Source:R	24685	10	3772397	3772883	487	0,00		
ENSRNOG00000002535	**Kif2b**	kinesin family member 2B (Kif2b), mR	287624	10	78940905	78942899	1995	0,00		
ENSRNOG00000002539	**Prm2**	protamine 2 (Prm2), mRNA [Source:R	25345	10	3776970	3777406	437	2,36	0,01	
ENSRNOG00000002540	**Rhoh**	ras homolog family member H (Rhoh),	305341	14	43781206	43812028	30823	5,96	0,36	
ENSRNOG00000002549	**Htr5b**	5-hydroxytryptamine (serotonin) recep	79247	13	42637728	42650516	12789	16,51	0,52	
ENSRNOG00000002550	**Atrx**	Transcriptional regulator ATRX [Sourc	246284	X	55946393	56063386	116994	1002,57	0,04	
ENSRNOG00000002555	**Serpinb7**	serpin peptidase inhibitor, clade B (ova	117092	13	32461746	32503184	41439	0,12	0,03	
ENSRNOG00000002560	**Fgf16**	fibroblast growth factor 16 (Fgf16), mF	60464	X	55910311	55920908	10598	4,22	-0,82	
ENSRNOG00000002564	**Prm3**	protamine 3 (Prm3), mRNA [Source:R	442921	10	3778043	3778557	515	0,00		
ENSRNOG00000002566	**Tnp2**	transition protein 2 (Tnp2), mRNA [Sou	24840	10	3780384	3781110	727	1,59	-0,02	
ENSRNOG00000002571	**Tekt5**	tektin 5 (Tekt5), mRNA [Source:RefSe	363538	10	4175003	4211706	36704	8,24	-0,56	

ENSRNOG00000002578	**Serpinb3a**	Protein Serpinb3a [Source:UniProtKB	498209	13	32303013	32307716	4704	0,00		
ENSRNOG00000002582	**Hand1**	heart and neural crest derivatives expı	59112	10	43050084	43052635	2552	0,00		
ENSRNOG00000002583	**Serpinb11**	serpin peptidase inhibitor, clade B (ova	304689	13	32388015	32406429	18415	0,00		
ENSRNOG00000002597	**LOC10036:**	hydroxysteroid (17-beta) dehydrogenase 6 (Hsd		7	2390324	2409187	18864	0,00		
ENSRNOG00000002598		HNF1 homeobox B [Source:MGI Symbol;Acc:M(		10	70778377	70829124	50748	1,44	-0,29	
ENSRNOG00000002599	**Grap**	GRB2-related adaptor protein (Grap),	363616	10	47723737	47742915	19179	0,97	0,41	
ENSRNOG00000002602	**Serpinb12**	serpin peptidase inhibitor, clade B (ova	304692	13	32119950	32147132	27183	0,28	0,12	
ENSRNOG00000002616	**Slc5a10**	solute carrier family 5 (sodium/sugar c	303205	10	47742920	47789792	46873	0,00		
ENSRNOG00000002624	**Edaradd**	Protein Edaradd; Similar to crinkled (F	498769	17	92462791	92502338	39548	1,12	-0,05	
ENSRNOG00000002628	**LOC10091(**	vomeronasal 2 receptor, 32 (Vom2r32	286981	1	68607184	68640948	33765	0,00		
ENSRNOG00000002636	**Abat**	4-aminobutyrate aminotransferase (Ab	81632	10	5895469	6002068	106600	2937,02	-0,53	
ENSRNOG00000002640	**Serpinb5**	serpin peptidase inhibitor, clade B (ova	116589	13	32053528	32073726	20199	2,48	-0,40	
ENSRNOG00000002641		vomeronasal 2 receptor 28 precursor	292547	1	70956193	70986046	29854	0,00		
ENSRNOG00000002657	**Pla2g4a**	phospholipase A2, group IVA (cytosoli	24653	13	72028204	72172613	144410	9,28	0,08	
ENSRNOG00000002659	**Ciita**	class II, major histocompatibility comp	85483	10	4037680	4083966	46287	0,14	-0,04	
ENSRNOG00000002661				10	4265033	4265816	784	0,72	0,04	
ENSRNOG00000002662	**Pbdc1**	polysaccharide biosynthesis domain c	363485	X	76840414	76845113	4700	456,07	-0,03	
ENSRNOG00000002691	**Shcbp1l**	SHC SH2-domain binding protein 1-lik	289101	13	75817697	75847988	30292	2,84	-0,47	
ENSRNOG00000002699		Uncharacterized protein [Source:UniProtKB/TrE		10	4503006	4503667	662	0,47	0,03	
ENSRNOG00000002701	**Hgd**	homogentisate 1, 2-dioxygenase (Hgd	360719	11	69075118	69126725	51608	5,60	-0,16	
ENSRNOG00000002712		Uncharacterized protein [Source:UniProtKB/TrE		13	87195318	87196539	1222	0,28	-0,06	
ENSRNOG00000002718	**Scgb3a1**	secretoglobin, family 3A, member 1 (S	338418	10	34905080	34906387	1308	0,59	0,03	
ENSRNOG00000002723	**Sele**	selectin E (Sele), mRNA [Source:RefS	25544	13	87235309	87242177	6869	1,60	0,05	
ENSRNOG00000002726	**LOC10036(**	Protein LOC100360993 [Source:UniP	1E+08	13	92548308	92548943	636	0,00		
ENSRNOG00000002728	**Btc**	betacellulin (Btc), mRNA [Source:RefS	64022	14	18142884	18181817	38934	1,83	-0,28	
ENSRNOG00000002730	**Rgs5**	regulator of G-protein signaling 5 (Rgs	54294	13	92595346	92631357	36012	1,17	0,17	
ENSRNOG00000002733				13	83373937	83374396	460	0,33	0,03	
ENSRNOG00000002754	**Areg**	amphiregulin (Areg), mRNA [Source:R	29183	14	18431492	18440735	9244	6,27	0,11	
ENSRNOG00000002760		Uncharacterized protein [Source:UniProtKB/TrE		13	69594212	69595105	894	2,14	-0,10	
ENSRNOG00000002768	**Tmem114**	transmembrane protein 114 (Tmem11	501675	10	6082816	6098029	15214	8,71	-0,64	
ENSRNOG00000002771	**Ereg**	epiregulin (Ereg), mRNA [Source:RefS	59325	14	18486956	18500731	13776	0,00		
ENSRNOG00000002776	**Sell**	selectin L (Sell), mRNA [Source:RefSe	29259	13	87249243	87267197	17955	0,00		
ENSRNOG00000002782	**Epgn**	Protein Epgn [Source:UniProtKB/TrEI	289515	14	18536111	18542821	6711	0,63	-0,01	
ENSRNOG00000002789	**LOC681406**			X	81982713	81983416	704	0,47	0,01	
ENSRNOG00000002792	**Cxcl2**	chemokine (C-X-C motif) ligand 2 (Cx(	114105	14	18640312	18642357	2046	0,14	-0,04	
ENSRNOG00000002794	**Selp**	selectin P (Selp), mRNA [Source:RefS	25651	13	87318111	87344163	26053	1,35	0,05	
ENSRNOG00000002802	**Cxcl1**	chemokine (C-X-C motif) ligand 1 (Cx(	81503	14	18653039	18654811	1773	6,91	0,05	
ENSRNOG00000002812	**Lhx1**	LIM homeobox 1 (Lhx1), mRNA [Sour(	257634	10	71752917	71758219	5303	10,33	0,04	

ENSRNOG00000002814	**Higd1b**	HIG1 hypoxia inducible domain family,	287738	10	90703634	90705402	1769	2,44	0,23	
ENSRNOG00000002815	**F5**	coagulation factor V (proaccelerin, lab	304929	13	87355980	87417680	61701	34,42	-1,00	
ENSRNOG00000002820	**LOC24906**	predicted gene 12253 [Source:MGI Sy	24906	10	43664047	43672276	8230	0,00		
ENSRNOG00000002829	**Ppbp**	pro-platelet basic protein (chemokine (	246358	14	18762075	18762879	805	0,00		
ENSRNOG00000002831	**Wfikkn2**	Protein Wfikkn2 [Source:UniProtKB/T	287631	10	81770067	81775078	5012	13,76	-0,85	
ENSRNOG00000002834	**Klb**	Protein Klb-ps1 [Source:UniProtKB/Tr	289625	14	44362636	44427932	65297	0,30	-0,02	
ENSRNOG00000002843	**Cxcl5**	chemokine (C-X-C motif) ligand 5 (Cx(	60665	14	18770998	18771910	913	0,72	0,04	
ENSRNOG00000002845	**LOC69128(**	RIKEN cDNA 4930504O13 gene [Sou	691286	10	43676763	43684898	8136	0,00		
ENSRNOG00000002858	**Olr1432**	olfactory receptor 1432 (Olr1432), mR	287321	10	44061159	44062130	972	0,00		
ENSRNOG00000002866	**Rassf6**	Ras association (RalGDS/AF-6) doma	305251	14	18902157	18932580	30424	4,20	-0,26	
ENSRNOG00000002878	**Afm**	afamin (Afm), mRNA [Source:RefSeq	282708	14	18987903	19039510	51608	1,61	-0,12	
ENSRNOG00000002889	**Afp**	alpha-fetoprotein (Afp), mRNA [Source	24177	14	19049096	19067264	18169	0,66	-0,03	
ENSRNOG00000002904	**Arr3**	arrestin 3, retinal (X-arrestin) (Arr3), m	171107	X	71311677	71323656	11980	7,19	-0,31	
ENSRNOG00000002906	**Nr1i2**	nuclear receptor subfamily 1, group I,	84385	11	67024186	67060494	36309	0,54	-0,09	
ENSRNOG00000002909				18	82954522	82955516	995	0,16	0,04	
ENSRNOG00000002911	**Alb**	albumin (Alb), mRNA [Source:RefSeq	24186	14	19083063	19098563	15501	4,65	-0,29	
ENSRNOG00000002916	**Car4**	carbonic anhydrase 4 (Car4), mRNA [S	29242	10	72179405	72188138	8734	0,70	-0,21	
ENSRNOG00000002917	**Sec14l5**	SEC14-like 5 (S. cerevisiae) (Sec14l5	287060	10	9359289	9397055	37767	4,20	-0,33	
ENSRNOG00000002921	**Akap4**	A kinase (PRKA) anchor protein 4 (Ak	79254	X	17082092	17091111	9020	0,00		
ENSRNOG00000002923	**Zcchc13**	Protein LOC302399; RCG38105 [Sou	302399	X	75298571	75299083	513	0,00		
ENSRNOG00000002925	**Tsx**	testis specific X-linked gene (Tsx), mR	29391	X	75079036	75089673	10638	2,30	-0,09	
ENSRNOG00000002930	**Ppl**	periplakin (Ppl), mRNA [Source:RefSe	302934	10	9411587	9457238	45652	11,01	-0,58	
ENSRNOG00000002936	**Golt1a**	golgi transport 1A (Golt1a), mRNA [So	498230	13	55590139	55602878	12740	1,30	-0,05	
ENSRNOG00000002937	**Ren**	renin (Ren), mRNA [Source:RefSeq m	24715	13	55555587	55566981	11395	6,52	-0,36	
ENSRNOG00000002947	**Dpt**	dermatopontin (Dpt), mRNA [Source:F	289178	13	87954464	87982420	27957	0,27	-0,03	
ENSRNOG00000002950	**Lyl1**	lymphoblastic leukemia derived seque	304663	19	36790826	36793693	2868	8,57	-0,56	
ENSRNOG00000002951	**Ccnb3**	Protein Ccnb3 [Source:UniProtKB/TrE	317389	X	17131037	17187119	56083	0,84	-0,36	
ENSRNOG00000002953	**P2ry4**	pyrimidinergic receptor P2Y, G-protein	63843	X	71298520	71299933	1414	11,11	-0,07	
ENSRNOG00000002960	**Ndp**	Norrie disease (pseudoglioma) (Ndp),	363443	X	7591522	7615969	24448	12,38	-0,58	
ENSRNOG00000002964	**Xcl1**	chemokine (C motif) ligand 1 (Xcl1), m	171371	13	88079753	88083196	3444	0,14	-0,04	
ENSRNOG00000002968	**Tnfsf4**	tumor necrosis factor (ligand) superfar	89814	13	84166838	84190558	23721	0,63	-0,01	
ENSRNOG00000002974	**Cdx4**	caudal type homeo box 4 (Cdx4), mRI	302400	X	74973425	74982011	8587	0,14	-0,04	
ENSRNOG00000002978	**Faslg**	Fas ligand (TNF superfamily, member	25385	13	84591721	84598981	7261	5,49	-0,18	
ENSRNOG00000002984	**Bmp15**	bone morphogenetic protein 15 (Bmp1	59302	X	17797360	17802423	5064	1,24	0,17	
ENSRNOG00000002986	**Efhc2**	EF-hand domain (C-terminal) containi	302507	X	6650827	6850639	199813	5,92	-0,40	
ENSRNOG00000003003	**Tbc1d19**	TBC1 domain family, member 19 (Tbc	289657	14	59531483	59644982	113500	611,33	0,12	
ENSRNOG00000003008	**LOC10036**	Protein LOC100361671 [Source:UniP	1E+08	X	74862391	74863023	633	0,42	-0,15	
ENSRNOG00000003009	**RGD15625**	Protein RGD1562521 [Source:UniPro	1E+08	X	74815736	74853468	37733	0,42	-0,16	

ENSRNOG00000003020	**Slc25a47**	solute carrier family 25, member 47 (S	299316		6	141932322	141938021	5700	3,08	-0,42	
ENSRNOG00000003021		Uncharacterized protein [Source:UniProtKB/TrE		X		9288757	9289305	549	0,00		
ENSRNOG00000003027				X		73514911	73515695	785	0,00		
ENSRNOG00000003030	**Awat2**	acyl-CoA wax alcohol acyltransferase	302425	X		71097874	71106134	8261	3,76	0,48	
ENSRNOG00000003035				X		70589758	70590224	467	0,00		
ENSRNOG00000003039	**Wnt3a**	wingless-type MMTV integration site fa	303181		10	45355934	45398618	42685	2,57	-0,51	
ENSRNOG00000003059	**Optc**	opticin (Optc), mRNA [Source:RefSeq	304802		13	55788831	55799588	10758	17,06	-0,51	
ENSRNOG00000003064	**Bst1**	bone marrow stromal cell antigen 1 (B	81506		14	71827040	71843323	16284	0,33	0,01	
ENSRNOG00000003067	**Npffr2**	neuropeptide FF receptor 2 (Npffr2), n	78964		14	20055407	20064042	8636	0,00		
ENSRNOG00000003080	**LOC69027**	Protein LOC690271 [Source:UniProtK	690271		13	104965563	104967179	1617	0,96	0,19	
ENSRNOG00000003085	**Mycs**	myc-like oncogene, s-myc protein (My	24581	X		18605539	18606831	1293	0,92	-0,07	
ENSRNOG00000003089					9	7026025	7026467	443	0,12	0,03	
ENSRNOG00000003094		cytidine monophospho-N-acetylneurar	361245		17	44449940	44506535	56596	0,28	-0,07	
ENSRNOG00000003095	**Fgfbp1**	fibroblast growth factor binding protein	64535		14	71679816	71680896	1081	6,58	-0,58	
ENSRNOG00000003096		Protein LOC100912068 [Source:UniP	303310		10	62192077	62194572	2496	1,89	-0,00	
ENSRNOG00000003110	**Mageb17**	melanoma antigen family B, 17 (Mage	302442	X		61434582	61438151	3570	0,00		
ENSRNOG00000003112					10	95437589	95438019	431	1,20	0,00	
ENSRNOG00000003115				X		60883009	60884026	1018	0,00		
ENSRNOG00000003119	**Gc**	group specific component (Gc), mRNA	24384		14	20173456	20209025	35570	0,14	-0,04	
ENSRNOG00000003132	**Mip**	major intrinsic protein of lens fiber (Mi	25480		7	2614664	2620388	5725	4,99	-0,01	
ENSRNOG00000003133		Uncharacterized protein [Source:UniProtKB/TrE		X		41780480	41781198	719	0,00		
ENSRNOG00000003135	**Fcrlb**	Fc receptor-like B (Fcrlb), mRNA [Sou	498275		13	93898241	93903745	5505	0,42	0,06	
ENSRNOG00000003136	**Fcrla**	Fc receptor-like A (Fcrla), mRNA [Sou	304965		13	93908889	93918565	9677	0,73	-0,02	
ENSRNOG00000003137	**Sept12**	septin 12 (Sept12), mRNA [Source:Re	363542		10	9541630	9551203	9574	0,19	0,04	
ENSRNOG00000003138	**LOC10091**	Fc gamma receptor II beta (LOC4982	116591		13	93924704	94068497	143794	0,12	0,03	
ENSRNOG00000003143	**RGD13101**	Protein RGD1310166 [Source:UniPro	303395		10	72391463	72402105	10643	1,77	0,19	
ENSRNOG00000003153	**Adcy10**	adenylate cyclase 10 (soluble) (Adcy1	59320		13	88600755	88667831	67077	5,24	-0,35	
ENSRNOG00000003158		predicted gene 8444 [Source:MGI Symbol;Acc:M		X		19761057	19761410	354	1,11	-0,40	
ENSRNOG00000003160	**LOC10090**	Ras-related GTP binding B (RragB), m	117043	X		19864639	19896314	31676	0,00		
ENSRNOG00000003164	**Pla2g10**	phospholipase A2, group X (Pla2g10),	29359		10	406661	417899	11239	0,28	-0,07	
ENSRNOG00000003170	**Nlrp3**	NLR family, pyrin domain containing 3	287362		10	45649991	45674234	24244	4,99	-0,69	
ENSRNOG00000003171	**Mpz**	myelin protein zero (Mpz), mRNA [Sou	24564		13	94151844	94157570	5727	1,28	-0,03	
ENSRNOG00000003173					10	22108808	22109415	608	0,92	0,07	
ENSRNOG00000003176	**Mixl1**	Mix paired-like homeobox 1 (Mixl1), m	289311		13	102298849	102302890	4042	0,00		
ENSRNOG00000003182					12	14582124	14583091	968	0,42	-0,15	
ENSRNOG00000003189	**Cited1**	Cbp/p300-interacting transactivator wi	64466	X		72970889	72975577	4689	153,09	0,12	
ENSRNOG00000003192		Protein LOC100364984; RCG35079, i	1E+08		10	104593141	104657878	64738	0,44	-0,08	
ENSRNOG00000003210	**Olr1463**	olfactory receptor 1463 (Olr1463), mR	287363		10	45696571	45697530	960	0,00		

ENSRNOG00000003221	**Myoc**	myocilin, trabecular meshwork inducib	81523	13	85409611	85420007	10397	5,41	0,07
ENSRNOG00000003227	**RGD15599**	Protein RGD1559951 [Source:UniPro	302528	X	12545756	12546043	288	0,12	0,03
ENSRNOG00000003233	**Serpinf2**	serpin peptidase inhibitor, clade F (alp	287527	10	61978965	61987049	8085	16,07	-0,53
ENSRNOG00000003244	**Ltc4s**	leukotriene C4 synthase (Ltc4s), mRN	114097	10	35498266	35500227	1962	0,57	-0,01
ENSRNOG00000003245	**Cacng1**	calcium channel, voltage-dependent, g	29658	10	95655314	95668486	13173	0,53	-0,06
ENSRNOG00000003246				13	65378194	65378765	572	0,44	0,00
ENSRNOG00000003263	**Lefty1**	left right determination factor 1 (Lefty1	498299	13	104212318	104215251	2934	0,35	0,13
ENSRNOG00000003266	**LOC10036**	RIKEN cDNA 1700023F06 gene [Sour	1E+08	10	91052450	91056568	4119	10,25	0,04
ENSRNOG00000003273	**Spata20**	spermatogenesis associated 20 (Spat	360604	10	82022659	82029499	6841	13,68	-0,49
ENSRNOG00000003275	**Spata32**	spermatogenesis associated 32 (Spat	287747	10	91062288	91073123	10836	1,70	0,13
ENSRNOG00000003283	**Rcsd1**	RCSD domain containing 1 (Rcsd1), n	360872	13	88734830	88797167	62338	4,64	-0,58
ENSRNOG00000003284	**Epn3**	epsin 3 (Epn3), mRNA [Source:RefSe	360605	10	82040631	82049009	8379	0,54	-0,09
ENSRNOG00000003287		F-box and WD repeat domain containi	303215	10	49038559	49066581	28023	7,24	0,03
ENSRNOG00000003294	**Cdrt4**	Protein Cdrt4 [Source:UniProtKB/TrEI	1E+08	10	49177492	49178569	1078	0,40	-0,03
ENSRNOG00000003298	**Cd247**	Cd247 molecule (Cd247), transcript va	25300	13	88876070	88950995	74926	1,10	0,22
ENSRNOG00000003299				10	25851797	25852164	368	0,26	-0,01
ENSRNOG00000003303		Uncharacterized protein [Source:UniProtKB/TrE		10	26667657	26668902	1246	0,16	0,04
ENSRNOG00000003304	**Chad**	chondroadherin (Chad), mRNA [Sourc	29195	10	82111981	82115751	3771	0,91	-0,06
ENSRNOG00000003305	**Cxcr3**	chemokine (C-X-C motif) receptor 3 (C	84475	X	72463682	72466332	2651	0,72	-0,19
ENSRNOG00000003327				10	53081756	53082314	559	0,14	-0,04
ENSRNOG00000003342		Uncharacterized protein [Source:UniProtKB/TrE		X	31392690	31393213	524	0,00	
ENSRNOG00000003345	**LOC30276**	plasmacytoma expressed transcript 2	302762	X	59350762	59353105	2344	0,00	
ENSRNOG00000003347	**Olr1016**	olfactory receptor 1016 (Olr1016), mR	288858	7	7285807	7286730	924	0,00	
ENSRNOG00000003354	**Pabpc5**	poly A binding protein, cytoplasmic 5 (	688518	X	92037339	92038700	1362	6,53	-0,20
ENSRNOG00000003355				13	76614331	76615228	898	0,00	
ENSRNOG00000003370	**Otc**	ornithine carbamoyltransferase (Otc),	25611	X	14314381	14390615	76235	4,55	-0,64
ENSRNOG00000003377	**Rbm31y**	Protein Rbm31y [Source:UniProtKB/T	302780	X	97940173	97941870	1698	0,00	
ENSRNOG00000003384	**Hs3st3b1**	heparan sulfate (glucosamine) 3-O-su	303218	10	50138478	50170551	32074	6,35	0,25
ENSRNOG00000003388	**Cenpf**	centromere protein F (Cenpf), mRNA [	257649	13	112758855	112804965	46111	447,40	0,22
ENSRNOG00000003404	**Olr947**	olfactory receptor 947 (Olr947), mRNA	288876	7	7559421	7560266	846	0,00	
ENSRNOG00000003409	**Ldoc1**	Protein Ldoc1 [Source:UniProtKB/TrE	367956	X	144834146	144834583	438	0,28	-0,12
ENSRNOG00000003430	**F9**	coagulation factor IX (F9), mRNA [Sou	24946	X	143127239	143171465	44227	0,56	-0,10
ENSRNOG00000003435		mcf.2 transforming sequence [Source:	317598	X	143189278	143320764	131487	15,68	-0,11
ENSRNOG00000003439		Interleukin-15 [Source:UniProtKB/Swi	25670	19	34532034	34598725	66692	4,51	-0,00
ENSRNOG00000003441	**Asb9**	ankyrin repeat and SOCS box-contain	367785	X	32074331	32110290	35960	0,25	0,05
ENSRNOG00000003452	**Asb11**	ankyrin repeat and SOCS box-contain	302666	X	32132257	32154178	21922	3,09	-0,63
ENSRNOG00000003456	**Actrt1**	actin-related protein T1 (Actrt1), mRN	302796	X	133306981	133308334	1354	0,00	
ENSRNOG00000003457	**Ccdc64b**	coiled-coil domain containing 64B (Cc	302964	10	12803741	12812178	8438	0,16	0,04

ENSRNOG00000003468	**Capn8**	calpain 8 (Capn8), mRNA [Source:Ref	170808	13	105915589	105977240	61652	1,19	0,03	
ENSRNOG00000003476	**Slc6a4**	solute carrier family 6 (neurotransmitte	25553	10	62851326	62872481	21156	8,99	-0,41	
ENSRNOG00000003480	**Aim2**	Protein Aim2 [Source:UniProtKB/TrEM	304987	13	96469430	96477880	8451	16,72	-0,36	
ENSRNOG00000003486	**Mnda**	myeloid cell nuclear differentiation anti	304988	13	96588441	96604826	16386	0,00		
ENSRNOG00000003498	**Olr1584**	olfactory receptor 1584 (Olr1584), mR	289247	13	96618117	96619061	945	0,00		
ENSRNOG00000003500	**Apoa2**	apolipoprotein A-II (Apoa2), mRNA [So	25649	13	94223782	94225667	1886	0,42	0,07	
ENSRNOG00000003501	**Lyzl6**	lysozyme-like 6 (Lyzl6), mRNA [Sourc	287751	10	91461088	91465885	4798	0,14	-0,04	
ENSRNOG00000003509	**Olr1588**	olfactory receptor 1588 (Olr1588), mR	289250	13	96663615	96664568	954	0,00		
ENSRNOG00000003510	**Fmo2**	flavin containing monooxygenase 2 (F	246245	13	85649741	85669947	20207	0,58	-0,12	
ENSRNOG00000003517	**Tbx2**	T-box 2 (Tbx2), mRNA [Source:RefSe	303398	10	73184218	73192261	8044	4,94	0,12	
ENSRNOG00000003518	**Olr1592**	olfactory receptor 1592 (Olr1592), mR	289254	13	96727285	96728208	924	0,19	0,04	
ENSRNOG00000003521	**Olr1593**	olfactory receptor 1593 (Olr1593), mR	289255	13	96743127	96744074	948	0,00		
ENSRNOG00000003526	**Sytl4**	synaptotagmin-like 4 (Sytl4), mRNA [S	140594	X	104599736	104625454	25719	3,18	0,38	
ENSRNOG00000003531	**LOC68578**	Protein LOC685789 [Source:UniProtK	685789	X	23156621	23159135	2515	0,00		
ENSRNOG00000003532	**Magea11**	melanoma antigen family A, 11 (Mage	302845	X	141875018	141880224	5207	2,23	-0,03	
ENSRNOG00000003543	**Gpr143**	G protein-coupled receptor 143 (Gpr1	302619	X	24084931	24109730	24800	4,41	-0,04	
ENSRNOG00000003544	**Tbx4**	T-box 4 (Tbx4), mRNA [Source:RefSe	303399	10	73235440	73265104	29665	3,37	0,09	
ENSRNOG00000003548	**LOC68521**	Protein LOC685217 [Source:UniProtK	685217	4	6018627	6019984	1358	0,00		
ENSRNOG00000003555	**Olr1597**	olfactory receptor 1597 (Olr1597), mR	405222	13	96862491	96863432	942	0,00		
ENSRNOG00000003560	**Olr1601**	olfactory receptor 1601 (Olr1601), mR	289261	13	96932848	96933801	954	0,00		
ENSRNOG00000003566	**Apoh**	apolipoprotein H (beta-2-glycoprotein	287774	10	96364126	96378117	13992	3,87	0,58	
ENSRNOG00000003569	**Gabra6**	gamma-aminobutyric acid (GABA) A re	29708	10	27691631	27706942	15312	0,14	-0,04	
ENSRNOG00000003575	**Mospd4**	motile sperm domain containing 4 (Mo	317161	18	40106381	40106956	576	0,00		
ENSRNOG00000003580	**LOC10090**	uncoupling protein 1 (mitochondrial, pr	24860	19	35434981	35442811	7831	0,00		
ENSRNOG00000003585	**Sytl5**	synaptotagmin-like 5 (Sytl5), mRNA [S	302538	X	14802671	14891273	88603	11,61	-0,32	
ENSRNOG00000003587	**Figf**	c-fos induced growth factor (Figf), mR	360457	X	32191704	32225537	33834	15,81	-1,06	
ENSRNOG00000003588		Uncharacterized protein [Source:UniProtKB/TrE		X	36598391	36601212	2822	0,00		
ENSRNOG00000003591				13	48181070	48181789	720	0,14	-0,04	
ENSRNOG00000003595		LIM homeobox protein 4 [Source:MGI Symbol;A		13	78279842	78300742	20901	6,72	-0,36	
ENSRNOG00000003596	**Itgb1bp2**	integrin beta 1 binding protein 2 (Itgb1	317258	X	72193464	72198310	4847	14,38	-0,09	
ENSRNOG00000003601	**RGD15644**	Protein RGD1564463 [Source:UniPro	289322	13	104385060	104392200	7141	0,87	0,40	
ENSRNOG00000003620	**Fmo3**	flavin containing monooxygenase 3 (F	84493	13	85732375	85750452	18078	0,00		
ENSRNOG00000003622	**Cybb**	cytochrome b-245, beta polypeptide (	66021	X	15359336	15392482	33147	9,27	-0,70	
ENSRNOG00000003629	**Nvl**	nuclear VCP-like (Nvl), mRNA [Source	289323	13	104407045	104464514	57470	338,88	0,23	
ENSRNOG00000003633	**Rcvrn**	recoverin (Rcvrn), mRNA [Source:Ref	140936	10	53992826	54000571	7746	1,89	-0,26	
ENSRNOG00000003637				X	54121641	54122153	513	0,56	-0,19	
ENSRNOG00000003648	**Cldn6**	claudin 6 (Cldn6), mRNA [Source:Ref	287098	10	12823135	12825907	2773	4,91	0,12	
ENSRNOG00000003666		Immunoglobulin joining chain; Protein Igj [Sourc		14	21087060	21093606	6547	14,26	-0,10	

ENSRNOG00000003668	**Olr1404**	olfactory receptor 1404 (Olr1404), mR	405065	10	36239004	36239951	948	0,00		
ENSRNOG00000003669	**Myocd**	myocardin (Myocd), mRNA [Source:R	246297	10	51439733	51534448	94716	0,58	-0,10	
ENSRNOG00000003671	**Prop1**	PROP paired-like homeobox 1 (Prop1	266738	10	36222695	36225156	2462	0,00		
ENSRNOG00000003672	**Enam**	enamelin (Enam), mRNA [Source:Ref	289525	14	21104462	21130928	26467	0,47	0,25	
ENSRNOG00000003681	**Lct**	lactase (Lct), mRNA [Source:RefSeq r	116569	13	50083799	50125203	41405	2,56	-0,32	
ENSRNOG00000003683	**Glp2r**	glucagon-like peptide 2 receptor (Glp2	60432	10	54006866	54070157	63292	1,25	0,09	
ENSRNOG00000003688		Protein RGD1563947; Similar to RIKEN cDNA 4		X	26149171	26150295	1125	0,00		
ENSRNOG00000003695	**RGD13105**	similar to calmegin (RGD1310572), m	304646	19	35474577	35510786	36210	2,68	-0,81	
ENSRNOG00000003698	**Mroh9**	Protein RGD1564745 [Source:UniPro	501864	13	85822779	85871847	49069	0,86	-0,27	
ENSRNOG00000003705		cytoplasmic tyrosine-protein kinase BM	367786	X	32357851	32417878	60028	13,15	0,14	
ENSRNOG00000003709	**Kmo**	kynurenine 3-monooxygenase (kynure	59113	13	98149430	98180385	30956	0,57	-0,01	
ENSRNOG00000003713	**Pbsn**	probasin (Pbsn), mRNA [Source:RefS	54193	X	45466157	45481633	15477	0,27	-0,03	
ENSRNOG00000003715		sushi repeat-containing protein SRPX	317181	X	104569972	104594594	24623	0,84	-0,10	
ENSRNOG00000003716	**Batf3**	basic leucine zipper transcription facto	60462	13	114308705	114320081	11377	6,23	-0,12	
ENSRNOG00000003718		Ameloblastin [Source:UniProtKB/Swis	25376	14	21151296	21164251	12956	1,62	0,10	
ENSRNOG00000003722	**Dusp27**	dual specificity phosphatase 27 (putati	498267	13	89180887	89208908	28022	1,05	-0,19	
ENSRNOG00000003734		CXXC finger protein 5 (Cxxc5), mRNA	681300	X	134617986	134621389	3404	2,81	0,60	
ENSRNOG00000003737	**Best2**	bestrophin 2 (Best2), mRNA [Source:F	364973	19	37098504	37105242	6739	0,96	0,07	
ENSRNOG00000003738	**Ush2a**	Usher syndrome 2A (autosomal reces	289369	13	111395505	111568482	172978	0,12	0,03	
ENSRNOG00000003740	**Gpa33**	glycoprotein A33 (transmembrane) (G	360873	13	89212323	89245475	33153	0,00		
ENSRNOG00000003744				10	28154666	28155503	838	0,47	-0,03	
ENSRNOG00000003748	**RGD15657**	similar to chromosome X open reading	501570	X	54590577	54609310	18734	0,39	-0,02	
ENSRNOG00000003758	**LOC363224**			9	49746215	49748382	2168	7,74	-0,09	
ENSRNOG00000003765	**Nr0b1**	nuclear receptor subfamily 0, group B,	58850	X	54937819	54941944	4126	5,98	-0,08	
ENSRNOG00000003776		Amelotin [Source:UniProtKB/Swiss-P	689404	14	21199166	21211724	12559	0,12	0,03	
ENSRNOG00000003777	**Chrne**	cholinergic receptor, nicotinic, epsilon	29422	10	56984504	56988822	4319	15,34	0,29	
ENSRNOG00000003783	**LOC68106**	RIKEN cDNA 1600014K23 gene [Sou	681066	X	53804780	53805328	549	0,00		
ENSRNOG00000003784	**Rilp**	Rab interacting lysosomal protein (Rilp	287531	10	64344730	64347883	3154	9,91	-0,52	
ENSRNOG00000003789				X	11729482	11731426	1945	1,15	-0,08	
ENSRNOG00000003790	**Mael**	maelstrom spermatogenic transposon	364039	13	89287037	89326509	39473	0,00		
ENSRNOG00000003791	**LOC689085**			10	97203491	97204143	653	0,59	0,09	
ENSRNOG00000003806	**Gpr65**	G-protein coupled receptor 65 (Gpr65	299242	6	131470143	131471156	1014	0,12	0,03	
ENSRNOG00000003807	**Wnt9b**	wingless-type MMTV integration site fa	303586	10	91552385	91574275	21891	7,63	0,34	
ENSRNOG00000003836	**Fam47a**	Protein Fam47a; RCG36469 [Source:	1E+08	X	46183976	46185271	1296	0,00		
ENSRNOG00000003860	**RGD15603**	Protein RGD1560334 [Source:UniPro	317454	X	44137337	44137963	627	0,00		
ENSRNOG00000003865	**Tmigd1**	transmembrane and immunoglobulin d	363654	10	62961333	62973377	12045	0,00		
ENSRNOG00000003872	**LOC10091**	solute carrier family 40 (iron-regulated	170840	9	52560181	52578109	17929	10,92	-0,21	
ENSRNOG00000003883	**LOC31745**	uncharacterized protein LOC317456	317456	X	44183614	44184572	959	0,28	-0,12	

ENSRNOG00000003884	**Acmsd**	aminocarboxymuconate semialdehyde	171385	13	49530262	49553512	23251	0,00		
ENSRNOG00000003886				X	110105717	110106371	655	0,00		
ENSRNOG00000003888	**Rgs13**	Protein Rgs13; Similar to RGS13 (Pre	498246	13	65957846	65983487	25642	2,62	-0,26	
ENSRNOG00000003891	**LOC10091(**	Putative preoptic regulatory factor 1 [S	1E+08	6	123739976	123740089	114	6,85	-0,10	
ENSRNOG00000003895	**Rgs1**	regulator of G-protein signaling 1 (Rgs	54289	13	66051576	66055901	4326	1,13	-0,29	
ENSRNOG00000003896	**Tnmd**	tenomodulin (Tnmd), mRNA [Source:F	64104	X	104520020	104535506	15487	0,16	0,04	
ENSRNOG00000003899	**Krt14**	keratin 14 (Krt14), mRNA [Source:Ref	287701	10	87910658	87914716	4059	0,66	0,22	
ENSRNOG00000003902	**Fabp6**	fatty acid binding protein 6, ileal (Fabp	25440	10	29030288	29034963	4676	0,19	0,04	
ENSRNOG00000003926		Uncharacterized protein [Source:UniProtKB/TrE		6	133189627	133189968	342	0,00		
ENSRNOG00000003944				X	36207817	36208382	566	0,25	0,05	
ENSRNOG00000003954		interleukin 2 receptor, gamma (Il2rg), r	140924	X	72015072	72022343	7272	0,61	-0,05	
ENSRNOG00000003959	**Rgs18**	regulator of G-protein signaling 18 (Rg	289076	13	66552686	66578436	25751	0,14	-0,04	
ENSRNOG00000003960	**Tmem27**	transmembrane protein 27 (Tmem27),	57395	X	32490603	32522837	32235	0,57	-0,19	
ENSRNOG00000003965		amelogenin, X-linked (Amelx), transcri	29160	X	26843989	26855286	11298	0,56	0,01	
ENSRNOG00000003969	**Fam20a**	family with sequence similarity 20, me	303635	10	97677360	97731346	53987	6,54	-0,43	
ENSRNOG00000003991		ankyrin and armadillo repeat containin	501138	9	52679229	52748446	69218	3,23	-0,53	
ENSRNOG00000003992	**Pik3r6**	phosphoinositide-3-kinase, regulatory	497932	10	54844591	54878389	33799	1,89	-0,50	
ENSRNOG00000004002	**RGD1560575**			10	67142374	67143132	759	10,56	-0,34	
ENSRNOG00000004009	**Xpnpep2**	X-prolyl aminopeptidase (aminopeptid	117522	X	135015010	135043522	28513	1,01	-0,14	
ENSRNOG00000004017				11	87191646	87192256	611	0,28	0,12	
ENSRNOG00000004018	**Tdrd5**	tudor domain containing 5 (Tdrd5), tra	289129	13	78797045	78845281	48237	16,48	0,33	
ENSRNOG00000004029	**Pvrl4**	poliovirus receptor-related 4 (Pvrl4), m	498281	13	94383718	94401716	17999	17,14	-0,23	
ENSRNOG00000004030	**Nphs2**	nephrosis 2, idiopathic, steroid-resista	170672	13	78853117	78865501	12385	0,00		
ENSRNOG00000004034				6	89028853	89029141	289	0,00		
ENSRNOG00000004053				10	17086916	17087501	586	0,28	-0,06	
ENSRNOG00000004057	**Ccdc88a**	Protein Ccdc88a [Source:UniProtKB/T	305605	14	113438294	113598052	159759	1472,43	-0,14	
ENSRNOG00000004062	**C4bpa**	complement component 4 binding pro	24235	13	52430756	52466244	35489	2,88	-0,44	
ENSRNOG00000004065		Uncharacterized protein [Source:UniProtKB/TrE		7	54973901	54975068	1168	0,62	0,10	
ENSRNOG00000004066	**LOC10091:**	RIKEN cDNA 1700056E22 gene [Sour	1E+08	13	108931488	108932192	705	2,33	-0,53	
ENSRNOG00000004075	**Ccdc42**	coiled-coil domain containing 42 (Ccdc	303234	10	54913271	54923448	10178	0,19	0,04	
ENSRNOG00000004092	**RGD15641**	Protein RGD1564148 [Source:UniPro	287415	10	54965069	54967509	2441	0,58	0,13	
ENSRNOG00000004101	**Marc1**	mitochondrial amidoxime reducing cor	690745	13	107375897	107398500	22604	0,27	-0,03	
ENSRNOG00000004108				6	98602720	98603698	979	2,42	-0,01	
ENSRNOG00000004122	**Kremen2**	kringle containing transmembrane pro	287102	10	12896141	12900598	4458	5,88	-0,30	
ENSRNOG00000004124	**Grpr**	gastrin releasing peptide receptor (Grp	24938	X	33083315	33123324	40010	8,70	-0,16	
ENSRNOG00000004125	**C4bpb**	complement component 4 binding pro	24236	13	52475837	52486554	10718	4,76	-0,00	
ENSRNOG00000004127		Uncharacterized protein [Source:UniProtKB/TrE		13	99285362	99312482	27121	0,16	0,04	
ENSRNOG00000004142	**LOC10091**	EF-hand calcium binding domain 9 (Ef	688481	10	17303083	17311280	8198	0,33	0,03	

ENSRNOG00000004144	**RGD1560234**			1	45262968	45264205	1238	7,04	0,06	
ENSRNOG00000004163		predicted gene 7589 [Source:MGI Symbol;Acc:M		6	99399798	99400307	510	0,00		
ENSRNOG00000004177	**Myo1a**	myosin IA (Myo1a), mRNA [Source:Re	299509	7	71177105	71191860	14756	1,02	-0,20	
ENSRNOG00000004188	**LOC29879!**	similar to 14-3-3 protein sigma (LOC2!	298795	6	33617273	33618544	1272	0,00		
ENSRNOG00000004192	**Arhgap30**	Rho GTPase activating protein 30 (Arl	498282	13	94402572	94423807	21236	4,72	-0,87	
ENSRNOG00000004197	**Asb12**	ankyrin repeat and SOCS box-contain	503446	X	65613516	65698971	85456	1,55	-0,02	
ENSRNOG00000004199	**LOC500610**			6	6138495	6139261	767	1,21	0,15	
ENSRNOG00000004202				6	676977	677392	416	1,27	0,20	
ENSRNOG00000004203		Peptidyl-prolyl cis-trans isomerase [Source:UniP		16	32542520	32678074	135555	6,45	-0,01	
ENSRNOG00000004209				X	33718724	33719744	1021	3,11	0,05	
ENSRNOG00000004210	**Osr1**	odd-skipped related 1 (Drosophila) (O:	298878	6	45083960	45086318	2359	10,20	-1,09	
ENSRNOG00000004216	**Lrr1**	leucine rich repeat protein 1 (Lrr1), mF	685860	6	100922215	100931215	9001	16,78	-0,09	
ENSRNOG00000004222	**S100g**	S100 calcium binding protein G (S100	24249	X	33790649	33793177	2529	0,00		
ENSRNOG00000004225	**Odf4**	outer dense fiber of sperm tails 4 (Odf	303236	10	55252753	55258298	5546	8,28	-0,33	
ENSRNOG00000004239	**LOC688546**			6	68894297	68895402	1106	13,55	0,43	
ENSRNOG00000004247	**Nhp2**	NHP2 ribonucleoprotein (Nhp2), mRN	287273	10	34748309	34751683	3375	0,00		
ENSRNOG00000004248	**Smarce1l**	Protein Smarce1l [Source:UniProtKB/	298972	6	68960832	68962009	1178	0,30	0,01	
ENSRNOG00000004249	**Tlr7**	toll-like receptor 7 (Tlr7), mRNA [Sour	317468	X	28883041	28908313	25273	2,00	-0,35	
ENSRNOG00000004262				6	152603420	152604228	809	3,64	0,51	
ENSRNOG00000004265	**RGD15597**	Protein RGD1559731 [Source:UniPro	501512	X	5476842	5525113	48272	0,12	0,03	
ENSRNOG00000004273	**Ifitm1**	interferon induced transmembrane pro	293618	1	220687945	220689004	1060	9,43	-1,01	
ENSRNOG00000004275	**Egfl6**	EGF-like-domain, multiple 6 (Egfl6), m	317470	X	29823779	29881339	57561	4,77	-0,17	
ENSRNOG00000004278	**Dlx3**	distal-less homeobox 3 (Dlx3), mRNA	287638	10	82748717	82754094	5378	0,00		
ENSRNOG00000004296	**Nt5c1b**	5'-nucleotidase, cytosolic IB (Nt5c1b),	298881	6	45851471	45868642	17172	2,61	0,02	
ENSRNOG00000004298	**Dpys**	dihydropyrimidinase (Dpys), mRNA [S	65135	7	78796405	78873621	77217	6,69	0,09	
ENSRNOG00000004302	**Pah**	phenylalanine hydroxylase (Pah), mRI	24616	7	28180707	28245424	64718	8,31	-0,70	
ENSRNOG00000004316	**Llph**	LLP homolog, long-term synaptic facili	299818	7	65224652	65227684	3033	0,41	-0,06	
[truncated: 1,509,630 more chars]
