# Supplementary material for: S-SCAM is essential for synapse formation
Source: Front Cell Neurosci. 2023 Nov 16;17:1182493. doi: 10.3389/fncel.2023.1182493 (PMC10690602; doi:10.3389/fncel.2023.1182493)
Supplement: Supplementary file 1 [file Data_Sheet_1.zip › Data Sheet 1/Suppl. Figure S5 Legend.pdf]

S5_Fig: Gene ontology analysis and visual representation of significantly altered terms classified by molecular function. Analysis performed using the GOrilla web application and show hierarchical connection between GO terms. Significance of GO enrichment is represented by color code.
